# The Geographical Coexist of the Migratory Birds, Ticks, and Nairobi Sheep Disease Virus May Potentially Contribute to the Passive Spreading of Nairobi Sheep Disease

**DOI:** 10.1155/2023/5598142

**Published:** 2023-10-30

**Authors:** KwangHyok Kim, HaoNing Wang, JinMyong Cha, XiaoLong Wang

**Affiliations:** ^1^College of Wildlife and Protected Area, Northeast Forestry University, Harbin, China; ^2^Branch of Biotechnology, State Academy of Sciences, Institute of Animal Genetic Engineering, Pyongyang, Democratic People's Republic of Korea; ^3^School of Geography and Tourism, Harbin University, Harbin, China; ^4^Kyeungsang Sariwon University of Agriculture, Sariwon, Democratic People's Republic of Korea; ^5^Key Laboratory of Wildlife Diseases and Biosecurity Management of Heilongjiang Province, Harbin, China

## Abstract

Nairobi sheep disease (NSD) is a hemorrhagic vector-borne disease of small ruminants caused by the Nairobi sheep disease virus (NSDV), also known as Ganjam virus (GV). NSDV and GV refer to the same virus. The NSDV has been identified in East Africa, India, Sri Lanka, and China, and NSDV vector ticks can be carried by birds. There is few research on the mechanism of the global cycle and spillover of NSDV. Based on the prediction of the high probability distribution areas of NSD by the maximum entropy model (MaxEnt), the possible passive transport routes of NSDV vector ticks by migratory birds were simulated for further evaluation. The transmission probability of NSDV vector ticks by migrating birds was calculated using evaluations of the parasitism intensity of ticks on migratory birds at start points, the flying burden of parasitized birds, and the attachment coefficient of ticks on birds during migration. A total of 31 potential transport routes were predicted, which, through interaction with each other, constitute a spreading network for NSDV. Seven species of migratory birds were predicted as intra or interregional carriers. Our study first provides measurable support for estimating the possibility of passive migration of NSDV vector ticks by migratory birds that may be potential carriers of ticks and proposes a transmission mechanism between all known natural foci and potential natural foci. These findings highlight the necessity of cooperation in the management of the NSDV in all known and potential natural foci located in different countries, with the aim of blocking global circulation in a cost-effective way. Furthermore, these findings may also contribute to the prevention of other similar diseases.

## 1. Introduction

The Nairobi sheep disease (NSD) is a must-reported zoonotic disease of the World Organization for Animal Health [1] caused by the Nairobi sheep disease virus (NSDV) of genus Nairovirus, family Bunyaviridae. There are seven serogroups within the Nairovirus genus, with the most important serogroups being the Crimean–Congo hemorrhagic fever group and the Nairobi sheep disease group [2]. NSDV is also known as Ganjam virus (GV) in Asia. GV was detected in Ganjam province, India, which was identified as an Asian strain of NSDV [3].

NSDV is the typical virus of the NSD serogroup. It causes hemorrhagic gastroenteritis, fever, abortion in sheep and goats [4], and febrile illness, nausea, vomiting, and headache in humans [3]. Mortality rates reach over 90% in susceptible populations [5].

This disease was first reported in Nairobi, Kenya, in 1910 [6], and it has since been found in other parts of Kenya, Uganda, Tanzania, Somalia, Ethiopia, Botswana, and Mozambique [5, 7]. NSDV has also been confirmed in Asia, including India (1954) [8], Sri Lanka (1996) [9], and China (2013) [10, 11]. It is transmitted through small ruminants, hard ticks (Ixodidae ticks), and the types of arthropods vary in each region [11]. Sheep and goats are the amplifying hosts for NSDV and are the only known vertebrate reservoirs [5], and no disease or viremia has been detected in cattle, buffalo, equids, or other mammals infected [12]. The virus was also isolated from mosquitoes (e.g., *Culex vishnui*), but no viral multiplication was observed [13]. Direct transmission of the virus between infected animals has not been established [6, 12], and it is known to be transmitted only by feeding competent infected ticks [14].

Since ticks cannot fly and the distance they can travel is limited to very short distances [15], the spread of ticks is dependent on the movement of the host [16]. The role of birds in the spread of ticks has long been emphasized, and seasonal shifts, particularly seasonal migration of birds between breeding and wintering grounds, can facilitate the spread of tick species and tick-borne diseases [17, 18]. All known arthropod vectors of NSDV can feed on birds at various stages of life, including nymphs and larvae (*Supplementary 1*). The detection of viruses of the genus *Orthonairovirus* from ticks collected from birds suggests that birds may be involved in the transmission of NSDV [19–21].

The introduction of the NSDV vector tick is recognized by many countries and regions as a potential invasiveness of NSD [7]. Although mechanisms of transmission through migratory birds have been investigated for other viral diseases belonging to the genus *Orthonairovirus*, including the Crimean–Congo hemorrhagic fever virus [22, 23], but no studies have been conducted to assess NSDV transmission based on migratory bird migration. Studies on NSD have mostly focused on classical epidemiological investigations and virological studies [11, 24]. Krasteva et al. [7] found that environmental factors such as soil moisture, livestock density, and precipitation act as key factors for NSDV circulation and pointed out Ethiopia, Malawi, Zimbabwe, Southeast China, Taiwan, and Vietnam as suitable regions for NSDV. This is, to our knowledge, the only study that contributed to the prediction of the risk distribution of NSDV. Based on NSDV detection points and tick and host distribution data, they identified potential disease transmission risk areas where NSDV could circulate. However, it did not include an interpretation of the correlation between known or potential natural foci. As the raising of small ruminants becomes more and more important in food security guarantee and poverty reduction globally, dissecting the transmission mechanism of NSDV is going to benefit the spreading block.

We utilized a niche model that combines ecological, geographic, and meteorological factors with NSDV reservoir and vector tick distribution data to predict the natural foci of NSD. Subsequently, we inferred relative probability values of NSDV vector tick spread for each migratory bird species based on the analysis of factors characterizing tick-to-bird affinity and the bird's ability to disperse ticks. This study is the first to reveal the spreading characteristics of NSDV within and among its natural foci based on predictions of the distribution range of NSDV vector ticks and the seasonal migration of migratory birds.

## 2. Materials and Methods

### 2.1. Natural Focus Prediction for NSD

#### 2.1.1. Differentiation of Prediction Areas

**Figure 1:**
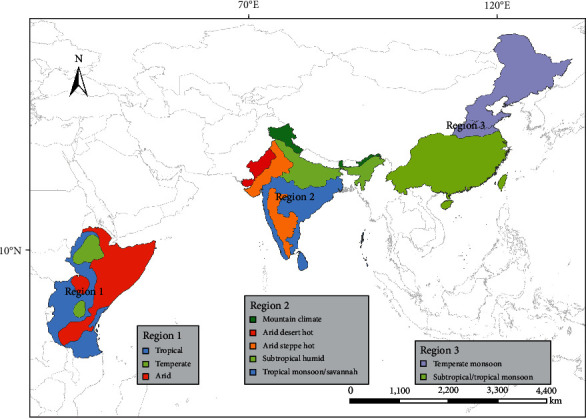
Classification of the research area's subregions based on climate traits.

Based on the regions reporting NSD, three distribution regions were obtained: East Africa (region 1), including Ethiopia, Kenya, Somalia, Tanzania, and Uganda; India and Sri Lanka (region 2), including India (with the exception of the desert region in northwest India where the distribution data of the NSDV vector tick species have not yet been confirmed) and Sri Lanka; and the part of China north of Qinling–Huaihe Line and east of Hu Line (northeast China) and the part of China south of Qinling–Huaihe Line and east of Hu Line (south China) (region 3). As various climatic conditions support gradients in species distribution and biodiversity within a region [25], we divided the three regions into subregions based on the Köppen climate classification map [26] for distribution prediction. Region 1 was divided into three subregions (tropical, arid, and temperate); region 2 was divided into five subregions (mountain climate, arid desert hot, arid steppe hot, subtropical humid, and tropical monsoon/savannah); and region 3 was divided into two subregions (temperate monsoon and subtropical/tropical monsoon) (Figure 1). Then, MaxEnt was supplied for each region separately.

#### 2.1.2. Data Collection and Preprocessing for MaxEnt

A literature review for NSD was performed on PubMed, Web of Science, Google Scholar, and China National Knowledge Infrastructure (CNKI) using by keywords “Nairobi Sheep Disease” and “Ganjam virus” in English or Chinese. A total of 309 NSD and NSDV point data were available (*Supplementary 2*). A total of 1,700 tick distribution locations were available within the study area (*Supplementary 3*).

The spatial autocorrelation minimizing and principal component analysis (PCA) were done as references [27]. The collinearity among environmental variables was assessed using the variance inflation factor (VIF) to avoid overfitting the model. A VIF value low 10 indicates that multicollinearity is acceptable [28].

Climate, terrain, and vegetation data were generated as environmental predictor categories for NSDV vector tick species habitat modeling. Density data for goats and sheep were employed as terrestrial vertebrate host data (Table 1). The preprocessing and calculation of all spatial data were conducted in ArcGIS 10.6 and projected in UTM-WGS-1984 with standard settings or resampling to 30 arc seconds [29].

#### 2.1.3. Distribution Data Collection of Migratory Birds

The distribution records of birds were obtained from Macaulay Library and eBird, while the records without accurate geographical coordinates or with disagreements were removed. A total of 29,955 distribution points for 35 species were available (*Supplementary 4*). Species attributes, red list categories, ecology, and behavior characteristics were extracted from the Birdlife International Data Zone and International Union for Conservation of Nature Red List of Threatened Species.

#### 2.1.4. Natural Foci Prediction

The distribution maps for each species of tick and NSD against regions 1, 2, and 3 were obtained by mosaic-processing the prediction map for each subregion [30]. Then, the layers of NSD, NSDV vector tick species distribution prediction, and the density of sheep and goats were overlaid to generate the distribution map of NSD. The overlay type was set to “and.” Areas with a risk value ≥0.5 were considered high-risk areas of NSD, i.e., the natural focus.

#### 2.1.5. MaxEnt Model Prediction

The MaxEnt model was used to map the suitable habitat ranges of NSD and NSDV vector tick species. Model setting refers to the study of Fekede et al. [27]. The relative contribution of the predictors for modeling was evaluated by the jackknife test and variable response curve [31], and the accuracy of the model was assessed by the area under the receiver operating characteristic curve (AUC) [32]. The general standard of AUC >0.8 was accepted to indicate a good model.

### 2.2. Estimated Transmission Trends of NSDV Vector Ticks by Migratory Birds

#### 2.2.1. The Starting and Ending Points of Migration

The start points and destinations of potential migration within each risk area were set as the southernmost and northernmost points of the NSD high-risk areas. Additionally, because the Andaman and Nicobar Islands are east to region 2 and there are migratory bird distribution points on those islands, the start points and destinations of migration were set between those islands and the westernmost high-risk area of region 2. The starting and ending points of potential migration between regions were set as the two nearest points among high-risk areas in the two regions. The Andaman and Nicobar Islands were also set as the start point and/or destination of migration in inter-region migration, considering they are stopover sites for migratory birds. If the actual seasonal migration route does not support the predicted migration route, the migration route is not set.

#### 2.2.2. Assessment of the Parasitism Intensity of Ticks on Migratory Birds in Start Points

The probability of a tick feeding on a bird is expressed as the relationship of bird nest location, bird foraging strategy, and bird population size as studies [33, 34]:

(1) $$\begin{array}{c} M_{A}=B_{N}\times B_{F}\times B_{P}\times K_{T}^{B}, \end{array}$$

where *B*_*N*_ is the weight factor according to nest location, *B*_*F*_ is the weight factor according to foraging strategy, *B*_*P*_ is the weight factor according to population size, and *K*_*T*_^*B*^ is the mean distribution probability of ticks at distribution points of migratory birds.

#### 2.2.3. Assessment of the Flying Burden Coefficient of Tick for Migratory Birds

The flight burden coefficient of ticks for migratory birds is expressed as the relationship between the bird's weight *W* and the tick intensity *N* as studies [35, 36]:

(2) $$\begin{array}{c} \alpha =e^{-{\left(\frac{N}{W}\right)}^{2}}. \end{array}$$

#### 2.2.4. Assessment of the Attachment Coefficient of Ticks during Migration

From a temporal perspective, the time the NSDV vector tick remains attached to the migratory bird's body surface and the time it takes for the migratory bird to move between points *A* and *B* within our regions are factors that determine whether migratory birds can successfully move ticks from *A* to *B*. The feeding period at that stage was taken into account as the migratory bird's attachment time since the hard ticks included in the study parasitize birds in the larval and nymphal stages.

In our study, the attachment coefficient of ticks during the migration of migratory birds from area *A* to area *B* was calculated by the following equation:

(3) $$\begin{array}{c} \beta =1-\tau \times \frac{D_{AB}}{D_{T}}, \end{array}$$

where *D*_*AB*_: date of migration of migratory birds from area *A* to *B*, *D*_*T*_: the attachment date of the tick on the body surface of the migratory bird, and *τ*: correction factor for *β*.

Birds with larger body sizes often have proportionately larger wing areas and wingspans, and these features can improve flight speed [37, 38]. The theoretical speed of movement of birds was estimated to be proportional to 15.9 × *W*^0.13^, where *W* is body mass [39].

*D*_*AB*_ is expressed as follows by the body mass (*W*), flight speed (*U*_*e*_), and flight distance (*D*_*L*_) of the migratory bird.

(4) $$\begin{array}{c} D_{AB}=\frac{D_{L}}{U_{e}}, \end{array}$$

(5) $$\begin{array}{c} U_{e}=15.9\times W^{0.13}. \end{array}$$

Therefore,

(6) $$\begin{array}{c} \beta =1-\frac{\tau \times D_{L}}{\left(15.9\times W^{0.13}\right)D_{T}}. \end{array}$$

#### 2.2.5. Assessment for Migration Probability

The possibility of intra/inter-regional transmission of NSDV vector ticks through birds' seasonal migration was evaluated by the probability that the ticks would parasite the migratory birds and the probability that the parasited birds would migrate to the area of interest. As the procedure of parasite and migration is too complicated to predict, we assumed: (i) birds migrate directly between the start point and termination without stop; (ii) the migration is not affected by the wind; and (iii) blood-sucking behavior of ticks reduces the stamina of migratory birds; and (iv) within region migration is set as north-south direction movement.

The probability that NSDV vector ticks move from area of interest *A* to *B* by migratory birds can be expressed as a host–parasitic relationship [40] as follows:

(7) $$\begin{array}{c} M_{\vec{AB}}=\alpha \times \beta \times M_{A}, \end{array}$$

where $M_{\vec{AB}}$ is the probability of a migratory bird carrying a tick from area *A* to area *B*; *M*_*A*_ is the probability of ticks feeding on migratory birds in area *A*; *α* is the tick load coefficient for migratory birds; *β* is attachment coefficient of ticks during the migration of migratory birds from area *A* to area *B*.

## 3. Results

### 3.1. Result of Natural Foci Prediction

#### 3.1.1. Result of Data Collection and Preprocessing for MaxEnt Models

In order to minimize spatial autocorrelation, filtering was performed using the minimum distance (10 km) between each pair of occurrence points [41, 42], resulting in 295 NSD and NSDV points and 1,391 tick distribution points (*Amblyomma variegatum* (*n* = 300), *Haemaphysalis intermedia* (*n* = 83), *Haemaphysalis longicornis* (*n* = 329), *Haemaphysalis wellingtoni* (*n* = 21), *Rhipicephalus appendiculatus* (*n* = 242), *Rhipicephalus haemaphysaloides* (*n* = 182), and *Rhipicephalus pulchellus* (*n* = 234)).

The VIF values of the predictors in the tick models and the NSD models were <10, meeting the criterion for low multicollinearity.

#### 3.1.2. Result of NSD and Vector Ticks Spatial Distribution Model

Model results of NSDV vector ticks are shown in *Supplementary 5*. The AUC values of 10 models were between 0.906 and 0.955, and another 16 were between 0.808 and 0.899. In NSD models, the AUC values of the two models were between 0.854 and 0.897, and another six were between 0.922 and 0.983 (*Supplementary 5*).

#### 3.1.3. Result of Natural Foci Prediction

**Figure 2:**
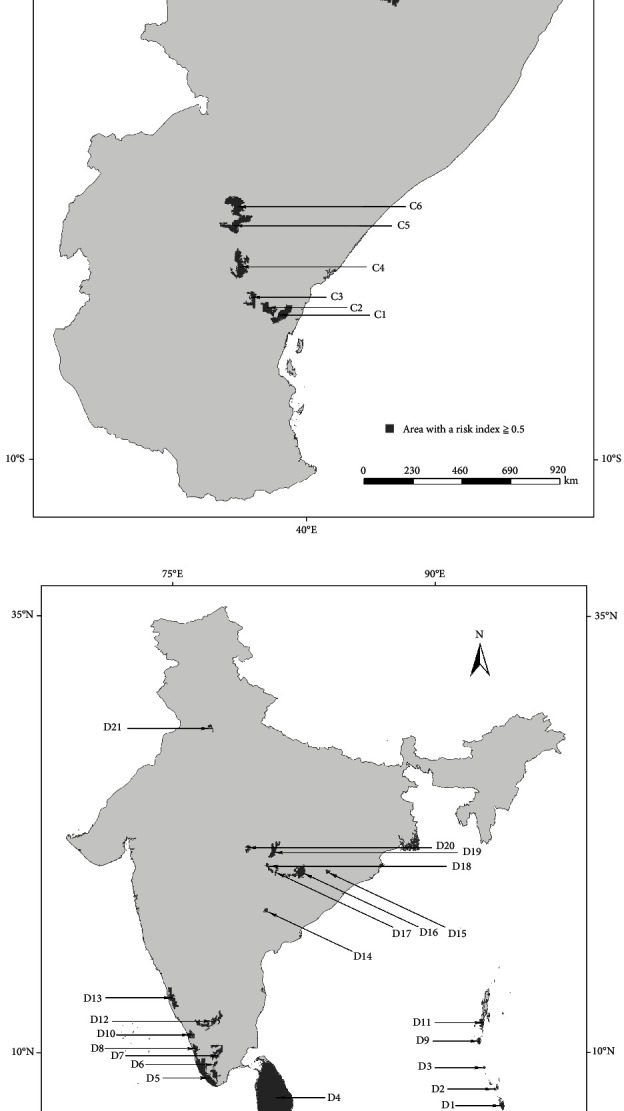
High-risk areas predicted by NSD spatial distribution model: (a) *A. variegatum*; (b) *R. appendiculatus*; (c) *R. pulchellus*; (d) *H. intermedia*; (e) *H. longicornis*; (f) *H. wellingtoni*; (g) *R. haemaphysaloides*.

High-risk areas (risk index ≥0.5) were extracted from the integrated NSD risk prediction map, and numbers were assigned based on the latitude values of the central points of each high-risk area and species of tick (Figure 2).

#### 3.1.4. Result of Data Collection and Preprocessing for Birds

There were a total of 103 species of birds that 7 species of NSDV vector ticks could parasitize on the body surface, and 41 of them were migratory birds. Of the 41 species of migratory birds, 6 species with no distribution points in the study area were excluded, and finally, 35 species were added to the list. Distribution points with the same longitude and latitude were integrated into one, and a total of 15,645 distribution points were extracted (S*upplementary 6*).

### 3.2. Result of Estimated Transmission Probability of NSDV Vector Ticks by Migratory Birds

#### 3.2.1. Results of the Starting and Ending Points of Migration Setting

For 18 species of migratory birds that meet the migration route setting conditions, the starting and ending points within and between regions were set, and as a result, 31 possible routes were obtained. Two routes (A1-A10 and A1-C7) in area 1 and 14 routes (D4-D21, D13-D11, D4-F17, D13-F6, D4-F9, D4-G15, D13-G13, F1-F17, F9-F7, F1-F9, F1-D21, F9-D11, F1-G15, and F9-G13) in area 2 were set as potential NSDV vector tick transport routes within the area, and no possible route could be set in region 3. As potential NSDV vector tick transport routes between regions, six routes (A9-D13, A9-D11, A9-F8, A9-F7, A9-G12, and A9-G13) were set between regions 1 and 2, eight routes (D11-E7, F17-E7, F7-E7, E1-D11, E1-F17, E1-F6, E1-G14, and E1-G13) between region 2 and 3, and one route (A9-E1) between region 1 and 3.

#### 3.2.2. Result of the Assessment of the Parasitism Intensity

For the parasitism intensity, please refer to the values of *M*_A_ in *Supplementary 7*. For *A. variegatum*, *H. intermedia*, and *H. wellingtoni*, Tree Pipit (*Anthus trivialis*), Indian Pitta (*Pitta brachyuran*), and Orange-headed Thrush (*Geokichla citrine*) showed the highest parasitic probability, respectively. For *H. longicornis*, only Black-faced Bunting (*Emberiza spodocephala*) was evaluated, and the value was relatively large at 0.644.

#### 3.2.3. Result of the Assessment of the Flying Burden Coefficient

The flight burden coefficient is shown in the *α* column of *Supplementary 7*. For *A. variegatum* and *H. intermedia*, Abdim's Stork (*Ciconia abdimii*) and Brahminy Starling (*Sturnus pagodarum*) showed the highest recovery values of tick flight burden, respectively. Black-faced Bunting (*Emberiza spodocephala*), the only migratory species evaluated for *H. longicornis*, had a value of 0.9849, which was the 4th lowest among all migratory species evaluated. For *H. wellingtoni*, the Clamorous Reed-warbler (*Acrocephalus stentoreus*) had the lowest value of 0.9246, and all other migratory birds had values of 0.99 or higher.

#### 3.2.4. Result of the Assessment of the Attachment Coefficient

The attachment coefficient is shown in the *β* column of *Supplementary 7*. Among the migratory species designated as *A. variegatum* carriers, Tree Pipit (*Anthus trivialis*), which migrated along route A1-A10, had the highest carrying probability value, and Cattle Egret (*Bubulcus ibis*), which migrated along route A9-E1, had the lowest value. For *H. intermedia*, the carrying probability value corresponding to the route D13-D11 of Brown Shrike (*Lanius cristatus*) was the highest, and the value of the route D11-E7 was the lowest. The carrying probability values of Black-faced Bunting (*Emberiza spodocephala*), a carrier of *H. longicornis*, were the highest in the route E1-F17 and the lowest in the route E1-F6. For *H. wellingtoni*, Orange-headed Thrush (*Geokichla citrine*) had the highest values when moving along the route F17-E7, and Western Koel (*Eudynamys scolopaceus*) had the lowest values when moving along the route F7-E7.

#### 3.2.5. Result of the Assessment of the Migration Probability of Ticks

**Figure 3:**
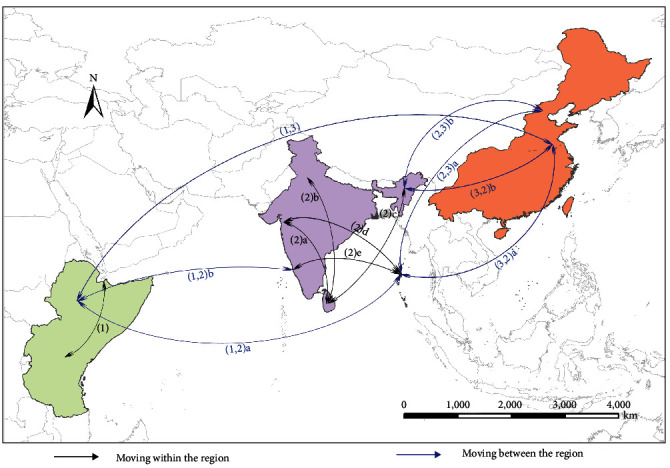
Schematic diagram of NSDV vector tick transmission by migratory birds.

The relative transport probabilities for the 31 possible migration routes of 18 species of migratory birds, which have the potential to act as intraregional or interregional carriers, are displayed in Table 2. Among the areas set as the starting and ending points of the movement route, overlapping areas were set to one point. Seven migratory bird species with the highest relative transport probabilities were selected as candidates for the 13 routes integrated with moving points (Table 3). The possible transport route of NSDV vector ticks within or between regions by migratory birds consisting of these 13 routes is shown in Figure 3. These routes shown in the schematic diagram can be interpreted as spreading routes within or between the natural foci of NSD formed by one or a combination of several.

By our analysis, the global spreading routes are a network and can be divided into six routes. They are: route 1: direct spreading route from the northern part of East Africa to East Asia (1,3); route 2: starts from the northern part of East Africa and stop at the Andaman and Nicobar islands, then splits into two to China ((1,2)a-(2,3)a and (1,2)a-(3,2)a); route 3: starts from northern part of east Africa with two stops at the southwest of India and the Andaman and Nicobar islands, then join the last half part of route 2 ((1,2)b-(2)e-(2,3)a and (1,2)b-(2)e-(3,2)a). route 4: starts from the west of India with a stop at the Andaman and Nicobar islands, finally join the last half part of route 2 ((2)d-(2,3)a and (2)d-(3,2)a); route 5: starts from west of India with two stops at Sri Lanka and northeast India, then splits into two to China ((2)a-(2)c-(2,3)b and (2)a-(2)c-(3,2)b); route 6: stats from north India with a stop in Sri Lanka and then join the last half part of route 5 ((2)b-(2)c-(2,3)b and (2)b-(2)c-(3,2)b). Within the East Africa, there is communication between the middle south and the north, while no evidence verified that they are involved into the global spreading circulation. In China, two areas were predicted in the global spreading circulations, but no communication between these two areas was confirmed.

*A. variegatum* (region 1), *H. intermedia* (region 2), *H. wellingtoni* (region 2), and *H. longicornis* (region 3), and Cattle Egret (*Bubulcus ibis*), Brown shrike (*Lanius cristatus*), Orange-headed thrush (*Geokichla citrina*), Indian Pitta (*Pitta brachyura*), Black-headed Cuckooshrike (*Lalage melanoptera*), and Black-faced Bunting (*Emberiza spodocephala*) are species involved into the global spreading circulations. As the northern part of east Africa, west and southwest India, the Andaman and Nicobar Islands, Sri Lanka, northeast India, and northeast China are important confluence regions, from the aspects of species similarity, we found on routes 2 and 3 sharing the same species of tick and birds. In addition, routes 4, 5, and 6 share the same species of ticks, and regarding migratory birds, migratory bird species of route 4 are included in route 6, and migratory species of route 6 are included in route 5.

## 4. Discussion

Based on the selection of potential natural focus areas of NSD in the study regions, the study developed a spread model of NSDV through migratory birds by connecting the focus areas with the migration routes of the birds. The study predicted 13 routes linking NSD high-risk areas of different tick species, and 7 migratory bird species have been predicted as candidates for carrying NSDV vector ticks.

In addition, eight subregions could be the starting or ending points of two or more migration routes. Specifically, the southern region of the main Ethiopian rift, including Awasa Lake, Abijatta Lake, Shalla Lake, and Langano Lake; Sri Lanka region; the northeastern region of India, including Arunachal Pradesh and Assam, western India, including Goa; the northern end of the Western Ghats of India, including the southern reaches of the Tapi river, Andaman and Nicobar Islands, the North China Plain, including Tianjin, and China's Shandong Hills. These areas may harbor two or more species of NSDV vector ticks, and at the same time, these areas lie within the migration ranges of migratory birds. Due to their environmental and geopolitical characteristics, these regions are likely to become a natural focus for NSD transmission.

In our results, the six routes recognized as global transmission routes of NSDV include tick species and migratory bird species related to NSD high-risk areas set as starting and ending points. Route 1 involves *A. variegatum* as a tick and *Bubulcus ibis* as a migratory bird, while routes 2 and 3 include ticks such as *A. variegatum*, *H. intermedia*, *H. wellingtoni*, and *H. longicornis* and migratory birds such as *Bubulcus ibis*, *Lanius cristatus*, *Geokichla citrina*, and *Emberiza spodocephala*. For hard ticks, routes 4, 5, and 6 all contain *H. intermedia*, *H. wellingtoni*, and *H. longicornis*. For migratory birds, route 4 includes *Geokichla citrina*, *Lanius cristatus*, and *Emberiza spodocephala*, route 5 includes *Pitta brachyura*, *Lalage melanoptera*, *Lanius cristatus*, *Geokichla citrina*, and *Emberiza spodocephala*, and route 6 includes *Pitta brachyura*, *Lanius cristatus*, *Geokichla citrina*, and *Emberiza spodocephala*. These pathways are networked and exhibit a reticulated NSDV circulatory pattern.

The migration route of migratory birds within or between natural foci in Figure 3 revealed the spreading of NSDV. In addition, under the premise that migratory birds are regarded as non-negligible carriers in the natural cycle of NSDV [19], they can be regarded as the future transmission route. Interlocking hypothetical propagation lines suggest that different NSDV vector ticks can be sequentially transferred to different regions by different migratory birds. In other words, it shows that even if transport is impossible with the migration of one species of migratory birds far away from each other, it can spread in stages due to the habitat overlap effect of several species of migratory birds and NSDV vector ticks.

Most notably, Andaman and Nicobar Islands belong to a high-risk area in the NSDV spreading cycle by *R. haemaphysaloides*, *H. intermedia*, and *H. wellingtoni* and lie on five migration routes linking regions 1, 2, and 3. They are also important stopover sites along the East Asian Australasian Flyway [43]. So, the Andaman and Nicobar Islands can play a role as a stepping stone in the global circulation of NSDV.

The transmission of infectious diseases is a process involving interactions among at least two, and often many, species. NSDV vector ticks are three-host types and may feed on different hosts in the larval, nymph, and adult stages [44, 45]. Their common host types and possible host types at each stage are shown in *Supplementary 8* [46]. It has been confirmed that NSDV is transovarially and transstadially transmitted by vector ticks [47]. In addition, it is known that tick larvae molt into nymphs while attached to the bird and attach to the host for a longer period of time, enabling transmission through the bird's long-distance movement [17]. NSDV vector ticks can share hosts in areas with overlapping habitats; for example, *A. variegatum*, *R. appendiculatus, R. pulchellus*, *R. haemaphysaloides*, and *H. intermedia* were collected from ruminants of the same species [48, 49]. In addition, interspecies transmission of pathogenic viruses was confirmed in ticks of different species feeding on the same host [50]. These facts suggest that the introduction of non-endemic ticks carried by migratory birds between the areas predicted as natural foci of NSD in our study could be a sufficient basis for the transmission of NSDV from one area to another.

The correlation between the natural circulation of NSDV and various environmental factors from niche model results as a natural focus of NSD is confirmed in this study. Land cover has become a major factor in tick species distribution and prediction of tick-borne diseases, as it affects tick-host density and interactions [51]. In our study, the land cover was identified as a significant predictor in the distribution models of the NSDV vector ticks and the NSD. This can be explained by the preferred habitat of hard ticks [16].

Altitude changes cause variations in climatic elements such as atmospheric temperature, water vapor, and soil moisture, which in turn cause variations in habitat and biodiversity. In general, ticks' densities have been shown to decrease with increasing altitude; the importance of elevation was emphasized in the species distribution model (SDM) of ticks [52]. In the NSDV vector ticks distribution model, elevation was expressed as an important predictor next to land cover. The distribution model results for *R. haemaphysaloides*, *H. intermedia*, and *H. wellingtoni* showed that elevation contributes as a predictor of NSDV vector ticks distribution in the mountain subregion of India in region 2.

It has been evaluated that there is a high correlation between the distribution of ticks and soil moisture [53]. If the relative humidity is less than 60%, the *Rhipicephalus* spp. larvae die [54], and increase the relative humidity, more questing ticks [55], and higher questing height [56]. In the areas where soil moisture was selected as a predictor in the tick SDM, no correlation with climate zone was confirmed, which can be seen in *Supplementary 5*. This suggests that the humidity that affects ticks is more dependent on the microclimate that directly affects the living space of ticks than on macroscopic environmental factors such as soil moisture or atmospheric humidity.

The seasonal migrations between breeding and wintering grounds of birds play a role in the long-distance dispersion of ticks. The importance of migratory birds in the introduction of exotic tick species and the spread of tick-borne pathogens was highlighted [57, 58]. The possible role of birds in the spread of tick-borne pathogens can vary and is influenced by a number of elements, including the physiology, ecology, behavior, and environmental aspects of the birds' habitat. A bird's nest provides a suitable microclimate, which makes it a preferred site for the bird's ectoparasites [59]. It was found that the abundance of ticks varies depending on the nest location and type [59, 60]. The bird's foraging strategy is also regarded as a key driver of tick parasitism [61]. Loss et al. [33] quantitatively evaluated the tick infection rate for birds based on the foraging strategy, migration strategy, and nest location. Tonelli and Dearborn [34] found that breeding range, migration timing, and propensity for tick attachment play important roles in the relative size of tick dispersal by songbird species. Brown et al. [62] found that virus prevalence varied with the vector and host group size, and Norte et al. [63] found that tick infestation caused increased stress in birds and decreased health indices.

The time spent for migration varies significantly based on the features of the bird (such as body mass, wing shape, and flight speed) [64, 65]. The flight speed is a characteristic value unique to each bird species, and different flight speeds are utilized according to the everyday routine movements, dispersal movements, migration, dispersive migration, irruptions, and nomadism [66]. Radar measurements and theoretical estimates are becoming how bird flight speeds can be obtained [39, 67]. Based on more thorough data, modeling outcomes that more precisely define the bird's flight speed have been suggested [68]. Birds with larger body sizes often have proportionately larger wing areas and wingspans, and these features can improve flight speed [38, 69]. The regression equation describing the relationship between flight speed and bird body mass presented in Alerstam et al. [39] was estimated from data from 138 bird species. These bird species taxonomically include the family to which the migratory species in our study belong, which allows a more accurate estimate of flight speed.

We have felt the need to highlight the difference from the fact that the first part of our findings can be seen as similar to that of Krasteva et al. [7]. Krasteva et al. [7] conducted a study to predict areas suitable for NSDV, and to the best of our knowledge, their data is the only one that identifies potential disease transmission risk areas related to NSD. They used five environmental variables, namely minimum temperature, annual precipitation, runoff, evapotranspiration, and soil moisture, in the model to estimate areas suitable for NSDV circulation. We used 67 climate data from CHELSA (climatologies at high resolution for the earth's land surface areas), which includes climate strata for various variables, such as monthly prediction, monthly mean temperature, monthly minimum temperature, monthly maximum temperature and annual trends, seasonality, extreme or limiting environmental variables. In addition, elevation, soil moisture, and land cover were selected as model predictors. Unlike their method of selecting environmental variables based on the requirements of hosts and ticks, we selected major predictors in the category of environmental predictors through PCA. The most important factors affecting multiparameter processes provide a much better estimation of the mutual relationship among the parameters and their evaluation, the accuracy, and efficiency of PCA-based modeling have been demonstrated [70, 71]. In addition, Based on the fact that various climatic conditions support gradients in species distribution and biodiversity within a region, we modeled the study area by dividing it into subregions according to the climatic zone [72, 73].

Some regions of East Africa and the Indian subcontinent included in the regions predicted by Krasteva et al. [7] were in agreement with our modeling results, but there were also discrepant regions. The difference in model predictors described above is the underlying cause of the difference in modeling results. Differences in tick data and modeling methods are also analyzed as causes that cannot be ignored. Krasteva et al. [7] used point data from NSDV-positive ticks, and we based all occurrence data on seven species of NSDV vector ticks. Unlike our method, which modeled tick species individually, Krasteva et al. [7] modeled several species of Ixodid ticks together. As a result, a tendency to overgeneralize the geographic distribution was expressed. The NSDV suitability region predicted by Krasteva et al. [7] covered a wider range than our results, including Malawi, Zimbabwe, southeastern China, Taiwan, and Vietnam. This may have to do with their setting a buffer zone for positive ticks and positive host points, while we pursuit to predict the most precise distribution areas. In the discussion section, it is noteworthy that Krasteva et al. [7] emphasized the importance of increased surveillance measures and suggested the potential for disease introduction by migratory birds.

Our methods are a general way for the assessment of the probability of migratory birds carrying ticks nowadays [33, 74, 75]. It contributes a way to reveal the potential spreading trend of NSD and other tick-borne diseases. The relative probability values presented in our tables are not used to determine whether migratory birds are successful in carrying ticks. It should be interpreted as a contrast value for several species, and the small probability values should not be ignored because an intermittent transmission may also bring huge harm.

The rise in tick-borne diseases emphasizes the need for preventive and control measures. Although there is no established vaccine against NSDV, the spatio-temporal model for NSD risk area prediction and research results in the field of tick biology provide a theoretical basis for effective control. Identification of high-risk areas can be a key component of tick-borne disease prevention, and surveys of migratory birds and ticks in their habitats in these areas provide a clearer picture of the size of the risk area. Avoidance of reservoir animals to NSD high-risk areas suggested in the results reduces the risk of tick exposure. Different types of vegetation management (brush removal, mowing, and removal of overstory vegetation) reduce the habitat of ticks, and the application of acaricides to vegetation decrease tick populations. Tick killing using parasitic wasps, nematodes, bacterial and fungal agents, and vertebrate and invertebrate predators is recognized as an environmentally friendly approach. It is recommended that veterinary institutions in different countries supervise and exchange information at different levels.

## 5. Conclusions

Our work focused on migratory birds that could be potential carriers of NSDV vector ticks and suggested a mechanism of transmission between all known and potential natural foci based on a quantitative assessment of their passive migration potential. Thirty-one potential transport routes constituting the NSDV dissemination network were predicted, and seven species of migratory birds were predicted to be intraregional or interregional carriers.

This study highlights the need for NSD management in focal areas believed to be disease-causing and the need for international cooperation to break the global circulation of the pathogen. This finding can also contribute to the research and prevention of other the same kinds of diseases.

## Figures and Tables

**Table 1:** Data layer and source used for NSD spatial distribution model.

Layer	Source	Value/categories	Variable/proxy
Climate			

Monthly P (prec1–12)	CHELSA	0–275 mm/month	Precipitation

Monthly mean T (temp1–12)	CHELSA	−32.6 to 37.3°C	Mean temperature

Monthly min T (tmin1–12)	CHELSA	−37.3 to 30.5°C	Minimum temperature

Monthly max T (tmax1–12)	CHELSA	−27.9 to 43.6°C	Maximum temperature

Bioclimatic (bio1–19)	CHELSA		Annual trends, seasonality, extreme, or limiting environmental variables

Terrain			

Elevation	ASTER-GDEM	−121 to 7,226 m a.s.l	Overall effect on host and tick

Soil moisture	GEOSS	0–0.00082 m^3^/m^3^	Ticks survival

Vegetation			

Land cover	ESA	Cropland (3), herbaceous, tree (9), shrubland (3), grassland, urban areas, bare areas (2), mosaic shrub and herbaceous cover, water bodies, permanent snow, and ice	Host animal food and refuge ticks host-seeking

Host			

Sheep density	GLW3	0–1,202.6 ind/km^2^	Host–disease interaction

Goat density	GLW3	0–2,768.9 ind/km^2^	Host–disease interaction

**Table 2:** Relative probability of transport of NSDV vector ticks by migratory bird.

Moving type	Region	Start point (*A*)	End point (*B*)	Birds species																			
				*Anthus trivialis*	*Bubulcus ibis*	*Ciconia abdimii*	*Acrocephalus dumetorum*	*Lanius cristatus*	*Pitta brachyura*	*Sturnus pagodarum*	*Accipiter badius*	*Acrocephalus dumetorum*	*Acrocephalus stentoreus*	*Alauda gulgula*	*Amaurornis phoenicurus*	*Circaetus gallicus*	*Eudynamys scolopaceus*	*Geokichla citrina*	*Lalage melanoptera*	*Pastor roseus*	*Saxicola caprata*	*Sturnus pagodarum*	*Emberiza spodocephala*
Moving within the region	Region 1	A1	A10	0.9452	0.5390	0.5343																	
		A1	C7		0.5387	0.5339																	
	Region 2	D4	D21				0.2958	0.4965	0.5493	0.2808													
		D13	D11					0.5124															
		D4	F17				0.3005	0.5057															
		D13	F6					0.5117															
		D4	F9						0.5692	0.2906													
		D4	G15				0.2958	0.4965	0.5492	0.2807													
		D13	G13					0.5121															
		F1	F17								0.2839	0.3613	0.2902	0.4247	0.4635		0.3598	0.5405			0.1656		
		F9	F7										0.2888	0.4227	0.4607		0.3576	0.5377		0.2315			
		F1	F9																0.3352	0.2283		0.3044	
		F1	D21								0.2810	0.3586	0.2878	0.4212	0.4586		0.3559	0.5356	0.3300	0.2274	0.1644	0.2993	
		F9	D11										0.2886	0.4223	0.4602		0.3572	0.5372		0.2281			
		F1	G15								0.2810	0.3586	0.2878	0.4212	0.4586		0.3559	0.5356	0.3300	0.2274	0.1644	0.2993	
		F9	G13										0.2885	0.4222	0.4600		0.3571	0.5370		0.2280			

Moving between the region	Region 1–Region 2	A9	D13		0.4956																		
		A9	D11		0.4576																		
		A9	F8		0.4960																		
		A9	F7		0.4577																		
		A9	G12		0.4934																		
		A9	G13		0.4574																		
	Region 2–Region 3	D11	E7					0.4872															
		F17	E7												0.4748	0.2083	0.3688	0.5516					
		F7	E7												0.4558		0.3538	0.5329					
		E1	D11																				0.5847
		E1	F17																				0.5973
		E1	F6																				0.5827
		E1	G14																				0.5970
		E1	G13																				0.5845
	Region 1–Region 3	A9	E1		0.4403																		

Tick species				*A. variegatum*			*H. intermedia*				*H. wellingtoni*												*H. longicornis*

**Table 3:** Main birds selected on the tick dispersal routes.

Moving path	Starting point	End point	NSDV-infected ticks carried by migratory birds	Migratory birds	Transport relative probability
(1)	A1	A10, C7	*Amblyomma variegatum*	*Anthus trivialis*	0.9452
(2)a	D4	F9	*Haemaphysalis intermedia*	*Pitta brachyura*	0.5493
	F1	F9	*Haemaphysalis wellingtoni*	*Lalage melanoptera*	0.3352
(2)b	D4	D21, G15	*Haemaphysalis intermedia*	*Pitta brachyura*	0.5493
	F1	D21, G15	*Haemaphysalis wellingtoni*	*Geokichla citrina*	0.5356
(2)c	D4	F17	*Haemaphysalis intermedia*	*Lanius cristatus*	0.5057
	F1	F17	*Haemaphysalis wellingtoni*	*Geokichla citrina*	0.5405
(2)d	F9	F7, D11, G13	*Haemaphysalis wellingtoni*	*Geokichla citrina*	0.5377
(2)e	D13	D11, F6, G13	*Haemaphysalis intermedia*	*Lanius cristatus*	0.5124
(1,2)a	A9	D11, F7, G13	*Amblyomma variegatum*	*Bubulcus ibis*	0.4576
(1,2)b	A9	D13, F8, G12	*Amblyomma variegatum*	*Bubulcus ibis*	0.4960
(2,3)a	D11	E7	*Haemaphysalis intermedia*	*Lanius cristatus*	0.4872
	F7	E7	*Haemaphysalis wellingtoni*	*Geokichla citrina*	0.5329
(2,3)b	F17	E7	*Haemaphysalis wellingtoni*	*Geokichla citrina*	0.5516
(3,2)a	E1	D11, F6, G13	*Haemaphysalis longicornis*	*Emberiza spodocephala*	0.5847
(3,2)b	E1	F17, G14	*Haemaphysalis longicornis*	*Emberiza spodocephala*	0.5973
(1,3)	A9	E1	*Amblyomma variegatum*	*Bubulcus ibis*	0.4403

## Data Availability

The data that support the findings of this study are available from the authors upon reasonable request.

## Supplementary Materials

Supplementary 1 Avian hosts that can be parasitized by NSDV vector tick species.

Supplementary 2 NSD and NSDV points data were used for the NSD spatial distribution model.

Supplementary 3 Ticks distribution data used for NSD spatial distribution model.

Supplementary 4 Distribution data of selected migratory birds in the study area.

Supplementary 5 Table S4: predictor contribution rate and AUC value in the tick model. Table S5: contribution rate and AUC value of predictor variables in the NSD model.

Supplementary 6 Migratory bird distribution data after preprocessing were used in the NSD spatial model.

Supplementary 7 Table S7: parasitism intensity of corresponding NSDV vector ticks on selected migratory birds. Table S8: flight burden coefficient of selected migratory birds. Table S9: attachment coefficient of ticks for selected migratory birds moving along the set routes.

Supplementary 8 Life cycle and host of NSDV vector ticks.
