# Supplementary material for: The Geographical Coexist of the Migratory Birds, Ticks, and Nairobi Sheep Disease Virus May Potentially Contribute to the Passive Spreading of Nairobi Sheep Disease
Source: Transbound Emerg Dis. 2023 Oct 30;2023:5598142. doi: 10.1155/2023/5598142 (PMC12016763; doi:10.1155/2023/5598142)
Supplement: Supplementary 1 — Avian hosts that can be parasitized by NSDV vector tick species. [file 5598142.f1.docx]

**Table S1. Avian hosts that can be parasitized by NSDV vector tick species**

| **Tick species** | **Bird species** | | **Movement patterns** | **Reference** |
| --- | --- | --- | --- | --- |
|  | Scientific name | Common name |  |  |
| *Amblyomma variegatum* | *Anthus trivialis* | Tree Pipit | Full Migrant | (Rollins et al., 2021) |
|  | *Bubo lacteus* | Verreaux's Eagle-owl | Not a Migrant | (Theiler 1959) |
|  | *Bubulcus ibis* | Cattle Egret | Full Migrant | (Corn et al., 1993, Corn 1996, Cicculli et al., 2019) |
|  | *Centropus superciliosus* | White-browed Coucal | Not a Migrant | (Theiler 1959) |
|  | *Centropus toulou* | Madagascar Coucal | Not a Migrant | (Theiler 1959) |
|  | *Ciconia abdimii* | Abdim's Stork | Full Migrant | (Theiler 1959) |
|  | *Corvus corax* | Common Raven | Not a Migrant | (Theiler 1959) |
|  | *Francolinus clappertoni* | Clapperton's Francolin | Not a Migrant | (Theiler 1959) |
|  | *Francolinus swainsonii* | Swainson's Francolin | Not a Migrant | (Zieger et al., 1998) |
|  | *Gyps africanus* | White-backed Vulture | Not a Migrant | (Theiler 1959) |
|  | *Hippolais icterina* | Icterine Warbler | Full Migrant | (Pascucci et al., 2019) |
|  | *Lophoceros nasutus* | African Grey Hornbill | Not a Migrant | (Theiler 1959) |
|  | *Numida meleagris* | Helmeted Guineafowl | Not a Migrant | (Theiler 1959, Zieger et al., 1998) |
|  | *Perdix perdix* | Grey Partridge | Not a Migrant | (Theiler 1959) |
|  | *Plectropterus gambensis* | Spur-winged Goose | Full Migrant | (Theiler 1959) |
|  | *Pternistis leucoscepus* | Yellow-necked Francolin | Not a Migrant | (Theiler 1959) |
|  | *Quiscalus lugubris* | Carib Grackle | Not a Migrant | (Corn et al., 1994, Corn 1996) |
|  | *Sagittarius serpentarius* | Secretarybird | Nomadic | (Theiler 1959) |
|  | *Tchagra senegalus* | Black-crowned Tchagra | Not a Migrant | (Theiler 1959) |
|  | *Tetrax tetrax* | Little Bustard | Full Migrant | (Theiler 1959) |
| *Haemaphysalis intermedia* | *Acridotheres fuscus* | Jungle Myna | Not a Migrant | (Rajagopalan 1972) |
|  | *Acridotheres tristis* | Common Myna | Not a Migrant | (Miranpuri et al., 1975) |
|  | *Acrocephalus dumetorum* | Blyth's Reed-warbler | Full Migrant | (Rajagopalan 1972, Miranpuri et al., 1975) |
|  | *Centropus sinensis* | Greater Coucal | Not a Migrant | (Rajagopalan 1972) |
|  | *Chloropsis aurifrons* | Golden-fronted Leafbird | Not a Migrant | (Rajagopalan 1972) |
|  | *Copsychus saularis* | Oriental Magpie-robin | Not a Migrant | (Miranpuri et al., 1975) |
|  | *Dinopium benghalense* | Black-rumped flameback | Not a Migrant | (Rajagopalan 1972) |
|  | *Francolinus pondicerianus* | Grey Francolin | Not a Migrant | (Miranpuri et al., 1975) |
|  | *Galerida malabarica* | Malabar Lark | Not a Migrant | (Rajagopalan 1972) |
|  | *Galloperdix lunulata* | Painted Spurfowl | Not a Migrant | (Miranpuri et al., 1975) |
|  | *Gallus sonneratii* | Grey Junglefowl | Not a Migrant | (Rajagopalan 1972) |
|  | *Lanius cristatus* | Brown Shrike | Full Migrant | (Miranpuri et al., 1975) |
|  | *Mirafra assamica* | Bengal Bushlark | Not a Migrant | (Miranpuri et al., 1975) |
|  | *Orthotomus sutorius* | Common Tailorbird | Not a Migrant | (Miranpuri et al., 1975) |
|  | *Passer domesticus* | House Sparrow | Not a Migrant | (Rajagopalan 1972) |
|  | *Pitta brachyura* | Indian Pitta | Full Migrant | (Miranpuri et al., 1975) |
|  | *Pycnonotus cafer* | Red-vented Bulbul | Not a Migrant | (Miranpuri et al., 1975) |
|  | *Saxicoloides fulicata* | Indian Robin | Not a Migrant | (Miranpuri et al., 1975) |
|  | *Sturnus pagodarum* | Brahminy Starling | Full Migrant | (Rajagopalan 1972, Miranpuri et al., 1975) |
|  | *Tephrodornis pondicerianus* | Common Woodshrike | Not a Migrant | (Miranpuri et al., 1975) |
|  | *Turdoides affinis* | Yellow-billed Babbler | Not a Migrant | (Miranpuri et al., 1975) |
|  | *Turdoides striata* | Jungle Babbler | Not a Migrant | (Rajagopalan 1972) |
|  | *Turdoides subrufus* | Rufous Babbler | Not a Migrant | (Rajagopalan 1972) |
|  | *Zoothera dauma* | Scaly Thrush | Full Migrant | (Rajagopalan 1972) |
| *Haemaphysalis longicornis* | *Apteryx australis* | Southern Brown Kiwi | Not a Migrant | (Heath et al., 1988) |
|  | *Branta canadensis* | Canada Goose | Full Migrant | (Zhao et al., 2020) |
|  | *Buteo jamaicensis* | Red-tailed Hawk | Full Migrant | (Zhao et al., 2020) |
|  | *Corvus corone* | Carrion Crow | Full Migrant | (Zhao et al., 2020) |
|  | *Cyanopica cyanus* | Asian Azure-winged Magpie | Not a Migrant | (Yamauchi 2001) |
|  | *Emberiza rustica* | Rustic Bunting | Full Migrant | (Yamauchi 2001) |
|  | *Emberiza spodocephala* | Black-faced Bunting | Full Migrant | (Yamauchi 2001, Zhao et al., 2020) |
|  | *Gallus gallus* | Red Junglefowl | Not a Migrant | (Zhao et al., 2020) |
|  | *Garrulus glandarius* | Eurasian Jay | Not a Migrant | (Ishiguro et al., 2000, Yamauchi 2001, Zhao et al., 2020) |
|  | *Halcyon coromanda* | Ruddy Kingfisher | Full Migrant | (Choi et al., 2014, Zhang et al., 2016, Zhao et al., 2020) |
|  | *Phasianus colchicus* | Common Pheasant | Not a Migrant | (Zhao et al., 2020) |
|  | *Pitta nympha* | Fairy Pitta | Full Migrant | (Choi et al., 2014, Zhang et al., 2016, Zhao et al., 2020) |
|  | *Rallus philippensis* | Buff-banded Rail | Full Migrant | (Heath et al., 1988) |
|  | *Spilopelia chinensis* | Eastern Spotted Dove | Full Migrant | (Zhao et al., 2020) |
|  | *Turdus cardis* | Japanese Thrush | Full Migrant | (Yamauchi 2001) |
|  | *Turdus hortulorum* | Grey-backed Thrush | Full Migrant | (Choi et al., 2014, Zhang et al., 2016, Zhao et al., 2020) |
|  | *Turdus naumanni* | Naumann's Thrush | Full Migrant | (Yamauchi 2001, Kang et al., 2013) |
|  | *Turdus pallidus* | Pale Thrush | Full Migrant | (Yamauchi 2001, Kang et al., 2013) |
|  | *Zoothera aurea* | White's Thrush | Full Migrant | (Choi et al., 2014, Zhang et al., 2016, Zhao et al., 2020) |
| *Haemaphysalis wellingtoni* | *Accipiter badius* | Shikra | Full Migrant | (Rajagopalan 1972) |
|  | *Acridotheres fuscus* | Jungle Myna | Not a Migrant | (Rajagopalan 1972) |
|  | *Acridotheres tristis* | Common Myna | Not a Migrant | (Rajagopalan 1972) |
|  | *Acrocephalus dumetorum* | Blyth's Reed-warbler | Full Migrant | (Rajagopalan 1972) |
|  | *Acrocephalus stentoreus* | Clamorous Reed-warbler | Full Migrant | (Rajagopalan 1972) |
|  | *Alauda gulgula* | Oriental Skylark | Full Migrant | (Rajagopalan 1972) |
|  | *Amaurornis phoenicurus* | White-breasted Waterhen | Full Migrant | (Rajagopalan 1972) |
|  | *Centropus sinensis* | Greater Coucal | Not a Migrant | (Rajagopalan 1972, Tanskul et al., 1983, Kuo et al., 2017) |
|  | *Circaetus gallicus* | Short-toed Snake-eagle | Full Migrant | (Rajagopalan 1972) |
|  | *Copsychus malabaricus* | White-rumped Shama | Not a Migrant | (Rajagopalan 1972) |
|  | *Copsychus saularis* | Oriental Magpie-robin | Not a Migrant | (Rajagopalan 1972) |
|  | *Corvus macrorhynchos* | Large-billed Crow | Not a Migrant | (Rajagopalan 1972) |
|  | *Dicrurus caerulescens* | White-bellied Drongo | Not a Migrant | (Rajagopalan 1972) |
|  | *Dicrurus paradiseus* | Greater Racquet-tailed Drongo | Not a Migrant | (Rajagopalan 1972) |
|  | *Eudynamys scolopaceus* | Western Koel | Full Migrant | (Rajagopalan 1972) |
|  | *Galerida malabarica* | Malabar Lark | Not a Migrant | (Rajagopalan 1972) |
|  | *Galloperdix spadicea* | Red Spurfowl | Not a Migrant | (Rajagopalan 1972) |
|  | *Gallus gallus* | Red Junglefowl | Not a Migrant | (Tanskul et al., 1983) |
|  | *Gallus sonneratii* | Grey Junglefowl | Not a Migrant | (Rajagopalan 1972) |
|  | *Garrulax leucolophus* | White-crested Laughingthrush | Not a Migrant | (Tanskul et al., 1983) |
|  | *Geokichla citrina* | Orange-headed Thrush | Full Migrant | (Rajagopalan 1972) |
|  | *Glaucidium cuculoides* | Asian Barred Owlet | Altitudinal Migrant | (Tanskul et al., 1983) |
|  | *Lalage melanoptera* | Black-headed Cuckooshrike | Full Migrant | (Rajagopalan 1972) |
|  | *Meleagris gallopavo* | Wild Turkey | Not a Migrant | (Tanskul et al., 1983) |
|  | *Mirafra erythroptera* | Indian Bushlark | Not a Migrant | (Rajagopalan 1972) |
|  | *Ocyceros griseus* | Malabar Grey Hornbill | Not a Migrant | (Rajagopalan 1972) |
|  | *Pastor roseus* | Rosy Starling | Full Migrant | (Rajagopalan 1972) |
|  | *Pavo cristatus* | Indian Peafowl | Not a Migrant | (Rajagopalan 1972) |
|  | *Pellorneum ruficeps* | Puff-throated Babbler | Not a Migrant | (Rajagopalan 1972) |
|  | *Pericrocotus flammeus* | Scarlet Minivet | Not a Migrant | (Rajagopalan 1972) |
|  | *Petronia xanthocollis* | Chestnut-shouldered Bush-sparrow | Not a Migrant | (Rajagopalan 1972) |
|  | *Pitta moluccensis* | Blue-winged Pitta | Full Migrant | (Tanskul et al., 1983) |
|  | *Pomatorhinus schisticeps* | White-browed Scimitar-babbler | Not a Migrant | (Rajagopalan 1972) |
|  | *Prinia sylvatica* | Jungle Prinia | Not a Migrant | (Rajagopalan 1972) |
|  | *Pycnonotus blanfordi* | Streak-eared Bulbul | Not a Migrant | (Tanskul et al., 1983) |
|  | *Pycnonotus cafer* | Red-vented Bulbul | Not a Migrant | (Rajagopalan 1972) |
|  | *Pycnonotus jocosus* | Red-whiskered Bulbul | Not a Migrant | (Rajagopalan 1972) |
|  | *Saxicola caprata* | Pied Bushchat | Full Migrant | (Rajagopalan 1972) |
|  | *Saxicoloides fulicata* | Indian Robin | Not a Migrant | (Rajagopalan 1972) |
|  | *Sturnus pagodarum* | Brahminy Starling | Full Migrant | (Rajagopalan 1972) |
|  | *Tephrodornis virgatus* | Large Woodshrike | Altitudinal Migrant | (Rajagopalan 1972) |
|  | *Turdoides affinis* | Yellow-billed Babbler | Not a Migrant | (Rajagopalan 1972) |
|  | *Turdoides striata* | Jungle Babbler | Not a Migrant | (Rajagopalan 1972) |
|  | *Turdus cardis* | Japanese Thrush | Full Migrant | (MIYAMOTO et al., 1993, Yamauchi 2001) |
|  | *Turdus merula* | Eurasian Blackbird | Full Migrant | (Rajagopalan 1972) |
|  | *Vanellus malabaricus* | Yellow-wattled Lapwing | Not a Migrant | (Rajagopalan 1972) |
| *Rhipicephalus appendiculatus* | *Campethera abingoni* | Golden-tailed Woodpecker | Not a Migrant | (Theiler 1959) |
|  | *Colius colius* | White-backed Mousebird | Not a Migrant | (Theiler 1959) |
|  | *Francolinus swainsonii* | Swainson's Francolin | Not a Migrant | (Zieger et al., 1998) |
|  | *Lamprotornis superbus* | Superb Starling | Not a Migrant | (Mwangi et al., 1991) |
|  | *Motacilla aguimp* | African Pied Wagtail | Not a Migrant | (Mwangi et al., 1991) |
|  | *Numida meleagris* | Helmeted Guineafowl | Not a Migrant | (Zieger et al., 1998, Walker et al., 2000) |
|  | *Prionops plumatus* | White-crested Helmetshrike | Not a Migrant | (Walker et al., 2000) |
| *Rhipicephalus haemaphysaloides* | *Argya striata* | Jungle Babbler | Not a Migrant | (Geevarghese et al., 1995) |
|  | *Centropus sinensis* | Greater Coucal | Not a Migrant | (Geevarghese et al., 1995) |
|  | *Turdus chrysolaus* | Brown-headed Thrush | Full Migrant | (Kuo et al., 2017) |
| *Rhipicephalus pulchellus* | *Pternistis leucoscepus* | Yellow-necked Francolin | Not a Migrant | (Theiler 1959, Walker et al., 2000) |
|  | *Struthio camelus* | Common Ostrich | Not a Migrant | (Walker et al., 2000) |

**References**

Choi, C.-Y., C.-W. Kang, E.-M. Kim, S. Lee, K.-H. Moon, M.-R. Oh, T. Yamauchi&Y.-M. Yun.(2014) Ticks collected from migratory birds, including a new record of Haemaphysalis formosensis, on Jeju Island, Korea. *Experimental and applied acarology*, *62*(4), 557-566

Cicculli, V., X. de Lamballerie, R. Charrel&A. Falchi.(2019) First molecular detection of Rickettsia africae in a tropical bont tick, Amblyomma variegatum, collected in Corsica, France. *Experimental and Applied Acarology*, *77*(2), 207-214

Corn, J. L.(1996) The role of wildlife in the ecology of the tropical bont tick (Amblyomma variegatum) in the West Indies.

Corn, J. L., N. Barré, B. Thiebot, T. E. Creekmore, G. I. Garris&V. F. Nettles.(1993) Potential role of cattle egrets, Bubulcus ibis (Ciconiformes: Ardeidae), in the dissemination of Amblyomma variegatum (Acari: Ixodidae) in the eastern Caribbean. *Journal of Medical Entomology*, *30*(6), 1029-1037

Corn, J. L., D. M. Kavanaugh, T. E. Creekmore&J. L. Robinson.(1994) Wildlife as hosts for ticks (Acari) in Antigua, West Indies. *Journal of medical entomology*, *31*(1), 57-61

Geevarghese, G.&V. Dhanda.(1995) Ixodid ticks of Maharashtra state, India. *Acarologia*, *36*(4), 309-313

Heath, A., J. Tenquist&D. Bishop.(1988) Bird hosts of the New Zealand cattle tick, Haemaphysalis longicornis. *New Zealand journal of zoology*, *15*(4), 585-586

Ishiguro, F., N. Takada, T. Masuzawa&T. Fukui.(2000) Prevalence of Lyme disease Borrelia spp. in ticks from migratory birds on the Japanese mainland. *Applied and Environmental Microbiology*, *66*(3), 982-986

Kang, J.-G., H.-C. Kim, C.-Y. Choi, H.-Y. Nam, H.-Y. Chae, S.-T. Chong, T. A. Klein, S. Ko&J.-S. Chae.(2013) Molecular detection of Anaplasma, Bartonella, and Borrelia species in ticks collected from migratory birds from Hong-do Island, Republic of Korea. *Vector-Borne and Zoonotic Diseases*, *13*(4), 215-225

Kuo, C.-C., Y.-F. Lin, C.-T. Yao, H.-C. Shih, L.-H. Chung, H.-C. Liao, Y.-C. Hsu&H.-C. Wang.(2017) Tick-borne pathogens in ticks collected from birds in Taiwan. *Parasites & vectors*, *10*(1), 1-13

Miranpuri, G. S., O. S. Bindra&V. Prasad.(1975) Tick fauna of north-western India (Acarina: Metastigmata). *International Journal of Acarology*, *1*(1), 31-54

MIYAMOTO, K., M. NAKAO, H. FUJITA&F. SATO.(1993) The ixodid ticks on migratory birds in Japan and the isolation of Lyme disease spirochetes from bird-feeding ticks. *Medical entomology and zoology*, *44*(4), 315-326

Mwangi, E. N., R. M. Newson&G. P. Kaaya.(1991) Predation of free-living engorged femaleRhipicephalus appendiculatus. *Experimental & applied acarology*, *12*(3), 153-162

Pascucci, I., M. Di Domenico, G. C. Dondona, A. Di Gennaro, A. Polci, A. C. Dondona, E. Mancuso, C. Cammà, G. Savini&J. G. Cecere.(2019) Assessing the role of migratory birds in the introduction of ticks and tick-borne pathogens from African countries: An Italian experience. *Ticks and tick-borne diseases*, *10*(6), 101272

Rajagopalan, P.(1972) Ixodid ticks (Acarina: Ixodidae) parasitizing wild birds in the Kyasanur forest disease area of Shimoga district, Mysore State, India. *Bombay Natur Hist Soc J*

Rollins, R. E., S. Schaper, C. Kahlhofer, D. Frangoulidis, A. F. Strauß, M. Cardinale, A. Springer, C. Strube, D. K. Bakkes&N. S. Becker.(2021) Ticks (Acari: Ixodidae) on birds migrating to the island of Ponza, Italy, and the tick-borne pathogens they carry. *Ticks and tick-borne diseases*, *12*(1), 101590

Tanskul, P., H. E. Stark&I. Inlao.(1983) A checklist of ticks of Thailand (Acari: Metastigmata: Ixodoidea). *Journal of Medical Entomology*, *20*(3), 330-341

Theiler, G.(1959) African ticks and birds. *Ostrich*, *30*(S1), 353-378

Walker, J. B., J. E. Keirans&I. G. Horak (2000). The genus Rhipicephalus (Acari, Ixodidae): a guide to the brown ticks of the world, Cambridge University Press.

Yamauchi, T.(2001) A bibliographical survey of host-parasite relationships between birds and ticks from Japan. *Bull. Hoshizaki Green Found.*, *5*, 271-308

Zhang, Y.-Z.&J. Xu.(2016) The emergence and cross species transmission of newly discovered tick-borne Bunyavirus in China. *Current opinion in virology*, *16*, 126-131

Zhao, L., J. Li, X. Cui, N. Jia, J. Wei, L. Xia, H. Wang, Y. Zhou, Q. Wang&X. Liu.(2020) Distribution of Haemaphysalis longicornis and associated pathogens: analysis of pooled data from a China field survey and global published data. *The Lancet Planetary health*, *4*(8), e320-e329

Zieger, U., A. Cauldwell, A. C. Uys&I. G. Horak.(1998) Ixodid tick infestations of wild birds and mammals on a game ranch in Central Province, Zambia.
