# Supplementary material for: The Geographical Coexist of the Migratory Birds, Ticks, and Nairobi Sheep Disease Virus May Potentially Contribute to the Passive Spreading of Nairobi Sheep Disease
Source: Transbound Emerg Dis. 2023 Oct 30;2023:5598142. doi: 10.1155/2023/5598142 (PMC12016763; doi:10.1155/2023/5598142)
Supplement: Supplementary 2 — NSD and NSDV points data were used for the NSD spatial distribution model. [file 5598142.f2.docx]

**Table S2. NSD and NSDV point data used for NSD spatial distribution model**

| **Country** | **Type** | **Longitude** | **Latitude** | **Reference** |
| --- | --- | --- | --- | --- |
| Kenya | Sheep, Goat | 35.524 | 1.494 | (Davies 1978b) |
| Kenya | Sheep, Goat | 35.517 | 1.384 | " |
| Kenya | Sheep, Goat | 35.402 | 1.346 | " |
| Kenya | Sheep, Goat | 35.270 | 1.247 | " |
| Kenya | Sheep, Goat | 35.616 | 1.315 | " |
| Kenya | Sheep, Goat | 35.699 | 1.205 | " |
| Kenya | Sheep, Goat | 35.690 | 1.111 | " |
| Kenya | Sheep, Goat | 35.569 | 0.885 | " |
| Kenya | Sheep, Goat | 35.562 | 0.788 | " |
| Kenya | Sheep, Goat | 35.430 | 0.758 | " |
| Kenya | Sheep, Goat | 35.572 | 0.676 | " |
| Kenya | Sheep, Goat | 35.574 | 0.581 | " |
| Kenya | Sheep, Goat | 35.600 | 0.452 | " |
| Kenya | Sheep, Goat | 35.442 | 0.376 | " |
| Kenya | Sheep, Goat | 35.314 | 0.771 | " |
| Kenya | Sheep, Goat | 35.257 | 0.606 | " |
| Kenya | Sheep, Goat | 34.573 | 0.685 | " |
| Kenya | Sheep, Goat | 34.755 | 0.716 | " |
| Kenya | Sheep, Goat | 34.803 | 0.635 | " |
| Kenya | Sheep, Goat | 34.341 | 0.043 | " |
| Kenya | Sheep, Goat | 34.556 | 0.165 | " |
| Kenya | Sheep, Goat | 35.009 | 0.190 | " |
| Kenya | Sheep, Goat | 35.190 | 0.399 | " |
| Kenya | Sheep, Goat | 35.165 | 0.336 | " |
| Kenya | Sheep, Goat | 35.156 | 0.264 | " |
| Kenya | Sheep, Goat | 35.193 | 0.218 | " |
| Kenya | Sheep, Goat | 35.152 | 0.138 | " |
| Kenya | Sheep, Goat | 35.214 | 0.085 | " |
| Kenya | Sheep, Goat | 35.285 | 0.173 | " |
| Kenya | Sheep, Goat | 35.782 | 0.426 | " |
| Kenya | Sheep, Goat | 35.839 | 0.276 | " |
| Kenya | Sheep, Goat | 34.240 | -0.838 | " |
| Kenya | Sheep, Goat | 34.286 | -0.718 | " |
| Kenya | Sheep, Goat | 35.546 | -0.236 | " |
| Kenya | Sheep, Goat | 35.665 | -0.235 | " |
| Kenya | Sheep, Goat | 35.536 | -0.330 | " |
| Kenya | Sheep, Goat | 34.859 | -0.506 | " |
| Kenya | Sheep, Goat | 35.037 | -0.465 | " |
| Kenya | Sheep, Goat | 35.192 | -0.494 | " |
| Kenya | Sheep, Goat | 34.763 | -0.875 | " |
| Kenya | Sheep, Goat | 34.831 | -0.807 | " |
| Kenya | Sheep, Goat | 35.007 | -0.755 | " |
| Kenya | Sheep, Goat | 35.138 | -0.826 | " |
| Kenya | Sheep, Goat | 35.149 | -0.935 | " |
| Kenya | Sheep, Goat | 35.016 | -0.948 | " |
| Kenya | Sheep, Goat | 35.066 | -1.037 | " |
| Kenya | Sheep, Goat | 34.873 | -1.127 | " |
| Kenya | Sheep, Goat | 34.868 | -1.299 | " |
| Kenya | Sheep, Goat | 35.101 | -1.107 | " |
| Kenya | Sheep, Goat | 35.056 | -1.203 | " |
| Kenya | Sheep, Goat | 35.185 | -1.287 | " |
| Kenya | Sheep, Goat | 35.307 | -1.132 | " |
| Kenya | Sheep, Goat | 35.321 | -1.312 | " |
| Kenya | Sheep, Goat | 35.472 | -1.175 | " |
| Kenya | Sheep, Goat | 35.501 | -0.966 | " |
| Kenya | Sheep, Goat | 35.82 | -1.00 | " |
| Kenya | Sheep, Goat | 36.039 | -1.049 | " |
| Kenya | Sheep, Goat | 35.838 | -1.202 | " |
| Kenya | Sheep, Goat | 35.639 | -1.544 | " |
| Kenya | Sheep, Goat | 35.88 | -1.437 | " |
| Kenya | Sheep, Goat | 35.727 | -1.827 | " |
| Kenya | Sheep, Goat | 35.873 | -1.781 | " |
| Kenya | Sheep, Goat | 36.820 | -0.114 | " |
| Kenya | Sheep, Goat | 36.921 | -0.202 | " |
| Kenya | Sheep, Goat | 36.918 | -0.399 | " |
| Kenya | Sheep, Goat | 36.957 | -0.477 | " |
| Kenya | Sheep, Goat | 36.918 | -0.560 | " |
| Kenya | Sheep, Goat | 36.500 | -0.738 | " |
| Kenya | Sheep, Goat | 36.476 | -0.912 | " |
| Kenya | Sheep, Goat | 36.695 | -0.902 | " |
| Kenya | Sheep, Goat | 36.677 | -1.009 | " |
| Kenya | Sheep, Goat | 36.774 | -0.967 | " |
| Kenya | Sheep, Goat | 36.843 | -1.042 | " |
| Kenya | Sheep, Goat | 36.789 | -1.084 | " |
| Kenya | Sheep, Goat | 36.845 | -1.125 | " |
| Kenya | Sheep, Goat | 36.877 | -1.184 | " |
| Kenya | Sheep, Goat | 36.699 | -1.195 | " |
| Kenya | Sheep, Goat | 37.010 | -1.048 | " |
| Kenya | Sheep, Goat | 36.953 | -0.851 | " |
| Kenya | Sheep, Goat | 37.064 | -0.960 | " |
| Kenya | Sheep, Goat | 37.091 | -0.863 | " |
| Kenya | Sheep, Goat | 36.954 | -0.700 | " |
| Kenya | Sheep, Goat | 37.037 | -0.622 | " |
| Kenya | Sheep, Goat | 37.077 | -0.725 | " |
| Kenya | Sheep, Goat | 37.150 | -0.651 | " |
| Kenya | Sheep, Goat | 37.184 | -0.559 | " |
| Kenya | Sheep, Goat | 37.240 | -0.765 | " |
| Kenya | Sheep, Goat | 37.260 | -0.559 | " |
| Kenya | Sheep, Goat | 37.187 | -1.116 | " |
| Kenya | Sheep, Goat | 37.087 | -1.174 | " |
| Kenya | Sheep, Goat | 36.947 | -1.236 | " |
| Kenya | Sheep, Goat | 37.044 | -1.228 | " |
| Kenya | Sheep, Goat | 37.008 | -1.280 | " |
| Kenya | Sheep, Goat | 36.728 | -1.394 | " |
| Kenya | Sheep, Goat | 37.343 | -1.157 | " |
| Kenya | Sheep, Goat | 37.051 | -1.518 | " |
| Kenya | Sheep, Goat | 37.146 | -1.474 | " |
| Kenya | Sheep, Goat | 37.346 | -1.498 | " |
| Kenya | Sheep, Goat | 37.236 | -1.915 | " |
| Kenya | Sheep, Goat | 37.327 | -0.809 | " |
| Kenya | Sheep, Goat | 37.409 | -0.769 | " |
| Kenya | Sheep, Goat | 37.376 | -0.657 | " |
| Kenya | Sheep, Goat | 37.334 | -0.557 | " |
| Kenya | Sheep, Goat | 37.510 | -0.582 | " |
| Kenya | Sheep, Goat | 37.664 | -0.671 | " |
| Kenya | Sheep, Goat | 37.712 | -0.759 | " |
| Kenya | Sheep, Goat | 37.842 | -0.530 | " |
| Kenya | Sheep, Goat | 37.634 | -0.482 | " |
| Kenya | Sheep, Goat | 37.714 | -0.387 | " |
| Kenya | Sheep, Goat | 37.528 | -0.347 | " |
| Kenya | Sheep, Goat | 37.691 | -0.299 | " |
| Kenya | Sheep, Goat | 37.722 | -0.230 | " |
| Kenya | Sheep, Goat | 37.638 | -0.183 | " |
| Kenya | Sheep, Goat | 37.725 | -0.139 | " |
| Kenya | Sheep, Goat | 37.693 | -0.072 | " |
| Kenya | Sheep, Goat | 37.625 | -0.038 | " |
| Kenya | Sheep, Goat | 37.696 | -0.004 | " |
| Kenya | Sheep, Goat | 37.796 | -0.021 | " |
| Kenya | Sheep, Goat | 37.864 | 0.004 | " |
| Kenya | Sheep, Goat | 37.958 | -0.323 | " |
| Kenya | Sheep, Goat | 38.062 | -0.387 | " |
| Kenya | Sheep, Goat | 37.926 | 0.099 | " |
| Kenya | Sheep, Goat | 37.825 | 0.127 | " |
| Kenya | Sheep, Goat | 37.885 | 0.192 | " |
| Kenya | Sheep, Goat | 37.993 | 0.192 | " |
| Kenya | Sheep, Goat | 37.987 | 0.283 | " |
| Kenya | Sheep, Goat | 37.486 | -2.927 | " |
| Kenya | Sheep, Goat | 37.623 | -2.919 | " |
| Kenya | Sheep, Goat | 37.715 | -3.052 | " |
| Kenya | Sheep, Goat | 37.748 | -3.431 | " |
| Kenya | Sheep, Goat | 37.903 | -3.395 | " |
| Kenya | Sheep, Goat | 37.797 | -3.486 | " |
| Kenya | Sheep, Goat | 38.049 | -3.466 | " |
| Kenya | Sheep, Goat | 38.145 | -3.491 | " |
| Kenya | Sheep, Goat | 38.333 | -3.550 | " |
| Kenya | Sheep, Goat | 38.38 | -3.588 | " |
| Kenya | Sheep, Goat | 38.461 | -3.620 | " |
| Kenya | Sheep, Goat | 38.385 | -3.459 | " |
| Kenya | Sheep, Goat | 38.455 | -3.402 | " |
| Kenya | Sheep, Goat | 38.479 | -3.546 | " |
| Kenya | Sheep, Goat | 38.602 | -3.566 | " |
| Kenya | Sheep, Goat | 38.651 | -3.649 | " |
| Kenya | Sheep, Goat | 38.700 | -3.819 | " |
| Kenya | Sheep, Goat | 38.674 | -3.870 | " |
| Kenya | Sheep, Goat | 38.754 | -3.886 | " |
| Kenya | Sheep, Goat | 39.581 | -4.235 | " |
| Kenya | Sheep, Goat | 39.616 | -4.010 | " |
| Kenya | Sheep, Goat | 39.676 | -4.015 | " |
| Kenya | Sheep, Goat | 39.701 | -3.961 | " |
| Kenya | Sheep, Goat | 39.735 | -3.891 | " |
| Kenya | Sheep, Goat | 39.790 | -3.759 | " |
| Kenya | Sheep, Goat | 39.561 | -3.646 | " |
| Kenya | Sheep, Goat | 39.777 | -3.630 | " |
| Kenya | Sheep, Goat | 39.852 | -3.588 | " |
| Kenya | Sheep, Goat | 39.905 | -3.458 | " |
| Kenya | Sheep, Goat | 35.034 | 0.282 | (Davies 1978a) |
| Kenya | Sheep, Goat | 35.097 | 0.163 | " |
| Kenya | Sheep, Goat | 35.097 | 0.617 | " |
| Kenya | Sheep, Goat | 35.253 | 0.536 | " |
| Kenya | Sheep, Goat | 36.740 | -0.096 | " |
| Kenya | Sheep, Goat | 36.957 | -0.187 | " |
| Kenya | Sheep, Goat | 36.698 | -0.288 | " |
| Kenya | Sheep, Goat | 37.164 | -0.428 | " |
| Kenya | Sheep, Goat | 36.355 | -1.147 | " |
| Kenya | Sheep, Goat | 36.513 | -0.929 | " |
| Kenya | Sheep, Goat | 36.617 | -0.879 | " |
| Kenya | Sheep, Goat | 36.515 | -1.249 | " |
| Kenya | Sheep, Goat | 36.55 | -1.36 | " |
| Kenya | Sheep, Goat | 36.607 | -1.293 | " |
| Kenya | Sheep, Goat | 36.619 | -1.191 | " |
| Kenya | Sheep, Goat | 36.601 | -1.086 | " |
| Kenya | Sheep, Goat | 36.713 | -1.130 | " |
| Kenya | Sheep, Goat | 36.700 | -1.239 | " |
| Kenya | Sheep, Goat | 36.693 | -1.345 | " |
| Kenya | Sheep, Goat | 36.739 | -1.027 | " |
| Kenya | Sheep, Goat | 36.829 | -0.977 | " |
| Kenya | Sheep, Goat | 36.817 | -1.088 | " |
| Kenya | Sheep, Goat | 36.798 | -1.205 | " |
| Kenya | Sheep, Goat | 36.777 | -1.301 | " |
| Kenya | Sheep, Goat | 36.881 | -1.161 | " |
| Kenya | Sheep, Goat | 36.916 | -1.059 | " |
| Kenya | Sheep, Goat | 36.998 | -0.844 | " |
| Kenya | Sheep, Goat | 36.996 | -0.979 | " |
| Kenya | Sheep, Goat | 36.993 | -1.110 | " |
| Kenya | Sheep, Goat | 37.095 | -1.046 | " |
| Kenya | Sheep, Goat | 37.084 | -1.191 | " |
| Kenya | Sheep, Goat | 36.957 | -1.206 | " |
| Kenya | Sheep, Goat | 36.88 | -1.28 | " |
| Kenya | Sheep, Goat | 36.836 | -1.379 | " |
| Kenya | Sheep, Goat | 36.772 | -1.420 | " |
| Kenya | Sheep, Goat | 36.938 | -1.359 | " |
| Kenya | Sheep, Goat | 36.911 | -1.452 | " |
| Kenya | Sheep, Goat | 36.992 | -1.442 | " |
| Kenya | Sheep, Goat | 37.090 | -1.402 | " |
| Kenya | Sheep, Goat | 37.134 | -1.312 | " |
| Kenya | Sheep, Goat | 37.034 | -1.291 | " |
| Kenya | Sheep, Goat | 37.240 | -0.969 | " |
| Kenya | Sheep, Goat | 37.420 | -0.984 | " |
| Kenya | Sheep, Goat | 37.185 | -1.647 | " |
| Kenya | Sheep, Goat | 37.176 | -1.759 | " |
| Kenya | Sheep, Goat | 37.297 | -1.775 | " |
| Kenya | Sheep, Goat | 37.439 | -2.857 | " |
| Kenya | Sheep, Goat | 37.578 | -2.886 | " |
| Kenya | Sheep, Goat | 37.668 | -2.977 | " |
| Kenya | Sheep, Goat | 39.529 | -4.271 | " |
| Kenya | Sheep, Goat | 39.601 | -4.087 | " |
| Kenya | Sheep, Goat | 39.483 | -4.034 | " |
| Kenya | Sheep, Goat | 39.580 | -4.008 | " |
| Kenya | Sheep, Goat | 39.673 | -3.988 | " |
| Kenya | Sheep, Goat | 39.624 | -3.912 | " |
| Kenya | Sheep, Goat | 39.727 | -3.915 | " |
| Kenya | Sheep, Goat | 39.512 | -3.903 | " |
| Kenya | Sheep, Goat | 39.411 | -3.873 | " |
| Kenya | Sheep, Goat | 39.583 | -3.812 | " |
| Kenya | Sheep, Goat | 39.671 | -3.827 | " |
| Kenya | Sheep, Goat | 39.765 | -3.816 | " |
| Kenya | Sheep, Goat | 39.573 | -3.700 | " |
| Kenya | Sheep, Goat | 39.657 | -3.719 | " |
| Kenya | Sheep, Goat | 39.474 | -3.780 | " |
| Kenya | Sheep, Goat | 39.764 | -3.707 | " |
| Kenya | Sheep, Goat | 39.642 | -3.615 | " |
| Kenya | Sheep, Goat | 39.730 | -3.551 | " |
| Kenya | Sheep, Goat | 39.821 | -3.609 | " |
| Kenya | Sheep, Goat | 39.757 | -3.452 | " |
| Kenya | Sheep, Goat | 39.877 | -3.469 | " |
| Somalia | Sheep, Goat | 43.113 | 10.340 | (Edelsten 1975) |
| Somalia | Sheep, Goat | 43.443 | 10.286 | " |
| Somalia | Sheep, Goat | 43.209 | 10.064 | " |
| Somalia | Sheep, Goat | 43.513 | 9.907 | " |
| Somalia | Sheep, Goat | 43.207 | 9.783 | " |
| Somalia | Sheep, Goat | 43.773 | 9.741 | " |
| Somalia | Sheep, Goat | 44.304 | 9.867 | " |
| Somalia | Sheep, Goat | 43.504 | 9.476 | " |
| Somalia | Sheep, Goat | 43.797 | 9.439 | " |
| Somalia | Sheep, Goat | 43.935 | 9.203 | " |
| Somalia | Sheep, Goat | 44.410 | 9.224 | " |
| Somalia | Sheep, Goat | 44.391 | 8.969 | " |
| Somalia | Sheep, Goat | 44.887 | 9.427 | " |
| Somalia | Sheep, Goat | 44.782 | 9.900 | " |
| Somalia | Sheep, Goat | 45.179 | 9.750 | " |
| Somalia | Sheep, Goat | 44.824 | 8.833 | " |
| Somalia | Sheep, Goat | 45.154 | 8.709 | " |
| Somalia | Sheep, Goat | 45.348 | 9.481 | " |
| Somalia | Sheep, Goat | 45.409 | 9.161 | " |
| Somalia | Sheep, Goat | 45.776 | 8.489 | " |
| Somalia | Sheep, Goat | 46.010 | 8.667 | " |
| Somalia | Sheep, Goat | 46.347 | 9.041 | " |
| Somalia | Sheep, Goat | 46.143 | 8.358 | " |
| Somalia | Sheep, Goat | 46.513 | 8.693 | " |
| Somalia | Sheep, Goat | 46.679 | 9.259 | " |
| Somalia | Sheep, Goat | 47.126 | 9.554 | " |
| Somalia | Sheep, Goat | 47.051 | 8.744 | " |
| Somalia | Sheep, Goat | 46.679 | 8.281 | " |
| Somalia | Sheep, Goat | 47.484 | 8.576 | " |
| Somalia | Sheep, Goat | 47.119 | 8.077 | " |
| Somalia | Sheep, Goat | 47.875 | 8.070 | " |
| Somalia | Sheep, Goat | 48.085 | 8.487 | " |
| Somalia | Sheep, Goat | 48.340 | 9.238 | " |
| Somalia | Sheep, Goat | 48.614 | 9.862 | " |
| Somalia | Sheep, Goat | 49.115 | 9.659 | " |
| Tanzania | Sheep, Goat | 36.682993 | -3.386925 | (Davies et al., 1978) |
| Tanzania | Sheep, Goat | 32.917452 | -2.51643 | " |
| Tanzania | Sheep, Goat | 36.48512 | -6.347795 | " |
| Tanzania | Sheep, Goat | 39.208328 | -6.792354 | " |
| Tanzania | Sheep, Goat | 35.69912 | -7.773094 | " |
| Tanzania | Sheep, Goat | 35.616667 | -2.05 | " |
| Tanzania | Sheep, Goat | 32.819733 | -5.042495 | " |
| Uganda | Sheep | 32.541517 | 0.422329 | (Weinbren et al., 1958) |
| Uganda | Goat | 33.126717 | 0.944785 | " |
| Uganda | Sheep | 32.246467 | 0.189173 | " |
| Uganda | Sheep | 30.654502 | -0.60716 | " |
| Uganda | Goat | 32.463708 | 0.051184 | " |
| Uganda | Sheep | 30.9417368 | 2.5544293 | (Terpstra 1969) |
| Uganda | Goat | 30.9417368 | 3.037925 | " |
| Uganda | Goat | 30.9403023 | 3.2873127 | " |
| Uganda | Goat | 31.1252135 | 3.1710085 | " |
| Uganda | Sheep, Goat | 30.9589496 | 3.4167992 | " |
| Uganda | Goat | 31.1975236 | 3.4448673 | " |
| Uganda | Sheep, Goat | 31.5826642 | 1.5939148 | " |
| Uganda | Sheep, Goat | 32.4467238 | 1.3489721 | " |
| Uganda | Sheep, Goat | 31.1710389 | 1.6343426 | " |
| Uganda | Sheep, Goat | 30.6199895 | 0.7137652 | " |
| Uganda | Sheep, Goat | 30.2974199 | 0.6832535 | " |
| Uganda | Sheep, Goat | 30.2051096 | 0.4870918 | " |
| Uganda | Goat | 30.0665236 | 0.3244003 | " |
| Uganda | Sheep, Goat | 29.8352303 | -0.5546336 | " |
| Uganda | Goat | 29.742604 | -0.8195253 | " |
| Uganda | Sheep, Goat | 30.043412 | -1.1326337 | " |
| Uganda | Sheep, Goat | 29.6499162 | -1.1538569 | " |
| Uganda | Sheep, Goat | 30.0202964 | -1.3504705 | " |
| Uganda | Sheep | 34.1865669 | 0.6782274 | " |
| Uganda | Goat | 34.0195827 | 0.4044731 | " |
| India | Tick | 85.051544 | 19.387388 | (Dandawate et al., 1969) |
| India | Goat | 84.582481 | 19.9358071 | " |
| India | Sheep | 75.8040022 | 13.0462575 | " |
| India | Goat | 74.9866287 | 33.7125334 | " |
| India | Tick | 74.7900264 | 19.4822619 | (Joshi et al., 2005) |
| India | Tick | 74.0301826 | 18.3463128 | " |
| India | Tick | 73.6138821 | 18.5243308 | " |
| India | Tick | 74.4659065 | 19.58041 | " |
| India | Tick | 75.1239547 | 15.3647083 | (Boshell et al., 1970) |
| India | Tick | 78.305268 | 12.305112 | " |
| India | Tick | 78.7378068 | 23.838805 | " |
| India | Sheep, Goat, Human | 80.093853 | 12.554789 | (Joshi et al., 1998) |
| Sri Lanka | Tick, Goat, Human | 79.969421 | 7.925625 | (Perera et al., 1996) |
| China | Tick | 40.866667 | 125.566667 | (Gong et al., 2015) |
| China | Tick | 43.024776 | 130.912019 | " |
| China | Tick | 40.154482 | 124.358137 | " |
| China | Tick | 32.668 | 113.246 | (Zhao et al., 2019) |

**References**

Boshell, J., P. Desai, C. Dandawate&M. Goverdhan.(1970) Isolation of Ganjam virus from ticks Haemaphysalis intermedia. *Indian Journal of Medical Research*, *58*(5), 561-562

Dandawate, C., T. Work, J. Webb&K. V. Shah.(1969) Isolation of Ganjam virus from a human case of febrile illness: a report of a laboratory infection and serological survey of human sera from three different states of India. *Indian Journal of Medical Research*, *57*(6), 975-982

Davies, F.(1978a) Nairobi sheep disease in Kenya. The isolation of virus from sheep and goats, ticks and possible maintenance hosts. *Epidemiology & Infection*, *81*(2), 259-265

Davies, F.(1978b) A survey of Nairobi sheep disease antibody in sheep and goats, wild ruminants and rodents within Kenya. *Epidemiology & Infection*, *81*(2), 251-258

Davies, F., J. Casals, D. Jesset&P. Ochieng.(1978) The serological relationships of Nairobi sheep disease virus. *Journal of Comparative Pathology*, *88*(4), 519-523

Edelsten, R.(1975) The distribution and prevalence of Nairobi sheep disease and other tick-borne infections of sheep and goats in northern Somalia. *Tropical Animal Health and Production*, *7*, 29-34

Gong, S., B. He, Z. Wang, L. Shang, F. Wei, Q. Liu&C. Tu.(2015) Nairobi sheep disease virus RNA in ixodid ticks, China, 2013. *Emerging infectious diseases*, *21*(4), 718

Joshi, M., S. Elankumaran, G. Joshi, A. Albert, V. Padbidri, B. M. Manohar, M. Ilkal, A. Undararaj, U. Umarani&A. Venugopalan.(1998) A post-epizootic survey of Rift Valley Fever-like illness among sheep at Veerapuram, Chennai, Tamil Nadu. *Indian Journal of Virology*, *14*(2), 155-157

Joshi, M., G. Geevarghese, G. Joshi, Y. Ghodke, D. Mourya&A. Mishra.(2005) Isolation of Ganjam virus from ticks collected off domestic animals around Pune, Maharashtra, India. *Journal of medical entomology*, *42*(2), 204-206

Perera, L., J. Peiris, D. Weilgama, C. Calisher&R. Shope.(1996) Nairobi sheep disease virus isolated from Haemaphysalis intermedia ticks collected in Sri Lanka. *Annals of Tropical Medicine & Parasitology*, *90*(1), 91-93

Terpstra, C.(1969) Nairobi sheep disease. Studies on virus properties, epizootiology and vaccination in Uganda. *Nairobi sheep disease. Studies on virus properties, epizootiology and vaccination in Uganda.*

Weinbren, M., R. Gourlay, W. Lumsden&B. M. Weinbren.(1958) An epizootic of Nairobi sheep disease in Uganda. *Journal of Comparative Pathology and Therapeutics*, *68*, 174-187

Zhao, Z., G. Hou, C. Zhang, J. Liu, L. Xu, W. Li, Z. Tan, C. Tu&B. He.(2019) Genomes and seroprevalence of severe fever with thrombocytopenia syndrome virus and Nairobi sheep disease virus in Haemaphysalis longicornis ticks and goats in Hubei, China. *Virology*, *529*, 234-245
