# Supplementary material for: The Geographical Coexist of the Migratory Birds, Ticks, and Nairobi Sheep Disease Virus May Potentially Contribute to the Passive Spreading of Nairobi Sheep Disease
Source: Transbound Emerg Dis. 2023 Oct 30;2023:5598142. doi: 10.1155/2023/5598142 (PMC12016763; doi:10.1155/2023/5598142)
Supplement: Supplementary 3 — Ticks distribution data used for NSD spatial distribution model. [file 5598142.f3.docx]

**Table S3. Ticks distribution data used for NSD spatial distribution model**

| **Tick species** | **Country** | **Longitude** | **Latitude** | **Reference** |
| --- | --- | --- | --- | --- |
| *Amblyomma variegatum* | Ethiopia | 37.199284 | 13.782886 | (Pegram et al., 1981) |
| *Amblyomma variegatum* | Ethiopia | 37.907816 | 13.607297 | " |
| *Amblyomma variegatum* | Ethiopia | 39.001578 | 14.059007 | " |
| *Amblyomma variegatum* | Ethiopia | 39.220044 | 13.264136 | " |
| *Amblyomma variegatum* | Ethiopia | 36.697128 | 12.987263 | " |
| *Amblyomma variegatum* | Ethiopia | 37.145801 | 12.873561 | " |
| *Amblyomma variegatum* | Ethiopia | 37.162974 | 12.555252 | " |
| *Amblyomma variegatum* | Ethiopia | 37.475537 | 12.759315 | " |
| *Amblyomma variegatum* | Ethiopia | 38.930073 | 11.160767 | " |
| *Amblyomma variegatum* | Ethiopia | 35.519294 | 11.193082 | " |
| *Amblyomma variegatum* | Ethiopia | 36.612121 | 11.386371 | " |
| *Amblyomma variegatum* | Ethiopia | 36.606607 | 10.862940 | " |
| *Amblyomma variegatum* | Ethiopia | 36.959341 | 10.385724 | " |
| *Amblyomma variegatum* | Ethiopia | 34.848868 | 10.015956 | " |
| *Amblyomma variegatum* | Ethiopia | 35.600462 | 8.717315 | " |
| *Amblyomma variegatum* | Ethiopia | 36.985470 | 8.442157 | " |
| *Amblyomma variegatum* | Ethiopia | 36.991088 | 9.476339 | " |
| *Amblyomma variegatum* | Ethiopia | 39.787659 | 10.433062 | " |
| *Amblyomma variegatum* | Ethiopia | 39.207853 | 10.425301 | " |
| *Amblyomma variegatum* | Ethiopia | 38.585004 | 10.436380 | " |
| *Amblyomma variegatum* | Ethiopia | 37.882637 | 9.853564 | " |
| *Amblyomma variegatum* | Ethiopia | 38.583746 | 9.643826 | " |
| *Amblyomma variegatum* | Ethiopia | 40.077641 | 9.246833 | " |
| *Amblyomma variegatum* | Ethiopia | 40.233461 | 8.593225 | " |
| *Amblyomma variegatum* | Ethiopia | 38.413335 | 8.376536 | " |
| *Amblyomma variegatum* | Ethiopia | 37.297821 | 8.383780 | " |
| *Amblyomma variegatum* | Ethiopia | 35.597057 | 7.377174 | " |
| *Amblyomma variegatum* | Ethiopia | 36.307741 | 7.581243 | " |
| *Amblyomma variegatum* | Ethiopia | 39.447169 | 8.117372 | " |
| *Amblyomma variegatum* | Ethiopia | 35.689761 | 8.483427 | " |
| *Amblyomma variegatum* | Ethiopia | 34.429356 | 7.805056 | " |
| *Amblyomma variegatum* | Ethiopia | 35.538143 | 7.752286 | " |
| *Amblyomma variegatum* | Ethiopia | 36.583422 | 7.530008 | " |
| *Amblyomma variegatum* | Ethiopia | 36.483433 | 6.998274 | " |
| *Amblyomma variegatum* | Ethiopia | 37.243528 | 6.975208 | " |
| *Amblyomma variegatum* | Ethiopia | 37.013013 | 6.511559 | " |
| *Amblyomma variegatum* | Ethiopia | 36.340976 | 5.208864 | " |
| *Amblyomma variegatum* | Ethiopia | 38.827235 | 7.314099 | " |
| *Amblyomma variegatum* | Ethiopia | 38.392094 | 6.476866 | " |
| *Amblyomma variegatum* | Ethiopia | 38.903154 | 5.974862 | " |
| *Amblyomma variegatum* | Ethiopia | 38.103245 | 4.821618 | " |
| *Amblyomma variegatum* | Ethiopia | 40.072651 | 4.700731 | " |
| *Amblyomma variegatum* | Ethiopia | 39.333416 | 6.547540 | " |
| *Amblyomma variegatum* | Ethiopia | 40.178744 | 5.829027 | " |
| *Amblyomma variegatum* | Ethiopia | 40.463286 | 10.115984 | " |
| *Amblyomma variegatum* | Ethiopia | 42.469304 | 9.631679 | " |
| *Amblyomma variegatum* | Ethiopia | 42.533602 | 8.343725 | " |
| *Amblyomma variegatum* | Ethiopia | 40.590460 | 7.978488 | " |
| *Amblyomma variegatum* | Ethiopia | 41.815495 | 7.927064 | " |
| *Amblyomma variegatum* | Ethiopia | 43.760611 | 6.914658 | " |
| *Amblyomma variegatum* | Ethiopia | 41.556393 | 6.832043 | " |
| *Amblyomma variegatum* | Ethiopia | 40.441032 | 10.727012 | (Mekonnen et al., 2001) |
| *Amblyomma variegatum* | Ethiopia | 40.987263 | 10.530019 | " |
| *Amblyomma variegatum* | Ethiopia | 39.843668 | 10.581094 | " |
| *Amblyomma variegatum* | Ethiopia | 39.665695 | 10.541557 | " |
| *Amblyomma variegatum* | Ethiopia | 39.91197 | 10.311425 | " |
| *Amblyomma variegatum* | Ethiopia | 39.935305 | 10.004295 | " |
| *Amblyomma variegatum* | Ethiopia | 39.712789 | 9.935454 | " |
| *Amblyomma variegatum* | Ethiopia | 39.633 | 9.757 | " |
| *Amblyomma variegatum* | Ethiopia | 39.187 | 9.935 | " |
| *Amblyomma variegatum* | Ethiopia | 39.074 | 10.06 | " |
| *Amblyomma variegatum* | Ethiopia | 39.129617 | 10.262119 | " |
| *Amblyomma variegatum* | Ethiopia | 38.990761 | 10.216174 | " |
| *Amblyomma variegatum* | Ethiopia | 38.907813 | 10.023615 | " |
| *Amblyomma variegatum* | Ethiopia | 39.603 | 9.477 | " |
| *Amblyomma variegatum* | Ethiopia | 39.74 | 9.227 | " |
| *Amblyomma variegatum* | Ethiopia | 39.493208 | 9.159882 | " |
| *Amblyomma variegatum* | Ethiopia | 39.400775 | 9.243136 | " |
| *Amblyomma variegatum* | Ethiopia | 39.678055 | 8.843295 | " |
| *Amblyomma variegatum* | Ethiopia | 40.070245 | 9.11876 | " |
| *Amblyomma variegatum* | Ethiopia | 38.767554 | 10.188297 | " |
| *Amblyomma variegatum* | Ethiopia | 38.606342 | 10.216384 | " |
| *Amblyomma variegatum* | Ethiopia | 38.586 | 9.983 | " |
| *Amblyomma variegatum* | Ethiopia | 38.412971 | 10.024669 | " |
| *Amblyomma variegatum* | Ethiopia | 38.3 | 9.977 | " |
| *Amblyomma variegatum* | Ethiopia | 38.144739 | 9.921977 | " |
| *Amblyomma variegatum* | Ethiopia | 38.375363 | 9.767586 | " |
| *Amblyomma variegatum* | Ethiopia | 38.711 | 9.59 | " |
| *Amblyomma variegatum* | Ethiopia | 38.775797 | 9.295696 | " |
| *Amblyomma variegatum* | Ethiopia | 39.057461 | 9.204869 | " |
| *Amblyomma variegatum* | Ethiopia | 38.911804 | 9.050426 | " |
| *Amblyomma variegatum* | Ethiopia | 39.234 | 9.027 | " |
| *Amblyomma variegatum* | Ethiopia | 38.895 | 8.825 | " |
| *Amblyomma variegatum* | Ethiopia | 38.987069 | 8.616974 | " |
| *Amblyomma variegatum* | Ethiopia | 39.181 | 8.646 | " |
| *Amblyomma variegatum* | Ethiopia | 39.675 | 8.724 | " |
| *Amblyomma variegatum* | Ethiopia | 38.985451 | 8.406104 | " |
| *Amblyomma variegatum* | Ethiopia | 38.888887 | 8.375428 | " |
| *Amblyomma variegatum* | Ethiopia | 38.773923 | 8.383365 | " |
| *Amblyomma variegatum* | Ethiopia | 38.925 | 8.267 | " |
| *Amblyomma variegatum* | Ethiopia | 38.808431 | 8.229213 | " |
| *Amblyomma variegatum* | Ethiopia | 38.877 | 8.172 | " |
| *Amblyomma variegatum* | Ethiopia | 38.789223 | 8.182798 | " |
| *Amblyomma variegatum* | Ethiopia | 38.616 | 7.85 | " |
| *Amblyomma variegatum* | Ethiopia | 38.562 | 7.333 | " |
| *Amblyomma variegatum* | Ethiopia | 38.277 | 7.291 | " |
| *Amblyomma variegatum* | Ethiopia | 39.340298 | 8.106154 | " |
| *Amblyomma variegatum* | Ethiopia | 39.142109 | 7.94952 | " |
| *Amblyomma variegatum* | Ethiopia | 37.9109 | 9.66447 | " |
| *Amblyomma variegatum* | Ethiopia | 38.461 | 9.509 | " |
| *Amblyomma variegatum* | Ethiopia | 38.479 | 9.355 | " |
| *Amblyomma variegatum* | Ethiopia | 38.052843 | 9.391735 | " |
| *Amblyomma variegatum* | Ethiopia | 38.425 | 9.087 | " |
| *Amblyomma variegatum* | Ethiopia | 38.175 | 9.039 | " |
| *Amblyomma variegatum* | Ethiopia | 37.479 | 9.129 | " |
| *Amblyomma variegatum* | Ethiopia | 37.164 | 9.146 | " |
| *Amblyomma variegatum* | Ethiopia | 38.717 | 8.843 | " |
| *Amblyomma variegatum* | Ethiopia | 38.58 | 8.855 | " |
| *Amblyomma variegatum* | Ethiopia | 37.658 | 8.843 | " |
| *Amblyomma variegatum* | Ethiopia | 38.027 | 8.546 | " |
| *Amblyomma variegatum* | Ethiopia | 37.545 | 8.688 | " |
| *Amblyomma variegatum* | Ethiopia | 37.442642 | 8.629115 | " |
| *Amblyomma variegatum* | Ethiopia | 38.574 | 8.404 | " |
| *Amblyomma variegatum* | Ethiopia | 38.231004 | 8.35206 | " |
| *Amblyomma variegatum* | Ethiopia | 37.646 | 8.374 | " |
| *Amblyomma variegatum* | Ethiopia | 37.765 | 8.243 | " |
| *Amblyomma variegatum* | Ethiopia | 38.497 | 8.1 | " |
| *Amblyomma variegatum* | Ethiopia | 38.330756 | 8.025629 | " |
| *Amblyomma variegatum* | Ethiopia | 38.06374 | 8.006561 | " |
| *Amblyomma variegatum* | Ethiopia | 37.765 | 8.082 | " |
| *Amblyomma variegatum* | Ethiopia | 38.211 | 7.809 | " |
| *Amblyomma variegatum* | Ethiopia | 37.979398 | 7.643986 | " |
| *Amblyomma variegatum* | Ethiopia | 37.710476 | 7.792703 | " |
| *Amblyomma variegatum* | Ethiopia | 38.254025 | 7.587777 | " |
| *Amblyomma variegatum* | Ethiopia | 38.152 | 7.464 | " |
| *Amblyomma variegatum* | Ethiopia | 37.963047 | 7.408363 | " |
| *Amblyomma variegatum* | Ethiopia | 37.85759 | 7.46293 | " |
| *Amblyomma variegatum* | Ethiopia | 37.768631 | 7.314863 | " |
| *Amblyomma variegatum* | Ethiopia | 37.508548 | 7.348933 | " |
| *Amblyomma variegatum* | Ethiopia | 38.021748 | 7.271136 | " |
| *Amblyomma variegatum* | Ethiopia | 37.883212 | 7.270417 | " |
| *Amblyomma variegatum* | Ethiopia | 36.765 | 13.685 | (Kaba 2022) |
| *Amblyomma variegatum* | Ethiopia | 39.720411 | 13.034773 | " |
| *Amblyomma variegatum* | Ethiopia | 39.583 | 12.829 | " |
| *Amblyomma variegatum* | Ethiopia | 37.288856 | 11.615466 | " |
| *Amblyomma variegatum* | Ethiopia | 39.689757 | 11.096316 | " |
| *Amblyomma variegatum* | Ethiopia | 36.889 | 10.906 | " |
| *Amblyomma variegatum* | Ethiopia | 39.391205 | 9.909163 | " |
| *Amblyomma variegatum* | Ethiopia | 36.189207 | 11.343924 | " |
| *Amblyomma variegatum* | Ethiopia | 36.388857 | 11.236021 | " |
| *Amblyomma variegatum* | Ethiopia | 36.28 | 11.02 | " |
| *Amblyomma variegatum* | Ethiopia | 36.813 | 9.65 | " |
| *Amblyomma variegatum* | Ethiopia | 38.291755 | 9.549779 | " |
| *Amblyomma variegatum* | Ethiopia | 42.067 | 9.421 | " |
| *Amblyomma variegatum* | Ethiopia | 35.89 | 8.707 | " |
| *Amblyomma variegatum* | Ethiopia | 37.032 | 9.098 | " |
| *Amblyomma variegatum* | Ethiopia | 38.488 | 9.107 | " |
| *Amblyomma variegatum* | Ethiopia | 36.368019 | 8.505942 | " |
| *Amblyomma variegatum* | Ethiopia | 38.707 | 8.831 | " |
| *Amblyomma variegatum* | Ethiopia | 40.903749 | 8.822629 | " |
| *Amblyomma variegatum* | Ethiopia | 42.530822 | 8.899372 | " |
| *Amblyomma variegatum* | Ethiopia | 37.814843 | 7.9345 | " |
| *Amblyomma variegatum* | Ethiopia | 38.722084 | 7.891322 | " |
| *Amblyomma variegatum* | Ethiopia | 39.097 | 7.984 | " |
| *Amblyomma variegatum* | Ethiopia | 37.784 | 7.527 | " |
| *Amblyomma variegatum* | Ethiopia | 37.801 | 6.731 | " |
| *Amblyomma variegatum* | Ethiopia | 38.163644 | 6.43179 | " |
| *Amblyomma variegatum* | Ethiopia | 38.696 | 6.741 | " |
| *Amblyomma variegatum* | Ethiopia | 44.597032 | 6.857478 | " |
| *Amblyomma variegatum* | Ethiopia | 38.092296 | 4.957656 | " |
| *Amblyomma variegatum* | Ethiopia | 38.40779 | 4.67582 | " |
| *Amblyomma variegatum* | Ethiopia | 37.998203 | 4.248911 | " |
| *Amblyomma variegatum* | Kenya | 36.715 | -1.148 | (Ngumi et al., 1997) |
| *Amblyomma variegatum* | Kenya | 37.388 | -1.275 | " |
| *Amblyomma variegatum* | Kenya | 36.106 | 0.578 | " |
| *Amblyomma variegatum* | Kenya | 34.393 | -0.184 | " |
| *Amblyomma variegatum* | Kenya | 35.865 | -1.618 | " |
| *Amblyomma variegatum* | Kenya | 37.203611 | 1.450762 | " |
| *Amblyomma variegatum* | Kenya | 34.202 | 0.298 | " |
| *Amblyomma variegatum* | Kenya | 36.982 | -0.945 | " |
| *Amblyomma variegatum* | Kenya | 34.989 | -0.336 | " |
| *Amblyomma variegatum* | Kenya | 37.565 | -1.427 | " |
| *Amblyomma variegatum* | Kenya | 39.629145 | -3.813214 | (Zulu et al., 1998) |
| *Amblyomma variegatum* | Kenya | 37.545135 | -1.899685 | " |
| *Amblyomma variegatum* | Kenya | 36.022173 | 0.61466 | (Omondi et al., 2017) |
| *Amblyomma variegatum* | Kenya | 36.097664 | 0.644607 | " |
| *Amblyomma variegatum* | Kenya | 36.137579 | 0.438299 | " |
| *Amblyomma variegatum* | Kenya | 34.190255 | -0.354661 | " |
| *Amblyomma variegatum* | Kenya | 34.206396 | -0.439786 | " |
| *Amblyomma variegatum* | Kenya | 34.012383 | -0.467772 | " |
| *Amblyomma variegatum* | Kenya | 39.698043 | 3.579901 | (Walker et al., 1987) |
| *Amblyomma variegatum* | Kenya | 35.543 | 1.523 | " |
| *Amblyomma variegatum* | Kenya | 36.630767 | 1.310484 | " |
| *Amblyomma variegatum* | Kenya | 34.59173 | 0.543018 | " |
| *Amblyomma variegatum* | Kenya | 35.505 | 0.546 | " |
| *Amblyomma variegatum* | Kenya | 36.546538 | 0.522074 | " |
| *Amblyomma variegatum* | Kenya | 37.575703 | 0.57092 | " |
| *Amblyomma variegatum* | Kenya | 34.512894 | -0.455936 | " |
| *Amblyomma variegatum* | Kenya | 35.466 | -0.456 | " |
| *Amblyomma variegatum* | Kenya | 36.545 | -0.456 | " |
| *Amblyomma variegatum* | Kenya | 37.533736 | -0.411367 | " |
| *Amblyomma variegatum* | Kenya | 39.616 | -0.393 | " |
| *Amblyomma variegatum* | Kenya | 35.44078 | -1.44786 | " |
| *Amblyomma variegatum* | Kenya | 36.48324 | -1.431526 | " |
| *Amblyomma variegatum* | Kenya | 37.316121 | -1.468291 | " |
| *Amblyomma variegatum* | Kenya | 38.544931 | -1.288017 | " |
| *Amblyomma variegatum* | Kenya | 40.032714 | -1.396601 | " |
| *Amblyomma variegatum* | Kenya | 37.81663 | -2.491963 | " |
| *Amblyomma variegatum* | Kenya | 40.504039 | -2.368631 | " |
| *Amblyomma variegatum* | Kenya | 38.561772 | -3.445595 | " |
| *Amblyomma variegatum* | Kenya | 39.825681 | -3.437072 | " |
| *Amblyomma variegatum* | Kenya | 34.489762 | 3.981148 | (Cumming 1999) |
| *Amblyomma variegatum* | Kenya | 36.921 | 2.738 | " |
| *Amblyomma variegatum* | Kenya | 35.304372 | 1.74894 | " |
| *Amblyomma variegatum* | Kenya | 35.079132 | 1.358702 | " |
| *Amblyomma variegatum* | Kenya | 35.552326 | 1.327952 | " |
| *Amblyomma variegatum* | Kenya | 36.579825 | 1.138274 | " |
| *Amblyomma variegatum* | Kenya | 34.742 | 0.987 | " |
| *Amblyomma variegatum* | Kenya | 35.122 | 0.901 | " |
| *Amblyomma variegatum* | Kenya | 35.493 | 0.949 | " |
| *Amblyomma variegatum* | Kenya | 36.144158 | 0.842646 | " |
| *Amblyomma variegatum* | Kenya | 34.47149 | 0.846281 | " |
| *Amblyomma variegatum* | Kenya | 35.069468 | 0.632357 | " |
| *Amblyomma variegatum* | Kenya | 36.070029 | 0.614526 | " |
| *Amblyomma variegatum* | Kenya | 36.86003 | 0.974573 | " |
| *Amblyomma variegatum* | Kenya | 36.449281 | 0.168496 | " |
| *Amblyomma variegatum* | Kenya | 36.257361 | 0.243588 | " |
| *Amblyomma variegatum* | Kenya | 36.517442 | -0.091931 | " |
| *Amblyomma variegatum* | Kenya | 37.174987 | -0.040806 | " |
| *Amblyomma variegatum* | Kenya | 36.934495 | -0.366319 | " |
| *Amblyomma variegatum* | Kenya | 37.542953 | 0.360724 | " |
| *Amblyomma variegatum* | Kenya | 37.4123 | 0.14329 | " |
| *Amblyomma variegatum* | Kenya | 37.842261 | 0.191019 | " |
| *Amblyomma variegatum* | Kenya | 37.559 | -0.079 | " |
| *Amblyomma variegatum* | Kenya | 37.887172 | -0.32326 | " |
| *Amblyomma variegatum* | Kenya | 37.473 | -0.527 | " |
| *Amblyomma variegatum* | Kenya | 35.368343 | 0.160188 | " |
| *Amblyomma variegatum* | Kenya | 34.865 | 0.292 | " |
| *Amblyomma variegatum* | Kenya | 34.484311 | 0.87268 | " |
| *Amblyomma variegatum* | Kenya | 34.210272 | -0.006286 | " |
| *Amblyomma variegatum* | Kenya | 34.654952 | -0.006449 | " |
| *Amblyomma variegatum* | Kenya | 35.331624 | -0.037343 | " |
| *Amblyomma variegatum* | Kenya | 35.789 | -0.079 | " |
| *Amblyomma variegatum* | Kenya | 34.167419 | -0.554642 | " |
| *Amblyomma variegatum* | Kenya | 34.523 | -0.527 | " |
| *Amblyomma variegatum* | Kenya | 34.718266 | -0.471389 | " |
| *Amblyomma variegatum* | Kenya | 34.871622 | -0.857674 | " |
| *Amblyomma variegatum* | Kenya | 34.408473 | -0.935027 | " |
| *Amblyomma variegatum* | Kenya | 34.959327 | -1.208008 | " |
| *Amblyomma variegatum* | Kenya | 35.300324 | -1.127481 | " |
| *Amblyomma variegatum* | Kenya | 35.762931 | -0.868058 | " |
| *Amblyomma variegatum* | Kenya | 36.026 | -1.136 | " |
| *Amblyomma variegatum* | Kenya | 35.025838 | -1.316289 | " |
| *Amblyomma variegatum* | Kenya | 35.444961 | -1.387316 | " |
| *Amblyomma variegatum* | Kenya | 35.953278 | -1.380287 | " |
| *Amblyomma variegatum* | Kenya | 35.573862 | -1.641246 | " |
| *Amblyomma variegatum* | Kenya | 35.931 | -1.773 | " |
| *Amblyomma variegatum* | Kenya | 36.436 | -0.917 | " |
| *Amblyomma variegatum* | Kenya | 36.197588 | -0.942924 | " |
| *Amblyomma variegatum* | Kenya | 36.664882 | -1.11997 | " |
| *Amblyomma variegatum* | Kenya | 36.969 | -1.002 | " |
| *Amblyomma variegatum* | Kenya | 37.469894 | -0.985055 | " |
| *Amblyomma variegatum* | Kenya | 38.014997 | -1.106913 | " |
| *Amblyomma variegatum* | Kenya | 38.111 | -1.221 | " |
| *Amblyomma variegatum* | Kenya | 38.066425 | -1.428945 | " |
| *Amblyomma variegatum* | Kenya | 36.632974 | -1.429493 | " |
| *Amblyomma variegatum* | Kenya | 37.058053 | -1.475006 | " |
| *Amblyomma variegatum* | Kenya | 37.519377 | -1.655187 | " |
| *Amblyomma variegatum* | Kenya | 36.759299 | -2.125307 | " |
| *Amblyomma variegatum* | Kenya | 37.014304 | -1.879977 | " |
| *Amblyomma variegatum* | Kenya | 37.386872 | -2.090149 | " |
| *Amblyomma variegatum* | Kenya | 37.575618 | -2.037735 | " |
| *Amblyomma variegatum* | Kenya | 37.897363 | -2.904138 | " |
| *Amblyomma variegatum* | Kenya | 37.723084 | -3.476276 | " |
| *Amblyomma variegatum* | Kenya | 38.568087 | -3.54347 | " |
| *Amblyomma variegatum* | Kenya | 38.498954 | -3.42198 | " |
| *Amblyomma variegatum* | Kenya | 38.287217 | -3.363467 | " |
| *Amblyomma variegatum* | Kenya | 39.717703 | -3.2502 | " |
| *Amblyomma variegatum* | Kenya | 39.817604 | -3.651405 | " |
| *Amblyomma variegatum* | Kenya | 39.38899 | -3.709484 | " |
| *Amblyomma variegatum* | Kenya | 39.454606 | -4.093602 | " |
| *Amblyomma variegatum* | Kenya | 39.109819 | -4.216849 | " |
| *Amblyomma variegatum* | Kenya | 39.359812 | -4.433119 | " |
| *Amblyomma variegatum* | Somalia | 43.468 | 11.234 | (Pegram 1976) |
| *Amblyomma variegatum* | Somalia | 43.525 | 10.949 | " |
| *Amblyomma variegatum* | Somalia | 43.087 | 10.202 | " |
| *Amblyomma variegatum* | Somalia | 43.321 | 9.854 | " |
| *Amblyomma variegatum* | Somalia | 43.53 | 9.892 | " |
| *Amblyomma variegatum* | Somalia | 43.335 | 9.654 | " |
| *Amblyomma variegatum* | Somalia | 43.601 | 9.721 | " |
| *Amblyomma variegatum* | Somalia | 43.511 | 9.554 | " |
| *Amblyomma variegatum* | Somalia | 43.706 | 9.545 | " |
| *Amblyomma variegatum* | Somalia | 43.949 | 9.597 | " |
| *Amblyomma variegatum* | Somalia | 44.215 | 9.54 | " |
| *Amblyomma variegatum* | Somalia | 43.73 | 9.355 | " |
| *Amblyomma variegatum* | Somalia | 44.063 | 9.383 | " |
| *Amblyomma variegatum* | Somalia | 44.182 | 9.121 | " |
| *Amblyomma variegatum* | Tanzania | 34.120076 | -1.176514 | (Isack et al., 2017) |
| *Amblyomma variegatum* | Tanzania | 34.001573 | -1.567698 | " |
| *Amblyomma variegatum* | Tanzania | 34.332024 | -1.283802 | " |
| *Amblyomma variegatum* | Tanzania | 34.567122 | -1.437009 | " |
| *Amblyomma variegatum* | Tanzania | 34.538715 | -1.646068 | " |
| *Amblyomma variegatum* | Tanzania | 34.452205 | -2.011897 | " |
| *Amblyomma variegatum* | Tanzania | 35.041135 | -1.903099 | " |
| *Amblyomma variegatum* | Tanzania | 34.872895 | -2.278013 | " |
| *Amblyomma variegatum* | Tanzania | 34.666219 | -4.012861 | " |
| *Amblyomma variegatum* | Tanzania | 34.761051 | -4.126501 | " |
| *Amblyomma variegatum* | Tanzania | 34.578555 | -4.257569 | " |
| *Amblyomma variegatum* | Tanzania | 34.657 | -4.356 | " |
| *Amblyomma variegatum* | Tanzania | 34.324519 | -4.256396 | " |
| *Amblyomma variegatum* | Tanzania | 34.418759 | -4.107199 | " |
| *Amblyomma variegatum* | Tanzania | 34.308 | -4.594 | " |
| *Amblyomma variegatum* | Tanzania | 34.211978 | -4.635598 | " |
| *Amblyomma variegatum* | Tanzania | 33.772 | -8.283 | " |
| *Amblyomma variegatum* | Tanzania | 33.814846 | -8.488892 | " |
| *Amblyomma variegatum* | Tanzania | 34.129 | -8.608 | " |
| *Amblyomma variegatum* | Tanzania | 34.288 | -8.417 | " |
| *Amblyomma variegatum* | Tanzania | 32.352891 | -8.437648 | " |
| *Amblyomma variegatum* | Tanzania | 32.327171 | -8.645651 | " |
| *Amblyomma variegatum* | Tanzania | 32.480046 | -8.711155 | " |
| *Amblyomma variegatum* | Tanzania | 32.557891 | -8.566648 | " |
| *Amblyomma variegatum* | Uganda | 32.315556 | 3.030059 | (Kaiser et al., 1991) |
| *Amblyomma variegatum* | Uganda | 34.687209 | 2.528039 | (Akure 2019) |
| *Amblyomma variegatum* | Uganda | 34.696837 | 2.56191 | " |
| *Amblyomma variegatum* | Uganda | 34.104411 | 3.06883 | " |
| *Amblyomma variegatum* | Uganda | 34.200103 | 3.00698 | " |
| *Haemaphysalis intermedia* | India | 76.538619 | 29.351063 | (Geevarghese et al., 2011) |
| *Haemaphysalis intermedia* | India | 76.862888 | 31.850894 | " |
| *Haemaphysalis intermedia* | India | 75.467496 | 33.327085 | " |
| *Haemaphysalis intermedia* | India | 81.926851 | 17.60714 | " |
| *Haemaphysalis intermedia* | India | 78.810918 | 15.621322 | " |
| *Haemaphysalis intermedia* | India | 84.756306 | 26.912443 | " |
| *Haemaphysalis intermedia* | India | 92.40892 | 25.891358 | " |
| *Haemaphysalis intermedia* | India | 77.019058 | 28.821284 | " |
| *Haemaphysalis intermedia* | India | 76.93683 | 28.520366 | " |
| *Haemaphysalis intermedia* | India | 74.205742 | 15.435895 | " |
| *Haemaphysalis intermedia* | India | 75.815561 | 30.460335 | " |
| *Haemaphysalis intermedia* | India | 73.577289 | 17.827633 | " |
| *Haemaphysalis intermedia* | India | 80.239746 | 22.36605 | " |
| *Haemaphysalis intermedia* | India | 75.597814 | 13.439496 | " |
| *Haemaphysalis intermedia* | India | 83.959915 | 20.042652 | " |
| *Haemaphysalis intermedia* | India | 78.818179 | 12.571848 | " |
| *Haemaphysalis intermedia* | India | 78.82924 | 10.788827 | (Ghosh et al., 2006) |
| *Haemaphysalis intermedia* | India | 78.93972 | 18.756938 | " |
| *Haemaphysalis intermedia* | India | 75.407656 | 13.867363 | " |
| *Haemaphysalis intermedia* | India | 76.275969 | 18.596422 | " |
| *Haemaphysalis intermedia* | India | 86.627103 | 21.093295 | " |
| *Haemaphysalis intermedia* | India | 77.709835 | 24.436045 | " |
| *Haemaphysalis intermedia* | India | 75.977603 | 30.722694 | " |
| *Haemaphysalis intermedia* | India | 77.459437 | 31.777949 | " |
| *Haemaphysalis intermedia* | India | 75.740654 | 33.438045 | " |
| *Haemaphysalis intermedia* | India | 86.826712 | 26.351703 | " |
| *Haemaphysalis intermedia* | India | 77.090205 | 28.781226 | " |
| *Haemaphysalis intermedia* | India | 26.313817 | 92.857179 | " |
| *Haemaphysalis intermedia* | India | 30.478076 | 77.089795 | (Miranpuri et al., 1975) |
| *Haemaphysalis intermedia* | India | 32.637543 | 76.226775 | " |
| *Haemaphysalis intermedia* | India | 31.962629 | 77.105431 | " |
| *Haemaphysalis intermedia* | India | 32.100076 | 76.266481 | " |
| *Haemaphysalis intermedia* | India | 34.076986 | 74.859197 | " |
| *Haemaphysalis intermedia* | India | 32.056974 | 75.426633 | " |
| *Haemaphysalis intermedia* | India | 31.625918 | 75.95501 | " |
| *Haemaphysalis intermedia* | India | 30.984667 | 76.528194 | " |
| *Haemaphysalis intermedia* | India | 12.590514 | 92.859895 | (Ghosh et al., 2007) |
| *Haemaphysalis intermedia* | India | 7.977527 | 93.374068 | " |
| *Haemaphysalis intermedia* | India | 15.502569 | 78.631129 | " |
| *Haemaphysalis intermedia* | India | 26.371163 | 93.161965 | " |
| *Haemaphysalis intermedia* | India | 26.607398 | 85.190502 | " |
| *Haemaphysalis intermedia* | India | 22.727586 | 82.897532 | " |
| *Haemaphysalis intermedia* | India | 29.685013 | 76.407875 | " |
| *Haemaphysalis intermedia* | India | 32.396928 | 76.135584 | " |
| *Haemaphysalis intermedia* | India | 15.185255 | 74.521154 | " |
| *Haemaphysalis intermedia* | India | 22.373007 | 80.627267 | " |
| *Haemaphysalis intermedia* | India | 20.268537 | 79.331825 | " |
| *Haemaphysalis intermedia* | India | 21.19428 | 84.256546 | " |
| *Haemaphysalis intermedia* | India | 31.073567 | 76.44965 | " |
| *Haemaphysalis intermedia* | India | 10.364366 | 77.647461 | " |
| *Haemaphysalis intermedia* | India | 13.944195 | 75.588058 | (Sadanandane et al., 2018) |
| *Haemaphysalis intermedia* | India | 11.875711 | 77.17432 | " |
| *Haemaphysalis intermedia* | India | 11.61998 | 75.955323 | " |
| *Haemaphysalis intermedia* | India | 11.368497 | 77.71505 | " |
| *Haemaphysalis intermedia* | Sri Lanka | 6.053458 | 80.232961 | (Diyes et al., 2015) |
| *Haemaphysalis intermedia* | Sri Lanka | 5.961211 | 80.575466 | " |
| *Haemaphysalis intermedia* | Sri Lanka | 6.719161 | 80.347308 | " |
| *Haemaphysalis intermedia* | Sri Lanka | 7.27956 | 80.641573 | " |
| *Haemaphysalis intermedia* | Sri Lanka | 6.597679 | 79.992537 | " |
| *Haemaphysalis intermedia* | Sri Lanka | 6.907749 | 79.878868 | " |
| *Haemaphysalis intermedia* | Sri Lanka | 7.080942 | 79.9896 | " |
| *Haemaphysalis intermedia* | Sri Lanka | 6.965337 | 80.77717 | " |
| *Haemaphysalis intermedia* | Sri Lanka | 7.49049 | 80.372199 | " |
| *Haemaphysalis intermedia* | Sri Lanka | 8.055551 | 79.832395 | " |
| *Haemaphysalis intermedia* | Sri Lanka | 6.163764 | 81.116147 | " |
| *Haemaphysalis intermedia* | Sri Lanka | 7.462336 | 80.634541 | " |
| *Haemaphysalis intermedia* | Sri Lanka | 6.97516 | 81.053524 | " |
| *Haemaphysalis intermedia* | Sri Lanka | 7.094818 | 81.359811 | " |
| *Haemaphysalis intermedia* | Sri Lanka | 9.348818 | 80.368021 | " |
| *Haemaphysalis intermedia* | Sri Lanka | 9.686033 | 80.01023 | " |
| *Haemaphysalis intermedia* | Sri Lanka | 8.347027 | 80.362638 | " |
| *Haemaphysalis intermedia* | Sri Lanka | 7.30219 | 81.657461 | " |
| *Haemaphysalis intermedia* | Sri Lanka | 7.739941 | 81.692827 | " |
| *Haemaphysalis intermedia* | Sri Lanka | 7.953833 | 81.005968 | " |
| *Haemaphysalis intermedia* | Sri Lanka | 8.717911 | 80.466345 | " |
| *Haemaphysalis intermedia* | Sri Lanka | 6.168543 | 81.143613 | (Bandaranayaka et al., 2022) |
| *Haemaphysalis intermedia* | Sri Lanka | 7.290185 | 81.648534 | " |
| *Haemaphysalis intermedia* | Sri Lanka | 8.550807 | 81.235232 | " |
| *Haemaphysalis intermedia* | Sri Lanka | 9.385572 | 80.413854 | " |
| *Haemaphysalis intermedia* | Sri Lanka | 8.306186 | 80.350439 | " |
| *Haemaphysalis intermedia* | Sri Lanka | 8.048837 | 79.83791 | " |
| *Haemaphysalis intermedia* | Sri Lanka | 7.747627 | 81.686369 | " |
| *Haemaphysalis intermedia* | Sri Lanka | 9.66708 | 80.042074 | " |
| *Haemaphysalis intermedia* | Sri Lanka | 6.626993 | 81.317936 | " |
| *Haemaphysalis intermedia* | Sri Lanka | 7.955364 | 81.005796 | " |
| *Haemaphysalis intermedia* | Sri Lanka | 9.001958 | 79.879316 | " |
| *Haemaphysalis intermedia* | Sri Lanka | 9.262446 | 80.770515 | " |
| *Haemaphysalis intermedia* | Sri Lanka | 6.987998 | 80.757752 | " |
| *Haemaphysalis intermedia* | Sri Lanka | 6.72223 | 80.34748 | " |
| *Haemaphysalis intermedia* | Sri Lanka | 6.973121 | 79.934462 | " |
| *Haemaphysalis intermedia* | Sri Lanka | 7.271897 | 80.64226 | " |
| *Haemaphysalis intermedia* | Sri Lanka | 7.251091 | 80.352073 | " |
| *Haemaphysalis intermedia* | Sri Lanka | 5.957444 | 80.566336 | " |
| *Haemaphysalis intermedia* | Sri Lanka | 6.075756 | 80.219184 | " |
| *Haemaphysalis intermedia* | Sri Lanka | 7.064045 | 80.03789 | " |
| *Haemaphysalis intermedia* | Sri Lanka | 6.594812 | 79.992194 | " |
| *Haemaphysalis intermedia* | Sri Lanka | 7.510573 | 80.369109 | " |
| *Haemaphysalis intermedia* | Sri Lanka | 6.976949 | 81.054479 | " |
| *Haemaphysalis intermedia* | Sri Lanka | 7.445059 | 80.637631 | " |
| *Haemaphysalis intermedia* | Sri Lanka | 7.171612 | 80.50262 | " |
| *Haemaphysalis intermedia* | Sri Lanka | 7.299312 | 80.506153 | " |
| *Haemaphysalis intermedia* | Sri Lanka | 7.467941 | 81.014843 | " |
| *Haemaphysalis intermedia* | Sri Lanka | 6.163209 | 81.113003 | " |
| *Haemaphysalis wellingtoni* | China | 99.391872 | 23.555417 | (DeSheng 2003) |
| *Haemaphysalis wellingtoni* | China | 101.568655 | 21.400144 | " |
| *Haemaphysalis wellingtoni* | China | 116.662613 | 26.273431 | (Liang 1996) |
| *Haemaphysalis wellingtoni* | China | 116.690079 | 26.579977 | (ZhenGuang 1999) |
| *Haemaphysalis wellingtoni* | China | 116.714665 | 26.391352 | " |
| *Haemaphysalis wellingtoni* | China | 116.963879 | 26.782433 | " |
| *Haemaphysalis wellingtoni* | China | 116.643077 | 26.484808 | (ZhenGuang 1999) |
| *Haemaphysalis wellingtoni* | China | 116.565551 | 26.341292 | " |
| *Haemaphysalis wellingtoni* | China | 121.662501 | 24.175983 | (Kuo et al., 2017) |
| *Haemaphysalis wellingtoni* | China | 121.526892 | 23.904576 | " |
| *Haemaphysalis wellingtoni* | China | 121.58309 | 24.169917 | " |
| *Haemaphysalis wellingtoni* | China | 121.505545 | 24.21831 | " |
| *Haemaphysalis wellingtoni* | China | 121.476641 | 24.21573 | " |
| *Haemaphysalis wellingtoni* | China | 121.446189 | 24.236232 | " |
| *Haemaphysalis wellingtoni* | China | 121.294641 | 24.145703 | " |
| *Haemaphysalis wellingtoni* | China | 118.396854 | 24.456264 | " |
| *Haemaphysalis wellingtoni* | India | 76.536629 | 13.226608 | (Geevarghese et al., 2011) |
| *Haemaphysalis wellingtoni* | India | 92.842161 | 12.558102 | " |
| *Haemaphysalis wellingtoni* | India | 93.373654 | 7.95497 | " |
| *Haemaphysalis wellingtoni* | India | 84.499227 | 21.331402 | " |
| *Haemaphysalis wellingtoni* | India | 92.891806 | 26.31904 | " |
| *Haemaphysalis wellingtoni* | India | 88.301666 | 23.401046 | " |
| *Haemaphysalis wellingtoni* | India | 76.183289 | 11.738328 | (Sadanandane et al., 2018) |
| *Haemaphysalis wellingtoni* | India | 75.075177 | 14.250245 | (Rajagopalan 1972) |
| *Rhipicephalus appendiculatus* | Kenya | 37.695426 | -3.054181 | (Kirung'o 2012) |
| *Rhipicephalus appendiculatus* | Kenya | 36.771378 | -2.482534 | " |
| *Rhipicephalus appendiculatus* | Kenya | 37.828695 | -2.787759 | " |
| *Rhipicephalus appendiculatus* | Kenya | 37.902236 | -2.592849 | " |
| *Rhipicephalus appendiculatus* | Kenya | 38.045262 | -2.375419 | " |
| *Rhipicephalus appendiculatus* | Kenya | 38.173065 | -2.026162 | " |
| *Rhipicephalus appendiculatus* | Kenya | 37.38253 | -2.139125 | " |
| *Rhipicephalus appendiculatus* | Kenya | 37.467781 | -1.9835 | " |
| *Rhipicephalus appendiculatus* | Kenya | 37.478193 | -1.958871 | " |
| *Rhipicephalus appendiculatus* | Kenya | 37.29445 | -1.970581 | " |
| *Rhipicephalus appendiculatus* | Kenya | 37.367172 | -1.88325 | " |
| *Rhipicephalus appendiculatus* | Kenya | 37.342184 | -1.804805 | " |
| *Rhipicephalus appendiculatus* | Kenya | 37.468149 | -1.810185 | " |
| *Rhipicephalus appendiculatus* | Kenya | 37.519909 | -1.817475 | " |
| *Rhipicephalus appendiculatus* | Kenya | 37.531559 | -1.894512 | " |
| *Rhipicephalus appendiculatus* | Kenya | 37.611247 | -1.821864 | " |
| *Rhipicephalus appendiculatus* | Kenya | 37.42632 | -1.680252 | " |
| *Rhipicephalus appendiculatus* | Kenya | 37.432701 | -1.641163 | " |
| *Rhipicephalus appendiculatus* | Kenya | 37.474294 | -1.591197 | " |
| *Rhipicephalus appendiculatus* | Kenya | 37.646345 | -1.674808 | " |
| *Rhipicephalus appendiculatus* | Kenya | 38.159022 | -1.601997 | " |
| *Rhipicephalus appendiculatus* | Kenya | 38.118131 | -1.485133 | " |
| *Rhipicephalus appendiculatus* | Kenya | 38.053029 | -1.338976 | " |
| *Rhipicephalus appendiculatus* | Kenya | 38.128765 | -1.261838 | " |
| *Rhipicephalus appendiculatus* | Kenya | 38.168125 | -1.2877 | " |
| *Rhipicephalus appendiculatus* | Kenya | 37.949237 | -1.33008 | " |
| *Rhipicephalus appendiculatus* | Kenya | 38.004771 | -1.15551 | " |
| *Rhipicephalus appendiculatus* | Kenya | 38.265806 | -1.022334 | " |
| *Rhipicephalus appendiculatus* | Kenya | 38.322762 | -1.030722 | " |
| *Rhipicephalus appendiculatus* | Kenya | 38.393132 | -1.108425 | " |
| *Rhipicephalus appendiculatus* | Kenya | 38.166085 | -0.788028 | " |
| *Rhipicephalus appendiculatus* | Kenya | 37.285331 | -1.677654 | " |
| *Rhipicephalus appendiculatus* | Kenya | 37.273096 | -1.551461 | " |
| *Rhipicephalus appendiculatus* | Kenya | 37.286236 | -1.501396 | " |
| *Rhipicephalus appendiculatus* | Kenya | 37.282904 | -1.46064 | " |
| *Rhipicephalus appendiculatus* | Kenya | 37.406704 | -1.439389 | " |
| *Rhipicephalus appendiculatus* | Kenya | 37.690681 | -1.494171 | " |
| *Rhipicephalus appendiculatus* | Kenya | 36.973406 | -1.535884 | " |
| *Rhipicephalus appendiculatus* | Kenya | 37.004768 | -1.477393 | " |
| *Rhipicephalus appendiculatus* | Kenya | 37.063479 | -1.338218 | " |
| *Rhipicephalus appendiculatus* | Kenya | 37.014652 | -1.349106 | " |
| *Rhipicephalus appendiculatus* | Kenya | 37.227414 | -1.141929 | " |
| *Rhipicephalus appendiculatus* | Kenya | 37.258824 | -1.11948 | " |
| *Rhipicephalus appendiculatus* | Kenya | 37.377819 | -1.139901 | " |
| *Rhipicephalus appendiculatus* | Kenya | 37.529683 | -1.138622 | " |
| *Rhipicephalus appendiculatus* | Kenya | 37.531297 | -1.227849 | " |
| *Rhipicephalus appendiculatus* | Kenya | 37.289364 | -0.824059 | " |
| *Rhipicephalus appendiculatus* | Kenya | 37.293842 | -0.855209 | " |
| *Rhipicephalus appendiculatus* | Kenya | 37.294701 | -0.894599 | " |
| *Rhipicephalus appendiculatus* | Kenya | 37.330802 | -0.882503 | " |
| *Rhipicephalus appendiculatus* | Kenya | 37.377497 | -0.868922 | " |
| *Rhipicephalus appendiculatus* | Kenya | 37.527438 | -0.950954 | " |
| *Rhipicephalus appendiculatus* | Kenya | 37.666708 | -0.85065 | " |
| *Rhipicephalus appendiculatus* | Kenya | 37.666685 | -0.88185 | " |
| *Rhipicephalus appendiculatus* | Kenya | 37.140591 | -1.88736 | " |
| *Rhipicephalus appendiculatus* | Kenya | 37.070331 | -1.906757 | " |
| *Rhipicephalus appendiculatus* | Kenya | 36.802396 | -1.885089 | " |
| *Rhipicephalus appendiculatus* | Kenya | 36.865666 | -1.681494 | " |
| *Rhipicephalus appendiculatus* | Kenya | 36.8383 | -1.506223 | " |
| *Rhipicephalus appendiculatus* | Kenya | 36.659527 | -1.627877 | " |
| *Rhipicephalus appendiculatus* | Kenya | 36.646106 | -1.530217 | " |
| *Rhipicephalus appendiculatus* | Kenya | 36.626689 | -1.426244 | " |
| *Rhipicephalus appendiculatus* | Kenya | 36.645978 | -1.396157 | " |
| *Rhipicephalus appendiculatus* | Kenya | 36.644292 | -1.312424 | " |
| *Rhipicephalus appendiculatus* | Kenya | 36.511391 | -1.318128 | " |
| *Rhipicephalus appendiculatus* | Kenya | 36.506362 | -1.401348 | " |
| *Rhipicephalus appendiculatus* | Kenya | 36.514221 | -1.485584 | " |
| *Rhipicephalus appendiculatus* | Kenya | 36.450521 | -1.225854 | " |
| *Rhipicephalus appendiculatus* | Kenya | 36.071295 | -1.483498 | " |
| *Rhipicephalus appendiculatus* | Kenya | 36.139356 | -1.565489 | " |
| *Rhipicephalus appendiculatus* | Kenya | 36.077522 | -1.900404 | " |
| *Rhipicephalus appendiculatus* | Kenya | 35.777217 | -1.891097 | " |
| *Rhipicephalus appendiculatus* | Kenya | 35.779454 | -1.582212 | " |
| *Rhipicephalus appendiculatus* | Kenya | 35.480666 | -1.777544 | " |
| *Rhipicephalus appendiculatus* | Kenya | 35.477567 | -1.706442 | " |
| *Rhipicephalus appendiculatus* | Kenya | 35.408644 | -1.601555 | " |
| *Rhipicephalus appendiculatus* | Kenya | 35.551037 | -1.530701 | " |
| *Rhipicephalus appendiculatus* | Kenya | 35.764821 | -1.38931 | " |
| *Rhipicephalus appendiculatus* | Kenya | 36.293469 | -1.119973 | " |
| *Rhipicephalus appendiculatus* | Kenya | 36.202994 | -1.206334 | " |
| *Rhipicephalus appendiculatus* | Kenya | 36.191878 | -0.970838 | " |
| *Rhipicephalus appendiculatus* | Kenya | 35.910082 | -0.746073 | " |
| *Rhipicephalus appendiculatus* | Kenya | 35.827513 | -0.766391 | " |
| *Rhipicephalus appendiculatus* | Kenya | 35.871298 | -0.780218 | " |
| *Rhipicephalus appendiculatus* | Kenya | 35.958884 | -0.908432 | " |
| *Rhipicephalus appendiculatus* | Kenya | 35.929821 | -0.97761 | " |
| *Rhipicephalus appendiculatus* | Kenya | 35.819847 | -0.954487 | " |
| *Rhipicephalus appendiculatus* | Kenya | 35.879878 | -0.948518 | " |
| *Rhipicephalus appendiculatus* | Kenya | 35.876206 | -0.968837 | " |
| *Rhipicephalus appendiculatus* | Kenya | 35.944242 | -1.021388 | " |
| *Rhipicephalus appendiculatus* | Kenya | 35.854991 | -1.043592 | " |
| *Rhipicephalus appendiculatus* | Kenya | 35.708714 | -0.947889 | " |
| *Rhipicephalus appendiculatus* | Kenya | 35.690149 | -1.025225 | " |
| *Rhipicephalus appendiculatus* | Kenya | 35.497118 | -1.006467 | " |
| *Rhipicephalus appendiculatus* | Kenya | 35.471511 | -1.049798 | " |
| *Rhipicephalus appendiculatus* | Kenya | 35.439232 | -1.096956 | " |
| *Rhipicephalus appendiculatus* | Kenya | 35.382552 | -1.08828 | " |
| *Rhipicephalus appendiculatus* | Kenya | 35.418617 | -1.121673 | " |
| *Rhipicephalus appendiculatus* | Kenya | 35.461921 | -1.21244 | " |
| *Rhipicephalus appendiculatus* | Kenya | 35.435217 | -1.232586 | " |
| *Rhipicephalus appendiculatus* | Kenya | 35.368819 | -1.198815 | " |
| *Rhipicephalus appendiculatus* | Kenya | 35.339458 | -1.213451 | " |
| *Rhipicephalus appendiculatus* | Kenya | 35.355408 | -1.221699 | " |
| *Rhipicephalus appendiculatus* | Kenya | 35.316588 | -1.258364 | " |
| *Rhipicephalus appendiculatus* | Kenya | 35.371861 | -1.266471 | " |
| *Rhipicephalus appendiculatus* | Kenya | 35.259348 | -1.563483 | " |
| *Rhipicephalus appendiculatus* | Kenya | 35.015668 | -1.316281 | " |
| *Rhipicephalus appendiculatus* | Kenya | 34.818802 | -1.384459 | " |
| *Rhipicephalus appendiculatus* | Kenya | 34.732323 | -1.338682 | " |
| *Rhipicephalus appendiculatus* | Kenya | 34.781661 | -1.179133 | " |
| *Rhipicephalus appendiculatus* | Kenya | 34.881368 | -1.208635 | " |
| *Rhipicephalus appendiculatus* | Kenya | 34.962986 | -1.212849 | " |
| *Rhipicephalus appendiculatus* | Kenya | 34.947211 | -1.153913 | " |
| *Rhipicephalus appendiculatus* | Kenya | 34.986563 | -1.110901 | " |
| *Rhipicephalus appendiculatus* | Kenya | 35.061694 | -1.135423 | " |
| *Rhipicephalus appendiculatus* | Kenya | 35.101109 | -1.135428 | " |
| *Rhipicephalus appendiculatus* | Kenya | 35.163782 | -1.112412 | " |
| *Rhipicephalus appendiculatus* | Kenya | 35.157846 | -1.140556 | " |
| *Rhipicephalus appendiculatus* | Kenya | 35.162381 | -1.165983 | " |
| *Rhipicephalus appendiculatus* | Kenya | 35.270718 | -1.03744 | " |
| *Rhipicephalus appendiculatus* | Kenya | 35.254665 | -1.011143 | " |
| *Rhipicephalus appendiculatus* | Kenya | 35.295791 | -0.896211 | " |
| *Rhipicephalus appendiculatus* | Kenya | 35.10982 | -0.78632 | " |
| *Rhipicephalus appendiculatus* | Kenya | 35.094592 | -0.877692 | " |
| *Rhipicephalus appendiculatus* | Kenya | 35.072282 | -0.910898 | " |
| *Rhipicephalus appendiculatus* | Kenya | 35.109691 | -0.994375 | " |
| *Rhipicephalus appendiculatus* | Kenya | 35.137507 | -1.086716 | " |
| *Rhipicephalus appendiculatus* | Kenya | 35.048803 | -1.01199 | " |
| *Rhipicephalus appendiculatus* | Kenya | 35.046559 | -1.031533 | " |
| *Rhipicephalus appendiculatus* | Kenya | 34.951021 | -0.944229 | " |
| *Rhipicephalus appendiculatus* | Kenya | 34.986711 | -0.998314 | " |
| *Rhipicephalus appendiculatus* | Kenya | 34.788354 | -0.977974 | " |
| *Rhipicephalus appendiculatus* | Kenya | 34.803866 | -1.036894 | " |
| *Rhipicephalus appendiculatus* | Kenya | 34.776117 | -1.041396 | " |
| *Rhipicephalus appendiculatus* | Kenya | 34.670496 | -1.027417 | " |
| *Rhipicephalus appendiculatus* | Kenya | 34.683372 | -1.053198 | " |
| *Rhipicephalus appendiculatus* | Kenya | 34.641484 | -1.066835 | " |
| *Rhipicephalus appendiculatus* | Kenya | 35.43058 | -0.41859 | " |
| *Rhipicephalus appendiculatus* | Kenya | 35.328575 | -0.42915 | " |
| *Rhipicephalus appendiculatus* | Kenya | 35.252398 | -0.44494 | " |
| *Rhipicephalus appendiculatus* | Kenya | 35.540655 | -0.349515 | " |
| *Rhipicephalus appendiculatus* | Kenya | 35.487796 | -0.284691 | " |
| *Rhipicephalus appendiculatus* | Kenya | 35.450301 | -0.256019 | " |
| *Rhipicephalus appendiculatus* | Kenya | 35.369103 | -0.310027 | " |
| *Rhipicephalus appendiculatus* | Kenya | 35.227303 | -0.255132 | " |
| *Rhipicephalus appendiculatus* | Kenya | 35.228826 | -0.291051 | " |
| *Rhipicephalus appendiculatus* | Kenya | 35.200703 | -0.314074 | " |
| *Rhipicephalus appendiculatus* | Kenya | 35.131642 | -0.229996 | " |
| *Rhipicephalus appendiculatus* | Kenya | 35.148956 | -0.260006 | " |
| *Rhipicephalus appendiculatus* | Kenya | 35.130194 | -0.296595 | " |
| *Rhipicephalus appendiculatus* | Kenya | 35.666062 | -0.226974 | " |
| *Rhipicephalus appendiculatus* | Kenya | 35.696685 | -0.128062 | " |
| *Rhipicephalus appendiculatus* | Kenya | 35.638934 | -0.135964 | " |
| *Rhipicephalus appendiculatus* | Kenya | 35.639145 | -0.073295 | " |
| *Rhipicephalus appendiculatus* | Kenya | 35.597929 | 0.03283 | " |
| *Rhipicephalus appendiculatus* | Kenya | 35.493832 | -0.034489 | " |
| *Rhipicephalus appendiculatus* | Kenya | 35.288723 | -0.07023 | " |
| *Rhipicephalus appendiculatus* | Kenya | 35.344697 | -0.020311 | " |
| *Rhipicephalus appendiculatus* | Kenya | 35.285769 | -0.012992 | " |
| *Rhipicephalus appendiculatus* | Kenya | 35.22033 | 0.11962 | " |
| *Rhipicephalus appendiculatus* | Kenya | 35.16639 | 0.2164 | " |
| *Rhipicephalus appendiculatus* | Kenya | 35.010267 | 0.240126 | " |
| *Rhipicephalus appendiculatus* | Kenya | 34.939273 | 0.502473 | " |
| *Rhipicephalus appendiculatus* | Kenya | 35.515463 | 0.168253 | " |
| *Rhipicephalus appendiculatus* | Kenya | 35.555346 | 0.223758 | " |
| *Rhipicephalus appendiculatus* | Kenya | 35.443861 | 0.231695 | " |
| *Rhipicephalus appendiculatus* | Kenya | 35.480947 | 0.238442 | " |
| *Rhipicephalus appendiculatus* | Kenya | 35.689294 | 0.260948 | " |
| *Rhipicephalus appendiculatus* | Kenya | 35.487882 | 0.395287 | " |
| *Rhipicephalus appendiculatus* | Kenya | 35.533553 | 0.454302 | " |
| *Rhipicephalus appendiculatus* | Kenya | 35.57353 | 0.464695 | " |
| *Rhipicephalus appendiculatus* | Kenya | 35.199251 | 0.449662 | " |
| *Rhipicephalus appendiculatus* | Kenya | 35.352463 | 0.413906 | " |
| *Rhipicephalus appendiculatus* | Kenya | 35.397403 | 0.565805 | " |
| *Rhipicephalus appendiculatus* | Kenya | 35.503004 | 0.617355 | " |
| *Rhipicephalus appendiculatus* | Kenya | 35.330328 | 0.651455 | " |
| *Rhipicephalus appendiculatus* | Kenya | 35.465826 | 0.652694 | " |
| *Rhipicephalus appendiculatus* | Kenya | 35.412675 | 0.700523 | " |
| *Rhipicephalus appendiculatus* | Kenya | 35.405482 | 0.733722 | " |
| *Rhipicephalus appendiculatus* | Kenya | 35.521494 | 0.708076 | " |
| *Rhipicephalus appendiculatus* | Kenya | 35.532733 | 0.817748 | " |
| *Rhipicephalus appendiculatus* | Kenya | 35.527867 | 0.866705 | " |
| *Rhipicephalus appendiculatus* | Kenya | 35.58839 | 0.909412 | " |
| *Rhipicephalus appendiculatus* | Kenya | 35.497122 | 0.896407 | " |
| *Rhipicephalus appendiculatus* | Kenya | 35.520709 | 0.883083 | " |
| *Rhipicephalus appendiculatus* | Kenya | 35.506637 | 0.856631 | " |
| *Rhipicephalus appendiculatus* | Kenya | 35.518968 | 0.834956 | " |
| *Rhipicephalus appendiculatus* | Kenya | 35.293949 | 0.674647 | " |
| *Rhipicephalus appendiculatus* | Kenya | 35.263162 | 0.794095 | " |
| *Rhipicephalus appendiculatus* | Kenya | 35.308382 | 0.882811 | " |
| *Rhipicephalus appendiculatus* | Kenya | 35.32428 | 0.857687 | " |
| *Rhipicephalus appendiculatus* | Kenya | 35.497707 | 1.025895 | " |
| *Rhipicephalus appendiculatus* | Kenya | 35.589599 | 1.132647 | " |
| *Rhipicephalus appendiculatus* | Kenya | 35.566832 | 1.232997 | " |
| *Rhipicephalus appendiculatus* | Kenya | 35.326503 | 1.110583 | " |
| *Rhipicephalus appendiculatus* | Kenya | 35.203239 | 1.120527 | " |
| *Rhipicephalus appendiculatus* | Kenya | 35.307813 | 1.0101 | " |
| *Rhipicephalus appendiculatus* | Kenya | 34.848861 | 0.855824 | " |
| *Rhipicephalus appendiculatus* | Kenya | 34.756601 | 0.990079 | " |
| *Rhipicephalus appendiculatus* | Kenya | 34.878131 | 1.022465 | " |
| *Rhipicephalus appendiculatus* | Kenya | 34.841371 | 1.07284 | " |
| *Rhipicephalus appendiculatus* | Kenya | 34.892744 | 1.064527 | " |
| *Rhipicephalus appendiculatus* | Kenya | 35.003738 | 1.0564 | " |
| *Rhipicephalus appendiculatus* | Kenya | 35.038334 | 1.248967 | " |
| *Rhipicephalus appendiculatus* | Kenya | 35.044694 | 1.156203 | " |
| *Rhipicephalus appendiculatus* | Kenya | 35.091012 | 1.137487 | " |
| *Rhipicephalus appendiculatus* | Kenya | 35.134433 | 1.182586 | " |
| *Rhipicephalus appendiculatus* | Kenya | 35.157708 | 1.193351 | " |
| *Rhipicephalus appendiculatus* | Kenya | 35.152148 | 1.215581 | " |
| *Rhipicephalus appendiculatus* | Kenya | 35.199216 | 1.182588 | " |
| *Rhipicephalus appendiculatus* | Kenya | 35.07452 | 1.44066 | " |
| *Rhipicephalus appendiculatus* | Kenya | 35.137633 | 1.323712 | " |
| *Rhipicephalus appendiculatus* | Kenya | 35.161181 | 1.484308 | " |
| *Rhipicephalus appendiculatus* | Kenya | 35.367946 | 1.580398 | " |
| *Rhipicephalus appendiculatus* | Kenya | 35.376739 | 1.458433 | " |
| *Rhipicephalus appendiculatus* | Kenya | 35.355434 | 1.400875 | " |
| *Rhipicephalus appendiculatus* | Kenya | 35.424560 | 1.418954 | " |
| *Rhipicephalus appendiculatus* | Kenya | 35.390439 | 1.368140 | " |
| *Rhipicephalus appendiculatus* | Kenya | 35.321580 | 1.318720 | " |
| *Rhipicephalus appendiculatus* | Kenya | 35.370582 | 1.271585 | " |
| *Rhipicephalus appendiculatus* | Kenya | 35.408894 | 1.295627 | " |
| *Rhipicephalus appendiculatus* | Kenya | 35.506834 | 1.354086 | " |
| *Rhipicephalus appendiculatus* | Kenya | 35.501663 | 1.307353 | " |
| *Rhipicephalus appendiculatus* | Kenya | 35.518861 | 1.500405 | " |
| *Rhipicephalus appendiculatus* | Kenya | 35.543763 | 1.552508 | " |
| *Rhipicephalus appendiculatus* | Kenya | 35.557782 | 1.487203 | " |
| *Rhipicephalus appendiculatus* | Kenya | 35.151140 | 1.723004 | " |
| *Rhipicephalus appendiculatus* | Kenya | 35.196339 | 1.722027 | " |
| *Rhipicephalus appendiculatus* | Kenya | 35.211515 | 1.700269 | " |
| *Rhipicephalus appendiculatus* | Kenya | 35.267702 | 1.780704 | " |
| *Rhipicephalus appendiculatus* | Kenya | 35.335220 | 1.826911 | " |
| *Rhipicephalus appendiculatus* | Kenya | 36.694016 | 1.227385 | " |
| *Rhipicephalus appendiculatus* | Kenya | 36.803275 | 1.072910 | " |
| *Rhipicephalus appendiculatus* | Kenya | 35.834362 | 0.719024 | " |
| *Rhipicephalus appendiculatus* | Kenya | 35.836519 | 0.701059 | " |
| *Rhipicephalus appendiculatus* | Kenya | 35.838276 | 0.676135 | " |
| *Rhipicephalus appendiculatus* | Kenya | 35.836145 | 0.646352 | " |
| *Rhipicephalus appendiculatus* | Kenya | 35.713789 | 0.174407 | " |
| *Rhipicephalus appendiculatus* | Kenya | 35.655064 | 0.106697 | " |
| *Rhipicephalus appendiculatus* | Kenya | 36.078937 | 0.354764 | " |
| *Rhipicephalus appendiculatus* | Kenya | 36.252084 | -0.845333 | " |
| *Rhipicephalus appendiculatus* | Kenya | 36.199587 | -0.690863 | " |
| *Rhipicephalus appendiculatus* | Kenya | 36.367107 | -0.698713 | " |
| *Rhipicephalus appendiculatus* | Kenya | 36.387490 | -0.561041 | " |
| *Rhipicephalus appendiculatus* | Kenya | 36.228393 | -0.405207 | " |
| *Rhipicephalus appendiculatus* | Kenya | 36.196400 | -0.239651 | " |
| *Rhipicephalus appendiculatus* | Kenya | 36.248476 | -0.361753 | " |
| *Rhipicephalus appendiculatus* | Kenya | 36.130667 | -0.334919 | " |
| *Rhipicephalus appendiculatus* | Kenya | 36.081294 | -0.233237 | " |
| *Rhipicephalus appendiculatus* | Kenya | 36.203706 | -0.078131 | " |
| *Rhipicephalus appendiculatus* | Kenya | 36.203587 | 0.257118 | " |
| *Rhipicephalus appendiculatus* | Kenya | 36.215376 | 0.230925 | " |
| *Rhipicephalus appendiculatus* | Kenya | 36.282055 | 0.216897 | " |
| *Rhipicephalus appendiculatus* | Kenya | 36.270385 | 0.242563 | " |
| *Rhipicephalus appendiculatus* | Kenya | 36.353303 | 0.362287 | " |
| *Rhipicephalus appendiculatus* | Kenya | 36.414500 | 0.229861 | " |
| *Rhipicephalus appendiculatus* | Kenya | 36.566454 | 0.294046 | " |
| *Rhipicephalus appendiculatus* | Kenya | 36.377125 | 0.048205 | " |
| *Rhipicephalus appendiculatus* | Kenya | 36.298706 | 0.033260 | " |
| *Rhipicephalus appendiculatus* | Kenya | 36.599846 | 0.633259 | " |
| *Rhipicephalus appendiculatus* | Kenya | 36.769511 | 0.494451 | " |
| *Rhipicephalus appendiculatus* | Kenya | 37.089556 | 0.211728 | " |
| *Rhipicephalus appendiculatus* | Kenya | 36.912269 | 0.012879 | " |
| *Rhipicephalus appendiculatus* | Kenya | 36.952319 | -0.084278 | " |
| *Rhipicephalus appendiculatus* | Kenya | 36.972140 | -0.055638 | " |
| *Rhipicephalus appendiculatus* | Kenya | 38.555440 | 0.458586 | " |
| *Rhipicephalus appendiculatus* | Kenya | 38.190719 | 0.233911 | " |
| *Rhipicephalus appendiculatus* | Kenya | 38.050283 | 0.332321 | " |
| *Rhipicephalus appendiculatus* | Kenya | 37.987823 | 0.404969 | " |
| *Rhipicephalus appendiculatus* | Kenya | 38.018510 | 0.325415 | " |
| *Rhipicephalus appendiculatus* | Kenya | 37.967139 | 0.299544 | " |
| *Rhipicephalus appendiculatus* | Kenya | 37.944018 | 0.283895 | " |
| *Rhipicephalus appendiculatus* | Kenya | 37.905611 | 0.262684 | " |
| *Rhipicephalus appendiculatus* | Kenya | 37.876535 | 0.240406 | " |
| *Rhipicephalus appendiculatus* | Kenya | 37.892816 | 0.183794 | " |
| *Rhipicephalus appendiculatus* | Kenya | 37.883902 | 0.082547 | " |
| *Rhipicephalus appendiculatus* | Kenya | 37.885030 | -0.028862 | " |
| *Rhipicephalus appendiculatus* | Kenya | 37.799456 | -0.153808 | " |
| *Rhipicephalus appendiculatus* | Kenya | 37.725991 | -0.088691 | " |
| *Rhipicephalus appendiculatus* | Kenya | 37.555563 | -0.066578 | " |
| *Rhipicephalus appendiculatus* | Kenya | 37.544249 | 0.042865 | " |
| *Rhipicephalus appendiculatus* | Kenya | 37.585389 | -0.328280 | " |
| *Rhipicephalus appendiculatus* | Kenya | 37.777051 | -0.240862 | " |
| *Rhipicephalus appendiculatus* | Kenya | 37.919728 | -0.187143 | " |
| *Rhipicephalus appendiculatus* | Kenya | 37.956887 | -0.251476 | " |
| *Rhipicephalus appendiculatus* | Kenya | 37.936454 | -0.348588 | " |
| *Rhipicephalus appendiculatus* | Kenya | 38.097475 | -0.207045 | " |
| *Rhipicephalus appendiculatus* | Kenya | 37.519486 | -0.345095 | " |
| *Rhipicephalus appendiculatus* | Kenya | 37.732504 | -0.453217 | " |
| *Rhipicephalus appendiculatus* | Kenya | 37.712508 | -0.554937 | " |
| *Rhipicephalus appendiculatus* | Kenya | 37.809763 | -0.694369 | " |
| *Rhipicephalus appendiculatus* | Kenya | 37.614518 | -0.687627 | " |
| *Rhipicephalus appendiculatus* | Kenya | 37.493040 | -0.802975 | " |
| *Rhipicephalus appendiculatus* | Kenya | 37.685147 | -0.556190 | " |
| *Rhipicephalus appendiculatus* | Kenya | 37.586847 | -0.616006 | " |
| *Rhipicephalus appendiculatus* | Kenya | 37.592726 | -0.467780 | " |
| *Rhipicephalus appendiculatus* | Kenya | 37.540944 | -0.462565 | " |
| *Rhipicephalus appendiculatus* | Kenya | 37.392948 | -0.336626 | " |
| *Rhipicephalus appendiculatus* | Kenya | 37.441707 | -0.351726 | " |
| *Rhipicephalus appendiculatus* | Kenya | 37.423286 | -0.402360 | " |
| *Rhipicephalus appendiculatus* | Kenya | 37.369017 | -0.360621 | " |
| *Rhipicephalus appendiculatus* | Kenya | 37.405607 | -0.404855 | " |
| *Rhipicephalus appendiculatus* | Kenya | 37.421599 | -0.692417 | " |
| *Rhipicephalus appendiculatus* | Kenya | 37.329117 | -0.641315 | " |
| *Rhipicephalus appendiculatus* | Kenya | 37.247027 | -0.449891 | " |
| *Rhipicephalus appendiculatus* | Kenya | 36.771939 | -0.201099 | " |
| *Rhipicephalus appendiculatus* | Kenya | 36.903380 | -0.248110 | " |
| *Rhipicephalus appendiculatus* | Kenya | 36.819875 | -0.316363 | " |
| *Rhipicephalus appendiculatus* | Kenya | 36.805361 | -0.382792 | " |
| *Rhipicephalus appendiculatus* | Kenya | 36.823222 | -0.484551 | " |
| *Rhipicephalus appendiculatus* | Kenya | 36.934421 | -0.325034 | " |
| *Rhipicephalus appendiculatus* | Kenya | 36.939838 | -0.368761 | " |
| *Rhipicephalus appendiculatus* | Kenya | 36.980178 | -0.297482 | " |
| *Rhipicephalus appendiculatus* | Kenya | 36.979680 | -0.329909 | " |
| *Rhipicephalus appendiculatus* | Kenya | 37.045726 | -0.231660 | " |
| *Rhipicephalus appendiculatus* | Kenya | 37.064299 | -0.332198 | " |
| *Rhipicephalus appendiculatus* | Kenya | 36.970510 | -0.532423 | " |
| *Rhipicephalus appendiculatus* | Kenya | 37.076878 | -0.575296 | " |
| *Rhipicephalus appendiculatus* | Kenya | 36.864163 | -0.701905 | " |
| *Rhipicephalus appendiculatus* | Kenya | 36.917886 | -0.706544 | " |
| *Rhipicephalus appendiculatus* | Kenya | 36.959318 | -0.715957 | " |
| *Rhipicephalus appendiculatus* | Kenya | 37.059420 | -0.745900 | " |
| *Rhipicephalus appendiculatus* | Kenya | 36.784185 | -0.638479 | " |
| *Rhipicephalus appendiculatus* | Kenya | 36.795457 | -0.685202 | " |
| *Rhipicephalus appendiculatus* | Kenya | 36.888454 | -0.726873 | " |
| *Rhipicephalus appendiculatus* | Kenya | 36.960715 | -0.768725 | " |
| *Rhipicephalus appendiculatus* | Kenya | 37.034013 | -0.796535 | " |
| *Rhipicephalus appendiculatus* | Kenya | 36.870811 | -0.772350 | " |
| *Rhipicephalus appendiculatus* | Kenya | 36.930880 | -0.803334 | " |
| *Rhipicephalus appendiculatus* | Kenya | 36.994934 | -0.834461 | " |
| *Rhipicephalus appendiculatus* | Kenya | 37.067351 | -0.860314 | " |
| *Rhipicephalus appendiculatus* | Kenya | 37.112380 | -1.069190 | " |
| *Rhipicephalus appendiculatus* | Kenya | 36.951593 | -0.856599 | " |
| *Rhipicephalus appendiculatus* | Kenya | 36.990089 | -0.869942 | " |
| *Rhipicephalus appendiculatus* | Kenya | 37.026834 | -0.897528 | " |
| *Rhipicephalus appendiculatus* | Kenya | 36.895552 | -0.960090 | " |
| *Rhipicephalus appendiculatus* | Kenya | 36.908122 | -0.974734 | " |
| *Rhipicephalus appendiculatus* | Kenya | 36.948267 | -1.035824 | " |
| *Rhipicephalus appendiculatus* | Kenya | 36.773747 | -1.367955 | " |
| *Rhipicephalus appendiculatus* | Kenya | 36.703039 | -1.354811 | " |
| *Rhipicephalus appendiculatus* | Kenya | 36.709628 | -1.300668 | " |
| *Rhipicephalus appendiculatus* | Kenya | 36.714608 | -1.268521 | " |
| *Rhipicephalus appendiculatus* | Kenya | 36.733081 | -1.310125 | " |
| *Rhipicephalus appendiculatus* | Kenya | 36.758832 | -1.320019 | " |
| *Rhipicephalus appendiculatus* | Kenya | 36.682809 | -1.043222 | " |
| *Rhipicephalus appendiculatus* | Kenya | 36.670543 | -0.872452 | " |
| *Rhipicephalus appendiculatus* | Kenya | 36.512393 | -0.517973 | " |
| *Rhipicephalus appendiculatus* | Kenya | 36.504989 | -0.416850 | " |
| *Rhipicephalus appendiculatus* | Kenya | 36.329402 | -0.122788 | " |
| *Rhipicephalus appendiculatus* | Kenya | 36.442802 | -0.098105 | " |
| *Rhipicephalus appendiculatus* | Kenya | 36.508475 | -0.238185 | " |
| *Rhipicephalus appendiculatus* | Kenya | 36.330270 | -0.299126 | " |
| *Rhipicephalus appendiculatus* | Somalia | 41.791501 | -0.772627 | (Cumming 1999) |
| *Rhipicephalus appendiculatus* | Somalia | 41.742544 | -0.813267 | " |
| *Rhipicephalus appendiculatus* | Somalia | 42.493804 | 2.900827 | " |
| *Rhipicephalus appendiculatus* | Somalia | 42.501412 | 3.260019 | " |
| *Rhipicephalus appendiculatus* | Tanzania | 33.984794 | -1.444527 | (Isack et al., 2017) |
| *Rhipicephalus appendiculatus* | Tanzania | 34.126779 | -1.204694 | " |
| *Rhipicephalus appendiculatus* | Tanzania | 34.279921 | -1.234927 | " |
| *Rhipicephalus appendiculatus* | Tanzania | 34.606524 | -1.44907 | " |
| *Rhipicephalus appendiculatus* | Tanzania | 34.586225 | -1.848579 | " |
| *Rhipicephalus appendiculatus* | Tanzania | 34.527270 | -2.22616 | " |
| *Rhipicephalus appendiculatus* | Tanzania | 34.745763 | -4.835057 | " |
| *Rhipicephalus appendiculatus* | Tanzania | 34.596154 | -4.888465 | " |
| *Rhipicephalus appendiculatus* | Tanzania | 34.685665 | -4.859297 | " |
| *Rhipicephalus appendiculatus* | Tanzania | 34.713410 | -4.981069 | " |
| *Rhipicephalus appendiculatus* | Tanzania | 34.611216 | -5.057176 | " |
| *Rhipicephalus appendiculatus* | Tanzania | 34.929028 | -4.825508 | " |
| *Rhipicephalus appendiculatus* | Tanzania | 35.007907 | -5.089898 | " |
| *Rhipicephalus appendiculatus* | Tanzania | 34.974019 | -4.782591 | " |
| *Rhipicephalus appendiculatus* | Tanzania | 33.28351 | -3.11198 | (Tatchell et al., 1986) |
| *Rhipicephalus appendiculatus* | Tanzania | 33.409027 | -3.556908 | " |
| *Rhipicephalus appendiculatus* | Tanzania | 33.423712 | -3.718297 | " |
| *Rhipicephalus appendiculatus* | Tanzania | 33.513579 | -3.364092 | " |
| *Rhipicephalus appendiculatus* | Tanzania | 33.348087 | -3.805323 | " |
| *Rhipicephalus appendiculatus* | Tanzania | 36.304517 | -7.925091 | " |
| *Rhipicephalus appendiculatus* | Tanzania | 35.263491 | -7.840056 | " |
| *Rhipicephalus appendiculatus* | Tanzania | 34.254596 | -8.692761 | " |
| *Rhipicephalus appendiculatus* | Uganda | 34.698249 | 2.524732 | (Byaruhanga et al., 2015) |
| *Rhipicephalus appendiculatus* | Uganda | 33.248865 | 3.183847 | " |
| *Rhipicephalus appendiculatus* | Uganda | 34.841131 | 2.464204 | " |
| *Rhipicephalus appendiculatus* | Uganda | 34.835078 | 2.387579 | " |
| *Rhipicephalus appendiculatus* | Uganda | 34.676570 | 2.297801 | " |
| *Rhipicephalus appendiculatus* | Uganda | 34.870170 | 2.240098 | " |
| *Rhipicephalus appendiculatus* | Uganda | 34.425937 | 2.621005 | " |
| *Rhipicephalus appendiculatus* | Uganda | 34.449571 | 2.588969 | " |
| *Rhipicephalus appendiculatus* | Uganda | 34.474196 | 2.572182 | " |
| *Rhipicephalus appendiculatus* | Uganda | 33.957498 | 3.315638 | " |
| *Rhipicephalus appendiculatus* | Uganda | 33.886935 | 3.321578 | " |
| *Rhipicephalus appendiculatus* | Uganda | 33.309733 | 3.178988 | " |
| *Rhipicephalus appendiculatus* | Uganda | 33.303353 | 3.158312 | " |
| *Rhipicephalus appendiculatus* | Uganda | 32.332281 | 2.765793 | (Kaiser et al., 1991) |
| *Rhipicephalus appendiculatus* | Uganda | 34.104446 | 3.068718 | (Akure 2019) |
| *Rhipicephalus appendiculatus* | Uganda | 34.204379 | 3.00841 | " |
| *Rhipicephalus haemaphysaloides* | China | 116.361465 | 31.444425 | (LiZhao 1993) |
| *Rhipicephalus haemaphysaloides* | China | 116.422613 | 30.850516 | " |
| *Rhipicephalus haemaphysaloides* | China | 118.415441 | 29.814544 | " |
| *Rhipicephalus haemaphysaloides* | China | 116.280115 | 30.467832 | " |
| *Rhipicephalus haemaphysaloides* | China | 118.462701 | 31.673253 | " |
| *Rhipicephalus haemaphysaloides* | China | 116.239583 | 30.437447 | (FengLin 2004) |
| *Rhipicephalus haemaphysaloides* | China | 118.304464 | 29.909625 | " |
| *Rhipicephalus haemaphysaloides* | China | 118.588607 | 31.63766 | " |
| *Rhipicephalus haemaphysaloides* | China | 117.209923 | 32.515515 | " |
| *Rhipicephalus haemaphysaloides* | China | 118.740699 | 30.997734 | " |
| *Rhipicephalus haemaphysaloides* | China | 117.020112 | 30.12803 | " |
| *Rhipicephalus haemaphysaloides* | China | 119.064763 | 31.870249 | (Jun 1998) |
| *Rhipicephalus haemaphysaloides* | China | 119.555993 | 31.739703 | " |
| *Rhipicephalus haemaphysaloides* | China | 118.51827 | 32.05437 | (BenHua 1964) |
| *Rhipicephalus haemaphysaloides* | China | 119.31945 | 31.814789 | (Jun 2000) |
| *Rhipicephalus haemaphysaloides* | China | 119.560017 | 31.73479 | " |
| *Rhipicephalus haemaphysaloides* | China | 121.029941 | 29.184082 | (JunHua 2010) |
| *Rhipicephalus haemaphysaloides* | China | 118.791202 | 29.042055 | (GuoPing 2021) |
| *Rhipicephalus haemaphysaloides* | China | 119.141479 | 29.004102 | " |
| *Rhipicephalus haemaphysaloides* | China | 118.492551 | 28.872843 | " |
| *Rhipicephalus haemaphysaloides* | China | 118.857892 | 29.043318 | " |
| *Rhipicephalus haemaphysaloides* | China | 119.150534 | 28.950828 | " |
| *Rhipicephalus haemaphysaloides* | China | 118.357446 | 28.869807 | " |
| *Rhipicephalus haemaphysaloides* | China | 122.132288 | 30.036468 | (JieNan 2021) |
| *Rhipicephalus haemaphysaloides* | China | 122.395069 | 30.007115 | " |
| *Rhipicephalus haemaphysaloides* | China | 122.204718 | 30.274646 | " |
| *Rhipicephalus haemaphysaloides* | China | 122.463628 | 30.719105 | " |
| *Rhipicephalus haemaphysaloides* | China | 120.737157 | 30.50747 | (Ting 2015) |
| *Rhipicephalus haemaphysaloides* | China | 119.665864 | 30.675737 | " |
| *Rhipicephalus haemaphysaloides* | China | 119.169254 | 29.574429 | (Zhe 2014) |
| *Rhipicephalus haemaphysaloides* | China | 119.836327 | 30.640198 | (Feng 2013) |
| *Rhipicephalus haemaphysaloides* | China | 119.710941 | 29.115116 | " |
| *Rhipicephalus haemaphysaloides* | China | 121.079959 | 29.231498 | " |
| *Rhipicephalus haemaphysaloides* | China | 120.771077 | 28.835508 | " |
| *Rhipicephalus haemaphysaloides* | China | 120.023368 | 29.166049 | (XinHong 2022) |
| *Rhipicephalus haemaphysaloides* | China | 115.774036 | 28.761168 | (XiaoQing 2019) |
| *Rhipicephalus haemaphysaloides* | China | 114.790257 | 28.45552 | (ShiQuan 2001) |
| *Rhipicephalus haemaphysaloides* | China | 115.368262 | 28.84402 | " |
| *Rhipicephalus haemaphysaloides* | China | 114.93298 | 28.269107 | " |
| *Rhipicephalus haemaphysaloides* | China | 115.30027 | 27.637658 | " |
| *Rhipicephalus haemaphysaloides* | China | 115.801632 | 28.864469 | " |
| *Rhipicephalus haemaphysaloides* | China | 115.425017 | 25.934064 | " |
| *Rhipicephalus haemaphysaloides* | China | 115.357025 | 28.406464 | " |
| *Rhipicephalus haemaphysaloides* | China | 115.608469 | 28.069547 | " |
| *Rhipicephalus haemaphysaloides* | China | 115.995378 | 28.709479 | (XiaoQing 2020) |
| *Rhipicephalus haemaphysaloides* | China | 118.014141 | 28.457831 | " |
| *Rhipicephalus haemaphysaloides* | China | 116.043734 | 29.631004 | " |
| *Rhipicephalus haemaphysaloides* | China | 114.649789 | 27.778165 | " |
| *Rhipicephalus haemaphysaloides* | China | 115.370886 | 27.683401 | " |
| *Rhipicephalus haemaphysaloides* | China | 117.446816 | 27.330295 | (Liang 1996) |
| *Rhipicephalus haemaphysaloides* | China | 118.062847 | 27.295648 | " |
| *Rhipicephalus haemaphysaloides* | China | 117.295927 | 27.579298 | " |
| *Rhipicephalus haemaphysaloides* | China | 118.561236 | 27.957856 | (HaoRong 1995) |
| *Rhipicephalus haemaphysaloides* | China | 117.57301 | 27.287411 | " |
| *Rhipicephalus haemaphysaloides* | China | 117.419534 | 27.482939 | " |
| *Rhipicephalus haemaphysaloides* | China | 117.642943 | 24.479209 | " |
| *Rhipicephalus haemaphysaloides* | China | 120.769369 | 24.54138 | (Jongejan et al., 2018) |
| *Rhipicephalus haemaphysaloides* | China | 121.335949 | 25.096034 | " |
| *Rhipicephalus haemaphysaloides* | China | 121.438858 | 25.077708 | " |
| *Rhipicephalus haemaphysaloides* | China | 121.552117 | 25.011974 | " |
| *Rhipicephalus haemaphysaloides* | China | 121.56304 | 25.097342 | " |
| *Rhipicephalus haemaphysaloides* | China | 121.560092 | 23.997823 | (Kuo et al., 2011) |
| *Rhipicephalus haemaphysaloides* | China | 121.655636 | 24.017135 | (Kuo et al., 2015) |
| *Rhipicephalus haemaphysaloides* | China | 121.157279 | 22.807082 | " |
| *Rhipicephalus haemaphysaloides* | China | 121.382461 | 24.976988 | " |
| *Rhipicephalus haemaphysaloides* | China | 120.80029 | 24.132045 | " |
| *Rhipicephalus haemaphysaloides* | China | 118.361882 | 24.443493 | " |
| *Rhipicephalus haemaphysaloides* | China | 121.51 | 25.02 | (Chao et al., 2019) |
| *Rhipicephalus haemaphysaloides* | China | 121.57 | 25.02 | " |
| *Rhipicephalus haemaphysaloides* | China | 121.56 | 25.08 | " |
| *Rhipicephalus haemaphysaloides* | China | 121.53 | 25.09 | " |
| *Rhipicephalus haemaphysaloides* | China | 121.58 | 25.09 | " |
| *Rhipicephalus haemaphysaloides* | China | 121.51 | 25.09 | " |
| *Rhipicephalus haemaphysaloides* | China | 121.58 | 25.10 | " |
| *Rhipicephalus haemaphysaloides* | China | 121.55 | 25.11 | " |
| *Rhipicephalus haemaphysaloides* | China | 121.54 | 25.07 | " |
| *Rhipicephalus haemaphysaloides* | China | 121.56 | 25.07 | " |
| *Rhipicephalus haemaphysaloides* | China | 121.50 | 25.05 | " |
| *Rhipicephalus haemaphysaloides* | China | 121.51 | 25.05 | " |
| *Rhipicephalus haemaphysaloides* | China | 121.50 | 25.02 | " |
| *Rhipicephalus haemaphysaloides* | China | 121.49 | 25.01 | " |
| *Rhipicephalus haemaphysaloides* | China | 121.48 | 25.03 | " |
| *Rhipicephalus haemaphysaloides* | China | 121.57 | 25.03 | " |
| *Rhipicephalus haemaphysaloides* | China | 121.53 | 25.03 | " |
| *Rhipicephalus haemaphysaloides* | China | 121.51 | 25.14 | " |
| *Rhipicephalus haemaphysaloides* | China | 121.50 | 25.13 | " |
| *Rhipicephalus haemaphysaloides* | China | 121.62 | 25.03 | " |
| *Rhipicephalus haemaphysaloides* | China | 121.60 | 25.08 | " |
| *Rhipicephalus haemaphysaloides* | China | 121.56 | 25.07 | " |
| *Rhipicephalus haemaphysaloides* | China | 121.57 | 24.98 | " |
| *Rhipicephalus haemaphysaloides* | China | 106.848509 | 22.31353 | (GuoHou 1995) |
| *Rhipicephalus haemaphysaloides* | China | 106.496382 | 23.953901 | " |
| *Rhipicephalus haemaphysaloides* | China | 108.371442 | 22.764767 | " |
| *Rhipicephalus haemaphysaloides* | China | 108.982341 | 19.158892 | (DingWei 2013) |
| *Rhipicephalus haemaphysaloides* | China | 110.784964 | 19.565685 | " |
| *Rhipicephalus haemaphysaloides* | China | 109.974414 | 19.731012 | " |
| *Rhipicephalus haemaphysaloides* | China | 109.469405 | 19.113467 | (Qian 2021) |
| *Rhipicephalus haemaphysaloides* | China | 109.131211 | 18.7525 | " |
| *Rhipicephalus haemaphysaloides* | China | 110.32 | 26.07 | (Liu et al., 2016) |
| *Rhipicephalus haemaphysaloides* | China | 111.288528 | 30.690241 | (YiRen 2001) |
| *Rhipicephalus haemaphysaloides* | China | 113.887217 | 29.933546 | " |
| *Rhipicephalus haemaphysaloides* | China | 112.092601 | 31.985155 | " |
| *Rhipicephalus haemaphysaloides* | China | 112.282898 | 31.911131 | " |
| *Rhipicephalus haemaphysaloides* | China | 111.773181 | 30.804208 | (ZhiJin 2002) |
| *Rhipicephalus haemaphysaloides* | China | 113.927749 | 29.987114 | (LiHua 1989) |
| *Rhipicephalus haemaphysaloides* | China | 114.424269 | 30.55444 | " |
| *Rhipicephalus haemaphysaloides* | China | 114.376685 | 29.688656 | (ZhongLing 1987) |
| *Rhipicephalus haemaphysaloides* | China | 114.508556 | 30.510588 | (LiHua 1986) |
| *Rhipicephalus haemaphysaloides* | China | 106.82314 | 26.629815 | (XiaoRong 1997) |
| *Rhipicephalus haemaphysaloides* | China | 106.387409 | 26.234673 | " |
| *Rhipicephalus haemaphysaloides* | China | 107.493562 | 26.704799 | " |
| *Rhipicephalus haemaphysaloides* | China | 104.394931 | 25.722161 | " |
| *Rhipicephalus haemaphysaloides* | China | 105.897787 | 26.163048 | " |
| *Rhipicephalus haemaphysaloides* | China | 105.374766 | 27.290984 | " |
| *Rhipicephalus haemaphysaloides* | China | 108.155229 | 28.266288 | " |
| *Rhipicephalus haemaphysaloides* | China | 106.580238 | 26.721755 | (LianKun 2011) |
| *Rhipicephalus haemaphysaloides* | China | 105.677622 | 28.540032 | " |
| *Rhipicephalus haemaphysaloides* | China | 105.375341 | 27.292012 | " |
| *Rhipicephalus haemaphysaloides* | China | 106.547181 | 26.740857 | " |
| *Rhipicephalus haemaphysaloides* | China | 104.50244 | 25.603356 | " |
| *Rhipicephalus haemaphysaloides* | China | 105.153999 | 27.272491 | " |
| *Rhipicephalus haemaphysaloides* | China | 105.965627 | 26.202732 | " |
| *Rhipicephalus haemaphysaloides* | China | 101.56305 | 21.494257 | (HaiBin 2010) |
| *Rhipicephalus haemaphysaloides* | China | 101.694174 | 21.192815 | " |
| *Rhipicephalus haemaphysaloides* | China | 101.708647 | 21.383183 | (DeSheng 2003) |
| *Rhipicephalus haemaphysaloides* | China | 100.41029 | 21.919717 | " |
| *Rhipicephalus haemaphysaloides* | China | 100.863072 | 22.012447 | " |
| *Rhipicephalus haemaphysaloides* | China | 101.040182 | 22.839667 | " |
| *Rhipicephalus haemaphysaloides* | China | 99.598348 | 22.631442 | " |
| *Rhipicephalus haemaphysaloides* | China | 99.874092 | 23.386994 | " |
| *Rhipicephalus haemaphysaloides* | China | 99.460574 | 23.554357 | " |
| *Rhipicephalus haemaphysaloides* | China | 99.155079 | 25.169407 | " |
| *Rhipicephalus haemaphysaloides* | China | 98.41624 | 25.022949 | " |
| *Rhipicephalus haemaphysaloides* | China | 103.30428 | 23.354046 | " |
| *Rhipicephalus haemaphysaloides* | China | 104.005237 | 22.604347 | " |
| *Rhipicephalus haemaphysaloides* | China | 98.278126 | 24.719176 | " |
| *Rhipicephalus haemaphysaloides* | China | 97.857378 | 24.678544 | " |
| *Rhipicephalus haemaphysaloides* | China | 99.473969 | 22.2794 | " |
| *Rhipicephalus haemaphysaloides* | China | 99.171949 | 23.143574 | " |
| *Rhipicephalus haemaphysaloides* | China | 102.921368 | 24.925862 | " |
| *Rhipicephalus haemaphysaloides* | China | 100.651625 | 22.203223 | (ZhengDa 2001) |
| *Rhipicephalus haemaphysaloides* | China | 101.589493 | 21.663281 | " |
| *Rhipicephalus haemaphysaloides* | China | 98.940537 | 23.254623 | " |
| *Rhipicephalus haemaphysaloides* | China | 97.662678 | 24.618236 | " |
| *Rhipicephalus haemaphysaloides* | China | 98.135377 | 25.153241 | " |
| *Rhipicephalus haemaphysaloides* | China | 100.785306 | 24.474756 | (KaiFei 2016) |
| *Rhipicephalus haemaphysaloides* | China | 97.916244 | 24.126546 | " |
| *Rhipicephalus haemaphysaloides* | China | 97.85824 | 24.712424 | " |
| *Rhipicephalus haemaphysaloides* | China | 105.567067 | 23.657069 | " |
| *Rhipicephalus haemaphysaloides* | China | 99.232593 | 24.071071 | (ZhiHai 2020) |
| *Rhipicephalus haemaphysaloides* | China | 100.340384 | 25.335992 | (HongXiang 2016) |
| *Rhipicephalus haemaphysaloides* | China | 100.53467 | 25.350642 | " |
| *Rhipicephalus haemaphysaloides* | China | 99.156265 | 25.16954 | " |
| *Rhipicephalus haemaphysaloides* | China | 99.273152 | 23.572377 | (DaoMao 1982) |
| *Rhipicephalus haemaphysaloides* | China | 99.08609 | 25.09299 | " |
| *Rhipicephalus haemaphysaloides* | China | 100.867672 | 22.00923 | " |
| *Rhipicephalus haemaphysaloides* | China | 99.84822 | 23.382483 | " |
| *Rhipicephalus haemaphysaloides* | China | 101.743142 | 21.484924 | " |
| *Rhipicephalus haemaphysaloides* | China | 85.989792 | 27.993572 | (YouZhi 2009) |
| *Rhipicephalus haemaphysaloides* | India | 76.594767 | 8.893891 | (Ghosh et al., 2006) |
| *Rhipicephalus haemaphysaloides* | India | 77.540026 | 11.631261 | " |
| *Rhipicephalus haemaphysaloides* | India | 77.714103 | 14.882048 | " |
| *Rhipicephalus haemaphysaloides* | India | 76.062092 | 16.736422 | " |
| *Rhipicephalus haemaphysaloides* | India | 75.095869 | 20.635066 | " |
| *Rhipicephalus haemaphysaloides* | India | 84.321996 | 21.286435 | " |
| *Rhipicephalus haemaphysaloides* | India | 80.124623 | 22.377485 | " |
| *Rhipicephalus haemaphysaloides* | India | 75.858482 | 27.008946 | " |
| *Rhipicephalus haemaphysaloides* | India | 76.441528 | 29.361945 | " |
| *Rhipicephalus haemaphysaloides* | India | 75.235225 | 31.073157 | " |
| *Rhipicephalus haemaphysaloides* | India | 76.859272 | 31.917634 | " |
| *Rhipicephalus haemaphysaloides* | India | 75.38593 | 33.174989 | " |
| *Rhipicephalus haemaphysaloides* | India | 79.813698 | 29.646262 | " |
| *Rhipicephalus haemaphysaloides* | India | 80.131279 | 26.819678 | " |
| *Rhipicephalus haemaphysaloides* | India | 85.174192 | 25.505144 | " |
| *Rhipicephalus haemaphysaloides* | India | 87.124942 | 22.734062 | " |
| *Rhipicephalus haemaphysaloides* | India | 88.395429 | 27.586775 | " |
| *Rhipicephalus haemaphysaloides* | India | 77.301971 | 28.574674 | " |
| *Rhipicephalus haemaphysaloides* | India | 93.223943 | 25.272791 | " |
| *Rhipicephalus haemaphysaloides* | India | 93.594062 | 27.957313 | " |
| *Rhipicephalus haemaphysaloides* | India | 76.125497 | 11.499648 | (Kumar et al., 2018) |
| *Rhipicephalus haemaphysaloides* | India | 82.103821 | 22.499821 | (Miranpuri et al., 1975) |
| *Rhipicephalus haemaphysaloides* | India | 76.265845 | 32.101078 | " |
| *Rhipicephalus haemaphysaloides* | India | 74.896629 | 34.100489 | " |
| *Rhipicephalus haemaphysaloides* | India | 75.148823 | 32.91469 | " |
| *Rhipicephalus haemaphysaloides* | India | 74.914749 | 31.592592 | " |
| *Rhipicephalus haemaphysaloides* | India | 75.382593 | 32.045489 | " |
| *Rhipicephalus haemaphysaloides* | India | 75.924466 | 31.661921 | " |
| *Rhipicephalus haemaphysaloides* | India | 75.384835 | 31.394661 | " |
| *Rhipicephalus haemaphysaloides* | India | 75.806227 | 30.944008 | " |
| *Rhipicephalus haemaphysaloides* | India | 76.359612 | 30.392253 | " |
| *Rhipicephalus haemaphysaloides* | India | 75.532217 | 31.358739 | " |
| *Rhipicephalus haemaphysaloides* | India | 76.529645 | 30.983967 | " |
| *Rhipicephalus haemaphysaloides* | India | 73.913212 | 29.909545 | " |
| *Rhipicephalus haemaphysaloides* | India | 78.007862 | 30.341707 | " |
| *Rhipicephalus haemaphysaloides* | Sri Lanka | 80.347477 | 6.730068 | (Diyes et al., 2015) |
| *Rhipicephalus haemaphysaloides* | Sri Lanka | 80.641573 | 7.282454 | " |
| *Rhipicephalus haemaphysaloides* | Sri Lanka | 80.352631 | 7.251261 | " |
| *Rhipicephalus haemaphysaloides* | Sri Lanka | 79.898869 | 6.92416 | " |
| *Rhipicephalus haemaphysaloides* | Sri Lanka | 79.992598 | 7.079286 | " |
| *Rhipicephalus haemaphysaloides* | Sri Lanka | 80.378548 | 7.459765 | " |
| *Rhipicephalus haemaphysaloides* | Sri Lanka | 79.838918 | 8.055891 | " |
| *Rhipicephalus haemaphysaloides* | Sri Lanka | 81.370191 | 6.672802 | " |
| *Rhipicephalus haemaphysaloides* | Sri Lanka | 80.36699 | 9.347802 | " |
| *Rhipicephalus haemaphysaloides* | Sri Lanka | 80.043384 | 9.66923 | " |
| *Rhipicephalus haemaphysaloides* | Sri Lanka | 80.360696 | 8.347257 | " |
| *Rhipicephalus haemaphysaloides* | Sri Lanka | 81.66431 | 7.742472 | " |
| *Rhipicephalus haemaphysaloides* | Sri Lanka | 81.006311 | 7.953833 | " |
| *Rhipicephalus haemaphysaloides* | Sri Lanka | 81.116147 | 6.163081 | (Bandaranayaka et al., 2022) |
| *Rhipicephalus haemaphysaloides* | Sri Lanka | 81.65789 | 7.301934 | " |
| *Rhipicephalus haemaphysaloides* | Sri Lanka | 81.189698 | 8.596999 | " |
| *Rhipicephalus haemaphysaloides* | Sri Lanka | 80.366449 | 9.34511 | " |
| *Rhipicephalus haemaphysaloides* | Sri Lanka | 80.355588 | 8.349413 | " |
| *Rhipicephalus haemaphysaloides* | Sri Lanka | 80.523842 | 8.773335 | " |
| *Rhipicephalus haemaphysaloides* | Sri Lanka | 79.838232 | 8.056231 | " |
| *Rhipicephalus haemaphysaloides* | Sri Lanka | 81.662755 | 7.742578 | " |
| *Rhipicephalus haemaphysaloides* | Sri Lanka | 80.044905 | 9.667248 | " |
| *Rhipicephalus haemaphysaloides* | Sri Lanka | 81.328892 | 6.63245 | " |
| *Rhipicephalus haemaphysaloides* | Sri Lanka | 81.005796 | 7.953663 | " |
| *Rhipicephalus haemaphysaloides* | Sri Lanka | 79.854211 | 9.009789 | " |
| *Rhipicephalus haemaphysaloides* | Sri Lanka | 80.756925 | 9.239702 | " |
| *Rhipicephalus haemaphysaloides* | Sri Lanka | 80.751551 | 6.982961 | " |
| *Rhipicephalus haemaphysaloides* | Sri Lanka | 80.347477 | 6.736546 | " |
| *Rhipicephalus haemaphysaloides* | Sri Lanka | 80.617788 | 7.292373 | " |
| *Rhipicephalus haemaphysaloides* | Sri Lanka | 80.345464 | 7.257647 | " |
| *Rhipicephalus haemaphysaloides* | Sri Lanka | 80.573342 | 5.96295 | " |
| *Rhipicephalus haemaphysaloides* | Sri Lanka | 79.986059 | 7.079015 | " |
| *Rhipicephalus haemaphysaloides* | Sri Lanka | 79.992537 | 6.596826 | " |
| *Rhipicephalus haemaphysaloides* | Sri Lanka | 80.333809 | 7.4688 | " |
| *Rhipicephalus haemaphysaloides* | Sri Lanka | 81.054263 | 6.972625 | " |
| *Rhipicephalus haemaphysaloides* | Sri Lanka | 80.607418 | 7.466164 | " |
| *Rhipicephalus pulchellus* | Ethiopia | 39.911 | 13.43 | (Pegram et al., 1981) |
| *Rhipicephalus pulchellus* | Ethiopia | 39.981964 | 12.888419 | " |
| *Rhipicephalus pulchellus* | Ethiopia | 39.806937 | 12.286744 | " |
| *Rhipicephalus pulchellus* | Ethiopia | 41.327718 | 11.92696 | " |
| *Rhipicephalus pulchellus* | Ethiopia | 39.542504 | 11.683483 | " |
| *Rhipicephalus pulchellus* | Ethiopia | 40.106546 | 11.520374 | " |
| *Rhipicephalus pulchellus* | Ethiopia | 40.239954 | 10.939755 | " |
| *Rhipicephalus pulchellus* | Ethiopia | 42.079834 | 10.261286 | " |
| *Rhipicephalus pulchellus* | Ethiopia | 40.618025 | 10.096318 | " |
| *Rhipicephalus pulchellus* | Ethiopia | 40.085387 | 10.030401 | " |
| *Rhipicephalus pulchellus* | Ethiopia | 39.70473 | 9.959701 | " |
| *Rhipicephalus pulchellus* | Ethiopia | 39.39871 | 9.326364 | " |
| *Rhipicephalus pulchellus* | Ethiopia | 42.102896 | 9.279076 | " |
| *Rhipicephalus pulchellus* | Ethiopia | 42.848657 | 9.1507 | " |
| *Rhipicephalus pulchellus* | Ethiopia | 38.678402 | 9.117317 | " |
| *Rhipicephalus pulchellus* | Ethiopia | 39.258366 | 8.7423 | " |
| *Rhipicephalus pulchellus* | Ethiopia | 41.149 | 8.448 | " |
| *Rhipicephalus pulchellus* | Ethiopia | 42.224 | 8.353 | " |
| *Rhipicephalus pulchellus* | Ethiopia | 40.413873 | 8.249877 | " |
| *Rhipicephalus pulchellus* | Ethiopia | 39.548716 | 8.062663 | " |
| *Rhipicephalus pulchellus* | Ethiopia | 42.555508 | 8.591345 | " |
| *Rhipicephalus pulchellus* | Ethiopia | 38.423438 | 7.611674 | " |
| *Rhipicephalus pulchellus* | Ethiopia | 45.810809 | 7.49107 | " |
| *Rhipicephalus pulchellus* | Ethiopia | 39.135253 | 7.30867 | " |
| *Rhipicephalus pulchellus* | Ethiopia | 41.225 | 7.066 | " |
| *Rhipicephalus pulchellus* | Ethiopia | 44.118 | 6.781 | " |
| *Rhipicephalus pulchellus* | Ethiopia | 37.813143 | 6.75516 | " |
| *Rhipicephalus pulchellus* | Ethiopia | 42.790526 | 6.620063 | " |
| *Rhipicephalus pulchellus* | Ethiopia | 39.407 | 6.322 | " |
| *Rhipicephalus pulchellus* | Ethiopia | 40.397 | 6.293 | " |
| *Rhipicephalus pulchellus* | Ethiopia | 38.049111 | 6.158144 | " |
| *Rhipicephalus pulchellus* | Ethiopia | 37.439629 | 6.10364 | " |
| *Rhipicephalus pulchellus* | Ethiopia | 44.967 | 5.692 | " |
| *Rhipicephalus pulchellus* | Ethiopia | 42.134891 | 5.482791 | " |
| *Rhipicephalus pulchellus* | Ethiopia | 37.281259 | 5.493132 | " |
| *Rhipicephalus pulchellus* | Ethiopia | 37.734 | 5.263 | " |
| *Rhipicephalus pulchellus* | Ethiopia | 36.439 | 5.025 | " |
| *Rhipicephalus pulchellus* | Ethiopia | 39.456 | 4.814 | " |
| *Rhipicephalus pulchellus* | Ethiopia | 39.828539 | 10.547496 | (Mekonnen et al., 2001) |
| *Rhipicephalus pulchellus* | Ethiopia | 39.876104 | 10.406041 | " |
| *Rhipicephalus pulchellus* | Ethiopia | 39.883555 | 10.208587 | " |
| *Rhipicephalus pulchellus* | Ethiopia | 39.846 | 10.089 | " |
| *Rhipicephalus pulchellus* | Ethiopia | 39.799245 | 10.010042 | " |
| *Rhipicephalus pulchellus* | Ethiopia | 39.135 | 9.954 | " |
| *Rhipicephalus pulchellus* | Ethiopia | 39.68405 | 9.943727 | " |
| *Rhipicephalus pulchellus* | Ethiopia | 40.020136 | 9.985777 | " |
| *Rhipicephalus pulchellus* | Ethiopia | 39.725758 | 9.33115 | " |
| *Rhipicephalus pulchellus* | Ethiopia | 39.826 | 9.283 | " |
| *Rhipicephalus pulchellus* | Ethiopia | 40.002739 | 9.190454 | " |
| *Rhipicephalus pulchellus* | Ethiopia | 39.737782 | 9.170258 | " |
| *Rhipicephalus pulchellus* | Ethiopia | 39.624063 | 9.156473 | " |
| *Rhipicephalus pulchellus* | Ethiopia | 38.371389 | 8.998695 | " |
| *Rhipicephalus pulchellus* | Ethiopia | 38.749694 | 8.916734 | " |
| *Rhipicephalus pulchellus* | Ethiopia | 39.372941 | 8.942746 | " |
| *Rhipicephalus pulchellus* | Ethiopia | 39.693871 | 8.963508 | " |
| *Rhipicephalus pulchellus* | Ethiopia | 39.855703 | 9.032844 | " |
| *Rhipicephalus pulchellus* | Ethiopia | 38.859427 | 8.814773 | " |
| *Rhipicephalus pulchellus* | Ethiopia | 39.856 | 8.778 | " |
| *Rhipicephalus pulchellus* | Ethiopia | 39.609172 | 8.723689 | " |
| *Rhipicephalus pulchellus* | Ethiopia | 38.964893 | 8.676707 | " |
| *Rhipicephalus pulchellus* | Ethiopia | 38.80055 | 8.645577 | " |
| *Rhipicephalus pulchellus* | Ethiopia | 39.131595 | 8.513479 | " |
| *Rhipicephalus pulchellus* | Ethiopia | 38.715496 | 7.948113 | " |
| *Rhipicephalus pulchellus* | Ethiopia | 36.766 | 13.692 | (Kaba 2022) |
| *Rhipicephalus pulchellus* | Ethiopia | 39.692 | 12.978 | " |
| *Rhipicephalus pulchellus* | Ethiopia | 39.585487 | 12.769394 | " |
| *Rhipicephalus pulchellus* | Ethiopia | 36.166 | 11.307 | " |
| *Rhipicephalus pulchellus* | Ethiopia | 36.373 | 11.25 | " |
| *Rhipicephalus pulchellus* | Ethiopia | 36.28 | 11.029 | " |
| *Rhipicephalus pulchellus* | Ethiopia | 37.051 | 9.103 | " |
| *Rhipicephalus pulchellus* | Ethiopia | 38.288 | 9.519 | " |
| *Rhipicephalus pulchellus* | Ethiopia | 38.693 | 8.805 | " |
| *Rhipicephalus pulchellus* | Ethiopia | 40.906 | 8.817 | " |
| *Rhipicephalus pulchellus* | Ethiopia | 42.06 | 9.412 | " |
| *Rhipicephalus pulchellus* | Ethiopia | 42.530704 | 8.892391 | " |
| *Rhipicephalus pulchellus* | Ethiopia | 37.765 | 7.532 | " |
| *Rhipicephalus pulchellus* | Ethiopia | 38.693 | 6.723 | " |
| *Rhipicephalus pulchellus* | Ethiopia | 44.417977 | 6.508021 | " |
| *Rhipicephalus pulchellus* | Ethiopia | 38.09805 | 4.94764 | " |
| *Rhipicephalus pulchellus* | Ethiopia | 38.414033 | 4.661898 | " |
| *Rhipicephalus pulchellus* | Ethiopia | 38.708981 | 4.754848 | " |
| *Rhipicephalus pulchellus* | Ethiopia | 42.316 | 4.925 | " |
| *Rhipicephalus pulchellus* | Kenya | 35.790987 | 4.467392 | (Walker et al., 2000) |
| *Rhipicephalus pulchellus* | Kenya | 35.059806 | 3.785183 | " |
| *Rhipicephalus pulchellus* | Kenya | 37.839866 | 3.580404 | " |
| *Rhipicephalus pulchellus* | Kenya | 38.6227 | 3.55689 | " |
| *Rhipicephalus pulchellus* | Kenya | 39.59334 | 3.385569 | " |
| *Rhipicephalus pulchellus* | Kenya | 40.906408 | 3.294023 | " |
| *Rhipicephalus pulchellus* | Kenya | 41.558226 | 3.473463 | " |
| *Rhipicephalus pulchellus* | Kenya | 36.786993 | 2.255772 | " |
| *Rhipicephalus pulchellus* | Kenya | 37.902568 | 2.252034 | " |
| *Rhipicephalus pulchellus* | Kenya | 39.556591 | 2.44253 | " |
| *Rhipicephalus pulchellus* | Kenya | 40.672417 | 2.407274 | " |
| *Rhipicephalus pulchellus* | Kenya | 36.542 | 1.491 | " |
| *Rhipicephalus pulchellus* | Kenya | 37.365706 | 1.439758 | " |
| *Rhipicephalus pulchellus* | Kenya | 39.009932 | 1.23368 | " |
| *Rhipicephalus pulchellus* | Kenya | 40.624205 | 1.364884 | " |
| *Rhipicephalus pulchellus* | Kenya | 35.467083 | 0.475382 | " |
| *Rhipicephalus pulchellus* | Kenya | 36.361266 | 0.360611 | " |
| *Rhipicephalus pulchellus* | Kenya | 37.496356 | 0.42155 | " |
| *Rhipicephalus pulchellus* | Kenya | 38.588997 | 0.422326 | " |
| *Rhipicephalus pulchellus* | Kenya | 39.601429 | 0.39722 | " |
| *Rhipicephalus pulchellus* | Kenya | 36.437921 | -0.569199 | " |
| *Rhipicephalus pulchellus* | Kenya | 37.469 | -0.577 | " |
| *Rhipicephalus pulchellus* | Kenya | 39.651902 | -0.504463 | " |
| *Rhipicephalus pulchellus* | Kenya | 36.634941 | -1.425081 | " |
| *Rhipicephalus pulchellus* | Kenya | 37.394368 | -1.539798 | " |
| *Rhipicephalus pulchellus* | Kenya | 38.421 | -1.554 | " |
| *Rhipicephalus pulchellus* | Kenya | 39.437998 | -1.578979 | " |
| *Rhipicephalus pulchellus* | Kenya | 40.474305 | -1.668753 | " |
| *Rhipicephalus pulchellus* | Kenya | 41.279577 | -1.419832 | " |
| *Rhipicephalus pulchellus* | Kenya | 37.317687 | -2.576 | " |
| *Rhipicephalus pulchellus* | Kenya | 38.374476 | -2.629347 | " |
| *Rhipicephalus pulchellus* | Kenya | 38.357642 | -3.589744 | " |
| *Rhipicephalus pulchellus* | Kenya | 39.396087 | -3.624422 | " |
| *Rhipicephalus pulchellus* | Kenya | 39.243074 | -4.530851 | " |
| *Rhipicephalus pulchellus* | Somalia | 43.433 | 11.242 | (Pegram 1976) |
| *Rhipicephalus pulchellus* | Somalia | 43.493 | 10.906 | " |
| *Rhipicephalus pulchellus* | Somalia | 43.283 | 10.735 | " |
| *Rhipicephalus pulchellus* | Somalia | 43.054 | 10.185 | " |
| *Rhipicephalus pulchellus* | Somalia | 43.258 | 10.15 | " |
| *Rhipicephalus pulchellus* | Somalia | 43.447 | 10.096 | " |
| *Rhipicephalus pulchellus* | Somalia | 43.54 | 10.253 | " |
| *Rhipicephalus pulchellus* | Somalia | 43.829 | 10.132 | " |
| *Rhipicephalus pulchellus* | Somalia | 44.061 | 10 | " |
| *Rhipicephalus pulchellus* | Somalia | 43.311 | 9.943 | " |
| *Rhipicephalus pulchellus* | Somalia | 43.497 | 9.907 | " |
| *Rhipicephalus pulchellus* | Somalia | 43.386 | 9.697 | " |
| *Rhipicephalus pulchellus* | Somalia | 43.59 | 9.722 | " |
| *Rhipicephalus pulchellus* | Somalia | 43.939 | 9.84 | " |
| *Rhipicephalus pulchellus* | Somalia | 44.368 | 10.297 | " |
| *Rhipicephalus pulchellus* | Somalia | 43.511 | 9.53 | " |
| *Rhipicephalus pulchellus* | Somalia | 43.729 | 9.544 | " |
| *Rhipicephalus pulchellus* | Somalia | 44.107 | 9.78 | " |
| *Rhipicephalus pulchellus* | Somalia | 43.961 | 9.623 | " |
| *Rhipicephalus pulchellus* | Somalia | 43.907 | 9.459 | " |
| *Rhipicephalus pulchellus* | Somalia | 43.729 | 9.344 | " |
| *Rhipicephalus pulchellus* | Somalia | 44.054 | 9.378 | " |
| *Rhipicephalus pulchellus* | Somalia | 44.175 | 9.58 | " |
| *Rhipicephalus pulchellus* | Somalia | 44.311 | 9.723 | " |
| *Rhipicephalus pulchellus* | Somalia | 44.414 | 9.866 | " |
| *Rhipicephalus pulchellus* | Somalia | 44.268 | 9.412 | " |
| *Rhipicephalus pulchellus* | Somalia | 44.446 | 9.38 | " |
| *Rhipicephalus pulchellus* | Somalia | 44.81 | 9.551 | " |
| *Rhipicephalus pulchellus* | Somalia | 45.189 | 10.401 | " |
| *Rhipicephalus pulchellus* | Somalia | 45.082 | 10.055 | " |
| *Rhipicephalus pulchellus* | Somalia | 44.928 | 9.926 | " |
| *Rhipicephalus pulchellus* | Somalia | 45.231 | 9.99 | " |
| *Rhipicephalus pulchellus* | Somalia | 45.478 | 9.969 | " |
| *Rhipicephalus pulchellus* | Somalia | 45.039 | 9.744 | " |
| *Rhipicephalus pulchellus* | Somalia | 45.271 | 9.791 | " |
| *Rhipicephalus pulchellus* | Somalia | 45.46 | 9.716 | " |
| *Rhipicephalus pulchellus* | Somalia | 44.921 | 9.392 | " |
| *Rhipicephalus pulchellus* | Somalia | 45.135 | 9.427 | " |
| *Rhipicephalus pulchellus* | Somalia | 45.467 | 9.484 | " |
| *Rhipicephalus pulchellus* | Somalia | 45.695 | 9.463 | " |
| *Rhipicephalus pulchellus* | Somalia | 45.303 | 9.334 | " |
| *Rhipicephalus pulchellus* | Somalia | 43.997 | 9.196 | " |
| *Rhipicephalus pulchellus* | Somalia | 44.111 | 9.121 | " |
| *Rhipicephalus pulchellus* | Somalia | 44.196 | 9.239 | " |
| *Rhipicephalus pulchellus* | Somalia | 44.307 | 9.039 | " |
| *Rhipicephalus pulchellus* | Somalia | 44.396 | 9.203 | " |
| *Rhipicephalus pulchellus* | Somalia | 44.589 | 9.196 | " |
| *Rhipicephalus pulchellus* | Somalia | 44.746 | 8.876 | " |
| *Rhipicephalus pulchellus* | Somalia | 44.971 | 8.798 | " |
| *Rhipicephalus pulchellus* | Somalia | 45.199 | 8.883 | " |
| *Rhipicephalus pulchellus* | Somalia | 45.481 | 8.741 | " |
| *Rhipicephalus pulchellus* | Somalia | 45.72 | 8.712 | " |
| *Rhipicephalus pulchellus* | Somalia | 45.87 | 9.076 | " |
| *Rhipicephalus pulchellus* | Somalia | 46.02 | 9.412 | " |
| *Rhipicephalus pulchellus* | Somalia | 46.088 | 9.24 | " |
| *Rhipicephalus pulchellus* | Somalia | 46.427 | 9.269 | " |
| *Rhipicephalus pulchellus* | Somalia | 46.256 | 8.987 | " |
| *Rhipicephalus pulchellus* | Somalia | 46.534 | 9.012 | " |
| *Rhipicephalus pulchellus* | Somalia | 46.813 | 8.988 | " |
| *Rhipicephalus pulchellus* | Somalia | 46.677 | 8.777 | " |
| *Rhipicephalus pulchellus* | Somalia | 46.691 | 8.556 | " |
| *Rhipicephalus pulchellus* | Somalia | 46.224 | 8.467 | " |
| *Rhipicephalus pulchellus* | Somalia | 46.984 | 9.484 | " |
| *Rhipicephalus pulchellus* | Somalia | 47.323 | 9.881 | " |
| *Rhipicephalus pulchellus* | Somalia | 47.68 | 9.798 | " |
| *Rhipicephalus pulchellus* | Somalia | 46.656 | 8.308 | " |
| *Rhipicephalus pulchellus* | Somalia | 46.898 | 8.219 | " |
| *Rhipicephalus pulchellus* | Somalia | 47.041 | 8.237 | " |
| *Rhipicephalus pulchellus* | Somalia | 47.17 | 8.401 | " |
| *Rhipicephalus pulchellus* | Somalia | 47.398 | 8.715 | " |
| *Rhipicephalus pulchellus* | Somalia | 47.448 | 8.436 | " |
| *Rhipicephalus pulchellus* | Somalia | 47.348 | 8.233 | " |
| *Rhipicephalus pulchellus* | Somalia | 47.191 | 8.09 | " |
| *Rhipicephalus pulchellus* | Somalia | 47.923 | 8.105 | " |
| *Rhipicephalus pulchellus* | Somalia | 48.155 | 8.329 | " |
| *Rhipicephalus pulchellus* | Somalia | 48.058 | 7.895 | " |
| *Rhipicephalus pulchellus* | Somalia | 48.644 | 8.495 | " |
| *Rhipicephalus pulchellus* | Somalia | 49.959 | 8.124 | " |
| *Rhipicephalus pulchellus* | Somalia | 49.559 | 8.599 | " |
| *Rhipicephalus pulchellus* | Somalia | 49.666 | 9.077 | " |
| *Rhipicephalus pulchellus* | Somalia | 48.481 | 9.235 | " |
| *Rhipicephalus pulchellus* | Somalia | 48.881 | 9.26 | " |
| *Rhipicephalus pulchellus* | Somalia | 48.096 | 9.534 | " |
| *Rhipicephalus pulchellus* | Somalia | 48.624 | 9.477 | " |
| *Rhipicephalus pulchellus* | Somalia | 48.927 | 9.584 | " |
| *Rhipicephalus pulchellus* | Somalia | 49.117 | 9.492 | " |
| *Rhipicephalus pulchellus* | Somalia | 47.18 | 10.041 | " |
| *Rhipicephalus pulchellus* | Somalia | 47.251 | 10.36 | " |
| *Rhipicephalus pulchellus* | Somalia | 47.08 | 10.689 | " |
| *Rhipicephalus pulchellus* | Somalia | 47.579 | 10.927 | " |
| *Rhipicephalus pulchellus* | Somalia | 47.494 | 10.527 | " |
| *Rhipicephalus pulchellus* | Somalia | 47.794 | 10.432 | " |
| *Rhipicephalus pulchellus* | Somalia | 48.017 | 10.108 | " |
| *Rhipicephalus pulchellus* | Somalia | 48.174 | 9.937 | " |
| *Rhipicephalus pulchellus* | Somalia | 48.612 | 10.532 | " |
| *Rhipicephalus pulchellus* | Somalia | 48.641 | 10.084 | " |
| *Rhipicephalus pulchellus* | Somalia | 49.193 | 10.432 | " |
| *Rhipicephalus pulchellus* | Somalia | 49.997 | 11.355 | " |
| *Rhipicephalus pulchellus* | Somalia | 49.921 | 10.784 | " |
| *Rhipicephalus pulchellus* | Somalia | 49.916 | 10.436 | " |
| *Rhipicephalus pulchellus* | Somalia | 50.363 | 10.313 | " |
| *Rhipicephalus pulchellus* | Somalia | 50.016 | 9.913 | " |
| *Rhipicephalus pulchellus* | Tanzania | 35.534398 | -2.499291 | (Walker et al., 2000) |
| *Rhipicephalus pulchellus* | Tanzania | 36.320614 | -2.582249 | " |
| *Rhipicephalus pulchellus* | Tanzania | 35.484503 | -3.359192 | " |
| *Rhipicephalus pulchellus* | Tanzania | 36.269311 | -3.589484 | " |
| *Rhipicephalus pulchellus* | Tanzania | 37.349314 | -3.553556 | " |
| *Rhipicephalus pulchellus* | Tanzania | 35.240013 | -4.621528 | " |
| *Rhipicephalus pulchellus* | Tanzania | 36.186781 | -4.512922 | " |
| *Rhipicephalus pulchellus* | Tanzania | 37.24 | -4.547 | " |
| *Rhipicephalus pulchellus* | Tanzania | 38.256 | -4.598 | " |
| *Rhipicephalus pulchellus* | Tanzania | 35.146 | -5.499 | " |
| *Rhipicephalus pulchellus* | Tanzania | 36.136457 | -5.537666 | " |
| *Rhipicephalus pulchellus* | Tanzania | 37.174966 | -5.531624 | " |
| *Rhipicephalus pulchellus* | Tanzania | 38.141 | -5.626 | " |
| *Rhipicephalus pulchellus* | Tanzania | 38.908134 | -5.537455 | " |
| *Rhipicephalus pulchellus* | Tanzania | 38.871016 | -6.617019 | " |
| *Rhipicephalus pulchellus* | Tanzania | 34.991445 | -7.460333 | " |
| *Rhipicephalus pulchellus* | Uganda | 34.67765 | 2.508923 | (Akure 2019) |
| *Rhipicephalus pulchellus* | Uganda | 34.680008 | 2.579096 | " |
| *Rhipicephalus pulchellus* | Uganda | 34.10154 | 3.068379 | " |
| *Rhipicephalus pulchellus* | Uganda | 34.2 | 3.006914 | " |
| *Haemaphysalis longicornis* | China | 117.557602 | 30.733459 | (XiangGuang 2019) |
| *Haemaphysalis longicornis* | China | 118.483398 | 31.740162 | " |
| *Haemaphysalis longicornis* | China | 117.747002 | 30.874538 | " |
| *Haemaphysalis longicornis* | China | 121.038852 | 29.251920 | (JunHua 2010) |
| *Haemaphysalis longicornis* | China | 118.985955 | 28.944243 | (GuoPing 2021) |
| *Haemaphysalis longicornis* | China | 118.944552 | 28.969806 | " |
| *Haemaphysalis longicornis* | China | 119.190921 | 29.042698 | " |
| *Haemaphysalis longicornis* | China | 118.625824 | 28.717276 | " |
| *Haemaphysalis longicornis* | China | 118.498875 | 28.876892 | " |
| *Haemaphysalis longicornis* | China | 118.399325 | 29.140033 | " |
| *Haemaphysalis longicornis* | China | 122.054674 | 30.064728 | " |
| *Haemaphysalis longicornis* | China | 122.201268 | 30.275395 | " |
| *Haemaphysalis longicornis* | China | 122.465065 | 30.716683 | " |
| *Haemaphysalis longicornis* | China | 116.971642 | 31.991150 | (XiangGuang 2019) |
| *Haemaphysalis longicornis* | China | 118.258507 | 29.728170 | " |
| *Haemaphysalis longicornis* | China | 118.359383 | 31.400869 | " |
| *Haemaphysalis longicornis* | China | 103.370000 | 30.590000 | (Wang et al., 2020) |
| *Haemaphysalis longicornis* | China | 117.333701 | 34.820736 | (Zhang et al., 2017) |
| *Haemaphysalis longicornis* | China | 118.348302 | 35.198972 | " |
| *Haemaphysalis longicornis* | China | 119.345205 | 35.697194 | " |
| *Haemaphysalis longicornis* | China | 117.091617 | 36.213778 | " |
| *Haemaphysalis longicornis* | China | 120.185732 | 36.522013 | " |
| *Haemaphysalis longicornis* | China | 117.117776 | 36.610179 | " |
| *Haemaphysalis longicornis* | China | 119.122093 | 36.773728 | " |
| *Haemaphysalis longicornis* | China | 115.168275 | 30.984256 | (Xu et al., 2021) |
| *Haemaphysalis longicornis* | China | 115.559476 | 31.024503 | " |
| *Haemaphysalis longicornis* | China | 115.596768 | 31.122413 | " |
| *Haemaphysalis longicornis* | China | 114.842411 | 31.306332 | " |
| *Haemaphysalis longicornis* | China | 113.517063 | 32.075162 | " |
| *Haemaphysalis longicornis* | China | 113.291019 | 32.234148 | " |
| *Haemaphysalis longicornis* | China | 118.316546 | 29.717623 | (LiZhao 1993) |
| *Haemaphysalis longicornis* | China | 118.234414 | 29.786079 | " |
| *Haemaphysalis longicornis* | China | 118.375628 | 29.861913 | " |
| *Haemaphysalis longicornis* | China | 118.420884 | 29.864033 | " |
| *Haemaphysalis longicornis* | China | 116.287589 | 30.462602 | " |
| *Haemaphysalis longicornis* | China | 118.449433 | 30.696412 | " |
| *Haemaphysalis longicornis* | China | 118.330404 | 32.298891 | " |
| *Haemaphysalis longicornis* | China | 116.929581 | 34.217282 | (FengLin 2004) |
| *Haemaphysalis longicornis* | China | 117.298626 | 31.889006 | " |
| *Haemaphysalis longicornis* | China | 116.286439 | 30.465591 | " |
| *Haemaphysalis longicornis* | China | 118.146461 | 30.292621 | " |
| *Haemaphysalis longicornis* | China | 115.943744 | 31.736286 | " |
| *Haemaphysalis longicornis* | China | 118.328844 | 32.265027 | " |
| *Haemaphysalis longicornis* | China | 116.585458 | 30.617116 | (DanDan 2020) |
| *Haemaphysalis longicornis* | China | 116.943513 | 31.047658 | " |
| *Haemaphysalis longicornis* | China | 118.442309 | 32.215297 | " |
| *Haemaphysalis longicornis* | China | 117.978981 | 32.794739 | " |
| *Haemaphysalis longicornis* | China | 108.909257 | 25.755716 | (XiaoRong 1997) |
| *Haemaphysalis longicornis* | China | 107.516426 | 25.822953 | " |
| *Haemaphysalis longicornis* | China | 108.526633 | 25.947553 | " |
| *Haemaphysalis longicornis* | China | 106.390139 | 26.220085 | " |
| *Haemaphysalis longicornis* | China | 107.529577 | 26.270913 | " |
| *Haemaphysalis longicornis* | China | 107.983733 | 26.562998 | " |
| *Haemaphysalis longicornis* | China | 107.244724 | 26.591113 | " |
| *Haemaphysalis longicornis* | China | 106.643766 | 26.648674 | " |
| *Haemaphysalis longicornis* | China | 104.283487 | 26.853208 | " |
| *Haemaphysalis longicornis* | China | 108.229456 | 27.512675 | " |
| *Haemaphysalis longicornis* | China | 107.033469 | 27.698265 | " |
| *Haemaphysalis longicornis* | China | 109.195245 | 27.731724 | " |
| *Haemaphysalis longicornis* | China | 108.432976 | 28.002886 | " |
| *Haemaphysalis longicornis* | China | 106.856638 | 28.138449 | " |
| *Haemaphysalis longicornis* | China | 106.222327 | 28.347701 | " |
| *Haemaphysalis longicornis* | China | 105.708524 | 28.600178 | " |
| *Haemaphysalis longicornis* | China | 106.114393 | 25.177476 | (YuLong 2022) |
| *Haemaphysalis longicornis* | China | 105.227207 | 25.423931 | " |
| *Haemaphysalis longicornis* | China | 105.209676 | 25.837405 | " |
| *Haemaphysalis longicornis* | China | 106.597055 | 26.848214 | " |
| *Haemaphysalis longicornis* | China | 117.893426 | 41.078033 | (XueBin 2020) |
| *Haemaphysalis longicornis* | China | 114.561728 | 36.619446 | (ZhiKun 1995) |
| *Haemaphysalis longicornis* | China | 114.346091 | 37.898352 | " |
| *Haemaphysalis longicornis* | China | 115.483955 | 39.358708 | " |
| *Haemaphysalis longicornis* | China | 115.719680 | 39.417058 | " |
| *Haemaphysalis longicornis* | China | 117.507748 | 40.418702 | " |
| *Haemaphysalis longicornis* | China | 115.491438 | 38.921883 | (WanChun 1994) |
| *Haemaphysalis longicornis* | China | 115.520750 | 37.612906 | (XueBin 2020) |
| *Haemaphysalis longicornis* | China | 115.279070 | 37.951128 | " |
| *Haemaphysalis longicornis* | China | 117.228788 | 38.035108 | " |
| *Haemaphysalis longicornis* | China | 115.586632 | 38.087530 | " |
| *Haemaphysalis longicornis* | China | 117.503337 | 38.155247 | " |
| *Haemaphysalis longicornis* | China | 114.158640 | 38.224869 | " |
| *Haemaphysalis longicornis* | China | 117.394256 | 38.374327 | " |
| *Haemaphysalis longicornis* | China | 114.132284 | 38.891532 | " |
| *Haemaphysalis longicornis* | China | 115.630280 | 39.423747 | " |
| *Haemaphysalis longicornis* | China | 115.985174 | 39.516592 | " |
| *Haemaphysalis longicornis* | China | 118.681842 | 39.523848 | " |
| *Haemaphysalis longicornis* | China | 118.087775 | 39.574867 | " |
| *Haemaphysalis longicornis* | China | 119.165598 | 39.880083 | " |
| *Haemaphysalis longicornis* | China | 118.333318 | 40.160273 | " |
| *Haemaphysalis longicornis* | China | 117.537033 | 40.445529 | " |
| *Haemaphysalis longicornis* | China | 118.139634 | 40.732723 | " |
| *Haemaphysalis longicornis* | China | 114.235077 | 37.405556 | (Shuai 2013) |
| *Haemaphysalis longicornis* | China | 114.082186 | 38.027473 | " |
| *Haemaphysalis longicornis* | China | 114.111300 | 38.151100 | " |
| *Haemaphysalis longicornis* | China | 114.220500 | 38.182600 | " |
| *Haemaphysalis longicornis* | China | 115.073466 | 38.491226 | " |
| *Haemaphysalis longicornis* | China | 114.128834 | 38.519639 | " |
| *Haemaphysalis longicornis* | China | 115.307851 | 39.219229 | " |
| *Haemaphysalis longicornis* | China | 115.420436 | 39.233528 | " |
| *Haemaphysalis longicornis* | China | 115.277785 | 38.955216 | (ZongYi 1997) |
| *Haemaphysalis longicornis* | China | 117.744093 | 39.959587 | " |
| *Haemaphysalis longicornis* | China | 112.479503 | 34.159843 | (Qian 2022) |
| *Haemaphysalis longicornis* | China | 112.179465 | 34.541047 | " |
| *Haemaphysalis longicornis* | China | 112.946449 | 35.110678 | " |
| *Haemaphysalis longicornis* | China | 113.094768 | 35.169282 | " |
| *Haemaphysalis longicornis* | China | 116.885974 | 32.373161 | (LiJun 2018) |
| *Haemaphysalis longicornis* | China | 112.590558 | 33.061943 | " |
| *Haemaphysalis longicornis* | China | 111.640830 | 33.723215 | " |
| *Haemaphysalis longicornis* | China | 112.960694 | 35.032407 | " |
| *Haemaphysalis longicornis* | China | 112.923975 | 35.161321 | " |
| *Haemaphysalis longicornis* | China | 113.195959 | 35.229909 | " |
| *Haemaphysalis longicornis* | China | 112.845491 | 35.242625 | " |
| *Haemaphysalis longicornis* | China | 115.421559 | 31.775864 | (GuangZhi 2020) |
| *Haemaphysalis longicornis* | China | 114.021855 | 32.799193 | " |
| *Haemaphysalis longicornis* | China | 114.000611 | 33.127062 | " |
| *Haemaphysalis longicornis* | China | 112.979939 | 34.371635 | " |
| *Haemaphysalis longicornis* | China | 112.441297 | 34.797892 | " |
| *Haemaphysalis longicornis* | China | 112.510932 | 32.979844 | (Yan 2021) |
| *Haemaphysalis longicornis* | China | 113.996344 | 33.026560 | " |
| *Haemaphysalis longicornis* | China | 113.154973 | 33.768929 | " |
| *Haemaphysalis longicornis* | China | 113.504549 | 33.845411 | " |
| *Haemaphysalis longicornis* | China | 115.633523 | 34.394244 | " |
| *Haemaphysalis longicornis* | China | 112.487595 | 34.561979 | " |
| *Haemaphysalis longicornis* | China | 113.573062 | 34.683179 | " |
| *Haemaphysalis longicornis* | China | 114.380818 | 36.052851 | " |
| *Haemaphysalis longicornis* | China | 114.140746 | 32.084689 | (Yang 2012) |
| *Haemaphysalis longicornis* | China | 112.491583 | 35.027046 | " |
| *Haemaphysalis longicornis* | China | 114.839816 | 31.641132 | (Qi 2015) |
| *Haemaphysalis longicornis* | China | 115.396263 | 31.773162 | " |
| *Haemaphysalis longicornis* | China | 114.916047 | 31.989696 | " |
| *Haemaphysalis longicornis* | China | 114.151670 | 32.080161 | " |
| *Haemaphysalis longicornis* | China | 114.035402 | 32.124755 | " |
| *Haemaphysalis longicornis* | China | 113.543700 | 32.136900 | " |
| *Haemaphysalis longicornis* | China | 113.373243 | 32.374520 | " |
| *Haemaphysalis longicornis* | China | 114.013322 | 32.963526 | " |
| *Haemaphysalis longicornis* | China | 111.834149 | 33.041277 | " |
| *Haemaphysalis longicornis* | China | 114.005641 | 33.125610 | " |
| *Haemaphysalis longicornis* | China | 114.001904 | 33.127062 | " |
| *Haemaphysalis longicornis* | China | 113.565041 | 33.280359 | " |
| *Haemaphysalis longicornis* | China | 111.471485 | 33.293038 | " |
| *Haemaphysalis longicornis* | China | 113.595511 | 33.441649 | " |
| *Haemaphysalis longicornis* | China | 114.040989 | 33.613383 | " |
| *Haemaphysalis longicornis* | China | 114.633458 | 33.682804 | " |
| *Haemaphysalis longicornis* | China | 113.276854 | 33.691498 | " |
| *Haemaphysalis longicornis* | China | 112.937142 | 33.723744 | " |
| *Haemaphysalis longicornis* | China | 111.662241 | 33.776099 | " |
| *Haemaphysalis longicornis* | China | 113.078554 | 33.853043 | " |
| *Haemaphysalis longicornis* | China | 113.829905 | 34.121082 | " |
| *Haemaphysalis longicornis* | China | 112.099886 | 34.134388 | " |
| *Haemaphysalis longicornis* | China | 113.513190 | 34.167026 | " |
| *Haemaphysalis longicornis* | China | 112.584700 | 34.295900 | " |
| *Haemaphysalis longicornis* | China | 115.615288 | 34.449947 | " |
| *Haemaphysalis longicornis* | China | 115.084011 | 34.456855 | " |
| *Haemaphysalis longicornis* | China | 113.066912 | 34.479339 | " |
| *Haemaphysalis longicornis* | China | 112.111483 | 34.507976 | " |
| *Haemaphysalis longicornis* | China | 112.428423 | 34.674156 | " |
| *Haemaphysalis longicornis* | China | 112.159722 | 34.714091 | " |
| *Haemaphysalis longicornis* | China | 112.812016 | 34.734999 | " |
| *Haemaphysalis longicornis* | China | 114.025314 | 34.758945 | " |
| *Haemaphysalis longicornis* | China | 114.766883 | 34.912666 | " |
| *Haemaphysalis longicornis* | China | 112.653565 | 35.081416 | " |
| *Haemaphysalis longicornis* | China | 114.204796 | 35.134553 | " |
| *Haemaphysalis longicornis* | China | 114.217264 | 35.139187 | " |
| *Haemaphysalis longicornis* | China | 113.093762 | 35.170227 | " |
| *Haemaphysalis longicornis* | China | 113.469533 | 35.242413 | " |
| *Haemaphysalis longicornis* | China | 113.815595 | 35.508632 | " |
| *Haemaphysalis longicornis* | China | 114.516929 | 35.581514 | " |
| *Haemaphysalis longicornis* | China | 114.095040 | 35.624694 | " |
| *Haemaphysalis longicornis* | China | 115.044783 | 35.739807 | " |
| *Haemaphysalis longicornis* | China | 114.275267 | 35.749177 | " |
| *Haemaphysalis longicornis* | China | 115.089348 | 35.886397 | " |
| *Haemaphysalis longicornis* | China | 114.293988 | 35.906948 | " |
| *Haemaphysalis longicornis* | China | 114.820485 | 36.001015 | " |
| *Haemaphysalis longicornis* | China | 114.824797 | 36.013861 | " |
| *Haemaphysalis longicornis* | China | 114.500014 | 36.070422 | " |
| *Haemaphysalis longicornis* | China | 113.743094 | 36.073219 | " |
| *Haemaphysalis longicornis* | China | 115.183787 | 36.090732 | " |
| *Haemaphysalis longicornis* | China | 131.844623 | 45.536953 | (DongHui 2006) |
| *Haemaphysalis longicornis* | China | 132.960713 | 45.753252 | " |
| *Haemaphysalis longicornis* | China | 111.791398 | 30.180338 | (YiRen 2001) |
| *Haemaphysalis longicornis* | China | 108.870613 | 30.303191 | " |
| *Haemaphysalis longicornis* | China | 113.126424 | 31.021128 | " |
| *Haemaphysalis longicornis* | China | 110.760629 | 31.344842 | " |
| *Haemaphysalis longicornis* | China | 115.435481 | 30.900416 | (ZhiYu 2021) |
| *Haemaphysalis longicornis* | China | 115.463659 | 30.927472 | " |
| *Haemaphysalis longicornis* | China | 113.329894 | 32.276860 | " |
| *Haemaphysalis longicornis* | China | 114.622490 | 31.290224 | (JinLin 1994) |
| *Haemaphysalis longicornis* | China | 111.141349 | 30.767972 | (ZhiJin 2002) |
| *Haemaphysalis longicornis* | China | 109.560160 | 26.214055 | (XiCheng 1997) |
| *Haemaphysalis longicornis* | China | 109.822971 | 28.224338 | " |
| *Haemaphysalis longicornis* | China | 109.624546 | 28.238895 | " |
| *Haemaphysalis longicornis* | China | 109.784087 | 28.251531 | " |
| *Haemaphysalis longicornis* | China | 109.839465 | 28.303894 | " |
| *Haemaphysalis longicornis* | China | 109.931031 | 28.306086 | " |
| *Haemaphysalis longicornis* | China | 109.666955 | 28.313193 | " |
| *Haemaphysalis longicornis* | China | 109.595913 | 28.332745 | " |
| *Haemaphysalis longicornis* | China | 109.592050 | 28.345600 | " |
| *Haemaphysalis longicornis* | China | 109.983621 | 28.359594 | " |
| *Haemaphysalis longicornis* | China | 109.720241 | 28.367430 | " |
| *Haemaphysalis longicornis* | China | 109.812211 | 28.409150 | " |
| *Haemaphysalis longicornis* | China | 109.869858 | 28.424264 | " |
| *Haemaphysalis longicornis* | China | 121.042811 | 29.710163 | (ShaoJun 2020) |
| *Haemaphysalis longicornis* | China | 121.144739 | 29.847580 | " |
| *Haemaphysalis longicornis* | China | 121.105581 | 30.004811 | " |
| *Haemaphysalis longicornis* | China | 121.227232 | 30.048866 | " |
| *Haemaphysalis longicornis* | China | 121.010451 | 30.139024 | " |
| *Haemaphysalis longicornis* | China | 127.347742 | 43.720310 | (HuanHuan 2018) |
| *Haemaphysalis longicornis* | China | 131.008201 | 44.379408 | " |
| *Haemaphysalis longicornis* | China | 132.514741 | 47.645736 | " |
| *Haemaphysalis longicornis* | China | 128.908485 | 47.749026 | " |
| *Haemaphysalis longicornis* | China | 129.400729 | 42.723653 | (JiXu 2017) |
| *Haemaphysalis longicornis* | China | 130.367225 | 42.866664 | " |
| *Haemaphysalis longicornis* | China | 129.486885 | 42.879743 | " |
| *Haemaphysalis longicornis* | China | 129.842049 | 42.998763 | " |
| *Haemaphysalis longicornis* | China | 130.412205 | 42.820546 | (LongZheng 2015) |
| *Haemaphysalis longicornis* | China | 129.641642 | 42.923719 | " |
| *Haemaphysalis longicornis* | China | 129.207389 | 43.114206 | " |
| *Haemaphysalis longicornis* | China | 129.228631 | 43.117911 | " |
| *Haemaphysalis longicornis* | China | 129.178385 | 43.167804 | " |
| *Haemaphysalis longicornis* | China | 126.176872 | 41.163896 | (Bo 2020) |
| *Haemaphysalis longicornis* | China | 127.454003 | 42.230861 | " |
| *Haemaphysalis longicornis* | China | 125.754157 | 42.285642 | " |
| *Haemaphysalis longicornis* | China | 126.064018 | 42.700029 | " |
| *Haemaphysalis longicornis* | China | 126.085586 | 42.949389 | " |
| *Haemaphysalis longicornis* | China | 128.235591 | 43.386469 | " |
| *Haemaphysalis longicornis* | China | 119.823561 | 31.341723 | (Jun 1998) |
| *Haemaphysalis longicornis* | China | 119.481155 | 31.402872 | " |
| *Haemaphysalis longicornis* | China | 119.559442 | 31.733807 | " |
| *Haemaphysalis longicornis* | China | 119.194406 | 31.957542 | " |
| *Haemaphysalis longicornis* | China | 118.626785 | 32.100875 | " |
| *Haemaphysalis longicornis* | China | 118.518818 | 33.033754 | " |
| *Haemaphysalis longicornis* | China | 117.515079 | 34.145052 | " |
| *Haemaphysalis longicornis* | China | 119.280653 | 34.610331 | " |
| *Haemaphysalis longicornis* | China | 120.514512 | 31.291442 | (ZhenHong 2020) |
| *Haemaphysalis longicornis* | China | 120.919323 | 31.396288 | " |
| *Haemaphysalis longicornis* | China | 120.724715 | 31.666178 | " |
| *Haemaphysalis longicornis* | China | 120.599680 | 31.150702 | (XueLiang 2019) |
| *Haemaphysalis longicornis* | China | 120.606309 | 31.258877 | " |
| *Haemaphysalis longicornis* | China | 120.544120 | 31.305666 | " |
| *Haemaphysalis longicornis* | China | 120.923060 | 31.395795 | " |
| *Haemaphysalis longicornis* | China | 121.104123 | 31.468666 | " |
| *Haemaphysalis longicornis* | China | 120.727589 | 31.661506 | " |
| *Haemaphysalis longicornis* | China | 120.537697 | 31.850719 | " |
| *Haemaphysalis longicornis* | China | 114.772194 | 24.936410 | (Qian 2019) |
| *Haemaphysalis longicornis* | China | 115.381108 | 25.960363 | " |
| *Haemaphysalis longicornis* | China | 115.431890 | 25.055574 | (XiaoQing 2019) |
| *Haemaphysalis longicornis* | China | 115.412567 | 27.749853 | " |
| *Haemaphysalis longicornis* | China | 114.379291 | 27.815744 | " |
| *Haemaphysalis longicornis* | China | 114.944523 | 27.850923 | " |
| *Haemaphysalis longicornis* | China | 115.876658 | 28.616931 | " |
| *Haemaphysalis longicornis* | China | 117.066752 | 28.688393 | " |
| *Haemaphysalis longicornis* | China | 115.368694 | 28.850885 | " |
| *Haemaphysalis longicornis* | China | 116.677795 | 29.006146 | " |
| *Haemaphysalis longicornis* | China | 117.865570 | 29.231348 | " |
| *Haemaphysalis longicornis* | China | 116.201064 | 29.260062 | " |
| *Haemaphysalis longicornis* | China | 115.073304 | 29.287178 | " |
| *Haemaphysalis longicornis* | China | 114.852826 | 24.935623 | (HongBin 2017) |
| *Haemaphysalis longicornis* | China | 115.786703 | 25.611258 | " |
| *Haemaphysalis longicornis* | China | 116.774201 | 27.929670 | " |
| *Haemaphysalis longicornis* | China | 114.319312 | 28.561296 | " |
| *Haemaphysalis longicornis* | China | 117.600453 | 28.936069 | " |
| *Haemaphysalis longicornis* | China | 117.014057 | 28.255097 | " |
| *Haemaphysalis longicornis* | China | 115.588501 | 28.840164 | " |
| *Haemaphysalis longicornis* | China | 115.392750 | 25.972187 | " |
| *Haemaphysalis longicornis* | China | 115.396245 | 25.117358 | " |
| *Haemaphysalis longicornis* | China | 114.318305 | 25.660854 | " |
| *Haemaphysalis longicornis* | China | 110.330704 | 31.441829 | (MingShe 2005) |
| *Haemaphysalis longicornis* | China | 124.041376 | 40.428431 | (YingZi 2013) |
| *Haemaphysalis longicornis* | China | 121.811808 | 39.272589 | (DongHai 2021) |
| *Haemaphysalis longicornis* | China | 123.793392 | 40.103602 | " |
| *Haemaphysalis longicornis* | China | 123.859381 | 40.318958 | " |
| *Haemaphysalis longicornis* | China | 124.111766 | 40.349403 | " |
| *Haemaphysalis longicornis* | China | 124.296329 | 40.350746 | " |
| *Haemaphysalis longicornis* | China | 123.880218 | 40.370229 | " |
| *Haemaphysalis longicornis* | China | 124.076904 | 40.447107 | " |
| *Haemaphysalis longicornis* | China | 124.482682 | 40.504806 | " |
| *Haemaphysalis longicornis* | China | 123.932592 | 40.530627 | " |
| *Haemaphysalis longicornis* | China | 124.258546 | 40.546077 | " |
| *Haemaphysalis longicornis* | China | 124.567264 | 40.610995 | " |
| *Haemaphysalis longicornis* | China | 123.997398 | 40.671493 | " |
| *Haemaphysalis longicornis* | China | 124.874981 | 40.729718 | " |
| *Haemaphysalis longicornis* | China | 123.929805 | 40.801944 | " |
| *Haemaphysalis longicornis* | China | 124.454535 | 40.907455 | " |
| *Haemaphysalis longicornis* | China | 125.012635 | 40.978234 | " |
| *Haemaphysalis longicornis* | China | 124.265062 | 40.983182 | " |
| *Haemaphysalis longicornis* | China | 125.368315 | 41.278931 | " |
| *Haemaphysalis longicornis* | China | 123.707351 | 42.215669 | " |
| *Haemaphysalis longicornis* | China | 124.773181 | 40.734494 | (Zhuo 2018) |
| *Haemaphysalis longicornis* | China | 124.161794 | 41.337377 | " |
| *Haemaphysalis longicornis* | China | 125.492808 | 41.018864 | (ChunMei 2000) |
| *Haemaphysalis longicornis* | China | 122.975105 | 39.677432 | (WeiXian 1987) |
| *Haemaphysalis longicornis* | China | 123.327357 | 40.304202 | " |
| *Haemaphysalis longicornis* | China | 120.343959 | 40.356096 | " |
| *Haemaphysalis longicornis* | China | 124.094124 | 40.438206 | " |
| *Haemaphysalis longicornis* | China | 120.767232 | 40.634899 | " |
| *Haemaphysalis longicornis* | China | 120.837362 | 40.700738 | " |
| *Haemaphysalis longicornis* | China | 124.774043 | 40.734056 | " |
| *Haemaphysalis longicornis* | China | 123.429240 | 40.992429 | " |
| *Haemaphysalis longicornis* | China | 125.297261 | 41.254596 | " |
| *Haemaphysalis longicornis* | China | 121.247582 | 41.555838 | " |
| *Haemaphysalis longicornis* | China | 121.730557 | 41.608668 | " |
| *Haemaphysalis longicornis* | China | 125.413026 | 41.611930 | " |
| *Haemaphysalis longicornis* | China | 125.367046 | 41.616999 | " |
| *Haemaphysalis longicornis* | China | 125.460182 | 41.624333 | " |
| *Haemaphysalis longicornis* | China | 124.890101 | 42.107967 | " |
| *Haemaphysalis longicornis* | China | 120.363219 | 40.345264 | (Zhen 2017) |
| *Haemaphysalis longicornis* | China | 120.768292 | 40.639771 | " |
| *Haemaphysalis longicornis* | China | 121.111821 | 41.122447 | " |
| *Haemaphysalis longicornis* | China | 121.349172 | 41.169856 | " |
| *Haemaphysalis longicornis* | China | 121.247295 | 41.555352 | " |
| *Haemaphysalis longicornis* | China | 120.442062 | 36.133631 | (KaiSan 1996) |
| *Haemaphysalis longicornis* | China | 119.922770 | 37.210158 | (Qiang 1996) |
| *Haemaphysalis longicornis* | China | 117.955876 | 35.561322 | (LiJuan 2007) |
| *Haemaphysalis longicornis* | China | 121.409653 | 37.516759 | (LiJuan 1997) |
| *Haemaphysalis longicornis* | China | 118.381934 | 35.273856 | (JuFeng 2015) |
| *Haemaphysalis longicornis* | China | 115.507671 | 35.321323 | " |
| *Haemaphysalis longicornis* | China | 120.567969 | 36.157131 | " |
| *Haemaphysalis longicornis* | China | 117.104552 | 36.607667 | " |
| *Haemaphysalis longicornis* | China | 116.142553 | 36.679661 | " |
| *Haemaphysalis longicornis* | China | 119.123854 | 36.772398 | " |
| *Haemaphysalis longicornis* | China | 118.083382 | 37.133481 | " |
| *Haemaphysalis longicornis* | China | 121.968868 | 37.205739 | " |
| *Haemaphysalis longicornis* | China | 116.357935 | 37.452653 | " |
| *Haemaphysalis longicornis* | China | 118.184891 | 36.194877 | (XiangYe 2015) |
| *Haemaphysalis longicornis* | China | 106.263478 | 32.839021 | (YinShu 2008) |
| *Haemaphysalis longicornis* | China | 107.021809 | 33.058914 | " |
| *Haemaphysalis longicornis* | China | 121.730727 | 31.059957 | (ZiXin 2022) |
| *Haemaphysalis longicornis* | China | 121.190782 | 31.066833 | " |
| *Haemaphysalis longicornis* | China | 121.427181 | 31.228267 | " |
| *Haemaphysalis longicornis* | China | 121.488355 | 31.691024 | " |
| *Haemaphysalis longicornis* | China | 123.589861 | 41.503323 | (Na 2014) |
| *Haemaphysalis longicornis* | China | 123.649414 | 41.941942 | " |
| *Haemaphysalis longicornis* | China | 123.331903 | 42.732869 | " |
| *Haemaphysalis longicornis* | China | 117.907099 | 35.255328 | (LiJuan 2000) |
| *Haemaphysalis longicornis* | China | 105.914891 | 31.727444 | " |
| *Haemaphysalis longicornis* | China | 120.384056 | 31.287941 | (DenGan 2018) |
| *Haemaphysalis longicornis* | China | 120.500713 | 31.297888 | " |
| *Haemaphysalis longicornis* | China | 120.352761 | 31.300731 | " |
| *Haemaphysalis longicornis* | China | 120.482962 | 31.330959 | " |
| *Haemaphysalis longicornis* | China | 120.473261 | 31.347553 | " |
| *Haemaphysalis longicornis* | China | 121.614154 | 38.896492 | (GuoPing 2008) |
| *Haemaphysalis longicornis* | China | 121.992825 | 39.618846 | " |
| *Haemaphysalis longicornis* | China | 122.975069 | 39.677515 | " |
| *Haemaphysalis longicornis* | China | 124.224145 | 40.010608 | " |
| *Haemaphysalis longicornis* | China | 123.309966 | 40.285437 | " |
| *Haemaphysalis longicornis* | China | 120.362788 | 40.345374 | " |
| *Haemaphysalis longicornis* | China | 123.967211 | 40.433044 | " |
| *Haemaphysalis longicornis* | China | 122.359325 | 40.443295 | " |
| *Haemaphysalis longicornis* | China | 120.758518 | 40.651596 | " |
| *Haemaphysalis longicornis* | China | 122.221345 | 40.657856 | " |
| *Haemaphysalis longicornis* | China | 120.838512 | 40.702925 | " |
| *Haemaphysalis longicornis* | China | 124.769156 | 40.733072 | " |
| *Haemaphysalis longicornis* | China | 121.164794 | 41.107456 | " |
| *Haemaphysalis longicornis* | China | 121.348885 | 41.171920 | " |
| *Haemaphysalis longicornis* | China | 125.404051 | 41.310217 | " |
| *Haemaphysalis longicornis* | China | 121.598146 | 41.401102 | " |
| *Haemaphysalis longicornis* | China | 121.248157 | 41.555676 | " |
| *Haemaphysalis longicornis* | China | 122.170294 | 41.646993 | " |
| *Haemaphysalis longicornis* | China | 102.780658 | 24.897416 | (KaiFei 2016) |
| *Haemaphysalis longicornis* | China | 102.635497 | 24.974044 | " |
| *Haemaphysalis longicornis* | China | 100.340196 | 25.185317 | " |
| *Haemaphysalis longicornis* | China | 100.156438 | 25.652783 | " |
| *Haemaphysalis longicornis* | China | 100.785144 | 26.686367 | " |
| *Haemaphysalis longicornis* | China | 103.709017 | 27.310711 | " |
| *Haemaphysalis longicornis* | China | 103.920652 | 27.733687 | " |
| *Haemaphysalis longicornis* | China | 119.928667 | 28.495251 | (Ting 2015) |
| *Haemaphysalis longicornis* | China | 120.688289 | 28.894724 | " |
| *Haemaphysalis longicornis* | China | 120.241121 | 29.375378 | " |
| *Haemaphysalis longicornis* | China | 121.943688 | 29.472461 | " |
| *Haemaphysalis longicornis* | China | 122.149813 | 30.278140 | " |
| *Haemaphysalis longicornis* | China | 119.645742 | 30.605143 | " |
| *Haemaphysalis longicornis* | China | 119.216109 | 29.503287 | (Zhe 2014) |
| *Haemaphysalis longicornis* | China | 119.930146 | 28.494613 | (YuQing 2015) |
| *Haemaphysalis longicornis* | China | 119.650521 | 27.540135 | (Feng 2013) |
| *Haemaphysalis longicornis* | China | 120.688522 | 28.126126 | " |
| *Haemaphysalis longicornis* | China | 120.770789 | 28.835508 | " |
| *Haemaphysalis longicornis* | China | 119.712378 | 29.116252 | " |
| *Haemaphysalis longicornis* | China | 121.027066 | 29.168945 | " |
| *Haemaphysalis longicornis* | China | 120.116077 | 29.279147 | " |
| *Haemaphysalis longicornis* | China | 122.170223 | 30.294107 | " |
| *Haemaphysalis longicornis* | China | 120.710998 | 30.538082 | " |
| *Haemaphysalis longicornis* | China | 120.899264 | 30.538343 | " |
| *Haemaphysalis longicornis* | China | 119.645455 | 30.604895 | " |
| *Haemaphysalis longicornis* | China | 120.323307 | 28.126885 | (LiPing 2006) |
| *Haemaphysalis longicornis* | China | 119.712665 | 29.115999 | " |
| *Haemaphysalis longicornis* | China | 118.597653 | 28.771494 | (GuoPing 2021) |
| *Haemaphysalis longicornis* | China | 118.484502 | 28.872843 | " |
| *Haemaphysalis longicornis* | China | 118.860336 | 28.952714 | " |
| *Haemaphysalis longicornis* | China | 118.979478 | 28.998622 | " |
| *Haemaphysalis longicornis* | China | 119.241945 | 29.083235 | " |
| *Haemaphysalis longicornis* | China | 118.396738 | 29.103558 | " |
| *Haemaphysalis longicornis* | China | 120.023315 | 29.165663 | (XinHong 2022) |
| *Haemaphysalis longicornis* | China | 120.076848 | 35.922344 | (GuangZhi 2021) |
| *Haemaphysalis longicornis* | China | 118.184675 | 36.193945 | " |
| *Haemaphysalis longicornis* | China | 117.055882 | 36.195037 | " |
| *Haemaphysalis longicornis* | China | 117.075043 | 36.682067 | " |

**References**

Akure, C. P. (2019). Tick species composition and associated haemoparasites of cattle in a semi-arid area of Karamoja, Uganda, University of Pretoria.

Bandaranayaka, K. O., U. I. Dissanayake&R. S. Rajakaruna.(2022) Diversity and geographic distribution of dog tick species in Sri Lanka and the life cycle of brown dog tick, Rhipicephalus sanguineus under laboratory conditions.

BenHua, Z.(1964) Studies on the infestation of ticks of domestic animals in NanKing(in chinese). *ACTA ZOOLOGICA SINICA*, *16*(1), 132-138. CNKI:SUN:BEAR.0.1964-01-013

Bo, S.(2020) An investigation of tick species in Changbai mountain area of Jilin province,China(in chinese). *Chinese Journal of Vector Biology and Control*, *31*(6), 667-671. 10.11853/j.issn.1003.8280.2020.06.008

Byaruhanga, C., N. Collins, D. Knobel, W. Kabasa&M. Oosthuizen.(2015) Endemic status of tick-borne infections and tick species diversity among transhumant zebu cattle in Karamoja region, Uganda: support for control approaches. *Veterinary Parasitology: Regional Studies and Reports*, *1*, 21-30

Chao, L.-L., C.-K. Hsieh, T.-Y. Ho&C.-M. Shih.(2019) First zootiological survey of hard ticks (Acari: Ixodidae) infesting dogs in northern Taiwan. *Experimental and Applied Acarology*, *77*(1), 105-115

ChunMei, W.(2000) Investigation of Parasitic Ticks of Surfaces on Cow from Huanren County Liaoning Province in China(in chinese). *Journal of Medical Pest Control*, *16*(10), 525-526. CNKI:SUN:YXDZ.0.2000-10-008

Cumming, G.(1999) Host distributions do not limit the species ranges of most African ticks (Acari: Ixodida). *Bulletin of Entomological Research*, *89*(4), 303-327

DanDan, S.(2020) Tick distribution in some epidemic areas of fever with thrombocytopenia in Anhui Province(in chinese ). *Anhui Journal of Preventive Medicine*, *26*(4), 267-280. 10.19837/j.cnki.ahyf.2020.04.006

DaoMao, P.(1982) NOTES ON LXODID TICKS FROM SOUTHWESTERN YUNNA WITH DESCRIPTION OF A NEW SPECIES OF HAEMPHYSALIS (ACARINA. LXODIDAE)(in chinese). *ZOOLOGICAL RESEARCH*, *3*, 45-51

DenGan, G.(2018) Survey of tick distribution and tick-borne pathogens in hilly scenic spots of Suzhou， China(in chinese). *Shanghai Journal of Preventive Medicine*, *30*(8), 652-655. 10.19428/j.cnki.sjpm.2018.18774

DeSheng, H.(2003) List of Livestock and Poultry Parasites in Yunnan Province (5)(in chinese). *Yunnan Journal of Animal Science and Veterinary Medicine*(1), 9-13. 10.3969/j.issn.1005-1341.2003.01.005

DingWei, S.(2013) Investigation of species of Ixodidae and a new record species of Ixodidae in Hainan province, China(in chinese). *Chinese Journal of Vector Biology and Control*, *24*(5), 442-443. 10.11853/j.issn.1003.4692.2013.05.018

Diyes, G.&R. Rajakaruna.(2015) Diversity and distribution of tick species infesting goats with two new host records from Sri Lanka. *Journal of the National Science Foundation of Sri Lanka*, *43*(3)

DongHai, Y.(2021) Investigation on the distribution of ticks and their main pathogens in some areas of Liaoning Province(in chinese). *Shenyang Agricultural University*(4), 1-54. 10.27327/d.cnki.gshnu.2020.000923

DongHui, F.(2006) A study on the population structure of ticks and their infected status by Lyme disease spirochetea at the 7 ports along the east of Heilongjiang Province(in chinese). *Port Health Control*, *11*(5), 40-42. 10.3969/j.issn.1008-5777.2006.05.014

Feng, L.(2013) Surveillance and study of tick⁃borne diseases in Zhejiang province, China(in chinese). *Chinese Journal of Vector Biology and Control*, *24*(1), 19-23. CNKI:SUN:ZMSK.0.2013-01-009

FengLin, L.(2004) List of Livestock and Poultry Parasites in Anhui Province (4) (6 Acarids and Insects <Part 1>)(in chinese). *Chinese Journal of Veterinary Parasitology*, *12*(3), 27-33. CNKI:SUN:ZSJB.0.2004-03-011

FengLin, L.(2004) List of Livestock and Poultry Parasites in Anhui Province (4) (6 Acarids and Insects <Part 2>)(in chinese). *Chinese Journal of Veterinary Parasitology*, *12*(4), 18-23. CNKI:SUN:ZSJB.0.2004-03-011

Geevarghese, G.&A. Mishra (2011). Haemaphysalis ticks of India, Elsevier.

Ghosh, S., P. Azhahianambi&J. de la Fuente.(2006) Control of ticks of ruminants, with special emphasis on livestock farming systems in India: present and future possibilities for integrated control—a review. *Experimental & applied acarology*, *40*(1), 49-66

Ghosh, S., G. C. Bansal, S. C. Gupta, D. Ray, M. Q. Khan, H. Irshad, M. Shahiduzzaman, U. Seitzer&J. S. Ahmed.(2007) Status of tick distribution in Bangladesh, India and Pakistan. *Parasitology research*, *101*(2), 207-216

GuangZhi, Z.(2020) SURVEY ON TICK DENSITY OF MILITARY TRAINING FIELDS IN HENAN PROVINCE(in chinese). *Acta Parasitologica Et Medica Entomologica Sinica*, *27*(2). 10.3969/j.issn.1005-0507.2020.02.008

GuangZhi, Z.(2021) Survey on the distribution of ticks in the field training area of troops stationed in Shandong Province(in chinese). *Chinese Journal of Hygienic Insecticides & Equipments*, *27*(2), 139-141. 10.19821/j.1671-2781.2021.02.013

GuoHou, L.(1995) A Survey of lxodidae distribution in Guangxi(in chinese). *Chinese Journal of Vector Biology and Control*, *6*(1), 14-16. CNKI:SUN:ZMSK.0.1995-01-007

GuoPing, C.(2021) Status of tick distribution and tick-borne pathogens in urban parks of Quzhou, Zhejiang, 2017–2019(in chinese). *Disease Surveillance*, *36*(9), 879-883. 10.3784/jbjc.202106010314

GuoPing, L.(2008) Distribution and medical importance of ticks in three provinces of northeast China(in chinese). *Chinese Journal of Hygienic Insecticides & Equipments*(1), 39-42. 10.3969/j.issn.1671-2781.2008.01.016

HaiBin, M.(2010) Distribution Survey of Ticks and Its Rodents in the Mohan Port of MengLa in Yunnan Province(in chinese). *Journal of Medical Pest Control*, *26*(8), 719-720. 10.3969/j.issn.1003-6245.2010.08.014

HaoRong, L.(1995) An Investigation on Ticks in Fujian(in chinese). *ENDEMIC DISEASES BULLETIN*, *10*(1), 50-52. 10.13215/ j. cnki.jbyfkztb. 1995.01.022

HongBin, X.(2017) Six newly recorded species of Ixodidae in Jiangxi Province，China(in chinese). *JOURNAL OF CENTRAL CHINA NORMAL UNIVERSITY(Nat.Sci.)*, *51*(6), 804-808. 10.19603/j.cnki.1000-1190.2017.06.014

HongXiang, Y.(2016) Tick species and genetic variants analysis of tick gene in Hengduan Mountains，west Yunnan Province，China(in chinese). *Chinese Journal of Zoonoses*, *32*(10), 865-870. 10.3969/j.issn.1002-2694.2016.010.003

HuanHuan, L.(2018) Survey on ticks species distribution and molecular epidemiology of three associated tick-borne microorganism in Jilin and Heilongjiang province (in chinese). *Jilin Agricultural University*(2), 1-65. CNKI:CDMD:2.1017.842699

Isack, I. K., M. Walter, C. Sebastian, K. Marja, H. Seong-Gu&S. Martin.(2017) Abundance and distribution of Ixodid tick species infesting cattle reared under traditional farming systems in Tanzania. *African Journal of Agricultural Research*, *12*(4), 286-299

JieNan, L.(2021) Survey of ticks and severe fever with thrombocytopenia syndrome in Zhoushan, Zhejiang, 2018–2020(in chinese). *Disease Surveillance*, *36*(9), 920-925. 10.3784/jbjc.202106200346

JinLin, Z.(1994) Investigation and Research on the Species and Seasonal Dynamics of Cattle Crickets in Hong'an County, Hubei Province(in chinese). *Chinese Journal of Veterinary Parasitology*, *2*(3), 39-40. CNKI:SUN:ZSJB.0.1994-03-017

JiXu, L.(2017) TICKS AND THEIR POTENTIAL INFECTION WITH SEVERE FEVER WITH THROMBOCYTOPENIA SYNDROMES VIRUS IN YANBIAN, JILIN(in chinese). *Acta Parasitologica Et Medica Entomologica Sinica*, *24*(2), 132-140. 10.3969/j.issn.1005-0507.2017.02.010

Jongejan, F., B.-L. Su, H.-J. Yang, L. Berger, J. Bevers, P.-C. Liu, J.-C. Fang, Y.-W. Cheng, C. Kraakman&N. Plaxton.(2018) Molecular evidence for the transovarial passage of Babesia gibsoni in Haemaphysalis hystricis (Acari: Ixodidae) ticks from Taiwan: a novel vector for canine babesiosis. *Parasites & vectors*, *11*(1), 1-8

JuFeng, Y.(2015) ldentification of tick species and epidemiological survey of ovine theileria parasiteinfection in Shandong province(in chinese). *Chinese Journal of Veterinary Science*, *35*(6), 930-937. 10.16303/j.cnki.1005-4545.2015.06.17

Jun, S.(1998) Investigation on Species Composition,Distribution , Wax and Wane of Ixodoidea in JiangSu(in chinese). *Medical Animal Control*, *14*(6), 39-40. CNKI:SUN:YXDZ.0.1998-06-013

Jun, S.(2000) Studies on Control and Ecology of Vectors of Lyme Disease in Jiangsu Province(in chinese). *Chinese Journal of Vector Biology and Control*, *11*(6), 458-461. 10.3969/j.issn.1003-4692.2000.06.018

JunHua, G.(2010) Surveillance of tick-borne infectiou disease in Tiantai of Zhejiang province(in chinese). *Disease Surveillance*, *25*(8), 635-637. 10.3784/j.issn.1003-9961.2010.08.015

Kaba, T.(2022) Geographical distribution of ixodid ticks and tick-borne pathogens of domestic animals in Ethiopia: a systematic review. *Parasites & Vectors*, *15*(1), 1-26

KaiFei, G.(2016) DISTRIBUTION AND FAUNA ANALYSIS OF TICKS IN YUNNAN PROVINCE, CHINA(in chinese). *Southwest Forestry University*(9), 1-74

KaiSan, W.(1996) Investigation and Analysis of the Transmitter of Theileria thurskyi in Qingdao City--Haemophora longhorn(in chinese). *Shangdong Journal of Animal Science and Veterinary Medicine*(3), 12. CNKI:SUN:DCMY.0.1996-03-004

Kaiser, M., R. Sutherst&A. Bourne.(1991) Tick (Acarina: Ixodidae) infestations on zebu cattle in northern Uganda. *Bulletin of Entomological Research*, *81*(3), 257-262

Kirung'o, M. N. (2012). Species Distribution Model: A Case Study of Brown Ear Tick (Rhipicephalus appendiculatus) in Kenya.

Kumar, K. A., R. Ravindran, J. Johns, G. Chandy, K. Rajagopal, L. Chandrasekhar, A. J. George&S. Ghosh.(2018) Ixodid tick vectors of wild mammals and reptiles of southern India. *Journal of Arthropod-borne diseases*, *12*(3), 276

Kuo, C.-C., C. Huang&H. C. Wang.(2011) Identification of potential hosts and vectors of scrub typhus and tick‐borne spotted fever group rickettsiae in eastern Taiwan. *Medical and veterinary entomology*, *25*(2), 169-177

Kuo, C.-C., Y.-F. Lin, C.-T. Yao, H.-C. Shih, L.-H. Chung, H.-C. Liao, Y.-C. Hsu&H.-C. Wang.(2017) Tick-borne pathogens in ticks collected from birds in Taiwan. *Parasites & vectors*, *10*(1), 1-13

Kuo, C.-C., P.-Y. Shu, J.-J. Mu, P.-L. Lee, Y.-W. Wu, C.-K. Chung&H.-C. Wang.(2015) Widespread Rickettsia spp. infections in ticks (Acari: Ixodoidea) in Taiwan. *Journal of medical entomology*, *52*(5), 1096-1102

Liang, P.(1996) Investigation on the Iost Animal and Transmission Vector of Lyme Disease in Fujian Province(in chinese). *Chinese Journal of Vector Biology and Control*, *7*(6), 437-439. CNKI:SUN:ZMSK.0.1996-06-014

Liang, P.(1996) Study on the Host Ahimal and Transmission Vector of Lyme Disease in Fujian Province(in chinese). *Strait Journal of Preventive Medicine*, *2*(2), 1-2. CNKI:SUN:HXYF.0.1996-02-000

LianKun, L.(2011) Species of Poultry Ticks and Its Dominant Species in Guizhou Province(in chinese). *Journal of Southwest China Normal University （Natural Science Edition） 36*(1), 99-101. 10.13718/j.cnki.xsxb.2011.01.040

LiHua, M.(1986) THE INVESTIGATION AND RESEARCH ON THE BABESIOSIS OF BUFFALO IN HUBEI PROVINCE Ⅲ. THE STUDY OF THE LIFE HISTORY OF THE TICK RHIPICEPHALUS HAEMAPHYSALOIDES HAEMAPHYSALOIDES SUPINO(in chinese). *Journal of Huazhong Agricultural University*, *5*(2), 157-162. 10.13300/j.cnki.hnlkxb.1986.02.009

LiHua, M.(1989) A survey of water buffalo babesiasis in HuBei province(IV)-The Observation of vermicules of babesia bovins in the Haemolymph of Rhipicephylus Haemaphysaloides Haemaphysaloides(in chinese). *Journal of Huazhong Agricultural University*, *8*(2), 151-154. 10.13300/j.cnki.hnlkxb.1989.02.010

LiJuan, W.(1997) First Isolation and Identification of Lyme Disease Spirochete in Shandong Province(in chinese). *Chinese Journal of Vector Biology and Control*, *8*(2), 127-128. CNKI:SUN:ZMSK.0.1997-02-022

LiJuan, W.(2000) The first discovery of endemic Lyme disease in Shandong province(in chinese). *Chinese Journal of Epidemiology*(4), 292-294. 10.3760/j.issn:0254-6450.2000.04.015

LiJuan, W.(2007) Seroepidemiological study of Lyme disease in Shandong province(in chinese). *Chinese Journal of Vector Biology and Control*, *18*(4), 306-308. 10.3969/j.issn.1003-4692.2007.04.014

LiJun, G.(2018) Investigation on the selectivity of Haemaphysalis longicornis for hosts in different geographic zoning of Henan(in chinese). *Henan Journal of preventive Medicine*, *29*(9), 671-673. 10.13515/j.cnki.hnjpm.1006-8414.2018.09.011

LiPing, J.(2006) Establishment and application of detection methods for tick-borne diseases in Zhejiang Province(in chinese). *Modern Practical Medicine*, *18*(11), 847-848. CNKI:SUN:NBYX.0.2006-11-047

Liu, L., T.-y. Cheng&F. Yan.(2016) Expression pattern of subA in different tissues and blood-feeding status in Haemaphysalis flava. *Experimental and Applied Acarology*, *70*(4), 511-522

LiZhao, X.(1993) A list of ticks in AnHui Province(in chinese). *Entomological Journal of East China*, *2*(2), 21-23

LongZheng, Y.(2015) Investigation on ticks in Yanbian natural habitat of Jilin Province(in chinese). *International Journal of Medical Parasitic Diseases*, *42*(1), 22-24. 10.3760/cma.j.issn.1673-4122.2015.01.006

Mekonnen, S., I. Hussein&B. Bedane.(2001) The distribution of ixodid ticks (Acari: Ixodidae) in central Ethiopia.

MingShe, L.(2005) Investigating the Distribution of Ticks Among Nine Provinces in China(in chinese). *Journal of Changzhi Medical College*, *19*(4), 249-250. 10.3969/j.issn.1006-0588.2005.04.004

Miranpuri, G. S., O. S. Bindra&V. Prasad.(1975) Tick fauna of north-western India (Acarina: Metastigmata). *International Journal of Acarology*, *1*(1), 31-54

Na, L.(2014) Detection of novel tick-borne Bunyavirus in Shenyang(in chinese). *Disease Surveillance*, *29*(5), 395-398. 10.3784/j.issn.1003-9961.2014.05.015

Ngumi, P., R. Rumberia, S. Williamson, K. Sumption, A. Lesan&D. Kariuki.(1997) Isolation of the causative agent of heartwater (Cowdria ruminantium) from three Amblyomma species in eight districts of Kenya. *Veterinary record*, *140*(1), 13-16

Omondi, D., D. K. Masiga, B. C. Fielding, E. Kariuki, Y. U. Ajamma, M. M. Mwamuye, D. O. Ouso&J. Villinger.(2017) Molecular detection of tick-borne pathogen diversities in ticks from livestock and reptiles along the shores and adjacent islands of Lake Victoria and Lake Baringo, Kenya. *Frontiers in Veterinary Science*, *4*, 73

Pegram, R.(1976) Ticks (Acarina, Ixodoidea) of the northern regions of the Somali Democratic Republic. *Bulletin of Entomological Research*, *66*(2), 345-363

Pegram, R. G., H. Hoogstraal&H. Y. Wassef.(1981) Ticks (Acari: Ixodoidea) of Ethiopia. I. Distribution, ecology and host relationships of species infesting livestock. *Bulletin of Entomological Research*, *71*(3), 339-359

Qi, Z.(2015) Investigation of species, temporal and spatial distribution of ticks in Henan province, China(in chinese). *Chinese Journal of Vector Biology and Control*, *26*(1), 75-77. 10.11853/j.issn.1003.4692.2015.01.000

Qian, C.(2022) Molecular epidemiological investigation of Anaplasma in cattle and tick-borne Anaplasma in parts of Henan(in chinese). *Henan Agricultural University*(04), 1-88. 10.27117/d.cnki.ghenu.2021.000326

Qian, H.(2019) Rickettsia and Ehrichia from Ticks: field and laboratory investigation in Ganzhou City，Jiangxi Provence，China(in chinese). *Chinese Journal of Zoonoses*, *35*(6), 518-524. 10.3969/j.issn.1002-2694.2019.00.077

Qian, W.(2021) Identification of Yunnan tick virus and epidemiological investigation on its co-infection(in chinese). *Shandong University*(10), 1-194. 10.27272/d.cnki.gshdu.2021.000493

Qiang, Y.(1996) Detection and Analysis of 16SrRNA of Anaplasma longhorn in Wetland of Laizhou Bay, Shandong Province(in chinese). *Chinese Journal of Zoonoses*(3), 12. CNKI:SUN:ZRSZ.0.2012-04-002

Rajagopalan, P.(1972) Ixodid ticks (Acarina: Ixodidae) parasitizing wild birds in the Kyasanur forest disease area of Shimoga district, Mysore State, India. *Bombay Natur Hist Soc J*

Sadanandane, C., M. Gokhale, A. Elango, P. Yadav, D. Mourya&P. Jambulingam.(2018) Prevalence and spatial distribution of Ixodid tick populations in the forest fringes of Western Ghats reported with human cases of Kyasanur forest disease and monkey deaths in South India. *Experimental and Applied Acarology*, *75*(1), 135-142

ShaoJun, H.(2020) High-throughput sequencing based analysis on diversity of pathogens carried by ticks in Yuyao, Zhejiang(in chinese). *DISEASE SURVEILLANCE*, *35*(7), 642-645. 10.3784/j.issn.1003-9961.2020.07.019

ShiQuan, L.(2001) Investigation on the species of parasites in Jiangxi mountains and sheep(in chinese). *Jiangxi Journal of Animal Husbandry & Veterinary Medicine*(4), 10-11. 10.3969/j.issn.1004-2342.2001.04.006

Shuai, F.(2013) Molecular epidemiological study of tick⁃borne spotted fever group Rickettsia in western mountain area of Hebei province, China(in chinese). *Chinese Journal of Vector Biology and Control*, *24*(4), 308-312. 10.11853/j.issn.1003.4692.2013.04.008

Tatchell, R.&E. Easton.(1986) Tick (Acari: Ixodidae) ecological studies in Tanzania. *Bulletin of Entomological Research*, *76*(2), 229-246

Ting, M.(2015) Survey of vectors and hosts of severe fever with thrombocytopenia syndrome virus in Zhejiang province, China(in chinese). *Chinese Journal of Vector Biology and Control*, *26*(4), 353-356. 10.11853/j.issn.1003.4692.2015.04.006

Walker, J. B., J. E. Keirans&I. G. Horak (2000). The genus Rhipicephalus (Acari, Ixodidae): a guide to the brown ticks of the world, Cambridge University Press.

Walker, J. B.&A. Olwage.(1987) The tick vectors of Cowdria ruminantium (Ixodoidea, Ixodidae, genus Amblyomma) and their distribution.

WanChun, T.(1994) Survey on Tick Vectors of Lyme Disease Spirochete in Hebei Province(in chinese ). *Journal of Medical Pest Control*, *10*(1), 33-35. CNKI:SUN:ZGGW.0.1995-06-008

Wang, L., X. Zhou, L. Deng, Y. Liu, Y. Li, Y. Chen, S. Huang, G. Li, Y. Huang&H. Zhang.(2020) Complete mitogenome of the giant panda tick Haemaphysalis longicornis (Ixodida: Ixodidae) and its phylogenetic implications. *Mitochondrial DNA Part B*, *5*(3), 3221-3223

WeiXian, L.(1987) THE ECOGEOGRAPHIICAL DISTRIBUTION OF TICKS IN LIAONING PROVINCE(in chinese). *Acta Entomologica Sinica*, *30*(2), 180-185. 10.16380/j.kcxb.1987.02.010

XiangGuang, Y.(2019) Investigation on ticks at 7 ports of entry in Anhui(in chinese). *Chinese Frontier Health Quarantine*, *42*(6), 409-411. 10.16408/j.1004-9770.2019.06.010

XiangYe, L.(2015) Investigation and Phylogenic Analysis of Anaplasma Derived from Hard Ticks in Yiyuan County , Shandong Province(in chinese). *Progress in Veterinary Medicine*, *36*(12), 103-107. 10.16437/j.cnki.1007-5038.2015.12.021

XiaoQing, L.(2019) Distribution of ixodid ticks in Jiangxi Province(in chinese). *Chinese Journal of Hygienic Insecticides & Equipments*, *25*(6), 555-561. CNKI:SUN:WSSC.0.2019-06-015

XiaoQing, L.(2020) Epidemiological studies on tick-borne pathogens in vectors and hosts in Poyang Lake region of Jiangxi Province(in chinese). *Chinese Journal of Hygienic Insecticides & Equipments*, *26*(2), 151-157. 10.19821/j.1671-2781.2020.02.017

XiaoRong, S.(1997) Studies on the Ticks and It's Transmission Disease in Guizhou Province(in chinese). *Chinese Journal of Vector Biology and Control*, *8*(4), 287-288. CNKI:SUN:ZMSK.0.1997-04-052

XiCheng, D.(1997) Some Biological Characteristics of Ticks and Piroplasmas of Cattle in Southmountain Farm Hunan Province(in chinese). *Journal of Shihezi University(Natural Science)*, *1*(2), 139-141. 10.13880/j.cnki.65-1174/n.1997.02.013

XinHong, Z.(2022) An analysis of the monitoring results of ticks in goats in Yiwu，Zhejiang province，China，2020(in chinese). *Chinese Journal of Vector Biology and Control*, *32*(2), 221-224. 10.11853/j.issn.1003.8280.2022.02.010

Xu, L., M. Guo, B. Hu, H. Zhou, W. Yang, L. Hui, R. Huang, J. Zhan, W. Shi&Y. Wu.(2021) Tick virome diversity in Hubei Province, China, and the influence of host ecology. *Virus evolution*, *7*(2), veab089

XueBin, T.(2020) Investigation and Analysis of Population Dynamics of Haemaphysalis longicornis in Yunfengling District, Chengde, Hebei Province(in chinese ). *JOURNAL OF HEBEI NORMAL UNIVERSITY(Natural Science)*, *44*(1), 57-61. 10.13763/j.cnki.jhebnu.nse.2020.01.009

XueBin, T.(2020) Study on species and distribution of ticks and population dynamics and life cycle of dominant ticks in Hebei Province(in chinese) *Chengde Medical University*(2), 1-74. 10.27691/d.cnki.gcdyx.2019.000005

XueLiang, C.(2019) An investigation of on-host ticks collected from domestic and small wild animals in Suzhou, Jiangsu province, China(in chinese). *Chinese Journal of Vector Biology and Control*, *30*(6), 689-692. 10.11853/j.issn.1003.8280.2019.06.022

Yan, Z.(2021) Species identification of hard ticks and molecular survey of Anaplasmas in partial areas of Henan Province(in chinese). *MODERN ANIMAL HUSBANDRY*, *5*(1), 27-34. 10.3969/j.issn.1005-0507.2020.02.008

Yang, L.(2012) Survey on ticks and detection of new bunyavirus in the endemic areas of fever, thrombocytopenia and leukopenia syndrome in Henan province(in chinese). *Zhengzhou University*(9), 1-67. 10.7666/d.y2102750

YingZi, H.(2013) Investigation and protection measures of acarids in tussah farms in Fengcheng, Liaoning(in chinese). *Liaoning Agricultural Sciences*(2), 50-51. 10.3969/j.issn.1002-1728.2013.02.015

YinShu, Y.(2008) Investgaiton on species and nature geographic distribution of ticks in Shaanxi Province(in chinese). *Chinese Journal of Hygienic Insecticides & Equipments*, *14*(2), 97-99. 10.3969/j.issn.1671-2781.2008.02.007

YiRen, L.(2001) The Tick Fauna in FuBei Province(in chinese). *ACTA ARACHNOLOGICA SINICA*, *10*(1), 18-21. 10.3969/j.issn.1005-9628.2001.01.007

YouZhi, Z.(2009) Species and geographical distribution of ticks in Tibet(in chinese). *Chinese Journal of Hygienic Insecticides & Equipments*, *15*(3), 244-245. 10.19821/j.1671-2781.2009.03.025

YuLong, X.(2022) An investigation of ticks and tick-borne bacteria in some areas of Guizhou province，China(in chinese ). *Chinese Journal of Vector Biology and Control*, *33*(1), 148-152. 10.11853/j.issn.1003.8280.2022.01.027

YuQing, L.(2015) Surveillance of tick-born infections during 2012-2013 in Lishui, Zhejiang(in chinese). *Chinese Journal of Vector Biology and Control*, *26*(5), 512-515. 10.11853/j.issn.1003.4692.2015.05.022

Zhang, R., A. Zhao, X. Wang&Z. Zhang.(2017) Diversity of tick species on domestic animals in Shandong Province, China, using DNA barcoding. *Experimental and Applied Acarology*, *73*(1), 79-89

Zhe, X.(2014) Comprehensive surveillance of tick⁃borne diseases in Jiande, Zhejiang province, China(in chinese). *Chinese Journal of Vector Biology and Control*, *25*(4), 350-353. 10.11853/j.issn.1003.4692.2014.04.018

Zhen, W.(2017) lnvestigation on the animal parasitic ticks andtick-borne zoonotic disease carrying situation in western of Liaoning Province(in chinese). *Jinzhou Medical University*(5), 1-56. CNKI:CDMD:2.1016.174860

ZhengDa, G.(2001) Composition and distribution of cicadas in the Hengduan mountain area of western Yunnan(in chinese). *Journal of Medical Pest Control*, *17*(1). 10.3969/j.issn.1003-6245.2001.01.013

ZhenGuang, C.(1999) Study on the Host Animal and Transmission vector of SFG in Nighua County Fujian Province of China(in chinese). *Chinese public health*, *15*(8), 684-685. 10.1088/0256-307X/16/12/025

ZhenGuang, C.(1999) Study on the Host Animals and Transmission Vectors of SFGR in Ninghua County，Fujian. *Strait Journal of Preventive Medicine*, *5*(2), 3-5

ZhenHong, M.(2020) An investigation of questing ticks collected from the habitats with different vegetation types in Suzhou, Jiangsu province, China(in chinese). *Chinese Journal of Vector Biology and Control*, *31*(1), 92-95. CNKI:SUN:ZMSK.0.2020-01-020

ZhiHai, H.(2020) Infection of Borrelia burgdorferi sensu lato of host mammals and vectors in western Yunnan(in chinese). *Dali University*, *1*, 1-65. 10.27811/d.cnki.gdixy.2019.000056

ZhiJin, L.(2002) A Survey of Tick in YichangHubei with the Delinitation of Zoogeographical Regions(in chinese). *Chinese Journal of Vector Biology and Control*, *13*(3), 200-201. 10.3969/j.issn.1003-4692.2002.03.013

ZhiKun, Z.(1995) Investigation on Lyme Disease in Hebei Province(in chinese). *Chinese Journal of Public Health*, *11*(6), 253-254

ZhiYu, L.(2021) Investigation of rickettsiae in ticks from the northeast of Hubei Province(in chinese). *Military Medical Sciences*, *45*(4), 262-266. 10.7644/j.issn.1674

ZhongLing, L.(1987) STUDY ON BABESIOSIS OF BUFFALOES IN HUBEI PROVINCE Ⅱ.EXPERIMENTAL INFECTION DEMONSTRATED THAT RHIPICEPHALUS HAEMAPHYSALOIDES HAEMAPHYSALOIDES TO BE THE VECTOR OF BABESIOSIS IN BUFFALOES(in chinese). *Acta Veterinaria et Zootechnica sinica*, *18*(3), 173-178

Zhuo, W.(2018) Molecular detection of spotted fever group rickettsiae in Haemaphysalis longicornis from the eastern mountains of Liaoning Province, China(in chinese). *Journal of Pathogen Biology*, *13*(6), 609-611. 10.13350/j.cjpb.180613

ZiXin, W.(2022) Ticks species，distribution and pathogens in Shanghai，China(in chinese). *Chinese Journal of Vector Biology and Control*, *33*(1), 120-124. 10.11853/j.issn.1003.8280.2022.01.022

ZongYi, W.(1997) Flora Investigation and Seasonal Dynamic Observation of Haemorida longhorn in Hebei Province(in chinese). *CHINESE JOURNAL OF VETERINARY SCIENCE AND TECHNOLOGY*, *27*(10), 17-19. 10.16656/j.issn.1673-4696.1997.10.008

Zulu, F., J. Okello-Onen, D. Punyua, S. Essuman&M. Malonza.(1998) A note on the ticks of domestic animals in Coast Province, Kenya. *International Journal of Tropical Insect Science*, *18*(2), 163-165
