# Supplementary material for: The Geographical Coexist of the Migratory Birds, Ticks, and Nairobi Sheep Disease Virus May Potentially Contribute to the Passive Spreading of Nairobi Sheep Disease
Source: Transbound Emerg Dis. 2023 Oct 30;2023:5598142. doi: 10.1155/2023/5598142 (PMC12016763; doi:10.1155/2023/5598142)
Supplement: Supplementary 5 — Table S4: predictor contribution rate and AUC value in the tick model. Table S5: contribution rate and AUC value of predictor variables in the NSD model. [file 5598142.f5.docx]

**Table S4. Predictor contribution rate and AUC value in the tick model.** (Tick species names are abbreviated; A.V=*A. variegatum*, R.A=*R. appendiculatus*, R.P=*R. pulchellus*, R.H=*R. haemaphysaloides*, H.I=*H. intermedia*, H.W=*H. wellingtoni*, H.L=*H. longicornis*)

| **Tick** | **Region(Subregion)** | **Land cover** | **Elevation** | **Soil Moisture** | | | | | | | | | | | | **temp1** | **prec12** | **bio9** | **prec1** | **temp4** | **temp12** | **bio2** | **bio6** | **bio7** | **bio16** | **prec2** | **prec4** | **prec7** | **prec9** | **temp2** | **temp6** | **tmax3** | **tmax6** | **tmax11** | **tmin8** | **tmin9** | **VIF** | **AUC** |
| --- | --- | --- | --- | --- | --- | --- | --- | --- | --- | --- | --- | --- | --- | --- | --- | --- | --- | --- | --- | --- | --- | --- | --- | --- | --- | --- | --- | --- | --- | --- | --- | --- | --- | --- | --- | --- | --- | --- |
|  |  |  |  | **Jan** | **Jul** | **Mar** | **Jun** | **Aug** | **Sep** | **Oct** | **Feb** | **Apr** | **May** | **Nov** | **Dec** |  |  |  |  |  |  |  |  |  |  |  |  |  |  |  |  |  |  |  |  |  |  |  |
| A. V | Region1(Arid) | 38.3 | 11.4 |  |  |  |  |  |  |  |  |  |  |  |  | 23.9 |  |  |  |  |  |  |  |  |  | 26.4 |  |  |  |  |  |  |  |  |  |  | 1.000-5.471 | 0.889 |
|  | Region1(Temperate) | 18.7 |  | 20.8 | 14.8 |  |  |  | 11.1 |  |  |  |  |  |  |  |  | 12.1 |  | 22.5 |  |  |  |  |  |  |  |  |  |  |  |  |  |  |  |  | 1.081-8.576 | 0.808 |
|  | Region1(Tropical) | 20.3 | 20.7 |  |  | 19.1 | 12.8 |  |  |  |  |  |  |  |  |  |  |  |  |  |  | 27.1 |  |  |  |  |  |  |  |  |  |  |  |  |  |  | 1.001-4.524 | 0.897 |
| R. A | Region1(Arid) | 50.1 |  |  |  |  |  |  |  |  |  |  |  |  |  |  |  |  |  |  |  |  |  |  |  |  |  |  | 20.0 |  |  |  |  |  |  | 29.9 | 1.000-1.000 | 0.906 |
|  | Region1(Temperate) | 21.5 |  | 13.5 |  |  |  |  |  |  |  |  |  |  |  |  | 27.8 |  | 37.2 |  |  |  |  |  |  |  |  |  |  |  |  |  |  |  |  |  | 1.092-3.021 | 0.899 |
|  | Region1(Tropical) | 12.0 |  |  |  | 19.2 |  |  |  | 21.5 |  |  |  |  |  |  |  |  |  |  |  |  |  |  |  |  |  |  |  |  |  |  |  |  | 47.3 |  | 2.541-7.142 | 0.930 |
| R. P | Region1(Arid) | 15.9 |  |  |  | 18.9 |  |  |  |  |  |  |  |  |  | 29.5 |  |  |  |  | 35.7 |  |  |  |  |  |  |  |  |  |  |  |  |  |  |  | 4.632-5.377 | 0.826 |
|  | Region1(Temperate) |  |  |  |  |  |  |  |  |  |  |  |  |  |  |  | 51.8 |  | 48.2 |  |  |  |  |  |  |  |  |  |  |  |  |  |  |  |  |  | 1.000-1.000 | 0.894 |
|  | Region1(Tropical) | 46.3 | 12.9 |  | 26.1 |  |  |  |  |  |  |  |  |  |  |  |  |  |  |  |  |  |  |  | 14.7 |  |  |  |  |  |  |  |  |  |  |  | 4.574-6.198 | 0.875 |
| R. H | Region2(Mountain Climate) | 41.7 | 30.5 |  |  |  |  |  |  |  |  |  |  |  |  |  |  |  |  | 27.8 |  |  |  |  |  |  |  |  |  |  |  |  |  |  |  |  | 1.000-1.000 | 0.928 |
|  | Region2(Arid Steppe Hot) | 43.4 |  | 41.5 |  |  |  |  |  |  |  |  |  |  |  | 15.1 |  |  |  |  |  |  |  |  |  |  |  |  |  |  |  |  |  |  |  |  | 1.000-1.000 | 0.896 |
|  | Region2(Sub-tropical Humid) | 74.4 |  |  |  |  |  |  |  |  |  | 11.9 |  |  |  |  |  |  |  |  |  |  |  |  |  |  |  | 13.7 |  |  |  |  |  |  |  |  | 1.000-1.000 | 0.892 |
|  | Region2(Tropical Monsoon/Savannah) |  | 24.0 |  |  |  |  |  |  |  |  |  |  |  | 76.0 |  |  |  |  |  |  |  |  |  |  |  |  |  |  |  |  |  |  |  |  |  | 1.000-1.000 | 0.955 |
|  | Region3(Subtropical/Tropical Monsoon) | 17.8 | 15.3 |  | 13.4 |  |  |  | 10.4 |  |  |  |  |  |  | 15.6 | 27.4 |  |  |  |  |  |  |  |  |  |  |  |  |  |  |  |  |  |  |  | 5.237-8.414 | 0.833 |
| H. I | Region2(Mountain Climate) | 35.7 | 26.3 |  |  |  |  |  |  |  | 10.8 |  | 27.2 |  |  |  |  |  |  |  |  |  |  |  |  |  |  |  |  |  |  |  |  |  |  |  | 3.216-5.429 | 0.937 |
|  | Region2(Arid Steppe Hot) | 78.1 |  |  |  |  |  |  |  |  |  |  |  |  |  |  |  |  |  |  |  |  |  |  |  |  |  |  |  |  |  | 21.9 |  |  |  |  | - | 0.871 |
|  | Region2(Sub-tropical Humid) | 89.8 |  |  |  |  |  |  |  |  |  |  |  |  |  |  |  |  |  |  |  |  |  | 10.2 |  |  |  |  |  |  |  |  |  |  |  |  | - | 0.858 |
|  | Region2(Tropical Monsoon/Savannah) | 24.1 |  | 60.9 |  |  |  |  |  |  |  |  |  | 15.0 |  |  |  |  |  |  |  |  |  |  |  |  |  |  |  |  |  |  |  |  |  |  | 1.000-1.000 | 0.909 |
| H. W | Region2(Mountain Climate) | 24.6 | 11.4 | 52.5 |  |  |  | 11.5 |  |  |  |  |  |  |  |  |  |  |  |  |  |  |  |  |  |  |  |  |  |  |  |  |  |  |  |  | 2.268-4.547 | 0.878 |
|  | Region2(Arid Steppe Hot) | 54.1 |  |  |  |  |  |  |  |  |  |  |  |  |  |  |  |  |  |  |  |  |  |  |  |  |  |  |  |  |  |  | 18.0 | 27.9 |  |  | 1.000-1.000 | 0.937 |
|  | Region2(Sub-tropical Humid) | 84.8 |  |  |  |  |  |  |  |  |  |  |  |  |  |  |  |  |  |  |  |  | 15.2 |  |  |  |  |  |  |  |  |  |  |  |  |  | - | 0.926 |
|  | Region2(Tropical Monsoon/Savannah) | 61.3 |  |  |  |  |  |  |  |  |  |  |  |  |  |  |  |  |  |  |  |  |  |  |  |  |  |  |  |  | 38.7 |  |  |  |  |  | - | 0.887 |
|  | Region3(Subtropical/Tropical Monsoon) | 23.2 |  | 40.0 |  |  | 23.9 |  |  |  |  |  |  |  |  |  |  |  |  |  |  |  |  |  |  |  | 12.9 |  |  |  |  |  |  |  |  |  | 3.336-6.124 | 0.937 |
| H. L | Region3(Temperate Monsoon) | 21.3 |  |  |  |  |  |  |  |  | 11.5 |  |  |  |  |  |  | 39.2 |  |  | 28.0 |  |  |  |  |  |  |  |  |  |  |  |  |  |  |  | 1.269-4.302 | 0.864 |
|  | Region3(Subtropical/Tropical Monsoon) | 40.6 | 11.4 |  | 18.3 |  |  |  |  |  |  |  |  |  |  | 17.4 |  |  |  |  |  |  |  |  |  |  |  |  |  | 12.3 |  |  |  |  |  |  | 6.533-6.588 | 0.832 |

**Table S5. Contribution rate and AUC value of predictor variables in NSD model**

| **Region** | **Land cover** | **Elevation** | **bio6** | **bio12** | **tmax8** | **bio3** | **bio14** | **prec5** | **prec6** | **prec8** | **tmax7** | **tmin3** | **tmin6** | **tmin9** | **VIF** | **AUC** |
| --- | --- | --- | --- | --- | --- | --- | --- | --- | --- | --- | --- | --- | --- | --- | --- | --- |
| Region1(Arid) | 40.7 |  |  |  |  |  |  |  |  |  |  | 46.3 | 13 |  | 1.000-1.000 | 0.922 |
| Region1(Temperate) | 15.4 |  |  |  |  |  | 34.6 |  |  | 50 |  |  |  |  | 1.000-1.000 | 0.977 |
| Region1(Tropical) | 43.5 |  |  |  |  |  |  |  | 25.3 |  |  |  |  | 31.2 | 1.000-1.000 | 0.897 |
| Region2(Mountain Climate) | 65.5 |  | 16.0 |  |  | 18.5 |  |  |  |  |  |  |  |  | 1.000-1.000 | 0.972 |
| Region2(Arid Steppe Hot) | 22.2 |  |  |  | 14.3 |  |  |  |  |  | 63.5 |  |  |  | 1.000-1.000 | 0.970 |
| Region2(Sub-tropical Humid) | 70.7 |  | 18.6 |  | 10.7 |  |  |  |  |  |  |  |  |  | 1.000-1.000 | 0.933 |
| Region2(Tropical Monsoon/Savannah) | 75.7 |  |  | 24.3 |  |  |  |  |  |  |  |  |  |  | - | 0.854 |
| Region3(Temperate Monsoon) | 21.6 |  |  | 12.7 |  |  |  | 65.7 |  |  |  |  |  |  | 1.000-1.000 | 0.983 |
