# Supplementary material for: The Geographical Coexist of the Migratory Birds, Ticks, and Nairobi Sheep Disease Virus May Potentially Contribute to the Passive Spreading of Nairobi Sheep Disease
Source: Transbound Emerg Dis. 2023 Oct 30;2023:5598142. doi: 10.1155/2023/5598142 (PMC12016763; doi:10.1155/2023/5598142)
Supplement: Supplementary 6 — Migratory bird distribution data after preprocessing were used in the NSD spatial model. [file 5598142.f6.docx]

**Table S6. Migratory bird distribution data after pre-processing used in the NSD spatial model**

| **№** | **Bird Species** | | **Related Tick Species** | **No. of distribution points** | | | **Total** |
| --- | --- | --- | --- | --- | --- | --- | --- |
|  | Scientific name | Common name |  | Region1 | Region2 | Region3 |  |
| 1 | *Accipiter badius* | Shikra | *H. wellingtoni* | 40 | 1023 | 16 | 1079 |
| 2 | *Acrocephalus dumetorum* | Blyth's Reed-warbler | *H. intermedia, H. wellingtoni* | 0 | 279 | 1 | 280 |
| 3 | *Acrocephalus stentoreus* | Clamorous Reed-warbler | *H. wellingtoni* | 0 | 123 | 1 | 124 |
| 4 | *Alauda gulgula* | Oriental Skylark | *H. wellingtoni* | 0 | 109 | 125 | 234 |
| 5 | *Amaurornis phoenicurus* | White-breasted Waterhen | *H. wellingtoni* | 0 | 635 | 465 | 1100 |
| 6 | *Anthus trivialis* | Tree Pipit | *A. variegatum* | 15 | 125 | 3 | 143 |
| 7 | *Bubulcus ibis* | Cattle Egret | *A. variegatum* | 360 | 1300 | 1225 | 2885 |
| 8 | *Ciconia abdimii* | Abdim's Stork | *A. variegatum* | 111 | 0 | 0 | 111 |
| 9 | *Circaetus gallicus* | Short-toed Snake-eagle | *H. wellingtoni* | 8 | 190 | 4 | 202 |
| 10 | *Corvus corone* | Carrion Crow | *H. longicornis* | 0 | 4 | 27 | 31 |
| 11 | *Emberiza rustica* | Rustic Bunting | *H. longicornis* | 0 | 0 | 96 | 96 |
| 12 | *Emberiza spodocephala* | Black-faced Bunting | *H. longicornis* | 0 | 3 | 288 | 291 |
| 13 | *Eudynamys scolopaceus* | Western Koel | *H. wellingtoni* | 0 | 830 | 261 | 1091 |
| 14 | *Geokichla citrina* | Orange-headed Thrush | *H. wellingtoni* | 0 | 330 | 35 | 365 |
| 15 | *Glaucidium cuculoides* | Asian Barred Owlet | *H. wellingtoni* | 0 | 127 | 60 | 187 |
| 16 | *Halcyon coromanda* | Ruddy Kingfisher | *H. longicornis* | 0 | 9 | 14 | 23 |
| 17 | *Lalage melanoptera* | Black-headed Cuckooshrike | *H. wellingtoni* | 0 | 164 | 0 | 164 |
| 18 | Lanius cristatus | Brown Shrike | *H. intermedia* | 0 | 579 | 860 | 1439 |
| 19 | *Pastor roseus* | Rosy Starling | *H. wellingtoni* | 0 | 289 | 7 | 296 |
| 20 | *Pitta brachyura* | Indian Pitta | *H. intermedia* | 0 | 258 | 0 | 258 |
| 21 | *Pitta moluccensis* | Blue-winged Pitta | *H. wellingtoni* | 0 | 0 | 3 | 3 |
| 22 | *Pitta nympha* | Fairy Pitta | *H. longicornis* | 0 | 0 | 54 | 54 |
| 23 | *Plectropterus gambensis* | Spur-winged Goose | *A. variegatum* | 114 | 0 | 0 | 114 |
| 24 | *Sagittarius serpentarius* | Secretarybird | *A. variegatum* | 641 | 0 | 0 | 641 |
| 25 | *Saxicola caprata* | Pied Bushchat | *H. wellingtoni* | 0 | 633 | 4 | 637 |
| 26 | *Spilopelia chinensis* | Eastern Spotted Dove | *H. longicornis* | 0 | 1113 | 1388 | 2501 |
| 27 | *Sturnus pagodarum* | Brahminy Starling | *H. intermedia, H. wellingtoni* | 0 | 557 | 1 | 558 |
| 28 | *Tephrodornis virgatus* | Large Woodshrike | *H. wellingtoni* | 0 | 28 | 8 | 36 |
| 29 | *Turdus cardis* | Japanese Thrush | *H. longicornis, H. wellingtoni* | 0 | 0 | 64 | 64 |
| 30 | *Turdus chrysolaus* | Brown-headed Thrush | *R. haemaphysaloides* | 0 | 0 | 170 | 170 |
| 31 | *Turdus hortulorum* | Grey-backed Thrush | *H. longicornis* | 0 | 0 | 101 | 101 |
| 32 | *Turdus naumanni* | Naumann's Thrush | *H. longicornis* | 0 | 0 | 62 | 62 |
| 33 | *Turdus pallidus* | Pale Thrush | *H. longicornis* | 0 | 0 | 146 | 146 |
| 34 | *Zoothera aurea* | White's Thrush | *H. longicornis* | 0 | 0 | 126 | 126 |
| 35 | *Zoothera dauma* | Scaly Thrush | *H. intermedia* | 0 | 21 | 12 | 33 |
| Total | | | | 1289 | 8729 | 5627 | 15645 |
