# Supplementary material for: The Geographical Coexist of the Migratory Birds, Ticks, and Nairobi Sheep Disease Virus May Potentially Contribute to the Passive Spreading of Nairobi Sheep Disease
Source: Transbound Emerg Dis. 2023 Oct 30;2023:5598142. doi: 10.1155/2023/5598142 (PMC12016763; doi:10.1155/2023/5598142)
Supplement: Supplementary 7 — Table S7: parasitism intensity of corresponding NSDV vector ticks on selected migratory birds. Table S8: flight burden coefficient of selected migratory birds. Table S9: attachment coefficient of ticks for selected migratory birds moving along the set routes. [file 5598142.f7.docx]

**Table S7. Parasitism intensity of corresponding NSDV vector ticks on selected migratory birds** $B_{N}$：Nesting location- ground (1), shrub (0.9), tree (0.8), building (0.7), cavity (0.6), cliff (0.5), burrow (0.5). $B_{F}$：Foraging strategy- ground forager (1), glean arboreal (0.8), sally ground (0.7), aquatic predator (0.5). $B_{P}$：Population Status- Least Concern (LC:1), Near Threatened (NT:0.7), Vulnerable (VU:0.4), Endangered (EN:0.1), Critically Endangered (CR:0.01), Extinct in the Wild (EW:0). $K_{T}^{B}$：Mean of tick distribution probability of migratory bird distribution points. $K_{T}^{B}$(N)：Normalized mean of tick distribution probability of migratory bird distribution points.

| **Ticks species** | **Birds species** | $\boldsymbol{B}_{\boldsymbol{N}}$ | $\boldsymbol{B}_{\boldsymbol{F}}$ | $\boldsymbol{B}_{\boldsymbol{P}}$ | $\boldsymbol{K}_{\boldsymbol{T}}^{\boldsymbol{B}}$ | $\boldsymbol{K}_{\boldsymbol{T}}^{\boldsymbol{B}}$**(N)** | $\boldsymbol{M}_{\mathbf{A}}$ |
| --- | --- | --- | --- | --- | --- | --- | --- |
|  |  |  |  |  |  |  |  |
| *Amblyomma variegatum* | *Anthus trivialis* | 1 | 1 | 1 | 0.5046 | 1.0000 | 1.0000 |
|  | *Bubulcus ibis* | 0.9 | 1 | 1 | 0.3214 | 0.6369 | 0.5732 |
|  | *Ciconia abdimii* | 0.8 | 1 | 1 | 0.3617 | 0.7168 | 0.5734 |
| *Haemaphysalis intermedia* | *Acrocephalus dumetorum* | 0.9 | 0.8 | 1 | 0.2813 | 0.5575 | 0.4014 |
|  | *Lanius cristatus* | 0.9 | 1 | 1 | 0.3127 | 0.6197 | 0.5577 |
|  | *Pitta brachyuran* | 0.8 | 1 | 1 | 0.3971 | 0.7870 | 0.6296 |
|  | *Sturnus pagodarum* | 0.8 | 1 | 1 | 0.1997 | 0.3958 | 0.3166 |
| *Haemaphysalis longicornis* | *Emberiza spodocephala* | 1 | 1 | 1 | 0.3252 | 0.6445 | 0.6445 |
| *Haemaphysalis wellingtoni* | *Accipiter badius* | 0.8 | 0.8 | 1 | 0.2381 | 0.4719 | 0.3020 |
|  | *Acrocephalus dumetorum* | 0.9 | 0.8 | 1 | 0.2668 | 0.5287 | 0.3807 |
|  | *Acrocephalus stentoreus* | 0.9 | 0.8 | 1 | 0.2314 | 0.4586 | 0.3302 |
|  | *Alauda gulgula* | 1 | 1 | 1 | 0.2247 | 0.4453 | 0.4453 |
|  | *Amaurornis phoenicurus* | 1 | 1 | 1 | 0.2486 | 0.4927 | 0.4927 |
|  | *Circaetus gallicus* | 0.8 | 0.7 | 1 | 0.1976 | 0.3916 | 0.2193 |
|  | *Lalage melanoptera* | 0.8 | 0.8 | 1 | 0.2760 | 0.5470 | 0.3501 |
|  | *Eudynamys scolopaceus* | 0.8 | 1 | 1 | 0.2416 | 0.4788 | 0.3830 |
|  | *Saxicola caprata* | 0.6 | 0.7 | 1 | 0.2091 | 0.4144 | 0.1740 |
|  | *Sturnus pagodarum* | 0.8 | 1 | 1 | 0.2014 | 0.3991 | 0.3193 |
|  | *Pastor roseus* | 0.5 | 1 | 1 | 0.2446 | 0.4847 | 0.2424 |
|  | *Geokichla citrina* | 0.9 | 1 | 1 | 0.3205 | 0.6352 | 0.5716 |

**Table S8. Flight burden coefficient of selected migratory birds.** W：Birds body mass. N：Number of Ticks per Infested Bird.

| **Ticks species** | **Birds species** | **W (g)** | **N** | | **α** |
| --- | --- | --- | --- | --- | --- |
|  |  |  | **Value** | **References** |  |
| *Amblyomma variegatum* | *Anthus trivialis* | 22.5 | 2.8 | (Kaiser et al., 1974, Mancuso et al., 2022) | 0.9846 |
|  | *Bubulcus ibis* | 391 | 5.3 | (Corn et al., 1993) | 0.9998 |
|  | *Ciconia abdimii* | 1398 | 5.0 | (Njila et al., 2019) | 1.0000 |
| *Haemaphysalis intermedia* | *Acrocephalus dumetorum* | 11.5 | 5.2 | (Rajagopalan 1972) | 0.8151 |
|  | *Lanius cristatus* | 31.7 | 2.0 | (Kang et al., 2013, Kwak et al., 2022) | 0.9960 |
|  | *Pitta brachyura* | 60 | 6.6 | (Rajagopalan 1972) | 0.9880 |
|  | *Sturnus pagodarum* | 49 | 1.6 | (Rajagopalan 1972) | 0.9989 |
| *Haemaphysalis longicornis* | *Emberiza spodocephala* | 17 | 2.1 | (Ishiguro et al., 2000, Seo et al., 2021) | 0.9849 |
| *Haemaphysalis wellingtoni* | *Accipiter badius* | 145 | 1.0 | (Rajagopalan 1972) | 1.0000 |
|  | *Acrocephalus dumetorum* | 12 | 1.2 | (Rajagopalan 1972) | 0.9900 |
|  | *Acrocephalus stentoreus* | 25 | 7.0 | (Rajagopalan 1972) | 0.9246 |
|  | *Alauda gulgula* | 26 | 1.0 | (Rajagopalan 1972) | 0.9985 |
|  | *Amaurornis phoenicurus* | 180 | 2.0 | (Rajagopalan 1972) | 0.9999 |
|  | *Circaetus gallicus* | 2350 | 2.0 | (Rajagopalan 1972) | 1.0000 |
|  | *Lalage melanoptera* | 30 | 1.4 | (Rajagopalan 1972) | 0.9978 |
|  | *Eudynamys scolopaceus* | 210 | 1.0 | (Rajagopalan 1972) | 1.0000 |
|  | *Saxicola caprata* | 15 | 1.0 | (Rajagopalan 1972) | 0.9956 |
|  | *Sturnus pagodarum* | 49 | 1.6 | (Rajagopalan 1972) | 0.9989 |
|  | *Pastor roseus* | 73.5 | 1.0 | (Rajagopalan 1972) | 0.9998 |
|  | *Geokichla citrina* | 53 | 3.8 | (Rajagopalan 1972) | 0.9949 |

**Table S9. Attachment coefficient of ticks for selected migratory birds moving along the set routes.** W：Birds body mass. $D_{L}$：Birds Flying distance. $D_{T}$：Ticks attachment times.

| **Ticks species** | **Birds species** | **W (g)** | **Start point** | **End point** | $\boldsymbol{D}_{\boldsymbol{L}}$ **(m)** | $\boldsymbol{D}_{\boldsymbol{T}}$ **(day)** | | **β** |
| --- | --- | --- | --- | --- | --- | --- | --- | --- |
|  |  |  |  |  |  | **Value** | **References** |  |
| *Amblyomma variegatum* | *Anthus trivialis* | 22.5 | A1 | A10 | 1694092 | 8 | (Socolovschi et al., 2009) | 0.9600 |
|  | *Bubulcus ibis* | 391 | A1 | A10 | 1694092 | 8 | (Socolovschi et al., 2009) | 0.9405 |
|  |  | 391 | A1 | C7 | 1710353 | 8 | (Socolovschi et al., 2009) | 0.9400 |
|  |  | 391 | A9 | D13 | 3907513 | 8 | (Socolovschi et al., 2009) | 0.8648 |
|  |  | 391 | A9 | D11 | 5841246 | 8 | (Socolovschi et al., 2009) | 0.7985 |
|  |  | 391 | A9 | F8 | 3887832 | 8 | (Socolovschi et al., 2009) | 0.8655 |
|  |  | 391 | A9 | F7 | 5837172 | 8 | (Socolovschi et al., 2009) | 0.7987 |
|  |  | 391 | A9 | G12 | 4017266 | 8 | (Socolovschi et al., 2009) | 0.8610 |
|  |  | 391 | A9 | G13 | 5851142 | 8 | (Socolovschi et al., 2009) | 0.7982 |
|  |  | 391 | A9 | E1 | 8105840 | 8 | (Socolovschi et al., 2009) | 0.7683 |
|  | *Ciconia abdimii* | 1398 | A1 | A10 | 1694092 | 8 | (Socolovschi et al., 2009) | 0.9318 |
|  |  | 1398 | A1 | C7 | 1710353 | 8 | (Socolovschi et al., 2009) | 0.9311 |
| *Haemaphysalis intermedia* | *Acrocephalus dumetorum* | 11.5 | D4 | D21 | 2693675 | 5 | (Geevarghese et al., 2011) | 0.9041 |
|  |  | 11.5 | D4 | F17 | 2276691 | 5 | (Geevarghese et al., 2011) | 0.9184 |
|  |  | 11.5 | D4 | G15 | 2694956 | 5 | (Geevarghese et al., 2011) | 0.9041 |
|  | *Lanius cristatus* | 31.7 | D4 | D21 | 2693675 | 5 | (Geevarghese et al., 2011) | 0.8939 |
|  |  | 31.7 | D13 | D11 | 1971564 | 5 | (Geevarghese et al., 2011) | 0.9225 |
|  |  | 31.7 | D4 | F17 | 2276691 | 5 | (Geevarghese et al., 2011) | 0.9104 |
|  |  | 31.7 | D13 | F6 | 2003248 | 5 | (Geevarghese et al., 2011) | 0.9212 |
|  |  | 31.7 | D4 | G15 | 2694956 | 5 | (Geevarghese et al., 2011) | 0.8939 |
|  |  | 31.7 | D13 | G13 | 1984252 | 5 | (Geevarghese et al., 2011) | 0.9219 |
|  |  | 31.7 | D11 | E7 | 3119142 | 5 | (Geevarghese et al., 2011) | 0.8771 |
|  | *Pitta brachyura* | 60 | D4 | D21 | 2693675 | 5 | (Geevarghese et al., 2011) | 0.8831 |
|  |  | 60 | D4 | F9 | 1948021 | 5 | (Geevarghese et al., 2011) | 0.9151 |
|  |  | 60 | D4 | G15 | 2694956 | 5 | (Geevarghese et al., 2011) | 0.8829 |
|  | *Sturnus pagodarum* | 49 | D4 | D21 | 2693675 | 5 | (Geevarghese et al., 2011) | 0.8879 |
|  |  | 49 | D4 | F9 | 1948021 | 5 | (Geevarghese et al., 2011) | 0.9189 |
|  |  | 49 | D4 | G15 | 2694956 | 5 | (Geevarghese et al., 2011) | 0.8876 |
| *Haemaphysalis longicornis* | *Emberiza spodocephala* | 17 | E1 | D11 | 2567559 | 6 | (Neilson 1980) | 0.9211 |
|  |  | 17 | E1 | F17 | 1913504 | 6 | (Neilson 1980) | 0.9410 |
|  |  | 17 | E1 | F6 | 2669458 | 6 | (Neilson 1980) | 0.9180 |
|  |  | 17 | E1 | G14 | 1928039 | 6 | (Neilson 1980) | 0.9405 |
|  |  | 17 | E1 | G13 | 2578515 | 6 | (Neilson 1980) | 0.9208 |
| *Haemaphysalis wellingtoni* | *Accipiter badius* | 145 | F1 | F17 | 2407021 | 10 | (Geevarghese et al., 2011) | 0.9401 |
|  |  | 145 | F1 | D21 | 2809738 | 10 | (Geevarghese et al., 2011) | 0.9305 |
|  |  | 145 | F1 | G15 | 2811056 | 10 | (Geevarghese et al., 2011) | 0.9305 |
|  | *Acrocephalus dumetorum* | 12 | F1 | F17 | 2407021 | 10 | (Geevarghese et al., 2011) | 0.9586 |
|  |  | 12 | F1 | D21 | 2809738 | 10 | (Geevarghese et al., 2011) | 0.9514 |
|  |  | 12 | F1 | G15 | 2811056 | 10 | (Geevarghese et al., 2011) | 0.9514 |
|  | *Acrocephalus stentoreus* | 25 | F1 | F17 | 2407021 | 10 | (Geevarghese et al., 2011) | 0.9506 |
|  |  | 25 | F9 | F7 | 2636241 | 10 | (Geevarghese et al., 2011) | 0.9459 |
|  |  | 25 | F1 | D21 | 2809738 | 10 | (Geevarghese et al., 2011) | 0.9427 |
|  |  | 25 | F9 | D11 | 2677351 | 10 | (Geevarghese et al., 2011) | 0.9453 |
|  |  | 25 | F1 | G15 | 2811056 | 10 | (Geevarghese et al., 2011) | 0.9427 |
|  |  | 25 | F9 | G13 | 2694276 | 10 | (Geevarghese et al., 2011) | 0.9449 |
|  | *Alauda gulgula* | 26 | F1 | F17 | 2407021 | 10 | (Geevarghese et al., 2011) | 0.9551 |
|  |  | 26 | F9 | F7 | 2636241 | 10 | (Geevarghese et al., 2011) | 0.9506 |
|  |  | 26 | F1 | D21 | 2809738 | 10 | (Geevarghese et al., 2011) | 0.9473 |
|  |  | 26 | F9 | D11 | 2677351 | 10 | (Geevarghese et al., 2011) | 0.9497 |
|  |  | 26 | F1 | G15 | 2811056 | 10 | (Geevarghese et al., 2011) | 0.9473 |
|  |  | 26 | F9 | G13 | 2694276 | 10 | (Geevarghese et al., 2011) | 0.9495 |
|  | *Amaurornis phoenicurus* | 180 | F1 | F17 | 2407021 | 10 | (Geevarghese et al., 2011) | 0.9408 |
|  |  | 180 | F9 | F7 | 2636241 | 10 | (Geevarghese et al., 2011) | 0.9352 |
|  |  | 180 | F1 | D21 | 2809738 | 10 | (Geevarghese et al., 2011) | 0.9309 |
|  |  | 180 | F9 | D11 | 2677351 | 10 | (Geevarghese et al., 2011) | 0.9341 |
|  |  | 180 | F1 | G15 | 2811056 | 10 | (Geevarghese et al., 2011) | 0.9309 |
|  |  | 180 | F9 | G13 | 2694276 | 10 | (Geevarghese et al., 2011) | 0.9337 |
|  |  | 180 | F17 | E7 | 1479724 | 10 | (Geevarghese et al., 2011) | 0.9638 |
|  |  | 180 | F7 | E7 | 3034407 | 10 | (Geevarghese et al., 2011) | 0.9252 |
|  | *Circaetus gallicus* | 2350 | F17 | E7 | 1479724 | 10 | (Geevarghese et al., 2011) | 0.9498 |
|  | *Lalage melanoptera* | 30 | F1 | F9 | 2049736 | 10 | (Geevarghese et al., 2011) | 0.9595 |
|  |  | 30 | F1 | D21 | 2809738 | 10 | (Geevarghese et al., 2011) | 0.9447 |
|  |  | 30 | F1 | G15 | 2811056 | 10 | (Geevarghese et al., 2011) | 0.9447 |
|  | *Eudynamys scolopaceus* | 210 | F1 | F17 | 2407021 | 10 | (Geevarghese et al., 2011) | 0.9394 |
|  |  | 210 | F9 | F7 | 2636241 | 10 | (Geevarghese et al., 2011) | 0.9337 |
|  |  | 210 | F1 | D21 | 2809738 | 10 | (Geevarghese et al., 2011) | 0.9292 |
|  |  | 210 | F9 | D11 | 2677351 | 10 | (Geevarghese et al., 2011) | 0.9326 |
|  |  | 210 | F1 | G15 | 2811056 | 10 | (Geevarghese et al., 2011) | 0.9292 |
|  |  | 210 | F9 | G13 | 2694276 | 10 | (Geevarghese et al., 2011) | 0.9324 |
|  |  | 210 | F17 | E7 | 1479724 | 10 | (Geevarghese et al., 2011) | 0.9629 |
|  |  | 210 | F7 | E7 | 3034407 | 10 | (Geevarghese et al., 2011) | 0.9238 |
|  | *Saxicola caprata* | 15 | F1 | F17 | 2407021 | 10 | (Geevarghese et al., 2011) | 0.9559 |
|  |  | 15 | F1 | D21 | 2809738 | 10 | (Geevarghese et al., 2011) | 0.9490 |
|  |  | 15 | F1 | G15 | 2811056 | 10 | (Geevarghese et al., 2011) | 0.9490 |
|  | *Sturnus pagodarum* | 49 | F1 | F9 | 2049736 | 10 | (Geevarghese et al., 2011) | 0.9543 |
|  |  | 49 | F1 | D21 | 2809738 | 10 | (Geevarghese et al., 2011) | 0.9384 |
|  |  | 49 | F1 | G15 | 2811056 | 10 | (Geevarghese et al., 2011) | 0.9384 |
|  | *Pastor roseus* | 73.5 | F1 | F9 | 2049736 | 10 | (Geevarghese et al., 2011) | 0.9552 |
|  |  | 73.5 | F9 | F7 | 2636241 | 10 | (Geevarghese et al., 2011) | 0.9420 |
|  |  | 73.5 | F1 | D21 | 2809738 | 10 | (Geevarghese et al., 2011) | 0.9383 |
|  |  | 73.5 | F9 | D11 | 2677351 | 10 | (Geevarghese et al., 2011) | 0.9412 |
|  |  | 73.5 | F1 | G15 | 2811056 | 10 | (Geevarghese et al., 2011) | 0.9383 |
|  |  | 73.5 | F9 | G13 | 2694276 | 10 | (Geevarghese et al., 2011) | 0.9408 |
|  | *Geokichla citrina* | 53 | F1 | F17 | 2407021 | 10 | (Geevarghese et al., 2011) | 0.9504 |
|  |  | 53 | F9 | F7 | 2636241 | 10 | (Geevarghese et al., 2011) | 0.9455 |
|  |  | 53 | F1 | D21 | 2809738 | 10 | (Geevarghese et al., 2011) | 0.9418 |
|  |  | 53 | F9 | D11 | 2677351 | 10 | (Geevarghese et al., 2011) | 0.9446 |
|  |  | 53 | F1 | G15 | 2811056 | 10 | (Geevarghese et al., 2011) | 0.9418 |
|  |  | 53 | F9 | G13 | 2694276 | 10 | (Geevarghese et al., 2011) | 0.9443 |
|  |  | 53 | F17 | E7 | 1479724 | 10 | (Geevarghese et al., 2011) | 0.9699 |
|  |  | 53 | F7 | E7 | 3034407 | 10 | (Geevarghese et al., 2011) | 0.9371 |

**References**

Corn, J. L., N. Barré, B. Thiebot, T. E. Creekmore, G. I. Garris&V. F. Nettles.(1993) Potential role of cattle egrets, Bubulcus ibis (Ciconiformes: Ardeidae), in the dissemination of Amblyomma variegatum (Acari: Ixodidae) in the eastern Caribbean. *Journal of Medical Entomology*, *30*(6), 1029-1037

Geevarghese, G.&A. Mishra (2011). Haemaphysalis ticks of India, Elsevier.

Ishiguro, F., N. Takada, T. Masuzawa&T. Fukui.(2000) Prevalence of Lyme disease Borrelia spp. in ticks from migratory birds on the Japanese mainland. *Applied and Environmental Microbiology*, *66*(3), 982-986. <https://doi.org/10.1128/AEM.66.3.982-986.2000>

Kaiser, M. N., H. Hoogstraal&G. E. Watson.(1974) Ticks (Ixodoidea) on migrating birds in Cyprus, fall 1967 and spring 1968, and epidemiological considerations. *Bulletin of Entomological research*, *64*(1), 97-110. <https://doi.org/10.1017/S0007485300027024>

Kang, J.-G., H.-C. Kim, C.-Y. Choi, H.-Y. Nam, H.-Y. Chae, S.-T. Chong, T. A. Klein, S. Ko&J.-S. Chae.(2013) Molecular detection of Anaplasma, Bartonella, and Borrelia species in ticks collected from migratory birds from Hong-do Island, Republic of Korea. *Vector-Borne and Zoonotic Diseases*, *13*(4), 215-225. <https://doi.org/10.1089/vbz.2012.1149>

Kwak, M. L.&A. Ng.(2022) The detection of three new Haemaphysalis ticks (Acari: Ixodidae) in Singapore and their potential threat for public health, companion animals, and wildlife. *Acarologia*, *62*(4), 927-940. <https://doi.org/10.24349/fz2l-kg9r>

Mancuso, E., L. Toma, I. Pascucci, S. G. d’Alessio, V. Marini, M. Quaglia, S. Riello, A. Ferri, F. Spina&L. Serra.(2022) Direct and Indirect Role of Migratory Birds in Spreading CCHFV and WNV: A Multidisciplinary Study on Three Stop-Over Islands in Italy. *Pathogens*, *11*(9), 1056. <https://doi.org/10.3390/pathogens11091056>

Neilson, F. J. A. (1980). An investigation into the ecology, biology, distribution and control of Haemaphysalis longicornis Neumann, 1901: a thesis presented in partial fulfilment of the requirements for the degree of Master of Veterinary Science at Massey University, Massey University.

Njila, H., J. Debi-Dore, S. Pukuma, A. Ombugadu, M. Dibal&M. Mafuyai.(2019) SURVEY OF ECTOPARASITES OF CAPTIVE BIRDS IN THE JOS MUSEUM ZOOLOGICAL GARDEN, NORTH CENTRAL NIGERIA. *AFRICAN JOURNAL OF NATURAL SCIENCES (AJNS) ISSN 1119-1104*, *20*

Rajagopalan, P.(1972) Ixodid ticks (Acarina: Ixodidae) parasitizing wild birds in the Kyasanur forest disease area of Shimoga district, Mysore State, India. *Bombay Natur Hist Soc J*

Seo, H.-J., J. Noh, H.-C. Kim, S.-T. Chong, T. A. Klein, C.-U. Park, C. Y. Choi, Y.-S. Kwon, M. Kim&S. Min.(2021) Molecular detection and phylogenetic analysis of Anaplasma and Borrelia species in ticks collected from migratory birds at Heuksan, Hong, and Nan islands, Republic of Korea. *Vector-Borne and Zoonotic Diseases*, *21*(1), 20-31. <https://doi.org/10.1089/vbz.2020.2629>

Socolovschi, C., T. Huynh, B. Davoust, J. Gomez, D. Raoult&P. Parola.(2009) Transovarial and trans-stadial transmission of Rickettsiae africae in Amblyomma variegatum ticks. *Clinical Microbiology and Infection*, *15*, 317-318. <https://doi.org/10.1111/j.1469-0691.2008.02278.x>
