# Supplementary material for: The Geographical Coexist of the Migratory Birds, Ticks, and Nairobi Sheep Disease Virus May Potentially Contribute to the Passive Spreading of Nairobi Sheep Disease
Source: Transbound Emerg Dis. 2023 Oct 30;2023:5598142. doi: 10.1155/2023/5598142 (PMC12016763; doi:10.1155/2023/5598142)
Supplement: Supplementary 8 — Life cycle and host of NSDV vector ticks. [file 5598142.f8.docx]

**Table S10. Life cycle and host of NSDV vector ticks**

| **Tick species** | **Life cycle** | **Host** | | | |
| --- | --- | --- | --- | --- | --- |
|  |  | Description | Larval | Nymph | Adult |
| *Amblyomma variegatum* | Three- host | Usual hosts for larvae, nymphs and adults are Bovidae. Squamata are considered exceptional hosts. | Mammalia (several orders), Aves (several orders), Chamaeleonidae | Varanidae, Viperidae, Chamaelaeonidae, Agamidae, Colubridae | Varanidae, Viperidae |
| *Haemaphysalis intermedia* | Three- host | Usual hosts for adult ticks are Bovidae. | Mammalia (several orders), Cuculidae, Phasianidae, Picidae, Columbidae | Mammalia (several orders), Passeriformes (several families), Cuculidae, Phasianidae, Muscicapidae | Mammalia (several orders), Passeriformes (several families), Cuculidae, Phasianidae, Muscicapidae |
| *Haemaphysalis longicornis* | Three- host | Usual hosts for adult ticks are Bovidae, Cervidae, and Equidae. Aves are considered exceptional hosts. | Mammalia (several orders), Passeriformes (several families), Phasianidae, Apterygidae, Rallidae, Laridae | Mammalia (several orders), Passeriformes (several families), Phasianidae, Apterygidae, Rallidae, Psittacidae | Mammalia (several orders), Phasianidae, Anatidae |
| *Haemaphysalis wellingtoni* | Three- host | Usual hosts for larvae, nymphs and adults are Phasianidae | Mammalia (several orders), Aves (several orders) | Mammalia (several orders), Aves (several orders) | Mammalia (several orders), Aves (several orders) |
| *Rhipicephalus appendiculatus* | Three- host | Usual hosts for larvae, nymphs and adults are Bovidae. Aves and Testudines are exceptional hosts. | Mammalia (several orders), Numididae, Phasianidae, Coliidae, Alcedinidae, Sturnidae, Picidae | Mammalia (several orders), Numididae, Phasianidae, Coliidae, Alcedinidae, Sturnidae, Picidae | Mammalia (several orders), Numididae, Phasianidae, Laniidae, Testudines (unknown order) |
| *Rhipicephalus haemaphysaloides* | Three- host | Aves are exceptional hosts. | Canidae, Muridae, Soricidae, Cuculidae, Timaliidae | Canidae, Muridae, Bovidae, Herpestidae, Soricidae, Cuculidae, Timaliidae | Canidae, Muridae, Bovidae |
| *Rhipicephalus pulchellus* | Three- host | Aves are considered exceptional hosts. | Mammalia (several orders) | Mammalia (several orders) | Mammalia (several orders), Phasianidae, Struthionidae |
