# Supplementary material for: The Geographical Coexist of the Migratory Birds, Ticks, and Nairobi Sheep Disease Virus May Potentially Contribute to the Passive Spreading of Nairobi Sheep Disease
Source: Transbound Emerg Dis. 2023 Oct 30;2023:5598142. doi: 10.1155/2023/5598142 (PMC12016763; doi:10.1155/2023/5598142)
Supplement: Supplementary 4 — Distribution data of selected migratory birds in the study area. [file 5598142.f4.pdf]

Distribution data of selected migratory birds in the study area (NIA-Number Identification Agreements NID-Number Identification Disagreements)

No	scientific name	common name	date	NIA	NID	latitude	longitude	url
1	Anthus trivialis	Tree Pipit	2021/11/8	1	0	-5.0997620	38.6302930	https://www.inaturalist.org/observations/105115923
2	Anthus trivialis	Tree Pipit	2018/11/29	1	0	-3.3614408	38.3355012	https://www.inaturalist.org/observations/19081708
3	Anthus trivialis	Tree Pipit	2018/1/25	1	0	-3.1540060	35.6765950	https://www.inaturalist.org/observations/16814311
4	Anthus trivialis	Tree Pipit	2020/1/3	0	0	-3.1522317	35.6756950	https://www.inaturalist.org/observations/37690476
5	Anthus trivialis	Tree Pipit	2018/1/3	1	0	-3.1520887	35.6770876	https://www.inaturalist.org/observations/16652333
6	Anthus trivialis	Tree Pipit	2022/1/10	0	0	-2.5758974	37.8364669	https://www.inaturalist.org/observations/112724499
7	Anthus trivialis	Tree Pipit	2022/1/10	0	0	-2.5758974	37.8364669	https://www.inaturalist.org/observations/112724504
8	Anthus trivialis	Tree Pipit	2022/1/10	1	0	-2.5758974	37.8364669	https://www.inaturalist.org/observations/112724514
9	Anthus trivialis	Tree Pipit	2022/1/11	0	0	-2.5293527	37.9017028	https://www.inaturalist.org/observations/112724613
10	Anthus trivialis	Tree Pipit	2010/12/31	2	0	-1.9716567	35.1699829	https://www.inaturalist.org/observations/7131279
11	Anthus trivialis	Tree Pipit	2019/4/11	1	0	-1.8749622	37.2338896	https://www.inaturalist.org/observations/22366674
12	Anthus trivialis	Tree Pipit	2017/12/14	1	0	-1.3597540	36.7200442	https://www.inaturalist.org/observations/9782237
13	Anthus trivialis	Tree Pipit	2005/3/20	1	0	-1.3520746	36.7119868	https://www.inaturalist.org/observations/135347383
14	Anthus trivialis	Tree Pipit	2016/3/9	1	0	-1.1812436	36.8247795	https://www.inaturalist.org/observations/36698383
15	Anthus trivialis	Tree Pipit	2019/12/31	0	0	-1.1419189	36.7932025	https://www.inaturalist.org/observations/37223330
16	Anthus trivialis	Tree Pipit	2019/3/9	1	0	-0.9439805	36.5960290	https://www.inaturalist.org/observations/22292179
17	Anthus trivialis	Tree Pipit	2019/3/9	0	0	-0.9434214	36.5984401	https://www.inaturalist.org/observations/108444061
18	Anthus trivialis	Tree Pipit	2021/2/26	1	0	-0.9431500	36.5940814	https://www.inaturalist.org/observations/70198488
19	Anthus trivialis	Tree Pipit	2022/3/14	0	0	-0.9428707	36.5945579	https://www.inaturalist.org/observations/109282428
20	Anthus trivialis	Tree Pipit	2019/3/9	1	0	-0.3205965	36.9329590	https://www.inaturalist.org/observations/28693221
21	Anthus trivialis	Tree Pipit	2019/3/17	1	0	1.7851000	34.7266300	https://www.inaturalist.org/observations/21777662
22	Anthus trivialis	Tree Pipit	2013/1/21	2	0	7.5364239	38.8105771	https://www.inaturalist.org/observations/7506893
23	Anthus trivialis	Tree Pipit	2022/11/30	0	0	8.8625127	76.9484628	https://www.inaturalist.org/observations/144972114

24	Anthus trivialis	Tree Pipit	2009/2/7	3	0	9.9206550	38.9024840	https://www.inaturalist.org/observations/37135333
25	Anthus trivialis	Tree Pipit	2021/12/21	1	0	10.0520622	76.3503845	https://www.inaturalist.org/observations/127077462
26	Anthus trivialis	Tree Pipit	2020/12/23	2	0	11.3204253	76.6246357	https://www.inaturalist.org/observations/120875767
27	Anthus trivialis	Tree Pipit	2016/12/25	1	0	11.3230384	79.2433528	https://www.inaturalist.org/observations/54540049
28	Anthus trivialis	Tree Pipit	2018/11/15	2	0	11.6525402	76.6066110	https://www.inaturalist.org/observations/82795733
29	Anthus trivialis	Tree Pipit	2019/1/23	0	0	11.6616667	76.6272222	https://www.inaturalist.org/observations/94108976
30	Anthus trivialis	Tree Pipit	2019/3/24	1	0	11.7206943	76.5807582	https://www.inaturalist.org/observations/82694547
31	Anthus trivialis	Tree Pipit	2022/12/3	0	0	11.9446843	75.3849381	https://www.inaturalist.org/observations/145187719
32	Anthus trivialis	Tree Pipit	2022/12/3	0	0	11.9446843	75.3849381	https://www.inaturalist.org/observations/145187895
33	Anthus trivialis	Tree Pipit	2022/12/2	0	0	11.9446843	75.3849381	https://www.inaturalist.org/observations/144972229
34	Anthus trivialis	Tree Pipit	2022/12/1	0	0	11.9446843	75.3849381	https://www.inaturalist.org/observations/144969927
35	Anthus trivialis	Tree Pipit	2022/12/1	0	0	11.9446843	75.3849381	https://www.inaturalist.org/observations/144969933
36	Anthus trivialis	Tree Pipit	2022/12/1	0	0	11.9446843	75.3849381	https://www.inaturalist.org/observations/144972339
37	Anthus trivialis	Tree Pipit	2022/11/30	0	0	11.9446843	75.3849381	https://www.inaturalist.org/observations/144910706
38	Anthus trivialis	Tree Pipit	2022/11/30	0	0	11.9446843	75.3849381	https://www.inaturalist.org/observations/144910727
39	Anthus trivialis	Tree Pipit	2022/11/30	0	0	11.9446843	75.3849381	https://www.inaturalist.org/observations/144915847
40	Anthus trivialis	Tree Pipit	2022/11/27	0	0	11.9446843	75.3849381	https://www.inaturalist.org/observations/144837333
41	Anthus trivialis	Tree Pipit	2022/11/21	0	0	11.9446843	75.3849381	https://www.inaturalist.org/observations/144354821
42	Anthus trivialis	Tree Pipit	2022/11/21	0	0	11.9446843	75.3849381	https://www.inaturalist.org/observations/144354829
43	Anthus trivialis	Tree Pipit	2022/11/21	0	0	11.9446843	75.3849381	https://www.inaturalist.org/observations/144354840
44	Anthus trivialis	Tree Pipit	2022/11/17	1	0	11.9446843	75.3849381	https://www.inaturalist.org/observations/144065539
45	Anthus trivialis	Tree Pipit	2022/11/14	0	0	11.9446843	75.3849381	https://www.inaturalist.org/observations/143986502
46	Anthus trivialis	Tree Pipit	2022/11/10	1	0	11.9446843	75.3849381	https://www.inaturalist.org/observations/143556573
47	Anthus trivialis	Tree Pipit	2022/11/10	0	0	11.9446843	75.3849381	https://www.inaturalist.org/observations/143786183
48	Anthus trivialis	Tree Pipit	2022/11/10	0	0	11.9446843	75.3849381	https://www.inaturalist.org/observations/143786189

49	Anthus trivialis	Tree Pipit	2022/11/10	0	0	11.9446843	75.3849381	https://www.inaturalist.org/observations/143786203
50	Anthus trivialis	Tree Pipit	2022/11/10	1	0	11.9446843	75.3849381	https://www.inaturalist.org/observations/143786218
51	Anthus trivialis	Tree Pipit	2022/11/10	0	0	11.9446843	75.3849381	https://www.inaturalist.org/observations/143786252
52	Anthus trivialis	Tree Pipit	2022/11/10	1	0	11.9446843	75.3849381	https://www.inaturalist.org/observations/143993605
53	Anthus trivialis	Tree Pipit	2022/11/10	1	0	11.9446843	75.3849381	https://www.inaturalist.org/observations/143993959
54	Anthus trivialis	Tree Pipit	2022/11/9	1	0	11.9446843	75.3849381	https://www.inaturalist.org/observations/143544716
55	Anthus trivialis	Tree Pipit	2022/11/9	1	0	11.9446843	75.3849381	https://www.inaturalist.org/observations/143544725
56	Anthus trivialis	Tree Pipit	2022/11/9	1	0	11.9446843	75.3849381	https://www.inaturalist.org/observations/143556446
57	Anthus trivialis	Tree Pipit	2022/11/8	1	0	11.9446843	75.3849381	https://www.inaturalist.org/observations/143257938
58	Anthus trivialis	Tree Pipit	2022/11/8	0	0	11.9446843	75.3849381	https://www.inaturalist.org/observations/143257969
59	Anthus trivialis	Tree Pipit	2022/11/8	0	0	11.9446843	75.3849381	https://www.inaturalist.org/observations/143858334
60	Anthus trivialis	Tree Pipit	2022/11/8	0	0	11.9446843	75.3849381	https://www.inaturalist.org/observations/143858341
61	Anthus trivialis	Tree Pipit	2022/10/26	0	0	11.9446843	75.3849381	https://www.inaturalist.org/observations/142205172
62	Anthus trivialis	Tree Pipit	2022/3/30	0	0	11.9446843	75.3849381	https://www.inaturalist.org/observations/116127256
63	Anthus trivialis	Tree Pipit	2020/12/16	2	0	11.9446846	75.3849381	https://www.inaturalist.org/observations/67564967
64	Anthus trivialis	Tree Pipit	2019/10/6	1	0	11.9446846	75.3849381	https://www.inaturalist.org/observations/63153870
65	Anthus trivialis	Tree Pipit	2019/10/15	1	0	12.0303367	75.2512233	https://www.inaturalist.org/observations/34573181
66	Anthus trivialis	Tree Pipit	2019/10/15	1	0	12.0303717	75.2513083	https://www.inaturalist.org/observations/34573180
67	Anthus trivialis	Tree Pipit	2019/10/15	1	0	12.0323467	75.2566564	https://www.inaturalist.org/observations/34573186
68	Anthus trivialis	Tree Pipit	2019/10/15	1	0	12.0323467	75.2566564	https://www.inaturalist.org/observations/34573187
69	Anthus trivialis	Tree Pipit	2019/10/15	1	0	12.0323467	75.2566564	https://www.inaturalist.org/observations/34573188
70	Anthus trivialis	Tree Pipit	2019/10/15	1	0	12.0323467	75.2566564	https://www.inaturalist.org/observations/34573189
71	Anthus trivialis	Tree Pipit	2019/10/15	1	0	12.0323467	75.2566564	https://www.inaturalist.org/observations/94061644
72	Anthus trivialis	Tree Pipit	2021/1/1	1	0	12.0466556	76.6268304	https://www.inaturalist.org/observations/67769482
73	Anthus trivialis	Tree Pipit	2009/1/27	0	0	12.1178461	77.7763615	https://www.inaturalist.org/observations/90633492

74	Anthus trivialis	Tree Pipit	2020/12/19	1	0	12.1262837	76.3923919	https://www.inaturalist.org/observations/93041016
75	Anthus trivialis	Tree Pipit	2012/12/22	0	0	12.1307476	76.4186495	https://www.inaturalist.org/observations/91846201
76	Anthus trivialis	Tree Pipit	2022/11/15	2	0	12.1806086	76.7262009	https://www.inaturalist.org/observations/142068881
77	Anthus trivialis	Tree Pipit	2022/11/15	2	0	12.1806086	76.7262009	https://www.inaturalist.org/observations/142068883
78	Anthus trivialis	Tree Pipit	2017/3/19	0	0	12.2252841	79.0746957	https://www.inaturalist.org/observations/95858026
79	Anthus trivialis	Tree Pipit	2022/12/8	1	0	12.2485521	76.5921897	https://www.inaturalist.org/observations/145115925
80	Anthus trivialis	Tree Pipit	2022/11/22	1	0	12.2485521	76.5921897	https://www.inaturalist.org/observations/142583513
81	Anthus trivialis	Tree Pipit	2021/12/7	0	0	12.2485521	76.5921897	https://www.inaturalist.org/observations/102652327
82	Anthus trivialis	Tree Pipit	2015/12/5	0	0	12.2561049	76.6134073	https://www.inaturalist.org/observations/98770789
83	Anthus trivialis	Tree Pipit	2015/11/15	0	0	12.2609374	76.5916724	https://www.inaturalist.org/observations/98771507
84	Anthus trivialis	Tree Pipit	2022/12/7	1	0	12.2626994	76.5676206	https://www.inaturalist.org/observations/145113814
85	Anthus trivialis	Tree Pipit	2022/12/4	1	0	12.2626994	76.5676206	https://www.inaturalist.org/observations/145112845
86	Anthus trivialis	Tree Pipit	2022/11/26	3	0	12.2626994	76.5676206	https://www.inaturalist.org/observations/143550275
87	Anthus trivialis	Tree Pipit	2022/11/13	1	0	12.2626994	76.5676206	https://www.inaturalist.org/observations/141885920
88	Anthus trivialis	Tree Pipit	2012/1/21	0	0	12.2693720	76.6119780	https://www.inaturalist.org/observations/94717024
89	Anthus trivialis	Tree Pipit	2014/12/28	0	0	12.2728479	76.2122647	https://www.inaturalist.org/observations/95548796
90	Anthus trivialis	Tree Pipit	2021/12/5	0	0	12.3092189	76.5556046	https://www.inaturalist.org/observations/102516677
91	Anthus trivialis	Tree Pipit	2022/1/13	1	0	12.3161900	76.5483528	https://www.inaturalist.org/observations/104855153
92	Anthus trivialis	Tree Pipit	2022/12/1	2	0	12.3162928	76.5503740	https://www.inaturalist.org/observations/143549932
93	Anthus trivialis	Tree Pipit	2020/11/22	0	0	12.3720686	76.6283475	https://www.inaturalist.org/observations/95014266
94	Anthus trivialis	Tree Pipit	2014/11/30	0	0	12.4331532	76.1928640	https://www.inaturalist.org/observations/145241294
95	Anthus trivialis	Tree Pipit	2018/3/17	1	0	12.4440489	76.4592855	https://www.inaturalist.org/observations/93474486
96	Anthus trivialis	Tree Pipit	2010/11/14	0	0	12.6478028	76.6229409	https://www.inaturalist.org/observations/93150604
97	Anthus trivialis	Tree Pipit	2020/10/2	2	0	12.6747309	75.0161820	https://www.inaturalist.org/observations/61917585
98	Anthus trivialis	Tree Pipit	2022/12/24	1	0	12.6788317	77.9162000	https://www.inaturalist.org/observations/144992572

99	Anthus trivialis	Tree Pipit	2022/12/24	1	0	12.6789900	77.9159817	https://www.inaturalist.org/observations/144992579
100	Anthus trivialis	Tree Pipit	2022/12/24	1	0	12.6790417	77.9159050	https://www.inaturalist.org/observations/144992795
101	Anthus trivialis	Tree Pipit	2022/12/24	1	0	12.6798550	77.9227000	https://www.inaturalist.org/observations/144982032
102	Anthus trivialis	Tree Pipit	2018/1/20	1	0	12.7575074	77.6516517	https://www.inaturalist.org/observations/65002139
103	Anthus trivialis	Tree Pipit	2016/1/9	1	0	12.8078755	77.6020038	https://www.inaturalist.org/observations/65637955
104	Anthus trivialis	Tree Pipit	2018/10/31	0	0	12.8236213	76.9098222	https://www.inaturalist.org/observations/88695058
105	Anthus trivialis	Tree Pipit	2019/2/2	2	0	12.8300625	79.7427179	https://www.inaturalist.org/observations/22612018
106	Anthus trivialis	Tree Pipit	2019/1/7	1	0	12.8557074	77.0751538	https://www.inaturalist.org/observations/89157963
107	Anthus trivialis	Tree Pipit	2021/3/21	2	0	12.8571865	77.5089110	https://www.inaturalist.org/observations/71692547
108	Anthus trivialis	Tree Pipit	2020/12/29	1	0	12.9599739	79.3672751	https://www.inaturalist.org/observations/73007442
109	Anthus trivialis	Tree Pipit	2022/1/13	2	0	12.9715987	77.5945627	https://www.inaturalist.org/observations/120864735
110	Anthus trivialis	Tree Pipit	2016/11/26	2	0	13.0033968	80.2251183	https://www.inaturalist.org/observations/22552703
111	Anthus trivialis	Tree Pipit	2016/12/25	1	0	13.0114447	80.0590803	https://www.inaturalist.org/observations/45163008
112	Anthus trivialis	Tree Pipit	2019/1/26	1	0	13.0826802	80.2707184	https://www.inaturalist.org/observations/135547091
113	Anthus trivialis	Tree Pipit	2019/4/9	2	0	13.3714183	77.6853033	https://www.inaturalist.org/observations/29238222
114	Anthus trivialis	Tree Pipit	2011/12/25	0	0	14.8809083	74.5068819	https://www.inaturalist.org/observations/96684167
115	Anthus trivialis	Tree Pipit	2019/1/28	0	0	15.0112879	76.5643027	https://www.inaturalist.org/observations/84832647
116	Anthus trivialis	Tree Pipit	2022/11/9	1	0	15.3935376	73.8207841	https://www.inaturalist.org/observations/141511817
117	Anthus trivialis	Tree Pipit	2019/1/27	0	0	16.0405923	73.5925378	https://www.inaturalist.org/observations/56497999
118	Anthus trivialis	Tree Pipit	2019/1/27	0	0	16.0405923	73.5925378	https://www.inaturalist.org/observations/56498004
119	Anthus trivialis	Tree Pipit	2020/3/7	0	0	16.2656091	77.3084366	https://www.inaturalist.org/observations/95627225
120	Anthus trivialis	Tree Pipit	2021/3/15	1	0	16.3600400	74.2958390	https://www.inaturalist.org/observations/71280390
121	Anthus trivialis	Tree Pipit	2016/11/12	2	0	16.7966026	74.2910357	https://www.inaturalist.org/observations/30795870
122	Anthus trivialis	Tree Pipit	2021/1/23	2	0	17.1900996	81.5334906	https://www.inaturalist.org/observations/114671432
123	Anthus trivialis	Tree Pipit	2017/3/7	1	0	17.8241997	75.8849796	https://www.inaturalist.org/observations/65454001

124	Anthus trivialis	Tree Pipit	2013/12/23	2	0	17.9789709	73.4106321	https://www.inaturalist.org/observations/104577279
125	Anthus trivialis	Tree Pipit	2019/1/5	0	0	18.1124372	79.0192997	https://www.inaturalist.org/observations/63620591
126	Anthus trivialis	Tree Pipit	2018/10/24	1	0	18.1453254	73.7935299	https://www.inaturalist.org/observations/84720898
127	Anthus trivialis	Tree Pipit	2018/10/7	1	0	18.1509732	73.7890851	https://www.inaturalist.org/observations/91547489
128	Anthus trivialis	Tree Pipit	2020/2/28	1	0	18.2760720	73.6227170	https://www.inaturalist.org/observations/63881501
129	Anthus trivialis	Tree Pipit	2014/2/4	1	0	18.3515415	74.3675446	https://www.inaturalist.org/observations/41677668
130	Anthus trivialis	Tree Pipit	2016/2/12	1	0	18.3565030	73.7507280	http://www.inaturalist.org/observations/2708021
131	Anthus trivialis	Tree Pipit	2018/2/5	3	0	18.3569695	73.7796896	https://www.inaturalist.org/observations/33243291
132	Anthus trivialis	Tree Pipit	2012/1/1	1	0	18.3662770	73.7558777	https://www.inaturalist.org/observations/79624650
133	Anthus trivialis	Tree Pipit	2019/2/3	1	0	18.3721362	73.7623948	https://www.inaturalist.org/observations/20246336
134	Anthus trivialis	Tree Pipit	2014/3/16	2	0	18.3735266	73.7677223	https://www.inaturalist.org/observations/56552171
135	Anthus trivialis	Tree Pipit	2014/3/16	3	0	18.3735266	73.7677223	https://www.inaturalist.org/observations/56552314
136	Anthus trivialis	Tree Pipit	2014/2/8	1	0	18.3749974	73.7697441	https://www.inaturalist.org/observations/130215883
137	Anthus trivialis	Tree Pipit	2014/2/8	2	0	18.3749974	73.7697441	https://www.inaturalist.org/observations/130216892
138	Anthus trivialis	Tree Pipit	2021/2/14	2	0	18.3758628	73.7691647	https://www.inaturalist.org/observations/75058877
139	Anthus trivialis	Tree Pipit	2021/2/13	2	0	18.3758628	73.7691647	https://www.inaturalist.org/observations/75058828
140	Anthus trivialis	Tree Pipit	2019/2/3	1	0	18.3758628	73.7691647	https://www.inaturalist.org/observations/44359008
141	Anthus trivialis	Tree Pipit	2019/2/3	1	0	18.3758628	73.7691647	https://www.inaturalist.org/observations/44359030
142	Anthus trivialis	Tree Pipit	2018/3/31	2	0	18.3758628	73.7691647	https://www.inaturalist.org/observations/44360068
143	Anthus trivialis	Tree Pipit	2018/3/31	0	0	18.3758628	73.7691647	https://www.inaturalist.org/observations/67006339
144	Anthus trivialis	Tree Pipit	2019/2/3	1	0	18.3758630	73.7691650	https://www.inaturalist.org/observations/40073872
145	Anthus trivialis	Tree Pipit	2022/2/24	1	0	18.3802793	73.7676430	https://www.inaturalist.org/observations/121368360
146	Anthus trivialis	Tree Pipit	2017/4/1	2	0	18.3802793	73.7676430	https://www.inaturalist.org/observations/41811009
147	Anthus trivialis	Tree Pipit	2017/4/1	2	0	18.3802793	73.7676430	https://www.inaturalist.org/observations/41811027
148	Anthus trivialis	Tree Pipit	2013/3/26	3	0	18.3802793	73.7676430	https://www.inaturalist.org/observations/127343141

149	Anthus trivialis	Tree Pipit	2013/2/26	2	0	18.3802793	73.7676430	https://www.inaturalist.org/observations/127343144
150	Anthus trivialis	Tree Pipit	2022/4/11	2	0	18.5010536	73.5137654	https://www.inaturalist.org/observations/121450022
151	Anthus trivialis	Tree Pipit	2017/2/10	1	0	18.5203887	73.8567438	https://www.inaturalist.org/observations/139601270
152	Anthus trivialis	Tree Pipit	2015/2/2	2	0	18.5204303	73.8567437	https://www.inaturalist.org/observations/38149359
153	Anthus trivialis	Tree Pipit	2021/10/28	1	0	18.5254899	73.8153845	https://www.inaturalist.org/observations/141470711
154	Anthus trivialis	Tree Pipit	2012/12/9	2	0	18.5320170	73.8165064	https://www.inaturalist.org/observations/128111191
155	Anthus trivialis	Tree Pipit	2021/3/27	3	0	18.5957704	73.9258938	https://www.inaturalist.org/observations/121118820
156	Anthus trivialis	Tree Pipit	2020/1/6	2	0	18.8132975	82.0189247	https://www.inaturalist.org/observations/37578255
157	Anthus trivialis	Tree Pipit	2018/4/1	1	0	19.0858505	73.7404306	https://www.inaturalist.org/observations/30471464
158	Anthus trivialis	Tree Pipit	2014/10/18	2	0	19.1505534	72.9564893	https://www.inaturalist.org/observations/57550580
159	Anthus trivialis	Tree Pipit	2014/10/18	2	0	19.1505534	72.9564893	https://www.inaturalist.org/observations/57550588
160	Anthus trivialis	Tree Pipit	2014/10/20	1	0	19.2022321	73.0025168	https://www.inaturalist.org/observations/38469767
161	Anthus trivialis	Tree Pipit	2015/2/15	2	0	19.2166700	73.0833300	http://www.inaturalist.org/observations/1301475
162	Anthus trivialis	Tree Pipit	2020/11/7	1	0	19.2194233	73.1248344	https://www.inaturalist.org/observations/64660739
163	Anthus trivialis	Tree Pipit	2020/11/7	1	0	19.2194233	73.1248344	https://www.inaturalist.org/observations/64660768
164	Anthus trivialis	Tree Pipit	2022/10/26	1	0	19.2266945	73.1143087	https://www.inaturalist.org/observations/140038190
165	Anthus trivialis	Tree Pipit	2018/12/28	2	0	19.2372539	72.9645546	https://www.inaturalist.org/observations/30320223
166	Anthus trivialis	Tree Pipit	2019/1/13	1	0	19.8240300	72.9218542	https://www.inaturalist.org/observations/19615362
167	Anthus trivialis	Tree Pipit	2022/10/30	3	0	19.9517790	73.7860274	https://www.inaturalist.org/observations/140505783
168	Anthus trivialis	Tree Pipit	2019/1/14	1	0	19.9974533	73.7898023	https://www.inaturalist.org/observations/79946146
169	Anthus trivialis	Tree Pipit	2019/1/11	1	0	19.9974533	73.7898023	https://www.inaturalist.org/observations/56498506
170	Anthus trivialis	Tree Pipit	2020/2/9	1	0	20.1529973	73.4499557	https://www.inaturalist.org/observations/92223075
171	Anthus trivialis	Tree Pipit	2021/11/23	0	0	20.2743384	79.0276479	https://www.inaturalist.org/observations/101759782
172	Anthus trivialis	Tree Pipit	2017/12/3	1	0	20.8253781	73.7007088	https://www.inaturalist.org/observations/31119843
173	Anthus trivialis	Tree Pipit	2021/1/28	1	0	21.0112840	81.4596970	https://www.inaturalist.org/observations/68723448

174	Anthus trivialis	Tree Pipit	2018/12/23	2	0	21.0146501	75.9084892	https://www.inaturalist.org/observations/54279467
175	Anthus trivialis	Tree Pipit	2021/1/28	1	0	21.0727375	81.4809042	https://www.inaturalist.org/observations/68723352
176	Anthus trivialis	Tree Pipit	2018/12/23	2	0	21.0738364	75.9456553	https://www.inaturalist.org/observations/54279250
177	Anthus trivialis	Tree Pipit	2021/5/19	1	0	21.0881974	80.6645796	https://www.inaturalist.org/observations/79375810
178	Anthus trivialis	Tree Pipit	2021/2/26	1	0	21.1862271	82.1094142	https://www.inaturalist.org/observations/70947648
179	Anthus trivialis	Tree Pipit	2020/5/20	1	0	21.1905709	81.2849169	https://www.inaturalist.org/observations/68752095
180	Anthus trivialis	Tree Pipit	2013/1/13	2	0	21.2269507	75.4812341	https://www.inaturalist.org/observations/85653687
181	Anthus trivialis	Tree Pipit	2021/11/14	1	0	21.4293093	76.3318085	https://www.inaturalist.org/observations/112489692
182	Anthus trivialis	Tree Pipit	2022/2/13	2	0	21.4351917	69.9807983	https://www.inaturalist.org/observations/120166919
183	Anthus trivialis	Tree Pipit	2018/12/10	1	0	21.6717166	82.0775522	https://www.inaturalist.org/observations/19004930
184	Anthus trivialis	Tree Pipit	2019/3/15	2	0	21.6775014	79.3397950	https://www.inaturalist.org/observations/52916336
185	Anthus trivialis	Tree Pipit	2021/2/2	0	0	22.1619405	73.4840496	https://www.inaturalist.org/observations/98494480
186	Anthus trivialis	Tree Pipit	2022/1/4	1	0	22.3135583	73.2773333	https://www.inaturalist.org/observations/120809260
187	Anthus trivialis	Tree Pipit	2022/1/4	3	0	22.3135700	73.2774083	https://www.inaturalist.org/observations/120809272
188	Anthus trivialis	Tree Pipit	2022/1/4	1	0	22.3135733	73.2773983	https://www.inaturalist.org/observations/120809262
189	Anthus trivialis	Tree Pipit	2022/1/4	2	0	22.3178442	73.2794845	https://www.inaturalist.org/observations/104429330
190	Anthus trivialis	Tree Pipit	2020/10/18	2	0	22.3178442	73.2794845	https://www.inaturalist.org/observations/62891669
191	Anthus trivialis	Tree Pipit	2021/1/19	1	0	22.3766340	70.0991232	https://www.inaturalist.org/observations/94235377
192	Anthus trivialis	Tree Pipit	2021/12/24	1	0	22.3880553	70.2220210	https://www.inaturalist.org/observations/136772361
193	Anthus trivialis	Tree Pipit	2022/3/5	2	0	22.4427777	114.0387911	https://www.inaturalist.org/observations/108496802
194	Anthus trivialis	Tree Pipit	2022/1/23	2	0	22.4917467	73.5633733	https://www.inaturalist.org/observations/120517107
195	Anthus trivialis	Tree Pipit	2022/3/16	1	0	22.4917555	114.0416703	https://www.inaturalist.org/observations/115135426
196	Anthus trivialis	Tree Pipit	2022/3/17	0	0	22.4932137	114.0451505	https://www.inaturalist.org/observations/118533835
197	Anthus trivialis	Tree Pipit	2022/3/4	0	0	22.4935050	114.0455799	https://www.inaturalist.org/observations/118533826
198	Anthus trivialis	Tree Pipit	2022/3/7	3	0	22.4950569	114.0339342	https://www.inaturalist.org/observations/108092470

199	Anthus trivialis	Tree Pipit	2022/3/6	1	0	22.4950724	114.0339231	https://www.inaturalist.org/observations/108101902
200	Anthus trivialis	Tree Pipit	2014/10/4	1	0	22.5645175	72.9288710	https://www.inaturalist.org/observations/39915535
201	Anthus trivialis	Tree Pipit	2022/1/30	1	0	22.9177673	77.7222939	https://www.inaturalist.org/observations/143852559
202	Anthus trivialis	Tree Pipit	2022/11/28	1	0	22.9916412	86.9999576	https://www.inaturalist.org/observations/143234303
203	Anthus trivialis	Tree Pipit	2022/11/28	1	0	22.9916521	86.9999962	https://www.inaturalist.org/observations/143234304
204	Anthus trivialis	Tree Pipit	2021/1/3	2	0	23.0121448	72.4354762	https://www.inaturalist.org/observations/67792438
205	Anthus trivialis	Tree Pipit	2021/1/22	1	0	23.2490759	75.7779662	https://www.inaturalist.org/observations/68485894
206	Anthus trivialis	Tree Pipit	2022/1/6	1	0	23.2639930	77.4968080	https://www.inaturalist.org/observations/104517615
207	Anthus trivialis	Tree Pipit	2022/10/8	2	0	23.2837629	71.7613757	https://www.inaturalist.org/observations/138364146
208	Anthus trivialis	Tree Pipit	2022/10/7	2	0	23.2843542	71.7611397	https://www.inaturalist.org/observations/138285336
209	Anthus trivialis	Tree Pipit	2008/11/3	2	0	23.3353213	71.7784869	https://www.inaturalist.org/observations/7417173
210	Anthus trivialis	Tree Pipit	2021/2/5	0	0	23.3728047	72.0390148	https://www.inaturalist.org/observations/69196974
211	Anthus trivialis	Tree Pipit	2021/2/5	1	0	23.3741274	72.0393457	https://www.inaturalist.org/observations/69193738
212	Anthus trivialis	Tree Pipit	2022/10/24	1	0	23.3743514	72.0394333	https://www.inaturalist.org/observations/139870593
213	Anthus trivialis	Tree Pipit	2022/10/24	1	0	23.3746466	72.0386377	https://www.inaturalist.org/observations/139870618
214	Anthus trivialis	Tree Pipit	2022/11/12	1	0	23.3746761	72.0384768	https://www.inaturalist.org/observations/141774169
215	Anthus trivialis	Tree Pipit	2022/11/12	1	0	23.3746761	72.0384768	https://www.inaturalist.org/observations/141774170
216	Anthus trivialis	Tree Pipit	2022/10/24	1	0	23.3748687	72.0388972	https://www.inaturalist.org/observations/139870615
217	Anthus trivialis	Tree Pipit	2021/2/14	1	0	23.3837311	72.0361921	https://www.inaturalist.org/observations/69676129
218	Anthus trivialis	Tree Pipit	2018/10/12	1	0	23.5862133	87.3245550	https://www.inaturalist.org/observations/17468297
219	Anthus trivialis	Tree Pipit	2017/1/9	1	0	23.6176353	87.2398899	https://www.inaturalist.org/observations/6140462
220	Anthus trivialis	Tree Pipit	2022/1/9	0	0	23.8011930	86.8275570	https://www.inaturalist.org/observations/104717608
221	Anthus trivialis	Tree Pipit	2021/12/23	0	0	24.4810528	73.6691660	https://www.inaturalist.org/observations/103518890
222	Anthus trivialis	Tree Pipit	2017/11/13	1	0	24.5854450	73.7124790	https://www.inaturalist.org/observations/31165114
223	Anthus trivialis	Tree Pipit	2022/10/16	0	0	24.5925909	72.7156274	https://www.inaturalist.org/observations/139519496

224	Anthus trivialis	Tree Pipit	2022/10/15	1	0	24.5925909	72.7156274	https://www.inaturalist.org/observations/139519414
225	Anthus trivialis	Tree Pipit	2022/10/15	1	0	24.5925909	72.7156274	https://www.inaturalist.org/observations/139519427
226	Anthus trivialis	Tree Pipit	2022/10/15	1	0	24.5925909	72.7156274	https://www.inaturalist.org/observations/139519431
227	Anthus trivialis	Tree Pipit	2020/2/2	2	0	24.6922245	75.5688610	https://www.inaturalist.org/observations/40595427
228	Anthus trivialis	Tree Pipit	2020/2/2	2	0	24.6940961	75.5698909	https://www.inaturalist.org/observations/40595413
229	Anthus trivialis	Tree Pipit	2020/2/1	2	0	24.6956557	75.5729808	https://www.inaturalist.org/observations/40595381
230	Anthus trivialis	Tree Pipit	2013/11/3	0	0	24.8421769	121.9412716	https://www.inaturalist.org/observations/62767187
231	Anthus trivialis	Tree Pipit	2021/4/2	2	0	25.3101124	91.9025971	https://www.inaturalist.org/observations/83498069
232	Anthus trivialis	Tree Pipit	2018/9/12	2	0	25.3314134	74.7759465	https://www.inaturalist.org/observations/121383772
233	Anthus trivialis	Tree Pipit	2020/2/27	1	0	26.0173274	76.5025742	https://www.inaturalist.org/observations/104673074
234	Anthus trivialis	Tree Pipit	2020/2/26	0	0	26.0173274	76.5025742	https://www.inaturalist.org/observations/104668520
235	Anthus trivialis	Tree Pipit	2017/11/26	1	0	26.9555478	78.3646801	https://www.inaturalist.org/observations/69975119
236	Anthus trivialis	Tree Pipit	2013/12/28	1	0	27.8094852	74.4351838	https://www.inaturalist.org/observations/36297247
237	Anthus trivialis	Tree Pipit	2013/1/14	1	0	28.4614365	76.8909361	https://www.inaturalist.org/observations/31516284
238	Anthus trivialis	Tree Pipit	2011/1/23	2	0	28.5324253	76.5517131	https://www.inaturalist.org/observations/31491915
239	Anthus trivialis	Tree Pipit	2017/12/6	1	0	29.3443090	79.5294318	https://www.inaturalist.org/observations/44373234
240	Anthus trivialis	Tree Pipit	2019/2/9	2	0	29.5062635	79.6164390	https://www.inaturalist.org/observations/37779449
241	Anthus trivialis	Tree Pipit	2016/12/13	1	0	29.5300138	78.7746603	https://www.inaturalist.org/observations/41680173
242	Anthus trivialis	Tree Pipit	2019/9/3	1	0	30.3411208	78.0014706	https://www.inaturalist.org/observations/32951099
243	Anthus trivialis	Tree Pipit	2017/9/14	1	0	30.6475926	78.9882083	https://www.inaturalist.org/observations/80464592
244	Anthus trivialis	Tree Pipit	2021/2/2	2	0	30.8029302	76.7298368	https://www.inaturalist.org/observations/134740655
245	Anthus trivialis	Tree Pipit	2018/8/26	0	0	30.8106399	76.7094924	https://www.inaturalist.org/observations/135481650
246	Anthus trivialis	Tree Pipit	2017/4/5	1	0	32.0419148	76.7199892	https://www.inaturalist.org/observations/64390660

No	scientific name	common name	date	NIA	NID	latitude	longitude	url
1	Bubulcus ibis	Cattle Egret	2014/4/6	2	0	-9.4875560	34.0237500	http://www.inaturalist.org/observations/610618
2	Bubulcus ibis	Cattle Egret	2014/4/26	1	0	-8.6001083	31.1840083	https://www.inaturalist.org/observations/71890161
3	Bubulcus ibis	Cattle Egret	2015/10/12	4	0	-8.1892000	36.6841050	http://www.inaturalist.org/observations/2211107
4	Bubulcus ibis	Cattle Egret	2016/10/11	4	0	-7.9785222	39.7290667	http://www.inaturalist.org/observations/4334928
5	Bubulcus ibis	Cattle Egret	2022/11/24	1	0	-7.9691861	39.7398430	https://www.inaturalist.org/observations/143722801
6	Bubulcus ibis	Cattle Egret	2008/1/5	5	0	-7.8698950	39.7549511	https://www.inaturalist.org/observations/7557358
7	Bubulcus ibis	Cattle Egret	2017/10/20	4	0	-7.7684300	38.2311233	https://www.inaturalist.org/observations/8640652
8	Bubulcus ibis	Cattle Egret	2017/10/21	2	0	-7.7660267	38.2323367	https://www.inaturalist.org/observations/8663925
9	Bubulcus ibis	Cattle Egret	2017/10/20	1	0	-7.7631535	38.2236995	https://www.inaturalist.org/observations/110898004
10	Bubulcus ibis	Cattle Egret	2017/10/21	1	0	-7.6787238	38.0504616	https://www.inaturalist.org/observations/107057081
11	Bubulcus ibis	Cattle Egret	2017/10/21	3	0	-7.6735950	38.0727183	https://www.inaturalist.org/observations/8741238
12	Bubulcus ibis	Cattle Egret	2011/11/14	2	0	-7.6672964	38.1540222	https://www.inaturalist.org/observations/68207074
13	Bubulcus ibis	Cattle Egret	2017/1/28	1	0	-7.6567900	38.0944667	https://www.inaturalist.org/observations/86568274
14	Bubulcus ibis	Cattle Egret	2014/6/4	1	0	-7.6566306	38.1118056	https://www.inaturalist.org/observations/34868393
15	Bubulcus ibis	Cattle Egret	2017/10/21	4	0	-7.6511850	38.1054783	https://www.inaturalist.org/observations/8684000
16	Bubulcus ibis	Cattle Egret	2017/10/21	3	0	-7.6494467	38.1057917	https://www.inaturalist.org/observations/8684003
17	Bubulcus ibis	Cattle Egret	2016/10/16	4	0	-7.6481889	38.1047694	http://www.inaturalist.org/observations/4427159
18	Bubulcus ibis	Cattle Egret	2017/7/24	4	0	-7.6429017	38.0442717	https://www.inaturalist.org/observations/7248179
19	Bubulcus ibis	Cattle Egret	2018/1/20	3	0	-7.3475662	37.1345766	https://www.inaturalist.org/observations/9866298
20	Bubulcus ibis	Cattle Egret	2012/12/10	2	0	-7.3203731	34.9300932	https://www.inaturalist.org/observations/24376732
21	Bubulcus ibis	Cattle Egret	2021/7/26	2	0	-6.9456008	31.2845259	https://www.inaturalist.org/observations/101079122
22	Bubulcus ibis	Cattle Egret	2021/9/28	2	0	-6.8981556	31.1865139	https://www.inaturalist.org/observations/97642833
23	Bubulcus ibis	Cattle Egret	2022/7/22	1	0	-6.8520287	37.6575913	https://www.inaturalist.org/observations/144376477
24	Bubulcus ibis	Cattle Egret	2014/7/14	3	0	-6.8510000	37.6390000	http://www.inaturalist.org/observations/4650900

25	Bubulcus ibis	Cattle Egret	2014/7/14	4	0	-6.8510000	37.6390000	http://www.inaturalist.org/observations/4650914
26	Bubulcus ibis	Cattle Egret	2018/6/3	2	0	-6.8170800	39.2910017	https://www.inaturalist.org/observations/14709036
27	Bubulcus ibis	Cattle Egret	2018/6/3	0	0	-6.8170383	39.2909333	https://www.inaturalist.org/observations/14709039
28	Bubulcus ibis	Cattle Egret	2017/10/25	2	0	-6.8125450	39.2982183	https://www.inaturalist.org/observations/8831483
29	Bubulcus ibis	Cattle Egret	2018/7/24	3	0	-6.8113244	37.6707231	https://www.inaturalist.org/observations/14644955
30	Bubulcus ibis	Cattle Egret	2017/10/15	4	0	-6.7821700	39.0375550	https://www.inaturalist.org/observations/8462590
31	Bubulcus ibis	Cattle Egret	2017/10/15	1	0	-6.7819201	39.0374488	https://www.inaturalist.org/observations/69894872
32	Bubulcus ibis	Cattle Egret	2019/6/21	2	0	-6.7814682	39.2052484	https://www.inaturalist.org/observations/28667908
33	Bubulcus ibis	Cattle Egret	2018/1/29	2	0	-6.7334853	39.2386365	https://www.inaturalist.org/observations/9726440
34	Bubulcus ibis	Cattle Egret	2022/6/8	1	0	-6.7322667	39.2381133	https://www.inaturalist.org/observations/123338754
35	Bubulcus ibis	Cattle Egret	2018/1/27	4	0	-6.4746326	38.8607025	https://www.inaturalist.org/observations/9714869
36	Bubulcus ibis	Cattle Egret	2018/6/2	2	0	-6.3243650	38.8158783	https://www.inaturalist.org/observations/14356977
37	Bubulcus ibis	Cattle Egret	2021/12/30	1	0	-6.2783067	39.3655850	https://www.inaturalist.org/observations/104471795
38	Bubulcus ibis	Cattle Egret	2020/12/20	1	0	-6.2707389	39.4184082	https://www.inaturalist.org/observations/67754538
39	Bubulcus ibis	Cattle Egret	2019/10/1	2	0	-6.2623754	39.4146368	https://www.inaturalist.org/observations/66054680
40	Bubulcus ibis	Cattle Egret	2018/9/14	6	0	-6.2036167	39.2083750	https://www.inaturalist.org/observations/16516994
41	Bubulcus ibis	Cattle Egret	2017/2/16	0	0	-6.1579134	39.1984099	https://www.inaturalist.org/observations/106108987
42	Bubulcus ibis	Cattle Egret	2019/3/4	4	0	-6.1145617	39.2546633	https://www.inaturalist.org/observations/21201700
43	Bubulcus ibis	Cattle Egret	2018/7/3	4	0	-6.1030920	39.2245606	https://www.inaturalist.org/observations/15006462
44	Bubulcus ibis	Cattle Egret	2018/7/3	3	0	-6.1030920	39.2245606	https://www.inaturalist.org/observations/15006464
45	Bubulcus ibis	Cattle Egret	2019/9/19	1	0	-6.0689507	39.3598352	https://www.inaturalist.org/observations/34028017
46	Bubulcus ibis	Cattle Egret	2019/9/17	1	0	-6.0268369	39.3979514	https://www.inaturalist.org/observations/33479476
47	Bubulcus ibis	Cattle Egret	2021/12/30	1	0	-6.0193667	39.3249000	https://www.inaturalist.org/observations/104471794
48	Bubulcus ibis	Cattle Egret	2022/1/4	1	0	-5.9938683	39.2674417	https://www.inaturalist.org/observations/104625721
49	Bubulcus ibis	Cattle Egret	2022/1/2	1	0	-5.9557667	39.3109117	https://www.inaturalist.org/observations/104570737

50	Bubulcus ibis	Cattle Egret	2022/5/2	1	0	-5.7370001	39.2911981	https://www.inaturalist.org/observations/114683557
51	Bubulcus ibis	Cattle Egret	2022/1/4	1	0	-5.7276167	39.3101900	https://www.inaturalist.org/observations/104625720
52	Bubulcus ibis	Cattle Egret	2021/2/15	1	0	-5.0942483	30.8220350	https://www.inaturalist.org/observations/70236604
53	Bubulcus ibis	Cattle Egret	2021/2/17	1	0	-5.0896147	30.8973471	https://www.inaturalist.org/observations/70890777
54	Bubulcus ibis	Cattle Egret	2021/2/17	1	0	-5.0680606	30.8810222	https://www.inaturalist.org/observations/71153834
55	Bubulcus ibis	Cattle Egret	2021/2/16	1	0	-5.0440908	30.9242083	https://www.inaturalist.org/observations/70432803
56	Bubulcus ibis	Cattle Egret	2021/2/16	1	0	-5.0434448	30.9322426	https://www.inaturalist.org/observations/70432834
57	Bubulcus ibis	Cattle Egret	2021/2/16	1	0	-5.0427995	30.9293708	https://www.inaturalist.org/observations/70432820
58	Bubulcus ibis	Cattle Egret	2009/1/9	4	0	-5.0319352	39.7755571	https://www.inaturalist.org/observations/7566718
59	Bubulcus ibis	Cattle Egret	2022/11/4	2	0	-4.9207817	39.6895454	https://www.inaturalist.org/observations/143774147
60	Bubulcus ibis	Cattle Egret	2021/11/12	1	0	-4.9003400	39.6781740	https://www.inaturalist.org/observations/106800204
61	Bubulcus ibis	Cattle Egret	2021/2/19	1	0	-4.5488619	33.1651808	https://www.inaturalist.org/observations/70593936
62	Bubulcus ibis	Cattle Egret	2021/2/20	1	0	-4.3983790	34.0608193	https://www.inaturalist.org/observations/71280210
63	Bubulcus ibis	Cattle Egret	2022/7/18	1	0	-4.0828333	36.1266383	https://www.inaturalist.org/observations/128363187
64	Bubulcus ibis	Cattle Egret	2007/10/19	3	0	-4.0425799	36.1215457	https://www.inaturalist.org/observations/19703508
65	Bubulcus ibis	Cattle Egret	2013/8/12	1	0	-4.0422507	36.1208931	https://www.inaturalist.org/observations/72276709
66	Bubulcus ibis	Cattle Egret	2022/1/26	1	0	-4.0227270	36.1210400	https://www.inaturalist.org/observations/107018806
67	Bubulcus ibis	Cattle Egret	2022/1/26	1	0	-4.0187070	36.1191430	https://www.inaturalist.org/observations/107018798
68	Bubulcus ibis	Cattle Egret	2017/2/2	1	0	-3.9889560	36.1293980	https://www.inaturalist.org/observations/92249408
69	Bubulcus ibis	Cattle Egret	2018/2/5	3	0	-3.7733503	35.9698341	https://www.inaturalist.org/observations/16659711
70	Bubulcus ibis	Cattle Egret	2021/5/20	1	0	-3.7006900	35.8632467	https://www.inaturalist.org/observations/80543858
71	Bubulcus ibis	Cattle Egret	2019/6/5	1	0	-3.6873305	35.7469595	https://www.inaturalist.org/observations/131334501
72	Bubulcus ibis	Cattle Egret	2019/6/6	1	0	-3.6473748	35.7455004	https://www.inaturalist.org/observations/131361641
73	Bubulcus ibis	Cattle Egret	2009/2/15	1	0	-3.6190593	35.8061779	https://www.inaturalist.org/observations/59802416
74	Bubulcus ibis	Cattle Egret	2022/1/11	1	0	-3.6177500	35.7381767	https://www.inaturalist.org/observations/105165956

75	Bubulcus ibis	Cattle Egret	2019/9/19	2	0	-3.6175318	35.7381038	https://www.inaturalist.org/observations/33465263
76	Bubulcus ibis	Cattle Egret	2019/11/16	1	0	-3.6145400	37.7760117	https://www.inaturalist.org/observations/37411563
77	Bubulcus ibis	Cattle Egret	2014/7/10	3	0	-3.5877105	35.8195483	https://www.inaturalist.org/observations/6620640
78	Bubulcus ibis	Cattle Egret	2019/10/26	2	0	-3.5668481	36.9836558	https://www.inaturalist.org/observations/105163073
79	Bubulcus ibis	Cattle Egret	2019/11/17	1	0	-3.5530056	38.1799861	https://www.inaturalist.org/observations/37411592
80	Bubulcus ibis	Cattle Egret	2019/11/15	1	0	-3.5420383	38.1429667	https://www.inaturalist.org/observations/37658444
81	Bubulcus ibis	Cattle Egret	2022/11/7	2	0	-3.5183795	37.3639294	https://www.inaturalist.org/observations/141907320
82	Bubulcus ibis	Cattle Egret	2022/11/17	1	0	-3.5183795	37.3639294	https://www.inaturalist.org/observations/143546000
83	Bubulcus ibis	Cattle Egret	2019/5/11	1	0	-3.5107457	36.7353385	https://www.inaturalist.org/observations/69796575
84	Bubulcus ibis	Cattle Egret	2017/4/19	5	0	-3.5000000	35.8333333	https://www.inaturalist.org/observations/6036681
85	Bubulcus ibis	Cattle Egret	2018/2/11	4	0	-3.4957341	35.7468419	https://www.inaturalist.org/observations/10001712
86	Bubulcus ibis	Cattle Egret	2019/9/26	2	0	-3.4786878	33.9378981	https://www.inaturalist.org/observations/34197188
87	Bubulcus ibis	Cattle Egret	2019/5/27	1	0	-3.4768414	35.6566458	https://www.inaturalist.org/observations/105210190
88	Bubulcus ibis	Cattle Egret	2015/2/8	2	0	-3.4741033	35.3495300	https://www.inaturalist.org/observations/23202486
89	Bubulcus ibis	Cattle Egret	2012/11/26	5	0	-3.4719654	35.8055757	https://www.inaturalist.org/observations/27611632
90	Bubulcus ibis	Cattle Egret	2012/11/27	2	0	-3.4719654	35.8055757	https://www.inaturalist.org/observations/27611683
91	Bubulcus ibis	Cattle Egret	2012/6/6	2	0	-3.4655450	35.7855790	http://www.inaturalist.org/observations/606557
92	Bubulcus ibis	Cattle Egret	2019/12/23	1	0	-3.4497456	35.8116243	https://www.inaturalist.org/observations/39882954
93	Bubulcus ibis	Cattle Egret	2018/12/29	3	0	-3.4494430	35.8057635	https://www.inaturalist.org/observations/57630606
94	Bubulcus ibis	Cattle Egret	2022/1/10	1	0	-3.4233450	36.3516800	https://www.inaturalist.org/observations/104712135
95	Bubulcus ibis	Cattle Egret	2019/2/11	3	0	-3.4227296	35.8148061	https://www.inaturalist.org/observations/43549176
96	Bubulcus ibis	Cattle Egret	2010/6/20	2	0	-3.4174140	35.8364799	https://www.inaturalist.org/observations/36732974
97	Bubulcus ibis	Cattle Egret	2008/7/22	1	0	-3.4163450	35.8349815	https://www.inaturalist.org/observations/68399735
98	Bubulcus ibis	Cattle Egret	2016/12/28	3	0	-3.4152179	35.8338545	https://www.inaturalist.org/observations/21381230
99	Bubulcus ibis	Cattle Egret	2019/9/29	1	0	-3.4147967	35.8347333	https://www.inaturalist.org/observations/35092977

100	Bubulcus ibis	Cattle Egret	2016/12/28	5	0	-3.4146690	35.8350297	https://www.inaturalist.org/observations/18818141
101	Bubulcus ibis	Cattle Egret	2013/2/10	0	0	-3.4138433	35.8383550	https://www.inaturalist.org/observations/34922162
102	Bubulcus ibis	Cattle Egret	2019/7/26	1	0	-3.4133489	35.8361624	https://www.inaturalist.org/observations/60464043
103	Bubulcus ibis	Cattle Egret	2019/4/28	1	0	-3.4124286	35.8267874	https://www.inaturalist.org/observations/128775123
104	Bubulcus ibis	Cattle Egret	2019/2/11	2	0	-3.4096779	35.8297921	https://www.inaturalist.org/observations/43547037
105	Bubulcus ibis	Cattle Egret	2019/7/26	1	0	-3.4094733	35.8402322	https://www.inaturalist.org/observations/60464013
106	Bubulcus ibis	Cattle Egret	2019/8/29	2	0	-3.4093203	37.4077853	https://www.inaturalist.org/observations/31917779
107	Bubulcus ibis	Cattle Egret	2018/7/10	3	0	-3.4034414	35.8201367	https://www.inaturalist.org/observations/14265737
108	Bubulcus ibis	Cattle Egret	2019/4/28	1	0	-3.3927223	35.8355422	https://www.inaturalist.org/observations/128775133
109	Bubulcus ibis	Cattle Egret	2018/10/26	2	0	-3.3862912	36.8621330	https://www.inaturalist.org/observations/107185708
110	Bubulcus ibis	Cattle Egret	2016/2/4	2	0	-3.3860360	35.8446300	http://www.inaturalist.org/observations/2784767
111	Bubulcus ibis	Cattle Egret	2022/2/6	1	0	-3.3808202	35.8538574	https://www.inaturalist.org/observations/106504371
112	Bubulcus ibis	Cattle Egret	2021/2/21	1	0	-3.3803758	36.8370165	https://www.inaturalist.org/observations/71781138
113	Bubulcus ibis	Cattle Egret	2007/1/24	1	0	-3.3702837	35.8463396	https://www.inaturalist.org/observations/143769156
114	Bubulcus ibis	Cattle Egret	2019/3/15	1	0	-3.3604281	36.6097706	https://www.inaturalist.org/observations/119546667
115	Bubulcus ibis	Cattle Egret	2019/9/2	1	0	-3.3461103	37.3406924	https://www.inaturalist.org/observations/31976147
116	Bubulcus ibis	Cattle Egret	2017/8/27	1	0	-3.3179828	36.8825110	https://www.inaturalist.org/observations/137149122
117	Bubulcus ibis	Cattle Egret	2022/10/31	1	0	-3.3109777	37.6922379	https://www.inaturalist.org/observations/140665697
118	Bubulcus ibis	Cattle Egret	2022/1/4	1	0	-3.2886671	39.0550547	https://www.inaturalist.org/observations/112714234
119	Bubulcus ibis	Cattle Egret	2014/9/14	2	0	-3.2499515	36.7499542	https://www.inaturalist.org/observations/10019908
120	Bubulcus ibis	Cattle Egret	2019/6/17	1	0	-3.2437483	36.8102826	https://www.inaturalist.org/observations/128232048
121	Bubulcus ibis	Cattle Egret	2019/2/26	2	0	-3.2304650	35.5438600	https://www.inaturalist.org/observations/21110240
122	Bubulcus ibis	Cattle Egret	2019/2/26	3	0	-3.2288200	35.5484517	https://www.inaturalist.org/observations/21110250
123	Bubulcus ibis	Cattle Egret	2018/5/21	5	0	-3.2261675	35.4466595	https://www.inaturalist.org/observations/18878671
124	Bubulcus ibis	Cattle Egret	2018/12/30	2	0	-3.2261675	35.4466595	https://www.inaturalist.org/observations/57864472

125	Bubulcus ibis	Cattle Egret	2014/11/17	4	0	-3.2102663	35.6005668	https://www.inaturalist.org/observations/19774440
126	Bubulcus ibis	Cattle Egret	2019/2/26	2	0	-3.2100400	35.5997283	https://www.inaturalist.org/observations/21109157
127	Bubulcus ibis	Cattle Egret	2013/3/6	1	0	-3.2094306	35.5985983	https://www.inaturalist.org/observations/98872354
128	Bubulcus ibis	Cattle Egret	2018/4/19	2	0	-3.2090545	35.5807452	https://www.inaturalist.org/observations/14482269
129	Bubulcus ibis	Cattle Egret	2019/6/26	1	0	-3.1967172	35.5814899	https://www.inaturalist.org/observations/33163616
130	Bubulcus ibis	Cattle Egret	2022/1/4	3	0	-3.1960257	35.5638640	https://www.inaturalist.org/observations/116447000
131	Bubulcus ibis	Cattle Egret	1993/12/27	1	0	-3.1884903	35.5725635	https://www.inaturalist.org/observations/135823408
132	Bubulcus ibis	Cattle Egret	2017/7/11	1	0	-3.1810280	35.5728850	https://www.inaturalist.org/observations/131295306
133	Bubulcus ibis	Cattle Egret	2021/4/12	3	0	-3.1808117	35.5716583	https://www.inaturalist.org/observations/80556000
134	Bubulcus ibis	Cattle Egret	2020/12/23	2	0	-3.1806918	35.5579722	https://www.inaturalist.org/observations/67786537
135	Bubulcus ibis	Cattle Egret	2020/12/23	1	0	-3.1806918	35.5579722	https://www.inaturalist.org/observations/67786553
136	Bubulcus ibis	Cattle Egret	2019/4/27	1	0	-3.1798950	35.5732555	https://www.inaturalist.org/observations/128030508
137	Bubulcus ibis	Cattle Egret	2022/8/11	1	0	-3.1795404	35.5049421	https://www.inaturalist.org/observations/134625817
138	Bubulcus ibis	Cattle Egret	2016/9/7	1	0	-3.1788920	35.5684429	https://www.inaturalist.org/observations/106135320
139	Bubulcus ibis	Cattle Egret	2007/1/27	2	0	-3.1768595	35.5750216	https://www.inaturalist.org/observations/143836079
140	Bubulcus ibis	Cattle Egret	2007/1/27	2	0	-3.1768595	35.5750216	https://www.inaturalist.org/observations/143836114
141	Bubulcus ibis	Cattle Egret	2012/4/15	1	0	-3.1743475	35.5645975	https://www.inaturalist.org/observations/43851118
142	Bubulcus ibis	Cattle Egret	2014/9/23	3	0	-3.1740030	35.5638920	http://www.inaturalist.org/observations/1988332
143	Bubulcus ibis	Cattle Egret	2017/2/28	4	0	-3.1735485	35.5745544	https://www.inaturalist.org/observations/9524641
144	Bubulcus ibis	Cattle Egret	2011/6/10	2	0	-3.1727217	35.5684436	https://www.inaturalist.org/observations/54829201
145	Bubulcus ibis	Cattle Egret	2021/10/16	2	0	-3.1723860	35.5231740	https://www.inaturalist.org/observations/100184144
146	Bubulcus ibis	Cattle Egret	2021/2/6	1	0	-3.1679226	35.5804599	https://www.inaturalist.org/observations/70506819
147	Bubulcus ibis	Cattle Egret	2020/12/24	2	0	-3.1659640	35.5351422	https://www.inaturalist.org/observations/82192715
148	Bubulcus ibis	Cattle Egret	2014/9/30	3	0	-3.1654450	35.5749820	http://www.inaturalist.org/observations/2511757
149	Bubulcus ibis	Cattle Egret	1991/3/12	5	0	-3.1617522	35.5876697	http://www.inaturalist.org/observations/4456394

150	Bubulcus ibis	Cattle Egret	2018/6/24	4	0	-3.1617522	35.5876697	https://www.inaturalist.org/observations/15092906
151	Bubulcus ibis	Cattle Egret	2019/7/16	3	0	-3.1617522	35.5876697	https://www.inaturalist.org/observations/30282154
152	Bubulcus ibis	Cattle Egret	2018/1/23	1	0	-3.1617522	35.5876697	https://www.inaturalist.org/observations/81701024
153	Bubulcus ibis	Cattle Egret	2009/2/25	1	0	-3.1617522	35.5876697	https://www.inaturalist.org/observations/92408022
154	Bubulcus ibis	Cattle Egret	2019/6/22	1	0	-3.1617522	35.5876697	https://www.inaturalist.org/observations/138735941
155	Bubulcus ibis	Cattle Egret	2007/2/10	1	0	-3.1617522	35.5876697	https://www.inaturalist.org/observations/142357876
156	Bubulcus ibis	Cattle Egret	2022/8/11	3	0	-3.1613842	35.5222620	https://www.inaturalist.org/observations/138434063
157	Bubulcus ibis	Cattle Egret	2019/7/27	1	0	-3.1590952	35.5498933	https://www.inaturalist.org/observations/60655856
158	Bubulcus ibis	Cattle Egret	2019/8/23	1	0	-3.1587970	35.5499340	https://www.inaturalist.org/observations/35919995
159	Bubulcus ibis	Cattle Egret	2017/11/10	3	0	-3.1586443	35.5501784	https://www.inaturalist.org/observations/19390270
160	Bubulcus ibis	Cattle Egret	2007/10/17	4	0	-3.1586220	35.5497866	https://www.inaturalist.org/observations/19686603
161	Bubulcus ibis	Cattle Egret	2018/10/21	5	0	-3.1586117	35.5500617	https://www.inaturalist.org/observations/18027665
162	Bubulcus ibis	Cattle Egret	2016/8/27	4	0	-3.1583242	35.5495609	https://www.inaturalist.org/observations/21262807
163	Bubulcus ibis	Cattle Egret	2022/1/15	1	0	-3.1578533	35.5159683	https://www.inaturalist.org/observations/105567361
164	Bubulcus ibis	Cattle Egret	2007/10/16	3	0	-3.1577880	35.5496998	https://www.inaturalist.org/observations/19684356
165	Bubulcus ibis	Cattle Egret	2014/11/18	3	0	-3.1575279	35.5501712	https://www.inaturalist.org/observations/19793603
166	Bubulcus ibis	Cattle Egret	2011/2/9	1	0	-3.1475746	35.5966606	https://www.inaturalist.org/observations/68921781
167	Bubulcus ibis	Cattle Egret	2022/10/2	2	0	-3.1133530	35.6052004	https://www.inaturalist.org/observations/139777525
168	Bubulcus ibis	Cattle Egret	2015/7/18	3	0	-3.0699469	35.4389479	http://www.inaturalist.org/observations/3054131
169	Bubulcus ibis	Cattle Egret	2019/6/3	2	0	-3.0332250	33.2096467	https://www.inaturalist.org/observations/27620390
170	Bubulcus ibis	Cattle Egret	2009/11/22	1	0	-2.9088530	35.3900710	https://www.inaturalist.org/observations/142132993
171	Bubulcus ibis	Cattle Egret	2019/6/1	2	0	-2.8698083	31.7235750	https://www.inaturalist.org/observations/27368482
172	Bubulcus ibis	Cattle Egret	2018/4/18	4	0	-2.7753233	35.1221800	https://www.inaturalist.org/observations/14214652
173	Bubulcus ibis	Cattle Egret	2019/9/14	2	0	-2.7070958	35.4594877	https://www.inaturalist.org/observations/33744612
174	Bubulcus ibis	Cattle Egret	2019/2/27	1	0	-2.7020278	37.3079694	https://www.inaturalist.org/observations/72194023

175	Bubulcus ibis	Cattle Egret	2018/8/30	2	0	-2.6869237	37.2906449	https://www.inaturalist.org/observations/19326351
176	Bubulcus ibis	Cattle Egret	2018/8/30	2	0	-2.6869237	37.2906449	https://www.inaturalist.org/observations/19326353
177	Bubulcus ibis	Cattle Egret	2018/8/30	2	0	-2.6869237	37.2906449	https://www.inaturalist.org/observations/19326354
178	Bubulcus ibis	Cattle Egret	2018/8/30	2	0	-2.6869237	37.2906449	https://www.inaturalist.org/observations/19326359
179	Bubulcus ibis	Cattle Egret	2018/8/30	2	0	-2.6869237	37.2906449	https://www.inaturalist.org/observations/19326364
180	Bubulcus ibis	Cattle Egret	2018/8/30	3	0	-2.6869237	37.2906449	https://www.inaturalist.org/observations/19326367
181	Bubulcus ibis	Cattle Egret	2018/8/30	2	0	-2.6869237	37.2906449	https://www.inaturalist.org/observations/19326374
182	Bubulcus ibis	Cattle Egret	2022/1/12	1	0	-2.6807486	37.2607387	https://www.inaturalist.org/observations/112727539
183	Bubulcus ibis	Cattle Egret	2014/11/11	4	0	-2.6751562	37.2946130	https://www.inaturalist.org/observations/19979526
184	Bubulcus ibis	Cattle Egret	2012/10/10	2	0	-2.6684379	37.2326660	https://www.inaturalist.org/observations/5119614
185	Bubulcus ibis	Cattle Egret	2011/4/22	1	0	-2.6656810	37.2817880	https://www.inaturalist.org/observations/50093415
186	Bubulcus ibis	Cattle Egret	2022/10/2	1	0	-2.6650945	37.3064952	https://www.inaturalist.org/observations/139959322
187	Bubulcus ibis	Cattle Egret	2021/8/27	1	0	-2.6639468	37.2756180	https://www.inaturalist.org/observations/98005065
188	Bubulcus ibis	Cattle Egret	2012/10/10	2	0	-2.6629507	37.2601318	https://www.inaturalist.org/observations/5119911
189	Bubulcus ibis	Cattle Egret	2018/8/31	2	0	-2.6627519	37.2978222	https://www.inaturalist.org/observations/19324367
190	Bubulcus ibis	Cattle Egret	2019/6/1	2	0	-2.6622067	31.7537250	https://www.inaturalist.org/observations/27367798
191	Bubulcus ibis	Cattle Egret	2021/8/27	1	0	-2.6621651	37.2334805	https://www.inaturalist.org/observations/98005148
192	Bubulcus ibis	Cattle Egret	2021/8/25	1	0	-2.6613203	37.3037707	https://www.inaturalist.org/observations/105862138
193	Bubulcus ibis	Cattle Egret	2022/10/2	1	0	-2.6612480	37.2970345	https://www.inaturalist.org/observations/139959347
194	Bubulcus ibis	Cattle Egret	2015/4/14	1	0	-2.6604655	37.2395324	https://www.inaturalist.org/observations/86832676
195	Bubulcus ibis	Cattle Egret	2011/2/18	1	0	-2.6602199	37.2115829	https://www.inaturalist.org/observations/108113129
196	Bubulcus ibis	Cattle Egret	2021/8/25	1	0	-2.6593608	37.2303970	https://www.inaturalist.org/observations/107424117
197	Bubulcus ibis	Cattle Egret	2018/8/30	3	0	-2.6590121	37.2388448	https://www.inaturalist.org/observations/19328953
198	Bubulcus ibis	Cattle Egret	2018/8/30	3	0	-2.6590121	37.2388448	https://www.inaturalist.org/observations/19328984
199	Bubulcus ibis	Cattle Egret	2018/8/30	3	0	-2.6590121	37.2388448	https://www.inaturalist.org/observations/19328985

200	Bubulcus ibis	Cattle Egret	2018/8/30	4	0	-2.6590121	37.2388448	https://www.inaturalist.org/observations/19328988
201	Bubulcus ibis	Cattle Egret	2017/6/3	2	0	-2.6584017	37.2919350	https://www.inaturalist.org/observations/135164718
202	Bubulcus ibis	Cattle Egret	2021/8/25	1	0	-2.6581373	37.2316994	https://www.inaturalist.org/observations/107424132
203	Bubulcus ibis	Cattle Egret	2017/11/17	5	0	-2.6554919	37.2275727	https://www.inaturalist.org/observations/19203342
204	Bubulcus ibis	Cattle Egret	2021/7/23	1	0	-2.6547444	37.2543848	https://www.inaturalist.org/observations/93006930
205	Bubulcus ibis	Cattle Egret	2022/10/3	2	0	-2.6542414	37.2612115	https://www.inaturalist.org/observations/140056951
206	Bubulcus ibis	Cattle Egret	2013/9/23	1	0	-2.6526742	37.2605650	https://www.inaturalist.org/observations/110104709
207	Bubulcus ibis	Cattle Egret	2019/1/7	2	0	-2.6520778	37.2748028	https://www.inaturalist.org/observations/60870334
208	Bubulcus ibis	Cattle Egret	2020/2/23	1	0	-2.6520157	37.2607672	https://www.inaturalist.org/observations/39322575
209	Bubulcus ibis	Cattle Egret	2012/10/10	2	0	-2.6519761	37.2271729	https://www.inaturalist.org/observations/5120040
210	Bubulcus ibis	Cattle Egret	2012/10/10	2	0	-2.6519761	37.2436523	https://www.inaturalist.org/observations/5119978
211	Bubulcus ibis	Cattle Egret	2021/9/14	1	0	-2.6517776	37.2750937	https://www.inaturalist.org/observations/94829555
212	Bubulcus ibis	Cattle Egret	2021/8/28	1	0	-2.6513919	37.2849455	https://www.inaturalist.org/observations/98020025
213	Bubulcus ibis	Cattle Egret	2010/10/23	1	0	-2.6511383	37.2753707	https://www.inaturalist.org/observations/131931046
214	Bubulcus ibis	Cattle Egret	2021/8/25	1	0	-2.6503564	37.2774348	https://www.inaturalist.org/observations/105859564
215	Bubulcus ibis	Cattle Egret	2019/10/18	3	0	-2.6502156	37.2434220	https://www.inaturalist.org/observations/41008105
216	Bubulcus ibis	Cattle Egret	2021/9/13	2	0	-2.6495399	37.2777156	https://www.inaturalist.org/observations/94729488
217	Bubulcus ibis	Cattle Egret	2017/8/30	5	0	-2.6491639	37.2317219	https://www.inaturalist.org/observations/7898467
218	Bubulcus ibis	Cattle Egret	2021/3/31	1	0	-2.6490049	37.2511226	https://www.inaturalist.org/observations/131183390
219	Bubulcus ibis	Cattle Egret	2014/12/24	4	0	-2.6474620	37.2428210	http://www.inaturalist.org/observations/1500562
220	Bubulcus ibis	Cattle Egret	2021/12/30	1	0	-2.6410689	37.1646500	https://www.inaturalist.org/observations/103947900
221	Bubulcus ibis	Cattle Egret	2018/4/4	3	0	-2.6366130	37.2244511	https://www.inaturalist.org/observations/32601591
222	Bubulcus ibis	Cattle Egret	2019/11/29	2	0	-2.6299650	34.7898250	https://www.inaturalist.org/observations/68811058
223	Bubulcus ibis	Cattle Egret	2015/9/28	1	0	-2.6077855	37.2076656	https://www.inaturalist.org/observations/134643681
224	Bubulcus ibis	Cattle Egret	2019/10/3	1	0	-2.5924467	37.2491183	https://www.inaturalist.org/observations/36427325

225	Bubulcus ibis	Cattle Egret	2019/6/1	2	0	-2.5880533	31.7255467	https://www.inaturalist.org/observations/27367789
226	Bubulcus ibis	Cattle Egret	2018/4/16	3	0	-2.5541217	34.9234117	https://www.inaturalist.org/observations/14051026
227	Bubulcus ibis	Cattle Egret	2009/1/9	1	0	-2.5403113	34.8312034	https://www.inaturalist.org/observations/135950818
228	Bubulcus ibis	Cattle Egret	2020/5/3	1	0	-2.5294503	37.3256144	https://www.inaturalist.org/observations/45067759
229	Bubulcus ibis	Cattle Egret	2018/1/28	1	0	-2.4880531	40.3032240	https://www.inaturalist.org/observations/91836977
230	Bubulcus ibis	Cattle Egret	2019/6/1	1	0	-2.4859433	31.7102417	https://www.inaturalist.org/observations/27193397
231	Bubulcus ibis	Cattle Egret	1996/2/4	1	0	-2.4542055	35.0066638	https://www.inaturalist.org/observations/69069114
232	Bubulcus ibis	Cattle Egret	2005/12/23	2	0	-2.3790378	34.8669253	https://www.inaturalist.org/observations/22348398
233	Bubulcus ibis	Cattle Egret	2007/1/28	1	0	-2.3541275	34.8221937	https://www.inaturalist.org/observations/143845788
234	Bubulcus ibis	Cattle Egret	2019/12/28	2	0	-2.3435563	36.0458437	https://www.inaturalist.org/observations/41142250
235	Bubulcus ibis	Cattle Egret	2019/12/28	2	0	-2.3435563	36.0458437	https://www.inaturalist.org/observations/40340883
236	Bubulcus ibis	Cattle Egret	2009/2/11	1	0	-2.3333333	34.8333333	https://www.inaturalist.org/observations/59796964
237	Bubulcus ibis	Cattle Egret	2018/8/22	0	0	-2.3333333	34.8333333	https://www.inaturalist.org/observations/37850710
238	Bubulcus ibis	Cattle Egret	2007/12/7	1	0	-2.3333333	34.8333333	https://www.inaturalist.org/observations/69758958
239	Bubulcus ibis	Cattle Egret	2011/3/10	2	0	-2.3333333	34.8333333	https://www.inaturalist.org/observations/107783866
240	Bubulcus ibis	Cattle Egret	2008/6/11	3	0	-2.2882841	40.1266621	https://www.inaturalist.org/observations/18475731
241	Bubulcus ibis	Cattle Egret	2020/1/2	1	0	-2.2498505	33.8467624	https://www.inaturalist.org/observations/121068402
242	Bubulcus ibis	Cattle Egret	2012/12/5	2	0	-2.2356060	34.9198720	http://www.inaturalist.org/observations/615615
243	Bubulcus ibis	Cattle Egret	2005/12/23	2	0	-2.1430103	34.9850333	https://www.inaturalist.org/observations/22335364
244	Bubulcus ibis	Cattle Egret	2009/11/23	1	0	-1.8936000	34.6856510	https://www.inaturalist.org/observations/142162779
245	Bubulcus ibis	Cattle Egret	2017/8/14	2	0	-1.8715841	37.2485859	https://www.inaturalist.org/observations/21868132
246	Bubulcus ibis	Cattle Egret	2017/6/22	1	0	-1.8698563	37.2469381	https://www.inaturalist.org/observations/21868134
247	Bubulcus ibis	Cattle Egret	2017/6/22	3	0	-1.8698407	37.2469390	https://www.inaturalist.org/observations/21868133
248	Bubulcus ibis	Cattle Egret	2020/5/13	1	0	-1.6454576	36.8659370	https://www.inaturalist.org/observations/45794457
249	Bubulcus ibis	Cattle Egret	2005/3/31	3	0	-1.6161530	31.6197530	http://www.inaturalist.org/observations/1977388

250	Bubulcus ibis	Cattle Egret	2019/5/31	1	0	-1.5905150	31.6709233	https://www.inaturalist.org/observations/27093447
251	Bubulcus ibis	Cattle Egret	2019/5/31	1	0	-1.5899067	31.6744117	https://www.inaturalist.org/observations/27093436
252	Bubulcus ibis	Cattle Egret	2019/5/31	2	0	-1.5026350	31.7412633	https://www.inaturalist.org/observations/27039510
253	Bubulcus ibis	Cattle Egret	2021/8/5	1	0	-1.4949250	37.0991900	https://www.inaturalist.org/observations/90041532
254	Bubulcus ibis	Cattle Egret	2019/6/6	4	0	-1.4786567	33.8055717	https://www.inaturalist.org/observations/28111095
255	Bubulcus ibis	Cattle Egret	2022/12/6	1	0	-1.4734607	37.0700486	https://www.inaturalist.org/observations/144354494
256	Bubulcus ibis	Cattle Egret	2021/12/21	1	0	-1.4482387	34.9624024	https://www.inaturalist.org/observations/112650960
257	Bubulcus ibis	Cattle Egret	2008/6/4	1	0	-1.4432517	35.2080989	https://www.inaturalist.org/observations/110433544
258	Bubulcus ibis	Cattle Egret	2008/6/4	1	0	-1.4432517	35.2080989	https://www.inaturalist.org/observations/131629881
259	Bubulcus ibis	Cattle Egret	2009/12/8	2	0	-1.4367698	35.0120897	https://www.inaturalist.org/observations/18044076
260	Bubulcus ibis	Cattle Egret	2022/4/2	1	0	-1.4130381	36.9306705	https://www.inaturalist.org/observations/110382067
261	Bubulcus ibis	Cattle Egret	2018/8/5	3	0	-1.3930934	36.8962707	https://www.inaturalist.org/observations/15318197
262	Bubulcus ibis	Cattle Egret	2021/7/12	2	0	-1.3882118	36.8915967	https://www.inaturalist.org/observations/86702476
263	Bubulcus ibis	Cattle Egret	2009/12/10	2	0	-1.3820834	36.8639374	http://www.inaturalist.org/observations/28296
264	Bubulcus ibis	Cattle Egret	2022/12/2	1	0	-1.3766714	36.8389792	https://www.inaturalist.org/observations/144482144
265	Bubulcus ibis	Cattle Egret	2022/12/2	1	0	-1.3766714	36.8389792	https://www.inaturalist.org/observations/144483155
266	Bubulcus ibis	Cattle Egret	2017/6/21	5	0	-1.3762541	36.8360892	https://www.inaturalist.org/observations/17364419
267	Bubulcus ibis	Cattle Egret	2022/6/22	1	0	-1.3706931	36.8506763	https://www.inaturalist.org/observations/131931422
268	Bubulcus ibis	Cattle Egret	2009/8/22	3	0	-1.3666667	36.8333333	https://www.inaturalist.org/observations/4959554
269	Bubulcus ibis	Cattle Egret	2017/9/8	4	0	-1.3666667	36.8333333	https://www.inaturalist.org/observations/8088361
270	Bubulcus ibis	Cattle Egret	2018/7/13	2	0	-1.3631818	36.8455411	https://www.inaturalist.org/observations/46646801
271	Bubulcus ibis	Cattle Egret	2019/6/13	1	0	-1.3624021	36.7964597	https://www.inaturalist.org/observations/52561340
272	Bubulcus ibis	Cattle Egret	2022/6/1	1	0	-1.3618845	36.8483800	https://www.inaturalist.org/observations/119851688
273	Bubulcus ibis	Cattle Egret	2018/7/20	1	0	-1.3615881	36.8451532	https://www.inaturalist.org/observations/38658542
274	Bubulcus ibis	Cattle Egret	2022/11/7	1	0	-1.3612073	36.8473286	https://www.inaturalist.org/observations/141531510

275	Bubulcus ibis	Cattle Egret	2017/3/5	2	0	-1.3608909	36.8107151	https://www.inaturalist.org/observations/5259088
276	Bubulcus ibis	Cattle Egret	2019/7/31	1	0	-1.3600610	36.8237662	https://www.inaturalist.org/observations/47011706
277	Bubulcus ibis	Cattle Egret	2019/4/28	3	0	-1.3585375	36.8530977	https://www.inaturalist.org/observations/23595570
278	Bubulcus ibis	Cattle Egret	2008/6/20	1	0	-1.3569641	36.8465564	https://www.inaturalist.org/observations/61542362
279	Bubulcus ibis	Cattle Egret	2018/6/28	1	0	-1.3569641	36.8465564	https://www.inaturalist.org/observations/64369315
280	Bubulcus ibis	Cattle Egret	2022/11/12	1	0	-1.3569641	36.8465564	https://www.inaturalist.org/observations/144468008
281	Bubulcus ibis	Cattle Egret	2022/12/25	1	0	-1.3569641	36.8465564	https://www.inaturalist.org/observations/145036027
282	Bubulcus ibis	Cattle Egret	2018/2/25	3	0	-1.3518633	36.8205300	https://www.inaturalist.org/observations/9988554
283	Bubulcus ibis	Cattle Egret	2022/6/24	1	0	-1.3508133	36.7987117	https://www.inaturalist.org/observations/125331272
284	Bubulcus ibis	Cattle Egret	2022/6/27	1	0	-1.3485139	36.7975379	https://www.inaturalist.org/observations/125325222
285	Bubulcus ibis	Cattle Egret	2022/6/25	2	0	-1.3480588	36.8150804	https://www.inaturalist.org/observations/123484989
286	Bubulcus ibis	Cattle Egret	2022/6/27	1	0	-1.3477761	36.7964818	https://www.inaturalist.org/observations/125309068
287	Bubulcus ibis	Cattle Egret	2017/7/3	1	0	-1.3468442	36.8502386	https://www.inaturalist.org/observations/108771346
288	Bubulcus ibis	Cattle Egret	2021/9/13	1	0	-1.3397181	36.8089449	https://www.inaturalist.org/observations/97139360
289	Bubulcus ibis	Cattle Egret	2022/10/14	2	0	-1.3396226	36.8101058	https://www.inaturalist.org/observations/138743031
290	Bubulcus ibis	Cattle Egret	2022/2/19	1	0	-1.3388672	36.8096324	https://www.inaturalist.org/observations/110309781
291	Bubulcus ibis	Cattle Egret	2018/8/4	3	0	-1.3381884	36.8095855	https://www.inaturalist.org/observations/15100438
292	Bubulcus ibis	Cattle Egret	2021/2/20	2	0	-1.3343397	36.7796825	https://www.inaturalist.org/observations/86422917
293	Bubulcus ibis	Cattle Egret	2022/6/29	2	0	-1.3342348	36.7153765	https://www.inaturalist.org/observations/124525186
294	Bubulcus ibis	Cattle Egret	2019/4/28	3	0	-1.3339467	36.7574806	https://www.inaturalist.org/observations/24160390
295	Bubulcus ibis	Cattle Egret	2018/8/19	3	0	-1.3310650	36.7096500	https://www.inaturalist.org/observations/15715138
296	Bubulcus ibis	Cattle Egret	2019/6/5	4	0	-1.3197607	34.9500236	https://www.inaturalist.org/observations/26658886
297	Bubulcus ibis	Cattle Egret	2018/8/30	2	0	-1.3079471	36.8040254	https://www.inaturalist.org/observations/16026542
298	Bubulcus ibis	Cattle Egret	2020/1/5	1	0	-1.3013221	35.0058454	https://www.inaturalist.org/observations/121086627
299	Bubulcus ibis	Cattle Egret	2019/12/22	1	0	-1.2920659	36.8219462	https://www.inaturalist.org/observations/39882956

300	Bubulcus ibis	Cattle Egret	2018/3/22	4	0	-1.2001686	35.1177979	https://www.inaturalist.org/observations/11187459
301	Bubulcus ibis	Cattle Egret	2021/11/21	1	0	-1.0954229	37.0168856	https://www.inaturalist.org/observations/101604010
302	Bubulcus ibis	Cattle Egret	2020/5/30	1	0	-1.0737650	35.2167600	https://www.inaturalist.org/observations/47883779
303	Bubulcus ibis	Cattle Egret	2019/7/18	4	0	-1.0718388	35.2555783	https://www.inaturalist.org/observations/29068900
304	Bubulcus ibis	Cattle Egret	2022/12/22	1	0	-1.0656889	35.2334226	https://www.inaturalist.org/observations/144843508
305	Bubulcus ibis	Cattle Egret	2013/1/13	3	0	-0.9573523	36.2582438	https://www.inaturalist.org/observations/83994828
306	Bubulcus ibis	Cattle Egret	2016/12/27	4	0	-0.8855660	36.3062100	https://www.inaturalist.org/observations/4921113
307	Bubulcus ibis	Cattle Egret	2017/2/21	2	0	-0.8576280	36.3650940	https://www.inaturalist.org/observations/33959818
308	Bubulcus ibis	Cattle Egret	2016/11/28	2	0	-0.8262800	36.3367500	https://www.inaturalist.org/observations/80691540
309	Bubulcus ibis	Cattle Egret	2022/9/30	2	0	-0.8165637	36.3237288	https://www.inaturalist.org/observations/137676358
310	Bubulcus ibis	Cattle Egret	1992/10/16	2	0	-0.8052417	36.3911222	https://www.inaturalist.org/observations/18868613
311	Bubulcus ibis	Cattle Egret	2022/10/1	2	0	-0.7850262	36.4147881	https://www.inaturalist.org/observations/139899849
312	Bubulcus ibis	Cattle Egret	2022/7/5	1	0	-0.7789079	36.4097941	https://www.inaturalist.org/observations/128167708
313	Bubulcus ibis	Cattle Egret	2022/6/18	0	0	-0.7709246	37.3613743	https://www.inaturalist.org/observations/122677982
314	Bubulcus ibis	Cattle Egret	2010/6/10	2	0	-0.7703224	36.3654331	https://www.inaturalist.org/observations/107637985
315	Bubulcus ibis	Cattle Egret	2022/7/3	1	0	-0.7674751	36.4071870	https://www.inaturalist.org/observations/131071658
316	Bubulcus ibis	Cattle Egret	2013/1/13	5	0	-0.6773340	34.7796030	https://www.inaturalist.org/observations/7543354
317	Bubulcus ibis	Cattle Egret	2017/5/1	1	0	-0.6161137	30.9801069	https://www.inaturalist.org/observations/140110821
318	Bubulcus ibis	Cattle Egret	2016/11/19	2	0	-0.5694167	29.7013889	http://www.inaturalist.org/observations/4675119
319	Bubulcus ibis	Cattle Egret	2022/10/14	2	0	-0.5583740	30.9806575	https://www.inaturalist.org/observations/139950795
320	Bubulcus ibis	Cattle Egret	2021/10/30	1	0	-0.5520000	36.5460000	https://www.inaturalist.org/observations/102253295
321	Bubulcus ibis	Cattle Egret	2021/4/25	1	0	-0.4785843	36.1174129	https://www.inaturalist.org/observations/75658611
322	Bubulcus ibis	Cattle Egret	2018/8/12	4	0	-0.4764208	36.2661483	https://www.inaturalist.org/observations/15341591
323	Bubulcus ibis	Cattle Egret	2018/8/12	3	0	-0.4762760	36.2661095	https://www.inaturalist.org/observations/15342032
324	Bubulcus ibis	Cattle Egret	2021/7/30	1	0	-0.4585700	36.0850231	https://www.inaturalist.org/observations/97303702

325	Bubulcus ibis	Cattle Egret	2016/3/16	2	0	-0.4500000	36.2500000	https://www.inaturalist.org/observations/23654614
326	Bubulcus ibis	Cattle Egret	2019/10/26	1	0	-0.4290789	34.2057276	https://www.inaturalist.org/observations/40832274
327	Bubulcus ibis	Cattle Egret	2020/3/15	1	0	-0.4284658	34.2060126	https://www.inaturalist.org/observations/110666998
328	Bubulcus ibis	Cattle Egret	2021/3/4	1	0	-0.4233639	36.1296167	https://www.inaturalist.org/observations/103962967
329	Bubulcus ibis	Cattle Egret	2022/9/29	1	0	-0.4225926	36.1145254	https://www.inaturalist.org/observations/139741467
330	Bubulcus ibis	Cattle Egret	2022/9/29	1	0	-0.4225839	36.1145206	https://www.inaturalist.org/observations/139741465
331	Bubulcus ibis	Cattle Egret	2005/3/29	1	0	-0.4203793	36.9457851	https://www.inaturalist.org/observations/123247804
332	Bubulcus ibis	Cattle Egret	2010/10/21	1	0	-0.4182971	36.1254471	https://www.inaturalist.org/observations/131907459
333	Bubulcus ibis	Cattle Egret	2018/7/20	2	0	-0.4181900	36.1140181	https://www.inaturalist.org/observations/21560116
334	Bubulcus ibis	Cattle Egret	2021/7/30	1	0	-0.4131114	36.1235514	https://www.inaturalist.org/observations/97298747
335	Bubulcus ibis	Cattle Egret	2014/10/11	1	0	-0.4104552	36.0711433	https://www.inaturalist.org/observations/66313407
336	Bubulcus ibis	Cattle Egret	2014/10/11	1	0	-0.4093461	36.0689669	https://www.inaturalist.org/observations/66313140
337	Bubulcus ibis	Cattle Egret	2014/8/13	1	0	-0.4088402	36.0937589	https://www.inaturalist.org/observations/142541355
338	Bubulcus ibis	Cattle Egret	2017/2/23	2	0	-0.4043190	36.1061920	https://www.inaturalist.org/observations/34030653
339	Bubulcus ibis	Cattle Egret	2019/1/6	1	0	-0.4034278	36.0671667	https://www.inaturalist.org/observations/58076100
340	Bubulcus ibis	Cattle Egret	2021/3/5	1	0	-0.3944278	36.1202056	https://www.inaturalist.org/observations/103822157
341	Bubulcus ibis	Cattle Egret	2012/12/15	5	0	-0.3777491	36.0891346	https://www.inaturalist.org/observations/7128759
342	Bubulcus ibis	Cattle Egret	2013/11/15	1	0	-0.3693169	36.1194440	https://www.inaturalist.org/observations/142278948
343	Bubulcus ibis	Cattle Egret	2005/3/24	1	0	-0.3680538	36.0507285	https://www.inaturalist.org/observations/102126591
344	Bubulcus ibis	Cattle Egret	2019/3/4	3	0	-0.3640150	36.1210533	https://www.inaturalist.org/observations/21395981
345	Bubulcus ibis	Cattle Egret	2014/5/19	2	0	-0.3634050	36.0865920	https://www.inaturalist.org/observations/54468800
346	Bubulcus ibis	Cattle Egret	2006/7/23	2	0	-0.3561660	36.1002110	https://www.inaturalist.org/observations/31994689
347	Bubulcus ibis	Cattle Egret	2013/8/22	1	0	-0.3561660	36.1002110	https://www.inaturalist.org/observations/75422081
348	Bubulcus ibis	Cattle Egret	2021/9/21	1	0	-0.3540000	36.1180000	https://www.inaturalist.org/observations/96384339
349	Bubulcus ibis	Cattle Egret	2013/2/13	3	0	-0.3390160	36.0671500	http://www.inaturalist.org/observations/2752148

350	Bubulcus ibis	Cattle Egret	2021/7/30	2	0	-0.3101587	36.0841572	https://www.inaturalist.org/observations/93628202
351	Bubulcus ibis	Cattle Egret	2022/8/13	1	0	-0.3101587	36.0841572	https://www.inaturalist.org/observations/135747776
352	Bubulcus ibis	Cattle Egret	2021/7/20	2	0	-0.3030988	36.0800260	https://www.inaturalist.org/observations/88721931
353	Bubulcus ibis	Cattle Egret	2021/7/30	1	0	-0.3030988	36.0800260	https://www.inaturalist.org/observations/95468065
354	Bubulcus ibis	Cattle Egret	2022/9/19	1	0	-0.2957997	36.1439285	https://www.inaturalist.org/observations/135669877
355	Bubulcus ibis	Cattle Egret	2019/3/11	2	0	-0.2388244	36.9039160	https://www.inaturalist.org/observations/101277674
356	Bubulcus ibis	Cattle Egret	2016/12/4	2	0	-0.2085750	29.8902637	https://www.inaturalist.org/observations/5186155
357	Bubulcus ibis	Cattle Egret	2021/8/4	1	0	-0.2031816	29.9051175	https://www.inaturalist.org/observations/94861322
358	Bubulcus ibis	Cattle Egret	2022/10/28	1	0	-0.2026350	29.8979667	https://www.inaturalist.org/observations/143276107
359	Bubulcus ibis	Cattle Egret	2019/7/30	5	0	-0.2004390	29.9091130	https://www.inaturalist.org/observations/31420296
360	Bubulcus ibis	Cattle Egret	2018/9/24	1	0	-0.1927666	29.9090633	https://www.inaturalist.org/observations/131990139
361	Bubulcus ibis	Cattle Egret	2021/7/24	1	0	-0.1907172	29.9088445	https://www.inaturalist.org/observations/109936813
362	Bubulcus ibis	Cattle Egret	2021/12/9	1	0	-0.1857032	29.9070966	https://www.inaturalist.org/observations/106216270
363	Bubulcus ibis	Cattle Egret	2019/9/7	1	0	-0.1823701	29.9400381	https://www.inaturalist.org/observations/142326321
364	Bubulcus ibis	Cattle Egret	2015/8/8	1	0	-0.1640715	30.0202536	https://www.inaturalist.org/observations/47058498
365	Bubulcus ibis	Cattle Egret	2015/8/9	1	0	-0.1582701	34.7532980	https://www.inaturalist.org/observations/117979339
366	Bubulcus ibis	Cattle Egret	2007/12/20	2	0	-0.1572050	30.0181940	https://www.inaturalist.org/observations/68886910
367	Bubulcus ibis	Cattle Egret	2021/12/24	1	0	-0.1370930	34.7385931	https://www.inaturalist.org/observations/112658279
368	Bubulcus ibis	Cattle Egret	2022/8/15	2	0	-0.1349917	30.0251821	https://www.inaturalist.org/observations/141845052
369	Bubulcus ibis	Cattle Egret	2022/8/15	2	0	-0.1349917	30.0251821	https://www.inaturalist.org/observations/141845054
370	Bubulcus ibis	Cattle Egret	2018/5/28	2	0	-0.1349914	30.0251821	https://www.inaturalist.org/observations/66087006
371	Bubulcus ibis	Cattle Egret	2019/8/7	3	0	-0.1259649	30.0239938	https://www.inaturalist.org/observations/46785535
372	Bubulcus ibis	Cattle Egret	2011/11/14	1	0	-0.1039008	32.6528365	https://www.inaturalist.org/observations/142280138
373	Bubulcus ibis	Cattle Egret	2013/8/9	4	0	-0.0771797	30.0620323	https://www.inaturalist.org/observations/105710639
374	Bubulcus ibis	Cattle Egret	2022/3/21	1	0	-0.0628840	34.7751900	https://www.inaturalist.org/observations/109228257

375	Bubulcus ibis	Cattle Egret	2021/2/28	1	0	-0.0470683	34.8298416	https://www.inaturalist.org/observations/70555593
376	Bubulcus ibis	Cattle Egret	2021/3/1	2	0	-0.0420652	30.1001743	https://www.inaturalist.org/observations/78678734
377	Bubulcus ibis	Cattle Egret	2022/10/9	1	0	-0.0393434	30.0822312	https://www.inaturalist.org/observations/139670110
378	Bubulcus ibis	Cattle Egret	2021/7/24	1	0	-0.0384974	30.1297534	https://www.inaturalist.org/observations/96772978
379	Bubulcus ibis	Cattle Egret	2005/1/14	3	0	-0.0235590	37.9061930	https://www.inaturalist.org/observations/35670491
380	Bubulcus ibis	Cattle Egret	2018/6/23	1	0	-0.0043140	36.9637340	https://www.inaturalist.org/observations/64304172
381	Bubulcus ibis	Cattle Egret	2017/8/31	5	0	-0.0042590	36.9636710	https://www.inaturalist.org/observations/7922721
382	Bubulcus ibis	Cattle Egret	2017/2/19	3	0	-0.0042590	36.9636710	https://www.inaturalist.org/observations/12655326
383	Bubulcus ibis	Cattle Egret	2017/2/19	2	0	0.0058358	36.9636705	https://www.inaturalist.org/observations/42014614
384	Bubulcus ibis	Cattle Egret	2017/2/19	2	0	0.0061228	36.9635647	https://www.inaturalist.org/observations/42014720
385	Bubulcus ibis	Cattle Egret	2015/12/27	3	0	0.0108043	36.8950065	https://www.inaturalist.org/observations/9850130
386	Bubulcus ibis	Cattle Egret	2019/10/12	1	0	0.0311128	36.9605877	https://www.inaturalist.org/observations/107593359
387	Bubulcus ibis	Cattle Egret	2019/10/12	1	0	0.0313778	36.9602895	https://www.inaturalist.org/observations/107593358
388	Bubulcus ibis	Cattle Egret	2019/7/31	2	0	0.0317579	36.9313169	https://www.inaturalist.org/observations/33943731
389	Bubulcus ibis	Cattle Egret	2019/10/12	1	0	0.0318457	36.9600960	https://www.inaturalist.org/observations/107593355
390	Bubulcus ibis	Cattle Egret	2019/10/12	1	0	0.0326454	36.9597821	https://www.inaturalist.org/observations/107593353
391	Bubulcus ibis	Cattle Egret	2019/3/14	2	0	0.0341937	36.9392678	https://www.inaturalist.org/observations/101280918
392	Bubulcus ibis	Cattle Egret	2019/8/9	3	0	0.0392651	36.9381027	https://www.inaturalist.org/observations/32384592
393	Bubulcus ibis	Cattle Egret	2022/7/18	2	0	0.0419917	36.9675467	https://www.inaturalist.org/observations/126923790
394	Bubulcus ibis	Cattle Egret	2019/10/12	1	0	0.0437742	36.9524212	https://www.inaturalist.org/observations/107593361
395	Bubulcus ibis	Cattle Egret	2017/2/10	3	0	0.0440000	32.4700000	https://www.inaturalist.org/observations/9073450
396	Bubulcus ibis	Cattle Egret	2022/9/18	2	0	0.0452150	36.9520250	https://www.inaturalist.org/observations/138070734
397	Bubulcus ibis	Cattle Egret	2020/1/14	0	0	0.0525914	32.4775427	https://www.inaturalist.org/observations/38395132
398	Bubulcus ibis	Cattle Egret	2019/3/12	1	0	0.0528704	32.4770920	https://www.inaturalist.org/observations/37448780
399	Bubulcus ibis	Cattle Egret	2011/11/14	1	0	0.0532180	32.4720700	https://www.inaturalist.org/observations/142229855

400	Bubulcus ibis	Cattle Egret	2022/1/15	2	0	0.0532297	32.4778479	https://www.inaturalist.org/observations/110462836
401	Bubulcus ibis	Cattle Egret	2014/6/18	1	0	0.0532814	32.4747726	https://www.inaturalist.org/observations/106521332
402	Bubulcus ibis	Cattle Egret	2018/11/8	4	0	0.0533726	32.4765119	https://www.inaturalist.org/observations/18323526
403	Bubulcus ibis	Cattle Egret	2022/11/8	1	0	0.0542590	32.4757871	https://www.inaturalist.org/observations/141688426
404	Bubulcus ibis	Cattle Egret	2016/9/5	1	0	0.0585490	32.3575860	https://www.inaturalist.org/observations/90184233
405	Bubulcus ibis	Cattle Egret	2016/11/21	2	0	0.0589417	32.4813111	http://www.inaturalist.org/observations/4679481
406	Bubulcus ibis	Cattle Egret	2022/9/13	1	0	0.0611033	32.4797633	https://www.inaturalist.org/observations/134839054
407	Bubulcus ibis	Cattle Egret	2019/6/24	3	0	0.0620109	32.4792744	https://www.inaturalist.org/observations/28329947
408	Bubulcus ibis	Cattle Egret	2018/4/17	1	0	0.0620109	32.4792744	https://www.inaturalist.org/observations/121083550
409	Bubulcus ibis	Cattle Egret	2020/1/26	1	0	0.0620170	32.4792743	https://www.inaturalist.org/observations/38178559
410	Bubulcus ibis	Cattle Egret	2017/1/31	1	0	0.0621414	32.4790192	https://www.inaturalist.org/observations/107289453
411	Bubulcus ibis	Cattle Egret	2022/11/1	1	0	0.0621755	32.4805326	https://www.inaturalist.org/observations/141535730
412	Bubulcus ibis	Cattle Egret	2022/2/3	1	0	0.0624418	32.4784184	https://www.inaturalist.org/observations/106985306
413	Bubulcus ibis	Cattle Egret	2011/2/18	1	0	0.0625063	32.4791813	https://www.inaturalist.org/observations/68266737
414	Bubulcus ibis	Cattle Egret	2022/7/26	2	0	0.0626824	32.4788896	https://www.inaturalist.org/observations/143007601
415	Bubulcus ibis	Cattle Egret	2019/3/14	2	0	0.0627798	32.4784184	https://www.inaturalist.org/observations/37452109
416	Bubulcus ibis	Cattle Egret	2020/1/15	1	0	0.0628017	32.4790548	https://www.inaturalist.org/observations/38285235
417	Bubulcus ibis	Cattle Egret	2020/1/15	1	0	0.0628017	32.4790548	https://www.inaturalist.org/observations/38285262
418	Bubulcus ibis	Cattle Egret	2022/11/11	1	0	0.0630312	32.4803707	https://www.inaturalist.org/observations/143935800
419	Bubulcus ibis	Cattle Egret	2019/6/25	1	0	0.0632984	32.4789096	https://www.inaturalist.org/observations/59114476
420	Bubulcus ibis	Cattle Egret	2021/6/19	1	0	0.0632984	32.4789096	https://www.inaturalist.org/observations/109741271
421	Bubulcus ibis	Cattle Egret	2022/8/2	1	0	0.0633139	32.4782186	https://www.inaturalist.org/observations/144668555
422	Bubulcus ibis	Cattle Egret	2011/2/23	2	0	0.0634021	32.4788557	https://www.inaturalist.org/observations/68268586
423	Bubulcus ibis	Cattle Egret	2019/3/9	2	0	0.0634271	32.4791886	https://www.inaturalist.org/observations/37253813
424	Bubulcus ibis	Cattle Egret	2019/8/24	1	0	0.0634271	32.4791886	https://www.inaturalist.org/observations/37641436

425	Bubulcus ibis	Cattle Egret	2019/3/14	1	0	0.0635308	32.4798346	https://www.inaturalist.org/observations/37462260
426	Bubulcus ibis	Cattle Egret	2019/3/14	2	0	0.0637239	32.4797917	https://www.inaturalist.org/observations/37456087
427	Bubulcus ibis	Cattle Egret	2019/3/14	1	0	0.0637239	32.4797917	https://www.inaturalist.org/observations/37456095
428	Bubulcus ibis	Cattle Egret	2022/7/14	2	0	0.0637465	32.4789975	https://www.inaturalist.org/observations/127112230
429	Bubulcus ibis	Cattle Egret	2022/11/4	1	0	0.0654923	32.3571008	https://www.inaturalist.org/observations/141677545
430	Bubulcus ibis	Cattle Egret	2021/6/20	1	0	0.0658396	32.3558765	https://www.inaturalist.org/observations/109909199
431	Bubulcus ibis	Cattle Egret	2018/9/19	1	0	0.0659349	32.3575884	https://www.inaturalist.org/observations/131883409
432	Bubulcus ibis	Cattle Egret	2019/2/10	4	0	0.0669650	32.4781483	https://www.inaturalist.org/observations/20265191
433	Bubulcus ibis	Cattle Egret	2019/7/27	4	0	0.0670470	32.3500160	https://www.inaturalist.org/observations/31020756
434	Bubulcus ibis	Cattle Egret	2022/11/1	1	0	0.0672315	32.4788928	https://www.inaturalist.org/observations/141529601
435	Bubulcus ibis	Cattle Egret	2013/3/18	2	0	0.0679400	32.3491100	https://www.inaturalist.org/observations/47500973
436	Bubulcus ibis	Cattle Egret	2016/2/28	1	0	0.0755880	32.3500500	https://www.inaturalist.org/observations/34627136
437	Bubulcus ibis	Cattle Egret	2021/7/22	1	0	0.0778770	36.0791095	https://www.inaturalist.org/observations/90636654
438	Bubulcus ibis	Cattle Egret	2019/7/25	4	0	0.0829230	32.4507720	https://www.inaturalist.org/observations/30808049
439	Bubulcus ibis	Cattle Egret	2013/3/17	1	0	0.0833333	32.3333333	https://www.inaturalist.org/observations/123209243
440	Bubulcus ibis	Cattle Egret	2013/3/17	1	0	0.0833333	32.3333333	https://www.inaturalist.org/observations/123210188
441	Bubulcus ibis	Cattle Egret	2021/1/24	2	0	0.0882865	32.9543354	https://www.inaturalist.org/observations/69570171
442	Bubulcus ibis	Cattle Egret	2013/8/29	1	0	0.0896280	38.1900290	https://www.inaturalist.org/observations/65570290
443	Bubulcus ibis	Cattle Egret	2021/10/7	1	0	0.0901535	32.3577133	https://www.inaturalist.org/observations/97787664
444	Bubulcus ibis	Cattle Egret	2022/7/27	1	0	0.1275443	32.4525833	https://www.inaturalist.org/observations/138497265
445	Bubulcus ibis	Cattle Egret	2016/2/4	3	0	0.1358550	32.4825680	http://www.inaturalist.org/observations/2833318
446	Bubulcus ibis	Cattle Egret	2022/3/13	1	0	0.1446237	32.7508529	https://www.inaturalist.org/observations/108502727
447	Bubulcus ibis	Cattle Egret	2011/8/10	2	0	0.1564263	30.0788069	https://www.inaturalist.org/observations/41133924
448	Bubulcus ibis	Cattle Egret	2022/8/14	2	0	0.1564263	30.0788069	https://www.inaturalist.org/observations/141927764
449	Bubulcus ibis	Cattle Egret	2021/6/21	2	0	0.1599883	32.5591433	https://www.inaturalist.org/observations/87364875

450	Bubulcus ibis	Cattle Egret	2021/6/12	1	0	0.1604417	32.5593800	https://www.inaturalist.org/observations/84298709
451	Bubulcus ibis	Cattle Egret	2017/5/15	2	0	0.1847213	38.1932071	https://www.inaturalist.org/observations/6820533
452	Bubulcus ibis	Cattle Egret	2018/12/9	4	0	0.1883006	37.4496832	https://www.inaturalist.org/observations/18915259
453	Bubulcus ibis	Cattle Egret	2021/10/19	1	0	0.1913988	37.4576745	https://www.inaturalist.org/observations/101689594
454	Bubulcus ibis	Cattle Egret	2018/8/21	1	0	0.1938264	38.1904831	https://www.inaturalist.org/observations/66511938
455	Bubulcus ibis	Cattle Egret	2022/7/2	1	0	0.2170215	37.4303548	https://www.inaturalist.org/observations/133501308
456	Bubulcus ibis	Cattle Egret	2020/11/17	1	0	0.2193777	32.5507161	https://www.inaturalist.org/observations/65332694
457	Bubulcus ibis	Cattle Egret	2021/11/23	1	0	0.2235740	36.9476110	https://www.inaturalist.org/observations/103363884
458	Bubulcus ibis	Cattle Egret	2019/6/11	1	0	0.2567966	36.8475634	https://www.inaturalist.org/observations/41164118
459	Bubulcus ibis	Cattle Egret	2022/4/29	1	0	0.2591030	36.9377731	https://www.inaturalist.org/observations/116802851
460	Bubulcus ibis	Cattle Egret	2021/6/21	2	0	0.2850783	32.5630867	https://www.inaturalist.org/observations/83904476
461	Bubulcus ibis	Cattle Egret	2022/2/3	2	0	0.2923660	36.8984604	https://www.inaturalist.org/observations/106369295
462	Bubulcus ibis	Cattle Egret	2019/4/4	3	0	0.2924420	36.8986050	https://www.inaturalist.org/observations/26409936
463	Bubulcus ibis	Cattle Egret	2014/1/5	2	0	0.3324188	32.3780443	https://www.inaturalist.org/observations/122624747
464	Bubulcus ibis	Cattle Egret	2019/5/23	2	0	0.3427970	36.8723244	https://www.inaturalist.org/observations/108489240
465	Bubulcus ibis	Cattle Egret	2013/8/4	1	0	0.3475964	32.5825197	https://www.inaturalist.org/observations/69492792
466	Bubulcus ibis	Cattle Egret	2014/12/19	3	0	0.3536190	32.6032390	http://www.inaturalist.org/observations/1499937
467	Bubulcus ibis	Cattle Egret	2019/8/13	3	0	0.3606063	36.7819505	https://www.inaturalist.org/observations/30919524
468	Bubulcus ibis	Cattle Egret	2020/1/22	1	0	0.3800967	32.5710917	https://www.inaturalist.org/observations/37841655
469	Bubulcus ibis	Cattle Egret	2011/3/2	1	0	0.4053550	33.1984361	https://www.inaturalist.org/observations/38693467
470	Bubulcus ibis	Cattle Egret	2022/4/8	1	0	0.4478563	33.2026121	https://www.inaturalist.org/observations/111957374
471	Bubulcus ibis	Cattle Egret	2009/3/10	3	0	0.4908943	36.2809303	http://www.inaturalist.org/observations/4632166
472	Bubulcus ibis	Cattle Egret	2007/8/3	2	0	0.4956443	33.3059840	https://www.inaturalist.org/observations/142863957
473	Bubulcus ibis	Cattle Egret	2019/6/9	3	0	0.5328783	33.8854267	https://www.inaturalist.org/observations/28353978
474	Bubulcus ibis	Cattle Egret	2020/1/13	1	0	0.5435383	33.0894083	https://www.inaturalist.org/observations/143520782

475	Bubulcus ibis	Cattle Egret	2019/6/14	1	0	0.5451353	37.5883645	https://www.inaturalist.org/observations/53348897
476	Bubulcus ibis	Cattle Egret	2017/12/31	3	0	0.5534717	35.3137817	https://www.inaturalist.org/observations/9563247
477	Bubulcus ibis	Cattle Egret	2019/6/15	2	0	0.5566629	37.6059708	https://www.inaturalist.org/observations/53824848
478	Bubulcus ibis	Cattle Egret	2014/1/2	6	0	0.5700383	35.3140167	https://www.inaturalist.org/observations/9023440
479	Bubulcus ibis	Cattle Egret	2010/9/9	1	0	0.5718048	36.0727166	https://www.inaturalist.org/observations/46178234
480	Bubulcus ibis	Cattle Egret	2019/3/9	2	0	0.5774645	37.4915773	https://www.inaturalist.org/observations/42293935
481	Bubulcus ibis	Cattle Egret	2020/7/17	1	0	0.5774698	37.4915770	https://www.inaturalist.org/observations/73962849
482	Bubulcus ibis	Cattle Egret	2019/8/4	6	0	0.5899190	37.5897930	https://www.inaturalist.org/observations/31866599
483	Bubulcus ibis	Cattle Egret	2010/9/9	2	0	0.5900000	36.0710000	https://www.inaturalist.org/observations/46052233
484	Bubulcus ibis	Cattle Egret	2021/8/9	2	0	0.6136856	37.6217401	https://www.inaturalist.org/observations/94037731
485	Bubulcus ibis	Cattle Egret	2010/9/9	2	0	0.6140000	36.0230000	https://www.inaturalist.org/observations/46329589
486	Bubulcus ibis	Cattle Egret	2008/9/26	3	0	0.6304548	36.0723283	https://www.inaturalist.org/observations/38093075
487	Bubulcus ibis	Cattle Egret	2007/3/3	1	0	0.6446745	36.0557215	https://www.inaturalist.org/observations/103095194
488	Bubulcus ibis	Cattle Egret	2022/11/14	1	0	0.8376569	30.1661420	https://www.inaturalist.org/observations/142201412
489	Bubulcus ibis	Cattle Egret	2017/12/27	3	0	0.8442617	35.2623183	https://www.inaturalist.org/observations/9764390
490	Bubulcus ibis	Cattle Egret	2014/6/5	3	0	1.2351800	30.7383730	http://www.inaturalist.org/observations/1232292
491	Bubulcus ibis	Cattle Egret	2016/2/17	1	0	1.4080530	32.1223620	https://www.inaturalist.org/observations/34527878
492	Bubulcus ibis	Cattle Egret	2022/10/13	1	0	1.5798625	31.1083441	https://www.inaturalist.org/observations/138640092
493	Bubulcus ibis	Cattle Egret	2022/9/29	2	0	1.6279243	32.0320373	https://www.inaturalist.org/observations/139257213
494	Bubulcus ibis	Cattle Egret	2009/6/17	4	0	1.9641080	33.0654144	https://www.inaturalist.org/observations/8679311
495	Bubulcus ibis	Cattle Egret	2019/2/7	1	0	2.1473243	31.8054199	https://www.inaturalist.org/observations/34929866
496	Bubulcus ibis	Cattle Egret	2016/2/5	2	0	2.1746008	31.3680305	https://www.inaturalist.org/observations/48498422
497	Bubulcus ibis	Cattle Egret	2011/1/22	2	0	2.2153333	31.8286667	https://www.inaturalist.org/observations/131547240
498	Bubulcus ibis	Cattle Egret	2018/8/13	1	0	2.2202780	31.4162061	https://www.inaturalist.org/observations/144237332
499	Bubulcus ibis	Cattle Egret	2010/11/24	1	0	2.2275088	31.4397285	https://www.inaturalist.org/observations/109417648

500	Bubulcus ibis	Cattle Egret	2017/6/10	4	0	2.2433497	31.4066488	https://www.inaturalist.org/observations/18599522
501	Bubulcus ibis	Cattle Egret	2014/12/26	2	0	2.2487981	31.4902262	https://www.inaturalist.org/observations/33545027
502	Bubulcus ibis	Cattle Egret	2005/8/13	1	0	2.2574146	31.6538776	https://www.inaturalist.org/observations/67457336
503	Bubulcus ibis	Cattle Egret	2019/9/23	1	0	2.2672525	31.6567405	https://www.inaturalist.org/observations/118147539
504	Bubulcus ibis	Cattle Egret	2017/12/23	1	0	2.2698179	31.3742086	https://www.inaturalist.org/observations/38135062
505	Bubulcus ibis	Cattle Egret	2013/8/2	4	0	2.2749452	31.5719605	https://www.inaturalist.org/observations/9765939
506	Bubulcus ibis	Cattle Egret	2012/9/26	1	0	2.2780283	31.3837900	https://www.inaturalist.org/observations/39838513
507	Bubulcus ibis	Cattle Egret	2018/7/23	2	0	2.2839481	31.5901681	https://www.inaturalist.org/observations/21560119
508	Bubulcus ibis	Cattle Egret	2020/3/21	2	0	2.2848899	31.5681891	https://www.inaturalist.org/observations/40767907
509	Bubulcus ibis	Cattle Egret	2019/6/14	3	0	2.2882450	31.4792629	https://www.inaturalist.org/observations/28325470
510	Bubulcus ibis	Cattle Egret	2019/3/16	1	0	2.2970912	31.4830860	https://www.inaturalist.org/observations/37520767
511	Bubulcus ibis	Cattle Egret	2021/6/3	2	0	2.2977633	31.4678700	https://www.inaturalist.org/observations/83114780
512	Bubulcus ibis	Cattle Egret	2019/6/23	1	0	2.3021875	31.4965960	https://www.inaturalist.org/observations/58820681
513	Bubulcus ibis	Cattle Egret	2019/8/27	0	0	2.3032015	31.4959486	https://www.inaturalist.org/observations/37772372
514	Bubulcus ibis	Cattle Egret	2021/6/14	1	0	2.3083800	31.4136183	https://www.inaturalist.org/observations/84816619
515	Bubulcus ibis	Cattle Egret	2021/6/14	1	0	2.3129017	31.4236233	https://www.inaturalist.org/observations/84834405
516	Bubulcus ibis	Cattle Egret	2021/6/13	1	0	2.3616833	31.4859967	https://www.inaturalist.org/observations/84522309
517	Bubulcus ibis	Cattle Egret	2021/6/13	1	0	2.3633550	31.4862067	https://www.inaturalist.org/observations/84522304
518	Bubulcus ibis	Cattle Egret	2020/3/21	1	0	2.3908133	31.6995039	https://www.inaturalist.org/observations/40363595
519	Bubulcus ibis	Cattle Egret	2018/4/25	2	0	2.3921095	32.1931399	https://www.inaturalist.org/observations/13685593
520	Bubulcus ibis	Cattle Egret	2022/10/25	1	0	2.4074950	31.5127717	https://www.inaturalist.org/observations/142468393
521	Bubulcus ibis	Cattle Egret	2018/9/16	1	0	3.7253055	33.7103398	https://www.inaturalist.org/observations/131782475
522	Bubulcus ibis	Cattle Egret	2017/2/3	2	0	3.7527171	33.7041264	https://www.inaturalist.org/observations/68337126
523	Bubulcus ibis	Cattle Egret	2021/6/18	1	0	3.7756400	33.6957567	https://www.inaturalist.org/observations/86264887
524	Bubulcus ibis	Cattle Egret	2022/4/30	1	0	6.0582872	37.6029851	https://www.inaturalist.org/observations/113869098

525	Bubulcus ibis	Cattle Egret	2015/10/20	3	0	7.0008472	38.5742417	https://www.inaturalist.org/observations/68220741
526	Bubulcus ibis	Cattle Egret	2012/8/15	3	0	7.0231230	38.4663100	http://www.inaturalist.org/observations/1710402
527	Bubulcus ibis	Cattle Egret	2014/1/12	1	0	7.0312974	38.4218849	https://www.inaturalist.org/observations/129326393
528	Bubulcus ibis	Cattle Egret	2014/1/12	1	0	7.0312974	38.4218849	https://www.inaturalist.org/observations/129457079
529	Bubulcus ibis	Cattle Egret	2014/1/28	2	0	7.0332720	38.4617550	https://www.inaturalist.org/observations/37371715
530	Bubulcus ibis	Cattle Egret	2022/6/2	1	0	7.9170451	38.7309685	https://www.inaturalist.org/observations/119845318
531	Bubulcus ibis	Cattle Egret	2014/1/12	2	0	8.4673473	39.1558159	https://www.inaturalist.org/observations/129326061
532	Bubulcus ibis	Cattle Egret	2015/1/11	1	0	8.7866855	38.9941937	https://www.inaturalist.org/observations/49924595
533	Bubulcus ibis	Cattle Egret	2021/11/2	2	0	8.8031473	38.7127037	https://www.inaturalist.org/observations/100161527
534	Bubulcus ibis	Cattle Egret	2022/9/11	1	0	8.8765678	38.8191185	https://www.inaturalist.org/observations/134567004
535	Bubulcus ibis	Cattle Egret	2014/1/23	1	0	9.0833333	40.0000000	https://www.inaturalist.org/observations/129457151
536	Bubulcus ibis	Cattle Egret	2016/4/23	4	0	9.1059892	40.0207901	https://www.inaturalist.org/observations/5238488
537	Bubulcus ibis	Cattle Egret	2012/12/20	1	0	9.1852784	38.7603778	https://www.inaturalist.org/observations/54415420
538	Bubulcus ibis	Cattle Egret	2014/2/22	1	0	9.2536585	34.8682286	https://www.inaturalist.org/observations/143529471
539	Bubulcus ibis	Cattle Egret	2012/12/20	3	0	9.3258450	38.7690350	https://www.inaturalist.org/observations/18677366
540	Bubulcus ibis	Cattle Egret	2015/7/22	4	0	9.3604944	42.8000972	http://www.inaturalist.org/observations/2130018
541	Bubulcus ibis	Cattle Egret	2011/7/6	2	0	9.6008180	43.3323530	http://www.inaturalist.org/observations/502550
542	Bubulcus ibis	Cattle Egret	2020/2/17	0	0	11.4901771	37.5833883	https://www.inaturalist.org/observations/39378397
543	Bubulcus ibis	Cattle Egret	2020/2/17	1	0	11.4901771	37.5833883	https://www.inaturalist.org/observations/39378418
544	Bubulcus ibis	Cattle Egret	2014/12/3	1	0	11.4905079	37.5803714	https://www.inaturalist.org/observations/46423513
545	Bubulcus ibis	Cattle Egret	2014/4/16	1	0	11.5742086	37.3613533	https://www.inaturalist.org/observations/58447447
546	Bubulcus ibis	Cattle Egret	2021/10/20	1	0	11.5742086	37.3613533	https://www.inaturalist.org/observations/103139432
547	Bubulcus ibis	Cattle Egret	2021/6/5	1	0	11.5742086	37.3613533	https://www.inaturalist.org/observations/104176200
548	Bubulcus ibis	Cattle Egret	2022/1/11	1	0	11.5742086	37.3613533	https://www.inaturalist.org/observations/104754178
549	Bubulcus ibis	Cattle Egret	2022/3/31	1	0	11.5742086	37.3613533	https://www.inaturalist.org/observations/109999521

550	Bubulcus ibis	Cattle Egret	2022/11/17	1	0	11.5742086	37.3613533	https://www.inaturalist.org/observations/142209113
551	Bubulcus ibis	Cattle Egret	2022/12/6	1	0	11.5742086	37.3613533	https://www.inaturalist.org/observations/143786736
552	Bubulcus ibis	Cattle Egret	2011/1/23	4	0	11.6013910	37.3912810	https://www.inaturalist.org/observations/9777343
553	Bubulcus ibis	Cattle Egret	2022/12/2	1	0	11.6104633	37.3780233	https://www.inaturalist.org/observations/143513324
554	Bubulcus ibis	Cattle Egret	2020/2/18	0	0	11.9642963	37.7034069	https://www.inaturalist.org/observations/39682860
555	Bubulcus ibis	Cattle Egret	2015/1/17	1	0	12.0265982	37.3034845	https://www.inaturalist.org/observations/50116472
556	Bubulcus ibis	Cattle Egret	2015/3/14	2	0	5.9424583	80.4521900	https://www.inaturalist.org/observations/18953623
557	Bubulcus ibis	Cattle Egret	2015/3/20	3	0	5.9424583	80.4521900	https://www.inaturalist.org/observations/18953658
558	Bubulcus ibis	Cattle Egret	2021/1/15	6	0	5.9548881	80.5375545	https://www.inaturalist.org/observations/68950646
559	Bubulcus ibis	Cattle Egret	2019/4/15	2	0	5.9833890	80.4669410	https://www.inaturalist.org/observations/22700108
560	Bubulcus ibis	Cattle Egret	2019/4/10	5	0	5.9845010	80.4666670	https://www.inaturalist.org/observations/22308938
561	Bubulcus ibis	Cattle Egret	2022/2/10	1	0	6.0007040	80.3352364	https://www.inaturalist.org/observations/109653762
562	Bubulcus ibis	Cattle Egret	2018/12/28	3	0	6.0107890	80.2437956	https://www.inaturalist.org/observations/19610102
563	Bubulcus ibis	Cattle Egret	2019/4/19	8	0	6.0249689	80.2162479	https://www.inaturalist.org/observations/40347652
564	Bubulcus ibis	Cattle Egret	2019/1/12	2	0	6.0271971	80.2164717	https://www.inaturalist.org/observations/144267471
565	Bubulcus ibis	Cattle Egret	2016/4/10	3	0	6.0279764	80.2174389	https://www.inaturalist.org/observations/127458399
566	Bubulcus ibis	Cattle Egret	2019/2/2	5	0	6.0294521	80.2138258	https://www.inaturalist.org/observations/20455573
567	Bubulcus ibis	Cattle Egret	2017/2/8	5	0	6.0300426	80.2156501	https://www.inaturalist.org/observations/5073991
568	Bubulcus ibis	Cattle Egret	2017/11/18	4	0	6.0301157	80.2168063	https://www.inaturalist.org/observations/22724414
569	Bubulcus ibis	Cattle Egret	2019/2/5	6	0	6.0303917	80.2166133	https://www.inaturalist.org/observations/20145420
570	Bubulcus ibis coromandus	Cattle Egret	2018/12/10	4	0	6.0304700	80.2161367	https://www.inaturalist.org/observations/19269795
571	Bubulcus ibis	Cattle Egret	2019/12/20	2	0	6.0322291	80.2152387	https://www.inaturalist.org/observations/38385507
572	Bubulcus ibis	Cattle Egret	2018/2/25	1	0	6.0328928	80.2167912	https://www.inaturalist.org/observations/136784136
573	Bubulcus ibis	Cattle Egret	2019/4/17	4	0	6.0340830	80.2142530	https://www.inaturalist.org/observations/22791468
574	Bubulcus ibis	Cattle Egret	2012/11/10	5	0	6.0369601	80.2161710	https://www.inaturalist.org/observations/41232138

575	Bubulcus ibis	Cattle Egret	2015/11/14	5	0	6.0451542	80.2213207	https://www.inaturalist.org/observations/63752283
576	Bubulcus ibis	Cattle Egret	2019/3/7	3	0	6.0480774	80.8566247	https://www.inaturalist.org/observations/22136110
577	Bubulcus ibis	Cattle Egret	2019/3/8	4	0	6.0511938	80.8587395	https://www.inaturalist.org/observations/22157712
578	Bubulcus ibis	Cattle Egret	2020/3/4	4	0	6.0535185	80.2209773	https://www.inaturalist.org/observations/39914160
579	Bubulcus ibis	Cattle Egret	2019/12/5	3	0	6.0631314	80.8732118	https://www.inaturalist.org/observations/104809845
580	Bubulcus ibis	Cattle Egret	2019/1/6	4	0	6.0882170	80.9397881	https://www.inaturalist.org/observations/28193208
581	Bubulcus ibis	Cattle Egret	2019/3/11	6	0	6.1214388	80.1133500	https://www.inaturalist.org/observations/21137973
582	Bubulcus ibis	Cattle Egret	2019/2/5	6	0	6.1219887	80.1125174	https://www.inaturalist.org/observations/20107449
583	Bubulcus ibis coromandus	Cattle Egret	2019/3/11	2	0	6.1240805	80.1148445	https://www.inaturalist.org/observations/21356511
584	Bubulcus ibis coromandus	Cattle Egret	2019/3/11	2	0	6.1240805	80.1148445	https://www.inaturalist.org/observations/57900761
585	Bubulcus ibis	Cattle Egret	2014/2/15	5	0	6.1394676	80.1062861	https://www.inaturalist.org/observations/39273170
586	Bubulcus ibis	Cattle Egret	2019/4/24	5	0	6.1394676	80.1062861	https://www.inaturalist.org/observations/49860678
587	Bubulcus ibis	Cattle Egret	2019/11/28	2	0	6.1756815	81.2163652	https://www.inaturalist.org/observations/37056691
588	Bubulcus ibis	Cattle Egret	2016/4/5	4	0	6.2008703	81.1984936	https://www.inaturalist.org/observations/127458353
589	Bubulcus ibis	Cattle Egret	2019/2/27	1	0	6.2028608	81.2457846	https://www.inaturalist.org/observations/124603677
590	Bubulcus ibis	Cattle Egret	2019/12/24	3	0	6.2640171	81.4112857	https://www.inaturalist.org/observations/36961034
591	Bubulcus ibis	Cattle Egret	2007/7/25	2	0	6.2899000	81.2919000	https://www.inaturalist.org/observations/53812591
592	Bubulcus ibis	Cattle Egret	2017/11/20	2	0	6.2913046	81.2759127	https://www.inaturalist.org/observations/22724921
593	Bubulcus ibis	Cattle Egret	2012/12/31	3	0	6.2913473	81.2863399	https://www.inaturalist.org/observations/30527165
594	Bubulcus ibis	Cattle Egret	2011/1/21	5	0	6.2930225	81.2667776	https://www.inaturalist.org/observations/57910369
595	Bubulcus ibis	Cattle Egret	2020/12/20	6	0	6.2957783	81.3127217	https://www.inaturalist.org/observations/66822123
596	Bubulcus ibis	Cattle Egret	2011/1/23	5	0	6.2960254	81.2706266	https://www.inaturalist.org/observations/57913380
597	Bubulcus ibis	Cattle Egret	2017/12/30	3	0	6.2970698	81.2775411	https://www.inaturalist.org/observations/14166397
598	Bubulcus ibis	Cattle Egret	2017/12/30	3	0	6.2970698	81.2775411	https://www.inaturalist.org/observations/14166398
599	Bubulcus ibis coromandus	Cattle Egret	2018/12/11	4	0	6.2976013	81.2711784	https://www.inaturalist.org/observations/19374210

600	Bubulcus ibis	Cattle Egret	2014/4/20	2	0	6.3113031	80.0322209	https://www.inaturalist.org/observations/19173320
601	Bubulcus ibis	Cattle Egret	2014/4/20	3	0	6.3119114	80.0312557	https://www.inaturalist.org/observations/19172589
602	Bubulcus ibis	Cattle Egret	2019/2/4	3	0	6.3228088	81.4705947	https://www.inaturalist.org/observations/20668082
603	Bubulcus ibis	Cattle Egret	2011/1/21	4	0	6.3232935	81.4778641	https://www.inaturalist.org/observations/57910785
604	Bubulcus ibis	Cattle Egret	2018/12/25	4	0	6.3562145	81.3726262	https://www.inaturalist.org/observations/19486763
605	Bubulcus ibis	Cattle Egret	2016/2/8	3	0	6.3897222	80.5013889	https://www.inaturalist.org/observations/35666786
606	Bubulcus ibis	Cattle Egret	2017/12/29	3	0	6.3966688	81.5206430	https://www.inaturalist.org/observations/14143330
607	Bubulcus ibis	Cattle Egret	2019/11/30	2	0	6.4115617	79.9989224	https://www.inaturalist.org/observations/104804201
608	Bubulcus ibis	Cattle Egret	2019/2/27	5	0	6.4174199	79.9964708	https://www.inaturalist.org/observations/20803017
609	Bubulcus ibis	Cattle Egret	2013/3/31	2	0	6.4256777	81.4199904	https://www.inaturalist.org/observations/105664591
610	Bubulcus ibis	Cattle Egret	2015/4/8	5	0	6.4299250	79.9994659	https://www.inaturalist.org/observations/20761617
611	Bubulcus ibis	Cattle Egret	2018/3/6	3	0	6.4456050	80.3010867	https://www.inaturalist.org/observations/10180279
612	Bubulcus ibis	Cattle Egret	2015/2/3	6	0	6.4632511	80.8750411	https://www.inaturalist.org/observations/65617516
613	Bubulcus ibis	Cattle Egret	2017/7/28	4	0	6.4815806	80.9321785	https://www.inaturalist.org/observations/30926966
614	Bubulcus ibis	Cattle Egret	2018/3/7	3	0	6.5177850	80.2236133	https://www.inaturalist.org/observations/10183057
615	Bubulcus ibis	Cattle Egret	2014/4/18	3	0	6.5199513	81.5657259	https://www.inaturalist.org/observations/19169793
616	Bubulcus ibis	Cattle Egret	2012/3/8	6	0	6.5392974	81.1789904	https://www.inaturalist.org/observations/64607956
617	Bubulcus ibis coromandus	Cattle Egret	2022/3/17	2	0	6.5853422	81.5614371	https://www.inaturalist.org/observations/108939092
618	Bubulcus ibis	Cattle Egret	2018/7/19	2	0	6.5853950	79.9607400	https://www.inaturalist.org/observations/15082357
619	Bubulcus ibis	Cattle Egret	2020/5/18	7	0	6.5857726	80.0374291	https://www.inaturalist.org/observations/46444602
620	Bubulcus ibis	Cattle Egret	2020/5/18	3	0	6.5857730	80.0374290	https://www.inaturalist.org/observations/46449470
621	Bubulcus ibis	Cattle Egret	2020/5/18	8	0	6.6071490	80.0079830	https://www.inaturalist.org/observations/46444411
622	Bubulcus ibis	Cattle Egret	2019/4/19	2	0	6.6266220	79.9438250	https://www.inaturalist.org/observations/22718614
623	Bubulcus ibis	Cattle Egret	2019/11/16	4	0	6.6273639	79.9409994	https://www.inaturalist.org/observations/101032394
624	Bubulcus ibis	Cattle Egret	2018/3/27	4	0	6.6782883	79.9197617	https://www.inaturalist.org/observations/18051747

625	Bubulcus ibis	Cattle Egret	2021/2/23	4	0	6.6804028	80.6959706	https://www.inaturalist.org/observations/70027729
626	Bubulcus ibis	Cattle Egret	2021/3/19	6	0	6.6835133	79.9187550	https://www.inaturalist.org/observations/71548927
627	Bubulcus ibis	Cattle Egret	2019/3/9	1	0	6.7503867	81.0996333	https://www.inaturalist.org/observations/21314351
628	Bubulcus ibis	Cattle Egret	2018/3/5	3	0	6.7861111	79.9669444	https://www.inaturalist.org/observations/10168585
629	Bubulcus ibis	Cattle Egret	2020/12/13	6	0	6.7937011	80.0933881	https://www.inaturalist.org/observations/67578844
630	Bubulcus ibis	Cattle Egret	2020/11/29	7	0	6.8017575	79.9227313	https://www.inaturalist.org/observations/66011274
631	Bubulcus ibis	Cattle Egret	2020/4/22	5	0	6.8289837	79.9809163	https://www.inaturalist.org/observations/42831797
632	Bubulcus ibis coromandus	Cattle Egret	2021/1/20	3	0	6.8332823	80.0898356	https://www.inaturalist.org/observations/71310401
633	Bubulcus ibis	Cattle Egret	2018/3/7	3	0	6.8443883	79.8912333	https://www.inaturalist.org/observations/10183070
634	Bubulcus ibis coromandus	Cattle Egret	2021/9/5	0	0	6.8445148	81.8229904	https://www.inaturalist.org/observations/93746294
635	Bubulcus ibis	Cattle Egret	2022/1/12	6	0	6.8450558	80.1034618	https://www.inaturalist.org/observations/104861719
636	Bubulcus ibis	Cattle Egret	2021/4/9	6	0	6.8453630	80.1037719	https://www.inaturalist.org/observations/79184644
637	Bubulcus ibis	Cattle Egret	2022/1/12	4	0	6.8453634	80.1037721	https://www.inaturalist.org/observations/104860993
638	Bubulcus ibis	Cattle Egret	2022/1/12	5	0	6.8459766	80.1040058	https://www.inaturalist.org/observations/104900255
639	Bubulcus ibis	Cattle Egret	2019/3/17	3	0	6.8592833	80.0311750	https://www.inaturalist.org/observations/21369679
640	Bubulcus ibis	Cattle Egret	2020/11/21	4	0	6.8758250	79.9300167	https://www.inaturalist.org/observations/65312935
641	Bubulcus ibis	Cattle Egret	2014/6/8	5	0	6.8921354	81.6942070	https://www.inaturalist.org/observations/69694375
642	Bubulcus ibis coromandus	Cattle Egret	2021/8/8	2	0	6.8923336	79.8959607	https://www.inaturalist.org/observations/94904703
643	Bubulcus ibis	Cattle Egret	2022/1/23	4	0	6.8994181	79.9785787	https://www.inaturalist.org/observations/105414511
644	Bubulcus ibis coromandus	Cattle Egret	2021/10/29	0	0	6.9016953	79.9110489	https://www.inaturalist.org/observations/99681272
645	Bubulcus ibis	Cattle Egret	2019/3/11	5	0	6.9032300	79.8681867	https://www.inaturalist.org/observations/21412646
646	Bubulcus ibis	Cattle Egret	2020/11/13	4	0	6.9068500	79.8641050	https://www.inaturalist.org/observations/64923302
647	Bubulcus ibis	Cattle Egret	2022/3/7	1	0	6.9085306	79.8773066	https://www.inaturalist.org/observations/108108147
648	Bubulcus ibis coromandus	Cattle Egret	2020/2/25	2	0	6.9127314	79.8613415	https://www.inaturalist.org/observations/39202579
649	Bubulcus ibis	Cattle Egret	2020/5/11	4	0	6.9133904	79.8617220	https://www.inaturalist.org/observations/45556865

650	Bubulcus ibis	Cattle Egret	2020/5/11	4	0	6.9133907	79.8617401	https://www.inaturalist.org/observations/45556863
651	Bubulcus ibis	Cattle Egret	2022/1/20	2	0	6.9148061	79.8630057	https://www.inaturalist.org/observations/105367505
652	Bubulcus ibis	Cattle Egret	2019/4/18	4	0	6.9165648	79.8534564	https://www.inaturalist.org/observations/33365449
653	Bubulcus ibis	Cattle Egret	2019/10/11	2	0	6.9173950	79.8546450	https://www.inaturalist.org/observations/34202820
654	Bubulcus ibis coromandus	Cattle Egret	2019/3/27	5	0	6.9176301	79.8528950	https://www.inaturalist.org/observations/61814692
655	Bubulcus ibis	Cattle Egret	2022/4/6	4	0	6.9183083	79.8502283	https://www.inaturalist.org/observations/110580363
656	Bubulcus ibis	Cattle Egret	2018/10/13	1	0	6.9270786	79.8612430	https://www.inaturalist.org/observations/17706761
657	Bubulcus ibis	Cattle Egret	2015/11/4	7	0	6.9270786	79.8612430	https://www.inaturalist.org/observations/63246589
658	Bubulcus ibis	Cattle Egret	2019/2/8	4	0	6.9679697	80.7693960	https://www.inaturalist.org/observations/20678227
659	Bubulcus ibis	Cattle Egret	2020/3/11	3	0	6.9682790	80.7678789	https://www.inaturalist.org/observations/142861719
660	Bubulcus ibis	Cattle Egret	2020/2/13	4	0	6.9690590	80.7682014	https://www.inaturalist.org/observations/39425621
661	Bubulcus ibis	Cattle Egret	2019/3/8	5	0	6.9697950	80.7686083	https://www.inaturalist.org/observations/21057797
662	Bubulcus ibis coromandus	Cattle Egret	2015/1/22	2	0	6.9712290	80.7648570	https://www.inaturalist.org/observations/35182761
663	Bubulcus ibis coromandus	Cattle Egret	2015/1/16	3	0	6.9865470	80.4240420	https://www.inaturalist.org/observations/35084697
664	Bubulcus ibis coromandus	Cattle Egret	2009/1/13	3	0	6.9870583	80.4277752	https://www.inaturalist.org/observations/6985867
665	Bubulcus ibis	Cattle Egret	2022/12/11	1	0	7.0249000	79.8619300	https://www.inaturalist.org/observations/144230142
666	Bubulcus ibis	Cattle Egret	2022/10/9	3	0	7.0667984	79.9040932	https://www.inaturalist.org/observations/139603663
667	Bubulcus ibis	Cattle Egret	2022/12/21	1	0	7.0667984	79.9040932	https://www.inaturalist.org/observations/144782343
668	Bubulcus ibis	Cattle Egret	2019/1/14	4	0	7.0699687	80.1128780	https://www.inaturalist.org/observations/32221545
669	Bubulcus ibis	Cattle Egret	2019/4/6	4	0	7.0772050	80.0434400	https://www.inaturalist.org/observations/22138412
670	Bubulcus ibis	Cattle Egret	2022/11/15	5	0	7.0890800	79.8966767	https://www.inaturalist.org/observations/142072393
671	Bubulcus ibis	Cattle Egret	2022/4/9	3	0	7.1740417	79.8873367	https://www.inaturalist.org/observations/110818395
672	Bubulcus ibis	Cattle Egret	2017/3/31	3	0	7.1747194	79.8887472	https://www.inaturalist.org/observations/136132542
673	Bubulcus ibis	Cattle Egret	2020/4/21	4	0	7.1804990	80.3123066	https://www.inaturalist.org/observations/42770049
674	Bubulcus ibis	Cattle Egret	2020/2/12	3	0	7.1848633	79.8961417	https://www.inaturalist.org/observations/38790170

675	Bubulcus ibis	Cattle Egret	2019/12/27	2	0	7.2234583	79.8390500	https://www.inaturalist.org/observations/37098002
676	Bubulcus ibis	Cattle Egret	2017/1/19	3	0	7.2267700	79.8394017	https://www.inaturalist.org/observations/110310346
677	Bubulcus ibis	Cattle Egret	2019/2/26	4	0	7.2392837	79.8499857	https://www.inaturalist.org/observations/21828073
678	Bubulcus ibis	Cattle Egret	2019/4/21	5	0	7.2475971	80.5971671	https://www.inaturalist.org/observations/22989137
679	Bubulcus ibis	Cattle Egret	2019/4/14	4	0	7.2486100	80.2344430	https://www.inaturalist.org/observations/22484623
680	Bubulcus ibis	Cattle Egret	2019/4/10	4	0	7.2568096	80.5958540	https://www.inaturalist.org/observations/22276279
681	Bubulcus ibis	Cattle Egret	2019/4/4	5	0	7.2585710	80.5983990	https://www.inaturalist.org/observations/22023973
682	Bubulcus ibis	Cattle Egret	2019/4/8	4	0	7.2586230	80.5972210	https://www.inaturalist.org/observations/22452503
683	Bubulcus ibis	Cattle Egret	2019/4/11	5	0	7.2588610	80.5973680	https://www.inaturalist.org/observations/22353556
684	Bubulcus ibis	Cattle Egret	2019/4/20	4	0	7.2599570	80.5959770	https://www.inaturalist.org/observations/22791543
685	Bubulcus ibis	Cattle Egret	2019/3/21	7	0	7.2600217	80.5961083	https://www.inaturalist.org/observations/21538670
686	Bubulcus ibis	Cattle Egret	2019/4/5	3	0	7.2602000	80.5955547	https://www.inaturalist.org/observations/22084654
687	Bubulcus ibis	Cattle Egret	2019/4/19	4	0	7.2610310	80.5989680	https://www.inaturalist.org/observations/22806473
688	Bubulcus ibis	Cattle Egret	2019/4/1	5	0	7.2612512	80.5990635	https://www.inaturalist.org/observations/22293980
689	Bubulcus ibis	Cattle Egret	2019/4/5	5	0	7.2664960	80.5523680	https://www.inaturalist.org/observations/22007975
690	Bubulcus ibis	Cattle Egret	2015/4/30	3	0	7.2785276	80.2106728	https://www.inaturalist.org/observations/33942955
691	Bubulcus ibis	Cattle Egret	2019/4/6	3	0	7.2789340	80.7132162	https://www.inaturalist.org/observations/22218459
692	Bubulcus ibis	Cattle Egret	2018/4/30	5	0	7.2803003	80.5396383	https://www.inaturalist.org/observations/37120680
693	Bubulcus ibis	Cattle Egret	2022/3/29	2	0	7.2872939	79.8869002	https://www.inaturalist.org/observations/131833603
694	Bubulcus ibis	Cattle Egret	2022/2/19	3	0	7.2877668	79.8876124	https://www.inaturalist.org/observations/107299046
695	Bubulcus ibis	Cattle Egret	2019/4/18	4	0	7.2892270	80.6437520	https://www.inaturalist.org/observations/22685622
696	Bubulcus ibis	Cattle Egret	2016/7/19	1	0	7.2893954	80.6446149	https://www.inaturalist.org/observations/16777047
697	Bubulcus ibis	Cattle Egret	2018/3/9	2	0	7.2901762	80.6428282	https://www.inaturalist.org/observations/10388064
698	Bubulcus ibis	Cattle Egret	2021/12/18	4	0	7.2974630	79.8821879	https://www.inaturalist.org/observations/103233126
699	Bubulcus ibis	Cattle Egret	2021/12/31	2	0	7.2974630	79.8821879	https://www.inaturalist.org/observations/104056686

700	Bubulcus ibis	Cattle Egret	2021/12/31	1	0	7.2974630	79.8821879	https://www.inaturalist.org/observations/104056909
701	Bubulcus ibis	Cattle Egret	2022/1/5	2	0	7.2974630	79.8821879	https://www.inaturalist.org/observations/104420032
702	Bubulcus ibis	Cattle Egret	2022/1/5	2	0	7.2974630	79.8821879	https://www.inaturalist.org/observations/104495565
703	Bubulcus ibis	Cattle Egret	2022/1/5	2	0	7.2974630	79.8821879	https://www.inaturalist.org/observations/104495697
704	Bubulcus ibis	Cattle Egret	2018/10/27	4	0	7.2976237	79.8457658	https://www.inaturalist.org/observations/18347866
705	Bubulcus ibis	Cattle Egret	2019/12/27	3	0	7.3006741	80.3837603	https://www.inaturalist.org/observations/37004329
706	Bubulcus ibis	Cattle Egret	2017/6/27	2	0	7.3014151	79.8466912	https://www.inaturalist.org/observations/142225179
707	Bubulcus ibis	Cattle Egret	2022/4/1	3	0	7.3080916	80.5967783	https://www.inaturalist.org/observations/110072088
708	Bubulcus ibis	Cattle Egret	2019/4/15	3	0	7.3676620	80.7011990	https://www.inaturalist.org/observations/22594378
709	Bubulcus ibis	Cattle Egret	2015/12/26	4	0	7.3902072	80.9872767	https://www.inaturalist.org/observations/46835661
710	Bubulcus ibis	Cattle Egret	2019/3/19	1	0	7.6412091	79.8015354	https://www.inaturalist.org/observations/112486466
711	Bubulcus ibis	Cattle Egret	2022/7/10	3	0	7.7057844	79.8119244	https://www.inaturalist.org/observations/125744699
712	Bubulcus ibis	Cattle Egret	2022/3/12	2	0	7.8114383	80.6134533	https://www.inaturalist.org/observations/109821237
713	Bubulcus ibis	Cattle Egret	2012/12/23	3	0	7.8184869	79.8307800	http://www.inaturalist.org/observations/177096
714	Bubulcus ibis	Cattle Egret	2019/1/3	1	0	7.8388137	80.6495905	https://www.inaturalist.org/observations/144267259
715	Bubulcus ibis	Cattle Egret	2011/3/11	6	0	7.8569575	80.6244459	https://www.inaturalist.org/observations/65947385
716	Bubulcus ibis	Cattle Egret	2014/4/12	6	0	7.8691282	80.7717969	https://www.inaturalist.org/observations/82784127
717	Bubulcus ibis	Cattle Egret	2015/11/5	7	0	7.8697216	80.7064906	https://www.inaturalist.org/observations/63248100
718	Bubulcus ibis	Cattle Egret	2015/11/5	6	0	7.8708139	80.7060615	https://www.inaturalist.org/observations/63248095
719	Bubulcus ibis	Cattle Egret	2017/11/29	3	0	7.8730540	80.7717970	https://www.inaturalist.org/observations/9951508
720	Bubulcus ibis	Cattle Egret	2017/8/22	4	0	7.8730540	80.7717970	https://www.inaturalist.org/observations/49650837
721	Bubulcus ibis	Cattle Egret	2014/12/16	2	0	7.8730540	80.7717970	https://www.inaturalist.org/observations/93801794
722	Bubulcus ibis coromandus	Cattle Egret	2022/5/10	1	0	7.8730540	80.7717970	https://www.inaturalist.org/observations/116513882
723	Bubulcus ibis coromandus	Cattle Egret	2022/5/10	1	0	7.8730540	80.7717970	https://www.inaturalist.org/observations/116513972
724	Bubulcus ibis	Cattle Egret	2018/12/21	4	0	7.8742170	80.6511287	https://www.inaturalist.org/observations/19439511

725	Bubulcus ibis	Cattle Egret	2019/3/20	1	0	7.8777860	80.7026284	https://www.inaturalist.org/observations/112537058
726	Bubulcus ibis	Cattle Egret	2012/11/2	3	0	7.8843230	80.6946805	https://www.inaturalist.org/observations/41177237
727	Bubulcus ibis	Cattle Egret	2014/4/14	4	0	7.8865081	80.7223981	https://www.inaturalist.org/observations/19155040
728	Bubulcus ibis	Cattle Egret	2014/2/4	5	0	7.8898600	80.7258500	http://www.inaturalist.org/observations/3849787
729	Bubulcus ibis	Cattle Egret	2022/3/11	3	0	7.8931581	80.6995372	https://www.inaturalist.org/observations/108343712
730	Bubulcus ibis	Cattle Egret	2018/8/16	6	0	7.9253154	80.7195179	https://www.inaturalist.org/observations/68689155
731	Bubulcus ibis coromandus	Cattle Egret	2014/1/22	1	0	7.9286743	80.9942658	https://www.inaturalist.org/observations/92603376
732	Bubulcus ibis	Cattle Egret	2019/8/27	1	0	7.9362865	80.7120101	https://www.inaturalist.org/observations/38068707
733	Bubulcus ibis	Cattle Egret	2019/2/13	4	0	7.9397678	80.9978699	https://www.inaturalist.org/observations/20690601
734	Bubulcus ibis coromandus	Cattle Egret	2019/2/7	4	0	7.9403384	81.0187984	https://www.inaturalist.org/observations/30735094
735	Bubulcus ibis	Cattle Egret	2020/2/6	2	0	7.9418300	80.9975583	https://www.inaturalist.org/observations/38380446
736	Bubulcus ibis	Cattle Egret	2019/1/3	1	0	7.9470051	81.0004796	https://www.inaturalist.org/observations/144267303
737	Bubulcus ibis	Cattle Egret	2019/3/4	3	0	7.9502533	80.9719167	https://www.inaturalist.org/observations/20946459
738	Bubulcus ibis	Cattle Egret	2020/1/26	5	0	7.9567603	80.7557307	https://www.inaturalist.org/observations/39528344
739	Bubulcus ibis	Cattle Egret	2022/2/1	1	0	7.9569250	80.7538222	https://www.inaturalist.org/observations/144442131
740	Bubulcus ibis	Cattle Egret	2019/2/6	4	0	7.9590717	80.7439500	https://www.inaturalist.org/observations/26051330
741	Bubulcus ibis	Cattle Egret	2022/1/12	2	0	7.9648233	81.0038017	https://www.inaturalist.org/observations/111399813
742	Bubulcus ibis	Cattle Egret	2013/2/28	4	0	7.9658333	81.0047222	https://www.inaturalist.org/observations/66505378
743	Bubulcus ibis	Cattle Egret	2022/4/10	1	0	7.9800361	80.7495791	https://www.inaturalist.org/observations/118568159
744	Bubulcus ibis coromandus	Cattle Egret	2014/1/22	2	0	8.0011335	80.9240381	https://www.inaturalist.org/observations/92596674
745	Bubulcus ibis coromandus	Cattle Egret	2019/8/5	1	0	8.0083000	80.5714667	https://www.inaturalist.org/observations/32681995
746	Bubulcus ibis coromandus	Cattle Egret	2019/8/5	1	0	8.0083028	80.5714667	https://www.inaturalist.org/observations/32681953
747	Bubulcus ibis coromandus	Cattle Egret	2019/8/5	2	0	8.0083639	80.5715889	https://www.inaturalist.org/observations/32681889
748	Bubulcus ibis coromandus	Cattle Egret	2014/1/20	1	0	8.0326298	80.8676898	https://www.inaturalist.org/observations/92599096
749	Bubulcus ibis	Cattle Egret	2017/7/11	3	0	8.0329625	80.8989864	https://www.inaturalist.org/observations/142687590

750	Bubulcus ibis	Cattle Egret	2019/3/4	5	0	8.0340472	80.8923262	https://www.inaturalist.org/observations/20971220
751	Bubulcus ibis coromandus	Cattle Egret	2013/3/4	1	0	8.0360923	80.7472065	https://www.inaturalist.org/observations/110773971
752	Bubulcus ibis	Cattle Egret	2015/12/25	5	0	8.0484617	80.8719083	https://www.inaturalist.org/observations/46835251
753	Bubulcus ibis	Cattle Egret	2018/12/27	2	0	8.0918333	77.5578433	https://www.inaturalist.org/observations/34522899
754	Bubulcus ibis	Cattle Egret	2018/12/26	1	0	8.0918830	77.5561340	https://www.inaturalist.org/observations/34522460
755	Bubulcus ibis	Cattle Egret	2018/12/25	2	0	8.0919650	77.5561718	https://www.inaturalist.org/observations/32339442
756	Bubulcus ibis coromandus	Cattle Egret	2018/11/13	1	0	8.1020442	77.5428195	https://www.inaturalist.org/observations/56766461
757	Bubulcus ibis	Cattle Egret	2019/4/10	5	0	8.1198350	79.7068220	https://www.inaturalist.org/observations/22321998
758	Bubulcus ibis	Cattle Egret	2018/9/3	1	0	8.1611963	80.9136200	https://www.inaturalist.org/observations/76572649
759	Bubulcus ibis	Cattle Egret	2019/5/3	3	0	8.1756296	77.2444253	https://www.inaturalist.org/observations/24417781
760	Bubulcus ibis	Cattle Egret	2022/2/12	4	0	8.1802414	77.7049798	https://www.inaturalist.org/observations/107473734
761	Bubulcus ibis	Cattle Egret	2022/8/4	2	0	8.1818092	77.3759178	https://www.inaturalist.org/observations/129396149
762	Bubulcus ibis	Cattle Egret	2014/8/9	4	0	8.1832857	77.4118996	https://www.inaturalist.org/observations/20048289
763	Bubulcus ibis	Cattle Egret	2020/2/5	2	0	8.2088728	80.0739193	https://www.inaturalist.org/observations/39241201
764	Bubulcus ibis	Cattle Egret	2021/11/9	2	0	8.2347075	77.4841504	https://www.inaturalist.org/observations/105038157
765	Bubulcus ibis	Cattle Egret	2019/2/7	4	0	8.3113518	80.4036508	https://www.inaturalist.org/observations/30608589
766	Bubulcus ibis	Cattle Egret	2016/2/19	2	0	8.3113518	80.4036508	https://www.inaturalist.org/observations/37070167
767	Bubulcus ibis	Cattle Egret	2009/10/29	1	0	8.3210629	80.4083149	https://www.inaturalist.org/observations/131794540
768	Bubulcus ibis	Cattle Egret	2022/4/13	1	0	8.3251833	80.4162354	https://www.inaturalist.org/observations/111616846
769	Bubulcus ibis	Cattle Egret	2019/3/2	3	0	8.3287250	80.3857717	https://www.inaturalist.org/observations/20877009
770	Bubulcus ibis	Cattle Egret	2019/3/2	2	0	8.3288583	80.3817750	https://www.inaturalist.org/observations/20929021
771	Bubulcus ibis	Cattle Egret	2015/4/25	6	0	8.3365180	80.4034420	http://www.inaturalist.org/observations/1449687
772	Bubulcus ibis	Cattle Egret	2016/3/26	3	0	8.3374126	80.3939863	https://www.inaturalist.org/observations/127458276
773	Bubulcus ibis	Cattle Egret	2016/1/18	3	0	8.3430416	80.3996624	https://www.inaturalist.org/observations/42004348
774	Bubulcus ibis	Cattle Egret	2020/2/26	2	0	8.3445550	80.3890917	https://www.inaturalist.org/observations/39246264

775	Bubulcus ibis	Cattle Egret	2019/3/2	5	0	8.3463667	80.3975133	https://www.inaturalist.org/observations/20918356
776	Bubulcus ibis	Cattle Egret	2022/1/29	1	0	8.3465556	80.3973444	https://www.inaturalist.org/observations/144431379
777	Bubulcus ibis	Cattle Egret	2021/11/12	5	0	8.3467579	80.3971566	https://www.inaturalist.org/observations/129547666
778	Bubulcus ibis	Cattle Egret	2017/2/11	2	0	8.3467718	77.7412704	https://www.inaturalist.org/observations/65345104
779	Bubulcus ibis	Cattle Egret	2021/11/12	5	0	8.3467866	80.3972443	https://www.inaturalist.org/observations/129547662
780	Bubulcus ibis	Cattle Egret	2017/1/22	3	0	8.3482072	80.3969722	https://www.inaturalist.org/observations/106707655
781	Bubulcus ibis	Cattle Egret	2015/4/4	3	0	8.3495161	80.3977806	https://www.inaturalist.org/observations/12171871
782	Bubulcus ibis	Cattle Egret	2019/2/15	4	0	8.3499116	80.4051644	https://www.inaturalist.org/observations/20733717
783	Bubulcus ibis	Cattle Egret	2019/3/1	6	0	8.3523367	80.4335633	https://www.inaturalist.org/observations/20853968
784	Bubulcus ibis	Cattle Egret	2014/3/10	6	0	8.3564700	80.4172600	http://www.inaturalist.org/observations/2477697
785	Bubulcus ibis coromandus	Cattle Egret	2016/2/5	5	0	8.3652984	80.5453313	http://www.inaturalist.org/observations/3776908
786	Bubulcus ibis	Cattle Egret	2019/3/27	4	0	8.3690140	80.0316080	https://www.inaturalist.org/observations/21818490
787	Bubulcus ibis	Cattle Egret	2022/1/28	1	0	8.3776306	80.0698139	https://www.inaturalist.org/observations/144428273
788	Bubulcus ibis	Cattle Egret	2009/12/29	1	0	8.3802974	81.0073693	https://www.inaturalist.org/observations/144670701
789	Bubulcus ibis	Cattle Egret	2019/3/15	2	0	8.3888922	76.9760433	https://www.inaturalist.org/observations/47557628
790	Bubulcus ibis coromandus	Cattle Egret	2017/2/24	1	0	8.3948786	76.9738000	https://www.inaturalist.org/observations/62025560
791	Bubulcus ibis	Cattle Egret	2022/2/12	2	0	8.4080133	78.0449383	https://www.inaturalist.org/observations/106982307
792	Bubulcus ibis	Cattle Egret	2022/12/14	2	0	8.4142351	77.6054508	https://www.inaturalist.org/observations/144336667
793	Bubulcus ibis	Cattle Egret	2022/11/5	1	0	8.4184449	77.6017534	https://www.inaturalist.org/observations/141226255
794	Bubulcus ibis	Cattle Egret	2020/2/4	1	0	8.4213683	80.0090667	https://www.inaturalist.org/observations/39206350
795	Bubulcus ibis	Cattle Egret	2022/11/5	3	0	8.4217118	77.6003657	https://www.inaturalist.org/observations/143007899
796	Bubulcus ibis	Cattle Egret	2020/2/4	2	0	8.4240600	80.0240750	https://www.inaturalist.org/observations/39206425
797	Bubulcus ibis coromandus	Cattle Egret	2021/11/1	1	0	8.4252933	81.2892268	https://www.inaturalist.org/observations/100314689
798	Bubulcus ibis	Cattle Egret	2022/12/6	2	0	8.4271284	77.5951464	https://www.inaturalist.org/observations/143773204
799	Bubulcus ibis	Cattle Egret	2022/4/9	3	0	8.4474674	77.4365987	https://www.inaturalist.org/observations/116241815

800	Bubulcus ibis coromandus	Cattle Egret	2020/2/20	3	0	8.4562965	80.0476383	https://www.inaturalist.org/observations/76978489
801	Bubulcus ibis coromandus	Cattle Egret	2016/4/8	2	0	8.4563723	80.0476383	https://www.inaturalist.org/observations/58182603
802	Bubulcus ibis	Cattle Egret	2021/4/24	2	0	8.4750836	76.9469977	https://www.inaturalist.org/observations/76560267
803	Bubulcus ibis	Cattle Egret	2022/4/9	2	0	8.4888581	77.5165953	https://www.inaturalist.org/observations/116241814
804	Bubulcus ibis	Cattle Egret	2019/6/30	5	0	8.4928000	79.9210283	https://www.inaturalist.org/observations/41874539
805	Bubulcus ibis	Cattle Egret	2022/2/12	2	0	8.4959367	77.7591617	https://www.inaturalist.org/observations/106982271
806	Bubulcus ibis	Cattle Egret	2021/12/8	2	0	8.5176839	77.4194642	https://www.inaturalist.org/observations/103937933
807	Bubulcus ibis	Cattle Egret	2022/4/24	4	0	8.5205839	76.9615020	https://www.inaturalist.org/observations/112659173
808	Bubulcus ibis	Cattle Egret	2020/11/9	2	0	8.5255879	76.9366376	https://www.inaturalist.org/observations/64522318
809	Bubulcus ibis	Cattle Egret	2020/10/30	1	0	8.5255879	76.9366376	https://www.inaturalist.org/observations/64911333
810	Bubulcus ibis	Cattle Egret	2018/7/6	4	0	8.5301284	76.9384593	https://www.inaturalist.org/observations/22993370
811	Bubulcus ibis	Cattle Egret	2021/12/8	2	0	8.5537723	77.4860075	https://www.inaturalist.org/observations/103871978
812	Bubulcus ibis	Cattle Egret	2020/12/13	1	0	8.5742943	81.2392542	https://www.inaturalist.org/observations/66770255
813	Bubulcus ibis coromandus	Cattle Egret	2021/11/15	1	0	8.5744914	81.2392286	https://www.inaturalist.org/observations/101513105
814	Bubulcus ibis	Cattle Egret	2020/12/12	1	0	8.5748405	81.2388892	https://www.inaturalist.org/observations/66468965
815	Bubulcus ibis coromandus	Cattle Egret	2017/7/24	3	0	8.5781906	81.2305958	https://www.inaturalist.org/observations/69815167
816	Bubulcus ibis	Cattle Egret	2015/12/30	2	0	8.5784570	77.7837772	https://www.inaturalist.org/observations/66133948
817	Bubulcus ibis coromandus	Cattle Egret	2021/1/23	2	0	8.5918420	81.1881065	https://www.inaturalist.org/observations/68539402
818	Bubulcus ibis	Cattle Egret	2010/1/3	2	0	8.6181193	81.2087554	https://www.inaturalist.org/observations/144736080
819	Bubulcus ibis	Cattle Egret	2021/1/16	2	0	8.6229920	76.9404192	https://www.inaturalist.org/observations/73869241
820	Bubulcus ibis	Cattle Egret	2017/12/12	3	0	8.6730208	77.1406030	https://www.inaturalist.org/observations/36013736
821	Bubulcus ibis	Cattle Egret	2019/3/2	5	0	8.6822594	77.1365292	https://www.inaturalist.org/observations/20876031
822	Bubulcus ibis	Cattle Egret	2019/9/15	3	0	8.7043734	77.7382355	https://www.inaturalist.org/observations/32746141
823	Bubulcus ibis	Cattle Egret	2019/9/11	2	0	8.7055597	77.7354965	https://www.inaturalist.org/observations/32519279
824	Bubulcus ibis	Cattle Egret	2019/8/26	1	0	8.7129427	81.1847467	https://www.inaturalist.org/observations/36302483

825	Bubulcus ibis	Cattle Egret	2015/10/28	3	0	8.7139130	77.7566520	https://www.inaturalist.org/observations/23908672
826	Bubulcus ibis	Cattle Egret	2022/5/17	6	0	8.7266894	77.4992483	https://www.inaturalist.org/observations/120897873
827	Bubulcus ibis	Cattle Egret	2021/6/11	3	0	8.7583206	78.1740862	https://www.inaturalist.org/observations/91492855
828	Bubulcus ibis	Cattle Egret	2020/11/8	2	0	8.8241876	76.8948713	https://www.inaturalist.org/observations/64423760
829	Bubulcus ibis	Cattle Egret	2019/2/17	3	0	8.8396306	76.7009785	https://www.inaturalist.org/observations/65980816
830	Bubulcus ibis	Cattle Egret	2022/5/11	3	0	8.8698046	76.6018342	https://www.inaturalist.org/observations/124895278
831	Bubulcus ibis	Cattle Egret	2022/2/12	2	0	8.8698387	76.6012281	https://www.inaturalist.org/observations/125581766
832	Bubulcus ibis	Cattle Egret	2021/12/15	3	0	8.8700590	76.6012548	https://www.inaturalist.org/observations/103742515
833	Bubulcus ibis	Cattle Egret	2021/7/3	2	0	8.8836214	76.7710048	https://www.inaturalist.org/observations/85738054
834	Bubulcus ibis	Cattle Egret	2019/5/12	3	0	8.9023003	76.6565543	https://www.inaturalist.org/observations/66039631
835	Bubulcus ibis	Cattle Egret	2020/11/7	3	0	8.9493433	76.6266537	https://www.inaturalist.org/observations/64830385
836	Bubulcus ibis	Cattle Egret	2020/11/4	3	0	8.9540327	76.6344726	https://www.inaturalist.org/observations/64168848
837	Bubulcus ibis	Cattle Egret	2022/2/5	2	0	9.0104191	76.6601530	https://www.inaturalist.org/observations/106715290
838	Bubulcus ibis	Cattle Egret	2022/2/5	1	0	9.0118650	76.6632262	https://www.inaturalist.org/observations/106826229
839	Bubulcus ibis	Cattle Egret	2022/2/5	1	0	9.0122041	76.6640416	https://www.inaturalist.org/observations/106826301
840	Bubulcus ibis	Cattle Egret	2022/2/5	2	0	9.0122274	76.6640203	https://www.inaturalist.org/observations/106727199
841	Bubulcus ibis	Cattle Egret	2022/2/5	0	0	9.0123736	76.6643849	https://www.inaturalist.org/observations/106927263
842	Bubulcus ibis	Cattle Egret	2022/1/14	2	0	9.1672573	77.8642807	https://www.inaturalist.org/observations/104913050
843	Bubulcus ibis	Cattle Egret	2022/2/26	3	0	9.2173550	76.6483467	https://www.inaturalist.org/observations/142757715
844	Bubulcus ibis	Cattle Egret	2018/4/2	5	0	9.2690996	76.6068292	https://www.inaturalist.org/observations/10568397
845	Bubulcus ibis	Cattle Egret	2022/12/5	1	0	9.2908502	76.6127703	https://www.inaturalist.org/observations/143714391
846	Bubulcus ibis coromandus	Cattle Egret	2022/12/18	2	0	9.2955532	76.6162296	https://www.inaturalist.org/observations/144720722
847	Bubulcus ibis	Cattle Egret	2022/2/27	2	0	9.3064783	76.4994700	https://www.inaturalist.org/observations/142757846
848	Bubulcus ibis	Cattle Egret	2022/5/15	2	0	9.3388450	76.6537483	https://www.inaturalist.org/observations/142804992
849	Bubulcus ibis	Cattle Egret	2022/2/26	2	0	9.3430733	76.8294450	https://www.inaturalist.org/observations/142757681

850	Bubulcus ibis	Cattle Egret	2021/12/29	5	0	9.3518830	76.6953863	https://www.inaturalist.org/observations/130740593
851	Bubulcus ibis	Cattle Egret	2019/4/8	4	0	9.3724197	76.5837965	https://www.inaturalist.org/observations/22279557
852	Bubulcus ibis	Cattle Egret	2015/11/23	3	0	9.4390053	76.5443431	https://www.inaturalist.org/observations/62789459
853	Bubulcus ibis	Cattle Egret	2021/4/8	1	0	9.4973892	78.1380145	https://www.inaturalist.org/observations/85361802
854	Bubulcus ibis	Cattle Egret	2022/1/7	2	0	9.5032478	76.5490581	https://www.inaturalist.org/observations/105242733
855	Bubulcus ibis	Cattle Egret	2020/1/26	3	0	9.5082532	76.3600795	https://www.inaturalist.org/observations/63700766
856	Bubulcus ibis	Cattle Egret	2013/12/25	1	0	9.5324114	76.5497972	https://www.inaturalist.org/observations/81670885
857	Bubulcus ibis	Cattle Egret	2013/11/24	1	0	9.5325021	76.5498402	https://www.inaturalist.org/observations/81672393
858	Bubulcus ibis	Cattle Egret	2013/11/18	1	0	9.5325233	76.5498402	https://www.inaturalist.org/observations/81672390
859	Bubulcus ibis	Cattle Egret	2019/1/20	3	0	9.5665322	76.4423940	https://www.inaturalist.org/observations/41031567
860	Bubulcus ibis	Cattle Egret	2019/2/12	5	0	9.5694798	76.4241721	https://www.inaturalist.org/observations/20510689
861	Bubulcus ibis	Cattle Egret	2020/12/26	3	0	9.5761528	76.4372750	https://www.inaturalist.org/observations/67380180
862	Bubulcus ibis	Cattle Egret	2022/5/2	3	0	9.5762508	76.4367763	https://www.inaturalist.org/observations/124512723
863	Bubulcus ibis	Cattle Egret	2014/11/6	1	0	9.5838466	77.1786689	https://www.inaturalist.org/observations/71554140
864	Bubulcus ibis	Cattle Egret	2022/5/1	1	0	9.5886855	76.4243447	https://www.inaturalist.org/observations/114134091
865	Bubulcus ibis	Cattle Egret	2022/11/1	2	0	9.5923806	76.4463639	https://www.inaturalist.org/observations/140814954
866	Bubulcus ibis	Cattle Egret	2018/12/19	3	0	9.5972099	76.5206807	https://www.inaturalist.org/observations/42833313
867	Bubulcus ibis	Cattle Egret	2018/12/17	3	0	9.5976625	76.5110900	https://www.inaturalist.org/observations/42832935
868	Bubulcus ibis	Cattle Egret	2019/4/15	1	0	9.5979879	76.5110398	https://www.inaturalist.org/observations/41148754
869	Bubulcus ibis	Cattle Egret	2022/9/13	0	0	9.5987360	76.3116235	https://www.inaturalist.org/observations/134878703
870	Bubulcus ibis	Cattle Egret	2020/10/4	2	0	9.6005863	76.3579400	https://www.inaturalist.org/observations/62467718
871	Bubulcus ibis	Cattle Egret	2020/10/10	3	0	9.6005863	76.3579400	https://www.inaturalist.org/observations/62474673
872	Bubulcus ibis	Cattle Egret	2022/4/14	3	0	9.6122720	76.4308660	https://www.inaturalist.org/observations/111439582
873	Bubulcus ibis	Cattle Egret	2018/4/28	4	0	9.6194354	76.3233948	https://www.inaturalist.org/observations/12593413
874	Bubulcus ibis	Cattle Egret	2019/12/26	1	0	9.6264099	76.4291613	https://www.inaturalist.org/observations/96979531

875	Bubulcus ibis	Cattle Egret	2018/1/13	3	0	9.6328464	76.4185395	https://www.inaturalist.org/observations/117705602
876	Bubulcus ibis	Cattle Egret	2020/5/29	3	0	9.6681611	76.3214656	https://www.inaturalist.org/observations/48640775
877	Bubulcus ibis	Cattle Egret	2022/9/3	5	0	9.6682036	76.3492569	https://www.inaturalist.org/observations/133597569
878	Bubulcus ibis	Cattle Egret	2012/12/25	3	0	9.6741360	76.3400960	https://www.inaturalist.org/observations/129508023
879	Bubulcus ibis	Cattle Egret	2021/5/12	2	0	9.7184883	76.3124481	https://www.inaturalist.org/observations/78919236
880	Bubulcus ibis	Cattle Egret	2018/6/11	4	0	9.7347238	77.2843678	https://www.inaturalist.org/observations/63810305
881	Bubulcus ibis	Cattle Egret	2020/9/20	3	0	9.7377848	77.2953755	https://www.inaturalist.org/observations/61275907
882	Bubulcus ibis	Cattle Egret	2021/5/12	3	0	9.7835306	76.3114966	https://www.inaturalist.org/observations/78919241
883	Bubulcus ibis	Cattle Egret	2022/12/21	1	0	9.7900056	76.3056083	https://www.inaturalist.org/observations/144788573
884	Bubulcus ibis	Cattle Egret	2019/5/9	1	0	9.7902181	76.3047531	https://www.inaturalist.org/observations/48629635
885	Bubulcus ibis	Cattle Egret	2019/5/9	1	0	9.7902750	76.3047031	https://www.inaturalist.org/observations/45343360
886	Bubulcus ibis	Cattle Egret	2022/12/6	2	0	9.7903583	76.3046917	https://www.inaturalist.org/observations/143777200
887	Bubulcus ibis	Cattle Egret	2022/12/12	2	0	9.7903750	76.3044361	https://www.inaturalist.org/observations/144737744
888	Bubulcus ibis	Cattle Egret	2022/12/6	1	0	9.7904083	76.3041833	https://www.inaturalist.org/observations/144904391
889	Bubulcus ibis	Cattle Egret	2020/1/11	3	0	9.7904614	76.3045547	https://www.inaturalist.org/observations/46815207
890	Bubulcus ibis	Cattle Egret	2019/6/29	1	0	9.7907414	76.3045000	https://www.inaturalist.org/observations/48934618
891	Bubulcus ibis	Cattle Egret	2022/12/17	1	0	9.7974309	76.2889039	https://www.inaturalist.org/observations/145176299
892	Bubulcus ibis	Cattle Egret	2022/12/16	1	0	9.7974309	76.2889039	https://www.inaturalist.org/observations/145196162
893	Bubulcus ibis	Cattle Egret	2022/12/16	0	0	9.7974309	76.2889039	https://www.inaturalist.org/observations/145229271
894	Bubulcus ibis	Cattle Egret	2022/12/16	1	0	9.7974309	76.2889039	https://www.inaturalist.org/observations/145232288
895	Bubulcus ibis	Cattle Egret	2022/12/16	0	0	9.7974309	76.2889039	https://www.inaturalist.org/observations/145239554
896	Bubulcus ibis	Cattle Egret	2022/12/16	0	0	9.7974309	76.2889039	https://www.inaturalist.org/observations/145240338
897	Bubulcus ibis	Cattle Egret	2020/5/31	3	0	9.7994514	76.2931064	https://www.inaturalist.org/observations/48013974
898	Bubulcus ibis	Cattle Egret	2022/10/8	3	0	9.8310481	76.7883827	https://www.inaturalist.org/observations/138016629
899	Bubulcus ibis	Cattle Egret	2021/12/7	2	0	9.8321461	76.7884413	https://www.inaturalist.org/observations/102771311

900	Bubulcus ibis	Cattle Egret	2022/3/13	1	0	9.8330226	76.7624094	https://www.inaturalist.org/observations/110464538
901	Bubulcus ibis	Cattle Egret	2021/11/4	2	0	9.8391083	76.7832926	https://www.inaturalist.org/observations/100221967
902	Bubulcus ibis	Cattle Egret	2021/11/28	2	0	9.8398721	76.7836510	https://www.inaturalist.org/observations/102415704
903	Bubulcus ibis	Cattle Egret	2018/12/5	1	0	9.8404457	76.7829539	https://www.inaturalist.org/observations/88838342
904	Bubulcus ibis	Cattle Egret	2018/11/17	3	0	9.8477869	76.7453861	https://www.inaturalist.org/observations/87107764
905	Bubulcus ibis	Cattle Egret	2019/1/6	2	0	9.8505611	76.7666936	https://www.inaturalist.org/observations/96801431
906	Bubulcus ibis	Cattle Egret	2018/12/5	1	0	9.8718695	76.7708848	https://www.inaturalist.org/observations/88838329
907	Bubulcus ibis	Cattle Egret	2018/12/4	1	0	9.8765716	76.7651581	https://www.inaturalist.org/observations/88147612
908	Bubulcus ibis	Cattle Egret	2019/1/12	3	0	9.8845431	76.7327851	https://www.inaturalist.org/observations/97982984
909	Bubulcus ibis	Cattle Egret	2018/12/4	2	0	9.8922581	76.7420961	https://www.inaturalist.org/observations/88147642
910	Bubulcus ibis	Cattle Egret	2021/11/18	2	0	9.8955535	76.7487912	https://www.inaturalist.org/observations/101384704
911	Bubulcus ibis	Cattle Egret	2021/11/21	2	0	9.8955535	76.7487912	https://www.inaturalist.org/observations/101639237
912	Bubulcus ibis	Cattle Egret	2021/10/24	2	0	9.9057067	76.7075246	https://www.inaturalist.org/observations/99715084
913	Bubulcus ibis	Cattle Egret	2013/11/8	2	0	9.9186293	76.7813133	https://www.inaturalist.org/observations/91676800
914	Bubulcus ibis	Cattle Egret	2020/4/17	3	0	9.9312328	76.2673041	https://www.inaturalist.org/observations/42383442
915	Bubulcus ibis	Cattle Egret	2020/4/28	2	0	9.9312328	76.2673041	https://www.inaturalist.org/observations/44131245
916	Bubulcus ibis	Cattle Egret	2022/4/12	0	0	9.9415822	78.1575322	https://www.inaturalist.org/observations/111188875
917	Bubulcus ibis	Cattle Egret	2022/4/12	1	0	9.9426174	78.1559461	https://www.inaturalist.org/observations/111189041
918	Bubulcus ibis	Cattle Egret	2022/4/12	2	0	9.9442621	78.1551947	https://www.inaturalist.org/observations/111188929
919	Bubulcus ibis	Cattle Egret	2015/3/5	2	0	9.9520550	76.3113843	https://www.inaturalist.org/observations/48524789
920	Bubulcus ibis	Cattle Egret	2019/8/28	4	0	9.9531329	76.4087609	https://www.inaturalist.org/observations/31664248
921	Bubulcus ibis	Cattle Egret	2019/12/19	2	0	9.9664914	76.2396314	https://www.inaturalist.org/observations/36768853
922	Bubulcus ibis	Cattle Egret	2013/12/11	3	0	9.9668582	76.2399809	https://www.inaturalist.org/observations/97920203
923	Bubulcus ibis	Cattle Egret	2022/12/8	3	0	9.9943972	76.5780778	https://www.inaturalist.org/observations/143919755
924	Bubulcus ibis	Cattle Egret	2019/1/25	4	0	10.0055251	76.8538196	https://www.inaturalist.org/observations/121632002

925	Bubulcus ibis	Cattle Egret	2020/11/11	4	0	10.0119353	76.3673193	https://www.inaturalist.org/observations/65016443
926	Bubulcus ibis	Cattle Egret	2020/3/7	1	0	10.0367767	76.8573850	https://www.inaturalist.org/observations/40039875
927	Bubulcus ibis	Cattle Egret	2019/11/26	1	0	10.0458527	76.6167212	https://www.inaturalist.org/observations/80851267
928	Bubulcus ibis	Cattle Egret	2022/12/17	1	0	10.0516871	76.3404863	https://www.inaturalist.org/observations/144524363
929	Bubulcus ibis	Cattle Egret	2022/1/16	3	0	10.0522167	76.3382767	https://www.inaturalist.org/observations/142746163
930	Bubulcus ibis	Cattle Egret	2020/12/24	2	0	10.0529872	76.3155969	https://www.inaturalist.org/observations/66989711
931	Bubulcus ibis	Cattle Egret	2022/11/3	1	0	10.0932833	76.2556617	https://www.inaturalist.org/observations/140960929
932	Bubulcus ibis	Cattle Egret	2021/5/8	2	0	10.0942918	76.2671806	https://www.inaturalist.org/observations/105245024
933	Bubulcus ibis	Cattle Egret	2016/4/16	4	0	10.1039800	76.7004600	http://www.inaturalist.org/observations/3594702
934	Bubulcus ibis	Cattle Egret	2014/11/4	1	0	10.1039803	76.7004605	https://www.inaturalist.org/observations/71554034
935	Bubulcus ibis	Cattle Egret	2020/12/12	2	0	10.1154737	76.6748884	https://www.inaturalist.org/observations/66517059
936	Bubulcus ibis	Cattle Egret	2020/12/12	1	0	10.1160964	76.6746976	https://www.inaturalist.org/observations/66996892
937	Bubulcus ibis	Cattle Egret	2022/5/10	5	0	10.1174654	76.6746856	https://www.inaturalist.org/observations/117595405
938	Bubulcus ibis	Cattle Egret	2022/2/5	3	0	10.1174900	76.3553688	https://www.inaturalist.org/observations/106155008
939	Bubulcus ibis	Cattle Egret	2021/11/3	1	0	10.1275216	76.3344964	https://www.inaturalist.org/observations/101473541
940	Bubulcus ibis	Cattle Egret	2019/5/15	3	0	10.1297816	76.6870809	https://www.inaturalist.org/observations/80905555
941	Bubulcus ibis	Cattle Egret	2022/12/15	2	0	10.1499581	76.3689709	https://www.inaturalist.org/observations/144409654
942	Bubulcus ibis	Cattle Egret	2018/6/8	3	0	10.2673954	76.2081252	https://www.inaturalist.org/observations/56645610
943	Bubulcus ibis	Cattle Egret	2021/6/5	3	0	10.2955218	76.2314037	https://www.inaturalist.org/observations/82049019
944	Bubulcus ibis	Cattle Egret	2021/12/2	5	0	10.3021427	76.4273844	https://www.inaturalist.org/observations/102439521
945	Bubulcus ibis	Cattle Egret	2022/4/12	2	0	10.3021915	76.4273844	https://www.inaturalist.org/observations/111198820
946	Bubulcus ibis coromandus	Cattle Egret	2011/12/16	4	0	10.3235908	76.9510030	https://www.inaturalist.org/observations/30495577
947	Bubulcus ibis	Cattle Egret	2015/10/18	1	0	10.3235908	76.9510030	https://www.inaturalist.org/observations/44519137
948	Bubulcus ibis	Cattle Egret	2021/3/29	3	0	10.3269734	76.9553967	https://www.inaturalist.org/observations/105206849
949	Bubulcus ibis	Cattle Egret	2015/5/4	4	0	10.3472648	76.2531318	https://www.inaturalist.org/observations/35271986

950	Bubulcus ibis	Cattle Egret	2015/5/10	3	0	10.3515740	76.2519754	https://www.inaturalist.org/observations/46835682
951	Bubulcus ibis	Cattle Egret	2022/5/1	2	0	10.3613150	76.2500967	https://www.inaturalist.org/observations/114746676
952	Bubulcus ibis	Cattle Egret	2022/5/1	3	0	10.3613150	76.2500967	https://www.inaturalist.org/observations/142801401
953	Bubulcus ibis	Cattle Egret	2019/6/18	2	0	10.3699133	76.2826892	https://www.inaturalist.org/observations/53833755
954	Bubulcus ibis	Cattle Egret	2021/10/31	1	0	10.4175799	76.2296578	https://www.inaturalist.org/observations/99880859
955	Bubulcus ibis	Cattle Egret	2021/10/31	1	0	10.4176170	76.2296766	https://www.inaturalist.org/observations/100250371
956	Bubulcus ibis	Cattle Egret	2021/10/31	2	0	10.4257830	76.2223830	https://www.inaturalist.org/observations/100293840
957	Bubulcus ibis	Cattle Egret	2020/5/18	4	0	10.4393498	76.1198433	https://www.inaturalist.org/observations/46326381
958	Bubulcus ibis	Cattle Egret	2021/11/1	0	0	10.4441280	76.2336700	https://www.inaturalist.org/observations/100040873
959	Bubulcus ibis	Cattle Egret	2014/12/29	1	0	10.4470060	76.8156911	https://www.inaturalist.org/observations/45582537
960	Bubulcus ibis	Cattle Egret	2021/11/3	3	0	10.4489203	76.2310257	https://www.inaturalist.org/observations/100128799
961	Bubulcus ibis	Cattle Egret	2020/11/29	2	0	10.4517100	76.1549286	https://www.inaturalist.org/observations/65822845
962	Bubulcus ibis	Cattle Egret	2022/12/18	1	0	10.4543123	76.1537499	https://www.inaturalist.org/observations/144864214
963	Bubulcus ibis	Cattle Egret	2017/5/24	1	0	10.5101373	76.1468568	https://www.inaturalist.org/observations/30260336
964	Bubulcus ibis	Cattle Egret	2020/11/7	3	0	10.5276314	76.2144347	https://www.inaturalist.org/observations/64367107
965	Bubulcus ibis	Cattle Egret	2018/4/13	6	0	10.5306050	76.1360700	https://www.inaturalist.org/observations/18016386
966	Bubulcus ibis	Cattle Egret	2020/1/6	2	0	10.5321017	76.1283967	https://www.inaturalist.org/observations/44162266
967	Bubulcus ibis	Cattle Egret	2020/1/29	2	0	10.5332333	76.1289917	https://www.inaturalist.org/observations/67019445
968	Bubulcus ibis	Cattle Egret	2018/5/24	2	0	10.5336781	76.1758381	https://www.inaturalist.org/observations/140128742
969	Bubulcus ibis	Cattle Egret	2021/4/26	2	0	10.5368165	76.0237187	https://www.inaturalist.org/observations/79491993
970	Bubulcus ibis	Cattle Egret	2021/12/18	2	0	10.5397262	76.1528520	https://www.inaturalist.org/observations/103245107
971	Bubulcus ibis	Cattle Egret	2021/1/3	2	0	10.5450770	76.1464903	https://www.inaturalist.org/observations/67513216
972	Bubulcus ibis	Cattle Egret	2020/2/23	0	0	10.5508767	76.1763967	https://www.inaturalist.org/observations/39213485
973	Bubulcus ibis	Cattle Egret	2021/4/15	3	0	10.5519758	76.1755809	https://www.inaturalist.org/observations/74334730
974	Bubulcus ibis	Cattle Egret	2022/3/26	4	0	10.5782382	76.0190886	https://www.inaturalist.org/observations/109656608

975	Bubulcus ibis	Cattle Egret	2022/5/6	2	0	10.5924689	76.0330121	https://www.inaturalist.org/observations/116967368
976	Bubulcus ibis	Cattle Egret	2021/5/8	2	0	10.6138270	76.1947792	https://www.inaturalist.org/observations/107043451
977	Bubulcus ibis	Cattle Egret	2021/5/22	3	0	10.6139988	76.1941462	https://www.inaturalist.org/observations/107045247
978	Bubulcus ibis	Cattle Egret	2018/12/14	5	0	10.6141210	76.1443262	https://www.inaturalist.org/observations/19033119
979	Bubulcus ibis	Cattle Egret	2020/5/31	2	0	10.6153063	76.1893091	https://www.inaturalist.org/observations/49757046
980	Bubulcus ibis	Cattle Egret	2020/5/5	4	0	10.6158836	76.1932527	https://www.inaturalist.org/observations/48354029
981	Bubulcus ibis	Cattle Egret	2022/3/18	2	0	10.6323317	76.8009967	https://www.inaturalist.org/observations/115064531
982	Bubulcus ibis	Cattle Egret	2022/3/18	3	0	10.6323317	76.8009967	https://www.inaturalist.org/observations/142799117
983	Bubulcus ibis	Cattle Egret	2022/5/5	3	0	10.6873587	78.6913244	https://www.inaturalist.org/observations/115508703
984	Bubulcus ibis	Cattle Egret	2021/12/24	2	0	10.7034567	79.3205533	https://www.inaturalist.org/observations/103745310
985	Bubulcus ibis	Cattle Egret	2016/12/29	3	0	10.7472960	76.4672130	https://www.inaturalist.org/observations/87008585
986	Bubulcus ibis	Cattle Egret	2016/12/28	3	0	10.7475660	76.4673670	https://www.inaturalist.org/observations/87008582
987	Bubulcus ibis	Cattle Egret	2016/12/17	3	0	10.7476287	76.4673708	https://www.inaturalist.org/observations/65301098
988	Bubulcus ibis	Cattle Egret	2015/11/23	3	0	10.7497424	76.3786039	https://www.inaturalist.org/observations/62789120
989	Bubulcus ibis	Cattle Egret	2022/1/1	4	0	10.7551158	76.6718444	https://www.inaturalist.org/observations/104101276
990	Bubulcus ibis	Cattle Egret	2020/2/15	1	0	10.7726572	76.2538196	https://www.inaturalist.org/observations/38853562
991	Bubulcus ibis	Cattle Egret	2020/2/15	1	0	10.7727686	76.2542289	https://www.inaturalist.org/observations/38853701
992	Bubulcus ibis	Cattle Egret	2020/4/17	1	0	10.7727724	79.1329936	https://www.inaturalist.org/observations/42749157
993	Bubulcus ibis	Cattle Egret	2017/11/9	4	0	10.7846735	76.6618214	https://www.inaturalist.org/observations/10518015
994	Bubulcus ibis	Cattle Egret	2022/5/11	5	0	10.7861367	75.9180217	https://www.inaturalist.org/observations/131548749
995	Bubulcus ibis	Cattle Egret	2021/3/10	1	0	10.7867270	76.6547931	https://www.inaturalist.org/observations/103955084
996	Bubulcus ibis	Cattle Egret	2016/1/24	5	0	10.7867300	76.6547930	https://www.inaturalist.org/observations/7279949
997	Bubulcus ibis	Cattle Egret	2021/2/16	3	0	10.7867303	76.6547932	https://www.inaturalist.org/observations/69921945
998	Bubulcus ibis	Cattle Egret	2022/11/15	4	0	10.8057297	76.1956646	https://www.inaturalist.org/observations/142901640
999	Bubulcus ibis	Cattle Egret	2022/3/13	3	0	10.8156917	76.8009017	https://www.inaturalist.org/observations/142797634

1000	Bubulcus ibis	Cattle Egret	2022/3/13	3	0	10.8401531	76.8356224	https://www.inaturalist.org/observations/115189758
1001	Bubulcus ibis	Cattle Egret	2022/3/13	3	0	10.8401531	76.8356224	https://www.inaturalist.org/observations/115189810
1002	Bubulcus ibis	Cattle Egret	2019/11/17	4	0	10.8656858	75.9843017	https://www.inaturalist.org/observations/67191609
1003	Bubulcus ibis	Cattle Egret	2021/5/23	4	0	10.8847499	76.0658477	https://www.inaturalist.org/observations/79949338
1004	Bubulcus ibis	Cattle Egret	2021/5/30	4	0	10.8849484	76.0658695	https://www.inaturalist.org/observations/80846005
1005	Bubulcus ibis	Cattle Egret	2021/5/30	3	0	10.8849491	76.0658725	https://www.inaturalist.org/observations/80846388
1006	Bubulcus ibis	Cattle Egret	2022/4/20	3	0	10.9567124	76.1757242	https://www.inaturalist.org/observations/112127014
1007	Bubulcus ibis	Cattle Egret	2020/10/31	2	0	10.9835556	77.5368528	https://www.inaturalist.org/observations/63890073
1008	Bubulcus ibis coromandus	Cattle Egret	2020/2/15	1	0	10.9938033	75.8762967	https://www.inaturalist.org/observations/38853760
1009	Bubulcus ibis	Cattle Egret	2020/5/27	1	0	11.0168445	76.9558321	https://www.inaturalist.org/observations/92664890
1010	Bubulcus ibis	Cattle Egret	2021/1/14	2	0	11.0953602	77.3750794	https://www.inaturalist.org/observations/69359399
1011	Bubulcus ibis	Cattle Egret	2022/2/12	3	0	11.1072083	76.6601750	https://www.inaturalist.org/observations/142754219
1012	Bubulcus ibis coromandus	Cattle Egret	2021/2/3	1	0	11.1275708	79.4543910	https://www.inaturalist.org/observations/70375240
1013	Bubulcus ibis	Cattle Egret	2020/12/25	1	0	11.1625398	75.8794249	https://www.inaturalist.org/observations/67196756
1014	Bubulcus ibis	Cattle Egret	2020/12/27	4	0	11.1625398	75.8794249	https://www.inaturalist.org/observations/67401093
1015	Bubulcus ibis	Cattle Egret	2020/6/2	2	0	11.2195874	76.9717326	https://www.inaturalist.org/observations/50131868
1016	Bubulcus ibis	Cattle Egret	2019/5/6	4	0	11.2341230	77.6586236	https://www.inaturalist.org/observations/38786110
1017	Bubulcus ibis	Cattle Egret	2022/2/13	1	0	11.2543865	77.6576908	https://www.inaturalist.org/observations/107477934
1018	Bubulcus ibis	Cattle Egret	2022/2/13	2	0	11.2543865	77.6576908	https://www.inaturalist.org/observations/107477962
1019	Bubulcus ibis	Cattle Egret	2022/2/13	2	0	11.2543865	77.6576908	https://www.inaturalist.org/observations/107477964
1020	Bubulcus ibis	Cattle Egret	2021/3/28	1	0	11.2587319	75.7804101	https://www.inaturalist.org/observations/83751710
1021	Bubulcus ibis	Cattle Egret	2021/12/23	2	0	11.2621500	79.5212085	https://www.inaturalist.org/observations/103529526
1022	Bubulcus ibis	Cattle Egret	2019/12/21	1	0	11.2757852	77.5829873	https://www.inaturalist.org/observations/86294149
1023	Bubulcus ibis	Cattle Egret	2019/10/16	3	0	11.2757855	77.5829873	https://www.inaturalist.org/observations/37124833
1024	Bubulcus ibis	Cattle Egret	2021/3/19	2	0	11.3043970	75.8029440	https://www.inaturalist.org/observations/71545828

1025	Bubulcus ibis	Cattle Egret	2022/2/6	2	0	11.3176603	79.2445983	https://www.inaturalist.org/observations/106317887
1026	Bubulcus ibis	Cattle Egret	2022/4/3	3	0	11.3222382	75.7479345	https://www.inaturalist.org/observations/138749339
1027	Bubulcus ibis	Cattle Egret	2022/4/3	3	0	11.3223102	75.7460028	https://www.inaturalist.org/observations/138749348
1028	Bubulcus ibis	Cattle Egret	2022/4/3	3	0	11.3226160	75.7463895	https://www.inaturalist.org/observations/138749343
1029	Bubulcus ibis	Cattle Egret	2019/1/15	2	0	11.3230384	79.2433528	https://www.inaturalist.org/observations/54716334
1030	Bubulcus ibis	Cattle Egret	2022/12/3	4	0	11.3230384	79.2433528	https://www.inaturalist.org/observations/143614492
1031	Bubulcus ibis	Cattle Egret	2022/12/4	4	0	11.3230384	79.2433528	https://www.inaturalist.org/observations/143652329
1032	Bubulcus ibis	Cattle Egret	2020/1/12	1	0	11.3230466	79.2433528	https://www.inaturalist.org/observations/82839968
1033	Bubulcus ibis	Cattle Egret	2022/1/13	3	0	11.3230466	79.2433528	https://www.inaturalist.org/observations/104866439
1034	Bubulcus ibis	Cattle Egret	2022/4/3	2	0	11.3233098	75.7465177	https://www.inaturalist.org/observations/138749344
1035	Bubulcus ibis	Cattle Egret	2021/1/10	1	0	11.3426158	79.2251352	https://www.inaturalist.org/observations/81837099
1036	Bubulcus ibis	Cattle Egret	2022/12/25	2	0	11.3681160	75.8361460	https://www.inaturalist.org/observations/145027842
1037	Bubulcus ibis	Cattle Egret	2018/4/20	4	0	11.5284281	76.5272980	https://www.inaturalist.org/observations/13019062
1038	Bubulcus ibis	Cattle Egret	2019/3/23	4	0	11.5766897	75.6322327	https://www.inaturalist.org/observations/21513962
1039	Bubulcus ibis	Cattle Egret	2020/12/25	3	0	11.5877341	78.7086467	https://www.inaturalist.org/observations/67110798
1040	Bubulcus ibis	Cattle Egret	2021/1/17	1	0	11.5901419	78.7165562	https://www.inaturalist.org/observations/142125251
1041	Bubulcus ibis	Cattle Egret	2022/4/10	3	0	11.6154601	92.6190327	https://www.inaturalist.org/observations/111493591
1042	Bubulcus ibis	Cattle Egret	2022/4/10	3	0	11.6154601	92.6190327	https://www.inaturalist.org/observations/111493592
1043	Bubulcus ibis	Cattle Egret	2021/4/4	1	0	11.6645959	78.1460141	https://www.inaturalist.org/observations/72876224
1044	Bubulcus ibis	Cattle Egret	2022/2/27	3	0	11.6645959	78.1460141	https://www.inaturalist.org/observations/107537066
1045	Bubulcus ibis	Cattle Egret	2022/4/16	1	0	11.6741251	78.1715488	https://www.inaturalist.org/observations/111619748
1046	Bubulcus ibis	Cattle Egret	2018/2/18	6	0	11.6874052	78.1806683	https://www.inaturalist.org/observations/57088475
1047	Bubulcus ibis	Cattle Egret	2020/10/30	2	0	11.6874052	78.1806683	https://www.inaturalist.org/observations/63812303
1048	Bubulcus ibis	Cattle Egret	2020/11/8	4	0	11.6874213	78.1806683	https://www.inaturalist.org/observations/64584558
1049	Bubulcus ibis	Cattle Egret	2021/2/13	1	0	11.6874213	78.1806683	https://www.inaturalist.org/observations/69667427

1050	Bubulcus ibis	Cattle Egret	2021/2/28	1	0	11.6874213	78.1806683	https://www.inaturalist.org/observations/70307244
1051	Bubulcus ibis	Cattle Egret	2021/3/28	1	0	11.6874213	78.1806683	https://www.inaturalist.org/observations/72243885
1052	Bubulcus ibis	Cattle Egret	2021/4/4	1	0	11.6874213	78.1806683	https://www.inaturalist.org/observations/72866067
1053	Bubulcus ibis	Cattle Egret	2021/4/4	1	0	11.6874213	78.1806683	https://www.inaturalist.org/observations/72866252
1054	Bubulcus ibis	Cattle Egret	2021/4/14	1	0	11.6874213	78.1806683	https://www.inaturalist.org/observations/73919916
1055	Bubulcus ibis	Cattle Egret	2021/4/14	1	0	11.6874213	78.1806683	https://www.inaturalist.org/observations/73919986
1056	Bubulcus ibis	Cattle Egret	2021/11/11	1	0	11.6874213	78.1806683	https://www.inaturalist.org/observations/100985304
1057	Bubulcus ibis	Cattle Egret	2021/11/28	1	0	11.6874213	78.1806683	https://www.inaturalist.org/observations/102079496
1058	Bubulcus ibis	Cattle Egret	2021/12/25	1	0	11.6874213	78.1806683	https://www.inaturalist.org/observations/103620467
1059	Bubulcus ibis	Cattle Egret	2022/5/1	1	0	11.6874213	78.1806683	https://www.inaturalist.org/observations/114130096
1060	Bubulcus ibis	Cattle Egret	2022/5/8	3	0	11.6874213	78.1806683	https://www.inaturalist.org/observations/116080660
1061	Bubulcus ibis	Cattle Egret	2022/6/5	3	0	11.6924019	78.1991896	https://www.inaturalist.org/observations/120296773
1062	Bubulcus ibis	Cattle Egret	2022/2/19	1	0	11.6976123	78.1806287	https://www.inaturalist.org/observations/107030734
1063	Bubulcus ibis	Cattle Egret	2021/3/21	2	0	11.6976560	78.1806287	https://www.inaturalist.org/observations/71684987
1064	Bubulcus ibis	Cattle Egret	2022/2/18	2	0	11.7031338	78.1877922	https://www.inaturalist.org/observations/106962955
1065	Bubulcus ibis	Cattle Egret	2021/11/21	2	0	11.7059375	78.2561577	https://www.inaturalist.org/observations/101693789
1066	Bubulcus ibis	Cattle Egret	2022/4/9	3	0	11.7092688	78.1893536	https://www.inaturalist.org/observations/110842021
1067	Bubulcus ibis	Cattle Egret	2022/10/1	1	0	11.7103509	78.1892034	https://www.inaturalist.org/observations/137237017
1068	Bubulcus ibis	Cattle Egret	2022/10/5	1	0	11.7104119	78.1890140	https://www.inaturalist.org/observations/137625383
1069	Bubulcus ibis	Cattle Egret	2022/3/12	3	0	11.7107393	78.1894515	https://www.inaturalist.org/observations/108429868
1070	Bubulcus ibis	Cattle Egret	2020/4/8	4	0	11.7119336	76.1638825	https://www.inaturalist.org/observations/43269356
1071	Bubulcus ibis	Cattle Egret	2022/1/2	3	0	11.7189508	78.2522095	https://www.inaturalist.org/observations/104380168
1072	Bubulcus ibis	Cattle Egret	2021/5/23	2	0	11.7341025	75.9376147	https://www.inaturalist.org/observations/80451337
1073	Bubulcus ibis	Cattle Egret	2021/3/19	2	0	11.7356162	78.2020388	https://www.inaturalist.org/observations/71538449
1074	Bubulcus ibis	Cattle Egret	2021/4/14	1	0	11.7356162	78.2020388	https://www.inaturalist.org/observations/73919845

1075	Bubulcus ibis	Cattle Egret	2018/4/29	4	0	11.7462051	75.4881761	https://www.inaturalist.org/observations/11711102
1076	Bubulcus ibis	Cattle Egret	2022/1/3	2	0	11.8049115	75.4368397	https://www.inaturalist.org/observations/104247079
1077	Bubulcus ibis	Cattle Egret	2022/10/7	3	0	11.8101363	75.4542766	https://www.inaturalist.org/observations/137851158
1078	Bubulcus ibis	Cattle Egret	2014/12/27	2	0	11.8263213	76.0666054	https://www.inaturalist.org/observations/105847565
1079	Bubulcus ibis	Cattle Egret	2019/12/23	1	0	11.8294520	75.5409521	https://www.inaturalist.org/observations/77643289
1080	Bubulcus ibis	Cattle Egret	2016/1/31	4	0	11.8328729	75.4641119	https://www.inaturalist.org/observations/47139127
1081	Bubulcus ibis	Cattle Egret	2017/2/9	1	0	11.8329186	93.0526120	https://www.inaturalist.org/observations/82026000
1082	Bubulcus ibis	Cattle Egret	2021/3/8	1	0	11.8413837	75.4203614	https://www.inaturalist.org/observations/70886617
1083	Bubulcus ibis	Cattle Egret	2006/11/16	4	0	11.8624817	77.1550204	https://www.inaturalist.org/observations/31931943
1084	Bubulcus ibis	Cattle Egret	2022/10/9	1	0	11.8634628	78.1229461	https://www.inaturalist.org/observations/138241575
1085	Bubulcus ibis	Cattle Egret	2021/4/11	2	0	11.8763714	75.3995285	https://www.inaturalist.org/observations/73589562
1086	Bubulcus ibis	Cattle Egret	2020/10/5	3	0	11.8819714	78.1446452	https://www.inaturalist.org/observations/62318070
1087	Bubulcus ibis	Cattle Egret	2021/11/3	2	0	11.8932289	78.1000181	https://www.inaturalist.org/observations/100134178
1088	Bubulcus ibis	Cattle Egret	2021/7/24	1	0	11.8993930	77.7720800	https://www.inaturalist.org/observations/88726543
1089	Bubulcus ibis	Cattle Egret	2020/5/14	6	0	11.9030545	75.3738686	https://www.inaturalist.org/observations/48310431
1090	Bubulcus ibis	Cattle Egret	2012/1/16	2	0	11.9048304	78.6166376	https://www.inaturalist.org/observations/97326715
1091	Bubulcus ibis	Cattle Egret	2021/5/11	4	0	11.9446843	75.3849381	https://www.inaturalist.org/observations/81266614
1092	Bubulcus ibis	Cattle Egret	2018/12/10	4	0	11.9446843	75.3849381	https://www.inaturalist.org/observations/97533279
1093	Bubulcus ibis	Cattle Egret	2018/12/10	4	0	11.9446843	75.3849381	https://www.inaturalist.org/observations/110002511
1094	Bubulcus ibis	Cattle Egret	2022/3/16	1	0	11.9446843	75.3849381	https://www.inaturalist.org/observations/110081975
1095	Bubulcus ibis	Cattle Egret	2022/6/27	2	0	11.9446843	75.3849381	https://www.inaturalist.org/observations/126454520
1096	Bubulcus ibis	Cattle Egret	2022/7/8	1	0	11.9446843	75.3849381	https://www.inaturalist.org/observations/127078812
1097	Bubulcus ibis	Cattle Egret	2022/7/8	2	0	11.9446843	75.3849381	https://www.inaturalist.org/observations/127081561
1098	Bubulcus ibis	Cattle Egret	2022/7/8	2	0	11.9446843	75.3849381	https://www.inaturalist.org/observations/127081887
1099	Bubulcus ibis	Cattle Egret	2022/4/9	1	0	11.9446843	75.3849381	https://www.inaturalist.org/observations/128117620

1100	Bubulcus ibis	Cattle Egret	2022/6/8	3	0	11.9446843	75.3849381	https://www.inaturalist.org/observations/128174589
1101	Bubulcus ibis	Cattle Egret	2022/11/13	2	0	11.9446843	75.3849381	https://www.inaturalist.org/observations/142122817
1102	Bubulcus ibis	Cattle Egret	2018/5/5	3	0	11.9446846	75.3849381	https://www.inaturalist.org/observations/83820057
1103	Bubulcus ibis	Cattle Egret	2018/7/8	4	0	11.9446846	75.3849381	https://www.inaturalist.org/observations/83820645
1104	Bubulcus ibis	Cattle Egret	2022/11/14	2	0	11.9760500	92.9875570	https://www.inaturalist.org/observations/142804603
1105	Bubulcus ibis	Cattle Egret	2020/4/18	4	0	11.9842763	75.3994546	https://www.inaturalist.org/observations/45567529
1106	Bubulcus ibis	Cattle Egret	2020/4/13	1	0	11.9871204	92.9480729	https://www.inaturalist.org/observations/48747655
1107	Bubulcus ibis	Cattle Egret	2020/11/6	3	0	11.9884395	75.3211505	https://www.inaturalist.org/observations/64500777
1108	Bubulcus ibis	Cattle Egret	2021/4/29	2	0	11.9887737	75.3196200	https://www.inaturalist.org/observations/75651818
1109	Bubulcus ibis	Cattle Egret	2022/2/21	3	0	11.9957617	75.3770567	https://www.inaturalist.org/observations/142757642
1110	Bubulcus ibis	Cattle Egret	2017/2/22	5	0	11.9971750	75.2618933	https://www.inaturalist.org/observations/30663948
1111	Bubulcus ibis	Cattle Egret	2018/10/28	3	0	12.0201921	75.4221501	https://www.inaturalist.org/observations/48519336
1112	Bubulcus ibis	Cattle Egret	2019/9/20	3	0	12.0276300	75.2640467	https://www.inaturalist.org/observations/33136183
1113	Bubulcus ibis	Cattle Egret	2021/9/9	3	0	12.0304062	75.2540228	https://www.inaturalist.org/observations/141774477
1114	Bubulcus ibis	Cattle Egret	2019/9/5	2	0	12.0313733	75.2525167	https://www.inaturalist.org/observations/32201046
1115	Bubulcus ibis	Cattle Egret	2013/5/10	1	0	12.0314216	76.1206821	https://www.inaturalist.org/observations/56002543
1116	Bubulcus ibis	Cattle Egret	2022/10/16	1	0	12.0323463	75.2566565	https://www.inaturalist.org/observations/141592821
1117	Bubulcus ibis	Cattle Egret	2019/10/15	2	0	12.0323467	75.2566564	https://www.inaturalist.org/observations/34573193
1118	Bubulcus ibis	Cattle Egret	2019/10/15	2	0	12.0323467	75.2566564	https://www.inaturalist.org/observations/34573194
1119	Bubulcus ibis	Cattle Egret	2019/10/15	2	0	12.0323467	75.2566564	https://www.inaturalist.org/observations/34573197
1120	Bubulcus ibis	Cattle Egret	2019/10/15	2	0	12.0323467	75.2566564	https://www.inaturalist.org/observations/34573198
1121	Bubulcus ibis	Cattle Egret	2019/10/15	2	0	12.0323467	75.2566564	https://www.inaturalist.org/observations/34573199
1122	Bubulcus ibis	Cattle Egret	2019/10/15	2	0	12.0323467	75.2566564	https://www.inaturalist.org/observations/34574173
1123	Bubulcus ibis	Cattle Egret	2019/10/15	4	0	12.0323467	75.2566564	https://www.inaturalist.org/observations/34574181
1124	Bubulcus ibis	Cattle Egret	2021/9/11	1	0	12.0323467	75.2566564	https://www.inaturalist.org/observations/94542588

1125	Bubulcus ibis	Cattle Egret	2022/6/1	3	0	12.0327400	75.2605217	https://www.inaturalist.org/observations/142805081
1126	Bubulcus ibis	Cattle Egret	2021/8/28	1	0	12.0328701	75.2575718	https://www.inaturalist.org/observations/93066428
1127	Bubulcus ibis	Cattle Egret	2019/5/9	4	0	12.0346900	75.2897750	https://www.inaturalist.org/observations/29242860
1128	Bubulcus ibis	Cattle Egret	2022/6/22	3	0	12.0372867	75.2580667	https://www.inaturalist.org/observations/126932748
1129	Bubulcus ibis	Cattle Egret	2020/4/10	1	0	12.0574917	75.2006266	https://www.inaturalist.org/observations/41837097
1130	Bubulcus ibis	Cattle Egret	2022/11/6	1	0	12.0659217	75.2122117	https://www.inaturalist.org/observations/141471657
1131	Bubulcus ibis	Cattle Egret	2021/6/7	2	0	12.0847065	75.2414651	https://www.inaturalist.org/observations/82178283
1132	Bubulcus ibis	Cattle Egret	2019/1/20	1	0	12.0941481	77.0211822	https://www.inaturalist.org/observations/97525617
1133	Bubulcus ibis	Cattle Egret	2018/6/14	4	0	12.1148946	75.8359685	https://www.inaturalist.org/observations/13451837
1134	Bubulcus ibis	Cattle Egret	2002/1/5	1	0	12.1307476	76.4186495	https://www.inaturalist.org/observations/91844738
1135	Bubulcus ibis	Cattle Egret	2014/7/27	3	0	12.1365023	76.6491264	https://www.inaturalist.org/observations/90007130
1136	Bubulcus ibis	Cattle Egret	2021/2/5	2	0	12.1398728	75.2196408	https://www.inaturalist.org/observations/69083606
1137	Bubulcus ibis	Cattle Egret	2021/2/1	1	0	12.1399275	75.2196584	https://www.inaturalist.org/observations/68907573
1138	Bubulcus ibis	Cattle Egret	2021/7/4	2	0	12.1494135	76.5574930	https://www.inaturalist.org/observations/85601233
1139	Bubulcus ibis	Cattle Egret	2022/4/10	2	0	12.1559500	75.1978417	https://www.inaturalist.org/observations/142799212
1140	Bubulcus ibis	Cattle Egret	2020/5/20	2	0	12.1608281	75.1632934	https://www.inaturalist.org/observations/142896958
1141	Bubulcus ibis	Cattle Egret	2020/5/28	2	0	12.1608281	75.1632934	https://www.inaturalist.org/observations/143564581
1142	Bubulcus ibis	Cattle Egret	2015/12/27	1	0	12.1642219	76.5425617	https://www.inaturalist.org/observations/85735948
1143	Bubulcus ibis	Cattle Egret	2022/11/15	2	0	12.1649444	75.1715556	https://www.inaturalist.org/observations/142039356
1144	Bubulcus ibis	Cattle Egret	2019/5/25	4	0	12.1661342	75.4605712	https://www.inaturalist.org/observations/32678296
1145	Bubulcus ibis	Cattle Egret	2014/7/6	2	0	12.1677012	76.5314817	https://www.inaturalist.org/observations/85732389
1146	Bubulcus ibis	Cattle Egret	2013/10/16	1	0	12.1677012	76.5314817	https://www.inaturalist.org/observations/85735534
1147	Bubulcus ibis	Cattle Egret	2020/9/27	1	0	12.1702110	76.5437370	https://www.inaturalist.org/observations/82525049
1148	Bubulcus ibis	Cattle Egret	2022/9/3	2	0	12.1800914	76.7419525	https://www.inaturalist.org/observations/133586747
1149	Bubulcus ibis	Cattle Egret	2022/12/18	1	0	12.1816982	77.7293856	https://www.inaturalist.org/observations/144598126

1150	Bubulcus ibis	Cattle Egret	2018/3/5	5	0	12.2006565	75.1665553	https://www.inaturalist.org/observations/18265228
1151	Bubulcus ibis	Cattle Egret	2022/8/17	2	0	12.2028258	75.8117695	https://www.inaturalist.org/observations/131851742
1152	Bubulcus ibis	Cattle Egret	2020/5/5	6	0	12.2124074	75.3552266	https://www.inaturalist.org/observations/83776470
1153	Bubulcus ibis	Cattle Egret	2021/6/10	1	0	12.2124735	76.6334254	https://www.inaturalist.org/observations/82395083
1154	Bubulcus ibis	Cattle Egret	2017/1/21	1	0	12.2124735	76.6334254	https://www.inaturalist.org/observations/88942324
1155	Bubulcus ibis	Cattle Egret	2019/8/22	1	0	12.2132993	76.6253001	https://www.inaturalist.org/observations/96557350
1156	Bubulcus ibis	Cattle Egret	2018/12/1	5	0	12.2148127	75.6579659	https://www.inaturalist.org/observations/19207966
1157	Bubulcus ibis	Cattle Egret	2017/11/16	3	0	12.2148127	75.6579659	https://www.inaturalist.org/observations/36726717
1158	Bubulcus ibis	Cattle Egret	2015/4/20	3	0	12.2227233	76.6272876	https://www.inaturalist.org/observations/142801522
1159	Bubulcus ibis	Cattle Egret	2014/10/4	2	0	12.2227233	76.6272876	https://www.inaturalist.org/observations/143348931
1160	Bubulcus ibis	Cattle Egret	2014/10/30	1	0	12.2227233	76.6272876	https://www.inaturalist.org/observations/143348937
1161	Bubulcus ibis	Cattle Egret	2014/11/9	1	0	12.2227233	76.6272876	https://www.inaturalist.org/observations/145242722
1162	Bubulcus ibis	Cattle Egret	2018/5/5	1	0	12.2252841	79.0746957	https://www.inaturalist.org/observations/96085182
1163	Bubulcus ibis	Cattle Egret	2022/5/29	3	0	12.2271678	76.6433448	https://www.inaturalist.org/observations/119198245
1164	Bubulcus ibis	Cattle Egret	2021/10/28	1	0	12.2409060	76.5979445	https://www.inaturalist.org/observations/99606928
1165	Bubulcus ibis	Cattle Egret	2020/12/20	2	0	12.2517833	75.2770317	https://www.inaturalist.org/observations/66994048
1166	Bubulcus ibis	Cattle Egret	2014/5/10	2	0	12.2555440	76.6118253	https://www.inaturalist.org/observations/94567357
1167	Bubulcus ibis	Cattle Egret	2011/4/14	2	0	12.2555440	76.6118253	https://www.inaturalist.org/observations/94571717
1168	Bubulcus ibis	Cattle Egret	2022/10/27	2	0	12.2556866	79.0302825	https://www.inaturalist.org/observations/140367932
1169	Bubulcus ibis	Cattle Egret	2022/11/5	1	0	12.2571479	78.8049910	https://www.inaturalist.org/observations/141546395
1170	Bubulcus ibis	Cattle Egret	2018/4/15	1	0	12.2693720	76.6119780	https://www.inaturalist.org/observations/94104163
1171	Bubulcus ibis	Cattle Egret	2012/11/11	2	0	12.2693720	76.6119780	https://www.inaturalist.org/observations/94706635
1172	Bubulcus ibis	Cattle Egret	2019/1/3	5	0	12.2703717	76.6091100	https://www.inaturalist.org/observations/19465631
1173	Bubulcus ibis	Cattle Egret	2013/11/26	1	0	12.2704249	76.7756307	https://www.inaturalist.org/observations/90401803
1174	Bubulcus ibis	Cattle Egret	2018/10/23	1	0	12.2752546	76.6701475	https://www.inaturalist.org/observations/88663075

1175	Bubulcus ibis	Cattle Egret	2020/2/3	2	0	12.2759998	76.8823880	https://www.inaturalist.org/observations/65747871
1176	Bubulcus ibis	Cattle Egret	2018/3/7	5	0	12.2822492	76.6161594	https://www.inaturalist.org/observations/10125148
1177	Bubulcus ibis	Cattle Egret	2018/2/19	4	0	12.2828660	76.6154766	https://www.inaturalist.org/observations/9931351
1178	Bubulcus ibis	Cattle Egret	2018/5/17	4	0	12.2830983	76.6149050	https://www.inaturalist.org/observations/12705985
1179	Bubulcus ibis	Cattle Egret	2018/5/12	4	0	12.2831067	76.6149033	https://www.inaturalist.org/observations/12443532
1180	Bubulcus ibis	Cattle Egret	2018/3/23	4	0	12.2832170	76.6148742	https://www.inaturalist.org/observations/10374289
1181	Bubulcus ibis	Cattle Egret	2018/3/22	5	0	12.2834405	76.6172791	https://www.inaturalist.org/observations/10357628
1182	Bubulcus ibis	Cattle Egret	2021/2/20	1	0	12.2841661	75.1602206	https://www.inaturalist.org/observations/69845484
1183	Bubulcus ibis	Cattle Egret	2021/11/14	0	0	12.2855820	76.5676071	https://www.inaturalist.org/observations/101074497
1184	Bubulcus ibis	Cattle Egret	2021/10/27	2	0	12.2942421	76.5653969	https://www.inaturalist.org/observations/99523415
1185	Bubulcus ibis	Cattle Egret	2022/3/4	4	0	12.2957999	76.6393805	https://www.inaturalist.org/observations/107886907
1186	Bubulcus ibis	Cattle Egret	2022/8/27	2	0	12.3092189	76.5556046	https://www.inaturalist.org/observations/132523169
1187	Bubulcus ibis	Cattle Egret	2022/10/23	1	0	12.3103953	76.6304409	https://www.inaturalist.org/observations/139962780
1188	Bubulcus ibis	Cattle Egret	2022/10/25	4	0	12.3103953	76.6304409	https://www.inaturalist.org/observations/140118579
1189	Bubulcus ibis	Cattle Egret	2018/2/18	2	0	12.3220310	75.8901176	https://www.inaturalist.org/observations/37518098
1190	Bubulcus ibis	Cattle Egret	2020/12/20	2	0	12.3474150	75.0893617	https://www.inaturalist.org/observations/66994794
1191	Bubulcus ibis	Cattle Egret	2020/6/15	2	0	12.3502010	76.5125120	https://www.inaturalist.org/observations/50019886
1192	Bubulcus ibis	Cattle Egret	2020/12/20	2	0	12.3516767	75.0940450	https://www.inaturalist.org/observations/66994780
1193	Bubulcus ibis	Cattle Egret	2019/1/22	1	0	12.3580693	76.6100719	https://www.inaturalist.org/observations/91848141
1194	Bubulcus ibis	Cattle Egret	2020/1/22	4	0	12.3679149	76.4807923	https://www.inaturalist.org/observations/38319695
1195	Bubulcus ibis	Cattle Egret	2021/10/29	2	0	12.4138944	75.7543102	https://www.inaturalist.org/observations/102531024
1196	Bubulcus ibis	Cattle Egret	2022/11/4	1	0	12.4157590	75.0450088	https://www.inaturalist.org/observations/141058585
1197	Bubulcus ibis	Cattle Egret	2017/9/13	1	0	12.4200282	76.6227068	https://www.inaturalist.org/observations/79896999
1198	Bubulcus ibis	Cattle Egret	2018/4/16	4	0	12.4237099	76.6540146	https://www.inaturalist.org/observations/13018957
1199	Bubulcus ibis	Cattle Egret	2018/4/16	3	0	12.4237099	76.6540146	https://www.inaturalist.org/observations/13018972

1200	Bubulcus ibis	Cattle Egret	2022/6/2	4	0	12.4239250	76.6547895	https://www.inaturalist.org/observations/120474031
1201	Bubulcus ibis	Cattle Egret	2022/6/25	3	0	12.4243205	76.6539623	https://www.inaturalist.org/observations/123363909
1202	Bubulcus ibis	Cattle Egret	2015/5/1	2	0	12.4243603	76.6563491	https://www.inaturalist.org/observations/105419538
1203	Bubulcus ibis	Cattle Egret	2010/5/9	1	0	12.4243603	76.6563492	https://www.inaturalist.org/observations/52821415
1204	Bubulcus ibis	Cattle Egret	2009/5/9	1	0	12.4243603	76.6563492	https://www.inaturalist.org/observations/55132011
1205	Bubulcus ibis	Cattle Egret	2019/10/6	1	0	12.4243678	76.6563491	https://www.inaturalist.org/observations/37087743
1206	Bubulcus ibis	Cattle Egret	2020/11/10	1	0	12.4253452	76.6561346	https://www.inaturalist.org/observations/96065697
1207	Bubulcus ibis	Cattle Egret	2021/11/23	2	0	12.4312957	75.7630562	https://www.inaturalist.org/observations/128754079
1208	Bubulcus ibis	Cattle Egret	2019/12/25	1	0	12.4349608	76.2400175	https://www.inaturalist.org/observations/92361086
1209	Bubulcus ibis	Cattle Egret	2021/11/16	2	0	12.4376023	75.6671417	https://www.inaturalist.org/observations/102292463
1210	Bubulcus ibis coromandus	Cattle Egret	2012/5/22	3	0	12.4410379	75.7948096	https://www.inaturalist.org/observations/36663873
1211	Bubulcus ibis	Cattle Egret	2011/7/23	1	0	12.4440489	76.4592855	https://www.inaturalist.org/observations/93239520
1212	Bubulcus ibis	Cattle Egret	2011/12/11	1	0	12.4676385	76.1001646	https://www.inaturalist.org/observations/84952253
1213	Bubulcus ibis coromandus	Cattle Egret	2021/11/5	2	0	12.4746265	78.1860066	https://www.inaturalist.org/observations/100312353
1214	Bubulcus ibis	Cattle Egret	2020/12/29	1	0	12.5113813	76.9903743	https://www.inaturalist.org/observations/96586149
1215	Bubulcus ibis	Cattle Egret	2017/2/11	2	0	12.5435660	79.8551078	https://www.inaturalist.org/observations/67508382
1216	Bubulcus ibis	Cattle Egret	2019/12/28	2	0	12.5454597	79.8560692	https://www.inaturalist.org/observations/48641524
1217	Bubulcus ibis	Cattle Egret	2022/12/25	2	0	12.5454597	79.8560692	https://www.inaturalist.org/observations/145048383
1218	Bubulcus ibis	Cattle Egret	2020/3/4	1	0	12.5454600	79.8560690	https://www.inaturalist.org/observations/89996440
1219	Bubulcus ibis	Cattle Egret	2022/1/5	3	0	12.5472833	79.8555586	https://www.inaturalist.org/observations/104403179
1220	Bubulcus ibis	Cattle Egret	2012/4/7	3	0	12.5798634	78.9029503	https://www.inaturalist.org/observations/89039248
1221	Bubulcus ibis	Cattle Egret	2017/2/11	3	0	12.5879709	79.8400503	https://www.inaturalist.org/observations/67458900
1222	Bubulcus ibis	Cattle Egret	2020/3/15	2	0	12.6124176	75.2078706	https://www.inaturalist.org/observations/62101633
1223	Bubulcus ibis	Cattle Egret	2022/6/21	6	0	12.6135233	76.0843750	https://www.inaturalist.org/observations/124086976
1224	Bubulcus ibis	Cattle Egret	2022/5/7	1	0	12.6360697	77.9794772	https://www.inaturalist.org/observations/119229953

1225	Bubulcus ibis	Cattle Egret	2020/11/14	1	0	12.6397621	80.2034449	https://www.inaturalist.org/observations/89428226
1226	Bubulcus ibis	Cattle Egret	2022/1/14	2	0	12.6558486	77.9677951	https://www.inaturalist.org/observations/107474614
1227	Bubulcus ibis	Cattle Egret	2022/1/19	2	0	12.6588053	79.8052360	https://www.inaturalist.org/observations/105613340
1228	Bubulcus ibis	Cattle Egret	2022/11/23	0	0	12.6747923	77.7654114	https://www.inaturalist.org/observations/142656455
1229	Bubulcus ibis	Cattle Egret	2022/11/26	2	0	12.6749917	77.7667883	https://www.inaturalist.org/observations/142900254
1230	Bubulcus ibis	Cattle Egret	2022/11/23	1	0	12.6751680	77.7653122	https://www.inaturalist.org/observations/142652796
1231	Bubulcus ibis	Cattle Egret	2022/11/26	2	0	12.6759167	77.7657700	https://www.inaturalist.org/observations/142894112
1232	Bubulcus ibis	Cattle Egret	2022/12/17	1	0	12.6781267	77.9266933	https://www.inaturalist.org/observations/144533436
1233	Bubulcus ibis	Cattle Egret	2022/12/24	1	0	12.6782167	77.9266117	https://www.inaturalist.org/observations/144981624
1234	Bubulcus ibis	Cattle Egret	2022/12/3	4	0	12.6785233	77.9257717	https://www.inaturalist.org/observations/143552653
1235	Bubulcus ibis	Cattle Egret	2022/2/20	1	0	12.6864903	77.6653652	https://www.inaturalist.org/observations/107473260
1236	Bubulcus ibis	Cattle Egret	2021/1/6	2	0	12.6961839	75.1097271	https://www.inaturalist.org/observations/67691004
1237	Bubulcus ibis	Cattle Egret	2022/1/9	1	0	12.7138497	77.8143883	https://www.inaturalist.org/observations/106980821
1238	Bubulcus ibis	Cattle Egret	2022/1/15	1	0	12.7138497	77.8143883	https://www.inaturalist.org/observations/106980827
1239	Bubulcus ibis	Cattle Egret	2022/1/26	1	0	12.7138497	77.8143883	https://www.inaturalist.org/observations/106980849
1240	Bubulcus ibis	Cattle Egret	2022/11/19	2	0	12.7138617	77.8144217	https://www.inaturalist.org/observations/142332117
1241	Bubulcus ibis	Cattle Egret	2021/12/19	1	0	12.7139035	77.8142702	https://www.inaturalist.org/observations/103307760
1242	Bubulcus ibis	Cattle Egret	2022/11/9	2	0	12.7139816	77.8135875	https://www.inaturalist.org/observations/141500930
1243	Bubulcus ibis	Cattle Egret	2015/4/4	3	0	12.7204878	77.5607733	https://www.inaturalist.org/observations/52037630
1244	Bubulcus ibis	Cattle Egret	2015/4/4	2	0	12.7204878	77.5607733	https://www.inaturalist.org/observations/52037641
1245	Bubulcus ibis	Cattle Egret	2022/10/30	1	0	12.7322332	79.0565819	https://www.inaturalist.org/observations/140868381
1246	Bubulcus ibis	Cattle Egret	2022/12/1	2	0	12.7326633	77.7900267	https://www.inaturalist.org/observations/143419649
1247	Bubulcus ibis	Cattle Egret	2022/4/10	2	0	12.7327741	77.7897038	https://www.inaturalist.org/observations/139409023
1248	Bubulcus ibis	Cattle Egret	2022/11/20	1	0	12.7395005	77.7864153	https://www.inaturalist.org/observations/142420722
1249	Bubulcus ibis	Cattle Egret	2022/2/6	1	0	12.7419889	77.5427487	https://www.inaturalist.org/observations/106232942

1250	Bubulcus ibis	Cattle Egret	2021/12/11	2	0	12.7450165	80.2053814	https://www.inaturalist.org/observations/102855885
1251	Bubulcus ibis	Cattle Egret	2022/2/6	1	0	12.7477105	77.4978000	https://www.inaturalist.org/observations/107039683
1252	Bubulcus ibis	Cattle Egret	2019/2/16	3	0	12.7513671	80.1889300	https://www.inaturalist.org/observations/22558661
1253	Bubulcus ibis	Cattle Egret	2020/12/31	3	0	12.7532017	77.3013674	https://www.inaturalist.org/observations/67378487
1254	Bubulcus ibis	Cattle Egret	2022/2/6	1	0	12.7547156	77.5309263	https://www.inaturalist.org/observations/106231873
1255	Bubulcus ibis	Cattle Egret	2020/11/9	0	0	12.7616258	75.2032168	https://www.inaturalist.org/observations/64708042
1256	Bubulcus ibis	Cattle Egret	2022/11/13	3	0	12.7667767	77.8669417	https://www.inaturalist.org/observations/141869126
1257	Bubulcus ibis	Cattle Egret	2022/5/1	1	0	12.7673560	77.8739691	https://www.inaturalist.org/observations/114131773
1258	Bubulcus ibis	Cattle Egret	2022/5/1	1	0	12.7673560	77.8739691	https://www.inaturalist.org/observations/114131825
1259	Bubulcus ibis	Cattle Egret	2022/5/1	1	0	12.7673560	77.8739691	https://www.inaturalist.org/observations/114131826
1260	Bubulcus ibis	Cattle Egret	2022/5/1	1	0	12.7673560	77.8739691	https://www.inaturalist.org/observations/114131837
1261	Bubulcus ibis	Cattle Egret	2021/12/12	1	0	12.7676155	77.8680224	https://www.inaturalist.org/observations/102921114
1262	Bubulcus ibis	Cattle Egret	2022/5/1	1	0	12.7684467	77.8821050	https://www.inaturalist.org/observations/114411245
1263	Bubulcus ibis	Cattle Egret	2022/5/1	1	0	12.7727850	77.8761533	https://www.inaturalist.org/observations/114395110
1264	Bubulcus ibis	Cattle Egret	2022/11/12	2	0	12.7735767	77.6177417	https://www.inaturalist.org/observations/141774072
1265	Bubulcus ibis	Cattle Egret	2016/5/7	6	0	12.7743726	77.6592099	https://www.inaturalist.org/observations/126172696
1266	Bubulcus ibis	Cattle Egret	2022/2/27	2	0	12.7779250	77.8612167	https://www.inaturalist.org/observations/107552795
1267	Bubulcus ibis	Cattle Egret	2022/5/6	5	0	12.7834509	77.8106894	https://www.inaturalist.org/observations/119229936
1268	Bubulcus ibis	Cattle Egret	2022/5/1	0	0	12.7838645	77.8927863	https://www.inaturalist.org/observations/114136746
1269	Bubulcus ibis	Cattle Egret	2021/4/9	1	0	12.7907739	78.3644940	https://www.inaturalist.org/observations/74040052
1270	Bubulcus ibis	Cattle Egret	2022/2/9	1	0	12.8071169	77.5320287	https://www.inaturalist.org/observations/106439345
1271	Bubulcus ibis	Cattle Egret	2022/2/9	3	0	12.8071518	77.5309799	https://www.inaturalist.org/observations/106445494
1272	Bubulcus ibis	Cattle Egret	2022/2/9	3	0	12.8081598	77.5301437	https://www.inaturalist.org/observations/106446026
1273	Bubulcus ibis	Cattle Egret	2021/12/31	1	0	12.8138533	76.9460964	https://www.inaturalist.org/observations/104182932
1274	Bubulcus ibis	Cattle Egret	2022/1/14	3	0	12.8225173	77.7294705	https://www.inaturalist.org/observations/104914940

1275	Bubulcus ibis	Cattle Egret	2020/12/24	1	0	12.8419594	80.1738604	https://www.inaturalist.org/observations/67620826
1276	Bubulcus ibis coromandus	Cattle Egret	2016/2/21	3	0	12.8420533	77.5891983	http://www.inaturalist.org/observations/2727129
1277	Bubulcus ibis	Cattle Egret	2020/1/23	0	0	12.8421681	77.7060194	https://www.inaturalist.org/observations/37863307
1278	Bubulcus ibis coromandus	Cattle Egret	2022/1/5	1	0	12.8427500	77.7244630	https://www.inaturalist.org/observations/109221759
1279	Bubulcus ibis	Cattle Egret	2021/11/21	1	0	12.8430343	80.1786427	https://www.inaturalist.org/observations/101611072
1280	Bubulcus ibis	Cattle Egret	2021/11/21	2	0	12.8430343	80.1786427	https://www.inaturalist.org/observations/101611075
1281	Bubulcus ibis	Cattle Egret	2021/12/17	3	0	12.8457562	77.7754195	https://www.inaturalist.org/observations/105364669
1282	Bubulcus ibis	Cattle Egret	2020/12/20	2	0	12.8571865	77.5089110	https://www.inaturalist.org/observations/66816598
1283	Bubulcus ibis	Cattle Egret	2021/3/7	1	0	12.8571865	77.5089110	https://www.inaturalist.org/observations/70743665
1284	Bubulcus ibis	Cattle Egret	2021/3/21	2	0	12.8571865	77.5089110	https://www.inaturalist.org/observations/71692543
1285	Bubulcus ibis	Cattle Egret	2021/10/31	2	0	12.8575976	77.7864057	https://www.inaturalist.org/observations/99865167
1286	Bubulcus ibis	Cattle Egret	2022/2/20	2	0	12.8575976	77.7864057	https://www.inaturalist.org/observations/107361839
1287	Bubulcus ibis	Cattle Egret	2019/11/10	1	0	12.8655653	77.5217171	https://www.inaturalist.org/observations/55132012
1288	Bubulcus ibis	Cattle Egret	2020/11/8	2	0	12.8658517	77.5682383	https://www.inaturalist.org/observations/64536356
1289	Bubulcus ibis	Cattle Egret	2020/9/26	2	0	12.8687750	77.6441266	https://www.inaturalist.org/observations/60812489
1290	Bubulcus ibis	Cattle Egret	2020/10/25	3	0	12.8717417	77.5797417	https://www.inaturalist.org/observations/63558045
1291	Bubulcus ibis	Cattle Egret	2021/1/1	2	0	12.8726501	77.5151777	https://www.inaturalist.org/observations/67399747
1292	Bubulcus ibis	Cattle Egret	2021/12/21	1	0	12.8741482	75.7644532	https://www.inaturalist.org/observations/103677714
1293	Bubulcus ibis	Cattle Egret	2021/12/22	1	0	12.8741482	75.7644532	https://www.inaturalist.org/observations/103679927
1294	Bubulcus ibis	Cattle Egret	2020/1/26	2	0	12.8745759	77.6307832	https://www.inaturalist.org/observations/61770247
1295	Bubulcus ibis	Cattle Egret	2020/12/20	3	0	12.8749875	77.6299856	https://www.inaturalist.org/observations/66818076
1296	Bubulcus ibis	Cattle Egret	2019/12/14	2	0	12.8807814	77.6382566	https://www.inaturalist.org/observations/36637554
1297	Bubulcus ibis	Cattle Egret	2021/4/13	3	0	12.8822217	77.4623467	https://www.inaturalist.org/observations/73831371
1298	Bubulcus ibis	Cattle Egret	2022/4/14	2	0	12.8858591	80.2335255	https://www.inaturalist.org/observations/111393388
1299	Bubulcus ibis	Cattle Egret	2018/12/30	2	0	12.8904140	77.5871900	https://www.inaturalist.org/observations/86154917

1300	Bubulcus ibis	Cattle Egret	2021/4/17	1	0	12.8905000	77.5640000	https://www.inaturalist.org/observations/80296052
1301	Bubulcus ibis	Cattle Egret	2019/3/5	2	0	12.8935419	80.2231306	https://www.inaturalist.org/observations/65623252
1302	Bubulcus ibis	Cattle Egret	2021/3/29	2	0	12.8951482	77.6625845	https://www.inaturalist.org/observations/72523889
1303	Bubulcus ibis	Cattle Egret	2021/4/20	1	0	12.8957306	80.2353222	https://www.inaturalist.org/observations/74683888
1304	Bubulcus ibis	Cattle Egret	2022/1/18	2	0	12.8961819	77.8145154	https://www.inaturalist.org/observations/137707607
1305	Bubulcus ibis	Cattle Egret	2021/4/23	1	0	12.8966167	80.2356806	https://www.inaturalist.org/observations/74911697
1306	Bubulcus ibis	Cattle Egret	2020/12/6	2	0	12.8991793	77.6669909	https://www.inaturalist.org/observations/67082905
1307	Bubulcus ibis	Cattle Egret	2022/4/15	4	0	12.8993628	80.2209489	https://www.inaturalist.org/observations/111614114
1308	Bubulcus ibis	Cattle Egret	2020/2/22	0	0	12.8998320	77.5409830	https://www.inaturalist.org/observations/39250640
1309	Bubulcus ibis	Cattle Egret	2022/1/29	1	0	12.9012250	80.2219209	https://www.inaturalist.org/observations/105818886
1310	Bubulcus ibis	Cattle Egret	2022/1/29	1	0	12.9012250	80.2219209	https://www.inaturalist.org/observations/105819601
1311	Bubulcus ibis	Cattle Egret	2021/1/17	2	0	12.9015717	80.2182350	https://www.inaturalist.org/observations/103082792
1312	Bubulcus ibis	Cattle Egret	2010/6/2	4	0	12.9046136	77.6149480	https://www.inaturalist.org/observations/28845427
1313	Bubulcus ibis	Cattle Egret	2020/7/6	1	0	12.9064508	77.6771124	https://www.inaturalist.org/observations/52228202
1314	Bubulcus ibis	Cattle Egret	2021/1/14	1	0	12.9108315	79.4011488	https://www.inaturalist.org/observations/82160900
1315	Bubulcus ibis	Cattle Egret	2022/1/23	3	0	12.9114730	77.6161230	https://www.inaturalist.org/observations/106435820
1316	Bubulcus ibis	Cattle Egret	2020/11/9	1	0	12.9124005	76.9362747	https://www.inaturalist.org/observations/97142376
1317	Bubulcus ibis	Cattle Egret	2021/10/29	1	0	12.9124005	76.9362747	https://www.inaturalist.org/observations/99771029
1318	Bubulcus ibis	Cattle Egret	2022/1/15	2	0	12.9132740	80.2293710	https://www.inaturalist.org/observations/105157960
1319	Bubulcus ibis	Cattle Egret	2022/1/18	2	0	12.9132740	80.2293710	https://www.inaturalist.org/observations/105437462
1320	Bubulcus ibis	Cattle Egret	2021/4/5	5	0	12.9137650	77.5190040	https://www.inaturalist.org/observations/108492919
1321	Bubulcus ibis	Cattle Egret	2021/11/2	1	0	12.9150945	77.4696980	https://www.inaturalist.org/observations/100161692
1322	Bubulcus ibis	Cattle Egret	2021/11/2	1	0	12.9150945	77.4696980	https://www.inaturalist.org/observations/100161693
1323	Bubulcus ibis	Cattle Egret	2022/4/2	2	0	12.9150945	77.4696980	https://www.inaturalist.org/observations/110268214
1324	Bubulcus ibis	Cattle Egret	2019/12/9	1	0	12.9153773	79.4483482	https://www.inaturalist.org/observations/134421759

1325	Bubulcus ibis	Cattle Egret	2020/4/18	2	0	12.9163766	77.6642086	https://www.inaturalist.org/observations/52782022
1326	Bubulcus ibis	Cattle Egret	2021/4/17	2	0	12.9169633	77.6471550	https://www.inaturalist.org/observations/80872179
1327	Bubulcus ibis	Cattle Egret	2022/3/5	2	0	12.9174276	80.2093854	https://www.inaturalist.org/observations/142077165
1328	Bubulcus ibis	Cattle Egret	2022/1/15	2	0	12.9180070	80.2256500	https://www.inaturalist.org/observations/105023838
1329	Bubulcus ibis	Cattle Egret	2022/4/15	2	0	12.9195652	77.6797409	https://www.inaturalist.org/observations/112497466
1330	Bubulcus ibis	Cattle Egret	2019/2/2	3	0	12.9348912	80.2137124	https://www.inaturalist.org/observations/64436336
1331	Bubulcus ibis	Cattle Egret	2020/5/10	2	0	12.9348913	80.2137123	https://www.inaturalist.org/observations/45508369
1332	Bubulcus ibis	Cattle Egret	2020/4/18	1	0	12.9375139	80.2054434	https://www.inaturalist.org/observations/44089230
1333	Bubulcus ibis	Cattle Egret	2019/11/26	4	0	12.9399258	80.2164549	https://www.inaturalist.org/observations/36216723
1334	Bubulcus ibis	Cattle Egret	2019/5/19	5	0	12.9427130	80.2107951	https://www.inaturalist.org/observations/25497555
1335	Bubulcus ibis	Cattle Egret	2022/3/5	3	0	12.9459686	80.2588535	https://www.inaturalist.org/observations/142077168
1336	Bubulcus ibis	Cattle Egret	2022/11/13	2	0	12.9462212	77.6487498	https://www.inaturalist.org/observations/142065980
1337	Bubulcus ibis	Cattle Egret	2015/4/16	4	0	12.9465761	80.2237660	https://www.inaturalist.org/observations/22597479
1338	Bubulcus ibis	Cattle Egret	2013/9/20	4	0	12.9465761	80.2237660	https://www.inaturalist.org/observations/22602984
1339	Bubulcus ibis	Cattle Egret	2013/11/1	4	0	12.9465761	80.2237660	https://www.inaturalist.org/observations/63147480
1340	Bubulcus ibis	Cattle Egret	2020/12/29	1	0	12.9465761	80.2237660	https://www.inaturalist.org/observations/83231601
1341	Bubulcus ibis	Cattle Egret	2018/9/23	1	0	12.9465761	80.2237660	https://www.inaturalist.org/observations/88817272
1342	Bubulcus ibis	Cattle Egret	2022/1/29	2	0	12.9465761	80.2237660	https://www.inaturalist.org/observations/106113747
1343	Bubulcus ibis	Cattle Egret	2020/2/14	0	0	12.9492500	77.5116800	https://www.inaturalist.org/observations/39019306
1344	Bubulcus ibis	Cattle Egret	2018/1/1	4	0	12.9494800	80.2216900	https://www.inaturalist.org/observations/22522551
1345	Bubulcus ibis	Cattle Egret	2019/1/1	4	0	12.9498297	74.8565613	https://www.inaturalist.org/observations/19397003
1346	Bubulcus ibis	Cattle Egret	2022/12/4	2	0	12.9580590	80.1519592	https://www.inaturalist.org/observations/143774062
1347	Bubulcus ibis	Cattle Egret	2022/11/16	3	0	12.9582659	77.7255356	https://www.inaturalist.org/observations/142153652
1348	Bubulcus ibis	Cattle Egret	2022/11/26	2	0	12.9582659	77.7255356	https://www.inaturalist.org/observations/143011922
1349	Bubulcus ibis	Cattle Egret	2022/11/29	2	0	12.9582659	77.7255356	https://www.inaturalist.org/observations/143706448

1350	Bubulcus ibis	Cattle Egret	2022/10/15	1	0	12.9582660	77.7255357	https://www.inaturalist.org/observations/138914853
1351	Bubulcus ibis	Cattle Egret	2022/10/17	4	0	12.9582660	77.7255357	https://www.inaturalist.org/observations/139120353
1352	Bubulcus ibis	Cattle Egret	2022/10/18	3	0	12.9582660	77.7255357	https://www.inaturalist.org/observations/139230764
1353	Bubulcus ibis	Cattle Egret	2022/10/27	3	0	12.9582660	77.7255357	https://www.inaturalist.org/observations/140244117
1354	Bubulcus ibis	Cattle Egret	2022/10/28	3	0	12.9582660	77.7255357	https://www.inaturalist.org/observations/140250763
1355	Bubulcus ibis	Cattle Egret	2022/11/21	4	0	12.9582660	77.7255357	https://www.inaturalist.org/observations/142583450
1356	Bubulcus ibis	Cattle Egret	2020/9/30	2	0	12.9609817	77.7083641	https://www.inaturalist.org/observations/61385247
1357	Bubulcus ibis	Cattle Egret	2022/5/20	2	0	12.9640703	80.2036849	https://www.inaturalist.org/observations/118326713
1358	Bubulcus ibis	Cattle Egret	2022/11/26	2	0	12.9658592	77.7069332	https://www.inaturalist.org/observations/143011529
1359	Bubulcus ibis	Cattle Egret	2021/11/21	2	0	12.9672627	74.8471248	https://www.inaturalist.org/observations/101761276
1360	Bubulcus ibis	Cattle Egret	2022/12/28	2	0	12.9714785	77.5945627	https://www.inaturalist.org/observations/145215793
1361	Bubulcus ibis	Cattle Egret	2019/11/15	1	0	12.9715987	77.5945627	https://www.inaturalist.org/observations/55132009
1362	Bubulcus ibis	Cattle Egret	2019/11/3	1	0	12.9715987	77.5945627	https://www.inaturalist.org/observations/55895079
1363	Bubulcus ibis	Cattle Egret	2021/9/19	3	0	12.9715987	77.5945627	https://www.inaturalist.org/observations/121977400
1364	Bubulcus ibis	Cattle Egret	2015/5/15	4	0	12.9719400	77.5936900	http://www.inaturalist.org/observations/1501121
1365	Bubulcus ibis	Cattle Egret	2015/5/15	4	0	12.9719400	77.5936900	http://www.inaturalist.org/observations/1501135
1366	Bubulcus ibis	Cattle Egret	2021/7/3	3	0	12.9721614	77.8659858	https://www.inaturalist.org/observations/85419135
1367	Bubulcus ibis	Cattle Egret	2021/1/1	3	0	12.9722790	77.5945627	https://www.inaturalist.org/observations/67504351
1368	Bubulcus ibis	Cattle Egret	2021/1/1	4	0	12.9722790	77.5945627	https://www.inaturalist.org/observations/70490438
1369	Bubulcus ibis	Cattle Egret	2018/5/7	5	0	12.9738583	77.6860967	https://www.inaturalist.org/observations/12219734
1370	Bubulcus ibis	Cattle Egret	2022/1/1	1	0	12.9739550	75.8681981	https://www.inaturalist.org/observations/104150576
1371	Bubulcus ibis	Cattle Egret	2020/12/4	2	0	12.9759467	77.6912783	https://www.inaturalist.org/observations/66177270
1372	Bubulcus ibis	Cattle Egret	2020/10/28	1	0	12.9760300	77.6911467	https://www.inaturalist.org/observations/63684634
1373	Bubulcus ibis	Cattle Egret	2019/10/12	2	0	12.9781300	77.6857300	https://www.inaturalist.org/observations/34235634
1374	Bubulcus ibis	Cattle Egret	2020/2/15	0	0	12.9784867	77.6881417	https://www.inaturalist.org/observations/38690960

1375	Bubulcus ibis	Cattle Egret	2018/10/25	4	0	12.9825445	77.6196229	https://www.inaturalist.org/observations/17844290
1376	Bubulcus ibis	Cattle Egret	2021/1/11	1	0	12.9843944	80.1896241	https://www.inaturalist.org/observations/67915320
1377	Bubulcus ibis	Cattle Egret	2020/11/5	3	0	12.9895811	77.4896404	https://www.inaturalist.org/observations/64226392
1378	Bubulcus ibis	Cattle Egret	2022/1/6	3	0	12.9907664	80.2376866	https://www.inaturalist.org/observations/104467528
1379	Bubulcus ibis	Cattle Egret	2022/1/28	1	0	12.9922535	80.1901478	https://www.inaturalist.org/observations/105733760
1380	Bubulcus ibis	Cattle Egret	2022/1/27	1	0	12.9926926	80.1904348	https://www.inaturalist.org/observations/105678046
1381	Bubulcus ibis	Cattle Egret	2014/2/18	6	0	12.9978673	80.0958252	http://www.inaturalist.org/observations/1495587
1382	Bubulcus ibis	Cattle Egret	2021/3/9	5	0	13.0075100	77.5108300	https://www.inaturalist.org/observations/108705150
1383	Bubulcus ibis	Cattle Egret	2020/12/18	2	0	13.0078522	80.1925295	https://www.inaturalist.org/observations/93231328
1384	Bubulcus ibis	Cattle Egret	2019/1/17	2	0	13.0111830	80.2755925	https://www.inaturalist.org/observations/109822324
1385	Bubulcus ibis	Cattle Egret	2022/4/9	3	0	13.0128872	80.2529269	https://www.inaturalist.org/observations/112546321
1386	Bubulcus ibis	Cattle Egret	2022/4/9	3	0	13.0128872	80.2529269	https://www.inaturalist.org/observations/112546326
1387	Bubulcus ibis	Cattle Egret	2022/4/9	3	0	13.0128872	80.2529269	https://www.inaturalist.org/observations/112546327
1388	Bubulcus ibis	Cattle Egret	2022/4/9	3	0	13.0128872	80.2529269	https://www.inaturalist.org/observations/112546329
1389	Bubulcus ibis	Cattle Egret	2022/12/3	2	0	13.0142867	77.6833237	https://www.inaturalist.org/observations/143712467
1390	Bubulcus ibis	Cattle Egret	2022/12/3	3	0	13.0142867	77.6833237	https://www.inaturalist.org/observations/143713955
1391	Bubulcus ibis	Cattle Egret	2021/10/27	2	0	13.0156222	77.5686950	https://www.inaturalist.org/observations/99767950
1392	Bubulcus ibis	Cattle Egret	2020/2/6	5	0	13.0167102	80.2363903	https://www.inaturalist.org/observations/109841407
1393	Bubulcus ibis	Cattle Egret	2019/1/13	3	0	13.0170655	75.6405138	https://www.inaturalist.org/observations/63629852
1394	Bubulcus ibis	Cattle Egret	2022/12/3	0	0	13.0191915	80.2646894	https://www.inaturalist.org/observations/143732951
1395	Bubulcus ibis	Cattle Egret	2021/10/21	1	0	13.0197095	80.1984177	https://www.inaturalist.org/observations/98856349
1396	Bubulcus ibis	Cattle Egret	2015/3/15	6	0	13.0279640	77.5688816	https://www.inaturalist.org/observations/20845974
1397	Bubulcus ibis	Cattle Egret	2021/2/8	1	0	13.0304639	77.5733306	https://www.inaturalist.org/observations/69236930
1398	Bubulcus ibis	Cattle Egret	2021/12/18	4	0	13.0390545	80.1419356	https://www.inaturalist.org/observations/103514536
1399	Bubulcus ibis	Cattle Egret	2021/12/8	2	0	13.0399610	77.6312850	https://www.inaturalist.org/observations/102707546

1400	Bubulcus ibis	Cattle Egret	2022/10/29	2	0	13.0459063	80.2532708	https://www.inaturalist.org/observations/140487631
1401	Bubulcus ibis	Cattle Egret	2022/10/29	2	0	13.0459063	80.2532708	https://www.inaturalist.org/observations/140487634
1402	Bubulcus ibis	Cattle Egret	2022/12/19	1	0	13.0459063	80.2532708	https://www.inaturalist.org/observations/144900897
1403	Bubulcus ibis	Cattle Egret	2022/12/19	1	0	13.0459063	80.2532708	https://www.inaturalist.org/observations/144900899
1404	Bubulcus ibis	Cattle Egret	2022/12/21	2	0	13.0459063	80.2532708	https://www.inaturalist.org/observations/145095171
1405	Bubulcus ibis	Cattle Egret	2022/11/15	1	0	13.0459110	80.2532705	https://www.inaturalist.org/observations/142298255
1406	Bubulcus ibis	Cattle Egret	2021/3/17	1	0	13.0463405	80.2533557	https://www.inaturalist.org/observations/90664150
1407	Bubulcus ibis	Cattle Egret	2021/11/16	1	0	13.0463405	80.2533557	https://www.inaturalist.org/observations/101320539
1408	Bubulcus ibis	Cattle Egret	2021/11/16	1	0	13.0463405	80.2533557	https://www.inaturalist.org/observations/101320555
1409	Bubulcus ibis	Cattle Egret	2021/11/16	1	0	13.0463405	80.2533557	https://www.inaturalist.org/observations/101320614
1410	Bubulcus ibis	Cattle Egret	2021/11/16	2	0	13.0463405	80.2533557	https://www.inaturalist.org/observations/101320658
1411	Bubulcus ibis	Cattle Egret	2021/11/16	2	0	13.0463405	80.2533557	https://www.inaturalist.org/observations/101320776
1412	Bubulcus ibis	Cattle Egret	2021/11/16	1	0	13.0463405	80.2533557	https://www.inaturalist.org/observations/101320795
1413	Bubulcus ibis	Cattle Egret	2021/10/29	1	0	13.0463405	80.2533557	https://www.inaturalist.org/observations/101988541
1414	Bubulcus ibis	Cattle Egret	2021/12/6	1	0	13.0463405	80.2533557	https://www.inaturalist.org/observations/102599115
1415	Bubulcus ibis	Cattle Egret	2021/12/6	1	0	13.0463405	80.2533557	https://www.inaturalist.org/observations/102599145
1416	Bubulcus ibis	Cattle Egret	2021/12/15	2	0	13.0463405	80.2533557	https://www.inaturalist.org/observations/103093741
1417	Bubulcus ibis	Cattle Egret	2021/12/20	3	0	13.0463405	80.2533557	https://www.inaturalist.org/observations/103402178
1418	Bubulcus ibis	Cattle Egret	2021/12/20	3	0	13.0463405	80.2533557	https://www.inaturalist.org/observations/103402621
1419	Bubulcus ibis	Cattle Egret	2021/12/18	1	0	13.0463405	80.2533557	https://www.inaturalist.org/observations/103409219
1420	Bubulcus ibis	Cattle Egret	2022/2/18	3	0	13.0463405	80.2533557	https://www.inaturalist.org/observations/106960828
1421	Bubulcus ibis	Cattle Egret	2022/2/18	3	0	13.0463405	80.2533557	https://www.inaturalist.org/observations/106960943
1422	Bubulcus ibis	Cattle Egret	2022/2/18	3	0	13.0463405	80.2533557	https://www.inaturalist.org/observations/106961217
1423	Bubulcus ibis	Cattle Egret	2022/2/18	4	0	13.0463405	80.2533557	https://www.inaturalist.org/observations/106961327
1424	Bubulcus ibis	Cattle Egret	2022/3/4	4	0	13.0463405	80.2533557	https://www.inaturalist.org/observations/107883498

1425	Bubulcus ibis	Cattle Egret	2022/1/5	3	0	13.0463405	80.2533557	https://www.inaturalist.org/observations/133468440
1426	Bubulcus ibis	Cattle Egret	2022/11/15	1	0	13.0463405	80.2533557	https://www.inaturalist.org/observations/142054882
1427	Bubulcus ibis	Cattle Egret	2022/11/16	1	0	13.0463405	80.2533557	https://www.inaturalist.org/observations/142131797
1428	Bubulcus ibis	Cattle Egret	2021/12/13	5	0	13.0463406	80.2533559	https://www.inaturalist.org/observations/108363800
1429	Bubulcus ibis	Cattle Egret	2022/12/19	2	0	13.0463406	80.2533559	https://www.inaturalist.org/observations/144922604
1430	Bubulcus ibis	Cattle Egret	2022/1/15	3	0	13.0463463	80.2533557	https://www.inaturalist.org/observations/132639219
1431	Bubulcus ibis	Cattle Egret	2022/8/29	3	0	13.0463496	80.2533557	https://www.inaturalist.org/observations/132794204
1432	Bubulcus ibis	Cattle Egret	2019/11/12	1	0	13.0473487	80.2540004	https://www.inaturalist.org/observations/89443291
1433	Bubulcus ibis	Cattle Egret	2021/2/19	2	0	13.0473487	80.2540004	https://www.inaturalist.org/observations/89748862
1434	Bubulcus ibis	Cattle Egret	2021/3/6	1	0	13.0473487	80.2540004	https://www.inaturalist.org/observations/89748908
1435	Bubulcus ibis	Cattle Egret	2020/2/14	1	0	13.0473487	80.2540004	https://www.inaturalist.org/observations/88274129
1436	Bubulcus ibis	Cattle Egret	2020/2/14	1	0	13.0473487	80.2540004	https://www.inaturalist.org/observations/89024839
1437	Bubulcus ibis	Cattle Egret	2021/10/7	2	0	13.0473533	80.2540001	https://www.inaturalist.org/observations/97436356
1438	Bubulcus ibis	Cattle Egret	2021/12/6	2	0	13.0473533	80.2540001	https://www.inaturalist.org/observations/102612663
1439	Bubulcus ibis	Cattle Egret	2021/12/13	2	0	13.0473533	80.2540001	https://www.inaturalist.org/observations/103373318
1440	Bubulcus ibis	Cattle Egret	2021/12/17	1	0	13.0473533	80.2540001	https://www.inaturalist.org/observations/103398657
1441	Bubulcus ibis	Cattle Egret	2021/12/18	2	0	13.0473533	80.2540001	https://www.inaturalist.org/observations/103398687
1442	Bubulcus ibis	Cattle Egret	2022/1/6	1	0	13.0473533	80.2540001	https://www.inaturalist.org/observations/104466456
1443	Bubulcus ibis	Cattle Egret	2022/3/10	4	0	13.0473533	80.2540001	https://www.inaturalist.org/observations/108351732
1444	Bubulcus ibis	Cattle Egret	2022/3/22	5	0	13.0473533	80.2540001	https://www.inaturalist.org/observations/109214624
1445	Bubulcus ibis	Cattle Egret	2022/4/6	4	0	13.0473533	80.2540001	https://www.inaturalist.org/observations/110562220
1446	Bubulcus ibis	Cattle Egret	2022/4/11	3	0	13.0473533	80.2540001	https://www.inaturalist.org/observations/111078858
1447	Bubulcus ibis	Cattle Egret	2022/11/25	1	0	13.0514455	80.2546562	https://www.inaturalist.org/observations/142825260
1448	Bubulcus ibis	Cattle Egret	2021/4/5	2	0	13.0548325	77.5424805	https://www.inaturalist.org/observations/72973641
1449	Bubulcus ibis	Cattle Egret	2022/1/7	2	0	13.0561236	77.7523708	https://www.inaturalist.org/observations/105414846

1450	Bubulcus ibis	Cattle Egret	2021/10/15	1	0	13.0582576	77.7527523	https://www.inaturalist.org/observations/99160663
1451	Bubulcus ibis coromandus	Cattle Egret	2021/1/31	2	0	13.0604558	77.6570840	https://www.inaturalist.org/observations/68902297
1452	Bubulcus ibis	Cattle Egret	2020/12/20	1	0	13.0702915	77.7695255	https://www.inaturalist.org/observations/105188115
1453	Bubulcus ibis	Cattle Egret	2019/4/7	3	0	13.0711600	77.5804750	https://www.inaturalist.org/observations/29238165
1454	Bubulcus ibis	Cattle Egret	2022/1/5	1	0	13.0826438	80.2707183	https://www.inaturalist.org/observations/143354246
1455	Bubulcus ibis	Cattle Egret	2022/1/6	1	0	13.0826438	80.2707183	https://www.inaturalist.org/observations/143356229
1456	Bubulcus ibis	Cattle Egret	2022/2/13	1	0	13.0826438	80.2707183	https://www.inaturalist.org/observations/143411528
1457	Bubulcus ibis	Cattle Egret	2021/2/16	1	0	13.0826802	80.2707184	https://www.inaturalist.org/observations/69650747
1458	Bubulcus ibis	Cattle Egret	2012/3/25	2	0	13.0826802	80.2707184	https://www.inaturalist.org/observations/89166947
1459	Bubulcus ibis	Cattle Egret	2020/2/7	2	0	13.0826802	80.2707184	https://www.inaturalist.org/observations/137421997
1460	Bubulcus ibis	Cattle Egret	2020/4/24	1	0	13.0845939	77.6457427	https://www.inaturalist.org/observations/43871378
1461	Bubulcus ibis	Cattle Egret	2022/11/29	3	0	13.1067415	80.0969511	https://www.inaturalist.org/observations/143227463
1462	Bubulcus ibis coromandus	Cattle Egret	2017/5/5	6	0	13.1105551	77.5963286	https://www.inaturalist.org/observations/6149841
1463	Bubulcus ibis	Cattle Egret	2021/3/24	1	0	13.1108173	77.5759123	https://www.inaturalist.org/observations/72082339
1464	Bubulcus ibis	Cattle Egret	2021/2/13	1	0	13.1169157	77.5360799	https://www.inaturalist.org/observations/69613259
1465	Bubulcus ibis	Cattle Egret	2020/12/26	3	0	13.1241361	79.9556422	https://www.inaturalist.org/observations/67087155
1466	Bubulcus ibis	Cattle Egret	2022/12/4	1	0	13.1502389	79.9631584	https://www.inaturalist.org/observations/143791892
1467	Bubulcus ibis	Cattle Egret	2017/4/16	2	0	13.2156583	75.9806550	https://www.inaturalist.org/observations/65517333
1468	Bubulcus ibis	Cattle Egret	2020/4/6	2	0	13.3004896	77.4170514	https://www.inaturalist.org/observations/57529569
1469	Bubulcus ibis	Cattle Egret	2020/11/18	4	0	13.3128754	77.5420005	https://www.inaturalist.org/observations/115836194
1470	Bubulcus ibis	Cattle Egret	2019/1/14	5	0	13.3456297	74.7942886	https://www.inaturalist.org/observations/19652982
1471	Bubulcus ibis	Cattle Egret	2020/1/13	2	0	13.3470147	74.7966744	https://www.inaturalist.org/observations/37558518
1472	Bubulcus ibis	Cattle Egret	2019/1/9	5	0	13.3473083	74.7940367	https://www.inaturalist.org/observations/19528295
1473	Bubulcus ibis	Cattle Egret	2020/1/8	2	0	13.3488320	74.7940710	https://www.inaturalist.org/observations/37543471
1474	Bubulcus ibis	Cattle Egret	2020/1/16	2	0	13.3489300	74.7929617	https://www.inaturalist.org/observations/37646397

1475	Bubulcus ibis	Cattle Egret	2020/1/13	2	0	13.3489959	74.7855559	https://www.inaturalist.org/observations/37583081
1476	Bubulcus ibis	Cattle Egret	2020/1/13	1	0	13.3490105	74.7929764	https://www.inaturalist.org/observations/37739337
1477	Bubulcus ibis	Cattle Egret	2020/1/15	1	0	13.3491231	74.7855559	https://www.inaturalist.org/observations/37739305
1478	Bubulcus ibis	Cattle Egret	2020/1/11	2	0	13.3495333	74.7928783	https://www.inaturalist.org/observations/37512700
1479	Bubulcus ibis	Cattle Egret	2019/2/9	5	0	13.3514767	74.7861083	https://www.inaturalist.org/observations/20219650
1480	Bubulcus ibis	Cattle Egret	2019/1/4	5	0	13.3515501	74.7926254	https://www.inaturalist.org/observations/19490613
1481	Bubulcus ibis	Cattle Egret	2020/1/7	3	0	13.3525321	74.7928224	https://www.inaturalist.org/observations/37361004
1482	Bubulcus ibis	Cattle Egret	2020/1/14	1	0	13.3525321	74.7928224	https://www.inaturalist.org/observations/37614451
1483	Bubulcus ibis	Cattle Egret	2020/10/24	2	0	13.3555464	80.3352364	https://www.inaturalist.org/observations/63445264
1484	Bubulcus ibis	Cattle Egret	2020/3/9	2	0	13.3618883	74.7163683	https://www.inaturalist.org/observations/40656825
1485	Bubulcus ibis	Cattle Egret	2022/11/10	1	0	13.3673824	74.8176881	https://www.inaturalist.org/observations/141886339
1486	Bubulcus ibis	Cattle Egret	2016/1/31	2	0	13.4078482	80.2979325	https://www.inaturalist.org/observations/73977804
1487	Bubulcus ibis	Cattle Egret	2021/1/4	0	0	13.4157405	75.2515063	https://www.inaturalist.org/observations/81534327
1488	Bubulcus ibis	Cattle Egret	2022/1/31	2	0	13.4166639	80.2906659	https://www.inaturalist.org/observations/105917850
1489	Bubulcus ibis	Cattle Egret	2022/1/31	2	0	13.4166639	80.2906659	https://www.inaturalist.org/observations/105917860
1490	Bubulcus ibis	Cattle Egret	2022/1/31	3	0	13.4166639	80.2906659	https://www.inaturalist.org/observations/105924281
1491	Bubulcus ibis	Cattle Egret	2022/1/31	1	0	13.4166639	80.2906659	https://www.inaturalist.org/observations/105976339
1492	Bubulcus ibis	Cattle Egret	2020/1/30	3	0	13.4485095	94.2651945	https://www.inaturalist.org/observations/38814713
1493	Bubulcus ibis	Cattle Egret	2022/2/26	3	0	13.4580902	80.2966653	https://www.inaturalist.org/observations/142075800
1494	Bubulcus ibis	Cattle Egret	2022/2/13	3	0	13.5058442	78.3336165	https://www.inaturalist.org/observations/106909197
1495	Bubulcus ibis	Cattle Egret	2022/12/16	1	0	13.5100618	75.1022199	https://www.inaturalist.org/observations/144461364
1496	Bubulcus ibis	Cattle Egret	2015/11/24	1	0	13.5193307	75.0904302	https://www.inaturalist.org/observations/144459613
1497	Bubulcus ibis	Cattle Egret	2022/3/14	1	0	13.5316750	79.3083783	https://www.inaturalist.org/observations/115057376
1498	Bubulcus ibis	Cattle Egret	2022/9/25	2	0	13.5822967	79.5517983	https://www.inaturalist.org/observations/143847791
1499	Bubulcus ibis	Cattle Egret	2022/9/25	1	0	13.5823350	79.5517883	https://www.inaturalist.org/observations/137832288

1500	Bubulcus ibis	Cattle Egret	2022/9/25	1	0	13.5916217	79.5497167	https://www.inaturalist.org/observations/143847743
1501	Bubulcus ibis	Cattle Egret	2022/3/21	4	0	13.5920478	79.4455109	https://www.inaturalist.org/observations/111891310
1502	Bubulcus ibis	Cattle Egret	2022/3/19	3	0	13.6012237	79.4361808	https://www.inaturalist.org/observations/111772996
1503	Bubulcus ibis	Cattle Egret	2016/3/14	1	0	13.6208983	74.6713720	https://www.inaturalist.org/observations/48312085
1504	Bubulcus ibis	Cattle Egret	2022/3/26	3	0	13.6370932	79.4465182	https://www.inaturalist.org/observations/109925553
1505	Bubulcus ibis	Cattle Egret	2022/3/26	3	0	13.6371111	79.4464857	https://www.inaturalist.org/observations/109925560
1506	Bubulcus ibis	Cattle Egret	2020/5/20	2	0	13.6440621	79.4511702	https://www.inaturalist.org/observations/47245904
1507	Bubulcus ibis	Cattle Egret	2019/12/9	1	0	13.6445646	79.4512329	https://www.inaturalist.org/observations/36631384
1508	Bubulcus ibis	Cattle Egret	2020/4/7	2	0	13.6482194	79.4512326	https://www.inaturalist.org/observations/43064271
1509	Bubulcus ibis	Cattle Egret	2022/3/20	2	0	13.6502436	79.4666079	https://www.inaturalist.org/observations/111174418
1510	Bubulcus ibis	Cattle Egret	2022/3/9	2	0	13.6504354	79.4669579	https://www.inaturalist.org/observations/108230151
1511	Bubulcus ibis	Cattle Egret	2021/10/13	2	0	13.6514544	79.4684563	https://www.inaturalist.org/observations/98083270
1512	Bubulcus ibis	Cattle Egret	2022/2/26	1	0	13.6518788	79.4707455	https://www.inaturalist.org/observations/115048059
1513	Bubulcus ibis	Cattle Egret	2022/2/26	1	0	13.6533078	79.4720710	https://www.inaturalist.org/observations/109822031
1514	Bubulcus ibis	Cattle Egret	2020/3/22	1	0	13.6538465	74.9337264	https://www.inaturalist.org/observations/51089746
1515	Bubulcus ibis	Cattle Egret	2022/4/10	1	0	13.6588313	79.4900322	https://www.inaturalist.org/observations/111054416
1516	Bubulcus ibis	Cattle Egret	2021/3/16	4	0	13.7158637	79.5961605	https://www.inaturalist.org/observations/71361327
1517	Bubulcus ibis	Cattle Egret	2021/3/16	3	0	13.7160001	79.5962626	https://www.inaturalist.org/observations/71361324
1518	Bubulcus ibis	Cattle Egret	2022/4/6	2	0	13.7162197	79.5990671	https://www.inaturalist.org/observations/110635963
1519	Bubulcus ibis	Cattle Egret	2016/1/30	1	0	13.7198764	80.1154694	https://www.inaturalist.org/observations/73979571
1520	Bubulcus ibis	Cattle Egret	2019/12/17	3	0	13.7242176	80.1234449	https://www.inaturalist.org/observations/65587758
1521	Bubulcus ibis	Cattle Egret	2019/11/4	1	0	13.7358951	75.6324272	https://www.inaturalist.org/observations/88654748
1522	Bubulcus ibis	Cattle Egret	2015/11/14	1	0	13.7400521	77.3232365	https://www.inaturalist.org/observations/96570778
1523	Bubulcus ibis	Cattle Egret	2020/11/14	1	0	13.7725628	79.4291224	https://www.inaturalist.org/observations/64839655
1524	Bubulcus ibis	Cattle Egret	2021/11/22	2	0	13.8219499	74.6425865	https://www.inaturalist.org/observations/141903019

1525	Bubulcus ibis	Cattle Egret	2017/2/5	5	0	13.9627732	77.6750869	https://www.inaturalist.org/observations/5135097
1526	Bubulcus ibis	Cattle Egret	2019/12/23	3	0	14.0998874	78.2941639	https://www.inaturalist.org/observations/36877137
1527	Bubulcus ibis	Cattle Egret	2021/10/31	1	0	14.2489372	74.6530699	https://www.inaturalist.org/observations/100599008
1528	Bubulcus ibis	Cattle Egret	2020/4/4	2	0	14.2876030	74.4450519	https://www.inaturalist.org/observations/41505927
1529	Bubulcus ibis	Cattle Egret	2019/1/26	1	0	14.3874785	75.8330591	https://www.inaturalist.org/observations/84829106
1530	Bubulcus ibis	Cattle Egret	2021/12/18	1	0	14.4672704	79.9885043	https://www.inaturalist.org/observations/103235689
1531	Bubulcus ibis	Cattle Egret	2011/2/9	1	0	14.5699919	79.0419822	https://www.inaturalist.org/observations/80829049
1532	Bubulcus ibis	Cattle Egret	2020/12/5	1	0	14.8187253	75.6110924	https://www.inaturalist.org/observations/90234692
1533	Bubulcus ibis	Cattle Egret	2021/3/17	5	0	14.8665642	74.1611552	https://www.inaturalist.org/observations/121108220
1534	Bubulcus ibis	Cattle Egret	2018/5/13	5	0	14.9902716	74.0386271	https://www.inaturalist.org/observations/13100717
1535	Bubulcus ibis	Cattle Egret	2012/5/16	5	0	14.9910530	74.1026802	https://www.inaturalist.org/observations/75530079
1536	Bubulcus ibis	Cattle Egret	2021/12/6	2	0	15.0093890	74.0293730	https://www.inaturalist.org/observations/102603183
1537	Bubulcus ibis	Cattle Egret	2019/4/23	5	0	15.1302516	76.2240123	https://www.inaturalist.org/observations/116077127
1538	Bubulcus ibis	Cattle Egret	2020/2/7	3	0	15.1575695	73.9464867	https://www.inaturalist.org/observations/38384459
1539	Bubulcus ibis	Cattle Egret	2014/1/21	1	0	15.1757125	73.9408350	https://www.inaturalist.org/observations/103477715
1540	Bubulcus ibis	Cattle Egret	2019/11/18	6	0	15.1811792	73.9496120	https://www.inaturalist.org/observations/36153981
1541	Bubulcus ibis	Cattle Egret	2020/4/4	2	0	15.2137626	73.9357281	https://www.inaturalist.org/observations/41677123
1542	Bubulcus ibis	Cattle Egret	2021/10/23	2	0	15.2159113	74.2195572	https://www.inaturalist.org/observations/106552938
1543	Bubulcus ibis	Cattle Egret	2018/5/16	2	0	15.2230556	73.9372222	https://www.inaturalist.org/observations/85063463
1544	Bubulcus ibis	Cattle Egret	2016/7/14	2	0	15.2395777	77.0574663	https://www.inaturalist.org/observations/137233649
1545	Bubulcus ibis	Cattle Egret	2016/11/3	1	0	15.2454651	73.9325010	https://www.inaturalist.org/observations/37781442
1546	Bubulcus ibis coromandus	Cattle Egret	2021/2/26	2	0	15.2620385	76.4803470	https://www.inaturalist.org/observations/70188165
1547	Bubulcus ibis	Cattle Egret	2020/12/6	1	0	15.2857066	74.9298191	https://www.inaturalist.org/observations/90235421
1548	Bubulcus ibis	Cattle Egret	2020/1/22	1	0	15.2865157	73.9409132	https://www.inaturalist.org/observations/38951651
1549	Bubulcus ibis	Cattle Egret	2019/3/1	4	0	15.2948634	73.9074282	https://www.inaturalist.org/observations/65494156

1550	Bubulcus ibis	Cattle Egret	2019/11/20	2	0	15.3002305	74.1239958	https://www.inaturalist.org/observations/65468717
1551	Bubulcus ibis	Cattle Egret	2020/12/7	2	0	15.3002305	74.1239958	https://www.inaturalist.org/observations/67693978
1552	Bubulcus ibis	Cattle Egret	2022/8/19	0	0	15.3172775	75.7138884	https://www.inaturalist.org/observations/132797069
1553	Bubulcus ibis	Cattle Egret	2022/10/4	2	0	15.3303778	76.4592517	https://www.inaturalist.org/observations/143010828
1554	Bubulcus ibis	Cattle Egret	2014/3/15	1	0	15.3350132	76.4600240	https://www.inaturalist.org/observations/81444035
1555	Bubulcus ibis	Cattle Egret	2003/3/17	2	0	15.3350132	76.4600240	https://www.inaturalist.org/observations/105666651
1556	Bubulcus ibis	Cattle Egret	2015/2/7	4	0	15.3376955	76.4630119	https://www.inaturalist.org/observations/42458852
1557	Bubulcus ibis	Cattle Egret	2020/11/20	4	0	15.3849015	75.1026649	https://www.inaturalist.org/observations/66245551
1558	Bubulcus ibis	Cattle Egret	2011/2/22	3	0	15.3911236	73.8781616	https://www.inaturalist.org/observations/46580498
1559	Bubulcus ibis	Cattle Egret	2008/11/17	2	0	15.3993440	73.8441730	https://www.inaturalist.org/observations/57421652
1560	Bubulcus ibis	Cattle Egret	2021/12/24	1	0	15.4515667	73.8503033	https://www.inaturalist.org/observations/103677073
1561	Bubulcus ibis	Cattle Egret	2014/11/18	2	0	15.4527767	74.0107378	https://www.inaturalist.org/observations/142408050
1562	Bubulcus ibis	Cattle Egret	2022/1/3	1	0	15.4650724	73.8656665	https://www.inaturalist.org/observations/105643636
1563	Bubulcus ibis	Cattle Egret	2019/12/22	2	0	15.4781590	73.8106269	https://www.inaturalist.org/observations/36845972
1564	Bubulcus ibis	Cattle Egret	2018/4/15	1	0	15.4935065	73.8171632	https://www.inaturalist.org/observations/47262168
1565	Bubulcus ibis	Cattle Egret	2018/12/15	2	0	15.5024167	73.9134167	https://www.inaturalist.org/observations/67967872
1566	Bubulcus ibis	Cattle Egret	2022/11/12	3	0	15.5032250	73.9126150	https://www.inaturalist.org/observations/141883186
1567	Bubulcus ibis	Cattle Egret	2022/5/20	5	0	15.5038769	73.9121918	https://www.inaturalist.org/observations/118141818
1568	Bubulcus ibis	Cattle Egret	2019/3/6	2	0	15.5134106	73.8222427	https://www.inaturalist.org/observations/21508071
1569	Bubulcus ibis	Cattle Egret	2017/6/1	3	0	15.5261977	73.8321404	https://www.inaturalist.org/observations/36403331
1570	Bubulcus ibis	Cattle Egret	2017/5/28	6	0	15.5634075	73.8051895	https://www.inaturalist.org/observations/36093376
1571	Bubulcus ibis	Cattle Egret	2022/4/9	3	0	15.5638262	73.8053098	https://www.inaturalist.org/observations/111154043
1572	Bubulcus ibis	Cattle Egret	2012/3/6	4	0	15.5817690	73.8489990	http://www.inaturalist.org/observations/1587787
1573	Bubulcus ibis	Cattle Egret	2021/5/2	1	0	15.5868106	73.7453296	https://www.inaturalist.org/observations/76447810
1574	Bubulcus ibis	Cattle Egret	2021/10/29	2	0	15.6843116	73.9668408	https://www.inaturalist.org/observations/100685743

1575	Bubulcus ibis coromandus	Cattle Egret	2022/11/17	0	0	15.7092388	74.2274459	https://www.inaturalist.org/observations/142676249
1576	Bubulcus ibis	Cattle Egret	2021/10/29	3	0	15.7245025	73.9775642	https://www.inaturalist.org/observations/104257193
1577	Bubulcus ibis	Cattle Egret	2020/7/31	4	0	15.8039250	78.0786694	https://www.inaturalist.org/observations/63330676
1578	Bubulcus ibis	Cattle Egret	2016/12/10	1	0	15.9498279	75.8160444	https://www.inaturalist.org/observations/82685072
1579	Bubulcus ibis	Cattle Egret	2020/1/1	2	0	16.0199067	75.8809899	https://www.inaturalist.org/observations/37333322
1580	Bubulcus ibis	Cattle Egret	2021/1/20	1	0	16.1842754	77.4099496	https://www.inaturalist.org/observations/81796003
1581	Bubulcus ibis	Cattle Egret	2020/12/27	1	0	16.1903698	77.3668809	https://www.inaturalist.org/observations/67122020
1582	Bubulcus ibis	Cattle Egret	2018/12/16	1	0	16.2209164	74.7489645	https://www.inaturalist.org/observations/90276728
1583	Bubulcus ibis	Cattle Egret	2015/7/22	7	0	16.2377925	80.1659372	https://www.inaturalist.org/observations/17832723
1584	Bubulcus ibis	Cattle Egret	2020/10/26	2	0	16.2693758	73.6784256	https://www.inaturalist.org/observations/63626282
1585	Bubulcus ibis	Cattle Egret	2014/5/21	1	0	16.3009744	80.4826479	https://www.inaturalist.org/observations/94112850
1586	Bubulcus ibis	Cattle Egret	2014/12/9	2	0	16.3061785	80.5129217	https://www.inaturalist.org/observations/88111015
1587	Bubulcus ibis	Cattle Egret	2013/5/19	2	0	16.3063074	80.5123720	https://www.inaturalist.org/observations/88104302
1588	Bubulcus ibis	Cattle Egret	2013/5/19	2	0	16.3063074	80.5123720	https://www.inaturalist.org/observations/88104306
1589	Bubulcus ibis	Cattle Egret	2014/12/1	1	0	16.3066195	80.5126851	https://www.inaturalist.org/observations/94110991
1590	Bubulcus ibis	Cattle Egret	2014/5/7	3	0	16.3068910	80.5125264	https://www.inaturalist.org/observations/90128776
1591	Bubulcus ibis	Cattle Egret	2014/5/21	2	0	16.3068910	80.5125264	https://www.inaturalist.org/observations/90240685
1592	Bubulcus ibis	Cattle Egret	2014/5/30	4	0	16.3068910	80.5125264	https://www.inaturalist.org/observations/90240973
1593	Bubulcus ibis	Cattle Egret	2014/6/16	1	0	16.3068910	80.5125264	https://www.inaturalist.org/observations/94112031
1594	Bubulcus ibis	Cattle Egret	2014/4/18	2	0	16.3081759	80.4422321	https://www.inaturalist.org/observations/89170137
1595	Bubulcus ibis	Cattle Egret	2020/11/16	2	0	16.3589350	74.3046760	https://www.inaturalist.org/observations/65214680
1596	Bubulcus ibis	Cattle Egret	2020/9/27	0	0	16.3631180	74.2915000	https://www.inaturalist.org/observations/60979747
1597	Bubulcus ibis	Cattle Egret	2015/7/22	5	0	16.3891696	80.1558853	https://www.inaturalist.org/observations/17832751
1598	Bubulcus ibis	Cattle Egret	2022/5/7	4	0	16.4072319	80.4031423	https://www.inaturalist.org/observations/115842294
1599	Bubulcus ibis	Cattle Egret	2012/7/13	2	0	16.4984380	80.5984582	https://www.inaturalist.org/observations/93589681

1600	Bubulcus ibis	Cattle Egret	2013/4/24	2	0	16.5003047	80.6011540	https://www.inaturalist.org/observations/88092037
1601	Bubulcus ibis	Cattle Egret	2012/2/28	1	0	16.5009947	80.5914539	https://www.inaturalist.org/observations/88029888
1602	Bubulcus ibis	Cattle Egret	2015/1/25	2	0	16.5046081	79.2783437	https://www.inaturalist.org/observations/94201313
1603	Bubulcus ibis	Cattle Egret	2022/5/8	0	0	16.5073362	80.6332328	https://www.inaturalist.org/observations/116218595
1604	Bubulcus ibis	Cattle Egret	2021/12/20	1	0	16.5158642	80.5911313	https://www.inaturalist.org/observations/103396095
1605	Bubulcus ibis	Cattle Egret	2020/11/29	3	0	16.5183747	81.2758326	https://www.inaturalist.org/observations/67455545
1606	Bubulcus ibis	Cattle Egret	2020/5/8	1	0	16.5214906	80.5908978	https://www.inaturalist.org/observations/45249581
1607	Bubulcus ibis	Cattle Egret	2011/12/13	2	0	16.5223155	80.5727870	https://www.inaturalist.org/observations/89648078
1608	Bubulcus ibis	Cattle Egret	2020/3/9	1	0	16.5269154	76.6576161	https://www.inaturalist.org/observations/95630936
1609	Bubulcus ibis	Cattle Egret	2022/2/1	1	0	16.5358633	81.4976300	https://www.inaturalist.org/observations/105946846
1610	Bubulcus ibis	Cattle Egret	2022/8/24	3	0	16.5383388	81.5003314	https://www.inaturalist.org/observations/132111606
1611	Bubulcus ibis	Cattle Egret	2021/10/23	2	0	16.5436780	81.4922860	https://www.inaturalist.org/observations/99070750
1612	Bubulcus ibis	Cattle Egret	2021/12/22	1	0	16.5448909	81.5212409	https://www.inaturalist.org/observations/103471949
1613	Bubulcus ibis	Cattle Egret	2021/12/22	1	0	16.5448909	81.5212409	https://www.inaturalist.org/observations/103531632
1614	Bubulcus ibis	Cattle Egret	2021/12/25	1	0	16.5448909	81.5212409	https://www.inaturalist.org/observations/103639029
1615	Bubulcus ibis	Cattle Egret	2022/5/7	1	0	16.5448909	81.5212409	https://www.inaturalist.org/observations/116117502
1616	Bubulcus ibis	Cattle Egret	2022/5/7	2	0	16.5448909	81.5212409	https://www.inaturalist.org/observations/116435564
1617	Bubulcus ibis	Cattle Egret	2022/5/7	2	0	16.5448909	81.5212409	https://www.inaturalist.org/observations/116437670
1618	Bubulcus ibis	Cattle Egret	2022/5/7	2	0	16.5448909	81.5212409	https://www.inaturalist.org/observations/116574957
1619	Bubulcus ibis	Cattle Egret	2022/5/7	1	0	16.5448909	81.5212409	https://www.inaturalist.org/observations/116718015
1620	Bubulcus ibis	Cattle Egret	2022/5/7	1	0	16.5448909	81.5212409	https://www.inaturalist.org/observations/116718629
1621	Bubulcus ibis	Cattle Egret	2022/5/7	3	0	16.5468896	81.4832253	https://www.inaturalist.org/observations/115842389
1622	Bubulcus ibis	Cattle Egret	2021/11/22	1	0	16.5559300	81.4912760	https://www.inaturalist.org/observations/102016153
1623	Bubulcus ibis	Cattle Egret	2021/11/22	1	0	16.5559300	81.4912760	https://www.inaturalist.org/observations/102017096
1624	Bubulcus ibis	Cattle Egret	2021/9/1	2	0	16.5567355	81.4942043	https://www.inaturalist.org/observations/99065973

1625	Bubulcus ibis	Cattle Egret	2021/12/25	1	0	16.5569433	81.4961333	https://www.inaturalist.org/observations/104647603
1626	Bubulcus ibis	Cattle Egret	2021/12/25	3	0	16.5569950	81.4941817	https://www.inaturalist.org/observations/111967110
1627	Bubulcus ibis	Cattle Egret	2022/2/4	2	0	16.5571917	81.4943100	https://www.inaturalist.org/observations/106119782
1628	Bubulcus ibis	Cattle Egret	2020/12/20	1	0	16.5577292	80.7269854	https://www.inaturalist.org/observations/92475027
1629	Bubulcus ibis	Cattle Egret	2020/9/1	3	0	16.5578752	80.7064398	https://www.inaturalist.org/observations/58258010
1630	Bubulcus ibis	Cattle Egret	2021/10/10	3	0	16.5581190	81.4981635	https://www.inaturalist.org/observations/97889568
1631	Bubulcus ibis	Cattle Egret	2020/12/6	1	0	16.5592179	80.7610010	https://www.inaturalist.org/observations/92452601
1632	Bubulcus ibis	Cattle Egret	2021/8/1	1	0	16.5665453	80.6189987	https://www.inaturalist.org/observations/89445113
1633	Bubulcus ibis	Cattle Egret	2021/12/5	2	0	16.5755915	80.6823019	https://www.inaturalist.org/observations/104498307
1634	Bubulcus ibis	Cattle Egret	2020/11/29	1	0	16.5758927	80.6886222	https://www.inaturalist.org/observations/92670072
1635	Bubulcus ibis	Cattle Egret	2021/12/24	2	0	16.5759672	79.3124022	https://www.inaturalist.org/observations/105600292
1636	Bubulcus ibis	Cattle Egret	2020/12/27	1	0	16.5957792	80.6181858	https://www.inaturalist.org/observations/90049745
1637	Bubulcus ibis	Cattle Egret	2020/12/27	1	0	16.6002639	80.6164944	https://www.inaturalist.org/observations/67125892
1638	Bubulcus ibis	Cattle Egret	2022/5/4	2	0	16.6174076	80.3606048	https://www.inaturalist.org/observations/116945948
1639	Bubulcus ibis	Cattle Egret	2017/12/21	6	0	16.6343900	73.3570047	https://www.inaturalist.org/observations/30732789
1640	Bubulcus ibis	Cattle Egret	2020/11/8	1	0	16.6357807	80.7980061	https://www.inaturalist.org/observations/92484760
1641	Bubulcus ibis	Cattle Egret	2020/11/8	1	0	16.6357807	80.7980061	https://www.inaturalist.org/observations/92484784
1642	Bubulcus ibis	Cattle Egret	2022/10/16	2	0	16.6398103	80.4851532	https://www.inaturalist.org/observations/140376593
1643	Bubulcus ibis	Cattle Egret	2022/2/19	3	0	16.6495021	80.4908577	https://www.inaturalist.org/observations/108083299
1644	Bubulcus ibis	Cattle Egret	2022/9/18	1	0	16.6511318	80.4669117	https://www.inaturalist.org/observations/135761402
1645	Bubulcus ibis	Cattle Egret	2018/3/11	4	0	16.6545164	73.5125814	https://www.inaturalist.org/observations/30862166
1646	Bubulcus ibis	Cattle Egret	2020/9/12	4	0	16.6616227	74.2408329	https://www.inaturalist.org/observations/59863561
1647	Bubulcus ibis	Cattle Egret	2021/7/9	2	0	16.6624481	80.6997220	https://www.inaturalist.org/observations/86538368
1648	Bubulcus ibis	Cattle Egret	2018/6/3	1	0	16.6646610	81.1840201	https://www.inaturalist.org/observations/96476607
1649	Bubulcus ibis	Cattle Egret	2015/11/22	2	0	16.6649730	81.1810684	https://www.inaturalist.org/observations/94303545

1650	Bubulcus ibis	Cattle Egret	2018/3/3	1	0	16.6649730	81.1810684	https://www.inaturalist.org/observations/96478149
1651	Bubulcus ibis	Cattle Egret	2017/10/20	1	0	16.6649730	81.1810684	https://www.inaturalist.org/observations/98112197
1652	Bubulcus ibis	Cattle Egret	2015/2/21	1	0	16.6651507	81.1822816	https://www.inaturalist.org/observations/94230965
1653	Bubulcus ibis	Cattle Egret	2018/3/10	4	0	16.6825881	73.5468581	https://www.inaturalist.org/observations/30858980
1654	Bubulcus ibis	Cattle Egret	2022/2/18	0	0	16.6919639	80.7736000	https://www.inaturalist.org/observations/106968151
1655	Bubulcus ibis	Cattle Egret	2021/12/30	1	0	16.6996611	80.7642333	https://www.inaturalist.org/observations/103957929
1656	Bubulcus ibis	Cattle Egret	2020/10/31	1	0	16.7129620	82.1079752	https://www.inaturalist.org/observations/63935031
1657	Bubulcus ibis	Cattle Egret	2020/3/9	1	0	16.7424318	77.1068781	https://www.inaturalist.org/observations/95631080
1658	Bubulcus ibis	Cattle Egret	2022/2/21	3	0	16.7433636	81.2167693	https://www.inaturalist.org/observations/107168715
1659	Bubulcus ibis	Cattle Egret	2020/11/6	1	0	16.7454198	74.2284334	https://www.inaturalist.org/observations/64422918
1660	Bubulcus ibis	Cattle Egret	2021/3/27	1	0	16.7480817	73.3307267	https://www.inaturalist.org/observations/72887308
1661	Bubulcus ibis	Cattle Egret	2022/5/10	5	0	16.7566737	81.6769713	https://www.inaturalist.org/observations/116381126
1662	Bubulcus ibis	Cattle Egret	2017/6/16	5	0	16.8523950	73.3204750	https://www.inaturalist.org/observations/30471075
1663	Bubulcus ibis	Cattle Egret	2021/5/19	1	0	16.8776035	80.9276858	https://www.inaturalist.org/observations/99640542
1664	Bubulcus ibis	Cattle Egret	2019/3/15	2	0	16.8860901	75.7093519	https://www.inaturalist.org/observations/48656053
1665	Bubulcus ibis	Cattle Egret	2011/10/19	2	0	16.9135908	81.8182663	https://www.inaturalist.org/observations/50745210
1666	Bubulcus ibis	Cattle Egret	2022/8/21	4	0	16.9361416	79.5084509	https://www.inaturalist.org/observations/132118896
1667	Bubulcus ibis	Cattle Egret	2021/5/10	1	0	16.9442253	74.4291135	https://www.inaturalist.org/observations/78200873
1668	Bubulcus ibis coromandus	Cattle Egret	2019/8/16	1	0	16.9840206	81.7836210	https://www.inaturalist.org/observations/31125755
1669	Bubulcus ibis	Cattle Egret	2022/1/15	2	0	16.9889430	82.2476168	https://www.inaturalist.org/observations/105019147
1670	Bubulcus ibis	Cattle Egret	2014/1/31	2	0	16.9930000	82.2720000	https://www.inaturalist.org/observations/35240921
1671	Bubulcus ibis	Cattle Egret	2018/5/8	3	0	16.9997067	73.3241014	https://www.inaturalist.org/observations/22764568
1672	Bubulcus ibis	Cattle Egret	2018/4/27	4	0	17.0009623	73.3221212	https://www.inaturalist.org/observations/11486471
1673	Bubulcus ibis	Cattle Egret	2020/3/25	2	0	17.0686131	81.8871384	https://www.inaturalist.org/observations/40691553
1674	Bubulcus ibis	Cattle Egret	2022/11/2	1	0	17.0699110	75.6877025	https://www.inaturalist.org/observations/141457838

1675	Bubulcus ibis	Cattle Egret	2021/12/25	1	0	17.1783353	80.4738868	https://www.inaturalist.org/observations/103617786
1676	Bubulcus ibis	Cattle Egret	2019/4/18	2	0	17.3270279	78.4104486	https://www.inaturalist.org/observations/22696598
1677	Bubulcus ibis	Cattle Egret	2022/1/7	8	0	17.3506780	78.4513068	https://www.inaturalist.org/observations/104522786
1678	Bubulcus ibis	Cattle Egret	2022/1/7	3	0	17.3506780	78.4513068	https://www.inaturalist.org/observations/104522787
1679	Bubulcus ibis	Cattle Egret	1980/1/1	2	0	17.3605399	78.5830721	https://www.inaturalist.org/observations/106677305
1680	Bubulcus ibis	Cattle Egret	2022/2/11	2	0	17.3616868	78.5810122	https://www.inaturalist.org/observations/106677304
1681	Bubulcus ibis	Cattle Egret	2022/2/11	2	0	17.3621783	78.5820421	https://www.inaturalist.org/observations/106677303
1682	Bubulcus ibis	Cattle Egret	2022/2/11	2	0	17.3621816	78.5816988	https://www.inaturalist.org/observations/106677302
1683	Bubulcus ibis	Cattle Egret	2022/2/11	2	0	17.3631613	78.5796389	https://www.inaturalist.org/observations/106677306
1684	Bubulcus ibis	Cattle Egret	2022/2/20	2	0	17.3657498	78.5823237	https://www.inaturalist.org/observations/107109066
1685	Bubulcus ibis	Cattle Egret	2019/4/29	2	0	17.3659770	77.8955797	https://www.inaturalist.org/observations/23856018
1686	Bubulcus ibis	Cattle Egret	2007/3/3	5	0	17.3727390	78.4785170	https://www.inaturalist.org/observations/18798180
1687	Bubulcus ibis	Cattle Egret	2022/11/27	3	0	17.3836184	78.3713540	https://www.inaturalist.org/observations/143132627
1688	Bubulcus ibis	Cattle Egret	2019/6/4	2	0	17.3970536	78.5197202	https://www.inaturalist.org/observations/26428823
1689	Bubulcus ibis	Cattle Egret	2020/7/5	3	0	17.4135013	78.5287355	https://www.inaturalist.org/observations/65023451
1690	Bubulcus ibis	Cattle Egret	2019/11/14	2	0	17.4168890	78.4386700	https://www.inaturalist.org/observations/58420054
1691	Bubulcus ibis	Cattle Egret	2022/2/5	2	0	17.4209212	78.4717085	https://www.inaturalist.org/observations/106160721
1692	Bubulcus ibis	Cattle Egret	2021/10/13	2	0	17.4212122	78.5408036	https://www.inaturalist.org/observations/98391060
1693	Bubulcus ibis	Cattle Egret	2022/7/31	3	0	17.4320751	78.4753877	https://www.inaturalist.org/observations/130092336
1694	Bubulcus ibis	Cattle Egret	2022/9/23	2	0	17.4320751	78.4753877	https://www.inaturalist.org/observations/137095129
1695	Bubulcus ibis	Cattle Egret	2019/8/18	6	0	17.4334618	78.5266567	https://www.inaturalist.org/observations/31033930
1696	Bubulcus ibis	Cattle Egret	2019/8/1	5	0	17.4340682	78.5257549	https://www.inaturalist.org/observations/30736404
1697	Bubulcus ibis	Cattle Egret	2017/12/1	5	0	17.4360208	78.3919117	https://www.inaturalist.org/observations/9014932
1698	Bubulcus ibis	Cattle Egret	2022/7/20	2	0	17.4460810	78.2221074	https://www.inaturalist.org/observations/140230610
1699	Bubulcus ibis	Cattle Egret	2022/10/12	0	0	17.4567789	78.2333743	https://www.inaturalist.org/observations/138816912

1700	Bubulcus ibis	Cattle Egret	2019/5/4	3	0	17.4697527	79.6930711	https://www.inaturalist.org/observations/24497681
1701	Bubulcus ibis	Cattle Egret	2020/12/3	2	0	17.4723432	78.7974250	https://www.inaturalist.org/observations/67861303
1702	Bubulcus ibis	Cattle Egret	2022/3/5	6	0	17.4846398	78.2429867	https://www.inaturalist.org/observations/107947011
1703	Bubulcus ibis	Cattle Egret	2022/3/5	5	0	17.4846398	78.2429867	https://www.inaturalist.org/observations/107947014
1704	Bubulcus ibis	Cattle Egret	2019/5/4	3	0	17.4851333	79.6855550	https://www.inaturalist.org/observations/24510432
1705	Bubulcus ibis	Cattle Egret	2022/2/19	1	0	17.4860569	78.3465311	https://www.inaturalist.org/observations/107240695
1706	Bubulcus ibis	Cattle Egret	2022/9/3	4	0	17.4860569	78.3465311	https://www.inaturalist.org/observations/133432833
1707	Bubulcus ibis	Cattle Egret	2022/9/3	5	0	17.4860569	78.3465311	https://www.inaturalist.org/observations/133432845
1708	Bubulcus ibis	Cattle Egret	2021/12/4	3	0	17.4860570	78.3465311	https://www.inaturalist.org/observations/102469055
1709	Bubulcus ibis	Cattle Egret	2021/12/8	2	0	17.4860570	78.3465311	https://www.inaturalist.org/observations/102805806
1710	Bubulcus ibis	Cattle Egret	2021/12/8	2	0	17.4860570	78.3465311	https://www.inaturalist.org/observations/102805812
1711	Bubulcus ibis	Cattle Egret	2020/7/1	3	0	17.4901217	78.5319050	https://www.inaturalist.org/observations/51565455
1712	Bubulcus ibis	Cattle Egret	2020/7/22	2	0	17.4952106	78.3134994	https://www.inaturalist.org/observations/53907535
1713	Bubulcus ibis	Cattle Egret	2022/11/6	1	0	17.4958478	77.9410251	https://www.inaturalist.org/observations/141255480
1714	Bubulcus ibis	Cattle Egret	2020/10/10	6	0	17.5175446	78.6512303	https://www.inaturalist.org/observations/62195376
1715	Bubulcus ibis	Cattle Egret	2022/9/6	2	0	17.5180951	78.2631080	https://www.inaturalist.org/observations/134276023
1716	Bubulcus ibis	Cattle Egret	2020/8/4	2	0	17.5224680	78.3344380	https://www.inaturalist.org/observations/56042724
1717	Bubulcus ibis	Cattle Egret	2021/10/29	1	0	17.5224792	78.3344383	https://www.inaturalist.org/observations/100986658
1718	Bubulcus ibis	Cattle Egret	2019/1/24	5	0	17.5238740	78.3889649	https://www.inaturalist.org/observations/19859519
1719	Bubulcus ibis	Cattle Egret	2021/10/3	3	0	17.5369001	78.6881403	https://www.inaturalist.org/observations/97882832
1720	Bubulcus ibis	Cattle Egret	2020/3/10	1	0	17.5712219	76.9075777	https://www.inaturalist.org/observations/95631477
1721	Bubulcus ibis	Cattle Egret	2022/6/12	1	0	17.6552448	78.0760688	https://www.inaturalist.org/observations/121375258
1722	Bubulcus ibis	Cattle Egret	2022/12/11	1	0	17.6595013	78.0602999	https://www.inaturalist.org/observations/144148590
1723	Bubulcus ibis	Cattle Egret	2013/8/2	2	0	17.6687912	80.8935925	https://www.inaturalist.org/observations/52544583
1724	Bubulcus ibis	Cattle Egret	2020/2/2	3	0	17.6804587	74.0182611	https://www.inaturalist.org/observations/66400706

1725	Bubulcus ibis	Cattle Egret	2020/12/16	4	0	17.6804639	74.0182610	https://www.inaturalist.org/observations/69146472
1726	Bubulcus ibis	Cattle Egret	2020/11/25	1	0	17.6804639	74.0182610	https://www.inaturalist.org/observations/71068309
1727	Bubulcus ibis	Cattle Egret	2020/11/25	1	0	17.6804639	74.0182610	https://www.inaturalist.org/observations/71068313
1728	Bubulcus ibis	Cattle Egret	2021/12/12	1	0	17.6866168	78.3273244	https://www.inaturalist.org/observations/104472175
1729	Bubulcus ibis	Cattle Egret	2019/1/31	5	0	17.7082806	73.9814453	https://www.inaturalist.org/observations/19986377
1730	Bubulcus ibis	Cattle Egret	2020/9/18	5	0	17.7880092	83.2118825	https://www.inaturalist.org/observations/59951062
1731	Bubulcus ibis	Cattle Egret	2022/5/5	3	0	17.8129020	83.4101520	https://www.inaturalist.org/observations/115553445
1732	Bubulcus ibis	Cattle Egret	2022/2/2	1	0	17.9325228	83.4267972	https://www.inaturalist.org/observations/107107267
1733	Bubulcus ibis	Cattle Egret	2019/6/1	2	0	17.9525830	80.0059790	https://www.inaturalist.org/observations/63635583
1734	Bubulcus ibis	Cattle Egret	2020/11/7	6	0	17.9918461	79.5591114	https://www.inaturalist.org/observations/64357484
1735	Bubulcus ibis	Cattle Egret	2020/8/2	1	0	17.9949490	78.3816220	https://www.inaturalist.org/observations/55353726
1736	Bubulcus ibis	Cattle Egret	2021/10/15	1	0	18.0529343	78.2618531	https://www.inaturalist.org/observations/99562760
1737	Bubulcus ibis	Cattle Egret	2021/1/18	4	0	18.0558778	83.3569250	https://www.inaturalist.org/observations/68259876
1738	Bubulcus ibis	Cattle Egret	2020/8/20	7	0	18.1073639	78.2307039	https://www.inaturalist.org/observations/57041750
1739	Bubulcus ibis	Cattle Egret	2018/11/11	3	0	18.1124370	79.0193000	https://www.inaturalist.org/observations/63556098
1740	Bubulcus ibis	Cattle Egret	2015/7/31	3	0	18.1124372	79.0192997	https://www.inaturalist.org/observations/63558558
1741	Bubulcus ibis	Cattle Egret	2018/6/21	2	0	18.1124372	79.0192997	https://www.inaturalist.org/observations/63573598
1742	Bubulcus ibis	Cattle Egret	2020/2/2	3	0	18.1404807	73.7908178	https://www.inaturalist.org/observations/66175050
1743	Bubulcus ibis	Cattle Egret	2022/2/2	4	0	18.1408172	73.7904463	https://www.inaturalist.org/observations/106016644
1744	Bubulcus ibis	Cattle Egret	2022/4/30	1	0	18.1408200	73.7904463	https://www.inaturalist.org/observations/114565071
1745	Bubulcus ibis	Cattle Egret	2020/11/5	5	0	18.1422866	73.7912332	https://www.inaturalist.org/observations/64235412
1746	Bubulcus ibis	Cattle Egret	2018/12/23	5	0	18.1724729	72.9888166	https://www.inaturalist.org/observations/19467068
1747	Bubulcus ibis	Cattle Egret	2020/3/10	5	0	18.1794381	74.6051258	https://www.inaturalist.org/observations/58676772
1748	Bubulcus ibis coromandus	Cattle Egret	2017/4/15	2	0	18.2143505	109.4959259	https://www.inaturalist.org/observations/5743387
1749	Bubulcus ibis	Cattle Egret	2021/5/14	2	0	18.2187857	73.8862876	https://www.inaturalist.org/observations/79173557

1750	Bubulcus ibis coromandus	Cattle Egret	2019/8/11	2	0	18.2524350	83.3379600	https://www.inaturalist.org/observations/34631395
1751	Bubulcus ibis	Cattle Egret	2018/11/27	1	0	18.2524850	74.7998117	https://www.inaturalist.org/observations/53841834
1752	Bubulcus ibis	Cattle Egret	2020/2/22	0	0	18.2633860	74.8042270	https://www.inaturalist.org/observations/39646955
1753	Bubulcus ibis	Cattle Egret	2018/5/13	1	0	18.2720439	74.7950705	https://www.inaturalist.org/observations/56207076
1754	Bubulcus ibis	Cattle Egret	2021/10/25	3	0	18.2726478	74.3206208	https://www.inaturalist.org/observations/121383465
1755	Bubulcus ibis	Cattle Egret	2020/8/13	1	0	18.2730400	84.0048438	https://www.inaturalist.org/observations/95508379
1756	Bubulcus ibis	Cattle Egret	2020/6/4	1	0	18.2730400	84.0048438	https://www.inaturalist.org/observations/95646473
1757	Bubulcus ibis	Cattle Egret	2021/8/19	1	0	18.2730400	84.0048438	https://www.inaturalist.org/observations/99488907
1758	Bubulcus ibis	Cattle Egret	2018/6/17	2	0	18.2751193	74.7962045	https://www.inaturalist.org/observations/54606150
1759	Bubulcus ibis	Cattle Egret	2018/11/20	3	0	18.2839133	74.7557633	https://www.inaturalist.org/observations/53840835
1760	Bubulcus ibis	Cattle Egret	2018/12/21	1	0	18.2844583	74.7555583	https://www.inaturalist.org/observations/53471185
1761	Bubulcus ibis	Cattle Egret	2019/1/23	2	0	18.2861664	74.7998481	https://www.inaturalist.org/observations/53344202
1762	Bubulcus ibis	Cattle Egret	2022/1/24	1	0	18.2889611	73.1335145	https://www.inaturalist.org/observations/105501696
1763	Bubulcus ibis	Cattle Egret	2012/3/9	2	0	18.2915827	74.7740800	https://www.inaturalist.org/observations/56113754
1764	Bubulcus ibis	Cattle Egret	2007/11/17	2	0	18.2929968	74.7720181	https://www.inaturalist.org/observations/44449798
1765	Bubulcus ibis	Cattle Egret	2022/1/29	1	0	18.3008734	73.1371371	https://www.inaturalist.org/observations/105763851
1766	Bubulcus ibis	Cattle Egret	2018/12/7	3	0	18.3266815	109.5392115	https://www.inaturalist.org/observations/19269236
1767	Bubulcus ibis	Cattle Egret	2018/9/29	2	0	18.3317534	109.5361216	https://www.inaturalist.org/observations/18979307
1768	Bubulcus ibis	Cattle Egret	2019/2/13	2	0	18.3376098	109.5874049	https://www.inaturalist.org/observations/20339268
1769	Bubulcus ibis	Cattle Egret	2016/8/15	2	0	18.3415649	74.3647834	https://www.inaturalist.org/observations/55508885
1770	Bubulcus ibis	Cattle Egret	2013/11/4	2	0	18.3465719	74.3639398	https://www.inaturalist.org/observations/57817560
1771	Bubulcus ibis	Cattle Egret	2018/12/29	3	0	18.3536280	109.6538814	https://www.inaturalist.org/observations/19321544
1772	Bubulcus ibis	Cattle Egret	2017/11/29	2	0	18.3560814	73.7029447	https://www.inaturalist.org/observations/55012809
1773	Bubulcus ibis	Cattle Egret	2014/3/16	5	0	18.3735266	73.7677223	https://www.inaturalist.org/observations/56552225
1774	Bubulcus ibis	Cattle Egret	2014/3/16	4	0	18.3735266	73.7677223	https://www.inaturalist.org/observations/56552234

1775	Bubulcus ibis	Cattle Egret	2017/11/5	3	0	18.3869637	74.1684625	https://www.inaturalist.org/observations/45665553
1776	Bubulcus ibis	Cattle Egret	2021/1/6	1	0	18.4029425	73.9955228	https://www.inaturalist.org/observations/70978241
1777	Bubulcus ibis	Cattle Egret	2021/3/29	3	0	18.4157565	73.3635013	https://www.inaturalist.org/observations/75178776
1778	Bubulcus ibis	Cattle Egret	2019/2/26	4	0	18.4260614	73.7635781	https://www.inaturalist.org/observations/53232684
1779	Bubulcus ibis	Cattle Egret	2022/11/7	2	0	18.4421428	73.7983443	https://www.inaturalist.org/observations/141357288
1780	Bubulcus ibis	Cattle Egret	2012/3/26	5	0	18.4564586	73.4506869	https://www.inaturalist.org/observations/17740835
1781	Bubulcus ibis	Cattle Egret	2018/2/2	2	0	18.4668531	73.4504300	https://www.inaturalist.org/observations/54822479
1782	Bubulcus ibis	Cattle Egret	2017/11/6	3	0	18.4842364	73.8260981	https://www.inaturalist.org/observations/55241909
1783	Bubulcus ibis	Cattle Egret	2019/5/11	7	0	18.4929055	73.8965665	https://www.inaturalist.org/observations/24969822
1784	Bubulcus ibis	Cattle Egret	2021/12/4	2	0	18.4932467	73.7964319	https://www.inaturalist.org/observations/102472713
1785	Bubulcus ibis	Cattle Egret	2021/12/4	2	0	18.4932726	73.7964266	https://www.inaturalist.org/observations/102472712
1786	Bubulcus ibis	Cattle Egret	2022/10/7	4	0	18.4943888	83.2828598	https://www.inaturalist.org/observations/137849747
1787	Bubulcus ibis	Cattle Egret	2013/8/4	4	0	18.4985537	73.5133839	https://www.inaturalist.org/observations/33243089
1788	Bubulcus ibis	Cattle Egret	2019/7/23	3	0	18.5006647	73.8530750	https://www.inaturalist.org/observations/52337062
1789	Bubulcus ibis	Cattle Egret	2017/10/28	2	0	18.5051631	73.9993697	https://www.inaturalist.org/observations/55245529
1790	Bubulcus ibis	Cattle Egret	2013/1/18	3	0	18.5052891	74.0001730	https://www.inaturalist.org/observations/56533307
1791	Bubulcus ibis	Cattle Egret	2013/1/18	3	0	18.5052891	74.0001730	https://www.inaturalist.org/observations/56533329
1792	Bubulcus ibis	Cattle Egret	2016/2/28	3	0	18.5059569	73.9965956	https://www.inaturalist.org/observations/48914806
1793	Bubulcus ibis	Cattle Egret	2016/2/28	2	0	18.5059569	73.9965956	https://www.inaturalist.org/observations/48914816
1794	Bubulcus ibis	Cattle Egret	2014/6/14	5	0	18.5305880	73.7863720	https://www.inaturalist.org/observations/17981152
1795	Bubulcus ibis	Cattle Egret	2013/12/12	2	0	18.5343969	73.7852922	https://www.inaturalist.org/observations/56545274
1796	Bubulcus ibis	Cattle Egret	2015/5/9	2	0	18.5343969	73.7852922	https://www.inaturalist.org/observations/141622223
1797	Bubulcus ibis coromandus	Cattle Egret	2019/8/11	2	0	18.5361117	83.3155550	https://www.inaturalist.org/observations/34631513
1798	Bubulcus ibis	Cattle Egret	2015/1/18	4	0	18.5443774	73.8907638	https://www.inaturalist.org/observations/109739892
1799	Bubulcus ibis	Cattle Egret	2014/4/20	1	0	18.5540312	73.7904576	https://www.inaturalist.org/observations/141622220

1800	Bubulcus ibis coromandus	Cattle Egret	2019/8/15	4	0	18.5715783	82.6107500	https://www.inaturalist.org/observations/35536949
1801	Bubulcus ibis	Cattle Egret	2022/2/2	5	0	18.5748139	79.8851639	https://www.inaturalist.org/observations/106017052
1802	Bubulcus ibis coromandus	Cattle Egret	2019/8/16	3	0	18.5907667	82.5674367	https://www.inaturalist.org/observations/35539499
1803	Bubulcus ibis	Cattle Egret	2016/9/10	4	0	18.5911292	73.9188200	http://www.inaturalist.org/observations/4077720
1804	Bubulcus ibis	Cattle Egret	2018/12/18	1	0	18.6416433	72.8969717	https://www.inaturalist.org/observations/53458630
1805	Bubulcus ibis	Cattle Egret	2019/4/2	3	0	18.6419981	72.8956747	https://www.inaturalist.org/observations/52661538
1806	Bubulcus ibis	Cattle Egret	2019/1/16	2	0	18.6420747	72.8956531	https://www.inaturalist.org/observations/53331310
1807	Bubulcus ibis	Cattle Egret	2020/5/24	2	0	18.6615809	72.8894230	https://www.inaturalist.org/observations/52270382
1808	Bubulcus ibis	Cattle Egret	2020/5/24	2	0	18.6619806	72.8894806	https://www.inaturalist.org/observations/102601443
1809	Bubulcus ibis	Cattle Egret	2020/10/30	2	0	18.6820507	76.9547653	https://www.inaturalist.org/observations/63809491
1810	Bubulcus ibis	Cattle Egret	2021/2/16	2	0	18.7035363	84.4041342	https://www.inaturalist.org/observations/69898749
1811	Bubulcus ibis coromandus	Cattle Egret	2019/8/11	2	0	18.7281100	83.3925283	https://www.inaturalist.org/observations/34631637
1812	Bubulcus ibis coromandus	Cattle Egret	2019/8/11	3	0	18.7281550	83.3926383	https://www.inaturalist.org/observations/34631123
1813	Bubulcus ibis coromandus	Cattle Egret	2006/5/29	1	0	18.7421813	72.8627658	https://www.inaturalist.org/observations/70485393
1814	Bubulcus ibis	Cattle Egret	2022/11/26	1	0	18.7786600	81.9971108	https://www.inaturalist.org/observations/143342285
1815	Bubulcus ibis	Cattle Egret	2018/7/4	6	0	18.7857495	81.9842046	https://www.inaturalist.org/observations/18161909
1816	Bubulcus ibis coromandus	Cattle Egret	2019/8/11	3	0	18.7927767	83.4289817	https://www.inaturalist.org/observations/34631780
1817	Bubulcus ibis coromandus	Cattle Egret	2019/8/11	3	0	18.8005383	83.4321250	https://www.inaturalist.org/observations/34631812
1818	Bubulcus ibis coromandus	Cattle Egret	2019/8/11	4	0	18.8005733	83.4322600	https://www.inaturalist.org/observations/34631150
1819	Bubulcus ibis coromandus	Cattle Egret	2019/8/11	3	0	18.8007900	83.4323100	https://www.inaturalist.org/observations/34631858
1820	Bubulcus ibis	Cattle Egret	2020/10/26	6	0	18.8214712	75.6470687	https://www.inaturalist.org/observations/63577030
1821	Bubulcus ibis	Cattle Egret	2018/3/29	2	0	18.8309158	110.3768921	https://www.inaturalist.org/observations/12161466
1822	Bubulcus ibis	Cattle Egret	2020/8/25	3	0	18.8312398	73.0703939	https://www.inaturalist.org/observations/57553226
1823	Bubulcus ibis	Cattle Egret	2020/1/10	4	0	18.8349686	80.6625493	https://www.inaturalist.org/observations/37578018
1824	Bubulcus ibis	Cattle Egret	2018/6/22	1	0	18.8366340	83.8506902	https://www.inaturalist.org/observations/84161783

1825	Bubulcus ibis	Cattle Egret	2022/3/29	3	0	18.8412056	81.3555495	https://www.inaturalist.org/observations/109928279
1826	Bubulcus ibis	Cattle Egret	2012/11/25	1	0	18.8910379	73.1219185	https://www.inaturalist.org/observations/41608774
1827	Bubulcus ibis	Cattle Egret	2008/2/9	4	0	18.8929120	73.1169840	https://www.inaturalist.org/observations/69133009
1828	Bubulcus ibis	Cattle Egret	2016/10/23	1	0	18.8999280	72.9520270	https://www.inaturalist.org/observations/100514356
1829	Bubulcus ibis	Cattle Egret	2011/11/27	2	0	18.9118144	72.8247150	https://www.inaturalist.org/observations/102933085
1830	Bubulcus ibis	Cattle Egret	2018/6/9	3	0	18.9157481	73.2936764	https://www.inaturalist.org/observations/54511188
1831	Bubulcus ibis	Cattle Egret	2015/1/25	4	0	18.9666700	72.8333300	http://www.inaturalist.org/observations/1239378
1832	Bubulcus ibis	Cattle Egret	2021/12/18	3	0	18.9785477	72.8359150	https://www.inaturalist.org/observations/105599154
1833	Bubulcus ibis	Cattle Egret	2017/12/3	1	0	18.9787099	72.8351209	https://www.inaturalist.org/observations/102405201
1834	Bubulcus ibis	Cattle Egret	2020/10/27	3	0	18.9919750	75.7158875	https://www.inaturalist.org/observations/63628564
1835	Bubulcus ibis coromandus	Cattle Egret	2019/8/11	2	0	18.9938850	83.4446400	https://www.inaturalist.org/observations/34634637
1836	Bubulcus ibis	Cattle Egret	2011/8/21	4	0	19.0021303	73.3026999	https://www.inaturalist.org/observations/36850786
1837	Bubulcus ibis	Cattle Egret	2022/6/22	4	0	19.0244600	77.9583067	https://www.inaturalist.org/observations/122924669
1838	Bubulcus ibis	Cattle Egret	2022/6/15	3	0	19.0247782	77.9570207	https://www.inaturalist.org/observations/122659838
1839	Bubulcus ibis	Cattle Egret	2022/6/17	3	0	19.0248633	77.9566133	https://www.inaturalist.org/observations/122128400
1840	Bubulcus ibis coromandus	Cattle Egret	2014/7/27	1	0	19.0267148	73.0615956	https://www.inaturalist.org/observations/88910005
1841	Bubulcus ibis	Cattle Egret	2018/1/5	5	0	19.0306568	72.8422744	https://www.inaturalist.org/observations/20938541
1842	Bubulcus ibis	Cattle Egret	2022/7/30	1	0	19.0358268	73.0412453	https://www.inaturalist.org/observations/129007857
1843	Bubulcus ibis	Cattle Egret	2013/3/3	3	0	19.0515824	72.8575411	https://www.inaturalist.org/observations/32856542
1844	Bubulcus ibis	Cattle Egret	2017/12/10	2	0	19.0522582	72.8627641	https://www.inaturalist.org/observations/102933796
1845	Bubulcus ibis	Cattle Egret	2017/9/9	2	0	19.0522583	72.8627641	https://www.inaturalist.org/observations/43306500
1846	Bubulcus ibis	Cattle Egret	2019/9/14	1	0	19.0615987	108.7246081	https://www.inaturalist.org/observations/32937021
1847	Bubulcus ibis	Cattle Egret	2019/9/14	2	0	19.0615987	108.7246081	https://www.inaturalist.org/observations/32937023
1848	Bubulcus ibis	Cattle Egret	2021/5/29	2	0	19.0616064	72.9069709	https://www.inaturalist.org/observations/84033260
1849	Bubulcus ibis	Cattle Egret	2019/9/8	1	0	19.0624417	72.8329000	https://www.inaturalist.org/observations/32332537

1850	Bubulcus ibis coromandus	Cattle Egret	2019/8/12	2	0	19.1039717	83.8859800	https://www.inaturalist.org/observations/34635808
1851	Bubulcus ibis coromandus	Cattle Egret	2022/2/17	0	0	19.1210867	77.3011583	https://www.inaturalist.org/observations/106902172
1852	Bubulcus ibis	Cattle Egret	2019/1/20	6	0	19.1242962	72.8343964	https://www.inaturalist.org/observations/19747582
1853	Bubulcus ibis	Cattle Egret	2020/9/6	4	0	19.1312500	72.8177633	https://www.inaturalist.org/observations/58706726
1854	Bubulcus ibis	Cattle Egret	2011/3/7	2	0	19.1321583	72.9011631	https://www.inaturalist.org/observations/66843351
1855	Bubulcus ibis	Cattle Egret	2020/10/28	1	0	19.1366280	110.3875590	https://www.inaturalist.org/observations/65982845
1856	Bubulcus ibis	Cattle Egret	2022/8/24	3	0	19.1416891	72.9626396	https://www.inaturalist.org/observations/132145412
1857	Bubulcus ibis	Cattle Egret	2016/7/13	6	0	19.1419038	72.9646254	https://www.inaturalist.org/observations/30478429
1858	Bubulcus ibis	Cattle Egret	2018/10/26	5	0	19.1419038	72.9646254	https://www.inaturalist.org/observations/31473348
1859	Bubulcus ibis	Cattle Egret	2020/10/10	3	0	19.1419055	72.9646284	https://www.inaturalist.org/observations/62186492
1860	Bubulcus ibis	Cattle Egret	2018/11/2	2	0	19.1419248	72.9646254	https://www.inaturalist.org/observations/56300018
1861	Bubulcus ibis	Cattle Egret	2018/11/7	2	0	19.1419248	72.9646254	https://www.inaturalist.org/observations/56300052
1862	Bubulcus ibis	Cattle Egret	2017/12/2	2	0	19.1419248	72.9646254	https://www.inaturalist.org/observations/56406526
1863	Bubulcus ibis	Cattle Egret	2019/5/8	2	0	19.1419248	72.9646254	https://www.inaturalist.org/observations/56496798
1864	Bubulcus ibis	Cattle Egret	2019/5/8	2	0	19.1419248	72.9646254	https://www.inaturalist.org/observations/56496803
1865	Bubulcus ibis	Cattle Egret	2020/6/18	4	0	19.1443969	72.8179441	https://www.inaturalist.org/observations/58146581
1866	Bubulcus ibis	Cattle Egret	2011/5/17	1	0	19.1473831	73.0406335	https://www.inaturalist.org/observations/41616272
1867	Bubulcus ibis	Cattle Egret	2022/10/1	3	0	19.1485040	73.1559946	https://www.inaturalist.org/observations/137480861
1868	Bubulcus ibis	Cattle Egret	2015/11/8	1	0	19.1592080	73.0572893	https://www.inaturalist.org/observations/101343647
1869	Bubulcus ibis	Cattle Egret	2020/9/21	1	0	19.1665044	73.0807402	https://www.inaturalist.org/observations/135214566
1870	Bubulcus ibis	Cattle Egret	2019/12/28	4	0	19.1729238	82.1504840	https://www.inaturalist.org/observations/37579716
1871	Bubulcus ibis	Cattle Egret	2020/1/13	0	0	19.1782430	108.6903734	https://www.inaturalist.org/observations/37834241
1872	Bubulcus ibis	Cattle Egret	2018/9/20	1	0	19.1834895	72.9780731	https://www.inaturalist.org/observations/56307367
1873	Bubulcus ibis	Cattle Egret	2022/10/15	5	0	19.1858766	73.1173198	https://www.inaturalist.org/observations/139116161
1874	Bubulcus ibis	Cattle Egret	2018/9/25	3	0	19.1883417	72.9805413	https://www.inaturalist.org/observations/136704934

1875	Bubulcus ibis	Cattle Egret	2019/5/26	5	0	19.1952752	73.0698015	https://www.inaturalist.org/observations/25809817
1876	Bubulcus ibis	Cattle Egret	2012/9/18	5	0	19.1977659	73.0749615	https://www.inaturalist.org/observations/66918387
1877	Bubulcus ibis	Cattle Egret	2020/3/7	3	0	19.2110382	72.8928026	https://www.inaturalist.org/observations/39839144
1878	Bubulcus ibis	Cattle Egret	2019/4/7	1	0	19.2202450	73.0747660	https://www.inaturalist.org/observations/22134993
1879	Bubulcus ibis coromandus	Cattle Egret	2021/8/10	1	0	19.2231167	72.8711250	https://www.inaturalist.org/observations/90629142
1880	Bubulcus ibis coromandus	Cattle Egret	2021/8/10	1	0	19.2237167	72.8704300	https://www.inaturalist.org/observations/90629098
1881	Bubulcus ibis	Cattle Egret	2018/4/22	5	0	19.2249910	72.8700597	https://www.inaturalist.org/observations/11445637
1882	Bubulcus ibis	Cattle Egret	2005/1/1	4	0	19.2263817	72.8828033	https://www.inaturalist.org/observations/8234480
1883	Bubulcus ibis	Cattle Egret	2021/9/18	2	0	19.2308790	73.1103494	https://www.inaturalist.org/observations/96443686
1884	Bubulcus ibis	Cattle Egret	2017/10/19	2	0	19.2337650	72.9445802	https://www.inaturalist.org/observations/56400421
1885	Bubulcus ibis	Cattle Egret	2020/11/28	2	0	19.2337650	72.9445802	https://www.inaturalist.org/observations/65746598
1886	Bubulcus ibis coromandus	Cattle Egret	2019/8/12	2	0	19.2483017	83.4680850	https://www.inaturalist.org/observations/34635025
1887	Bubulcus ibis coromandus	Cattle Egret	2019/8/12	2	0	19.2483033	83.4680400	https://www.inaturalist.org/observations/34634975
1888	Bubulcus ibis	Cattle Egret	2010/4/11	2	0	19.2569711	73.1239534	https://www.inaturalist.org/observations/42475100
1889	Bubulcus ibis	Cattle Egret	2022/4/30	1	0	19.2612261	73.2235652	https://www.inaturalist.org/observations/113771372
1890	Bubulcus ibis	Cattle Egret	2021/9/21	1	0	19.2651176	73.1723893	https://www.inaturalist.org/observations/117560861
1891	Bubulcus ibis coromandus	Cattle Egret	2019/8/13	3	0	19.2842233	83.4319667	https://www.inaturalist.org/observations/34636412
1892	Bubulcus ibis	Cattle Egret	2009/7/11	5	0	19.2854060	72.9403270	https://www.inaturalist.org/observations/69811762
1893	Bubulcus ibis	Cattle Egret	2021/5/24	3	0	19.2855606	72.8343878	https://www.inaturalist.org/observations/80078813
1894	Bubulcus ibis	Cattle Egret	2021/5/4	2	0	19.3042315	84.8039285	https://www.inaturalist.org/observations/77249441
1895	Bubulcus ibis	Cattle Egret	2021/8/3	3	0	19.3210756	84.7969919	https://www.inaturalist.org/observations/89747581
1896	Bubulcus ibis	Cattle Egret	2021/10/6	1	0	19.3263653	84.7918260	https://www.inaturalist.org/observations/97338661
1897	Bubulcus ibis coromandus	Cattle Egret	2019/8/13	3	0	19.3486100	83.4452017	https://www.inaturalist.org/observations/34636568
1898	Bubulcus ibis	Cattle Egret	2017/12/31	4	0	19.3824677	72.7717775	https://www.inaturalist.org/observations/30817774
1899	Bubulcus ibis	Cattle Egret	2017/10/8	2	0	19.3824677	72.7717775	https://www.inaturalist.org/observations/42584311

1900	Bubulcus ibis	Cattle Egret	2018/10/14	2	0	19.3824677	72.7717775	https://www.inaturalist.org/observations/42587072
1901	Bubulcus ibis coromandus	Cattle Egret	2021/8/7	1	0	19.4022783	72.7731467	https://www.inaturalist.org/observations/90232190
1902	Bubulcus ibis coromandus	Cattle Egret	2021/8/7	1	0	19.4025717	72.7724300	https://www.inaturalist.org/observations/90232109
1903	Bubulcus ibis	Cattle Egret	2021/9/20	1	0	19.4066277	72.8394477	https://www.inaturalist.org/observations/97217296
1904	Bubulcus ibis coromandus	Cattle Egret	2019/8/19	3	0	19.4173217	85.0717633	https://www.inaturalist.org/observations/35542719
1905	Bubulcus ibis	Cattle Egret	2022/5/16	4	0	19.4192068	81.6567947	https://www.inaturalist.org/observations/117397661
1906	Bubulcus ibis	Cattle Egret	2019/6/5	6	0	19.4220610	72.8913374	https://www.inaturalist.org/observations/27014661
1907	Bubulcus ibis	Cattle Egret	2017/4/7	1	0	19.4392830	108.8474500	https://www.inaturalist.org/observations/64917616
1908	Bubulcus ibis	Cattle Egret	2021/10/4	1	0	19.4392830	108.8474500	https://www.inaturalist.org/observations/98014399
1909	Bubulcus ibis coromandus	Cattle Egret	2019/8/13	3	0	19.4813433	83.5224250	https://www.inaturalist.org/observations/34637217
1910	Bubulcus ibis	Cattle Egret	2019/8/13	3	0	19.4813533	83.5224333	https://www.inaturalist.org/observations/34637218
1911	Bubulcus ibis	Cattle Egret	2015/3/7	5	0	19.4835620	73.2595983	https://www.inaturalist.org/observations/60056393
1912	Bubulcus ibis coromandus	Cattle Egret	2019/8/13	3	0	19.4881700	83.4693817	https://www.inaturalist.org/observations/34636919
1913	Bubulcus ibis	Cattle Egret	2022/4/13	2	0	19.5009285	73.0146642	https://www.inaturalist.org/observations/111430853
1914	Bubulcus ibis	Cattle Egret	2022/10/27	3	0	19.5078544	73.0187007	https://www.inaturalist.org/observations/143648802
1915	Bubulcus ibis	Cattle Egret	2022/5/21	3	0	19.5155424	83.5074497	https://www.inaturalist.org/observations/117965384
1916	Bubulcus ibis	Cattle Egret	2019/4/21	1	0	19.5453272	73.2630382	https://www.inaturalist.org/observations/44126242
1917	Bubulcus ibis	Cattle Egret	2022/10/16	2	0	19.5618896	81.6756363	https://www.inaturalist.org/observations/139597551
1918	Bubulcus ibis	Cattle Egret	2022/7/3	2	0	19.5867119	81.6708908	https://www.inaturalist.org/observations/124872774
1919	Bubulcus ibis coromandus	Cattle Egret	2019/8/5	2	0	19.5943379	81.2857457	https://www.inaturalist.org/observations/32234731
1920	Bubulcus ibis	Cattle Egret	2022/1/25	1	0	19.7529942	109.2186265	https://www.inaturalist.org/observations/122940534
1921	Bubulcus ibis	Cattle Egret	2020/10/2	1	0	19.7766099	109.2008285	https://www.inaturalist.org/observations/64423111
1922	Bubulcus ibis	Cattle Egret	2019/9/30	2	0	19.8500287	82.6125997	https://www.inaturalist.org/observations/33636132
1923	Bubulcus ibis	Cattle Egret	2021/5/21	1	0	19.8653412	82.6968384	https://www.inaturalist.org/observations/79648041
1924	Bubulcus ibis	Cattle Egret	2021/5/21	1	0	19.8689393	82.6293576	https://www.inaturalist.org/observations/79615429

1925	Bubulcus ibis coromandus	Cattle Egret	2019/8/20	4	0	19.8865350	86.0959633	https://www.inaturalist.org/observations/35545801
1926	Bubulcus ibis	Cattle Egret	2013/11/17	1	0	19.8870563	86.0940468	https://www.inaturalist.org/observations/68547890
1927	Bubulcus ibis	Cattle Egret	2012/3/31	4	0	19.8871450	86.0948750	http://www.inaturalist.org/observations/699325
1928	Bubulcus ibis	Cattle Egret	2016/12/12	2	0	19.8878935	86.0948427	https://www.inaturalist.org/observations/110217226
1929	Bubulcus ibis	Cattle Egret	2022/4/1	3	0	19.8891016	86.0946126	https://www.inaturalist.org/observations/110169139
1930	Bubulcus ibis	Cattle Egret	2021/2/23	3	0	19.8944738	82.6608819	https://www.inaturalist.org/observations/70020668
1931	Bubulcus ibis	Cattle Egret	2018/4/29	3	0	19.8956382	82.6658096	https://www.inaturalist.org/observations/15994901
1932	Bubulcus ibis	Cattle Egret	2018/10/4	4	0	19.8961867	82.6523300	https://www.inaturalist.org/observations/17270432
1933	Bubulcus ibis	Cattle Egret	2019/3/25	3	0	19.8968267	82.6557783	https://www.inaturalist.org/observations/21583159
1934	Bubulcus ibis	Cattle Egret	2020/10/28	3	0	19.9081766	75.3221694	https://www.inaturalist.org/observations/63718753
1935	Bubulcus ibis	Cattle Egret	2021/5/20	4	0	19.9137036	82.6432294	https://www.inaturalist.org/observations/79511616
1936	Bubulcus ibis	Cattle Egret	2021/4/18	2	0	19.9194047	85.4254317	https://www.inaturalist.org/observations/101935740
1937	Bubulcus ibis	Cattle Egret	2021/4/13	1	0	19.9280896	82.4361736	https://www.inaturalist.org/observations/73834908
1938	Bubulcus ibis	Cattle Egret	2022/8/5	3	0	19.9745197	82.4021317	https://www.inaturalist.org/observations/129622661
1939	Bubulcus ibis	Cattle Egret	2018/12/18	3	0	20.0086157	74.1323815	https://www.inaturalist.org/observations/24757641
1940	Bubulcus ibis	Cattle Egret	2018/12/18	3	0	20.0089632	74.1323479	https://www.inaturalist.org/observations/24757627
1941	Bubulcus ibis	Cattle Egret	2021/11/7	1	0	20.1127338	79.1144726	https://www.inaturalist.org/observations/100492927
1942	Bubulcus ibis	Cattle Egret	2019/9/27	3	0	20.1702862	85.6848068	https://www.inaturalist.org/observations/33487038
1943	Bubulcus ibis	Cattle Egret	2022/9/27	1	0	20.1719856	85.6863695	https://www.inaturalist.org/observations/136687482
1944	Bubulcus ibis coromandus	Cattle Egret	2015/6/16	5	0	20.2006682	79.3418884	https://www.inaturalist.org/observations/12182688
1945	Bubulcus ibis	Cattle Egret	2010/5/25	2	0	20.2483827	79.3606890	https://www.inaturalist.org/observations/55252623
1946	Bubulcus ibis	Cattle Egret	2016/6/4	3	0	20.2483827	79.3606890	https://www.inaturalist.org/observations/55505166
1947	Bubulcus ibis	Cattle Egret	2022/5/8	3	0	20.2483827	79.3606890	https://www.inaturalist.org/observations/135770805
1948	Bubulcus ibis	Cattle Egret	2022/1/6	3	0	20.3051463	85.8388361	https://www.inaturalist.org/observations/104441505
1949	Bubulcus ibis	Cattle Egret	2022/7/25	1	0	20.3334433	81.7633283	https://www.inaturalist.org/observations/129117053

1950	Bubulcus ibis	Cattle Egret	2014/6/25	3	0	20.3400256	79.3059006	https://www.inaturalist.org/observations/58578981
1951	Bubulcus ibis	Cattle Egret	2018/5/14	3	0	20.3496852	79.3252978	https://www.inaturalist.org/observations/52550526
1952	Bubulcus ibis	Cattle Egret	2016/12/16	1	0	20.3959093	85.8272958	https://www.inaturalist.org/observations/110401498
1953	Bubulcus ibis	Cattle Egret	2021/7/18	1	0	20.6291153	81.6395438	https://www.inaturalist.org/observations/134156712
1954	Bubulcus ibis	Cattle Egret	2020/9/13	5	0	20.6661768	77.1049693	https://www.inaturalist.org/observations/59414796
1955	Bubulcus ibis	Cattle Egret	2020/9/13	3	0	20.6662071	77.1054101	https://www.inaturalist.org/observations/59414797
1956	Bubulcus ibis	Cattle Egret	2020/8/13	3	0	20.6926384	77.0173962	https://www.inaturalist.org/observations/56299543
1957	Bubulcus ibis	Cattle Egret	2020/8/13	2	0	20.6927670	77.0174452	https://www.inaturalist.org/observations/56299115
1958	Bubulcus ibis	Cattle Egret	2019/11/5	1	0	20.7012302	116.7280890	https://www.inaturalist.org/observations/35411721
1959	Bubulcus ibis	Cattle Egret	2021/11/11	1	0	20.7226997	81.1902362	https://www.inaturalist.org/observations/100865444
1960	Bubulcus ibis	Cattle Egret	2021/1/24	1	0	20.7347168	81.2031671	https://www.inaturalist.org/observations/143848742
1961	Bubulcus ibis	Cattle Egret	2021/2/9	0	0	20.8200227	81.8379760	https://www.inaturalist.org/observations/69297083
1962	Bubulcus ibis	Cattle Egret	2022/11/30	3	0	20.8270286	81.7131929	https://www.inaturalist.org/observations/143341689
1963	Bubulcus ibis	Cattle Egret	2022/8/23	4	0	20.8280568	82.0597946	https://www.inaturalist.org/observations/133573196
1964	Bubulcus ibis	Cattle Egret	2022/8/23	4	0	20.8280568	82.0597946	https://www.inaturalist.org/observations/133573197
1965	Bubulcus ibis	Cattle Egret	2021/11/9	2	0	20.8303945	81.7084430	https://www.inaturalist.org/observations/100836751
1966	Bubulcus ibis	Cattle Egret	2012/11/28	1	0	20.8417114	77.9147708	https://www.inaturalist.org/observations/85593389
1967	Bubulcus ibis	Cattle Egret	2021/7/10	1	0	20.8431415	85.1349711	https://www.inaturalist.org/observations/87368313
1968	Bubulcus ibis	Cattle Egret	2021/8/1	2	0	20.8584668	72.8180534	https://www.inaturalist.org/observations/90136311
1969	Bubulcus ibis	Cattle Egret	2021/11/12	2	0	20.9060992	81.8024682	https://www.inaturalist.org/observations/100976671
1970	Bubulcus ibis	Cattle Egret	2022/9/12	2	0	20.9197844	81.7901506	https://www.inaturalist.org/observations/134714185
1971	Bubulcus ibis	Cattle Egret	2020/11/9	2	0	20.9241199	72.9093170	https://www.inaturalist.org/observations/64730101
1972	Bubulcus ibis	Cattle Egret	2021/10/2	1	0	20.9431270	86.1315510	https://www.inaturalist.org/observations/96829912
1973	Bubulcus ibis	Cattle Egret	2022/7/10	4	0	20.9748546	75.5383760	https://www.inaturalist.org/observations/126361289
1974	Bubulcus ibis	Cattle Egret	2021/1/3	2	0	20.9794586	75.5647869	https://www.inaturalist.org/observations/75133471

1975	Bubulcus ibis	Cattle Egret	2020/12/17	3	0	20.9819874	75.5385000	https://www.inaturalist.org/observations/66969741
1976	Bubulcus ibis	Cattle Egret	2020/12/16	2	0	20.9892264	75.5456102	https://www.inaturalist.org/observations/66959513
1977	Bubulcus ibis	Cattle Egret	2021/1/30	1	0	21.0055478	81.4759190	https://www.inaturalist.org/observations/68795992
1978	Bubulcus ibis	Cattle Egret	2020/10/28	1	0	21.0365310	79.0294290	https://www.inaturalist.org/observations/63899981
1979	Bubulcus ibis	Cattle Egret	2020/8/25	3	0	21.0451917	75.8385050	https://www.inaturalist.org/observations/57526126
1980	Bubulcus ibis	Cattle Egret	2022/9/10	1	0	21.0459495	79.0228323	https://www.inaturalist.org/observations/134431270
1981	Bubulcus ibis	Cattle Egret	2020/5/25	2	0	21.0571850	75.8421583	https://www.inaturalist.org/observations/51022797
1982	Bubulcus ibis	Cattle Egret	2021/3/26	1	0	21.0943248	81.0434379	https://www.inaturalist.org/observations/72103403
1983	Bubulcus ibis coromandus	Cattle Egret	2021/1/24	2	0	21.0973559	81.0324030	https://www.inaturalist.org/observations/70797961
1984	Bubulcus ibis	Cattle Egret	2011/10/17	5	0	21.1243054	70.8241507	https://www.inaturalist.org/observations/75530076
1985	Bubulcus ibis	Cattle Egret	2014/2/4	2	0	21.1243054	70.8241507	https://www.inaturalist.org/observations/78322199
1986	Bubulcus ibis	Cattle Egret	2011/10/20	4	0	21.1243054	70.8241507	https://www.inaturalist.org/observations/122262668
1987	Bubulcus ibis	Cattle Egret	2020/7/15	3	0	21.1397493	72.7677978	https://www.inaturalist.org/observations/53217766
1988	Bubulcus ibis	Cattle Egret	2022/9/10	1	0	21.1457620	79.0881545	https://www.inaturalist.org/observations/134431339
1989	Bubulcus ibis	Cattle Egret	2016/6/28	4	0	21.1458005	79.0881545	https://www.inaturalist.org/observations/56120178
1990	Bubulcus ibis	Cattle Egret	2020/2/2	1	0	21.1870321	80.7618636	https://www.inaturalist.org/observations/41530275
1991	Bubulcus ibis	Cattle Egret	2021/1/28	1	0	21.1879299	81.3598150	https://www.inaturalist.org/observations/68709693
1992	Bubulcus ibis	Cattle Egret	2021/2/28	3	0	21.1921990	81.7207810	https://www.inaturalist.org/observations/121441745
1993	Bubulcus ibis	Cattle Egret	2022/7/26	6	0	21.1921990	81.7207810	https://www.inaturalist.org/observations/129294160
1994	Bubulcus ibis	Cattle Egret	2017/8/26	2	0	21.1940648	81.3509414	https://www.inaturalist.org/observations/89188798
1995	Bubulcus ibis	Cattle Egret	2020/5/31	3	0	21.2047328	78.9851469	https://www.inaturalist.org/observations/65261292
1996	Bubulcus ibis	Cattle Egret	2022/6/4	4	0	21.2084446	81.3377379	https://www.inaturalist.org/observations/123869927
1997	Bubulcus ibis	Cattle Egret	2020/2/26	3	0	21.2097899	81.4368154	https://www.inaturalist.org/observations/51404353
1998	Bubulcus ibis	Cattle Egret	2022/10/18	2	0	21.2456966	81.5995950	https://www.inaturalist.org/observations/139198102
1999	Bubulcus ibis	Cattle Egret	2020/2/26	3	0	21.2467117	81.6033325	https://www.inaturalist.org/observations/39918151

2000	Bubulcus ibis	Cattle Egret	2022/6/15	4	0	21.2468599	81.5974452	https://www.inaturalist.org/observations/121903980
2001	Bubulcus ibis	Cattle Egret	2022/8/7	0	0	21.2474248	81.6457994	https://www.inaturalist.org/observations/129995202
2002	Bubulcus ibis	Cattle Egret	2022/8/7	3	0	21.2474248	81.6457994	https://www.inaturalist.org/observations/129995203
2003	Bubulcus ibis	Cattle Egret	2020/6/30	4	0	21.2513844	81.6296413	https://www.inaturalist.org/observations/61108413
2004	Bubulcus ibis	Cattle Egret	2022/7/12	3	0	21.2587629	81.6237831	https://www.inaturalist.org/observations/129098892
2005	Bubulcus ibis	Cattle Egret	2022/7/12	2	0	21.2587629	81.6237831	https://www.inaturalist.org/observations/129098893
2006	Bubulcus ibis	Cattle Egret	2022/7/31	4	0	21.2587629	81.6237831	https://www.inaturalist.org/observations/129304471
2007	Bubulcus ibis	Cattle Egret	2022/8/6	4	0	21.2587629	81.6237831	https://www.inaturalist.org/observations/129991793
2008	Bubulcus ibis	Cattle Egret	2022/8/6	4	0	21.2587629	81.6237831	https://www.inaturalist.org/observations/129991795
2009	Bubulcus ibis	Cattle Egret	2022/8/6	5	0	21.2587629	81.6237831	https://www.inaturalist.org/observations/129991796
2010	Bubulcus ibis	Cattle Egret	2022/8/6	5	0	21.2587629	81.6237831	https://www.inaturalist.org/observations/129991797
2011	Bubulcus ibis	Cattle Egret	2022/8/8	4	0	21.2587629	81.6237831	https://www.inaturalist.org/observations/130111385
2012	Bubulcus ibis	Cattle Egret	2022/8/16	5	0	21.2587629	81.6237831	https://www.inaturalist.org/observations/131339930
2013	Bubulcus ibis	Cattle Egret	2022/8/16	5	0	21.2587629	81.6237831	https://www.inaturalist.org/observations/131340050
2014	Bubulcus ibis	Cattle Egret	2022/8/21	4	0	21.2587629	81.6237831	https://www.inaturalist.org/observations/131788379
2015	Bubulcus ibis	Cattle Egret	2022/8/21	5	0	21.2587629	81.6237831	https://www.inaturalist.org/observations/131788380
2016	Bubulcus ibis	Cattle Egret	2022/8/21	4	0	21.2587629	81.6237831	https://www.inaturalist.org/observations/131788424
2017	Bubulcus ibis	Cattle Egret	2022/8/21	2	0	21.2587629	81.6237831	https://www.inaturalist.org/observations/131788462
2018	Bubulcus ibis	Cattle Egret	2022/8/23	3	0	21.2587629	81.6237831	https://www.inaturalist.org/observations/133427623
2019	Bubulcus ibis	Cattle Egret	2022/8/26	3	0	21.2587629	81.6237831	https://www.inaturalist.org/observations/133427626
2020	Bubulcus ibis	Cattle Egret	2022/8/26	2	0	21.2587629	81.6237831	https://www.inaturalist.org/observations/133427628
2021	Bubulcus ibis	Cattle Egret	2022/8/26	2	0	21.2587629	81.6237831	https://www.inaturalist.org/observations/133427630
2022	Bubulcus ibis	Cattle Egret	2022/8/26	4	0	21.2587629	81.6237831	https://www.inaturalist.org/observations/133427632
2023	Bubulcus ibis	Cattle Egret	2022/8/26	3	0	21.2587629	81.6237831	https://www.inaturalist.org/observations/133427633
2024	Bubulcus ibis	Cattle Egret	2022/8/26	2	0	21.2587629	81.6237831	https://www.inaturalist.org/observations/133427638

2025	Bubulcus ibis	Cattle Egret	2022/8/26	2	0	21.2587629	81.6237831	https://www.inaturalist.org/observations/133427640
2026	Bubulcus ibis	Cattle Egret	2022/8/23	2	0	21.2587629	81.6237831	https://www.inaturalist.org/observations/133427641
2027	Bubulcus ibis	Cattle Egret	2022/9/2	4	0	21.2587629	81.6237831	https://www.inaturalist.org/observations/134198430
2028	Bubulcus ibis	Cattle Egret	2021/7/30	3	0	21.2600146	81.3585033	https://www.inaturalist.org/observations/89650917
2029	Bubulcus ibis	Cattle Egret	2021/8/27	1	0	21.2600146	81.3585033	https://www.inaturalist.org/observations/93383479
2030	Bubulcus ibis	Cattle Egret	2022/7/5	3	0	21.2674596	81.6184959	https://www.inaturalist.org/observations/124881421
2031	Bubulcus ibis	Cattle Egret	2022/7/8	2	0	21.2766770	81.6446049	https://www.inaturalist.org/observations/125802347
2032	Bubulcus ibis	Cattle Egret	2022/11/30	0	0	21.2920803	82.3515606	https://www.inaturalist.org/observations/143721703
2033	Bubulcus ibis	Cattle Egret	2020/10/25	3	0	21.2977170	80.3498170	https://www.inaturalist.org/observations/63689563
2034	Bubulcus ibis	Cattle Egret	2022/8/10	0	0	21.3038044	81.6362152	https://www.inaturalist.org/observations/130207229
2035	Bubulcus ibis	Cattle Egret	2022/8/10	2	0	21.3040504	81.6382523	https://www.inaturalist.org/observations/130213314
2036	Bubulcus ibis	Cattle Egret	2022/8/24	0	0	21.3040543	81.6382523	https://www.inaturalist.org/observations/132160077
2037	Bubulcus ibis	Cattle Egret	2022/7/15	2	0	21.3042240	81.6385198	https://www.inaturalist.org/observations/126446399
2038	Bubulcus ibis	Cattle Egret	2022/8/4	0	0	21.3042240	81.6385198	https://www.inaturalist.org/observations/129418218
2039	Bubulcus ibis	Cattle Egret	2022/8/4	0	0	21.3042240	81.6385198	https://www.inaturalist.org/observations/129418668
2040	Bubulcus ibis	Cattle Egret	2022/9/2	3	0	21.3321217	81.7229883	https://www.inaturalist.org/observations/133308008
2041	Bubulcus ibis	Cattle Egret	2019/2/24	1	0	21.4124738	72.6830156	https://www.inaturalist.org/observations/88249067
2042	Bubulcus ibis	Cattle Egret	2022/6/28	5	0	21.4487577	81.1579638	https://www.inaturalist.org/observations/124278503
2043	Bubulcus ibis	Cattle Egret	2022/6/4	3	0	21.4749138	79.3212058	https://www.inaturalist.org/observations/121057090
2044	Bubulcus ibis	Cattle Egret	2019/6/5	2	0	21.5201321	81.0295853	https://www.inaturalist.org/observations/74532388
2045	Bubulcus ibis	Cattle Egret	2020/6/6	2	0	21.5265545	70.4586736	https://www.inaturalist.org/observations/48625570
2046	Bubulcus ibis coromandus	Cattle Egret	2021/7/16	1	0	21.5387410	70.5027647	https://www.inaturalist.org/observations/87232023
2047	Bubulcus ibis	Cattle Egret	2020/2/14	1	0	21.5631303	88.2595023	https://www.inaturalist.org/observations/40946508
2048	Bubulcus ibis	Cattle Egret	2020/2/14	0	0	21.5631304	88.2595022	https://www.inaturalist.org/observations/39744039
2049	Bubulcus ibis	Cattle Egret	2020/2/14	4	0	21.5650823	88.2490489	https://www.inaturalist.org/observations/39919825

2050	Bubulcus ibis	Cattle Egret	2020/2/14	1	0	21.5706191	88.2430140	https://www.inaturalist.org/observations/58235233
2051	Bubulcus ibis	Cattle Egret	2020/2/14	1	0	21.5706191	88.2430140	https://www.inaturalist.org/observations/58235235
2052	Bubulcus ibis	Cattle Egret	2020/2/14	1	0	21.5706191	88.2430140	https://www.inaturalist.org/observations/58235237
2053	Bubulcus ibis coromandus	Cattle Egret	2021/2/7	0	0	21.5794933	88.2512208	https://www.inaturalist.org/observations/97936591
2054	Bubulcus ibis	Cattle Egret	2015/12/29	2	0	21.6391102	87.5531080	https://www.inaturalist.org/observations/61106553
2055	Bubulcus ibis	Cattle Egret	2018/10/15	5	0	21.6519658	71.8581503	https://www.inaturalist.org/observations/66366094
2056	Bubulcus ibis	Cattle Egret	2020/5/17	4	0	21.6622222	71.8525000	https://www.inaturalist.org/observations/46373543
2057	Bubulcus ibis	Cattle Egret	2020/3/8	2	0	21.7257909	73.0074705	https://www.inaturalist.org/observations/63693886
2058	Bubulcus ibis	Cattle Egret	2014/4/9	3	0	21.7560486	72.1257072	https://www.inaturalist.org/observations/39749665
2059	Bubulcus ibis	Cattle Egret	2021/1/23	2	0	21.7705556	108.1697222	https://www.inaturalist.org/observations/68490423
2060	Bubulcus ibis	Cattle Egret	2021/5/21	1	0	21.8190129	83.3121738	https://www.inaturalist.org/observations/79642152
2061	Bubulcus ibis	Cattle Egret	2021/5/7	1	0	21.8190713	83.3120839	https://www.inaturalist.org/observations/79921565
2062	Bubulcus ibis	Cattle Egret	2021/8/4	1	0	21.8193250	83.3120850	https://www.inaturalist.org/observations/89949271
2063	Bubulcus ibis	Cattle Egret	2021/8/8	1	0	21.8194217	83.3118133	https://www.inaturalist.org/observations/92207590
2064	Bubulcus ibis	Cattle Egret	2022/11/26	2	0	21.8493162	84.0479835	https://www.inaturalist.org/observations/142895453
2065	Bubulcus ibis	Cattle Egret	2022/11/26	1	0	21.8493268	84.0480390	https://www.inaturalist.org/observations/142895416
2066	Bubulcus ibis	Cattle Egret	2021/5/21	2	0	21.8507517	81.8227156	https://www.inaturalist.org/observations/79566128
2067	Bubulcus ibis	Cattle Egret	2019/1/2	5	0	21.8839254	80.5136326	https://www.inaturalist.org/observations/19435645
2068	Bubulcus ibis	Cattle Egret	2020/6/4	3	0	21.8884500	83.4030040	https://www.inaturalist.org/observations/69038619
2069	Bubulcus ibis	Cattle Egret	2020/12/22	1	0	21.8973977	83.3949631	https://www.inaturalist.org/observations/79749214
2070	Bubulcus ibis	Cattle Egret	2019/10/12	1	0	21.9260667	120.8475633	https://www.inaturalist.org/observations/34292507
2071	Bubulcus ibis	Cattle Egret	2019/10/14	1	0	21.9272344	120.8479628	https://www.inaturalist.org/observations/39690114
2072	Bubulcus ibis	Cattle Egret	2019/10/3	1	0	21.9365685	120.7320223	https://www.inaturalist.org/observations/33976774
2073	Bubulcus ibis	Cattle Egret	2019/10/3	1	0	21.9366665	120.7321167	https://www.inaturalist.org/observations/34066219
2074	Bubulcus ibis	Cattle Egret	2019/10/7	1	0	21.9422628	120.8272304	https://www.inaturalist.org/observations/34336399

2075	Bubulcus ibis	Cattle Egret	2019/9/16	1	0	21.9437800	120.8248579	https://www.inaturalist.org/observations/32827480
2076	Bubulcus ibis	Cattle Egret	2021/10/22	1	0	21.9444301	120.8245252	https://www.inaturalist.org/observations/99202460
2077	Bubulcus ibis	Cattle Egret	2022/9/18	2	0	21.9448278	120.7944111	https://www.inaturalist.org/observations/135632387
2078	Bubulcus ibis	Cattle Egret	2016/10/28	4	0	21.9487122	120.7805264	http://www.inaturalist.org/observations/4696248
2079	Bubulcus ibis coromandus	Cattle Egret	2020/10/18	1	0	21.9531559	120.8226981	https://www.inaturalist.org/observations/63374887
2080	Bubulcus ibis	Cattle Egret	2021/10/10	1	0	21.9533948	120.8233666	https://www.inaturalist.org/observations/97886498
2081	Bubulcus ibis	Cattle Egret	2022/1/9	1	0	21.9563698	120.8189188	https://www.inaturalist.org/observations/104693615
2082	Bubulcus ibis	Cattle Egret	2019/9/16	1	0	21.9563800	120.8189034	https://www.inaturalist.org/observations/32827488
2083	Bubulcus ibis	Cattle Egret	2019/10/8	2	0	21.9699614	120.7490319	https://www.inaturalist.org/observations/34095719
2084	Bubulcus ibis	Cattle Egret	2019/12/1	1	0	21.9704520	120.7485440	https://www.inaturalist.org/observations/45761759
2085	Bubulcus ibis	Cattle Egret	2019/11/18	2	0	21.9791195	120.7277359	https://www.inaturalist.org/observations/35853242
2086	Bubulcus ibis	Cattle Egret	2019/11/14	1	0	21.9829044	120.7353493	https://www.inaturalist.org/observations/35917168
2087	Bubulcus ibis	Cattle Egret	2019/11/12	1	0	21.9873501	120.7399138	https://www.inaturalist.org/observations/35607449
2088	Bubulcus ibis	Cattle Egret	2021/10/20	2	0	22.0002783	81.2208400	https://www.inaturalist.org/observations/98792655
2089	Bubulcus ibis	Cattle Egret	2018/10/10	1	0	22.0015984	120.8190189	https://www.inaturalist.org/observations/17491530
2090	Bubulcus ibis	Cattle Egret	2019/10/5	1	0	22.0131431	120.8047458	https://www.inaturalist.org/observations/87650544
2091	Bubulcus ibis	Cattle Egret	2021/9/5	1	0	22.0131925	81.2214228	https://www.inaturalist.org/observations/93968588
2092	Bubulcus ibis	Cattle Egret	2019/10/5	1	0	22.0132781	120.8049242	https://www.inaturalist.org/observations/87649739
2093	Bubulcus ibis	Cattle Egret	2022/7/7	4	0	22.0158899	72.1254608	https://www.inaturalist.org/observations/131547379
2094	Bubulcus ibis	Cattle Egret	2021/10/10	1	0	22.0190372	120.8429264	https://www.inaturalist.org/observations/97886503
2095	Bubulcus ibis	Cattle Egret	2011/10/10	2	0	22.0192131	120.8425447	https://www.inaturalist.org/observations/59457244
2096	Bubulcus ibis	Cattle Egret	2019/10/19	0	0	22.0220870	120.8349630	https://www.inaturalist.org/observations/38084280
2097	Bubulcus ibis	Cattle Egret	2019/10/11	0	0	22.0238833	120.8611000	https://www.inaturalist.org/observations/37973803
2098	Bubulcus ibis	Cattle Egret	2017/4/16	3	0	22.0264389	73.0906827	https://www.inaturalist.org/observations/89290705
2099	Bubulcus ibis	Cattle Egret	2019/9/8	1	0	22.0295120	120.7777817	https://www.inaturalist.org/observations/32333157

2100	Bubulcus ibis	Cattle Egret	2019/9/28	1	0	22.0328670	120.7169331	https://www.inaturalist.org/observations/33587915
2101	Bubulcus ibis	Cattle Egret	2020/9/5	1	0	22.0341968	121.5743335	https://www.inaturalist.org/observations/59033952
2102	Bubulcus ibis	Cattle Egret	2022/9/19	2	0	22.0346950	121.5737770	https://www.inaturalist.org/observations/136532858
2103	Bubulcus ibis	Cattle Egret	2017/5/22	3	0	22.0435616	121.5484180	https://www.inaturalist.org/observations/6547455
2104	Bubulcus ibis	Cattle Egret	2011/1/26	2	0	22.0441079	72.0202813	https://www.inaturalist.org/observations/59217790
2105	Bubulcus ibis	Cattle Egret	2021/8/20	1	0	22.0578650	82.0578733	https://www.inaturalist.org/observations/93701539
2106	Bubulcus ibis	Cattle Egret	2021/8/20	1	0	22.0584233	82.0635517	https://www.inaturalist.org/observations/93694719
2107	Bubulcus ibis	Cattle Egret	2021/8/20	1	0	22.0584233	82.0635517	https://www.inaturalist.org/observations/93694721
2108	Bubulcus ibis	Cattle Egret	2021/9/3	1	0	22.0585133	82.2098783	https://www.inaturalist.org/observations/93737515
2109	Bubulcus ibis	Cattle Egret	2019/11/12	1	0	22.0585284	120.7299035	https://www.inaturalist.org/observations/35607866
2110	Bubulcus ibis	Cattle Egret	2019/2/2	3	0	22.0597151	82.0434380	https://www.inaturalist.org/observations/60722946
2111	Bubulcus ibis	Cattle Egret	2019/7/12	4	0	22.0597151	82.0434380	https://www.inaturalist.org/observations/62090775
2112	Bubulcus ibis	Cattle Egret	2008/10/19	1	0	22.0612933	121.5111341	https://www.inaturalist.org/observations/126207270
2113	Bubulcus ibis	Cattle Egret	2021/9/5	0	0	22.0630517	82.2011000	https://www.inaturalist.org/observations/93742342
2114	Bubulcus ibis	Cattle Egret	2021/8/31	1	0	22.0659000	82.2051650	https://www.inaturalist.org/observations/93439021
2115	Bubulcus ibis	Cattle Egret	2021/8/31	1	0	22.0659000	82.2051650	https://www.inaturalist.org/observations/93439074
2116	Bubulcus ibis	Cattle Egret	2019/9/1	2	0	22.0720830	120.7188988	https://www.inaturalist.org/observations/31903295
2117	Bubulcus ibis	Cattle Egret	2019/4/7	1	0	22.0740291	120.7223755	https://www.inaturalist.org/observations/22133802
2118	Bubulcus ibis	Cattle Egret	2016/3/14	1	0	22.0773072	120.7326128	https://www.inaturalist.org/observations/82393381
2119	Bubulcus ibis	Cattle Egret	2020/9/5	1	0	22.0774898	121.5080571	https://www.inaturalist.org/observations/106670650
2120	Bubulcus ibis	Cattle Egret	2019/6/30	2	0	22.0795040	121.5188441	https://www.inaturalist.org/observations/28199701
2121	Bubulcus ibis	Cattle Egret	2019/10/15	3	0	22.0796545	82.1409152	https://www.inaturalist.org/observations/60488891
2122	Bubulcus ibis	Cattle Egret	2019/6/30	1	0	22.0800492	121.5285098	https://www.inaturalist.org/observations/28600315
2123	Bubulcus ibis	Cattle Egret	2022/4/16	2	0	22.0912181	88.7615667	https://www.inaturalist.org/observations/112701772
2124	Bubulcus ibis	Cattle Egret	2021/11/28	1	0	22.1033917	120.7920972	https://www.inaturalist.org/observations/102068567

2125	Bubulcus ibis	Cattle Egret	2022/12/14	1	0	22.1119457	120.8760168	https://www.inaturalist.org/observations/144334453
2126	Bubulcus ibis	Cattle Egret	2022/12/14	2	0	22.1123176	120.8773247	https://www.inaturalist.org/observations/144727819
2127	Bubulcus ibis	Cattle Egret	2013/10/26	4	0	22.1191298	80.6743047	https://www.inaturalist.org/observations/69360382
2128	Bubulcus ibis	Cattle Egret	2019/9/1	1	0	22.1260510	82.1425522	https://www.inaturalist.org/observations/32244086
2129	Bubulcus ibis	Cattle Egret	2021/8/16	1	0	22.1261445	82.1430395	https://www.inaturalist.org/observations/93694754
2130	Bubulcus ibis	Cattle Egret	2021/9/5	0	0	22.1281650	82.1424983	https://www.inaturalist.org/observations/93746555
2131	Bubulcus ibis	Cattle Egret	2021/8/30	1	0	22.1282217	82.1437117	https://www.inaturalist.org/observations/93206875
2132	Bubulcus ibis	Cattle Egret	2021/8/30	1	0	22.1291300	82.1410217	https://www.inaturalist.org/observations/93206952
2133	Bubulcus ibis	Cattle Egret	2022/5/3	2	0	22.1292283	120.7841195	https://www.inaturalist.org/observations/117393492
2134	Bubulcus ibis	Cattle Egret	2022/5/3	2	0	22.1292669	120.7841568	https://www.inaturalist.org/observations/117393493
2135	Bubulcus ibis	Cattle Egret	2021/8/30	1	0	22.1298850	82.1425800	https://www.inaturalist.org/observations/93206938
2136	Bubulcus ibis	Cattle Egret	2019/9/10	3	0	22.1299364	82.1361662	https://www.inaturalist.org/observations/32519955
2137	Bubulcus ibis	Cattle Egret	2022/5/3	2	0	22.1302403	120.7837341	https://www.inaturalist.org/observations/117393495
2138	Bubulcus ibis	Cattle Egret	2022/5/3	1	0	22.1307917	120.7830592	https://www.inaturalist.org/observations/117393506
2139	Bubulcus ibis	Cattle Egret	2022/5/3	1	0	22.1311171	120.7834531	https://www.inaturalist.org/observations/117393489
2140	Bubulcus ibis	Cattle Egret	2021/9/1	2	0	22.1321883	82.0463317	https://www.inaturalist.org/observations/93451114
2141	Bubulcus ibis	Cattle Egret	2017/5/11	1	0	22.1623045	114.1919963	https://www.inaturalist.org/observations/58299384
2142	Bubulcus ibis	Cattle Egret	2009/5/2	0	0	22.1650590	114.2548540	https://www.inaturalist.org/observations/39483730
2143	Bubulcus ibis	Cattle Egret	2013/4/7	1	0	22.1651360	114.2550490	https://www.inaturalist.org/observations/37551298
2144	Bubulcus ibis	Cattle Egret	2022/4/30	1	0	22.1652941	114.2551501	https://www.inaturalist.org/observations/114052284
2145	Bubulcus ibis	Cattle Egret	2022/5/3	1	0	22.1653661	114.2549622	https://www.inaturalist.org/observations/115249581
2146	Bubulcus ibis	Cattle Egret	2022/4/30	1	0	22.1655021	114.2554062	https://www.inaturalist.org/observations/115517731
2147	Bubulcus ibis	Cattle Egret	2020/4/5	1	0	22.1660801	114.2616417	https://www.inaturalist.org/observations/45564566
2148	Bubulcus ibis	Cattle Egret	2020/5/3	1	0	22.1689984	114.2594818	https://www.inaturalist.org/observations/53104149
2149	Bubulcus ibis	Cattle Egret	2021/9/25	1	0	22.1692320	114.2598530	https://www.inaturalist.org/observations/96885175

2150	Bubulcus ibis	Cattle Egret	2018/1/19	7	0	22.1778762	69.3906560	https://www.inaturalist.org/observations/30347557
2151	Bubulcus ibis	Cattle Egret	2016/11/5	1	0	22.1960974	114.1398459	https://www.inaturalist.org/observations/35942458
2152	Bubulcus ibis	Cattle Egret	2021/10/11	1	0	22.2005170	113.5477910	https://www.inaturalist.org/observations/97867092
2153	Bubulcus ibis coromandus	Cattle Egret	2015/10/16	1	0	22.2030155	114.1309155	https://www.inaturalist.org/observations/32100335
2154	Bubulcus ibis	Cattle Egret	2022/9/22	1	0	22.2039649	113.9616873	https://www.inaturalist.org/observations/136093780
2155	Bubulcus ibis	Cattle Egret	2020/4/23	1	0	22.2042536	114.1274529	https://www.inaturalist.org/observations/43016874
2156	Bubulcus ibis	Cattle Egret	2021/10/11	1	0	22.2043717	114.1256805	https://www.inaturalist.org/observations/97875021
2157	Bubulcus ibis	Cattle Egret	2019/11/1	1	0	22.2043874	114.1250667	https://www.inaturalist.org/observations/35159525
2158	Bubulcus ibis coromandus	Cattle Egret	2020/4/18	1	0	22.2046305	114.1264604	https://www.inaturalist.org/observations/42658799
2159	Bubulcus ibis	Cattle Egret	2020/10/27	2	0	22.2095444	120.7293917	https://www.inaturalist.org/observations/63617450
2160	Bubulcus ibis	Cattle Egret	2020/11/8	1	0	22.2100465	114.2549018	https://www.inaturalist.org/observations/64413871
2161	Bubulcus ibis	Cattle Egret	2022/8/17	2	0	22.2163750	120.7990333	https://www.inaturalist.org/observations/132530811
2162	Bubulcus ibis	Cattle Egret	2017/5/23	4	0	22.2168381	114.2143814	https://www.inaturalist.org/observations/19352422
2163	Bubulcus ibis	Cattle Egret	2022/8/23	1	0	22.2173361	120.8026167	https://www.inaturalist.org/observations/132367080
2164	Bubulcus ibis	Cattle Egret	2017/10/18	2	0	22.2191783	113.9180517	https://www.inaturalist.org/observations/8453425
2165	Bubulcus ibis	Cattle Egret	2021/12/15	1	0	22.2191853	113.9184304	https://www.inaturalist.org/observations/103094226
2166	Bubulcus ibis coromandus	Cattle Egret	2018/4/23	3	0	22.2197143	113.9150734	https://www.inaturalist.org/observations/11498021
2167	Bubulcus ibis	Cattle Egret	2020/4/27	2	0	22.2198773	113.9173066	https://www.inaturalist.org/observations/43847089
2168	Bubulcus ibis	Cattle Egret	2020/4/27	2	0	22.2199245	113.9172626	https://www.inaturalist.org/observations/44082636
2169	Bubulcus ibis	Cattle Egret	2020/4/27	2	0	22.2200000	113.9166667	https://www.inaturalist.org/observations/43846611
2170	Bubulcus ibis	Cattle Egret	2020/4/27	1	0	22.2200000	113.9172222	https://www.inaturalist.org/observations/44476981
2171	Bubulcus ibis	Cattle Egret	2020/9/22	2	0	22.2200211	113.9173376	https://www.inaturalist.org/observations/60510958
2172	Bubulcus ibis	Cattle Egret	2019/10/21	1	0	22.2200620	113.9137750	https://www.inaturalist.org/observations/34815060
2173	Bubulcus ibis	Cattle Egret	2020/4/27	1	0	22.2200883	113.9173800	https://www.inaturalist.org/observations/43899396
2174	Bubulcus ibis	Cattle Egret	2020/4/27	1	0	22.2201655	113.9175070	https://www.inaturalist.org/observations/43846613

2175	Bubulcus ibis	Cattle Egret	2020/4/26	1	0	22.2202033	113.9174500	https://www.inaturalist.org/observations/43576822
2176	Bubulcus ibis	Cattle Egret	2022/9/17	1	0	22.2202131	113.9169266	https://www.inaturalist.org/observations/135494346
2177	Bubulcus ibis	Cattle Egret	2020/4/7	2	0	22.2202967	113.9163667	https://www.inaturalist.org/observations/41606352
2178	Bubulcus ibis	Cattle Egret	2022/4/14	2	0	22.2203700	113.9159500	https://www.inaturalist.org/observations/111391917
2179	Bubulcus ibis	Cattle Egret	2019/10/21	1	0	22.2203900	113.9176370	https://www.inaturalist.org/observations/34992277
2180	Bubulcus ibis	Cattle Egret	2022/4/30	2	0	22.2204083	113.9158950	https://www.inaturalist.org/observations/115353464
2181	Bubulcus ibis	Cattle Egret	2020/4/27	1	0	22.2204559	113.9145034	https://www.inaturalist.org/observations/43915368
2182	Bubulcus ibis	Cattle Egret	2019/10/10	1	0	22.2204694	113.9156779	https://www.inaturalist.org/observations/34153425
2183	Bubulcus ibis coromandus	Cattle Egret	2020/3/30	1	0	22.2204800	113.9160617	https://www.inaturalist.org/observations/41021399
2184	Bubulcus ibis	Cattle Egret	2022/5/2	2	0	22.2208098	113.9154175	https://www.inaturalist.org/observations/115585447
2185	Bubulcus ibis	Cattle Egret	2022/9/22	1	0	22.2208899	113.9155024	https://www.inaturalist.org/observations/137033287
2186	Bubulcus ibis	Cattle Egret	2020/5/23	1	0	22.2211603	114.1168317	https://www.inaturalist.org/observations/46948894
2187	Bubulcus ibis coromandus	Cattle Egret	2020/4/27	0	0	22.2214058	113.9195496	https://www.inaturalist.org/observations/43914645
2188	Bubulcus ibis	Cattle Egret	2022/4/30	1	0	22.2214700	113.9150433	https://www.inaturalist.org/observations/115839555
2189	Bubulcus ibis	Cattle Egret	2022/4/30	1	0	22.2214700	113.9150433	https://www.inaturalist.org/observations/115839557
2190	Bubulcus ibis	Cattle Egret	2022/10/25	2	0	22.2214700	120.8084550	https://www.inaturalist.org/observations/139956750
2191	Bubulcus ibis	Cattle Egret	2022/11/5	3	0	22.2216347	114.1155529	https://www.inaturalist.org/observations/141124202
2192	Bubulcus ibis	Cattle Egret	2018/10/31	4	0	22.2219028	113.9189015	https://www.inaturalist.org/observations/17981379
2193	Bubulcus ibis	Cattle Egret	2020/4/27	1	0	22.2230150	113.9147990	https://www.inaturalist.org/observations/43934409
2194	Bubulcus ibis coromandus	Cattle Egret	2020/4/19	1	0	22.2251437	113.8469127	https://www.inaturalist.org/observations/42833508
2195	Bubulcus ibis coromandus	Cattle Egret	2020/11/8	1	0	22.2260939	113.8471622	https://www.inaturalist.org/observations/64663891
2196	Bubulcus ibis	Cattle Egret	2022/4/15	1	0	22.2262841	113.8943053	https://www.inaturalist.org/observations/111493302
2197	Bubulcus ibis	Cattle Egret	2020/4/25	2	0	22.2265713	113.8479232	https://www.inaturalist.org/observations/44096209
2198	Bubulcus ibis	Cattle Egret	2021/10/16	1	0	22.2267993	113.9041579	https://www.inaturalist.org/observations/98494283
2199	Bubulcus ibis	Cattle Egret	2021/10/12	1	0	22.2271115	114.1132518	https://www.inaturalist.org/observations/97989741

2200	Bubulcus ibis	Cattle Egret	2021/10/26	1	0	22.2273300	113.9326467	https://www.inaturalist.org/observations/99436771
2201	Bubulcus ibis	Cattle Egret	2020/12/30	2	0	22.2277570	113.9322090	https://www.inaturalist.org/observations/67277098
2202	Bubulcus ibis coromandus	Cattle Egret	2017/11/8	2	0	22.2280463	114.1148145	https://www.inaturalist.org/observations/8729392
2203	Bubulcus ibis coromandus	Cattle Egret	2022/5/2	0	0	22.2283222	113.8918146	https://www.inaturalist.org/observations/114990597
2204	Bubulcus ibis	Cattle Egret	2020/4/21	2	0	22.2285058	113.9233828	https://www.inaturalist.org/observations/86982871
2205	Bubulcus ibis coromandus	Cattle Egret	2020/10/7	2	0	22.2289130	113.9364470	https://www.inaturalist.org/observations/61903584
2206	Bubulcus ibis	Cattle Egret	2022/4/26	1	0	22.2308173	113.9678530	https://www.inaturalist.org/observations/113089006
2207	Bubulcus ibis	Cattle Egret	2021/5/1	1	0	22.2358977	114.1138811	https://www.inaturalist.org/observations/76017898
2208	Bubulcus ibis	Cattle Egret	2022/5/2	1	0	22.2361533	114.1914833	https://www.inaturalist.org/observations/115046367
2209	Bubulcus ibis	Cattle Egret	2020/4/26	2	0	22.2384529	114.1170340	https://www.inaturalist.org/observations/43574280
2210	Bubulcus ibis	Cattle Egret	2021/2/5	1	0	22.2388305	113.9906638	https://www.inaturalist.org/observations/69139066
2211	Bubulcus ibis	Cattle Egret	2018/5/6	2	0	22.2389361	114.2229778	https://www.inaturalist.org/observations/12188540
2212	Bubulcus ibis	Cattle Egret	2021/5/3	3	0	22.2390540	114.2232440	https://www.inaturalist.org/observations/76957411
2213	Bubulcus ibis	Cattle Egret	2021/5/3	2	0	22.2391821	114.2233356	https://www.inaturalist.org/observations/76867907
2214	Bubulcus ibis	Cattle Egret	2021/5/3	1	0	22.2393867	114.2232750	https://www.inaturalist.org/observations/76851283
2215	Bubulcus ibis	Cattle Egret	2021/5/3	1	0	22.2396081	114.2234093	https://www.inaturalist.org/observations/76851334
2216	Bubulcus ibis	Cattle Egret	2021/11/21	2	0	22.2397114	113.9768132	https://www.inaturalist.org/observations/123193220
2217	Bubulcus ibis	Cattle Egret	2019/8/24	2	0	22.2403423	113.9784547	https://www.inaturalist.org/observations/31392261
2218	Bubulcus ibis coromandus	Cattle Egret	2019/10/5	1	0	22.2405731	113.9764745	https://www.inaturalist.org/observations/33879643
2219	Bubulcus ibis coromandus	Cattle Egret	2020/1/4	1	0	22.2408945	113.9764599	https://www.inaturalist.org/observations/37302099
2220	Bubulcus ibis	Cattle Egret	2019/12/15	1	0	22.2411203	113.9743986	https://www.inaturalist.org/observations/47483871
2221	Bubulcus ibis	Cattle Egret	2022/2/3	1	0	22.2411295	113.9750055	https://www.inaturalist.org/observations/106065784
2222	Bubulcus ibis	Cattle Egret	2016/3/20	1	0	22.2414488	113.9776663	https://www.inaturalist.org/observations/49217086
2223	Bubulcus ibis	Cattle Egret	2021/5/1	2	0	22.2415669	113.9747405	https://www.inaturalist.org/observations/76517585
2224	Bubulcus ibis	Cattle Egret	2009/11/22	1	0	22.2416380	113.9752500	https://www.inaturalist.org/observations/140744812

2225	Bubulcus ibis	Cattle Egret	2012/4/21	2	0	22.2417602	113.9740333	https://www.inaturalist.org/observations/104640498
2226	Bubulcus ibis coromandus	Cattle Egret	2019/5/13	2	0	22.2417811	113.9752602	https://www.inaturalist.org/observations/25162503
2227	Bubulcus ibis	Cattle Egret	2021/4/18	4	0	22.2418731	81.4292385	https://www.inaturalist.org/observations/75160132
2228	Bubulcus ibis	Cattle Egret	2021/12/19	2	0	22.2419061	113.9749893	https://www.inaturalist.org/observations/103519988
2229	Bubulcus ibis	Cattle Egret	2021/11/29	1	0	22.2420550	113.9749249	https://www.inaturalist.org/observations/102351916
2230	Bubulcus ibis	Cattle Egret	2010/1/1	0	0	22.2420990	113.9758680	https://www.inaturalist.org/observations/39205066
2231	Bubulcus ibis	Cattle Egret	2011/10/16	0	0	22.2424110	113.9796950	https://www.inaturalist.org/observations/38376690
2232	Bubulcus ibis coromandus	Cattle Egret	2020/4/25	1	0	22.2428602	113.9794520	https://www.inaturalist.org/observations/43307870
2233	Bubulcus ibis	Cattle Egret	2021/11/19	1	0	22.2428912	113.9795939	https://www.inaturalist.org/observations/101544572
2234	Bubulcus ibis	Cattle Egret	2020/9/5	2	0	22.2428950	113.9795300	https://www.inaturalist.org/observations/58574224
2235	Bubulcus ibis	Cattle Egret	2020/11/1	1	0	22.2429136	113.9785716	https://www.inaturalist.org/observations/64041762
2236	Bubulcus ibis	Cattle Egret	2020/11/1	1	0	22.2429689	113.9783057	https://www.inaturalist.org/observations/64104488
2237	Bubulcus ibis	Cattle Egret	2020/5/16	1	0	22.2433760	113.9753490	https://www.inaturalist.org/observations/46170631
2238	Bubulcus ibis	Cattle Egret	2022/5/2	2	0	22.2437797	113.9796724	https://www.inaturalist.org/observations/114554653
2239	Bubulcus ibis	Cattle Egret	2021/9/1	2	0	22.2440833	82.0194250	https://www.inaturalist.org/observations/93451117
2240	Bubulcus ibis	Cattle Egret	2022/4/17	2	0	22.2445060	113.9791870	https://www.inaturalist.org/observations/111776058
2241	Bubulcus ibis	Cattle Egret	2021/9/25	2	0	22.2445640	113.9799394	https://www.inaturalist.org/observations/101526719
2242	Bubulcus ibis	Cattle Egret	2019/11/7	1	0	22.2446897	114.1761282	https://www.inaturalist.org/observations/35403161
2243	Bubulcus ibis	Cattle Egret	2019/11/7	1	0	22.2453598	114.1760136	https://www.inaturalist.org/observations/35442412
2244	Bubulcus ibis	Cattle Egret	2021/4/1	2	0	22.2454610	113.9778410	https://www.inaturalist.org/observations/72600220
2245	Bubulcus ibis	Cattle Egret	2021/5/5	1	0	22.2460767	114.1449555	https://www.inaturalist.org/observations/77897113
2246	Bubulcus ibis	Cattle Egret	2021/9/30	1	0	22.2461668	113.9810414	https://www.inaturalist.org/observations/96705191
2247	Bubulcus ibis	Cattle Egret	2018/4/17	3	0	22.2465782	114.1882553	https://www.inaturalist.org/observations/11102847
2248	Bubulcus ibis	Cattle Egret	2021/12/31	1	0	22.2472558	114.1871953	https://www.inaturalist.org/observations/104031864
2249	Bubulcus ibis	Cattle Egret	2021/4/28	4	0	22.2474748	114.1745500	https://www.inaturalist.org/observations/75539908

2250	Bubulcus ibis	Cattle Egret	2020/10/29	1	0	22.2486502	114.1750102	https://www.inaturalist.org/observations/63752405
2251	Bubulcus ibis	Cattle Egret	2021/6/26	2	0	22.2492755	114.1399515	https://www.inaturalist.org/observations/84514554
2252	Bubulcus ibis coromandus	Cattle Egret	2018/4/29	2	0	22.2505294	113.8653958	https://www.inaturalist.org/observations/11835973
2253	Bubulcus ibis	Cattle Egret	2020/11/15	1	0	22.2532069	114.1587005	https://www.inaturalist.org/observations/64917278
2254	Bubulcus ibis	Cattle Egret	2022/11/7	2	0	22.2532450	114.1583862	https://www.inaturalist.org/observations/141420313
2255	Bubulcus ibis	Cattle Egret	2020/5/23	1	0	22.2541247	114.1616040	https://www.inaturalist.org/observations/46934266
2256	Bubulcus ibis	Cattle Egret	2021/10/23	2	0	22.2545358	114.1596100	https://www.inaturalist.org/observations/99057382
2257	Bubulcus ibis	Cattle Egret	2022/12/18	1	0	22.2554529	114.3989675	https://www.inaturalist.org/observations/144590922
2258	Bubulcus ibis	Cattle Egret	2022/11/5	1	0	22.2558267	114.1563847	https://www.inaturalist.org/observations/141164474
2259	Bubulcus ibis	Cattle Egret	2021/11/11	2	0	22.2566258	113.9878304	https://www.inaturalist.org/observations/100841173
2260	Bubulcus ibis	Cattle Egret	2021/4/30	2	0	22.2566667	114.1313888	https://www.inaturalist.org/observations/75737371
2261	Bubulcus ibis	Cattle Egret	2020/5/3	1	0	22.2591691	114.2100571	https://www.inaturalist.org/observations/44736852
2262	Bubulcus ibis	Cattle Egret	2020/12/9	2	0	22.2594707	114.1301740	https://www.inaturalist.org/observations/66325619
2263	Bubulcus ibis	Cattle Egret	2022/10/16	1	0	22.2600871	114.1665406	https://www.inaturalist.org/observations/138968929
2264	Bubulcus ibis	Cattle Egret	2022/4/29	1	0	22.2623939	113.9932688	https://www.inaturalist.org/observations/114141721
2265	Bubulcus ibis	Cattle Egret	2021/5/29	2	0	22.2627768	113.9571599	https://www.inaturalist.org/observations/81258312
2266	Bubulcus ibis	Cattle Egret	2020/4/25	1	0	22.2633700	113.9923333	https://www.inaturalist.org/observations/43513099
2267	Bubulcus ibis	Cattle Egret	2021/1/23	2	0	22.2635486	113.9922533	https://www.inaturalist.org/observations/68479447
2268	Bubulcus ibis	Cattle Egret	2020/3/25	1	0	22.2643648	113.9904766	https://www.inaturalist.org/observations/40747343
2269	Bubulcus ibis	Cattle Egret	2018/10/1	3	0	22.2643698	114.1365256	https://www.inaturalist.org/observations/17094557
2270	Bubulcus ibis coromandus	Cattle Egret	2018/5/11	2	0	22.2648909	114.1575314	https://www.inaturalist.org/observations/12340906
2271	Bubulcus ibis	Cattle Egret	2020/10/3	1	0	22.2660320	113.9947441	https://www.inaturalist.org/observations/61827898
2272	Bubulcus ibis	Cattle Egret	2021/3/13	1	0	22.2660320	113.9947441	https://www.inaturalist.org/observations/72175830
2273	Bubulcus ibis	Cattle Egret	2022/9/29	1	0	22.2660833	114.2500750	https://www.inaturalist.org/observations/136914957
2274	Bubulcus ibis coromandus	Cattle Egret	2022/4/4	1	0	22.2662178	113.9920624	https://www.inaturalist.org/observations/110391643

2275	Bubulcus ibis	Cattle Egret	2021/5/3	2	0	22.2664984	113.9417510	https://www.inaturalist.org/observations/76903885
2276	Bubulcus ibis	Cattle Egret	2022/4/28	1	0	22.2667780	113.9417507	https://www.inaturalist.org/observations/113241551
2277	Bubulcus ibis	Cattle Egret	2022/9/24	2	0	22.2667780	113.9417510	https://www.inaturalist.org/observations/136561498
2278	Bubulcus ibis	Cattle Egret	2016/5/27	2	0	22.2686701	114.1232643	https://www.inaturalist.org/observations/10339992
2279	Bubulcus ibis	Cattle Egret	2020/11/7	1	0	22.2692512	113.9923600	https://www.inaturalist.org/observations/64584799
2280	Bubulcus ibis	Cattle Egret	2020/4/26	1	0	22.2695451	113.9901829	https://www.inaturalist.org/observations/43566564
2281	Bubulcus ibis	Cattle Egret	2012/9/26	2	0	22.2699674	113.9924498	https://www.inaturalist.org/observations/24180178
2282	Bubulcus ibis	Cattle Egret	2022/5/2	1	0	22.2705632	113.9924699	https://www.inaturalist.org/observations/114551916
2283	Bubulcus ibis	Cattle Egret	2020/9/27	1	0	22.2706450	113.9888580	https://www.inaturalist.org/observations/62009122
2284	Bubulcus ibis	Cattle Egret	2021/5/2	2	0	22.2718450	113.9964967	https://www.inaturalist.org/observations/113725365
2285	Bubulcus ibis	Cattle Egret	2020/10/8	1	0	22.2721187	113.9931363	https://www.inaturalist.org/observations/62101772
2286	Bubulcus ibis	Cattle Egret	2020/9/27	1	0	22.2724770	113.9952670	https://www.inaturalist.org/observations/62008994
2287	Bubulcus ibis	Cattle Egret	2022/4/30	1	0	22.2726467	113.9962917	https://www.inaturalist.org/observations/113767718
2288	Bubulcus ibis coromandus	Cattle Egret	2019/4/2	3	0	22.2739913	113.9953780	https://www.inaturalist.org/observations/22070491
2289	Bubulcus ibis	Cattle Egret	2020/5/11	2	0	22.2764950	114.1891950	https://www.inaturalist.org/observations/45567076
2290	Bubulcus ibis	Cattle Egret	2021/5/2	2	0	22.2766737	114.2968257	https://www.inaturalist.org/observations/76481490
2291	Bubulcus ibis	Cattle Egret	2013/5/4	3	0	22.2783151	114.1746950	http://www.inaturalist.org/observations/3917208
2292	Bubulcus ibis	Cattle Egret	2022/5/7	1	0	22.2786867	114.1272138	https://www.inaturalist.org/observations/115894547
2293	Bubulcus ibis	Cattle Egret	2021/9/25	1	0	22.2801005	113.9327465	https://www.inaturalist.org/observations/96175981
2294	Bubulcus ibis	Cattle Egret	2021/5/16	1	0	22.2814917	114.1877217	https://www.inaturalist.org/observations/78975597
2295	Bubulcus ibis coromandus	Cattle Egret	2021/5/18	3	0	22.2824950	114.1881867	https://www.inaturalist.org/observations/91069215
2296	Bubulcus ibis coromandus	Cattle Egret	2018/1/3	2	0	22.2837050	114.0005583	https://www.inaturalist.org/observations/9370882
2297	Bubulcus ibis	Cattle Egret	2021/5/15	1	0	22.2872064	114.1617807	https://www.inaturalist.org/observations/78883712
2298	Bubulcus ibis	Cattle Egret	2019/10/8	3	0	22.2875276	114.0044913	https://www.inaturalist.org/observations/34106327
2299	Bubulcus ibis	Cattle Egret	2022/4/30	1	0	22.2887174	114.2830025	https://www.inaturalist.org/observations/114437018

2300	Bubulcus ibis	Cattle Egret	2020/5/4	1	0	22.2944817	114.2702546	https://www.inaturalist.org/observations/44876957
2301	Bubulcus ibis	Cattle Egret	2021/5/2	1	0	22.2948236	114.0132228	https://www.inaturalist.org/observations/76452749
2302	Bubulcus ibis	Cattle Egret	2021/5/1	1	0	22.2950727	114.0118565	https://www.inaturalist.org/observations/76451315
2303	Bubulcus ibis	Cattle Egret	2020/4/25	1	0	22.2965706	114.2762291	https://www.inaturalist.org/observations/44097440
2304	Bubulcus ibis	Cattle Egret	2020/4/27	1	0	22.2975480	114.2760973	https://www.inaturalist.org/observations/44133913
2305	Bubulcus ibis	Cattle Egret	2021/9/1	1	0	22.3004583	82.0822050	https://www.inaturalist.org/observations/93442900
2306	Bubulcus ibis	Cattle Egret	2022/11/22	1	0	22.3039526	114.2678195	https://www.inaturalist.org/observations/142607639
2307	Bubulcus ibis	Cattle Egret	2021/6/25	2	0	22.3041572	114.2666935	https://www.inaturalist.org/observations/87076909
2308	Bubulcus ibis	Cattle Egret	2021/10/11	2	0	22.3044076	114.2668263	https://www.inaturalist.org/observations/97915540
2309	Bubulcus ibis	Cattle Egret	2021/10/10	1	0	22.3045430	114.2670055	https://www.inaturalist.org/observations/97749622
2310	Bubulcus ibis	Cattle Egret	2022/10/12	1	0	22.3047034	114.2692966	https://www.inaturalist.org/observations/138508628
2311	Bubulcus ibis	Cattle Egret	2021/10/10	1	0	22.3048798	114.2162088	https://www.inaturalist.org/observations/101082004
2312	Bubulcus ibis	Cattle Egret	2022/11/3	2	0	22.3050162	114.2666605	https://www.inaturalist.org/observations/140986548
2313	Bubulcus ibis	Cattle Egret	2019/4/12	3	0	22.3061513	114.2177895	https://www.inaturalist.org/observations/23972491
2314	Bubulcus ibis	Cattle Egret	2022/5/29	1	0	22.3066077	114.0391545	https://www.inaturalist.org/observations/119218132
2315	Bubulcus ibis	Cattle Egret	2014/10/24	2	0	22.3071588	73.1812187	https://www.inaturalist.org/observations/56857619
2316	Bubulcus ibis	Cattle Egret	2015/8/27	5	0	22.3071590	73.1812190	http://www.inaturalist.org/observations/1926979
2317	Bubulcus ibis	Cattle Egret	2022/5/7	2	0	22.3073130	114.2653916	https://www.inaturalist.org/observations/136544807
2318	Bubulcus ibis	Cattle Egret	2021/5/12	2	0	22.3073830	114.2196025	https://www.inaturalist.org/observations/78489184
2319	Bubulcus ibis	Cattle Egret	2022/10/6	1	0	22.3085163	114.2652298	https://www.inaturalist.org/observations/137773162
2320	Bubulcus ibis	Cattle Egret	2021/4/20	2	0	22.3119357	114.2568776	https://www.inaturalist.org/observations/75159701
2321	Bubulcus ibis	Cattle Egret	2020/6/18	3	0	22.3126839	73.2853817	https://www.inaturalist.org/observations/61619809
2322	Bubulcus ibis	Cattle Egret	2021/5/2	4	0	22.3127159	73.2853817	https://www.inaturalist.org/observations/105207519
2323	Bubulcus ibis	Cattle Egret	2021/5/1	1	0	22.3137476	114.2750841	https://www.inaturalist.org/observations/87220223
2324	Bubulcus ibis	Cattle Egret	2022/3/20	3	0	22.3138700	73.2763667	https://www.inaturalist.org/observations/119749592

2325	Bubulcus ibis coromandus	Cattle Egret	2022/4/26	2	0	22.3186796	114.0428344	https://www.inaturalist.org/observations/113202282
2326	Bubulcus ibis	Cattle Egret	2020/10/6	5	0	22.3219737	114.0378249	https://www.inaturalist.org/observations/61856930
2327	Bubulcus ibis	Cattle Egret	2020/10/11	2	0	22.3228664	73.5606995	https://www.inaturalist.org/observations/62279113
2328	Bubulcus ibis	Cattle Egret	2020/9/27	5	0	22.3261689	72.6354001	https://www.inaturalist.org/observations/61073030
2329	Bubulcus ibis	Cattle Egret	2019/10/1	2	0	22.3323748	114.1828934	https://www.inaturalist.org/observations/33837183
2330	Bubulcus ibis	Cattle Egret	2019/5/7	4	0	22.3326396	114.1831987	https://www.inaturalist.org/observations/24722354
2331	Bubulcus ibis	Cattle Egret	2022/4/30	1	0	22.3327928	114.1830179	https://www.inaturalist.org/observations/113728382
2332	Bubulcus ibis	Cattle Egret	2020/5/14	3	0	22.3331557	114.1833241	https://www.inaturalist.org/observations/45857737
2333	Bubulcus ibis	Cattle Egret	2021/3/19	1	0	22.3340777	80.6112046	https://www.inaturalist.org/observations/73364930
2334	Bubulcus ibis	Cattle Egret	2012/5/17	3	0	22.3345132	80.6115130	https://www.inaturalist.org/observations/55791236
2335	Bubulcus ibis	Cattle Egret	2019/2/12	1	0	22.3345132	80.6115130	https://www.inaturalist.org/observations/90522215
2336	Bubulcus ibis	Cattle Egret	2020/10/9	3	0	22.3346469	114.1375696	https://www.inaturalist.org/observations/82384475
2337	Bubulcus ibis	Cattle Egret	2020/10/17	2	0	22.3416178	114.1598017	https://www.inaturalist.org/observations/62774830
2338	Bubulcus ibis	Cattle Egret	2019/2/13	0	0	22.3444650	80.4552699	https://www.inaturalist.org/observations/90508030
2339	Bubulcus ibis	Cattle Egret	2018/4/29	1	0	22.3508385	113.5833626	https://www.inaturalist.org/observations/142749179
2340	Bubulcus ibis	Cattle Egret	2021/5/8	1	0	22.3509056	114.2383972	https://www.inaturalist.org/observations/77989570
2341	Bubulcus ibis	Cattle Egret	2022/2/20	2	0	22.3518233	73.5907133	https://www.inaturalist.org/observations/120020524
2342	Bubulcus ibis coromandus	Cattle Egret	2020/4/14	3	0	22.3554492	73.1357684	https://www.inaturalist.org/observations/44058941
2343	Bubulcus ibis	Cattle Egret	2020/4/17	2	0	22.3555940	114.1054437	https://www.inaturalist.org/observations/42387363
2344	Bubulcus ibis	Cattle Egret	2020/4/27	1	0	22.3558983	114.1056299	https://www.inaturalist.org/observations/43866372
2345	Bubulcus ibis	Cattle Egret	2020/5/2	1	0	22.3562691	114.1056228	https://www.inaturalist.org/observations/44661433
2346	Bubulcus ibis	Cattle Egret	2020/5/6	1	0	22.3565247	114.1059689	https://www.inaturalist.org/observations/45047753
2347	Bubulcus ibis	Cattle Egret	2021/1/31	1	0	22.3566645	88.3899022	https://www.inaturalist.org/observations/104113869
2348	Bubulcus ibis	Cattle Egret	2020/4/13	1	0	22.3570594	114.1037166	https://www.inaturalist.org/observations/43907926
2349	Bubulcus ibis	Cattle Egret	2022/3/19	5	0	22.3594235	82.7500594	https://www.inaturalist.org/observations/121760218

2350	Bubulcus ibis	Cattle Egret	2021/4/17	1	0	22.3602516	82.7551168	https://www.inaturalist.org/observations/75729763
2351	Bubulcus ibis	Cattle Egret	2022/3/29	1	0	22.3631217	73.5859317	https://www.inaturalist.org/observations/119566004
2352	Bubulcus ibis	Cattle Egret	2022/3/29	2	0	22.3637167	73.5856617	https://www.inaturalist.org/observations/119566014
2353	Bubulcus ibis	Cattle Egret	2021/5/22	3	0	22.3671452	82.1403410	https://www.inaturalist.org/observations/79757849
2354	Bubulcus ibis	Cattle Egret	2022/4/17	2	0	22.3678947	114.2702018	https://www.inaturalist.org/observations/112038189
2355	Bubulcus ibis	Cattle Egret	2018/12/26	3	0	22.3685873	114.0599175	https://www.inaturalist.org/observations/19308909
2356	Bubulcus ibis	Cattle Egret	2021/4/25	2	0	22.3723169	114.0229796	https://www.inaturalist.org/observations/75152882
2357	Bubulcus ibis	Cattle Egret	2021/11/28	1	0	22.3724292	113.9343161	https://www.inaturalist.org/observations/102083337
2358	Bubulcus ibis	Cattle Egret	2021/12/1	1	0	22.3724292	113.9343161	https://www.inaturalist.org/observations/102347593
2359	Bubulcus ibis	Cattle Egret	2021/12/1	1	0	22.3724292	113.9343161	https://www.inaturalist.org/observations/102347788
2360	Bubulcus ibis	Cattle Egret	2022/4/8	1	0	22.3726735	113.9314323	https://www.inaturalist.org/observations/111098471
2361	Bubulcus ibis	Cattle Egret	2020/4/26	1	0	22.3731484	114.1245003	https://www.inaturalist.org/observations/43550973
2362	Bubulcus ibis	Cattle Egret	2018/11/2	3	0	22.3734420	114.3366443	https://www.inaturalist.org/observations/23087920
2363	Bubulcus ibis	Cattle Egret	2021/11/11	2	0	22.3740888	114.2650540	https://www.inaturalist.org/observations/100833720
2364	Bubulcus ibis	Cattle Egret	2021/11/13	2	0	22.3740889	114.2650538	https://www.inaturalist.org/observations/101190108
2365	Bubulcus ibis	Cattle Egret	2021/11/13	3	0	22.3740889	114.2650538	https://www.inaturalist.org/observations/101190154
2366	Bubulcus ibis	Cattle Egret	2022/11/11	1	0	22.3740930	114.2650538	https://www.inaturalist.org/observations/141742495
2367	Bubulcus ibis	Cattle Egret	2017/10/14	2	0	22.3740970	114.2668680	https://www.inaturalist.org/observations/8406819
2368	Bubulcus ibis	Cattle Egret	2021/11/6	1	0	22.3741069	114.2650437	https://www.inaturalist.org/observations/100584348
2369	Bubulcus ibis	Cattle Egret	2018/10/20	5	0	22.3743858	114.2651443	https://www.inaturalist.org/observations/17674344
2370	Bubulcus ibis	Cattle Egret	2021/10/30	1	0	22.3744641	114.2638850	https://www.inaturalist.org/observations/99780885
2371	Bubulcus ibis	Cattle Egret	2021/5/1	1	0	22.3744862	114.2652298	https://www.inaturalist.org/observations/76008370
2372	Bubulcus ibis	Cattle Egret	2022/12/14	2	0	22.3746005	114.2649633	https://www.inaturalist.org/observations/144741106
2373	Bubulcus ibis	Cattle Egret	2022/12/18	2	0	22.3746480	114.2649208	https://www.inaturalist.org/observations/144590612
2374	Bubulcus ibis coromandus	Cattle Egret	2022/12/19	2	0	22.3747279	114.2643173	https://www.inaturalist.org/observations/144768850

2375	Bubulcus ibis	Cattle Egret	2021/9/7	1	0	22.3751289	114.1839496	https://www.inaturalist.org/observations/93982725
2376	Bubulcus ibis	Cattle Egret	2020/5/3	2	0	22.3755552	114.1264790	https://www.inaturalist.org/observations/44676796
2377	Bubulcus ibis	Cattle Egret	2022/11/3	1	0	22.3758269	73.1440339	https://www.inaturalist.org/observations/140974377
2378	Bubulcus ibis	Cattle Egret	2021/11/11	2	0	22.3772205	114.2645229	https://www.inaturalist.org/observations/100848304
2379	Bubulcus ibis	Cattle Egret	2020/4/12	2	0	22.3775892	114.1269604	https://www.inaturalist.org/observations/41973848
2380	Bubulcus ibis	Cattle Egret	2022/4/15	1	0	22.3801768	114.2772490	https://www.inaturalist.org/observations/114772481
2381	Bubulcus ibis	Cattle Egret	2017/11/1	4	0	22.3802367	114.2678046	https://www.inaturalist.org/observations/20075806
2382	Bubulcus ibis	Cattle Egret	2021/5/1	1	0	22.3811185	114.2651807	https://www.inaturalist.org/observations/76822763
2383	Bubulcus ibis	Cattle Egret	2017/5/8	1	0	22.3846410	114.1445800	https://www.inaturalist.org/observations/39829194
2384	Bubulcus ibis	Cattle Egret	2021/11/6	1	0	22.3849500	114.2730867	https://www.inaturalist.org/observations/101074292
2385	Bubulcus ibis	Cattle Egret	2020/10/31	2	0	22.3851416	114.1982366	https://www.inaturalist.org/observations/63868864
2386	Bubulcus ibis coromandus	Cattle Egret	2020/11/15	2	0	22.3856604	114.2751246	https://www.inaturalist.org/observations/64910993
2387	Bubulcus ibis	Cattle Egret	2021/4/30	1	0	22.3862601	114.1012398	https://www.inaturalist.org/observations/76854773
2388	Bubulcus ibis	Cattle Egret	2021/5/14	2	0	22.3869739	114.1453990	https://www.inaturalist.org/observations/142516864
2389	Bubulcus ibis	Cattle Egret	2019/5/5	3	0	22.3878049	114.1456546	https://www.inaturalist.org/observations/25804385
2390	Bubulcus ibis	Cattle Egret	2021/2/14	3	0	22.3882383	120.6062117	https://www.inaturalist.org/observations/69553598
2391	Bubulcus ibis	Cattle Egret	2021/2/12	1	0	22.3884700	120.6067467	https://www.inaturalist.org/observations/69450664
2392	Bubulcus ibis	Cattle Egret	2019/5/14	4	0	22.3886201	114.1457999	https://www.inaturalist.org/observations/26316657
2393	Bubulcus ibis	Cattle Egret	2022/4/3	2	0	22.3889416	114.3158947	https://www.inaturalist.org/observations/113734015
2394	Bubulcus ibis	Cattle Egret	2021/4/21	1	0	22.3923206	114.1492880	https://www.inaturalist.org/observations/74756264
2395	Bubulcus ibis	Cattle Egret	2022/12/23	2	0	22.3926331	114.3485651	https://www.inaturalist.org/observations/144909735
2396	Bubulcus ibis coromandus	Cattle Egret	2021/5/2	2	0	22.3939467	114.1520833	https://www.inaturalist.org/observations/76449591
2397	Bubulcus ibis	Cattle Egret	2020/4/28	1	0	22.3939957	114.1515447	https://www.inaturalist.org/observations/49731246
2398	Bubulcus ibis	Cattle Egret	2021/5/1	1	0	22.3944747	114.1520859	https://www.inaturalist.org/observations/76035485
2399	Bubulcus ibis	Cattle Egret	2021/5/1	2	0	22.3944949	114.1520834	https://www.inaturalist.org/observations/76037917

2400	Bubulcus ibis	Cattle Egret	2021/5/1	2	0	22.3945538	114.1523597	https://www.inaturalist.org/observations/76035797
2401	Bubulcus ibis	Cattle Egret	2019/1/29	3	0	22.3946033	120.6016617	https://www.inaturalist.org/observations/19942404
2402	Bubulcus ibis	Cattle Egret	2021/4/21	1	0	22.3948456	114.1509368	https://www.inaturalist.org/observations/74756267
2403	Bubulcus ibis	Cattle Egret	2022/10/23	2	0	22.3950340	114.1116758	https://www.inaturalist.org/observations/139946578
2404	Bubulcus ibis	Cattle Egret	2021/5/1	1	0	22.3954333	114.1524289	https://www.inaturalist.org/observations/76063076
2405	Bubulcus ibis	Cattle Egret	2021/11/2	0	0	22.3957383	114.2843700	https://www.inaturalist.org/observations/100270573
2406	Bubulcus ibis	Cattle Egret	2019/5/15	2	0	22.3958470	114.1509348	https://www.inaturalist.org/observations/34050936
2407	Bubulcus ibis	Cattle Egret	2004/4/4	2	0	22.3979422	114.2054321	https://www.inaturalist.org/observations/8555575
2408	Bubulcus ibis	Cattle Egret	2020/4/30	1	0	22.3980878	114.3191005	https://www.inaturalist.org/observations/44347892
2409	Bubulcus ibis	Cattle Egret	2020/4/17	1	0	22.4033431	114.3257760	https://www.inaturalist.org/observations/42452156
2410	Bubulcus ibis	Cattle Egret	2022/11/9	2	0	22.4034537	114.2327395	https://www.inaturalist.org/observations/141533324
2411	Bubulcus ibis	Cattle Egret	2021/11/7	1	0	22.4055298	114.2774164	https://www.inaturalist.org/observations/100472296
2412	Bubulcus ibis	Cattle Egret	2019/4/4	1	0	22.4057283	120.6192083	https://www.inaturalist.org/observations/21977350
2413	Bubulcus ibis	Cattle Egret	2019/9/23	4	0	22.4075518	72.5307071	https://www.inaturalist.org/observations/33241108
2414	Bubulcus ibis	Cattle Egret	2021/10/20	2	0	22.4081906	113.9842909	https://www.inaturalist.org/observations/111183817
2415	Bubulcus ibis	Cattle Egret	2022/11/6	2	0	22.4084695	114.3435211	https://www.inaturalist.org/observations/141273798
2416	Bubulcus ibis	Cattle Egret	2022/10/30	2	0	22.4092222	114.0313944	https://www.inaturalist.org/observations/140466163
2417	Bubulcus ibis	Cattle Egret	2021/5/1	1	0	22.4108700	114.2730626	https://www.inaturalist.org/observations/76799430
2418	Bubulcus ibis	Cattle Egret	2021/3/31	1	0	22.4173764	114.0849424	https://www.inaturalist.org/observations/72575890
2419	Bubulcus ibis	Cattle Egret	2018/11/17	5	0	22.4174960	114.2691930	https://www.inaturalist.org/observations/18622961
2420	Bubulcus ibis	Cattle Egret	2020/4/26	3	0	22.4182804	114.2121010	https://www.inaturalist.org/observations/43563573
2421	Bubulcus ibis coromandus	Cattle Egret	2022/2/3	0	0	22.4184005	114.2768225	https://www.inaturalist.org/observations/106063159
2422	Bubulcus ibis	Cattle Egret	2022/1/4	1	0	22.4215201	114.0635009	https://www.inaturalist.org/observations/104380396
2423	Bubulcus ibis	Cattle Egret	2018/2/23	2	0	22.4230000	88.2864000	https://www.inaturalist.org/observations/69879104
2424	Bubulcus ibis	Cattle Egret	2022/2/2	2	0	22.4234295	114.0473415	https://www.inaturalist.org/observations/106021081

2425	Bubulcus ibis	Cattle Egret	2018/4/30	4	0	22.4237741	113.9408656	https://www.inaturalist.org/observations/11864701
2426	Bubulcus ibis	Cattle Egret	2019/12/15	1	0	22.4238753	113.9406253	https://www.inaturalist.org/observations/36666207
2427	Bubulcus ibis	Cattle Egret	2018/4/30	4	0	22.4239527	113.9410158	https://www.inaturalist.org/observations/11864679
2428	Bubulcus ibis	Cattle Egret	2020/5/1	1	0	22.4271975	114.2917887	https://www.inaturalist.org/observations/44446402
2429	Bubulcus ibis	Cattle Egret	2020/9/27	1	0	22.4275760	114.0874146	https://www.inaturalist.org/observations/60981042
2430	Bubulcus ibis	Cattle Egret	2020/9/27	1	0	22.4275760	114.0874146	https://www.inaturalist.org/observations/60981066
2431	Bubulcus ibis	Cattle Egret	2020/11/1	1	0	22.4275760	114.0874146	https://www.inaturalist.org/observations/64017943
2432	Bubulcus ibis	Cattle Egret	2021/5/30	3	0	22.4275969	114.0887397	https://www.inaturalist.org/observations/81057685
2433	Bubulcus ibis	Cattle Egret	2022/9/24	1	0	22.4309572	120.4929402	https://www.inaturalist.org/observations/136389299
2434	Bubulcus ibis	Cattle Egret	2022/5/7	2	0	22.4310578	114.0851528	https://www.inaturalist.org/observations/115875974
2435	Bubulcus ibis	Cattle Egret	2021/11/18	2	0	22.4317281	114.3271367	https://www.inaturalist.org/observations/101361399
2436	Bubulcus ibis	Cattle Egret	2018/4/27	3	0	22.4318028	114.2557220	https://www.inaturalist.org/observations/11477386
2437	Bubulcus ibis	Cattle Egret	2022/2/6	1	0	22.4318666	114.0057069	https://www.inaturalist.org/observations/106355465
2438	Bubulcus ibis	Cattle Egret	2020/4/30	1	0	22.4361937	114.3309272	https://www.inaturalist.org/observations/44359163
2439	Bubulcus ibis	Cattle Egret	2021/11/5	1	0	22.4398887	114.0256179	https://www.inaturalist.org/observations/128124595
2440	Bubulcus ibis	Cattle Egret	2021/5/7	2	0	22.4408680	114.2824839	https://www.inaturalist.org/observations/77706933
2441	Bubulcus ibis	Cattle Egret	2021/4/29	1	0	22.4414985	114.2861028	https://www.inaturalist.org/observations/75633658
2442	Bubulcus ibis	Cattle Egret	2020/5/9	1	0	22.4422689	114.2856858	https://www.inaturalist.org/observations/45453197
2443	Bubulcus ibis	Cattle Egret	2020/4/11	2	0	22.4423218	114.1655064	https://www.inaturalist.org/observations/96035079
2444	Bubulcus ibis	Cattle Egret	2017/3/14	6	0	22.4425478	78.1831039	https://www.inaturalist.org/observations/5574265
2445	Bubulcus ibis	Cattle Egret	2015/8/9	1	0	22.4439509	120.4916027	https://www.inaturalist.org/observations/63755297
2446	Bubulcus ibis	Cattle Egret	2021/12/29	1	0	22.4445376	114.0222076	https://www.inaturalist.org/observations/107938743
2447	Bubulcus ibis	Cattle Egret	2022/5/8	2	0	22.4445376	114.0222076	https://www.inaturalist.org/observations/141152185
2448	Bubulcus ibis	Cattle Egret	2020/5/8	1	0	22.4449472	114.1778290	https://www.inaturalist.org/observations/46487400
2449	Bubulcus ibis	Cattle Egret	2020/4/25	2	0	22.4450990	114.1385870	https://www.inaturalist.org/observations/44733978

2450	Bubulcus ibis	Cattle Egret	2020/4/16	1	0	22.4451402	114.1385703	https://www.inaturalist.org/observations/45146389
2451	Bubulcus ibis coromandus	Cattle Egret	2020/4/26	1	0	22.4452337	114.1374250	https://www.inaturalist.org/observations/43613244
2452	Bubulcus ibis	Cattle Egret	2018/12/15	4	0	22.4452440	114.1379470	https://www.inaturalist.org/observations/19418766
2453	Bubulcus ibis coromandus	Cattle Egret	2020/12/19	2	0	22.4452840	114.1373856	https://www.inaturalist.org/observations/66901717
2454	Bubulcus ibis	Cattle Egret	2022/4/17	1	0	22.4453277	114.1378574	https://www.inaturalist.org/observations/114618024
2455	Bubulcus ibis	Cattle Egret	2019/3/31	2	0	22.4454506	114.1375517	https://www.inaturalist.org/observations/22186227
2456	Bubulcus ibis coromandus	Cattle Egret	2021/1/16	2	0	22.4454740	114.1373964	https://www.inaturalist.org/observations/68205205
2457	Bubulcus ibis	Cattle Egret	2019/1/10	3	0	22.4458243	114.1377284	https://www.inaturalist.org/observations/19549207
2458	Bubulcus ibis	Cattle Egret	2018/12/15	4	0	22.4458719	114.1377393	https://www.inaturalist.org/observations/19129871
2459	Bubulcus ibis	Cattle Egret	2021/4/17	1	0	22.4465010	120.5056193	https://www.inaturalist.org/observations/74509584
2460	Bubulcus ibis	Cattle Egret	2021/4/19	2	0	22.4472939	120.5070208	https://www.inaturalist.org/observations/74516869
2461	Bubulcus ibis	Cattle Egret	2021/4/19	1	0	22.4474117	120.5067050	https://www.inaturalist.org/observations/74503151
2462	Bubulcus ibis	Cattle Egret	2020/6/8	1	0	22.4490861	120.5103667	https://www.inaturalist.org/observations/48911170
2463	Bubulcus ibis	Cattle Egret	2020/5/15	1	0	22.4497719	120.5101648	https://www.inaturalist.org/observations/46059589
2464	Bubulcus ibis	Cattle Egret	2021/5/1	1	0	22.4554798	114.1454727	https://www.inaturalist.org/observations/76121618
2465	Bubulcus ibis	Cattle Egret	2022/12/1	1	0	22.4586717	114.3258883	https://www.inaturalist.org/observations/143536460
2466	Bubulcus ibis	Cattle Egret	2022/5/8	1	0	22.4590854	114.1956386	https://www.inaturalist.org/observations/116114336
2467	Bubulcus ibis	Cattle Egret	2015/12/5	2	0	22.4606419	114.0041996	https://www.inaturalist.org/observations/24473928
2468	Bubulcus ibis	Cattle Egret	2015/12/5	2	0	22.4606419	114.0041996	https://www.inaturalist.org/observations/24475422
2469	Bubulcus ibis	Cattle Egret	2015/12/5	2	0	22.4606419	114.0041996	https://www.inaturalist.org/observations/24502661
2470	Bubulcus ibis	Cattle Egret	2016/12/3	3	0	22.4606419	114.0041996	https://www.inaturalist.org/observations/28596036
2471	Bubulcus ibis	Cattle Egret	2021/11/15	0	0	22.4606467	114.3289000	https://www.inaturalist.org/observations/101176779
2472	Bubulcus ibis	Cattle Egret	2021/11/15	0	0	22.4606533	114.3286750	https://www.inaturalist.org/observations/101176682
2473	Bubulcus ibis	Cattle Egret	2021/11/15	0	0	22.4606997	114.3284814	https://www.inaturalist.org/observations/101160025
2474	Bubulcus ibis	Cattle Egret	2020/4/6	1	0	22.4607117	114.3288033	https://www.inaturalist.org/observations/43250622

2475	Bubulcus ibis	Cattle Egret	2021/7/1	1	0	22.4622430	114.0383940	https://www.inaturalist.org/observations/85407331
2476	Bubulcus ibis	Cattle Egret	2020/12/20	4	0	22.4653450	114.0370081	https://www.inaturalist.org/observations/122321859
2477	Bubulcus ibis	Cattle Egret	2022/11/16	1	0	22.4659717	114.0369600	https://www.inaturalist.org/observations/142143610
2478	Bubulcus ibis	Cattle Egret	2022/11/30	1	0	22.4660323	114.0388417	https://www.inaturalist.org/observations/143350502
2479	Bubulcus ibis	Cattle Egret	2021/7/4	3	0	22.4671648	114.0408427	https://www.inaturalist.org/observations/85601221
2480	Bubulcus ibis	Cattle Egret	2022/5/1	1	0	22.4687693	114.0102363	https://www.inaturalist.org/observations/114140686
2481	Bubulcus ibis	Cattle Egret	2021/6/27	3	0	22.4692630	114.0097302	https://www.inaturalist.org/observations/84736908
2482	Bubulcus ibis coromandus	Cattle Egret	2022/5/14	2	0	22.4693050	114.0055083	https://www.inaturalist.org/observations/118112367
2483	Bubulcus ibis	Cattle Egret	2019/11/17	1	0	22.4695341	114.0100226	https://www.inaturalist.org/observations/35766054
2484	Bubulcus ibis	Cattle Egret	2021/8/29	1	0	22.4695539	114.0095730	https://www.inaturalist.org/observations/92999006
2485	Bubulcus ibis	Cattle Egret	2022/5/7	1	0	22.4697464	114.0087050	https://www.inaturalist.org/observations/116057668
2486	Bubulcus ibis	Cattle Egret	2019/3/30	3	0	22.4697770	114.0298200	https://www.inaturalist.org/observations/21968752
2487	Bubulcus ibis	Cattle Egret	2021/7/10	1	0	22.4698812	88.3228301	https://www.inaturalist.org/observations/86401885
2488	Bubulcus ibis	Cattle Egret	2020/12/20	1	0	22.4700011	88.3321069	https://www.inaturalist.org/observations/66870952
2489	Bubulcus ibis	Cattle Egret	2022/5/7	2	0	22.4700585	114.0065831	https://www.inaturalist.org/observations/116381234
2490	Bubulcus ibis	Cattle Egret	2022/5/2	2	0	22.4704533	114.0062722	https://www.inaturalist.org/observations/114603090
2491	Bubulcus ibis	Cattle Egret	2021/5/2	1	0	22.4706804	114.0240430	https://www.inaturalist.org/observations/77446711
2492	Bubulcus ibis	Cattle Egret	2018/4/28	2	0	22.4708396	114.0100580	https://www.inaturalist.org/observations/11587171
2493	Bubulcus ibis	Cattle Egret	2022/3/29	1	0	22.4709693	114.0427050	https://www.inaturalist.org/observations/109820088
2494	Bubulcus ibis	Cattle Egret	2022/4/16	1	0	22.4709693	114.0427050	https://www.inaturalist.org/observations/111913098
2495	Bubulcus ibis coromandus	Cattle Egret	2021/5/1	2	0	22.4709700	114.0427050	https://www.inaturalist.org/observations/77913656
2496	Bubulcus ibis	Cattle Egret	2021/11/22	1	0	22.4709700	114.0427050	https://www.inaturalist.org/observations/101875551
2497	Bubulcus ibis	Cattle Egret	2022/4/7	2	0	22.4715833	114.3628217	https://www.inaturalist.org/observations/139424706
2498	Bubulcus ibis	Cattle Egret	2021/1/26	2	0	22.4721862	88.3262712	https://www.inaturalist.org/observations/68690380
2499	Bubulcus ibis	Cattle Egret	2022/9/28	0	0	22.4723483	88.3255283	https://www.inaturalist.org/observations/138097842

2500	Bubulcus ibis	Cattle Egret	2021/5/1	1	0	22.4724081	114.2352434	https://www.inaturalist.org/observations/76072023
2501	Bubulcus ibis	Cattle Egret	2022/12/4	1	0	22.4730590	114.0221330	https://www.inaturalist.org/observations/143780076
2502	Bubulcus ibis	Cattle Egret	2020/12/26	1	0	22.4733420	114.0224100	https://www.inaturalist.org/observations/67127742
2503	Bubulcus ibis	Cattle Egret	2022/5/2	1	0	22.4733900	114.0241167	https://www.inaturalist.org/observations/115557077
2504	Bubulcus ibis	Cattle Egret	2020/4/30	1	0	22.4735981	114.0245656	https://www.inaturalist.org/observations/44349722
2505	Bubulcus ibis	Cattle Egret	2020/5/8	3	0	22.4736241	88.3129264	https://www.inaturalist.org/observations/131994674
2506	Bubulcus ibis	Cattle Egret	2020/5/8	2	0	22.4736278	88.3129801	https://www.inaturalist.org/observations/131995627
2507	Bubulcus ibis	Cattle Egret	2021/11/15	0	0	22.4736848	114.3356287	https://www.inaturalist.org/observations/101177310
2508	Bubulcus ibis	Cattle Egret	2022/4/30	1	0	22.4741931	114.0371807	https://www.inaturalist.org/observations/115128397
2509	Bubulcus ibis	Cattle Egret	2022/4/3	1	0	22.4742136	114.0376858	https://www.inaturalist.org/observations/110261682
2510	Bubulcus ibis	Cattle Egret	2022/4/5	1	0	22.4742200	114.0377500	https://www.inaturalist.org/observations/110481691
2511	Bubulcus ibis	Cattle Egret	2022/11/25	1	0	22.4743580	114.0365370	https://www.inaturalist.org/observations/143041552
2512	Bubulcus ibis	Cattle Egret	2022/4/5	1	0	22.4744500	114.0372292	https://www.inaturalist.org/observations/110470029
2513	Bubulcus ibis	Cattle Egret	2019/4/9	3	0	22.4750580	114.0359250	https://www.inaturalist.org/observations/24709390
2514	Bubulcus ibis	Cattle Egret	2020/8/4	1	0	22.4810495	114.0348385	https://www.inaturalist.org/observations/55675214
2515	Bubulcus ibis	Cattle Egret	2020/11/13	2	0	22.4835629	114.3097785	https://www.inaturalist.org/observations/65148038
2516	Bubulcus ibis	Cattle Egret	2019/4/1	2	0	22.4852300	114.0487750	https://www.inaturalist.org/observations/21870031
2517	Bubulcus ibis	Cattle Egret	2018/4/30	6	0	22.4867120	114.0401200	https://www.inaturalist.org/observations/12040029
2518	Bubulcus ibis	Cattle Egret	2022/5/2	1	0	22.4867865	114.0402426	https://www.inaturalist.org/observations/115523493
2519	Bubulcus ibis	Cattle Egret	2016/5/14	4	0	22.4868110	114.0397340	https://www.inaturalist.org/observations/30347030
2520	Bubulcus ibis	Cattle Egret	2021/5/1	1	0	22.4868173	114.0400287	https://www.inaturalist.org/observations/77233096
2521	Bubulcus ibis	Cattle Egret	2021/5/4	2	0	22.4868793	114.0373177	https://www.inaturalist.org/observations/81982108
2522	Bubulcus ibis	Cattle Egret	2022/6/3	2	0	22.4869230	114.1023871	https://www.inaturalist.org/observations/121689882
2523	Bubulcus ibis	Cattle Egret	2021/9/29	1	0	22.4878600	114.0368420	https://www.inaturalist.org/observations/96760508
2524	Bubulcus ibis	Cattle Egret	2022/12/5	1	0	22.4882994	114.1401527	https://www.inaturalist.org/observations/143715273

2525	Bubulcus ibis	Cattle Egret	2020/6/12	1	0	22.4893345	114.1234939	https://www.inaturalist.org/observations/67647390
2526	Bubulcus ibis	Cattle Egret	2022/6/3	0	0	22.4905674	114.0435368	https://www.inaturalist.org/observations/119992347
2527	Bubulcus ibis	Cattle Egret	2012/9/16	1	0	22.4905723	72.5082813	https://www.inaturalist.org/observations/40530376
2528	Bubulcus ibis	Cattle Egret	2020/4/26	1	0	22.4909898	114.0381139	https://www.inaturalist.org/observations/43616385
2529	Bubulcus ibis	Cattle Egret	2020/9/29	1	0	22.4920389	114.0446297	https://www.inaturalist.org/observations/61226728
2530	Bubulcus ibis	Cattle Egret	2020/4/26	2	0	22.4924030	114.0441019	https://www.inaturalist.org/observations/43563260
2531	Bubulcus ibis coromandus	Cattle Egret	2022/5/6	1	0	22.4926962	114.0369254	https://www.inaturalist.org/observations/115728275
2532	Bubulcus ibis coromandus	Cattle Egret	2018/9/4	3	0	22.4928483	114.1040517	https://www.inaturalist.org/observations/16317817
2533	Bubulcus ibis	Cattle Egret	2021/4/24	1	0	22.4932617	114.0452278	https://www.inaturalist.org/observations/75018406
2534	Bubulcus ibis coromandus	Cattle Egret	2018/8/4	4	0	22.4933453	114.1057735	https://www.inaturalist.org/observations/15096481
2535	Bubulcus ibis	Cattle Egret	2022/1/23	3	0	22.4934467	73.4826117	https://www.inaturalist.org/observations/120517019
2536	Bubulcus ibis coromandus	Cattle Egret	2011/3/28	2	0	22.4934710	114.0349640	https://www.inaturalist.org/observations/10431707
2537	Bubulcus ibis ibis	Cattle Egret	2022/9/24	0	0	22.4935998	114.1055489	https://www.inaturalist.org/observations/136239156
2538	Bubulcus ibis	Cattle Egret	2022/1/23	3	0	22.4936000	73.4826667	https://www.inaturalist.org/observations/120517030
2539	Bubulcus ibis	Cattle Egret	2020/4/25	1	0	22.4938673	114.0403715	https://www.inaturalist.org/observations/44098438
2540	Bubulcus ibis	Cattle Egret	2020/4/26	2	0	22.4938673	114.0403715	https://www.inaturalist.org/observations/44143594
2541	Bubulcus ibis	Cattle Egret	2022/5/2	1	0	22.4938673	114.0403715	https://www.inaturalist.org/observations/116033907
2542	Bubulcus ibis	Cattle Egret	2021/6/6	3	0	22.4939667	114.0461883	https://www.inaturalist.org/observations/82136217
2543	Bubulcus ibis	Cattle Egret	2009/9/5	1	0	22.4941650	114.0517510	https://www.inaturalist.org/observations/141038934
2544	Bubulcus ibis	Cattle Egret	2017/4/29	2	0	22.4941850	114.0534180	https://www.inaturalist.org/observations/6159695
2545	Bubulcus ibis coromandus	Cattle Egret	2018/8/4	3	0	22.4945367	114.1043731	https://www.inaturalist.org/observations/15096462
2546	Bubulcus ibis	Cattle Egret	2020/10/20	1	0	22.4945824	120.5126099	https://www.inaturalist.org/observations/63170089
2547	Bubulcus ibis	Cattle Egret	2020/7/12	1	0	22.4945986	114.0283136	https://www.inaturalist.org/observations/56017294
2548	Bubulcus ibis	Cattle Egret	2019/4/28	1	0	22.4946767	114.1094317	https://www.inaturalist.org/observations/23689666
2549	Bubulcus ibis	Cattle Egret	2010/5/9	1	0	22.4947010	114.0530550	https://www.inaturalist.org/observations/68752823

2550	Bubulcus ibis	Cattle Egret	2011/3/25	2	0	22.4947300	114.0529460	https://www.inaturalist.org/observations/67850003
2551	Bubulcus ibis	Cattle Egret	2021/11/3	1	0	22.4948440	114.0473540	https://www.inaturalist.org/observations/100486590
2552	Bubulcus ibis	Cattle Egret	2019/12/22	2	0	22.4948580	114.0555900	https://www.inaturalist.org/observations/36882528
2553	Bubulcus ibis	Cattle Egret	2014/4/29	1	0	22.4950121	114.0696131	https://www.inaturalist.org/observations/64232589
2554	Bubulcus ibis	Cattle Egret	2010/4/25	1	0	22.4950470	114.0556630	https://www.inaturalist.org/observations/68946880
2555	Bubulcus ibis	Cattle Egret	2020/4/27	1	0	22.4950569	114.0339342	https://www.inaturalist.org/observations/43847405
2556	Bubulcus ibis	Cattle Egret	2020/4/27	1	0	22.4950569	114.0339342	https://www.inaturalist.org/observations/43847414
2557	Bubulcus ibis	Cattle Egret	2020/6/5	1	0	22.4950569	114.0339342	https://www.inaturalist.org/observations/48785544
2558	Bubulcus ibis	Cattle Egret	2020/7/26	1	0	22.4950569	114.0339342	https://www.inaturalist.org/observations/54379685
2559	Bubulcus ibis	Cattle Egret	2021/11/20	2	0	22.4950613	114.0339342	https://www.inaturalist.org/observations/101533740
2560	Bubulcus ibis	Cattle Egret	2022/5/28	1	0	22.4950613	114.0339342	https://www.inaturalist.org/observations/119157286
2561	Bubulcus ibis	Cattle Egret	2020/11/24	1	0	22.4950940	114.0530410	https://www.inaturalist.org/observations/65795315
2562	Bubulcus ibis	Cattle Egret	2021/9/7	1	0	22.4951430	114.0547904	https://www.inaturalist.org/observations/94319191
2563	Bubulcus ibis	Cattle Egret	2022/5/7	1	0	22.4951821	114.0297599	https://www.inaturalist.org/observations/116410858
2564	Bubulcus ibis	Cattle Egret	2022/9/11	1	0	22.4954420	114.0540910	https://www.inaturalist.org/observations/134837972
2565	Bubulcus ibis	Cattle Egret	2018/4/29	2	0	22.4956875	114.0530853	https://www.inaturalist.org/observations/11761782
2566	Bubulcus ibis	Cattle Egret	2019/10/27	1	0	22.4956925	88.2714903	https://www.inaturalist.org/observations/64162044
2567	Bubulcus ibis	Cattle Egret	2019/6/2	3	0	22.4962987	114.1055925	https://www.inaturalist.org/observations/26234337
2568	Bubulcus ibis	Cattle Egret	2022/4/20	2	0	22.4966988	120.4998604	https://www.inaturalist.org/observations/112147978
2569	Bubulcus ibis	Cattle Egret	2022/4/24	1	0	22.4970365	120.4986418	https://www.inaturalist.org/observations/112817692
2570	Bubulcus ibis coromandus	Cattle Egret	2020/4/26	1	0	22.4987239	114.0449105	https://www.inaturalist.org/observations/43562864
2571	Bubulcus ibis	Cattle Egret	2020/4/25	2	0	22.4989095	114.0274326	https://www.inaturalist.org/observations/43342325
2572	Bubulcus ibis	Cattle Egret	2020/4/25	2	0	22.4989888	114.0274541	https://www.inaturalist.org/observations/43342460
2573	Bubulcus ibis coromandus	Cattle Egret	2019/4/27	2	0	22.4990751	114.0276574	https://www.inaturalist.org/observations/23410664
2574	Bubulcus ibis	Cattle Egret	2019/11/6	1	0	22.4991667	114.0556367	https://www.inaturalist.org/observations/35606140

2575	Bubulcus ibis	Cattle Egret	2020/4/25	2	0	22.4991772	114.0274487	https://www.inaturalist.org/observations/43342602
2576	Bubulcus ibis	Cattle Egret	2020/4/25	1	0	22.4991920	114.0273253	https://www.inaturalist.org/observations/44346315
2577	Bubulcus ibis	Cattle Egret	2021/4/11	1	0	22.4999379	114.1907692	https://www.inaturalist.org/observations/76063245
2578	Bubulcus ibis	Cattle Egret	2021/1/17	1	0	22.5000000	114.1166667	https://www.inaturalist.org/observations/68324557
2579	Bubulcus ibis	Cattle Egret	2015/4/3	1	0	22.5000000	114.1166667	https://www.inaturalist.org/observations/68356658
2580	Bubulcus ibis	Cattle Egret	2020/10/25	1	0	22.5000000	114.1166667	https://www.inaturalist.org/observations/114516223
2581	Bubulcus ibis coromandus	Cattle Egret	2017/2/24	1	0	22.5000000	114.1166667	https://www.inaturalist.org/observations/129286930
2582	Bubulcus ibis	Cattle Egret	2022/1/20	1	0	22.5000000	114.1166670	https://www.inaturalist.org/observations/105354379
2583	Bubulcus ibis	Cattle Egret	2022/1/20	1	0	22.5000000	114.1166670	https://www.inaturalist.org/observations/105354380
2584	Bubulcus ibis	Cattle Egret	2022/1/20	1	0	22.5000000	114.1166670	https://www.inaturalist.org/observations/105354533
2585	Bubulcus ibis	Cattle Egret	2022/10/15	2	0	22.5012120	114.0721549	https://www.inaturalist.org/observations/138989718
2586	Bubulcus ibis	Cattle Egret	2021/5/1	2	0	22.5014568	114.0578203	https://www.inaturalist.org/observations/77380313
2587	Bubulcus ibis	Cattle Egret	2022/4/12	1	0	22.5015247	114.0720189	https://www.inaturalist.org/observations/111182590
2588	Bubulcus ibis	Cattle Egret	2019/11/2	0	0	22.5018802	114.0425071	https://www.inaturalist.org/observations/35186541
2589	Bubulcus ibis	Cattle Egret	2019/11/9	2	0	22.5024300	114.0609283	https://www.inaturalist.org/observations/35640311
2590	Bubulcus ibis	Cattle Egret	2022/4/10	1	0	22.5029609	114.0702515	https://www.inaturalist.org/observations/111050947
2591	Bubulcus ibis	Cattle Egret	2021/11/12	1	0	22.5033596	114.0692343	https://www.inaturalist.org/observations/101004953
2592	Bubulcus ibis	Cattle Egret	2021/11/15	1	0	22.5040343	88.3989157	https://www.inaturalist.org/observations/102364625
2593	Bubulcus ibis	Cattle Egret	2015/11/15	1	0	22.5044480	114.0676820	https://www.inaturalist.org/observations/36405047
2594	Bubulcus ibis	Cattle Egret	2019/11/27	1	0	22.5046410	114.0662260	https://www.inaturalist.org/observations/36238330
2595	Bubulcus ibis	Cattle Egret	2019/12/2	1	0	22.5046660	114.0681800	https://www.inaturalist.org/observations/36376099
2596	Bubulcus ibis	Cattle Egret	2021/10/22	1	0	22.5049136	114.1200014	https://www.inaturalist.org/observations/99110340
2597	Bubulcus ibis	Cattle Egret	2022/10/15	1	0	22.5050000	114.1196564	https://www.inaturalist.org/observations/138984675
2598	Bubulcus ibis	Cattle Egret	2021/11/6	1	0	22.5053351	114.1192158	https://www.inaturalist.org/observations/100461745
2599	Bubulcus ibis	Cattle Egret	2018/12/1	5	0	22.5058614	114.1165391	https://www.inaturalist.org/observations/18770430

2600	Bubulcus ibis	Cattle Egret	2021/5/1	2	0	22.5059750	114.1109850	https://www.inaturalist.org/observations/76921948
2601	Bubulcus ibis	Cattle Egret	2020/4/27	2	0	22.5060460	114.2430130	https://www.inaturalist.org/observations/44348435
2602	Bubulcus ibis	Cattle Egret	2020/11/23	1	0	22.5062030	114.1122130	https://www.inaturalist.org/observations/66653551
2603	Bubulcus ibis	Cattle Egret	2021/5/1	2	0	22.5062317	114.1105650	https://www.inaturalist.org/observations/76921956
2604	Bubulcus ibis	Cattle Egret	2020/2/18	0	0	22.5063683	114.1116233	https://www.inaturalist.org/observations/38980747
2605	Bubulcus ibis	Cattle Egret	2009/4/5	1	0	22.5069120	114.1183420	https://www.inaturalist.org/observations/39872473
2606	Bubulcus ibis	Cattle Egret	2008/11/30	1	0	22.5069320	114.1184600	https://www.inaturalist.org/observations/40012775
2607	Bubulcus ibis	Cattle Egret	2020/4/30	1	0	22.5071366	114.1127184	https://www.inaturalist.org/observations/44365104
2608	Bubulcus ibis	Cattle Egret	2020/4/27	1	0	22.5074438	114.2430017	https://www.inaturalist.org/observations/44468447
2609	Bubulcus ibis coromandus	Cattle Egret	2018/12/1	1	0	22.5074472	114.1151658	https://www.inaturalist.org/observations/33620317
2610	Bubulcus ibis	Cattle Egret	2010/4/6	0	0	22.5076450	114.1179770	https://www.inaturalist.org/observations/38703795
2611	Bubulcus ibis	Cattle Egret	2019/11/23	1	0	22.5076804	114.1182498	https://www.inaturalist.org/observations/35969873
2612	Bubulcus ibis	Cattle Egret	2017/12/6	3	0	22.5077337	114.0746737	https://www.inaturalist.org/observations/10613533
2613	Bubulcus ibis coromandus	Cattle Egret	2021/3/24	1	0	22.5077535	114.1134840	https://www.inaturalist.org/observations/73066277
2614	Bubulcus ibis coromandus	Cattle Egret	2021/5/3	1	0	22.5077535	114.1134840	https://www.inaturalist.org/observations/77371886
2615	Bubulcus ibis	Cattle Egret	2020/11/11	2	0	22.5077663	114.1127856	https://www.inaturalist.org/observations/64678476
2616	Bubulcus ibis	Cattle Egret	2019/9/21	1	0	22.5077751	114.1155592	https://www.inaturalist.org/observations/47387877
2617	Bubulcus ibis	Cattle Egret	2010/10/12	2	0	22.5078530	114.1179900	https://www.inaturalist.org/observations/109641917
2618	Bubulcus ibis	Cattle Egret	2021/4/1	1	0	22.5079233	114.1119574	https://www.inaturalist.org/observations/72592110
2619	Bubulcus ibis	Cattle Egret	2020/10/28	1	0	22.5079633	114.1126862	https://www.inaturalist.org/observations/63748667
2620	Bubulcus ibis	Cattle Egret	2018/4/29	4	0	22.5079770	114.1124019	https://www.inaturalist.org/observations/11945909
2621	Bubulcus ibis	Cattle Egret	2020/1/11	1	0	22.5080228	114.1182401	https://www.inaturalist.org/observations/37478298
2622	Bubulcus ibis	Cattle Egret	2019/11/23	1	0	22.5080916	114.1180670	https://www.inaturalist.org/observations/35969953
2623	Bubulcus ibis	Cattle Egret	2017/1/7	2	0	22.5081003	114.1148655	https://www.inaturalist.org/observations/28779844
2624	Bubulcus ibis	Cattle Egret	2017/1/7	2	0	22.5081003	114.1148655	https://www.inaturalist.org/observations/28780042

2625	Bubulcus ibis	Cattle Egret	2017/1/7	2	0	22.5081003	114.1148655	https://www.inaturalist.org/observations/28780480
2626	Bubulcus ibis	Cattle Egret	2017/1/7	2	0	22.5081003	114.1148655	https://www.inaturalist.org/observations/28780665
2627	Bubulcus ibis	Cattle Egret	2022/4/19	1	0	22.5081702	114.2822495	https://www.inaturalist.org/observations/112002890
2628	Bubulcus ibis	Cattle Egret	2009/9/6	1	0	22.5082210	114.1181460	https://www.inaturalist.org/observations/140651640
2629	Bubulcus ibis	Cattle Egret	2017/4/1	3	0	22.5082600	114.1149727	https://www.inaturalist.org/observations/29625592
2630	Bubulcus ibis	Cattle Egret	2017/4/1	2	0	22.5082600	114.1149727	https://www.inaturalist.org/observations/29626120
2631	Bubulcus ibis	Cattle Egret	2017/4/1	3	0	22.5082600	114.1149727	https://www.inaturalist.org/observations/29626349
2632	Bubulcus ibis	Cattle Egret	2017/4/1	3	0	22.5082600	114.1149727	https://www.inaturalist.org/observations/29691851
2633	Bubulcus ibis	Cattle Egret	2017/12/6	3	0	22.5085204	114.1130571	https://www.inaturalist.org/observations/10550583
2634	Bubulcus ibis coromandus	Cattle Egret	2020/4/28	1	0	22.5086366	114.1170924	https://www.inaturalist.org/observations/44102736
2635	Bubulcus ibis	Cattle Egret	2021/2/18	1	0	22.5086934	114.1125763	https://www.inaturalist.org/observations/71859788
2636	Bubulcus ibis	Cattle Egret	2021/10/31	2	0	22.5087374	114.1137251	https://www.inaturalist.org/observations/100154683
2637	Bubulcus ibis	Cattle Egret	2010/4/24	0	0	22.5087540	114.1127970	https://www.inaturalist.org/observations/38703673
2638	Bubulcus ibis	Cattle Egret	2017/1/21	3	0	22.5087952	114.1129771	https://www.inaturalist.org/observations/20958618
2639	Bubulcus ibis	Cattle Egret	2020/11/28	2	0	22.5087952	114.1129771	https://www.inaturalist.org/observations/65808729
2640	Bubulcus ibis	Cattle Egret	2010/2/19	2	0	22.5087952	114.1129771	https://www.inaturalist.org/observations/105487028
2641	Bubulcus ibis	Cattle Egret	2020/12/9	1	0	22.5087953	114.1129769	https://www.inaturalist.org/observations/66330539
2642	Bubulcus ibis	Cattle Egret	2021/3/29	1	0	22.5087953	114.1129769	https://www.inaturalist.org/observations/72353261
2643	Bubulcus ibis	Cattle Egret	2020/12/25	1	0	22.5087953	114.1129769	https://www.inaturalist.org/observations/101687177
2644	Bubulcus ibis	Cattle Egret	2022/12/4	1	0	22.5087953	114.1129769	https://www.inaturalist.org/observations/143934311
2645	Bubulcus ibis	Cattle Egret	2022/1/30	2	0	22.5087993	114.1129769	https://www.inaturalist.org/observations/105834720
2646	Bubulcus ibis	Cattle Egret	2017/5/6	1	0	22.5088176	114.1130161	https://www.inaturalist.org/observations/72428786
2647	Bubulcus ibis coromandus	Cattle Egret	2021/5/1	1	0	22.5089130	114.1131781	https://www.inaturalist.org/observations/76194672
2648	Bubulcus ibis	Cattle Egret	2022/6/19	5	0	22.5089786	73.4740233	https://www.inaturalist.org/observations/125050991
2649	Bubulcus ibis	Cattle Egret	2021/5/2	1	0	22.5091103	114.1132025	https://www.inaturalist.org/observations/76562427

2650	Bubulcus ibis	Cattle Egret	2020/4/20	1	0	22.5091210	114.1145410	https://www.inaturalist.org/observations/44678426
2651	Bubulcus ibis	Cattle Egret	2019/10/30	1	0	22.5091278	114.1134967	https://www.inaturalist.org/observations/35191030
2652	Bubulcus ibis	Cattle Egret	2021/11/22	1	0	22.5091400	88.3475618	https://www.inaturalist.org/observations/115399059
2653	Bubulcus ibis	Cattle Egret	2021/4/6	1	0	22.5092379	114.1126638	https://www.inaturalist.org/observations/73092668
2654	Bubulcus ibis	Cattle Egret	2020/12/21	2	0	22.5092696	114.1174133	https://www.inaturalist.org/observations/66914687
2655	Bubulcus ibis	Cattle Egret	2020/12/5	1	0	22.5092940	114.1111382	https://www.inaturalist.org/observations/69293533
2656	Bubulcus ibis	Cattle Egret	2018/12/28	4	0	22.5095221	114.1117867	https://www.inaturalist.org/observations/19271717
2657	Bubulcus ibis	Cattle Egret	2020/4/28	1	0	22.5097212	114.1144926	https://www.inaturalist.org/observations/44103865
2658	Bubulcus ibis	Cattle Egret	2020/1/21	0	0	22.5097888	114.1120050	https://www.inaturalist.org/observations/38029217
2659	Bubulcus ibis	Cattle Egret	2020/11/23	1	0	22.5098360	114.1120220	https://www.inaturalist.org/observations/65572942
2660	Bubulcus ibis	Cattle Egret	2021/10/30	1	0	22.5098409	114.1173412	https://www.inaturalist.org/observations/99858148
2661	Bubulcus ibis coromandus	Cattle Egret	2021/2/14	1	0	22.5099152	114.1146079	https://www.inaturalist.org/observations/73931956
2662	Bubulcus ibis	Cattle Egret	2021/12/20	2	0	22.5100063	88.3602349	https://www.inaturalist.org/observations/103361474
2663	Bubulcus ibis	Cattle Egret	2021/3/9	1	0	22.5100753	114.1173678	https://www.inaturalist.org/observations/70945696
2664	Bubulcus ibis	Cattle Egret	2019/3/22	1	0	22.5102417	114.1106325	https://www.inaturalist.org/observations/62556974
2665	Bubulcus ibis coromandus	Cattle Egret	2018/5/5	3	0	22.5102442	114.1157841	https://www.inaturalist.org/observations/12186062
2666	Bubulcus ibis	Cattle Egret	2019/11/10	2	0	22.5102840	114.1126220	https://www.inaturalist.org/observations/35774716
2667	Bubulcus ibis	Cattle Egret	2019/3/22	1	0	22.5103297	114.1106307	https://www.inaturalist.org/observations/62556977
2668	Bubulcus ibis	Cattle Egret	2020/11/22	1	0	22.5104402	114.1113534	https://www.inaturalist.org/observations/65407657
2669	Bubulcus ibis	Cattle Egret	2022/12/23	1	0	22.5105068	114.1165002	https://www.inaturalist.org/observations/145189124
2670	Bubulcus ibis	Cattle Egret	2021/12/28	2	0	22.5106467	114.1160028	https://www.inaturalist.org/observations/103810179
2671	Bubulcus ibis	Cattle Egret	2020/11/9	1	0	22.5107259	114.1135189	https://www.inaturalist.org/observations/64519229
2672	Bubulcus ibis coromandus	Cattle Egret	2021/5/8	1	0	22.5108539	114.1126198	https://www.inaturalist.org/observations/78041937
2673	Bubulcus ibis	Cattle Egret	2020/11/14	1	0	22.5115050	114.1129250	https://www.inaturalist.org/observations/64840245
2674	Bubulcus ibis	Cattle Egret	2020/2/16	1	0	22.5118360	114.1083690	https://www.inaturalist.org/observations/110954708

2675	Bubulcus ibis	Cattle Egret	2021/5/20	2	0	22.5119633	114.1309858	https://www.inaturalist.org/observations/79530114
2676	Bubulcus ibis	Cattle Egret	2020/11/30	3	0	22.5136049	88.3600248	https://www.inaturalist.org/observations/65877182
2677	Bubulcus ibis	Cattle Egret	2020/11/30	2	0	22.5136569	88.3599298	https://www.inaturalist.org/observations/65877119
2678	Bubulcus ibis	Cattle Egret	2020/11/30	4	0	22.5136582	88.3599468	https://www.inaturalist.org/observations/65876964
2679	Bubulcus ibis	Cattle Egret	2020/11/30	3	0	22.5136756	88.3598372	https://www.inaturalist.org/observations/65876956
2680	Bubulcus ibis	Cattle Egret	2021/8/3	1	0	22.5142553	114.1145997	https://www.inaturalist.org/observations/89754022
2681	Bubulcus ibis	Cattle Egret	2020/2/25	0	0	22.5142875	114.1130517	https://www.inaturalist.org/observations/39397839
2682	Bubulcus ibis coromandus	Cattle Egret	2021/4/15	1	0	22.5143347	114.1123681	https://www.inaturalist.org/observations/76015319
2683	Bubulcus ibis	Cattle Egret	2013/4/14	1	0	22.5144623	114.0536317	https://www.inaturalist.org/observations/37551181
2684	Bubulcus ibis	Cattle Egret	2020/3/22	1	0	22.5154198	114.1054685	https://www.inaturalist.org/observations/40569028
2685	Bubulcus ibis	Cattle Egret	2022/1/8	3	0	22.5180643	114.2707296	https://www.inaturalist.org/observations/104566556
2686	Bubulcus ibis	Cattle Egret	2013/7/21	1	0	22.5188470	114.1134050	https://www.inaturalist.org/observations/74120108
2687	Bubulcus ibis	Cattle Egret	2021/5/1	1	0	22.5188950	114.2164420	https://www.inaturalist.org/observations/76072825
2688	Bubulcus ibis	Cattle Egret	2021/10/23	1	0	22.5188950	114.2164420	https://www.inaturalist.org/observations/99464637
2689	Bubulcus ibis	Cattle Egret	2022/7/3	4	0	22.5191120	72.9702309	https://www.inaturalist.org/observations/124504895
2690	Bubulcus ibis	Cattle Egret	2019/4/28	4	0	22.5191633	114.1132250	https://www.inaturalist.org/observations/23561738
2691	Bubulcus ibis	Cattle Egret	2021/10/15	1	0	22.5195450	114.2159250	https://www.inaturalist.org/observations/98263400
2692	Bubulcus ibis	Cattle Egret	2014/4/26	4	0	22.5202920	114.1153840	https://www.inaturalist.org/observations/19684117
2693	Bubulcus ibis	Cattle Egret	2022/11/1	1	0	22.5206256	114.2140818	https://www.inaturalist.org/observations/140860001
2694	Bubulcus ibis	Cattle Egret	2022/4/15	1	0	22.5206290	114.2138563	https://www.inaturalist.org/observations/111507990
2695	Bubulcus ibis	Cattle Egret	2020/9/3	1	0	22.5208616	114.2087624	https://www.inaturalist.org/observations/58584446
2696	Bubulcus ibis	Cattle Egret	2015/4/2	1	0	22.5209277	114.2097701	https://www.inaturalist.org/observations/47943718
2697	Bubulcus ibis	Cattle Egret	2020/10/8	1	0	22.5210766	114.2163295	https://www.inaturalist.org/observations/62869453
2698	Bubulcus ibis	Cattle Egret	2020/5/28	1	0	22.5210979	114.2099162	https://www.inaturalist.org/observations/48105166
2699	Bubulcus ibis	Cattle Egret	2021/1/15	1	0	22.5231263	114.2160089	https://www.inaturalist.org/observations/68099297

2700	Bubulcus ibis	Cattle Egret	2022/5/19	1	0	22.5232328	114.2926371	https://www.inaturalist.org/observations/118313893
2701	Bubulcus ibis	Cattle Egret	2018/5/3	3	0	22.5236683	114.1142080	https://www.inaturalist.org/observations/12083735
2702	Bubulcus ibis	Cattle Egret	2021/1/28	2	0	22.5239160	114.2175468	https://www.inaturalist.org/observations/68753674
2703	Bubulcus ibis coromandus	Cattle Egret	2019/3/17	2	0	22.5251125	114.0859660	https://www.inaturalist.org/observations/21317026
2704	Bubulcus ibis	Cattle Egret	2020/12/27	1	0	22.5253750	114.2595700	https://www.inaturalist.org/observations/67128757
2705	Bubulcus ibis	Cattle Egret	2018/4/29	3	0	22.5255694	114.2589196	https://www.inaturalist.org/observations/11724265
2706	Bubulcus ibis	Cattle Egret	2020/11/15	2	0	22.5259354	114.2593443	https://www.inaturalist.org/observations/64916000
2707	Bubulcus ibis	Cattle Egret	2020/12/31	1	0	22.5262717	114.1111055	https://www.inaturalist.org/observations/67345150
2708	Bubulcus ibis	Cattle Egret	2021/11/27	2	0	22.5270220	114.0945800	https://www.inaturalist.org/observations/102161081
2709	Bubulcus ibis	Cattle Egret	2019/11/24	1	0	22.5273307	114.2286761	https://www.inaturalist.org/observations/38554122
2710	Bubulcus ibis	Cattle Egret	2009/4/10	1	0	22.5274510	114.2107270	https://www.inaturalist.org/observations/144723083
2711	Bubulcus ibis	Cattle Egret	2020/2/28	2	0	22.5274641	72.9336914	https://www.inaturalist.org/observations/53570974
2712	Bubulcus ibis	Cattle Egret	2021/11/6	2	0	22.5280100	114.2337703	https://www.inaturalist.org/observations/102236289
2713	Bubulcus ibis	Cattle Egret	2020/12/13	1	0	22.5282596	114.2226215	https://www.inaturalist.org/observations/66506006
2714	Bubulcus ibis	Cattle Egret	2020/12/13	1	0	22.5286063	114.2238448	https://www.inaturalist.org/observations/66506005
2715	Bubulcus ibis coromandus	Cattle Egret	2019/4/28	3	0	22.5297906	114.2345895	https://www.inaturalist.org/observations/24328039
2716	Bubulcus ibis	Cattle Egret	2020/5/3	1	0	22.5299029	114.2352897	https://www.inaturalist.org/observations/44691247
2717	Bubulcus ibis	Cattle Egret	2021/1/22	1	0	22.5317317	114.2368715	https://www.inaturalist.org/observations/68482340
2718	Bubulcus ibis	Cattle Egret	2022/11/5	4	0	22.5349018	114.1347252	https://www.inaturalist.org/observations/141151762
2719	Bubulcus ibis	Cattle Egret	2020/6/5	2	0	22.5360843	120.5888128	https://www.inaturalist.org/observations/48542435
2720	Bubulcus ibis coromandus	Cattle Egret	2018/10/20	3	0	22.5372929	114.1442404	https://www.inaturalist.org/observations/17668520
2721	Bubulcus ibis	Cattle Egret	2020/10/1	1	0	22.5387202	120.5903149	https://www.inaturalist.org/observations/61565116
2722	Bubulcus ibis	Cattle Egret	2022/8/13	3	0	22.5389191	88.3331132	https://www.inaturalist.org/observations/130730901
2723	Bubulcus ibis	Cattle Egret	2022/1/19	1	0	22.5413258	114.0352223	https://www.inaturalist.org/observations/105305747
2724	Bubulcus ibis	Cattle Egret	2021/5/3	1	0	22.5428599	114.0595600	https://www.inaturalist.org/observations/106361601

2725	Bubulcus ibis	Cattle Egret	2020/4/18	1	0	22.5444392	113.9611403	https://www.inaturalist.org/observations/66562588
2726	Bubulcus ibis	Cattle Egret	2020/10/31	1	0	22.5544381	114.1228762	https://www.inaturalist.org/observations/140626370
2727	Bubulcus ibis	Cattle Egret	2022/12/26	1	0	22.5551837	114.3024965	https://www.inaturalist.org/observations/145108540
2728	Bubulcus ibis	Cattle Egret	2021/12/22	1	0	22.5574981	88.2917216	https://www.inaturalist.org/observations/104274285
2729	Bubulcus ibis	Cattle Egret	2020/10/20	2	0	22.5641146	120.5376474	https://www.inaturalist.org/observations/63170099
2730	Bubulcus ibis	Cattle Egret	2022/2/3	1	0	22.5669294	114.1146683	https://www.inaturalist.org/observations/120511076
2731	Bubulcus ibis	Cattle Egret	2022/10/17	1	0	22.5678829	120.3671519	https://www.inaturalist.org/observations/139088921
2732	Bubulcus ibis	Cattle Egret	2022/10/29	2	0	22.5697778	120.3670742	https://www.inaturalist.org/observations/140371842
2733	Bubulcus ibis	Cattle Egret	2022/2/2	1	0	22.5727475	114.1595979	https://www.inaturalist.org/observations/106014339
2734	Bubulcus ibis	Cattle Egret	2022/2/27	1	0	22.5728759	114.0604130	https://www.inaturalist.org/observations/107564210
2735	Bubulcus ibis	Cattle Egret	2022/7/16	1	0	22.5732915	120.4545224	https://www.inaturalist.org/observations/140837560
2736	Bubulcus ibis	Cattle Egret	2020/10/3	1	0	22.5749086	114.1982562	https://www.inaturalist.org/observations/62479958
2737	Bubulcus ibis	Cattle Egret	2018/3/12	3	0	22.5774552	88.4508430	https://www.inaturalist.org/observations/23117683
2738	Bubulcus ibis	Cattle Egret	2020/10/28	1	0	22.5783367	120.3490703	https://www.inaturalist.org/observations/63964481
2739	Bubulcus ibis	Cattle Egret	2022/2/17	3	0	22.5796821	120.3506109	https://www.inaturalist.org/observations/106908025
2740	Bubulcus ibis	Cattle Egret	2021/4/11	1	0	22.5798846	114.0845043	https://www.inaturalist.org/observations/76063244
2741	Bubulcus ibis	Cattle Egret	2022/4/21	2	0	22.5811626	114.1891720	https://www.inaturalist.org/observations/113104959
2742	Bubulcus ibis	Cattle Egret	2022/2/27	3	0	22.5841697	72.9499866	https://www.inaturalist.org/observations/107563325
2743	Bubulcus ibis	Cattle Egret	2022/2/8	1	0	22.5841972	120.3416103	https://www.inaturalist.org/observations/106380364
2744	Bubulcus ibis	Cattle Egret	2021/12/25	1	0	22.5845547	120.3966746	https://www.inaturalist.org/observations/103754530
2745	Bubulcus ibis	Cattle Egret	2022/10/24	2	0	22.5853735	120.5781298	https://www.inaturalist.org/observations/139849359
2746	Bubulcus ibis	Cattle Egret	2011/10/29	5	0	22.5864293	88.4135719	https://www.inaturalist.org/observations/25504212
2747	Bubulcus ibis	Cattle Egret	2014/11/9	2	0	22.5866805	88.4165709	https://www.inaturalist.org/observations/60171422
2748	Bubulcus ibis	Cattle Egret	2014/11/9	3	0	22.5866805	88.4165709	https://www.inaturalist.org/observations/60171428
2749	Bubulcus ibis	Cattle Egret	2014/11/9	3	0	22.5866805	88.4165709	https://www.inaturalist.org/observations/60171431

2750	Bubulcus ibis	Cattle Egret	2022/2/8	1	0	22.5870131	120.3525318	https://www.inaturalist.org/observations/106380356
2751	Bubulcus ibis	Cattle Egret	2021/2/1	1	0	22.5871357	88.4151858	https://www.inaturalist.org/observations/69797246
2752	Bubulcus ibis	Cattle Egret	2021/1/3	1	0	22.5872247	120.3919090	https://www.inaturalist.org/observations/67570219
2753	Bubulcus ibis	Cattle Egret	2020/12/8	1	0	22.5917702	114.0609882	https://www.inaturalist.org/observations/66462901
2754	Bubulcus ibis	Cattle Egret	2022/10/26	1	0	22.5921073	120.3535845	https://www.inaturalist.org/observations/140032976
2755	Bubulcus ibis	Cattle Egret	2021/5/4	1	0	22.5929210	120.3541610	https://www.inaturalist.org/observations/77435390
2756	Bubulcus ibis	Cattle Egret	2021/3/8	1	0	22.5930124	120.3537183	https://www.inaturalist.org/observations/70936176
2757	Bubulcus ibis	Cattle Egret	2022/11/14	3	0	22.5933816	120.3537737	https://www.inaturalist.org/observations/142114379
2758	Bubulcus ibis	Cattle Egret	2022/10/26	1	0	22.5949401	120.3554571	https://www.inaturalist.org/observations/140032987
2759	Bubulcus ibis	Cattle Egret	2018/10/24	2	0	22.5962638	120.9638488	https://www.inaturalist.org/observations/17871534
2760	Bubulcus ibis	Cattle Egret	2022/10/8	2	0	22.5988966	87.9754847	https://www.inaturalist.org/observations/138841706
2761	Bubulcus ibis	Cattle Egret	2022/2/27	1	0	22.5999339	114.1265767	https://www.inaturalist.org/observations/107564114
2762	Bubulcus ibis	Cattle Egret	2021/9/18	1	0	22.6065647	120.3560270	https://www.inaturalist.org/observations/95393003
2763	Bubulcus ibis	Cattle Egret	2022/11/19	3	0	22.6066880	87.3107300	https://www.inaturalist.org/observations/142375179
2764	Bubulcus ibis	Cattle Egret	2022/2/18	2	0	22.6073900	73.5559967	https://www.inaturalist.org/observations/119838463
2765	Bubulcus ibis	Cattle Egret	2020/11/21	5	0	22.6091925	88.4644508	https://www.inaturalist.org/observations/108032723
2766	Bubulcus ibis	Cattle Egret	2021/12/14	1	0	22.6126840	120.3583920	https://www.inaturalist.org/observations/103091320
2767	Bubulcus ibis	Cattle Egret	2021/5/12	2	0	22.6160709	120.3769398	https://www.inaturalist.org/observations/78566612
2768	Bubulcus ibis	Cattle Egret	2020/5/19	1	0	22.6165151	120.3750482	https://www.inaturalist.org/observations/46450586
2769	Bubulcus ibis	Cattle Egret	2017/10/30	2	0	22.6172395	120.3392708	https://www.inaturalist.org/observations/12607365
2770	Bubulcus ibis	Cattle Egret	2022/9/24	2	0	22.6180946	120.3774648	https://www.inaturalist.org/observations/136238547
2771	Bubulcus ibis	Cattle Egret	2020/4/28	1	0	22.6181997	120.2688029	https://www.inaturalist.org/observations/44104928
2772	Bubulcus ibis	Cattle Egret	2017/11/16	4	0	22.6197682	120.3395529	https://www.inaturalist.org/observations/9482372
2773	Bubulcus ibis	Cattle Egret	2018/9/7	3	0	22.6197682	120.3395529	https://www.inaturalist.org/observations/16344098
2774	Bubulcus ibis	Cattle Egret	2017/10/26	2	0	22.6197682	120.3395529	https://www.inaturalist.org/observations/20022047

2775	Bubulcus ibis	Cattle Egret	2017/9/14	2	0	22.6197682	120.3395529	https://www.inaturalist.org/observations/20022123
2776	Bubulcus ibis	Cattle Egret	2022/5/9	1	0	22.6197682	120.3395529	https://www.inaturalist.org/observations/116415514
2777	Bubulcus ibis	Cattle Egret	2022/10/5	1	0	22.6197682	120.3395529	https://www.inaturalist.org/observations/137603851
2778	Bubulcus ibis	Cattle Egret	2019/12/8	1	0	22.6197749	120.3395498	https://www.inaturalist.org/observations/36460185
2779	Bubulcus ibis	Cattle Egret	2020/4/9	1	0	22.6197749	120.3395498	https://www.inaturalist.org/observations/41758276
2780	Bubulcus ibis	Cattle Egret	2021/11/17	1	0	22.6197947	120.3395176	https://www.inaturalist.org/observations/101396220
2781	Bubulcus ibis	Cattle Egret	2018/9/7	3	0	22.6200035	120.3392204	https://www.inaturalist.org/observations/16343948
2782	Bubulcus ibis	Cattle Egret	2017/8/21	2	0	22.6200348	120.3413361	https://www.inaturalist.org/observations/12607363
2783	Bubulcus ibis	Cattle Egret	2020/8/17	2	0	22.6201469	120.3382546	https://www.inaturalist.org/observations/56725388
2784	Bubulcus ibis	Cattle Egret	2020/8/17	2	0	22.6202163	120.3382439	https://www.inaturalist.org/observations/56725387
2785	Bubulcus ibis	Cattle Egret	2020/8/17	2	0	22.6202460	120.3382653	https://www.inaturalist.org/observations/56725390
2786	Bubulcus ibis	Cattle Egret	2018/1/31	1	0	22.6205425	120.3401328	https://www.inaturalist.org/observations/123676029
2787	Bubulcus ibis	Cattle Egret	2021/12/3	3	0	22.6208915	88.4031475	https://www.inaturalist.org/observations/102449063
2788	Bubulcus ibis	Cattle Egret	2018/1/11	1	0	22.6209799	120.3406018	https://www.inaturalist.org/observations/123674626
2789	Bubulcus ibis	Cattle Egret	2020/8/17	2	0	22.6212686	120.3394563	https://www.inaturalist.org/observations/56725342
2790	Bubulcus ibis	Cattle Egret	2022/2/14	3	0	22.6216397	120.3637671	https://www.inaturalist.org/observations/106734009
2791	Bubulcus ibis	Cattle Egret	2021/5/8	1	0	22.6219754	120.3874398	https://www.inaturalist.org/observations/78316560
2792	Bubulcus ibis	Cattle Egret	2018/12/31	5	0	22.6221200	120.3816300	https://www.inaturalist.org/observations/19368388
2793	Bubulcus ibis	Cattle Egret	2021/11/3	1	0	22.6226225	120.3375489	https://www.inaturalist.org/observations/100124240
2794	Bubulcus ibis	Cattle Egret	2021/11/3	1	0	22.6226364	120.3374899	https://www.inaturalist.org/observations/100124243
2795	Bubulcus ibis	Cattle Egret	2021/11/3	1	0	22.6232668	120.3380564	https://www.inaturalist.org/observations/100118278
2796	Bubulcus ibis	Cattle Egret	2021/2/18	1	0	22.6234857	120.3860903	https://www.inaturalist.org/observations/69809474
2797	Bubulcus ibis	Cattle Egret	2022/2/14	3	0	22.6254623	120.3657416	https://www.inaturalist.org/observations/106733989
2798	Bubulcus ibis	Cattle Egret	2021/2/25	1	0	22.6256145	120.5972734	https://www.inaturalist.org/observations/70249055
2799	Bubulcus ibis	Cattle Egret	2021/3/23	1	0	22.6261957	120.5973131	https://www.inaturalist.org/observations/72010149

2800	Bubulcus ibis	Cattle Egret	2020/4/26	1	0	22.6317444	120.2615250	https://www.inaturalist.org/observations/43533867
2801	Bubulcus ibis	Cattle Egret	2022/10/19	1	0	22.6326610	114.4388724	https://www.inaturalist.org/observations/141769992
2802	Bubulcus ibis	Cattle Egret	2022/4/13	1	0	22.6335387	114.4394060	https://www.inaturalist.org/observations/112014833
2803	Bubulcus ibis	Cattle Egret	2022/9/26	3	0	22.6348141	120.3007438	https://www.inaturalist.org/observations/136560678
2804	Bubulcus ibis	Cattle Egret	2022/9/26	1	0	22.6366119	120.2775345	https://www.inaturalist.org/observations/136548360
2805	Bubulcus ibis	Cattle Egret	2019/12/27	1	0	22.6386398	120.6154847	https://www.inaturalist.org/observations/36991486
2806	Bubulcus ibis	Cattle Egret	2018/3/29	1	0	22.6387801	120.2966167	https://www.inaturalist.org/observations/15777795
2807	Bubulcus ibis	Cattle Egret	2020/9/24	2	0	22.6395594	120.6167975	https://www.inaturalist.org/observations/60579827
2808	Bubulcus ibis	Cattle Egret	2021/7/24	1	0	22.6397039	120.6141167	https://www.inaturalist.org/observations/88936571
2809	Bubulcus ibis	Cattle Egret	2022/7/2	1	0	22.6411256	120.3587671	https://www.inaturalist.org/observations/124366743
2810	Bubulcus ibis	Cattle Egret	2020/12/20	3	0	22.6413102	120.6168718	https://www.inaturalist.org/observations/66915262
2811	Bubulcus ibis	Cattle Egret	2020/10/1	1	0	22.6414771	120.6063102	https://www.inaturalist.org/observations/61565107
2812	Bubulcus ibis	Cattle Egret	2022/1/1	2	0	22.6442951	120.2765004	https://www.inaturalist.org/observations/106011578
2813	Bubulcus ibis	Cattle Egret	2021/2/27	1	0	22.6445400	120.2821964	https://www.inaturalist.org/observations/70736065
2814	Bubulcus ibis	Cattle Egret	2022/4/4	3	0	22.6450053	120.2751066	https://www.inaturalist.org/observations/120821134
2815	Bubulcus ibis	Cattle Egret	2022/4/4	3	0	22.6453809	120.2749435	https://www.inaturalist.org/observations/120821135
2816	Bubulcus ibis	Cattle Egret	2022/4/4	3	0	22.6454924	120.2742312	https://www.inaturalist.org/observations/120821137
2817	Bubulcus ibis	Cattle Egret	2022/12/19	1	0	22.6457333	120.2744244	https://www.inaturalist.org/observations/144661178
2818	Bubulcus ibis	Cattle Egret	2020/10/23	1	0	22.6461642	120.2860095	https://www.inaturalist.org/observations/63383266
2819	Bubulcus ibis	Cattle Egret	2020/9/24	2	0	22.6463816	120.6054510	https://www.inaturalist.org/observations/60579823
2820	Bubulcus ibis	Cattle Egret	2019/10/28	1	0	22.6485519	120.6043375	https://www.inaturalist.org/observations/35005452
2821	Bubulcus ibis	Cattle Egret	2020/11/16	1	0	22.6500210	120.2953140	https://www.inaturalist.org/observations/64997778
2822	Bubulcus ibis	Cattle Egret	2020/11/10	1	0	22.6521160	120.4419136	https://www.inaturalist.org/observations/64727211
2823	Bubulcus ibis	Cattle Egret	2015/10/22	1	0	22.6523001	120.3501560	https://www.inaturalist.org/observations/132011997
2824	Bubulcus ibis	Cattle Egret	2021/2/7	1	0	22.6525433	120.3500904	https://www.inaturalist.org/observations/69199320

2825	Bubulcus ibis	Cattle Egret	2020/9/27	1	0	22.6529065	120.3499119	https://www.inaturalist.org/observations/62772888
2826	Bubulcus ibis	Cattle Egret	2022/4/17	2	0	22.6551967	120.2849633	https://www.inaturalist.org/observations/111783725
2827	Bubulcus ibis	Cattle Egret	2021/12/28	1	0	22.6553400	120.2736580	https://www.inaturalist.org/observations/103937060
2828	Bubulcus ibis	Cattle Egret	2022/1/4	1	0	22.6554930	120.2736370	https://www.inaturalist.org/observations/104332368
2829	Bubulcus ibis	Cattle Egret	2022/4/16	2	0	22.6562843	87.7358856	https://www.inaturalist.org/observations/111605865
2830	Bubulcus ibis	Cattle Egret	2018/3/31	4	0	22.6564323	120.2862630	https://www.inaturalist.org/observations/10524874
2831	Bubulcus ibis	Cattle Egret	2021/10/27	1	0	22.6568268	120.2846776	https://www.inaturalist.org/observations/99543873
2832	Bubulcus ibis	Cattle Egret	2022/7/10	1	0	22.6574690	120.3473279	https://www.inaturalist.org/observations/125602471
2833	Bubulcus ibis	Cattle Egret	2018/4/7	3	0	22.6574895	120.2884249	https://www.inaturalist.org/observations/13579824
2834	Bubulcus ibis	Cattle Egret	2021/10/23	2	0	22.6575956	120.2864372	https://www.inaturalist.org/observations/99047763
2835	Bubulcus ibis	Cattle Egret	2021/10/23	1	0	22.6578364	120.2859267	https://www.inaturalist.org/observations/99051143
2836	Bubulcus ibis	Cattle Egret	2021/9/6	1	0	22.6578967	120.2855317	https://www.inaturalist.org/observations/94067020
2837	Bubulcus ibis	Cattle Egret	2018/10/21	2	0	22.6579573	120.2859455	https://www.inaturalist.org/observations/17711770
2838	Bubulcus ibis	Cattle Egret	2021/5/30	2	0	22.6580583	120.2857389	https://www.inaturalist.org/observations/80849468
2839	Bubulcus ibis	Cattle Egret	2022/11/13	1	0	22.6583361	120.2974361	https://www.inaturalist.org/observations/141860091
2840	Bubulcus ibis	Cattle Egret	2021/1/1	1	0	22.6583472	120.2871083	https://www.inaturalist.org/observations/67386936
2841	Bubulcus ibis	Cattle Egret	2022/12/23	1	0	22.6592888	120.2857224	https://www.inaturalist.org/observations/144900286
2842	Bubulcus ibis	Cattle Egret	2017/3/14	5	0	22.6601952	78.1549358	https://www.inaturalist.org/observations/8085938
2843	Bubulcus ibis	Cattle Egret	2018/4/22	1	0	22.6603607	120.3538060	https://www.inaturalist.org/observations/82125546
2844	Bubulcus ibis	Cattle Egret	2022/3/25	2	0	22.6615961	120.2842622	https://www.inaturalist.org/observations/109438568
2845	Bubulcus ibis	Cattle Egret	2022/11/10	2	0	22.6623198	120.4288502	https://www.inaturalist.org/observations/141606540
2846	Bubulcus ibis	Cattle Egret	2021/10/26	2	0	22.6626406	120.3342819	https://www.inaturalist.org/observations/99536484
2847	Bubulcus ibis	Cattle Egret	2018/10/29	2	0	22.6631947	120.4283324	https://www.inaturalist.org/observations/17930208
2848	Bubulcus ibis	Cattle Egret	2022/11/10	1	0	22.6637346	120.4288814	https://www.inaturalist.org/observations/141606541
2849	Bubulcus ibis	Cattle Egret	2022/11/8	1	0	22.6683962	120.3273373	https://www.inaturalist.org/observations/141434954

2850	Bubulcus ibis	Cattle Egret	2022/8/19	2	0	22.6692760	120.3272870	https://www.inaturalist.org/observations/131461351
2851	Bubulcus ibis	Cattle Egret	2022/2/1	1	0	22.6693004	120.3283059	https://www.inaturalist.org/observations/106016446
2852	Bubulcus ibis	Cattle Egret	2021/9/17	1	0	22.6716787	120.3487035	https://www.inaturalist.org/observations/95980796
2853	Bubulcus ibis	Cattle Egret	2021/11/12	2	0	22.6752599	120.2903257	https://www.inaturalist.org/observations/101188541
2854	Bubulcus ibis	Cattle Egret	2020/2/11	0	0	22.6758611	120.2902028	https://www.inaturalist.org/observations/38529375
2855	Bubulcus ibis	Cattle Egret	2022/9/10	3	0	22.6759333	120.2897667	https://www.inaturalist.org/observations/134551429
2856	Bubulcus ibis	Cattle Egret	2020/6/12	0	0	22.6760056	120.2937556	https://www.inaturalist.org/observations/49296001
2857	Bubulcus ibis	Cattle Egret	2019/12/17	3	0	22.6760807	120.2903519	https://www.inaturalist.org/observations/36720498
2858	Bubulcus ibis	Cattle Egret	2021/10/17	1	0	22.6764050	120.2908667	https://www.inaturalist.org/observations/98493943
2859	Bubulcus ibis	Cattle Egret	2020/11/24	1	0	22.6764136	120.2877198	https://www.inaturalist.org/observations/65513892
2860	Bubulcus ibis	Cattle Egret	2020/11/24	1	0	22.6764200	120.2883300	https://www.inaturalist.org/observations/65530072
2861	Bubulcus ibis	Cattle Egret	2020/11/24	1	0	22.6765241	120.2874408	https://www.inaturalist.org/observations/65513891
2862	Bubulcus ibis	Cattle Egret	2021/10/3	1	0	22.6766550	120.2887117	https://www.inaturalist.org/observations/97020751
2863	Bubulcus ibis	Cattle Egret	2020/12/1	1	0	22.6772385	120.2936385	https://www.inaturalist.org/observations/65924684
2864	Bubulcus ibis	Cattle Egret	2020/12/1	0	0	22.6772633	120.2936550	https://www.inaturalist.org/observations/65925416
2865	Bubulcus ibis	Cattle Egret	2020/12/1	1	0	22.6773149	120.2935693	https://www.inaturalist.org/observations/65924690
2866	Bubulcus ibis	Cattle Egret	2020/12/1	1	0	22.6774835	120.2939813	https://www.inaturalist.org/observations/65924720
2867	Bubulcus ibis	Cattle Egret	2020/2/18	2	0	22.6779079	72.8816376	https://www.inaturalist.org/observations/38788168
2868	Bubulcus ibis	Cattle Egret	2020/12/1	1	0	22.6786200	120.2944117	https://www.inaturalist.org/observations/65927296
2869	Bubulcus ibis	Cattle Egret	2020/12/1	1	0	22.6786333	120.2943800	https://www.inaturalist.org/observations/65927117
2870	Bubulcus ibis	Cattle Egret	2020/12/1	0	0	22.6786591	120.2944964	https://www.inaturalist.org/observations/65925581
2871	Bubulcus ibis	Cattle Egret	2020/12/1	2	0	22.6786687	120.2943993	https://www.inaturalist.org/observations/65925592
2872	Bubulcus ibis	Cattle Egret	2020/12/1	2	0	22.6786887	120.2944735	https://www.inaturalist.org/observations/65925607
2873	Bubulcus ibis	Cattle Egret	2020/12/1	1	0	22.6787025	120.2948848	https://www.inaturalist.org/observations/65924806
2874	Bubulcus ibis	Cattle Egret	2020/12/1	2	0	22.6787150	120.2944510	https://www.inaturalist.org/observations/65925578

2875	Bubulcus ibis	Cattle Egret	2019/11/6	3	0	22.6787643	120.2939987	https://www.inaturalist.org/observations/35362518
2876	Bubulcus ibis	Cattle Egret	2020/12/1	1	0	22.6790558	120.2934775	https://www.inaturalist.org/observations/65926970
2877	Bubulcus ibis	Cattle Egret	2020/2/26	1	0	22.6795367	72.8800800	https://www.inaturalist.org/observations/39235813
2878	Bubulcus ibis	Cattle Egret	2020/2/15	2	0	22.6796285	72.8801579	https://www.inaturalist.org/observations/38661688
2879	Bubulcus ibis	Cattle Egret	2020/2/26	2	0	22.6798286	72.8803330	https://www.inaturalist.org/observations/39236364
2880	Bubulcus ibis	Cattle Egret	2020/2/18	2	0	22.6802291	72.8797936	https://www.inaturalist.org/observations/39310229
2881	Bubulcus ibis	Cattle Egret	2020/2/15	2	0	22.6802447	72.8801988	https://www.inaturalist.org/observations/38737123
2882	Bubulcus ibis	Cattle Egret	2018/2/24	4	0	22.6805540	120.2982190	https://www.inaturalist.org/observations/18349851
2883	Bubulcus ibis	Cattle Egret	2020/12/1	1	0	22.6820571	120.2954764	https://www.inaturalist.org/observations/65932258
2884	Bubulcus ibis	Cattle Egret	2022/12/3	1	0	22.6824316	120.4513265	https://www.inaturalist.org/observations/143546997
2885	Bubulcus ibis	Cattle Egret	2022/1/26	2	0	22.6827273	120.2982377	https://www.inaturalist.org/observations/105661999
2886	Bubulcus ibis	Cattle Egret	2017/11/5	2	0	22.6832085	120.2983753	https://www.inaturalist.org/observations/13110887
2887	Bubulcus ibis	Cattle Egret	2019/11/20	1	0	22.6843948	120.2950058	https://www.inaturalist.org/observations/35881764
2888	Bubulcus ibis	Cattle Egret	2020/12/20	3	0	22.6846140	88.3208530	https://www.inaturalist.org/observations/66907122
2889	Bubulcus ibis	Cattle Egret	2019/8/10	3	0	22.6847534	120.6397781	https://www.inaturalist.org/observations/30512487
2890	Bubulcus ibis	Cattle Egret	2020/12/20	3	0	22.6849034	88.3206299	https://www.inaturalist.org/observations/67046463
2891	Bubulcus ibis	Cattle Egret	2020/12/13	2	0	22.6857761	88.3213234	https://www.inaturalist.org/observations/66576896
2892	Bubulcus ibis	Cattle Egret	2020/12/13	2	0	22.6857761	88.3213234	https://www.inaturalist.org/observations/66657992
2893	Bubulcus ibis	Cattle Egret	2019/12/2	2	0	22.6861324	120.6380367	https://www.inaturalist.org/observations/36287684
2894	Bubulcus ibis	Cattle Egret	2020/1/12	1	0	22.6864773	120.3611275	https://www.inaturalist.org/observations/37516400
2895	Bubulcus ibis	Cattle Egret	2021/8/25	1	0	22.6866520	120.3258116	https://www.inaturalist.org/observations/92547561
2896	Bubulcus ibis	Cattle Egret	2020/2/16	3	0	22.6871710	88.3207484	https://www.inaturalist.org/observations/38788022
2897	Bubulcus ibis	Cattle Egret	2019/10/20	2	0	22.6873660	120.9916050	https://www.inaturalist.org/observations/34760903
2898	Bubulcus ibis	Cattle Egret	2019/11/15	2	0	22.6876917	120.9905783	https://www.inaturalist.org/observations/35691862
2899	Bubulcus ibis	Cattle Egret	2020/2/18	1	0	22.6880868	72.8388435	https://www.inaturalist.org/observations/38788210

2900	Bubulcus ibis	Cattle Egret	2022/2/6	2	0	22.6913324	120.3270691	https://www.inaturalist.org/observations/106228433
2901	Bubulcus ibis	Cattle Egret	2020/2/17	2	0	22.6915853	72.8633636	https://www.inaturalist.org/observations/39235893
2902	Bubulcus ibis	Cattle Egret	2022/6/22	3	0	22.6926909	120.3293555	https://www.inaturalist.org/observations/122920978
2903	Bubulcus ibis	Cattle Egret	2020/3/18	2	0	22.6962939	121.0194579	https://www.inaturalist.org/observations/40170462
2904	Bubulcus ibis	Cattle Egret	2021/10/16	1	0	22.7014351	120.6442598	https://www.inaturalist.org/observations/98482571
2905	Bubulcus ibis	Cattle Egret	2020/10/16	1	0	22.7025183	120.2965836	https://www.inaturalist.org/observations/62790391
2906	Bubulcus ibis	Cattle Egret	2021/2/26	2	0	22.7030528	120.2944833	https://www.inaturalist.org/observations/70201246
2907	Bubulcus ibis	Cattle Egret	2020/9/25	1	0	22.7055552	120.3686839	https://www.inaturalist.org/observations/62116466
2908	Bubulcus ibis	Cattle Egret	2017/4/11	2	0	22.7062335	121.0454047	https://www.inaturalist.org/observations/9112001
2909	Bubulcus ibis	Cattle Egret	2019/4/15	2	0	22.7070320	120.3541150	https://www.inaturalist.org/observations/24574913
2910	Bubulcus ibis	Cattle Egret	2020/9/25	1	0	22.7076039	120.3670532	https://www.inaturalist.org/observations/62116357
2911	Bubulcus ibis	Cattle Egret	2015/11/4	2	0	22.7098880	120.4910102	https://www.inaturalist.org/observations/15195194
2912	Bubulcus ibis	Cattle Egret	2021/7/25	1	0	22.7101790	120.5072810	https://www.inaturalist.org/observations/88936541
2913	Bubulcus ibis	Cattle Egret	2020/10/3	1	0	22.7103058	120.5085450	https://www.inaturalist.org/observations/61565131
2914	Bubulcus ibis	Cattle Egret	2019/1/25	4	0	22.7109509	120.5086736	https://www.inaturalist.org/observations/19859417
2915	Bubulcus ibis	Cattle Egret	2020/5/15	1	0	22.7110427	120.5092624	https://www.inaturalist.org/observations/46095331
2916	Bubulcus ibis	Cattle Egret	2021/4/18	1	0	22.7130460	115.4472769	https://www.inaturalist.org/observations/80077984
2917	Bubulcus ibis	Cattle Egret	2020/2/22	1	0	22.7156184	120.4740539	https://www.inaturalist.org/observations/39276862
2918	Bubulcus ibis	Cattle Egret	2018/5/18	2	0	22.7160375	120.4734477	https://www.inaturalist.org/observations/14067169
2919	Bubulcus ibis	Cattle Egret	2021/4/18	1	0	22.7163368	115.4698410	https://www.inaturalist.org/observations/80082426
2920	Bubulcus ibis	Cattle Egret	2018/9/8	3	0	22.7173338	120.4716315	https://www.inaturalist.org/observations/16350953
2921	Bubulcus ibis	Cattle Egret	2022/8/20	2	0	22.7195750	120.5150735	https://www.inaturalist.org/observations/131558762
2922	Bubulcus ibis	Cattle Egret	2021/9/28	1	0	22.7196059	120.5150199	https://www.inaturalist.org/observations/96675166
2923	Bubulcus ibis	Cattle Egret	2020/11/10	1	0	22.7198351	120.5149066	https://www.inaturalist.org/observations/64727210
2924	Bubulcus ibis	Cattle Egret	2021/4/18	1	0	22.7204564	115.4649094	https://www.inaturalist.org/observations/80082435

2925	Bubulcus ibis	Cattle Egret	2022/10/18	1	0	22.7266556	120.2854722	https://www.inaturalist.org/observations/139212121
2926	Bubulcus ibis	Cattle Egret	2022/10/18	3	0	22.7295917	120.2865917	https://www.inaturalist.org/observations/139212210
2927	Bubulcus ibis	Cattle Egret	2022/9/15	1	0	22.7320972	120.3123111	https://www.inaturalist.org/observations/135115938
2928	Bubulcus ibis	Cattle Egret	2020/7/13	2	0	22.7338956	120.3148057	https://www.inaturalist.org/observations/52937889
2929	Bubulcus ibis	Cattle Egret	2020/7/13	1	0	22.7340737	120.3144623	https://www.inaturalist.org/observations/52937884
2930	Bubulcus ibis	Cattle Egret	2022/11/11	2	0	22.7398165	88.3797724	https://www.inaturalist.org/observations/141692786
2931	Bubulcus ibis coromandus	Cattle Egret	2022/5/5	2	0	22.7418412	88.3787428	https://www.inaturalist.org/observations/126971030
2932	Bubulcus ibis coromandus	Cattle Egret	2022/5/5	1	0	22.7422716	88.3786308	https://www.inaturalist.org/observations/127052410
2933	Bubulcus ibis	Cattle Egret	2013/1/27	1	0	22.7449690	120.6378320	https://www.inaturalist.org/observations/82256048
2934	Bubulcus ibis	Cattle Egret	2020/9/26	2	0	22.7555316	120.2695268	https://www.inaturalist.org/observations/129821822
2935	Bubulcus ibis	Cattle Egret	2021/10/2	1	0	22.7714136	120.3360592	https://www.inaturalist.org/observations/97025957
2936	Bubulcus ibis	Cattle Egret	2022/4/4	1	0	22.7832231	120.2455033	https://www.inaturalist.org/observations/145239230
2937	Bubulcus ibis	Cattle Egret	2022/8/31	3	0	22.7860667	72.6429133	https://www.inaturalist.org/observations/133092041
2938	Bubulcus ibis	Cattle Egret	2022/6/26	4	0	22.7924536	88.2959795	https://www.inaturalist.org/observations/123489186
2939	Bubulcus ibis	Cattle Egret	2022/1/3	1	0	22.7940654	121.1258666	https://www.inaturalist.org/observations/106015880
2940	Bubulcus ibis	Cattle Egret	2020/11/7	1	0	22.7943000	120.2704722	https://www.inaturalist.org/observations/64339640
2941	Bubulcus ibis	Cattle Egret	2020/12/22	1	0	22.7980874	86.9464955	https://www.inaturalist.org/observations/71212858
2942	Bubulcus ibis	Cattle Egret	2018/2/13	1	0	22.7981700	120.5506700	https://www.inaturalist.org/observations/27987173
2943	Bubulcus ibis	Cattle Egret	2022/8/21	3	0	22.7982682	88.2680172	https://www.inaturalist.org/observations/134753057
2944	Bubulcus ibis	Cattle Egret	2020/8/30	1	0	22.8041604	88.3339234	https://www.inaturalist.org/observations/68067117
2945	Bubulcus ibis	Cattle Egret	2022/11/27	1	0	22.8049618	88.3320821	https://www.inaturalist.org/observations/143043924
2946	Bubulcus ibis	Cattle Egret	2022/10/9	3	0	22.8056934	88.3314236	https://www.inaturalist.org/observations/139247246
2947	Bubulcus ibis coromandus	Cattle Egret	2014/3/7	1	0	22.8072175	121.1283559	https://www.inaturalist.org/observations/92613662
2948	Bubulcus ibis	Cattle Egret	2019/7/25	2	0	22.8116197	120.2484208	https://www.inaturalist.org/observations/29609636
2949	Bubulcus ibis	Cattle Egret	2007/3/17	2	0	22.8188528	72.0444822	https://www.inaturalist.org/observations/54906883

2950	Bubulcus ibis	Cattle Egret	2012/7/9	3	0	22.8194461	72.0634448	https://www.inaturalist.org/observations/32857431
2951	Bubulcus ibis	Cattle Egret	2012/1/5	2	0	22.8209477	72.0571863	https://www.inaturalist.org/observations/32858588
2952	Bubulcus ibis	Cattle Egret	2019/7/25	2	0	22.8211143	120.2459948	https://www.inaturalist.org/observations/29609629
2953	Bubulcus ibis	Cattle Egret	2019/7/25	1	0	22.8214672	120.2526906	https://www.inaturalist.org/observations/29609539
2954	Bubulcus ibis	Cattle Egret	2019/7/25	2	0	22.8218217	120.2515672	https://www.inaturalist.org/observations/29609545
2955	Bubulcus ibis	Cattle Egret	2021/10/23	1	0	22.8293647	120.5807216	https://www.inaturalist.org/observations/134945308
2956	Bubulcus ibis	Cattle Egret	2022/8/14	1	0	22.8337406	120.1999909	https://www.inaturalist.org/observations/136122312
2957	Bubulcus ibis	Cattle Egret	2020/7/10	1	0	22.8351055	120.2152492	https://www.inaturalist.org/observations/53329344
2958	Bubulcus ibis	Cattle Egret	2022/2/24	2	0	22.8359176	120.2114582	https://www.inaturalist.org/observations/107369511
2959	Bubulcus ibis	Cattle Egret	2021/4/1	2	0	22.8379777	121.0871310	https://www.inaturalist.org/observations/73160903
2960	Bubulcus ibis	Cattle Egret	2020/7/20	2	0	22.8383464	120.2681317	https://www.inaturalist.org/observations/54497067
2961	Bubulcus ibis	Cattle Egret	2020/5/15	1	0	22.8521867	120.5440717	https://www.inaturalist.org/observations/46471971
2962	Bubulcus ibis	Cattle Egret	2021/1/8	1	0	22.8581167	120.5052000	https://www.inaturalist.org/observations/67771698
2963	Bubulcus ibis	Cattle Egret	2020/5/26	1	0	22.8605600	120.5377417	https://www.inaturalist.org/observations/47593601
2964	Bubulcus ibis	Cattle Egret	2003/8/30	2	0	22.8619063	120.4985999	https://www.inaturalist.org/observations/25565641
2965	Bubulcus ibis	Cattle Egret	2019/3/4	4	0	22.8729980	86.9291783	https://www.inaturalist.org/observations/20943797
2966	Bubulcus ibis	Cattle Egret	2020/6/18	1	0	22.8856525	120.5814579	https://www.inaturalist.org/observations/50049810
2967	Bubulcus ibis	Cattle Egret	2019/12/29	1	0	22.8860485	120.6195140	https://www.inaturalist.org/observations/49696918
2968	Bubulcus ibis	Cattle Egret	2020/4/26	2	0	22.8870521	120.6186096	https://www.inaturalist.org/observations/43921347
2969	Bubulcus ibis	Cattle Egret	2020/10/8	1	0	22.8880984	120.6200589	https://www.inaturalist.org/observations/62300729
2970	Bubulcus ibis	Cattle Egret	2021/12/2	1	0	22.8887161	120.6202318	https://www.inaturalist.org/observations/102350550
2971	Bubulcus ibis	Cattle Egret	2020/7/10	3	0	22.8916350	120.5797133	https://www.inaturalist.org/observations/52577586
2972	Bubulcus ibis	Cattle Egret	2020/4/9	1	0	22.8963606	113.8897043	https://www.inaturalist.org/observations/41749429
2973	Bubulcus ibis	Cattle Egret	2020/6/14	2	0	22.8965883	86.2061027	https://www.inaturalist.org/observations/50759051
2974	Bubulcus ibis	Cattle Egret	2020/12/17	1	0	22.9029176	120.4676079	https://www.inaturalist.org/observations/66688925

2975	Bubulcus ibis	Cattle Egret	2020/12/17	1	0	22.9034772	120.4664506	https://www.inaturalist.org/observations/66689089
2976	Bubulcus ibis	Cattle Egret	2021/12/18	2	0	22.9041573	83.9127756	https://www.inaturalist.org/observations/105911697
2977	Bubulcus ibis	Cattle Egret	2022/4/21	1	0	22.9072287	120.3116619	https://www.inaturalist.org/observations/131187066
2978	Bubulcus ibis	Cattle Egret	2020/11/24	1	0	22.9082217	120.5556281	https://www.inaturalist.org/observations/65581074
2979	Bubulcus ibis	Cattle Egret	2022/6/14	1	0	22.9089844	120.5559981	https://www.inaturalist.org/observations/121740514
2980	Bubulcus ibis	Cattle Egret	2020/10/8	1	0	22.9091084	120.5560175	https://www.inaturalist.org/observations/62300737
2981	Bubulcus ibis	Cattle Egret	2020/4/14	2	0	22.9099598	120.2494888	https://www.inaturalist.org/observations/43198653
2982	Bubulcus ibis	Cattle Egret	2020/10/27	2	0	22.9103553	87.8193577	https://www.inaturalist.org/observations/64912049
2983	Bubulcus ibis	Cattle Egret	2020/4/26	2	0	22.9104156	120.5560964	https://www.inaturalist.org/observations/43924765
2984	Bubulcus ibis	Cattle Egret	2021/6/14	1	0	22.9143258	88.3877838	https://www.inaturalist.org/observations/82927741
2985	Bubulcus ibis	Cattle Egret	2021/6/10	1	0	22.9144600	88.3879391	https://www.inaturalist.org/observations/82357959
2986	Bubulcus ibis	Cattle Egret	2020/10/17	1	0	22.9151851	120.3121874	https://www.inaturalist.org/observations/62790401
2987	Bubulcus ibis	Cattle Egret	2021/4/24	1	0	22.9164841	121.2546398	https://www.inaturalist.org/observations/75157647
2988	Bubulcus ibis	Cattle Egret	2018/4/3	1	0	22.9167225	120.3088149	https://www.inaturalist.org/observations/140770472
2989	Bubulcus ibis	Cattle Egret	2017/8/19	4	0	22.9181583	87.0708947	https://www.inaturalist.org/observations/17177130
2990	Bubulcus ibis	Cattle Egret	2020/1/28	0	0	22.9187735	120.5546179	https://www.inaturalist.org/observations/38078192
2991	Bubulcus ibis	Cattle Egret	2021/12/18	1	0	22.9195417	120.5564028	https://www.inaturalist.org/observations/103239412
2992	Bubulcus ibis	Cattle Egret	2020/8/22	1	0	22.9198862	120.3110829	https://www.inaturalist.org/observations/57541625
2993	Bubulcus ibis	Cattle Egret	2020/4/25	2	0	22.9200555	120.2630456	https://www.inaturalist.org/observations/44093660
2994	Bubulcus ibis	Cattle Egret	2020/10/30	1	0	22.9206782	120.2629421	https://www.inaturalist.org/observations/64040769
2995	Bubulcus ibis	Cattle Egret	2021/5/22	2	0	22.9215534	120.2931242	https://www.inaturalist.org/observations/81827036
2996	Bubulcus ibis	Cattle Egret	2021/10/3	1	0	22.9223503	120.2623489	https://www.inaturalist.org/observations/101074299
2997	Bubulcus ibis	Cattle Egret	2021/11/9	1	0	22.9230366	120.3462601	https://www.inaturalist.org/observations/100663950
2998	Bubulcus ibis	Cattle Egret	2018/8/31	1	0	22.9237771	120.2861550	https://www.inaturalist.org/observations/142661995
2999	Bubulcus ibis	Cattle Egret	2021/10/3	1	0	22.9246564	120.2596836	https://www.inaturalist.org/observations/101074892

3000	Bubulcus ibis	Cattle Egret	2021/8/28	1	0	22.9248922	120.3088382	https://www.inaturalist.org/observations/94556207
3001	Bubulcus ibis	Cattle Egret	2018/4/1	1	0	22.9258324	120.3054669	https://www.inaturalist.org/observations/140769441
3002	Bubulcus ibis	Cattle Egret	2018/3/30	1	0	22.9264187	120.3058180	https://www.inaturalist.org/observations/140768610
3003	Bubulcus ibis	Cattle Egret	2018/3/30	1	0	22.9264187	120.3058180	https://www.inaturalist.org/observations/140768620
3004	Bubulcus ibis	Cattle Egret	2018/9/29	3	0	22.9264855	120.2945299	https://www.inaturalist.org/observations/17027146
3005	Bubulcus ibis	Cattle Egret	2021/9/29	1	0	22.9268349	120.5765331	https://www.inaturalist.org/observations/96669909
3006	Bubulcus ibis	Cattle Egret	2021/10/3	1	0	22.9270017	120.2648061	https://www.inaturalist.org/observations/101074238
3007	Bubulcus ibis	Cattle Egret	2020/3/7	0	0	22.9275159	120.2601551	https://www.inaturalist.org/observations/39642754
3008	Bubulcus ibis	Cattle Egret	2019/5/8	2	0	22.9282064	120.5855164	https://www.inaturalist.org/observations/24888008
3009	Bubulcus ibis	Cattle Egret	2020/4/25	1	0	22.9282295	120.2619467	https://www.inaturalist.org/observations/43319190
3010	Bubulcus ibis	Cattle Egret	2018/4/3	1	0	22.9286758	120.3035673	https://www.inaturalist.org/observations/81601354
3011	Bubulcus ibis	Cattle Egret	2020/4/25	1	0	22.9307439	120.2656288	https://www.inaturalist.org/observations/43282574
3012	Bubulcus ibis	Cattle Egret	2020/4/25	1	0	22.9307923	120.2656700	https://www.inaturalist.org/observations/43282521
3013	Bubulcus ibis	Cattle Egret	2021/8/1	2	0	22.9316189	120.2619082	https://www.inaturalist.org/observations/90000660
3014	Bubulcus ibis	Cattle Egret	2020/12/31	1	0	22.9317216	120.2622303	https://www.inaturalist.org/observations/67352345
3015	Bubulcus ibis	Cattle Egret	2021/4/2	1	0	22.9317483	120.2623125	https://www.inaturalist.org/observations/101077047
3016	Bubulcus ibis	Cattle Egret	2020/3/7	0	0	22.9317694	120.2622927	https://www.inaturalist.org/observations/39642924
3017	Bubulcus ibis	Cattle Egret	2013/6/12	1	0	22.9319405	120.7349950	https://www.inaturalist.org/observations/82255625
3018	Bubulcus ibis coromandus	Cattle Egret	2022/11/27	1	0	22.9325869	120.5922250	https://www.inaturalist.org/observations/143034826
3019	Bubulcus ibis	Cattle Egret	2020/1/4	1	0	22.9336345	120.3010776	https://www.inaturalist.org/observations/37296210
3020	Bubulcus ibis	Cattle Egret	2018/11/23	4	0	22.9345750	120.2223883	https://www.inaturalist.org/observations/19150436
3021	Bubulcus ibis	Cattle Egret	2020/7/24	3	0	22.9346428	120.2249069	https://www.inaturalist.org/observations/55463212
3022	Bubulcus ibis	Cattle Egret	2018/6/12	4	0	22.9389734	120.3121138	https://www.inaturalist.org/observations/56306892
3023	Bubulcus ibis	Cattle Egret	2020/4/10	1	0	22.9447715	121.1279735	https://www.inaturalist.org/observations/42924041
3024	Bubulcus ibis	Cattle Egret	2020/7/24	1	0	22.9571032	120.6046131	https://www.inaturalist.org/observations/54517433

3025	Bubulcus ibis	Cattle Egret	2021/12/11	1	0	22.9614366	88.4588717	https://www.inaturalist.org/observations/102865118
3026	Bubulcus ibis	Cattle Egret	2018/2/15	2	0	22.9648102	86.8953406	https://www.inaturalist.org/observations/55191156
3027	Bubulcus ibis	Cattle Egret	2022/12/12	1	0	22.9702255	120.2227959	https://www.inaturalist.org/observations/144216667
3028	Bubulcus ibis	Cattle Egret	2022/12/12	1	0	22.9705920	120.2228642	https://www.inaturalist.org/observations/144215592
3029	Bubulcus ibis	Cattle Egret	2018/1/16	3	0	22.9711281	86.9893694	https://www.inaturalist.org/observations/50588558
3030	Bubulcus ibis	Cattle Egret	2022/12/12	1	0	22.9715547	120.2234193	https://www.inaturalist.org/observations/144215534
3031	Bubulcus ibis	Cattle Egret	2019/11/30	3	0	22.9719929	120.2225874	https://www.inaturalist.org/observations/36697080
3032	Bubulcus ibis	Cattle Egret	2022/12/12	2	0	22.9723335	120.2236398	https://www.inaturalist.org/observations/144215547
3033	Bubulcus ibis	Cattle Egret	2021/12/13	2	0	22.9724679	120.2230730	https://www.inaturalist.org/observations/102967090
3034	Bubulcus ibis	Cattle Egret	2021/10/31	1	0	22.9725081	120.2236244	https://www.inaturalist.org/observations/99845691
3035	Bubulcus ibis	Cattle Egret	2022/12/12	1	0	22.9725692	120.2232851	https://www.inaturalist.org/observations/144213616
3036	Bubulcus ibis	Cattle Egret	2020/6/13	1	0	22.9759885	120.2068502	https://www.inaturalist.org/observations/49548937
3037	Bubulcus ibis	Cattle Egret	2022/5/11	1	0	22.9867569	87.8549755	https://www.inaturalist.org/observations/122766226
3038	Bubulcus ibis	Cattle Egret	2022/5/11	1	0	22.9867569	87.8549755	https://www.inaturalist.org/observations/122766228
3039	Bubulcus ibis	Cattle Egret	2017/11/11	4	0	22.9869463	86.9974977	https://www.inaturalist.org/observations/13358560
3040	Bubulcus ibis	Cattle Egret	2019/7/30	6	0	22.9888698	87.0049462	https://www.inaturalist.org/observations/30618614
3041	Bubulcus ibis	Cattle Egret	2020/9/11	2	0	22.9893178	87.0031154	https://www.inaturalist.org/observations/59228190
3042	Bubulcus ibis	Cattle Egret	2020/1/3	5	0	22.9897931	87.0078094	https://www.inaturalist.org/observations/37265126
3043	Bubulcus ibis	Cattle Egret	2020/12/5	1	0	22.9902307	87.0070984	https://www.inaturalist.org/observations/66144407
3044	Bubulcus ibis	Cattle Egret	2020/9/11	3	0	22.9902857	87.0011198	https://www.inaturalist.org/observations/59228206
3045	Bubulcus ibis	Cattle Egret	2020/11/1	3	0	22.9906304	86.9976302	https://www.inaturalist.org/observations/63950859
3046	Bubulcus ibis	Cattle Egret	2020/11/23	3	0	22.9911744	87.0005846	https://www.inaturalist.org/observations/65470598
3047	Bubulcus ibis	Cattle Egret	2020/11/1	2	0	22.9912364	86.9976365	https://www.inaturalist.org/observations/63950878
3048	Bubulcus ibis	Cattle Egret	2019/6/11	2	0	22.9995830	120.2193463	https://www.inaturalist.org/observations/26866374
3049	Bubulcus ibis	Cattle Egret	2022/11/27	1	0	23.0011351	120.1547248	https://www.inaturalist.org/observations/143050894

3050	Bubulcus ibis	Cattle Egret	2022/5/10	1	0	23.0025659	120.1534229	https://www.inaturalist.org/observations/116405454
3051	Bubulcus ibis	Cattle Egret	2020/4/9	1	0	23.0025909	87.5912821	https://www.inaturalist.org/observations/41759229
3052	Bubulcus ibis	Cattle Egret	2020/9/14	1	0	23.0031649	88.4425837	https://www.inaturalist.org/observations/89541222
3053	Bubulcus ibis	Cattle Egret	2018/4/15	1	0	23.0040768	120.1486067	https://www.inaturalist.org/observations/140378371
3054	Bubulcus ibis	Cattle Egret	2019/3/16	4	0	23.0071503	120.1728981	https://www.inaturalist.org/observations/21281811
3055	Bubulcus ibis	Cattle Egret	2020/5/24	4	0	23.0157564	120.1806950	https://www.inaturalist.org/observations/47501973
3056	Bubulcus ibis	Cattle Egret	2020/5/24	2	0	23.0165364	120.1799533	https://www.inaturalist.org/observations/47502057
3057	Bubulcus ibis	Cattle Egret	2019/12/8	1	0	23.0220376	120.2024483	https://www.inaturalist.org/observations/45952447
3058	Bubulcus ibis	Cattle Egret	2021/3/12	1	0	23.0238305	120.3555300	https://www.inaturalist.org/observations/71042284
3059	Bubulcus ibis	Cattle Egret	2021/6/27	2	0	23.0248651	120.2753520	https://www.inaturalist.org/observations/85047730
3060	Bubulcus ibis	Cattle Egret	2022/10/10	2	0	23.0265436	120.3506773	https://www.inaturalist.org/observations/138228758
3061	Bubulcus ibis	Cattle Egret	2020/7/1	1	0	23.0400000	121.1738889	https://www.inaturalist.org/observations/51784752
3062	Bubulcus ibis	Cattle Egret	2021/5/3	2	0	23.0469040	120.1176992	https://www.inaturalist.org/observations/76918515
3063	Bubulcus ibis	Cattle Egret	2020/11/6	2	0	23.0524042	120.1542920	https://www.inaturalist.org/observations/64282864
3064	Bubulcus ibis	Cattle Egret	2022/3/18	3	0	23.0607738	72.5640573	https://www.inaturalist.org/observations/124892564
3065	Bubulcus ibis	Cattle Egret	2020/12/2	1	0	23.0650913	120.1965133	https://www.inaturalist.org/observations/65990956
3066	Bubulcus ibis	Cattle Egret	2020/12/2	1	0	23.0651353	120.1965026	https://www.inaturalist.org/observations/65990955
3067	Bubulcus ibis	Cattle Egret	2019/7/30	2	0	23.0675317	120.1822079	https://www.inaturalist.org/observations/29972890
3068	Bubulcus ibis	Cattle Egret	2021/4/11	1	0	23.0689677	120.0899255	https://www.inaturalist.org/observations/73825794
3069	Bubulcus ibis	Cattle Egret	2014/8/14	2	0	23.0824343	120.2602694	https://www.inaturalist.org/observations/18894763
3070	Bubulcus ibis	Cattle Egret	2019/4/10	1	0	23.0839003	120.1693100	https://www.inaturalist.org/observations/28347087
3071	Bubulcus ibis	Cattle Egret	2019/6/21	2	0	23.0865492	120.1789934	https://www.inaturalist.org/observations/50613338
3072	Bubulcus ibis	Cattle Egret	2019/10/13	1	0	23.0873265	120.3597355	https://www.inaturalist.org/observations/85317833
3073	Bubulcus ibis	Cattle Egret	2021/1/9	1	0	23.0875946	72.4433898	https://www.inaturalist.org/observations/67788428
3074	Bubulcus ibis	Cattle Egret	2019/10/11	3	0	23.0877247	120.3594081	https://www.inaturalist.org/observations/34254929

3075	Bubulcus ibis	Cattle Egret	2019/4/27	3	0	23.0884500	120.0996250	https://www.inaturalist.org/observations/23308444
3076	Bubulcus ibis	Cattle Egret	2021/5/14	1	0	23.0889225	120.1972328	https://www.inaturalist.org/observations/78993839
3077	Bubulcus ibis	Cattle Egret	2020/10/30	2	0	23.0890734	120.3586948	https://www.inaturalist.org/observations/64032841
3078	Bubulcus ibis	Cattle Egret	2016/8/28	1	0	23.0910710	120.1067000	https://www.inaturalist.org/observations/85041643
3079	Bubulcus ibis	Cattle Egret	2018/9/3	2	0	23.0911020	120.1079680	https://www.inaturalist.org/observations/17423736
3080	Bubulcus ibis	Cattle Egret	2020/9/20	1	0	23.0970249	120.3608434	https://www.inaturalist.org/observations/60162867
3081	Bubulcus ibis	Cattle Egret	2019/7/22	3	0	23.0983759	120.2710297	https://www.inaturalist.org/observations/29424128
3082	Bubulcus ibis	Cattle Egret	2022/4/20	3	0	23.0989862	120.3614117	https://www.inaturalist.org/observations/112147546
3083	Bubulcus ibis	Cattle Egret	2021/12/17	2	0	23.0999596	120.3617580	https://www.inaturalist.org/observations/103181530
3084	Bubulcus ibis	Cattle Egret	2021/8/17	1	0	23.1002733	120.1190648	https://www.inaturalist.org/observations/107246449
3085	Bubulcus ibis	Cattle Egret	2021/7/24	2	0	23.1045184	72.4822550	https://www.inaturalist.org/observations/88345663
3086	Bubulcus ibis	Cattle Egret	2020/7/14	2	0	23.1115980	120.1863317	https://www.inaturalist.org/observations/53100069
3087	Bubulcus ibis	Cattle Egret	2020/7/14	1	0	23.1137763	120.1861433	https://www.inaturalist.org/observations/53033562
3088	Bubulcus ibis	Cattle Egret	2020/7/14	1	0	23.1137763	120.1861433	https://www.inaturalist.org/observations/53099527
3089	Bubulcus ibis	Cattle Egret	2022/7/30	1	0	23.1175061	121.2202330	https://www.inaturalist.org/observations/128520195
3090	Bubulcus ibis coromandus	Cattle Egret	2014/3/16	1	0	23.1195166	120.1011836	https://www.inaturalist.org/observations/92899974
3091	Bubulcus ibis	Cattle Egret	2021/2/7	1	0	23.1224563	116.4995150	https://www.inaturalist.org/observations/69245772
3092	Bubulcus ibis	Cattle Egret	2019/6/16	4	0	23.1236377	72.0527421	https://www.inaturalist.org/observations/27203389
3093	Bubulcus ibis	Cattle Egret	2019/7/22	3	0	23.1260000	120.2768100	https://www.inaturalist.org/observations/29424125
3094	Bubulcus ibis	Cattle Egret	2021/2/7	1	0	23.1261222	116.4887331	https://www.inaturalist.org/observations/69245997
3095	Bubulcus ibis	Cattle Egret	2022/6/16	6	0	23.1319243	79.9647135	https://www.inaturalist.org/observations/121981262
3096	Bubulcus ibis	Cattle Egret	2021/12/9	3	0	23.1335733	71.7842533	https://www.inaturalist.org/observations/121873611
3097	Bubulcus ibis	Cattle Egret	2022/12/7	1	0	23.1371766	120.1412678	https://www.inaturalist.org/observations/143840802
3098	Bubulcus ibis	Cattle Egret	2019/11/10	2	0	23.1372866	72.4065810	https://www.inaturalist.org/observations/102293916
3099	Bubulcus ibis	Cattle Egret	2021/3/26	1	0	23.1425542	120.1147810	https://www.inaturalist.org/observations/72417309

3100	Bubulcus ibis	Cattle Egret	2019/7/21	6	0	23.1488389	72.3865917	https://www.inaturalist.org/observations/29263344
3101	Bubulcus ibis	Cattle Egret	2021/12/23	1	0	23.1515806	120.2607167	https://www.inaturalist.org/observations/103525103
3102	Bubulcus ibis	Cattle Egret	2020/3/18	5	0	23.1520947	86.9231261	https://www.inaturalist.org/observations/40182641
3103	Bubulcus ibis	Cattle Egret	2022/9/19	2	0	23.1544160	121.2855249	https://www.inaturalist.org/observations/140124920
3104	Bubulcus ibis	Cattle Egret	2022/9/19	2	0	23.1544160	121.2855249	https://www.inaturalist.org/observations/140126403
3105	Bubulcus ibis	Cattle Egret	2022/8/1	2	0	23.1544160	121.2855249	https://www.inaturalist.org/observations/140131280
3106	Bubulcus ibis	Cattle Egret	2022/8/8	2	0	23.1544160	121.2855249	https://www.inaturalist.org/observations/140224252
3107	Bubulcus ibis	Cattle Egret	2022/8/15	2	0	23.1544160	121.2855249	https://www.inaturalist.org/observations/140227890
3108	Bubulcus ibis	Cattle Egret	2022/8/8	1	0	23.1544160	121.2855249	https://www.inaturalist.org/observations/140232161
3109	Bubulcus ibis	Cattle Egret	2022/8/15	2	0	23.1544160	121.2855249	https://www.inaturalist.org/observations/140232751
3110	Bubulcus ibis	Cattle Egret	2022/10/24	2	0	23.1544160	121.2855249	https://www.inaturalist.org/observations/143923492
3111	Bubulcus ibis	Cattle Egret	2022/8/12	1	0	23.1544160	121.2855249	https://www.inaturalist.org/observations/143924557
3112	Bubulcus ibis	Cattle Egret	2022/8/26	1	0	23.1544160	121.2855249	https://www.inaturalist.org/observations/143924573
3113	Bubulcus ibis	Cattle Egret	2022/9/2	1	0	23.1544160	121.2855249	https://www.inaturalist.org/observations/143924585
3114	Bubulcus ibis	Cattle Egret	2022/10/17	1	0	23.1544160	121.2855249	https://www.inaturalist.org/observations/143926620
3115	Bubulcus ibis	Cattle Egret	2022/9/9	1	0	23.1544160	121.2855249	https://www.inaturalist.org/observations/143927543
3116	Bubulcus ibis	Cattle Egret	2022/11/7	1	0	23.1544160	121.2855249	https://www.inaturalist.org/observations/143929060
3117	Bubulcus ibis	Cattle Egret	2022/11/18	2	0	23.1544160	121.2855249	https://www.inaturalist.org/observations/143929704
3118	Bubulcus ibis	Cattle Egret	2022/11/18	2	0	23.1544160	121.2855249	https://www.inaturalist.org/observations/143929705
3119	Bubulcus ibis	Cattle Egret	2022/9/23	2	0	23.1544160	121.2855249	https://www.inaturalist.org/observations/143982504
3120	Bubulcus ibis	Cattle Egret	2022/9/23	3	0	23.1544160	121.2855249	https://www.inaturalist.org/observations/143982505
3121	Bubulcus ibis	Cattle Egret	2022/10/7	1	0	23.1544160	121.2855249	https://www.inaturalist.org/observations/143982519
3122	Bubulcus ibis	Cattle Egret	2022/11/18	2	0	23.1544160	121.2855249	https://www.inaturalist.org/observations/143982571
3123	Bubulcus ibis	Cattle Egret	2022/11/7	2	0	23.1544160	121.2855249	https://www.inaturalist.org/observations/143983454
3124	Bubulcus ibis	Cattle Egret	2022/10/31	0	0	23.1544160	121.2855249	https://www.inaturalist.org/observations/143989885

3125	Bubulcus ibis	Cattle Egret	2022/8/26	1	0	23.1544160	121.2855249	https://www.inaturalist.org/observations/143990401
3126	Bubulcus ibis	Cattle Egret	2022/9/12	2	0	23.1544160	121.2855249	https://www.inaturalist.org/observations/143994216
3127	Bubulcus ibis	Cattle Egret	2022/9/16	2	0	23.1544160	121.2855249	https://www.inaturalist.org/observations/143994393
3128	Bubulcus ibis	Cattle Egret	2022/10/28	1	0	23.1544160	121.2855249	https://www.inaturalist.org/observations/143994430
3129	Bubulcus ibis	Cattle Egret	2022/10/10	1	0	23.1544160	121.2855249	https://www.inaturalist.org/observations/144000495
3130	Bubulcus ibis	Cattle Egret	2022/10/10	1	0	23.1544160	121.2855249	https://www.inaturalist.org/observations/144000499
3131	Bubulcus ibis	Cattle Egret	2022/8/1	1	0	23.1544160	121.2855249	https://www.inaturalist.org/observations/144002861
3132	Bubulcus ibis	Cattle Egret	2022/11/18	1	0	23.1544160	121.2855249	https://www.inaturalist.org/observations/144004697
3133	Bubulcus ibis	Cattle Egret	2022/8/15	0	0	23.1544160	121.2855249	https://www.inaturalist.org/observations/144004823
3134	Bubulcus ibis	Cattle Egret	2022/11/14	2	0	23.1544160	121.2855249	https://www.inaturalist.org/observations/144007344
3135	Bubulcus ibis	Cattle Egret	2022/8/8	0	0	23.1544160	121.2855249	https://www.inaturalist.org/observations/144047223
3136	Bubulcus ibis	Cattle Egret	2022/8/8	1	0	23.1544160	121.2855249	https://www.inaturalist.org/observations/144059631
3137	Bubulcus ibis	Cattle Egret	2022/8/29	1	0	23.1544160	121.2855249	https://www.inaturalist.org/observations/144067256
3138	Bubulcus ibis	Cattle Egret	2022/11/7	1	0	23.1544160	121.2855249	https://www.inaturalist.org/observations/144067997
3139	Bubulcus ibis	Cattle Egret	2022/11/14	0	0	23.1544160	121.2855249	https://www.inaturalist.org/observations/144068054
3140	Bubulcus ibis	Cattle Egret	2022/8/15	1	0	23.1544160	121.2855249	https://www.inaturalist.org/observations/144068211
3141	Bubulcus ibis	Cattle Egret	2022/10/17	1	0	23.1544160	121.2855249	https://www.inaturalist.org/observations/144068952
3142	Bubulcus ibis	Cattle Egret	2022/8/22	1	0	23.1544160	121.2855249	https://www.inaturalist.org/observations/144073526
3143	Bubulcus ibis	Cattle Egret	2022/11/7	1	0	23.1544160	121.2855249	https://www.inaturalist.org/observations/144119287
3144	Bubulcus ibis	Cattle Egret	2022/11/14	2	0	23.1544160	121.2855249	https://www.inaturalist.org/observations/144222397
3145	Bubulcus ibis	Cattle Egret	2022/10/3	2	0	23.1544160	121.2855249	https://www.inaturalist.org/observations/144468978
3146	Bubulcus ibis	Cattle Egret	2020/11/6	1	0	23.1665130	120.2103283	https://www.inaturalist.org/observations/64282774
3147	Bubulcus ibis	Cattle Egret	2020/9/22	6	0	23.1673382	72.4632904	https://www.inaturalist.org/observations/61087391
3148	Bubulcus ibis	Cattle Egret	2022/9/9	2	0	23.1740641	121.2476137	https://www.inaturalist.org/observations/143992086
3149	Bubulcus ibis	Cattle Egret	2019/4/30	2	0	23.1764597	75.7885164	https://www.inaturalist.org/observations/79290806

3150	Bubulcus ibis	Cattle Egret	2019/11/14	2	0	23.1805109	120.3098550	https://www.inaturalist.org/observations/35658952
3151	Bubulcus ibis	Cattle Egret	2019/10/13	1	0	23.1830622	120.3127808	https://www.inaturalist.org/observations/34297724
3152	Bubulcus ibis	Cattle Egret	2019/11/14	2	0	23.1834949	120.3118604	https://www.inaturalist.org/observations/35658884
3153	Bubulcus ibis	Cattle Egret	2022/10/1	1	0	23.1835819	120.3117432	https://www.inaturalist.org/observations/137133334
3154	Bubulcus ibis	Cattle Egret	2019/12/22	1	0	23.1839394	120.3111321	https://www.inaturalist.org/observations/49225459
3155	Bubulcus ibis	Cattle Egret	2019/11/14	2	0	23.1844540	120.3131102	https://www.inaturalist.org/observations/35670235
3156	Bubulcus ibis	Cattle Egret	2004/6/22	5	0	23.1844640	120.3131150	https://www.inaturalist.org/observations/16280112
3157	Bubulcus ibis	Cattle Egret	2018/5/14	2	0	23.1844640	120.3131150	https://www.inaturalist.org/observations/16313020
3158	Bubulcus ibis	Cattle Egret	2019/11/19	2	0	23.1852044	120.3173588	https://www.inaturalist.org/observations/35875759
3159	Bubulcus ibis	Cattle Egret	2016/5/20	2	0	23.1855682	120.3149284	https://www.inaturalist.org/observations/25609639
3160	Bubulcus ibis	Cattle Egret	2022/7/24	2	0	23.1858126	120.3257110	https://www.inaturalist.org/observations/127840631
3161	Bubulcus ibis	Cattle Egret	2021/1/11	2	0	23.1865200	120.3269576	https://www.inaturalist.org/observations/81001499
3162	Bubulcus ibis	Cattle Egret	2021/4/3	2	0	23.1878146	120.3614342	https://www.inaturalist.org/observations/76012993
3163	Bubulcus ibis	Cattle Egret	2022/4/5	2	0	23.1898030	120.1860780	https://www.inaturalist.org/observations/110816680
3164	Bubulcus ibis	Cattle Egret	2022/4/5	2	0	23.1898030	120.1860780	https://www.inaturalist.org/observations/110816681
3165	Bubulcus ibis	Cattle Egret	2022/4/5	2	0	23.1898030	120.1860780	https://www.inaturalist.org/observations/110817077
3166	Bubulcus ibis	Cattle Egret	2019/9/3	1	0	23.1898901	120.3106319	https://www.inaturalist.org/observations/32211451
3167	Bubulcus ibis	Cattle Egret	2018/5/12	1	0	23.1944809	120.2954653	https://www.inaturalist.org/observations/81666364
3168	Bubulcus ibis	Cattle Egret	2018/12/10	6	0	23.1954449	72.6445127	https://www.inaturalist.org/observations/18934216
3169	Bubulcus ibis	Cattle Egret	2021/12/4	1	0	23.1976667	120.3701944	https://www.inaturalist.org/observations/102454688
3170	Bubulcus ibis	Cattle Egret	2018/5/12	1	0	23.1979951	120.2975531	https://www.inaturalist.org/observations/141776970
3171	Bubulcus ibis	Cattle Egret	2020/8/30	3	0	23.1990683	77.0833068	https://www.inaturalist.org/observations/58037340
3172	Bubulcus ibis	Cattle Egret	2020/8/30	3	0	23.1990683	77.0833068	https://www.inaturalist.org/observations/58037343
3173	Bubulcus ibis	Cattle Egret	2021/6/19	1	0	23.2066161	120.3595428	https://www.inaturalist.org/observations/85069041
3174	Bubulcus ibis	Cattle Egret	2021/10/21	2	0	23.2067580	77.4601620	https://www.inaturalist.org/observations/99201434

3175	Bubulcus ibis	Cattle Egret	2021/10/21	3	0	23.2067580	77.4601620	https://www.inaturalist.org/observations/99203471
3176	Bubulcus ibis	Cattle Egret	2021/5/2	1	0	23.2069536	120.3571222	https://www.inaturalist.org/observations/77417875
3177	Bubulcus ibis	Cattle Egret	2021/5/2	1	0	23.2069540	120.3571220	https://www.inaturalist.org/observations/77418060
3178	Bubulcus ibis	Cattle Egret	2021/12/5	1	0	23.2077244	120.3456719	https://www.inaturalist.org/observations/106529148
3179	Bubulcus ibis	Cattle Egret	2021/12/13	3	0	23.2094480	77.4581710	https://www.inaturalist.org/observations/102994354
3180	Bubulcus ibis	Cattle Egret	2022/1/17	3	0	23.2094480	77.4581710	https://www.inaturalist.org/observations/107292665
3181	Bubulcus ibis	Cattle Egret	2021/7/3	2	0	23.2102150	120.3584180	https://www.inaturalist.org/observations/86527573
3182	Bubulcus ibis	Cattle Egret	2021/7/3	1	0	23.2102150	120.3584180	https://www.inaturalist.org/observations/86527729
3183	Bubulcus ibis	Cattle Egret	2021/7/3	1	0	23.2102150	120.3584180	https://www.inaturalist.org/observations/86527990
3184	Bubulcus ibis	Cattle Egret	2021/7/3	2	0	23.2102150	120.3584180	https://www.inaturalist.org/observations/86528738
3185	Bubulcus ibis	Cattle Egret	2021/7/3	2	0	23.2102150	120.3584180	https://www.inaturalist.org/observations/86529184
3186	Bubulcus ibis	Cattle Egret	2021/7/3	1	0	23.2102150	120.3584180	https://www.inaturalist.org/observations/86529334
3187	Bubulcus ibis	Cattle Egret	2021/6/19	1	0	23.2102150	120.3584181	https://www.inaturalist.org/observations/85069313
3188	Bubulcus ibis	Cattle Egret	2020/4/25	1	0	23.2119375	120.0882707	https://www.inaturalist.org/observations/44593919
3189	Bubulcus ibis coromandus	Cattle Egret	2018/6/18	5	0	23.2131681	72.6541668	https://www.inaturalist.org/observations/13545815
3190	Bubulcus ibis	Cattle Egret	2022/12/10	2	0	23.2155879	77.4386844	https://www.inaturalist.org/observations/144075015
3191	Bubulcus ibis coromandus	Cattle Egret	2022/8/6	2	0	23.2209441	120.1374854	https://www.inaturalist.org/observations/130058358
3192	Bubulcus ibis	Cattle Egret	2022/8/6	1	0	23.2209737	120.1381655	https://www.inaturalist.org/observations/129806396
3193	Bubulcus ibis	Cattle Egret	2022/7/15	1	0	23.2210138	120.1395402	https://www.inaturalist.org/observations/126666385
3194	Bubulcus ibis	Cattle Egret	2020/12/15	3	0	23.2224588	87.8812170	https://www.inaturalist.org/observations/70434748
3195	Bubulcus ibis	Cattle Egret	2020/3/19	2	0	23.2290031	73.7407708	https://www.inaturalist.org/observations/40224044
3196	Bubulcus ibis	Cattle Egret	2021/12/30	1	0	23.2290090	77.3640260	https://www.inaturalist.org/observations/104105520
3197	Bubulcus ibis	Cattle Egret	2022/1/3	2	0	23.2290090	77.3640260	https://www.inaturalist.org/observations/104269993
3198	Bubulcus ibis	Cattle Egret	2019/2/14	5	0	23.2322917	69.7006967	https://www.inaturalist.org/observations/28682869
3199	Bubulcus ibis	Cattle Egret	2019/8/25	1	0	23.2330079	120.5074307	https://www.inaturalist.org/observations/35079950

3200	Bubulcus ibis	Cattle Egret	2020/6/13	2	0	23.2335341	120.1114428	https://www.inaturalist.org/observations/54735965
3201	Bubulcus ibis	Cattle Egret	2020/2/17	0	0	23.2350383	120.1262550	https://www.inaturalist.org/observations/38745905
3202	Bubulcus ibis	Cattle Egret	2022/8/29	1	0	23.2372972	120.1342833	https://www.inaturalist.org/observations/133180045
3203	Bubulcus ibis	Cattle Egret	2022/8/29	2	0	23.2374222	120.1337306	https://www.inaturalist.org/observations/133180032
3204	Bubulcus ibis	Cattle Egret	2022/8/29	1	0	23.2374222	120.1337306	https://www.inaturalist.org/observations/133180033
3205	Bubulcus ibis	Cattle Egret	2022/8/29	1	0	23.2374472	120.1338944	https://www.inaturalist.org/observations/133180042
3206	Bubulcus ibis	Cattle Egret	2022/8/24	1	0	23.2378028	120.1279917	https://www.inaturalist.org/observations/133293163
3207	Bubulcus ibis	Cattle Egret	2022/8/29	1	0	23.2388944	120.1277556	https://www.inaturalist.org/observations/133180827
3208	Bubulcus ibis	Cattle Egret	2022/8/29	1	0	23.2393222	120.1276306	https://www.inaturalist.org/observations/133180826
3209	Bubulcus ibis	Cattle Egret	2022/8/29	1	0	23.2393222	120.1276306	https://www.inaturalist.org/observations/133180871
3210	Bubulcus ibis	Cattle Egret	2021/8/17	1	0	23.2408392	120.1310749	https://www.inaturalist.org/observations/107249226
3211	Bubulcus ibis	Cattle Egret	2022/7/27	2	0	23.2437309	120.1500894	https://www.inaturalist.org/observations/129541156
3212	Bubulcus ibis	Cattle Egret	2020/6/20	2	0	23.2474867	70.6927100	https://www.inaturalist.org/observations/72992935
3213	Bubulcus ibis	Cattle Egret	2017/11/20	2	0	23.2519444	87.0636111	https://www.inaturalist.org/observations/136252932
3214	Bubulcus ibis	Cattle Egret	2022/11/26	1	0	23.2519749	87.0637436	https://www.inaturalist.org/observations/142901171
3215	Bubulcus ibis	Cattle Egret	2022/11/8	2	0	23.2522318	87.0638837	https://www.inaturalist.org/observations/141444415
3216	Bubulcus ibis	Cattle Egret	2022/12/19	2	0	23.2533797	87.0656768	https://www.inaturalist.org/observations/144789282
3217	Bubulcus ibis	Cattle Egret	2019/5/25	3	0	23.2590571	69.3973671	https://www.inaturalist.org/observations/124551725
3218	Bubulcus ibis	Cattle Egret	2022/9/29	1	0	23.2602962	71.7383389	https://www.inaturalist.org/observations/137395283
3219	Bubulcus ibis	Cattle Egret	2018/3/27	4	0	23.2665206	86.7485350	https://www.inaturalist.org/observations/31127109
3220	Bubulcus ibis	Cattle Egret	2019/4/18	3	0	23.2730997	77.2350874	https://www.inaturalist.org/observations/22649759
3221	Bubulcus ibis	Cattle Egret	2020/4/23	1	0	23.2776950	120.1699967	https://www.inaturalist.org/observations/43059972
3222	Bubulcus ibis	Cattle Egret	2020/4/23	1	0	23.2777217	120.1702333	https://www.inaturalist.org/observations/43065540
3223	Bubulcus ibis	Cattle Egret	2022/10/5	2	0	23.2788625	71.7567401	https://www.inaturalist.org/observations/138081939
3224	Bubulcus ibis	Cattle Egret	2022/4/20	2	0	23.2788908	120.1889688	https://www.inaturalist.org/observations/112134031

3225	Bubulcus ibis	Cattle Egret	2014/7/19	3	0	23.2851280	120.3188176	https://www.inaturalist.org/observations/15099321
3226	Bubulcus ibis	Cattle Egret	2022/9/30	3	0	23.2885995	71.7631131	https://www.inaturalist.org/observations/137732339
3227	Bubulcus ibis	Cattle Egret	2019/8/16	2	0	23.2915173	77.2708817	https://www.inaturalist.org/observations/30971995
3228	Bubulcus ibis	Cattle Egret	2022/10/22	1	0	23.2986250	120.2458056	https://www.inaturalist.org/observations/139582715
3229	Bubulcus ibis	Cattle Egret	2021/8/17	1	0	23.2993602	120.5855794	https://www.inaturalist.org/observations/91807402
3230	Bubulcus ibis	Cattle Egret	2018/5/26	4	0	23.3145485	86.3660284	https://www.inaturalist.org/observations/13242662
3231	Bubulcus ibis	Cattle Egret	2022/10/23	2	0	23.3172090	120.2691044	https://www.inaturalist.org/observations/139712009
3232	Bubulcus ibis	Cattle Egret	2021/12/31	1	0	23.3203430	121.3254007	https://www.inaturalist.org/observations/105137883
3233	Bubulcus ibis	Cattle Egret	2021/12/31	1	0	23.3206156	121.3259052	https://www.inaturalist.org/observations/105137880
3234	Bubulcus ibis	Cattle Egret	2022/10/18	2	0	23.3306250	120.3178660	https://www.inaturalist.org/observations/139312834
3235	Bubulcus ibis	Cattle Egret	2017/4/8	3	0	23.3306718	77.7961911	https://www.inaturalist.org/observations/72768469
3236	Bubulcus ibis	Cattle Egret	2020/8/27	2	0	23.3339942	120.4588058	https://www.inaturalist.org/observations/65384430
3237	Bubulcus ibis	Cattle Egret	2020/12/6	2	0	23.3417527	77.7796349	https://www.inaturalist.org/observations/66177351
3238	Bubulcus ibis	Cattle Egret	2021/9/28	0	0	23.3436583	120.1729722	https://www.inaturalist.org/observations/96741498
3239	Bubulcus ibis	Cattle Egret	2022/2/5	1	0	23.3457709	120.2804571	https://www.inaturalist.org/observations/107304294
3240	Bubulcus ibis	Cattle Egret	2021/9/4	2	0	23.3482410	77.7812580	https://www.inaturalist.org/observations/93734778
3241	Bubulcus ibis	Cattle Egret	2021/2/6	2	0	23.3528182	77.7815200	https://www.inaturalist.org/observations/69132593
3242	Bubulcus ibis	Cattle Egret	2018/11/1	3	0	23.3535361	120.4730033	https://www.inaturalist.org/observations/18004619
3243	Bubulcus ibis	Cattle Egret	2020/4/26	2	0	23.3593430	120.1935920	https://www.inaturalist.org/observations/44102149
3244	Bubulcus ibis	Cattle Egret	2018/7/28	3	0	23.3714733	121.3297831	https://www.inaturalist.org/observations/18418021
3245	Bubulcus ibis	Cattle Egret	2018/6/20	3	0	23.3727640	121.3240830	https://www.inaturalist.org/observations/18417963
3246	Bubulcus ibis	Cattle Egret	2022/9/11	1	0	23.3743763	72.0390454	https://www.inaturalist.org/observations/134549321
3247	Bubulcus ibis	Cattle Egret	2021/2/5	1	0	23.3759072	72.0407140	https://www.inaturalist.org/observations/69195805
3248	Bubulcus ibis	Cattle Egret	2021/1/8	3	0	23.3759670	72.0405510	https://www.inaturalist.org/observations/67736212
3249	Bubulcus ibis	Cattle Egret	2022/10/2	2	0	23.3766280	72.0397630	https://www.inaturalist.org/observations/138094787

3250	Bubulcus ibis	Cattle Egret	2022/7/24	2	0	23.3767877	72.0396400	https://www.inaturalist.org/observations/127673159
3251	Bubulcus ibis	Cattle Egret	2022/11/3	1	0	23.3797841	120.6271371	https://www.inaturalist.org/observations/140938463
3252	Bubulcus ibis	Cattle Egret	2022/11/3	1	0	23.3800346	120.6270319	https://www.inaturalist.org/observations/140940526
3253	Bubulcus ibis	Cattle Egret	2021/1/13	2	0	23.3800711	120.3369829	https://www.inaturalist.org/observations/68015117
3254	Bubulcus ibis	Cattle Egret	2019/5/25	4	0	23.3905943	71.5433121	https://www.inaturalist.org/observations/34454682
3255	Bubulcus ibis	Cattle Egret	2022/10/17	1	0	23.4040070	121.2168466	https://www.inaturalist.org/observations/143908423
3256	Bubulcus ibis	Cattle Egret	2022/10/24	0	0	23.4040070	121.2168466	https://www.inaturalist.org/observations/143919955
3257	Bubulcus ibis	Cattle Egret	2022/11/14	0	0	23.4040070	121.2168466	https://www.inaturalist.org/observations/143921089
3258	Bubulcus ibis	Cattle Egret	2022/9/26	0	0	23.4040070	121.2168466	https://www.inaturalist.org/observations/143921345
3259	Bubulcus ibis	Cattle Egret	2022/10/17	0	0	23.4040070	121.2168466	https://www.inaturalist.org/observations/143922383
3260	Bubulcus ibis	Cattle Egret	2022/9/2	1	0	23.4040070	121.2168466	https://www.inaturalist.org/observations/144073647
3261	Bubulcus ibis	Cattle Egret	2022/9/19	3	0	23.4040070	121.2168466	https://www.inaturalist.org/observations/144202340
3262	Bubulcus ibis	Cattle Egret	2022/9/19	2	0	23.4040070	121.2168466	https://www.inaturalist.org/observations/144203168
3263	Bubulcus ibis	Cattle Egret	2022/11/14	2	0	23.4040070	121.2168466	https://www.inaturalist.org/observations/144220006
3264	Bubulcus ibis	Cattle Egret	2022/7/3	1	0	23.4084389	121.3404028	https://www.inaturalist.org/observations/124551016
3265	Bubulcus ibis	Cattle Egret	2022/7/3	1	0	23.4084389	121.3404028	https://www.inaturalist.org/observations/124551098
3266	Bubulcus ibis	Cattle Egret	2022/7/3	1	0	23.4084389	121.3404028	https://www.inaturalist.org/observations/124551195
3267	Bubulcus ibis	Cattle Egret	2022/7/3	1	0	23.4084389	121.3404028	https://www.inaturalist.org/observations/124551336
3268	Bubulcus ibis	Cattle Egret	2022/12/23	0	0	23.4160301	120.1708828	https://www.inaturalist.org/observations/144912728
3269	Bubulcus ibis	Cattle Egret	2020/4/27	1	0	23.4194518	120.3002205	https://www.inaturalist.org/observations/44020860
3270	Bubulcus ibis	Cattle Egret	2022/8/23	1	0	23.4197850	120.4964183	https://www.inaturalist.org/observations/132087094
3271	Bubulcus ibis	Cattle Egret	2022/4/15	1	0	23.4234639	121.3558522	https://www.inaturalist.org/observations/120113753
3272	Bubulcus ibis	Cattle Egret	2022/4/15	1	0	23.4239411	121.3553193	https://www.inaturalist.org/observations/120113754
3273	Bubulcus ibis	Cattle Egret	2022/4/15	2	0	23.4262795	121.3563389	https://www.inaturalist.org/observations/126194495
3274	Bubulcus ibis	Cattle Egret	2022/4/15	2	0	23.4268260	121.3571357	https://www.inaturalist.org/observations/126194496

3275	Bubulcus ibis	Cattle Egret	2019/11/3	1	0	23.4272493	88.3912935	https://www.inaturalist.org/observations/95556955
3276	Bubulcus ibis	Cattle Egret	2021/4/2	1	0	23.4292730	116.3828052	https://www.inaturalist.org/observations/72676528
3277	Bubulcus ibis	Cattle Egret	2019/5/15	2	0	23.4366670	120.1633143	https://www.inaturalist.org/observations/25184627
3278	Bubulcus ibis	Cattle Egret	2020/10/27	1	0	23.4415417	120.5991383	https://www.inaturalist.org/observations/63619183
3279	Bubulcus ibis	Cattle Egret	2019/4/1	1	0	23.4468155	88.3289999	https://www.inaturalist.org/observations/100292556
3280	Bubulcus ibis	Cattle Egret	2018/12/31	5	0	23.4469000	120.3518300	https://www.inaturalist.org/observations/19328425
3281	Bubulcus ibis	Cattle Egret	2022/2/8	1	0	23.4485400	121.3987200	https://www.inaturalist.org/observations/106383561
3282	Bubulcus ibis	Cattle Egret	2022/2/10	1	0	23.4495017	121.3980117	https://www.inaturalist.org/observations/106475675
3283	Bubulcus ibis	Cattle Egret	2021/11/28	1	0	23.4510450	120.4844857	https://www.inaturalist.org/observations/102063863
3284	Bubulcus ibis	Cattle Egret	2022/10/20	1	0	23.4531884	120.5594478	https://www.inaturalist.org/observations/139850630
3285	Bubulcus ibis	Cattle Egret	2022/4/21	1	0	23.4531950	120.5596633	https://www.inaturalist.org/observations/117694067
3286	Bubulcus ibis	Cattle Egret	2014/1/2	1	0	23.4532766	120.5603104	https://www.inaturalist.org/observations/98086548
3287	Bubulcus ibis	Cattle Egret	2012/10/18	1	0	23.4532929	120.5593186	https://www.inaturalist.org/observations/118893793
3288	Bubulcus ibis	Cattle Egret	2022/7/29	0	0	23.4533178	120.5598138	https://www.inaturalist.org/observations/135634130
3289	Bubulcus ibis	Cattle Egret	2012/10/17	1	0	23.4533245	120.5593109	https://www.inaturalist.org/observations/118887242
3290	Bubulcus ibis	Cattle Egret	2020/8/18	1	0	23.4534796	120.5592370	https://www.inaturalist.org/observations/60689544
3291	Bubulcus ibis	Cattle Egret	2015/5/7	1	0	23.4534833	120.5588996	https://www.inaturalist.org/observations/97962326
3292	Bubulcus ibis	Cattle Egret	2015/1/28	1	0	23.4534833	120.5588996	https://www.inaturalist.org/observations/97962334
3293	Bubulcus ibis	Cattle Egret	2022/9/22	1	0	23.4535054	120.5579636	https://www.inaturalist.org/observations/136008977
3294	Bubulcus ibis	Cattle Egret	2020/11/9	1	0	23.4535353	120.5591051	https://www.inaturalist.org/observations/64762081
3295	Bubulcus ibis	Cattle Egret	2019/8/28	1	0	23.4537516	120.5595193	https://www.inaturalist.org/observations/42377287
3296	Bubulcus ibis	Cattle Egret	2020/4/25	1	0	23.4558511	120.1779884	https://www.inaturalist.org/observations/43583888
3297	Bubulcus ibis	Cattle Egret	2022/9/1	1	0	23.4569806	121.3811745	https://www.inaturalist.org/observations/140238884
3298	Bubulcus ibis	Cattle Egret	2021/4/17	1	0	23.4600203	120.4815421	https://www.inaturalist.org/observations/74334276
3299	Bubulcus ibis	Cattle Egret	2022/6/11	1	0	23.4604820	120.1983882	https://www.inaturalist.org/observations/121378159

3300	Bubulcus ibis	Cattle Egret	2019/6/29	3	0	23.4622002	120.4780655	https://www.inaturalist.org/observations/27878070
3301	Bubulcus ibis	Cattle Egret	2020/1/15	3	0	23.4622270	120.4780665	https://www.inaturalist.org/observations/40122532
3302	Bubulcus ibis	Cattle Egret	2019/4/29	1	0	23.4665876	120.1663997	https://www.inaturalist.org/observations/23899102
3303	Bubulcus ibis	Cattle Egret	2019/4/29	1	0	23.4695809	120.4825658	https://www.inaturalist.org/observations/23914714
3304	Bubulcus ibis	Cattle Egret	2021/4/15	1	0	23.4696010	120.1486159	https://www.inaturalist.org/observations/76561604
3305	Bubulcus ibis	Cattle Egret	2022/10/3	2	0	23.4710183	120.4856638	https://www.inaturalist.org/observations/137383877
3306	Bubulcus ibis	Cattle Egret	2018/10/4	4	0	23.4713917	120.4869300	https://www.inaturalist.org/observations/17343851
3307	Bubulcus ibis	Cattle Egret	2022/7/5	2	0	23.4716467	120.4843967	https://www.inaturalist.org/observations/124842514
3308	Bubulcus ibis	Cattle Egret	2019/11/24	2	0	23.4731747	120.4796848	https://www.inaturalist.org/observations/36011008
3309	Bubulcus ibis	Cattle Egret	2020/6/17	1	0	23.4748103	120.4508681	https://www.inaturalist.org/observations/51599042
3310	Bubulcus ibis	Cattle Egret	2020/8/6	1	0	23.4750833	120.4516139	https://www.inaturalist.org/observations/55583885
3311	Bubulcus ibis	Cattle Egret	2021/4/15	1	0	23.4757590	120.1576381	https://www.inaturalist.org/observations/76556988
3312	Bubulcus ibis	Cattle Egret	2019/2/17	5	0	23.4815564	87.3030978	https://www.inaturalist.org/observations/20418558
3313	Bubulcus ibis	Cattle Egret	2022/4/23	0	0	23.4817883	120.2536797	https://www.inaturalist.org/observations/112495756
3314	Bubulcus ibis	Cattle Egret	2022/2/10	2	0	23.4832560	77.5046100	https://www.inaturalist.org/observations/107300776
3315	Bubulcus ibis	Cattle Egret	2022/2/10	2	0	23.4832560	77.5046100	https://www.inaturalist.org/observations/107304068
3316	Bubulcus ibis	Cattle Egret	2022/5/7	2	0	23.4870870	120.1896943	https://www.inaturalist.org/observations/116942101
3317	Bubulcus ibis	Cattle Egret	2020/10/5	1	0	23.4916116	120.4466990	https://www.inaturalist.org/observations/61763131
3318	Bubulcus ibis	Cattle Egret	2020/3/1	0	0	23.4934032	120.1569634	https://www.inaturalist.org/observations/39397077
3319	Bubulcus ibis	Cattle Egret	2021/5/5	1	0	23.4936117	120.1519250	https://www.inaturalist.org/observations/79243100
3320	Bubulcus ibis	Cattle Egret	2020/1/23	0	0	23.4938033	120.1523300	https://www.inaturalist.org/observations/37907649
3321	Bubulcus ibis	Cattle Egret	2021/1/5	1	0	23.4941600	120.1538200	https://www.inaturalist.org/observations/67635215
3322	Bubulcus ibis	Cattle Egret	2021/2/2	1	0	23.4941750	120.1539250	https://www.inaturalist.org/observations/68975341
3323	Bubulcus ibis	Cattle Egret	2021/11/17	1	0	23.4947083	120.1568817	https://www.inaturalist.org/observations/101652951
3324	Bubulcus ibis	Cattle Egret	2018/1/25	1	0	23.4947918	120.1549515	https://www.inaturalist.org/observations/123738140

3325	Bubulcus ibis	Cattle Egret	2022/2/9	1	0	23.4948992	120.1566441	https://www.inaturalist.org/observations/106479791
3326	Bubulcus ibis	Cattle Egret	2020/4/27	2	0	23.4951800	120.2959100	https://www.inaturalist.org/observations/43891283
3327	Bubulcus ibis	Cattle Egret	2021/6/4	1	0	23.4959474	120.1538465	https://www.inaturalist.org/observations/84824706
3328	Bubulcus ibis	Cattle Egret	2020/4/27	1	0	23.4959989	120.1555550	https://www.inaturalist.org/observations/44097224
3329	Bubulcus ibis	Cattle Egret	2021/4/16	1	0	23.4966260	120.1683772	https://www.inaturalist.org/observations/78568842
3330	Bubulcus ibis	Cattle Egret	2020/4/25	1	0	23.4970550	120.1683433	https://www.inaturalist.org/observations/43873887
3331	Bubulcus ibis	Cattle Egret	2020/2/13	0	0	23.4981833	120.1529800	https://www.inaturalist.org/observations/38625253
3332	Bubulcus ibis	Cattle Egret	2013/7/8	5	0	23.4993110	87.3051740	http://www.inaturalist.org/observations/611724
3333	Bubulcus ibis	Cattle Egret	2020/4/10	1	0	23.5057164	120.1782418	https://www.inaturalist.org/observations/41820557
3334	Bubulcus ibis	Cattle Egret	2019/10/8	2	0	23.5075590	120.3907470	https://www.inaturalist.org/observations/35086597
3335	Bubulcus ibis	Cattle Egret	2021/11/28	1	0	23.5075700	120.1485717	https://www.inaturalist.org/observations/102081237
3336	Bubulcus ibis	Cattle Egret	2019/4/29	1	0	23.5079227	120.1619869	https://www.inaturalist.org/observations/23876340
3337	Bubulcus ibis	Cattle Egret	2022/5/9	2	0	23.5148656	120.2053900	https://www.inaturalist.org/observations/116234369
3338	Bubulcus ibis	Cattle Egret	2020/12/19	1	0	23.5159493	120.5663260	https://www.inaturalist.org/observations/68101764
3339	Bubulcus ibis	Cattle Egret	2020/1/19	0	0	23.5164183	120.1900917	https://www.inaturalist.org/observations/37772103
3340	Bubulcus ibis	Cattle Egret	2016/8/7	5	0	23.5166786	121.4158686	http://www.inaturalist.org/observations/3820336
3341	Bubulcus ibis	Cattle Egret	2021/1/24	1	0	23.5167683	120.1935883	https://www.inaturalist.org/observations/68637770
3342	Bubulcus ibis	Cattle Egret	2020/1/19	1	0	23.5200200	120.1921733	https://www.inaturalist.org/observations/37772102
3343	Bubulcus ibis	Cattle Egret	2022/1/23	1	0	23.5207099	120.2165634	https://www.inaturalist.org/observations/107233807
3344	Bubulcus ibis	Cattle Egret	2021/1/24	1	0	23.5282067	120.2325783	https://www.inaturalist.org/observations/68638505
3345	Bubulcus ibis	Cattle Egret	2020/1/19	0	0	23.5331183	120.2172650	https://www.inaturalist.org/observations/37772146
3346	Bubulcus ibis	Cattle Egret	2020/1/19	0	0	23.5352167	120.2374967	https://www.inaturalist.org/observations/37772153
3347	Bubulcus ibis	Cattle Egret	2019/10/26	1	0	23.5368684	120.1856485	https://www.inaturalist.org/observations/34913454
3348	Bubulcus ibis	Cattle Egret	2020/4/30	1	0	23.5380907	120.1690899	https://www.inaturalist.org/observations/44407090
3349	Bubulcus ibis	Cattle Egret	2019/5/10	1	0	23.5382140	120.1860694	https://www.inaturalist.org/observations/25051177

3350	Bubulcus ibis	Cattle Egret	2018/12/23	2	0	23.5395741	120.1732206	https://www.inaturalist.org/observations/19170904
3351	Bubulcus ibis	Cattle Egret	2020/11/16	1	0	23.5397666	120.7255334	https://www.inaturalist.org/observations/67551362
3352	Bubulcus ibis	Cattle Egret	2021/11/3	2	0	23.5401217	72.7179850	https://www.inaturalist.org/observations/122138156
3353	Bubulcus ibis	Cattle Egret	2022/10/22	1	0	23.5406888	120.4384720	https://www.inaturalist.org/observations/140621437
3354	Bubulcus ibis	Cattle Egret	2020/8/29	2	0	23.5409027	88.6614231	https://www.inaturalist.org/observations/58123594
3355	Bubulcus ibis	Cattle Egret	2020/11/20	4	0	23.5410133	120.6890054	https://www.inaturalist.org/observations/65256138
3356	Bubulcus ibis	Cattle Egret	2020/9/25	2	0	23.5410247	120.6890054	https://www.inaturalist.org/observations/64764288
3357	Bubulcus ibis	Cattle Egret	2020/9/25	1	0	23.5410250	120.6890060	https://www.inaturalist.org/observations/65930772
3358	Bubulcus ibis	Cattle Egret	2022/3/15	1	0	23.5431665	120.2028875	https://www.inaturalist.org/observations/108656269
3359	Bubulcus ibis	Cattle Egret	2021/4/13	1	0	23.5441302	120.2046856	https://www.inaturalist.org/observations/74032947
3360	Bubulcus ibis	Cattle Egret	2021/5/20	1	0	23.5442275	120.2044286	https://www.inaturalist.org/observations/79636940
3361	Bubulcus ibis	Cattle Egret	2020/9/28	1	0	23.5496900	120.6347683	https://www.inaturalist.org/observations/61216910
3362	Bubulcus ibis	Cattle Egret	2021/4/13	2	0	23.5504508	120.2060911	https://www.inaturalist.org/observations/74032966
3363	Bubulcus ibis	Cattle Egret	2022/3/15	1	0	23.5506903	120.2074594	https://www.inaturalist.org/observations/108656312
3364	Bubulcus ibis	Cattle Egret	2019/9/14	1	0	23.5531279	120.6328322	https://www.inaturalist.org/observations/32686106
3365	Bubulcus ibis	Cattle Egret	2018/4/1	2	0	23.5578093	119.6031578	https://www.inaturalist.org/observations/105322188
3366	Bubulcus ibis	Cattle Egret	2020/3/17	2	0	23.5583800	120.1824367	https://www.inaturalist.org/observations/40119319
3367	Bubulcus ibis	Cattle Egret	2017/4/30	2	0	23.5588997	119.6111205	https://www.inaturalist.org/observations/105322178
3368	Bubulcus ibis	Cattle Egret	2021/5/12	1	0	23.5611737	120.1643022	https://www.inaturalist.org/observations/78672190
3369	Bubulcus ibis	Cattle Egret	2022/4/24	1	0	23.5630492	120.1632065	https://www.inaturalist.org/observations/112809823
3370	Bubulcus ibis	Cattle Egret	2021/2/4	1	0	23.5663983	120.1974867	https://www.inaturalist.org/observations/69370019
3371	Bubulcus ibis	Cattle Egret	2016/12/20	4	0	23.5666908	87.3034771	https://www.inaturalist.org/observations/5936338
3372	Bubulcus ibis	Cattle Egret	2021/1/10	1	0	23.5669276	120.1636277	https://www.inaturalist.org/observations/67887549
3373	Bubulcus ibis	Cattle Egret	2020/1/15	0	0	23.5674650	120.2430800	https://www.inaturalist.org/observations/37772497
3374	Bubulcus ibis	Cattle Egret	2020/4/25	1	0	23.5675853	120.1658789	https://www.inaturalist.org/observations/44296069

3375	Bubulcus ibis	Cattle Egret	2022/4/30	1	0	23.5695172	120.1649084	https://www.inaturalist.org/observations/114454821
3376	Bubulcus ibis	Cattle Egret	2020/4/29	1	0	23.5696133	120.1649483	https://www.inaturalist.org/observations/44292645
3377	Bubulcus ibis	Cattle Egret	2022/2/22	1	0	23.5699367	120.2186879	https://www.inaturalist.org/observations/107300329
3378	Bubulcus ibis	Cattle Egret	2021/12/8	1	0	23.5699517	120.2193350	https://www.inaturalist.org/observations/103066815
3379	Bubulcus ibis	Cattle Egret	2022/3/25	1	0	23.5699540	120.1635690	https://www.inaturalist.org/observations/125135632
3380	Bubulcus ibis	Cattle Egret	2022/11/21	2	0	23.5701917	120.4926000	https://www.inaturalist.org/observations/142517943
3381	Bubulcus ibis	Cattle Egret	2021/4/20	1	0	23.5710349	120.1717198	https://www.inaturalist.org/observations/74716801
3382	Bubulcus ibis	Cattle Egret	2022/5/9	1	0	23.5721674	120.1866538	https://www.inaturalist.org/observations/116245606
3383	Bubulcus ibis	Cattle Egret	2022/5/9	1	0	23.5722260	120.1866549	https://www.inaturalist.org/observations/116245584
3384	Bubulcus ibis	Cattle Egret	2020/3/9	2	0	23.5732767	120.1687517	https://www.inaturalist.org/observations/39832543
3385	Bubulcus ibis	Cattle Egret	2022/4/24	2	0	23.5736166	120.1656834	https://www.inaturalist.org/observations/112813037
3386	Bubulcus ibis	Cattle Egret	2022/4/19	2	0	23.5749986	120.1593501	https://www.inaturalist.org/observations/112033793
3387	Bubulcus ibis	Cattle Egret	2020/4/28	1	0	23.5755317	120.1982000	https://www.inaturalist.org/observations/44290990
3388	Bubulcus ibis	Cattle Egret	2018/2/24	2	0	23.5768330	119.5851031	https://www.inaturalist.org/observations/105322187
3389	Bubulcus ibis	Cattle Egret	2020/4/17	1	0	23.5776300	120.2058750	https://www.inaturalist.org/observations/42737934
3390	Bubulcus ibis	Cattle Egret	2022/2/22	1	0	23.5788830	120.2200262	https://www.inaturalist.org/observations/107299938
3391	Bubulcus ibis	Cattle Egret	2022/5/11	1	0	23.5790778	120.2163556	https://www.inaturalist.org/observations/116530655
3392	Bubulcus ibis	Cattle Egret	2020/4/17	2	0	23.5791383	120.2151967	https://www.inaturalist.org/observations/42738486
3393	Bubulcus ibis	Cattle Egret	2017/11/15	2	0	23.5795220	119.5837630	https://www.inaturalist.org/observations/105322186
3394	Bubulcus ibis	Cattle Egret	2022/3/3	1	0	23.5811905	120.1645183	https://www.inaturalist.org/observations/108089853
3395	Bubulcus ibis	Cattle Egret	2020/9/25	2	0	23.5820381	120.5587701	https://www.inaturalist.org/observations/65256510
3396	Bubulcus ibis	Cattle Egret	2022/3/2	1	0	23.5848748	120.2394496	https://www.inaturalist.org/observations/107984118
3397	Bubulcus ibis	Cattle Egret	2022/11/4	2	0	23.5851901	121.5027404	https://www.inaturalist.org/observations/143863513
3398	Bubulcus ibis	Cattle Egret	2022/11/4	2	0	23.5851901	121.5027404	https://www.inaturalist.org/observations/143863514
3399	Bubulcus ibis	Cattle Egret	2022/11/11	2	0	23.5851901	121.5027404	https://www.inaturalist.org/observations/143863516

3400	Bubulcus ibis	Cattle Egret	2022/9/2	2	0	23.5851901	121.5027404	https://www.inaturalist.org/observations/143912644
3401	Bubulcus ibis	Cattle Egret	2022/9/30	1	0	23.5851901	121.5027404	https://www.inaturalist.org/observations/143914795
3402	Bubulcus ibis	Cattle Egret	2022/10/21	2	0	23.5851901	121.5027404	https://www.inaturalist.org/observations/143914819
3403	Bubulcus ibis	Cattle Egret	2022/10/21	1	0	23.5851901	121.5027404	https://www.inaturalist.org/observations/143914824
3404	Bubulcus ibis	Cattle Egret	2022/10/21	1	0	23.5851901	121.5027404	https://www.inaturalist.org/observations/143914829
3405	Bubulcus ibis	Cattle Egret	2022/10/28	1	0	23.5851901	121.5027404	https://www.inaturalist.org/observations/143914849
3406	Bubulcus ibis	Cattle Egret	2022/11/4	1	0	23.5851901	121.5027404	https://www.inaturalist.org/observations/143914859
3407	Bubulcus ibis	Cattle Egret	2022/9/10	1	0	23.5856426	120.5147755	https://www.inaturalist.org/observations/134369804
3408	Bubulcus ibis	Cattle Egret	2018/12/6	2	0	23.5874655	120.2511646	https://www.inaturalist.org/observations/18852157
3409	Bubulcus ibis	Cattle Egret	2020/2/13	0	0	23.5905367	120.2438567	https://www.inaturalist.org/observations/38625362
3410	Bubulcus ibis	Cattle Egret	2022/5/1	1	0	23.5910484	120.5984203	https://www.inaturalist.org/observations/114225440
3411	Bubulcus ibis	Cattle Egret	2018/6/24	3	0	23.5931930	120.3019990	https://www.inaturalist.org/observations/13760608
3412	Bubulcus ibis	Cattle Egret	2020/12/9	1	0	23.5944411	120.2372257	https://www.inaturalist.org/observations/66634589
3413	Bubulcus ibis	Cattle Egret	2021/3/14	1	0	23.5975580	120.3359310	https://www.inaturalist.org/observations/71220442
3414	Bubulcus ibis	Cattle Egret	2021/12/8	1	0	23.5985183	120.2360550	https://www.inaturalist.org/observations/103066823
3415	Bubulcus ibis	Cattle Egret	2019/1/27	2	0	23.5991394	120.1728520	https://www.inaturalist.org/observations/19903799
3416	Bubulcus ibis	Cattle Egret	2019/1/28	3	0	23.5993719	120.2424807	https://www.inaturalist.org/observations/19927432
3417	Bubulcus ibis	Cattle Egret	2021/3/11	2	0	23.5994451	120.2799848	https://www.inaturalist.org/observations/71041137
3418	Bubulcus ibis	Cattle Egret	2020/1/21	0	0	23.6000446	120.3362094	https://www.inaturalist.org/observations/37843218
3419	Bubulcus ibis	Cattle Egret	2022/1/17	2	0	23.6042102	120.2311797	https://www.inaturalist.org/observations/108111790
3420	Bubulcus ibis	Cattle Egret	2022/2/5	1	0	23.6051028	120.4018417	https://www.inaturalist.org/observations/106165093
3421	Bubulcus ibis	Cattle Egret	2021/12/2	1	0	23.6057591	120.2413642	https://www.inaturalist.org/observations/102627988
3422	Bubulcus ibis	Cattle Egret	2021/12/8	1	0	23.6059950	120.2505533	https://www.inaturalist.org/observations/103113576
3423	Bubulcus ibis	Cattle Egret	2020/9/19	1	0	23.6062354	120.2378823	https://www.inaturalist.org/observations/60040558
3424	Bubulcus ibis	Cattle Egret	2021/4/30	1	0	23.6074694	120.2874056	https://www.inaturalist.org/observations/75769446

3425	Bubulcus ibis	Cattle Egret	2019/4/28	1	0	23.6099088	120.4609830	https://www.inaturalist.org/observations/23584232
3426	Bubulcus ibis	Cattle Egret	2020/3/6	1	0	23.6105790	120.2633421	https://www.inaturalist.org/observations/39604432
3427	Bubulcus ibis	Cattle Egret	2020/4/27	4	0	23.6107786	120.4586018	https://www.inaturalist.org/observations/44038304
3428	Bubulcus ibis	Cattle Egret	2019/10/9	1	0	23.6108983	120.2441133	https://www.inaturalist.org/observations/34105929
3429	Bubulcus ibis coromandus	Cattle Egret	2020/12/23	1	0	23.6109600	120.2609083	https://www.inaturalist.org/observations/67028359
3430	Bubulcus ibis	Cattle Egret	2021/11/24	2	0	23.6120033	120.2530883	https://www.inaturalist.org/observations/102239254
3431	Bubulcus ibis	Cattle Egret	2021/2/1	1	0	23.6128503	120.3397260	https://www.inaturalist.org/observations/68916421
3432	Bubulcus ibis	Cattle Egret	2022/1/7	2	0	23.6130845	120.3403689	https://www.inaturalist.org/observations/104506601
3433	Bubulcus ibis	Cattle Egret	2019/5/9	1	0	23.6132622	120.1459199	https://www.inaturalist.org/observations/24831151
3434	Bubulcus ibis	Cattle Egret	2020/2/3	0	0	23.6133050	120.1807483	https://www.inaturalist.org/observations/38282496
3435	Bubulcus ibis	Cattle Egret	2022/9/12	1	0	23.6141872	120.6449510	https://www.inaturalist.org/observations/134711118
3436	Bubulcus ibis	Cattle Egret	2020/4/14	1	0	23.6142250	120.5223383	https://www.inaturalist.org/observations/42146326
3437	Bubulcus ibis	Cattle Egret	2021/9/5	1	0	23.6164063	120.3766558	https://www.inaturalist.org/observations/93645878
3438	Bubulcus ibis	Cattle Egret	2018/12/11	2	0	23.6166304	120.2626554	https://www.inaturalist.org/observations/18958347
3439	Bubulcus ibis	Cattle Egret	2021/1/21	1	0	23.6166800	120.2675317	https://www.inaturalist.org/observations/68403695
3440	Bubulcus ibis	Cattle Egret	2020/3/5	1	0	23.6173300	120.2615850	https://www.inaturalist.org/observations/39604427
3441	Bubulcus ibis	Cattle Egret	2019/1/8	3	0	23.6181694	120.2282391	https://www.inaturalist.org/observations/19513237
3442	Bubulcus ibis	Cattle Egret	2020/3/9	3	0	23.6187300	120.2626817	https://www.inaturalist.org/observations/39832545
3443	Bubulcus ibis	Cattle Egret	2021/9/10	1	0	23.6191801	120.6514376	https://www.inaturalist.org/observations/96883105
3444	Bubulcus ibis	Cattle Egret	2021/3/24	1	0	23.6195177	120.2605910	https://www.inaturalist.org/observations/71934986
3445	Bubulcus ibis	Cattle Egret	2022/12/23	1	0	23.6197636	120.2565476	https://www.inaturalist.org/observations/144924348
3446	Bubulcus ibis	Cattle Egret	2022/6/2	1	0	23.6197761	120.2615389	https://www.inaturalist.org/observations/120969589
3447	Bubulcus ibis	Cattle Egret	2022/1/17	1	0	23.6199111	120.2610574	https://www.inaturalist.org/observations/108110610
3448	Bubulcus ibis	Cattle Egret	2022/5/2	2	0	23.6199800	120.2614133	https://www.inaturalist.org/observations/114495974
3449	Bubulcus ibis	Cattle Egret	2021/5/4	2	0	23.6201483	120.2624583	https://www.inaturalist.org/observations/78402326

3450	Bubulcus ibis	Cattle Egret	2021/4/26	1	0	23.6201678	120.2615948	https://www.inaturalist.org/observations/75267292
3451	Bubulcus ibis	Cattle Egret	2021/4/30	1	0	23.6203075	120.2615124	https://www.inaturalist.org/observations/75770509
3452	Bubulcus ibis	Cattle Egret	2020/5/15	2	0	23.6203193	120.2615912	https://www.inaturalist.org/observations/45944184
3453	Bubulcus ibis	Cattle Egret	2020/4/8	1	0	23.6209600	120.2522817	https://www.inaturalist.org/observations/41673626
3454	Bubulcus ibis	Cattle Egret	2018/12/23	2	0	23.6210883	120.2549950	https://www.inaturalist.org/observations/19171000
3455	Bubulcus ibis	Cattle Egret	2020/1/15	0	0	23.6215233	120.2363567	https://www.inaturalist.org/observations/37772515
3456	Bubulcus ibis	Cattle Egret	2022/1/14	2	0	23.6215600	120.2258220	https://www.inaturalist.org/observations/108104672
3457	Bubulcus ibis	Cattle Egret	2022/3/2	1	0	23.6218362	120.2414849	https://www.inaturalist.org/observations/107981024
3458	Bubulcus ibis	Cattle Egret	2018/12/19	2	0	23.6231378	120.2601019	https://www.inaturalist.org/observations/19152488
3459	Bubulcus ibis	Cattle Egret	2019/1/16	2	0	23.6232610	120.2405549	https://www.inaturalist.org/observations/19709366
3460	Bubulcus ibis	Cattle Egret	2018/1/28	2	0	23.6239200	120.2287470	https://www.inaturalist.org/observations/14414946
3461	Bubulcus ibis	Cattle Egret	2020/1/13	1	0	23.6240959	119.5974265	https://www.inaturalist.org/observations/37558636
3462	Bubulcus ibis	Cattle Egret	2018/1/28	2	0	23.6247730	120.1991060	https://www.inaturalist.org/observations/14414943
3463	Bubulcus ibis	Cattle Egret	2021/11/26	1	0	23.6247833	120.3729900	https://www.inaturalist.org/observations/102236214
3464	Bubulcus ibis	Cattle Egret	2020/4/17	2	0	23.6253554	120.5245152	https://www.inaturalist.org/observations/42383004
3465	Bubulcus ibis	Cattle Egret	2009/8/31	2	0	23.6253596	120.2678766	https://www.inaturalist.org/observations/18692974
3466	Bubulcus ibis	Cattle Egret	2019/12/25	1	0	23.6266305	120.2346750	https://www.inaturalist.org/observations/36987670
3467	Bubulcus ibis	Cattle Egret	2020/8/19	1	0	23.6268944	120.3094528	https://www.inaturalist.org/observations/56909342
3468	Bubulcus ibis	Cattle Egret	2022/6/10	1	0	23.6271181	120.2505345	https://www.inaturalist.org/observations/121064315
3469	Bubulcus ibis	Cattle Egret	2022/7/19	2	0	23.6285750	120.2941933	https://www.inaturalist.org/observations/127233247
3470	Bubulcus ibis	Cattle Egret	2018/11/19	3	0	23.6288771	120.3320195	https://www.inaturalist.org/observations/18494053
3471	Bubulcus ibis	Cattle Egret	2021/9/29	0	0	23.6303700	120.4050917	https://www.inaturalist.org/observations/96633133
3472	Bubulcus ibis	Cattle Egret	2022/7/19	0	0	23.6304017	120.2918083	https://www.inaturalist.org/observations/127233194
3473	Bubulcus ibis	Cattle Egret	2020/5/14	2	0	23.6324901	120.3003080	https://www.inaturalist.org/observations/45944175
3474	Bubulcus ibis	Cattle Egret	2022/3/26	1	0	23.6327531	120.2888914	https://www.inaturalist.org/observations/109518121

3475	Bubulcus ibis	Cattle Egret	2020/9/26	3	0	23.6328033	120.2645600	https://www.inaturalist.org/observations/60779968
3476	Bubulcus ibis	Cattle Egret	2021/11/12	1	0	23.6336861	120.4840861	https://www.inaturalist.org/observations/100915546
3477	Bubulcus ibis	Cattle Egret	2021/1/27	1	0	23.6339583	120.1584150	https://www.inaturalist.org/observations/85719379
3478	Bubulcus ibis	Cattle Egret	2020/6/23	1	0	23.6342433	120.4007667	https://www.inaturalist.org/observations/50712787
3479	Bubulcus ibis	Cattle Egret	2021/5/28	1	0	23.6343013	120.5047960	https://www.inaturalist.org/observations/80803799
3480	Bubulcus ibis	Cattle Egret	2022/3/2	1	0	23.6345096	120.2397392	https://www.inaturalist.org/observations/107981017
3481	Bubulcus ibis	Cattle Egret	2020/3/7	0	0	23.6355207	120.4921832	https://www.inaturalist.org/observations/39646969
3482	Bubulcus ibis	Cattle Egret	2020/4/17	3	0	23.6359839	120.5237441	https://www.inaturalist.org/observations/42380800
3483	Bubulcus ibis	Cattle Egret	2020/8/22	1	0	23.6367447	120.5420475	https://www.inaturalist.org/observations/57220159
3484	Bubulcus ibis	Cattle Egret	2022/5/23	1	0	23.6367721	120.5236780	https://www.inaturalist.org/observations/118342263
3485	Bubulcus ibis	Cattle Egret	2022/2/24	2	0	23.6369394	120.3091315	https://www.inaturalist.org/observations/107391697
3486	Bubulcus ibis	Cattle Egret	2022/5/23	1	0	23.6370043	120.5229091	https://www.inaturalist.org/observations/118345777
3487	Bubulcus ibis	Cattle Egret	2022/9/2	1	0	23.6370233	120.4827949	https://www.inaturalist.org/observations/133301068
3488	Bubulcus ibis	Cattle Egret	2022/6/4	1	0	23.6371047	120.4934540	https://www.inaturalist.org/observations/120127776
3489	Bubulcus ibis	Cattle Egret	2021/2/2	1	0	23.6389327	120.3078328	https://www.inaturalist.org/observations/68963564
3490	Bubulcus ibis	Cattle Egret	2021/10/5	1	0	23.6392116	120.4091549	https://www.inaturalist.org/observations/97217529
3491	Bubulcus ibis	Cattle Egret	2021/8/16	1	0	23.6393806	120.4938000	https://www.inaturalist.org/observations/91378244
3492	Bubulcus ibis	Cattle Egret	2021/6/5	4	0	23.6394462	120.5184266	https://www.inaturalist.org/observations/81652773
3493	Bubulcus ibis	Cattle Egret	2019/10/22	2	0	23.6395078	120.5336711	https://www.inaturalist.org/observations/45460485
3494	Bubulcus ibis	Cattle Egret	2020/2/12	0	0	23.6400076	120.1588300	https://www.inaturalist.org/observations/38625833
3495	Bubulcus ibis	Cattle Egret	1980/1/1	1	0	23.6409235	120.5604128	https://www.inaturalist.org/observations/64029172
3496	Bubulcus ibis	Cattle Egret	2020/3/31	2	0	23.6422150	120.2986883	https://www.inaturalist.org/observations/41209366
3497	Bubulcus ibis	Cattle Egret	2020/2/12	0	0	23.6427006	120.1691419	https://www.inaturalist.org/observations/38625850
3498	Bubulcus ibis	Cattle Egret	2022/11/6	1	0	23.6449083	120.6585250	https://www.inaturalist.org/observations/141255033
3499	Bubulcus ibis	Cattle Egret	2022/7/5	1	0	23.6451501	120.4836566	https://www.inaturalist.org/observations/124840803

3500	Bubulcus ibis	Cattle Egret	2021/8/24	2	0	23.6451694	120.4887444	https://www.inaturalist.org/observations/92315812
3501	Bubulcus ibis	Cattle Egret	2017/1/23	2	0	23.6459240	120.3195770	https://www.inaturalist.org/observations/47487506
3502	Bubulcus ibis	Cattle Egret	1980/1/1	1	0	23.6459716	120.5585388	https://www.inaturalist.org/observations/64029220
3503	Bubulcus ibis	Cattle Egret	2021/12/6	1	0	23.6468750	120.2905200	https://www.inaturalist.org/observations/102659093
3504	Bubulcus ibis	Cattle Egret	2020/4/22	1	0	23.6480186	120.3496820	https://www.inaturalist.org/observations/42821109
3505	Bubulcus ibis	Cattle Egret	2022/10/28	1	0	23.6483274	120.3249148	https://www.inaturalist.org/observations/140225952
3506	Bubulcus ibis	Cattle Egret	2022/10/28	1	0	23.6483344	120.3249846	https://www.inaturalist.org/observations/140226013
3507	Bubulcus ibis	Cattle Egret	2020/10/14	1	0	23.6486528	120.2696694	https://www.inaturalist.org/observations/62549741
3508	Bubulcus ibis	Cattle Egret	2020/2/20	0	0	23.6489233	120.2796233	https://www.inaturalist.org/observations/38948899
3509	Bubulcus ibis	Cattle Egret	1980/1/1	1	0	23.6492478	120.5600469	https://www.inaturalist.org/observations/64029238
3510	Bubulcus ibis	Cattle Egret	2022/7/15	1	0	23.6508882	120.2596599	https://www.inaturalist.org/observations/126308971
3511	Bubulcus ibis	Cattle Egret	2020/5/7	1	0	23.6514418	120.2863081	https://www.inaturalist.org/observations/45246383
3512	Bubulcus ibis	Cattle Egret	2020/2/18	0	0	23.6527333	120.2262317	https://www.inaturalist.org/observations/38816321
3513	Bubulcus ibis	Cattle Egret	2021/12/9	1	0	23.6543942	120.2657867	https://www.inaturalist.org/observations/103113583
3514	Bubulcus ibis	Cattle Egret	2021/12/21	1	0	23.6545026	120.3025412	https://www.inaturalist.org/observations/103408555
3515	Bubulcus ibis	Cattle Egret	2020/4/17	1	0	23.6547983	120.3065067	https://www.inaturalist.org/observations/42736418
3516	Bubulcus ibis	Cattle Egret	2021/7/25	2	0	23.6550342	120.5082708	https://www.inaturalist.org/observations/88507040
3517	Bubulcus ibis	Cattle Egret	2021/11/2	1	0	23.6554173	120.2857043	https://www.inaturalist.org/observations/100026878
3518	Bubulcus ibis	Cattle Egret	2021/1/13	1	0	23.6557867	120.3019467	https://www.inaturalist.org/observations/68267287
3519	Bubulcus ibis	Cattle Egret	2022/1/23	1	0	23.6584314	120.3048063	https://www.inaturalist.org/observations/106494559
3520	Bubulcus ibis	Cattle Egret	2021/4/22	2	0	23.6594941	120.2312797	https://www.inaturalist.org/observations/74814733
3521	Bubulcus ibis	Cattle Egret	2020/1/14	0	0	23.6604500	120.3168300	https://www.inaturalist.org/observations/37772724
3522	Bubulcus ibis	Cattle Egret	2022/9/11	1	0	23.6605944	120.6553034	https://www.inaturalist.org/observations/138666988
3523	Bubulcus ibis	Cattle Egret	2021/1/28	3	0	23.6606015	120.3641031	https://www.inaturalist.org/observations/68742464
3524	Bubulcus ibis	Cattle Egret	2021/1/8	2	0	23.6608461	120.3132078	https://www.inaturalist.org/observations/67834279

3525	Bubulcus ibis	Cattle Egret	2020/11/25	1	0	23.6615833	120.3056183	https://www.inaturalist.org/observations/65565856
3526	Bubulcus ibis	Cattle Egret	2022/12/4	1	0	23.6626574	120.2756140	https://www.inaturalist.org/observations/144396808
3527	Bubulcus ibis	Cattle Egret	2021/10/19	1	0	23.6626687	120.5402802	https://www.inaturalist.org/observations/98679972
3528	Bubulcus ibis	Cattle Egret	2021/10/19	1	0	23.6627925	120.5399709	https://www.inaturalist.org/observations/98679251
3529	Bubulcus ibis	Cattle Egret	2021/10/19	1	0	23.6633125	120.5392455	https://www.inaturalist.org/observations/98679769
3530	Bubulcus ibis	Cattle Egret	2018/4/24	2	0	23.6635503	120.2837712	https://www.inaturalist.org/observations/22993082
3531	Bubulcus ibis	Cattle Egret	2021/12/6	1	0	23.6650824	120.2842236	https://www.inaturalist.org/observations/102659409
3532	Bubulcus ibis	Cattle Egret	2022/1/13	1	0	23.6679676	120.2336403	https://www.inaturalist.org/observations/108104409
3533	Bubulcus ibis	Cattle Egret	2022/1/17	3	0	23.6704089	120.2346525	https://www.inaturalist.org/observations/108110098
3534	Bubulcus ibis	Cattle Egret	2021/2/18	1	0	23.6763035	120.2776564	https://www.inaturalist.org/observations/69757667
3535	Bubulcus ibis	Cattle Egret	2020/5/1	1	0	23.6767750	120.2981583	https://www.inaturalist.org/observations/45245437
3536	Bubulcus ibis	Cattle Egret	2021/11/24	1	0	23.6775483	120.3160883	https://www.inaturalist.org/observations/102239240
3537	Bubulcus ibis	Cattle Egret	2020/8/17	1	0	23.6780306	120.2331778	https://www.inaturalist.org/observations/56704900
3538	Bubulcus ibis	Cattle Egret	2019/11/1	4	0	23.6784294	120.5553965	https://www.inaturalist.org/observations/35161628
3539	Bubulcus ibis	Cattle Egret	2019/11/6	2	0	23.6788096	120.5550207	https://www.inaturalist.org/observations/35668304
3540	Bubulcus ibis	Cattle Egret	2019/10/23	3	0	23.6788808	120.5560419	https://www.inaturalist.org/observations/35672676
3541	Bubulcus ibis	Cattle Egret	2019/11/8	2	0	23.6788872	120.5550643	https://www.inaturalist.org/observations/35668130
3542	Bubulcus ibis	Cattle Egret	2019/11/4	2	0	23.6791191	120.5559038	https://www.inaturalist.org/observations/35353646
3543	Bubulcus ibis	Cattle Egret	2018/11/6	4	0	23.6792967	120.5569083	https://www.inaturalist.org/observations/18162057
3544	Bubulcus ibis	Cattle Egret	2019/12/26	2	0	23.6793669	120.5563641	https://www.inaturalist.org/observations/37417349
3545	Bubulcus ibis	Cattle Egret	2021/8/19	2	0	23.6794181	120.1768681	https://www.inaturalist.org/observations/91729200
3546	Bubulcus ibis	Cattle Egret	2021/5/27	1	0	23.6799081	120.1654188	https://www.inaturalist.org/observations/80456118
3547	Bubulcus ibis	Cattle Egret	2020/10/23	2	0	23.6803300	120.5595700	https://www.inaturalist.org/observations/65254508
3548	Bubulcus ibis	Cattle Egret	2020/3/3	1	0	23.6812500	120.4279717	https://www.inaturalist.org/observations/39485274
3549	Bubulcus ibis	Cattle Egret	2022/1/13	1	0	23.6821370	120.4335300	https://www.inaturalist.org/observations/108103428

3550	Bubulcus ibis	Cattle Egret	2019/12/12	1	0	23.6826958	120.4758519	https://www.inaturalist.org/observations/36587562
3551	Bubulcus ibis	Cattle Egret	2021/5/13	1	0	23.6845135	116.3061076	https://www.inaturalist.org/observations/78545290
3552	Bubulcus ibis	Cattle Egret	2015/2/26	3	0	23.6855503	81.0258168	https://www.inaturalist.org/observations/69187560
3553	Bubulcus ibis	Cattle Egret	2021/11/12	1	0	23.6857798	120.6207728	https://www.inaturalist.org/observations/102745371
3554	Bubulcus ibis	Cattle Egret	2022/5/17	1	0	23.6884790	120.3105207	https://www.inaturalist.org/observations/117417662
3555	Bubulcus ibis coromandus	Cattle Egret	2019/2/23	4	0	23.6895245	120.5210019	https://www.inaturalist.org/observations/20780019
3556	Bubulcus ibis	Cattle Egret	2019/2/23	4	0	23.6898856	120.5207779	https://www.inaturalist.org/observations/20780008
3557	Bubulcus ibis	Cattle Egret	2020/4/20	1	0	23.6903950	120.2397917	https://www.inaturalist.org/observations/42670417
3558	Bubulcus ibis	Cattle Egret	2020/2/12	1	0	23.6919059	120.3145398	https://www.inaturalist.org/observations/38559602
3559	Bubulcus ibis	Cattle Egret	2022/3/9	1	0	23.6940398	120.4514207	https://www.inaturalist.org/observations/108334598
3560	Bubulcus ibis	Cattle Egret	2021/12/22	1	0	23.6950821	120.2045653	https://www.inaturalist.org/observations/103460730
3561	Bubulcus ibis	Cattle Egret	2021/2/4	1	0	23.6959517	120.3214467	https://www.inaturalist.org/observations/69369982
3562	Bubulcus ibis	Cattle Egret	2020/10/20	1	0	23.6960144	120.5925293	https://www.inaturalist.org/observations/63076975
3563	Bubulcus ibis	Cattle Egret	2018/10/7	4	0	23.6961917	120.5093917	https://www.inaturalist.org/observations/17272675
3564	Bubulcus ibis	Cattle Egret	2018/10/7	5	0	23.6961917	120.5093917	https://www.inaturalist.org/observations/17272682
3565	Bubulcus ibis	Cattle Egret	2022/4/29	1	0	23.6970045	120.3874655	https://www.inaturalist.org/observations/113343678
3566	Bubulcus ibis	Cattle Egret	2019/9/14	1	0	23.6978100	120.9605150	https://www.inaturalist.org/observations/48335871
3567	Bubulcus ibis coromandus	Cattle Egret	2017/2/16	2	0	23.6978100	120.9605150	https://www.inaturalist.org/observations/129287049
3568	Bubulcus ibis	Cattle Egret	2020/4/2	1	0	23.6984325	120.3592839	https://www.inaturalist.org/observations/41213427
3569	Bubulcus ibis	Cattle Egret	2020/4/19	1	0	23.7027250	120.4633967	https://www.inaturalist.org/observations/44231116
3570	Bubulcus ibis	Cattle Egret	2020/3/4	0	0	23.7029869	120.4164975	https://www.inaturalist.org/observations/39523561
3571	Bubulcus ibis	Cattle Egret	2020/10/20	1	0	23.7031846	120.5946382	https://www.inaturalist.org/observations/63076967
3572	Bubulcus ibis	Cattle Egret	2020/5/5	3	0	23.7038541	120.4635798	https://www.inaturalist.org/observations/44964188
3573	Bubulcus ibis	Cattle Egret	2020/3/24	2	0	23.7039667	120.3356000	https://www.inaturalist.org/observations/40683679
3574	Bubulcus ibis	Cattle Egret	2022/5/28	1	0	23.7040131	120.4623814	https://www.inaturalist.org/observations/119039215

3575	Bubulcus ibis	Cattle Egret	2021/2/20	1	0	23.7040976	120.4634454	https://www.inaturalist.org/observations/69855746
3576	Bubulcus ibis	Cattle Egret	2020/3/12	1	0	23.7045083	120.4628700	https://www.inaturalist.org/observations/39875100
3577	Bubulcus ibis	Cattle Egret	2020/3/12	1	0	23.7045980	120.4625069	https://www.inaturalist.org/observations/39875076
3578	Bubulcus ibis	Cattle Egret	2020/5/9	1	0	23.7062372	120.3525933	https://www.inaturalist.org/observations/45626131
3579	Bubulcus ibis	Cattle Egret	2020/5/4	1	0	23.7064155	120.3534194	https://www.inaturalist.org/observations/45627901
3580	Bubulcus ibis	Cattle Egret	2020/5/3	1	0	23.7067224	120.3531702	https://www.inaturalist.org/observations/44691851
3581	Bubulcus ibis	Cattle Egret	2021/4/18	4	0	23.7071511	120.4101033	https://www.inaturalist.org/observations/74354436
3582	Bubulcus ibis	Cattle Egret	2021/2/22	1	0	23.7093716	120.5040526	https://www.inaturalist.org/observations/70025647
3583	Bubulcus ibis	Cattle Egret	2019/12/14	1	0	23.7094729	120.5883823	https://www.inaturalist.org/observations/36639938
3584	Bubulcus ibis	Cattle Egret	2020/12/27	1	0	23.7137133	120.3726050	https://www.inaturalist.org/observations/67114940
3585	Bubulcus ibis	Cattle Egret	2020/3/24	1	0	23.7140146	120.3433459	https://www.inaturalist.org/observations/40620770
3586	Bubulcus ibis	Cattle Egret	2021/12/25	1	0	23.7150150	120.3905881	https://www.inaturalist.org/observations/103626677
3587	Bubulcus ibis	Cattle Egret	2021/2/15	1	0	23.7170783	120.3669633	https://www.inaturalist.org/observations/69622177
3588	Bubulcus ibis	Cattle Egret	2020/3/15	1	0	23.7172029	120.4343317	https://www.inaturalist.org/observations/40009211
3589	Bubulcus ibis	Cattle Egret	2019/6/25	1	0	23.7196744	120.3383222	https://www.inaturalist.org/observations/27633103
3590	Bubulcus ibis	Cattle Egret	2020/4/14	1	0	23.7211550	120.3701650	https://www.inaturalist.org/observations/42155829
3591	Bubulcus ibis	Cattle Egret	2020/7/5	1	0	23.7221417	120.3664867	https://www.inaturalist.org/observations/51999358
3592	Bubulcus ibis	Cattle Egret	2020/6/25	1	0	23.7232017	120.3082050	https://www.inaturalist.org/observations/50873191
3593	Bubulcus ibis	Cattle Egret	2022/2/27	1	0	23.7242631	120.4221197	https://www.inaturalist.org/observations/107534166
3594	Bubulcus ibis	Cattle Egret	2022/4/30	1	0	23.7252609	120.6263092	https://www.inaturalist.org/observations/114204833
3595	Bubulcus ibis	Cattle Egret	2020/5/21	1	0	23.7255396	120.6273282	https://www.inaturalist.org/observations/46725252
3596	Bubulcus ibis	Cattle Egret	2022/3/10	1	0	23.7256828	120.3579093	https://www.inaturalist.org/observations/108308111
3597	Bubulcus ibis	Cattle Egret	2021/9/12	1	0	23.7261981	120.4142900	https://www.inaturalist.org/observations/94528453
3598	Bubulcus ibis	Cattle Egret	2022/8/21	2	0	23.7262631	120.4231331	https://www.inaturalist.org/observations/132808589
3599	Bubulcus ibis	Cattle Egret	2020/10/4	2	0	23.7263091	120.4219457	https://www.inaturalist.org/observations/61650603

3600	Bubulcus ibis	Cattle Egret	2021/12/17	1	0	23.7263303	120.4717929	https://www.inaturalist.org/observations/103172809
3601	Bubulcus ibis	Cattle Egret	2022/3/13	1	0	23.7269284	120.3558316	https://www.inaturalist.org/observations/108490418
3602	Bubulcus ibis	Cattle Egret	2019/6/23	1	0	23.7278785	120.6220697	https://www.inaturalist.org/observations/32399534
3603	Bubulcus ibis	Cattle Egret	2019/7/20	2	0	23.7278820	120.6220777	https://www.inaturalist.org/observations/32399532
3604	Bubulcus ibis	Cattle Egret	2019/7/14	1	0	23.7278834	120.6220657	https://www.inaturalist.org/observations/32399533
3605	Bubulcus ibis	Cattle Egret	2019/8/24	1	0	23.7278871	120.6220858	https://www.inaturalist.org/observations/32399536
3606	Bubulcus ibis	Cattle Egret	2013/5/5	3	0	23.7286082	120.6161466	https://www.inaturalist.org/observations/19485801
3607	Bubulcus ibis	Cattle Egret	2022/7/2	0	0	23.7292651	120.3904102	https://www.inaturalist.org/observations/124458030
3608	Bubulcus ibis	Cattle Egret	2020/2/2	0	0	23.7310667	120.3584117	https://www.inaturalist.org/observations/38245504
3609	Bubulcus ibis	Cattle Egret	2019/11/19	2	0	23.7315020	120.3767130	https://www.inaturalist.org/observations/35846029
3610	Bubulcus ibis	Cattle Egret	2018/3/18	2	0	23.7315182	120.3652644	https://www.inaturalist.org/observations/109292128
3611	Bubulcus ibis	Cattle Egret	2020/12/16	0	0	23.7315633	120.3870250	https://www.inaturalist.org/observations/66647134
3612	Bubulcus ibis	Cattle Egret	2021/10/29	1	0	23.7323767	120.5121350	https://www.inaturalist.org/observations/100071258
3613	Bubulcus ibis	Cattle Egret	2020/6/30	2	0	23.7325807	120.3878668	https://www.inaturalist.org/observations/51536433
3614	Bubulcus ibis	Cattle Egret	2020/12/16	1	0	23.7328204	120.3861877	https://www.inaturalist.org/observations/66648506
3615	Bubulcus ibis	Cattle Egret	2019/1/25	2	0	23.7329966	120.3821925	https://www.inaturalist.org/observations/19872283
3616	Bubulcus ibis	Cattle Egret	2021/12/4	1	0	23.7337150	120.3795700	https://www.inaturalist.org/observations/102628875
3617	Bubulcus ibis	Cattle Egret	2021/3/14	3	0	23.7337614	120.4328412	https://www.inaturalist.org/observations/71697431
3618	Bubulcus ibis	Cattle Egret	2021/12/4	1	0	23.7339833	120.3798050	https://www.inaturalist.org/observations/102628876
3619	Bubulcus ibis	Cattle Egret	2020/5/24	1	0	23.7342367	120.4085812	https://www.inaturalist.org/observations/47125803
3620	Bubulcus ibis	Cattle Egret	2021/6/26	3	0	23.7354967	120.3834186	https://www.inaturalist.org/observations/84533136
3621	Bubulcus ibis	Cattle Egret	2019/1/30	2	0	23.7358018	120.3915688	https://www.inaturalist.org/observations/19982891
3622	Bubulcus ibis	Cattle Egret	2021/12/4	2	0	23.7365117	120.3839733	https://www.inaturalist.org/observations/102629885
3623	Bubulcus ibis	Cattle Egret	2020/12/14	0	0	23.7408767	120.6582200	https://www.inaturalist.org/observations/66558573
3624	Bubulcus ibis	Cattle Egret	2022/3/13	1	0	23.7414188	120.3926605	https://www.inaturalist.org/observations/108487605

3625	Bubulcus ibis	Cattle Egret	2021/1/7	3	0	23.7421111	86.1047146	https://www.inaturalist.org/observations/67701136
3626	Bubulcus ibis	Cattle Egret	2018/7/27	2	0	23.7428122	120.5782015	https://www.inaturalist.org/observations/15067712
3627	Bubulcus ibis	Cattle Egret	2022/3/1	1	0	23.7433944	121.4602861	https://www.inaturalist.org/observations/107700089
3628	Bubulcus ibis	Cattle Egret	2020/12/8	1	0	23.7444067	120.3476017	https://www.inaturalist.org/observations/66279806
3629	Bubulcus ibis	Cattle Egret	2021/11/12	2	0	23.7452593	121.4787019	https://www.inaturalist.org/observations/101158123
3630	Bubulcus ibis	Cattle Egret	2020/4/19	1	0	23.7466672	119.6150084	https://www.inaturalist.org/observations/44343265
3631	Bubulcus ibis	Cattle Egret	2020/7/21	0	0	23.7475694	120.2300167	https://www.inaturalist.org/observations/53978380
3632	Bubulcus ibis	Cattle Egret	2022/1/13	1	0	23.7540400	120.5251900	https://www.inaturalist.org/observations/108103921
3633	Bubulcus ibis	Cattle Egret	2017/1/19	3	0	23.7540674	121.4711656	https://www.inaturalist.org/observations/18878884
3634	Bubulcus ibis	Cattle Egret	2022/4/29	1	0	23.7541140	120.6147190	https://www.inaturalist.org/observations/115809549
3635	Bubulcus ibis	Cattle Egret	2020/1/24	0	0	23.7547483	120.5365033	https://www.inaturalist.org/observations/37905251
3636	Bubulcus ibis	Cattle Egret	2022/2/22	1	0	23.7585645	120.5263170	https://www.inaturalist.org/observations/107298417
3637	Bubulcus ibis	Cattle Egret	2020/6/13	2	0	23.7590059	120.3828571	https://www.inaturalist.org/observations/49758754
3638	Bubulcus ibis	Cattle Egret	2022/4/11	1	0	23.7599510	120.5511013	https://www.inaturalist.org/observations/111038608
3639	Bubulcus ibis	Cattle Egret	2020/3/21	1	0	23.7608297	121.4825163	https://www.inaturalist.org/observations/41698757
3640	Bubulcus ibis	Cattle Egret	2021/2/20	1	0	23.7609000	120.5998317	https://www.inaturalist.org/observations/69845365
3641	Bubulcus ibis	Cattle Egret	2020/3/21	1	0	23.7610859	121.4811009	https://www.inaturalist.org/observations/41698760
3642	Bubulcus ibis	Cattle Egret	2020/3/21	1	0	23.7616234	121.4795208	https://www.inaturalist.org/observations/41698770
3643	Bubulcus ibis	Cattle Egret	2022/10/21	2	0	23.7624662	120.2302261	https://www.inaturalist.org/observations/139483614
3644	Bubulcus ibis	Cattle Egret	2021/2/27	1	0	23.7647167	121.4705785	https://www.inaturalist.org/observations/70247452
3645	Bubulcus ibis	Cattle Egret	2022/4/21	2	0	23.7687429	120.2571700	https://www.inaturalist.org/observations/112232762
3646	Bubulcus ibis	Cattle Egret	2022/5/22	1	0	23.7689725	120.5440977	https://www.inaturalist.org/observations/118155136
3647	Bubulcus ibis	Cattle Egret	2022/2/22	1	0	23.7703092	120.5291789	https://www.inaturalist.org/observations/107298420
3648	Bubulcus ibis	Cattle Egret	2020/5/9	1	0	23.7703750	120.3980450	https://www.inaturalist.org/observations/45345938
3649	Bubulcus ibis	Cattle Egret	2022/4/21	1	0	23.7709090	120.2244128	https://www.inaturalist.org/observations/112254624

3650	Bubulcus ibis	Cattle Egret	2022/2/22	1	0	23.7717678	120.5223842	https://www.inaturalist.org/observations/107298082
3651	Bubulcus ibis	Cattle Egret	2021/1/25	1	0	23.7718005	120.6266411	https://www.inaturalist.org/observations/68586957
3652	Bubulcus ibis	Cattle Egret	2020/12/8	1	0	23.7731433	120.3782150	https://www.inaturalist.org/observations/66279054
3653	Bubulcus ibis	Cattle Egret	2022/9/8	2	0	23.7737166	120.6728383	https://www.inaturalist.org/observations/134127736
3654	Bubulcus ibis	Cattle Egret	2021/4/27	1	0	23.7781903	120.5597267	https://www.inaturalist.org/observations/75499340
3655	Bubulcus ibis	Cattle Egret	2021/1/15	1	0	23.7788933	120.4252533	https://www.inaturalist.org/observations/68262201
3656	Bubulcus ibis	Cattle Egret	2021/8/5	1	0	23.7792056	120.5773722	https://www.inaturalist.org/observations/90002973
3657	Bubulcus ibis	Cattle Egret	2021/8/5	1	0	23.7827531	120.5361223	https://www.inaturalist.org/observations/90003289
3658	Bubulcus ibis	Cattle Egret	2021/5/2	2	0	23.7881283	120.2280917	https://www.inaturalist.org/observations/76505667
3659	Bubulcus ibis	Cattle Egret	2019/10/28	3	0	23.7904309	120.4697838	https://www.inaturalist.org/observations/35437751
3660	Bubulcus ibis	Cattle Egret	2020/11/11	1	0	23.7946467	120.4655183	https://www.inaturalist.org/observations/64657444
3661	Bubulcus ibis	Cattle Egret	2022/3/17	1	0	23.7960508	120.2905246	https://www.inaturalist.org/observations/108774719
3662	Bubulcus ibis	Cattle Egret	2022/1/23	2	0	23.7965078	120.3686882	https://www.inaturalist.org/observations/105395107
3663	Bubulcus ibis	Cattle Egret	2022/5/26	0	0	23.7971028	120.4321972	https://www.inaturalist.org/observations/118825211
3664	Bubulcus ibis	Cattle Egret	2020/12/16	1	0	23.7985383	120.3165933	https://www.inaturalist.org/observations/66643878
3665	Bubulcus ibis	Cattle Egret	2021/3/4	1	0	23.7997217	120.2308533	https://www.inaturalist.org/observations/70551628
3666	Bubulcus ibis	Cattle Egret	2022/10/12	1	0	23.8064408	120.8500904	https://www.inaturalist.org/observations/138435951
3667	Bubulcus ibis	Cattle Egret	2022/7/12	1	0	23.8065102	120.3116069	https://www.inaturalist.org/observations/125891283
3668	Bubulcus ibis	Cattle Egret	2022/12/4	0	0	23.8084873	120.3129121	https://www.inaturalist.org/observations/143635141
3669	Bubulcus ibis	Cattle Egret	2022/2/21	3	0	23.8107734	121.5288055	https://www.inaturalist.org/observations/110070791
3670	Bubulcus ibis	Cattle Egret	2019/4/7	1	0	23.8120284	120.2975221	https://www.inaturalist.org/observations/22144840
3671	Bubulcus ibis	Cattle Egret	2020/1/16	1	0	23.8123269	120.7403293	https://www.inaturalist.org/observations/37632269
3672	Bubulcus ibis	Cattle Egret	2021/3/14	1	0	23.8126370	120.2408759	https://www.inaturalist.org/observations/71201712
3673	Bubulcus ibis	Cattle Egret	2020/5/22	1	0	23.8157444	120.3942326	https://www.inaturalist.org/observations/46801005
3674	Bubulcus ibis	Cattle Egret	2020/5/29	1	0	23.8171685	120.3823719	https://www.inaturalist.org/observations/47940673

3675	Bubulcus ibis	Cattle Egret	2022/5/1	1	0	23.8173165	120.3624718	https://www.inaturalist.org/observations/115706934
3676	Bubulcus ibis	Cattle Egret	2020/2/11	1	0	23.8181462	120.3865607	https://www.inaturalist.org/observations/38529653
3677	Bubulcus ibis	Cattle Egret	2022/9/5	1	0	23.8197043	120.5961690	https://www.inaturalist.org/observations/133723372
3678	Bubulcus ibis	Cattle Egret	2022/2/21	3	0	23.8239394	121.5240004	https://www.inaturalist.org/observations/110070790
3679	Bubulcus ibis	Cattle Egret	2022/5/5	1	0	23.8268000	120.8006833	https://www.inaturalist.org/observations/115513344
3680	Bubulcus ibis	Cattle Egret	2022/4/17	2	0	23.8268200	120.8006583	https://www.inaturalist.org/observations/112450470
3681	Bubulcus ibis	Cattle Egret	2021/5/11	2	0	23.8271838	120.8004958	https://www.inaturalist.org/observations/78327891
3682	Bubulcus ibis	Cattle Egret	2021/10/25	1	0	23.8276147	120.5866257	https://www.inaturalist.org/observations/99309601
3683	Bubulcus ibis	Cattle Egret	2018/10/21	3	0	23.8288220	120.6889600	https://www.inaturalist.org/observations/19670688
3684	Bubulcus ibis	Cattle Egret	2021/3/31	1	0	23.8297939	120.9015654	https://www.inaturalist.org/observations/72516935
3685	Bubulcus ibis	Cattle Egret	2015/6/30	2	0	23.8308734	120.5259960	https://www.inaturalist.org/observations/13519117
3686	Bubulcus ibis	Cattle Egret	2019/8/31	1	0	23.8310635	120.5253905	https://www.inaturalist.org/observations/31973758
3687	Bubulcus ibis	Cattle Egret	2022/5/19	1	0	23.8398024	120.8030794	https://www.inaturalist.org/observations/117661336
3688	Bubulcus ibis	Cattle Egret	2020/7/15	2	0	23.8405750	120.9068028	https://www.inaturalist.org/observations/53109954
3689	Bubulcus ibis	Cattle Egret	2018/7/24	4	0	23.8422069	86.2384829	https://www.inaturalist.org/observations/14726978
3690	Bubulcus ibis	Cattle Egret	2005/5/8	2	0	23.8483614	120.3089541	https://www.inaturalist.org/observations/13332450
3691	Bubulcus ibis	Cattle Egret	2020/10/31	2	0	23.8495067	77.9370969	https://www.inaturalist.org/observations/64918317
3692	Bubulcus ibis	Cattle Egret	2022/1/23	2	0	23.8503882	121.4771243	https://www.inaturalist.org/observations/105482735
3693	Bubulcus ibis	Cattle Egret	2022/1/23	2	0	23.8503882	121.4771243	https://www.inaturalist.org/observations/105482738
3694	Bubulcus ibis	Cattle Egret	2022/7/31	1	0	23.8513813	120.9018457	https://www.inaturalist.org/observations/128730991
3695	Bubulcus ibis	Cattle Egret	2022/7/29	1	0	23.8520298	120.9022521	https://www.inaturalist.org/observations/141984944
3696	Bubulcus ibis	Cattle Egret	2021/4/19	1	0	23.8563524	120.7786586	https://www.inaturalist.org/observations/74704908
3697	Bubulcus ibis	Cattle Egret	2021/5/11	1	0	23.8575467	120.7803591	https://www.inaturalist.org/observations/78317232
3698	Bubulcus ibis	Cattle Egret	2018/9/20	1	0	23.8579352	120.9428644	https://www.inaturalist.org/observations/81811604
3699	Bubulcus ibis	Cattle Egret	2022/7/3	3	0	23.8582528	120.8552139	https://www.inaturalist.org/observations/144009878

3700	Bubulcus ibis	Cattle Egret	2018/8/7	3	0	23.8584049	120.4816737	https://www.inaturalist.org/observations/15314395
3701	Bubulcus ibis	Cattle Egret	2022/8/29	2	0	23.8593411	121.5598345	https://www.inaturalist.org/observations/139398150
3702	Bubulcus ibis	Cattle Egret	2020/7/15	1	0	23.8619417	120.9008861	https://www.inaturalist.org/observations/53113319
3703	Bubulcus ibis	Cattle Egret	2020/10/12	2	0	23.8621229	120.9015809	https://www.inaturalist.org/observations/62373773
3704	Bubulcus ibis	Cattle Egret	2022/1/23	1	0	23.8723010	86.9150760	https://www.inaturalist.org/observations/105429647
3705	Bubulcus ibis	Cattle Egret	2018/9/29	0	0	23.8736656	120.9223934	https://www.inaturalist.org/observations/37770108
3706	Bubulcus ibis	Cattle Egret	2020/5/2	1	0	23.8798679	121.5506854	https://www.inaturalist.org/observations/44604989
3707	Bubulcus ibis	Cattle Egret	2021/10/22	2	0	23.8837678	84.1917750	https://www.inaturalist.org/observations/98976530
3708	Bubulcus ibis	Cattle Egret	2020/5/8	1	0	23.8908892	121.5408042	https://www.inaturalist.org/observations/45259149
3709	Bubulcus ibis	Cattle Egret	2020/10/28	1	0	23.8911287	121.5350655	https://www.inaturalist.org/observations/67299909
3710	Bubulcus ibis	Cattle Egret	2019/10/3	2	0	23.8921411	121.5357268	https://www.inaturalist.org/observations/33790303
3711	Bubulcus ibis	Cattle Egret	2021/11/3	1	0	23.8924448	121.5362810	https://www.inaturalist.org/observations/100400558
3712	Bubulcus ibis	Cattle Egret	2019/10/17	2	0	23.8930550	121.5450130	https://www.inaturalist.org/observations/34489309
3713	Bubulcus ibis	Cattle Egret	2019/10/17	1	0	23.8932694	121.5450784	https://www.inaturalist.org/observations/34489737
3714	Bubulcus ibis	Cattle Egret	2020/10/20	1	0	23.8939602	121.5347851	https://www.inaturalist.org/observations/63469459
3715	Bubulcus ibis	Cattle Egret	2014/5/15	4	0	23.8941759	121.5484643	https://www.inaturalist.org/observations/12264699
3716	Bubulcus ibis	Cattle Egret	2020/10/7	1	0	23.8942900	121.5440567	https://www.inaturalist.org/observations/63470734
3717	Bubulcus ibis	Cattle Egret	2021/11/6	1	0	23.8950393	121.5451450	https://www.inaturalist.org/observations/100374361
3718	Bubulcus ibis	Cattle Egret	2022/10/1	1	0	23.8958389	121.5449553	https://www.inaturalist.org/observations/137125227
3719	Bubulcus ibis	Cattle Egret	2020/7/9	1	0	23.8962978	121.5451427	https://www.inaturalist.org/observations/54427620
3720	Bubulcus ibis	Cattle Egret	2020/7/9	1	0	23.8963051	121.5452500	https://www.inaturalist.org/observations/54427623
3721	Bubulcus ibis	Cattle Egret	2020/4/14	2	0	23.8963543	121.5452992	https://www.inaturalist.org/observations/42158228
3722	Bubulcus ibis	Cattle Egret	2020/7/9	1	0	23.8963626	121.5452698	https://www.inaturalist.org/observations/54427611
3723	Bubulcus ibis	Cattle Egret	2020/7/9	2	0	23.8963640	121.5452768	https://www.inaturalist.org/observations/54427624
3724	Bubulcus ibis	Cattle Egret	2019/9/29	2	0	23.8963674	121.5449125	https://www.inaturalist.org/observations/33584840

3725	Bubulcus ibis	Cattle Egret	2020/5/5	1	0	23.8963725	121.5452272	https://www.inaturalist.org/observations/44971117
3726	Bubulcus ibis	Cattle Egret	2020/5/2	3	0	23.8963740	121.5453444	https://www.inaturalist.org/observations/44541811
3727	Bubulcus ibis	Cattle Egret	2020/5/18	1	0	23.8963761	121.5452004	https://www.inaturalist.org/observations/46363096
3728	Bubulcus ibis	Cattle Egret	2020/5/18	3	0	23.8963829	121.5451605	https://www.inaturalist.org/observations/46363090
3729	Bubulcus ibis	Cattle Egret	2020/10/27	2	0	23.8963876	121.5452113	https://www.inaturalist.org/observations/63675691
3730	Bubulcus ibis	Cattle Egret	2019/10/17	2	0	23.8964240	121.5448720	https://www.inaturalist.org/observations/34489298
3731	Bubulcus ibis	Cattle Egret	2020/4/29	4	0	23.8964833	121.5448550	https://www.inaturalist.org/observations/44238186
3732	Bubulcus ibis	Cattle Egret	2020/4/29	3	0	23.8965233	121.5448233	https://www.inaturalist.org/observations/44238181
3733	Bubulcus ibis	Cattle Egret	2019/11/30	2	0	23.8965994	120.5821831	https://www.inaturalist.org/observations/37366305
3734	Bubulcus ibis	Cattle Egret	2020/11/12	1	0	23.8966290	121.5476763	https://www.inaturalist.org/observations/64709463
3735	Bubulcus ibis	Cattle Egret	2019/10/17	2	0	23.8967200	121.5451486	https://www.inaturalist.org/observations/34489724
3736	Bubulcus ibis	Cattle Egret	2021/11/24	0	0	23.8967220	121.5364185	https://www.inaturalist.org/observations/102153058
3737	Bubulcus ibis	Cattle Egret	2019/9/29	2	0	23.8967269	121.5458883	https://www.inaturalist.org/observations/33591729
3738	Bubulcus ibis	Cattle Egret	2019/10/9	2	0	23.8967389	121.5458819	https://www.inaturalist.org/observations/34118476
3739	Bubulcus ibis	Cattle Egret	2020/9/26	2	0	23.8968601	121.5461619	https://www.inaturalist.org/observations/61129838
3740	Bubulcus ibis	Cattle Egret	2017/2/28	4	0	23.8969441	120.9369431	https://www.inaturalist.org/observations/5215502
3741	Bubulcus ibis	Cattle Egret	2020/9/26	2	0	23.8969562	121.5461771	https://www.inaturalist.org/observations/61129837
3742	Bubulcus ibis	Cattle Egret	2020/10/26	1	0	23.8969837	121.5457470	https://www.inaturalist.org/observations/63579796
3743	Bubulcus ibis	Cattle Egret	2019/10/9	1	0	23.8970095	121.5471359	https://www.inaturalist.org/observations/34489536
3744	Bubulcus ibis	Cattle Egret	2020/10/28	1	0	23.8972133	121.5472850	https://www.inaturalist.org/observations/63675679
3745	Bubulcus ibis	Cattle Egret	2019/10/4	2	0	23.8973999	121.5342177	https://www.inaturalist.org/observations/34104159
3746	Bubulcus ibis	Cattle Egret	2019/9/29	2	0	23.8976021	121.5509933	https://www.inaturalist.org/observations/33584815
3747	Bubulcus ibis	Cattle Egret	2021/12/1	1	0	23.8976303	121.5467370	https://www.inaturalist.org/observations/102291280
3748	Bubulcus ibis	Cattle Egret	2021/12/1	1	0	23.8979530	121.5464526	https://www.inaturalist.org/observations/102291312
3749	Bubulcus ibis	Cattle Egret	2022/6/3	2	0	23.8979538	121.5413977	https://www.inaturalist.org/observations/120009374

3750	Bubulcus ibis	Cattle Egret	2021/12/13	1	0	23.8983528	121.5462222	https://www.inaturalist.org/observations/103003086
3751	Bubulcus ibis	Cattle Egret	2022/12/24	1	0	23.8992556	121.5496778	https://www.inaturalist.org/observations/144972970
3752	Bubulcus ibis	Cattle Egret	2019/11/6	2	0	23.8993830	121.5413470	https://www.inaturalist.org/observations/35367337
3753	Bubulcus ibis	Cattle Egret	2019/11/6	2	0	23.8993833	121.5413350	https://www.inaturalist.org/observations/35366359
3754	Bubulcus ibis	Cattle Egret	2015/10/11	2	0	23.8994826	121.5413886	https://www.inaturalist.org/observations/12229662
3755	Bubulcus ibis	Cattle Egret	2022/2/24	1	0	23.8998966	121.5249341	https://www.inaturalist.org/observations/107423072
3756	Bubulcus ibis	Cattle Egret	2022/2/24	2	0	23.8998966	121.5249341	https://www.inaturalist.org/observations/107476777
3757	Bubulcus ibis	Cattle Egret	2022/2/24	1	0	23.8998966	121.5249341	https://www.inaturalist.org/observations/107476787
3758	Bubulcus ibis	Cattle Egret	2019/12/19	1	0	23.8999010	121.5425390	https://www.inaturalist.org/observations/36768103
3759	Bubulcus ibis	Cattle Egret	2019/10/8	1	0	23.8999140	121.5503460	https://www.inaturalist.org/observations/34060125
3760	Bubulcus ibis	Cattle Egret	2019/10/10	2	0	23.9000847	121.5418801	https://www.inaturalist.org/observations/34164439
3761	Bubulcus ibis	Cattle Egret	2019/10/9	1	0	23.9004022	121.5458220	https://www.inaturalist.org/observations/34740358
3762	Bubulcus ibis	Cattle Egret	2019/10/14	2	0	23.9004515	121.5420310	https://www.inaturalist.org/observations/34362498
3763	Bubulcus ibis	Cattle Egret	2020/11/20	1	0	23.9005116	121.5392496	https://www.inaturalist.org/observations/65251776
3764	Bubulcus ibis	Cattle Egret	2022/10/15	1	0	23.9005583	121.5364722	https://www.inaturalist.org/observations/138799946
3765	Bubulcus ibis	Cattle Egret	2022/10/15	1	0	23.9005639	121.5365111	https://www.inaturalist.org/observations/138800024
3766	Bubulcus ibis	Cattle Egret	2019/10/10	2	0	23.9005810	121.5366520	https://www.inaturalist.org/observations/34155401
3767	Bubulcus ibis	Cattle Egret	2022/10/15	1	0	23.9006444	121.5366278	https://www.inaturalist.org/observations/138800203
3768	Bubulcus ibis	Cattle Egret	2020/11/7	1	0	23.9006677	121.5486777	https://www.inaturalist.org/observations/64444449
3769	Bubulcus ibis	Cattle Egret	2019/10/25	2	0	23.9006967	121.5384805	https://www.inaturalist.org/observations/35015155
3770	Bubulcus ibis	Cattle Egret	2019/10/25	2	0	23.9007061	121.5384288	https://www.inaturalist.org/observations/34874614
3771	Bubulcus ibis	Cattle Egret	2019/10/10	2	0	23.9007132	121.5420245	https://www.inaturalist.org/observations/34155416
3772	Bubulcus ibis	Cattle Egret	2020/5/17	2	0	23.9007573	121.5364921	https://www.inaturalist.org/observations/46469347
3773	Bubulcus ibis	Cattle Egret	2020/4/25	2	0	23.9007717	121.5423650	https://www.inaturalist.org/observations/43288737
3774	Bubulcus ibis	Cattle Egret	2019/10/10	2	0	23.9007889	121.5362898	https://www.inaturalist.org/observations/34164435

3775	Bubulcus ibis	Cattle Egret	2019/12/25	2	0	23.9008080	121.5461350	https://www.inaturalist.org/observations/36931993
3776	Bubulcus ibis	Cattle Egret	2019/10/14	3	0	23.9008580	121.5423970	https://www.inaturalist.org/observations/34365665
3777	Bubulcus ibis	Cattle Egret	2022/4/2	1	0	23.9008887	121.5457708	https://www.inaturalist.org/observations/119842724
3778	Bubulcus ibis	Cattle Egret	2019/10/25	2	0	23.9009300	121.5396583	https://www.inaturalist.org/observations/34854517
3779	Bubulcus ibis	Cattle Egret	2019/12/21	1	0	23.9009776	121.5456041	https://www.inaturalist.org/observations/36883375
3780	Bubulcus ibis	Cattle Egret	2020/11/14	2	0	23.9009857	121.5423445	https://www.inaturalist.org/observations/64840781
3781	Bubulcus ibis	Cattle Egret	2022/4/22	2	0	23.9013585	121.5388503	https://www.inaturalist.org/observations/119551389
3782	Bubulcus ibis	Cattle Egret	2020/4/24	3	0	23.9015146	121.5383491	https://www.inaturalist.org/observations/47111673
3783	Bubulcus ibis	Cattle Egret	2019/10/10	2	0	23.9016298	121.5365225	https://www.inaturalist.org/observations/34164422
3784	Bubulcus ibis	Cattle Egret	2020/4/24	2	0	23.9016867	121.5385159	https://www.inaturalist.org/observations/43025307
3785	Bubulcus ibis	Cattle Egret	2019/10/9	2	0	23.9019190	121.5387510	https://www.inaturalist.org/observations/34111532
3786	Bubulcus ibis	Cattle Egret	2019/11/22	2	0	23.9020258	121.5472101	https://www.inaturalist.org/observations/35930636
3787	Bubulcus ibis	Cattle Egret	2020/4/24	3	0	23.9020761	121.5382218	https://www.inaturalist.org/observations/47111678
3788	Bubulcus ibis	Cattle Egret	2020/4/24	3	0	23.9021257	121.5370400	https://www.inaturalist.org/observations/47111680
3789	Bubulcus ibis	Cattle Egret	2020/4/24	3	0	23.9022265	121.5385613	https://www.inaturalist.org/observations/43025305
3790	Bubulcus ibis	Cattle Egret	2020/4/24	3	0	23.9023090	121.5386166	https://www.inaturalist.org/observations/47111672
3791	Bubulcus ibis	Cattle Egret	2020/4/24	3	0	23.9027628	121.5383742	https://www.inaturalist.org/observations/47111674
3792	Bubulcus ibis	Cattle Egret	2018/5/13	2	0	23.9029617	121.5366367	https://www.inaturalist.org/observations/12408128
3793	Bubulcus ibis	Cattle Egret	2019/9/29	2	0	23.9031045	121.5470554	https://www.inaturalist.org/observations/33584811
3794	Bubulcus ibis	Cattle Egret	2022/4/2	2	0	23.9049597	121.5412397	https://www.inaturalist.org/observations/110162250
3795	Bubulcus ibis	Cattle Egret	2021/2/26	1	0	23.9064350	91.3131860	https://www.inaturalist.org/observations/80062590
3796	Bubulcus ibis	Cattle Egret	2018/8/19	2	0	23.9066938	121.5283312	https://www.inaturalist.org/observations/38982837
3797	Bubulcus ibis	Cattle Egret	2018/8/4	3	0	23.9068823	120.9357594	https://www.inaturalist.org/observations/15285259
3798	Bubulcus ibis	Cattle Egret	2020/1/6	1	0	23.9096830	121.5417440	https://www.inaturalist.org/observations/37395060
3799	Bubulcus ibis	Cattle Egret	2021/11/19	2	0	23.9097307	121.5538534	https://www.inaturalist.org/observations/101451728

3800	Bubulcus ibis	Cattle Egret	2021/10/21	1	0	23.9135857	121.5416760	https://www.inaturalist.org/observations/98861894
3801	Bubulcus ibis	Cattle Egret	2014/4/4	3	0	23.9226115	120.3179570	https://www.inaturalist.org/observations/11264246
3802	Bubulcus ibis	Cattle Egret	2020/8/5	1	0	23.9287417	120.3160722	https://www.inaturalist.org/observations/55471149
3803	Bubulcus ibis	Cattle Egret	2022/9/13	1	0	23.9312179	120.3217486	https://www.inaturalist.org/observations/134855733
3804	Bubulcus ibis	Cattle Egret	2022/9/13	1	0	23.9312179	120.3217486	https://www.inaturalist.org/observations/134855782
3805	Bubulcus ibis	Cattle Egret	2022/8/31	1	0	23.9315422	120.3146901	https://www.inaturalist.org/observations/133164347
3806	Bubulcus ibis	Cattle Egret	2022/11/4	1	0	23.9340368	120.7766800	https://www.inaturalist.org/observations/141058356
3807	Bubulcus ibis	Cattle Egret	2020/5/2	1	0	23.9350400	121.6032354	https://www.inaturalist.org/observations/44603378
3808	Bubulcus ibis	Cattle Egret	2022/9/13	1	0	23.9371026	120.3144883	https://www.inaturalist.org/observations/134820445
3809	Bubulcus ibis	Cattle Egret	2020/4/14	1	0	23.9402067	120.3166567	https://www.inaturalist.org/observations/42155838
3810	Bubulcus ibis	Cattle Egret	2021/10/7	1	0	23.9483690	121.5544547	https://www.inaturalist.org/observations/97435043
3811	Bubulcus ibis	Cattle Egret	2020/6/13	1	0	23.9504082	120.9682669	https://www.inaturalist.org/observations/49403332
3812	Bubulcus ibis	Cattle Egret	2021/11/22	2	0	23.9517912	120.9289827	https://www.inaturalist.org/observations/108223512
3813	Bubulcus ibis	Cattle Egret	2020/4/5	2	0	23.9532399	120.4078138	https://www.inaturalist.org/observations/41432636
3814	Bubulcus ibis	Cattle Egret	2022/11/13	1	0	23.9561888	120.3351409	https://www.inaturalist.org/observations/144187677
3815	Bubulcus ibis	Cattle Egret	2022/11/13	1	0	23.9561932	120.3351405	https://www.inaturalist.org/observations/144187678
3816	Bubulcus ibis	Cattle Egret	2022/11/13	1	0	23.9561958	120.3351421	https://www.inaturalist.org/observations/144187679
3817	Bubulcus ibis	Cattle Egret	2022/12/11	1	0	23.9565025	120.3369255	https://www.inaturalist.org/observations/144144540
3818	Bubulcus ibis	Cattle Egret	2020/11/7	1	0	23.9651371	120.9673768	https://www.inaturalist.org/observations/64349155
3819	Bubulcus ibis	Cattle Egret	2020/11/7	1	0	23.9651371	120.9673768	https://www.inaturalist.org/observations/64349162
3820	Bubulcus ibis	Cattle Egret	2020/11/16	1	0	23.9651371	120.9673768	https://www.inaturalist.org/observations/65005455
3821	Bubulcus ibis	Cattle Egret	2020/11/16	1	0	23.9651371	120.9673768	https://www.inaturalist.org/observations/65005456
3822	Bubulcus ibis	Cattle Egret	2020/11/23	1	0	23.9651371	120.9673768	https://www.inaturalist.org/observations/65464498
3823	Bubulcus ibis	Cattle Egret	2020/11/23	1	0	23.9651371	120.9673768	https://www.inaturalist.org/observations/65464499
3824	Bubulcus ibis	Cattle Egret	2020/11/24	1	0	23.9651371	120.9673768	https://www.inaturalist.org/observations/65522766

3825	Bubulcus ibis	Cattle Egret	2020/11/24	2	0	23.9651371	120.9673768	https://www.inaturalist.org/observations/65522768
3826	Bubulcus ibis	Cattle Egret	2020/11/27	1	0	23.9651371	120.9673768	https://www.inaturalist.org/observations/65686024
3827	Bubulcus ibis	Cattle Egret	2020/12/5	2	0	23.9651371	120.9673768	https://www.inaturalist.org/observations/66285045
3828	Bubulcus ibis	Cattle Egret	2022/7/2	3	0	23.9657093	120.9897034	https://www.inaturalist.org/observations/128436132
3829	Bubulcus ibis	Cattle Egret	2022/7/2	3	0	23.9657168	120.9896980	https://www.inaturalist.org/observations/128436133
3830	Bubulcus ibis	Cattle Egret	2022/7/2	3	0	23.9657266	120.9896980	https://www.inaturalist.org/observations/128436134
3831	Bubulcus ibis	Cattle Egret	2013/11/16	4	0	23.9668750	120.9662170	https://www.inaturalist.org/observations/9264741
3832	Bubulcus ibis	Cattle Egret	2009/6/25	3	0	23.9846346	120.4214386	https://www.inaturalist.org/observations/19284783
3833	Bubulcus ibis	Cattle Egret	2021/7/29	2	0	23.9860133	120.7238167	https://www.inaturalist.org/observations/89289992
3834	Bubulcus ibis	Cattle Egret	2022/3/5	1	0	23.9861467	72.9691250	https://www.inaturalist.org/observations/120118678
3835	Bubulcus ibis	Cattle Egret	2022/3/5	2	0	23.9866017	72.9682100	https://www.inaturalist.org/observations/120122843
3836	Bubulcus ibis	Cattle Egret	2019/3/10	2	0	23.9888598	120.5765290	https://www.inaturalist.org/observations/21105474
3837	Bubulcus ibis	Cattle Egret	2021/3/15	2	0	23.9888817	120.7258517	https://www.inaturalist.org/observations/71349888
3838	Bubulcus ibis	Cattle Egret	2019/3/10	2	0	23.9894451	120.5784425	https://www.inaturalist.org/observations/21105473
3839	Bubulcus ibis	Cattle Egret	2022/6/27	1	0	23.9914083	120.7383483	https://www.inaturalist.org/observations/124356545
3840	Bubulcus ibis	Cattle Egret	2022/6/27	1	0	23.9914617	120.7382817	https://www.inaturalist.org/observations/124356544
3841	Bubulcus ibis	Cattle Egret	2021/12/1	2	0	23.9933000	120.7379983	https://www.inaturalist.org/observations/102804754
3842	Bubulcus ibis	Cattle Egret	2022/8/8	1	0	23.9934067	120.7379783	https://www.inaturalist.org/observations/129936247
3843	Bubulcus ibis	Cattle Egret	2022/8/8	2	0	23.9934067	120.7379783	https://www.inaturalist.org/observations/129936249
3844	Bubulcus ibis	Cattle Egret	2014/7/5	1	0	23.9943397	120.7716826	https://www.inaturalist.org/observations/49315213
3845	Bubulcus ibis	Cattle Egret	2022/6/27	2	0	23.9972783	120.7221400	https://www.inaturalist.org/observations/124356550
3846	Bubulcus ibis	Cattle Egret	2022/8/8	3	0	23.9981550	120.7342150	https://www.inaturalist.org/observations/129936256
3847	Bubulcus ibis	Cattle Egret	2020/5/1	1	0	23.9998515	120.5726130	https://www.inaturalist.org/observations/44469167
3848	Bubulcus ibis	Cattle Egret	2019/4/9	2	0	24.0007985	120.5735332	https://www.inaturalist.org/observations/22267910
3849	Bubulcus ibis	Cattle Egret	2019/11/9	1	0	24.0011100	120.5346430	https://www.inaturalist.org/observations/35475423

3850	Bubulcus ibis	Cattle Egret	2019/3/21	5	0	24.0018103	120.5682324	https://www.inaturalist.org/observations/21512265
3851	Bubulcus ibis	Cattle Egret	2019/9/7	1	0	24.0019138	120.5325620	https://www.inaturalist.org/observations/32251550
3852	Bubulcus ibis	Cattle Egret	2019/9/7	2	0	24.0022667	120.5321329	https://www.inaturalist.org/observations/32251608
3853	Bubulcus ibis	Cattle Egret	2020/11/16	1	0	24.0030172	120.5686962	https://www.inaturalist.org/observations/65006214
3854	Bubulcus ibis	Cattle Egret	2019/7/24	4	0	24.0037743	120.5325703	https://www.inaturalist.org/observations/29442478
3855	Bubulcus ibis	Cattle Egret	2019/6/15	2	0	24.0038550	120.5322533	https://www.inaturalist.org/observations/27022980
3856	Bubulcus ibis	Cattle Egret	2019/6/15	3	0	24.0043000	120.5352483	https://www.inaturalist.org/observations/27023305
3857	Bubulcus ibis	Cattle Egret	2019/7/7	2	0	24.0043017	120.5352283	https://www.inaturalist.org/observations/28341765
3858	Bubulcus ibis	Cattle Egret	2019/8/28	1	0	24.0052650	120.5342800	https://www.inaturalist.org/observations/31826734
3859	Bubulcus ibis	Cattle Egret	2019/8/28	2	0	24.0052667	120.5342900	https://www.inaturalist.org/observations/31826712
3860	Bubulcus ibis	Cattle Egret	2019/4/23	3	0	24.0054864	120.5335794	https://www.inaturalist.org/observations/22940272
3861	Bubulcus ibis	Cattle Egret	2020/2/10	1	0	24.0058205	120.5675562	https://www.inaturalist.org/observations/38503085
3862	Bubulcus ibis	Cattle Egret	2022/12/5	1	0	24.0070983	120.7046683	https://www.inaturalist.org/observations/143859256
3863	Bubulcus ibis	Cattle Egret	2018/12/18	3	0	24.0088129	120.5631787	https://www.inaturalist.org/observations/19649786
3864	Bubulcus ibis	Cattle Egret	2022/1/14	2	0	24.0090083	120.7179650	https://www.inaturalist.org/observations/105249745
3865	Bubulcus ibis	Cattle Egret	2021/3/15	2	0	24.0090283	120.7181083	https://www.inaturalist.org/observations/71349872
3866	Bubulcus ibis	Cattle Egret	2021/4/12	1	0	24.0091250	120.7191367	https://www.inaturalist.org/observations/74942659
3867	Bubulcus ibis	Cattle Egret	2021/3/15	2	0	24.0091250	120.7196633	https://www.inaturalist.org/observations/71349874
3868	Bubulcus ibis	Cattle Egret	2022/11/18	1	0	24.0091367	120.7192033	https://www.inaturalist.org/observations/143859228
3869	Bubulcus ibis	Cattle Egret	2022/1/14	1	0	24.0091400	120.7194617	https://www.inaturalist.org/observations/105249749
3870	Bubulcus ibis	Cattle Egret	2022/12/9	1	0	24.0091683	120.7192567	https://www.inaturalist.org/observations/144005187
3871	Bubulcus ibis	Cattle Egret	2022/9/12	1	0	24.0092250	120.7199500	https://www.inaturalist.org/observations/137510217
3872	Bubulcus ibis	Cattle Egret	2022/9/12	1	0	24.0092433	120.7199483	https://www.inaturalist.org/observations/137510219
3873	Bubulcus ibis	Cattle Egret	2022/8/29	2	0	24.0097833	120.7198983	https://www.inaturalist.org/observations/133862358
3874	Bubulcus ibis	Cattle Egret	2022/10/12	2	0	24.0121250	120.4706417	https://www.inaturalist.org/observations/140037103

3875	Bubulcus ibis	Cattle Egret	2020/4/25	2	0	24.0122898	120.4810217	https://www.inaturalist.org/observations/43333474
3876	Bubulcus ibis	Cattle Egret	2020/4/25	2	0	24.0128558	120.4769686	https://www.inaturalist.org/observations/43333477
3877	Bubulcus ibis	Cattle Egret	2020/4/26	1	0	24.0137197	120.3718728	https://www.inaturalist.org/observations/44345107
3878	Bubulcus ibis	Cattle Egret	2022/7/15	2	0	24.0187020	120.6174246	https://www.inaturalist.org/observations/136016319
3879	Bubulcus ibis	Cattle Egret	2019/6/13	1	0	24.0210101	120.3738643	https://www.inaturalist.org/observations/36499900
3880	Bubulcus ibis	Cattle Egret	2018/11/10	3	0	24.0265675	120.4729903	https://www.inaturalist.org/observations/18314372
3881	Bubulcus ibis	Cattle Egret	2019/6/13	1	0	24.0282505	120.4197376	https://www.inaturalist.org/observations/36499878
3882	Bubulcus ibis	Cattle Egret	2022/10/28	1	0	24.0288096	120.4733412	https://www.inaturalist.org/observations/140621016
3883	Bubulcus ibis	Cattle Egret	2022/12/15	1	0	24.0300200	120.6287317	https://www.inaturalist.org/observations/145190447
3884	Bubulcus ibis	Cattle Egret	2022/3/4	2	0	24.0314139	120.6882806	https://www.inaturalist.org/observations/107874895
3885	Bubulcus ibis	Cattle Egret	2019/6/13	1	0	24.0359849	120.3818686	https://www.inaturalist.org/observations/36499920
3886	Bubulcus ibis	Cattle Egret	2018/1/14	3	0	24.0371540	120.4683328	https://www.inaturalist.org/observations/9482019
3887	Bubulcus ibis	Cattle Egret	2022/12/15	1	0	24.0372067	120.6277450	https://www.inaturalist.org/observations/145190480
3888	Bubulcus ibis	Cattle Egret	2018/10/22	2	0	24.0378922	120.3837514	https://www.inaturalist.org/observations/18430347
3889	Bubulcus ibis	Cattle Egret	2020/4/3	1	0	24.0380917	120.3852600	https://www.inaturalist.org/observations/41312962
3890	Bubulcus ibis	Cattle Egret	2020/7/5	1	0	24.0404450	120.3870467	https://www.inaturalist.org/observations/59689817
3891	Bubulcus ibis	Cattle Egret	2021/4/20	1	0	24.0418973	120.3797898	https://www.inaturalist.org/observations/93714180
3892	Bubulcus ibis	Cattle Egret	2022/4/2	2	0	24.0444879	88.7105797	https://www.inaturalist.org/observations/117279861
3893	Bubulcus ibis	Cattle Egret	2020/1/30	3	0	24.0454286	70.1455805	https://www.inaturalist.org/observations/51869623
3894	Bubulcus ibis	Cattle Egret	2020/7/11	1	0	24.0457317	120.6329250	https://www.inaturalist.org/observations/53049583
3895	Bubulcus ibis	Cattle Egret	2021/4/17	1	0	24.0462188	120.4152574	https://www.inaturalist.org/observations/74277717
3896	Bubulcus ibis	Cattle Egret	2015/4/3	2	0	24.0469257	88.7105357	https://www.inaturalist.org/observations/117567649
3897	Bubulcus ibis	Cattle Egret	2021/8/17	3	0	24.0555951	120.4705290	https://www.inaturalist.org/observations/91610034
3898	Bubulcus ibis	Cattle Egret	2021/8/18	2	0	24.0569332	120.4683737	https://www.inaturalist.org/observations/91713237
3899	Bubulcus ibis	Cattle Egret	2021/4/17	1	0	24.0679323	120.4523265	https://www.inaturalist.org/observations/74277716

3900	Bubulcus ibis	Cattle Egret	2021/5/1	1	0	24.0764306	120.6950072	https://www.inaturalist.org/observations/76033364
3901	Bubulcus ibis	Cattle Egret	2015/11/23	2	0	24.0782745	88.6916530	https://www.inaturalist.org/observations/117575923
3902	Bubulcus ibis	Cattle Egret	2021/5/24	2	0	24.1122550	120.6720017	https://www.inaturalist.org/observations/84674983
3903	Bubulcus ibis	Cattle Egret	2021/1/10	1	0	24.1125264	120.6716556	https://www.inaturalist.org/observations/67864883
3904	Bubulcus ibis	Cattle Egret	2021/4/17	1	0	24.1129565	120.4309930	https://www.inaturalist.org/observations/74277719
3905	Bubulcus ibis	Cattle Egret	2021/6/12	1	0	24.1149493	120.4432680	https://www.inaturalist.org/observations/82612748
3906	Bubulcus ibis	Cattle Egret	2020/5/12	1	0	24.1169201	120.4696670	https://www.inaturalist.org/observations/45667866
3907	Bubulcus ibis	Cattle Egret	2020/12/14	2	0	24.1174612	120.6764217	https://www.inaturalist.org/observations/66566706
3908	Bubulcus ibis	Cattle Egret	2018/9/20	1	0	24.1223795	121.2724378	https://www.inaturalist.org/observations/68532372
3909	Bubulcus ibis	Cattle Egret	2016/10/14	3	0	24.1228000	120.6737367	https://www.inaturalist.org/observations/18530126
3910	Bubulcus ibis	Cattle Egret	2022/2/25	2	0	24.1235000	120.6752250	https://www.inaturalist.org/observations/107673851
3911	Bubulcus ibis	Cattle Egret	2021/12/19	2	0	24.1235722	120.6783617	https://www.inaturalist.org/observations/103290199
3912	Bubulcus ibis	Cattle Egret	2021/11/23	1	0	24.1241777	120.6743957	https://www.inaturalist.org/observations/101748846
3913	Bubulcus ibis	Cattle Egret	2022/1/7	1	0	24.1241840	120.6747890	https://www.inaturalist.org/observations/104503167
3914	Bubulcus ibis	Cattle Egret	2020/7/15	3	0	24.1244017	82.7791167	https://www.inaturalist.org/observations/72994325
3915	Bubulcus ibis	Cattle Egret	2020/12/20	2	0	24.1344893	120.6274346	https://www.inaturalist.org/observations/66954705
3916	Bubulcus ibis	Cattle Egret	2020/11/26	1	0	24.1370886	120.6911319	https://www.inaturalist.org/observations/66965651
3917	Bubulcus ibis	Cattle Egret	2020/11/26	1	0	24.1371140	120.6911158	https://www.inaturalist.org/observations/66965671
3918	Bubulcus ibis	Cattle Egret	2020/4/17	1	0	24.1371287	120.6913607	https://www.inaturalist.org/observations/43271317
3919	Bubulcus ibis	Cattle Egret	2022/4/8	2	0	24.1373128	120.6909640	https://www.inaturalist.org/observations/129112994
3920	Bubulcus ibis	Cattle Egret	2020/12/26	1	0	24.1374214	120.6911408	https://www.inaturalist.org/observations/78279085
3921	Bubulcus ibis	Cattle Egret	2022/6/20	2	0	24.1448133	120.6301967	https://www.inaturalist.org/observations/122813750
3922	Bubulcus ibis	Cattle Egret	2021/12/14	1	0	24.1485790	120.7299527	https://www.inaturalist.org/observations/103260647
3923	Bubulcus ibis	Cattle Egret	2019/10/29	3	0	24.1579475	121.6215106	https://www.inaturalist.org/observations/36523168
3924	Bubulcus ibis	Cattle Egret	2019/1/5	3	0	24.1587497	120.7044859	https://www.inaturalist.org/observations/19675748

3925	Bubulcus ibis	Cattle Egret	2021/7/16	1	0	24.1710500	120.7698283	https://www.inaturalist.org/observations/87246730
3926	Bubulcus ibis	Cattle Egret	2020/7/14	1	0	24.1759517	120.6305350	https://www.inaturalist.org/observations/53133306
3927	Bubulcus ibis	Cattle Egret	2022/9/13	1	0	24.1777632	120.6566000	https://www.inaturalist.org/observations/134816428
3928	Bubulcus ibis	Cattle Egret	2022/9/1	1	0	24.1779848	120.6564003	https://www.inaturalist.org/observations/133186148
3929	Bubulcus ibis	Cattle Egret	2020/4/21	3	0	24.1788690	120.6014960	https://www.inaturalist.org/observations/42737969
3930	Bubulcus ibis	Cattle Egret	2022/11/1	1	0	24.1788802	120.6561878	https://www.inaturalist.org/observations/140748110
3931	Bubulcus ibis	Cattle Egret	2022/12/25	1	0	24.1789551	120.6001434	https://www.inaturalist.org/observations/145029835
3932	Bubulcus ibis	Cattle Egret	2022/12/14	1	0	24.1789660	120.6009150	https://www.inaturalist.org/observations/144346127
3933	Bubulcus ibis	Cattle Egret	2022/10/25	3	0	24.1792756	120.6552911	https://www.inaturalist.org/observations/139943585
3934	Bubulcus ibis	Cattle Egret	2022/9/27	2	0	24.1793694	120.6002445	https://www.inaturalist.org/observations/136668059
3935	Bubulcus ibis	Cattle Egret	2022/10/18	1	0	24.1796310	120.6610279	https://www.inaturalist.org/observations/139211145
3936	Bubulcus ibis	Cattle Egret	2020/4/26	3	0	24.1799789	120.6291647	https://www.inaturalist.org/observations/43518404
3937	Bubulcus ibis	Cattle Egret	2022/10/25	1	0	24.1800217	120.6556217	https://www.inaturalist.org/observations/139948310
3938	Bubulcus ibis	Cattle Egret	2022/10/25	2	0	24.1801794	120.6555971	https://www.inaturalist.org/observations/139948013
3939	Bubulcus ibis	Cattle Egret	2021/5/11	1	0	24.1801867	120.6015550	https://www.inaturalist.org/observations/78959268
3940	Bubulcus ibis	Cattle Egret	2022/11/1	2	0	24.1804667	120.6549883	https://www.inaturalist.org/observations/140847351
3941	Bubulcus ibis	Cattle Egret	2022/5/10	2	0	24.1805467	120.5995554	https://www.inaturalist.org/observations/116357882
3942	Bubulcus ibis	Cattle Egret	2022/11/1	2	0	24.1806037	120.6557625	https://www.inaturalist.org/observations/140847246
3943	Bubulcus ibis	Cattle Egret	2019/6/6	2	0	24.1807356	120.6093388	https://www.inaturalist.org/observations/26475816
3944	Bubulcus ibis	Cattle Egret	2022/11/1	2	0	24.1809328	120.6555766	https://www.inaturalist.org/observations/140847383
3945	Bubulcus ibis	Cattle Egret	2021/11/5	1	0	24.1810543	120.9835511	https://www.inaturalist.org/observations/100286718
3946	Bubulcus ibis	Cattle Egret	2022/11/1	1	0	24.1810955	120.6550098	https://www.inaturalist.org/observations/140748240
3947	Bubulcus ibis	Cattle Egret	2022/11/1	2	0	24.1813486	120.6552266	https://www.inaturalist.org/observations/140847287
3948	Bubulcus ibis	Cattle Egret	2019/6/5	2	0	24.1815698	120.6020398	https://www.inaturalist.org/observations/26430177
3949	Bubulcus ibis	Cattle Egret	2022/5/2	1	0	24.1826867	121.4947133	https://www.inaturalist.org/observations/114571999

3950	Bubulcus ibis	Cattle Egret	2022/9/20	1	0	24.1841147	120.6551427	https://www.inaturalist.org/observations/135767049
3951	Bubulcus ibis	Cattle Egret	2022/9/20	2	0	24.1843186	120.6545890	https://www.inaturalist.org/observations/135743649
3952	Bubulcus ibis	Cattle Egret	2014/8/31	1	0	24.1844975	88.2849045	https://www.inaturalist.org/observations/93178985
3953	Bubulcus ibis	Cattle Egret	2022/10/18	2	0	24.1877435	120.6534075	https://www.inaturalist.org/observations/139203210
3954	Bubulcus ibis	Cattle Egret	2022/8/23	1	0	24.1878350	120.6532808	https://www.inaturalist.org/observations/131984253
3955	Bubulcus ibis	Cattle Egret	2022/8/23	1	0	24.1878994	120.6532682	https://www.inaturalist.org/observations/131980156
3956	Bubulcus ibis	Cattle Egret	2022/10/18	1	0	24.1882633	120.6523450	https://www.inaturalist.org/observations/139211067
3957	Bubulcus ibis	Cattle Egret	2022/10/18	2	0	24.1885439	120.6531870	https://www.inaturalist.org/observations/139211179
3958	Bubulcus ibis	Cattle Egret	2022/10/18	1	0	24.1890220	120.6537587	https://www.inaturalist.org/observations/139203219
3959	Bubulcus ibis	Cattle Egret	2022/10/18	1	0	24.1890332	120.6532740	https://www.inaturalist.org/observations/139203235
3960	Bubulcus ibis	Cattle Egret	2022/10/18	2	0	24.1891373	120.6535130	https://www.inaturalist.org/observations/139211199
3961	Bubulcus ibis	Cattle Egret	2022/10/18	1	0	24.1896939	120.6532128	https://www.inaturalist.org/observations/139210687
3962	Bubulcus ibis	Cattle Egret	2022/10/18	1	0	24.1898196	120.6535014	https://www.inaturalist.org/observations/139210721
3963	Bubulcus ibis	Cattle Egret	2022/10/18	1	0	24.1900950	120.6520117	https://www.inaturalist.org/observations/139210885
3964	Bubulcus ibis	Cattle Egret	2021/10/9	2	0	24.1916575	120.5518389	https://www.inaturalist.org/observations/99623784
3965	Bubulcus ibis	Cattle Egret	2020/6/1	2	0	24.2016457	120.8118214	https://www.inaturalist.org/observations/48076816
3966	Bubulcus ibis	Cattle Egret	2022/10/22	1	0	24.2058005	120.5977433	https://www.inaturalist.org/observations/139603045
3967	Bubulcus ibis	Cattle Egret	2022/10/22	1	0	24.2060645	120.5976291	https://www.inaturalist.org/observations/139600013
3968	Bubulcus ibis	Cattle Egret	2022/10/22	1	0	24.2062516	120.5973122	https://www.inaturalist.org/observations/139600003
3969	Bubulcus ibis	Cattle Egret	2019/1/11	4	0	24.2062519	120.8201742	https://www.inaturalist.org/observations/19634177
3970	Bubulcus ibis coromandus	Cattle Egret	2019/5/7	1	0	24.2062519	120.8201742	https://www.inaturalist.org/observations/27247714
3971	Bubulcus ibis coromandus	Cattle Egret	2020/4/24	1	0	24.2062519	120.8201742	https://www.inaturalist.org/observations/44088365
3972	Bubulcus ibis	Cattle Egret	2020/4/26	1	0	24.2062519	120.8201742	https://www.inaturalist.org/observations/44728339
3973	Bubulcus ibis	Cattle Egret	2020/9/9	2	0	24.2067533	120.5947867	https://www.inaturalist.org/observations/59133039
3974	Bubulcus ibis	Cattle Egret	2022/11/12	1	0	24.2067902	120.5985381	https://www.inaturalist.org/observations/141766215

3975	Bubulcus ibis	Cattle Egret	2022/10/23	1	0	24.2068481	120.5985619	https://www.inaturalist.org/observations/139711772
3976	Bubulcus ibis	Cattle Egret	2022/11/12	1	0	24.2070577	120.5984263	https://www.inaturalist.org/observations/141773180
3977	Bubulcus ibis	Cattle Egret	2019/10/27	1	0	24.2071233	120.5999282	https://www.inaturalist.org/observations/34936660
3978	Bubulcus ibis	Cattle Egret	2022/11/12	1	0	24.2072133	120.5985523	https://www.inaturalist.org/observations/141773188
3979	Bubulcus ibis	Cattle Egret	2022/11/12	1	0	24.2072467	120.5985981	https://www.inaturalist.org/observations/141766217
3980	Bubulcus ibis	Cattle Egret	2022/11/12	2	0	24.2072911	120.5985759	https://www.inaturalist.org/observations/141775816
3981	Bubulcus ibis	Cattle Egret	2022/11/27	1	0	24.2074073	120.5973030	https://www.inaturalist.org/observations/143162165
3982	Bubulcus ibis	Cattle Egret	2022/10/10	1	0	24.2074716	120.5987616	https://www.inaturalist.org/observations/138225629
3983	Bubulcus ibis	Cattle Egret	2022/11/27	2	0	24.2075945	120.5972135	https://www.inaturalist.org/observations/143021755
3984	Bubulcus ibis	Cattle Egret	2022/12/11	4	0	24.2076655	120.5978223	https://www.inaturalist.org/observations/144121568
3985	Bubulcus ibis	Cattle Egret	2022/5/26	1	0	24.2078321	120.5988161	https://www.inaturalist.org/observations/118748260
3986	Bubulcus ibis	Cattle Egret	2022/8/5	1	0	24.2078321	120.5988161	https://www.inaturalist.org/observations/129501238
3987	Bubulcus ibis	Cattle Egret	2022/7/17	1	0	24.2079413	120.5981886	https://www.inaturalist.org/observations/126623246
3988	Bubulcus ibis	Cattle Egret	2022/7/16	1	0	24.2079622	120.5980405	https://www.inaturalist.org/observations/126423351
3989	Bubulcus ibis	Cattle Egret	2022/10/10	0	0	24.2079716	120.5979084	https://www.inaturalist.org/observations/138202043
3990	Bubulcus ibis	Cattle Egret	2022/10/29	1	0	24.2079895	120.5966997	https://www.inaturalist.org/observations/140358607
3991	Bubulcus ibis	Cattle Egret	2022/7/1	1	0	24.2080583	120.5981000	https://www.inaturalist.org/observations/124186100
3992	Bubulcus ibis	Cattle Egret	2022/10/2	1	0	24.2080843	120.5978851	https://www.inaturalist.org/observations/137250537
3993	Bubulcus ibis	Cattle Egret	2022/10/1	1	0	24.2081450	120.5980550	https://www.inaturalist.org/observations/137220258
3994	Bubulcus ibis	Cattle Egret	2022/11/20	2	0	24.2081516	120.5972482	https://www.inaturalist.org/observations/142439688
3995	Bubulcus ibis	Cattle Egret	2022/4/25	1	0	24.2081785	120.5978506	https://www.inaturalist.org/observations/112783164
3996	Bubulcus ibis	Cattle Egret	2022/8/17	1	0	24.2082002	120.5932740	https://www.inaturalist.org/observations/139623762
3997	Bubulcus ibis	Cattle Egret	2019/5/25	3	0	24.2082140	120.5965686	https://www.inaturalist.org/observations/25741579
3998	Bubulcus ibis	Cattle Egret	2022/6/25	1	0	24.2082896	120.5985684	https://www.inaturalist.org/observations/123338062
3999	Bubulcus ibis	Cattle Egret	2022/10/26	1	0	24.2085596	120.5976717	https://www.inaturalist.org/observations/140034394

4000	Bubulcus ibis	Cattle Egret	2021/10/2	3	0	24.2086053	120.5991929	https://www.inaturalist.org/observations/120287189
4001	Bubulcus ibis	Cattle Egret	2022/6/4	1	0	24.2086138	120.5986067	https://www.inaturalist.org/observations/120111110
4002	Bubulcus ibis	Cattle Egret	2022/6/4	1	0	24.2086184	120.5986361	https://www.inaturalist.org/observations/120113029
4003	Bubulcus ibis	Cattle Egret	2022/11/20	2	0	24.2086571	120.5978084	https://www.inaturalist.org/observations/142439677
4004	Bubulcus ibis	Cattle Egret	2021/4/17	1	0	24.2087295	120.5978198	https://www.inaturalist.org/observations/94617035
4005	Bubulcus ibis	Cattle Egret	2022/10/26	2	0	24.2087353	120.5978423	https://www.inaturalist.org/observations/140029602
4006	Bubulcus ibis	Cattle Egret	2022/7/9	1	0	24.2087391	120.5986415	https://www.inaturalist.org/observations/125393459
4007	Bubulcus ibis	Cattle Egret	2022/10/1	1	0	24.2087444	120.5987482	https://www.inaturalist.org/observations/137215350
4008	Bubulcus ibis	Cattle Egret	2022/11/20	3	0	24.2087508	120.5988159	https://www.inaturalist.org/observations/142530786
4009	Bubulcus ibis	Cattle Egret	2021/10/23	2	0	24.2088797	120.5986322	https://www.inaturalist.org/observations/120297857
4010	Bubulcus ibis	Cattle Egret	2019/10/20	2	0	24.2089020	120.5956749	https://www.inaturalist.org/observations/34676907
4011	Bubulcus ibis	Cattle Egret	2022/9/24	1	0	24.2089083	120.5991550	https://www.inaturalist.org/observations/136230054
4012	Bubulcus ibis	Cattle Egret	2022/9/24	1	0	24.2089335	120.5991839	https://www.inaturalist.org/observations/136229930
4013	Bubulcus ibis	Cattle Egret	2022/10/1	1	0	24.2089383	120.5993417	https://www.inaturalist.org/observations/137097015
4014	Bubulcus ibis	Cattle Egret	2022/10/29	1	0	24.2090087	120.5977006	https://www.inaturalist.org/observations/140356869
4015	Bubulcus ibis	Cattle Egret	2022/9/4	1	0	24.2090248	120.5989829	https://www.inaturalist.org/observations/133557648
4016	Bubulcus ibis	Cattle Egret	2022/9/24	2	0	24.2092790	120.5995190	https://www.inaturalist.org/observations/136230689
4017	Bubulcus ibis	Cattle Egret	2022/10/23	2	0	24.2092798	120.5988882	https://www.inaturalist.org/observations/139730228
4018	Bubulcus ibis	Cattle Egret	2022/10/23	2	0	24.2093229	120.5995459	https://www.inaturalist.org/observations/139746264
4019	Bubulcus ibis	Cattle Egret	2022/7/16	1	0	24.2093318	120.5975515	https://www.inaturalist.org/observations/126420876
4020	Bubulcus ibis	Cattle Egret	2022/10/23	2	0	24.2095033	120.5988333	https://www.inaturalist.org/observations/139816741
4021	Bubulcus ibis	Cattle Egret	2022/10/23	3	0	24.2095108	120.5987332	https://www.inaturalist.org/observations/139711586
4022	Bubulcus ibis	Cattle Egret	2021/11/5	1	0	24.2095968	120.6020720	https://www.inaturalist.org/observations/102362081
4023	Bubulcus ibis	Cattle Egret	2022/1/31	1	0	24.2099682	120.5980186	https://www.inaturalist.org/observations/116561625
4024	Bubulcus ibis	Cattle Egret	2021/4/24	1	0	24.2104466	120.5976708	https://www.inaturalist.org/observations/119199132

4025	Bubulcus ibis	Cattle Egret	2019/8/20	1	0	24.2105774	120.5955653	https://www.inaturalist.org/observations/33520156
4026	Bubulcus ibis	Cattle Egret	2018/6/27	2	0	24.2247970	120.7021211	https://www.inaturalist.org/observations/17606720
4027	Bubulcus ibis	Cattle Egret	2022/4/21	3	0	24.2379718	121.3882545	https://www.inaturalist.org/observations/116455124
4028	Bubulcus ibis	Cattle Egret	2022/5/24	1	0	24.2401887	120.7955177	https://www.inaturalist.org/observations/118513287
4029	Bubulcus ibis	Cattle Egret	2022/5/24	1	0	24.2402484	120.7956771	https://www.inaturalist.org/observations/118513094
4030	Bubulcus ibis	Cattle Egret	2019/8/10	2	0	24.2414588	120.6613676	https://www.inaturalist.org/observations/30522611
4031	Bubulcus ibis coromandus	Cattle Egret	2021/7/18	0	0	24.2425500	120.6769167	https://www.inaturalist.org/observations/87682546
4032	Bubulcus ibis	Cattle Egret	2019/11/8	1	0	24.2647983	120.8213817	https://www.inaturalist.org/observations/35489031
4033	Bubulcus ibis	Cattle Egret	2022/9/5	2	0	24.2657950	120.6052283	https://www.inaturalist.org/observations/133862366
4034	Bubulcus ibis	Cattle Egret	2022/7/23	1	0	24.2665793	120.9569926	https://www.inaturalist.org/observations/134813752
4035	Bubulcus ibis	Cattle Egret	2022/7/23	2	0	24.2923343	120.8969776	https://www.inaturalist.org/observations/134813748
4036	Bubulcus ibis	Cattle Egret	2022/4/21	2	0	24.2983163	121.3087099	https://www.inaturalist.org/observations/116455126
4037	Bubulcus ibis	Cattle Egret	2020/7/2	1	0	24.3047500	120.5480967	https://www.inaturalist.org/observations/51835484
4038	Bubulcus ibis	Cattle Egret	2019/7/26	3	0	24.3058796	120.6457694	https://www.inaturalist.org/observations/31219802
4039	Bubulcus ibis	Cattle Egret	2022/4/21	2	0	24.3081882	121.3131106	https://www.inaturalist.org/observations/116455138
4040	Bubulcus ibis	Cattle Egret	2018/4/4	1	0	24.3129340	120.5459347	https://www.inaturalist.org/observations/51749863
4041	Bubulcus ibis	Cattle Egret	2021/6/13	3	0	24.3150717	120.7310400	https://www.inaturalist.org/observations/83354862
4042	Bubulcus ibis	Cattle Egret	2022/6/21	0	0	24.3250675	120.5569917	https://www.inaturalist.org/observations/123155411
4043	Bubulcus ibis	Cattle Egret	2022/6/21	1	0	24.3257541	120.5568145	https://www.inaturalist.org/observations/122921080
4044	Bubulcus ibis	Cattle Egret	2022/7/23	1	0	24.3278673	120.8982551	https://www.inaturalist.org/observations/134813754
4045	Bubulcus ibis	Cattle Egret	2022/12/20	1	0	24.3458567	120.6723500	https://www.inaturalist.org/observations/145190498
4046	Bubulcus ibis	Cattle Egret	2018/12/2	6	0	24.3526742	92.3448154	https://www.inaturalist.org/observations/19982622
4047	Bubulcus ibis	Cattle Egret	2019/2/8	4	0	24.3526742	92.3448154	https://www.inaturalist.org/observations/20444517
4048	Bubulcus ibis	Cattle Egret	2019/2/8	4	0	24.3526742	92.3448154	https://www.inaturalist.org/observations/20444523
4049	Bubulcus ibis	Cattle Egret	2019/2/8	5	0	24.3526742	92.3448154	https://www.inaturalist.org/observations/20444597

4050	Bubulcus ibis	Cattle Egret	2019/2/8	5	0	24.3526742	92.3448154	https://www.inaturalist.org/observations/20444601
4051	Bubulcus ibis	Cattle Egret	2021/12/3	2	0	24.3526742	92.3448154	https://www.inaturalist.org/observations/104960841
4052	Bubulcus ibis	Cattle Egret	2020/9/20	2	0	24.3526778	92.3448139	https://www.inaturalist.org/observations/64098350
4053	Bubulcus ibis	Cattle Egret	2021/4/16	1	0	24.3526803	92.3448153	https://www.inaturalist.org/observations/81702931
4054	Bubulcus ibis	Cattle Egret	2018/11/14	6	0	24.3528077	92.3446028	https://www.inaturalist.org/observations/19413597
4055	Bubulcus ibis	Cattle Egret	2018/3/23	4	0	24.3543130	92.3528410	https://www.inaturalist.org/observations/13604523
4056	Bubulcus ibis	Cattle Egret	2022/9/16	2	0	24.3608599	120.9495181	https://www.inaturalist.org/observations/138675519
4057	Bubulcus ibis	Cattle Egret	2022/4/21	3	0	24.3652564	121.3354313	https://www.inaturalist.org/observations/116455123
4058	Bubulcus ibis	Cattle Egret	2022/4/3	1	0	24.3784603	120.6585700	https://www.inaturalist.org/observations/110268377
4059	Bubulcus ibis	Cattle Egret	2022/3/16	2	0	24.4003343	120.6743998	https://www.inaturalist.org/observations/108795792
4060	Bubulcus ibis	Cattle Egret	2021/9/14	1	0	24.4084633	118.2962800	https://www.inaturalist.org/observations/95123788
4061	Bubulcus ibis	Cattle Egret	2018/11/30	4	0	24.4245041	120.7760217	https://www.inaturalist.org/observations/30656832
4062	Bubulcus ibis	Cattle Egret	2018/6/3	4	0	24.4271460	92.3366300	https://www.inaturalist.org/observations/14538026
4063	Bubulcus ibis	Cattle Egret	2019/4/15	1	0	24.4279737	118.2351192	https://www.inaturalist.org/observations/69449197
4064	Bubulcus ibis	Cattle Egret	2020/2/11	2	0	24.4281351	92.3354226	https://www.inaturalist.org/observations/46470123
4065	Bubulcus ibis	Cattle Egret	2020/2/12	2	0	24.4281351	92.3354226	https://www.inaturalist.org/observations/46567678
4066	Bubulcus ibis	Cattle Egret	2020/3/8	1	0	24.4294076	92.3333012	https://www.inaturalist.org/observations/39793389
4067	Bubulcus ibis	Cattle Egret	2018/1/30	4	0	24.4298200	92.3332290	https://www.inaturalist.org/observations/13604512
4068	Bubulcus ibis	Cattle Egret	2018/1/30	4	0	24.4319100	92.3308080	https://www.inaturalist.org/observations/13252270
4069	Bubulcus ibis	Cattle Egret	2019/5/26	5	0	24.4320197	92.3306894	https://www.inaturalist.org/observations/26659181
4070	Bubulcus ibis	Cattle Egret	2010/3/31	2	0	24.4320200	92.3306890	https://www.inaturalist.org/observations/103465503
4071	Bubulcus ibis	Cattle Egret	2010/6/7	2	0	24.4320200	92.3306890	https://www.inaturalist.org/observations/103466137
4072	Bubulcus ibis	Cattle Egret	2010/8/24	1	0	24.4320200	92.3306890	https://www.inaturalist.org/observations/103510271
4073	Bubulcus ibis	Cattle Egret	2010/12/3	2	0	24.4320200	92.3306890	https://www.inaturalist.org/observations/103620966
4074	Bubulcus ibis	Cattle Egret	2010/12/27	2	0	24.4320200	92.3306890	https://www.inaturalist.org/observations/103622192

4075	Bubulcus ibis	Cattle Egret	2019/2/6	2	0	24.4324204	120.6216558	https://www.inaturalist.org/observations/20152869
4076	Bubulcus ibis	Cattle Egret	2020/5/23	1	0	24.4330273	92.3337174	https://www.inaturalist.org/observations/46969647
4077	Bubulcus ibis	Cattle Egret	2019/9/26	4	0	24.4338515	92.3284793	https://www.inaturalist.org/observations/34063096
4078	Bubulcus ibis	Cattle Egret	2020/9/22	1	0	24.4460215	118.2475554	https://www.inaturalist.org/observations/63295906
4079	Bubulcus ibis	Cattle Egret	2022/10/7	1	0	24.4475789	120.6415303	https://www.inaturalist.org/observations/137825994
4080	Bubulcus ibis	Cattle Egret	2022/9/11	2	0	24.4478281	120.8812254	https://www.inaturalist.org/observations/134540641
4081	Bubulcus ibis	Cattle Egret	2022/9/11	2	0	24.4483711	120.8810440	https://www.inaturalist.org/observations/134540705
4082	Bubulcus ibis	Cattle Egret	2015/12/30	2	0	24.4484060	82.9790306	https://www.inaturalist.org/observations/59107310
4083	Bubulcus ibis	Cattle Egret	2022/9/13	2	0	24.4486917	120.6430117	https://www.inaturalist.org/observations/134832980
4084	Bubulcus ibis	Cattle Egret	2011/6/2	1	0	24.4488116	118.3738469	https://www.inaturalist.org/observations/55863919
4085	Bubulcus ibis	Cattle Egret	2021/5/3	2	0	24.4523840	118.4402000	https://www.inaturalist.org/observations/79461270
4086	Bubulcus ibis	Cattle Egret	2022/10/7	2	0	24.4535503	120.6508775	https://www.inaturalist.org/observations/137826006
4087	Bubulcus ibis	Cattle Egret	2022/9/13	1	0	24.4557517	120.6489083	https://www.inaturalist.org/observations/134832961
4088	Bubulcus ibis	Cattle Egret	2021/10/24	3	0	24.4566498	118.0913079	https://www.inaturalist.org/observations/99229027
4089	Bubulcus ibis	Cattle Egret	2021/12/1	1	0	24.4602133	121.7930267	https://www.inaturalist.org/observations/104697600
4090	Bubulcus ibis	Cattle Egret	2018/2/6	1	0	24.4612517	121.8058110	https://www.inaturalist.org/observations/137514449
4091	Bubulcus ibis	Cattle Egret	2020/4/27	1	0	24.4659020	121.8018680	https://www.inaturalist.org/observations/44235201
4092	Bubulcus ibis	Cattle Egret	2020/4/27	1	0	24.4659020	121.8018680	https://www.inaturalist.org/observations/44235214
4093	Bubulcus ibis	Cattle Egret	2020/4/27	2	0	24.4659020	121.8018680	https://www.inaturalist.org/observations/44235254
4094	Bubulcus ibis	Cattle Egret	2020/4/27	1	0	24.4659020	121.8018680	https://www.inaturalist.org/observations/44235255
4095	Bubulcus ibis	Cattle Egret	2020/4/27	1	0	24.4659020	121.8018680	https://www.inaturalist.org/observations/44235256
4096	Bubulcus ibis	Cattle Egret	2020/4/27	2	0	24.4659020	121.8018680	https://www.inaturalist.org/observations/44235265
4097	Bubulcus ibis	Cattle Egret	2018/12/29	3	0	24.4757656	118.4563815	https://www.inaturalist.org/observations/19330585
4098	Bubulcus ibis	Cattle Egret	2011/3/20	2	0	24.4796320	92.3240080	https://www.inaturalist.org/observations/103684120
4099	Bubulcus ibis	Cattle Egret	2011/8/14	2	0	24.4796320	92.3240080	https://www.inaturalist.org/observations/103694248

4100	Bubulcus ibis	Cattle Egret	2018/8/15	4	0	24.4809080	92.3230080	https://www.inaturalist.org/observations/15604733
4101	Bubulcus ibis	Cattle Egret	2019/5/18	1	0	24.4825014	118.4362529	https://www.inaturalist.org/observations/25554642
4102	Bubulcus ibis	Cattle Egret	2020/2/9	3	0	24.4829440	92.2907123	https://www.inaturalist.org/observations/46468684
4103	Bubulcus ibis	Cattle Egret	2020/3/1	4	0	24.4829440	92.2907123	https://www.inaturalist.org/observations/46705500
4104	Bubulcus ibis	Cattle Egret	2020/3/1	4	0	24.4829440	92.2907123	https://www.inaturalist.org/observations/46705503
4105	Bubulcus ibis	Cattle Egret	2017/2/6	1	0	24.4850581	118.4075186	https://www.inaturalist.org/observations/57000434
4106	Bubulcus ibis	Cattle Egret	2021/2/21	3	0	24.4965459	92.3143072	https://www.inaturalist.org/observations/70075040
4107	Bubulcus ibis	Cattle Egret	2021/10/24	1	0	24.4977727	92.3147675	https://www.inaturalist.org/observations/102744497
4108	Bubulcus ibis	Cattle Egret	2020/3/1	4	0	24.4978148	92.3147678	https://www.inaturalist.org/observations/39439283
4109	Bubulcus ibis	Cattle Egret	2019/3/14	1	0	24.4996750	120.7790900	https://www.inaturalist.org/observations/62184163
4110	Bubulcus ibis	Cattle Egret	2021/4/25	3	0	24.5042400	120.8303317	https://www.inaturalist.org/observations/75155567
4111	Bubulcus ibis	Cattle Egret	2019/9/22	1	0	24.5156554	118.1744357	https://www.inaturalist.org/observations/62694136
4112	Bubulcus ibis	Cattle Egret	2019/10/3	3	0	24.5195610	73.7380134	https://www.inaturalist.org/observations/33785276
4113	Bubulcus ibis	Cattle Egret	2022/5/7	2	0	24.5460992	120.7919511	https://www.inaturalist.org/observations/115909209
4114	Bubulcus ibis	Cattle Egret	2021/4/30	1	0	24.5516250	118.2320520	https://www.inaturalist.org/observations/83479782
4115	Bubulcus ibis	Cattle Egret	2017/5/28	4	0	24.5743112	121.1057378	https://www.inaturalist.org/observations/29490025
4116	Bubulcus ibis	Cattle Egret	2019/12/26	0	0	24.5797039	73.6806226	https://www.inaturalist.org/observations/37721144
4117	Bubulcus ibis coromandus	Cattle Egret	2019/7/6	2	0	24.5918805	74.1064635	https://www.inaturalist.org/observations/31905258
4118	Bubulcus ibis	Cattle Egret	2010/5/15	5	0	24.5934510	73.6672350	http://www.inaturalist.org/observations/321893
4119	Bubulcus ibis	Cattle Egret	2022/4/4	1	0	24.5948033	121.8442467	https://www.inaturalist.org/observations/110375826
4120	Bubulcus ibis	Cattle Egret	2019/4/13	3	0	24.6030127	73.6858378	https://www.inaturalist.org/observations/42079237
4121	Bubulcus ibis	Cattle Egret	2020/2/29	1	0	24.6044159	120.8261776	https://www.inaturalist.org/observations/41229824
4122	Bubulcus ibis coromandus	Cattle Egret	2012/8/19	3	0	24.6072879	92.3834412	http://www.inaturalist.org/observations/115298
4123	Bubulcus ibis	Cattle Egret	2012/1/1	4	0	24.6072880	92.3834410	http://www.inaturalist.org/observations/409336
4124	Bubulcus ibis	Cattle Egret	2021/10/9	1	0	24.6099284	121.8377710	https://www.inaturalist.org/observations/97598332

4125	Bubulcus ibis	Cattle Egret	2021/10/9	2	0	24.6099591	121.8378160	https://www.inaturalist.org/observations/97598807
4126	Bubulcus ibis	Cattle Egret	2021/10/9	2	0	24.6100185	121.8377649	https://www.inaturalist.org/observations/97599162
4127	Bubulcus ibis	Cattle Egret	2021/9/14	1	0	24.6103207	121.8413917	https://www.inaturalist.org/observations/94770385
4128	Bubulcus ibis	Cattle Egret	2021/9/9	2	0	24.6105377	121.8403946	https://www.inaturalist.org/observations/94171977
4129	Bubulcus ibis	Cattle Egret	2021/8/29	1	0	24.6109380	121.8404580	https://www.inaturalist.org/observations/92865143
4130	Bubulcus ibis	Cattle Egret	2021/8/29	1	0	24.6110020	121.8411154	https://www.inaturalist.org/observations/92865139
4131	Bubulcus ibis	Cattle Egret	2021/10/10	1	0	24.6123474	121.8433470	https://www.inaturalist.org/observations/97733090
4132	Bubulcus ibis	Cattle Egret	2022/6/3	2	0	24.6142767	121.8551983	https://www.inaturalist.org/observations/120300893
4133	Bubulcus ibis	Cattle Egret	2021/10/10	1	0	24.6147506	121.8452638	https://www.inaturalist.org/observations/97734025
4134	Bubulcus ibis	Cattle Egret	2022/1/12	1	0	24.6219311	121.8012218	https://www.inaturalist.org/observations/106914145
4135	Bubulcus ibis	Cattle Egret	2022/11/9	1	0	24.6238140	121.8413320	https://www.inaturalist.org/observations/143962469
4136	Bubulcus ibis	Cattle Egret	2022/5/5	1	0	24.6239264	121.7935293	https://www.inaturalist.org/observations/123206679
4137	Bubulcus ibis	Cattle Egret	2022/4/3	3	0	24.6242336	121.7527406	https://www.inaturalist.org/observations/138677877
4138	Bubulcus ibis	Cattle Egret	2022/8/4	1	0	24.6267466	121.8452110	https://www.inaturalist.org/observations/144241740
4139	Bubulcus ibis	Cattle Egret	2021/10/17	1	0	24.6294065	121.7892717	https://www.inaturalist.org/observations/102230645
4140	Bubulcus ibis	Cattle Egret	2020/4/24	2	0	24.6304040	121.7496000	https://www.inaturalist.org/observations/43098802
4141	Bubulcus ibis	Cattle Egret	2020/6/10	2	0	24.6313822	121.7606081	https://www.inaturalist.org/observations/49106138
4142	Bubulcus ibis	Cattle Egret	2021/4/14	1	0	24.6314784	121.7602775	https://www.inaturalist.org/observations/75025834
4143	Bubulcus ibis	Cattle Egret	2021/5/18	2	0	24.6315408	121.7604956	https://www.inaturalist.org/observations/79218352
4144	Bubulcus ibis	Cattle Egret	2021/4/17	1	0	24.6316102	121.7614797	https://www.inaturalist.org/observations/74311591
4145	Bubulcus ibis	Cattle Egret	2020/5/9	1	0	24.6319190	121.7537404	https://www.inaturalist.org/observations/54501611
4146	Bubulcus ibis	Cattle Egret	2022/6/15	3	0	24.6335498	121.7617958	https://www.inaturalist.org/observations/121841726
4147	Bubulcus ibis	Cattle Egret	2020/5/15	2	0	24.6335976	121.7617398	https://www.inaturalist.org/observations/45937748
4148	Bubulcus ibis	Cattle Egret	2021/4/26	1	0	24.6375111	121.7908857	https://www.inaturalist.org/observations/75293845
4149	Bubulcus ibis	Cattle Egret	2020/9/8	1	0	24.6395573	121.7803942	https://www.inaturalist.org/observations/59025771

4150	Bubulcus ibis	Cattle Egret	2022/5/15	2	0	24.6436056	121.5966083	https://www.inaturalist.org/observations/117114447
4151	Bubulcus ibis	Cattle Egret	2022/10/15	2	0	24.6436194	121.5967139	https://www.inaturalist.org/observations/138795322
4152	Bubulcus ibis	Cattle Egret	2020/8/22	1	0	24.6444467	121.7740243	https://www.inaturalist.org/observations/58128433
4153	Bubulcus ibis	Cattle Egret	2022/8/24	1	0	24.6502538	121.8247537	https://www.inaturalist.org/observations/144555443
4154	Bubulcus ibis	Cattle Egret	2022/9/7	2	0	24.6505683	121.8257673	https://www.inaturalist.org/observations/144492530
4155	Bubulcus ibis coromandus	Cattle Egret	2021/5/1	1	0	24.6522354	121.6460088	https://www.inaturalist.org/observations/77212607
4156	Bubulcus ibis	Cattle Egret	2021/7/23	1	0	24.6522438	121.8142176	https://www.inaturalist.org/observations/92549963
4157	Bubulcus ibis	Cattle Egret	2019/4/28	2	0	24.6524678	121.8227495	https://www.inaturalist.org/observations/23594143
4158	Bubulcus ibis	Cattle Egret	2021/5/1	1	0	24.6529257	121.6454434	https://www.inaturalist.org/observations/94706001
4159	Bubulcus ibis	Cattle Egret	2022/4/26	1	0	24.6541935	121.8236600	https://www.inaturalist.org/observations/123202941
4160	Bubulcus ibis	Cattle Egret	2021/9/13	1	0	24.6556895	121.8166933	https://www.inaturalist.org/observations/100860445
4161	Bubulcus ibis	Cattle Egret	2018/8/31	3	0	24.6562932	121.8196613	https://www.inaturalist.org/observations/19888605
4162	Bubulcus ibis	Cattle Egret	2020/5/13	1	0	24.6565435	121.7508923	https://www.inaturalist.org/observations/47072458
4163	Bubulcus ibis	Cattle Egret	2020/10/25	1	0	24.6566351	121.7284344	https://www.inaturalist.org/observations/65139449
4164	Bubulcus ibis	Cattle Egret	2019/4/28	2	0	24.6566904	121.8138408	https://www.inaturalist.org/observations/23589118
4165	Bubulcus ibis	Cattle Egret	2019/4/28	2	0	24.6566904	121.8138408	https://www.inaturalist.org/observations/23589121
4166	Bubulcus ibis	Cattle Egret	2019/4/28	2	0	24.6566904	121.8138408	https://www.inaturalist.org/observations/23589122
4167	Bubulcus ibis	Cattle Egret	2019/4/28	2	0	24.6571840	121.8215950	https://www.inaturalist.org/observations/23658765
4168	Bubulcus ibis	Cattle Egret	2019/4/28	2	0	24.6578801	121.8213201	https://www.inaturalist.org/observations/23534862
4169	Bubulcus ibis	Cattle Egret	2020/4/25	2	0	24.6591400	121.0230630	https://www.inaturalist.org/observations/43274531
4170	Bubulcus ibis	Cattle Egret	2020/7/16	1	0	24.6592873	121.8205473	https://www.inaturalist.org/observations/54424673
4171	Bubulcus ibis	Cattle Egret	2020/7/8	1	0	24.6599954	121.8194148	https://www.inaturalist.org/observations/53481995
4172	Bubulcus ibis	Cattle Egret	2020/4/25	2	0	24.6615049	121.6189375	https://www.inaturalist.org/observations/43286613
4173	Bubulcus ibis	Cattle Egret	2022/8/5	3	0	24.6624387	121.8170625	https://www.inaturalist.org/observations/131532169
4174	Bubulcus ibis	Cattle Egret	2018/10/22	2	0	24.6628246	121.8164544	https://www.inaturalist.org/observations/17739209

4175	Bubulcus ibis	Cattle Egret	2019/4/28	1	0	24.6632325	121.8178269	https://www.inaturalist.org/observations/23582480
4176	Bubulcus ibis	Cattle Egret	2019/9/23	2	0	24.6632325	121.8178269	https://www.inaturalist.org/observations/33250141
4177	Bubulcus ibis	Cattle Egret	2019/9/23	1	0	24.6632325	121.8178269	https://www.inaturalist.org/observations/33250156
4178	Bubulcus ibis	Cattle Egret	2019/9/23	2	0	24.6632325	121.8178269	https://www.inaturalist.org/observations/33250157
4179	Bubulcus ibis	Cattle Egret	2019/9/23	2	0	24.6632325	121.8178269	https://www.inaturalist.org/observations/33250708
4180	Bubulcus ibis	Cattle Egret	2020/3/23	2	0	24.6638197	121.8127244	https://www.inaturalist.org/observations/40638325
4181	Bubulcus ibis	Cattle Egret	2021/9/24	1	0	24.6654654	121.8142605	https://www.inaturalist.org/observations/101402132
4182	Bubulcus ibis	Cattle Egret	2022/5/2	1	0	24.6656950	121.8128683	https://www.inaturalist.org/observations/115564213
4183	Bubulcus ibis	Cattle Egret	2018/8/20	3	0	24.6658455	121.8189427	https://www.inaturalist.org/observations/15717668
4184	Bubulcus ibis	Cattle Egret	2022/5/1	1	0	24.6659375	121.8138243	https://www.inaturalist.org/observations/115536695
4185	Bubulcus ibis	Cattle Egret	2021/12/13	3	0	24.6662274	121.7937469	https://www.inaturalist.org/observations/102981535
4186	Bubulcus ibis	Cattle Egret	2019/12/19	4	0	24.6666943	77.1464197	https://www.inaturalist.org/observations/66196024
4187	Bubulcus ibis	Cattle Egret	2019/6/22	2	0	24.6679117	121.7608417	https://www.inaturalist.org/observations/27410798
4188	Bubulcus ibis	Cattle Egret	2022/12/25	2	0	24.6686770	121.6330285	https://www.inaturalist.org/observations/145156018
4189	Bubulcus ibis	Cattle Egret	2020/10/2	2	0	24.6694135	121.7336199	https://www.inaturalist.org/observations/61470851
4190	Bubulcus ibis	Cattle Egret	2020/10/2	2	0	24.6694135	121.7336199	https://www.inaturalist.org/observations/61470863
4191	Bubulcus ibis	Cattle Egret	2021/10/30	1	0	24.6704302	121.8130198	https://www.inaturalist.org/observations/103819775
4192	Bubulcus ibis	Cattle Egret	2018/6/18	1	0	24.6732772	121.6719047	https://www.inaturalist.org/observations/50844262
4193	Bubulcus ibis	Cattle Egret	2022/5/11	3	0	24.6735569	121.8168382	https://www.inaturalist.org/observations/116525587
4194	Bubulcus ibis	Cattle Egret	2021/2/7	1	0	24.6736550	121.8170017	https://www.inaturalist.org/observations/69195263
4195	Bubulcus ibis	Cattle Egret	2020/9/7	1	0	24.6768680	121.7443059	https://www.inaturalist.org/observations/58818933
4196	Bubulcus ibis	Cattle Egret	2017/6/26	2	0	24.6775439	121.7745553	https://www.inaturalist.org/observations/17406936
4197	Bubulcus ibis	Cattle Egret	2020/9/2	1	0	24.6787317	121.7464288	https://www.inaturalist.org/observations/58318412
4198	Bubulcus ibis	Cattle Egret	2021/6/11	1	0	24.6790577	121.7557202	https://www.inaturalist.org/observations/83908149
4199	Bubulcus ibis	Cattle Egret	2021/6/21	1	0	24.6797703	121.7554860	https://www.inaturalist.org/observations/84174497

4200	Bubulcus ibis	Cattle Egret	2021/5/29	1	0	24.6801029	121.7556522	https://www.inaturalist.org/observations/81846358
4201	Bubulcus ibis	Cattle Egret	2019/8/10	3	0	24.6813529	121.7535321	https://www.inaturalist.org/observations/30522227
4202	Bubulcus ibis	Cattle Egret	2022/4/28	1	0	24.6815024	121.7627584	https://www.inaturalist.org/observations/113195162
4203	Bubulcus ibis	Cattle Egret	2019/4/22	2	0	24.6827423	121.7735229	https://www.inaturalist.org/observations/23109615
4204	Bubulcus ibis	Cattle Egret	2021/8/13	1	0	24.6828222	121.7738528	https://www.inaturalist.org/observations/93011739
4205	Bubulcus ibis	Cattle Egret	2022/3/19	4	0	24.6828740	121.7508760	https://www.inaturalist.org/observations/109344912
4206	Bubulcus ibis	Cattle Egret	2020/5/9	1	0	24.6828842	121.7524759	https://www.inaturalist.org/observations/45322294
4207	Bubulcus ibis	Cattle Egret	2020/7/2	1	0	24.6829182	121.7531973	https://www.inaturalist.org/observations/56794235
4208	Bubulcus ibis	Cattle Egret	2020/7/2	1	0	24.6829182	121.7531973	https://www.inaturalist.org/observations/56794262
4209	Bubulcus ibis	Cattle Egret	2020/7/2	1	0	24.6829182	121.7531973	https://www.inaturalist.org/observations/56794265
4210	Bubulcus ibis	Cattle Egret	2020/5/5	2	0	24.6832316	121.7530948	https://www.inaturalist.org/observations/46459176
4211	Bubulcus ibis	Cattle Egret	2018/6/30	4	0	24.6833126	120.9328192	https://www.inaturalist.org/observations/18858034
4212	Bubulcus ibis	Cattle Egret	2020/2/26	0	0	24.6840994	121.7557775	https://www.inaturalist.org/observations/39524186
4213	Bubulcus ibis	Cattle Egret	2020/4/27	2	0	24.6842814	121.7723536	https://www.inaturalist.org/observations/44236301
4214	Bubulcus ibis	Cattle Egret	2020/4/27	2	0	24.6842814	121.7723536	https://www.inaturalist.org/observations/44236335
4215	Bubulcus ibis	Cattle Egret	2020/4/27	2	0	24.6842814	121.7723536	https://www.inaturalist.org/observations/44236355
4216	Bubulcus ibis	Cattle Egret	2020/4/27	2	0	24.6842814	121.7723536	https://www.inaturalist.org/observations/44236420
4217	Bubulcus ibis	Cattle Egret	2020/4/27	2	0	24.6842814	121.7723536	https://www.inaturalist.org/observations/44236435
4218	Bubulcus ibis	Cattle Egret	2020/4/27	2	0	24.6842814	121.7723536	https://www.inaturalist.org/observations/44236436
4219	Bubulcus ibis	Cattle Egret	2020/4/27	1	0	24.6842814	121.7723536	https://www.inaturalist.org/observations/44236441
4220	Bubulcus ibis	Cattle Egret	2020/4/27	2	0	24.6842814	121.7723536	https://www.inaturalist.org/observations/44236543
4221	Bubulcus ibis	Cattle Egret	2018/7/17	3	0	24.6853362	121.7739356	https://www.inaturalist.org/observations/14456809
4222	Bubulcus ibis	Cattle Egret	2022/4/2	2	0	24.6856050	121.7536083	https://www.inaturalist.org/observations/110148535
4223	Bubulcus ibis	Cattle Egret	2022/8/4	1	0	24.6856066	121.7538959	https://www.inaturalist.org/observations/134831667
4224	Bubulcus ibis	Cattle Egret	2017/5/21	3	0	24.6918694	121.0111344	https://www.inaturalist.org/observations/9255779

4225	Bubulcus ibis	Cattle Egret	2021/9/20	2	0	24.6940782	121.8221800	https://www.inaturalist.org/observations/95525025
4226	Bubulcus ibis	Cattle Egret	2021/9/20	2	0	24.6940782	121.8221800	https://www.inaturalist.org/observations/95525088
4227	Bubulcus ibis	Cattle Egret	2021/9/20	1	0	24.6940782	121.8221800	https://www.inaturalist.org/observations/95525117
4228	Bubulcus ibis	Cattle Egret	2021/9/20	2	0	24.6940782	121.8221800	https://www.inaturalist.org/observations/95525125
4229	Bubulcus ibis	Cattle Egret	2021/9/20	2	0	24.6940782	121.8221800	https://www.inaturalist.org/observations/95525130
4230	Bubulcus ibis	Cattle Egret	2021/9/20	2	0	24.6940782	121.8221800	https://www.inaturalist.org/observations/95525141
4231	Bubulcus ibis	Cattle Egret	2021/9/20	2	0	24.6940782	121.8221800	https://www.inaturalist.org/observations/95525184
4232	Bubulcus ibis	Cattle Egret	2021/9/20	2	0	24.6940782	121.8221800	https://www.inaturalist.org/observations/95525201
4233	Bubulcus ibis	Cattle Egret	2019/7/24	3	0	24.6960935	121.0996050	https://www.inaturalist.org/observations/29623551
4234	Bubulcus ibis	Cattle Egret	2019/7/24	2	0	24.6963254	121.0995266	https://www.inaturalist.org/observations/29622672
4235	Bubulcus ibis	Cattle Egret	2020/7/3	1	0	24.6969846	121.7636985	https://www.inaturalist.org/observations/53371814
4236	Bubulcus ibis	Cattle Egret	2020/9/10	1	0	24.6986096	121.7627180	https://www.inaturalist.org/observations/59114256
4237	Bubulcus ibis	Cattle Egret	2022/6/15	2	0	24.7021073	121.7377502	https://www.inaturalist.org/observations/121893459
4238	Bubulcus ibis	Cattle Egret	2022/6/15	1	0	24.7021073	121.7377502	https://www.inaturalist.org/observations/121991252
4239	Bubulcus ibis	Cattle Egret	2020/10/14	1	0	24.7036382	121.7553478	https://www.inaturalist.org/observations/62697660
4240	Bubulcus ibis	Cattle Egret	2020/4/24	2	0	24.7043771	121.1574586	https://www.inaturalist.org/observations/43223578
4241	Bubulcus ibis	Cattle Egret	2022/4/30	1	0	24.7044201	121.1553074	https://www.inaturalist.org/observations/113800000
4242	Bubulcus ibis	Cattle Egret	2022/5/1	1	0	24.7045882	121.1172828	https://www.inaturalist.org/observations/114073713
4243	Bubulcus ibis	Cattle Egret	2022/5/1	1	0	24.7047723	121.1169647	https://www.inaturalist.org/observations/115127775
4244	Bubulcus ibis	Cattle Egret	2020/10/7	1	0	24.7067950	121.7620910	https://www.inaturalist.org/observations/62481381
4245	Bubulcus ibis	Cattle Egret	2020/4/22	1	0	24.7068963	121.1566573	https://www.inaturalist.org/observations/44359385
4246	Bubulcus ibis coromandus	Cattle Egret	2022/5/21	2	0	24.7076629	121.8296195	https://www.inaturalist.org/observations/118152554
4247	Bubulcus ibis coromandus	Cattle Egret	2022/5/21	2	0	24.7076629	121.8296195	https://www.inaturalist.org/observations/118152556
4248	Bubulcus ibis	Cattle Egret	2020/6/27	1	0	24.7083633	121.7852550	https://www.inaturalist.org/observations/53609346
4249	Bubulcus ibis	Cattle Egret	2020/4/25	2	0	24.7092411	121.7228444	https://www.inaturalist.org/observations/43286767

4250	Bubulcus ibis	Cattle Egret	2020/4/25	1	0	24.7092411	121.7228444	https://www.inaturalist.org/observations/43286812
4251	Bubulcus ibis	Cattle Egret	2020/4/25	2	0	24.7092411	121.7228444	https://www.inaturalist.org/observations/43286841
4252	Bubulcus ibis	Cattle Egret	2021/5/1	1	0	24.7102779	121.7612015	https://www.inaturalist.org/observations/77632096
4253	Bubulcus ibis	Cattle Egret	2022/7/5	1	0	24.7116444	121.0008156	https://www.inaturalist.org/observations/124741676
4254	Bubulcus ibis coromandus	Cattle Egret	2021/3/17	1	0	24.7138583	121.6887500	https://www.inaturalist.org/observations/77715889
4255	Bubulcus ibis	Cattle Egret	2022/8/10	1	0	24.7173669	121.8152529	https://www.inaturalist.org/observations/143886964
4256	Bubulcus ibis	Cattle Egret	2021/6/14	2	0	24.7176056	121.1016389	https://www.inaturalist.org/observations/83630437
4257	Bubulcus ibis	Cattle Egret	2022/10/5	1	0	24.7181148	121.8154919	https://www.inaturalist.org/observations/143887019
4258	Bubulcus ibis	Cattle Egret	2020/4/22	2	0	24.7185273	121.1147496	https://www.inaturalist.org/observations/42827047
4259	Bubulcus ibis	Cattle Egret	2022/8/24	1	0	24.7190472	121.8157266	https://www.inaturalist.org/observations/143877952
4260	Bubulcus ibis	Cattle Egret	2022/8/10	1	0	24.7194791	121.8159126	https://www.inaturalist.org/observations/143877942
4261	Bubulcus ibis	Cattle Egret	2020/3/31	1	0	24.7202024	121.7144297	https://www.inaturalist.org/observations/41608199
4262	Bubulcus ibis	Cattle Egret	2019/1/21	2	0	24.7205496	121.8050125	https://www.inaturalist.org/observations/19869648
4263	Bubulcus ibis	Cattle Egret	2022/8/22	2	0	24.7207651	121.8078730	https://www.inaturalist.org/observations/141599271
4264	Bubulcus ibis	Cattle Egret	2021/8/26	1	0	24.7208872	121.7105954	https://www.inaturalist.org/observations/97226677
4265	Bubulcus ibis	Cattle Egret	2020/6/1	1	0	24.7209624	121.7351283	https://www.inaturalist.org/observations/48903818
4266	Bubulcus ibis	Cattle Egret	2020/4/25	3	0	24.7220146	121.6872714	https://www.inaturalist.org/observations/43306273
4267	Bubulcus ibis	Cattle Egret	2020/4/25	3	0	24.7220146	121.6872714	https://www.inaturalist.org/observations/43306275
4268	Bubulcus ibis	Cattle Egret	2020/4/25	3	0	24.7220146	121.6872714	https://www.inaturalist.org/observations/43306277
4269	Bubulcus ibis	Cattle Egret	2020/4/25	3	0	24.7220146	121.6872714	https://www.inaturalist.org/observations/43306298
4270	Bubulcus ibis	Cattle Egret	2022/9/5	2	0	24.7220483	121.8127031	https://www.inaturalist.org/observations/141599577
4271	Bubulcus ibis	Cattle Egret	2022/9/19	2	0	24.7221971	121.8126726	https://www.inaturalist.org/observations/141599996
4272	Bubulcus ibis	Cattle Egret	2022/8/9	2	0	24.7223078	121.8100134	https://www.inaturalist.org/observations/141599076
4273	Bubulcus ibis	Cattle Egret	2022/11/3	2	0	24.7224261	121.8081719	https://www.inaturalist.org/observations/141695615
4274	Bubulcus ibis	Cattle Egret	2022/8/7	0	0	24.7227130	121.8118950	https://www.inaturalist.org/observations/143983667

4275	Bubulcus ibis	Cattle Egret	2022/9/18	2	0	24.7227130	121.8118950	https://www.inaturalist.org/observations/143984310
4276	Bubulcus ibis	Cattle Egret	2022/8/29	2	0	24.7228739	121.8094982	https://www.inaturalist.org/observations/141599362
4277	Bubulcus ibis	Cattle Egret	2022/8/27	2	0	24.7232880	121.8107820	https://www.inaturalist.org/observations/144533000
4278	Bubulcus ibis	Cattle Egret	2022/9/3	2	0	24.7232880	121.8107820	https://www.inaturalist.org/observations/144533550
4279	Bubulcus ibis	Cattle Egret	2020/9/5	1	0	24.7240999	121.8205968	https://www.inaturalist.org/observations/60295708
4280	Bubulcus ibis	Cattle Egret	2021/2/4	2	0	24.7243301	121.6935466	https://www.inaturalist.org/observations/69077780
4281	Bubulcus ibis	Cattle Egret	2022/8/8	2	0	24.7254450	121.8124710	https://www.inaturalist.org/observations/144590355
4282	Bubulcus ibis	Cattle Egret	2022/6/4	1	0	24.7255233	121.7629500	https://www.inaturalist.org/observations/120146744
4283	Bubulcus ibis	Cattle Egret	2022/5/1	1	0	24.7268791	121.7451773	https://www.inaturalist.org/observations/114167200
4284	Bubulcus ibis	Cattle Egret	2022/5/1	2	0	24.7268791	121.7451773	https://www.inaturalist.org/observations/114167214
4285	Bubulcus ibis	Cattle Egret	2022/5/1	1	0	24.7268791	121.7451773	https://www.inaturalist.org/observations/114167219
4286	Bubulcus ibis	Cattle Egret	2022/11/13	2	0	24.7276752	121.7565281	https://www.inaturalist.org/observations/141879206
4287	Bubulcus ibis	Cattle Egret	2021/5/29	1	0	24.7276883	121.7636850	https://www.inaturalist.org/observations/80859893
4288	Bubulcus ibis	Cattle Egret	2022/8/29	1	0	24.7279150	121.8043950	https://www.inaturalist.org/observations/145183836
4289	Bubulcus ibis	Cattle Egret	2020/4/25	1	0	24.7288804	121.7447866	https://www.inaturalist.org/observations/43285252
4290	Bubulcus ibis	Cattle Egret	2022/10/4	2	0	24.7289650	121.8096300	https://www.inaturalist.org/observations/144465535
4291	Bubulcus ibis	Cattle Egret	2022/9/10	0	0	24.7292740	121.8034530	https://www.inaturalist.org/observations/145241801
4292	Bubulcus ibis	Cattle Egret	2022/9/10	0	0	24.7292740	121.8034530	https://www.inaturalist.org/observations/145241806
4293	Bubulcus ibis	Cattle Egret	2022/9/19	3	0	24.7301425	121.7447745	https://www.inaturalist.org/observations/139720355
4294	Bubulcus ibis	Cattle Egret	2022/9/19	3	0	24.7301425	121.7447745	https://www.inaturalist.org/observations/139720389
4295	Bubulcus ibis	Cattle Egret	2022/9/19	3	0	24.7301425	121.7447745	https://www.inaturalist.org/observations/139720404
4296	Bubulcus ibis	Cattle Egret	2022/9/19	3	0	24.7301425	121.7447745	https://www.inaturalist.org/observations/139720475
4297	Bubulcus ibis	Cattle Egret	2022/11/25	1	0	24.7301425	121.7447745	https://www.inaturalist.org/observations/142977680
4298	Bubulcus ibis	Cattle Egret	2022/10/14	1	0	24.7303089	121.7446929	https://www.inaturalist.org/observations/139703487
4299	Bubulcus ibis	Cattle Egret	2022/10/14	2	0	24.7303089	121.7446929	https://www.inaturalist.org/observations/139703530

4300	Bubulcus ibis	Cattle Egret	2022/8/8	2	0	24.7305430	121.8091720	https://www.inaturalist.org/observations/144344145
4301	Bubulcus ibis	Cattle Egret	2022/9/11	0	0	24.7314359	121.8044785	https://www.inaturalist.org/observations/134562294
4302	Bubulcus ibis	Cattle Egret	2020/4/19	1	0	24.7315067	121.7467983	https://www.inaturalist.org/observations/42562763
4303	Bubulcus ibis	Cattle Egret	2022/10/22	1	0	24.7315830	121.8087620	https://www.inaturalist.org/observations/144358064
4304	Bubulcus ibis	Cattle Egret	2022/8/27	2	0	24.7321480	121.8092070	https://www.inaturalist.org/observations/144342928
4305	Bubulcus ibis	Cattle Egret	2022/5/1	3	0	24.7326067	121.7612600	https://www.inaturalist.org/observations/116017812
4306	Bubulcus ibis	Cattle Egret	2022/11/11	1	0	24.7356906	121.7313630	https://www.inaturalist.org/observations/141771322
4307	Bubulcus ibis	Cattle Egret	2022/11/11	1	0	24.7356906	121.7313630	https://www.inaturalist.org/observations/141771324
4308	Bubulcus ibis	Cattle Egret	2021/1/1	2	0	24.7358688	121.7403967	https://www.inaturalist.org/observations/67390279
4309	Bubulcus ibis	Cattle Egret	2021/6/13	4	0	24.7375469	121.7387748	https://www.inaturalist.org/observations/83590890
4310	Bubulcus ibis	Cattle Egret	2021/6/13	4	0	24.7375469	121.7387748	https://www.inaturalist.org/observations/83590891
4311	Bubulcus ibis	Cattle Egret	2022/5/1	1	0	24.7381293	121.6625320	https://www.inaturalist.org/observations/115109092
4312	Bubulcus ibis	Cattle Egret	2022/9/17	2	0	24.7415130	121.8110750	https://www.inaturalist.org/observations/140851982
4313	Bubulcus ibis	Cattle Egret	2020/2/18	1	0	24.7423242	121.7520630	https://www.inaturalist.org/observations/58948189
4314	Bubulcus ibis	Cattle Egret	2022/10/14	1	0	24.7426501	121.7366867	https://www.inaturalist.org/observations/139606252
4315	Bubulcus ibis	Cattle Egret	2022/1/26	2	0	24.7427843	121.7376526	https://www.inaturalist.org/observations/105752898
4316	Bubulcus ibis	Cattle Egret	2021/7/26	2	0	24.7431958	121.7351417	https://www.inaturalist.org/observations/89575356
4317	Bubulcus ibis	Cattle Egret	2022/10/14	2	0	24.7434701	121.7340994	https://www.inaturalist.org/observations/139606992
4318	Bubulcus ibis	Cattle Egret	2022/9/10	2	0	24.7437540	121.6970210	https://www.inaturalist.org/observations/134455412
4319	Bubulcus ibis coromandus	Cattle Egret	2021/1/24	1	0	24.7437857	121.7371475	https://www.inaturalist.org/observations/68758447
4320	Bubulcus ibis	Cattle Egret	2021/7/23	2	0	24.7442204	121.7396494	https://www.inaturalist.org/observations/89465583
4321	Bubulcus ibis	Cattle Egret	2021/7/24	1	0	24.7443373	121.7395100	https://www.inaturalist.org/observations/89466320
4322	Bubulcus ibis	Cattle Egret	2021/7/24	2	0	24.7443373	121.7395100	https://www.inaturalist.org/observations/89466334
4323	Bubulcus ibis	Cattle Egret	2021/12/7	2	0	24.7449240	121.7207109	https://www.inaturalist.org/observations/104077180
4324	Bubulcus ibis	Cattle Egret	2021/12/12	2	0	24.7449240	121.7207109	https://www.inaturalist.org/observations/104077445

4325	Bubulcus ibis coromandus	Cattle Egret	2021/6/28	1	0	24.7452448	121.7325735	https://www.inaturalist.org/observations/87523949
4326	Bubulcus ibis	Cattle Egret	2022/6/14	1	0	24.7459275	121.7453209	https://www.inaturalist.org/observations/121704901
4327	Bubulcus ibis	Cattle Egret	2020/5/10	1	0	24.7462462	121.7499538	https://www.inaturalist.org/observations/46064296
4328	Bubulcus ibis	Cattle Egret	2020/5/10	1	0	24.7462462	121.7499538	https://www.inaturalist.org/observations/46064299
4329	Bubulcus ibis	Cattle Egret	2021/5/29	2	0	24.7475278	121.0870000	https://www.inaturalist.org/observations/80680493
4330	Bubulcus ibis	Cattle Egret	2019/9/14	1	0	24.7493605	121.6386489	https://www.inaturalist.org/observations/32753056
4331	Bubulcus ibis	Cattle Egret	2019/9/14	4	0	24.7493605	121.6386489	https://www.inaturalist.org/observations/32753058
4332	Bubulcus ibis	Cattle Egret	2022/7/4	4	0	24.7507701	84.3713374	https://www.inaturalist.org/observations/126072104
4333	Bubulcus ibis	Cattle Egret	2019/4/13	3	0	24.7510579	121.7217589	https://www.inaturalist.org/observations/22505627
4334	Bubulcus ibis	Cattle Egret	2019/10/13	2	0	24.7530067	121.6357350	https://www.inaturalist.org/observations/34292943
4335	Bubulcus ibis	Cattle Egret	2019/10/13	2	0	24.7530083	121.6357367	https://www.inaturalist.org/observations/34292941
4336	Bubulcus ibis	Cattle Egret	2019/10/13	2	0	24.7531283	121.6354600	https://www.inaturalist.org/observations/34292937
4337	Bubulcus ibis	Cattle Egret	2019/10/13	2	0	24.7531533	121.6355900	https://www.inaturalist.org/observations/34292939
4338	Bubulcus ibis	Cattle Egret	2019/10/13	2	0	24.7531650	121.6356183	https://www.inaturalist.org/observations/34292940
4339	Bubulcus ibis	Cattle Egret	2021/2/18	1	0	24.7538940	121.6357550	https://www.inaturalist.org/observations/69862343
4340	Bubulcus ibis	Cattle Egret	2022/2/26	2	0	24.7545174	121.7754512	https://www.inaturalist.org/observations/108792015
4341	Bubulcus ibis	Cattle Egret	2022/1/26	2	0	24.7562125	121.6488014	https://www.inaturalist.org/observations/105745200
4342	Bubulcus ibis	Cattle Egret	2020/4/27	1	0	24.7581248	121.2088363	https://www.inaturalist.org/observations/43800743
4343	Bubulcus ibis	Cattle Egret	2021/8/16	1	0	24.7623634	121.0560201	https://www.inaturalist.org/observations/91383453
4344	Bubulcus ibis	Cattle Egret	2021/8/16	2	0	24.7624269	121.0559546	https://www.inaturalist.org/observations/91379719
4345	Bubulcus ibis	Cattle Egret	2021/1/30	1	0	24.7625077	121.7502886	https://www.inaturalist.org/observations/69041417
4346	Bubulcus ibis	Cattle Egret	2022/9/13	1	0	24.7630000	121.7505717	https://www.inaturalist.org/observations/134835192
4347	Bubulcus ibis	Cattle Egret	2020/4/27	1	0	24.7672222	121.0255556	https://www.inaturalist.org/observations/43848274
4348	Bubulcus ibis	Cattle Egret	2022/10/25	3	0	24.7690246	121.6055112	https://www.inaturalist.org/observations/139938811
4349	Bubulcus ibis	Cattle Egret	2021/8/7	2	0	24.7695690	121.7993168	https://www.inaturalist.org/observations/137483639

4350	Bubulcus ibis	Cattle Egret	2021/8/7	3	0	24.7695690	121.7993168	https://www.inaturalist.org/observations/137483641
4351	Bubulcus ibis	Cattle Egret	2021/9/1	2	0	24.7695690	121.7993168	https://www.inaturalist.org/observations/137608033
4352	Bubulcus ibis	Cattle Egret	2021/9/29	2	0	24.7695690	121.7993168	https://www.inaturalist.org/observations/137609355
4353	Bubulcus ibis	Cattle Egret	2021/9/29	2	0	24.7695690	121.7993168	https://www.inaturalist.org/observations/137609356
4354	Bubulcus ibis	Cattle Egret	2021/9/29	2	0	24.7695690	121.7993168	https://www.inaturalist.org/observations/137609357
4355	Bubulcus ibis	Cattle Egret	2021/9/29	2	0	24.7695690	121.7993168	https://www.inaturalist.org/observations/137609358
4356	Bubulcus ibis	Cattle Egret	2020/5/2	2	0	24.7707520	121.0250341	https://www.inaturalist.org/observations/44557029
4357	Bubulcus ibis	Cattle Egret	2022/1/30	1	0	24.7722964	121.7777827	https://www.inaturalist.org/observations/110237360
4358	Bubulcus ibis	Cattle Egret	2020/5/12	1	0	24.7731693	121.0460689	https://www.inaturalist.org/observations/46195025
4359	Bubulcus ibis	Cattle Egret	2022/5/22	2	0	24.7798208	121.7174390	https://www.inaturalist.org/observations/118171582
4360	Bubulcus ibis	Cattle Egret	2022/5/22	2	0	24.7798208	121.7174390	https://www.inaturalist.org/observations/118171583
4361	Bubulcus ibis	Cattle Egret	2022/5/22	2	0	24.7798208	121.7174390	https://www.inaturalist.org/observations/118171584
4362	Bubulcus ibis	Cattle Egret	2022/5/22	2	0	24.7798208	121.7174390	https://www.inaturalist.org/observations/118171588
4363	Bubulcus ibis	Cattle Egret	2020/7/1	2	0	24.7843201	121.0001280	https://www.inaturalist.org/observations/51557369
4364	Bubulcus ibis	Cattle Egret	2019/7/26	3	0	24.7848148	120.9945450	https://www.inaturalist.org/observations/29554727
4365	Bubulcus ibis	Cattle Egret	2022/4/26	1	0	24.7864682	120.9140893	https://www.inaturalist.org/observations/113054134
4366	Bubulcus ibis	Cattle Egret	2022/9/25	2	0	24.7873383	120.9840917	https://www.inaturalist.org/observations/136390730
4367	Bubulcus ibis	Cattle Egret	2021/5/12	1	0	24.7892056	121.0349808	https://www.inaturalist.org/observations/78441030
4368	Bubulcus ibis	Cattle Egret	2020/6/7	1	0	24.7900290	120.9905460	https://www.inaturalist.org/observations/48729724
4369	Bubulcus ibis	Cattle Egret	2022/7/3	1	0	24.7908950	120.9868083	https://www.inaturalist.org/observations/124519095
4370	Bubulcus ibis	Cattle Egret	2022/6/20	1	0	24.7909817	120.9877770	https://www.inaturalist.org/observations/124724144
4371	Bubulcus ibis	Cattle Egret	2021/5/8	3	0	24.7910383	120.9866417	https://www.inaturalist.org/observations/77856321
4372	Bubulcus ibis	Cattle Egret	2021/5/8	2	0	24.7910550	120.9866633	https://www.inaturalist.org/observations/77856304
4373	Bubulcus ibis	Cattle Egret	2021/6/20	1	0	24.7911494	120.9962118	https://www.inaturalist.org/observations/83758118
4374	Bubulcus ibis	Cattle Egret	2022/5/22	2	0	24.7911879	121.7193978	https://www.inaturalist.org/observations/118164829

4375	Bubulcus ibis coromandus	Cattle Egret	2022/5/22	2	0	24.7911879	121.7193978	https://www.inaturalist.org/observations/118164858
4376	Bubulcus ibis coromandus	Cattle Egret	2022/5/22	2	0	24.7911879	121.7193978	https://www.inaturalist.org/observations/118164859
4377	Bubulcus ibis coromandus	Cattle Egret	2022/5/22	2	0	24.7911879	121.7193978	https://www.inaturalist.org/observations/118164860
4378	Bubulcus ibis	Cattle Egret	2021/6/20	1	0	24.7911964	120.9962511	https://www.inaturalist.org/observations/83758538
4379	Bubulcus ibis	Cattle Egret	2021/6/20	1	0	24.7913900	120.9961240	https://www.inaturalist.org/observations/83758438
4380	Bubulcus ibis	Cattle Egret	2022/5/17	1	0	24.7915234	120.9963087	https://www.inaturalist.org/observations/117428525
4381	Bubulcus ibis	Cattle Egret	2022/5/21	1	0	24.7915724	120.9962129	https://www.inaturalist.org/observations/117974265
4382	Bubulcus ibis	Cattle Egret	2022/3/29	2	0	24.7918700	120.9919133	https://www.inaturalist.org/observations/111416930
4383	Bubulcus ibis	Cattle Egret	2021/6/21	1	0	24.7921486	120.9920554	https://www.inaturalist.org/observations/83946828
4384	Bubulcus ibis	Cattle Egret	2020/5/29	1	0	24.7923023	120.9921472	https://www.inaturalist.org/observations/47686392
4385	Bubulcus ibis	Cattle Egret	2018/5/10	3	0	24.7924200	120.9833300	https://www.inaturalist.org/observations/17309855
4386	Bubulcus ibis	Cattle Egret	2019/9/1	2	0	24.7924300	121.7425967	https://www.inaturalist.org/observations/32143878
4387	Bubulcus ibis	Cattle Egret	2020/1/8	2	0	24.7926825	121.7403539	https://www.inaturalist.org/observations/37422473
4388	Bubulcus ibis	Cattle Egret	2022/9/15	1	0	24.7930033	120.9837417	https://www.inaturalist.org/observations/144233291
4389	Bubulcus ibis	Cattle Egret	2022/4/29	1	0	24.7931812	120.9797777	https://www.inaturalist.org/observations/113290539
4390	Bubulcus ibis	Cattle Egret	2021/3/14	3	0	24.7932950	121.7420433	https://www.inaturalist.org/observations/71209790
4391	Bubulcus ibis	Cattle Egret	2020/8/27	1	0	24.7938462	120.9819180	https://www.inaturalist.org/observations/57733129
4392	Bubulcus ibis	Cattle Egret	2022/4/29	2	0	24.7943200	120.9926383	https://www.inaturalist.org/observations/135879822
4393	Bubulcus ibis	Cattle Egret	2015/10/15	5	0	24.7944110	121.7639040	https://www.inaturalist.org/observations/9320379
4394	Bubulcus ibis	Cattle Egret	2020/8/23	1	0	24.7957239	120.9170664	https://www.inaturalist.org/observations/57408253
4395	Bubulcus ibis	Cattle Egret	2020/4/27	1	0	24.7972978	120.9169134	https://www.inaturalist.org/observations/43869099
4396	Bubulcus ibis	Cattle Egret	2019/11/8	2	0	24.7973375	121.7408514	https://www.inaturalist.org/observations/35626348
4397	Bubulcus ibis	Cattle Egret	2022/7/8	2	0	24.7975128	120.9157321	https://www.inaturalist.org/observations/128910960
4398	Bubulcus ibis	Cattle Egret	2020/5/25	1	0	24.7979787	120.9820769	https://www.inaturalist.org/observations/47245271
4399	Bubulcus ibis	Cattle Egret	2021/4/25	1	0	24.7980367	121.7383100	https://www.inaturalist.org/observations/76520044

4400	Bubulcus ibis	Cattle Egret	2021/4/11	2	0	24.7992850	121.7389600	https://www.inaturalist.org/observations/73596991
4401	Bubulcus ibis	Cattle Egret	2021/7/7	1	0	24.7999517	121.8036433	https://www.inaturalist.org/observations/86395662
4402	Bubulcus ibis	Cattle Egret	2022/6/10	1	0	24.8003180	121.0328031	https://www.inaturalist.org/observations/121800319
4403	Bubulcus ibis	Cattle Egret	2022/6/10	1	0	24.8006789	121.0319234	https://www.inaturalist.org/observations/121800320
4404	Bubulcus ibis	Cattle Egret	2022/6/10	1	0	24.8020771	121.0308762	https://www.inaturalist.org/observations/121800342
4405	Bubulcus ibis	Cattle Egret	2020/4/19	2	0	24.8034067	120.9881698	https://www.inaturalist.org/observations/42544624
4406	Bubulcus ibis	Cattle Egret	2020/4/25	2	0	24.8047160	121.0285607	https://www.inaturalist.org/observations/43345409
4407	Bubulcus ibis	Cattle Egret	2021/7/19	1	0	24.8052211	120.9354411	https://www.inaturalist.org/observations/87677051
4408	Bubulcus ibis	Cattle Egret	2020/7/15	1	0	24.8057430	121.0181020	https://www.inaturalist.org/observations/53340048
4409	Bubulcus ibis	Cattle Egret	2020/12/6	1	0	24.8062077	121.7836207	https://www.inaturalist.org/observations/66324734
4410	Bubulcus ibis	Cattle Egret	2020/7/22	1	0	24.8071150	121.0165350	https://www.inaturalist.org/observations/54483457
4411	Bubulcus ibis	Cattle Egret	2020/4/26	2	0	24.8086291	121.8025017	https://www.inaturalist.org/observations/43587222
4412	Bubulcus ibis	Cattle Egret	2022/5/7	1	0	24.8087367	121.0139389	https://www.inaturalist.org/observations/115869130
4413	Bubulcus ibis	Cattle Egret	2022/6/3	1	0	24.8102488	121.0549421	https://www.inaturalist.org/observations/121801127
4414	Bubulcus ibis	Cattle Egret	2019/9/20	2	0	24.8129164	121.0394222	https://www.inaturalist.org/observations/33046328
4415	Bubulcus ibis	Cattle Egret	2019/10/10	1	0	24.8133823	102.7890955	https://www.inaturalist.org/observations/34146757
4416	Bubulcus ibis	Cattle Egret	2019/10/22	1	0	24.8160297	102.7863365	https://www.inaturalist.org/observations/34734235
4417	Bubulcus ibis	Cattle Egret	2021/8/1	3	0	24.8165756	121.8129932	https://www.inaturalist.org/observations/89484633
4418	Bubulcus ibis	Cattle Egret	2021/8/1	2	0	24.8165756	121.8129932	https://www.inaturalist.org/observations/89484635
4419	Bubulcus ibis	Cattle Egret	2019/7/16	1	0	24.8170167	121.2519400	https://www.inaturalist.org/observations/65881770
4420	Bubulcus ibis	Cattle Egret	2020/7/23	1	0	24.8251950	120.9792250	https://www.inaturalist.org/observations/54485270
4421	Bubulcus ibis	Cattle Egret	2020/5/20	1	0	24.8253733	121.7559067	https://www.inaturalist.org/observations/49696576
4422	Bubulcus ibis	Cattle Egret	2021/4/10	1	0	24.8259139	121.4436497	https://www.inaturalist.org/observations/73475395
4423	Bubulcus ibis	Cattle Egret	2020/8/8	1	0	24.8292667	120.9813467	https://www.inaturalist.org/observations/55783579
4424	Bubulcus ibis	Cattle Egret	2022/3/26	1	0	24.8299348	120.9176764	https://www.inaturalist.org/observations/109521153

4425	Bubulcus ibis	Cattle Egret	2018/12/4	3	0	24.8312222	121.7764583	https://www.inaturalist.org/observations/19151088
4426	Bubulcus ibis	Cattle Egret	2020/5/22	1	0	24.8317833	121.7702117	https://www.inaturalist.org/observations/46947247
4427	Bubulcus ibis	Cattle Egret	2019/11/18	1	0	24.8374913	121.7537901	https://www.inaturalist.org/observations/35972141
4428	Bubulcus ibis	Cattle Egret	2022/6/15	2	0	24.8388061	121.7803631	https://www.inaturalist.org/observations/125754889
4429	Bubulcus ibis	Cattle Egret	2020/5/2	2	0	24.8394645	121.7974941	https://www.inaturalist.org/observations/44577993
4430	Bubulcus ibis	Cattle Egret	2022/5/1	1	0	24.8414931	120.9207690	https://www.inaturalist.org/observations/114133313
4431	Bubulcus ibis	Cattle Egret	2018/6/17	2	0	24.8423918	121.2877526	https://www.inaturalist.org/observations/13542444
4432	Bubulcus ibis	Cattle Egret	2022/6/17	1	0	24.8431133	121.9439550	https://www.inaturalist.org/observations/122140125
4433	Bubulcus ibis	Cattle Egret	2020/4/25	2	0	24.8507411	121.2278049	https://www.inaturalist.org/observations/43273436
4434	Bubulcus ibis	Cattle Egret	2020/4/25	2	0	24.8508371	121.2277287	https://www.inaturalist.org/observations/43273452
4435	Bubulcus ibis	Cattle Egret	2022/3/18	2	0	24.8837843	121.2273602	https://www.inaturalist.org/observations/108850128
4436	Bubulcus ibis	Cattle Egret	2022/8/2	0	0	24.8849908	121.6661249	https://www.inaturalist.org/observations/129048358
4437	Bubulcus ibis	Cattle Egret	2022/8/2	0	0	24.8851067	121.6661433	https://www.inaturalist.org/observations/129087893
4438	Bubulcus ibis	Cattle Egret	2022/8/2	0	0	24.8852031	121.6659993	https://www.inaturalist.org/observations/129048307
4439	Bubulcus ibis	Cattle Egret	2020/10/4	3	0	24.8865783	121.1361305	https://www.inaturalist.org/observations/61651728
4440	Bubulcus ibis	Cattle Egret	2020/4/26	1	0	24.8895710	121.1587061	https://www.inaturalist.org/observations/43612022
4441	Bubulcus ibis	Cattle Egret	2022/9/17	2	0	24.8922417	121.0586778	https://www.inaturalist.org/observations/135349761
4442	Bubulcus ibis	Cattle Egret	2016/10/6	1	0	24.8998862	121.5541077	https://www.inaturalist.org/observations/33295333
4443	Bubulcus ibis	Cattle Egret	2021/9/17	1	0	24.8999772	121.3695834	https://www.inaturalist.org/observations/95736003
4444	Bubulcus ibis	Cattle Egret	2019/4/29	2	0	24.9001246	121.7325098	https://www.inaturalist.org/observations/24236822
4445	Bubulcus ibis	Cattle Egret	2020/5/16	1	0	24.9073181	121.3092804	https://www.inaturalist.org/observations/46059605
4446	Bubulcus ibis	Cattle Egret	2022/9/11	1	0	24.9120165	121.3005561	https://www.inaturalist.org/observations/135397398
4447	Bubulcus ibis	Cattle Egret	2022/3/30	1	0	24.9141433	121.3050255	https://www.inaturalist.org/observations/109894175
4448	Bubulcus ibis	Cattle Egret	2019/7/28	2	0	24.9160577	121.8403661	https://www.inaturalist.org/observations/29701673
4449	Bubulcus ibis	Cattle Egret	2022/10/4	1	0	24.9165307	121.1251004	https://www.inaturalist.org/observations/137529396

4450	Bubulcus ibis	Cattle Egret	2020/6/6	1	0	24.9186639	121.1267604	https://www.inaturalist.org/observations/49132247
4451	Bubulcus ibis	Cattle Egret	2021/12/23	2	0	24.9248820	121.1776610	https://www.inaturalist.org/observations/103549830
4452	Bubulcus ibis	Cattle Egret	2022/5/4	2	0	24.9267158	121.2343334	https://www.inaturalist.org/observations/115329593
4453	Bubulcus ibis	Cattle Egret	2022/8/20	4	0	24.9285919	121.3944946	https://www.inaturalist.org/observations/131745772
4454	Bubulcus ibis	Cattle Egret	2020/5/16	2	0	24.9332367	121.3683917	https://www.inaturalist.org/observations/46485156
4455	Bubulcus ibis	Cattle Egret	2022/3/1	2	0	24.9337267	121.0781483	https://www.inaturalist.org/observations/144774549
4456	Bubulcus ibis	Cattle Egret	2020/4/21	1	0	24.9344289	121.7130037	https://www.inaturalist.org/observations/48901918
4457	Bubulcus ibis	Cattle Egret	2014/7/13	1	0	24.9350844	121.7107613	https://www.inaturalist.org/observations/70893104
4458	Bubulcus ibis	Cattle Egret	2022/8/11	1	0	24.9357183	121.7129131	https://www.inaturalist.org/observations/131348835
4459	Bubulcus ibis	Cattle Egret	2022/4/17	1	0	24.9357434	121.7131031	https://www.inaturalist.org/observations/111756029
4460	Bubulcus ibis	Cattle Egret	2022/4/17	1	0	24.9358033	121.7132117	https://www.inaturalist.org/observations/111911721
4461	Bubulcus ibis	Cattle Egret	2020/4/18	1	0	24.9358783	121.7131717	https://www.inaturalist.org/observations/43641378
4462	Bubulcus ibis	Cattle Egret	2019/4/21	1	0	24.9359036	121.7131424	https://www.inaturalist.org/observations/23028393
4463	Bubulcus ibis	Cattle Egret	2012/5/6	2	0	24.9361302	121.7132967	https://www.inaturalist.org/observations/45677112
4464	Bubulcus ibis	Cattle Egret	2021/8/17	2	0	24.9363667	121.2108000	https://www.inaturalist.org/observations/91499072
4465	Bubulcus ibis	Cattle Egret	2019/10/24	1	0	24.9375443	121.3632410	https://www.inaturalist.org/observations/34820248
4466	Bubulcus ibis	Cattle Egret	2022/8/3	3	0	24.9377367	121.0283233	https://www.inaturalist.org/observations/129253261
4467	Bubulcus ibis	Cattle Egret	2022/8/3	3	0	24.9377783	121.0283233	https://www.inaturalist.org/observations/129253262
4468	Bubulcus ibis	Cattle Egret	2022/11/14	2	0	24.9399650	121.0276533	https://www.inaturalist.org/observations/141967889
4469	Bubulcus ibis	Cattle Egret	2019/10/25	1	0	24.9401034	121.3654202	https://www.inaturalist.org/observations/34862984
4470	Bubulcus ibis	Cattle Egret	2019/10/23	1	0	24.9404507	121.3659592	https://www.inaturalist.org/observations/34774150
4471	Bubulcus ibis	Cattle Egret	2019/10/23	2	0	24.9411833	121.3676755	https://www.inaturalist.org/observations/34771478
4472	Bubulcus ibis	Cattle Egret	2019/10/25	2	0	24.9412514	121.3680864	https://www.inaturalist.org/observations/34864018
4473	Bubulcus ibis	Cattle Egret	2019/10/23	1	0	24.9412911	121.3681914	https://www.inaturalist.org/observations/34773763
4474	Bubulcus ibis	Cattle Egret	2019/10/23	2	0	24.9412918	121.3681946	https://www.inaturalist.org/observations/34771384

4475	Bubulcus ibis	Cattle Egret	2019/10/23	2	0	24.9413637	121.3677413	https://www.inaturalist.org/observations/34771543
4476	Bubulcus ibis	Cattle Egret	2019/10/25	2	0	24.9416024	121.3685966	https://www.inaturalist.org/observations/34863976
4477	Bubulcus ibis	Cattle Egret	2019/10/23	1	0	24.9417085	121.3669512	https://www.inaturalist.org/observations/34773678
4478	Bubulcus ibis	Cattle Egret	2019/10/23	2	0	24.9417137	121.3680908	https://www.inaturalist.org/observations/34771463
4479	Bubulcus ibis	Cattle Egret	2019/10/23	2	0	24.9418099	121.3669888	https://www.inaturalist.org/observations/34771295
4480	Bubulcus ibis	Cattle Egret	2019/10/23	1	0	24.9418283	121.3671944	https://www.inaturalist.org/observations/34771359
4481	Bubulcus ibis	Cattle Egret	2019/10/23	2	0	24.9418329	121.3671015	https://www.inaturalist.org/observations/34771468
4482	Bubulcus ibis	Cattle Egret	2019/10/23	1	0	24.9418530	121.3668528	https://www.inaturalist.org/observations/34774095
4483	Bubulcus ibis	Cattle Egret	2019/10/23	1	0	24.9418838	121.3661603	https://www.inaturalist.org/observations/34773777
4484	Bubulcus ibis	Cattle Egret	2019/10/23	2	0	24.9419117	121.3666917	https://www.inaturalist.org/observations/34771528
4485	Bubulcus ibis	Cattle Egret	2019/10/23	1	0	24.9419217	121.3666533	https://www.inaturalist.org/observations/34771797
4486	Bubulcus ibis	Cattle Egret	2019/10/23	2	0	24.9419219	121.3666517	https://www.inaturalist.org/observations/34771082
4487	Bubulcus ibis	Cattle Egret	2019/10/23	1	0	24.9419242	121.3665388	https://www.inaturalist.org/observations/34774205
4488	Bubulcus ibis	Cattle Egret	2019/10/23	2	0	24.9419293	121.3660423	https://www.inaturalist.org/observations/34773766
4489	Bubulcus ibis	Cattle Egret	2019/10/23	1	0	24.9419417	121.3666167	https://www.inaturalist.org/observations/34772540
4490	Bubulcus ibis	Cattle Egret	2019/10/23	1	0	24.9419583	121.3664383	https://www.inaturalist.org/observations/34774345
4491	Bubulcus ibis	Cattle Egret	2019/10/23	2	0	24.9419612	121.3666547	https://www.inaturalist.org/observations/34771553
4492	Bubulcus ibis	Cattle Egret	2019/10/23	2	0	24.9419629	121.3663940	https://www.inaturalist.org/observations/34771381
4493	Bubulcus ibis	Cattle Egret	2019/10/23	2	0	24.9419704	121.3673882	https://www.inaturalist.org/observations/34771389
4494	Bubulcus ibis	Cattle Egret	2019/10/23	1	0	24.9419785	121.3664856	https://www.inaturalist.org/observations/35007561
4495	Bubulcus ibis	Cattle Egret	2019/10/23	2	0	24.9419820	121.3665536	https://www.inaturalist.org/observations/34771067
4496	Bubulcus ibis	Cattle Egret	2019/10/23	1	0	24.9420007	121.3661878	https://www.inaturalist.org/observations/34774666
4497	Bubulcus ibis	Cattle Egret	2019/10/23	2	0	24.9420036	121.3684409	https://www.inaturalist.org/observations/34832992
4498	Bubulcus ibis	Cattle Egret	2019/10/23	2	0	24.9420242	121.3663431	https://www.inaturalist.org/observations/34771126
4499	Bubulcus ibis	Cattle Egret	2019/10/25	1	0	24.9420420	121.3683233	https://www.inaturalist.org/observations/34864052

4500	Bubulcus ibis	Cattle Egret	2019/10/23	2	0	24.9420451	121.3673371	https://www.inaturalist.org/observations/34771283
4501	Bubulcus ibis	Cattle Egret	2020/6/5	1	0	24.9420500	121.3674700	https://www.inaturalist.org/observations/48598568
4502	Bubulcus ibis	Cattle Egret	2019/10/24	2	0	24.9421040	121.3689330	https://www.inaturalist.org/observations/34809559
4503	Bubulcus ibis	Cattle Egret	2019/10/24	2	0	24.9421572	121.3690630	https://www.inaturalist.org/observations/34808692
4504	Bubulcus ibis	Cattle Egret	2016/8/13	3	0	24.9421787	121.3701172	http://www.inaturalist.org/observations/3966678
4505	Bubulcus ibis	Cattle Egret	2019/10/23	1	0	24.9421852	121.3690880	https://www.inaturalist.org/observations/34771027
4506	Bubulcus ibis	Cattle Egret	2019/10/23	3	0	24.9423142	121.3693161	https://www.inaturalist.org/observations/34871970
4507	Bubulcus ibis	Cattle Egret	2019/10/23	2	0	24.9423414	121.3710240	https://www.inaturalist.org/observations/34774861
4508	Bubulcus ibis	Cattle Egret	2020/6/2	3	0	24.9423467	121.3675833	https://www.inaturalist.org/observations/48199193
4509	Bubulcus ibis	Cattle Egret	2019/10/25	1	0	24.9423730	121.3686995	https://www.inaturalist.org/observations/34864004
4510	Bubulcus ibis	Cattle Egret	2020/5/6	2	0	24.9426449	121.7108612	https://www.inaturalist.org/observations/45233087
4511	Bubulcus ibis	Cattle Egret	2019/10/25	2	0	24.9426583	121.3664962	https://www.inaturalist.org/observations/34860790
4512	Bubulcus ibis	Cattle Egret	2019/10/23	2	0	24.9428469	121.3677263	https://www.inaturalist.org/observations/34771382
4513	Bubulcus ibis	Cattle Egret	2019/10/23	1	0	24.9429809	121.3693045	https://www.inaturalist.org/observations/34930646
4514	Bubulcus ibis	Cattle Egret	2019/10/23	2	0	24.9433814	121.3698173	https://www.inaturalist.org/observations/34771883
4515	Bubulcus ibis	Cattle Egret	2020/7/3	1	0	24.9434083	121.3676300	https://www.inaturalist.org/observations/51786677
4516	Bubulcus ibis	Cattle Egret	2019/10/27	2	0	24.9442722	121.3681463	https://www.inaturalist.org/observations/34947874
4517	Bubulcus ibis	Cattle Egret	2019/10/26	2	0	24.9451160	121.3691177	https://www.inaturalist.org/observations/34892930
4518	Bubulcus ibis	Cattle Egret	2019/10/25	2	0	24.9451450	121.3686257	https://www.inaturalist.org/observations/34861846
4519	Bubulcus ibis	Cattle Egret	2018/5/6	3	0	24.9452187	121.3678746	https://www.inaturalist.org/observations/12189208
4520	Bubulcus ibis	Cattle Egret	2019/10/23	2	0	24.9470898	121.3806755	https://www.inaturalist.org/observations/35050191
4521	Bubulcus ibis	Cattle Egret	2021/12/8	1	0	24.9513544	121.5001340	https://www.inaturalist.org/observations/102704141
4522	Bubulcus ibis	Cattle Egret	2021/4/13	2	0	24.9514817	121.5001057	https://www.inaturalist.org/observations/73934814
4523	Bubulcus ibis	Cattle Egret	2022/11/29	3	0	24.9515259	121.4998465	https://www.inaturalist.org/observations/143269239
4524	Bubulcus ibis	Cattle Egret	2021/11/17	2	0	24.9515377	121.4998332	https://www.inaturalist.org/observations/101311808

4525	Bubulcus ibis	Cattle Egret	2022/11/28	2	0	24.9515591	121.4997897	https://www.inaturalist.org/observations/143180558
4526	Bubulcus ibis	Cattle Egret	2021/11/30	2	0	24.9515897	121.5001355	https://www.inaturalist.org/observations/102231148
4527	Bubulcus ibis	Cattle Egret	2021/11/29	2	0	24.9517736	121.4999562	https://www.inaturalist.org/observations/102130782
4528	Bubulcus ibis	Cattle Egret	2018/5/10	3	0	24.9527667	121.3013633	https://www.inaturalist.org/observations/19482359
4529	Bubulcus ibis	Cattle Egret	2022/8/23	2	0	24.9554722	121.4917861	https://www.inaturalist.org/observations/131987739
4530	Bubulcus ibis	Cattle Egret	2022/5/29	2	0	24.9584992	121.1906346	https://www.inaturalist.org/observations/119369677
4531	Bubulcus ibis	Cattle Egret	2022/3/5	2	0	24.9588200	121.5264500	https://www.inaturalist.org/observations/107928907
4532	Bubulcus ibis	Cattle Egret	2020/5/1	2	0	24.9589407	121.5431052	https://www.inaturalist.org/observations/44743704
4533	Bubulcus ibis	Cattle Egret	2006/9/3	3	0	24.9602016	121.5276659	https://www.inaturalist.org/observations/16706906
4534	Bubulcus ibis	Cattle Egret	2021/4/11	2	0	24.9608926	121.2181881	https://www.inaturalist.org/observations/73608069
4535	Bubulcus ibis	Cattle Egret	2020/6/8	1	0	24.9647000	121.1935667	https://www.inaturalist.org/observations/51900422
4536	Bubulcus ibis	Cattle Egret	2017/4/11	4	0	24.9674636	121.2681906	https://www.inaturalist.org/observations/5646095
4537	Bubulcus ibis coromandus	Cattle Egret	2017/6/5	2	0	24.9677122	121.2669992	https://www.inaturalist.org/observations/6506311
4538	Bubulcus ibis	Cattle Egret	2020/7/15	3	0	24.9677367	121.1945200	https://www.inaturalist.org/observations/53464818
4539	Bubulcus ibis	Cattle Egret	2020/7/10	1	0	24.9680283	121.1930300	https://www.inaturalist.org/observations/52629112
4540	Bubulcus ibis	Cattle Egret	2017/4/12	4	0	24.9680528	121.2683662	https://www.inaturalist.org/observations/5657476
4541	Bubulcus ibis	Cattle Egret	2017/5/5	3	0	24.9681627	121.2688134	https://www.inaturalist.org/observations/6097403
4542	Bubulcus ibis	Cattle Egret	2022/6/10	1	0	24.9683755	121.4106643	https://www.inaturalist.org/observations/121387725
4543	Bubulcus ibis	Cattle Egret	2022/5/1	2	0	24.9684854	121.1918692	https://www.inaturalist.org/observations/114179796
4544	Bubulcus ibis	Cattle Egret	2018/8/22	4	0	24.9690083	121.1937117	https://www.inaturalist.org/observations/15776824
4545	Bubulcus ibis	Cattle Egret	2022/3/13	3	0	24.9694028	121.5328518	https://www.inaturalist.org/observations/108493429
4546	Bubulcus ibis	Cattle Egret	2021/5/1	2	0	24.9697744	121.1950774	https://www.inaturalist.org/observations/76005757
4547	Bubulcus ibis	Cattle Egret	2019/9/23	2	0	24.9698203	121.2679243	https://www.inaturalist.org/observations/33231751
4548	Bubulcus ibis	Cattle Egret	2019/7/14	2	0	24.9704060	121.1942307	https://www.inaturalist.org/observations/29000206
4549	Bubulcus ibis	Cattle Egret	2020/6/26	2	0	24.9704500	121.1919340	https://www.inaturalist.org/observations/51036252

4550	Bubulcus ibis	Cattle Egret	2020/6/26	2	0	24.9704500	121.1919340	https://www.inaturalist.org/observations/71924487
4551	Bubulcus ibis	Cattle Egret	2019/5/30	6	0	24.9792011	121.4713697	https://www.inaturalist.org/observations/26046128
4552	Bubulcus ibis	Cattle Egret	2021/12/7	1	0	24.9845799	121.5725543	https://www.inaturalist.org/observations/102671347
4553	Bubulcus ibis	Cattle Egret	2012/7/10	1	0	24.9867251	121.7068489	https://www.inaturalist.org/observations/67324882
4554	Bubulcus ibis	Cattle Egret	2021/10/6	1	0	24.9888588	121.5362404	https://www.inaturalist.org/observations/97652722
4555	Bubulcus ibis	Cattle Egret	2022/2/7	1	0	24.9895201	121.5362112	https://www.inaturalist.org/observations/107258550
4556	Bubulcus ibis	Cattle Egret	2020/6/18	2	0	24.9896953	121.5554720	https://www.inaturalist.org/observations/50022801
4557	Bubulcus ibis	Cattle Egret	2022/2/7	1	0	24.9905452	121.5363069	https://www.inaturalist.org/observations/107258549
4558	Bubulcus ibis	Cattle Egret	2020/12/6	1	0	24.9907562	121.5926678	https://www.inaturalist.org/observations/105608442
4559	Bubulcus ibis	Cattle Egret	2021/4/14	2	0	24.9915450	121.5895217	https://www.inaturalist.org/observations/74582920
4560	Bubulcus ibis	Cattle Egret	2019/7/30	3	0	24.9915610	121.5889136	https://www.inaturalist.org/observations/29890916
4561	Bubulcus ibis	Cattle Egret	2022/5/1	3	0	24.9919802	121.5451351	https://www.inaturalist.org/observations/116730594
4562	Bubulcus ibis	Cattle Egret	2021/12/22	1	0	24.9920088	121.5368435	https://www.inaturalist.org/observations/103476104
4563	Bubulcus ibis	Cattle Egret	2019/9/22	2	0	24.9924955	121.5907773	https://www.inaturalist.org/observations/33173540
4564	Bubulcus ibis	Cattle Egret	2020/1/17	1	0	24.9924957	121.5908480	https://www.inaturalist.org/observations/37673985
4565	Bubulcus ibis	Cattle Egret	2020/2/12	2	0	24.9926000	121.5906008	https://www.inaturalist.org/observations/38558230
4566	Bubulcus ibis	Cattle Egret	2020/5/24	2	0	24.9926250	121.5906050	https://www.inaturalist.org/observations/47120113
4567	Bubulcus ibis	Cattle Egret	2022/7/19	2	0	24.9926540	121.5906190	https://www.inaturalist.org/observations/126960077
4568	Bubulcus ibis	Cattle Egret	2020/8/23	2	0	24.9926717	121.5908800	https://www.inaturalist.org/observations/57301010
4569	Bubulcus ibis	Cattle Egret	2020/3/4	1	0	24.9932262	121.5901251	https://www.inaturalist.org/observations/40921272
4570	Bubulcus ibis	Cattle Egret	2020/3/4	1	0	24.9932287	121.5901282	https://www.inaturalist.org/observations/40921274
4571	Bubulcus ibis	Cattle Egret	2011/11/14	2	0	24.9935985	121.5845901	https://www.inaturalist.org/observations/130588359
4572	Bubulcus ibis	Cattle Egret	2017/5/29	2	0	24.9941434	121.5878325	https://www.inaturalist.org/observations/105322183
4573	Bubulcus ibis	Cattle Egret	2017/5/29	2	0	24.9942330	121.5884189	https://www.inaturalist.org/observations/105322182
4574	Bubulcus ibis coromandus	Cattle Egret	2022/8/3	3	0	24.9942870	121.5860032	https://www.inaturalist.org/observations/129608774

4575	Bubulcus ibis	Cattle Egret	2017/12/30	2	0	24.9949343	121.5887316	https://www.inaturalist.org/observations/112856902
4576	Bubulcus ibis	Cattle Egret	2021/2/23	2	0	24.9949854	121.5871226	https://www.inaturalist.org/observations/75020404
4577	Bubulcus ibis	Cattle Egret	2008/11/21	1	0	24.9950525	121.5856276	https://www.inaturalist.org/observations/81252117
4578	Bubulcus ibis	Cattle Egret	2020/8/29	1	0	24.9950667	121.5874972	https://www.inaturalist.org/observations/57914180
4579	Bubulcus ibis coromandus	Cattle Egret	2019/3/2	4	0	24.9951459	121.5872260	https://www.inaturalist.org/observations/20872395
4580	Bubulcus ibis	Cattle Egret	2022/3/6	1	0	24.9951550	121.5875083	https://www.inaturalist.org/observations/108006984
4581	Bubulcus ibis	Cattle Egret	2020/1/28	0	0	24.9951750	121.5872722	https://www.inaturalist.org/observations/38118639
4582	Bubulcus ibis	Cattle Egret	2019/7/7	1	0	24.9952383	121.5856467	https://www.inaturalist.org/observations/73187721
4583	Bubulcus ibis	Cattle Egret	2020/3/4	1	0	24.9952613	121.5871867	https://www.inaturalist.org/observations/40921347
4584	Bubulcus ibis	Cattle Egret	2022/8/20	2	0	24.9952615	121.5870639	https://www.inaturalist.org/observations/131700503
4585	Bubulcus ibis	Cattle Egret	2017/12/9	6	0	24.9952621	121.5870130	https://www.inaturalist.org/observations/9099457
4586	Bubulcus ibis	Cattle Egret	2018/8/7	1	0	24.9952631	121.5871793	https://www.inaturalist.org/observations/111437783
4587	Bubulcus ibis	Cattle Egret	2019/3/6	2	0	24.9952827	121.5868956	https://www.inaturalist.org/observations/20991602
4588	Bubulcus ibis	Cattle Egret	2018/9/26	2	0	24.9952927	121.5870361	https://www.inaturalist.org/observations/17028368
4589	Bubulcus ibis	Cattle Egret	2014/8/16	6	0	24.9952930	121.5874900	http://www.inaturalist.org/observations/1078376
4590	Bubulcus ibis	Cattle Egret	2022/11/10	1	0	24.9953044	121.5873192	https://www.inaturalist.org/observations/141594824
4591	Bubulcus ibis	Cattle Egret	2016/7/23	2	0	24.9953093	121.5868703	https://www.inaturalist.org/observations/105322177
4592	Bubulcus ibis	Cattle Egret	2020/3/3	1	0	24.9953460	121.5869228	https://www.inaturalist.org/observations/39485310
4593	Bubulcus ibis	Cattle Egret	2020/3/4	1	0	24.9953490	121.5871560	https://www.inaturalist.org/observations/39743396
4594	Bubulcus ibis	Cattle Egret	2020/3/4	1	0	24.9953575	121.5871251	https://www.inaturalist.org/observations/40921334
4595	Bubulcus ibis	Cattle Egret	2022/4/9	1	0	24.9953667	121.5872637	https://www.inaturalist.org/observations/110832182
4596	Bubulcus ibis	Cattle Egret	2018/9/26	2	0	24.9955467	121.5837950	https://www.inaturalist.org/observations/17021949
4597	Bubulcus ibis	Cattle Egret	2017/5/6	3	0	24.9967300	121.5779583	https://www.inaturalist.org/observations/6109565
4598	Bubulcus ibis	Cattle Egret	2019/1/9	7	0	24.9967583	121.5778050	https://www.inaturalist.org/observations/19534751
4599	Bubulcus ibis	Cattle Egret	2022/8/20	2	0	24.9969730	121.5826256	https://www.inaturalist.org/observations/131700412

4600	Bubulcus ibis	Cattle Egret	2022/10/4	1	0	24.9973075	121.5815184	https://www.inaturalist.org/observations/137478518
4601	Bubulcus ibis	Cattle Egret	2020/7/30	1	0	24.9975033	121.5830682	https://www.inaturalist.org/observations/54820474
4602	Bubulcus ibis	Cattle Egret	2020/1/13	2	0	24.9977466	121.5859408	https://www.inaturalist.org/observations/37647981
4603	Bubulcus ibis	Cattle Egret	2018/8/26	3	0	24.9977583	121.5803059	https://www.inaturalist.org/observations/16225118
4604	Bubulcus ibis	Cattle Egret	2021/9/11	1	0	24.9980725	121.5816589	https://www.inaturalist.org/observations/96689689
4605	Bubulcus ibis	Cattle Egret	2020/10/9	1	0	24.9980725	121.5816589	https://www.inaturalist.org/observations/134374259
4606	Bubulcus ibis	Cattle Egret	2013/8/29	1	0	24.9980730	121.5816590	https://www.inaturalist.org/observations/106974522
4607	Bubulcus ibis	Cattle Egret	2018/7/24	2	0	24.9981393	121.5796094	https://www.inaturalist.org/observations/14876013
4608	Bubulcus ibis	Cattle Egret	2019/7/7	2	0	24.9982383	121.5814533	https://www.inaturalist.org/observations/73585558
4609	Bubulcus ibis	Cattle Egret	2021/4/23	3	0	24.9982578	121.5819646	https://www.inaturalist.org/observations/74915017
4610	Bubulcus ibis	Cattle Egret	2019/9/14	1	0	24.9983469	121.5810358	https://www.inaturalist.org/observations/34293090
4611	Bubulcus ibis	Cattle Egret	2021/4/23	3	0	24.9984436	121.5815926	https://www.inaturalist.org/observations/74915054
4612	Bubulcus ibis coromandus	Cattle Egret	2021/5/16	2	0	24.9984951	121.4458178	https://www.inaturalist.org/observations/78962091
4613	Bubulcus ibis	Cattle Egret	2020/2/14	2	0	24.9986580	121.5812070	https://www.inaturalist.org/observations/49543209
4614	Bubulcus ibis	Cattle Egret	2008/10/18	2	0	24.9987461	121.5823333	https://www.inaturalist.org/observations/79221079
4615	Bubulcus ibis	Cattle Egret	2020/4/15	1	0	24.9987890	121.5815960	https://www.inaturalist.org/observations/102180877
4616	Bubulcus ibis	Cattle Egret	2015/7/24	4	0	24.9988534	121.5810682	http://www.inaturalist.org/observations/3687502
4617	Bubulcus ibis	Cattle Egret	2019/4/20	1	0	24.9998304	121.4386032	https://www.inaturalist.org/observations/23911462
4618	Bubulcus ibis	Cattle Egret	2018/8/26	2	0	25.0009561	121.5856959	https://www.inaturalist.org/observations/16225124
4619	Bubulcus ibis	Cattle Egret	2020/6/12	2	0	25.0021050	121.3282355	https://www.inaturalist.org/observations/49289967
4620	Bubulcus ibis	Cattle Egret	2017/4/15	2	0	25.0025291	121.9299730	https://www.inaturalist.org/observations/36014487
4621	Bubulcus ibis	Cattle Egret	2020/4/19	1	0	25.0069883	121.5295767	https://www.inaturalist.org/observations/42550369
4622	Bubulcus ibis	Cattle Egret	2021/11/12	1	0	25.0071983	121.5364246	https://www.inaturalist.org/observations/103135076
4623	Bubulcus ibis	Cattle Egret	2021/11/4	2	0	25.0075361	121.5353550	https://www.inaturalist.org/observations/100399770
4624	Bubulcus ibis	Cattle Egret	2021/11/10	1	0	25.0077099	121.5904230	https://www.inaturalist.org/observations/101275610

4625	Bubulcus ibis	Cattle Egret	2022/4/17	1	0	25.0080544	121.5292940	https://www.inaturalist.org/observations/115309793
4626	Bubulcus ibis	Cattle Egret	2020/3/11	2	0	25.0091730	121.4912580	https://www.inaturalist.org/observations/40165941
4627	Bubulcus ibis	Cattle Egret	2022/11/19	1	0	25.0094675	121.6217000	https://www.inaturalist.org/observations/142345598
4628	Bubulcus ibis	Cattle Egret	2019/9/20	1	0	25.0099025	121.5316517	https://www.inaturalist.org/observations/33075570
4629	Bubulcus ibis	Cattle Egret	2019/9/20	2	0	25.0099563	121.5319440	https://www.inaturalist.org/observations/33075578
4630	Bubulcus ibis	Cattle Egret	2022/10/19	2	0	25.0101028	121.5318919	https://www.inaturalist.org/observations/141447206
4631	Bubulcus ibis	Cattle Egret	2020/2/10	0	0	25.0105880	121.5920171	https://www.inaturalist.org/observations/38713312
4632	Bubulcus ibis	Cattle Egret	2020/2/10	0	0	25.0105880	121.5920171	https://www.inaturalist.org/observations/38713313
4633	Bubulcus ibis	Cattle Egret	2019/9/23	1	0	25.0108727	121.5283775	https://www.inaturalist.org/observations/33248439
4634	Bubulcus ibis	Cattle Egret	2019/5/26	1	0	25.0112862	98.4532197	https://www.inaturalist.org/observations/36405450
4635	Bubulcus ibis	Cattle Egret	2022/12/8	0	0	25.0119371	121.2230982	https://www.inaturalist.org/observations/143913592
4636	Bubulcus ibis	Cattle Egret	2020/4/30	1	0	25.0141656	121.4681628	https://www.inaturalist.org/observations/61459053
4637	Bubulcus ibis	Cattle Egret	2021/12/4	1	0	25.0153628	121.9231214	https://www.inaturalist.org/observations/104770968
4638	Bubulcus ibis	Cattle Egret	2020/7/24	2	0	25.0153667	121.5382383	https://www.inaturalist.org/observations/54153018
4639	Bubulcus ibis	Cattle Egret	2021/12/13	1	0	25.0155715	121.9183244	https://www.inaturalist.org/observations/103051054
4640	Bubulcus ibis	Cattle Egret	2009/5/31	2	0	25.0160000	98.4660000	https://www.inaturalist.org/observations/106145140
4641	Bubulcus ibis	Cattle Egret	2018/8/20	4	0	25.0160269	121.5397382	https://www.inaturalist.org/observations/20433740
4642	Bubulcus ibis	Cattle Egret	2020/4/24	4	0	25.0160606	121.3419446	https://www.inaturalist.org/observations/43033733
4643	Bubulcus ibis	Cattle Egret	2020/11/7	1	0	25.0163531	121.9241364	https://www.inaturalist.org/observations/64453433
4644	Bubulcus ibis	Cattle Egret	2020/4/29	1	0	25.0168761	121.5538394	https://www.inaturalist.org/observations/44205092
4645	Bubulcus ibis	Cattle Egret	2019/4/11	4	0	25.0169615	121.5203104	https://www.inaturalist.org/observations/22302785
4646	Bubulcus ibis	Cattle Egret	2022/10/21	1	0	25.0170556	121.5337240	https://www.inaturalist.org/observations/143865179
4647	Bubulcus ibis	Cattle Egret	2020/1/23	1	0	25.0173405	121.5397518	https://www.inaturalist.org/observations/38144914
4648	Bubulcus ibis	Cattle Egret	2021/7/21	2	0	25.0174472	121.4910050	https://www.inaturalist.org/observations/88125610
4649	Bubulcus ibis	Cattle Egret	2022/4/28	2	0	25.0176465	121.4921155	https://www.inaturalist.org/observations/113203876

4650	Bubulcus ibis	Cattle Egret	2021/10/10	1	0	25.0177725	121.9268616	https://www.inaturalist.org/observations/104103185
4651	Bubulcus ibis	Cattle Egret	2019/10/14	2	0	25.0178799	121.5375364	https://www.inaturalist.org/observations/36702885
4652	Bubulcus ibis	Cattle Egret	2021/4/11	1	0	25.0179078	121.9235144	https://www.inaturalist.org/observations/73718678
4653	Bubulcus ibis	Cattle Egret	2022/6/19	1	0	25.0179593	121.5178668	https://www.inaturalist.org/observations/122432210
4654	Bubulcus ibis	Cattle Egret	2011/12/18	1	0	25.0186671	121.9244101	https://www.inaturalist.org/observations/55987552
4655	Bubulcus ibis	Cattle Egret	2022/11/13	1	0	25.0188603	121.9212331	https://www.inaturalist.org/observations/142273859
4656	Bubulcus ibis	Cattle Egret	2020/12/2	1	0	25.0190388	121.5373467	https://www.inaturalist.org/observations/65992318
4657	Bubulcus ibis	Cattle Egret	2020/12/2	1	0	25.0190555	121.5373467	https://www.inaturalist.org/observations/65992331
4658	Bubulcus ibis	Cattle Egret	2021/5/15	2	0	25.0191111	121.5371861	https://www.inaturalist.org/observations/79768948
4659	Bubulcus ibis	Cattle Egret	2021/4/30	1	0	25.0196638	121.5373617	https://www.inaturalist.org/observations/76586663
4660	Bubulcus ibis	Cattle Egret	2022/5/18	1	0	25.0197182	121.5171539	https://www.inaturalist.org/observations/122583749
4661	Bubulcus ibis	Cattle Egret	2022/9/15	3	0	25.0197862	121.5373700	https://www.inaturalist.org/observations/136393004
4662	Bubulcus ibis	Cattle Egret	2019/3/12	1	0	25.0197943	121.5381804	https://www.inaturalist.org/observations/48523002
4663	Bubulcus ibis	Cattle Egret	2020/5/31	1	0	25.0198756	121.9244019	https://www.inaturalist.org/observations/47967487
4664	Bubulcus ibis	Cattle Egret	2020/12/19	1	0	25.0198767	121.5378183	https://www.inaturalist.org/observations/66769195
4665	Bubulcus ibis	Cattle Egret	2021/2/16	2	0	25.0199083	121.5377417	https://www.inaturalist.org/observations/69708679
4666	Bubulcus ibis	Cattle Egret	2022/6/12	1	0	25.0199169	121.5373988	https://www.inaturalist.org/observations/121352716
4667	Bubulcus ibis	Cattle Egret	2020/11/6	2	0	25.0199433	121.5370567	https://www.inaturalist.org/observations/64289960
4668	Bubulcus ibis	Cattle Egret	2022/9/29	2	0	25.0199555	121.5377800	https://www.inaturalist.org/observations/136892654
4669	Bubulcus ibis	Cattle Egret	2021/2/16	2	0	25.0199667	121.5376200	https://www.inaturalist.org/observations/69708687
4670	Bubulcus ibis	Cattle Egret	2022/10/6	2	0	25.0200003	121.5377347	https://www.inaturalist.org/observations/137726792
4671	Bubulcus ibis	Cattle Egret	2020/2/9	1	0	25.0200432	121.5376094	https://www.inaturalist.org/observations/41212882
4672	Bubulcus ibis	Cattle Egret	2019/7/7	1	0	25.0201250	121.5376967	https://www.inaturalist.org/observations/49036710
4673	Bubulcus ibis	Cattle Egret	2020/7/18	1	0	25.0202083	121.5374133	https://www.inaturalist.org/observations/53452327
4674	Bubulcus ibis	Cattle Egret	2021/12/13	1	0	25.0202333	121.5374833	https://www.inaturalist.org/observations/102969851

4675	Bubulcus ibis	Cattle Egret	2020/9/15	2	0	25.0202917	121.5380083	https://www.inaturalist.org/observations/60949287
4676	Bubulcus ibis	Cattle Egret	2020/6/19	2	0	25.0202951	121.5370649	https://www.inaturalist.org/observations/50242003
4677	Bubulcus ibis	Cattle Egret	2022/10/26	2	0	25.0203080	121.5377160	https://www.inaturalist.org/observations/140507276
4678	Bubulcus ibis	Cattle Egret	2022/10/26	2	0	25.0203080	121.5377160	https://www.inaturalist.org/observations/140507277
4679	Bubulcus ibis	Cattle Egret	2022/10/26	2	0	25.0203080	121.5377160	https://www.inaturalist.org/observations/140507278
4680	Bubulcus ibis	Cattle Egret	2022/10/26	2	0	25.0203080	121.5377160	https://www.inaturalist.org/observations/140507280
4681	Bubulcus ibis	Cattle Egret	2022/10/26	2	0	25.0203080	121.5377160	https://www.inaturalist.org/observations/140507282
4682	Bubulcus ibis	Cattle Egret	2022/3/31	2	0	25.0203333	121.5381383	https://www.inaturalist.org/observations/109995615
4683	Bubulcus ibis	Cattle Egret	2018/5/30	2	0	25.0203806	121.5372778	https://www.inaturalist.org/observations/13055559
4684	Bubulcus ibis	Cattle Egret	2021/2/21	1	0	25.0203862	121.5378500	https://www.inaturalist.org/observations/69911940
4685	Bubulcus ibis	Cattle Egret	2022/1/18	1	0	25.0204340	121.5376962	https://www.inaturalist.org/observations/121402808
4686	Bubulcus ibis	Cattle Egret	2022/9/5	1	0	25.0204384	121.5212704	https://www.inaturalist.org/observations/133735313
4687	Bubulcus ibis	Cattle Egret	2021/2/6	1	0	25.0204610	121.5379582	https://www.inaturalist.org/observations/69132929
4688	Bubulcus ibis	Cattle Egret	2022/4/4	2	0	25.0204620	121.5379333	https://www.inaturalist.org/observations/110377604
4689	Bubulcus ibis	Cattle Egret	2021/3/27	1	0	25.0204722	121.5379867	https://www.inaturalist.org/observations/72143346
4690	Bubulcus ibis	Cattle Egret	2021/3/27	1	0	25.0204917	121.5379950	https://www.inaturalist.org/observations/72143326
4691	Bubulcus ibis	Cattle Egret	2019/7/24	3	0	25.0205383	121.5371617	https://www.inaturalist.org/observations/29501769
4692	Bubulcus ibis	Cattle Egret	2022/9/23	1	0	25.0205778	121.5379117	https://www.inaturalist.org/observations/136558794
4693	Bubulcus ibis	Cattle Egret	2021/12/4	1	0	25.0207550	121.9227289	https://www.inaturalist.org/observations/104772485
4694	Bubulcus ibis	Cattle Egret	2022/2/19	1	0	25.0207612	121.5371500	https://www.inaturalist.org/observations/124250575
4695	Bubulcus ibis	Cattle Egret	2021/3/26	3	0	25.0207901	121.5376012	https://www.inaturalist.org/observations/72074305
4696	Bubulcus ibis	Cattle Egret	2021/10/27	3	0	25.0209388	121.5377117	https://www.inaturalist.org/observations/99525556
4697	Bubulcus ibis	Cattle Egret	2022/8/11	2	0	25.0209638	121.5566417	https://www.inaturalist.org/observations/130362833
4698	Bubulcus ibis	Cattle Egret	2021/5/3	1	0	25.0211668	121.5551359	https://www.inaturalist.org/observations/76902622
4699	Bubulcus ibis	Cattle Egret	2022/2/11	1	0	25.0216136	121.5076081	https://www.inaturalist.org/observations/106549592

4700	Bubulcus ibis	Cattle Egret	2018/9/5	2	0	25.0216491	121.5046322	https://www.inaturalist.org/observations/16220900
4701	Bubulcus ibis	Cattle Egret	2019/4/28	3	0	25.0217358	121.5054628	https://www.inaturalist.org/observations/23540319
4702	Bubulcus ibis	Cattle Egret	2021/12/4	1	0	25.0217403	121.9231181	https://www.inaturalist.org/observations/104772481
4703	Bubulcus ibis	Cattle Egret	2020/4/25	2	0	25.0222144	121.5145347	https://www.inaturalist.org/observations/43264246
4704	Bubulcus ibis	Cattle Egret	2022/2/11	2	0	25.0224158	121.5134431	https://www.inaturalist.org/observations/106597203
4705	Bubulcus ibis	Cattle Egret	2022/3/6	1	0	25.0224330	121.5135108	https://www.inaturalist.org/observations/108103938
4706	Bubulcus ibis	Cattle Egret	2020/7/5	1	0	25.0224948	121.5060164	https://www.inaturalist.org/observations/51981814
4707	Bubulcus ibis	Cattle Egret	2018/9/16	1	0	25.0225604	121.5048841	https://www.inaturalist.org/observations/16577348
4708	Bubulcus ibis	Cattle Egret	2022/7/26	3	0	25.0227242	121.5056725	https://www.inaturalist.org/observations/127949201
4709	Bubulcus ibis	Cattle Egret	2022/10/20	1	0	25.0227972	121.5370717	https://www.inaturalist.org/observations/139504007
4710	Bubulcus ibis	Cattle Egret	2022/6/25	2	0	25.0229793	121.5042655	https://www.inaturalist.org/observations/123315484
4711	Bubulcus ibis	Cattle Egret	2020/12/19	2	0	25.0230556	121.5052778	https://www.inaturalist.org/observations/67078729
4712	Bubulcus ibis	Cattle Egret	2018/7/4	4	0	25.0233510	121.5056968	https://www.inaturalist.org/observations/14033279
4713	Bubulcus ibis	Cattle Egret	2021/11/13	2	0	25.0243122	121.5051099	https://www.inaturalist.org/observations/100985068
4714	Bubulcus ibis	Cattle Egret	2019/7/10	2	0	25.0243612	121.5459788	https://www.inaturalist.org/observations/28544424
4715	Bubulcus ibis	Cattle Egret	2021/7/13	2	0	25.0244055	121.5053467	https://www.inaturalist.org/observations/86852763
4716	Bubulcus ibis	Cattle Egret	2021/4/9	1	0	25.0244095	121.5061899	https://www.inaturalist.org/observations/73371978
4717	Bubulcus ibis	Cattle Egret	2019/11/10	3	0	25.0244400	121.5039933	https://www.inaturalist.org/observations/35526901
4718	Bubulcus ibis	Cattle Egret	2019/11/10	2	0	25.0245300	121.5065800	https://www.inaturalist.org/observations/35526929
4719	Bubulcus ibis	Cattle Egret	2022/9/10	5	0	25.0245736	121.5049620	https://www.inaturalist.org/observations/134556483
4720	Bubulcus ibis	Cattle Egret	2021/10/6	1	0	25.0245896	121.5066268	https://www.inaturalist.org/observations/97567577
4721	Bubulcus ibis	Cattle Egret	2019/10/26	2	0	25.0246100	121.5039817	https://www.inaturalist.org/observations/35081950
4722	Bubulcus ibis	Cattle Egret	2022/11/14	2	0	25.0246484	121.3036543	https://www.inaturalist.org/observations/142663612
4723	Bubulcus ibis	Cattle Egret	2021/11/13	1	0	25.0246704	121.5062664	https://www.inaturalist.org/observations/100984992
4724	Bubulcus ibis	Cattle Egret	2019/7/7	2	0	25.0247803	121.5075449	https://www.inaturalist.org/observations/28350118

4725	Bubulcus ibis	Cattle Egret	2018/8/3	2	0	25.0248750	121.5057367	https://www.inaturalist.org/observations/15067676
4726	Bubulcus ibis	Cattle Egret	2021/11/13	1	0	25.0252415	121.5072247	https://www.inaturalist.org/observations/100984566
4727	Bubulcus ibis	Cattle Egret	2018/10/13	4	0	25.0253868	121.5073929	https://www.inaturalist.org/observations/17459113
4728	Bubulcus ibis	Cattle Egret	2020/7/13	1	0	25.0259305	121.5357133	https://www.inaturalist.org/observations/53018587
4729	Bubulcus ibis	Cattle Egret	2020/7/12	1	0	25.0266778	121.5362333	https://www.inaturalist.org/observations/52766819
4730	Bubulcus ibis	Cattle Egret	2022/9/20	1	0	25.0267639	121.5345839	https://www.inaturalist.org/observations/135731681
4731	Bubulcus ibis	Cattle Egret	2022/8/1	2	0	25.0269051	121.5347596	https://www.inaturalist.org/observations/128917916
4732	Bubulcus ibis	Cattle Egret	2020/2/13	0	0	25.0270760	121.5342584	https://www.inaturalist.org/observations/39039112
4733	Bubulcus ibis	Cattle Egret	2020/5/23	1	0	25.0271695	121.5362333	https://www.inaturalist.org/observations/46934566
4734	Bubulcus ibis	Cattle Egret	2022/10/26	2	0	25.0271702	121.5372238	https://www.inaturalist.org/observations/140030923
4735	Bubulcus ibis	Cattle Egret	2022/3/9	2	0	25.0276589	121.2958000	https://www.inaturalist.org/observations/112520175
4736	Bubulcus ibis	Cattle Egret	2022/8/24	2	0	25.0282083	121.5347806	https://www.inaturalist.org/observations/132112885
4737	Bubulcus ibis	Cattle Egret	2017/7/7	2	0	25.0282224	121.5358179	https://www.inaturalist.org/observations/105322184
4738	Bubulcus ibis	Cattle Egret	2020/3/24	2	0	25.0284789	121.5353288	https://www.inaturalist.org/observations/105972854
4739	Bubulcus ibis	Cattle Egret	2018/11/24	3	0	25.0288306	121.5358417	https://www.inaturalist.org/observations/18594150
4740	Bubulcus ibis	Cattle Egret	2018/9/23	3	0	25.0289943	121.5350482	https://www.inaturalist.org/observations/16824145
4741	Bubulcus ibis	Cattle Egret	2020/10/18	1	0	25.0290868	121.5366797	https://www.inaturalist.org/observations/62864717
4742	Bubulcus ibis	Cattle Egret	2017/3/29	1	0	25.0292030	121.9295743	https://www.inaturalist.org/observations/81720211
4743	Bubulcus ibis	Cattle Egret	2018/5/30	2	0	25.0293167	121.5349972	https://www.inaturalist.org/observations/13055545
4744	Bubulcus ibis	Cattle Egret	2022/5/22	1	0	25.0293436	121.5355021	https://www.inaturalist.org/observations/118147233
4745	Bubulcus ibis	Cattle Egret	2020/7/2	1	0	25.0293805	121.3659967	https://www.inaturalist.org/observations/51698934
4746	Bubulcus ibis	Cattle Egret	2020/8/7	1	0	25.0296500	121.5368528	https://www.inaturalist.org/observations/55673006
4747	Bubulcus ibis	Cattle Egret	2022/7/23	4	0	25.0297195	121.5358800	https://www.inaturalist.org/observations/127472928
4748	Bubulcus ibis	Cattle Egret	2019/12/24	1	0	25.0301199	121.5360118	https://www.inaturalist.org/observations/36912313
4749	Bubulcus ibis	Cattle Egret	2017/12/11	3	0	25.0302386	121.5346822	https://www.inaturalist.org/observations/14446361

4750	Bubulcus ibis	Cattle Egret	2019/9/2	2	0	25.0302486	121.5357277	https://www.inaturalist.org/observations/32098446
4751	Bubulcus ibis	Cattle Egret	2021/9/28	1	0	25.0303148	121.5369549	https://www.inaturalist.org/observations/96595266
4752	Bubulcus ibis	Cattle Egret	2020/4/21	1	0	25.0304324	121.5369143	https://www.inaturalist.org/observations/42731809
4753	Bubulcus ibis	Cattle Egret	2019/9/15	3	0	25.0305126	121.5360486	https://www.inaturalist.org/observations/32753099
4754	Bubulcus ibis	Cattle Egret	2020/3/15	2	0	25.0307342	121.5339731	https://www.inaturalist.org/observations/40011464
4755	Bubulcus ibis coromandus	Cattle Egret	2018/11/29	4	0	25.0307778	121.5352472	https://www.inaturalist.org/observations/19151024
4756	Bubulcus ibis	Cattle Egret	2022/9/7	1	0	25.0309119	121.5359387	https://www.inaturalist.org/observations/137354695
4757	Bubulcus ibis	Cattle Egret	2020/1/25	0	0	25.0309150	121.5349050	https://www.inaturalist.org/observations/38237123
4758	Bubulcus ibis	Cattle Egret	2019/12/7	1	0	25.0309322	121.5347290	https://www.inaturalist.org/observations/36424698
4759	Bubulcus ibis	Cattle Egret	2021/9/21	1	0	25.0309799	121.5349807	https://www.inaturalist.org/observations/95659396
4760	Bubulcus ibis	Cattle Egret	2021/9/21	1	0	25.0309800	121.5349800	https://www.inaturalist.org/observations/95659394
4761	Bubulcus ibis	Cattle Egret	2022/10/2	2	0	25.0309846	121.5352397	https://www.inaturalist.org/observations/137250748
4762	Bubulcus ibis	Cattle Egret	2021/9/21	1	0	25.0309866	121.5349766	https://www.inaturalist.org/observations/95659395
4763	Bubulcus ibis	Cattle Egret	2021/9/1	1	0	25.0310083	121.5350367	https://www.inaturalist.org/observations/93308068
4764	Bubulcus ibis	Cattle Egret	2022/5/4	2	0	25.0310254	121.5361392	https://www.inaturalist.org/observations/116526960
4765	Bubulcus ibis	Cattle Egret	2022/5/4	2	0	25.0310254	121.5361392	https://www.inaturalist.org/observations/116526964
4766	Bubulcus ibis	Cattle Egret	2021/8/23	1	0	25.0310382	121.5355015	https://www.inaturalist.org/observations/94730907
4767	Bubulcus ibis	Cattle Egret	2020/10/27	1	0	25.0310474	121.5348358	https://www.inaturalist.org/observations/64165535
4768	Bubulcus ibis	Cattle Egret	2019/1/3	3	0	25.0310489	121.5348567	https://www.inaturalist.org/observations/19389999
4769	Bubulcus ibis	Cattle Egret	2020/3/22	1	0	25.0310516	121.5348558	https://www.inaturalist.org/observations/40637364
4770	Bubulcus ibis	Cattle Egret	2019/2/6	3	0	25.0310528	121.5350200	https://www.inaturalist.org/observations/20150495
4771	Bubulcus ibis	Cattle Egret	2017/4/25	1	0	25.0310587	121.5356475	https://www.inaturalist.org/observations/94729175
4772	Bubulcus ibis coromandus	Cattle Egret	2018/5/25	4	0	25.0310614	121.5350314	https://www.inaturalist.org/observations/12797954
4773	Bubulcus ibis	Cattle Egret	2020/2/13	0	0	25.0310691	121.5345284	https://www.inaturalist.org/observations/39039471
4774	Bubulcus ibis	Cattle Egret	2018/12/30	3	0	25.0310771	121.5349972	https://www.inaturalist.org/observations/19351829

4775	Bubulcus ibis	Cattle Egret	2019/1/3	3	0	25.0310784	121.5346579	https://www.inaturalist.org/observations/19389975
4776	Bubulcus ibis	Cattle Egret	2021/9/7	1	0	25.0310841	121.5342783	https://www.inaturalist.org/observations/93974254
4777	Bubulcus ibis	Cattle Egret	2018/6/2	3	0	25.0310898	121.5346216	https://www.inaturalist.org/observations/13034036
4778	Bubulcus ibis	Cattle Egret	2018/12/30	4	0	25.0310943	121.5349022	https://www.inaturalist.org/observations/19351834
4779	Bubulcus ibis	Cattle Egret	2019/1/26	1	0	25.0310983	121.5351759	https://www.inaturalist.org/observations/94729536
4780	Bubulcus ibis	Cattle Egret	2018/5/5	2	0	25.0311111	121.5347222	https://www.inaturalist.org/observations/113828893
4781	Bubulcus ibis	Cattle Egret	2021/4/18	3	0	25.0311167	121.5347883	https://www.inaturalist.org/observations/74353124
4782	Bubulcus ibis	Cattle Egret	2019/9/29	2	0	25.0311266	121.5370834	https://www.inaturalist.org/observations/34192885
4783	Bubulcus ibis	Cattle Egret	2022/7/23	5	0	25.0311472	121.5329750	https://www.inaturalist.org/observations/128411633
4784	Bubulcus ibis	Cattle Egret	2021/9/18	2	0	25.0311506	121.5341716	https://www.inaturalist.org/observations/97130728
4785	Bubulcus ibis	Cattle Egret	2020/3/14	1	0	25.0311623	121.5349503	https://www.inaturalist.org/observations/43533095
4786	Bubulcus ibis	Cattle Egret	2018/9/23	4	0	25.0311999	121.5346928	https://www.inaturalist.org/observations/16824508
4787	Bubulcus ibis	Cattle Egret	2020/4/16	3	0	25.0312628	121.5349492	https://www.inaturalist.org/observations/71311438
4788	Bubulcus ibis	Cattle Egret	2021/10/12	2	0	25.0313050	121.5349640	https://www.inaturalist.org/observations/97986142
4789	Bubulcus ibis	Cattle Egret	2016/5/9	2	0	25.0313065	121.5348155	https://www.inaturalist.org/observations/67911158
4790	Bubulcus ibis	Cattle Egret	2018/5/29	2	0	25.0313110	121.5345329	https://www.inaturalist.org/observations/12940122
4791	Bubulcus ibis	Cattle Egret	2022/10/4	1	0	25.0313193	121.5353328	https://www.inaturalist.org/observations/137519133
4792	Bubulcus ibis	Cattle Egret	2021/6/17	1	0	25.0313501	121.5348493	https://www.inaturalist.org/observations/83385447
4793	Bubulcus ibis	Cattle Egret	2021/5/2	1	0	25.0313528	121.5339367	https://www.inaturalist.org/observations/76976570
4794	Bubulcus ibis	Cattle Egret	2021/3/18	1	0	25.0313618	121.5345281	https://www.inaturalist.org/observations/79497033
4795	Bubulcus ibis	Cattle Egret	2019/4/12	1	0	25.0313680	121.5350092	https://www.inaturalist.org/observations/22401496
4796	Bubulcus ibis	Cattle Egret	2021/6/17	2	0	25.0314050	121.5348516	https://www.inaturalist.org/observations/83385297
4797	Bubulcus ibis	Cattle Egret	2020/4/26	1	0	25.0314487	121.5336104	https://www.inaturalist.org/observations/43550364
4798	Bubulcus ibis	Cattle Egret	2019/10/17	1	0	25.0314631	121.5352373	https://www.inaturalist.org/observations/34956664
4799	Bubulcus ibis	Cattle Egret	2021/7/4	1	0	25.0314692	121.5349990	https://www.inaturalist.org/observations/85583489

4800	Bubulcus ibis	Cattle Egret	2021/8/22	1	0	25.0315042	121.5347418	https://www.inaturalist.org/observations/92574330
4801	Bubulcus ibis	Cattle Egret	2021/8/22	2	0	25.0315042	121.5347418	https://www.inaturalist.org/observations/92574340
4802	Bubulcus ibis	Cattle Egret	2019/6/2	3	0	25.0315098	121.5349676	https://www.inaturalist.org/observations/26286615
4803	Bubulcus ibis	Cattle Egret	2019/2/28	1	0	25.0315252	121.5351149	https://www.inaturalist.org/observations/32211116
4804	Bubulcus ibis coromandus	Cattle Egret	2019/11/2	1	0	25.0315371	121.5342822	https://www.inaturalist.org/observations/35188062
4805	Bubulcus ibis	Cattle Egret	2022/2/28	1	0	25.0316055	121.5343550	https://www.inaturalist.org/observations/107629255
4806	Bubulcus ibis	Cattle Egret	2020/9/12	2	0	25.0316485	121.5353031	https://www.inaturalist.org/observations/59438453
4807	Bubulcus ibis	Cattle Egret	2022/11/11	2	0	25.0316732	121.5343352	https://www.inaturalist.org/observations/141679508
4808	Bubulcus ibis	Cattle Egret	2022/2/26	1	0	25.0317121	121.5343896	https://www.inaturalist.org/observations/107483856
4809	Bubulcus ibis	Cattle Egret	2018/6/9	2	0	25.0317146	121.5348474	https://www.inaturalist.org/observations/13253881
4810	Bubulcus ibis	Cattle Egret	2021/6/13	2	0	25.0317167	121.5341543	https://www.inaturalist.org/observations/82813854
4811	Bubulcus ibis	Cattle Egret	2018/5/28	3	0	25.0317217	121.5347797	https://www.inaturalist.org/observations/12922266
4812	Bubulcus ibis	Cattle Egret	2019/12/21	1	0	25.0317850	121.5343733	https://www.inaturalist.org/observations/122151031
4813	Bubulcus ibis	Cattle Egret	2022/3/26	1	0	25.0317865	121.5369210	https://www.inaturalist.org/observations/109522659
4814	Bubulcus ibis	Cattle Egret	2019/3/19	2	0	25.0318226	121.5353238	https://www.inaturalist.org/observations/21384064
4815	Bubulcus ibis	Cattle Egret	2020/9/1	1	0	25.0318806	121.5342556	https://www.inaturalist.org/observations/58226387
4816	Bubulcus ibis	Cattle Egret	2018/7/28	2	0	25.0319550	121.5345529	https://www.inaturalist.org/observations/14923394
4817	Bubulcus ibis	Cattle Egret	2018/7/28	2	0	25.0319574	121.5345475	https://www.inaturalist.org/observations/14923395
4818	Bubulcus ibis	Cattle Egret	2022/4/23	2	0	25.0320050	121.5345233	https://www.inaturalist.org/observations/112511299
4819	Bubulcus ibis	Cattle Egret	2022/8/24	1	0	25.0320389	121.5334139	https://www.inaturalist.org/observations/132112791
4820	Bubulcus ibis	Cattle Egret	2020/2/8	0	0	25.0320550	121.5348860	https://www.inaturalist.org/observations/38838352
4821	Bubulcus ibis	Cattle Egret	2022/11/22	2	0	25.0320612	121.5347133	https://www.inaturalist.org/observations/142586220
4822	Bubulcus ibis	Cattle Egret	2021/9/21	1	0	25.0320938	121.5350746	https://www.inaturalist.org/observations/95659398
4823	Bubulcus ibis	Cattle Egret	2022/8/27	2	0	25.0321083	121.5337450	https://www.inaturalist.org/observations/135668332
4824	Bubulcus ibis	Cattle Egret	2021/3/17	2	0	25.0321293	121.5350869	https://www.inaturalist.org/observations/77726380

4825	Bubulcus ibis	Cattle Egret	2022/7/23	2	0	25.0321778	121.5346750	https://www.inaturalist.org/observations/127448235
4826	Bubulcus ibis	Cattle Egret	2021/9/18	2	0	25.0322789	121.5344972	https://www.inaturalist.org/observations/97130727
4827	Bubulcus ibis	Cattle Egret	2021/5/8	2	0	25.0324347	121.5349054	https://www.inaturalist.org/observations/78335761
4828	Bubulcus ibis	Cattle Egret	2020/4/25	1	0	25.0324350	121.5349054	https://www.inaturalist.org/observations/43607942
4829	Bubulcus ibis	Cattle Egret	2019/3/21	1	0	25.0324350	121.5349054	https://www.inaturalist.org/observations/65111927
4830	Bubulcus ibis	Cattle Egret	2019/1/25	1	0	25.0324350	121.5349054	https://www.inaturalist.org/observations/82639147
4831	Bubulcus ibis coromandus	Cattle Egret	2021/8/22	1	0	25.0324350	121.5349054	https://www.inaturalist.org/observations/92678066
4832	Bubulcus ibis	Cattle Egret	2021/10/12	3	0	25.0325021	121.5344483	https://www.inaturalist.org/observations/97985808
4833	Bubulcus ibis	Cattle Egret	2020/9/2	1	0	25.0325310	121.5800718	https://www.inaturalist.org/observations/63688893
4834	Bubulcus ibis	Cattle Egret	2021/6/15	1	0	25.0326205	121.4588944	https://www.inaturalist.org/observations/83226365
4835	Bubulcus ibis	Cattle Egret	2021/10/12	3	0	25.0328353	121.5350765	https://www.inaturalist.org/observations/97985715
4836	Bubulcus ibis	Cattle Egret	2018/5/30	2	0	25.0328528	121.5336222	https://www.inaturalist.org/observations/13055549
4837	Bubulcus ibis	Cattle Egret	2016/5/31	5	0	25.0329636	121.5654268	http://www.inaturalist.org/observations/4449826
4838	Bubulcus ibis	Cattle Egret	2022/8/14	1	0	25.0329636	121.5654268	https://www.inaturalist.org/observations/130788325
4839	Bubulcus ibis	Cattle Egret	2022/5/6	1	0	25.0329945	121.5336833	https://www.inaturalist.org/observations/115694604
4840	Bubulcus ibis	Cattle Egret	2020/9/29	1	0	25.0330407	121.5349426	https://www.inaturalist.org/observations/63149485
4841	Bubulcus ibis	Cattle Egret	2022/4/21	3	0	25.0331695	121.5340208	https://www.inaturalist.org/observations/112248621
4842	Bubulcus ibis	Cattle Egret	2021/9/17	2	0	25.0331758	121.5342201	https://www.inaturalist.org/observations/95114005
4843	Bubulcus ibis	Cattle Egret	2021/12/18	1	0	25.0332398	121.5355371	https://www.inaturalist.org/observations/103572842
4844	Bubulcus ibis	Cattle Egret	2020/12/29	2	0	25.0332452	121.5359325	https://www.inaturalist.org/observations/67498508
4845	Bubulcus ibis	Cattle Egret	2021/5/12	1	0	25.0333549	121.5358874	https://www.inaturalist.org/observations/78535745
4846	Bubulcus ibis	Cattle Egret	2020/11/9	1	0	25.0333919	121.5361557	https://www.inaturalist.org/observations/64508181
4847	Bubulcus ibis	Cattle Egret	2018/2/14	2	0	25.0334317	121.5654175	https://www.inaturalist.org/observations/61791230
4848	Bubulcus ibis coromandus	Cattle Egret	2019/10/30	3	0	25.0334358	121.5336075	https://www.inaturalist.org/observations/36738084
4849	Bubulcus ibis	Cattle Egret	2021/4/14	1	0	25.0334949	121.5808411	https://www.inaturalist.org/observations/73922524

4850	Bubulcus ibis	Cattle Egret	2021/6/30	1	0	25.0340112	121.4823383	https://www.inaturalist.org/observations/85050844
4851	Bubulcus ibis	Cattle Egret	2021/1/28	1	0	25.0344982	121.5350571	https://www.inaturalist.org/observations/68712913
4852	Bubulcus ibis	Cattle Egret	2022/3/20	2	0	25.0350313	121.4844438	https://www.inaturalist.org/observations/109844912
4853	Bubulcus ibis	Cattle Egret	2020/3/11	2	0	25.0351630	121.4823810	https://www.inaturalist.org/observations/40165952
4854	Bubulcus ibis coromandus	Cattle Egret	2012/3/21	3	0	25.0357880	121.5216626	https://www.inaturalist.org/observations/19822247
4855	Bubulcus ibis	Cattle Egret	2021/5/27	1	0	25.0362028	121.4819717	https://www.inaturalist.org/observations/82779158
4856	Bubulcus ibis	Cattle Egret	2012/7/7	2	0	25.0364500	121.5197000	https://www.inaturalist.org/observations/10429933
4857	Bubulcus ibis	Cattle Egret	2012/7/7	2	0	25.0364500	121.5197000	https://www.inaturalist.org/observations/124432259
4858	Bubulcus ibis	Cattle Egret	2021/9/24	1	0	25.0366380	121.4907191	https://www.inaturalist.org/observations/98800936
4859	Bubulcus ibis	Cattle Egret	2019/10/7	1	0	25.0371590	121.4977670	https://www.inaturalist.org/observations/34068087
4860	Bubulcus ibis	Cattle Egret	2020/4/17	1	0	25.0372160	121.5103269	https://www.inaturalist.org/observations/61527839
4861	Bubulcus ibis	Cattle Egret	2018/12/18	4	0	25.0377888	121.4914121	https://www.inaturalist.org/observations/19201113
4862	Bubulcus ibis	Cattle Egret	2020/11/14	1	0	25.0379117	121.2620760	https://www.inaturalist.org/observations/68752395
4863	Bubulcus ibis	Cattle Egret	2022/10/11	2	0	25.0383106	121.4941478	https://www.inaturalist.org/observations/138610862
4864	Bubulcus ibis	Cattle Egret	2018/12/29	3	0	25.0383641	121.4914895	https://www.inaturalist.org/observations/19837935
4865	Bubulcus ibis	Cattle Egret	2018/11/11	4	0	25.0383641	121.4914895	https://www.inaturalist.org/observations/19984070
4866	Bubulcus ibis	Cattle Egret	2017/7/28	1	0	25.0384131	121.4912955	https://www.inaturalist.org/observations/65978781
4867	Bubulcus ibis	Cattle Egret	2018/11/24	4	0	25.0385433	121.4945617	https://www.inaturalist.org/observations/18623078
4868	Bubulcus ibis	Cattle Egret	2019/11/10	1	0	25.0386602	121.4943160	https://www.inaturalist.org/observations/36102310
4869	Bubulcus ibis coromandus	Cattle Egret	2020/1/22	3	0	25.0387025	121.4952208	https://www.inaturalist.org/observations/37941164
4870	Bubulcus ibis	Cattle Egret	2019/3/25	3	0	25.0390524	121.4932467	https://www.inaturalist.org/observations/26529953
4871	Bubulcus ibis	Cattle Egret	2017/5/27	1	0	25.0391699	121.8630124	https://www.inaturalist.org/observations/115745051
4872	Bubulcus ibis coromandus	Cattle Egret	2017/4/22	1	0	25.0400870	121.4955160	https://www.inaturalist.org/observations/136567359
4873	Bubulcus ibis	Cattle Egret	2020/4/17	1	0	25.0413304	121.4594610	https://www.inaturalist.org/observations/61543720
4874	Bubulcus ibis	Cattle Egret	2021/4/30	2	0	25.0414362	121.5907750	https://www.inaturalist.org/observations/75772596

4875	Bubulcus ibis	Cattle Egret	2022/7/23	1	0	25.0417720	121.5917830	https://www.inaturalist.org/observations/129140842
4876	Bubulcus ibis	Cattle Egret	2022/10/13	1	0	25.0421121	121.5150042	https://www.inaturalist.org/observations/139441246
4877	Bubulcus ibis	Cattle Egret	2019/10/15	3	0	25.0421852	121.6145477	https://www.inaturalist.org/observations/34404087
4878	Bubulcus ibis	Cattle Egret	2020/2/18	0	0	25.0422267	121.5913201	https://www.inaturalist.org/observations/38793249
4879	Bubulcus ibis	Cattle Egret	2020/9/13	2	0	25.0427620	121.5914549	https://www.inaturalist.org/observations/59417726
4880	Bubulcus ibis	Cattle Egret	2021/1/10	1	0	25.0440980	121.5925654	https://www.inaturalist.org/observations/67832376
4881	Bubulcus ibis	Cattle Egret	2021/8/12	2	0	25.0443460	121.5920244	https://www.inaturalist.org/observations/90868438
4882	Bubulcus ibis	Cattle Egret	2021/8/4	3	0	25.0450499	121.5925247	https://www.inaturalist.org/observations/89881923
4883	Bubulcus ibis	Cattle Egret	2018/3/11	3	0	25.0473313	121.5040742	https://www.inaturalist.org/observations/10200718
4884	Bubulcus ibis	Cattle Egret	2020/7/11	1	0	25.0519840	121.6080464	https://www.inaturalist.org/observations/52644747
4885	Bubulcus ibis	Cattle Egret	2021/9/11	1	0	25.0535917	121.5739283	https://www.inaturalist.org/observations/94400583
4886	Bubulcus ibis	Cattle Egret	2020/3/22	1	0	25.0583833	121.5972967	https://www.inaturalist.org/observations/40498235
4887	Bubulcus ibis	Cattle Egret	2020/4/9	1	0	25.0584862	121.5971450	https://www.inaturalist.org/observations/42563934
4888	Bubulcus ibis	Cattle Egret	2019/11/20	1	0	25.0599623	121.5723202	https://www.inaturalist.org/observations/35878856
4889	Bubulcus ibis	Cattle Egret	2022/5/29	1	0	25.0601055	121.6053300	https://www.inaturalist.org/observations/119220417
4890	Bubulcus ibis	Cattle Egret	2019/11/13	2	0	25.0609183	121.2339967	https://www.inaturalist.org/observations/66140185
4891	Bubulcus ibis	Cattle Egret	2022/5/6	3	0	25.0627777	121.4608440	https://www.inaturalist.org/observations/116314401
4892	Bubulcus ibis	Cattle Egret	2022/5/6	3	0	25.0627869	121.4607518	https://www.inaturalist.org/observations/116314413
4893	Bubulcus ibis	Cattle Egret	2020/10/17	1	0	25.0633722	121.5405417	https://www.inaturalist.org/observations/63479216
4894	Bubulcus ibis	Cattle Egret	2019/8/23	2	0	25.0634445	121.6507550	https://www.inaturalist.org/observations/31321692
4895	Bubulcus ibis	Cattle Egret	2020/10/3	1	0	25.0636079	121.5397264	https://www.inaturalist.org/observations/64023260
4896	Bubulcus ibis	Cattle Egret	2020/5/27	1	0	25.0663839	121.6081951	https://www.inaturalist.org/observations/47478941
4897	Bubulcus ibis	Cattle Egret	2017/2/20	0	0	25.0688665	121.6485231	https://www.inaturalist.org/observations/39045881
4898	Bubulcus ibis	Cattle Egret	2022/12/6	1	0	25.0712566	121.5967139	https://www.inaturalist.org/observations/143796164
4899	Bubulcus ibis	Cattle Egret	2021/12/5	1	0	25.0716168	121.6212408	https://www.inaturalist.org/observations/102521890

4900	Bubulcus ibis	Cattle Egret	2020/6/1	2	0	25.0720750	121.5897133	https://www.inaturalist.org/observations/48101466
4901	Bubulcus ibis	Cattle Egret	2022/7/20	2	0	25.0730050	121.5295580	https://www.inaturalist.org/observations/127088048
4902	Bubulcus ibis	Cattle Egret	2022/8/6	1	0	25.0741587	121.6181481	https://www.inaturalist.org/observations/129677178
4903	Bubulcus ibis	Cattle Egret	2022/11/25	0	0	25.0745817	121.5334550	https://www.inaturalist.org/observations/142799954
4904	Bubulcus ibis	Cattle Egret	2022/4/13	1	0	25.0759528	121.6750444	https://www.inaturalist.org/observations/111261646
4905	Bubulcus ibis	Cattle Egret	2021/11/17	2	0	25.0759833	121.6604000	https://www.inaturalist.org/observations/101388861
4906	Bubulcus ibis	Cattle Egret	2021/8/10	3	0	25.0764112	121.6740283	https://www.inaturalist.org/observations/90631811
4907	Bubulcus ibis	Cattle Egret	2022/4/5	1	0	25.0766810	121.6743106	https://www.inaturalist.org/observations/110473450
4908	Bubulcus ibis	Cattle Egret	2020/7/9	1	0	25.0772874	121.5251127	https://www.inaturalist.org/observations/52451216
4909	Bubulcus ibis	Cattle Egret	2020/10/4	1	0	25.0788050	121.4711198	https://www.inaturalist.org/observations/62995327
4910	Bubulcus ibis	Cattle Egret	2022/10/1	1	0	25.0807733	121.6033723	https://www.inaturalist.org/observations/137304780
4911	Bubulcus ibis	Cattle Egret	2022/4/20	1	0	25.0810017	121.6709101	https://www.inaturalist.org/observations/112122767
4912	Bubulcus ibis	Cattle Egret	2022/5/9	2	0	25.0810017	121.6709101	https://www.inaturalist.org/observations/116214068
4913	Bubulcus ibis	Cattle Egret	2022/5/9	2	0	25.0810419	121.6708190	https://www.inaturalist.org/observations/117898166
4914	Bubulcus ibis	Cattle Egret	2021/9/19	1	0	25.0810524	121.2030961	https://www.inaturalist.org/observations/95567093
4915	Bubulcus ibis	Cattle Egret	2022/4/4	1	0	25.0816043	121.6052766	https://www.inaturalist.org/observations/110494929
4916	Bubulcus ibis	Cattle Egret	2022/2/7	1	0	25.0819990	121.6740684	https://www.inaturalist.org/observations/106404427
4917	Bubulcus ibis	Cattle Egret	2022/4/9	3	0	25.0820206	121.6243691	https://www.inaturalist.org/observations/110838457
4918	Bubulcus ibis	Cattle Egret	2022/2/9	2	0	25.0820278	121.5841917	https://www.inaturalist.org/observations/106494954
4919	Bubulcus ibis	Cattle Egret	2019/6/15	2	0	25.0820555	121.4274583	https://www.inaturalist.org/observations/27008293
4920	Bubulcus ibis coromandus	Cattle Egret	2019/7/4	3	0	25.0822038	121.5855796	https://www.inaturalist.org/observations/28158016
4921	Bubulcus ibis	Cattle Egret	2013/10/14	1	0	25.0822805	121.6044519	https://www.inaturalist.org/observations/70856134
4922	Bubulcus ibis	Cattle Egret	2021/9/1	1	0	25.0823301	121.5828206	https://www.inaturalist.org/observations/93346135
4923	Bubulcus ibis	Cattle Egret	2011/5/22	1	0	25.0828320	121.2000043	https://www.inaturalist.org/observations/55786160
4924	Bubulcus ibis	Cattle Egret	2019/4/16	1	0	25.0832639	121.1866736	https://www.inaturalist.org/observations/22555977

4925	Bubulcus ibis	Cattle Egret	2019/4/16	1	0	25.0832639	121.1866736	https://www.inaturalist.org/observations/22555979
4926	Bubulcus ibis	Cattle Egret	2019/4/16	1	0	25.0832639	121.1866736	https://www.inaturalist.org/observations/22556095
4927	Bubulcus ibis	Cattle Egret	2019/4/18	2	0	25.0832639	121.1866736	https://www.inaturalist.org/observations/22702742
4928	Bubulcus ibis	Cattle Egret	2019/4/18	1	0	25.0832639	121.1866736	https://www.inaturalist.org/observations/22702775
4929	Bubulcus ibis	Cattle Egret	2022/7/10	2	0	25.0833417	121.5819033	https://www.inaturalist.org/observations/125596494
4930	Bubulcus ibis	Cattle Egret	2011/5/8	1	0	25.0835025	121.1985131	https://www.inaturalist.org/observations/55784426
4931	Bubulcus ibis	Cattle Egret	2022/5/28	1	0	25.0835667	121.6838617	https://www.inaturalist.org/observations/119016155
4932	Bubulcus ibis	Cattle Egret	2021/10/26	1	0	25.0836986	121.6790902	https://www.inaturalist.org/observations/99418558
4933	Bubulcus ibis	Cattle Egret	2011/5/15	2	0	25.0839523	121.1995403	https://www.inaturalist.org/observations/55785575
4934	Bubulcus ibis	Cattle Egret	2012/8/4	1	0	25.0841437	121.2003596	https://www.inaturalist.org/observations/56490066
4935	Bubulcus ibis	Cattle Egret	2022/5/18	1	0	25.0843628	121.6630151	https://www.inaturalist.org/observations/117539065
4936	Bubulcus ibis	Cattle Egret	2013/9/7	1	0	25.0844936	121.1995859	https://www.inaturalist.org/observations/56824078
4937	Bubulcus ibis	Cattle Egret	2018/1/11	2	0	25.0850101	121.2013835	https://www.inaturalist.org/observations/9481964
4938	Bubulcus ibis	Cattle Egret	2022/4/27	1	0	25.0851083	121.6630700	https://www.inaturalist.org/observations/113065429
4939	Bubulcus ibis	Cattle Egret	2011/8/7	1	0	25.0852321	121.2004335	https://www.inaturalist.org/observations/55875444
4940	Bubulcus ibis	Cattle Egret	2012/5/26	1	0	25.0856894	121.1965609	https://www.inaturalist.org/observations/56400382
4941	Bubulcus ibis	Cattle Egret	2021/10/15	5	0	25.0867921	121.6812150	https://www.inaturalist.org/observations/98240690
4942	Bubulcus ibis	Cattle Egret	2021/8/22	3	0	25.0870583	121.6811450	https://www.inaturalist.org/observations/92040945
4943	Bubulcus ibis	Cattle Egret	2020/4/25	2	0	25.0871673	121.5131215	https://www.inaturalist.org/observations/43276868
4944	Bubulcus ibis	Cattle Egret	2021/5/11	2	0	25.0873336	121.1762525	https://www.inaturalist.org/observations/78337417
4945	Bubulcus ibis	Cattle Egret	2019/7/1	1	0	25.0883317	121.6215295	https://www.inaturalist.org/observations/27988853
4946	Bubulcus ibis	Cattle Egret	2022/10/13	4	0	25.0893514	121.5982751	https://www.inaturalist.org/observations/138601358
4947	Bubulcus ibis	Cattle Egret	2020/1/12	2	0	25.0894417	121.5983800	https://www.inaturalist.org/observations/37516398
4948	Bubulcus ibis	Cattle Egret	2021/10/28	1	0	25.0897083	121.1811550	https://www.inaturalist.org/observations/100055658
4949	Bubulcus ibis	Cattle Egret	1980/1/1	1	0	25.0907480	121.5821835	https://www.inaturalist.org/observations/24066305

4950	Bubulcus ibis	Cattle Egret	2019/7/31	4	0	25.0908220	121.5814099	https://www.inaturalist.org/observations/30236970
4951	Bubulcus ibis	Cattle Egret	2019/4/7	2	0	25.0908722	121.5817341	https://www.inaturalist.org/observations/22125587
4952	Bubulcus ibis	Cattle Egret	2020/8/22	1	0	25.0909036	121.5832522	https://www.inaturalist.org/observations/58820054
4953	Bubulcus ibis	Cattle Egret	2019/10/18	1	0	25.0909066	121.5824582	https://www.inaturalist.org/observations/40685855
4954	Bubulcus ibis	Cattle Egret	2019/7/25	3	0	25.0909287	121.5825936	https://www.inaturalist.org/observations/31157287
4955	Bubulcus ibis	Cattle Egret	2019/6/17	2	0	25.0909300	121.5824392	https://www.inaturalist.org/observations/27563674
4956	Bubulcus ibis	Cattle Egret	2019/6/18	4	0	25.0909396	121.5827066	https://www.inaturalist.org/observations/27243707
4957	Bubulcus ibis	Cattle Egret	2019/9/2	2	0	25.0909649	121.5825039	https://www.inaturalist.org/observations/40334567
4958	Bubulcus ibis	Cattle Egret	2019/6/25	2	0	25.0909740	121.5825475	https://www.inaturalist.org/observations/28102284
4959	Bubulcus ibis	Cattle Egret	2019/7/2	2	0	25.0909752	121.5825840	https://www.inaturalist.org/observations/33127691
4960	Bubulcus ibis	Cattle Egret	2019/6/4	3	0	25.0909874	121.5827521	https://www.inaturalist.org/observations/26908266
4961	Bubulcus ibis	Cattle Egret	2019/5/13	1	0	25.0910243	121.5826950	https://www.inaturalist.org/observations/25102297
4962	Bubulcus ibis	Cattle Egret	2019/6/20	3	0	25.0910552	121.5827501	https://www.inaturalist.org/observations/27315904
4963	Bubulcus ibis	Cattle Egret	2018/8/28	2	0	25.0910610	121.5817390	https://www.inaturalist.org/observations/16223040
4964	Bubulcus ibis	Cattle Egret	2019/6/25	2	0	25.0910631	121.5826531	https://www.inaturalist.org/observations/28051304
4965	Bubulcus ibis	Cattle Egret	2019/6/25	2	0	25.0910672	121.5824244	https://www.inaturalist.org/observations/28101705
4966	Bubulcus ibis	Cattle Egret	2019/4/30	1	0	25.0910689	121.5826561	https://www.inaturalist.org/observations/24359949
4967	Bubulcus ibis	Cattle Egret	2018/9/18	2	0	25.0910691	121.5826014	https://www.inaturalist.org/observations/16652186
4968	Bubulcus ibis	Cattle Egret	2019/7/16	4	0	25.0910759	121.5827164	https://www.inaturalist.org/observations/28989303
4969	Bubulcus ibis	Cattle Egret	2018/10/2	3	0	25.0910961	121.5813012	https://www.inaturalist.org/observations/20989772
4970	Bubulcus ibis	Cattle Egret	2020/6/25	1	0	25.0911311	121.5820799	https://www.inaturalist.org/observations/50861322
4971	Bubulcus ibis	Cattle Egret	2021/7/16	2	0	25.0911918	121.5816784	https://www.inaturalist.org/observations/121544014
4972	Bubulcus ibis	Cattle Egret	2019/3/26	1	0	25.0912987	121.5812968	https://www.inaturalist.org/observations/21932741
4973	Bubulcus ibis	Cattle Egret	2022/12/27	1	0	25.0913138	121.5970300	https://www.inaturalist.org/observations/145181859
4974	Bubulcus ibis	Cattle Egret	2022/11/20	3	0	25.0913671	121.5818608	https://www.inaturalist.org/observations/142439909

4975	Bubulcus ibis	Cattle Egret	2022/1/10	1	0	25.0915518	121.5829691	https://www.inaturalist.org/observations/106306218
4976	Bubulcus ibis	Cattle Egret	2019/7/12	0	0	25.0917975	121.4920321	https://www.inaturalist.org/observations/38419494
4977	Bubulcus ibis	Cattle Egret	2021/7/17	2	0	25.0917975	121.4920321	https://www.inaturalist.org/observations/87389259
4978	Bubulcus ibis	Cattle Egret	2022/5/18	1	0	25.0917975	121.4920321	https://www.inaturalist.org/observations/117699561
4979	Bubulcus ibis	Cattle Egret	2022/5/18	1	0	25.0917975	121.4920321	https://www.inaturalist.org/observations/118896152
4980	Bubulcus ibis	Cattle Egret	2021/4/23	3	0	25.0930615	121.5323848	https://www.inaturalist.org/observations/77550743
4981	Bubulcus ibis	Cattle Egret	2022/8/28	1	0	25.0930710	121.5323991	https://www.inaturalist.org/observations/133132296
4982	Bubulcus ibis	Cattle Egret	2018/10/6	4	0	25.0938699	121.4523313	https://www.inaturalist.org/observations/18409165
4983	Bubulcus ibis	Cattle Egret	2021/5/2	3	0	25.0947659	121.5303680	https://www.inaturalist.org/observations/76409521
4984	Bubulcus ibis	Cattle Egret	2020/6/17	1	0	25.0950492	121.5246077	https://www.inaturalist.org/observations/51069482
4985	Bubulcus ibis	Cattle Egret	2022/2/27	2	0	25.0957694	121.4595635	https://www.inaturalist.org/observations/107635184
4986	Bubulcus ibis	Cattle Egret	2022/1/10	2	0	25.0960244	121.4594422	https://www.inaturalist.org/observations/104706098
4987	Bubulcus ibis coromandus	Cattle Egret	2018/12/21	3	0	25.0964657	121.4549263	https://www.inaturalist.org/observations/19140794
4988	Bubulcus ibis	Cattle Egret	2021/6/8	1	0	25.0966055	121.5170237	https://www.inaturalist.org/observations/82127640
4989	Bubulcus ibis	Cattle Egret	2021/5/22	1	0	25.0970178	121.5173067	https://www.inaturalist.org/observations/79744399
4990	Bubulcus ibis	Cattle Egret	2020/8/9	4	0	25.0971559	76.5343019	https://www.inaturalist.org/observations/64850401
4991	Bubulcus ibis	Cattle Egret	2022/3/6	4	0	25.0975877	121.4569688	https://www.inaturalist.org/observations/108024704
4992	Bubulcus ibis	Cattle Egret	2022/8/7	2	0	25.0975963	121.4556268	https://www.inaturalist.org/observations/132114844
4993	Bubulcus ibis	Cattle Egret	2020/1/15	1	0	25.0976125	121.4556566	https://www.inaturalist.org/observations/37743175
4994	Bubulcus ibis	Cattle Egret	2022/4/6	2	0	25.0977091	121.4570255	https://www.inaturalist.org/observations/110559895
4995	Bubulcus ibis	Cattle Egret	2018/9/2	4	0	25.0979390	121.4557610	https://www.inaturalist.org/observations/16136763
4996	Bubulcus ibis	Cattle Egret	2020/5/7	1	0	25.0980330	121.4567492	https://www.inaturalist.org/observations/48312312
4997	Bubulcus ibis	Cattle Egret	2021/7/3	2	0	25.0982919	121.4566395	https://www.inaturalist.org/observations/86007843
4998	Bubulcus ibis	Cattle Egret	2018/10/31	4	0	25.0983181	121.5391693	https://www.inaturalist.org/observations/17986892
4999	Bubulcus ibis	Cattle Egret	2021/6/15	1	0	25.0983617	121.5167294	https://www.inaturalist.org/observations/83105283

5000	Bubulcus ibis	Cattle Egret	2021/1/16	2	0	25.0987006	121.4543006	https://www.inaturalist.org/observations/68995689
5001	Bubulcus ibis	Cattle Egret	2022/10/11	1	0	25.0987149	121.4551976	https://www.inaturalist.org/observations/139503710
5002	Bubulcus ibis coromandus	Cattle Egret	2022/10/10	0	0	25.0987682	121.4552767	https://www.inaturalist.org/observations/139503292
5003	Bubulcus ibis	Cattle Egret	2022/10/11	1	0	25.0988558	121.4552181	https://www.inaturalist.org/observations/139503713
5004	Bubulcus ibis	Cattle Egret	2019/4/13	2	0	25.0989009	121.4542140	https://www.inaturalist.org/observations/24281665
5005	Bubulcus ibis	Cattle Egret	2019/7/26	3	0	25.0989964	121.6299412	https://www.inaturalist.org/observations/29573658
5006	Bubulcus ibis	Cattle Egret	2021/10/15	1	0	25.0991359	121.4555660	https://www.inaturalist.org/observations/100127588
5007	Bubulcus ibis	Cattle Egret	2009/10/30	2	0	25.0992391	121.4726938	https://www.inaturalist.org/observations/55448671
5008	Bubulcus ibis	Cattle Egret	2019/8/5	3	0	25.0994234	121.4612692	https://www.inaturalist.org/observations/30741564
5009	Bubulcus ibis	Cattle Egret	2021/11/23	1	0	25.0996112	121.5294717	https://www.inaturalist.org/observations/101742770
5010	Bubulcus ibis	Cattle Egret	2020/4/25	1	0	25.0997806	121.4543960	https://www.inaturalist.org/observations/44146941
5011	Bubulcus ibis	Cattle Egret	2020/12/19	1	0	25.0999917	121.5297700	https://www.inaturalist.org/observations/66774524
5012	Bubulcus ibis	Cattle Egret	2022/4/23	1	0	25.1005164	121.5272334	https://www.inaturalist.org/observations/114018686
5013	Bubulcus ibis	Cattle Egret	2018/12/29	3	0	25.1005753	121.5364444	https://www.inaturalist.org/observations/19351478
5014	Bubulcus ibis	Cattle Egret	2013/4/4	1	0	25.1006370	121.2215779	https://www.inaturalist.org/observations/56720706
5015	Bubulcus ibis	Cattle Egret	2020/8/27	2	0	25.1016280	121.5303002	https://www.inaturalist.org/observations/57714304
5016	Bubulcus ibis	Cattle Egret	2022/7/3	3	0	25.1045417	121.5306700	https://www.inaturalist.org/observations/141078657
5017	Bubulcus ibis	Cattle Egret	2021/7/1	2	0	25.1051422	121.4615024	https://www.inaturalist.org/observations/86007754
5018	Bubulcus ibis	Cattle Egret	2020/7/7	2	0	25.1057888	121.4592863	https://www.inaturalist.org/observations/53263117
5019	Bubulcus ibis	Cattle Egret	2021/5/1	0	0	25.1073950	121.5046760	https://www.inaturalist.org/observations/76159399
5020	Bubulcus ibis	Cattle Egret	2021/5/2	1	0	25.1074295	121.5673584	https://www.inaturalist.org/observations/76733882
5021	Bubulcus ibis	Cattle Egret	2021/5/2	1	0	25.1083565	121.5709235	https://www.inaturalist.org/observations/76378108
5022	Bubulcus ibis	Cattle Egret	2022/1/31	3	0	25.1092617	121.4564739	https://www.inaturalist.org/observations/105969213
5023	Bubulcus ibis	Cattle Egret	2018/6/30	3	0	25.1095474	121.4654884	https://www.inaturalist.org/observations/13912080
5024	Bubulcus ibis	Cattle Egret	2021/6/24	1	0	25.1096010	121.5124084	https://www.inaturalist.org/observations/84262145

5025	Bubulcus ibis	Cattle Egret	2018/6/30	3	0	25.1097728	121.4638748	https://www.inaturalist.org/observations/13912076
5026	Bubulcus ibis	Cattle Egret	2020/7/7	1	0	25.1098060	121.4564700	https://www.inaturalist.org/observations/53263361
5027	Bubulcus ibis	Cattle Egret	2018/6/30	3	0	25.1100684	121.4650622	https://www.inaturalist.org/observations/13912081
5028	Bubulcus ibis	Cattle Egret	2020/7/7	1	0	25.1105644	121.4583359	https://www.inaturalist.org/observations/53259465
5029	Bubulcus ibis	Cattle Egret	2019/10/20	3	0	25.1108303	121.4625778	https://www.inaturalist.org/observations/35528724
5030	Bubulcus ibis	Cattle Egret	2020/7/7	1	0	25.1113028	121.4595107	https://www.inaturalist.org/observations/53259408
5031	Bubulcus ibis	Cattle Egret	2022/12/7	1	0	25.1117462	121.4968227	https://www.inaturalist.org/observations/143867902
5032	Bubulcus ibis	Cattle Egret	2022/4/1	1	0	25.1120066	121.5526675	https://www.inaturalist.org/observations/137004554
5033	Bubulcus ibis	Cattle Egret	2020/4/25	3	0	25.1123093	121.4906270	https://www.inaturalist.org/observations/44101317
5034	Bubulcus ibis	Cattle Egret	2020/4/25	1	0	25.1123093	121.4906270	https://www.inaturalist.org/observations/56193076
5035	Bubulcus ibis	Cattle Egret	2020/7/8	1	0	25.1125109	121.4608765	https://www.inaturalist.org/observations/53356303
5036	Bubulcus ibis	Cattle Egret	2020/7/8	1	0	25.1134606	121.4589533	https://www.inaturalist.org/observations/53347436
5037	Bubulcus ibis	Cattle Egret	2022/5/26	2	0	25.1142393	121.6264143	https://www.inaturalist.org/observations/118754112
5038	Bubulcus ibis	Cattle Egret	2019/4/7	3	0	25.1152746	121.4725225	https://www.inaturalist.org/observations/71050709
5039	Bubulcus ibis	Cattle Egret	2021/8/21	2	0	25.1162132	121.4645783	https://www.inaturalist.org/observations/91924139
5040	Bubulcus ibis	Cattle Egret	2020/6/7	1	0	25.1167730	121.4712180	https://www.inaturalist.org/observations/48743871
5041	Bubulcus ibis	Cattle Egret	2019/7/25	2	0	25.1169848	121.4719391	https://www.inaturalist.org/observations/36368917
5042	Bubulcus ibis	Cattle Egret	2018/7/4	2	0	25.1183775	121.4719110	https://www.inaturalist.org/observations/58215919
5043	Bubulcus ibis	Cattle Egret	2018/7/4	2	0	25.1184222	121.4718015	https://www.inaturalist.org/observations/58215918
5044	Bubulcus ibis	Cattle Egret	2018/8/28	3	0	25.1185778	121.4697717	https://www.inaturalist.org/observations/17003772
5045	Bubulcus ibis	Cattle Egret	2014/3/16	4	0	25.1187890	121.4712260	http://www.inaturalist.org/observations/574076
5046	Bubulcus ibis coromandus	Cattle Egret	2016/3/20	0	0	25.1188888	121.4708331	https://www.inaturalist.org/observations/134122984
5047	Bubulcus ibis	Cattle Egret	2012/11/17	1	0	25.1188889	121.4708333	https://www.inaturalist.org/observations/70852020
5048	Bubulcus ibis	Cattle Egret	2021/8/21	2	0	25.1188889	121.4708333	https://www.inaturalist.org/observations/92573619
5049	Bubulcus ibis	Cattle Egret	2022/2/6	1	0	25.1188889	121.4708333	https://www.inaturalist.org/observations/108280473

5050	Bubulcus ibis	Cattle Egret	2018/9/21	3	0	25.1188989	121.4707983	https://www.inaturalist.org/observations/16904651
5051	Bubulcus ibis	Cattle Egret	2019/4/17	4	0	25.1188989	121.4707983	https://www.inaturalist.org/observations/24388620
5052	Bubulcus ibis	Cattle Egret	2018/8/15	2	0	25.1188990	121.4707980	https://www.inaturalist.org/observations/15476654
5053	Bubulcus ibis	Cattle Egret	2018/8/15	2	0	25.1188990	121.4707980	https://www.inaturalist.org/observations/15476707
5054	Bubulcus ibis	Cattle Egret	2017/8/20	2	0	25.1189966	121.4714349	https://www.inaturalist.org/observations/105322185
5055	Bubulcus ibis	Cattle Egret	2019/10/20	1	0	25.1190017	121.4706817	https://www.inaturalist.org/observations/34727130
5056	Bubulcus ibis	Cattle Egret	2021/11/2	1	0	25.1190383	121.4715020	https://www.inaturalist.org/observations/100125528
5057	Bubulcus ibis	Cattle Egret	2022/11/12	2	0	25.1190536	121.4722114	https://www.inaturalist.org/observations/141903108
5058	Bubulcus ibis	Cattle Egret	2019/10/19	1	0	25.1190710	121.4716857	https://www.inaturalist.org/observations/34727158
5059	Bubulcus ibis	Cattle Egret	2019/10/19	1	0	25.1191120	121.4704666	https://www.inaturalist.org/observations/34568248
5060	Bubulcus ibis	Cattle Egret	2019/10/20	1	0	25.1191141	121.4712884	https://www.inaturalist.org/observations/34735278
5061	Bubulcus ibis	Cattle Egret	2018/4/28	4	0	25.1191715	121.4716130	https://www.inaturalist.org/observations/11585873
5062	Bubulcus ibis	Cattle Egret	2022/4/3	1	0	25.1191903	121.4370921	https://www.inaturalist.org/observations/123792949
5063	Bubulcus ibis	Cattle Egret	2018/4/28	3	0	25.1192057	121.4716579	https://www.inaturalist.org/observations/11585963
5064	Bubulcus ibis	Cattle Egret	2017/2/11	4	0	25.1192267	121.4705180	https://www.inaturalist.org/observations/5134722
5065	Bubulcus ibis	Cattle Egret	2020/3/5	2	0	25.1192338	121.4715473	https://www.inaturalist.org/observations/40942315
5066	Bubulcus ibis	Cattle Egret	2020/4/26	1	0	25.1192346	121.4716564	https://www.inaturalist.org/observations/43559833
5067	Bubulcus ibis	Cattle Egret	2018/7/15	4	0	25.1192448	121.4708721	https://www.inaturalist.org/observations/14370880
5068	Bubulcus ibis	Cattle Egret	2018/12/2	2	0	25.1193333	121.4709700	https://www.inaturalist.org/observations/18776449
5069	Bubulcus ibis	Cattle Egret	2018/10/27	1	0	25.1193390	121.4710998	https://www.inaturalist.org/observations/99630453
5070	Bubulcus ibis	Cattle Egret	2020/10/4	1	0	25.1193946	121.4214828	https://www.inaturalist.org/observations/63206588
5071	Bubulcus ibis	Cattle Egret	2022/5/29	1	0	25.1194055	121.4698950	https://www.inaturalist.org/observations/122429825
5072	Bubulcus ibis	Cattle Egret	2020/9/18	2	0	25.1194445	121.4726550	https://www.inaturalist.org/observations/59944044
5073	Bubulcus ibis	Cattle Egret	2021/5/2	1	0	25.1194638	121.4721617	https://www.inaturalist.org/observations/76493976
5074	Bubulcus ibis	Cattle Egret	2021/12/5	1	0	25.1195248	121.4746376	https://www.inaturalist.org/observations/102510806

5075	Bubulcus ibis	Cattle Egret	2019/10/19	1	0	25.1198336	121.4711141	https://www.inaturalist.org/observations/34570062
5076	Bubulcus ibis coromandus	Cattle Egret	2017/12/27	2	0	25.1200540	98.5584740	https://www.inaturalist.org/observations/9372289
5077	Bubulcus ibis	Cattle Egret	2019/9/25	2	0	25.1202049	121.5529159	https://www.inaturalist.org/observations/35268909
5078	Bubulcus ibis	Cattle Egret	2020/4/17	1	0	25.1250739	121.4560150	https://www.inaturalist.org/observations/61546702
5079	Bubulcus ibis	Cattle Egret	2021/4/2	1	0	25.1251483	121.4807203	https://www.inaturalist.org/observations/72706574
5080	Bubulcus ibis	Cattle Egret	2021/4/2	1	0	25.1252139	121.4810394	https://www.inaturalist.org/observations/72706573
5081	Bubulcus ibis	Cattle Egret	2021/4/4	1	0	25.1290113	121.4884336	https://www.inaturalist.org/observations/73195504
5082	Bubulcus ibis	Cattle Egret	2021/4/2	1	0	25.1308611	121.4909514	https://www.inaturalist.org/observations/72706545
5083	Bubulcus ibis	Cattle Egret	2021/4/2	1	0	25.1322125	121.4888961	https://www.inaturalist.org/observations/72706552
5084	Bubulcus ibis	Cattle Egret	2019/7/7	3	0	25.1332479	121.5728156	https://www.inaturalist.org/observations/28352288
5085	Bubulcus ibis	Cattle Egret	2021/5/28	1	0	25.1332612	121.4591667	https://www.inaturalist.org/observations/80572411
5086	Bubulcus ibis	Cattle Egret	2021/5/28	1	0	25.1332612	121.4591667	https://www.inaturalist.org/observations/80572472
5087	Bubulcus ibis	Cattle Egret	2014/4/20	7	0	25.1336060	121.4699130	http://www.inaturalist.org/observations/635558
5088	Bubulcus ibis	Cattle Egret	2022/5/8	1	0	25.1341133	121.4718170	https://www.inaturalist.org/observations/116035015
5089	Bubulcus ibis	Cattle Egret	2021/4/2	2	0	25.1343817	121.4895828	https://www.inaturalist.org/observations/72706549
5090	Bubulcus ibis	Cattle Egret	2020/5/9	1	0	25.1350476	121.4895831	https://www.inaturalist.org/observations/45479386
5091	Bubulcus ibis	Cattle Egret	2020/10/21	1	0	25.1390722	121.4573450	https://www.inaturalist.org/observations/63141811
5092	Bubulcus ibis	Cattle Egret	2020/11/29	1	0	25.1398000	121.4733500	https://www.inaturalist.org/observations/65794200
5093	Bubulcus ibis	Cattle Egret	2018/9/25	2	0	25.1436722	121.5223083	https://www.inaturalist.org/observations/16899841
5094	Bubulcus ibis	Cattle Egret	2022/5/8	1	0	25.1467190	121.4825270	https://www.inaturalist.org/observations/116059910
5095	Bubulcus ibis	Cattle Egret	2020/4/28	1	0	25.1480397	121.4579679	https://www.inaturalist.org/observations/44087052
5096	Bubulcus ibis	Cattle Egret	2020/4/28	1	0	25.1480500	121.4579467	https://www.inaturalist.org/observations/44086970
5097	Bubulcus ibis	Cattle Egret	2020/4/28	2	0	25.1480945	121.4579842	https://www.inaturalist.org/observations/44086806
5098	Bubulcus ibis	Cattle Egret	2019/9/28	2	0	25.1491416	121.5098995	https://www.inaturalist.org/observations/67284755
5099	Bubulcus ibis	Cattle Egret	2020/6/6	1	0	25.1514028	121.5058533	https://www.inaturalist.org/observations/48647891

5100	Bubulcus ibis coromandus	Cattle Egret	2018/5/19	2	0	25.1541772	121.4584705	https://www.inaturalist.org/observations/12763362
5101	Bubulcus ibis	Cattle Egret	2020/5/29	1	0	25.1549141	121.5040167	https://www.inaturalist.org/observations/47971283
5102	Bubulcus ibis	Cattle Egret	2021/5/3	2	0	25.1559354	121.5475336	https://www.inaturalist.org/observations/76873999
5103	Bubulcus ibis	Cattle Egret	2021/5/3	3	0	25.1560833	121.5461800	https://www.inaturalist.org/observations/76868182
5104	Bubulcus ibis	Cattle Egret	2014/5/3	5	0	25.1569770	121.4571570	http://www.inaturalist.org/observations/655320
5105	Bubulcus ibis	Cattle Egret	2022/10/21	1	0	25.1583219	121.5070701	https://www.inaturalist.org/observations/142905339
5106	Bubulcus ibis	Cattle Egret	2021/9/6	2	0	25.1632370	121.4166718	https://www.inaturalist.org/observations/103676420
5107	Bubulcus ibis	Cattle Egret	2021/7/31	2	0	25.1632528	121.4166221	https://www.inaturalist.org/observations/99630456
5108	Bubulcus ibis	Cattle Egret	2022/9/6	1	0	25.1636733	121.5731550	https://www.inaturalist.org/observations/133926327
5109	Bubulcus ibis	Cattle Egret	2021/12/22	1	0	25.1639108	121.5781515	https://www.inaturalist.org/observations/136561207
5110	Bubulcus ibis	Cattle Egret	2021/12/29	1	0	25.1639108	121.5781515	https://www.inaturalist.org/observations/136563540
5111	Bubulcus ibis	Cattle Egret	2021/9/21	2	0	25.1646105	121.5756502	https://www.inaturalist.org/observations/95654756
5112	Bubulcus ibis	Cattle Egret	2022/9/16	3	0	25.1646141	121.5764334	https://www.inaturalist.org/observations/135333151
5113	Bubulcus ibis	Cattle Egret	2018/10/1	3	0	25.1655217	121.4142350	https://www.inaturalist.org/observations/19047034
5114	Bubulcus ibis	Cattle Egret	2022/8/27	2	0	25.1655521	121.5721436	https://www.inaturalist.org/observations/132476991
5115	Bubulcus ibis	Cattle Egret	2020/2/19	0	0	25.1656613	121.4129410	https://www.inaturalist.org/observations/39206753
5116	Bubulcus ibis	Cattle Egret	2022/4/13	2	0	25.1660328	121.4186939	https://www.inaturalist.org/observations/111305933
5117	Bubulcus ibis	Cattle Egret	2018/7/4	3	0	25.1745867	121.4501800	https://www.inaturalist.org/observations/14343082
5118	Bubulcus ibis	Cattle Egret	2022/7/10	1	0	25.1747833	121.4494833	https://www.inaturalist.org/observations/125775740
5119	Bubulcus ibis	Cattle Egret	2021/5/3	4	0	25.1768250	121.5384833	https://www.inaturalist.org/observations/76877689
5120	Bubulcus ibis	Cattle Egret	2021/5/3	5	0	25.1771204	121.5379785	https://www.inaturalist.org/observations/76874214
5121	Bubulcus ibis	Cattle Egret	2021/5/7	2	0	25.1781359	121.5683183	https://www.inaturalist.org/observations/77697919
5122	Bubulcus ibis	Cattle Egret	2020/9/1	1	0	25.1781525	121.4295455	https://www.inaturalist.org/observations/58215021
5123	Bubulcus ibis	Cattle Egret	2020/5/13	1	0	25.1831499	121.4883285	https://www.inaturalist.org/observations/56379310
5124	Bubulcus ibis	Cattle Egret	2016/7/29	1	0	25.1834499	121.4269459	https://www.inaturalist.org/observations/39050406

5125	Bubulcus ibis	Cattle Egret	2020/5/8	1	0	25.1858777	121.4272656	https://www.inaturalist.org/observations/46218086
5126	Bubulcus ibis	Cattle Egret	2019/9/11	2	0	25.1872467	121.4539717	https://www.inaturalist.org/observations/32511006
5127	Bubulcus ibis	Cattle Egret	2018/9/12	1	0	25.1874875	121.4551203	https://www.inaturalist.org/observations/16443333
5128	Bubulcus ibis	Cattle Egret	2018/9/12	3	0	25.1875315	121.4567715	https://www.inaturalist.org/observations/16442404
5129	Bubulcus ibis	Cattle Egret	2019/9/11	2	0	25.1877200	121.4536217	https://www.inaturalist.org/observations/32510899
5130	Bubulcus ibis	Cattle Egret	2012/9/22	2	0	25.1942462	121.5609363	https://www.inaturalist.org/observations/70850829
5131	Bubulcus ibis	Cattle Egret	2022/6/16	3	0	25.2031639	75.7922781	https://www.inaturalist.org/observations/121972199
5132	Bubulcus ibis	Cattle Egret	2014/5/12	1	0	25.2071533	110.3476974	https://www.inaturalist.org/observations/116083079
5133	Bubulcus ibis	Cattle Egret	2022/12/3	4	0	25.2208730	75.8361810	https://www.inaturalist.org/observations/143557722
5134	Bubulcus ibis	Cattle Egret	2015/9/5	1	0	25.2308046	121.6324989	https://www.inaturalist.org/observations/58826154
5135	Bubulcus ibis coromandus	Cattle Egret	2021/1/15	1	0	25.2308240	121.6328422	https://www.inaturalist.org/observations/68403414
5136	Bubulcus ibis	Cattle Egret	2015/3/21	1	0	25.2308919	121.6323916	https://www.inaturalist.org/observations/57611402
5137	Bubulcus ibis	Cattle Egret	2020/11/20	1	0	25.2309710	121.6317430	https://www.inaturalist.org/observations/65456631
5138	Bubulcus ibis	Cattle Egret	2022/4/9	1	0	25.2310543	121.6315540	https://www.inaturalist.org/observations/115869199
5139	Bubulcus ibis	Cattle Egret	2022/4/9	1	0	25.2310860	121.6315544	https://www.inaturalist.org/observations/115869206
5140	Bubulcus ibis	Cattle Egret	2022/8/17	2	0	25.2394435	121.6152244	https://www.inaturalist.org/observations/131169091
5141	Bubulcus ibis	Cattle Egret	2022/8/16	3	0	25.2398271	121.6148979	https://www.inaturalist.org/observations/131057962
5142	Bubulcus ibis	Cattle Egret	2022/8/16	2	0	25.2398272	121.6148980	https://www.inaturalist.org/observations/131042830
5143	Bubulcus ibis	Cattle Egret	2022/8/18	2	0	25.2398272	121.6148980	https://www.inaturalist.org/observations/131306502
5144	Bubulcus ibis	Cattle Egret	2022/8/19	1	0	25.2398272	121.6148980	https://www.inaturalist.org/observations/131431466
5145	Bubulcus ibis	Cattle Egret	2022/8/22	2	0	25.2398307	121.6148979	https://www.inaturalist.org/observations/131858962
5146	Bubulcus ibis	Cattle Egret	2022/9/7	1	0	25.2398307	121.6148979	https://www.inaturalist.org/observations/133997577
5147	Bubulcus ibis	Cattle Egret	2022/8/17	2	0	25.2401728	121.6138330	https://www.inaturalist.org/observations/131169083
5148	Bubulcus ibis	Cattle Egret	2022/8/17	2	0	25.2402032	121.6137074	https://www.inaturalist.org/observations/131169086
5149	Bubulcus ibis	Cattle Egret	2022/8/17	2	0	25.2402032	121.6137074	https://www.inaturalist.org/observations/131169088

5150	Bubulcus ibis	Cattle Egret	2022/8/17	2	0	25.2402032	121.6137074	https://www.inaturalist.org/observations/131169089
5151	Bubulcus ibis	Cattle Egret	2022/8/17	2	0	25.2402627	121.6139408	https://www.inaturalist.org/observations/131169084
5152	Bubulcus ibis	Cattle Egret	2022/8/17	2	0	25.2402627	121.6139408	https://www.inaturalist.org/observations/131169085
5153	Bubulcus ibis	Cattle Egret	2022/8/17	2	0	25.2403783	121.6137445	https://www.inaturalist.org/observations/131169090
5154	Bubulcus ibis	Cattle Egret	2022/8/11	2	0	25.2410606	121.6147399	https://www.inaturalist.org/observations/130586550
5155	Bubulcus ibis	Cattle Egret	2022/8/26	2	0	25.2411615	121.6134981	https://www.inaturalist.org/observations/132369594
5156	Bubulcus ibis	Cattle Egret	2022/5/10	2	0	25.2413522	121.6171678	https://www.inaturalist.org/observations/127069392
5157	Bubulcus ibis	Cattle Egret	2022/8/24	1	0	25.2435921	121.6147058	https://www.inaturalist.org/observations/132258147
5158	Bubulcus ibis	Cattle Egret	2022/5/6	1	0	25.2442750	121.6156300	https://www.inaturalist.org/observations/115656080
5159	Bubulcus ibis	Cattle Egret	2022/5/8	1	0	25.2444628	121.6166497	https://www.inaturalist.org/observations/116018567
5160	Bubulcus ibis	Cattle Egret	2020/11/15	3	0	25.2677187	82.9912581	https://www.inaturalist.org/observations/64929430
5161	Bubulcus ibis	Cattle Egret	2011/11/1	1	0	25.3047508	75.4118450	https://www.inaturalist.org/observations/45528652
5162	Bubulcus ibis	Cattle Egret	2016/3/31	2	0	25.3080738	83.0063801	https://www.inaturalist.org/observations/59942711
5163	Bubulcus ibis	Cattle Egret	2016/3/31	2	0	25.3199654	83.0142009	https://www.inaturalist.org/observations/91084203
5164	Bubulcus ibis	Cattle Egret	2018/2/20	2	0	25.3233000	83.0331000	https://www.inaturalist.org/observations/69874975
5165	Bubulcus ibis	Cattle Egret	2021/2/13	4	0	25.4115995	81.8485844	https://www.inaturalist.org/observations/69490787
5166	Bubulcus ibis	Cattle Egret	2018/9/9	2	0	25.5963363	85.1005811	https://www.inaturalist.org/observations/21745449
5167	Bubulcus ibis	Cattle Egret	2022/2/9	4	0	25.6063227	85.8823039	https://www.inaturalist.org/observations/106415996
5168	Bubulcus ibis	Cattle Egret	2019/12/25	2	0	25.6367363	88.1199865	https://www.inaturalist.org/observations/71412849
5169	Bubulcus ibis	Cattle Egret	2022/12/13	1	0	25.6517540	90.8032181	https://www.inaturalist.org/observations/144418748
5170	Bubulcus ibis	Cattle Egret	2021/1/24	1	0	25.6517761	90.8032180	https://www.inaturalist.org/observations/77565096
5171	Bubulcus ibis	Cattle Egret	2022/9/5	1	0	25.6714886	100.2122690	https://www.inaturalist.org/observations/133746538
5172	Bubulcus ibis	Cattle Egret	2021/8/3	1	0	25.6956992	100.2075542	https://www.inaturalist.org/observations/91217391
5173	Bubulcus ibis	Cattle Egret	2022/6/24	1	0	25.7034092	100.1662833	https://www.inaturalist.org/observations/123321791
5174	Bubulcus ibis	Cattle Egret	2014/5/23	2	0	25.7429167	100.1628611	https://www.inaturalist.org/observations/68418538

5175	Bubulcus ibis	Cattle Egret	2022/4/24	1	0	25.8450652	100.1294354	https://www.inaturalist.org/observations/112653467
5176	Bubulcus ibis	Cattle Egret	2018/11/24	5	0	25.8661376	93.4667024	https://www.inaturalist.org/observations/18591289
5177	Bubulcus ibis	Cattle Egret	2021/5/14	1	0	25.9516318	119.9683432	https://www.inaturalist.org/observations/83947955
5178	Bubulcus ibis	Cattle Egret	2022/6/3	1	0	26.0053583	119.6236549	https://www.inaturalist.org/observations/132116155
5179	Bubulcus ibis	Cattle Egret	2009/1/16	1	0	26.0173274	76.5025742	https://www.inaturalist.org/observations/48209306
5180	Bubulcus ibis	Cattle Egret	2022/6/3	1	0	26.0218349	119.6314567	https://www.inaturalist.org/observations/132116182
5181	Bubulcus ibis	Cattle Egret	2022/6/3	1	0	26.0220100	119.6308433	https://www.inaturalist.org/observations/132116188
5182	Bubulcus ibis	Cattle Egret	2015/5/26	5	0	26.0284019	76.4651526	https://www.inaturalist.org/observations/140701080
5183	Bubulcus ibis	Cattle Egret	2021/6/14	2	0	26.0554350	119.1892940	https://www.inaturalist.org/observations/82977389
5184	Bubulcus ibis	Cattle Egret	2021/6/15	2	0	26.0586530	119.1948590	https://www.inaturalist.org/observations/84636199
5185	Bubulcus ibis	Cattle Egret	2022/6/21	1	0	26.0608300	119.1879950	https://www.inaturalist.org/observations/122896873
5186	Bubulcus ibis	Cattle Egret	2020/2/28	0	0	26.0658552	76.4332443	https://www.inaturalist.org/observations/39745260
5187	Bubulcus ibis	Cattle Egret	2020/2/20	2	0	26.1104778	91.6351579	https://www.inaturalist.org/observations/46574660
5188	Bubulcus ibis	Cattle Egret	2006/2/9	3	0	26.1120000	91.6520000	https://www.inaturalist.org/observations/104851128
5189	Bubulcus ibis coromandus	Cattle Egret	2013/3/11	2	0	26.1120000	91.6770000	https://www.inaturalist.org/observations/37662701
5190	Bubulcus ibis coromandus	Cattle Egret	2013/3/11	1	0	26.1120000	91.6770000	https://www.inaturalist.org/observations/37662762
5191	Bubulcus ibis	Cattle Egret	2012/11/25	2	0	26.1130203	91.6560049	https://www.inaturalist.org/observations/60751624
5192	Bubulcus ibis	Cattle Egret	2019/4/2	4	0	26.1141729	91.6784492	https://www.inaturalist.org/observations/97304581
5193	Bubulcus ibis	Cattle Egret	2019/4/2	5	0	26.1145833	91.6768750	https://www.inaturalist.org/observations/25777967
5194	Bubulcus ibis	Cattle Egret	2016/6/12	1	0	26.1176941	91.6493532	https://www.inaturalist.org/observations/95938587
5195	Bubulcus ibis	Cattle Egret	2015/3/22	2	0	26.1243663	91.6680913	https://www.inaturalist.org/observations/68903335
5196	Bubulcus ibis	Cattle Egret	2020/5/7	1	0	26.1407242	119.9270926	https://www.inaturalist.org/observations/46065503
5197	Bubulcus ibis coromandus	Cattle Egret	2013/4/15	5	0	26.1482138	91.8351113	https://www.inaturalist.org/observations/7458822
5198	Bubulcus ibis	Cattle Egret	2022/4/6	2	0	26.1538020	91.6578865	https://www.inaturalist.org/observations/110648090
5199	Bubulcus ibis	Cattle Egret	2018/5/1	3	0	26.1549926	91.6685871	https://www.inaturalist.org/observations/59611117

5200	Bubulcus ibis	Cattle Egret	2022/5/23	1	0	26.1558843	119.9401461	https://www.inaturalist.org/observations/119411254
5201	Bubulcus ibis	Cattle Egret	2018/9/8	4	0	26.1635190	91.7807250	https://www.inaturalist.org/observations/16412175
5202	Bubulcus ibis	Cattle Egret	2018/9/8	5	0	26.1635190	91.7807250	https://www.inaturalist.org/observations/19484616
5203	Bubulcus ibis	Cattle Egret	2021/6/12	2	0	26.1659034	75.7962851	https://www.inaturalist.org/observations/82653472
5204	Bubulcus ibis	Cattle Egret	2018/6/14	4	0	26.1712302	91.7779827	https://www.inaturalist.org/observations/13445228
5205	Bubulcus ibis	Cattle Egret	2021/2/16	4	0	26.1801793	91.7352279	https://www.inaturalist.org/observations/75628462
5206	Bubulcus ibis	Cattle Egret	2013/2/10	0	0	26.1878680	91.6912937	https://www.inaturalist.org/observations/103620253
5207	Bubulcus ibis	Cattle Egret	2019/4/22	5	0	26.1889012	91.7468533	https://www.inaturalist.org/observations/23340923
5208	Bubulcus ibis	Cattle Egret	2020/4/10	3	0	26.1893606	91.7532970	https://www.inaturalist.org/observations/54023447
5209	Bubulcus ibis	Cattle Egret	2021/5/8	2	0	26.1954583	78.1979450	https://www.inaturalist.org/observations/78179113
5210	Bubulcus ibis	Cattle Egret	2018/9/12	5	0	26.1964284	91.7012290	https://www.inaturalist.org/observations/39875623
5211	Bubulcus ibis	Cattle Egret	2015/12/16	1	0	26.2173732	91.6334666	https://www.inaturalist.org/observations/90516750
5212	Bubulcus ibis	Cattle Egret	2015/12/16	3	0	26.2189207	91.6329246	https://www.inaturalist.org/observations/129148177
5213	Bubulcus ibis	Cattle Egret	2018/9/15	4	0	26.2419229	73.0045427	https://www.inaturalist.org/observations/16546456
5214	Bubulcus ibis	Cattle Egret	2018/8/20	5	0	26.2424894	73.0030896	https://www.inaturalist.org/observations/16447403
5215	Bubulcus ibis	Cattle Egret	2017/7/6	4	0	26.2426620	73.0033752	https://www.inaturalist.org/observations/16526820
5216	Bubulcus ibis	Cattle Egret	2018/7/22	4	0	26.2438652	73.0017880	https://www.inaturalist.org/observations/16526730
5217	Bubulcus ibis	Cattle Egret	2019/12/29	0	0	26.2527772	76.3410819	https://www.inaturalist.org/observations/37782847
5218	Bubulcus ibis	Cattle Egret	2022/8/12	5	0	26.2983975	74.7354242	https://www.inaturalist.org/observations/132148379
5219	Bubulcus ibis	Cattle Egret	2020/4/20	3	0	26.3131661	79.7396268	https://www.inaturalist.org/observations/55903825
5220	Bubulcus ibis	Cattle Egret	2015/7/23	5	0	26.3345034	91.2241497	https://www.inaturalist.org/observations/19121995
5221	Bubulcus ibis	Cattle Egret	2018/4/30	1	0	26.3686778	120.4883333	https://www.inaturalist.org/observations/56823058
5222	Bubulcus ibis	Cattle Egret	2019/4/21	2	0	26.3696038	120.4923492	https://www.inaturalist.org/observations/22932193
5223	Bubulcus ibis	Cattle Egret	2022/5/23	1	0	26.3805803	120.4772464	https://www.inaturalist.org/observations/118442792
5224	Bubulcus ibis	Cattle Egret	2021/4/27	1	0	26.3817056	120.4763286	https://www.inaturalist.org/observations/75406474

5225	Bubulcus ibis	Cattle Egret	2022/10/8	1	0	26.4234217	106.3695259	https://www.inaturalist.org/observations/142895329
5226	Bubulcus ibis	Cattle Egret	2022/9/30	1	0	26.4661960	106.6786976	https://www.inaturalist.org/observations/142150435
5227	Bubulcus ibis	Cattle Egret	2008/9/9	3	0	26.4698596	74.6305978	https://www.inaturalist.org/observations/107925921
5228	Bubulcus ibis	Cattle Egret	2020/10/18	1	0	26.4847219	74.6252591	https://www.inaturalist.org/observations/63454148
5229	Bubulcus ibis	Cattle Egret	2020/11/7	1	0	26.4852730	74.6268070	https://www.inaturalist.org/observations/64344085
5230	Bubulcus ibis	Cattle Egret	2022/4/1	2	0	26.4906468	80.2823524	https://www.inaturalist.org/observations/119700520
5231	Bubulcus ibis	Cattle Egret	2022/4/11	1	0	26.5093412	106.6497552	https://www.inaturalist.org/observations/112045268
5232	Bubulcus ibis	Cattle Egret	2022/4/21	1	0	26.5093412	106.6497552	https://www.inaturalist.org/observations/112256100
5233	Bubulcus ibis	Cattle Egret	2022/11/10	1	0	26.5093412	106.6497552	https://www.inaturalist.org/observations/142908364
5234	Bubulcus ibis	Cattle Egret	2019/12/10	3	0	26.5252813	92.9938080	https://www.inaturalist.org/observations/39928340
5235	Bubulcus ibis	Cattle Egret	2020/2/15	5	0	26.5442267	77.9607250	https://www.inaturalist.org/observations/50950039
5236	Bubulcus ibis	Cattle Egret	2021/1/15	1	0	26.5758957	93.0872373	https://www.inaturalist.org/observations/69969824
5237	Bubulcus ibis	Cattle Egret	2006/2/6	1	0	26.5760000	93.1890000	https://www.inaturalist.org/observations/104830871
5238	Bubulcus ibis	Cattle Egret	2017/11/8	1	0	26.5775310	93.1711220	https://www.inaturalist.org/observations/82655839
5239	Bubulcus ibis	Cattle Egret	2021/10/5	1	0	26.5775344	93.1711217	https://www.inaturalist.org/observations/105599146
5240	Bubulcus ibis	Cattle Egret	2020/12/6	2	0	26.5775842	93.1709772	https://www.inaturalist.org/observations/70860416
5241	Bubulcus ibis	Cattle Egret	2021/9/12	1	0	26.5830638	106.8501414	https://www.inaturalist.org/observations/94910219
5242	Bubulcus ibis	Cattle Egret	2006/2/3	3	0	26.5870000	93.4130000	https://www.inaturalist.org/observations/104802915
5243	Bubulcus ibis coromandus	Cattle Egret	2013/4/23	2	0	26.5961627	93.2970644	https://www.inaturalist.org/observations/59039277
5244	Bubulcus ibis	Cattle Egret	2018/11/27	6	0	26.5965967	93.3954400	https://www.inaturalist.org/observations/19761636
5245	Bubulcus ibis	Cattle Egret	2017/11/25	1	0	26.6022374	78.6406833	https://www.inaturalist.org/observations/70000168
5246	Bubulcus ibis	Cattle Egret	2018/4/26	4	0	26.6282310	92.7804530	https://www.inaturalist.org/observations/13604494
5247	Bubulcus ibis	Cattle Egret	2017/9/7	2	0	26.6288820	92.7796770	https://www.inaturalist.org/observations/49002056
5248	Bubulcus ibis	Cattle Egret	2021/8/24	1	0	26.6335984	106.6335758	https://www.inaturalist.org/observations/92891696
5249	Bubulcus ibis	Cattle Egret	2021/12/8	3	0	26.6409927	94.1913931	https://www.inaturalist.org/observations/105602053

5250	Bubulcus ibis	Cattle Egret	2021/5/3	1	0	26.6468098	106.5474618	https://www.inaturalist.org/observations/78330384
5251	Bubulcus ibis	Cattle Egret	2021/8/24	1	0	26.6468098	106.5474618	https://www.inaturalist.org/observations/92891754
5252	Bubulcus ibis	Cattle Egret	2021/8/24	1	0	26.6468098	106.5474618	https://www.inaturalist.org/observations/92891799
5253	Bubulcus ibis	Cattle Egret	2020/2/6	3	0	26.6511191	93.4060271	https://www.inaturalist.org/observations/104723629
5254	Bubulcus ibis	Cattle Egret	2018/4/26	5	0	26.6528495	92.7925592	https://www.inaturalist.org/observations/19635555
5255	Bubulcus ibis	Cattle Egret	2018/4/26	5	0	26.6528495	92.7925592	https://www.inaturalist.org/observations/19635560
5256	Bubulcus ibis	Cattle Egret	2018/4/26	5	0	26.6528495	92.7925592	https://www.inaturalist.org/observations/19635584
5257	Bubulcus ibis	Cattle Egret	2018/4/26	5	0	26.6528495	92.7925592	https://www.inaturalist.org/observations/19635587
5258	Bubulcus ibis	Cattle Egret	2018/4/26	4	0	26.6528495	92.7925592	https://www.inaturalist.org/observations/19635590
5259	Bubulcus ibis	Cattle Egret	2020/8/25	1	0	26.6557583	106.5278056	https://www.inaturalist.org/observations/58008274
5260	Bubulcus ibis coromandus	Cattle Egret	2021/12/23	3	0	26.6742782	88.3711277	https://www.inaturalist.org/observations/103521647
5261	Bubulcus ibis	Cattle Egret	2014/3/23	2	0	26.6773400	93.5499050	https://www.inaturalist.org/observations/37728952
5262	Bubulcus ibis	Cattle Egret	2022/1/5	3	0	26.6967233	93.9817064	https://www.inaturalist.org/observations/105666060
5263	Bubulcus ibis	Cattle Egret	2022/4/19	2	0	26.6967317	93.9808753	https://www.inaturalist.org/observations/112263383
5264	Bubulcus ibis	Cattle Egret	2021/4/26	2	0	26.6970423	93.9780539	https://www.inaturalist.org/observations/76844217
5265	Bubulcus ibis	Cattle Egret	2021/12/7	2	0	26.7079444	88.7062222	https://www.inaturalist.org/observations/104292212
5266	Bubulcus ibis	Cattle Egret	2022/4/15	1	0	26.7346049	106.6555595	https://www.inaturalist.org/observations/112145992
5267	Bubulcus ibis	Cattle Egret	2019/12/11	2	0	26.7551754	94.1504895	https://www.inaturalist.org/observations/39929358
5268	Bubulcus ibis	Cattle Egret	2021/9/27	1	0	26.7732111	80.9651889	https://www.inaturalist.org/observations/96380217
5269	Bubulcus ibis	Cattle Egret	2021/11/18	1	0	26.7878430	81.0696360	https://www.inaturalist.org/observations/101686916
5270	Bubulcus ibis	Cattle Egret	2022/7/5	2	0	26.8129389	75.8416917	https://www.inaturalist.org/observations/124834684
5271	Bubulcus ibis	Cattle Egret	2022/2/4	1	0	26.8132534	92.5867691	https://www.inaturalist.org/observations/106112346
5272	Bubulcus ibis	Cattle Egret	2022/7/26	3	0	26.8187617	81.0153417	https://www.inaturalist.org/observations/127947446
5273	Bubulcus ibis	Cattle Egret	2021/9/12	1	0	26.8441166	88.2402630	https://www.inaturalist.org/observations/94916014
5274	Bubulcus ibis	Cattle Egret	2020/8/25	2	0	26.8477981	91.8578757	https://www.inaturalist.org/observations/57637997

5275	Bubulcus ibis	Cattle Egret	2016/3/23	4	0	26.8492163	94.2062761	https://www.inaturalist.org/observations/107583855
5276	Bubulcus ibis	Cattle Egret	2022/8/15	3	0	26.8549114	75.8328411	https://www.inaturalist.org/observations/132146925
5277	Bubulcus ibis	Cattle Egret	2022/8/15	4	0	26.8549114	75.8328411	https://www.inaturalist.org/observations/132146931
5278	Bubulcus ibis	Cattle Egret	2022/7/19	2	0	26.8554416	75.8323418	https://www.inaturalist.org/observations/129019097
5279	Bubulcus ibis	Cattle Egret	2022/7/19	3	0	26.8554416	75.8323418	https://www.inaturalist.org/observations/129019099
5280	Bubulcus ibis	Cattle Egret	2022/3/12	3	0	26.8661050	76.3212389	https://www.inaturalist.org/observations/108575551
5281	Bubulcus ibis	Cattle Egret	2016/2/11	1	0	26.8789073	77.3611549	https://www.inaturalist.org/observations/47912711
5282	Bubulcus ibis	Cattle Egret	2020/10/25	3	0	26.8859148	80.9998057	https://www.inaturalist.org/observations/63458711
5283	Bubulcus ibis	Cattle Egret	2013/10/23	3	0	26.8939118	78.2629704	https://www.inaturalist.org/observations/69357593
5284	Bubulcus ibis	Cattle Egret	2020/1/17	1	0	26.9101227	70.8085675	https://www.inaturalist.org/observations/92469014
5285	Bubulcus ibis	Cattle Egret	2016/9/16	1	0	26.9127701	75.7872708	https://www.inaturalist.org/observations/117960430
5286	Bubulcus ibis	Cattle Egret	2022/7/18	2	0	26.9168605	75.8647354	https://www.inaturalist.org/observations/128746131
5287	Bubulcus ibis	Cattle Egret	2022/7/18	1	0	26.9168605	75.8647354	https://www.inaturalist.org/observations/128746132
5288	Bubulcus ibis	Cattle Egret	2011/12/2	3	0	26.9435588	94.2000242	https://www.inaturalist.org/observations/51793961
5289	Bubulcus ibis	Cattle Egret	2020/7/16	1	0	26.9479558	94.2940664	https://www.inaturalist.org/observations/93498333
5290	Bubulcus ibis	Cattle Egret	2020/2/25	2	0	26.9533458	80.9256023	https://www.inaturalist.org/observations/47944963
5291	Bubulcus ibis	Cattle Egret	2017/9/1	5	0	26.9602633	78.9659717	https://www.inaturalist.org/observations/7735419
5292	Bubulcus ibis	Cattle Egret	2022/8/21	5	0	26.9620915	75.8510534	https://www.inaturalist.org/observations/132165062
5293	Bubulcus ibis	Cattle Egret	2022/8/21	5	0	26.9621717	75.8543582	https://www.inaturalist.org/observations/132164990
5294	Bubulcus ibis	Cattle Egret	2020/5/23	1	0	26.9648137	113.1888404	https://www.inaturalist.org/observations/47511476
5295	Bubulcus ibis	Cattle Egret	2018/10/25	2	0	26.9710528	94.2088866	https://www.inaturalist.org/observations/48524021
5296	Bubulcus ibis	Cattle Egret	2017/11/26	2	0	26.9814258	78.2020337	https://www.inaturalist.org/observations/69975109
5297	Bubulcus ibis	Cattle Egret	2022/3/31	5	0	27.0069294	78.9825507	https://www.inaturalist.org/observations/125193747
5298	Bubulcus ibis	Cattle Egret	2018/10/25	2	0	27.0271279	94.1008616	https://www.inaturalist.org/observations/48524045
5299	Bubulcus ibis	Cattle Egret	2020/10/3	7	0	27.1127763	77.9924221	https://www.inaturalist.org/observations/61767364

5300	Bubulcus ibis	Cattle Egret	2022/9/19	1	0	27.1176170	78.0245071	https://www.inaturalist.org/observations/141154838
5301	Bubulcus ibis	Cattle Egret	2018/2/11	1	0	27.1238106	77.9912762	https://www.inaturalist.org/observations/42150112
5302	Bubulcus ibis	Cattle Egret	2020/7/30	2	0	27.1307559	75.6128126	https://www.inaturalist.org/observations/54840368
5303	Bubulcus ibis	Cattle Egret	2011/1/27	4	0	27.1569200	77.5236510	https://www.inaturalist.org/observations/24371115
5304	Bubulcus ibis	Cattle Egret	2019/9/25	4	0	27.1569200	77.5236510	https://www.inaturalist.org/observations/33784148
5305	Bubulcus ibis	Cattle Egret	2013/9/27	4	0	27.1569200	77.5236510	https://www.inaturalist.org/observations/41359941
5306	Bubulcus ibis	Cattle Egret	2013/2/10	4	0	27.1592690	77.5231999	https://www.inaturalist.org/observations/6870063
5307	Bubulcus ibis coromandus	Cattle Egret	2014/12/7	1	0	27.1592690	77.5231999	https://www.inaturalist.org/observations/43032183
5308	Bubulcus ibis	Cattle Egret	2018/7/17	2	0	27.1592690	77.5231999	https://www.inaturalist.org/observations/46906071
5309	Bubulcus ibis	Cattle Egret	2009/1/17	2	0	27.1592690	77.5231999	https://www.inaturalist.org/observations/48211544
5310	Bubulcus ibis	Cattle Egret	2009/1/17	0	0	27.1592690	77.5231999	https://www.inaturalist.org/observations/48211552
5311	Bubulcus ibis	Cattle Egret	2008/12/2	2	0	27.1592690	77.5231999	https://www.inaturalist.org/observations/55245756
5312	Bubulcus ibis	Cattle Egret	2008/9/5	2	0	27.1629346	77.5231999	https://www.inaturalist.org/observations/107804205
5313	Bubulcus ibis	Cattle Egret	2006/1/14	1	0	27.1650000	77.5250000	https://www.inaturalist.org/observations/104654447
5314	Bubulcus ibis	Cattle Egret	2006/1/9	5	0	27.1670000	77.5180000	https://www.inaturalist.org/observations/9771282
5315	Bubulcus ibis	Cattle Egret	2011/12/11	3	0	27.1697148	77.5141323	https://www.inaturalist.org/observations/21936811
5316	Bubulcus ibis coromandus	Cattle Egret	2019/8/10	4	0	27.1711883	77.9721567	https://www.inaturalist.org/observations/34630848
5317	Bubulcus ibis	Cattle Egret	2019/1/2	4	0	27.1714607	78.0418633	https://www.inaturalist.org/observations/120645413
5318	Bubulcus ibis	Cattle Egret	2018/2/9	3	0	27.1721422	78.0413076	https://www.inaturalist.org/observations/22096124
5319	Bubulcus ibis coromandus	Cattle Egret	2019/3/31	1	0	27.1725168	78.0420283	https://www.inaturalist.org/observations/24551826
5320	Bubulcus ibis	Cattle Egret	2011/4/10	1	0	27.1726167	78.0422750	https://www.inaturalist.org/observations/68534107
5321	Bubulcus ibis	Cattle Egret	2019/1/30	6	0	27.1726283	78.0422333	https://www.inaturalist.org/observations/20510694
5322	Bubulcus ibis	Cattle Egret	2020/2/23	1	0	27.1727210	78.0418057	https://www.inaturalist.org/observations/39209016
5323	Bubulcus ibis	Cattle Egret	2011/4/1	5	0	27.1729096	78.0418490	http://www.inaturalist.org/observations/240984
5324	Bubulcus ibis	Cattle Egret	2014/12/31	1	0	27.1729150	78.0427667	https://www.inaturalist.org/observations/74663446

5325	Bubulcus ibis	Cattle Egret	2018/10/24	6	0	27.1730590	78.0421380	https://www.inaturalist.org/observations/131465499
5326	Bubulcus ibis	Cattle Egret	2014/3/31	4	0	27.1730668	78.0420801	https://www.inaturalist.org/observations/127164151
5327	Bubulcus ibis	Cattle Egret	2020/1/2	2	0	27.1731873	78.0423660	https://www.inaturalist.org/observations/37187225
5328	Bubulcus ibis	Cattle Egret	2020/2/23	1	0	27.1731977	78.0432580	https://www.inaturalist.org/observations/104564161
5329	Bubulcus ibis	Cattle Egret	2020/1/2	1	0	27.1732349	78.0423508	https://www.inaturalist.org/observations/37187874
5330	Bubulcus ibis	Cattle Egret	2014/5/24	2	0	27.1732981	78.0421639	https://www.inaturalist.org/observations/69290603
5331	Bubulcus ibis	Cattle Egret	2012/5/8	2	0	27.1735202	78.0427601	https://www.inaturalist.org/observations/63352379
5332	Bubulcus ibis	Cattle Egret	2016/12/15	3	0	27.1735986	78.0425928	https://www.inaturalist.org/observations/37316422
5333	Bubulcus ibis	Cattle Egret	2019/3/3	1	0	27.1742106	78.0423010	https://www.inaturalist.org/observations/49614270
5334	Bubulcus ibis	Cattle Egret	2022/5/30	2	0	27.1744516	78.0423520	https://www.inaturalist.org/observations/119397851
5335	Bubulcus ibis	Cattle Egret	2019/1/10	2	0	27.1749594	78.0419860	https://www.inaturalist.org/observations/52147721
5336	Bubulcus ibis	Cattle Egret	2016/11/17	4	0	27.1750090	78.0420906	http://www.inaturalist.org/observations/4683330
5337	Bubulcus ibis	Cattle Egret	2010/7/15	5	0	27.1751360	78.0420874	https://www.inaturalist.org/observations/108791426
5338	Bubulcus ibis	Cattle Egret	2022/7/12	6	0	27.1751448	78.0421422	https://www.inaturalist.org/observations/127471866
5339	Bubulcus ibis coromandus	Cattle Egret	2019/8/10	4	0	27.1753833	78.0421250	https://www.inaturalist.org/observations/34630135
5340	Bubulcus ibis coromandus	Cattle Egret	2019/8/10	1	0	27.1754100	78.0421317	https://www.inaturalist.org/observations/34629585
5341	Bubulcus ibis	Cattle Egret	2019/3/3	1	0	27.1764585	78.0333516	https://www.inaturalist.org/observations/49616863
5342	Bubulcus ibis	Cattle Egret	2017/11/27	3	0	27.1765000	78.0418000	https://www.inaturalist.org/observations/69972846
5343	Bubulcus ibis	Cattle Egret	2017/10/22	2	0	27.1766701	78.0080745	https://www.inaturalist.org/observations/45732597
5344	Bubulcus ibis	Cattle Egret	2020/3/17	2	0	27.1779567	77.5187083	https://www.inaturalist.org/observations/47555227
5345	Bubulcus ibis	Cattle Egret	2016/2/11	2	0	27.1795329	78.0211121	https://www.inaturalist.org/observations/47874005
5346	Bubulcus ibis	Cattle Egret	2021/6/14	1	0	27.1877630	113.8286601	https://www.inaturalist.org/observations/85224169
5347	Bubulcus ibis	Cattle Egret	2021/7/14	1	0	27.1884310	113.8287110	https://www.inaturalist.org/observations/88641079
5348	Bubulcus ibis	Cattle Egret	2007/1/16	5	0	27.1985527	77.4842119	https://www.inaturalist.org/observations/8266722
5349	Bubulcus ibis	Cattle Egret	2013/12/25	1	0	27.2072208	77.5131798	https://www.inaturalist.org/observations/67015050

5350	Bubulcus ibis	Cattle Egret	2022/9/4	3	0	27.2151817	77.5029995	https://www.inaturalist.org/observations/133726642
5351	Bubulcus ibis	Cattle Egret	2004/12/17	3	0	27.2151863	77.5029996	https://www.inaturalist.org/observations/70912619
5352	Bubulcus ibis	Cattle Egret	2020/6/9	2	0	27.2236655	78.0162381	https://www.inaturalist.org/observations/48968975
5353	Bubulcus ibis	Cattle Egret	2016/11/13	2	0	27.3194314	95.5103451	https://www.inaturalist.org/observations/105926512
5354	Bubulcus ibis	Cattle Egret	2011/2/22	4	0	27.4728078	77.9936547	https://www.inaturalist.org/observations/17855207
5355	Bubulcus ibis	Cattle Egret	2016/4/17	5	0	27.5788683	95.3917459	https://www.inaturalist.org/observations/19516788
5356	Bubulcus ibis	Cattle Egret	2021/5/10	1	0	27.5946867	117.3927817	https://www.inaturalist.org/observations/88837490
5357	Bubulcus ibis	Cattle Egret	2022/7/27	1	0	27.7441896	108.8195120	https://www.inaturalist.org/observations/130074821
5358	Bubulcus ibis	Cattle Egret	2017/7/14	1	0	27.7971559	74.4337676	https://www.inaturalist.org/observations/105545965
5359	Bubulcus ibis	Cattle Egret	2019/5/18	1	0	27.8774330	108.6575429	https://www.inaturalist.org/observations/38632138
5360	Bubulcus ibis	Cattle Egret	2022/6/8	3	0	27.8965988	74.6296082	https://www.inaturalist.org/observations/120751774
5361	Bubulcus ibis	Cattle Egret	2019/12/12	2	0	27.9634793	73.3826585	https://www.inaturalist.org/observations/43258534
5362	Bubulcus ibis	Cattle Egret	2022/7/17	4	0	27.9646908	76.3773719	https://www.inaturalist.org/observations/126634113
5363	Bubulcus ibis	Cattle Egret	2017/1/16	4	0	28.0229348	73.3119159	https://www.inaturalist.org/observations/5013160
5364	Bubulcus ibis	Cattle Egret	2017/7/18	3	0	28.1095242	114.4883430	https://www.inaturalist.org/observations/7489595
5365	Bubulcus ibis	Cattle Egret	2017/7/4	4	0	28.2190841	79.5248499	https://www.inaturalist.org/observations/6915858
5366	Bubulcus ibis	Cattle Egret	2017/7/4	4	0	28.2775593	79.5842536	https://www.inaturalist.org/observations/6915908
5367	Bubulcus ibis	Cattle Egret	2022/1/22	3	0	28.2850431	77.4554456	https://www.inaturalist.org/observations/105352717
5368	Bubulcus ibis coromandus	Cattle Egret	2017/6/23	6	0	28.3104448	79.2182128	https://www.inaturalist.org/observations/6764178
5369	Bubulcus ibis	Cattle Egret	2017/7/14	4	0	28.3272212	79.2968738	https://www.inaturalist.org/observations/7102710
5370	Bubulcus ibis	Cattle Egret	2018/11/6	1	0	28.3412469	77.9253153	https://www.inaturalist.org/observations/91083638
5371	Bubulcus ibis	Cattle Egret	2021/8/5	1	0	28.3504718	77.5564339	https://www.inaturalist.org/observations/90053720
5372	Bubulcus ibis	Cattle Egret	2020/2/17	0	0	28.3733370	77.5415446	https://www.inaturalist.org/observations/38748706
5373	Bubulcus ibis	Cattle Egret	2019/8/16	5	0	28.3940667	79.4589250	https://www.inaturalist.org/observations/31264590
5374	Bubulcus ibis	Cattle Egret	2021/6/11	2	0	28.3954316	77.0596726	https://www.inaturalist.org/observations/82564065

5375	Bubulcus ibis coromandus	Cattle Egret	2021/7/17	3	0	28.4522965	77.5038890	https://www.inaturalist.org/observations/87368250
5376	Bubulcus ibis	Cattle Egret	2021/8/3	1	0	28.4522965	77.5038890	https://www.inaturalist.org/observations/89777075
5377	Bubulcus ibis	Cattle Egret	2022/11/12	2	0	28.4543910	119.9192730	https://www.inaturalist.org/observations/141763352
5378	Bubulcus ibis	Cattle Egret	2020/2/10	2	0	28.4564579	76.8890506	https://www.inaturalist.org/observations/61820093
5379	Bubulcus ibis	Cattle Egret	2020/1/4	4	0	28.4668117	76.8918933	https://www.inaturalist.org/observations/65639112
5380	Bubulcus ibis	Cattle Egret	2016/10/9	3	0	28.4685990	77.2907380	http://www.inaturalist.org/observations/4306472
5381	Bubulcus ibis	Cattle Egret	2011/2/5	5	0	28.4695998	76.9795532	https://www.inaturalist.org/observations/31497889
5382	Bubulcus ibis	Cattle Egret	2011/8/8	5	0	28.4695998	76.9795532	https://www.inaturalist.org/observations/31511912
5383	Bubulcus ibis	Cattle Egret	2010/7/31	5	0	28.4695998	76.9795532	https://www.inaturalist.org/observations/31576943
5384	Bubulcus ibis	Cattle Egret	2022/5/24	3	0	28.4713993	77.4804993	https://www.inaturalist.org/observations/118506379
5385	Bubulcus ibis	Cattle Egret	2020/9/24	2	0	28.5126977	77.4841858	https://www.inaturalist.org/observations/60819254
5386	Bubulcus ibis	Cattle Egret	2020/8/21	1	0	28.5355161	77.3910265	https://www.inaturalist.org/observations/57536199
5387	Bubulcus ibis	Cattle Egret	2020/7/9	4	0	28.5359756	77.3910266	https://www.inaturalist.org/observations/55149377
5388	Bubulcus ibis	Cattle Egret	2020/7/9	5	0	28.5359756	77.3910266	https://www.inaturalist.org/observations/55149793
5389	Bubulcus ibis	Cattle Egret	2021/8/8	1	0	28.5514466	77.3185215	https://www.inaturalist.org/observations/90364331
5390	Bubulcus ibis	Cattle Egret	2009/4/11	4	0	28.5525772	77.5548100	https://www.inaturalist.org/observations/31585017
5391	Bubulcus ibis	Cattle Egret	2009/7/15	5	0	28.5572098	77.2127891	https://www.inaturalist.org/observations/31583632
5392	Bubulcus ibis	Cattle Egret	2020/1/20	2	0	28.5889922	77.3174716	https://www.inaturalist.org/observations/105275209
5393	Bubulcus ibis	Cattle Egret	2019/12/25	2	0	28.5914554	77.2417831	https://www.inaturalist.org/observations/36936798
5394	Bubulcus ibis	Cattle Egret	2019/2/18	2	0	28.5944436	77.2453998	https://www.inaturalist.org/observations/33783687
5395	Bubulcus ibis	Cattle Egret	2021/7/9	1	0	28.5944950	77.0275414	https://www.inaturalist.org/observations/88651532
5396	Bubulcus ibis	Cattle Egret	2021/6/28	1	0	28.5944950	77.0275414	https://www.inaturalist.org/observations/88651945
5397	Bubulcus ibis	Cattle Egret	2021/12/31	4	0	28.6010180	77.3789320	https://www.inaturalist.org/observations/104022274
5398	Bubulcus ibis	Cattle Egret	2020/12/14	4	0	28.6015864	77.2484270	https://www.inaturalist.org/observations/66569063
5399	Bubulcus ibis	Cattle Egret	2021/10/24	1	0	28.6053969	77.3395587	https://www.inaturalist.org/observations/99189527

5400	Bubulcus ibis	Cattle Egret	2021/6/27	1	0	28.6067011	77.3388959	https://www.inaturalist.org/observations/84660944
5401	Bubulcus ibis coromandus	Cattle Egret	2021/1/29	2	0	28.6072118	77.5930828	https://www.inaturalist.org/observations/68755323
5402	Bubulcus ibis	Cattle Egret	2020/6/20	1	0	28.6362565	77.0791108	https://www.inaturalist.org/observations/50375112
5403	Bubulcus ibis	Cattle Egret	2019/12/25	1	0	28.6413346	77.2428131	https://www.inaturalist.org/observations/36936796
5404	Bubulcus ibis	Cattle Egret	2022/7/1	4	0	28.6550170	77.2436433	https://www.inaturalist.org/observations/134113162
5405	Bubulcus ibis	Cattle Egret	2019/3/20	1	0	28.6634302	77.2465782	https://www.inaturalist.org/observations/39880640
5406	Bubulcus ibis	Cattle Egret	2020/11/13	1	0	28.6683598	77.1905289	https://www.inaturalist.org/observations/64785052
5407	Bubulcus ibis	Cattle Egret	2020/11/5	1	0	28.6686039	77.1902542	https://www.inaturalist.org/observations/64508564
5408	Bubulcus ibis	Cattle Egret	2021/11/15	2	0	28.6862738	77.2217831	https://www.inaturalist.org/observations/101130817
5409	Bubulcus ibis	Cattle Egret	2022/6/10	4	0	28.6870230	77.3177246	https://www.inaturalist.org/observations/126957298
5410	Bubulcus ibis	Cattle Egret	2022/3/8	1	0	28.8289391	78.6854228	https://www.inaturalist.org/observations/108245617
5411	Bubulcus ibis	Cattle Egret	2022/7/23	3	0	28.8389459	78.6990397	https://www.inaturalist.org/observations/128125687
5412	Bubulcus ibis	Cattle Egret	2020/9/16	2	0	28.8833810	79.7594390	https://www.inaturalist.org/observations/59802218
5413	Bubulcus ibis	Cattle Egret	2021/9/26	1	0	28.9065560	77.3570730	https://www.inaturalist.org/observations/96333887
5414	Bubulcus ibis	Cattle Egret	2022/8/8	6	0	28.9068340	77.3551656	https://www.inaturalist.org/observations/134338796
5415	Bubulcus ibis	Cattle Egret	2022/8/8	3	0	28.9068340	77.3551656	https://www.inaturalist.org/observations/134338797
5416	Bubulcus ibis	Cattle Egret	2022/8/8	3	0	28.9068340	77.3551656	https://www.inaturalist.org/observations/134338802
5417	Bubulcus ibis	Cattle Egret	2022/8/8	4	0	28.9068340	77.3551656	https://www.inaturalist.org/observations/134338803
5418	Bubulcus ibis	Cattle Egret	2021/12/11	1	0	28.9301090	77.6983390	https://www.inaturalist.org/observations/102858647
5419	Bubulcus ibis	Cattle Egret	2021/3/7	2	0	28.9384166	79.8055316	https://www.inaturalist.org/observations/70730101
5420	Bubulcus ibis	Cattle Egret	2021/9/25	2	0	28.9392160	79.7964220	https://www.inaturalist.org/observations/96076137
5421	Bubulcus ibis	Cattle Egret	2020/9/19	3	0	28.9400508	79.7911786	https://www.inaturalist.org/observations/60061340
5422	Bubulcus ibis	Cattle Egret	2021/5/1	2	0	28.9409797	79.8156668	https://www.inaturalist.org/observations/76122709
5423	Bubulcus ibis	Cattle Egret	2021/5/7	1	0	28.9416923	79.7990265	https://www.inaturalist.org/observations/78193854
5424	Bubulcus ibis	Cattle Egret	2020/9/15	4	0	28.9436474	79.8115845	https://www.inaturalist.org/observations/59660000

5425	Bubulcus ibis	Cattle Egret	2020/9/19	3	0	28.9459915	79.8119125	https://www.inaturalist.org/observations/60061605
5426	Bubulcus ibis	Cattle Egret	2021/4/30	2	0	28.9463711	79.7883911	https://www.inaturalist.org/observations/75755619
5427	Bubulcus ibis	Cattle Egret	2021/9/26	1	0	28.9475861	79.8102417	https://www.inaturalist.org/observations/96200516
5428	Bubulcus ibis	Cattle Egret	2021/5/2	1	0	28.9495481	79.7604903	https://www.inaturalist.org/observations/76417803
5429	Bubulcus ibis	Cattle Egret	2021/9/27	0	0	28.9516110	79.7545330	https://www.inaturalist.org/observations/96482870
5430	Bubulcus ibis	Cattle Egret	2021/9/25	1	0	28.9529571	79.8004303	https://www.inaturalist.org/observations/96045625
5431	Bubulcus ibis	Cattle Egret	2022/4/16	2	0	28.9532610	79.7808130	https://www.inaturalist.org/observations/144592613
5432	Bubulcus ibis	Cattle Egret	2022/4/16	2	0	28.9532610	79.7808130	https://www.inaturalist.org/observations/144592614
5433	Bubulcus ibis	Cattle Egret	2021/5/15	2	0	28.9534784	79.7999816	https://www.inaturalist.org/observations/78939479
5434	Bubulcus ibis	Cattle Egret	2021/3/19	1	0	28.9535972	79.8007250	https://www.inaturalist.org/observations/71523202
5435	Bubulcus ibis	Cattle Egret	2021/3/26	2	0	28.9537703	79.8000228	https://www.inaturalist.org/observations/73318425
5436	Bubulcus ibis	Cattle Egret	2021/9/25	1	0	28.9547801	79.7808132	https://www.inaturalist.org/observations/96082986
5437	Bubulcus ibis	Cattle Egret	2020/6/22	2	0	28.9550467	77.7553633	https://www.inaturalist.org/observations/50532934
5438	Bubulcus ibis	Cattle Egret	2020/9/13	4	0	28.9551111	79.8024167	https://www.inaturalist.org/observations/59417113
5439	Bubulcus ibis	Cattle Egret	2021/9/24	3	0	28.9574717	79.8077935	https://www.inaturalist.org/observations/95940927
5440	Bubulcus ibis	Cattle Egret	2021/9/26	1	0	28.9580556	79.8075000	https://www.inaturalist.org/observations/96249593
5441	Bubulcus ibis	Cattle Egret	2021/5/1	1	0	28.9599133	79.8027267	https://www.inaturalist.org/observations/76399814
5442	Bubulcus ibis	Cattle Egret	2021/7/22	2	0	28.9609705	79.8053242	https://www.inaturalist.org/observations/88230247
5443	Bubulcus ibis	Cattle Egret	2021/8/25	2	0	28.9619218	79.8054885	https://www.inaturalist.org/observations/92457952
5444	Bubulcus ibis	Cattle Egret	2022/7/8	3	0	28.9619528	79.8055250	https://www.inaturalist.org/observations/125243500
5445	Bubulcus ibis	Cattle Egret	2022/9/4	5	0	28.9619556	79.8053472	https://www.inaturalist.org/observations/133571760
5446	Bubulcus ibis	Cattle Egret	2021/8/17	2	0	28.9620577	79.8054875	https://www.inaturalist.org/observations/91543339
5447	Bubulcus ibis	Cattle Egret	2021/7/19	2	0	28.9627667	79.8055891	https://www.inaturalist.org/observations/87711311
5448	Bubulcus ibis	Cattle Egret	2022/7/9	3	0	28.9628056	79.8056139	https://www.inaturalist.org/observations/125391485
5449	Bubulcus ibis	Cattle Egret	2022/7/25	3	0	28.9628722	79.8055194	https://www.inaturalist.org/observations/127867495

5450	Bubulcus ibis	Cattle Egret	2021/12/26	2	0	28.9628913	79.8054553	https://www.inaturalist.org/observations/103689602
5451	Bubulcus ibis	Cattle Egret	2021/10/18	3	0	28.9630673	79.8054482	https://www.inaturalist.org/observations/98932025
5452	Bubulcus ibis	Cattle Egret	2022/7/4	5	0	28.9636197	79.8049946	https://www.inaturalist.org/observations/124657813
5453	Bubulcus ibis	Cattle Egret	2021/12/22	3	0	28.9638541	79.8041474	https://www.inaturalist.org/observations/103466642
5454	Bubulcus ibis	Cattle Egret	2021/6/30	2	0	28.9640870	79.8039620	https://www.inaturalist.org/observations/85064255
5455	Bubulcus ibis	Cattle Egret	2021/9/25	1	0	28.9640884	79.8000793	https://www.inaturalist.org/observations/96242768
5456	Bubulcus ibis	Cattle Egret	2021/6/17	3	0	28.9643862	79.8042932	https://www.inaturalist.org/observations/83366619
5457	Bubulcus ibis	Cattle Egret	2021/5/2	1	0	28.9702660	79.7443690	https://www.inaturalist.org/observations/76422507
5458	Bubulcus ibis	Cattle Egret	2021/9/26	1	0	28.9711111	79.7652778	https://www.inaturalist.org/observations/96207003
5459	Bubulcus ibis	Cattle Egret	2020/9/12	3	0	28.9721228	79.7649295	https://www.inaturalist.org/observations/59858876
5460	Bubulcus ibis	Cattle Egret	2021/5/2	2	0	28.9738283	79.7815139	https://www.inaturalist.org/observations/76442833
5461	Bubulcus ibis	Cattle Egret	2021/5/23	1	0	28.9806415	79.7906226	https://www.inaturalist.org/observations/79937824
5462	Bubulcus ibis	Cattle Egret	2021/5/1	1	0	28.9861949	79.8044588	https://www.inaturalist.org/observations/77529558
5463	Bubulcus ibis	Cattle Egret	2021/9/19	2	0	28.9898254	79.7740969	https://www.inaturalist.org/observations/95357929
5464	Bubulcus ibis	Cattle Egret	2022/10/4	2	0	28.9998194	77.0443361	https://www.inaturalist.org/observations/137636535
5465	Bubulcus ibis	Cattle Egret	2019/4/25	1	0	29.0228626	79.4879412	https://www.inaturalist.org/observations/48679230
5466	Bubulcus ibis	Cattle Egret	2021/12/24	4	0	29.0233950	79.4847070	https://www.inaturalist.org/observations/103571241
5467	Bubulcus ibis	Cattle Egret	2020/12/27	1	0	29.0256540	79.4810130	https://www.inaturalist.org/observations/67125347
5468	Bubulcus ibis coromandus	Cattle Egret	2019/5/8	1	0	29.0835373	117.7638963	https://www.inaturalist.org/observations/31774353
5469	Bubulcus ibis	Cattle Egret	2021/6/18	1	0	29.1549699	117.8568600	https://www.inaturalist.org/observations/109620455
5470	Bubulcus ibis	Cattle Egret	2017/9/5	4	0	29.1889388	78.9520039	https://www.inaturalist.org/observations/8086438
5471	Bubulcus ibis	Cattle Egret	2021/3/17	2	0	29.2182639	79.5129768	https://www.inaturalist.org/observations/104498069
5472	Bubulcus ibis	Cattle Egret	2020/6/26	3	0	29.2331723	79.0121700	https://www.inaturalist.org/observations/50973236
5473	Bubulcus ibis	Cattle Egret	2020/6/13	1	0	29.2337143	79.0116822	https://www.inaturalist.org/observations/49427356
5474	Bubulcus ibis	Cattle Egret	2021/9/12	3	0	29.2877629	79.1082079	https://www.inaturalist.org/observations/96682418

5475	Bubulcus ibis	Cattle Egret	2013/1/27	5	0	29.3803039	79.4635658	https://www.inaturalist.org/observations/31520522
5476	Bubulcus ibis	Cattle Egret	2022/12/6	1	0	29.3980225	79.0831306	https://www.inaturalist.org/observations/144402420
5477	Bubulcus ibis coromandus	Cattle Egret	2020/6/16	0	0	29.4331733	103.6106813	https://www.inaturalist.org/observations/49810664
5478	Bubulcus ibis	Cattle Egret	2017/3/10	1	0	29.4565213	79.1482658	https://www.inaturalist.org/observations/102513165
5479	Bubulcus ibis	Cattle Egret	2020/8/22	1	0	29.5135270	76.9072850	https://www.inaturalist.org/observations/57213230
5480	Bubulcus ibis	Cattle Egret	2020/5/12	1	0	29.5143712	76.9098098	https://www.inaturalist.org/observations/57235908
5481	Bubulcus ibis	Cattle Egret	2020/5/2	2	0	29.6882676	76.9977508	https://www.inaturalist.org/observations/44592925
5482	Bubulcus ibis	Cattle Egret	2021/6/7	1	0	29.7677150	121.3306461	https://www.inaturalist.org/observations/82006130
5483	Bubulcus ibis	Cattle Egret	2022/8/7	2	0	29.7687213	78.0100331	https://www.inaturalist.org/observations/129827269
5484	Bubulcus ibis	Cattle Egret	2021/4/25	1	0	29.7687278	121.3284692	https://www.inaturalist.org/observations/75157943
5485	Bubulcus ibis	Cattle Egret	2020/10/29	1	0	29.8042699	76.4039016	https://www.inaturalist.org/observations/63753397
5486	Bubulcus ibis	Cattle Egret	2020/9/2	3	0	29.8173494	76.4173782	https://www.inaturalist.org/observations/60044452
5487	Bubulcus ibis	Cattle Egret	2017/8/19	4	0	29.9040146	78.1786499	https://www.inaturalist.org/observations/139173224
5488	Bubulcus ibis	Cattle Egret	2022/8/3	1	0	29.9562359	77.5525393	https://www.inaturalist.org/observations/129241228
5489	Bubulcus ibis	Cattle Egret	2020/6/11	2	0	30.0826290	105.2598309	https://www.inaturalist.org/observations/143670814
5490	Bubulcus ibis	Cattle Egret	2019/4/2	4	0	30.0985837	77.8056682	https://www.inaturalist.org/observations/36864952
5491	Bubulcus ibis	Cattle Egret	2021/8/21	4	0	30.2093250	120.1381305	https://www.inaturalist.org/observations/92050461
5492	Bubulcus ibis	Cattle Egret	2022/9/5	2	0	30.2386474	119.7501659	https://www.inaturalist.org/observations/133744569
5493	Bubulcus ibis	Cattle Egret	2021/9/21	2	0	30.2418194	120.1580064	https://www.inaturalist.org/observations/95750351
5494	Bubulcus ibis	Cattle Egret	2011/4/17	2	0	30.2430278	119.7888221	https://www.inaturalist.org/observations/27004049
5495	Bubulcus ibis	Cattle Egret	2021/7/25	1	0	30.2480546	119.9504986	https://www.inaturalist.org/observations/88505877
5496	Bubulcus ibis	Cattle Egret	2020/7/1	4	0	30.2671897	78.0075192	https://www.inaturalist.org/observations/51710443
5497	Bubulcus ibis	Cattle Egret	2019/5/4	1	0	30.2691765	120.0665091	https://www.inaturalist.org/observations/38790350
5498	Bubulcus ibis coromandus	Cattle Egret	2015/6/11	6	0	30.2833210	77.9728840	http://www.inaturalist.org/observations/1617501
5499	Bubulcus ibis	Cattle Egret	2020/9/5	3	0	30.3028788	78.0826231	https://www.inaturalist.org/observations/58583140

5500	Bubulcus ibis	Cattle Egret	2020/9/22	3	0	30.3169593	78.0321919	https://www.inaturalist.org/observations/60401252
5501	Bubulcus ibis	Cattle Egret	2021/12/12	2	0	30.3177011	78.0593419	https://www.inaturalist.org/observations/115096814
5502	Bubulcus ibis	Cattle Egret	2022/7/10	4	0	30.3390529	77.8813074	https://www.inaturalist.org/observations/141167015
5503	Bubulcus ibis	Cattle Egret	2022/7/12	1	0	30.3390979	77.8811049	https://www.inaturalist.org/observations/141271746
5504	Bubulcus ibis	Cattle Egret	2022/8/5	2	0	30.3454610	77.9929978	https://www.inaturalist.org/observations/129496936
5505	Bubulcus ibis	Cattle Egret	2022/8/3	2	0	30.3455695	77.9942051	https://www.inaturalist.org/observations/129364656
5506	Bubulcus ibis	Cattle Egret	2022/6/27	1	0	30.3521115	119.9318164	https://www.inaturalist.org/observations/125913142
5507	Bubulcus ibis	Cattle Egret	2021/4/21	1	0	30.3644134	76.7880194	https://www.inaturalist.org/observations/75049162
5508	Bubulcus ibis	Cattle Egret	2021/5/25	1	0	30.3644134	76.7880194	https://www.inaturalist.org/observations/82802681
5509	Bubulcus ibis	Cattle Egret	2020/9/3	3	0	30.3644203	76.7880194	https://www.inaturalist.org/observations/58419013
5510	Bubulcus ibis	Cattle Egret	2020/9/3	3	0	30.3644203	76.7880194	https://www.inaturalist.org/observations/58419106
5511	Bubulcus ibis coromandus	Cattle Egret	2018/8/3	3	0	30.3924654	120.0064385	https://www.inaturalist.org/observations/18135428
5512	Bubulcus ibis coromandus	Cattle Egret	2020/5/17	0	0	30.4482469	77.7549491	https://www.inaturalist.org/observations/134571487
5513	Bubulcus ibis	Cattle Egret	2020/5/17	2	0	30.4482665	77.7549536	https://www.inaturalist.org/observations/107528613
5514	Bubulcus ibis	Cattle Egret	2021/11/5	1	0	30.4597892	78.0643723	https://www.inaturalist.org/observations/100316914
5515	Bubulcus ibis	Cattle Egret	2018/11/18	6	0	30.5913084	74.8115340	https://www.inaturalist.org/observations/18510012
5516	Bubulcus ibis	Cattle Egret	2022/9/1	1	0	30.6139998	76.9326166	https://www.inaturalist.org/observations/133347768
5517	Bubulcus ibis	Cattle Egret	2019/5/12	3	0	30.6509950	76.4604667	https://www.inaturalist.org/observations/124431173
5518	Bubulcus ibis	Cattle Egret	2017/8/15	3	0	30.6634083	76.7273026	https://www.inaturalist.org/observations/7565759
5519	Bubulcus ibis	Cattle Egret	2020/7/21	5	0	30.6778332	76.7091771	https://www.inaturalist.org/observations/131553174
5520	Bubulcus ibis	Cattle Egret	2020/8/3	2	0	30.7097342	76.7236264	https://www.inaturalist.org/observations/55250007
5521	Bubulcus ibis	Cattle Egret	2022/6/5	3	0	30.7367000	76.6447915	https://www.inaturalist.org/observations/120287373
5522	Bubulcus ibis	Cattle Egret	2020/9/22	1	0	30.8908140	121.5894270	https://www.inaturalist.org/observations/60407875
5523	Bubulcus ibis	Cattle Egret	2022/9/23	1	0	30.9375684	121.8706514	https://www.inaturalist.org/observations/136929226
5524	Bubulcus ibis	Cattle Egret	2012/7/22	4	0	31.0732496	75.4011421	https://www.inaturalist.org/observations/133036746

5525	Bubulcus ibis	Cattle Egret	2021/4/21	2	0	31.0746570	121.1487090	https://www.inaturalist.org/observations/74802688
5526	Bubulcus ibis	Cattle Egret	2013/12/27	2	0	31.1434568	74.9513447	https://www.inaturalist.org/observations/36295596
5527	Bubulcus ibis	Cattle Egret	2021/10/16	2	0	31.1731566	120.5365409	https://www.inaturalist.org/observations/98337125
5528	Bubulcus ibis	Cattle Egret	2020/8/5	3	0	31.1771214	121.6054250	https://www.inaturalist.org/observations/55476313
5529	Bubulcus ibis coromandus	Cattle Egret	2005/5/19	1	0	31.1940585	121.3645503	https://www.inaturalist.org/observations/140623785
5530	Bubulcus ibis	Cattle Egret	2020/7/19	1	0	31.2557047	119.6091068	https://www.inaturalist.org/observations/102759277
5531	Bubulcus ibis	Cattle Egret	2020/7/20	2	0	31.2922157	119.6257733	https://www.inaturalist.org/observations/102844646
5532	Bubulcus ibis	Cattle Egret	2022/6/23	1	0	31.3051500	119.0879100	https://www.inaturalist.org/observations/126303953
5533	Bubulcus ibis	Cattle Egret	2022/7/20	1	0	31.3106801	118.4866622	https://www.inaturalist.org/observations/127084580
5534	Bubulcus ibis	Cattle Egret	2018/7/15	4	0	31.3260152	75.5761829	https://www.inaturalist.org/observations/14468532
5535	Bubulcus ibis	Cattle Egret	2021/5/30	2	0	31.3534489	121.2010990	https://www.inaturalist.org/observations/80857619
5536	Bubulcus ibis	Cattle Egret	2021/5/30	1	0	31.3587950	121.1916117	https://www.inaturalist.org/observations/80859054
5537	Bubulcus ibis	Cattle Egret	2021/4/20	1	0	31.3733215	120.3829097	https://www.inaturalist.org/observations/74610211
5538	Bubulcus ibis	Cattle Egret	2021/6/7	2	0	31.3989048	120.8331070	https://www.inaturalist.org/observations/81985888
5539	Bubulcus ibis	Cattle Egret	2021/6/28	1	0	31.4000000	119.4000000	https://www.inaturalist.org/observations/84952428
5540	Bubulcus ibis	Cattle Egret	2020/10/11	1	0	31.4474084	120.7836914	https://www.inaturalist.org/observations/62275646
5541	Bubulcus ibis	Cattle Egret	2020/5/19	1	0	31.4936160	120.5066830	https://www.inaturalist.org/observations/46490708
5542	Bubulcus ibis	Cattle Egret	2020/5/19	1	0	31.4936160	120.5066830	https://www.inaturalist.org/observations/46490714
5543	Bubulcus ibis	Cattle Egret	2020/5/19	1	0	31.4936160	120.5066830	https://www.inaturalist.org/observations/46490718
5544	Bubulcus ibis	Cattle Egret	2021/6/19	1	0	31.4954617	119.0880750	https://www.inaturalist.org/observations/83672227
5545	Bubulcus ibis	Cattle Egret	2022/7/21	2	0	31.5161391	118.9297398	https://www.inaturalist.org/observations/127224447
5546	Bubulcus ibis	Cattle Egret	2022/7/21	1	0	31.5161391	118.9297398	https://www.inaturalist.org/observations/127370381
5547	Bubulcus ibis	Cattle Egret	2020/7/18	1	0	31.5506430	121.8118395	https://www.inaturalist.org/observations/127972140
5548	Bubulcus ibis	Cattle Egret	2021/6/19	1	0	31.5619683	118.9780000	https://www.inaturalist.org/observations/87858124
5549	Bubulcus ibis	Cattle Egret	2022/6/9	0	0	31.6044300	120.7362583	https://www.inaturalist.org/observations/120900114

5550	Bubulcus ibis	Cattle Egret	2020/5/9	1	0	31.6058452	121.5399884	https://www.inaturalist.org/observations/131073137
5551	Bubulcus ibis	Cattle Egret	2021/5/1	1	0	31.6437266	119.2473831	https://www.inaturalist.org/observations/77293831
5552	Bubulcus ibis	Cattle Egret	2020/5/22	0	0	31.6473502	121.5942645	https://www.inaturalist.org/observations/131060453
5553	Bubulcus ibis	Cattle Egret	2021/5/3	1	0	31.6496686	119.2150937	https://www.inaturalist.org/observations/77310330
5554	Bubulcus ibis	Cattle Egret	2021/5/3	1	0	31.6496800	119.2150905	https://www.inaturalist.org/observations/77310288
5555	Bubulcus ibis	Cattle Egret	2021/5/3	1	0	31.6496800	119.2150905	https://www.inaturalist.org/observations/77310291
5556	Bubulcus ibis	Cattle Egret	2021/5/3	1	0	31.6496941	119.2150891	https://www.inaturalist.org/observations/77310388
5557	Bubulcus ibis	Cattle Egret	2021/5/2	1	0	31.6498940	119.2158416	https://www.inaturalist.org/observations/77293880
5558	Bubulcus ibis	Cattle Egret	2021/5/3	1	0	31.6499577	119.2152554	https://www.inaturalist.org/observations/77310299
5559	Bubulcus ibis	Cattle Egret	2020/5/15	0	0	31.7446912	121.6369506	https://www.inaturalist.org/observations/131061538
5560	Bubulcus ibis	Cattle Egret	2020/6/6	1	0	31.7471277	121.7197619	https://www.inaturalist.org/observations/127470962
5561	Bubulcus ibis	Cattle Egret	2022/6/30	0	0	31.7869616	118.6246386	https://www.inaturalist.org/observations/126304474
5562	Bubulcus ibis	Cattle Egret	2021/8/25	1	0	31.8381683	118.5103367	https://www.inaturalist.org/observations/92647187
5563	Bubulcus ibis	Cattle Egret	2021/8/25	1	0	31.8381683	118.5103367	https://www.inaturalist.org/observations/92647188
5564	Bubulcus ibis	Cattle Egret	2021/8/25	2	0	31.8381683	118.5103367	https://www.inaturalist.org/observations/92647190
5565	Bubulcus ibis	Cattle Egret	2015/9/4	1	0	31.8894128	118.8157261	https://www.inaturalist.org/observations/141880866
5566	Bubulcus ibis	Cattle Egret	2021/1/10	4	0	32.0269411	75.3939902	https://www.inaturalist.org/observations/68003661
5567	Bubulcus ibis	Cattle Egret	2020/9/24	1	0	32.0724850	118.8968030	https://www.inaturalist.org/observations/67986789
5568	Bubulcus ibis	Cattle Egret	2021/8/29	2	0	32.0880585	118.8343277	https://www.inaturalist.org/observations/142067379
5569	Bubulcus ibis	Cattle Egret	2021/9/4	2	0	32.0880623	118.8344421	https://www.inaturalist.org/observations/142067173
5570	Bubulcus ibis	Cattle Egret	2020/5/12	3	0	32.0880707	76.5496703	https://www.inaturalist.org/observations/45677930
5571	Bubulcus ibis	Cattle Egret	2022/10/2	1	0	32.1802131	118.8592194	https://www.inaturalist.org/observations/141088159
5572	Bubulcus ibis	Cattle Egret	2022/1/2	1	0	32.1936400	118.9874167	https://www.inaturalist.org/observations/120273510
5573	Bubulcus ibis	Cattle Egret	2019/7/15	2	0	32.2008679	76.3621045	https://www.inaturalist.org/observations/105433105
5574	Bubulcus ibis	Cattle Egret	2021/11/11	1	0	32.2086350	76.3324250	https://www.inaturalist.org/observations/100817085

5575	Bubulcus ibis	Cattle Egret	2015/6/21	3	0	32.2190420	76.3234037	https://www.inaturalist.org/observations/30503288
5576	Bubulcus ibis	Cattle Egret	2022/7/16	1	0	32.3210648	118.8084889	https://www.inaturalist.org/observations/126613259
5577	Bubulcus ibis	Cattle Egret	2022/5/15	1	0	32.3222199	118.8215500	https://www.inaturalist.org/observations/126652587
5578	Bubulcus ibis	Cattle Egret	2021/5/3	1	0	32.4564171	119.5849762	https://www.inaturalist.org/observations/142515920
5579	Bubulcus ibis coromandus	Cattle Egret	2020/9/6	1	0	32.4687653	119.5685043	https://www.inaturalist.org/observations/142862838
5580	Bubulcus ibis	Cattle Egret	2020/9/18	1	0	32.6607250	119.5136033	https://www.inaturalist.org/observations/60049290
5581	Bubulcus ibis	Cattle Egret	2022/3/4	3	0	32.7252407	74.8502005	https://www.inaturalist.org/observations/108009360
5582	Bubulcus ibis	Cattle Egret	2021/4/14	3	0	32.7266016	74.8570259	https://www.inaturalist.org/observations/74398319
5583	Bubulcus ibis	Cattle Egret	2021/7/17	1	0	33.0748250	114.1276267	https://www.inaturalist.org/observations/91264523
5584	Bubulcus ibis	Cattle Egret	2016/7/17	4	0	33.6300189	78.3159844	https://www.inaturalist.org/observations/10524036
5585	Bubulcus ibis	Cattle Egret	2016/7/8	2	0	34.0731818	77.6392939	https://www.inaturalist.org/observations/93828543
5586	Bubulcus ibis	Cattle Egret	2022/6/17	1	0	34.3950870	117.4822351	https://www.inaturalist.org/observations/122741067
5587	Bubulcus ibis	Cattle Egret	2021/10/6	1	0	36.1239816	120.5891187	https://www.inaturalist.org/observations/103686875
5588	Bubulcus ibis	Cattle Egret	2022/5/18	1	0	36.2696842	120.4419373	https://www.inaturalist.org/observations/118001874
5589	Bubulcus ibis	Cattle Egret	2021/10/10	2	0	36.3656260	120.6806710	https://www.inaturalist.org/observations/105560123
5590	Bubulcus ibis	Cattle Egret	2020/9/14	2	0	36.3700000	120.6813000	https://www.inaturalist.org/observations/105147745
5591	Bubulcus ibis	Cattle Egret	2021/6/4	1	0	37.6037050	118.7203794	https://www.inaturalist.org/observations/142604552
5592	Bubulcus ibis	Cattle Egret	2021/5/2	1	0	39.0215852	117.1556932	https://www.inaturalist.org/observations/143181072
5593	Bubulcus ibis	Cattle Egret	2017/5/16	2	0	39.8715900	123.8947300	https://www.inaturalist.org/observations/6680710
5594	Bubulcus ibis	Cattle Egret	2017/5/11	2	0	39.8949213	123.8968563	https://www.inaturalist.org/observations/6678764
5595	Bubulcus ibis	Cattle Egret	2022/9/18	1	0	40.1363097	116.3055089	https://www.inaturalist.org/observations/136427804
5596	Bubulcus ibis	Cattle Egret	2022/10/1	1	0	40.1374204	116.3039022	https://www.inaturalist.org/observations/137351092
5597	Bubulcus ibis	Cattle Egret	2021/5/22	1	0	40.1534374	116.6746867	https://www.inaturalist.org/observations/80621989
5598	Bubulcus ibis	Cattle Egret	2021/9/5	1	0	40.1927778	116.6713062	https://www.inaturalist.org/observations/94322632

No	scientific name	common name	date	NIA	NID	latitude	longitude	url
1	Ciconia abdimii	Abdim's Stork	2013/1/24	1	0	-9.0863660	33.9527031	https://www.inaturalist.org/observations/79513326
2	Ciconia abdimii	Abdim's Stork	2011/2/10	3	0	-7.6732877	34.9308096	https://www.inaturalist.org/observations/56763861
3	Ciconia abdimii	Abdim's Stork	2021/2/20	1	0	-4.4989233	35.4490354	https://www.inaturalist.org/observations/71567109
4	Ciconia abdimii	Abdim's Stork	2021/2/5	3	0	-3.6936433	35.9285756	https://www.inaturalist.org/observations/70384504
5	Ciconia abdimii	Abdim's Stork	2019/3/25	1	0	-3.6190593	35.8061779	https://www.inaturalist.org/observations/66984417
6	Ciconia abdimii	Abdim's Stork	2016/2/16	2	0	-3.6190593	35.8061779	https://www.inaturalist.org/observations/137402794
7	Ciconia abdimii	Abdim's Stork	2021/2/20	2	0	-3.6157223	36.0349918	https://www.inaturalist.org/observations/71697327
8	Ciconia abdimii	Abdim's Stork	2019/2/27	3	0	-3.6145917	36.0366550	https://www.inaturalist.org/observations/21113473
9	Ciconia abdimii	Abdim's Stork	2021/4/9	2	0	-3.6139717	36.0376817	https://www.inaturalist.org/observations/80408696
10	Ciconia abdimii	Abdim's Stork	2016/1/24	7	0	-3.4249514	36.3731931	https://www.inaturalist.org/observations/34413215
11	Ciconia abdimii	Abdim's Stork	2015/2/13	4	0	-3.4060236	36.2945433	http://www.inaturalist.org/observations/1231426
12	Ciconia abdimii	Abdim's Stork	2019/1/29	2	0	-3.4045738	36.8825776	https://www.inaturalist.org/observations/117964337
13	Ciconia abdimii	Abdim's Stork	2002/2/4	4	0	-3.3989600	36.2363430	http://www.inaturalist.org/observations/528731
14	Ciconia abdimii	Abdim's Stork	2019/3/7	4	0	-3.3951186	36.8660231	https://www.inaturalist.org/observations/104963750
15	Ciconia abdimii	Abdim's Stork	2019/12/23	0	0	-3.3869254	36.6829927	https://www.inaturalist.org/observations/39699426
16	Ciconia abdimii	Abdim's Stork	2020/1/23	2	0	-3.3869254	36.6829927	https://www.inaturalist.org/observations/39701312
17	Ciconia abdimii	Abdim's Stork	2019/12/23	1	0	-3.3869254	36.6829927	https://www.inaturalist.org/observations/40208740
18	Ciconia abdimii	Abdim's Stork	2018/1/20	2	0	-3.3869254	36.6829927	https://www.inaturalist.org/observations/81063625
19	Ciconia abdimii	Abdim's Stork	2020/1/23	2	0	-3.3861415	36.6809381	https://www.inaturalist.org/observations/39701315
20	Ciconia abdimii	Abdim's Stork	2019/3/8	1	0	-3.3859232	36.8625436	https://www.inaturalist.org/observations/120300528
21	Ciconia abdimii	Abdim's Stork	2018/3/29	3	0	-3.3542634	38.8256903	https://www.inaturalist.org/observations/10735181
22	Ciconia abdimii	Abdim's Stork	2015/2/27	3	0	-3.3462130	38.8220227	https://www.inaturalist.org/observations/106813279
23	Ciconia abdimii	Abdim's Stork	2005/2/13	0	0	-3.2500000	35.5200000	https://www.inaturalist.org/observations/37948498
24	Ciconia abdimii	Abdim's Stork	2009/2/24	4	0	-3.2415440	35.4877780	http://www.inaturalist.org/observations/1522926

25	Ciconia abdimii	Abdim's Stork	2022/1/15	3	0	-3.2292383	35.5585617	https://www.inaturalist.org/observations/105570043
26	Ciconia abdimii	Abdim's Stork	2016/3/28	6	0	-3.2224833	35.5576783	http://conabio.inaturalist.org/observations/2843022
27	Ciconia abdimii	Abdim's Stork	2003/1/25	4	0	-3.2137424	35.5582429	https://www.inaturalist.org/observations/40890482
28	Ciconia abdimii	Abdim's Stork	2022/1/15	3	0	-3.2083133	35.5808283	https://www.inaturalist.org/observations/105569943
29	Ciconia abdimii	Abdim's Stork	2019/2/23	4	0	-3.1995829	35.6479178	https://www.inaturalist.org/observations/142916118
30	Ciconia abdimii	Abdim's Stork	2017/1/30	4	0	-3.1992630	35.5879130	https://www.inaturalist.org/observations/5935558
31	Ciconia abdimii	Abdim's Stork	2019/12/31	3	0	-3.1956379	35.5650104	https://www.inaturalist.org/observations/120935906
32	Ciconia abdimii	Abdim's Stork	2020/12/24	3	0	-3.1897682	35.5060183	https://www.inaturalist.org/observations/82192709
33	Ciconia abdimii	Abdim's Stork	2018/1/25	3	0	-3.1884899	35.5753099	https://www.inaturalist.org/observations/16814038
34	Ciconia abdimii	Abdim's Stork	2018/2/6	2	0	-3.1871191	35.6171955	https://www.inaturalist.org/observations/142070130
35	Ciconia abdimii	Abdim's Stork	2015/1/1	2	0	-3.1836908	35.6213151	https://www.inaturalist.org/observations/21381386
36	Ciconia abdimii	Abdim's Stork	2020/12/23	2	0	-3.1806918	35.5579722	https://www.inaturalist.org/observations/67786560
37	Ciconia abdimii	Abdim's Stork	2010/12/27	4	0	-3.1802810	35.5757904	https://www.inaturalist.org/observations/7131275
38	Ciconia abdimii	Abdim's Stork	2020/2/12	2	0	-3.1795780	35.5670703	https://www.inaturalist.org/observations/49695890
39	Ciconia abdimii	Abdim's Stork	2015/2/16	4	0	-3.1793370	35.6108530	https://www.inaturalist.org/observations/34840787
40	Ciconia abdimii	Abdim's Stork	2019/2/26	2	0	-3.1789333	35.5686200	https://www.inaturalist.org/observations/21108264
41	Ciconia abdimii	Abdim's Stork	2017/2/19	4	0	-3.1782060	35.5684440	https://www.inaturalist.org/observations/6098418
42	Ciconia abdimii	Abdim's Stork	2017/2/19	4	0	-3.1782060	35.5711900	https://www.inaturalist.org/observations/6137034
43	Ciconia abdimii	Abdim's Stork	2007/1/26	2	0	-3.1781359	35.5659136	https://www.inaturalist.org/observations/143846616
44	Ciconia abdimii	Abdim's Stork	2007/1/27	3	0	-3.1768595	35.5750216	https://www.inaturalist.org/observations/143836050
45	Ciconia abdimii	Abdim's Stork	2007/1/27	3	0	-3.1768595	35.5750216	https://www.inaturalist.org/observations/143836065
46	Ciconia abdimii	Abdim's Stork	2019/3/2	2	0	-3.1768222	35.5121611	https://www.inaturalist.org/observations/80211739
47	Ciconia abdimii	Abdim's Stork	2019/2/26	2	0	-3.1768100	35.5618517	https://www.inaturalist.org/observations/21108260
48	Ciconia abdimii	Abdim's Stork	2016/2/22	6	0	-3.1764100	35.5898684	https://www.inaturalist.org/observations/13077839
49	Ciconia abdimii	Abdim's Stork	2022/1/4	1	0	-3.1747725	35.5260985	https://www.inaturalist.org/observations/116446990

50	Ciconia abdimii	Abdim's Stork	2012/4/15	2	0	-3.1743475	35.5645975	https://www.inaturalist.org/observations/43851122
51	Ciconia abdimii	Abdim's Stork	2017/2/28	5	0	-3.1721773	35.5718078	https://www.inaturalist.org/observations/9524531
52	Ciconia abdimii	Abdim's Stork	2011/3/8	1	0	-3.1666667	35.5833333	https://www.inaturalist.org/observations/102079947
53	Ciconia abdimii	Abdim's Stork	2014/3/29	2	0	-3.1646992	35.6034402	https://www.inaturalist.org/observations/64893593
54	Ciconia abdimii	Abdim's Stork	2019/2/26	3	0	-3.1633900	35.5000800	https://www.inaturalist.org/observations/21106417
55	Ciconia abdimii	Abdim's Stork	2009/2/14	2	0	-3.1617522	35.5876697	https://www.inaturalist.org/observations/59800242
56	Ciconia abdimii	Abdim's Stork	2018/1/23	2	0	-3.1617522	35.5876697	https://www.inaturalist.org/observations/81636907
57	Ciconia abdimii	Abdim's Stork	2018/1/23	2	0	-3.1617522	35.5876697	https://www.inaturalist.org/observations/81695576
58	Ciconia abdimii	Abdim's Stork	2011/2/20	5	0	-3.1617522	35.5876697	https://www.inaturalist.org/observations/108305456
59	Ciconia abdimii	Abdim's Stork	2011/2/20	4	0	-3.1617522	35.5876697	https://www.inaturalist.org/observations/108367433
60	Ciconia abdimii	Abdim's Stork	2022/2/23	4	0	-3.1617522	35.5876697	https://www.inaturalist.org/observations/108427170
61	Ciconia abdimii	Abdim's Stork	2007/2/10	2	0	-3.1617522	35.5876697	https://www.inaturalist.org/observations/142357883
62	Ciconia abdimii	Abdim's Stork	2019/2/26	3	0	-3.1606200	35.5693250	https://www.inaturalist.org/observations/21107545
63	Ciconia abdimii	Abdim's Stork	2019/2/26	2	0	-3.1579050	35.5771650	https://www.inaturalist.org/observations/21107539
64	Ciconia abdimii	Abdim's Stork	2022/1/15	2	0	-3.1575388	35.5287467	https://www.inaturalist.org/observations/105567810
65	Ciconia abdimii	Abdim's Stork	2020/2/14	2	0	-3.1558038	35.5956790	https://www.inaturalist.org/observations/134512520
66	Ciconia abdimii	Abdim's Stork	2019/2/26	3	0	-3.1509750	35.5027650	https://www.inaturalist.org/observations/21106422
67	Ciconia abdimii	Abdim's Stork	2022/1/15	2	0	-3.1504467	35.5981033	https://www.inaturalist.org/observations/105568814
68	Ciconia abdimii	Abdim's Stork	2019/2/26	3	0	-3.1499100	35.5824733	https://www.inaturalist.org/observations/21107530
69	Ciconia abdimii	Abdim's Stork	2014/3/9	3	0	-3.1483140	35.5400840	https://www.inaturalist.org/observations/5380622
70	Ciconia abdimii	Abdim's Stork	2016/2/6	5	0	-3.1397230	35.5879250	http://www.inaturalist.org/observations/2785855
71	Ciconia abdimii	Abdim's Stork	2013/3/6	1	0	-3.1367088	35.6052753	https://www.inaturalist.org/observations/98872344
72	Ciconia abdimii	Abdim's Stork	2019/2/23	4	0	-3.1274995	35.5838264	https://www.inaturalist.org/observations/142916130
73	Ciconia abdimii	Abdim's Stork	2019/3/4	3	0	-3.1244554	36.6970852	https://www.inaturalist.org/observations/106722012
74	Ciconia abdimii	Abdim's Stork	2013/1/8	4	0	-3.1219672	35.5864398	https://www.inaturalist.org/observations/142611991

75	Ciconia abdimii	Abdim's Stork	2016/1/10	5	0	-3.0199429	34.9969000	https://www.inaturalist.org/observations/9867211
76	Ciconia abdimii	Abdim's Stork	2020/2/12	2	0	-2.9908754	34.9937318	https://www.inaturalist.org/observations/134466262
77	Ciconia abdimii	Abdim's Stork	2020/2/24	3	0	-2.9890143	35.3534836	https://www.inaturalist.org/observations/70439839
78	Ciconia abdimii	Abdim's Stork	2018/1/21	2	0	-2.9388654	36.3136140	https://www.inaturalist.org/observations/81234989
79	Ciconia abdimii	Abdim's Stork	2020/2/10	2	0	-2.8580960	34.9990756	https://www.inaturalist.org/observations/108501996
80	Ciconia abdimii	Abdim's Stork	2020/2/7	2	0	-2.8459512	35.0239097	https://www.inaturalist.org/observations/40899867
81	Ciconia abdimii	Abdim's Stork	2020/12/27	3	0	-2.6943221	38.1673059	https://www.inaturalist.org/observations/67448052
82	Ciconia abdimii	Abdim's Stork	2010/12/16	5	0	-2.6532816	37.2607672	https://www.inaturalist.org/observations/34852473
83	Ciconia abdimii	Abdim's Stork	2007/1/12	2	0	-2.6526870	37.2605650	https://www.inaturalist.org/observations/49132767
84	Ciconia abdimii	Abdim's Stork	2018/4/7	2	0	-2.4927410	34.8287696	https://www.inaturalist.org/observations/107803453
85	Ciconia abdimii	Abdim's Stork	2001/2/14	3	0	-2.4361300	34.8205900	https://www.inaturalist.org/observations/139838633
86	Ciconia abdimii	Abdim's Stork	2019/12/25	2	0	-2.3333333	34.8333333	https://www.inaturalist.org/observations/39701317
87	Ciconia abdimii	Abdim's Stork	2019/12/25	2	0	-2.3284604	34.8266602	https://www.inaturalist.org/observations/39701318
88	Ciconia abdimii	Abdim's Stork	2019/12/25	2	0	-2.3284604	34.8266602	https://www.inaturalist.org/observations/39701319
89	Ciconia abdimii	Abdim's Stork	2019/12/25	2	0	-2.3284604	34.8266602	https://www.inaturalist.org/observations/39701320
90	Ciconia abdimii	Abdim's Stork	2019/12/25	2	0	-2.3284604	34.8266602	https://www.inaturalist.org/observations/39701321
91	Ciconia abdimii	Abdim's Stork	2021/12/31	1	0	-2.2495147	38.3376639	https://www.inaturalist.org/observations/104638388
92	Ciconia abdimii	Abdim's Stork	2011/11/22	3	0	-1.8936000	34.6856510	https://www.inaturalist.org/observations/142380171
93	Ciconia abdimii	Abdim's Stork	2022/11/19	4	0	-1.6017180	34.8404410	https://www.inaturalist.org/observations/144456033
94	Ciconia abdimii	Abdim's Stork	2019/5/22	2	0	-1.6016957	35.2140610	https://www.inaturalist.org/observations/25572349
95	Ciconia abdimii	Abdim's Stork	2022/3/26	4	0	-1.5269045	35.2034632	https://www.inaturalist.org/observations/110847400
96	Ciconia abdimii	Abdim's Stork	2016/11/24	3	0	-1.4239980	35.0747570	https://www.inaturalist.org/observations/8329858
97	Ciconia abdimii	Abdim's Stork	2022/3/21	2	0	-1.1349610	35.2635520	https://www.inaturalist.org/observations/109622630
98	Ciconia abdimii	Abdim's Stork	2022/12/22	2	0	-1.0726778	35.2282917	https://www.inaturalist.org/observations/144834451
99	Ciconia abdimii	Abdim's Stork	2022/12/22	2	0	-1.0723972	35.2273550	https://www.inaturalist.org/observations/144835669

100	Ciconia abdimii	Abdim's Stork	2022/12/22	2	0	-1.0718917	35.2276533	https://www.inaturalist.org/observations/144841903
101	Ciconia abdimii	Abdim's Stork	2022/12/22	3	0	-1.0718361	35.2276642	https://www.inaturalist.org/observations/144837836
102	Ciconia abdimii	Abdim's Stork	2022/12/22	3	0	-1.0694842	35.2272571	https://www.inaturalist.org/observations/144841980
103	Ciconia abdimii	Abdim's Stork	2022/11/3	2	0	-0.6350750	30.5296950	https://www.inaturalist.org/observations/143788011
104	Ciconia abdimii	Abdim's Stork	2022/11/3	2	0	-0.6350750	30.5296950	https://www.inaturalist.org/observations/143788012
105	Ciconia abdimii	Abdim's Stork	2022/11/3	1	0	-0.6349883	30.5300833	https://www.inaturalist.org/observations/143788015
106	Ciconia abdimii	Abdim's Stork	2022/11/3	2	0	-0.6349883	30.5300833	https://www.inaturalist.org/observations/143788018
107	Ciconia abdimii	Abdim's Stork	2022/4/18	3	0	-0.4227050	36.1295612	https://www.inaturalist.org/observations/111896988
108	Ciconia abdimii	Abdim's Stork	2017/5/28	3	0	-0.4148460	36.1192335	https://www.inaturalist.org/observations/106960496
109	Ciconia abdimii	Abdim's Stork	2022/10/28	3	0	-0.2001500	30.0880250	https://www.inaturalist.org/observations/143256643
110	Ciconia abdimii	Abdim's Stork	2022/11/22	2	0	-0.1337649	29.8806415	https://www.inaturalist.org/observations/145240154
111	Ciconia abdimii	Abdim's Stork	2020/2/23	3	0	-0.1273833	34.7461467	https://www.inaturalist.org/observations/39100607
112	Ciconia abdimii	Abdim's Stork	2022/11/8	2	0	0.0533796	32.4757864	https://www.inaturalist.org/observations/141688777
113	Ciconia abdimii	Abdim's Stork	2021/2/28	1	0	0.1014194	37.3818361	https://www.inaturalist.org/observations/104511897
114	Ciconia abdimii	Abdim's Stork	2017/3/4	5	0	0.1101470	37.4341180	https://www.inaturalist.org/observations/34314755
115	Ciconia abdimii	Abdim's Stork	2015/12/4	5	0	0.1345660	37.0330350	http://www.inaturalist.org/observations/2449929
116	Ciconia abdimii	Abdim's Stork	2020/11/18	1	0	0.2194528	32.5506839	https://www.inaturalist.org/observations/65333669
117	Ciconia abdimii	Abdim's Stork	2015/4/13	4	0	0.6320551	36.0567202	https://www.inaturalist.org/observations/36344307
118	Ciconia abdimii	Abdim's Stork	2008/10/27	2	0	0.8968830	30.3666099	https://www.inaturalist.org/observations/6480519
119	Ciconia abdimii	Abdim's Stork	2015/3/22	2	0	1.4481100	32.0790480	http://www.inaturalist.org/observations/1422394
120	Ciconia abdimii	Abdim's Stork	2019/3/11	2	0	1.5706617	34.6096954	https://www.inaturalist.org/observations/21777664
121	Ciconia abdimii	Abdim's Stork	2019/3/16	1	0	2.1118125	31.7204817	https://www.inaturalist.org/observations/37478193
122	Ciconia abdimii	Abdim's Stork	2010/2/6	5	0	2.1457680	31.8069054	https://www.inaturalist.org/observations/35997283
123	Ciconia abdimii	Abdim's Stork	2016/2/4	1	0	2.1457680	31.8069054	https://www.inaturalist.org/observations/48400861
124	Ciconia abdimii	Abdim's Stork	2015/3/14	3	0	2.1802592	31.8246382	https://www.inaturalist.org/observations/44695164

125	Ciconia abdimii	Abdim's Stork	2015/3/14	3	0	2.1802592	31.8246382	https://www.inaturalist.org/observations/44695165
126	Ciconia abdimii	Abdim's Stork	2014/3/4	3	0	2.2566618	31.6464412	https://www.inaturalist.org/observations/127478710
127	Ciconia abdimii	Abdim's Stork	2014/3/4	3	0	2.2566618	31.6464412	https://www.inaturalist.org/observations/127479220
128	Ciconia abdimii	Abdim's Stork	2014/3/3	3	0	2.2568501	31.6472367	https://www.inaturalist.org/observations/127476648
129	Ciconia abdimii	Abdim's Stork	2017/2/6	3	0	2.2646565	31.4594793	https://www.inaturalist.org/observations/68337424
130	Ciconia abdimii	Abdim's Stork	2019/3/17	2	0	2.2722716	31.6290075	https://www.inaturalist.org/observations/37478194
131	Ciconia abdimii	Abdim's Stork	2014/2/6	1	0	2.2749810	31.6733120	https://www.inaturalist.org/observations/38220490
132	Ciconia abdimii	Abdim's Stork	2016/2/17	3	0	2.2829800	31.5649210	https://www.inaturalist.org/observations/34527953
133	Ciconia abdimii	Abdim's Stork	2015/3/23	4	0	2.3152690	31.4326660	http://www.inaturalist.org/observations/1434489
134	Ciconia abdimii	Abdim's Stork	2016/2/17	4	0	2.3245830	31.4758580	https://www.inaturalist.org/observations/34530495
135	Ciconia abdimii	Abdim's Stork	2020/3/21	1	0	2.3623605	31.6486481	https://www.inaturalist.org/observations/40363618
136	Ciconia abdimii	Abdim's Stork	2015/12/10	4	0	2.3837630	35.6478190	http://www.inaturalist.org/observations/2896370
137	Ciconia abdimii	Abdim's Stork	2016/2/13	5	0	2.3940972	31.5077639	http://www.inaturalist.org/observations/2829227
138	Ciconia abdimii	Abdim's Stork	2020/3/9	3	0	2.3949738	31.5020599	https://www.inaturalist.org/observations/70506257
139	Ciconia abdimii	Abdim's Stork	2017/2/4	4	0	2.3968084	31.5524408	https://www.inaturalist.org/observations/107322093
140	Ciconia abdimii	Abdim's Stork	2014/3/4	3	0	2.4797333	31.5433517	https://www.inaturalist.org/observations/127479333
141	Ciconia abdimii	Abdim's Stork	2014/3/4	3	0	2.4797333	31.5433517	https://www.inaturalist.org/observations/127479355
142	Ciconia abdimii	Abdim's Stork	2020/3/21	1	0	3.4125083	33.4764533	https://www.inaturalist.org/observations/40348157
143	Ciconia abdimii	Abdim's Stork	2020/3/21	1	0	3.4549972	33.5148934	https://www.inaturalist.org/observations/40347622
144	Ciconia abdimii	Abdim's Stork	2020/3/20	1	0	3.7154749	33.6734490	https://www.inaturalist.org/observations/40279251
145	Ciconia abdimii	Abdim's Stork	2022/11/14	3	0	3.7357391	33.6978290	https://www.inaturalist.org/observations/144324472
146	Ciconia abdimii	Abdim's Stork	2019/3/23	1	0	5.9122114	37.5000231	https://www.inaturalist.org/observations/47403085
147	Ciconia abdimii	Abdim's Stork	2015/4/26	6	0	6.0362383	37.5898239	http://www.inaturalist.org/observations/2458659
148	Ciconia abdimii	Abdim's Stork	2022/4/29	3	0	6.2936819	37.7659368	https://www.inaturalist.org/observations/113535617
149	Ciconia abdimii	Abdim's Stork	2011/1/13	6	0	6.7442590	39.6784970	https://www.inaturalist.org/observations/9777133

150	Ciconia abdimii	Abdim's Stork	2014/1/29	1	0	6.9902290	38.4964730	https://www.inaturalist.org/observations/37371675
151	Ciconia abdimii	Abdim's Stork	2020/3/6	2	0	7.0068049	39.0592387	https://www.inaturalist.org/observations/82396450
152	Ciconia abdimii	Abdim's Stork	2022/4/5	2	0	7.0565319	38.4962654	https://www.inaturalist.org/observations/110477663
153	Ciconia abdimii	Abdim's Stork	2019/3/18	2	0	8.0189136	38.7232903	https://www.inaturalist.org/observations/21671383
154	Ciconia abdimii	Abdim's Stork	2014/4/20	1	0	8.2302215	38.8022698	https://www.inaturalist.org/observations/58449643
155	Ciconia abdimii	Abdim's Stork	2014/1/23	2	0	8.9066289	39.9815855	https://www.inaturalist.org/observations/141702189
156	Ciconia abdimii	Abdim's Stork	2014/1/18	1	0	9.3320580	40.2934520	https://www.inaturalist.org/observations/37268073
157	Ciconia abdimii	Abdim's Stork	2021/5/2	2	0	10.6854238	34.4361550	https://www.inaturalist.org/observations/76543807
158	Ciconia abdimii	Abdim's Stork	2020/2/17	3	0	11.4901771	37.5833883	https://www.inaturalist.org/observations/39378411
159	Ciconia abdimii	Abdim's Stork	2021/6/28	1	0	11.5742086	37.3613533	https://www.inaturalist.org/observations/103813860
160	Ciconia abdimii	Abdim's Stork	2022/3/15	3	0	11.5742086	37.3613533	https://www.inaturalist.org/observations/108644187
161	Ciconia abdimii	Abdim's Stork	2022/7/10	3	0	11.5742086	37.3613533	https://www.inaturalist.org/observations/125585867
162	Ciconia abdimii	Abdim's Stork	2019/12/3	2	0	12.9605384	36.1701223	https://www.inaturalist.org/observations/98508580

No	scientific name	common name	date	NIA	NID	latitude	longitude	url
1	Plectropterus gambensis	Spur-winged Goose	2004/11/13	1	0	-7.7956150	36.6941690	https://www.inaturalist.org/observations/58236463
2	Plectropterus gambensis	Spur-winged Goose	2017/10/21	5	0	-7.7661700	38.2326867	https://www.inaturalist.org/observations/8663737
3	Plectropterus gambensis	Spur-winged Goose	2017/7/25	4	0	-7.7659100	38.2324950	https://www.inaturalist.org/observations/7248443
4	Plectropterus gambensis	Spur-winged Goose	2012/12/10	4	0	-7.3203731	34.9300932	https://www.inaturalist.org/observations/24376717
5	Plectropterus gambensis	Spur-winged Goose	2021/9/28	2	0	-6.8981556	31.1865139	https://www.inaturalist.org/observations/97643246
6	Plectropterus gambensis	Spur-winged Goose	2011/11/7	1	0	-6.8980932	31.1658998	https://www.inaturalist.org/observations/97748593
7	Plectropterus gambensis	Spur-winged Goose	2011/11/9	1	0	-6.8257239	31.1279349	https://www.inaturalist.org/observations/67104251
8	Plectropterus gambensis	Spur-winged Goose	1980/1/1	1	0	-5.7661159	31.9102672	https://www.inaturalist.org/observations/70935185
9	Plectropterus gambensis	Spur-winged Goose	2021/2/15	2	0	-5.0944000	30.8227333	https://www.inaturalist.org/observations/70273378
10	Plectropterus gambensis	Spur-winged Goose	2021/2/17	1	0	-5.0759550	30.8899356	https://www.inaturalist.org/observations/70984438
11	Plectropterus gambensis	Spur-winged Goose	2021/2/17	1	0	-5.0758721	30.8899797	https://www.inaturalist.org/observations/70984436
12	Plectropterus gambensis	Spur-winged Goose	2021/2/15	1	0	-5.0541819	30.9550488	https://www.inaturalist.org/observations/70185125
13	Plectropterus gambensis	Spur-winged Goose	2021/2/16	1	0	-5.0425588	30.9298460	https://www.inaturalist.org/observations/70432823
14	Plectropterus gambensis	Spur-winged Goose	2021/2/19	1	0	-5.0068566	31.4153008	https://www.inaturalist.org/observations/70593975
15	Plectropterus gambensis	Spur-winged Goose	2017/7/18	4	0	-4.1656368	38.1665039	https://www.inaturalist.org/observations/40803898
16	Plectropterus gambensis	Spur-winged Goose	2022/7/19	1	0	-4.0880050	36.1368783	https://www.inaturalist.org/observations/128476157
17	Plectropterus gambensis	Spur-winged Goose	2017/9/23	4	0	-4.0677100	36.1203833	https://www.inaturalist.org/observations/31317591
18	Plectropterus gambensis	Spur-winged Goose	2007/10/19	3	0	-4.0523092	36.1216489	https://www.inaturalist.org/observations/19703510
19	Plectropterus gambensis	Spur-winged Goose	2018/8/31	4	0	-4.0503233	36.1207333	https://www.inaturalist.org/observations/20145305
20	Plectropterus gambensis	Spur-winged Goose	2017/2/2	3	0	-4.0498910	36.1221880	https://www.inaturalist.org/observations/27028604
21	Plectropterus gambensis	Spur-winged Goose	2018/10/19	3	0	-4.0234467	36.1211267	https://www.inaturalist.org/observations/18013474
22	Plectropterus gambensis	Spur-winged Goose	2016/9/4	5	0	-4.0218281	36.1218998	https://www.inaturalist.org/observations/39995383

23	Plectropterus gambensis	Spur-winged Goose	2021/10/30	2	0	-4.0056666	35.9787556	https://www.inaturalist.org/observations/101641484
24	Plectropterus gambensis	Spur-winged Goose	2022/11/13	1	0	-4.0056291	35.9787341	https://www.inaturalist.org/observations/144050197
25	Plectropterus gambensis	Spur-winged Goose	2021/9/12	1	0	-3.9892191	37.7672892	https://www.inaturalist.org/observations/95052405
26	Plectropterus gambensis	Spur-winged Goose	2022/12/16	1	0	-3.9831380	38.0431108	https://www.inaturalist.org/observations/145053691
27	Plectropterus gambensis	Spur-winged Goose	2015/10/4	1	0	-3.9506743	35.8661255	https://www.inaturalist.org/observations/75087459
28	Plectropterus gambensis	Spur-winged Goose	2019/1/18	3	0	-3.9057791	37.8629110	https://www.inaturalist.org/observations/49699285
29	Plectropterus gambensis	Spur-winged Goose	2007/10/19	3	0	-3.8293150	35.9815980	http://www.inaturalist.org/observations/18363
30	Plectropterus gambensis	Spur-winged Goose	2019/10/20	4	0	-3.7744500	35.9734000	https://www.inaturalist.org/observations/35938994
31	Plectropterus gambensis	Spur-winged Goose	2021/12/5	2	0	-3.7711283	35.9704767	https://www.inaturalist.org/observations/104806115
32	Plectropterus gambensis	Spur-winged Goose	2022/1/25	1	0	-3.7694230	35.9687790	https://www.inaturalist.org/observations/106903145
33	Plectropterus gambensis	Spur-winged Goose	2019/10/26	2	0	-3.6993190	36.9025825	https://www.inaturalist.org/observations/145176203
34	Plectropterus gambensis	Spur-winged Goose	2022/4/16	1	0	-3.6235004	37.7829847	https://www.inaturalist.org/observations/112059344
35	Plectropterus gambensis	Spur-winged Goose	2019/9/11	3	0	-3.6190593	35.8061779	https://www.inaturalist.org/observations/39509438
36	Plectropterus gambensis	Spur-winged Goose	2019/11/18	3	0	-3.6147017	38.2255450	https://www.inaturalist.org/observations/37411610
37	Plectropterus gambensis	Spur-winged Goose	2019/11/17	2	0	-3.5308333	38.1697389	https://www.inaturalist.org/observations/37411587
38	Plectropterus gambensis	Spur-winged Goose	2019/11/17	3	0	-3.5308278	38.1697472	https://www.inaturalist.org/observations/37411589
39	Plectropterus gambensis	Spur-winged Goose	2022/4/10	1	0	-3.5210218	38.2621545	https://www.inaturalist.org/observations/112854409
40	Plectropterus gambensis	Spur-winged Goose	2022/4/10	1	0	-3.5199520	38.2431860	https://www.inaturalist.org/observations/112854384
41	Plectropterus gambensis	Spur-winged Goose	2019/12/23	3	0	-3.4497456	35.8116243	https://www.inaturalist.org/observations/38713450
42	Plectropterus gambensis	Spur-winged Goose	2019/8/17	5	0	-3.4317957	35.8001111	https://www.inaturalist.org/observations/33436722
43	Plectropterus gambensis	Spur-winged Goose	2010/10/27	2	0	-3.4156303	38.6505499	https://www.inaturalist.org/observations/132034600
44	Plectropterus gambensis	Spur-winged Goose	2018/1/24	2	0	-3.4125589	35.8383455	https://www.inaturalist.org/observations/81896260
45	Plectropterus gambensis	Spur-winged Goose	2011/3/6	2	0	-3.4117411	35.8364167	https://www.inaturalist.org/observations/106711631

46	Plectropterus gambensis	Spur-winged Goose	2022/1/10	2	0	-3.4085300	35.8289800	https://www.inaturalist.org/observations/104719054
47	Plectropterus gambensis	Spur-winged Goose	2015/5/14	2	0	-3.3588039	36.8372193	https://www.inaturalist.org/observations/69748671
48	Plectropterus gambensis	Spur-winged Goose	2020/6/14	1	0	-3.3436925	36.9596761	https://www.inaturalist.org/observations/104649537
49	Plectropterus gambensis	Spur-winged Goose	2021/7/31	1	0	-3.2934304	36.8782651	https://www.inaturalist.org/observations/94641410
50	Plectropterus gambensis	Spur-winged Goose	2021/11/2	1	0	-3.2884430	36.8374290	https://www.inaturalist.org/observations/102929405
51	Plectropterus gambensis	Spur-winged Goose	2019/1/12	1	0	-3.2676599	36.8955189	https://www.inaturalist.org/observations/116047811
52	Plectropterus gambensis	Spur-winged Goose	2014/9/6	4	0	-3.2529510	36.7925260	http://www.inaturalist.org/observations/1048330
53	Plectropterus gambensis	Spur-winged Goose	2015/12/31	4	0	-3.2316798	35.5430374	https://www.inaturalist.org/observations/18817813
54	Plectropterus gambensis	Spur-winged Goose	2019/6/14	1	0	-3.2279236	35.5291148	https://www.inaturalist.org/observations/128218820
55	Plectropterus gambensis	Spur-winged Goose	2015/12/31	3	0	-3.2124841	35.5799443	https://www.inaturalist.org/observations/21380994
56	Plectropterus gambensis	Spur-winged Goose	2018/1/25	3	0	-3.2123130	35.6024319	https://www.inaturalist.org/observations/16814035
57	Plectropterus gambensis	Spur-winged Goose	2018/4/19	4	0	-3.2054933	35.5845267	https://www.inaturalist.org/observations/14282026
58	Plectropterus gambensis	Spur-winged Goose	2018/4/19	3	0	-3.2037883	35.5882367	https://www.inaturalist.org/observations/14282008
59	Plectropterus gambensis	Spur-winged Goose	1993/12/27	2	0	-3.1884903	35.5725635	https://www.inaturalist.org/observations/135823395
60	Plectropterus gambensis	Spur-winged Goose	2020/9/22	1	0	-3.1809490	35.5560838	https://www.inaturalist.org/observations/62803034
61	Plectropterus gambensis	Spur-winged Goose	2020/12/23	1	0	-3.1806918	35.5579722	https://www.inaturalist.org/observations/67786541
62	Plectropterus gambensis	Spur-winged Goose	2017/1/13	2	0	-3.1802695	35.6946503	https://www.inaturalist.org/observations/82927519
63	Plectropterus gambensis	Spur-winged Goose	2021/11/8	1	0	-3.1795778	35.5622629	https://www.inaturalist.org/observations/102447759
64	Plectropterus gambensis	Spur-winged Goose	2021/7/20	1	0	-3.1788418	35.5577688	https://www.inaturalist.org/observations/88528539
65	Plectropterus gambensis	Spur-winged Goose	2019/4/27	1	0	-3.1783838	35.5624741	https://www.inaturalist.org/observations/126815159
66	Plectropterus gambensis	Spur-winged Goose	2022/8/1	3	0	-3.1768207	35.5472944	https://www.inaturalist.org/observations/129435264
67	Plectropterus gambensis	Spur-winged Goose	2021/12/8	1	0	-3.1722450	35.5241717	https://www.inaturalist.org/observations/104864650
68	Plectropterus gambensis	Spur-winged Goose	2022/1/15	1	0	-3.1720617	35.5459450	https://www.inaturalist.org/observations/105574505

69	Plectropterus gambensis	Spur-winged Goose	2016/6/17	1	0	-3.1716816	35.5419229	https://www.inaturalist.org/observations/141322768
70	Plectropterus gambensis	Spur-winged Goose	2022/1/15	1	0	-3.1699333	35.5344500	https://www.inaturalist.org/observations/105618124
71	Plectropterus gambensis	Spur-winged Goose	2022/1/15	2	0	-3.1698000	35.5343200	https://www.inaturalist.org/observations/105618332
72	Plectropterus gambensis	Spur-winged Goose	2021/10/16	2	0	-3.1697470	35.5337070	https://www.inaturalist.org/observations/100184059
73	Plectropterus gambensis	Spur-winged Goose	1999/2/26	5	0	-3.1617522	35.5876697	https://www.inaturalist.org/observations/8707754
74	Plectropterus gambensis	Spur-winged Goose	2015/2/19	3	0	-3.1617522	35.5876697	https://www.inaturalist.org/observations/23371968
75	Plectropterus gambensis	Spur-winged Goose	2012/12/1	5	0	-3.1617522	35.5876697	https://www.inaturalist.org/observations/27633217
76	Plectropterus gambensis	Spur-winged Goose	2011/1/1	1	0	-3.1617522	35.5876697	https://www.inaturalist.org/observations/54519362
77	Plectropterus gambensis gambensis	Spur-winged Goose	2009/2/14	1	0	-3.1617522	35.5876697	https://www.inaturalist.org/observations/59799980
78	Plectropterus gambensis	Spur-winged Goose	2022/2/23	2	0	-3.1617522	35.5876697	https://www.inaturalist.org/observations/108427570
79	Plectropterus gambensis	Spur-winged Goose	2022/1/15	1	0	-3.1601638	35.5567117	https://www.inaturalist.org/observations/105571272
80	Plectropterus gambensis	Spur-winged Goose	2022/1/4	1	0	-3.1600322	35.5329649	https://www.inaturalist.org/observations/116446993
81	Plectropterus gambensis	Spur-winged Goose	2019/2/21	1	0	-3.1430551	35.5605355	https://www.inaturalist.org/observations/142700552
82	Plectropterus gambensis	Spur-winged Goose	2017/2/26	3	0	-3.0908621	35.3492959	https://www.inaturalist.org/observations/9517711
83	Plectropterus gambensis	Spur-winged Goose	2022/1/12	1	0	-2.6714614	37.2377641	https://www.inaturalist.org/observations/112727554
84	Plectropterus gambensis	Spur-winged Goose	2017/7/9	1	0	-2.6662336	37.3039095	https://www.inaturalist.org/observations/77938096
85	Plectropterus gambensis	Spur-winged Goose	2008/7/15	1	0	-2.6605841	37.2886007	https://www.inaturalist.org/observations/103964273
86	Plectropterus gambensis	Spur-winged Goose	2022/1/11	1	0	-2.6528566	37.2545370	https://www.inaturalist.org/observations/112727452
87	Plectropterus gambensis	Spur-winged Goose	2017/7/25	1	0	-2.6526870	37.2605650	https://www.inaturalist.org/observations/62024095
88	Plectropterus gambensis	Spur-winged Goose	2022/7/27	2	0	-2.6526870	37.2605650	https://www.inaturalist.org/observations/137402085
89	Plectropterus gambensis	Spur-winged Goose	2022/10/5	2	0	-2.6526705	37.2606207	https://www.inaturalist.org/observations/144751142
90	Plectropterus gambensis	Spur-winged Goose	2022/10/3	1	0	-2.6523230	37.2746134	https://www.inaturalist.org/observations/140056880
91	Plectropterus gambensis	Spur-winged Goose	2021/6/19	2	0	-2.6519967	37.2748950	https://www.inaturalist.org/observations/83918772

92	Plectropterus gambensis	Spur-winged Goose	2018/8/31	3	0	-2.6501483	37.2817034	https://www.inaturalist.org/observations/19324426
93	Plectropterus gambensis	Spur-winged Goose	2021/8/25	1	0	-2.6495839	37.2771959	https://www.inaturalist.org/observations/107424146
94	Plectropterus gambensis	Spur-winged Goose	2018/8/31	3	0	-2.6483864	37.2787999	https://www.inaturalist.org/observations/19324428
95	Plectropterus gambensis	Spur-winged Goose	2022/10/3	1	0	-2.6482810	37.2827970	https://www.inaturalist.org/observations/139999177
96	Plectropterus gambensis	Spur-winged Goose	2017/5/21	5	0	-2.6478966	37.2805530	https://www.inaturalist.org/observations/6828237
97	Plectropterus gambensis	Spur-winged Goose	2018/9/1	3	0	-2.6394583	37.2283200	https://www.inaturalist.org/observations/18601103
98	Plectropterus gambensis	Spur-winged Goose	2021/3/18	1	0	-2.6349101	37.2592888	https://www.inaturalist.org/observations/98268743
99	Plectropterus gambensis	Spur-winged Goose	2013/4/21	5	0	-1.4769805	35.0927769	https://www.inaturalist.org/observations/43172175
100	Plectropterus gambensis	Spur-winged Goose	2019/5/22	4	0	-1.4478450	34.9621697	https://www.inaturalist.org/observations/25574503
101	Plectropterus gambensis	Spur-winged Goose	2021/8/30	2	0	-1.4478217	34.9621878	https://www.inaturalist.org/observations/105866474
102	Plectropterus gambensis	Spur-winged Goose	2022/12/22	1	0	-1.0695164	35.2271643	https://www.inaturalist.org/observations/144834410
103	Plectropterus gambensis	Spur-winged Goose	2013/3/19	1	0	-0.6747625	30.9245216	https://www.inaturalist.org/observations/123268569
104	Plectropterus gambensis	Spur-winged Goose	2014/1/5	1	0	0.0684377	32.4329086	https://www.inaturalist.org/observations/122624752
105	Plectropterus gambensis	Spur-winged Goose	2021/7/18	1	0	0.0716261	32.3503480	https://www.inaturalist.org/observations/96537161
106	Plectropterus gambensis	Spur-winged Goose	2022/2/3	2	0	0.0718994	32.3678398	https://www.inaturalist.org/observations/106969831
107	Plectropterus gambensis	Spur-winged Goose	2022/12/18	2	0	0.0833332	32.3333333	https://www.inaturalist.org/observations/144590801
108	Plectropterus gambensis	Spur-winged Goose	2014/12/22	5	0	0.0833333	32.3333333	https://www.inaturalist.org/observations/33471174
109	Plectropterus gambensis	Spur-winged Goose	2016/12/18	1	0	0.1109314	35.9082956	https://www.inaturalist.org/observations/78796021
110	Plectropterus gambensis	Spur-winged Goose	2021/6/22	1	0	0.1587833	32.5704433	https://www.inaturalist.org/observations/87524011
111	Plectropterus gambensis	Spur-winged Goose	2009/8/14	3	0	0.2401261	36.3681356	https://www.inaturalist.org/observations/35466441
112	Plectropterus gambensis	Spur-winged Goose	2019/6/9	3	0	0.5332617	33.8830850	https://www.inaturalist.org/observations/28353971
113	Plectropterus gambensis	Spur-winged Goose	2021/2/10	2	0	0.6257530	36.7887520	https://www.inaturalist.org/observations/69553531
114	Plectropterus gambensis	Spur-winged Goose	2019/6/24	2	0	1.4253069	32.0763415	https://www.inaturalist.org/observations/59031535

115	Plectropterus gambensis	Spur-winged Goose	2015/3/15	3	0	2.1802592	31.8246382	https://www.inaturalist.org/observations/44695184
116	Plectropterus gambensis	Spur-winged Goose	2017/6/10	2	0	2.2437343	31.4094600	https://www.inaturalist.org/observations/18599704
117	Plectropterus gambensis	Spur-winged Goose	2014/3/3	1	0	2.2568501	31.6472367	https://www.inaturalist.org/observations/127475497
118	Plectropterus gambensis	Spur-winged Goose	2014/3/3	3	0	2.2568501	31.6472367	https://www.inaturalist.org/observations/127476178
119	Plectropterus gambensis gambensis	Spur-winged Goose	2016/2/18	3	0	2.2646770	31.6577720	https://www.inaturalist.org/observations/34534242
120	Plectropterus gambensis gambensis	Spur-winged Goose	2016/2/18	3	0	2.2660530	31.6355130	https://www.inaturalist.org/observations/34533885
121	Plectropterus gambensis	Spur-winged Goose	2019/3/17	3	0	2.2720489	31.6214449	https://www.inaturalist.org/observations/37520592
122	Plectropterus gambensis	Spur-winged Goose	2019/3/17	4	0	2.2720592	31.6231356	https://www.inaturalist.org/observations/37495428
123	Plectropterus gambensis	Spur-winged Goose	2019/9/23	2	0	2.2741305	31.6712367	https://www.inaturalist.org/observations/69190505
124	Plectropterus gambensis gambensis	Spur-winged Goose	2014/2/6	2	0	2.2749810	31.6733120	https://www.inaturalist.org/observations/38220480
125	Plectropterus gambensis	Spur-winged Goose	2019/3/9	4	0	2.2750334	31.6324056	https://www.inaturalist.org/observations/38660290
126	Plectropterus gambensis	Spur-winged Goose	2016/2/13	5	0	2.2805592	31.5726104	http://www.inaturalist.org/observations/2829310
127	Plectropterus gambensis	Spur-winged Goose	2021/6/14	2	0	2.3121150	31.4243367	https://www.inaturalist.org/observations/84834407
128	Plectropterus gambensis	Spur-winged Goose	2017/2/3	2	0	2.3968084	31.5524408	https://www.inaturalist.org/observations/107292788
129	Plectropterus gambensis gambensis	Spur-winged Goose	2014/1/28	2	0	7.0332720	38.4617550	https://www.inaturalist.org/observations/37371707
130	Plectropterus gambensis	Spur-winged Goose	2011/1/6	5	0	7.0485600	38.4586720	https://www.inaturalist.org/observations/9776842
131	Plectropterus gambensis	Spur-winged Goose	2012/1/9	2	0	7.0795884	38.4790939	https://www.inaturalist.org/observations/109664085
132	Plectropterus gambensis	Spur-winged Goose	2011/1/11	5	0	7.0913620	38.4821250	https://www.inaturalist.org/observations/9777096
133	Plectropterus gambensis	Spur-winged Goose	2007/10/22	1	0	7.5938756	38.6365075	https://www.inaturalist.org/observations/123957976
134	Plectropterus gambensis	Spur-winged Goose	2012/12/26	4	0	7.5981830	38.7480920	https://www.inaturalist.org/observations/18677409
135	Plectropterus gambensis	Spur-winged Goose	2015/8/6	4	0	7.8299605	38.6608887	https://www.inaturalist.org/observations/9513319
136	Plectropterus gambensis	Spur-winged Goose	2019/3/20	4	0	7.9180287	38.7276310	https://www.inaturalist.org/observations/21532057
137	Plectropterus gambensis	Spur-winged Goose	2022/11/5	2	0	7.9265599	38.7267493	https://www.inaturalist.org/observations/142245476

138	Plectropterus gambensis gambensis	Spur-winged Goose	2019/11/14	5	0	7.9266229	38.7268537	https://www.inaturalist.org/observations/36761262
139	Plectropterus gambensis	Spur-winged Goose	2017/11/26	3	0	7.9272554	38.7270334	https://www.inaturalist.org/observations/42253134
140	Plectropterus gambensis	Spur-winged Goose	2018/11/17	4	0	8.0073084	38.8414602	https://www.inaturalist.org/observations/18610559
141	Plectropterus gambensis	Spur-winged Goose	2019/3/19	4	0	8.0073084	38.8414602	https://www.inaturalist.org/observations/21516838
142	Plectropterus gambensis	Spur-winged Goose	2018/11/17	2	0	8.0073084	38.8414602	https://www.inaturalist.org/observations/112412478
143	Plectropterus gambensis	Spur-winged Goose	2018/9/12	1	0	8.3399056	38.9871139	https://www.inaturalist.org/observations/63080950
144	Plectropterus gambensis	Spur-winged Goose	2018/9/12	1	0	8.3399222	38.9870889	https://www.inaturalist.org/observations/63080949
145	Plectropterus gambensis	Spur-winged Goose	2014/1/12	2	0	8.4673473	39.1558159	https://www.inaturalist.org/observations/106934494
146	Plectropterus gambensis	Spur-winged Goose	2014/2/22	1	0	9.2536585	34.8682286	https://www.inaturalist.org/observations/143529469
147	Plectropterus gambensis	Spur-winged Goose	2019/9/17	2	0	11.5742086	37.3613533	https://www.inaturalist.org/observations/58044525
148	Plectropterus gambensis	Spur-winged Goose	2014/4/16	1	0	11.5742086	37.3613533	https://www.inaturalist.org/observations/58448211
149	Plectropterus gambensis	Spur-winged Goose	2021/10/16	2	0	11.5742086	37.3613533	https://www.inaturalist.org/observations/103243580
150	Plectropterus gambensis	Spur-winged Goose	2022/2/11	1	0	11.5742086	37.3613533	https://www.inaturalist.org/observations/106540808
151	Plectropterus gambensis	Spur-winged Goose	2022/2/22	1	0	11.5742086	37.3613533	https://www.inaturalist.org/observations/107244552
152	Plectropterus gambensis	Spur-winged Goose	2022/3/31	1	0	11.5742086	37.3613533	https://www.inaturalist.org/observations/109999522
153	Plectropterus gambensis	Spur-winged Goose	2022/5/12	2	0	11.5742086	37.3613533	https://www.inaturalist.org/observations/116674198
154	Plectropterus gambensis	Spur-winged Goose	2022/5/12	2	0	11.5742086	37.3613533	https://www.inaturalist.org/observations/116674202
155	Plectropterus gambensis	Spur-winged Goose	2022/6/3	1	0	11.5742086	37.3613533	https://www.inaturalist.org/observations/119999773
156	Plectropterus gambensis	Spur-winged Goose	2022/8/21	2	0	11.5742086	37.3613533	https://www.inaturalist.org/observations/131882954
157	Plectropterus gambensis	Spur-winged Goose	2022/10/19	1	0	11.5742086	37.3613533	https://www.inaturalist.org/observations/139313669
158	Plectropterus gambensis	Spur-winged Goose	2022/10/20	1	0	11.5742086	37.3613533	https://www.inaturalist.org/observations/139412300
159	Plectropterus gambensis	Spur-winged Goose	2022/12/6	2	0	11.5742086	37.3613533	https://www.inaturalist.org/observations/143786744
160	Plectropterus gambensis	Spur-winged Goose	2018/4/14	4	0	11.5762266	37.4056419	https://www.inaturalist.org/observations/11075640

161	Plectropterus gambensis	Spur-winged Goose	2012/8/4	3	0	11.6199617	37.4084061	https://www.inaturalist.org/observations/65945130
162	Plectropterus gambensis	Spur-winged Goose	2020/2/26	1	0	11.6323363	37.3784637	https://www.inaturalist.org/observations/81148234
163	Plectropterus gambensis	Spur-winged Goose	2019/10/29	5	0	11.9495828	37.3020045	https://www.inaturalist.org/observations/35197980
164	Plectropterus gambensis gambensis	Spur-winged Goose	2020/2/18	2	0	11.9642963	37.7034069	https://www.inaturalist.org/observations/39682857
165	Plectropterus gambensis	Spur-winged Goose	2020/2/18	3	0	11.9642963	37.7034069	https://www.inaturalist.org/observations/39682865
166	Plectropterus gambensis	Spur-winged Goose	2020/2/27	1	0	12.1483100	37.7491390	https://www.inaturalist.org/observations/81405192

No	scientific name	common name	date	NIA	NID	latitude	longitude	url
1	Sagittarius serpentarius	Secretarybird	2021/3/31	5	0	-7.7017421	34.9391613	https://www.inaturalist.org/observations/72544739
2	Sagittarius serpentarius	Secretarybird	2022/8/28	3	0	-7.2943196	37.0615315	https://www.inaturalist.org/observations/134145885
3	Sagittarius serpentarius	Secretarybird	2019/7/2	4	0	-7.0270224	34.7721793	https://www.inaturalist.org/observations/80452736
4	Sagittarius serpentarius	Secretarybird	2022/3/23	6	0	-6.2080520	34.9838272	https://www.inaturalist.org/observations/110407539
5	Sagittarius serpentarius	Secretarybird	2017/6/19	4	0	-4.3997598	36.3734062	https://www.inaturalist.org/observations/19220562
6	Sagittarius serpentarius	Secretarybird	2018/11/13	4	0	-4.3642007	36.0845695	https://www.inaturalist.org/observations/18388559
7	Sagittarius serpentarius	Secretarybird	2017/11/23	5	0	-4.2694456	36.2245204	https://www.inaturalist.org/observations/9074582
8	Sagittarius serpentarius	Secretarybird	2017/9/23	6	0	-4.1857123	36.1199497	https://www.inaturalist.org/observations/33292851
9	Sagittarius serpentarius	Secretarybird	2022/1/3	6	0	-4.1790935	35.8976950	https://www.inaturalist.org/observations/116168190
10	Sagittarius serpentarius	Secretarybird	2018/8/31	5	0	-4.1570504	36.1837945	https://www.inaturalist.org/observations/20145309
11	Sagittarius serpentarius	Secretarybird	2022/7/11	5	0	-4.1495579	35.8132236	https://www.inaturalist.org/observations/127679530
12	Sagittarius serpentarius	Secretarybird	2019/7/17	6	0	-4.1434478	36.1306310	https://www.inaturalist.org/observations/108829930
13	Sagittarius serpentarius	Secretarybird	2021/10/30	4	0	-4.1427459	35.8888423	https://www.inaturalist.org/observations/101641850
14	Sagittarius serpentarius	Secretarybird	2018/2/10	6	0	-4.1321840	38.0992794	https://www.inaturalist.org/observations/40803117
15	Sagittarius serpentarius	Secretarybird	2020/2/18	5	0	-4.1307444	35.9760259	https://www.inaturalist.org/observations/134638591
16	Sagittarius serpentarius	Secretarybird	2022/7/11	6	0	-4.1067568	35.9653482	https://www.inaturalist.org/observations/129412701
17	Sagittarius serpentarius	Secretarybird	2020/4/2	6	0	-4.0934114	38.0264980	https://www.inaturalist.org/observations/42403059
18	Sagittarius serpentarius	Secretarybird	2002/2/5	8	0	-4.0619377	36.1215069	http://www.inaturalist.org/observations/560977
19	Sagittarius serpentarius	Secretarybird	2021/7/11	3	0	-4.0437290	35.8326790	https://www.inaturalist.org/observations/122153343
20	Sagittarius serpentarius	Secretarybird	2018/6/20	4	0	-4.0270983	36.0624104	https://www.inaturalist.org/observations/13769589
21	Sagittarius serpentarius	Secretarybird	2017/11/15	4	0	-4.0044793	35.8040323	https://www.inaturalist.org/observations/19468389
22	Sagittarius serpentarius	Secretarybird	2007/10/19	8	0	-3.9864962	35.8598035	http://www.inaturalist.org/observations/18339

23	Sagittarius serpentarius	Secretarybird	2020/12/22	5	0	-3.9673379	35.9486686	https://www.inaturalist.org/observations/67760375
24	Sagittarius serpentarius	Secretarybird	2021/9/12	4	0	-3.9587271	37.6620376	https://www.inaturalist.org/observations/95123690
25	Sagittarius serpentarius	Secretarybird	2016/4/30	5	0	-3.9265239	36.0774640	https://www.inaturalist.org/observations/80770750
26	Sagittarius serpentarius	Secretarybird	2018/6/7	5	0	-3.9165302	35.9817506	https://www.inaturalist.org/observations/15919496
27	Sagittarius serpentarius	Secretarybird	2015/7/22	5	0	-3.9108858	36.1495948	https://www.inaturalist.org/observations/126045617
28	Sagittarius serpentarius	Secretarybird	2022/7/17	4	0	-3.9046788	36.1245170	https://www.inaturalist.org/observations/128194468
29	Sagittarius serpentarius	Secretarybird	2021/10/30	4	0	-3.8901333	36.0821772	https://www.inaturalist.org/observations/103610565
30	Sagittarius serpentarius	Secretarybird	2019/9/23	3	0	-3.8776805	37.9704706	https://www.inaturalist.org/observations/35913028
31	Sagittarius serpentarius	Secretarybird	2017/12/20	6	0	-3.8593268	35.9035752	https://www.inaturalist.org/observations/38129191
32	Sagittarius serpentarius	Secretarybird	2013/6/6	4	0	-3.8566830	36.0841107	https://www.inaturalist.org/observations/85741112
33	Sagittarius serpentarius	Secretarybird	2017/12/20	7	0	-3.8540435	35.9422628	https://www.inaturalist.org/observations/38129280
34	Sagittarius serpentarius	Secretarybird	2019/8/4	3	0	-3.8394736	35.9050298	https://www.inaturalist.org/observations/70637161
35	Sagittarius serpentarius	Secretarybird	2019/8/16	7	0	-3.8297194	35.8084759	https://www.inaturalist.org/observations/33434967
36	Sagittarius serpentarius	Secretarybird	2019/8/16	6	0	-3.8253379	35.9160854	https://www.inaturalist.org/observations/33434858
37	Sagittarius serpentarius	Secretarybird	2015/3/1	5	0	-3.8160526	35.8766766	https://www.inaturalist.org/observations/67721066
38	Sagittarius serpentarius	Secretarybird	2019/7/31	4	0	-3.8154787	35.8389143	https://www.inaturalist.org/observations/68993017
39	Sagittarius serpentarius	Secretarybird	2017/7/5	5	0	-3.8142238	38.8332557	https://www.inaturalist.org/observations/104784632
40	Sagittarius serpentarius	Secretarybird	2021/4/14	7	0	-3.8122358	37.8792407	https://www.inaturalist.org/observations/105830985
41	Sagittarius serpentarius	Secretarybird	2021/6/28	5	0	-3.7998367	35.8081829	https://www.inaturalist.org/observations/85097022
42	Sagittarius serpentarius	Secretarybird	2017/7/5	6	0	-3.7956638	38.6069255	https://www.inaturalist.org/observations/8660665
43	Sagittarius serpentarius	Secretarybird	2022/10/27	2	0	-3.7493761	36.0625189	https://www.inaturalist.org/observations/140291651
44	Sagittarius serpentarius	Secretarybird	2018/2/5	5	0	-3.7474509	36.1092995	https://www.inaturalist.org/observations/16659865
45	Sagittarius serpentarius	Secretarybird	2014/11/20	4	0	-3.7362264	36.1259894	https://www.inaturalist.org/observations/19734583

46	Sagittarius serpentarius	Secretarybird	2015/2/14	4	0	-3.7247968	35.8939759	https://www.inaturalist.org/observations/34865404
47	Sagittarius serpentarius	Secretarybird	2021/4/11	6	0	-3.7144286	35.9982457	https://www.inaturalist.org/observations/80523726
48	Sagittarius serpentarius	Secretarybird	2019/7/28	6	0	-3.7079137	35.9782495	https://www.inaturalist.org/observations/60904388
49	Sagittarius serpentarius	Secretarybird	2022/1/7	4	0	-3.6931413	38.8876736	https://www.inaturalist.org/observations/112719994
50	Sagittarius serpentarius	Secretarybird	2017/7/4	8	0	-3.6748762	38.7988033	https://www.inaturalist.org/observations/7694555
51	Sagittarius serpentarius	Secretarybird	2019/8/18	4	0	-3.6482738	38.1452043	https://www.inaturalist.org/observations/34317370
52	Sagittarius serpentarius	Secretarybird	2021/10/30	5	0	-3.6333306	35.8553165	https://www.inaturalist.org/observations/102323115
53	Sagittarius serpentarius	Secretarybird	2018/10/19	7	0	-3.6297252	36.0928804	https://www.inaturalist.org/observations/18012871
54	Sagittarius serpentarius	Secretarybird	2022/1/16	5	0	-3.6144815	36.1394832	https://www.inaturalist.org/observations/105664719
55	Sagittarius serpentarius	Secretarybird	2018/6/15	6	0	-3.5755946	36.0115507	https://www.inaturalist.org/observations/15092153
56	Sagittarius serpentarius	Secretarybird	2022/4/11	3	0	-3.5754492	38.3966604	https://www.inaturalist.org/observations/111066455
57	Sagittarius serpentarius	Secretarybird	2019/11/24	3	0	-3.5720635	38.0905069	https://www.inaturalist.org/observations/37411629
58	Sagittarius serpentarius	Secretarybird	2018/11/11	4	0	-3.5708821	38.8172578	https://www.inaturalist.org/observations/78652202
59	Sagittarius serpentarius	Secretarybird	2020/2/26	4	0	-3.5653387	38.3379108	https://www.inaturalist.org/observations/39323751
60	Sagittarius serpentarius	Secretarybird	2014/12/29	7	0	-3.5593725	38.2372831	http://www.inaturalist.org/observations/1200884
61	Sagittarius serpentarius	Secretarybird	2019/11/18	3	0	-3.5271675	38.0142747	https://www.inaturalist.org/observations/37411597
62	Sagittarius serpentarius	Secretarybird	2019/11/18	3	0	-3.5135843	38.0470615	https://www.inaturalist.org/observations/37411595
63	Sagittarius serpentarius	Secretarybird	2019/11/16	4	0	-3.5024725	37.9596985	https://www.inaturalist.org/observations/37411555
64	Sagittarius serpentarius	Secretarybird	2020/7/20	5	0	-3.4768280	38.9812003	https://www.inaturalist.org/observations/54040957
65	Sagittarius serpentarius	Secretarybird	2010/10/27	5	0	-3.4535247	38.6357698	https://www.inaturalist.org/observations/132043521
66	Sagittarius serpentarius	Secretarybird	2015/2/27	4	0	-3.4424437	38.6442839	https://www.inaturalist.org/observations/106813327
67	Sagittarius serpentarius	Secretarybird	2022/7/22	4	0	-3.4349283	36.0074877	https://www.inaturalist.org/observations/127496204
68	Sagittarius serpentarius	Secretarybird	2022/4/11	4	0	-3.4211326	38.2503370	https://www.inaturalist.org/observations/111066121

69	Sagittarius serpentarius	Secretarybird	2020/2/25	4	0	-3.3744551	37.7702434	https://www.inaturalist.org/observations/39322899
70	Sagittarius serpentarius	Secretarybird	2013/11/14	6	0	-3.3658985	38.6004942	https://www.inaturalist.org/observations/18775861
71	Sagittarius serpentarius	Secretarybird	2005/2/13	5	0	-3.2991491	35.5842202	https://www.inaturalist.org/observations/37948472
72	Sagittarius serpentarius	Secretarybird	2015/3/23	6	0	-3.2771391	35.6927741	https://www.inaturalist.org/observations/67735237
73	Sagittarius serpentarius	Secretarybird	2013/3/6	6	0	-3.2695263	35.4629209	https://www.inaturalist.org/observations/98756036
74	Sagittarius serpentarius	Secretarybird	2018/4/7	5	0	-3.2669562	38.4536809	https://www.inaturalist.org/observations/63258775
75	Sagittarius serpentarius	Secretarybird	2019/8/23	4	0	-3.2571307	35.4836324	https://www.inaturalist.org/observations/35920811
76	Sagittarius serpentarius	Secretarybird	2014/11/6	4	0	-3.2317500	38.6864619	https://www.inaturalist.org/observations/92700137
77	Sagittarius serpentarius	Secretarybird	2022/1/3	4	0	-3.2293787	38.6763461	https://www.inaturalist.org/observations/112714066
78	Sagittarius serpentarius	Secretarybird	2020/8/19	3	0	-3.2206604	38.5777878	https://www.inaturalist.org/observations/106415689
79	Sagittarius serpentarius	Secretarybird	2018/4/20	5	0	-3.2156918	35.4183357	https://www.inaturalist.org/observations/14482308
80	Sagittarius serpentarius	Secretarybird	2017/11/10	5	0	-3.2095839	35.4620279	https://www.inaturalist.org/observations/19370176
81	Sagittarius serpentarius	Secretarybird	2015/8/23	4	0	-3.2088851	35.4812263	https://www.inaturalist.org/observations/30321398
82	Sagittarius serpentarius	Secretarybird	2020/1/27	5	0	-3.1986320	35.1959858	https://www.inaturalist.org/observations/93222393
83	Sagittarius serpentarius	Secretarybird	2019/8/1	5	0	-3.1970543	35.5828302	https://www.inaturalist.org/observations/32938838
84	Sagittarius serpentarius	Secretarybird	2007/10/24	7	0	-3.1906431	35.6277505	https://www.inaturalist.org/observations/32955892
85	Sagittarius serpentarius	Secretarybird	2019/12/31	5	0	-3.1829185	35.5449569	https://www.inaturalist.org/observations/120935905
86	Sagittarius serpentarius	Secretarybird	2011/3/8	4	0	-3.1786482	34.9986156	https://www.inaturalist.org/observations/107139637
87	Sagittarius serpentarius	Secretarybird	2014/3/9	8	0	-3.1773551	35.5773939	https://www.inaturalist.org/observations/5385452
88	Sagittarius serpentarius	Secretarybird	2019/2/23	2	0	-3.1730203	35.4054891	https://www.inaturalist.org/observations/142916133
89	Sagittarius serpentarius	Secretarybird	2017/11/10	4	0	-3.1697167	35.4282790	https://www.inaturalist.org/observations/19389248
90	Sagittarius serpentarius	Secretarybird	2013/6/12	7	0	-3.1687882	35.5475341	https://www.inaturalist.org/observations/82707441
91	Sagittarius serpentarius	Secretarybird	2020/1/9	5	0	-3.1665749	35.4561068	https://www.inaturalist.org/observations/70373241

92	Sagittarius serpentarius	Secretarybird	2018/2/1	4	0	-3.1659281	35.5361222	https://www.inaturalist.org/observations/20818244
93	Sagittarius serpentarius	Secretarybird	2017/2/19	8	0	-3.1635978	35.5419880	https://www.inaturalist.org/observations/6098406
94	Sagittarius serpentarius	Secretarybird	2014/3/30	4	0	-3.1631842	35.3758562	https://www.inaturalist.org/observations/64895943
95	Sagittarius serpentarius	Secretarybird	2017/1/11	4	0	-3.1629025	35.7365425	https://www.inaturalist.org/observations/82927491
96	Sagittarius serpentarius	Secretarybird	2022/7/11	3	0	-3.1625391	35.5437513	https://www.inaturalist.org/observations/125770696
97	Sagittarius serpentarius	Secretarybird	2015/10/6	5	0	-3.1624838	35.5328185	https://www.inaturalist.org/observations/75123709
98	Sagittarius serpentarius	Secretarybird	2010/12/19	5	0	-3.1615378	38.6826424	https://www.inaturalist.org/observations/34853665
99	Sagittarius serpentarius	Secretarybird	2019/12/22	8	0	-3.1488051	35.5097720	https://www.inaturalist.org/observations/56902773
100	Sagittarius serpentarius	Secretarybird	2011/3/9	4	0	-3.1476951	34.8127318	https://www.inaturalist.org/observations/107146028
101	Sagittarius serpentarius	Secretarybird	2012/12/3	6	0	-3.1345225	34.8657944	https://www.inaturalist.org/observations/27634354
102	Sagittarius serpentarius	Secretarybird	2019/2/14	5	0	-3.1301163	38.0820411	https://www.inaturalist.org/observations/49698988
103	Sagittarius serpentarius	Secretarybird	2019/8/17	6	0	-3.1278727	35.5034658	https://www.inaturalist.org/observations/107254088
104	Sagittarius serpentarius	Secretarybird	2019/7/16	5	0	-3.1111348	35.4503966	https://www.inaturalist.org/observations/30282653
105	Sagittarius serpentarius	Secretarybird	2005/12/21	4	0	-3.1074477	35.5650903	https://www.inaturalist.org/observations/22335248
106	Sagittarius serpentarius	Secretarybird	2019/1/23	7	0	-3.1063355	38.8417345	https://www.inaturalist.org/observations/19838569
107	Sagittarius serpentarius	Secretarybird	2014/8/19	5	0	-3.1016156	36.0205269	https://www.inaturalist.org/observations/70385089
108	Sagittarius serpentarius	Secretarybird	2018/6/23	4	0	-3.1000555	35.5151874	https://www.inaturalist.org/observations/13999083
109	Sagittarius serpentarius	Secretarybird	2020/2/11	4	0	-3.0982007	35.1744551	https://www.inaturalist.org/observations/109088366
110	Sagittarius serpentarius	Secretarybird	2018/10/21	6	0	-3.0958190	35.5446175	https://www.inaturalist.org/observations/18028016
111	Sagittarius serpentarius	Secretarybird	2008/8/10	8	0	-3.0894760	35.4220806	https://www.inaturalist.org/observations/7131270
112	Sagittarius serpentarius	Secretarybird	2017/5/28	6	0	-3.0867823	35.4582444	https://www.inaturalist.org/observations/135144965
113	Sagittarius serpentarius	Secretarybird	2017/11/2	1	0	-3.0848076	35.4977507	https://www.inaturalist.org/observations/142937310
114	Sagittarius serpentarius	Secretarybird	2014/9/30	6	0	-3.0842282	35.4356589	http://www.inaturalist.org/observations/1065693

115	Sagittarius serpentarius	Secretarybird	2017/11/2	3	0	-3.0816918	35.4444800	https://www.inaturalist.org/observations/142938046
116	Sagittarius serpentarius	Secretarybird	2020/1/2	6	0	-3.0799248	35.5428094	https://www.inaturalist.org/observations/37688528
117	Sagittarius serpentarius	Secretarybird	2006/11/27	5	0	-3.0725795	35.4871195	http://www.inaturalist.org/observations/2362015
118	Sagittarius serpentarius	Secretarybird	2011/9/10	6	0	-3.0710974	35.5832272	https://www.inaturalist.org/observations/130018455
119	Sagittarius serpentarius	Secretarybird	2022/1/15	4	0	-3.0699727	35.5220813	https://www.inaturalist.org/observations/105513677
120	Sagittarius serpentarius	Secretarybird	2019/10/1	8	0	-3.0683329	35.5650985	https://www.inaturalist.org/observations/33938225
121	Sagittarius serpentarius	Secretarybird	2015/9/16	4	0	-3.0682850	35.4825753	https://www.inaturalist.org/observations/27269313
122	Sagittarius serpentarius	Secretarybird	2007/8/10	4	0	-3.0552779	35.5629483	https://www.inaturalist.org/observations/103391863
123	Sagittarius serpentarius	Secretarybird	2004/11/9	4	0	-3.0530726	35.5874016	https://www.inaturalist.org/observations/81510574
124	Sagittarius serpentarius	Secretarybird	2018/8/21	4	0	-3.0513394	35.5100389	https://www.inaturalist.org/observations/74429462
125	Sagittarius serpentarius	Secretarybird	2018/4/16	4	0	-3.0474175	35.1333491	https://www.inaturalist.org/observations/14117021
126	Sagittarius serpentarius	Secretarybird	2018/7/13	4	0	-3.0438640	35.5071801	https://www.inaturalist.org/observations/14596450
127	Sagittarius serpentarius	Secretarybird	2019/9/24	6	0	-3.0387369	35.4292184	https://www.inaturalist.org/observations/49717610
128	Sagittarius serpentarius	Secretarybird	2022/1/4	4	0	-3.0380856	35.4475317	https://www.inaturalist.org/observations/116446975
129	Sagittarius serpentarius	Secretarybird	2013/2/13	4	0	-3.0364702	35.1799868	https://www.inaturalist.org/observations/34923685
130	Sagittarius serpentarius	Secretarybird	2017/11/11	4	0	-3.0346430	35.5318401	https://www.inaturalist.org/observations/19434764
131	Sagittarius serpentarius	Secretarybird	2018/7/27	4	0	-3.0258473	35.5912076	https://www.inaturalist.org/observations/14819329
132	Sagittarius serpentarius	Secretarybird	2019/11/3	6	0	-3.0195984	35.5376665	https://www.inaturalist.org/observations/35937704
133	Sagittarius serpentarius	Secretarybird	2017/11/2	2	0	-3.0187148	35.4678254	https://www.inaturalist.org/observations/142937312
134	Sagittarius serpentarius	Secretarybird	2017/12/4	5	0	-3.0138526	35.4117044	https://www.inaturalist.org/observations/51997861
135	Sagittarius serpentarius	Secretarybird	2018/1/27	4	0	-3.0011126	34.9346529	https://www.inaturalist.org/observations/16850647
136	Sagittarius serpentarius	Secretarybird	2017/8/10	4	0	-3.0006443	35.4159774	https://www.inaturalist.org/observations/17209271
137	Sagittarius serpentarius	Secretarybird	2018/11/15	5	0	-3.0004492	35.5035266	https://www.inaturalist.org/observations/21691599

138	Sagittarius serpentarius	Secretarybird	2017/7/12	5	0	-3.0001535	35.5405792	https://www.inaturalist.org/observations/9300384
139	Sagittarius serpentarius	Secretarybird	2011/6/10	6	0	-3.0001321	35.4903229	https://www.inaturalist.org/observations/50964108
140	Sagittarius serpentarius	Secretarybird	2018/8/28	5	0	-2.9862087	34.8408805	https://www.inaturalist.org/observations/19269713
141	Sagittarius serpentarius	Secretarybird	2014/9/4	7	0	-2.9836828	35.0592777	http://www.inaturalist.org/observations/2748328
142	Sagittarius serpentarius	Secretarybird	2019/6/25	6	0	-2.9801601	36.4744781	https://www.inaturalist.org/observations/131329417
143	Sagittarius serpentarius	Secretarybird	2019/11/29	3	0	-2.9758948	33.8788347	https://www.inaturalist.org/observations/68811946
144	Sagittarius serpentarius	Secretarybird	2019/10/23	4	0	-2.9751223	35.1015305	https://www.inaturalist.org/observations/35413320
145	Sagittarius serpentarius	Secretarybird	2020/6/24	1	0	-2.9725247	37.8082769	https://www.inaturalist.org/observations/51558638
146	Sagittarius serpentarius	Secretarybird	2020/2/10	3	0	-2.9383259	35.0419746	https://www.inaturalist.org/observations/108501997
147	Sagittarius serpentarius	Secretarybird	2015/3/21	5	0	-2.9380030	36.9547812	https://www.inaturalist.org/observations/50765035
148	Sagittarius serpentarius	Secretarybird	2007/10/14	5	0	-2.9379377	34.6964551	https://www.inaturalist.org/observations/19669216
149	Sagittarius serpentarius	Secretarybird	2014/9/1	5	0	-2.9325648	36.7751827	https://www.inaturalist.org/observations/12104979
150	Sagittarius serpentarius	Secretarybird	2021/4/16	6	0	-2.9321552	35.1471663	https://www.inaturalist.org/observations/82612987
151	Sagittarius serpentarius	Secretarybird	2015/8/19	4	0	-2.9080677	38.8429278	https://www.inaturalist.org/observations/19351705
152	Sagittarius serpentarius	Secretarybird	2017/2/26	5	0	-2.9029057	35.0424950	https://www.inaturalist.org/observations/9519338
153	Sagittarius serpentarius	Secretarybird	2020/4/4	7	0	-2.8971144	35.1924330	https://www.inaturalist.org/observations/41405257
154	Sagittarius serpentarius	Secretarybird	2021/12/1	4	0	-2.8867590	35.4277108	https://www.inaturalist.org/observations/102308086
155	Sagittarius serpentarius	Secretarybird	2016/2/7	10	0	-2.8850678	35.0086331	http://www.inaturalist.org/observations/2786910
156	Sagittarius serpentarius	Secretarybird	2022/9/13	1	0	-2.8773416	38.1597936	https://www.inaturalist.org/observations/145036168
157	Sagittarius serpentarius	Secretarybird	2022/7/25	4	0	-2.8720404	38.1129751	https://www.inaturalist.org/observations/137400497
158	Sagittarius serpentarius	Secretarybird	2022/1/12	4	0	-2.8679595	34.8242250	https://www.inaturalist.org/observations/105277501
159	Sagittarius serpentarius	Secretarybird	2018/4/17	4	0	-2.8631912	34.8402644	https://www.inaturalist.org/observations/14117270
160	Sagittarius serpentarius	Secretarybird	2022/7/28	5	0	-2.8522500	38.0628332	https://www.inaturalist.org/observations/128281526

161	Sagittarius serpentarius	Secretarybird	2022/1/28	4	0	-2.8489602	35.2330240	https://www.inaturalist.org/observations/107240131
162	Sagittarius serpentarius	Secretarybird	2013/7/2	6	0	-2.8428892	37.8631014	http://www.inaturalist.org/observations/727244
163	Sagittarius serpentarius	Secretarybird	2017/2/25	10	0	-2.8427017	34.8159661	https://www.inaturalist.org/observations/6509733
164	Sagittarius serpentarius	Secretarybird	2019/11/30	6	0	-2.8380641	35.0900326	https://www.inaturalist.org/observations/68919703
165	Sagittarius serpentarius	Secretarybird	2022/1/28	5	0	-2.8316361	34.9411990	https://www.inaturalist.org/observations/107299086
166	Sagittarius serpentarius	Secretarybird	2022/1/29	6	0	-2.8314630	35.0573530	https://www.inaturalist.org/observations/107422473
167	Sagittarius serpentarius	Secretarybird	1986/3/17	5	0	-2.8299633	38.0336848	https://www.inaturalist.org/observations/63830392
168	Sagittarius serpentarius	Secretarybird	2017/1/27	4	0	-2.8200903	34.8226547	https://www.inaturalist.org/observations/91572042
169	Sagittarius serpentarius	Secretarybird	2013/11/12	3	0	-2.8189306	35.3559058	https://www.inaturalist.org/observations/35665316
170	Sagittarius serpentarius	Secretarybird	2021/7/20	4	0	-2.8082119	35.3948537	https://www.inaturalist.org/observations/88817735
171	Sagittarius serpentarius	Secretarybird	1990/10/18	7	0	-2.8065989	35.3606562	http://www.inaturalist.org/observations/4484397
172	Sagittarius serpentarius	Secretarybird	1993/12/28	6	0	-2.7996426	34.8710350	https://www.inaturalist.org/observations/135828251
173	Sagittarius serpentarius	Secretarybird	2022/6/25	3	0	-2.7988292	34.8801339	https://www.inaturalist.org/observations/125140860
174	Sagittarius serpentarius	Secretarybird	2017/5/20	11	0	-2.7968547	37.2383923	https://www.inaturalist.org/observations/6812086
175	Sagittarius serpentarius	Secretarybird	2015/9/4	6	0	-2.7941743	34.8505378	https://www.inaturalist.org/observations/67365190
176	Sagittarius serpentarius	Secretarybird	2012/6/9	8	0	-2.7927532	34.8245521	http://www.inaturalist.org/observations/509220
177	Sagittarius serpentarius	Secretarybird	2021/9/9	7	0	-2.7894082	34.7688350	https://www.inaturalist.org/observations/100431815
178	Sagittarius serpentarius	Secretarybird	2016/6/8	5	0	-2.7879252	37.3281217	https://www.inaturalist.org/observations/141637632
179	Sagittarius serpentarius	Secretarybird	2021/7/20	4	0	-2.7872174	37.3371140	https://www.inaturalist.org/observations/88426999
180	Sagittarius serpentarius	Secretarybird	2020/12/16	4	0	-2.7871017	37.2848571	https://www.inaturalist.org/observations/79055004
181	Sagittarius serpentarius	Secretarybird	2017/7/8	5	0	-2.7848725	37.2095693	https://www.inaturalist.org/observations/77851869
182	Sagittarius serpentarius	Secretarybird	2010/5/3	8	0	-2.7848705	37.3741552	https://www.inaturalist.org/observations/7091090
183	Sagittarius serpentarius	Secretarybird	2021/9/8	4	0	-2.7766713	34.7077444	https://www.inaturalist.org/observations/100606642

184	Sagittarius serpentarius	Secretarybird	2019/6/25	5	0	-2.7763340	37.3616006	https://www.inaturalist.org/observations/37077912
185	Sagittarius serpentarius	Secretarybird	2018/3/9	6	0	-2.7755510	37.2445237	https://www.inaturalist.org/observations/56918820
186	Sagittarius serpentarius	Secretarybird	2014/12/23	4	0	-2.7663193	37.3593758	https://www.inaturalist.org/observations/65563211
187	Sagittarius serpentarius	Secretarybird	2021/8/10	4	0	-2.7632035	38.5949003	https://www.inaturalist.org/observations/136176612
188	Sagittarius serpentarius	Secretarybird	2017/8/30	8	0	-2.7631050	37.3205458	https://www.inaturalist.org/observations/7891887
189	Sagittarius serpentarius	Secretarybird	2021/6/18	5	0	-2.7517541	37.3648243	https://www.inaturalist.org/observations/83480300
190	Sagittarius serpentarius	Secretarybird	2020/8/18	3	0	-2.7453235	38.0036805	https://www.inaturalist.org/observations/106379091
191	Sagittarius serpentarius	Secretarybird	2016/8/12	4	0	-2.7337672	34.9572670	https://www.inaturalist.org/observations/21242041
192	Sagittarius serpentarius	Secretarybird	2022/4/12	5	0	-2.7331937	37.3891618	https://www.inaturalist.org/observations/111508970
193	Sagittarius serpentarius	Secretarybird	2021/2/21	5	0	-2.7330922	34.8613121	https://www.inaturalist.org/observations/73402518
194	Sagittarius serpentarius	Secretarybird	2022/1/30	4	0	-2.7279541	34.8280649	https://www.inaturalist.org/observations/107686960
195	Sagittarius serpentarius	Secretarybird	2011/4/22	5	0	-2.7265468	37.2360658	https://www.inaturalist.org/observations/50192050
196	Sagittarius serpentarius	Secretarybird	2022/9/22	4	0	-2.7258266	34.8751863	https://www.inaturalist.org/observations/136134852
197	Sagittarius serpentarius	Secretarybird	2007/2/7	2	0	-2.7224982	35.0623704	https://www.inaturalist.org/observations/137128017
198	Sagittarius serpentarius	Secretarybird	2018/2/9	5	0	-2.7213436	37.2975999	https://www.inaturalist.org/observations/10003046
199	Sagittarius serpentarius	Secretarybird	2016/9/5	3	0	-2.7212797	34.7694056	https://www.inaturalist.org/observations/106133548
200	Sagittarius serpentarius	Secretarybird	2021/9/26	4	0	-2.7189390	34.9644995	https://www.inaturalist.org/observations/96865088
201	Sagittarius serpentarius	Secretarybird	2018/10/18	5	0	-2.7177351	37.2069348	https://www.inaturalist.org/observations/17983655
202	Sagittarius serpentarius	Secretarybird	2022/7/21	3	0	-2.7144675	35.0121877	https://www.inaturalist.org/observations/128677384
203	Sagittarius serpentarius	Secretarybird	2017/6/1	5	0	-2.7043557	37.3623458	https://www.inaturalist.org/observations/17853818
204	Sagittarius serpentarius	Secretarybird	2018/3/24	6	0	-2.7033182	37.3289779	https://www.inaturalist.org/observations/10565638
205	Sagittarius serpentarius	Secretarybird	2018/7/13	7	0	-2.7031486	34.8698788	https://www.inaturalist.org/observations/14423687
206	Sagittarius serpentarius	Secretarybird	2018/10/24	7	0	-2.7025414	34.6431638	https://www.inaturalist.org/observations/18038102

207	Sagittarius serpentarius	Secretarybird	2013/8/16	4	0	-2.7007006	37.2461372	https://www.inaturalist.org/observations/68802808
208	Sagittarius serpentarius	Secretarybird	2013/6/24	7	0	-2.6984050	37.3809688	https://www.inaturalist.org/observations/56965140
209	Sagittarius serpentarius	Secretarybird	2017/8/30	9	0	-2.6915847	37.2101763	https://www.inaturalist.org/observations/7898917
210	Sagittarius serpentarius	Secretarybird	2017/8/20	8	0	-2.6804950	37.0183473	https://www.inaturalist.org/observations/7749309
211	Sagittarius serpentarius	Secretarybird	2021/12/3	3	0	-2.6789944	34.7873504	https://www.inaturalist.org/observations/102416026
212	Sagittarius serpentarius	Secretarybird	2007/10/1	4	0	-2.6776760	34.9090771	https://www.inaturalist.org/observations/25949955
213	Sagittarius serpentarius	Secretarybird	2021/8/7	4	0	-2.6731022	37.2708217	https://www.inaturalist.org/observations/94410771
214	Sagittarius serpentarius	Secretarybird	2017/11/18	6	0	-2.6676483	37.2507323	https://www.inaturalist.org/observations/19208155
215	Sagittarius serpentarius	Secretarybird	2018/9/15	5	0	-2.6657178	37.2933105	https://www.inaturalist.org/observations/53669734
216	Sagittarius serpentarius	Secretarybird	2021/5/24	6	0	-2.6655650	38.5021148	https://www.inaturalist.org/observations/82251934
217	Sagittarius serpentarius	Secretarybird	2021/12/4	4	0	-2.6611845	37.3196760	https://www.inaturalist.org/observations/102762828
218	Sagittarius serpentarius	Secretarybird	2021/3/17	4	0	-2.6565106	37.3358933	https://www.inaturalist.org/observations/98468353
219	Sagittarius serpentarius	Secretarybird	2018/2/16	4	0	-2.6532562	37.3786888	https://www.inaturalist.org/observations/16312405
220	Sagittarius serpentarius	Secretarybird	2018/7/13	4	0	-2.6500783	34.9880872	https://www.inaturalist.org/observations/15193918
221	Sagittarius serpentarius	Secretarybird	2005/12/22	4	0	-2.6474154	34.9587072	https://www.inaturalist.org/observations/21642568
222	Sagittarius serpentarius	Secretarybird	2019/6/26	5	0	-2.6457784	34.9286655	https://www.inaturalist.org/observations/29662595
223	Sagittarius serpentarius	Secretarybird	2010/6/13	4	0	-2.6454681	34.9261659	https://www.inaturalist.org/observations/36057829
224	Sagittarius serpentarius	Secretarybird	2022/5/15	6	0	-2.6431435	37.2593368	https://www.inaturalist.org/observations/117584425
225	Sagittarius serpentarius	Secretarybird	2019/9/17	6	0	-2.6373631	37.1947709	https://www.inaturalist.org/observations/35704610
226	Sagittarius serpentarius	Secretarybird	2020/5/10	3	0	-2.6344274	37.3350280	https://www.inaturalist.org/observations/45516496
227	Sagittarius serpentarius	Secretarybird	2018/8/30	4	0	-2.6305124	37.2160551	https://www.inaturalist.org/observations/19326379
228	Sagittarius serpentarius	Secretarybird	2022/10/3	3	0	-2.6281130	37.2965144	https://www.inaturalist.org/observations/140056988
229	Sagittarius serpentarius	Secretarybird	2017/9/26	5	0	-2.6250514	37.3304270	https://www.inaturalist.org/observations/70273061

230	Sagittarius serpentarius	Secretarybird	2015/9/28	5	0	-2.6202732	37.2107180	https://www.inaturalist.org/observations/134607781
231	Sagittarius serpentarius	Secretarybird	2022/10/5	2	0	-2.6185566	37.3436543	https://www.inaturalist.org/observations/144751923
232	Sagittarius serpentarius	Secretarybird	2018/8/7	7	0	-2.6181262	35.1604659	https://www.inaturalist.org/observations/15397665
233	Sagittarius serpentarius	Secretarybird	2019/10/19	7	0	-2.6172752	37.3863703	https://www.inaturalist.org/observations/34732820
234	Sagittarius serpentarius	Secretarybird	2015/3/23	10	0	-2.6089161	34.9756094	https://www.inaturalist.org/observations/6887996
235	Sagittarius serpentarius	Secretarybird	2009/1/9	6	0	-2.6076455	34.8058456	https://www.inaturalist.org/observations/135951409
236	Sagittarius serpentarius	Secretarybird	2019/8/8	5	0	-2.6042137	37.2514338	https://www.inaturalist.org/observations/31625521
237	Sagittarius serpentarius	Secretarybird	2007/10/10	4	0	-2.6029955	37.2272888	https://www.inaturalist.org/observations/19543459
238	Sagittarius serpentarius	Secretarybird	2019/5/16	3	0	-2.6026430	35.3024589	https://www.inaturalist.org/observations/69769062
239	Sagittarius serpentarius	Secretarybird	2019/8/22	6	0	-2.5968989	34.8229872	https://www.inaturalist.org/observations/32776527
240	Sagittarius serpentarius	Secretarybird	2022/7/20	4	0	-2.5948565	34.8315430	https://www.inaturalist.org/observations/128518466
241	Sagittarius serpentarius	Secretarybird	2018/4/12	5	0	-2.5928934	34.8035032	https://www.inaturalist.org/observations/13624890
242	Sagittarius serpentarius	Secretarybird	2019/10/3	4	0	-2.5926140	37.2447580	https://www.inaturalist.org/observations/36427323
243	Sagittarius serpentarius	Secretarybird	2022/7/20	4	0	-2.5925277	35.1473529	https://www.inaturalist.org/observations/128521198
244	Sagittarius serpentarius	Secretarybird	2018/5/17	5	0	-2.5905038	34.9072928	https://www.inaturalist.org/observations/13009563
245	Sagittarius serpentarius	Secretarybird	2017/9/20	6	0	-2.5867039	34.8007376	https://www.inaturalist.org/observations/33292850
246	Sagittarius serpentarius	Secretarybird	2015/8/14	4	0	-2.5848390	34.8914850	https://www.inaturalist.org/observations/14374042
247	Sagittarius serpentarius	Secretarybird	2018/10/22	7	0	-2.5741771	34.9549582	https://www.inaturalist.org/observations/18028385
248	Sagittarius serpentarius	Secretarybird	2013/11/13	4	0	-2.5718847	34.9673349	https://www.inaturalist.org/observations/35665334
249	Sagittarius serpentarius	Secretarybird	2018/5/17	6	0	-2.5702385	34.9141147	https://www.inaturalist.org/observations/18878695
250	Sagittarius serpentarius	Secretarybird	2007/11/10	4	0	-2.5665024	34.8837344	https://www.inaturalist.org/observations/124223540
251	Sagittarius serpentarius	Secretarybird	2007/11/11	4	0	-2.5597654	38.5249331	https://www.inaturalist.org/observations/19714179
252	Sagittarius serpentarius	Secretarybird	2015/9/21	4	0	-2.5556532	34.9233743	https://www.inaturalist.org/observations/12315219

253	Sagittarius serpentarius	Secretarybird	2021/8/15	4	0	-2.5521700	34.6320298	https://www.inaturalist.org/observations/94919311
254	Sagittarius serpentarius	Secretarybird	2013/7/28	11	0	-2.5490960	34.8061183	http://inaturalist.ca/observations/6435764
255	Sagittarius serpentarius	Secretarybird	2018/7/15	7	0	-2.5488256	34.8143659	https://www.inaturalist.org/observations/15223403
256	Sagittarius serpentarius	Secretarybird	2021/11/3	7	0	-2.5411199	34.7306342	https://www.inaturalist.org/observations/100128831
257	Sagittarius serpentarius	Secretarybird	1996/1/31	2	0	-2.5374537	34.8598490	https://www.inaturalist.org/observations/68358338
258	Sagittarius serpentarius	Secretarybird	2022/7/21	8	0	-2.5354195	34.7524737	https://www.inaturalist.org/observations/131796740
259	Sagittarius serpentarius	Secretarybird	2019/8/22	6	0	-2.5353769	34.9771596	https://www.inaturalist.org/observations/34071847
260	Sagittarius serpentarius	Secretarybird	2018/10/26	6	0	-2.5337298	34.9914558	https://www.inaturalist.org/observations/18053730
261	Sagittarius serpentarius	Secretarybird	2019/1/1	6	0	-2.5271332	34.6946829	https://www.inaturalist.org/observations/57927568
262	Sagittarius serpentarius	Secretarybird	2021/9/8	5	0	-2.5218692	34.9330301	https://www.inaturalist.org/observations/94358907
263	Sagittarius serpentarius	Secretarybird	2022/10/2	3	0	-2.5189454	34.9571306	https://www.inaturalist.org/observations/139811306
264	Sagittarius serpentarius	Secretarybird	2018/7/1	4	0	-2.5075753	35.0654605	https://www.inaturalist.org/observations/13930658
265	Sagittarius serpentarius	Secretarybird	2022/7/21	4	0	-2.5066276	34.9177158	https://www.inaturalist.org/observations/128136725
266	Sagittarius serpentarius	Secretarybird	2021/9/9	5	0	-2.5049620	34.8452794	https://www.inaturalist.org/observations/100428942
267	Sagittarius serpentarius	Secretarybird	2008/7/28	3	0	-2.5001567	34.8927986	https://www.inaturalist.org/observations/68408942
268	Sagittarius serpentarius	Secretarybird	2019/9/19	5	0	-2.4976495	34.8513423	https://www.inaturalist.org/observations/34006012
269	Sagittarius serpentarius	Secretarybird	2019/11/1	5	0	-2.4925616	35.1761202	https://www.inaturalist.org/observations/35456008
270	Sagittarius serpentarius	Secretarybird	2010/8/2	4	0	-2.4804536	34.8412257	https://www.inaturalist.org/observations/68535637
271	Sagittarius serpentarius	Secretarybird	2021/12/25	4	0	-2.4769190	34.9047727	https://www.inaturalist.org/observations/104564967
272	Sagittarius serpentarius	Secretarybird	2020/1/1	5	0	-2.4763748	34.9604479	https://www.inaturalist.org/observations/120954780
273	Sagittarius serpentarius	Secretarybird	2013/11/13	5	0	-2.4730941	34.9424285	https://www.inaturalist.org/observations/35665350
274	Sagittarius serpentarius	Secretarybird	2020/7/9	3	0	-2.4713594	34.6680027	https://www.inaturalist.org/observations/105375806
275	Sagittarius serpentarius	Secretarybird	2015/12/22	7	0	-2.4702500	38.4221012	https://www.inaturalist.org/observations/7643543

276	Sagittarius serpentarius	Secretarybird	2011/3/15	3	0	-2.4680388	34.9462312	https://www.inaturalist.org/observations/38770955
277	Sagittarius serpentarius	Secretarybird	2014/6/22	8	0	-2.4660207	34.7632445	https://www.inaturalist.org/observations/6620633
278	Sagittarius serpentarius	Secretarybird	2019/6/12	4	0	-2.4657824	34.5969462	https://www.inaturalist.org/observations/128057811
279	Sagittarius serpentarius	Secretarybird	2016/2/21	7	0	-2.4528554	34.8635270	https://www.inaturalist.org/observations/12519420
280	Sagittarius serpentarius	Secretarybird	2022/7/22	5	0	-2.4505477	34.9366253	https://www.inaturalist.org/observations/128725463
281	Sagittarius serpentarius	Secretarybird	2022/1/14	4	0	-2.4451152	34.8852549	https://www.inaturalist.org/observations/105443064
282	Sagittarius serpentarius	Secretarybird	2005/1/2	5	0	-2.4372683	34.9609331	https://www.inaturalist.org/observations/24432960
283	Sagittarius serpentarius	Secretarybird	2020/2/3	6	0	-2.4339603	34.7571432	https://www.inaturalist.org/observations/38385064
284	Sagittarius serpentarius	Secretarybird	2022/11/19	3	0	-2.4266817	34.8241597	https://www.inaturalist.org/observations/144163788
285	Sagittarius serpentarius	Secretarybird	2019/8/13	3	0	-2.4149575	35.0171374	https://www.inaturalist.org/observations/45890390
286	Sagittarius serpentarius	Secretarybird	1993/12/29	5	0	-2.4145113	34.9317555	https://www.inaturalist.org/observations/135833222
287	Sagittarius serpentarius	Secretarybird	2019/10/10	4	0	-2.4093762	34.8214924	https://www.inaturalist.org/observations/108170519
288	Sagittarius serpentarius	Secretarybird	2022/11/18	3	0	-2.4077222	34.8382231	https://www.inaturalist.org/observations/144344202
289	Sagittarius serpentarius	Secretarybird	2019/2/4	3	0	-2.4062529	34.9487026	https://www.inaturalist.org/observations/137617358
290	Sagittarius serpentarius	Secretarybird	2008/9/6	4	0	-2.3980366	34.9132973	https://www.inaturalist.org/observations/112628278
291	Sagittarius serpentarius	Secretarybird	2007/12/7	3	0	-2.3963598	34.8374115	https://www.inaturalist.org/observations/69758955
292	Sagittarius serpentarius	Secretarybird	2011/7/11	5	0	-2.3946690	34.8948900	https://www.inaturalist.org/observations/55129479
293	Sagittarius serpentarius	Secretarybird	2020/12/24	4	0	-2.3937953	34.7642780	https://www.inaturalist.org/observations/67791635
294	Sagittarius serpentarius	Secretarybird	2019/9/17	6	0	-2.3931391	34.5722285	https://www.inaturalist.org/observations/33465227
295	Sagittarius serpentarius	Secretarybird	2022/7/12	4	0	-2.3929366	34.9707298	https://www.inaturalist.org/observations/127278994
296	Sagittarius serpentarius	Secretarybird	2017/9/23	4	0	-2.3893936	34.9332146	https://www.inaturalist.org/observations/69624609
297	Sagittarius serpentarius	Secretarybird	2009/2/26	5	0	-2.3868252	34.9878664	https://www.inaturalist.org/observations/92408380
298	Sagittarius serpentarius	Secretarybird	2018/11/25	4	0	-2.3789041	34.9738928	https://www.inaturalist.org/observations/96480482

299	Sagittarius serpentarius	Secretarybird	2011/2/21	6	0	-2.3734183	34.4178035	https://www.inaturalist.org/observations/109229551
300	Sagittarius serpentarius	Secretarybird	2018/8/22	4	0	-2.3695055	34.6332983	https://www.inaturalist.org/observations/134307244
301	Sagittarius serpentarius	Secretarybird	2018/9/5	6	0	-2.3669546	34.9457726	https://www.inaturalist.org/observations/16698286
302	Sagittarius serpentarius	Secretarybird	2017/7/14	4	0	-2.3633325	34.8878423	https://www.inaturalist.org/observations/69122626
303	Sagittarius serpentarius	Secretarybird	2016/9/19	4	0	-2.3624738	34.9087313	https://www.inaturalist.org/observations/98199890
304	Sagittarius serpentarius	Secretarybird	2019/6/29	5	0	-2.3622092	34.9991000	https://www.inaturalist.org/observations/34869161
305	Sagittarius serpentarius	Secretarybird	2017/7/14	4	0	-2.3617751	34.9524124	https://www.inaturalist.org/observations/50949635
306	Sagittarius serpentarius	Secretarybird	2021/11/3	5	0	-2.3565106	34.9090577	https://www.inaturalist.org/observations/102130042
307	Sagittarius serpentarius	Secretarybird	2017/11/22	4	0	-2.3504484	34.7271406	https://www.inaturalist.org/observations/19388827
308	Sagittarius serpentarius	Secretarybird	2017/7/25	5	0	-2.3414190	34.8286669	https://www.inaturalist.org/observations/136913665
309	Sagittarius serpentarius	Secretarybird	2019/6/12	4	0	-2.3412376	34.9902511	https://www.inaturalist.org/observations/128060099
310	Sagittarius serpentarius	Secretarybird	2018/1/22	4	0	-2.3392631	34.7461291	https://www.inaturalist.org/observations/81490923
311	Sagittarius serpentarius	Secretarybird	2019/12/26	6	0	-2.3387242	34.9155800	https://www.inaturalist.org/observations/40657733
312	Sagittarius serpentarius	Secretarybird	2010/10/31	5	0	-2.3269659	34.8173033	https://www.inaturalist.org/observations/23049300
313	Sagittarius serpentarius	Secretarybird	2007/6/22	5	0	-2.3233922	34.8543916	https://www.inaturalist.org/observations/56786945
314	Sagittarius serpentarius	Secretarybird	2015/10/8	4	0	-2.3213022	34.8693419	https://www.inaturalist.org/observations/79592841
315	Sagittarius serpentarius	Secretarybird	2019/9/8	4	0	-2.3212098	34.9834096	https://www.inaturalist.org/observations/102681580
316	Sagittarius serpentarius	Secretarybird	2020/2/25	4	0	-2.3137517	34.8978499	https://www.inaturalist.org/observations/70440246
317	Sagittarius serpentarius	Secretarybird	2010/4/28	5	0	-2.3113852	34.6671841	https://www.inaturalist.org/observations/136003402
318	Sagittarius serpentarius	Secretarybird	2018/7/2	5	0	-2.3107549	34.4211672	https://www.inaturalist.org/observations/14782108
319	Sagittarius serpentarius	Secretarybird	2012/8/7	5	0	-2.3072956	34.9192972	https://www.inaturalist.org/observations/69941311
320	Sagittarius serpentarius	Secretarybird	2019/12/24	6	0	-2.3050717	34.8255838	https://www.inaturalist.org/observations/40657734
321	Sagittarius serpentarius	Secretarybird	2019/9/7	5	0	-2.3044817	34.8636226	https://www.inaturalist.org/observations/39509559

322	Sagittarius serpentarius	Secretarybird	2016/1/13	5	0	-2.2973077	34.9616923	https://www.inaturalist.org/observations/9867734
323	Sagittarius serpentarius	Secretarybird	2015/8/3	5	0	-2.2962794	34.9385838	https://www.inaturalist.org/observations/35452435
324	Sagittarius serpentarius	Secretarybird	2008/9/6	5	0	-2.2816835	34.9437348	https://www.inaturalist.org/observations/112628591
325	Sagittarius serpentarius	Secretarybird	1982/10/1	7	0	-2.2785813	34.6086085	http://www.inaturalist.org/observations/585372
326	Sagittarius serpentarius	Secretarybird	2016/10/18	6	0	-2.2783954	34.8344346	https://www.inaturalist.org/observations/40045461
327	Sagittarius serpentarius	Secretarybird	2018/4/7	6	0	-2.2774956	34.6064238	https://www.inaturalist.org/observations/107803459
328	Sagittarius serpentarius	Secretarybird	2021/9/9	6	0	-2.2740570	34.6512240	https://www.inaturalist.org/observations/100858353
329	Sagittarius serpentarius	Secretarybird	2019/10/1	4	0	-2.2659793	34.9312630	https://www.inaturalist.org/observations/35656787
330	Sagittarius serpentarius	Secretarybird	2018/8/22	4	0	-2.2625689	34.8930865	https://www.inaturalist.org/observations/37891471
331	Sagittarius serpentarius	Secretarybird	2017/5/28	6	0	-2.2540967	34.7526666	https://www.inaturalist.org/observations/106959937
332	Sagittarius serpentarius	Secretarybird	2017/11/6	5	0	-2.2534205	34.4070100	https://www.inaturalist.org/observations/19347541
333	Sagittarius serpentarius	Secretarybird	2014/11/14	4	0	-2.2441481	34.8140049	https://www.inaturalist.org/observations/20023620
334	Sagittarius serpentarius	Secretarybird	2022/1/20	4	0	-2.2423280	37.0728681	https://www.inaturalist.org/observations/105441452
335	Sagittarius serpentarius	Secretarybird	2016/8/8	4	0	-2.2344365	34.8749997	https://www.inaturalist.org/observations/22102855
336	Sagittarius serpentarius	Secretarybird	2022/1/13	5	0	-2.2202920	34.6368465	https://www.inaturalist.org/observations/105312229
337	Sagittarius serpentarius	Secretarybird	2009/2/13	6	0	-2.2177302	34.9634595	https://www.inaturalist.org/observations/59799416
338	Sagittarius serpentarius	Secretarybird	2011/11/24	5	0	-2.2167101	34.9059167	https://www.inaturalist.org/observations/21512035
339	Sagittarius serpentarius	Secretarybird	2019/6/13	4	0	-2.2031634	34.8214177	https://www.inaturalist.org/observations/128062635
340	Sagittarius serpentarius	Secretarybird	2018/1/22	4	0	-2.2002902	34.7889883	https://www.inaturalist.org/observations/81375048
341	Sagittarius serpentarius	Secretarybird	2012/4/19	4	0	-2.1528862	35.0609502	https://www.inaturalist.org/observations/85742144
342	Sagittarius serpentarius	Secretarybird	2009/9/29	9	0	-2.1402263	34.6354387	http://www.inaturalist.org/observations/871121
343	Sagittarius serpentarius	Secretarybird	2007/7/14	6	0	-2.1380086	34.9190908	https://www.inaturalist.org/observations/57898667
344	Sagittarius serpentarius	Secretarybird	2017/9/19	6	0	-2.0956670	35.1802895	https://www.inaturalist.org/observations/33292849

345	Sagittarius serpentarius	Secretarybird	2018/6/25	4	0	-2.0927366	34.6321706	https://www.inaturalist.org/observations/15092474
346	Sagittarius serpentarius	Secretarybird	1998/11/10	7	0	-2.0901537	35.1162059	http://www.inaturalist.org/observations/4397774
347	Sagittarius serpentarius	Secretarybird	2007/9/24	6	0	-2.0834189	34.9023214	https://www.inaturalist.org/observations/56284679
348	Sagittarius serpentarius	Secretarybird	2017/9/19	6	0	-2.0625183	35.0422347	https://www.inaturalist.org/observations/33292848
349	Sagittarius serpentarius	Secretarybird	2022/9/3	5	0	-2.0508973	35.4492543	https://www.inaturalist.org/observations/134410564
350	Sagittarius serpentarius	Secretarybird	2007/7/14	5	0	-2.0107121	34.9068867	https://www.inaturalist.org/observations/57898668
351	Sagittarius serpentarius	Secretarybird	2012/9/24	6	0	-2.0089840	35.5901277	http://www.inaturalist.org/observations/3121689
352	Sagittarius serpentarius	Secretarybird	2019/6/30	5	0	-2.0085062	34.3156340	https://www.inaturalist.org/observations/27926922
353	Sagittarius serpentarius	Secretarybird	2016/9/11	5	0	-2.0016592	38.4402838	https://www.inaturalist.org/observations/44775035
354	Sagittarius serpentarius	Secretarybird	2009/11/23	4	0	-1.9943024	34.7661685	https://www.inaturalist.org/observations/142163690
355	Sagittarius serpentarius	Secretarybird	2015/1/4	5	0	-1.9884124	34.6617758	https://www.inaturalist.org/observations/117290570
356	Sagittarius serpentarius	Secretarybird	2013/6/28	6	0	-1.9808450	34.6610262	https://www.inaturalist.org/observations/110598989
357	Sagittarius serpentarius	Secretarybird	2011/11/8	4	0	-1.9694730	34.8893158	https://www.inaturalist.org/observations/28402615
358	Sagittarius serpentarius	Secretarybird	2016/5/25	6	0	-1.9562954	34.7740779	https://www.inaturalist.org/observations/131340305
359	Sagittarius serpentarius	Secretarybird	2016/9/11	5	0	-1.9514916	35.0410238	https://www.inaturalist.org/observations/9108688
360	Sagittarius serpentarius	Secretarybird	2018/6/10	4	0	-1.9498328	34.7195541	https://www.inaturalist.org/observations/44392003
361	Sagittarius serpentarius	Secretarybird	2019/12/17	5	0	-1.9428441	34.6279569	https://www.inaturalist.org/observations/36932143
362	Sagittarius serpentarius	Secretarybird	2019/3/25	6	0	-1.9346326	34.6381874	https://www.inaturalist.org/observations/66970606
363	Sagittarius serpentarius	Secretarybird	2018/8/26	5	0	-1.9178953	34.6323382	https://www.inaturalist.org/observations/52023174
364	Sagittarius serpentarius	Secretarybird	2019/2/28	5	0	-1.9066571	34.7239981	https://www.inaturalist.org/observations/57950406
365	Sagittarius serpentarius	Secretarybird	2022/8/7	5	0	-1.9054579	35.0222236	https://www.inaturalist.org/observations/133458357
366	Sagittarius serpentarius	Secretarybird	2013/6/28	5	0	-1.8978396	34.6429777	https://www.inaturalist.org/observations/110597972
367	Sagittarius serpentarius	Secretarybird	2015/7/21	7	0	-1.8963046	34.8041566	http://www.inaturalist.org/observations/3054113

368	Sagittarius serpentarius	Secretarybird	2016/12/28	5	0	-1.8953977	34.7535554	https://www.inaturalist.org/observations/51996305
369	Sagittarius serpentarius	Secretarybird	2017/10/7	5	0	-1.8381752	37.2488066	https://www.inaturalist.org/observations/21766521
370	Sagittarius serpentarius	Secretarybird	2009/11/22	6	0	-1.8178778	34.7994925	https://www.inaturalist.org/observations/142142922
371	Sagittarius serpentarius	Secretarybird	2018/7/19	7	0	-1.7951412	35.3380829	https://www.inaturalist.org/observations/15255484
372	Sagittarius serpentarius	Secretarybird	2019/8/20	7	0	-1.7699452	34.9567112	https://www.inaturalist.org/observations/33449552
373	Sagittarius serpentarius	Secretarybird	2022/9/9	5	0	-1.7689333	34.8800116	https://www.inaturalist.org/observations/135350469
374	Sagittarius serpentarius	Secretarybird	2019/8/11	6	0	-1.7554575	35.3843330	https://www.inaturalist.org/observations/32118097
375	Sagittarius serpentarius	Secretarybird	2016/9/24	5	0	-1.7547412	37.0189039	http://www.inaturalist.org/observations/4197185
376	Sagittarius serpentarius	Secretarybird	1988/8/19	6	0	-1.7270277	35.3250445	https://www.inaturalist.org/observations/42769655
377	Sagittarius serpentarius	Secretarybird	2007/10/5	5	0	-1.7007018	35.3448356	https://www.inaturalist.org/observations/19483942
378	Sagittarius serpentarius	Secretarybird	2022/7/11	3	0	-1.6870865	35.2254973	https://www.inaturalist.org/observations/125748021
379	Sagittarius serpentarius	Secretarybird	2019/8/14	5	0	-1.6710414	35.5210973	https://www.inaturalist.org/observations/32185819
380	Sagittarius serpentarius	Secretarybird	2020/12/12	4	0	-1.6527294	35.3683317	https://www.inaturalist.org/observations/79046787
381	Sagittarius serpentarius	Secretarybird	2010/10/20	4	0	-1.6470499	35.3182607	https://www.inaturalist.org/observations/131629444
382	Sagittarius serpentarius	Secretarybird	2022/10/22	2	0	-1.6423119	35.2071977	https://www.inaturalist.org/observations/139612716
383	Sagittarius serpentarius	Secretarybird	2018/9/30	5	0	-1.6381414	35.3044397	https://www.inaturalist.org/observations/17130486
384	Sagittarius serpentarius	Secretarybird	2017/9/17	6	0	-1.6132753	35.1657815	https://www.inaturalist.org/observations/33292847
385	Sagittarius serpentarius	Secretarybird	2018/8/25	5	0	-1.6028075	35.3150732	https://www.inaturalist.org/observations/19234226
386	Sagittarius serpentarius	Secretarybird	2015/5/26	4	0	-1.5999375	35.0254756	https://www.inaturalist.org/observations/22945716
387	Sagittarius serpentarius	Secretarybird	2019/3/8	5	0	-1.5981142	35.1774650	https://www.inaturalist.org/observations/22164409
388	Sagittarius serpentarius	Secretarybird	2022/9/18	5	0	-1.5927897	35.3966170	https://www.inaturalist.org/observations/135552497
389	Sagittarius serpentarius	Secretarybird	2007/3/6	4	0	-1.5895403	35.1027932	https://www.inaturalist.org/observations/103099658
390	Sagittarius serpentarius	Secretarybird	2018/6/25	6	0	-1.5892521	35.1019790	https://www.inaturalist.org/observations/64348067

391	Sagittarius serpentarius	Secretarybird	2022/6/20	4	0	-1.5882626	35.3558234	https://www.inaturalist.org/observations/144607671
392	Sagittarius serpentarius	Secretarybird	2021/12/23	3	0	-1.5881290	35.0798247	https://www.inaturalist.org/observations/134839399
393	Sagittarius serpentarius	Secretarybird	2008/9/30	6	0	-1.5865095	35.1948149	https://www.inaturalist.org/observations/38096412
394	Sagittarius serpentarius	Secretarybird	2011/3/1	3	0	-1.5812340	35.2145328	https://www.inaturalist.org/observations/122170266
395	Sagittarius serpentarius	Secretarybird	2007/10/7	4	0	-1.5810741	35.2925826	https://www.inaturalist.org/observations/19526433
396	Sagittarius serpentarius	Secretarybird	2022/9/18	4	0	-1.5783412	34.9656640	https://www.inaturalist.org/observations/135551952
397	Sagittarius serpentarius	Secretarybird	2017/4/21	4	0	-1.5778817	35.0572423	https://www.inaturalist.org/observations/36198219
398	Sagittarius serpentarius	Secretarybird	2012/9/30	5	0	-1.5773035	35.3438114	https://www.inaturalist.org/observations/101697093
399	Sagittarius serpentarius	Secretarybird	2018/7/16	4	0	-1.5729416	35.1169189	https://www.inaturalist.org/observations/38682951
400	Sagittarius serpentarius	Secretarybird	2019/12/26	5	0	-1.5692667	35.0994872	https://www.inaturalist.org/observations/40210395
401	Sagittarius serpentarius	Secretarybird	2022/7/10	4	0	-1.5648878	35.0980051	https://www.inaturalist.org/observations/128125604
402	Sagittarius serpentarius	Secretarybird	2014/10/15	4	0	-1.5630900	35.1813811	https://www.inaturalist.org/observations/66406699
403	Sagittarius serpentarius	Secretarybird	2008/7/21	11	0	-1.5570656	35.3009109	http://www.inaturalist.org/observations/1834115
404	Sagittarius serpentarius	Secretarybird	2010/6/13	5	0	-1.5568382	35.0981263	https://www.inaturalist.org/observations/107889880
405	Sagittarius serpentarius	Secretarybird	2010/5/17	5	0	-1.5557495	34.9803156	https://www.inaturalist.org/observations/46192325
406	Sagittarius serpentarius	Secretarybird	2016/12/12	4	0	-1.5517003	35.1235052	https://www.inaturalist.org/observations/52418511
407	Sagittarius serpentarius	Secretarybird	2018/2/12	6	0	-1.5500762	35.3177336	https://www.inaturalist.org/observations/9844753
408	Sagittarius serpentarius	Secretarybird	2017/9/5	7	0	-1.5455445	35.0759167	https://www.inaturalist.org/observations/8039116
409	Sagittarius serpentarius	Secretarybird	2019/8/5	6	0	-1.5453528	35.3090528	https://www.inaturalist.org/observations/34214693
410	Sagittarius serpentarius	Secretarybird	2018/3/4	5	0	-1.5422891	35.1492013	https://www.inaturalist.org/observations/54149035
411	Sagittarius serpentarius	Secretarybird	2020/2/27	5	0	-1.5416391	35.3251158	https://www.inaturalist.org/observations/137753006
412	Sagittarius serpentarius	Secretarybird	2018/8/25	4	0	-1.5384074	35.0209429	https://www.inaturalist.org/observations/64458504
413	Sagittarius serpentarius	Secretarybird	2008/7/18	6	0	-1.5376544	35.1491749	https://www.inaturalist.org/observations/61636993

414	Sagittarius serpentarius	Secretarybird	2022/9/30	2	0	-1.5376192	35.2480356	https://www.inaturalist.org/observations/139850446
415	Sagittarius serpentarius	Secretarybird	2020/6/5	5	0	-1.5352191	36.9838344	https://www.inaturalist.org/observations/48626759
416	Sagittarius serpentarius	Secretarybird	2018/4/15	3	0	-1.5345073	35.0261115	https://www.inaturalist.org/observations/68722072
417	Sagittarius serpentarius	Secretarybird	2013/8/24	3	0	-1.5320070	35.0821996	https://www.inaturalist.org/observations/76941724
418	Sagittarius serpentarius	Secretarybird	2015/2/18	7	0	-1.5313533	35.1967252	https://www.inaturalist.org/observations/100392950
419	Sagittarius serpentarius	Secretarybird	2006/10/8	5	0	-1.5307427	34.8807309	https://www.inaturalist.org/observations/114685456
420	Sagittarius serpentarius	Secretarybird	2009/8/25	7	0	-1.5307231	35.1614427	https://www.inaturalist.org/observations/27927439
421	Sagittarius serpentarius	Secretarybird	2020/7/23	5	0	-1.5298424	36.8801943	https://www.inaturalist.org/observations/54301688
422	Sagittarius serpentarius	Secretarybird	2019/7/21	4	0	-1.5294307	35.2950732	https://www.inaturalist.org/observations/75627167
423	Sagittarius serpentarius	Secretarybird	2008/4/8	4	0	-1.5289921	34.8879615	https://www.inaturalist.org/observations/39232234
424	Sagittarius serpentarius	Secretarybird	2022/5/23	6	0	-1.5285556	35.1229243	https://www.inaturalist.org/observations/126529870
425	Sagittarius serpentarius	Secretarybird	2022/3/27	6	0	-1.5277087	35.3225569	https://www.inaturalist.org/observations/111654972
426	Sagittarius serpentarius	Secretarybird	2012/5/22	4	0	-1.5275973	35.2344473	https://www.inaturalist.org/observations/101339630
427	Sagittarius serpentarius	Secretarybird	2015/8/17	6	0	-1.5229557	34.8318741	https://www.inaturalist.org/observations/36693129
428	Sagittarius serpentarius	Secretarybird	2018/1/14	4	0	-1.5218244	35.0649649	https://www.inaturalist.org/observations/38145949
429	Sagittarius serpentarius	Secretarybird	2014/7/20	10	0	-1.5158826	35.0224376	http://www.inaturalist.org/observations/1500334
430	Sagittarius serpentarius	Secretarybird	2019/8/17	5	0	-1.5103477	35.3239645	https://www.inaturalist.org/observations/136516114
431	Sagittarius serpentarius	Secretarybird	2017/7/8	9	0	-1.5101322	35.0778966	https://www.inaturalist.org/observations/7261147
432	Sagittarius serpentarius	Secretarybird	2019/12/24	4	0	-1.5046232	35.1897734	https://www.inaturalist.org/observations/99077766
433	Sagittarius serpentarius	Secretarybird	2014/10/15	4	0	-1.5046202	35.0115030	https://www.inaturalist.org/observations/66406700
434	Sagittarius serpentarius	Secretarybird	2019/9/6	7	0	-1.4958438	35.1758184	https://www.inaturalist.org/observations/32424437
435	Sagittarius serpentarius	Secretarybird	2021/8/1	3	0	-1.4952314	35.0283579	https://www.inaturalist.org/observations/125316853
436	Sagittarius serpentarius	Secretarybird	2022/8/8	4	0	-1.4935173	35.1006549	https://www.inaturalist.org/observations/141738605

437	Sagittarius serpentarius	Secretarybird	2020/3/5	5	0	-1.4932585	35.1833950	https://www.inaturalist.org/observations/40099854
438	Sagittarius serpentarius	Secretarybird	2011/8/19	6	0	-1.4899859	35.0809752	https://www.inaturalist.org/observations/41187658
439	Sagittarius serpentarius	Secretarybird	2022/9/29	3	0	-1.4893468	35.2291963	https://www.inaturalist.org/observations/139783191
440	Sagittarius serpentarius	Secretarybird	2019/8/8	6	0	-1.4875398	35.1111956	https://www.inaturalist.org/observations/32197852
441	Sagittarius serpentarius	Secretarybird	2016/8/30	4	0	-1.4861970	35.3108462	https://www.inaturalist.org/observations/106110194
442	Sagittarius serpentarius	Secretarybird	2014/8/28	7	0	-1.4817342	35.0972444	http://www.inaturalist.org/observations/2748343
443	Sagittarius serpentarius	Secretarybird	2021/9/6	4	0	-1.4784313	34.9823221	https://www.inaturalist.org/observations/98122946
444	Sagittarius serpentarius	Secretarybird	2019/5/22	5	0	-1.4776923	35.2783386	https://www.inaturalist.org/observations/25571100
445	Sagittarius serpentarius	Secretarybird	2019/8/8	5	0	-1.4767673	35.0354700	https://www.inaturalist.org/observations/32248319
446	Sagittarius serpentarius	Secretarybird	2009/12/9	5	0	-1.4742685	34.9049552	https://www.inaturalist.org/observations/18040087
447	Sagittarius serpentarius	Secretarybird	2004/8/7	3	0	-1.4738953	35.1870615	https://www.inaturalist.org/observations/123243155
448	Sagittarius serpentarius	Secretarybird	2021/9/2	5	0	-1.4731815	35.2646553	https://www.inaturalist.org/observations/94108748
449	Sagittarius serpentarius	Secretarybird	2022/6/22	4	0	-1.4730029	35.1784463	https://www.inaturalist.org/observations/128013557
450	Sagittarius serpentarius	Secretarybird	2012/7/15	5	0	-1.4714900	34.8682302	https://www.inaturalist.org/observations/118761557
451	Sagittarius serpentarius	Secretarybird	2013/8/20	2	0	-1.4687460	35.0805535	https://www.inaturalist.org/observations/68263290
452	Sagittarius serpentarius	Secretarybird	2022/9/30	3	0	-1.4674076	35.1043608	https://www.inaturalist.org/observations/139850491
453	Sagittarius serpentarius	Secretarybird	2014/8/21	4	0	-1.4650944	35.0461422	https://www.inaturalist.org/observations/80984777
454	Sagittarius serpentarius	Secretarybird	2022/3/31	3	0	-1.4650841	36.9443647	https://www.inaturalist.org/observations/110270379
455	Sagittarius serpentarius	Secretarybird	2019/8/29	7	0	-1.4626834	35.0493411	https://www.inaturalist.org/observations/43172076
456	Sagittarius serpentarius	Secretarybird	2022/7/24	5	0	-1.4591744	35.0700226	https://www.inaturalist.org/observations/128916425
457	Sagittarius serpentarius	Secretarybird	1987/10/4	4	0	-1.4569053	35.3545177	https://www.inaturalist.org/observations/104431449
458	Sagittarius serpentarius	Secretarybird	2022/10/5	0	0	-1.4550131	35.2407487	https://www.inaturalist.org/observations/137641248
459	Sagittarius serpentarius	Secretarybird	2022/6/28	5	0	-1.4541801	35.3721680	https://www.inaturalist.org/observations/125303307

460	Sagittarius serpentarius	Secretarybird	2008/7/14	7	0	-1.4520841	35.0836614	https://www.inaturalist.org/observations/61636973
461	Sagittarius serpentarius	Secretarybird	2018/2/13	6	0	-1.4491101	35.1506290	https://www.inaturalist.org/observations/23027016
462	Sagittarius serpentarius	Secretarybird	2015/2/24	5	0	-1.4482810	35.1914988	https://www.inaturalist.org/observations/106812635
463	Sagittarius serpentarius	Secretarybird	2005/1/24	6	0	-1.4454774	35.1789028	https://www.inaturalist.org/observations/35669613
464	Sagittarius serpentarius	Secretarybird	2022/5/23	5	0	-1.4436143	35.1765204	https://www.inaturalist.org/observations/130960269
465	Sagittarius serpentarius	Secretarybird	2019/9/20	4	0	-1.4435619	35.0218556	https://www.inaturalist.org/observations/66190146
466	Sagittarius serpentarius	Secretarybird	2018/10/2	5	0	-1.4389642	35.1339387	https://www.inaturalist.org/observations/17917390
467	Sagittarius serpentarius	Secretarybird	2014/7/9	4	0	-1.4386231	34.8160589	https://www.inaturalist.org/observations/20841814
468	Sagittarius serpentarius	Secretarybird	2013/10/30	9	0	-1.4288307	35.1089210	http://www.inaturalist.org/observations/863751
469	Sagittarius serpentarius	Secretarybird	2019/4/28	4	0	-1.4256444	36.8661391	https://www.inaturalist.org/observations/23586611
470	Sagittarius serpentarius	Secretarybird	2021/12/18	5	0	-1.4251364	35.1929578	https://www.inaturalist.org/observations/132928773
471	Sagittarius serpentarius	Secretarybird	2022/12/7	2	0	-1.4232230	37.1940733	https://www.inaturalist.org/observations/144354510
472	Sagittarius serpentarius	Secretarybird	2018/11/25	6	0	-1.4231863	35.0598388	https://www.inaturalist.org/observations/18737143
473	Sagittarius serpentarius	Secretarybird	2021/8/21	3	0	-1.4203622	35.0437175	https://www.inaturalist.org/observations/97440598
474	Sagittarius serpentarius	Secretarybird	2018/12/15	5	0	-1.4174767	34.9339307	https://www.inaturalist.org/observations/23117775
475	Sagittarius serpentarius	Secretarybird	2021/9/17	4	0	-1.4171288	35.0126778	https://www.inaturalist.org/observations/95132497
476	Sagittarius serpentarius	Secretarybird	2010/5/18	5	0	-1.4142440	34.8671355	https://www.inaturalist.org/observations/65196844
477	Sagittarius serpentarius	Secretarybird	2022/9/28	6	0	-1.4133581	35.0857858	https://www.inaturalist.org/observations/141554298
478	Sagittarius serpentarius	Secretarybird	2019/6/3	5	0	-1.4129504	35.2317847	https://www.inaturalist.org/observations/52456229
479	Sagittarius serpentarius	Secretarybird	2021/12/31	2	0	-1.4121152	35.2541695	https://www.inaturalist.org/observations/104029698
480	Sagittarius serpentarius	Secretarybird	2022/6/29	3	0	-1.4111037	35.1328469	https://www.inaturalist.org/observations/133513435
481	Sagittarius serpentarius	Secretarybird	2018/7/21	5	0	-1.4096014	34.9911006	https://www.inaturalist.org/observations/53797641
482	Sagittarius serpentarius	Secretarybird	2015/12/20	6	0	-1.4015150	34.8535931	https://www.inaturalist.org/observations/9622730

483	Sagittarius serpentarius	Secretarybird	2008/1/5	2	0	-1.3936331	36.9683439	https://www.inaturalist.org/observations/145243119
484	Sagittarius serpentarius	Secretarybird	2017/7/31	6	0	-1.3884582	35.1302351	https://www.inaturalist.org/observations/62028245
485	Sagittarius serpentarius	Secretarybird	2019/7/16	3	0	-1.3883437	35.2496627	https://www.inaturalist.org/observations/29075602
486	Sagittarius serpentarius	Secretarybird	2021/11/13	4	0	-1.3871434	35.2000497	https://www.inaturalist.org/observations/104031209
487	Sagittarius serpentarius	Secretarybird	2017/9/8	7	0	-1.3846853	36.8805613	https://www.inaturalist.org/observations/8088104
488	Sagittarius serpentarius	Secretarybird	2018/7/16	4	0	-1.3844485	34.8912603	https://www.inaturalist.org/observations/46652750
489	Sagittarius serpentarius	Secretarybird	2018/5/31	4	0	-1.3809082	35.1817491	https://www.inaturalist.org/observations/47914625
490	Sagittarius serpentarius	Secretarybird	2019/3/12	5	0	-1.3736878	35.0275743	https://www.inaturalist.org/observations/42292851
491	Sagittarius serpentarius	Secretarybird	2017/11/5	4	0	-1.3725623	34.8979762	https://www.inaturalist.org/observations/19312077
492	Sagittarius serpentarius	Secretarybird	2021/6/25	4	0	-1.3724905	34.8677497	https://www.inaturalist.org/observations/86841834
493	Sagittarius serpentarius	Secretarybird	2019/1/27	5	0	-1.3724122	36.9383793	https://www.inaturalist.org/observations/110170638
494	Sagittarius serpentarius	Secretarybird	2018/7/13	6	0	-1.3718528	36.8196346	https://www.inaturalist.org/observations/38512404
495	Sagittarius serpentarius	Secretarybird	2019/11/9	5	0	-1.3710644	36.6419767	https://www.inaturalist.org/observations/35840130
496	Sagittarius serpentarius	Secretarybird	2022/6/24	5	0	-1.3704223	36.9623914	https://www.inaturalist.org/observations/125331307
497	Sagittarius serpentarius	Secretarybird	2018/12/30	5	0	-1.3674714	36.9982961	https://www.inaturalist.org/observations/108948641
498	Sagittarius serpentarius	Secretarybird	2022/6/22	5	0	-1.3664362	35.0293680	https://www.inaturalist.org/observations/128016511
499	Sagittarius serpentarius	Secretarybird	2009/12/21	8	0	-1.3646613	36.6443458	http://www.inaturalist.org/observations/28284
500	Sagittarius serpentarius	Secretarybird	2017/7/31	6	0	-1.3645577	35.1456555	https://www.inaturalist.org/observations/62028244
501	Sagittarius serpentarius	Secretarybird	2018/4/15	4	0	-1.3645122	36.9819231	https://www.inaturalist.org/observations/14285666
502	Sagittarius serpentarius	Secretarybird	2015/12/23	5	0	-1.3634560	36.8928306	https://www.inaturalist.org/observations/9838439
503	Sagittarius serpentarius	Secretarybird	2022/8/7	5	0	-1.3612579	36.9338041	https://www.inaturalist.org/observations/141738540
504	Sagittarius serpentarius	Secretarybird	1994/5/21	6	0	-1.3524682	36.7045634	https://www.inaturalist.org/observations/54813687
505	Sagittarius serpentarius	Secretarybird	2019/5/17	5	0	-1.3484361	35.2965224	https://www.inaturalist.org/observations/38414007

506	Sagittarius serpentarius	Secretarybird	2018/1/7	5	0	-1.3412852	34.8458995	https://www.inaturalist.org/observations/9767034
507	Sagittarius serpentarius	Secretarybird	2015/11/27	7	0	-1.3382753	36.9740822	https://www.inaturalist.org/observations/38546792
508	Sagittarius serpentarius	Secretarybird	2016/5/3	3	0	-1.3375666	36.8165712	https://www.inaturalist.org/observations/87156182
509	Sagittarius serpentarius	Secretarybird	2022/2/19	4	0	-1.3355325	36.8824321	https://www.inaturalist.org/observations/110371635
510	Sagittarius serpentarius	Secretarybird	2022/2/4	3	0	-1.3352637	36.9396100	https://www.inaturalist.org/observations/144481650
511	Sagittarius serpentarius	Secretarybird	2020/1/31	4	0	-1.3284925	35.2469622	https://www.inaturalist.org/observations/130008366
512	Sagittarius serpentarius	Secretarybird	2021/1/15	6	0	-1.3274822	34.8836013	https://www.inaturalist.org/observations/120819899
513	Sagittarius serpentarius	Secretarybird	2022/6/21	4	0	-1.3229822	35.1480317	https://www.inaturalist.org/observations/127996189
514	Sagittarius serpentarius	Secretarybird	2021/7/30	4	0	-1.3218798	36.8854830	https://www.inaturalist.org/observations/101935680
515	Sagittarius serpentarius	Secretarybird	2019/7/31	4	0	-1.3203771	36.8400718	https://www.inaturalist.org/observations/47011708
516	Sagittarius serpentarius	Secretarybird	2021/8/30	5	0	-1.3161544	34.9835366	https://www.inaturalist.org/observations/106565342
517	Sagittarius serpentarius	Secretarybird	2013/10/24	5	0	-1.3148321	36.8184047	https://www.inaturalist.org/observations/9790160
518	Sagittarius serpentarius	Secretarybird	2019/1/2	7	0	-1.3147447	36.9515354	https://www.inaturalist.org/observations/67852776
519	Sagittarius serpentarius	Secretarybird	2022/12/12	5	0	-1.3143476	35.2092838	https://www.inaturalist.org/observations/144649069
520	Sagittarius serpentarius	Secretarybird	2020/1/5	3	0	-1.3139182	34.8101863	https://www.inaturalist.org/observations/121086618
521	Sagittarius serpentarius	Secretarybird	1994/10/5	5	0	-1.3101582	35.7469322	https://www.inaturalist.org/observations/54888479
522	Sagittarius serpentarius	Secretarybird	2009/6/20	4	0	-1.3099523	36.6615604	https://www.inaturalist.org/observations/36298031
523	Sagittarius serpentarius	Secretarybird	2017/8/1	6	0	-1.3086339	35.1338930	https://www.inaturalist.org/observations/62029150
524	Sagittarius serpentarius	Secretarybird	2022/6/22	5	0	-1.3083063	36.9117718	https://www.inaturalist.org/observations/131931416
525	Sagittarius serpentarius	Secretarybird	2009/9/24	6	0	-1.3081595	34.9213414	https://www.inaturalist.org/observations/45659347
526	Sagittarius serpentarius	Secretarybird	2021/12/21	4	0	-1.3061823	34.9261307	https://www.inaturalist.org/observations/112650979
527	Sagittarius serpentarius	Secretarybird	2021/8/4	5	0	-1.3036464	35.0227121	https://www.inaturalist.org/observations/93626189
528	Sagittarius serpentarius	Secretarybird	2019/3/22	6	0	-1.3011362	35.0278106	https://www.inaturalist.org/observations/101346674

529	Sagittarius serpentarius	Secretarybird	2021/12/20	4	0	-1.2891340	34.9751018	https://www.inaturalist.org/observations/112650851
530	Sagittarius serpentarius	Secretarybird	2015/11/7	7	0	-1.2822935	36.9445736	http://www.inaturalist.org/observations/2357389
531	Sagittarius serpentarius	Secretarybird	2017/1/30	4	0	-1.2808836	35.3644724	https://www.inaturalist.org/observations/17555665
532	Sagittarius serpentarius	Secretarybird	2014/8/29	7	0	-1.2796929	34.8594911	https://www.inaturalist.org/observations/63837325
533	Sagittarius serpentarius	Secretarybird	2017/9/6	8	0	-1.2766414	35.1903868	https://www.inaturalist.org/observations/8066091
534	Sagittarius serpentarius	Secretarybird	2017/1/27	5	0	-1.2745098	36.8319713	https://www.inaturalist.org/observations/17553443
535	Sagittarius serpentarius	Secretarybird	2019/4/14	6	0	-1.2731935	36.9611391	https://www.inaturalist.org/observations/110188193
536	Sagittarius serpentarius	Secretarybird	2022/6/27	5	0	-1.2711986	36.9673620	https://www.inaturalist.org/observations/125304250
537	Sagittarius serpentarius	Secretarybird	2017/7/30	5	0	-1.2703014	35.0615933	https://www.inaturalist.org/observations/99766455
538	Sagittarius serpentarius	Secretarybird	2014/8/7	5	0	-1.2694246	35.0272745	https://www.inaturalist.org/observations/106517996
539	Sagittarius serpentarius	Secretarybird	2020/8/21	6	0	-1.2685561	36.9105263	https://www.inaturalist.org/observations/57305800
540	Sagittarius serpentarius	Secretarybird	2021/7/12	4	0	-1.2685558	36.9890377	https://www.inaturalist.org/observations/86699037
541	Sagittarius serpentarius	Secretarybird	2019/3/14	7	0	-1.2685465	35.0256830	https://www.inaturalist.org/observations/33741751
542	Sagittarius serpentarius	Secretarybird	2017/9/8	5	0	-1.2681860	35.3100086	https://www.inaturalist.org/observations/41921173
543	Sagittarius serpentarius	Secretarybird	2015/4/9	9	0	-1.2670848	36.9297579	http://www.inaturalist.org/observations/1445353
544	Sagittarius serpentarius	Secretarybird	2018/9/7	4	0	-1.2628461	35.1588388	https://www.inaturalist.org/observations/16354562
545	Sagittarius serpentarius	Secretarybird	2016/10/14	6	0	-1.2580026	36.8858239	http://www.inaturalist.org/observations/4657826
546	Sagittarius serpentarius	Secretarybird	2018/7/13	6	0	-1.2536226	36.9909198	https://www.inaturalist.org/observations/46646836
547	Sagittarius serpentarius	Secretarybird	2022/12/25	1	0	-1.2526900	36.8093345	https://www.inaturalist.org/observations/145035346
548	Sagittarius serpentarius	Secretarybird	2015/7/6	5	0	-1.2514068	34.9534335	https://www.inaturalist.org/observations/40657894
549	Sagittarius serpentarius	Secretarybird	2015/4/2	8	0	-1.2449593	35.1765695	http://www.inaturalist.org/observations/1382569
550	Sagittarius serpentarius	Secretarybird	2019/3/5	6	0	-1.2355839	35.0505784	https://www.inaturalist.org/observations/42294385
551	Sagittarius serpentarius	Secretarybird	2019/7/30	5	0	-1.2351937	36.6604843	https://www.inaturalist.org/observations/49182323

552	Sagittarius serpentarius	Secretarybird	2021/7/20	4	0	-1.2292556	36.9968873	https://www.inaturalist.org/observations/91859745
553	Sagittarius serpentarius	Secretarybird	2019/2/23	4	0	-1.2249367	35.3883975	https://www.inaturalist.org/observations/71975005
554	Sagittarius serpentarius	Secretarybird	2021/3/26	5	0	-1.2215665	35.1055949	https://www.inaturalist.org/observations/104698526
555	Sagittarius serpentarius	Secretarybird	2019/10/10	5	0	-1.2186567	36.6525944	https://www.inaturalist.org/observations/34256128
556	Sagittarius serpentarius	Secretarybird	2019/8/3	5	0	-1.2093576	34.9464075	https://www.inaturalist.org/observations/46799514
557	Sagittarius serpentarius	Secretarybird	2015/8/1	6	0	-1.2042573	36.6760611	https://www.inaturalist.org/observations/60781307
558	Sagittarius serpentarius	Secretarybird	2017/11/24	4	0	-1.2024166	36.8898283	https://www.inaturalist.org/observations/27270542
559	Sagittarius serpentarius	Secretarybird	2013/8/2	7	0	-1.2012669	34.9177410	https://www.inaturalist.org/observations/38569978
560	Sagittarius serpentarius	Secretarybird	2019/11/7	5	0	-1.1580390	35.2301128	https://www.inaturalist.org/observations/35941502
561	Sagittarius serpentarius	Secretarybird	2019/5/18	4	0	-1.1237454	35.3717802	https://www.inaturalist.org/observations/29125399
562	Sagittarius serpentarius	Secretarybird	2020/2/12	3	0	-1.1104619	35.1511589	https://www.inaturalist.org/observations/39034490
563	Sagittarius serpentarius	Secretarybird	2019/11/7	6	0	-1.0942572	35.2350065	https://www.inaturalist.org/observations/35941713
564	Sagittarius serpentarius	Secretarybird	2022/5/31	4	0	-1.0471971	35.0886638	https://www.inaturalist.org/observations/128421676
565	Sagittarius serpentarius	Secretarybird	2019/11/7	5	0	-1.0388258	35.2445915	https://www.inaturalist.org/observations/35916342
566	Sagittarius serpentarius	Secretarybird	2022/11/13	2	0	-1.0229680	35.2691970	https://www.inaturalist.org/observations/141903765
567	Sagittarius serpentarius	Secretarybird	2021/7/22	5	0	-0.9540823	36.3739463	https://www.inaturalist.org/observations/106038214
568	Sagittarius serpentarius	Secretarybird	2014/1/17	9	0	-0.9465428	36.3670757	http://www.inaturalist.org/observations/4019403
569	Sagittarius serpentarius	Secretarybird	2022/4/10	3	0	-0.9128155	36.2770117	https://www.inaturalist.org/observations/111106404
570	Sagittarius serpentarius	Secretarybird	2014/11/6	5	0	-0.8908588	36.2517767	https://www.inaturalist.org/observations/19539154
571	Sagittarius serpentarius	Secretarybird	2021/1/30	4	0	-0.8813976	36.3637454	https://www.inaturalist.org/observations/68803852
572	Sagittarius serpentarius	Secretarybird	2019/11/3	5	0	-0.8714394	36.3318807	https://www.inaturalist.org/observations/61625252
573	Sagittarius serpentarius	Secretarybird	2022/7/2	4	0	-0.8614261	36.2680871	https://www.inaturalist.org/observations/131114299
574	Sagittarius serpentarius	Secretarybird	2021/7/19	4	0	-0.8160255	36.2568157	https://www.inaturalist.org/observations/88698642

575	Sagittarius serpentarius	Secretarybird	2018/10/28	6	0	-0.7713268	36.3919780	https://www.inaturalist.org/observations/17891590
576	Sagittarius serpentarius	Secretarybird	2018/11/24	4	0	-0.6427220	36.2475662	https://www.inaturalist.org/observations/64867165
577	Sagittarius serpentarius	Secretarybird	2018/8/29	5	0	-0.6363530	36.4793580	https://www.inaturalist.org/observations/106197504
578	Sagittarius serpentarius	Secretarybird	2007/10/8	5	0	-0.6156544	36.1526391	https://www.inaturalist.org/observations/19526747
579	Sagittarius serpentarius	Secretarybird	2021/4/27	5	0	-0.5824818	36.3733146	https://www.inaturalist.org/observations/75663517
580	Sagittarius serpentarius	Secretarybird	2021/3/5	4	0	-0.5441341	36.1560443	https://www.inaturalist.org/observations/103822129
581	Sagittarius serpentarius	Secretarybird	1988/8/23	1	0	-0.5337372	36.1064366	https://www.inaturalist.org/observations/144395841
582	Sagittarius serpentarius	Secretarybird	2013/8/16	3	0	-0.5291634	36.3206798	https://www.inaturalist.org/observations/68216117
583	Sagittarius serpentarius	Secretarybird	2005/9/28	4	0	-0.5081413	36.1732282	https://www.inaturalist.org/observations/53291811
584	Sagittarius serpentarius	Secretarybird	2016/3/13	4	0	-0.5006131	36.3635746	https://www.inaturalist.org/observations/23647503
585	Sagittarius serpentarius	Secretarybird	2015/11/10	3	0	-0.4778561	35.0537801	https://www.inaturalist.org/observations/102295190
586	Sagittarius serpentarius	Secretarybird	2017/2/23	6	0	-0.4286497	36.1699826	https://www.inaturalist.org/observations/34030891
587	Sagittarius serpentarius	Secretarybird	2012/7/18	8	0	-0.4082258	36.1971113	https://www.inaturalist.org/observations/8212950
588	Sagittarius serpentarius	Secretarybird	2018/8/28	4	0	-0.4021597	36.1303294	https://www.inaturalist.org/observations/19311435
589	Sagittarius serpentarius	Secretarybird	2013/8/22	4	0	-0.3750722	36.1065632	https://www.inaturalist.org/observations/75408977
590	Sagittarius serpentarius	Secretarybird	2012/7/25	4	0	-0.3721916	36.0826560	https://www.inaturalist.org/observations/67377756
591	Sagittarius serpentarius	Secretarybird	2022/5/19	5	0	-0.3473339	36.0852270	https://www.inaturalist.org/observations/118686746
592	Sagittarius serpentarius	Secretarybird	2019/7/10	6	0	-0.3343044	36.1698875	https://www.inaturalist.org/observations/33067183
593	Sagittarius serpentarius	Secretarybird	2018/1/25	4	0	-0.3292126	36.0610682	https://www.inaturalist.org/observations/107246167
594	Sagittarius serpentarius	Secretarybird	2005/1/3	4	0	-0.3227299	36.1100750	https://www.inaturalist.org/observations/78493960
595	Sagittarius serpentarius	Secretarybird	2011/7/5	4	0	-0.3007868	36.1347262	https://www.inaturalist.org/observations/48659211
596	Sagittarius serpentarius	Secretarybird	2021/9/4	4	0	-0.2649171	36.9877273	https://www.inaturalist.org/observations/106565665
597	Sagittarius serpentarius	Secretarybird	2018/4/2	7	0	-0.2563834	36.0966754	https://www.inaturalist.org/observations/10566683

598	Sagittarius serpentarius	Secretarybird	2009/11/30	6	0	-0.2524509	36.0922906	https://www.inaturalist.org/observations/67712667
599	Sagittarius serpentarius	Secretarybird	2019/3/12	3	0	-0.2341856	36.8561431	https://www.inaturalist.org/observations/101340407
600	Sagittarius serpentarius	Secretarybird	2018/8/2	5	0	-0.1697482	36.9824020	https://www.inaturalist.org/observations/15765338
601	Sagittarius serpentarius	Secretarybird	2017/4/13	4	0	-0.1681043	30.1274929	https://www.inaturalist.org/observations/45517661
602	Sagittarius serpentarius	Secretarybird	2005/1/14	4	0	-0.0709719	37.8362019	https://www.inaturalist.org/observations/35669605
603	Sagittarius serpentarius	Secretarybird	2019/10/2	6	0	-0.0338362	36.9680113	https://www.inaturalist.org/observations/34296245
604	Sagittarius serpentarius	Secretarybird	2010/9/18	10	0	-0.0147034	36.8904850	http://www.inaturalist.org/observations/4493516
605	Sagittarius serpentarius	Secretarybird	2022/1/18	6	0	0.0021332	36.9493009	https://www.inaturalist.org/observations/120334508
606	Sagittarius serpentarius	Secretarybird	2018/8/10	4	0	0.0089160	36.8185556	https://www.inaturalist.org/observations/15826969
607	Sagittarius serpentarius	Secretarybird	2022/7/18	6	0	0.0175373	36.8015165	https://www.inaturalist.org/observations/126923801
608	Sagittarius serpentarius	Secretarybird	2022/6/27	5	0	0.0257317	38.1976938	https://www.inaturalist.org/observations/130942930
609	Sagittarius serpentarius	Secretarybird	2009/9/10	7	0	0.0286099	36.9213669	http://inaturalist.ca/observations/4798248
610	Sagittarius serpentarius	Secretarybird	2020/1/7	4	0	0.0486536	38.0041114	https://www.inaturalist.org/observations/68934434
611	Sagittarius serpentarius	Secretarybird	2007/8/12	8	0	0.0507586	36.7945202	https://www.inaturalist.org/observations/5393909
612	Sagittarius serpentarius	Secretarybird	2013/8/29	4	0	0.0666504	38.1005207	https://www.inaturalist.org/observations/65569732
613	Sagittarius serpentarius	Secretarybird	2014/10/2	3	0	0.0752240	36.8974641	https://www.inaturalist.org/observations/65978102
614	Sagittarius serpentarius	Secretarybird	2015/8/4	4	0	0.0798283	36.9922327	https://www.inaturalist.org/observations/105593414
615	Sagittarius serpentarius	Secretarybird	2022/6/27	4	0	0.0816037	38.0917318	https://www.inaturalist.org/observations/130941409
616	Sagittarius serpentarius	Secretarybird	2015/8/4	4	0	0.0944238	36.9266471	https://www.inaturalist.org/observations/105593420
617	Sagittarius serpentarius	Secretarybird	2014/8/28	6	0	0.1277004	36.9385049	https://www.inaturalist.org/observations/66930810
618	Sagittarius serpentarius	Secretarybird	2014/10/3	4	0	0.1370600	36.8372910	https://www.inaturalist.org/observations/65987071
619	Sagittarius serpentarius	Secretarybird	2020/2/13	5	0	0.1595794	36.9883097	https://www.inaturalist.org/observations/38610448
620	Sagittarius serpentarius	Secretarybird	2022/8/12	7	0	0.1876222	36.9859869	https://www.inaturalist.org/observations/135165150

621	Sagittarius serpentarius	Secretarybird	2018/12/9	6	0	0.1909767	37.5539211	https://www.inaturalist.org/observations/109918591
622	Sagittarius serpentarius	Secretarybird	2009/8/14	4	0	0.2073530	36.2319780	https://www.inaturalist.org/observations/35468390
623	Sagittarius serpentarius	Secretarybird	2016/5/11	3	0	0.2131266	36.8236225	https://www.inaturalist.org/observations/90850263
624	Sagittarius serpentarius	Secretarybird	2018/5/28	4	0	0.2384809	37.3445382	https://www.inaturalist.org/observations/45899605
625	Sagittarius serpentarius	Secretarybird	2010/2/15	5	0	0.3186979	37.4770473	https://www.inaturalist.org/observations/14236967
626	Sagittarius serpentarius	Secretarybird	2019/5/24	4	0	0.3314335	37.5863607	https://www.inaturalist.org/observations/70293485
627	Sagittarius serpentarius	Secretarybird	2014/7/5	5	0	0.3391944	37.7432605	https://www.inaturalist.org/observations/48463884
628	Sagittarius serpentarius	Secretarybird	2014/5/31	4	0	0.3472277	36.9122450	https://www.inaturalist.org/observations/71097128
629	Sagittarius serpentarius	Secretarybird	2020/1/25	3	0	0.3635187	36.8784193	https://www.inaturalist.org/observations/46597728
630	Sagittarius serpentarius	Secretarybird	2008/6/20	7	0	0.3737655	36.9956997	http://www.inaturalist.org/observations/3013217
631	Sagittarius serpentarius	Secretarybird	2019/5/9	5	0	0.3775417	36.9359857	https://www.inaturalist.org/observations/108461471
632	Sagittarius serpentarius	Secretarybird	2012/2/15	4	0	0.3784771	37.5972749	https://www.inaturalist.org/observations/71496903
633	Sagittarius serpentarius	Secretarybird	2019/3/9	6	0	0.4094597	37.5785281	https://www.inaturalist.org/observations/42293931
634	Sagittarius serpentarius	Secretarybird	2018/6/2	3	0	0.4213994	37.4430460	https://www.inaturalist.org/observations/66087909
635	Sagittarius serpentarius	Secretarybird	2019/2/3	5	0	0.4410139	37.5100947	https://www.inaturalist.org/observations/53940255
636	Sagittarius serpentarius	Secretarybird	2012/4/28	8	0	0.4442076	37.9113628	https://www.inaturalist.org/observations/7131885
637	Sagittarius serpentarius	Secretarybird	2020/1/5	3	0	0.4492489	37.5833215	https://www.inaturalist.org/observations/68930942
638	Sagittarius serpentarius	Secretarybird	2019/6/15	7	0	0.4496836	37.6200511	https://www.inaturalist.org/observations/53824873
639	Sagittarius serpentarius	Secretarybird	2013/8/19	5	0	0.4551236	37.4436710	https://www.inaturalist.org/observations/74362738
640	Sagittarius serpentarius	Secretarybird	2019/9/13	5	0	0.4566932	37.6113887	https://www.inaturalist.org/observations/34378915
641	Sagittarius serpentarius	Secretarybird	2019/7/31	6	0	0.4622454	37.5634531	https://www.inaturalist.org/observations/33952809
642	Sagittarius serpentarius	Secretarybird	2018/7/15	5	0	0.5096179	37.7622566	https://www.inaturalist.org/observations/36710119
643	Sagittarius serpentarius	Secretarybird	2014/5/16	6	0	0.5163328	37.5818628	https://www.inaturalist.org/observations/54462495

644	Sagittarius serpentarius	Secretarybird	1994/10/2	5	0	0.5170775	37.4662676	https://www.inaturalist.org/observations/54885308
645	Sagittarius serpentarius	Secretarybird	2016/5/11	4	0	0.5222602	37.7667418	https://www.inaturalist.org/observations/87115558
646	Sagittarius serpentarius	Secretarybird	2022/12/8	3	0	0.5228367	36.9253623	https://www.inaturalist.org/observations/144553203
647	Sagittarius serpentarius	Secretarybird	2019/11/6	4	0	0.5228560	37.7649494	https://www.inaturalist.org/observations/39965869
648	Sagittarius serpentarius	Secretarybird	2021/4/22	5	0	0.5319296	37.7275744	https://www.inaturalist.org/observations/79975555
649	Sagittarius serpentarius	Secretarybird	2019/2/3	6	0	0.5399151	37.4452009	https://www.inaturalist.org/observations/56609844
650	Sagittarius serpentarius	Secretarybird	2021/3/1	3	0	0.5410640	37.5537424	https://www.inaturalist.org/observations/98270049
651	Sagittarius serpentarius	Secretarybird	2019/2/4	5	0	0.5424707	37.5708761	https://www.inaturalist.org/observations/56610111
652	Sagittarius serpentarius	Secretarybird	2019/2/3	7	0	0.5656739	37.5279200	https://www.inaturalist.org/observations/56609841
653	Sagittarius serpentarius	Secretarybird	2018/8/19	5	0	0.5838816	37.6130378	https://www.inaturalist.org/observations/66474866
654	Sagittarius serpentarius	Secretarybird	2018/10/11	4	0	0.6152973	37.5030834	https://www.inaturalist.org/observations/17917474
655	Sagittarius serpentarius	Secretarybird	2018/7/20	4	0	0.6421964	37.8267362	https://www.inaturalist.org/observations/99167917
656	Sagittarius serpentarius	Secretarybird	2014/8/11	4	0	0.6458724	37.5705107	https://www.inaturalist.org/observations/142538444
657	Sagittarius serpentarius	Secretarybird	2017/2/28	7	0	0.6814332	37.7731788	https://www.inaturalist.org/observations/34245463
658	Sagittarius serpentarius	Secretarybird	1999/2/19	6	0	0.6869954	37.5832998	https://www.inaturalist.org/observations/8718211
659	Sagittarius serpentarius	Secretarybird	2002/7/11	3	0	0.6951674	37.8900047	https://www.inaturalist.org/observations/100914058
660	Sagittarius serpentarius	Secretarybird	2012/7/23	5	0	0.7007121	37.4871913	https://www.inaturalist.org/observations/67328674
661	Sagittarius serpentarius	Secretarybird	2019/2/12	6	0	0.7031222	37.7016520	https://www.inaturalist.org/observations/55466953
662	Sagittarius serpentarius	Secretarybird	2015/4/18	10	0	0.7075348	37.6529080	https://www.inaturalist.org/observations/6484986
663	Sagittarius serpentarius	Secretarybird	2012/12/13	4	0	0.7090356	37.7986217	https://www.inaturalist.org/observations/86704887
664	Sagittarius serpentarius	Secretarybird	2008/8/11	8	0	0.7391725	37.7920462	https://www.inaturalist.org/observations/28423731
665	Sagittarius serpentarius	Secretarybird	2016/1/8	4	0	0.7392277	37.9649815	https://www.inaturalist.org/observations/36198261
666	Sagittarius serpentarius	Secretarybird	2018/10/27	5	0	0.7442958	35.0340351	https://www.inaturalist.org/observations/21556498

667	Sagittarius serpentarius	Secretarybird	2010/8/11	6	0	0.7896243	37.4461605	https://www.inaturalist.org/observations/62229782
668	Sagittarius serpentarius	Secretarybird	2018/10/11	3	0	1.2174256	36.8544992	https://www.inaturalist.org/observations/18051353
669	Sagittarius serpentarius	Secretarybird	2017/8/24	2	0	3.6672708	33.9826612	https://www.inaturalist.org/observations/141983036
670	Sagittarius serpentarius	Secretarybird	2009/7/17	4	0	5.9493078	37.4013791	https://www.inaturalist.org/observations/34123038
671	Sagittarius serpentarius	Secretarybird	2015/1/14	5	0	9.0882502	40.0641417	https://www.inaturalist.org/observations/50012657
672	Sagittarius serpentarius	Secretarybird	2020/1/29	6	0	9.1848950	39.8074162	https://www.inaturalist.org/observations/38110830
673	Sagittarius serpentarius	Secretarybird	2018/7/23	5	0	9.1881085	40.1308918	https://www.inaturalist.org/observations/18735617
674	Sagittarius serpentarius	Secretarybird	2014/1/18	5	0	9.2597506	40.3991365	https://www.inaturalist.org/observations/37268072
675	Sagittarius serpentarius	Secretarybird	2020/2/28	4	0	9.3212186	40.3962196	https://www.inaturalist.org/observations/40287120
676	Sagittarius serpentarius	Secretarybird	2019/11/21	4	0	9.3383174	40.2561452	https://www.inaturalist.org/observations/68768773
677	Sagittarius serpentarius	Secretarybird	2018/11/14	3	0	9.3679930	40.2300280	https://www.inaturalist.org/observations/69006729
678	Sagittarius serpentarius	Secretarybird	2018/11/7	4	0	9.8131893	40.4656736	https://www.inaturalist.org/observations/111168343

No	scientific name	common name	date	NIA	NID	latitude	longitude	url
1	Acrocephalus dumetorum	Blyth's Reed-warbler	2020/2/16	3	0	6.17859000	81.21962833	https://www.inaturalist.org/observations/39488263
2	Acrocephalus dumetorum	Blyth's Reed-warbler	2018/12/6	0	0	6.83038301	80.80678377	https://www.inaturalist.org/observations/108652005
3	Acrocephalus dumetorum	Blyth's Reed-warbler	2017/3/10	1	0	6.86313523	81.04749101	https://www.inaturalist.org/observations/142455135
4	Acrocephalus dumetorum	Blyth's Reed-warbler	2022/3/7	0	0	6.88414533	79.94816098	https://www.inaturalist.org/observations/108174415
5	Acrocephalus dumetorum	Blyth's Reed-warbler	2018/12/7	0	0	6.97090863	80.76750357	https://www.inaturalist.org/observations/108652635
6	Acrocephalus dumetorum	Blyth's Reed-warbler	2017/12/31	1	0	6.97169770	80.76833577	https://www.inaturalist.org/observations/15184939
7	Acrocephalus dumetorum	Blyth's Reed-warbler	2022/11/19	0	0	7.06679840	79.90409320	https://www.inaturalist.org/observations/142361211
8	Acrocephalus dumetorum	Blyth's Reed-warbler	2020/11/22	1	0	7.13474511	80.59778418	https://www.inaturalist.org/observations/66242596
9	Acrocephalus dumetorum	Blyth's Reed-warbler	2018/12/4	0	0	8.04018789	80.74074319	https://www.inaturalist.org/observations/108586996
10	Acrocephalus dumetorum	Blyth's Reed-warbler	2016/12/25	2	0	8.18328570	77.41189960	https://www.inaturalist.org/observations/92355279
11	Acrocephalus dumetorum	Blyth's Reed-warbler	2017/2/22	2	0	8.39415968	76.97278194	https://www.inaturalist.org/observations/63686884
12	Acrocephalus dumetorum	Blyth's Reed-warbler	2022/4/8	1	0	8.43790040	77.57431871	https://www.inaturalist.org/observations/142139555
13	Acrocephalus dumetorum	Blyth's Reed-warbler	2022/2/28	1	0	8.45086033	76.98304385	https://www.inaturalist.org/observations/107683653
14	Acrocephalus dumetorum	Blyth's Reed-warbler	2017/11/14	2	0	8.49622960	77.75593700	https://www.inaturalist.org/observations/92338197
15	Acrocephalus dumetorum	Blyth's Reed-warbler	2018/3/17	2	0	8.59458890	77.35903530	https://www.inaturalist.org/observations/92216226
16	Acrocephalus dumetorum	Blyth's Reed-warbler	2022/1/2	2	0	9.29508057	76.51290703	https://www.inaturalist.org/observations/104579711
17	Acrocephalus dumetorum	Blyth's Reed-warbler	2013/2/19	3	0	9.49782600	77.18444800	https://www.inaturalist.org/observations/9791010
18	Acrocephalus dumetorum	Blyth's Reed-warbler	2022/3/31	3	0	9.58566092	76.52925868	https://www.inaturalist.org/observations/125780837
19	Acrocephalus dumetorum	Blyth's Reed-warbler	2018/12/22	1	0	9.59766245	76.51108997	https://www.inaturalist.org/observations/42827867
20	Acrocephalus dumetorum	Blyth's Reed-warbler	2018/11/17	1	0	9.59766245	76.51108997	https://www.inaturalist.org/observations/42830621
21	Acrocephalus dumetorum	Blyth's Reed-warbler	2018/12/2	1	0	9.59766245	76.51108997	https://www.inaturalist.org/observations/42833031
22	Acrocephalus dumetorum	Blyth's Reed-warbler	2022/3/12	2	0	9.75227019	76.84708163	https://www.inaturalist.org/observations/109025706

23	Acrocephalus dumetorum	Blyth's Reed-warbler	2022/3/29	2	0	9.79168429	76.88711400	https://www.inaturalist.org/observations/110475494
24	Acrocephalus dumetorum	Blyth's Reed-warbler	2022/12/17	1	0	9.79743095	76.28890391	https://www.inaturalist.org/observations/145195391
25	Acrocephalus dumetorum	Blyth's Reed-warbler	2020/11/17	2	0	9.79747423	76.28890693	https://www.inaturalist.org/observations/132781205
26	Acrocephalus dumetorum	Blyth's Reed-warbler	2015/2/21	1	0	9.81380760	76.92973540	https://www.inaturalist.org/observations/91652452
27	Acrocephalus dumetorum	Blyth's Reed-warbler	2021/2/21	3	0	9.83242131	76.78719599	https://www.inaturalist.org/observations/98355844
28	Acrocephalus dumetorum	Blyth's Reed-warbler	2021/12/15	1	0	9.83949625	76.78310320	https://www.inaturalist.org/observations/103102426
29	Acrocephalus dumetorum	Blyth's Reed-warbler	2018/12/22	1	0	9.83961495	76.78247295	https://www.inaturalist.org/observations/93562390
30	Acrocephalus dumetorum	Blyth's Reed-warbler	2021/12/5	1	0	9.83967275	76.78260484	https://www.inaturalist.org/observations/102768160
31	Acrocephalus dumetorum	Blyth's Reed-warbler	2018/11/21	1	0	9.88868643	76.72812668	https://www.inaturalist.org/observations/87113706
32	Acrocephalus dumetorum	Blyth's Reed-warbler	2018/12/13	1	0	9.88868643	76.72812668	https://www.inaturalist.org/observations/91985092
33	Acrocephalus dumetorum	Blyth's Reed-warbler	2018/12/30	3	0	9.88868643	76.72812668	https://www.inaturalist.org/observations/95771886
34	Acrocephalus dumetorum	Blyth's Reed-warbler	2022/11/27	1	0	9.89081268	76.69041719	https://www.inaturalist.org/observations/143184828
35	Acrocephalus dumetorum	Blyth's Reed-warbler	2019/2/9	1	0	9.90143090	76.79712470	https://www.inaturalist.org/observations/91655521
36	Acrocephalus dumetorum	Blyth's Reed-warbler	2012/3/25	2	0	9.95205500	76.31138429	https://www.inaturalist.org/observations/48679323
37	Acrocephalus dumetorum	Blyth's Reed-warbler	2014/12/28	1	0	10.00872499	77.16182171	https://www.inaturalist.org/observations/79197314
38	Acrocephalus dumetorum	Blyth's Reed-warbler	2022/12/17	1	0	10.05168713	76.34048633	https://www.inaturalist.org/observations/144524372
39	Acrocephalus dumetorum	Blyth's Reed-warbler	2022/12/17	1	0	10.05168713	76.34048633	https://www.inaturalist.org/observations/144524373
40	Acrocephalus dumetorum	Blyth's Reed-warbler	2022/12/5	0	0	10.05435524	76.34151746	https://www.inaturalist.org/observations/143708523
41	Acrocephalus dumetorum	Blyth's Reed-warbler	2015/1/30	1	0	10.06137300	77.08433000	https://www.inaturalist.org/observations/35324389
42	Acrocephalus dumetorum	Blyth's Reed-warbler	2016/11/27	1	0	10.06646078	77.01271880	https://www.inaturalist.org/observations/59861035
43	Acrocephalus dumetorum	Blyth's Reed-warbler	2019/2/2	3	0	10.08695674	76.95485840	https://www.inaturalist.org/observations/121908105
44	Acrocephalus dumetorum	Blyth's Reed-warbler	2017/4/1	1	0	10.08893330	77.05952480	https://www.inaturalist.org/observations/64362622
45	Acrocephalus dumetorum	Blyth's Reed-warbler	2021/1/30	1	0	10.08897943	77.05952469	https://www.inaturalist.org/observations/68922048

46	Acrocephalus dumetorum	Blyth's Reed-warbler	2020/12/3	1	0	10.09851993	76.33183468	https://www.inaturalist.org/observations/66607096
47	Acrocephalus dumetorum	Blyth's Reed-warbler	2018/12/16	1	0	10.10398030	76.70046050	https://www.inaturalist.org/observations/32152995
48	Acrocephalus dumetorum	Blyth's Reed-warbler	2017/12/20	2	0	10.12067400	76.75534700	https://www.inaturalist.org/observations/9914839
49	Acrocephalus dumetorum	Blyth's Reed-warbler	2020/1/12	1	0	10.13098780	76.24104930	https://www.inaturalist.org/observations/41221905
50	Acrocephalus dumetorum	Blyth's Reed-warbler	2020/1/18	1	0	10.13098780	76.24104930	https://www.inaturalist.org/observations/41221931
51	Acrocephalus dumetorum	Blyth's Reed-warbler	2019/12/21	2	0	10.13098780	76.24104930	https://www.inaturalist.org/observations/41279046
52	Acrocephalus dumetorum	Blyth's Reed-warbler	2019/12/21	1	0	10.13098780	76.24104930	https://www.inaturalist.org/observations/41279064
53	Acrocephalus dumetorum	Blyth's Reed-warbler	2016/12/10	2	0	10.13431600	76.69413600	https://www.inaturalist.org/observations/19624646
54	Acrocephalus dumetorum	Blyth's Reed-warbler	2016/12/10	1	0	10.13576843	76.66057829	https://www.inaturalist.org/observations/5216245
55	Acrocephalus dumetorum	Blyth's Reed-warbler	2022/1/15	1	0	10.14991250	76.36897560	https://www.inaturalist.org/observations/104959270
56	Acrocephalus dumetorum	Blyth's Reed-warbler	2022/12/4	1	0	10.15322610	76.33767860	https://www.inaturalist.org/observations/143622386
57	Acrocephalus dumetorum	Blyth's Reed-warbler	2022/12/22	0	0	10.15322610	76.33767860	https://www.inaturalist.org/observations/144927429
58	Acrocephalus dumetorum	Blyth's Reed-warbler	2016/12/20	1	0	10.16706259	76.76549037	https://www.inaturalist.org/observations/66798802
59	Acrocephalus dumetorum	Blyth's Reed-warbler	2020/12/14	1	0	10.24769080	76.33484780	https://www.inaturalist.org/observations/143784835
60	Acrocephalus dumetorum	Blyth's Reed-warbler	2020/12/14	1	0	10.24769080	76.33484780	https://www.inaturalist.org/observations/143785101
61	Acrocephalus dumetorum	Blyth's Reed-warbler	2016/11/10	1	0	10.31222761	76.25731722	https://www.inaturalist.org/observations/35271299
62	Acrocephalus dumetorum	Blyth's Reed-warbler	2017/3/10	2	0	10.32697340	76.95539670	https://www.inaturalist.org/observations/92335036
63	Acrocephalus dumetorum	Blyth's Reed-warbler	2021/3/27	1	0	10.32697340	76.95539670	https://www.inaturalist.org/observations/105202395
64	Acrocephalus dumetorum	Blyth's Reed-warbler	2021/3/31	2	0	10.32697340	76.95539670	https://www.inaturalist.org/observations/105256430
65	Acrocephalus dumetorum	Blyth's Reed-warbler	2020/1/1	1	0	10.34644550	76.26877896	https://www.inaturalist.org/observations/41674314
66	Acrocephalus dumetorum	Blyth's Reed-warbler	2019/10/17	3	0	10.45731290	76.15495400	https://www.inaturalist.org/observations/102271948
67	Acrocephalus dumetorum	Blyth's Reed-warbler	2016/12/18	1	0	10.74748521	76.46730135	https://www.inaturalist.org/observations/65617744
68	Acrocephalus dumetorum	Blyth's Reed-warbler	2016/12/28	3	0	10.74785979	76.46728928	https://www.inaturalist.org/observations/87190204

69	Acrocephalus dumetorum	Blyth's Reed-warbler	2016/12/30	3	0	10.74785979	76.46728928	https://www.inaturalist.org/observations/87190205
70	Acrocephalus dumetorum	Blyth's Reed-warbler	2022/11/12	1	0	10.78576333	75.91389667	https://www.inaturalist.org/observations/142464962
71	Acrocephalus dumetorum	Blyth's Reed-warbler	2019/1/29	1	0	10.88777170	76.07321890	https://www.inaturalist.org/observations/67239996
72	Acrocephalus dumetorum	Blyth's Reed-warbler	2012/11/11	2	0	10.98905300	77.02274400	https://www.inaturalist.org/observations/98510675
73	Acrocephalus dumetorum	Blyth's Reed-warbler	2020/11/25	1	0	11.01684450	76.95583210	https://www.inaturalist.org/observations/88778873
74	Acrocephalus dumetorum	Blyth's Reed-warbler	2020/11/10	1	0	11.07894586	79.83002432	https://www.inaturalist.org/observations/64835885
75	Acrocephalus dumetorum	Blyth's Reed-warbler	2013/2/13	3	0	11.10483300	76.76827750	http://www.inaturalist.org/observations/204215
76	Acrocephalus dumetorum	Blyth's Reed-warbler	2019/10/16	1	0	11.27578553	77.58298729	https://www.inaturalist.org/observations/37212458
77	Acrocephalus dumetorum	Blyth's Reed-warbler	2018/3/18	2	0	11.43910397	78.03879409	https://www.inaturalist.org/observations/105405183
78	Acrocephalus dumetorum	Blyth's Reed-warbler	2018/1/7	2	0	11.46102600	77.92968750	https://www.inaturalist.org/observations/58736606
79	Acrocephalus dumetorum	Blyth's Reed-warbler	2016/11/12	2	0	11.63966497	75.93735350	https://www.inaturalist.org/observations/46986410
80	Acrocephalus dumetorum	Blyth's Reed-warbler	2021/2/14	1	0	11.68742128	78.18066828	https://www.inaturalist.org/observations/69551110
81	Acrocephalus dumetorum	Blyth's Reed-warbler	2021/2/28	1	0	11.68742128	78.18066828	https://www.inaturalist.org/observations/70307168
82	Acrocephalus dumetorum	Blyth's Reed-warbler	2021/11/7	2	0	11.68742128	78.18066828	https://www.inaturalist.org/observations/100985059
83	Acrocephalus dumetorum	Blyth's Reed-warbler	2020/10/31	2	0	11.71324820	76.17053120	https://www.inaturalist.org/observations/63900264
84	Acrocephalus dumetorum	Blyth's Reed-warbler	2015/1/20	1	0	11.74423266	78.05355422	https://www.inaturalist.org/observations/143067257
85	Acrocephalus dumetorum	Blyth's Reed-warbler	2022/11/30	1	0	11.77711166	76.78599972	https://www.inaturalist.org/observations/144841318
86	Acrocephalus dumetorum	Blyth's Reed-warbler	2015/3/15	2	0	11.93829479	76.24339692	https://www.inaturalist.org/observations/63686882
87	Acrocephalus dumetorum	Blyth's Reed-warbler	2022/11/25	1	0	11.94468399	75.38493812	https://www.inaturalist.org/observations/144724503
88	Acrocephalus dumetorum	Blyth's Reed-warbler	2021/12/31	2	0	11.94468432	75.38493812	https://www.inaturalist.org/observations/110830576
89	Acrocephalus dumetorum	Blyth's Reed-warbler	2022/3/29	2	0	11.94468432	75.38493812	https://www.inaturalist.org/observations/113079293
90	Acrocephalus dumetorum	Blyth's Reed-warbler	2022/3/23	0	0	11.94468432	75.38493812	https://www.inaturalist.org/observations/116440081
91	Acrocephalus dumetorum	Blyth's Reed-warbler	2022/3/23	0	0	11.94468432	75.38493812	https://www.inaturalist.org/observations/116441739

92	Acrocephalus dumetorum	Blyth's Reed-warbler	2022/3/28	0	0	11.94468432	75.38493812	https://www.inaturalist.org/observations/116444922
93	Acrocephalus dumetorum	Blyth's Reed-warbler	2022/3/28	0	0	11.94468432	75.38493812	https://www.inaturalist.org/observations/116444987
94	Acrocephalus dumetorum	Blyth's Reed-warbler	2022/3/29	0	0	11.94468432	75.38493812	https://www.inaturalist.org/observations/116446229
95	Acrocephalus dumetorum	Blyth's Reed-warbler	2022/3/22	1	0	11.94468432	75.38493812	https://www.inaturalist.org/observations/116447494
96	Acrocephalus dumetorum	Blyth's Reed-warbler	2022/3/23	1	0	11.94468432	75.38493812	https://www.inaturalist.org/observations/116515501
97	Acrocephalus dumetorum	Blyth's Reed-warbler	2022/3/28	1	0	11.94468432	75.38493812	https://www.inaturalist.org/observations/116538984
98	Acrocephalus dumetorum	Blyth's Reed-warbler	2021/3/9	2	0	11.94468432	75.38493812	https://www.inaturalist.org/observations/122789040
99	Acrocephalus dumetorum	Blyth's Reed-warbler	2021/3/9	1	0	11.94468432	75.38493812	https://www.inaturalist.org/observations/122789124
100	Acrocephalus dumetorum	Blyth's Reed-warbler	2021/3/9	2	0	11.94468432	75.38493812	https://www.inaturalist.org/observations/122789240
101	Acrocephalus dumetorum	Blyth's Reed-warbler	2021/3/14	3	0	11.94468432	75.38493812	https://www.inaturalist.org/observations/122789434
102	Acrocephalus dumetorum	Blyth's Reed-warbler	2021/3/24	2	0	11.94468432	75.38493812	https://www.inaturalist.org/observations/125427649
103	Acrocephalus dumetorum	Blyth's Reed-warbler	2022/1/2	1	0	11.94468432	75.38493812	https://www.inaturalist.org/observations/125767698
104	Acrocephalus dumetorum	Blyth's Reed-warbler	2022/4/9	1	0	11.94468432	75.38493812	https://www.inaturalist.org/observations/128118219
105	Acrocephalus dumetorum	Blyth's Reed-warbler	2022/4/9	1	0	11.94468432	75.38493812	https://www.inaturalist.org/observations/128121554
106	Acrocephalus dumetorum	Blyth's Reed-warbler	2022/11/19	0	0	11.94468432	75.38493812	https://www.inaturalist.org/observations/144223890
107	Acrocephalus dumetorum	Blyth's Reed-warbler	2022/11/22	0	0	11.94468432	75.38493812	https://www.inaturalist.org/observations/144412901
108	Acrocephalus dumetorum	Blyth's Reed-warbler	2022/11/10	2	0	11.94468432	75.38493812	https://www.inaturalist.org/observations/144725282
109	Acrocephalus dumetorum	Blyth's Reed-warbler	2022/11/29	0	0	11.94468432	75.38493812	https://www.inaturalist.org/observations/144910398
110	Acrocephalus dumetorum	Blyth's Reed-warbler	2022/11/29	0	0	11.94468432	75.38493812	https://www.inaturalist.org/observations/144910449
111	Acrocephalus dumetorum	Blyth's Reed-warbler	2022/11/29	0	0	11.94468432	75.38493812	https://www.inaturalist.org/observations/144910736
112	Acrocephalus dumetorum	Blyth's Reed-warbler	2020/11/22	1	0	11.94468464	75.38493812	https://www.inaturalist.org/observations/67564921
113	Acrocephalus dumetorum	Blyth's Reed-warbler	2020/12/15	0	0	11.94468464	75.38493812	https://www.inaturalist.org/observations/67595105
114	Acrocephalus dumetorum	Blyth's Reed-warbler	2020/12/5	1	0	11.94468464	75.38493812	https://www.inaturalist.org/observations/82715471

115	Acrocephalus dumetorum	Blyth's Reed-warbler	2021/12/24	1	0	12.02686493	75.36451109	https://www.inaturalist.org/observations/103829514
116	Acrocephalus dumetorum	Blyth's Reed-warbler	2017/3/14	1	0	12.03000078	75.20706394	https://www.inaturalist.org/observations/30603840
117	Acrocephalus dumetorum	Blyth's Reed-warbler	2017/3/18	1	0	12.03000078	75.20706394	https://www.inaturalist.org/observations/30603898
118	Acrocephalus dumetorum	Blyth's Reed-warbler	2020/12/19	1	0	12.05749173	75.20062657	https://www.inaturalist.org/observations/66999102
119	Acrocephalus dumetorum	Blyth's Reed-warbler	2022/2/10	1	0	12.05749173	75.20062657	https://www.inaturalist.org/observations/142750937
120	Acrocephalus dumetorum	Blyth's Reed-warbler	2021/12/12	1	0	12.16529924	75.42718928	https://www.inaturalist.org/observations/119894439
121	Acrocephalus dumetorum	Blyth's Reed-warbler	2021/12/12	1	0	12.16529924	75.42718928	https://www.inaturalist.org/observations/119989746
122	Acrocephalus dumetorum	Blyth's Reed-warbler	2021/12/12	1	0	12.16529924	75.42718928	https://www.inaturalist.org/observations/119989909
123	Acrocephalus dumetorum	Blyth's Reed-warbler	2010/3/14	3	0	12.21050906	75.83248603	http://www.inaturalist.org/observations/115978
124	Acrocephalus dumetorum	Blyth's Reed-warbler	2015/12/15	2	0	12.22272330	76.62728760	https://www.inaturalist.org/observations/142816116
125	Acrocephalus dumetorum	Blyth's Reed-warbler	2017/4/25	1	0	12.22930016	79.05346120	https://www.inaturalist.org/observations/95943643
126	Acrocephalus dumetorum	Blyth's Reed-warbler	2022/11/23	1	0	12.24090600	76.59794450	https://www.inaturalist.org/observations/142674852
127	Acrocephalus dumetorum	Blyth's Reed-warbler	2022/12/24	1	0	12.24180672	76.73531771	https://www.inaturalist.org/observations/145115081
128	Acrocephalus dumetorum	Blyth's Reed-warbler	2020/11/14	1	0	12.26269942	76.56762058	https://www.inaturalist.org/observations/95042455
129	Acrocephalus dumetorum	Blyth's Reed-warbler	2012/1/19	2	0	12.26937200	76.61197800	https://www.inaturalist.org/observations/94717002
130	Acrocephalus dumetorum	Blyth's Reed-warbler	2022/1/5	1	0	12.26937200	76.61197800	https://www.inaturalist.org/observations/104401072
131	Acrocephalus dumetorum	Blyth's Reed-warbler	2019/1/5	2	0	12.27601167	76.61604333	https://www.inaturalist.org/observations/19465638
132	Acrocephalus dumetorum	Blyth's Reed-warbler	2018/2/8	2	0	12.28291627	76.61502596	https://www.inaturalist.org/observations/10259720
133	Acrocephalus dumetorum	Blyth's Reed-warbler	2021/3/15	1	0	12.28427231	76.68319375	https://www.inaturalist.org/observations/81010626
134	Acrocephalus dumetorum	Blyth's Reed-warbler	2022/11/7	1	0	12.31071867	76.63349030	https://www.inaturalist.org/observations/141359619
135	Acrocephalus dumetorum	Blyth's Reed-warbler	2022/1/7	1	0	12.32277930	76.91736680	https://www.inaturalist.org/observations/104579896
136	Acrocephalus dumetorum	Blyth's Reed-warbler	2021/2/14	2	0	12.35037258	77.84846985	https://www.inaturalist.org/observations/92550760
137	Acrocephalus dumetorum	Blyth's Reed-warbler	2021/3/13	3	0	12.40395219	77.85719705	https://www.inaturalist.org/observations/126170854

138	Acrocephalus dumetorum	Blyth's Reed-warbler	2013/6/19	2	0	12.41666670	77.75000000	https://www.inaturalist.org/observations/64289422
139	Acrocephalus dumetorum	Blyth's Reed-warbler	2020/3/28	0	0	12.42843735	77.65531272	https://www.inaturalist.org/observations/65196607
140	Acrocephalus dumetorum	Blyth's Reed-warbler	2021/1/23	1	0	12.44703279	76.58019383	https://www.inaturalist.org/observations/68592254
141	Acrocephalus dumetorum	Blyth's Reed-warbler	2017/2/10	2	0	12.54545970	79.85606940	https://www.inaturalist.org/observations/92332973
142	Acrocephalus dumetorum	Blyth's Reed-warbler	2013/1/26	1	0	12.64780281	76.62294091	https://www.inaturalist.org/observations/98110935
143	Acrocephalus dumetorum	Blyth's Reed-warbler	2022/12/3	1	0	12.67869667	77.92445833	https://www.inaturalist.org/observations/143552270
144	Acrocephalus dumetorum	Blyth's Reed-warbler	2022/2/21	1	0	12.68289207	77.81519048	https://www.inaturalist.org/observations/107158160
145	Acrocephalus dumetorum	Blyth's Reed-warbler	2022/1/1	1	0	12.71384972	77.81438833	https://www.inaturalist.org/observations/106980791
146	Acrocephalus dumetorum	Blyth's Reed-warbler	2016/2/9	1	0	12.73534289	77.79045663	https://www.inaturalist.org/observations/141769089
147	Acrocephalus dumetorum	Blyth's Reed-warbler	2017/11/30	2	0	12.73534289	77.79045663	https://www.inaturalist.org/observations/141769333
148	Acrocephalus dumetorum	Blyth's Reed-warbler	2016/12/4	1	0	12.73795236	77.01309715	https://www.inaturalist.org/observations/82405088
149	Acrocephalus dumetorum	Blyth's Reed-warbler	2016/12/4	1	0	12.73795236	77.01309715	https://www.inaturalist.org/observations/82405092
150	Acrocephalus dumetorum	Blyth's Reed-warbler	2015/12/23	2	0	12.74091270	77.82529230	https://www.inaturalist.org/observations/33655043
151	Acrocephalus dumetorum	Blyth's Reed-warbler	2016/2/9	2	0	12.74091270	77.82529230	https://www.inaturalist.org/observations/33655046
152	Acrocephalus dumetorum	Blyth's Reed-warbler	2018/11/17	1	0	12.78126397	77.66285777	https://www.inaturalist.org/observations/18675692
153	Acrocephalus dumetorum	Blyth's Reed-warbler	2016/2/21	2	0	12.80202000	77.57435667	http://www.inaturalist.org/observations/2729075
154	Acrocephalus dumetorum	Blyth's Reed-warbler	2016/2/21	1	0	12.80216833	77.57481667	http://www.inaturalist.org/observations/2729077
155	Acrocephalus dumetorum	Blyth's Reed-warbler	2021/3/14	1	0	12.80648870	77.52726540	https://www.inaturalist.org/observations/71202011
156	Acrocephalus dumetorum	Blyth's Reed-warbler	2018/1/14	1	0	12.81385331	76.94609644	https://www.inaturalist.org/observations/96773247
157	Acrocephalus dumetorum	Blyth's Reed-warbler	2018/2/25	2	0	12.81385331	76.94609644	https://www.inaturalist.org/observations/96773476
158	Acrocephalus dumetorum	Blyth's Reed-warbler	2021/3/24	1	0	12.81744369	76.94470741	https://www.inaturalist.org/observations/81047425
159	Acrocephalus dumetorum	Blyth's Reed-warbler	2022/2/26	3	0	12.81813820	74.86750717	https://www.inaturalist.org/observations/108010683
160	Acrocephalus dumetorum	Blyth's Reed-warbler	2018/11/25	0	0	12.82046687	77.72457819	https://www.inaturalist.org/observations/67003288

161	Acrocephalus dumetorum	Blyth's Reed-warbler	2022/1/24	2	0	12.83896200	77.65182300	https://www.inaturalist.org/observations/106053772
162	Acrocephalus dumetorum	Blyth's Reed-warbler	2022/12/22	0	0	12.84550402	77.72021143	https://www.inaturalist.org/observations/144859510
163	Acrocephalus dumetorum	Blyth's Reed-warbler	2021/12/12	1	0	12.85121818	77.49016882	https://www.inaturalist.org/observations/102922077
164	Acrocephalus dumetorum	Blyth's Reed-warbler	2022/11/27	2	0	12.85431470	77.50994530	https://www.inaturalist.org/observations/143801409
165	Acrocephalus dumetorum	Blyth's Reed-warbler	2021/3/21	1	0	12.85718650	77.50891100	https://www.inaturalist.org/observations/71692530
166	Acrocephalus dumetorum	Blyth's Reed-warbler	2021/4/4	1	0	12.85718650	77.50891100	https://www.inaturalist.org/observations/72895901
167	Acrocephalus dumetorum	Blyth's Reed-warbler	2020/3/15	1	0	12.86430660	77.52127510	https://www.inaturalist.org/observations/55021331
168	Acrocephalus dumetorum	Blyth's Reed-warbler	2020/3/15	1	0	12.86556530	77.52171710	https://www.inaturalist.org/observations/55021333
169	Acrocephalus dumetorum	Blyth's Reed-warbler	2021/12/22	1	0	12.87414820	75.76445320	https://www.inaturalist.org/observations/103679445
170	Acrocephalus dumetorum	Blyth's Reed-warbler	2022/2/27	3	0	12.87880300	79.13077500	https://www.inaturalist.org/observations/129006275
171	Acrocephalus dumetorum	Blyth's Reed-warbler	2021/3/27	1	0	12.87892400	77.53009120	https://www.inaturalist.org/observations/72168377
172	Acrocephalus dumetorum	Blyth's Reed-warbler	2021/4/3	1	0	12.87896584	77.52959767	https://www.inaturalist.org/observations/72766372
173	Acrocephalus dumetorum	Blyth's Reed-warbler	2022/2/19	1	0	12.87958292	77.52957622	https://www.inaturalist.org/observations/107035737
174	Acrocephalus dumetorum	Blyth's Reed-warbler	2022/2/19	1	0	12.87958292	77.52957622	https://www.inaturalist.org/observations/107035741
175	Acrocephalus dumetorum	Blyth's Reed-warbler	2020/12/24	0	0	12.87959337	77.52949039	https://www.inaturalist.org/observations/67033374
176	Acrocephalus dumetorum	Blyth's Reed-warbler	2021/1/23	1	0	12.87976072	77.52975861	https://www.inaturalist.org/observations/68483077
177	Acrocephalus dumetorum	Blyth's Reed-warbler	2018/12/25	2	0	12.88035107	77.52930391	https://www.inaturalist.org/observations/19213991
178	Acrocephalus dumetorum	Blyth's Reed-warbler	2022/4/14	1	0	12.88585912	80.23352545	https://www.inaturalist.org/observations/111392345
179	Acrocephalus dumetorum	Blyth's Reed-warbler	2016/4/18	1	0	12.88662979	77.67267399	https://www.inaturalist.org/observations/79309837
180	Acrocephalus dumetorum	Blyth's Reed-warbler	2018/12/22	1	0	12.89490040	77.59362590	https://www.inaturalist.org/observations/19229595
181	Acrocephalus dumetorum	Blyth's Reed-warbler	2019/2/7	5	0	12.90075342	80.22180051	https://www.inaturalist.org/observations/29122635
182	Acrocephalus dumetorum	Blyth's Reed-warbler	2020/3/28	2	0	12.90898530	77.49020970	https://www.inaturalist.org/observations/41221046
183	Acrocephalus dumetorum	Blyth's Reed-warbler	2021/1/15	2	0	12.91083150	79.40114880	https://www.inaturalist.org/observations/82160905

184	Acrocephalus dumetorum	Blyth's Reed-warbler	2021/1/16	0	0	12.91083150	79.40114880	https://www.inaturalist.org/observations/82160920
185	Acrocephalus dumetorum	Blyth's Reed-warbler	2021/1/17	1	0	12.91083150	79.40114880	https://www.inaturalist.org/observations/82160932
186	Acrocephalus dumetorum	Blyth's Reed-warbler	2021/4/18	1	0	12.91693093	77.66491146	https://www.inaturalist.org/observations/74495250
187	Acrocephalus dumetorum	Blyth's Reed-warbler	2022/11/19	1	0	12.91831260	77.68009700	https://www.inaturalist.org/observations/142377131
188	Acrocephalus dumetorum	Blyth's Reed-warbler	2019/2/2	2	0	12.92352831	80.22974152	https://www.inaturalist.org/observations/22614363
189	Acrocephalus dumetorum	Blyth's Reed-warbler	2020/11/27	1	0	12.92493080	80.10000260	https://www.inaturalist.org/observations/65685359
190	Acrocephalus dumetorum	Blyth's Reed-warbler	2019/12/25	1	0	12.92904566	80.17216120	https://www.inaturalist.org/observations/36940820
191	Acrocephalus dumetorum	Blyth's Reed-warbler	2017/1/21	1	0	12.93352999	80.16560955	https://www.inaturalist.org/observations/48674456
192	Acrocephalus dumetorum	Blyth's Reed-warbler	2020/11/28	1	0	12.93365513	80.16540974	https://www.inaturalist.org/observations/65750786
193	Acrocephalus dumetorum	Blyth's Reed-warbler	2019/2/9	3	0	12.93475220	80.16483650	https://www.inaturalist.org/observations/22939351
194	Acrocephalus dumetorum	Blyth's Reed-warbler	2021/12/22	2	0	12.94663116	80.22376589	https://www.inaturalist.org/observations/103526563
195	Acrocephalus dumetorum	Blyth's Reed-warbler	2016/2/22	2	0	12.94747667	77.58131500	http://www.inaturalist.org/observations/2729091
196	Acrocephalus dumetorum	Blyth's Reed-warbler	2022/12/16	1	0	12.95016470	77.74091620	https://www.inaturalist.org/observations/144731612
197	Acrocephalus dumetorum	Blyth's Reed-warbler	2022/12/16	1	0	12.95016470	77.74091620	https://www.inaturalist.org/observations/144731613
198	Acrocephalus dumetorum	Blyth's Reed-warbler	2022/1/29	2	0	12.95023600	74.86877440	https://www.inaturalist.org/observations/107315659
199	Acrocephalus dumetorum	Blyth's Reed-warbler	2022/11/17	2	0	12.95826591	77.72553563	https://www.inaturalist.org/observations/142223363
200	Acrocephalus dumetorum	Blyth's Reed-warbler	2022/11/21	1	0	12.95826600	77.72553570	https://www.inaturalist.org/observations/142583446
201	Acrocephalus dumetorum	Blyth's Reed-warbler	2022/11/5	1	0	12.96098599	77.70836413	https://www.inaturalist.org/observations/141135196
202	Acrocephalus dumetorum	Blyth's Reed-warbler	2022/11/6	1	0	12.96098599	77.70836413	https://www.inaturalist.org/observations/141239267
203	Acrocephalus dumetorum	Blyth's Reed-warbler	2020/1/26	1	0	12.97159870	77.59456270	https://www.inaturalist.org/observations/55021332
204	Acrocephalus dumetorum	Blyth's Reed-warbler	2020/4/6	0	0	12.97159870	77.59456270	https://www.inaturalist.org/observations/55895106
205	Acrocephalus dumetorum	Blyth's Reed-warbler	2010/12/7	2	0	12.98302954	80.26426727	https://www.inaturalist.org/observations/22593185
206	Acrocephalus dumetorum	Blyth's Reed-warbler	2016/11/27	1	0	13.01428665	77.68332370	https://www.inaturalist.org/observations/139102193

207	Acrocephalus dumetorum	Blyth's Reed-warbler	2022/12/3	0	0	13.01428665	77.68332370	https://www.inaturalist.org/observations/143713124
208	Acrocephalus dumetorum	Blyth's Reed-warbler	2016/2/20	2	0	13.02121667	77.57052333	http://www.inaturalist.org/observations/2729062
209	Acrocephalus dumetorum	Blyth's Reed-warbler	2016/2/20	2	0	13.02171167	77.57008000	http://www.inaturalist.org/observations/2729063
210	Acrocephalus dumetorum	Blyth's Reed-warbler	2016/2/20	2	0	13.02171500	77.57007167	http://www.inaturalist.org/observations/2729064
211	Acrocephalus dumetorum	Blyth's Reed-warbler	2020/12/17	1	0	13.02566907	77.56803554	https://www.inaturalist.org/observations/143785460
212	Acrocephalus dumetorum	Blyth's Reed-warbler	2022/2/25	3	0	13.03361997	77.57284864	https://www.inaturalist.org/observations/109819503
213	Acrocephalus dumetorum	Blyth's Reed-warbler	2022/3/13	2	0	13.03395440	77.57267691	https://www.inaturalist.org/observations/110474630
214	Acrocephalus dumetorum	Blyth's Reed-warbler	2022/1/31	1	0	13.06473088	77.74819393	https://www.inaturalist.org/observations/106228084
215	Acrocephalus dumetorum	Blyth's Reed-warbler	2021/1/6	2	0	13.06558420	80.16077680	https://www.inaturalist.org/observations/83649561
216	Acrocephalus dumetorum	Blyth's Reed-warbler	2019/1/1	2	0	13.06606274	77.77125120	https://www.inaturalist.org/observations/19396736
217	Acrocephalus dumetorum	Blyth's Reed-warbler	2022/11/27	1	0	13.07029114	77.76952554	https://www.inaturalist.org/observations/143172896
218	Acrocephalus dumetorum	Blyth's Reed-warbler	2022/11/27	1	0	13.07029114	77.76952554	https://www.inaturalist.org/observations/143179596
219	Acrocephalus dumetorum	Blyth's Reed-warbler	2022/12/17	2	0	13.07029150	77.76952550	https://www.inaturalist.org/observations/144996125
220	Acrocephalus dumetorum	Blyth's Reed-warbler	2019/4/7	2	0	13.07135833	77.58154000	https://www.inaturalist.org/observations/29238172
221	Acrocephalus dumetorum	Blyth's Reed-warbler	2015/4/19	2	0	13.07979148	77.58405043	https://www.inaturalist.org/observations/29642001
222	Acrocephalus dumetorum	Blyth's Reed-warbler	2021/2/24	2	0	13.08007180	77.57851510	https://www.inaturalist.org/observations/117713529
223	Acrocephalus dumetorum	Blyth's Reed-warbler	2019/12/20	1	0	13.08736710	77.60993500	https://www.inaturalist.org/observations/45945945
224	Acrocephalus dumetorum	Blyth's Reed-warbler	2019/12/20	0	0	13.08736710	77.60993500	https://www.inaturalist.org/observations/45945946
225	Acrocephalus dumetorum	Blyth's Reed-warbler	2019/12/20	1	0	13.08736710	77.60993500	https://www.inaturalist.org/observations/45945947
226	Acrocephalus dumetorum	Blyth's Reed-warbler	2019/12/20	1	0	13.08736710	77.60993500	https://www.inaturalist.org/observations/45945950
227	Acrocephalus dumetorum	Blyth's Reed-warbler	2019/12/20	1	0	13.08736710	77.60993500	https://www.inaturalist.org/observations/45950668
228	Acrocephalus dumetorum	Blyth's Reed-warbler	2019/12/20	1	0	13.08736710	77.60993500	https://www.inaturalist.org/observations/45950767
229	Acrocephalus dumetorum	Blyth's Reed-warbler	2021/2/24	2	0	13.08835418	77.48489857	https://www.inaturalist.org/observations/70072394

230	Acrocephalus dumetorum	Blyth's Reed-warbler	2022/2/27	1	0	13.12287510	80.18053180	https://www.inaturalist.org/observations/107697019
231	Acrocephalus dumetorum	Blyth's Reed-warbler	2017/1/15	1	0	13.14914667	80.05789630	https://www.inaturalist.org/observations/45849464
232	Acrocephalus dumetorum	Blyth's Reed-warbler	2019/1/5	1	0	13.15918863	77.57209618	https://www.inaturalist.org/observations/19486843
233	Acrocephalus dumetorum	Blyth's Reed-warbler	2021/1/19	1	0	13.24022230	75.25405600	https://www.inaturalist.org/observations/69714070
234	Acrocephalus dumetorum	Blyth's Reed-warbler	2022/11/10	1	0	13.36738239	74.81768807	https://www.inaturalist.org/observations/141886348
235	Acrocephalus dumetorum	Blyth's Reed-warbler	2019/4/13	1	0	13.37197088	77.68109337	https://www.inaturalist.org/observations/22727957
236	Acrocephalus dumetorum	Blyth's Reed-warbler	2016/11/23	2	0	13.41773820	80.31852520	https://www.inaturalist.org/observations/92321606
237	Acrocephalus dumetorum	Blyth's Reed-warbler	2022/2/10	0	0	13.60480000	79.37305000	https://www.inaturalist.org/observations/112237291
238	Acrocephalus dumetorum	Blyth's Reed-warbler	2019/2/15	1	0	13.66004482	79.48968047	https://www.inaturalist.org/observations/55889045
239	Acrocephalus dumetorum	Blyth's Reed-warbler	2020/11/9	1	0	13.71792977	79.32248655	https://www.inaturalist.org/observations/64726119
240	Acrocephalus dumetorum	Blyth's Reed-warbler	2019/2/9	1	0	13.72629778	80.13074047	https://www.inaturalist.org/observations/55886782
241	Acrocephalus dumetorum	Blyth's Reed-warbler	2019/11/17	1	0	13.75772780	79.47021270	https://www.inaturalist.org/observations/35763437
242	Acrocephalus dumetorum	Blyth's Reed-warbler	2014/1/8	1	0	14.86613746	74.43937294	https://www.inaturalist.org/observations/108583919
243	Acrocephalus dumetorum	Blyth's Reed-warbler	2019/1/20	1	0	14.86613746	74.43937294	https://www.inaturalist.org/observations/109113923
244	Acrocephalus dumetorum	Blyth's Reed-warbler	2018/11/30	3	0	14.86613770	74.43937290	https://www.inaturalist.org/observations/126085000
245	Acrocephalus dumetorum	Blyth's Reed-warbler	2011/12/25	2	0	14.88090830	74.50688190	https://www.inaturalist.org/observations/96684175
246	Acrocephalus dumetorum	Blyth's Reed-warbler	2011/12/6	1	0	15.23611910	74.61728190	https://www.inaturalist.org/observations/41279911
247	Acrocephalus dumetorum	Blyth's Reed-warbler	2016/1/2	1	0	15.23611910	74.61728190	https://www.inaturalist.org/observations/41282799
248	Acrocephalus dumetorum	Blyth's Reed-warbler	2016/1/2	1	0	15.23611910	74.61728190	https://www.inaturalist.org/observations/41282800
249	Acrocephalus dumetorum	Blyth's Reed-warbler	2019/3/8	1	0	15.23611910	74.61728190	https://www.inaturalist.org/observations/102237327
250	Acrocephalus dumetorum	Blyth's Reed-warbler	2019/3/7	1	0	15.28426770	74.53021590	https://www.inaturalist.org/observations/56497356
251	Acrocephalus dumetorum	Blyth's Reed-warbler	2019/3/7	1	0	15.28426770	74.53021590	https://www.inaturalist.org/observations/56497357
252	Acrocephalus dumetorum	Blyth's Reed-warbler	2019/3/7	1	0	15.28426770	74.53021590	https://www.inaturalist.org/observations/56497360

253	Acrocephalus dumetorum	Blyth's Reed-warbler	2019/3/8	2	0	15.28426770	74.53021590	https://www.inaturalist.org/observations/56497415
254	Acrocephalus dumetorum	Blyth's Reed-warbler	2019/3/8	2	0	15.28426770	74.53021590	https://www.inaturalist.org/observations/56497420
255	Acrocephalus dumetorum	Blyth's Reed-warbler	2019/3/8	1	0	15.28426770	74.53021590	https://www.inaturalist.org/observations/56497434
256	Acrocephalus dumetorum	Blyth's Reed-warbler	2016/11/3	2	0	15.52680097	73.87086728	https://www.inaturalist.org/observations/37781509
257	Acrocephalus dumetorum	Blyth's Reed-warbler	2018/1/14	2	0	15.67789078	73.71019577	https://www.inaturalist.org/observations/19495903
258	Acrocephalus dumetorum	Blyth's Reed-warbler	2022/1/29	1	0	15.85416167	80.50854833	https://www.inaturalist.org/observations/105837258
259	Acrocephalus dumetorum	Blyth's Reed-warbler	2021/2/7	1	0	16.20022766	77.37198327	https://www.inaturalist.org/observations/81795462
260	Acrocephalus dumetorum	Blyth's Reed-warbler	2015/3/19	1	0	16.30689104	80.51252635	https://www.inaturalist.org/observations/87981026
261	Acrocephalus dumetorum	Blyth's Reed-warbler	2017/3/18	2	0	16.30689104	80.51252635	https://www.inaturalist.org/observations/98109606
262	Acrocephalus dumetorum	Blyth's Reed-warbler	2021/3/14	1	0	16.36004000	74.29583900	https://www.inaturalist.org/observations/71221806
263	Acrocephalus dumetorum	Blyth's Reed-warbler	2017/3/18	1	0	16.36906170	78.72911010	https://www.inaturalist.org/observations/136244504
264	Acrocephalus dumetorum	Blyth's Reed-warbler	2022/1/28	1	0	16.53580545	81.49850350	https://www.inaturalist.org/observations/105703681
265	Acrocephalus dumetorum	Blyth's Reed-warbler	2022/1/2	1	0	16.53976228	81.50539845	https://www.inaturalist.org/observations/104237069
266	Acrocephalus dumetorum	Blyth's Reed-warbler	2022/10/30	2	0	16.55473667	81.49606000	https://www.inaturalist.org/observations/140496486
267	Acrocephalus dumetorum	Blyth's Reed-warbler	2020/11/22	1	0	16.55493600	80.72975710	https://www.inaturalist.org/observations/92664026
268	Acrocephalus dumetorum	Blyth's Reed-warbler	2021/12/5	1	0	16.57559146	80.68230191	https://www.inaturalist.org/observations/104498315
269	Acrocephalus dumetorum	Blyth's Reed-warbler	2015/1/24	1	0	16.58784260	79.32380108	https://www.inaturalist.org/observations/87981027
270	Acrocephalus dumetorum	Blyth's Reed-warbler	2022/2/13	2	0	16.64674448	80.81858563	https://www.inaturalist.org/observations/106796050
271	Acrocephalus dumetorum	Blyth's Reed-warbler	2020/11/21	1	0	16.71847642	74.23877053	https://www.inaturalist.org/observations/65324605
272	Acrocephalus dumetorum	Blyth's Reed-warbler	2020/11/5	0	0	16.86603983	75.73659186	https://www.inaturalist.org/observations/64237411
273	Acrocephalus dumetorum	Blyth's Reed-warbler	2019/12/8	1	0	16.89121745	75.51323051	https://www.inaturalist.org/observations/48517650
274	Acrocephalus dumetorum	Blyth's Reed-warbler	2020/11/1	1	0	16.90655242	75.57091586	https://www.inaturalist.org/observations/63971089
275	Acrocephalus dumetorum	Blyth's Reed-warbler	2015/1/26	3	0	16.94806230	73.51523810	https://www.inaturalist.org/observations/107811356

276	Acrocephalus dumetorum	Blyth's Reed-warbler	2007/3/6	1	0	17.25430100	78.68076700	https://www.inaturalist.org/observations/26805253
277	Acrocephalus dumetorum	Blyth's Reed-warbler	2021/1/5	1	0	17.43004140	78.38945940	https://www.inaturalist.org/observations/69370906
278	Acrocephalus dumetorum	Blyth's Reed-warbler	2021/12/19	1	0	17.63782000	78.06452667	https://www.inaturalist.org/observations/121718423
279	Acrocephalus dumetorum	Blyth's Reed-warbler	2021/12/19	1	0	17.64092333	78.06890667	https://www.inaturalist.org/observations/121718371
280	Acrocephalus dumetorum	Blyth's Reed-warbler	2020/12/17	1	0	17.68046390	74.01826100	https://www.inaturalist.org/observations/69548177
281	Acrocephalus dumetorum	Blyth's Reed-warbler	2020/12/3	2	0	17.68046390	74.01826100	https://www.inaturalist.org/observations/105501151
282	Acrocephalus dumetorum	Blyth's Reed-warbler	2012/3/10	2	0	18.29158270	74.77408000	https://www.inaturalist.org/observations/56113760
283	Acrocephalus dumetorum	Blyth's Reed-warbler	2014/1/5	1	0	18.29894780	74.75425710	https://www.inaturalist.org/observations/41029782
284	Acrocephalus dumetorum	Blyth's Reed-warbler	2014/1/5	1	0	18.29894780	74.75425710	https://www.inaturalist.org/observations/41029798
285	Acrocephalus dumetorum	Blyth's Reed-warbler	2020/11/29	2	0	18.35312390	75.63916110	https://www.inaturalist.org/observations/75145471
286	Acrocephalus dumetorum	Blyth's Reed-warbler	2018/3/31	1	0	18.37586280	73.76916470	https://www.inaturalist.org/observations/44360079
287	Acrocephalus dumetorum	Blyth's Reed-warbler	2021/3/12	1	0	18.37586280	73.76916470	https://www.inaturalist.org/observations/71121907
288	Acrocephalus dumetorum	Blyth's Reed-warbler	2021/12/29	1	0	18.44690320	73.43054390	https://www.inaturalist.org/observations/104093577
289	Acrocephalus dumetorum	Blyth's Reed-warbler	2017/11/5	1	0	18.45783185	73.77355814	https://www.inaturalist.org/observations/8705764
290	Acrocephalus dumetorum	Blyth's Reed-warbler	2020/11/29	2	0	18.46242915	73.92131663	https://www.inaturalist.org/observations/66282175
291	Acrocephalus dumetorum	Blyth's Reed-warbler	2022/10/8	0	0	18.49064098	73.79870586	https://www.inaturalist.org/observations/137966214
292	Acrocephalus dumetorum	Blyth's Reed-warbler	2014/3/16	2	0	18.49183800	73.83643600	https://www.inaturalist.org/observations/17632411
293	Acrocephalus dumetorum	Blyth's Reed-warbler	2021/4/3	2	0	18.50920750	73.42647610	https://www.inaturalist.org/observations/75190582
294	Acrocephalus dumetorum	Blyth's Reed-warbler	2022/4/1	0	0	18.52210100	73.81377150	https://www.inaturalist.org/observations/139765298
295	Acrocephalus dumetorum	Blyth's Reed-warbler	2020/10/26	1	0	18.54855361	73.77981750	https://www.inaturalist.org/observations/63554455
296	Acrocephalus dumetorum	Blyth's Reed-warbler	2016/1/2	2	0	18.55403120	73.79045760	https://www.inaturalist.org/observations/141622230
297	Acrocephalus dumetorum	Blyth's Reed-warbler	2022/11/24	1	0	18.55811186	73.80363978	https://www.inaturalist.org/observations/143025482
298	Acrocephalus dumetorum	Blyth's Reed-warbler	2022/11/21	2	0	18.56481574	73.79278847	https://www.inaturalist.org/observations/142513151

299	Acrocephalus dumetorum	Blyth's Reed-warbler	2022/11/15	1	0	18.58766526	73.77658497	https://www.inaturalist.org/observations/142049923
300	Acrocephalus dumetorum	Blyth's Reed-warbler	2022/11/15	1	0	18.58766526	73.77658497	https://www.inaturalist.org/observations/142049927
301	Acrocephalus dumetorum	Blyth's Reed-warbler	2013/1/28	1	0	18.69440338	72.93132734	https://www.inaturalist.org/observations/104517108
302	Acrocephalus dumetorum	Blyth's Reed-warbler	2014/1/5	2	0	18.69448717	72.93082081	https://www.inaturalist.org/observations/104383279
303	Acrocephalus dumetorum	Blyth's Reed-warbler	2013/12/12	2	0	18.69463490	72.93121948	https://www.inaturalist.org/observations/104699650
304	Acrocephalus dumetorum	Blyth's Reed-warbler	2013/3/9	1	0	18.69467398	72.93123224	https://www.inaturalist.org/observations/104561561
305	Acrocephalus dumetorum	Blyth's Reed-warbler	2013/2/11	2	0	18.69475638	72.93139969	https://www.inaturalist.org/observations/53130032
306	Acrocephalus dumetorum	Blyth's Reed-warbler	2019/2/14	1	0	18.69554150	72.92532641	https://www.inaturalist.org/observations/103305851
307	Acrocephalus dumetorum	Blyth's Reed-warbler	2021/1/19	1	0	18.70122810	73.38855260	https://www.inaturalist.org/observations/78431690
308	Acrocephalus dumetorum	Blyth's Reed-warbler	2019/1/3	1	0	18.98865870	73.27117900	https://www.inaturalist.org/observations/102181499
309	Acrocephalus dumetorum	Blyth's Reed-warbler	2018/10/21	1	0	19.00678040	73.01469960	https://www.inaturalist.org/observations/44122295
310	Acrocephalus dumetorum	Blyth's Reed-warbler	2020/12/27	0	0	19.00678040	73.01469960	https://www.inaturalist.org/observations/67797514
311	Acrocephalus dumetorum	Blyth's Reed-warbler	2020/12/27	2	0	19.00678040	73.01469960	https://www.inaturalist.org/observations/75146521
312	Acrocephalus dumetorum	Blyth's Reed-warbler	2020/11/15	1	0	19.01659100	73.00593960	https://www.inaturalist.org/observations/64923591
313	Acrocephalus dumetorum	Blyth's Reed-warbler	2016/4/17	1	0	19.08397427	73.53655184	https://www.inaturalist.org/observations/7000713
314	Acrocephalus dumetorum	Blyth's Reed-warbler	2013/12/29	1	0	19.14190550	72.96462840	https://www.inaturalist.org/observations/41421625
315	Acrocephalus dumetorum	Blyth's Reed-warbler	2018/2/25	1	0	19.14190550	72.96462840	https://www.inaturalist.org/observations/56117017
316	Acrocephalus dumetorum	Blyth's Reed-warbler	2018/2/25	1	0	19.14190550	72.96462840	https://www.inaturalist.org/observations/56117022
317	Acrocephalus dumetorum	Blyth's Reed-warbler	2018/2/25	1	0	19.14190550	72.96462840	https://www.inaturalist.org/observations/56117026
318	Acrocephalus dumetorum	Blyth's Reed-warbler	2021/3/29	2	0	19.14190550	72.96462840	https://www.inaturalist.org/observations/72351687
319	Acrocephalus dumetorum	Blyth's Reed-warbler	2021/3/29	1	0	19.14190550	72.96462840	https://www.inaturalist.org/observations/72351695
320	Acrocephalus dumetorum	Blyth's Reed-warbler	2018/3/29	2	0	19.14192483	72.96462536	https://www.inaturalist.org/observations/56123704
321	Acrocephalus dumetorum	Blyth's Reed-warbler	2013/3/17	1	0	19.15055340	72.95648930	https://www.inaturalist.org/observations/41422458

322	Acrocephalus dumetorum	Blyth's Reed-warbler	2015/3/1	1	0	19.15055340	72.95648930	https://www.inaturalist.org/observations/41424517
323	Acrocephalus dumetorum	Blyth's Reed-warbler	2018/4/8	1	0	19.15055340	72.95648930	https://www.inaturalist.org/observations/44873461
324	Acrocephalus dumetorum	Blyth's Reed-warbler	2019/12/10	3	0	19.19094485	72.98145019	https://www.inaturalist.org/observations/136701597
325	Acrocephalus dumetorum	Blyth's Reed-warbler	2021/9/26	0	0	19.23063870	73.11032094	https://www.inaturalist.org/observations/96441676
326	Acrocephalus dumetorum	Blyth's Reed-warbler	2018/4/27	3	0	19.23119333	72.87065685	https://www.inaturalist.org/observations/11725673
327	Acrocephalus dumetorum	Blyth's Reed-warbler	2019/12/15	1	0	19.23376500	72.94458020	https://www.inaturalist.org/observations/36818606
328	Acrocephalus dumetorum	Blyth's Reed-warbler	2020/1/12	1	0	19.23653973	72.99878794	https://www.inaturalist.org/observations/37519188
329	Acrocephalus dumetorum	Blyth's Reed-warbler	2019/1/24	1	0	19.23700000	72.96400000	https://www.inaturalist.org/observations/56498317
330	Acrocephalus dumetorum	Blyth's Reed-warbler	2018/10/28	1	0	19.24515270	72.99792940	https://www.inaturalist.org/observations/75050259
331	Acrocephalus dumetorum	Blyth's Reed-warbler	2010/4/11	1	0	19.25697110	73.12395340	https://www.inaturalist.org/observations/42475130
332	Acrocephalus dumetorum	Blyth's Reed-warbler	2012/12/16	1	0	19.26803820	73.14045390	https://www.inaturalist.org/observations/41427654
333	Acrocephalus dumetorum	Blyth's Reed-warbler	2012/12/16	1	0	19.26803820	73.14045390	https://www.inaturalist.org/observations/41427680
334	Acrocephalus dumetorum	Blyth's Reed-warbler	2021/3/18	1	0	19.40330945	72.95786753	https://www.inaturalist.org/observations/71727627
335	Acrocephalus dumetorum	Blyth's Reed-warbler	2013/5/5	1	0	19.80419430	72.74955380	https://www.inaturalist.org/observations/32856367
336	Acrocephalus dumetorum	Blyth's Reed-warbler	2019/7/14	1	0	19.86903254	75.36057964	https://www.inaturalist.org/observations/110496497
337	Acrocephalus dumetorum	Blyth's Reed-warbler	2012/1/29	1	0	19.89522918	85.44423379	https://www.inaturalist.org/observations/20845384
338	Acrocephalus dumetorum	Blyth's Reed-warbler	2020/10/31	2	0	19.90734438	75.32153975	https://www.inaturalist.org/observations/63879789
339	Acrocephalus dumetorum	Blyth's Reed-warbler	2020/10/31	2	0	19.90734438	75.32153975	https://www.inaturalist.org/observations/63879904
340	Acrocephalus dumetorum	Blyth's Reed-warbler	2021/11/7	1	0	19.95247138	73.78284965	https://www.inaturalist.org/observations/100474505
341	Acrocephalus dumetorum	Blyth's Reed-warbler	2020/2/4	0	0	20.71172732	81.53439816	https://www.inaturalist.org/observations/38312274
342	Acrocephalus dumetorum	Blyth's Reed-warbler	2020/12/24	1	0	20.99373835	75.54336984	https://www.inaturalist.org/observations/67001828
343	Acrocephalus dumetorum	Blyth's Reed-warbler	2022/2/20	2	0	20.99947680	81.45188700	https://www.inaturalist.org/observations/141545829
344	Acrocephalus dumetorum	Blyth's Reed-warbler	2021/4/15	1	0	21.02616249	81.45505574	https://www.inaturalist.org/observations/74212656

345	Acrocephalus dumetorum	Blyth's Reed-warbler	2021/4/13	1	0	21.02649171	81.45980425	https://www.inaturalist.org/observations/73832387
346	Acrocephalus dumetorum	Blyth's Reed-warbler	2021/4/21	1	0	21.04034356	81.44709159	https://www.inaturalist.org/observations/74823136
347	Acrocephalus dumetorum	Blyth's Reed-warbler	2020/11/26	0	0	21.04208167	75.84359833	https://www.inaturalist.org/observations/65646483
348	Acrocephalus dumetorum	Blyth's Reed-warbler	2021/12/4	3	0	21.04218167	75.84752167	https://www.inaturalist.org/observations/102537829
349	Acrocephalus dumetorum	Blyth's Reed-warbler	2021/8/26	1	0	21.04749085	75.85351635	https://www.inaturalist.org/observations/92652764
350	Acrocephalus dumetorum	Blyth's Reed-warbler	2021/7/7	2	0	21.05914308	81.46057203	https://www.inaturalist.org/observations/86139903
351	Acrocephalus dumetorum	Blyth's Reed-warbler	2021/2/20	1	0	21.07367672	81.44190855	https://www.inaturalist.org/observations/69991729
352	Acrocephalus dumetorum	Blyth's Reed-warbler	2018/3/15	1	0	21.19406481	81.35094143	https://www.inaturalist.org/observations/95121458
353	Acrocephalus dumetorum	Blyth's Reed-warbler	2022/4/20	0	0	21.19406481	81.35094143	https://www.inaturalist.org/observations/143367858
354	Acrocephalus dumetorum	Blyth's Reed-warbler	2021/4/15	1	0	21.20607404	81.60739850	https://www.inaturalist.org/observations/74646664
355	Acrocephalus dumetorum	Blyth's Reed-warbler	2019/1/14	1	0	21.21456422	81.44687936	https://www.inaturalist.org/observations/78354030
356	Acrocephalus dumetorum	Blyth's Reed-warbler	2021/4/30	3	0	21.89483203	87.57942710	https://www.inaturalist.org/observations/116139886
357	Acrocephalus dumetorum	Blyth's Reed-warbler	2014/9/1	3	0	22.04370016	72.02045186	https://www.inaturalist.org/observations/47810962
358	Acrocephalus dumetorum	Blyth's Reed-warbler	2020/12/1	1	0	22.16194050	73.48404960	https://www.inaturalist.org/observations/96581850
359	Acrocephalus dumetorum	Blyth's Reed-warbler	2021/1/31	1	0	22.16194453	73.48404955	https://www.inaturalist.org/observations/68998406
360	Acrocephalus dumetorum	Blyth's Reed-warbler	2017/2/3	1	0	22.24669167	80.60390500	https://www.inaturalist.org/observations/5182915
361	Acrocephalus dumetorum	Blyth's Reed-warbler	2020/10/18	0	0	22.31784418	73.27948451	https://www.inaturalist.org/observations/62887123
362	Acrocephalus dumetorum	Blyth's Reed-warbler	2021/5/24	1	0	22.31839874	73.27914856	https://www.inaturalist.org/observations/136287192
363	Acrocephalus dumetorum	Blyth's Reed-warbler	2014/1/23	2	0	22.37974600	114.18960200	https://www.inaturalist.org/observations/20544447
364	Acrocephalus dumetorum	Blyth's Reed-warbler	2021/12/24	1	0	22.38805530	70.22202100	https://www.inaturalist.org/observations/136772372
365	Acrocephalus dumetorum	Blyth's Reed-warbler	2018/2/28	1	0	22.42240000	88.28990000	https://www.inaturalist.org/observations/69906736
366	Acrocephalus dumetorum	Blyth's Reed-warbler	2022/4/24	2	0	22.42260016	88.38098578	https://www.inaturalist.org/observations/113199431
367	Acrocephalus dumetorum	Blyth's Reed-warbler	2022/4/24	1	0	22.42260016	88.38098578	https://www.inaturalist.org/observations/115066837

368	Acrocephalus dumetorum	Blyth's Reed-warbler	2018/2/28	1	0	22.42380000	88.28850000	https://www.inaturalist.org/observations/69906750
369	Acrocephalus dumetorum	Blyth's Reed-warbler	2018/2/28	1	0	22.42390000	88.28850000	https://www.inaturalist.org/observations/69906745
370	Acrocephalus dumetorum	Blyth's Reed-warbler	2020/12/6	1	0	22.44354797	88.30676754	https://www.inaturalist.org/observations/66296713
371	Acrocephalus dumetorum	Blyth's Reed-warbler	2020/5/6	1	0	22.47355595	88.31298541	https://www.inaturalist.org/observations/131766666
372	Acrocephalus dumetorum	Blyth's Reed-warbler	2020/5/5	1	0	22.47363774	88.31293177	https://www.inaturalist.org/observations/131762167
373	Acrocephalus dumetorum	Blyth's Reed-warbler	2018/11/6	3	0	22.51223175	88.35585258	https://www.inaturalist.org/observations/97878470
374	Acrocephalus dumetorum	Blyth's Reed-warbler	2021/12/31	0	0	22.51422540	70.15451650	https://www.inaturalist.org/observations/136536625
375	Acrocephalus dumetorum	Blyth's Reed-warbler	2020/12/4	0	0	22.51743010	70.15337860	https://www.inaturalist.org/observations/136535263
376	Acrocephalus dumetorum	Blyth's Reed-warbler	2020/4/26	5	0	22.53662761	88.36617405	https://www.inaturalist.org/observations/126317224
377	Acrocephalus dumetorum	Blyth's Reed-warbler	2021/8/15	1	0	22.54981204	72.60791503	https://www.inaturalist.org/observations/91361664
378	Acrocephalus dumetorum	Blyth's Reed-warbler	2019/12/24	1	0	22.55218750	72.61806250	https://www.inaturalist.org/observations/56410562
379	Acrocephalus dumetorum	Blyth's Reed-warbler	2017/1/8	1	0	22.58162751	88.28499781	https://www.inaturalist.org/observations/100292549
380	Acrocephalus dumetorum	Blyth's Reed-warbler	2022/11/22	1	0	22.60932126	88.46348039	https://www.inaturalist.org/observations/143944337
381	Acrocephalus dumetorum	Blyth's Reed-warbler	2021/4/9	2	0	22.62015305	73.45790841	https://www.inaturalist.org/observations/97887966
382	Acrocephalus dumetorum	Blyth's Reed-warbler	2021/4/9	2	0	22.62015305	73.45790841	https://www.inaturalist.org/observations/104174610
383	Acrocephalus dumetorum	Blyth's Reed-warbler	2021/10/17	1	0	22.62017833	73.45859667	https://www.inaturalist.org/observations/122151713
384	Acrocephalus dumetorum	Blyth's Reed-warbler	2021/12/31	1	0	22.62020333	73.45865333	https://www.inaturalist.org/observations/121711709
385	Acrocephalus dumetorum	Blyth's Reed-warbler	2021/12/23	0	0	22.62022833	73.45850667	https://www.inaturalist.org/observations/121714552
386	Acrocephalus dumetorum	Blyth's Reed-warbler	2022/3/11	3	0	22.62025333	73.45839333	https://www.inaturalist.org/observations/119756696
387	Acrocephalus dumetorum	Blyth's Reed-warbler	2022/1/11	1	0	22.62025833	73.45830833	https://www.inaturalist.org/observations/120791432
388	Acrocephalus dumetorum	Blyth's Reed-warbler	2021/9/13	3	0	22.62031333	73.45877000	https://www.inaturalist.org/observations/122275236
389	Acrocephalus dumetorum	Blyth's Reed-warbler	2021/12/30	2	0	22.62037667	73.45848500	https://www.inaturalist.org/observations/121714648
390	Acrocephalus dumetorum	Blyth's Reed-warbler	2022/1/19	1	0	22.62043500	73.45850333	https://www.inaturalist.org/observations/120521863

391	Acrocephalus dumetorum	Blyth's Reed-warbler	2022/1/30	2	0	22.62052833	73.45849000	https://www.inaturalist.org/observations/120510467
392	Acrocephalus dumetorum	Blyth's Reed-warbler	2022/1/2	0	0	22.62060667	73.45881500	https://www.inaturalist.org/observations/121711734
393	Acrocephalus dumetorum	Blyth's Reed-warbler	2022/2/6	1	0	22.62061333	73.45854167	https://www.inaturalist.org/observations/120502433
394	Acrocephalus dumetorum	Blyth's Reed-warbler	2021/9/18	0	0	22.62063167	73.45871167	https://www.inaturalist.org/observations/122273250
395	Acrocephalus dumetorum	Blyth's Reed-warbler	2021/9/14	0	0	22.62070167	73.45878000	https://www.inaturalist.org/observations/122275224
396	Acrocephalus dumetorum	Blyth's Reed-warbler	2022/1/7	1	0	22.62540553	88.41226369	https://www.inaturalist.org/observations/104557486
397	Acrocephalus dumetorum	Blyth's Reed-warbler	2022/1/9	1	0	22.74791167	74.33546167	https://www.inaturalist.org/observations/120793624
398	Acrocephalus dumetorum	Blyth's Reed-warbler	2021/11/28	2	0	22.80416658	88.33392341	https://www.inaturalist.org/observations/126925296
399	Acrocephalus dumetorum	Blyth's Reed-warbler	2022/9/4	1	0	22.83463307	72.06067298	https://www.inaturalist.org/observations/135760028
400	Acrocephalus dumetorum	Blyth's Reed-warbler	2022/1/9	1	0	22.83819000	74.12402500	https://www.inaturalist.org/observations/120791413
401	Acrocephalus dumetorum	Blyth's Reed-warbler	2022/1/25	2	0	22.84517637	88.34970742	https://www.inaturalist.org/observations/105600711
402	Acrocephalus dumetorum	Blyth's Reed-warbler	2019/5/5	1	0	22.84519989	88.31391814	https://www.inaturalist.org/observations/100462169
403	Acrocephalus dumetorum	Blyth's Reed-warbler	2015/2/7	3	0	22.94958100	86.80465200	http://www.inaturalist.org/observations/1251066
404	Acrocephalus dumetorum	Blyth's Reed-warbler	2021/1/24	2	0	22.95275200	88.53729439	https://www.inaturalist.org/observations/103892009
405	Acrocephalus dumetorum	Blyth's Reed-warbler	2022/1/2	1	0	22.95505943	88.53746586	https://www.inaturalist.org/observations/104752088
406	Acrocephalus dumetorum	Blyth's Reed-warbler	2022/1/2	1	0	22.95505943	88.53746586	https://www.inaturalist.org/observations/104753270
407	Acrocephalus dumetorum	Blyth's Reed-warbler	2019/11/23	1	0	22.95507394	88.53744507	https://www.inaturalist.org/observations/36220487
408	Acrocephalus dumetorum	Blyth's Reed-warbler	2019/11/23	2	0	22.95507394	88.53744507	https://www.inaturalist.org/observations/36220512
409	Acrocephalus dumetorum	Blyth's Reed-warbler	2019/3/25	2	0	22.98689440	87.00177045	https://www.inaturalist.org/observations/21591586
410	Acrocephalus dumetorum	Blyth's Reed-warbler	2017/11/10	1	0	22.98881745	87.00500550	https://www.inaturalist.org/observations/13388133
411	Acrocephalus dumetorum	Blyth's Reed-warbler	2020/11/23	0	0	22.99064958	86.99983091	https://www.inaturalist.org/observations/65470600
412	Acrocephalus dumetorum	Blyth's Reed-warbler	2021/11/28	1	0	22.99106239	87.00014346	https://www.inaturalist.org/observations/102160699
413	Acrocephalus dumetorum	Blyth's Reed-warbler	2019/3/27	2	0	23.18608990	86.74776600	https://www.inaturalist.org/observations/21700620

414	Acrocephalus dumetorum	Blyth's Reed-warbler	2021/10/25	1	0	23.20675800	77.46016200	https://www.inaturalist.org/observations/100852712
415	Acrocephalus dumetorum	Blyth's Reed-warbler	2015/1/2	2	0	23.24199970	69.66693240	https://www.inaturalist.org/observations/69256996
416	Acrocephalus dumetorum	Blyth's Reed-warbler	2015/11/25	2	0	23.24681200	87.81896300	http://www.inaturalist.org/observations/2425354
417	Acrocephalus dumetorum	Blyth's Reed-warbler	2019/4/21	1	0	23.35274181	69.23164426	https://www.inaturalist.org/observations/137051675
418	Acrocephalus dumetorum	Blyth's Reed-warbler	2019/4/7	1	0	23.47233001	87.30178515	https://www.inaturalist.org/observations/22135914
419	Acrocephalus dumetorum	Blyth's Reed-warbler	2018/2/15	0	0	23.57704690	87.34384290	https://www.inaturalist.org/observations/9956666
420	Acrocephalus dumetorum	Blyth's Reed-warbler	2018/11/19	1	0	23.58879833	87.33467500	https://www.inaturalist.org/observations/18498447
421	Acrocephalus dumetorum	Blyth's Reed-warbler	2018/3/1	1	0	23.58936200	87.36866610	https://www.inaturalist.org/observations/12712106
422	Acrocephalus dumetorum	Blyth's Reed-warbler	2018/12/8	1	0	23.59155167	87.34298333	https://www.inaturalist.org/observations/18897488
423	Acrocephalus dumetorum	Blyth's Reed-warbler	2022/2/17	1	0	23.63010180	86.92689188	https://www.inaturalist.org/observations/106916943
424	Acrocephalus dumetorum	Blyth's Reed-warbler	2021/12/11	2	0	25.19313889	73.91811661	https://www.inaturalist.org/observations/102870106
425	Acrocephalus dumetorum	Blyth's Reed-warbler	2022/10/26	0	0	25.27482974	85.45689650	https://www.inaturalist.org/observations/141752440
426	Acrocephalus dumetorum	Blyth's Reed-warbler	2022/4/28	1	0	25.31011236	91.90259714	https://www.inaturalist.org/observations/113493492
427	Acrocephalus dumetorum	Blyth's Reed-warbler	2022/4/20	1	0	25.31100830	91.90020829	https://www.inaturalist.org/observations/112532810
428	Acrocephalus dumetorum	Blyth's Reed-warbler	2022/5/1	0	0	25.31105588	91.90021701	https://www.inaturalist.org/observations/114107511
429	Acrocephalus dumetorum	Blyth's Reed-warbler	2018/4/2	3	0	25.45090200	92.20891090	https://www.inaturalist.org/observations/19526156
430	Acrocephalus dumetorum	Blyth's Reed-warbler	2018/4/2	3	0	25.45090200	92.20891100	https://www.inaturalist.org/observations/15715801
431	Acrocephalus dumetorum	Blyth's Reed-warbler	2013/2/8	0	0	26.58517567	93.17021897	https://www.inaturalist.org/observations/79802203
432	Acrocephalus dumetorum	Blyth's Reed-warbler	2022/10/22	0	0	26.85045930	75.76277020	https://www.inaturalist.org/observations/139655459
433	Acrocephalus dumetorum	Blyth's Reed-warbler	2019/3/11	1	0	27.15692000	77.52365100	https://www.inaturalist.org/observations/42552797
434	Acrocephalus dumetorum	Blyth's Reed-warbler	2012/11/13	1	0	27.15926900	77.52319990	https://www.inaturalist.org/observations/31165299
435	Acrocephalus dumetorum	Blyth's Reed-warbler	2021/2/13	1	0	27.15926900	77.52319990	https://www.inaturalist.org/observations/70138153
436	Acrocephalus dumetorum	Blyth's Reed-warbler	2014/10/18	2	0	27.15926900	77.52319990	https://www.inaturalist.org/observations/88831600

437	Acrocephalus dumetorum	Blyth's Reed-warbler	2022/3/13	2	0	27.15926900	77.52319990	https://www.inaturalist.org/observations/109825126
438	Acrocephalus dumetorum	Blyth's Reed-warbler	2017/2/14	1	0	27.18076000	77.51707833	https://www.inaturalist.org/observations/5235295
439	Acrocephalus dumetorum	Blyth's Reed-warbler	2017/2/15	3	0	27.18532833	77.50642167	https://www.inaturalist.org/observations/5241245
440	Acrocephalus dumetorum	Blyth's Reed-warbler	2019/10/13	2	0	28.49084489	77.25999915	https://www.inaturalist.org/observations/34300687
441	Acrocephalus dumetorum	Blyth's Reed-warbler	2021/4/23	1	0	28.51617579	77.38643602	https://www.inaturalist.org/observations/78328150
442	Acrocephalus dumetorum	Blyth's Reed-warbler	2020/4/22	2	0	28.56816830	77.33184749	https://www.inaturalist.org/observations/43078991
443	Acrocephalus dumetorum	Blyth's Reed-warbler	2021/9/4	2	0	28.63864380	77.07206000	https://www.inaturalist.org/observations/93716353
444	Acrocephalus dumetorum	Blyth's Reed-warbler	2022/5/6	0	0	28.63864380	77.07206000	https://www.inaturalist.org/observations/115862355
445	Acrocephalus dumetorum	Blyth's Reed-warbler	2022/9/15	1	0	28.68702300	77.31772460	https://www.inaturalist.org/observations/135081861
446	Acrocephalus dumetorum	Blyth's Reed-warbler	2021/5/3	1	0	29.61269140	78.81177360	https://www.inaturalist.org/observations/79267806
447	Acrocephalus dumetorum	Blyth's Reed-warbler	2021/5/3	1	0	29.61269140	78.81177360	https://www.inaturalist.org/observations/79741845
448	Acrocephalus dumetorum	Blyth's Reed-warbler	2021/5/3	1	0	29.61269140	78.81177360	https://www.inaturalist.org/observations/79961333
449	Acrocephalus dumetorum	Blyth's Reed-warbler	2021/5/20	1	0	30.31770112	78.05934191	https://www.inaturalist.org/observations/92883819
450	Acrocephalus dumetorum	Blyth's Reed-warbler	2020/5/6	2	0	30.31843249	78.05791095	https://www.inaturalist.org/observations/45250008

No	scientific name	common name	date	NIA	NID	latitude	longitude	url
1	Lanius cristatus	Brown Shrike	2021/12/30	3	0	5.98347500	80.37292500	https://www.inaturalist.org/observations/104021688
2	Lanius cristatus	Brown Shrike	2022/2/13	1	0	6.01270000	80.26061111	https://www.inaturalist.org/observations/144619426
3	Lanius cristatus	Brown Shrike	2019/3/6	3	0	6.04893152	80.85721313	https://www.inaturalist.org/observations/22085745
4	Lanius cristatus	Brown Shrike	2019/12/22	4	0	6.05903785	80.31588508	https://www.inaturalist.org/observations/95056597
5	Lanius cristatus	Brown Shrike	2020/2/15	4	0	6.07710000	80.93132000	https://www.inaturalist.org/observations/39488086
6	Lanius cristatus	Brown Shrike	2018/3/25	4	0	6.11125892	81.06659424	https://www.inaturalist.org/observations/69669029
7	Lanius cristatus	Brown Shrike	2020/1/24	4	0	6.17507693	81.21627774	https://www.inaturalist.org/observations/41178570
8	Lanius cristatus	Brown Shrike	2016/4/6	2	0	6.17612083	81.21685028	https://www.inaturalist.org/observations/127818017
9	Lanius cristatus	Brown Shrike	2020/11/28	4	0	6.18266536	81.20492807	https://www.inaturalist.org/observations/65824194
10	Lanius cristatus	Brown Shrike	2022/2/21	3	0	6.18458018	81.20182462	https://www.inaturalist.org/observations/108886349
11	Lanius cristatus	Brown Shrike	2020/2/16	5	0	6.18561167	81.22480333	https://www.inaturalist.org/observations/39488321
12	Lanius cristatus	Brown Shrike	2015/11/11	4	0	6.19742233	81.21126595	https://www.inaturalist.org/observations/63709844
13	Lanius cristatus	Brown Shrike	2015/11/11	4	0	6.19742233	81.21126595	https://www.inaturalist.org/observations/63709845
14	Lanius cristatus	Brown Shrike	2020/1/28	2	0	6.19884100	81.21095500	https://www.inaturalist.org/observations/38134195
15	Lanius cristatus	Brown Shrike	2018/12/14	3	0	6.19901600	81.21048100	https://www.inaturalist.org/observations/19099040
16	Lanius cristatus	Brown Shrike	2019/10/19	4	0	6.22418210	80.11383690	https://www.inaturalist.org/observations/34767291
17	Lanius cristatus	Brown Shrike	2020/10/20	6	0	6.23051895	80.11060282	https://www.inaturalist.org/observations/64459962
18	Lanius cristatus	Brown Shrike	2018/10/24	3	0	6.26849837	81.38951598	https://www.inaturalist.org/observations/18136668
19	Lanius cristatus lucionensis	Brown Shrike	2020/1/28	2	0	6.27841200	81.40194100	https://www.inaturalist.org/observations/38542473
20	Lanius cristatus	Brown Shrike	2020/1/31	4	0	6.27842205	81.40193224	https://www.inaturalist.org/observations/40045621
21	Lanius cristatus	Brown Shrike	2013/11/24	4	0	6.28666155	81.28148924	https://www.inaturalist.org/observations/6985904
22	Lanius cristatus	Brown Shrike	2017/2/2	1	0	6.29283657	81.43048704	https://www.inaturalist.org/observations/136212810

23	Lanius cristatus	Brown Shrike	2018/1/13	6	0	6.30744333	81.44434500	https://www.inaturalist.org/observations/41520801
24	Lanius cristatus	Brown Shrike	2015/1/20	3	0	6.30763000	81.44494833	https://www.inaturalist.org/observations/106737599
25	Lanius cristatus	Brown Shrike	2022/4/25	2	0	6.31255320	81.40243271	https://www.inaturalist.org/observations/116471828
26	Lanius cristatus	Brown Shrike	2022/2/17	1	0	6.32828580	81.43741518	https://www.inaturalist.org/observations/107540981
27	Lanius cristatus	Brown Shrike	2022/2/17	2	0	6.33247519	81.42259564	https://www.inaturalist.org/observations/107543129
28	Lanius cristatus	Brown Shrike	2022/2/7	0	0	6.34159167	81.49611667	https://www.inaturalist.org/observations/144499134
29	Lanius cristatus	Brown Shrike	2020/1/14	1	0	6.34417211	81.49769615	https://www.inaturalist.org/observations/132846711
30	Lanius cristatus	Brown Shrike	2022/2/10	1	0	6.35115833	80.56822778	https://www.inaturalist.org/observations/144613114
31	Lanius cristatus	Brown Shrike	2014/2/20	5	0	6.35778780	80.48895520	https://www.inaturalist.org/observations/39273358
32	Lanius cristatus	Brown Shrike	2022/2/9	3	0	6.37562400	80.53881870	https://www.inaturalist.org/observations/108381340
33	Lanius cristatus	Brown Shrike	2022/3/21	3	0	6.37600167	81.49217833	https://www.inaturalist.org/observations/120876802
34	Lanius cristatus	Brown Shrike	2018/1/13	5	0	6.38295000	81.48565000	https://www.inaturalist.org/observations/41520809
35	Lanius cristatus	Brown Shrike	2020/1/24	3	0	6.38847009	80.59493680	https://www.inaturalist.org/observations/37917938
36	Lanius cristatus	Brown Shrike	2020/1/24	3	0	6.38867764	80.59485208	https://www.inaturalist.org/observations/37910876
37	Lanius cristatus	Brown Shrike	2020/1/24	3	0	6.38889442	80.59526121	https://www.inaturalist.org/observations/37917905
38	Lanius cristatus	Brown Shrike	2020/1/24	3	0	6.38972333	80.50138861	https://www.inaturalist.org/observations/38251244
39	Lanius cristatus	Brown Shrike	2021/1/2	3	0	6.38981822	80.59686628	https://www.inaturalist.org/observations/68941725
40	Lanius cristatus	Brown Shrike	2022/2/9	1	0	6.39036389	80.59811667	https://www.inaturalist.org/observations/144613100
41	Lanius cristatus	Brown Shrike	2014/2/13	3	0	6.39821167	81.46314335	https://www.inaturalist.org/observations/37173239
42	Lanius cristatus	Brown Shrike	2019/2/10	3	0	6.41855041	80.82393799	https://www.inaturalist.org/observations/89523285
43	Lanius cristatus	Brown Shrike	2019/1/10	2	0	6.42642101	80.81708538	https://www.inaturalist.org/observations/144267449
44	Lanius cristatus	Brown Shrike	2017/2/6	5	0	6.42676979	81.30793531	https://www.inaturalist.org/observations/54041226
45	Lanius cristatus	Brown Shrike	2017/2/7	7	0	6.42677800	81.30804700	https://www.inaturalist.org/observations/56921114

46	Lanius cristatus	Brown Shrike	2017/2/7	5	0	6.42678642	81.30804420	https://www.inaturalist.org/observations/54041264
47	Lanius cristatus	Brown Shrike	2012/12/11	7	0	6.42678642	81.30804420	https://www.inaturalist.org/observations/54634044
48	Lanius cristatus	Brown Shrike	2017/12/25	3	0	6.43305149	80.41435658	https://www.inaturalist.org/observations/10466415
49	Lanius cristatus	Brown Shrike	2016/4/16	3	0	6.43603510	80.41958790	https://www.inaturalist.org/observations/58176620
50	Lanius cristatus	Brown Shrike	2020/1/27	5	0	6.44045560	80.89099847	https://www.inaturalist.org/observations/38023489
51	Lanius cristatus	Brown Shrike	2022/2/6	2	0	6.44771150	80.89262100	https://www.inaturalist.org/observations/107503376
52	Lanius cristatus	Brown Shrike	2018/12/10	3	0	6.45353771	80.89625078	https://www.inaturalist.org/observations/19327595
53	Lanius cristatus	Brown Shrike	2012/3/5	4	0	6.45460310	80.89238783	https://www.inaturalist.org/observations/64525765
54	Lanius cristatus	Brown Shrike	2019/12/23	4	0	6.45794226	81.21933959	https://www.inaturalist.org/observations/36961029
55	Lanius cristatus	Brown Shrike	2022/2/6	1	0	6.46331389	80.91565000	https://www.inaturalist.org/observations/144484839
56	Lanius cristatus	Brown Shrike	2022/2/6	1	0	6.46663889	80.92591944	https://www.inaturalist.org/observations/144484893
57	Lanius cristatus	Brown Shrike	2020/2/17	2	0	6.47201478	80.87867316	https://www.inaturalist.org/observations/90820124
58	Lanius cristatus	Brown Shrike	2017/12/27	3	0	6.48910000	80.86580000	https://www.inaturalist.org/observations/10793886
59	Lanius cristatus	Brown Shrike	2011/1/20	4	0	6.49425641	80.89584688	https://www.inaturalist.org/observations/57908091
60	Lanius cristatus	Brown Shrike	2021/12/5	3	0	6.52084466	80.88433664	https://www.inaturalist.org/observations/102529930
61	Lanius cristatus	Brown Shrike	2021/12/6	3	0	6.52469802	80.88317592	https://www.inaturalist.org/observations/102600633
62	Lanius cristatus	Brown Shrike	2020/3/31	5	0	6.53272000	80.14151117	https://www.inaturalist.org/observations/41688166
63	Lanius cristatus	Brown Shrike	2020/10/26	6	0	6.60163391	79.95267637	https://www.inaturalist.org/observations/63801388
64	Lanius cristatus	Brown Shrike	2020/4/2	4	0	6.60175598	79.95271125	https://www.inaturalist.org/observations/41340655
65	Lanius cristatus	Brown Shrike	2021/10/23	4	0	6.74757920	80.36864880	https://www.inaturalist.org/observations/99073785
66	Lanius cristatus	Brown Shrike	2022/3/19	2	0	6.77216000	80.88449167	https://www.inaturalist.org/observations/110185533
67	Lanius cristatus	Brown Shrike	2021/12/28	2	0	6.83660663	79.89268285	https://www.inaturalist.org/observations/104167785
68	Lanius cristatus	Brown Shrike	2022/1/20	2	0	6.83667750	80.09696419	https://www.inaturalist.org/observations/129575515

69	Lanius cristatus	Brown Shrike	2017/3/10	2	0	6.86313523	81.04749101	https://www.inaturalist.org/observations/142455138
70	Lanius cristatus	Brown Shrike	2019/10/5	3	0	6.87289047	81.04663700	https://www.inaturalist.org/observations/34401991
71	Lanius cristatus	Brown Shrike	2022/1/15	1	0	6.87783813	79.93120844	https://www.inaturalist.org/observations/107104367
72	Lanius cristatus	Brown Shrike	2020/11/26	7	0	6.88675520	79.94759990	https://www.inaturalist.org/observations/65680128
73	Lanius cristatus	Brown Shrike	2022/1/23	3	0	6.89659474	79.97853443	https://www.inaturalist.org/observations/105413857
74	Lanius cristatus	Brown Shrike	2022/11/5	1	0	6.89857435	79.97868331	https://www.inaturalist.org/observations/141132633
75	Lanius cristatus	Brown Shrike	2022/2/6	2	0	6.89930413	79.97858430	https://www.inaturalist.org/observations/106219009
76	Lanius cristatus	Brown Shrike	2020/1/4	4	0	6.90063073	81.06519210	https://www.inaturalist.org/observations/37294938
77	Lanius cristatus	Brown Shrike	2015/1/29	7	0	6.92777336	80.82151070	https://www.inaturalist.org/observations/49033617
78	Lanius cristatus	Brown Shrike	2019/4/24	3	0	6.96797380	80.77155570	https://www.inaturalist.org/observations/23026035
79	Lanius cristatus	Brown Shrike	2014/11/24	4	0	6.96905630	80.76820450	https://www.inaturalist.org/observations/71308499
80	Lanius cristatus	Brown Shrike	2018/12/7	2	0	6.97006624	80.76761347	https://www.inaturalist.org/observations/108652634
81	Lanius cristatus	Brown Shrike	2018/1/1	3	0	6.97169770	80.76833577	https://www.inaturalist.org/observations/15184940
82	Lanius cristatus	Brown Shrike	2017/10/28	6	0	7.26392179	80.58622513	https://www.inaturalist.org/observations/37120630
83	Lanius cristatus	Brown Shrike	2022/2/4	2	0	7.27313333	80.59778333	https://www.inaturalist.org/observations/144447935
84	Lanius cristatus	Brown Shrike	2018/10/27	2	0	7.28426445	79.84239872	https://www.inaturalist.org/observations/18347905
85	Lanius cristatus	Brown Shrike	2015/10/19	3	0	7.30329573	80.64245566	https://www.inaturalist.org/observations/37924470
86	Lanius cristatus	Brown Shrike	2015/11/21	3	0	7.30329573	80.64245566	https://www.inaturalist.org/observations/38069738
87	Lanius cristatus	Brown Shrike	2020/2/10	4	0	7.34284167	80.77466333	https://www.inaturalist.org/observations/39424822
88	Lanius cristatus	Brown Shrike	2020/2/9	3	0	7.37781833	80.81302500	https://www.inaturalist.org/observations/39424519
89	Lanius cristatus	Brown Shrike	2022/10/15	1	0	7.47767174	80.32696598	https://www.inaturalist.org/observations/138811722
90	Lanius cristatus	Brown Shrike	2017/11/29	4	0	7.87305400	80.77179700	https://www.inaturalist.org/observations/9951502
91	Lanius cristatus	Brown Shrike	2017/11/29	4	0	7.87305400	80.77179700	https://www.inaturalist.org/observations/9951571

92	Lanius cristatus	Brown Shrike	2018/12/1	7	0	7.95499918	80.76522529	https://www.inaturalist.org/observations/68546241
93	Lanius cristatus	Brown Shrike	2019/11/7	2	0	7.95739229	80.75552622	https://www.inaturalist.org/observations/80341827
94	Lanius cristatus	Brown Shrike	2018/12/3	2	0	7.96932370	80.76097739	https://www.inaturalist.org/observations/108584828
95	Lanius cristatus	Brown Shrike	2020/2/7	2	0	8.02230833	80.82710333	https://www.inaturalist.org/observations/39243463
96	Lanius cristatus lucionensis	Brown Shrike	2018/12/16	2	0	8.03263702	80.74727805	https://www.inaturalist.org/observations/19569536
97	Lanius cristatus	Brown Shrike	2021/10/2	2	0	8.12902970	77.36402570	https://www.inaturalist.org/observations/98698063
98	Lanius cristatus	Brown Shrike	2020/2/5	3	0	8.20876667	80.07209000	https://www.inaturalist.org/observations/39241213
99	Lanius cristatus	Brown Shrike	2021/11/10	2	0	8.24014151	77.50143631	https://www.inaturalist.org/observations/104865048
100	Lanius cristatus	Brown Shrike	2021/11/10	2	0	8.27058248	77.56396682	https://www.inaturalist.org/observations/104865072
101	Lanius cristatus	Brown Shrike	2021/11/10	2	0	8.27683059	77.54436770	https://www.inaturalist.org/observations/104864073
102	Lanius cristatus	Brown Shrike	2021/11/10	2	0	8.34177640	77.50973452	https://www.inaturalist.org/observations/104865063
103	Lanius cristatus	Brown Shrike	2021/11/8	1	0	8.34281898	77.56256002	https://www.inaturalist.org/observations/105085907
104	Lanius cristatus	Brown Shrike	2017/10/16	2	0	8.37812190	77.40802860	https://www.inaturalist.org/observations/61497302
105	Lanius cristatus	Brown Shrike	2021/11/10	2	0	8.38176511	77.42395356	https://www.inaturalist.org/observations/104865073
106	Lanius cristatus	Brown Shrike	2021/11/8	3	0	8.39959276	77.53527051	https://www.inaturalist.org/observations/102535064
107	Lanius cristatus	Brown Shrike	2022/3/27	1	0	8.41101220	77.39138770	https://www.inaturalist.org/observations/110469982
108	Lanius cristatus	Brown Shrike	2020/12/16	2	0	8.44109221	76.98304720	https://www.inaturalist.org/observations/81162999
109	Lanius cristatus	Brown Shrike	2021/11/20	2	0	8.44176744	76.98298082	https://www.inaturalist.org/observations/101508870
110	Lanius cristatus	Brown Shrike	2022/1/17	1	0	8.44442489	76.98398128	https://www.inaturalist.org/observations/105139690
111	Lanius cristatus	Brown Shrike	2022/1/16	2	0	8.44503245	76.98461562	https://www.inaturalist.org/observations/105081104
112	Lanius cristatus	Brown Shrike	2022/2/28	4	0	8.44885559	76.98348004	https://www.inaturalist.org/observations/107686557
113	Lanius cristatus	Brown Shrike	2021/10/23	3	0	8.45391490	76.97329270	https://www.inaturalist.org/observations/99543673
114	Lanius cristatus	Brown Shrike	2022/1/26	1	0	8.49270650	77.75093030	https://www.inaturalist.org/observations/105901526

115	Lanius cristatus	Brown Shrike	2020/1/19	3	0	8.52243527	76.90322399	https://www.inaturalist.org/observations/67015623
116	Lanius cristatus	Brown Shrike	2020/12/12	0	0	8.57353356	81.23815978	https://www.inaturalist.org/observations/66466091
117	Lanius cristatus	Brown Shrike	2022/10/27	1	0	8.68834000	77.30946740	https://www.inaturalist.org/observations/141257452
118	Lanius cristatus	Brown Shrike	2022/11/2	1	0	8.68834000	77.30946740	https://www.inaturalist.org/observations/141259565
119	Lanius cristatus	Brown Shrike	2020/2/22	3	0	8.68989630	77.17067240	https://www.inaturalist.org/observations/39243332
120	Lanius cristatus	Brown Shrike	2015/10/27	3	0	8.69969770	77.63132000	https://www.inaturalist.org/observations/23905867
121	Lanius cristatus	Brown Shrike	2022/3/17	4	0	8.75994220	77.11687530	https://www.inaturalist.org/observations/108936503
122	Lanius cristatus	Brown Shrike	2019/4/2	6	0	8.77136208	81.13615356	https://www.inaturalist.org/observations/61253497
123	Lanius cristatus	Brown Shrike	2020/12/16	2	0	8.87002778	76.60139722	https://www.inaturalist.org/observations/66755195
124	Lanius cristatus	Brown Shrike	2018/10/17	5	0	8.87006725	76.60143861	https://www.inaturalist.org/observations/65888075
125	Lanius cristatus	Brown Shrike	2022/2/14	1	0	8.93173360	79.92146080	https://www.inaturalist.org/observations/107748946
126	Lanius cristatus	Brown Shrike	2022/2/5	1	0	9.01135633	76.66208893	https://www.inaturalist.org/observations/106836245
127	Lanius cristatus	Brown Shrike	2019/1/6	4	0	9.29536875	76.51159113	https://www.inaturalist.org/observations/65921296
128	Lanius cristatus	Brown Shrike	2022/4/30	3	0	9.30757790	77.06589460	https://www.inaturalist.org/observations/130740276
129	Lanius cristatus	Brown Shrike	2021/10/28	3	0	9.40061000	76.55001500	https://www.inaturalist.org/observations/102158889
130	Lanius cristatus	Brown Shrike	2018/1/21	2	0	9.40206960	76.42560510	https://www.inaturalist.org/observations/67397429
131	Lanius cristatus	Brown Shrike	2019/1/24	4	0	9.46458000	76.97067806	https://www.inaturalist.org/observations/47238636
132	Lanius cristatus	Brown Shrike	2022/2/19	2	0	9.53872270	76.51124710	https://www.inaturalist.org/observations/129432377
133	Lanius cristatus	Brown Shrike	2022/2/19	3	0	9.53872270	76.51124710	https://www.inaturalist.org/observations/131229049
134	Lanius cristatus	Brown Shrike	2021/1/26	4	0	9.54311003	76.50820199	https://www.inaturalist.org/observations/68752069
135	Lanius cristatus	Brown Shrike	2020/10/8	4	0	9.54385793	76.62595097	https://www.inaturalist.org/observations/62477950
136	Lanius cristatus	Brown Shrike	2014/12/25	2	0	9.56286103	76.41840311	https://www.inaturalist.org/observations/78549784
137	Lanius cristatus	Brown Shrike	2018/4/4	5	0	9.56561399	76.43993062	https://www.inaturalist.org/observations/66075736

138	Lanius cristatus	Brown Shrike	2018/12/29	4	0	9.56801469	76.44109807	https://www.inaturalist.org/observations/66031722
139	Lanius cristatus	Brown Shrike	2019/2/8	4	0	9.57872431	76.52783509	https://www.inaturalist.org/observations/41014346
140	Lanius cristatus	Brown Shrike	2012/12/14	6	0	9.58529902	77.18081024	https://www.inaturalist.org/observations/124864523
141	Lanius cristatus	Brown Shrike	2021/3/23	3	0	9.58566092	76.52925868	https://www.inaturalist.org/observations/129501724
142	Lanius cristatus	Brown Shrike	2019/12/14	4	0	9.59766245	76.51108997	https://www.inaturalist.org/observations/43051016
143	Lanius cristatus	Brown Shrike	2020/1/24	3	0	9.60466890	77.17205976	https://www.inaturalist.org/observations/59451149
144	Lanius cristatus	Brown Shrike	2020/3/13	3	0	9.66464288	76.33603070	https://www.inaturalist.org/observations/39923775
145	Lanius cristatus	Brown Shrike	2020/3/13	3	0	9.66466121	76.33604976	https://www.inaturalist.org/observations/39923495
146	Lanius cristatus	Brown Shrike	2022/11/22	3	0	9.70601240	76.47655193	https://www.inaturalist.org/observations/142599758
147	Lanius cristatus	Brown Shrike	2022/10/8	1	0	9.79519590	76.87057160	https://www.inaturalist.org/observations/138003369
148	Lanius cristatus	Brown Shrike	2022/4/17	2	0	9.83123041	76.78898888	https://www.inaturalist.org/observations/114303449
149	Lanius cristatus	Brown Shrike	2021/10/17	3	0	9.83178303	76.78950939	https://www.inaturalist.org/observations/98513240
150	Lanius cristatus	Brown Shrike	2021/11/28	2	0	9.83314184	76.77787217	https://www.inaturalist.org/observations/102415696
151	Lanius cristatus	Brown Shrike	2022/10/8	2	0	9.83935417	76.78313078	https://www.inaturalist.org/observations/137986965
152	Lanius cristatus	Brown Shrike	2021/11/4	2	0	9.83981250	76.78269480	https://www.inaturalist.org/observations/100221971
153	Lanius cristatus	Brown Shrike	2021/11/18	2	0	9.84002040	76.78343068	https://www.inaturalist.org/observations/101631590
154	Lanius cristatus	Brown Shrike	2021/10/31	4	0	9.84071111	76.78055129	https://www.inaturalist.org/observations/99882700
155	Lanius cristatus	Brown Shrike	2022/10/18	0	0	9.84891429	76.71430050	https://www.inaturalist.org/observations/139843464
156	Lanius cristatus	Brown Shrike	2019/1/2	2	0	9.87536058	76.72690920	https://www.inaturalist.org/observations/96385171
157	Lanius cristatus	Brown Shrike	2022/4/7	2	0	9.89081268	76.69041719	https://www.inaturalist.org/observations/114273756
158	Lanius cristatus	Brown Shrike	2022/11/27	1	0	9.89081268	76.69041719	https://www.inaturalist.org/observations/143184830
159	Lanius cristatus	Brown Shrike	2021/10/9	3	0	9.89245159	76.66443767	https://www.inaturalist.org/observations/97673536
160	Lanius cristatus	Brown Shrike	2021/10/5	5	0	9.89555351	76.74879118	https://www.inaturalist.org/observations/97231352

161	Lanius cristatus	Brown Shrike	2021/10/13	1	0	9.89555351	76.74879118	https://www.inaturalist.org/observations/98109952
162	Lanius cristatus	Brown Shrike	2019/10/8	3	0	9.93188362	78.14039302	https://www.inaturalist.org/observations/105339082
163	Lanius cristatus	Brown Shrike	2021/1/9	4	0	9.94835159	78.14931694	https://www.inaturalist.org/observations/67792918
164	Lanius cristatus	Brown Shrike	2014/3/25	3	0	9.95205500	76.31138429	https://www.inaturalist.org/observations/48544035
165	Lanius cristatus	Brown Shrike	2013/12/26	4	0	9.95205500	76.31138429	https://www.inaturalist.org/observations/48609783
166	Lanius cristatus	Brown Shrike	2021/12/20	3	0	10.05133978	76.33867749	https://www.inaturalist.org/observations/104655365
167	Lanius cristatus	Brown Shrike	2022/11/12	0	0	10.05168713	76.34048633	https://www.inaturalist.org/observations/141764264
168	Lanius cristatus	Brown Shrike	2022/11/12	1	0	10.05168713	76.34048633	https://www.inaturalist.org/observations/141764290
169	Lanius cristatus	Brown Shrike	2022/12/17	1	0	10.05168713	76.34048633	https://www.inaturalist.org/observations/144524367
170	Lanius cristatus	Brown Shrike	2022/12/17	1	0	10.05168713	76.34048633	https://www.inaturalist.org/observations/144524369
171	Lanius cristatus	Brown Shrike	2020/1/31	3	0	10.06363333	76.33896667	https://www.inaturalist.org/observations/67019358
172	Lanius cristatus	Brown Shrike	2021/2/7	2	0	10.06455535	76.60683319	https://www.inaturalist.org/observations/69265365
173	Lanius cristatus	Brown Shrike	2019/2/2	5	0	10.07226635	76.95704289	https://www.inaturalist.org/observations/49501574
174	Lanius cristatus	Brown Shrike	2021/11/11	2	0	10.07269066	77.05676342	https://www.inaturalist.org/observations/100935195
175	Lanius cristatus lucionensis	Brown Shrike	2020/1/28	1	0	10.09834893	76.71375134	https://www.inaturalist.org/observations/39236016
176	Lanius cristatus	Brown Shrike	2018/12/15	5	0	10.10398030	76.70046050	https://www.inaturalist.org/observations/32149420
177	Lanius cristatus	Brown Shrike	2018/12/15	3	0	10.10398030	76.70046050	https://www.inaturalist.org/observations/56312204
178	Lanius cristatus	Brown Shrike	2018/3/8	3	0	10.10398030	76.70046050	https://www.inaturalist.org/observations/91676801
179	Lanius cristatus	Brown Shrike	2019/11/29	4	0	10.10404183	76.70046039	https://www.inaturalist.org/observations/50386277
180	Lanius cristatus	Brown Shrike	2016/12/7	7	0	10.10634769	76.70470314	http://www.inaturalist.org/observations/4729889
181	Lanius cristatus	Brown Shrike	2022/4/18	2	0	10.12089500	76.75847500	https://www.inaturalist.org/observations/114784180
182	Lanius cristatus	Brown Shrike	2016/12/21	6	0	10.12811910	76.75529410	https://www.inaturalist.org/observations/66206541
183	Lanius cristatus	Brown Shrike	2017/3/29	3	0	10.12979140	76.68711840	https://www.inaturalist.org/observations/64265751

184	Lanius cristatus	Brown Shrike	2020/1/16	3	0	10.12994139	76.68765639	https://www.inaturalist.org/observations/51554490
185	Lanius cristatus	Brown Shrike	2020/2/9	4	0	10.13098780	76.24104930	https://www.inaturalist.org/observations/41221294
186	Lanius cristatus	Brown Shrike	2014/1/30	4	0	10.13316447	76.68874613	https://www.inaturalist.org/observations/68455414
187	Lanius cristatus	Brown Shrike	2022/10/24	3	0	10.13517211	76.38848886	https://www.inaturalist.org/observations/139951106
188	Lanius cristatus	Brown Shrike	2021/10/17	2	0	10.15322610	76.33767860	https://www.inaturalist.org/observations/98458182
189	Lanius cristatus	Brown Shrike	2022/11/9	1	0	10.15322610	76.33767860	https://www.inaturalist.org/observations/141515071
190	Lanius cristatus	Brown Shrike	2022/11/20	1	0	10.15322610	76.33767860	https://www.inaturalist.org/observations/142426701
191	Lanius cristatus	Brown Shrike	2021/11/7	4	0	10.15587909	76.37910426	https://www.inaturalist.org/observations/100844916
192	Lanius cristatus	Brown Shrike	2021/12/27	1	0	10.19424410	77.99670430	https://www.inaturalist.org/observations/104639605
193	Lanius cristatus	Brown Shrike	2020/12/14	0	0	10.24769080	76.33484780	https://www.inaturalist.org/observations/143784850
194	Lanius cristatus	Brown Shrike	2021/12/25	1	0	10.29753000	79.81047333	https://www.inaturalist.org/observations/103752216
195	Lanius cristatus	Brown Shrike	2019/10/6	1	0	10.29911240	79.84561000	https://www.inaturalist.org/observations/89439550
196	Lanius cristatus	Brown Shrike	2022/11/28	2	0	10.30214265	76.42738435	https://www.inaturalist.org/observations/143180082
197	Lanius cristatus	Brown Shrike	2018/4/3	4	0	10.30313333	76.99105556	https://www.inaturalist.org/observations/66524199
198	Lanius cristatus	Brown Shrike	2011/2/22	5	0	10.32359080	76.95100300	https://www.inaturalist.org/observations/30498099
199	Lanius cristatus	Brown Shrike	2019/10/12	2	0	10.32697340	76.95539670	https://www.inaturalist.org/observations/55132008
200	Lanius cristatus	Brown Shrike	2021/3/31	2	0	10.32697340	76.95539670	https://www.inaturalist.org/observations/105256419
201	Lanius cristatus	Brown Shrike	2021/4/1	2	0	10.32697340	76.95539670	https://www.inaturalist.org/observations/105256437
202	Lanius cristatus	Brown Shrike	2018/11/10	4	0	10.37499116	76.21854052	https://www.inaturalist.org/observations/18552047
203	Lanius cristatus	Brown Shrike	2020/1/7	4	0	10.39389333	76.23106500	https://www.inaturalist.org/observations/44164534
204	Lanius cristatus	Brown Shrike	2020/1/7	3	0	10.39861746	76.23055806	https://www.inaturalist.org/observations/44164679
205	Lanius cristatus	Brown Shrike	2020/2/1	4	0	10.43878000	76.17306000	https://www.inaturalist.org/observations/67039867
206	Lanius cristatus	Brown Shrike	2019/1/6	4	0	10.46427294	76.15506904	https://www.inaturalist.org/observations/100827075

207	Lanius cristatus	Brown Shrike	2019/10/18	3	0	10.46817167	76.16493333	https://www.inaturalist.org/observations/35016188
208	Lanius cristatus	Brown Shrike	2014/12/14	5	0	10.49318983	76.21332647	https://www.inaturalist.org/observations/35271984
209	Lanius cristatus	Brown Shrike	2020/1/6	3	0	10.53299675	76.12853717	https://www.inaturalist.org/observations/44162309
210	Lanius cristatus	Brown Shrike	2020/1/5	4	0	10.53324000	76.12897333	https://www.inaturalist.org/observations/44104620
211	Lanius cristatus	Brown Shrike	2022/1/2	2	0	10.53333000	76.12899833	https://www.inaturalist.org/observations/142732383
212	Lanius cristatus	Brown Shrike	2019/1/20	2	0	10.53367812	76.17583808	https://www.inaturalist.org/observations/139315302
213	Lanius cristatus	Brown Shrike	2020/1/6	4	0	10.53370167	76.12897000	https://www.inaturalist.org/observations/44162296
214	Lanius cristatus	Brown Shrike	2020/1/29	4	0	10.53382000	76.12891667	https://www.inaturalist.org/observations/67019449
215	Lanius cristatus	Brown Shrike	2020/1/29	4	0	10.53397500	76.12849167	https://www.inaturalist.org/observations/67019451
216	Lanius cristatus	Brown Shrike	2022/4/16	2	0	10.53478239	76.14606136	https://www.inaturalist.org/observations/114783978
217	Lanius cristatus	Brown Shrike	2018/10/30	3	0	10.53595206	76.12947280	https://www.inaturalist.org/observations/18264232
218	Lanius cristatus	Brown Shrike	2020/1/29	2	0	10.53759667	76.12866167	https://www.inaturalist.org/observations/67019455
219	Lanius cristatus	Brown Shrike	2020/1/5	4	0	10.54159500	76.15447000	https://www.inaturalist.org/observations/44104674
220	Lanius cristatus	Brown Shrike	2020/10/18	0	0	10.54587602	77.21157745	https://www.inaturalist.org/observations/63228863
221	Lanius cristatus	Brown Shrike	2020/1/8	4	0	10.55849333	76.13233833	https://www.inaturalist.org/observations/44169572
222	Lanius cristatus	Brown Shrike	2018/11/11	5	0	10.63750217	76.30570555	https://www.inaturalist.org/observations/18669415
223	Lanius cristatus	Brown Shrike	2016/12/17	5	0	10.74762869	76.46737076	https://www.inaturalist.org/observations/65301099
224	Lanius cristatus	Brown Shrike	2016/2/2	5	0	10.78673000	76.65479300	https://www.inaturalist.org/observations/7265797
225	Lanius cristatus	Brown Shrike	2016/1/24	4	0	10.78673000	76.65479300	https://www.inaturalist.org/observations/7279947
226	Lanius cristatus	Brown Shrike	2018/12/11	5	0	10.81531178	79.61054452	https://www.inaturalist.org/observations/29355023
227	Lanius cristatus	Brown Shrike	2021/4/4	3	0	10.88499352	76.06601469	https://www.inaturalist.org/observations/72870090
228	Lanius cristatus	Brown Shrike	2019/1/23	3	0	10.88777170	76.07321890	https://www.inaturalist.org/observations/67397361
229	Lanius cristatus	Brown Shrike	2021/12/27	2	0	11.01684450	76.95583210	https://www.inaturalist.org/observations/106483152

230	Lanius cristatus	Brown Shrike	2022/10/13	1	0	11.04169167	79.35541944	https://www.inaturalist.org/observations/138595566
231	Lanius cristatus	Brown Shrike	2021/10/17	2	0	11.07430916	79.85120773	https://www.inaturalist.org/observations/99189029
232	Lanius cristatus	Brown Shrike	2021/10/23	2	0	11.07760112	79.85221289	https://www.inaturalist.org/observations/99084753
233	Lanius cristatus	Brown Shrike	2021/1/10	2	0	11.07894586	79.83002432	https://www.inaturalist.org/observations/88412642
234	Lanius cristatus	Brown Shrike	2022/11/25	3	0	11.10722080	79.76986505	https://www.inaturalist.org/observations/142813028
235	Lanius cristatus	Brown Shrike	2020/1/11	3	0	11.10909344	79.69935045	https://www.inaturalist.org/observations/39694601
236	Lanius cristatus	Brown Shrike	2021/12/29	2	0	11.11571108	79.69141044	https://www.inaturalist.org/observations/103888818
237	Lanius cristatus	Brown Shrike	2020/10/31	1	0	11.16739935	77.11214610	https://www.inaturalist.org/observations/107244965
238	Lanius cristatus	Brown Shrike	2019/12/31	3	0	11.23409110	77.65862357	https://www.inaturalist.org/observations/37130643
239	Lanius cristatus	Brown Shrike	2019/1/17	3	0	11.23986174	79.29021332	https://www.inaturalist.org/observations/54719406
240	Lanius cristatus	Brown Shrike	2022/12/4	2	0	11.32303840	79.24335279	https://www.inaturalist.org/observations/143681736
241	Lanius cristatus	Brown Shrike	2021/1/10	3	0	11.32304662	79.24335279	https://www.inaturalist.org/observations/68215981
242	Lanius cristatus	Brown Shrike	2022/1/16	1	0	11.32304662	79.24335279	https://www.inaturalist.org/observations/105022611
243	Lanius cristatus	Brown Shrike	2017/1/20	2	0	11.35775292	76.80015383	https://www.inaturalist.org/observations/56913215
244	Lanius cristatus	Brown Shrike	2017/12/10	4	0	11.50746108	92.70247341	https://www.inaturalist.org/observations/9558269
245	Lanius cristatus	Brown Shrike	2019/1/29	2	0	11.54115090	76.02178310	https://www.inaturalist.org/observations/136678673
246	Lanius cristatus	Brown Shrike	2022/3/13	3	0	11.55938546	76.22768249	https://www.inaturalist.org/observations/125461787
247	Lanius cristatus lucionensis	Brown Shrike	2014/2/6	2	0	11.58776161	92.61504186	https://www.inaturalist.org/observations/68651720
248	Lanius cristatus	Brown Shrike	2017/12/14	4	0	11.59478800	92.69443000	https://www.inaturalist.org/observations/9558324
249	Lanius cristatus	Brown Shrike	2017/12/14	4	0	11.59478800	92.69443000	https://www.inaturalist.org/observations/9558325
250	Lanius cristatus lucionensis	Brown Shrike	2022/4/6	1	0	11.61543688	92.61952009	https://www.inaturalist.org/observations/111487566
251	Lanius cristatus lucionensis	Brown Shrike	2022/4/10	1	0	11.61546007	92.61903273	https://www.inaturalist.org/observations/111493590
252	Lanius cristatus	Brown Shrike	2017/2/11	1	0	11.62337740	92.72648280	https://www.inaturalist.org/observations/82143305

253	Lanius cristatus	Brown Shrike	2022/11/13	1	0	11.62337740	92.72648280	https://www.inaturalist.org/observations/142804594
254	Lanius cristatus	Brown Shrike	2022/2/23	2	0	11.66642990	92.67092900	https://www.inaturalist.org/observations/107880805
255	Lanius cristatus	Brown Shrike	2022/12/22	1	0	11.67495647	92.74170678	https://www.inaturalist.org/observations/144906877
256	Lanius cristatus	Brown Shrike	2021/1/28	2	0	11.68742128	78.18066828	https://www.inaturalist.org/observations/68717097
257	Lanius cristatus	Brown Shrike	2016/12/28	4	0	11.70338737	76.61443630	https://www.inaturalist.org/observations/68734609
258	Lanius cristatus	Brown Shrike	2020/4/26	4	0	11.71193359	76.16388246	https://www.inaturalist.org/observations/52940225
259	Lanius cristatus	Brown Shrike	2021/3/14	3	0	11.72005289	78.17852888	https://www.inaturalist.org/observations/71200089
260	Lanius cristatus	Brown Shrike	2013/2/10	3	0	11.72019670	92.73345460	https://www.inaturalist.org/observations/31318491
261	Lanius cristatus	Brown Shrike	2019/3/17	2	0	11.72019670	92.73345460	https://www.inaturalist.org/observations/51213103
262	Lanius cristatus	Brown Shrike	2017/2/4	1	0	11.72019670	92.73345460	https://www.inaturalist.org/observations/81990698
263	Lanius cristatus	Brown Shrike	2018/3/23	1	0	11.72019670	92.73345460	https://www.inaturalist.org/observations/101158876
264	Lanius cristatus	Brown Shrike	2021/12/18	2	0	11.72031814	92.73336411	https://www.inaturalist.org/observations/103519126
265	Lanius cristatus	Brown Shrike	2018/11/11	4	0	11.72301270	76.88286200	https://www.inaturalist.org/observations/18449309
266	Lanius cristatus lucionensis	Brown Shrike	2022/2/7	1	0	11.74008670	92.65864010	https://www.inaturalist.org/observations/141982369
267	Lanius cristatus	Brown Shrike	2022/2/21	3	0	11.75915032	75.55387102	https://www.inaturalist.org/observations/107195895
268	Lanius cristatus	Brown Shrike	2020/10/11	2	0	11.77978499	76.46468047	https://www.inaturalist.org/observations/62468627
269	Lanius cristatus	Brown Shrike	2021/1/12	2	0	11.79206521	76.02630056	https://www.inaturalist.org/observations/68312891
270	Lanius cristatus	Brown Shrike	2022/11/26	0	0	11.79515333	92.71206333	https://www.inaturalist.org/observations/144524174
271	Lanius cristatus	Brown Shrike	2022/2/21	2	0	11.79615407	92.71869267	https://www.inaturalist.org/observations/107877460
272	Lanius cristatus	Brown Shrike	2020/10/7	1	0	11.81766270	76.93148365	https://www.inaturalist.org/observations/86542891
273	Lanius cristatus	Brown Shrike	2017/2/9	0	0	11.83291860	93.05261200	https://www.inaturalist.org/observations/82025999
274	Lanius cristatus	Brown Shrike	2013/2/10	4	0	11.83969440	92.65382550	https://www.inaturalist.org/observations/31317760
275	Lanius cristatus	Brown Shrike	2017/11/13	2	0	11.86345120	78.12294640	https://www.inaturalist.org/observations/143067265

276	Lanius cristatus	Brown Shrike	2021/4/10	1	0	11.90235333	77.12364500	https://www.inaturalist.org/observations/87129092
277	Lanius cristatus	Brown Shrike	2021/1/24	3	0	11.93320530	79.42678450	https://www.inaturalist.org/observations/69401932
278	Lanius cristatus	Brown Shrike	2022/11/5	4	0	11.94352100	76.47872500	https://www.inaturalist.org/observations/143829659
279	Lanius cristatus	Brown Shrike	2018/11/11	2	0	11.94468432	75.38493812	https://www.inaturalist.org/observations/123696862
280	Lanius cristatus	Brown Shrike	2021/3/28	2	0	11.94468432	75.38493812	https://www.inaturalist.org/observations/125429652
281	Lanius cristatus	Brown Shrike	2022/9/30	1	0	11.94468432	75.38493812	https://www.inaturalist.org/observations/140358261
282	Lanius cristatus	Brown Shrike	2022/10/8	0	0	11.94468432	75.38493812	https://www.inaturalist.org/observations/140950550
283	Lanius cristatus	Brown Shrike	2022/10/16	1	0	11.94468432	75.38493812	https://www.inaturalist.org/observations/141602584
284	Lanius cristatus	Brown Shrike	2022/11/12	1	0	11.94468432	75.38493812	https://www.inaturalist.org/observations/143856729
285	Lanius cristatus	Brown Shrike	2022/11/14	1	0	11.94468432	75.38493812	https://www.inaturalist.org/observations/143987385
286	Lanius cristatus	Brown Shrike	2022/11/15	0	0	11.94468432	75.38493812	https://www.inaturalist.org/observations/143993207
287	Lanius cristatus	Brown Shrike	2022/11/21	2	0	11.94468432	75.38493812	https://www.inaturalist.org/observations/144354853
288	Lanius cristatus	Brown Shrike	2022/11/24	1	0	11.94468432	75.38493812	https://www.inaturalist.org/observations/144718306
289	Lanius cristatus	Brown Shrike	2022/12/4	0	0	11.94468432	75.38493812	https://www.inaturalist.org/observations/145246831
290	Lanius cristatus	Brown Shrike	2019/3/3	2	0	11.94468464	75.38493812	https://www.inaturalist.org/observations/83735583
291	Lanius cristatus	Brown Shrike	2018/3/21	3	0	11.97605030	92.98755650	https://www.inaturalist.org/observations/100761313
292	Lanius cristatus	Brown Shrike	2020/4/6	6	0	11.98427631	75.39945457	https://www.inaturalist.org/observations/44390439
293	Lanius cristatus	Brown Shrike	2020/10/15	2	0	11.99509720	79.82337110	https://www.inaturalist.org/observations/109834907
294	Lanius cristatus	Brown Shrike	2017/1/15	6	0	11.99563240	77.14276400	https://www.inaturalist.org/observations/19862824
295	Lanius cristatus	Brown Shrike	2019/3/1	3	0	11.99979140	75.95646020	https://www.inaturalist.org/observations/69987105
296	Lanius cristatus	Brown Shrike	2022/11/27	0	0	12.01230833	75.59171833	https://www.inaturalist.org/observations/143036222
297	Lanius cristatus	Brown Shrike	2022/12/4	0	0	12.01653305	92.98663907	https://www.inaturalist.org/observations/144472278
298	Lanius cristatus	Brown Shrike	2017/2/19	3	0	12.02593667	75.23667833	https://www.inaturalist.org/observations/30663934

299	Lanius cristatus	Brown Shrike	2017/3/15	4	0	12.03000078	75.20706394	https://www.inaturalist.org/observations/30603877
300	Lanius cristatus	Brown Shrike	2017/3/17	6	0	12.03000078	75.20706394	https://www.inaturalist.org/observations/30603896
301	Lanius cristatus	Brown Shrike	2017/2/19	5	0	12.03114000	75.22991833	https://www.inaturalist.org/observations/30663918
302	Lanius cristatus	Brown Shrike	2011/12/27	3	0	12.03126193	76.12014771	https://www.inaturalist.org/observations/66843333
303	Lanius cristatus	Brown Shrike	2022/10/3	3	0	12.03234633	75.25665645	https://www.inaturalist.org/observations/140749362
304	Lanius cristatus	Brown Shrike	2022/11/20	1	0	12.03234633	75.25665645	https://www.inaturalist.org/observations/144346104
305	Lanius cristatus	Brown Shrike	2019/10/15	4	0	12.03234670	75.25665640	https://www.inaturalist.org/observations/34574193
306	Lanius cristatus	Brown Shrike	2020/12/6	3	0	12.03234670	75.25665640	https://www.inaturalist.org/observations/66999029
307	Lanius cristatus	Brown Shrike	2017/2/7	1	0	12.03747360	92.98714120	https://www.inaturalist.org/observations/82010705
308	Lanius cristatus	Brown Shrike	2022/10/25	1	0	12.04009244	75.29748503	https://www.inaturalist.org/observations/142200219
309	Lanius cristatus	Brown Shrike	2022/10/25	1	0	12.04009244	75.29748503	https://www.inaturalist.org/observations/142200313
310	Lanius cristatus	Brown Shrike	2022/10/25	1	0	12.04009244	75.29748503	https://www.inaturalist.org/observations/142200322
311	Lanius cristatus	Brown Shrike	2021/10/2	1	0	12.10347138	75.23360022	https://www.inaturalist.org/observations/96904729
312	Lanius cristatus	Brown Shrike	2021/10/2	2	0	12.10347138	75.23360022	https://www.inaturalist.org/observations/96904746
313	Lanius cristatus	Brown Shrike	2020/2/17	2	0	12.10459333	75.23454833	https://www.inaturalist.org/observations/39321478
314	Lanius cristatus	Brown Shrike	2021/1/16	2	0	12.14557700	75.79543000	https://www.inaturalist.org/observations/68266722
315	Lanius cristatus	Brown Shrike	2021/10/9	1	0	12.15306351	76.54041270	https://www.inaturalist.org/observations/97635438
316	Lanius cristatus	Brown Shrike	2022/1/26	3	0	12.15771983	76.47221448	https://www.inaturalist.org/observations/105649524
317	Lanius cristatus	Brown Shrike	2022/11/13	2	0	12.16529924	75.42718928	https://www.inaturalist.org/observations/143931147
318	Lanius cristatus	Brown Shrike	2022/2/6	1	0	12.17181333	75.56614833	https://www.inaturalist.org/observations/142750897
319	Lanius cristatus	Brown Shrike	2016/3/9	0	0	12.19110400	76.65484000	https://www.inaturalist.org/observations/82672218
320	Lanius cristatus	Brown Shrike	2016/3/9	2	0	12.19113000	76.65513500	https://www.inaturalist.org/observations/139622991
321	Lanius cristatus	Brown Shrike	2015/9/27	0	0	12.20282576	75.81176954	https://www.inaturalist.org/observations/92880064

322	Lanius cristatus	Brown Shrike	2019/11/9	1	0	12.20282576	75.81176954	https://www.inaturalist.org/observations/93598699
323	Lanius cristatus	Brown Shrike	2022/2/26	2	0	12.21247353	76.63342537	https://www.inaturalist.org/observations/107462963
324	Lanius cristatus	Brown Shrike	2014/10/4	1	0	12.22272330	76.62728760	https://www.inaturalist.org/observations/143348914
325	Lanius cristatus	Brown Shrike	2014/12/16	1	0	12.22272330	76.62728760	https://www.inaturalist.org/observations/145244247
326	Lanius cristatus	Brown Shrike	2016/3/21	1	0	12.22966583	79.09139769	https://www.inaturalist.org/observations/96065123
327	Lanius cristatus	Brown Shrike	2022/10/11	2	0	12.23409360	76.68548729	https://www.inaturalist.org/observations/139112069
328	Lanius cristatus	Brown Shrike	2020/12/20	3	0	12.24937667	75.27790833	https://www.inaturalist.org/observations/66994058
329	Lanius cristatus	Brown Shrike	2020/12/20	3	0	12.25179333	75.27723500	https://www.inaturalist.org/observations/66994049
330	Lanius cristatus	Brown Shrike	2020/12/20	3	0	12.25224833	75.27641333	https://www.inaturalist.org/observations/66994046
331	Lanius cristatus	Brown Shrike	2016/9/22	2	0	12.25554399	76.61182531	https://www.inaturalist.org/observations/94567368
332	Lanius cristatus	Brown Shrike	2018/10/25	1	0	12.26005298	76.77324214	https://www.inaturalist.org/observations/90361052
333	Lanius cristatus	Brown Shrike	2020/10/4	1	0	12.26269942	76.56762058	https://www.inaturalist.org/observations/95042441
334	Lanius cristatus	Brown Shrike	2020/10/28	1	0	12.26937200	76.61197800	https://www.inaturalist.org/observations/94224573
335	Lanius cristatus	Brown Shrike	2012/1/14	2	0	12.26937200	76.61197800	https://www.inaturalist.org/observations/94711070
336	Lanius cristatus	Brown Shrike	2014/10/4	1	0	12.26937200	76.61197800	https://www.inaturalist.org/observations/94790694
337	Lanius cristatus	Brown Shrike	2009/4/7	1	0	12.27042491	76.77563072	https://www.inaturalist.org/observations/90362855
338	Lanius cristatus	Brown Shrike	2011/3/5	0	0	12.27042491	76.77563072	https://www.inaturalist.org/observations/92668440
339	Lanius cristatus	Brown Shrike	2015/10/25	1	0	12.27525460	76.67014750	https://www.inaturalist.org/observations/86684973
340	Lanius cristatus	Brown Shrike	2013/11/15	2	0	12.27525460	76.67014750	https://www.inaturalist.org/observations/87713327
341	Lanius cristatus	Brown Shrike	2020/10/22	2	0	12.27525460	76.67014750	https://www.inaturalist.org/observations/88506934
342	Lanius cristatus	Brown Shrike	2015/9/27	0	0	12.28234021	76.32413459	https://www.inaturalist.org/observations/92777199
343	Lanius cristatus	Brown Shrike	2022/4/3	3	0	12.28297667	76.70857500	https://www.inaturalist.org/observations/110454663
344	Lanius cristatus	Brown Shrike	2021/10/27	1	0	12.29424209	76.56539693	https://www.inaturalist.org/observations/99523414

345	Lanius cristatus	Brown Shrike	2021/12/5	1	0	12.30921890	76.55560459	https://www.inaturalist.org/observations/102516666
346	Lanius cristatus	Brown Shrike	2022/12/1	2	0	12.31629282	76.55037403	https://www.inaturalist.org/observations/143549934
347	Lanius cristatus	Brown Shrike	2022/11/27	2	0	12.33606885	77.81784051	https://www.inaturalist.org/observations/143637983
348	Lanius cristatus	Brown Shrike	2017/9/2	2	0	12.35159000	77.64564667	https://www.inaturalist.org/observations/67073853
349	Lanius cristatus	Brown Shrike	2022/11/27	2	0	12.36190500	77.86991167	https://www.inaturalist.org/observations/143014633
350	Lanius cristatus	Brown Shrike	2020/1/6	3	0	12.37254312	77.05188964	https://www.inaturalist.org/observations/37817929
351	Lanius cristatus	Brown Shrike	2022/11/27	0	0	12.39316667	77.86548167	https://www.inaturalist.org/observations/143013503
352	Lanius cristatus	Brown Shrike	2018/3/4	3	0	12.39834324	76.68988894	https://www.inaturalist.org/observations/64928592
353	Lanius cristatus	Brown Shrike	2021/3/6	4	0	12.41225289	77.85643991	https://www.inaturalist.org/observations/126171352
354	Lanius cristatus	Brown Shrike	2020/11/30	5	0	12.41666670	77.75000000	https://www.inaturalist.org/observations/66007336
355	Lanius cristatus	Brown Shrike	2022/4/18	2	0	12.42362760	76.65678918	https://www.inaturalist.org/observations/112122627
356	Lanius cristatus	Brown Shrike	2020/11/10	0	0	12.42534519	76.65613462	https://www.inaturalist.org/observations/96065696
357	Lanius cristatus	Brown Shrike	2022/2/7	2	0	12.43443611	75.77248985	https://www.inaturalist.org/observations/115755902
358	Lanius cristatus	Brown Shrike	2017/1/8	1	0	12.43496081	76.24001749	https://www.inaturalist.org/observations/95551684
359	Lanius cristatus	Brown Shrike	2018/1/1	4	0	12.49405097	76.97686707	https://www.inaturalist.org/observations/9355512
360	Lanius cristatus	Brown Shrike	2022/2/7	2	0	12.55537854	75.62993635	https://www.inaturalist.org/observations/115755905
361	Lanius cristatus	Brown Shrike	2021/11/7	0	0	12.56661654	75.78098942	https://www.inaturalist.org/observations/141344918
362	Lanius cristatus	Brown Shrike	2021/10/29	2	0	12.59597395	75.69609692	https://www.inaturalist.org/observations/102531025
363	Lanius cristatus	Brown Shrike	2014/12/21	2	0	12.63641730	76.99314520	https://www.inaturalist.org/observations/97980225
364	Lanius cristatus	Brown Shrike	2022/11/13	1	0	12.67455667	75.01822500	https://www.inaturalist.org/observations/144538336
365	Lanius cristatus	Brown Shrike	2017/12/3	2	0	12.67588107	77.76815173	https://www.inaturalist.org/observations/139617195
366	Lanius cristatus	Brown Shrike	2016/2/2	1	0	12.67591249	77.77037641	https://www.inaturalist.org/observations/83273835
367	Lanius cristatus	Brown Shrike	2017/12/3	1	0	12.67598563	77.76972776	https://www.inaturalist.org/observations/82665805

368	Lanius cristatus	Brown Shrike	2022/12/24	1	0	12.67689333	77.91016667	https://www.inaturalist.org/observations/144995660
369	Lanius cristatus	Brown Shrike	2021/10/3	2	0	12.67749487	77.91260335	https://www.inaturalist.org/observations/96993928
370	Lanius cristatus	Brown Shrike	2017/7/9	1	0	12.70834886	77.53787189	https://www.inaturalist.org/observations/66426131
371	Lanius cristatus	Brown Shrike	2022/2/19	2	0	12.71269193	77.80709054	https://www.inaturalist.org/observations/107472805
372	Lanius cristatus	Brown Shrike	2021/2/23	2	0	12.71317244	77.68367574	https://www.inaturalist.org/observations/70140313
373	Lanius cristatus	Brown Shrike	2022/2/19	2	0	12.71383000	77.80799167	https://www.inaturalist.org/observations/107120044
374	Lanius cristatus	Brown Shrike	2018/1/21	3	0	12.74535601	77.44834727	https://www.inaturalist.org/observations/65003474
375	Lanius cristatus	Brown Shrike	2019/2/15	4	0	12.74967697	80.19756265	https://www.inaturalist.org/observations/22558639
376	Lanius cristatus	Brown Shrike	2022/10/8	2	0	12.75028167	77.59918000	https://www.inaturalist.org/observations/137942687
377	Lanius cristatus	Brown Shrike	2021/10/17	2	0	12.76346401	78.43047477	https://www.inaturalist.org/observations/98479982
378	Lanius cristatus	Brown Shrike	2013/12/15	1	0	12.76865694	77.65885333	https://www.inaturalist.org/observations/102935086
379	Lanius cristatus	Brown Shrike	2020/10/17	3	0	12.76885310	77.57913580	https://www.inaturalist.org/observations/62774519
380	Lanius cristatus	Brown Shrike	2022/10/29	2	0	12.77747860	80.18868540	https://www.inaturalist.org/observations/140362363
381	Lanius cristatus	Brown Shrike	2022/10/29	0	0	12.78970747	80.22158425	https://www.inaturalist.org/observations/140364920
382	Lanius cristatus	Brown Shrike	2022/4/17	2	0	12.82063293	77.73091888	https://www.inaturalist.org/observations/112348137
383	Lanius cristatus	Brown Shrike	2022/1/14	3	0	12.82251700	77.72947100	https://www.inaturalist.org/observations/104914884
384	Lanius cristatus	Brown Shrike	2018/10/27	4	0	12.82582814	77.73390044	https://www.inaturalist.org/observations/17928198
385	Lanius cristatus	Brown Shrike	2021/1/14	1	0	12.82702320	80.24032100	https://www.inaturalist.org/observations/89642251
386	Lanius cristatus	Brown Shrike	2020/11/24	4	0	12.83179501	79.18678171	https://www.inaturalist.org/observations/73001489
387	Lanius cristatus	Brown Shrike	2021/10/26	1	0	12.83521880	80.20108040	https://www.inaturalist.org/observations/99766560
388	Lanius cristatus	Brown Shrike	2021/1/1	2	0	12.84064788	75.38225658	https://www.inaturalist.org/observations/67442460
389	Lanius cristatus	Brown Shrike	2021/12/22	1	0	12.84064788	75.38225658	https://www.inaturalist.org/observations/103685961
390	Lanius cristatus	Brown Shrike	2021/1/2	3	0	12.84197704	80.17383234	https://www.inaturalist.org/observations/67620522

391	Lanius cristatus	Brown Shrike	2021/1/4	2	0	12.84203140	80.17380851	https://www.inaturalist.org/observations/67572433
392	Lanius cristatus	Brown Shrike	2021/12/17	4	0	12.84575615	77.77541950	https://www.inaturalist.org/observations/105364656
393	Lanius cristatus	Brown Shrike	2020/11/5	1	0	12.85018083	77.49715928	https://www.inaturalist.org/observations/64289044
394	Lanius cristatus	Brown Shrike	2019/1/18	5	0	12.85502749	80.22369534	https://www.inaturalist.org/observations/19839674
395	Lanius cristatus	Brown Shrike	2020/10/4	3	0	12.85552100	79.11450818	https://www.inaturalist.org/observations/61669445
396	Lanius cristatus	Brown Shrike	2021/3/7	2	0	12.85718650	77.50891100	https://www.inaturalist.org/observations/70743653
397	Lanius cristatus	Brown Shrike	2021/10/31	1	0	12.85718650	77.50891100	https://www.inaturalist.org/observations/99861594
398	Lanius cristatus	Brown Shrike	2022/1/6	2	0	12.85755790	77.78640570	https://www.inaturalist.org/observations/104757420
399	Lanius cristatus	Brown Shrike	2021/2/7	2	0	12.86391821	79.14074186	https://www.inaturalist.org/observations/69259285
400	Lanius cristatus	Brown Shrike	2018/10/19	2	0	12.87414820	75.76445320	https://www.inaturalist.org/observations/65005757
401	Lanius cristatus	Brown Shrike	2020/10/26	2	0	12.87894620	77.52977930	https://www.inaturalist.org/observations/63565926
402	Lanius cristatus	Brown Shrike	2021/1/23	3	0	12.87976072	77.52975861	https://www.inaturalist.org/observations/68483083
403	Lanius cristatus	Brown Shrike	2021/11/20	1	0	12.88400499	77.46290982	https://www.inaturalist.org/observations/101698226
404	Lanius cristatus	Brown Shrike	2020/10/24	3	0	12.89047740	80.22585740	https://www.inaturalist.org/observations/63370595
405	Lanius cristatus	Brown Shrike	2019/10/14	2	0	12.89231570	80.18889170	https://www.inaturalist.org/observations/109824204
406	Lanius cristatus	Brown Shrike	2018/12/10	2	0	12.90098770	80.22793010	https://www.inaturalist.org/observations/109822136
407	Lanius cristatus	Brown Shrike	2018/12/10	2	0	12.90098770	80.22793010	https://www.inaturalist.org/observations/109822141
408	Lanius cristatus	Brown Shrike	2021/2/21	3	0	12.90402881	80.21086426	https://www.inaturalist.org/observations/109835100
409	Lanius cristatus	Brown Shrike	2020/4/12	3	0	12.90898530	77.49020970	https://www.inaturalist.org/observations/44570198
410	Lanius cristatus	Brown Shrike	2014/11/15	2	0	12.91499040	80.07201710	https://www.inaturalist.org/observations/63147471
411	Lanius cristatus	Brown Shrike	2020/10/19	1	0	12.91509453	77.46969799	https://www.inaturalist.org/observations/85940196
412	Lanius cristatus	Brown Shrike	2018/11/18	0	0	12.91831260	77.68009700	https://www.inaturalist.org/observations/65007286
413	Lanius cristatus	Brown Shrike	2022/10/29	2	0	12.92070700	77.64144180	https://www.inaturalist.org/observations/140480609

414	Lanius cristatus	Brown Shrike	2022/10/29	2	0	12.92070700	77.64144180	https://www.inaturalist.org/observations/140480613
415	Lanius cristatus	Brown Shrike	2018/12/28	5	0	12.92898480	80.17216130	https://www.inaturalist.org/observations/24588728
416	Lanius cristatus	Brown Shrike	2020/11/1	3	0	12.93562489	80.16648799	https://www.inaturalist.org/observations/65695765
417	Lanius cristatus	Brown Shrike	2019/11/17	6	0	12.93625261	80.21877129	https://www.inaturalist.org/observations/35776617
418	Lanius cristatus	Brown Shrike	2020/10/24	2	0	12.94693690	80.22006387	https://www.inaturalist.org/observations/64022206
419	Lanius cristatus	Brown Shrike	2019/2/7	5	0	12.94989475	80.22223569	https://www.inaturalist.org/observations/29175475
420	Lanius cristatus	Brown Shrike	2013/10/13	4	0	12.95812020	80.11922810	https://www.inaturalist.org/observations/22941879
421	Lanius cristatus	Brown Shrike	2013/10/13	2	0	12.95812020	80.11922810	https://www.inaturalist.org/observations/63147470
422	Lanius cristatus	Brown Shrike	2022/10/17	2	0	12.95826600	77.72553570	https://www.inaturalist.org/observations/139120367
423	Lanius cristatus	Brown Shrike	2022/10/28	1	0	12.95826600	77.72553570	https://www.inaturalist.org/observations/140250771
424	Lanius cristatus	Brown Shrike	2022/10/30	3	0	12.95826600	77.72553570	https://www.inaturalist.org/observations/140509851
425	Lanius cristatus	Brown Shrike	2022/11/1	2	0	12.95826600	77.72553570	https://www.inaturalist.org/observations/140750613
426	Lanius cristatus	Brown Shrike	2022/11/6	2	0	12.95827212	77.72553563	https://www.inaturalist.org/observations/141266145
427	Lanius cristatus	Brown Shrike	2022/11/8	1	0	12.95827212	77.72553563	https://www.inaturalist.org/observations/141507066
428	Lanius cristatus	Brown Shrike	2022/11/8	1	0	12.95827212	77.72553563	https://www.inaturalist.org/observations/141507775
429	Lanius cristatus	Brown Shrike	2021/2/21	1	0	12.97100393	80.15854289	https://www.inaturalist.org/observations/89428405
430	Lanius cristatus	Brown Shrike	2016/4/18	3	0	12.97137625	80.22727560	https://www.inaturalist.org/observations/63744579
431	Lanius cristatus	Brown Shrike	2019/9/27	3	0	12.97147851	77.59456269	https://www.inaturalist.org/observations/123058843
432	Lanius cristatus	Brown Shrike	2022/3/24	4	0	12.97159870	77.59456270	https://www.inaturalist.org/observations/120963717
433	Lanius cristatus	Brown Shrike	2022/3/24	4	0	12.97159870	77.59456270	https://www.inaturalist.org/observations/123299016
434	Lanius cristatus	Brown Shrike	2020/2/15	2	0	12.97895401	77.68667026	https://www.inaturalist.org/observations/38676990
435	Lanius cristatus	Brown Shrike	2018/10/7	5	0	12.98903043	77.66248455	https://www.inaturalist.org/observations/18330009
436	Lanius cristatus	Brown Shrike	2021/12/23	3	0	12.99243120	80.23289680	https://www.inaturalist.org/observations/105193887

437	Lanius cristatus	Brown Shrike	2021/12/23	1	0	12.99309831	80.23631651	https://www.inaturalist.org/observations/103619268
438	Lanius cristatus	Brown Shrike	2020/2/25	5	0	12.99333120	80.23610841	https://www.inaturalist.org/observations/39235959
439	Lanius cristatus	Brown Shrike	2022/1/26	2	0	12.99547418	80.23978467	https://www.inaturalist.org/observations/105620071
440	Lanius cristatus	Brown Shrike	2022/2/15	2	0	13.00029229	80.26669834	https://www.inaturalist.org/observations/106787398
441	Lanius cristatus	Brown Shrike	2022/2/15	2	0	13.00029229	80.26669834	https://www.inaturalist.org/observations/106842102
442	Lanius cristatus	Brown Shrike	2022/3/27	2	0	13.00492510	80.23787250	https://www.inaturalist.org/observations/109762065
443	Lanius cristatus	Brown Shrike	2021/1/22	1	0	13.01136871	77.05193785	https://www.inaturalist.org/observations/81701105
444	Lanius cristatus	Brown Shrike	2021/3/13	2	0	13.01856108	77.56938737	https://www.inaturalist.org/observations/105159026
445	Lanius cristatus	Brown Shrike	2020/1/19	5	0	13.02185970	77.56714230	https://www.inaturalist.org/observations/37946403
446	Lanius cristatus	Brown Shrike	2022/10/8	2	0	13.02209419	77.57052463	https://www.inaturalist.org/observations/138792499
447	Lanius cristatus	Brown Shrike	2020/9/23	3	0	13.02262227	77.56902755	https://www.inaturalist.org/observations/60499322
448	Lanius cristatus	Brown Shrike	2022/2/4	4	0	13.02398150	80.24441080	https://www.inaturalist.org/observations/107742109
449	Lanius cristatus	Brown Shrike	2021/10/14	2	0	13.03378717	77.57310610	https://www.inaturalist.org/observations/105659135
450	Lanius cristatus	Brown Shrike	2019/10/3	2	0	13.04734870	80.25400040	https://www.inaturalist.org/observations/52560343
451	Lanius cristatus	Brown Shrike	2022/1/7	2	0	13.05498860	77.75384199	https://www.inaturalist.org/observations/105414314
452	Lanius cristatus	Brown Shrike	2018/10/27	4	0	13.07029150	77.76952550	https://www.inaturalist.org/observations/18056412
453	Lanius cristatus	Brown Shrike	2016/2/25	2	0	13.08268020	80.27071840	https://www.inaturalist.org/observations/106017438
454	Lanius cristatus	Brown Shrike	2020/2/22	2	0	13.08268020	80.27071840	https://www.inaturalist.org/observations/137422270
455	Lanius cristatus	Brown Shrike	2017/12/24	2	0	13.14916756	80.05697362	https://www.inaturalist.org/observations/45849795
456	Lanius cristatus	Brown Shrike	2018/11/3	1	0	13.15000000	77.49000000	https://www.inaturalist.org/observations/18052017
457	Lanius cristatus	Brown Shrike	2020/10/18	3	0	13.16666700	80.17152800	https://www.inaturalist.org/observations/62869750
458	Lanius cristatus	Brown Shrike	2021/11/23	1	0	13.19559767	75.41076850	https://www.inaturalist.org/observations/102602088
459	Lanius cristatus	Brown Shrike	2021/3/14	1	0	13.34708333	74.79470000	https://www.inaturalist.org/observations/74335986

460	Lanius cristatus	Brown Shrike	2022/11/10	1	0	13.36738239	74.81768807	https://www.inaturalist.org/observations/141886337
461	Lanius cristatus	Brown Shrike	2018/1/26	1	0	13.40196890	78.05513840	https://www.inaturalist.org/observations/103460414
462	Lanius cristatus	Brown Shrike	2019/10/19	1	0	13.50592299	75.08894210	https://www.inaturalist.org/observations/81826336
463	Lanius cristatus	Brown Shrike	2019/10/18	1	0	13.50725830	75.09014373	https://www.inaturalist.org/observations/81826338
464	Lanius cristatus	Brown Shrike	2022/2/10	3	0	13.60274913	79.38015391	https://www.inaturalist.org/observations/112236732
465	Lanius cristatus	Brown Shrike	2020/12/30	1	0	13.62480300	74.69174600	https://www.inaturalist.org/observations/102882051
466	Lanius cristatus	Brown Shrike	2019/1/15	4	0	13.64892008	79.46691055	https://www.inaturalist.org/observations/35667301
467	Lanius cristatus	Brown Shrike	2019/1/15	3	0	13.65911635	79.48097763	https://www.inaturalist.org/observations/55805468
468	Lanius cristatus	Brown Shrike	2022/10/20	1	0	13.74931164	79.59864515	https://www.inaturalist.org/observations/139452132
469	Lanius cristatus	Brown Shrike	2021/3/11	1	0	13.75959646	79.47082987	https://www.inaturalist.org/observations/71541628
470	Lanius cristatus	Brown Shrike	2022/11/26	4	0	14.57646140	75.90992930	https://www.inaturalist.org/observations/143499946
471	Lanius cristatus	Brown Shrike	2020/11/8	3	0	14.66023904	74.90529943	https://www.inaturalist.org/observations/64513047
472	Lanius cristatus	Brown Shrike	2012/10/21	0	0	14.79638640	74.49969580	https://www.inaturalist.org/observations/96562080
473	Lanius cristatus	Brown Shrike	2022/10/31	3	0	14.81259020	76.80688695	https://www.inaturalist.org/observations/141458539
474	Lanius cristatus	Brown Shrike	2021/12/30	1	0	14.86613746	74.43937294	https://www.inaturalist.org/observations/103946069
475	Lanius cristatus	Brown Shrike	2021/3/12	1	0	15.23611910	74.61728190	https://www.inaturalist.org/observations/101927807
476	Lanius cristatus	Brown Shrike	2020/2/14	1	0	15.29123000	74.52274069	https://www.inaturalist.org/observations/104348024
477	Lanius cristatus	Brown Shrike	2018/10/27	2	0	15.29932650	74.12399600	https://www.inaturalist.org/observations/56304116
478	Lanius cristatus	Brown Shrike	2019/1/30	1	0	15.33501320	76.46002400	https://www.inaturalist.org/observations/84849991
479	Lanius cristatus	Brown Shrike	2020/11/19	2	0	15.38125989	75.10026568	https://www.inaturalist.org/observations/65757368
480	Lanius cristatus	Brown Shrike	2020/11/20	3	0	15.38490147	75.10266490	https://www.inaturalist.org/observations/66245553
481	Lanius cristatus	Brown Shrike	2020/11/20	4	0	15.38490147	75.10266490	https://www.inaturalist.org/observations/66245558
482	Lanius cristatus	Brown Shrike	2020/12/6	1	0	15.41523300	75.00598370	https://www.inaturalist.org/observations/90237904

483	Lanius cristatus	Brown Shrike	2022/10/19	2	0	15.43591242	74.98346306	https://www.inaturalist.org/observations/139331200
484	Lanius cristatus	Brown Shrike	2021/12/19	1	0	15.86827397	74.49491445	https://www.inaturalist.org/observations/103416152
485	Lanius cristatus	Brown Shrike	2022/2/9	3	0	15.86843167	80.50994500	https://www.inaturalist.org/observations/106438221
486	Lanius cristatus	Brown Shrike	2020/10/25	1	0	16.19958797	77.37883100	https://www.inaturalist.org/observations/81854450
487	Lanius cristatus	Brown Shrike	2014/10/15	2	0	16.30627144	80.51330510	https://www.inaturalist.org/observations/88111330
488	Lanius cristatus	Brown Shrike	2015/11/7	2	0	16.30689104	80.51252635	https://www.inaturalist.org/observations/87981040
489	Lanius cristatus	Brown Shrike	2014/2/26	2	0	16.30689104	80.51252635	https://www.inaturalist.org/observations/89171157
490	Lanius cristatus	Brown Shrike	2014/11/1	1	0	16.30689104	80.51252635	https://www.inaturalist.org/observations/94113063
491	Lanius cristatus	Brown Shrike	2020/11/27	2	0	16.36004000	74.29583900	https://www.inaturalist.org/observations/65687468
492	Lanius cristatus	Brown Shrike	2021/11/28	2	0	16.36906182	78.72910995	https://www.inaturalist.org/observations/102148493
493	Lanius cristatus	Brown Shrike	2013/11/18	2	0	16.40966969	80.70889656	https://www.inaturalist.org/observations/88106308
494	Lanius cristatus	Brown Shrike	2013/9/30	2	0	16.41060994	80.70892527	https://www.inaturalist.org/observations/88105643
495	Lanius cristatus	Brown Shrike	2021/11/7	3	0	16.44354010	80.57479460	https://www.inaturalist.org/observations/100846716
496	Lanius cristatus	Brown Shrike	2021/12/24	0	0	16.49170000	80.59191167	https://www.inaturalist.org/observations/103565949
497	Lanius cristatus	Brown Shrike	2012/3/29	1	0	16.49979669	80.59578562	https://www.inaturalist.org/observations/93593874
498	Lanius cristatus	Brown Shrike	2021/4/23	1	0	16.52286891	81.46555457	https://www.inaturalist.org/observations/95520605
499	Lanius cristatus	Brown Shrike	2022/1/2	2	0	16.52400500	81.51144833	https://www.inaturalist.org/observations/111965690
500	Lanius cristatus	Brown Shrike	2022/1/2	2	0	16.52400500	81.51144833	https://www.inaturalist.org/observations/113232252
501	Lanius cristatus	Brown Shrike	2022/1/2	1	0	16.52400667	81.51145500	https://www.inaturalist.org/observations/137427170
502	Lanius cristatus	Brown Shrike	2020/12/10	3	0	16.52985159	80.59233785	https://www.inaturalist.org/observations/66369911
503	Lanius cristatus	Brown Shrike	2022/2/1	1	0	16.53600167	81.50061500	https://www.inaturalist.org/observations/105946795
504	Lanius cristatus	Brown Shrike	2022/2/1	2	0	16.53600167	81.50061500	https://www.inaturalist.org/observations/114298155
505	Lanius cristatus	Brown Shrike	2022/2/25	2	0	16.53624833	81.49770667	https://www.inaturalist.org/observations/107406046

506	Lanius cristatus	Brown Shrike	2022/10/25	0	0	16.53886000	81.50652833	https://www.inaturalist.org/observations/139980850
507	Lanius cristatus	Brown Shrike	2022/10/25	2	0	16.53900000	81.50651500	https://www.inaturalist.org/observations/139980803
508	Lanius cristatus	Brown Shrike	2022/1/25	1	0	16.53938833	81.50571833	https://www.inaturalist.org/observations/113231258
509	Lanius cristatus	Brown Shrike	2022/1/25	1	0	16.53958667	81.50563833	https://www.inaturalist.org/observations/105548829
510	Lanius cristatus	Brown Shrike	2022/1/25	1	0	16.53960865	81.50545511	https://www.inaturalist.org/observations/105664603
511	Lanius cristatus	Brown Shrike	2022/1/25	1	0	16.53972918	81.50553592	https://www.inaturalist.org/observations/105569836
512	Lanius cristatus	Brown Shrike	2022/1/2	1	0	16.53974750	81.50541723	https://www.inaturalist.org/observations/104187908
513	Lanius cristatus	Brown Shrike	2022/1/2	1	0	16.53975682	81.50542159	https://www.inaturalist.org/observations/104166632
514	Lanius cristatus	Brown Shrike	2022/1/25	2	0	16.53976518	81.50555670	https://www.inaturalist.org/observations/111964625
515	Lanius cristatus	Brown Shrike	2022/1/25	2	0	16.53996734	81.50542762	https://www.inaturalist.org/observations/111964433
516	Lanius cristatus	Brown Shrike	2021/12/25	1	0	16.54489085	81.52124092	https://www.inaturalist.org/observations/103636399
517	Lanius cristatus	Brown Shrike	2021/2/4	5	0	16.54518332	81.70476638	https://www.inaturalist.org/observations/94458236
518	Lanius cristatus	Brown Shrike	2021/11/20	1	0	16.54553500	81.50563497	https://www.inaturalist.org/observations/102087159
519	Lanius cristatus	Brown Shrike	2021/12/25	1	0	16.55298167	81.50232167	https://www.inaturalist.org/observations/112076210
520	Lanius cristatus	Brown Shrike	2021/12/25	1	0	16.55302833	81.50233167	https://www.inaturalist.org/observations/104648425
521	Lanius cristatus	Brown Shrike	2021/12/25	2	0	16.55340333	81.50225500	https://www.inaturalist.org/observations/112981002
522	Lanius cristatus	Brown Shrike	2021/12/25	2	0	16.55340833	81.50225500	https://www.inaturalist.org/observations/104750303
523	Lanius cristatus lucionensis	Brown Shrike	2021/4/19	1	0	16.55345036	81.54328331	https://www.inaturalist.org/observations/93127339
524	Lanius cristatus	Brown Shrike	2020/10/7	2	0	16.55360559	80.58353109	https://www.inaturalist.org/observations/61961132
525	Lanius cristatus	Brown Shrike	2020/11/22	1	0	16.55493600	80.72975710	https://www.inaturalist.org/observations/92663999
526	Lanius cristatus	Brown Shrike	2020/11/22	1	0	16.55493600	80.72975710	https://www.inaturalist.org/observations/92664014
527	Lanius cristatus	Brown Shrike	2022/1/18	2	0	16.55633280	81.49540018	https://www.inaturalist.org/observations/105258156
528	Lanius cristatus	Brown Shrike	2021/12/31	2	0	16.55692000	81.49239333	https://www.inaturalist.org/observations/104112523

529	Lanius cristatus	Brown Shrike	2021/12/31	1	0	16.55692167	81.49237500	https://www.inaturalist.org/observations/104114528
530	Lanius cristatus	Brown Shrike	2021/12/31	1	0	16.55692167	81.49237500	https://www.inaturalist.org/observations/104411232
531	Lanius cristatus	Brown Shrike	2021/12/31	3	0	16.55692167	81.49237500	https://www.inaturalist.org/observations/111418534
532	Lanius cristatus	Brown Shrike	2022/1/2	1	0	16.55696667	81.49576167	https://www.inaturalist.org/observations/104411864
533	Lanius cristatus	Brown Shrike	2022/1/2	2	0	16.55696667	81.49576167	https://www.inaturalist.org/observations/111317285
534	Lanius cristatus	Brown Shrike	2021/12/19	2	0	16.55697105	81.49618272	https://www.inaturalist.org/observations/103344107
535	Lanius cristatus	Brown Shrike	2021/10/10	2	0	16.55811900	81.49816353	https://www.inaturalist.org/observations/97871276
536	Lanius cristatus	Brown Shrike	2021/10/10	3	0	16.55811900	81.49816353	https://www.inaturalist.org/observations/97895783
537	Lanius cristatus	Brown Shrike	2021/10/28	1	0	16.55811900	81.49816353	https://www.inaturalist.org/observations/101397457
538	Lanius cristatus	Brown Shrike	2021/11/4	2	0	16.55811900	81.49816353	https://www.inaturalist.org/observations/101457506
539	Lanius cristatus	Brown Shrike	2022/1/8	1	0	16.55811900	81.49816353	https://www.inaturalist.org/observations/104566630
540	Lanius cristatus	Brown Shrike	2020/12/6	1	0	16.55921794	80.76100097	https://www.inaturalist.org/observations/92452618
541	Lanius cristatus	Brown Shrike	2020/9/15	2	0	16.56202333	80.69468833	https://www.inaturalist.org/observations/59677547
542	Lanius cristatus	Brown Shrike	2020/10/6	3	0	16.56472500	80.71651333	https://www.inaturalist.org/observations/61845159
543	Lanius cristatus	Brown Shrike	2022/2/21	2	0	16.56483167	81.51749167	https://www.inaturalist.org/observations/107167077
544	Lanius cristatus	Brown Shrike	2022/2/21	2	0	16.56486000	81.51743000	https://www.inaturalist.org/observations/115843673
545	Lanius cristatus	Brown Shrike	2021/12/31	2	0	16.56821883	81.49002437	https://www.inaturalist.org/observations/104020982
546	Lanius cristatus	Brown Shrike	2022/1/20	1	0	16.56874833	81.56501333	https://www.inaturalist.org/observations/113231436
547	Lanius cristatus	Brown Shrike	2022/1/20	2	0	16.56876167	81.56510667	https://www.inaturalist.org/observations/105250631
548	Lanius cristatus	Brown Shrike	2020/11/29	1	0	16.57589266	80.68862217	https://www.inaturalist.org/observations/92670071
549	Lanius cristatus	Brown Shrike	2021/12/27	1	0	16.58994444	80.78369444	https://www.inaturalist.org/observations/103753926
550	Lanius cristatus	Brown Shrike	2020/12/27	1	0	16.59577924	80.61818580	https://www.inaturalist.org/observations/90049785
551	Lanius cristatus	Brown Shrike	2022/2/18	4	0	16.60132391	80.58064514	https://www.inaturalist.org/observations/107609864

552	Lanius cristatus	Brown Shrike	2020/10/24	2	0	16.62393664	80.61844333	https://www.inaturalist.org/observations/63390780
553	Lanius cristatus	Brown Shrike	2014/3/2	2	0	16.63942370	80.79750281	https://www.inaturalist.org/observations/89171359
554	Lanius cristatus	Brown Shrike	2021/9/26	2	0	16.64284764	80.48669251	https://www.inaturalist.org/observations/96472489
555	Lanius cristatus	Brown Shrike	2022/2/13	2	0	16.64674448	80.81858563	https://www.inaturalist.org/observations/106796061
556	Lanius cristatus	Brown Shrike	2021/9/26	1	0	16.64878167	80.49127164	https://www.inaturalist.org/observations/96203060
557	Lanius cristatus	Brown Shrike	2021/9/26	1	0	16.65175000	80.49308164	https://www.inaturalist.org/observations/96203079
558	Lanius cristatus	Brown Shrike	2022/2/1	1	0	16.65248889	80.78360833	https://www.inaturalist.org/observations/105958111
559	Lanius cristatus	Brown Shrike	2022/1/24	1	0	16.65258889	80.78359444	https://www.inaturalist.org/observations/105493547
560	Lanius cristatus	Brown Shrike	2022/3/6	4	0	16.65391605	80.46723317	https://www.inaturalist.org/observations/108249451
561	Lanius cristatus	Brown Shrike	2022/3/13	4	0	16.66112798	80.47830352	https://www.inaturalist.org/observations/108944395
562	Lanius cristatus	Brown Shrike	2017/10/20	3	0	16.68100634	81.16978168	https://www.inaturalist.org/observations/64937496
563	Lanius cristatus	Brown Shrike	2020/2/1	5	0	16.68739388	74.20737126	https://www.inaturalist.org/observations/38323667
564	Lanius cristatus	Brown Shrike	2022/3/22	0	0	16.69952222	80.76473056	https://www.inaturalist.org/observations/109278354
565	Lanius cristatus	Brown Shrike	2022/1/15	2	0	16.74047667	81.34118667	https://www.inaturalist.org/observations/105037010
566	Lanius cristatus	Brown Shrike	2022/1/15	1	0	16.74047667	81.34118667	https://www.inaturalist.org/observations/105562216
567	Lanius cristatus	Brown Shrike	2016/11/14	4	0	16.79660260	74.29103570	https://www.inaturalist.org/observations/30797174
568	Lanius cristatus	Brown Shrike	2019/11/8	2	0	16.88650185	75.70990551	https://www.inaturalist.org/observations/48859252
569	Lanius cristatus	Brown Shrike	2019/11/28	2	0	16.88650185	75.70990551	https://www.inaturalist.org/observations/48867770
570	Lanius cristatus	Brown Shrike	2019/12/11	3	0	16.88650185	75.70990551	https://www.inaturalist.org/observations/48893550
571	Lanius cristatus	Brown Shrike	2021/7/31	1	0	17.18246227	78.79171371	https://www.inaturalist.org/observations/90165539
572	Lanius cristatus	Brown Shrike	2022/10/4	1	0	17.31866950	78.35862240	https://www.inaturalist.org/observations/137601826
573	Lanius cristatus	Brown Shrike	2018/12/3	4	0	17.32689501	74.76560354	https://www.inaturalist.org/observations/38323696
574	Lanius cristatus	Brown Shrike	2022/9/21	2	0	17.35067800	78.45130680	https://www.inaturalist.org/observations/137029037

575	Lanius cristatus	Brown Shrike	2021/12/26	3	0	17.37626560	78.29886370	https://www.inaturalist.org/observations/104753086
576	Lanius cristatus	Brown Shrike	2019/10/6	3	0	17.38113500	78.31638070	https://www.inaturalist.org/observations/63639371
577	Lanius cristatus	Brown Shrike	2022/11/18	2	0	17.38977301	78.37702700	https://www.inaturalist.org/observations/142678461
578	Lanius cristatus	Brown Shrike	2022/12/4	0	0	17.43763720	78.47661520	https://www.inaturalist.org/observations/143719007
579	Lanius cristatus	Brown Shrike	2020/9/26	3	0	17.45536450	78.13116660	https://www.inaturalist.org/observations/63702486
580	Lanius cristatus	Brown Shrike	2022/10/12	2	0	17.45677890	78.23337430	https://www.inaturalist.org/observations/138816890
581	Lanius cristatus	Brown Shrike	2014/12/11	5	0	17.49904151	81.38165502	https://www.inaturalist.org/observations/66881021
582	Lanius cristatus	Brown Shrike	2021/12/11	2	0	17.59683001	81.68592447	https://www.inaturalist.org/observations/103518176
583	Lanius cristatus	Brown Shrike	2018/9/21	0	0	17.59833550	81.71354750	https://www.inaturalist.org/observations/88959723
584	Lanius cristatus	Brown Shrike	2021/9/27	1	0	17.59839778	81.71354759	https://www.inaturalist.org/observations/96362753
585	Lanius cristatus cristatus	Brown Shrike	2021/1/2	3	0	17.63556488	74.55682956	https://www.inaturalist.org/observations/67545186
586	Lanius cristatus	Brown Shrike	2021/12/19	4	0	17.63773667	78.06593500	https://www.inaturalist.org/observations/121718491
587	Lanius cristatus	Brown Shrike	2021/12/19	3	0	17.63997000	78.06640167	https://www.inaturalist.org/observations/121718393
588	Lanius cristatus	Brown Shrike	2022/11/21	2	0	17.65524480	78.07606880	https://www.inaturalist.org/observations/142740839
589	Lanius cristatus	Brown Shrike	2019/11/1	1	0	17.67067090	81.70914260	https://www.inaturalist.org/observations/92889121
590	Lanius cristatus	Brown Shrike	2012/7/5	3	0	17.68058160	75.31550350	https://www.inaturalist.org/observations/36498940
591	Lanius cristatus	Brown Shrike	2020/12/13	4	0	17.68292450	73.97297640	https://www.inaturalist.org/observations/71123132
592	Lanius cristatus	Brown Shrike	2022/2/27	3	0	17.68662580	78.32732450	https://www.inaturalist.org/observations/107689563
593	Lanius cristatus	Brown Shrike	2018/3/10	3	0	18.10760008	79.01929948	https://www.inaturalist.org/observations/63558713
594	Lanius cristatus	Brown Shrike	2015/9/18	3	0	18.11243720	79.01929970	https://www.inaturalist.org/observations/63558587
595	Lanius cristatus	Brown Shrike	2017/12/20	2	0	18.24826365	109.51652530	https://www.inaturalist.org/observations/12195701
596	Lanius cristatus	Brown Shrike	2016/9/20	2	0	18.25284700	109.51190900	http://www.inaturalist.org/observations/4164251
597	Lanius cristatus	Brown Shrike	2016/10/2	2	0	18.25284700	109.51190900	http://www.inaturalist.org/observations/4258947

598	Lanius cristatus	Brown Shrike	2016/10/21	2	0	18.25284700	109.51190900	http://www.inaturalist.org/observations/4410781
599	Lanius cristatus	Brown Shrike	2021/8/31	0	0	18.33159650	73.85287140	https://www.inaturalist.org/observations/93447483
600	Lanius cristatus	Brown Shrike	2021/9/8	2	0	18.34631280	74.03018260	https://www.inaturalist.org/observations/94793159
601	Lanius cristatus	Brown Shrike	2016/12/24	1	0	18.34631280	74.03018260	https://www.inaturalist.org/observations/142000194
602	Lanius cristatus	Brown Shrike	2019/9/16	2	0	18.34931939	109.48923760	https://www.inaturalist.org/observations/32942859
603	Lanius cristatus	Brown Shrike	2018/9/14	2	0	18.41934871	109.70811540	https://www.inaturalist.org/observations/16522771
604	Lanius cristatus	Brown Shrike	2018/3/25	2	0	18.50044746	108.73168950	https://www.inaturalist.org/observations/12194440
605	Lanius cristatus	Brown Shrike	2018/12/24	2	0	18.50429210	108.72980230	https://www.inaturalist.org/observations/19229901
606	Lanius cristatus	Brown Shrike	2018/12/24	2	0	18.50429210	108.72980230	https://www.inaturalist.org/observations/19229905
607	Lanius cristatus	Brown Shrike	2022/12/6	0	0	18.52313546	73.83825734	https://www.inaturalist.org/observations/143787364
608	Lanius cristatus	Brown Shrike	2018/2/11	2	0	18.53878928	108.71635440	https://www.inaturalist.org/observations/9832708
609	Lanius cristatus	Brown Shrike	2021/8/23	1	0	18.54588745	110.12078690	https://www.inaturalist.org/observations/92223444
610	Lanius cristatus	Brown Shrike	2014/10/6	1	0	18.55403120	73.79045760	https://www.inaturalist.org/observations/142674495
611	Lanius cristatus	Brown Shrike	2018/2/9	2	0	18.60315524	108.71063240	https://www.inaturalist.org/observations/10243247
612	Lanius cristatus	Brown Shrike	2018/2/9	2	0	18.60315524	108.71063240	https://www.inaturalist.org/observations/10243252
613	Lanius cristatus	Brown Shrike	2021/2/17	2	0	18.70353626	84.40413415	https://www.inaturalist.org/observations/69898936
614	Lanius cristatus	Brown Shrike	2022/11/25	0	0	18.77866000	81.99711080	https://www.inaturalist.org/observations/143342290
615	Lanius cristatus	Brown Shrike	2018/10/6	2	0	18.99888640	109.07169050	https://www.inaturalist.org/observations/17669997
616	Lanius cristatus	Brown Shrike	2019/10/4	2	0	19.04998226	108.70949620	https://www.inaturalist.org/observations/35530305
617	Lanius cristatus	Brown Shrike	2019/9/14	2	0	19.06159868	108.72460810	https://www.inaturalist.org/observations/32937029
618	Lanius cristatus	Brown Shrike	2020/1/15	3	0	19.06696688	109.13983110	https://www.inaturalist.org/observations/37780935
619	Lanius cristatus	Brown Shrike	2022/11/27	2	0	19.09509667	82.02064833	https://www.inaturalist.org/observations/143169921
620	Lanius cristatus	Brown Shrike	2020/9/14	2	0	19.24391718	110.45773940	https://www.inaturalist.org/observations/64852557

621	Lanius cristatus	Brown Shrike	2022/1/15	0	0	19.73844333	109.30207170	https://www.inaturalist.org/observations/125272349
622	Lanius cristatus	Brown Shrike	2019/10/16	5	0	19.74911955	81.39411327	https://www.inaturalist.org/observations/34865564
623	Lanius cristatus	Brown Shrike	2019/12/16	3	0	19.86660882	86.11101589	https://www.inaturalist.org/observations/40352141
624	Lanius cristatus	Brown Shrike	2021/5/21	2	0	19.91370362	82.64322940	https://www.inaturalist.org/observations/79633204
625	Lanius cristatus	Brown Shrike	2021/11/27	2	0	20.04638000	110.20959670	https://www.inaturalist.org/observations/101991893
626	Lanius cristatus	Brown Shrike	2016/12/19	2	0	20.40032712	85.82660573	https://www.inaturalist.org/observations/110266631
627	Lanius cristatus	Brown Shrike	2019/1/19	3	0	20.66559832	81.55340530	https://www.inaturalist.org/observations/37777269
628	Lanius cristatus	Brown Shrike	2020/10/31	4	0	20.67119885	77.09259412	https://www.inaturalist.org/observations/63876633
629	Lanius cristatus	Brown Shrike	2011/8/31	2	0	20.69950011	116.72639220	https://www.inaturalist.org/observations/40014085
630	Lanius cristatus	Brown Shrike	2021/3/31	2	0	20.97076402	81.49931554	https://www.inaturalist.org/observations/72511954
631	Lanius cristatus cristatus	Brown Shrike	2021/3/15	2	0	20.98462279	75.53618079	https://www.inaturalist.org/observations/71349186
632	Lanius cristatus cristatus	Brown Shrike	2021/1/1	4	0	20.98960524	75.54502911	https://www.inaturalist.org/observations/67400583
633	Lanius cristatus	Brown Shrike	2021/5/4	1	0	21.01266268	81.45391613	https://www.inaturalist.org/observations/77238765
634	Lanius cristatus	Brown Shrike	2022/10/5	2	0	21.01379191	81.45405293	https://www.inaturalist.org/observations/142429826
635	Lanius cristatus	Brown Shrike	2021/10/10	1	0	21.04801466	75.86916804	https://www.inaturalist.org/observations/97763450
636	Lanius cristatus	Brown Shrike	2021/9/21	1	0	21.08152254	81.40923254	https://www.inaturalist.org/observations/95730977
637	Lanius cristatus	Brown Shrike	2021/10/18	1	0	21.08662762	81.50319301	https://www.inaturalist.org/observations/98594572
638	Lanius cristatus	Brown Shrike	2021/10/25	0	0	21.16231734	81.44367814	https://www.inaturalist.org/observations/99851753
639	Lanius cristatus	Brown Shrike	2020/11/21	3	0	21.20267383	80.74586727	https://www.inaturalist.org/observations/66920768
640	Lanius cristatus	Brown Shrike	2021/4/16	1	0	21.20626565	81.60784308	https://www.inaturalist.org/observations/74646570
641	Lanius cristatus	Brown Shrike	2021/4/16	0	0	21.20948261	81.43681504	https://www.inaturalist.org/observations/74212045
642	Lanius cristatus	Brown Shrike	2019/1/1	0	0	21.21123234	81.43891957	https://www.inaturalist.org/observations/139509645
643	Lanius cristatus	Brown Shrike	2018/3/16	4	0	21.28470333	81.68143000	https://www.inaturalist.org/observations/10257074

644	Lanius cristatus	Brown Shrike	2019/12/19	3	0	21.32356052	81.73493903	https://www.inaturalist.org/observations/37580814
645	Lanius cristatus	Brown Shrike	2020/1/26	3	0	21.40602820	81.86887510	https://www.inaturalist.org/observations/121766486
646	Lanius cristatus	Brown Shrike	2018/10/29	4	0	21.42021477	81.89239595	https://www.inaturalist.org/observations/17959181
647	Lanius cristatus	Brown Shrike	2019/10/8	2	0	21.45187800	109.05539500	https://www.inaturalist.org/observations/34316859
648	Lanius cristatus	Brown Shrike	2016/4/27	3	0	21.63576500	87.56219167	https://www.inaturalist.org/observations/61388327
649	Lanius cristatus	Brown Shrike	2015/1/16	2	0	21.63595490	87.56299431	https://www.inaturalist.org/observations/58693791
650	Lanius cristatus	Brown Shrike	2015/1/16	4	0	21.63653006	87.56187777	https://www.inaturalist.org/observations/61137081
651	Lanius cristatus	Brown Shrike	2016/2/3	3	0	21.64377000	87.56583000	https://www.inaturalist.org/observations/58690647
652	Lanius cristatus	Brown Shrike	2015/1/1	4	0	21.64459142	87.57297024	https://www.inaturalist.org/observations/61329617
653	Lanius cristatus	Brown Shrike	2021/1/9	1	0	21.65192759	111.74996620	https://www.inaturalist.org/observations/67785814
654	Lanius cristatus	Brown Shrike	2014/12/26	4	0	21.65805361	87.57264234	https://www.inaturalist.org/observations/61329615
655	Lanius cristatus	Brown Shrike	2021/2/22	2	0	21.66869363	81.31583367	https://www.inaturalist.org/observations/69991858
656	Lanius cristatus	Brown Shrike	2019/3/16	2	0	21.77178306	79.27518306	https://www.inaturalist.org/observations/52923214
657	Lanius cristatus	Brown Shrike	2020/10/3	2	0	21.80763510	83.31094413	https://www.inaturalist.org/observations/79921563
658	Lanius cristatus	Brown Shrike	2021/2/2	3	0	21.83238812	81.80266734	https://www.inaturalist.org/observations/68952481
659	Lanius cristatus	Brown Shrike	2018/1/25	3	0	21.83585950	88.88420110	https://www.inaturalist.org/observations/55579884
660	Lanius cristatus	Brown Shrike	2021/2/21	1	0	21.83628651	88.88347154	https://www.inaturalist.org/observations/81263984
661	Lanius cristatus	Brown Shrike	2021/4/26	3	0	21.89534424	87.58025111	https://www.inaturalist.org/observations/116141090
662	Lanius cristatus	Brown Shrike	2021/4/26	3	0	21.89534853	87.58030936	https://www.inaturalist.org/observations/116141091
663	Lanius cristatus	Brown Shrike	2021/4/26	2	0	21.89535911	87.58029961	https://www.inaturalist.org/observations/116141098
664	Lanius cristatus	Brown Shrike	2019/10/11	2	0	21.91855167	120.85027830	https://www.inaturalist.org/observations/37983821
665	Lanius cristatus	Brown Shrike	2020/11/25	1	0	21.93277000	101.24932330	https://www.inaturalist.org/observations/67952555
666	Lanius cristatus	Brown Shrike	2021/10/9	1	0	21.93558164	120.82187430	https://www.inaturalist.org/observations/97650657

667	Lanius cristatus	Brown Shrike	2019/9/21	2	0	21.94351540	120.82663510	https://www.inaturalist.org/observations/33186969
668	Lanius cristatus	Brown Shrike	2022/9/11	1	0	21.94833070	120.77975160	https://www.inaturalist.org/observations/134748395
669	Lanius cristatus	Brown Shrike	2022/9/12	1	0	21.94833070	120.77975160	https://www.inaturalist.org/observations/135381331
670	Lanius cristatus	Brown Shrike	2022/9/11	1	0	21.94861116	120.79219270	https://www.inaturalist.org/observations/134575945
671	Lanius cristatus	Brown Shrike	2022/9/17	1	0	21.95339470	120.82338470	https://www.inaturalist.org/observations/136118713
672	Lanius cristatus	Brown Shrike	2022/9/17	2	0	21.95339481	120.82338470	https://www.inaturalist.org/observations/135464115
673	Lanius cristatus	Brown Shrike	2019/9/16	2	0	21.95407721	120.82299410	https://www.inaturalist.org/observations/32827500
674	Lanius cristatus	Brown Shrike	2019/10/10	2	0	21.96980678	120.78820710	https://www.inaturalist.org/observations/34455064
675	Lanius cristatus	Brown Shrike	2019/12/1	2	0	21.97045200	120.74854400	https://www.inaturalist.org/observations/45839438
676	Lanius cristatus	Brown Shrike	2019/12/1	2	0	21.98540628	120.73557030	https://www.inaturalist.org/observations/45758933
677	Lanius cristatus	Brown Shrike	2019/10/18	2	0	21.98652649	120.74059300	https://www.inaturalist.org/observations/37870612
678	Lanius cristatus	Brown Shrike	2019/11/5	2	0	21.98802170	120.74014560	https://www.inaturalist.org/observations/35315946
679	Lanius cristatus	Brown Shrike	2020/9/24	2	0	21.99066624	120.82589590	https://www.inaturalist.org/observations/60686293
680	Lanius cristatus	Brown Shrike	2019/12/1	2	0	21.99081262	120.73646230	https://www.inaturalist.org/observations/45760046
681	Lanius cristatus	Brown Shrike	2019/9/20	2	0	21.99992774	120.73493720	https://www.inaturalist.org/observations/46697676
682	Lanius cristatus	Brown Shrike	2022/2/24	4	0	22.00770389	120.74879310	https://www.inaturalist.org/observations/107357609
683	Lanius cristatus	Brown Shrike	2019/9/22	1	0	22.01362480	120.74186660	https://www.inaturalist.org/observations/33249837
684	Lanius cristatus	Brown Shrike	2019/10/14	3	0	22.01946274	120.74030730	https://www.inaturalist.org/observations/34357808
685	Lanius cristatus	Brown Shrike	2020/12/29	3	0	22.02055472	120.83856430	https://www.inaturalist.org/observations/67236255
686	Lanius cristatus	Brown Shrike	2019/10/12	3	0	22.02140427	120.83965300	https://www.inaturalist.org/observations/34304812
687	Lanius cristatus lucionensis	Brown Shrike	2018/10/11	2	0	22.02741333	120.85542330	https://www.inaturalist.org/observations/17496294
688	Lanius cristatus	Brown Shrike	2020/9/4	1	0	22.02974084	121.57996890	https://www.inaturalist.org/observations/106670964
689	Lanius cristatus	Brown Shrike	2018/1/21	4	0	22.03464342	72.02090860	https://www.inaturalist.org/observations/30384153

690	Lanius cristatus	Brown Shrike	2019/10/7	2	0	22.03823871	120.84705140	https://www.inaturalist.org/observations/34431357
691	Lanius cristatus	Brown Shrike	2020/9/10	2	0	22.04356160	121.54841800	https://www.inaturalist.org/observations/61667012
692	Lanius cristatus	Brown Shrike	2017/4/27	1	0	22.04356200	121.54841800	https://www.inaturalist.org/observations/137095544
693	Lanius cristatus	Brown Shrike	2019/12/22	2	0	22.04410790	72.02028130	https://www.inaturalist.org/observations/55904382
694	Lanius cristatus	Brown Shrike	2017/12/23	4	0	22.04410790	72.02028130	https://www.inaturalist.org/observations/56417961
695	Lanius cristatus	Brown Shrike	2019/3/8	3	0	22.05971510	82.04343800	https://www.inaturalist.org/observations/60035823
696	Lanius cristatus	Brown Shrike	2021/8/20	1	0	22.06030667	82.05720000	https://www.inaturalist.org/observations/93631071
697	Lanius cristatus	Brown Shrike	2021/8/15	1	0	22.06740500	82.20358000	https://www.inaturalist.org/observations/93615052
698	Lanius cristatus	Brown Shrike	2020/10/11	2	0	22.06829602	111.69132310	https://www.inaturalist.org/observations/62380747
699	Lanius cristatus	Brown Shrike	2019/11/10	3	0	22.07965450	82.14091520	https://www.inaturalist.org/observations/60429511
700	Lanius cristatus	Brown Shrike	2022/9/8	2	0	22.08022943	121.50541170	https://www.inaturalist.org/observations/134693172
701	Lanius cristatus	Brown Shrike	2020/12/2	2	0	22.08111512	120.74698960	https://www.inaturalist.org/observations/65983180
702	Lanius cristatus	Brown Shrike	2022/11/10	0	0	22.08983705	86.11712795	https://www.inaturalist.org/observations/141670871
703	Lanius cristatus	Brown Shrike	2020/5/3	2	0	22.10111302	113.52423450	https://www.inaturalist.org/observations/67285905
704	Lanius cristatus	Brown Shrike	2020/4/25	2	0	22.12408911	113.56960850	https://www.inaturalist.org/observations/43283810
705	Lanius cristatus	Brown Shrike	2021/1/4	2	0	22.12580633	82.14299995	https://www.inaturalist.org/observations/80077553
706	Lanius cristatus	Brown Shrike	2020/4/30	2	0	22.12597217	113.56092000	https://www.inaturalist.org/observations/44346440
707	Lanius cristatus	Brown Shrike	2018/3/26	4	0	22.12762692	88.83064270	https://www.inaturalist.org/observations/10812250
708	Lanius cristatus	Brown Shrike	2021/9/27	1	0	22.13425500	113.56922830	https://www.inaturalist.org/observations/118814072
709	Lanius cristatus	Brown Shrike	2019/5/4	2	0	22.13543368	113.57524400	https://www.inaturalist.org/observations/24505358
710	Lanius cristatus	Brown Shrike	2022/12/24	0	0	22.15155460	113.55906620	https://www.inaturalist.org/observations/144986042
711	Lanius cristatus	Brown Shrike	2022/12/24	0	0	22.15309575	113.55939210	https://www.inaturalist.org/observations/144986492
712	Lanius cristatus	Brown Shrike	2021/1/22	2	0	22.15349450	113.56004500	https://www.inaturalist.org/observations/68443386

713	Lanius cristatus	Brown Shrike	2020/4/24	2	0	22.15354589	113.56046860	https://www.inaturalist.org/observations/43052186
714	Lanius cristatus	Brown Shrike	2021/1/25	1	0	22.15355275	113.56100540	https://www.inaturalist.org/observations/84331109
715	Lanius cristatus	Brown Shrike	2022/11/27	0	0	22.15365317	113.56037350	https://www.inaturalist.org/observations/143040336
716	Lanius cristatus	Brown Shrike	2021/2/20	2	0	22.15367167	113.56038830	https://www.inaturalist.org/observations/69854253
717	Lanius cristatus	Brown Shrike	2022/9/21	1	0	22.15383698	113.55961770	https://www.inaturalist.org/observations/136159285
718	Lanius cristatus	Brown Shrike	2021/1/7	2	0	22.15389167	113.55999670	https://www.inaturalist.org/observations/67698278
719	Lanius cristatus	Brown Shrike	2009/5/2	1	0	22.16114300	114.25610200	https://www.inaturalist.org/observations/142733619
720	Lanius cristatus	Brown Shrike	2022/5/2	1	0	22.16364493	114.25430310	https://www.inaturalist.org/observations/114634850
721	Lanius cristatus	Brown Shrike	2015/5/12	1	0	22.16457574	114.26517650	https://www.inaturalist.org/observations/36282792
722	Lanius cristatus	Brown Shrike	2022/5/3	1	0	22.16461556	114.25668580	https://www.inaturalist.org/observations/115251206
723	Lanius cristatus	Brown Shrike	2021/5/19	1	0	22.16484500	114.25445000	https://www.inaturalist.org/observations/112945194
724	Lanius cristatus	Brown Shrike	2021/5/4	1	0	22.16541533	114.25548770	https://www.inaturalist.org/observations/77418910
725	Lanius cristatus	Brown Shrike	2022/5/2	2	0	22.16553672	114.25812560	https://www.inaturalist.org/observations/114697850
726	Lanius cristatus	Brown Shrike	2022/5/2	3	0	22.16650481	114.25467360	https://www.inaturalist.org/observations/114686681
727	Lanius cristatus	Brown Shrike	2022/5/3	1	0	22.16923240	114.25985330	https://www.inaturalist.org/observations/115285471
728	Lanius cristatus	Brown Shrike	2019/5/22	1	0	22.19873721	113.54387280	https://www.inaturalist.org/observations/95652232
729	Lanius cristatus	Brown Shrike	2022/5/19	1	0	22.19891632	113.55263020	https://www.inaturalist.org/observations/117731095
730	Lanius cristatus	Brown Shrike	2020/10/1	2	0	22.20009756	113.54785430	https://www.inaturalist.org/observations/61367605
731	Lanius cristatus	Brown Shrike	2020/9/29	2	0	22.20035685	113.54783110	https://www.inaturalist.org/observations/61206613
732	Lanius cristatus	Brown Shrike	2021/9/27	1	0	22.20048284	113.54796560	https://www.inaturalist.org/observations/96320081
733	Lanius cristatus	Brown Shrike	2022/5/6	1	0	22.20059104	113.54806420	https://www.inaturalist.org/observations/115714664
734	Lanius cristatus	Brown Shrike	2021/5/10	1	0	22.20159912	113.54779260	https://www.inaturalist.org/observations/78194696
735	Lanius cristatus	Brown Shrike	2022/5/2	1	0	22.20737259	113.54790130	https://www.inaturalist.org/observations/114631870

736	Lanius cristatus	Brown Shrike	2019/5/9	2	0	22.20793573	113.54793940	https://www.inaturalist.org/observations/24829896
737	Lanius cristatus	Brown Shrike	2022/10/16	0	0	22.20797993	113.54771370	https://www.inaturalist.org/observations/138968030
738	Lanius cristatus	Brown Shrike	2022/5/2	2	0	22.20810583	114.26028110	https://www.inaturalist.org/observations/114641050
739	Lanius cristatus	Brown Shrike	2019/4/29	2	0	22.20814557	113.54790490	https://www.inaturalist.org/observations/23888044
740	Lanius cristatus	Brown Shrike	2022/5/2	1	0	22.20814681	114.26028110	https://www.inaturalist.org/observations/114641347
741	Lanius cristatus	Brown Shrike	2022/5/2	2	0	22.20814681	114.26028110	https://www.inaturalist.org/observations/114643303
742	Lanius cristatus	Brown Shrike	2022/5/2	1	0	22.20814681	114.26028110	https://www.inaturalist.org/observations/114643483
743	Lanius cristatus	Brown Shrike	2022/5/2	2	0	22.20814681	114.26028110	https://www.inaturalist.org/observations/114643752
744	Lanius cristatus	Brown Shrike	2020/10/4	2	0	22.20818619	113.54777820	https://www.inaturalist.org/observations/62254048
745	Lanius cristatus	Brown Shrike	2019/5/10	2	0	22.20829281	113.54777630	https://www.inaturalist.org/observations/24925852
746	Lanius cristatus	Brown Shrike	2019/5/11	2	0	22.20833055	113.54799910	https://www.inaturalist.org/observations/24932566
747	Lanius cristatus	Brown Shrike	2022/9/25	1	0	22.20834712	114.06232370	https://www.inaturalist.org/observations/136393197
748	Lanius cristatus	Brown Shrike	2019/5/9	2	0	22.20886695	113.54750840	https://www.inaturalist.org/observations/27844352
749	Lanius cristatus	Brown Shrike	2020/12/27	2	0	22.21527652	113.54376320	https://www.inaturalist.org/observations/67129564
750	Lanius cristatus	Brown Shrike	2021/1/2	3	0	22.22018390	114.03896700	https://www.inaturalist.org/observations/67705347
751	Lanius cristatus	Brown Shrike	2020/4/28	1	0	22.22379026	114.07255430	https://www.inaturalist.org/observations/119050024
752	Lanius cristatus	Brown Shrike	2014/5/10	3	0	22.22488200	114.11450900	http://www.inaturalist.org/observations/673657
753	Lanius cristatus	Brown Shrike	2020/4/25	2	0	22.22558286	113.84743970	https://www.inaturalist.org/observations/44097928
754	Lanius cristatus	Brown Shrike	2019/9/22	2	0	22.22558929	113.84657540	https://www.inaturalist.org/observations/33798337
755	Lanius cristatus	Brown Shrike	2020/4/26	2	0	22.22575924	114.11271990	https://www.inaturalist.org/observations/43657485
756	Lanius cristatus lucionensis	Brown Shrike	2018/4/29	2	0	22.22854524	113.84872290	https://www.inaturalist.org/observations/11836314
757	Lanius cristatus	Brown Shrike	2019/10/22	3	0	22.23378053	114.17086370	https://www.inaturalist.org/observations/34727730
758	Lanius cristatus	Brown Shrike	2022/9/22	2	0	22.23416619	113.99995530	https://www.inaturalist.org/observations/137032429

759	Lanius cristatus	Brown Shrike	2018/10/1	3	0	22.23565303	114.11055010	https://www.inaturalist.org/observations/17304402
760	Lanius cristatus	Brown Shrike	2022/5/2	3	0	22.23756167	114.19342830	https://www.inaturalist.org/observations/115046322
761	Lanius cristatus	Brown Shrike	2022/5/2	1	0	22.24044698	114.15030410	https://www.inaturalist.org/observations/114617792
762	Lanius cristatus	Brown Shrike	2017/1/9	2	0	22.24057100	113.97447700	https://www.inaturalist.org/observations/11448660
763	Lanius cristatus	Brown Shrike	2021/2/16	2	0	22.24513333	114.17613830	https://www.inaturalist.org/observations/69662098
764	Lanius cristatus	Brown Shrike	2021/4/16	3	0	22.24517475	114.17603970	https://www.inaturalist.org/observations/74612051
765	Lanius cristatus	Brown Shrike	2020/4/4	2	0	22.24882591	113.94221310	https://www.inaturalist.org/observations/41510990
766	Lanius cristatus	Brown Shrike	2019/12/20	3	0	22.25207297	114.13359720	https://www.inaturalist.org/observations/36799470
767	Lanius cristatus	Brown Shrike	2021/5/3	1	0	22.25643409	114.38741320	https://www.inaturalist.org/observations/77147655
768	Lanius cristatus	Brown Shrike	2022/10/2	1	0	22.25657785	114.29453500	https://www.inaturalist.org/observations/137485249
769	Lanius cristatus	Brown Shrike	2020/12/9	2	0	22.26402283	120.66604960	https://www.inaturalist.org/observations/66319982
770	Lanius cristatus	Brown Shrike	2022/2/16	1	0	22.26668946	113.99247000	https://www.inaturalist.org/observations/106848270
771	Lanius cristatus	Brown Shrike	2022/5/6	1	0	22.26670755	114.18333330	https://www.inaturalist.org/observations/115711217
772	Lanius cristatus	Brown Shrike	2022/5/6	1	0	22.26670755	114.18333330	https://www.inaturalist.org/observations/115711265
773	Lanius cristatus	Brown Shrike	2022/9/24	2	0	22.26677799	113.94175100	https://www.inaturalist.org/observations/136561112
774	Lanius cristatus	Brown Shrike	2022/10/4	1	0	22.26917459	120.88325010	https://www.inaturalist.org/observations/142204376
775	Lanius cristatus	Brown Shrike	2010/4/18	1	0	22.26955200	113.99153200	https://www.inaturalist.org/observations/109742105
776	Lanius cristatus	Brown Shrike	2017/2/3	4	0	22.27172000	80.62366833	https://www.inaturalist.org/observations/5183081
777	Lanius cristatus	Brown Shrike	2022/10/3	1	0	22.27226794	120.86929960	https://www.inaturalist.org/observations/142575769
778	Lanius cristatus	Brown Shrike	2022/5/4	1	0	22.27251639	114.20635630	https://www.inaturalist.org/observations/115321182
779	Lanius cristatus	Brown Shrike	2017/2/3	5	0	22.27365667	80.62430500	https://www.inaturalist.org/observations/5183275
780	Lanius cristatus	Brown Shrike	2021/5/6	1	0	22.27664222	114.12369110	https://www.inaturalist.org/observations/77604939
781	Lanius cristatus	Brown Shrike	2021/5/7	1	0	22.27728610	114.20568420	https://www.inaturalist.org/observations/77713107

782	Lanius cristatus	Brown Shrike	2021/10/25	2	0	22.27741027	114.12196530	https://www.inaturalist.org/observations/99322331
783	Lanius cristatus	Brown Shrike	2013/5/4	4	0	22.27831510	114.17469500	http://www.inaturalist.org/observations/3952650
784	Lanius cristatus	Brown Shrike	2022/5/9	1	0	22.28015589	114.21083730	https://www.inaturalist.org/observations/116250535
785	Lanius cristatus	Brown Shrike	2022/5/2	1	0	22.28190391	114.22214070	https://www.inaturalist.org/observations/114611769
786	Lanius cristatus	Brown Shrike	2019/5/11	3	0	22.28511810	114.14567570	https://www.inaturalist.org/observations/25094987
787	Lanius cristatus	Brown Shrike	2020/4/26	2	0	22.28589986	114.15608180	https://www.inaturalist.org/observations/43657168
788	Lanius cristatus	Brown Shrike	2021/11/1	1	0	22.28986391	114.29977800	https://www.inaturalist.org/observations/99943357
789	Lanius cristatus	Brown Shrike	2019/4/20	2	0	22.29608708	114.16911580	https://www.inaturalist.org/observations/67290694
790	Lanius cristatus	Brown Shrike	2020/4/26	2	0	22.29646921	114.27601600	https://www.inaturalist.org/observations/44103508
791	Lanius cristatus	Brown Shrike	2020/4/25	2	0	22.29682969	114.27676330	https://www.inaturalist.org/observations/44097782
792	Lanius cristatus	Brown Shrike	2021/1/9	2	0	22.29780667	114.27308170	https://www.inaturalist.org/observations/67792893
793	Lanius cristatus	Brown Shrike	2020/4/27	2	0	22.29794861	114.27712070	https://www.inaturalist.org/observations/44134312
794	Lanius cristatus	Brown Shrike	2019/12/7	2	0	22.29837828	114.27637440	https://www.inaturalist.org/observations/36427709
795	Lanius cristatus lucionensis	Brown Shrike	2022/1/23	0	0	22.29881230	114.17217460	https://www.inaturalist.org/observations/108810576
796	Lanius cristatus	Brown Shrike	2021/11/24	2	0	22.29925966	114.17064640	https://www.inaturalist.org/observations/101927980
797	Lanius cristatus	Brown Shrike	2020/4/27	2	0	22.29970387	114.16995910	https://www.inaturalist.org/observations/43880376
798	Lanius cristatus	Brown Shrike	2022/5/3	1	0	22.29973972	114.18644810	https://www.inaturalist.org/observations/115062536
799	Lanius cristatus	Brown Shrike	2022/2/7	3	0	22.30028392	114.17016090	https://www.inaturalist.org/observations/106309974
800	Lanius cristatus	Brown Shrike	2022/1/15	1	0	22.30043871	114.17019250	https://www.inaturalist.org/observations/104965803
801	Lanius cristatus	Brown Shrike	2022/5/7	2	0	22.30045267	114.17007710	https://www.inaturalist.org/observations/115883950
802	Lanius cristatus	Brown Shrike	2020/1/17	2	0	22.30073330	114.17000590	https://www.inaturalist.org/observations/37678126
803	Lanius cristatus	Brown Shrike	2019/4/28	3	0	22.30075370	114.17005020	https://www.inaturalist.org/observations/23821630
804	Lanius cristatus	Brown Shrike	2022/1/15	1	0	22.30094837	114.16964060	https://www.inaturalist.org/observations/104961228

805	Lanius cristatus	Brown Shrike	2022/1/15	1	0	22.30120727	114.16931960	https://www.inaturalist.org/observations/104968717
806	Lanius cristatus	Brown Shrike	2022/11/7	2	0	22.30218367	114.25506990	https://www.inaturalist.org/observations/141422731
807	Lanius cristatus	Brown Shrike	2022/2/24	1	0	22.30219985	114.25525760	https://www.inaturalist.org/observations/107363610
808	Lanius cristatus	Brown Shrike	2022/11/11	1	0	22.30220404	114.25502930	https://www.inaturalist.org/observations/141899958
809	Lanius cristatus	Brown Shrike	2022/1/25	1	0	22.30227452	114.25492270	https://www.inaturalist.org/observations/105560744
810	Lanius cristatus	Brown Shrike	2022/12/24	1	0	22.30270386	114.25495910	https://www.inaturalist.org/observations/144969701
811	Lanius cristatus	Brown Shrike	2022/12/23	2	0	22.30273833	114.25496670	https://www.inaturalist.org/observations/144901359
812	Lanius cristatus	Brown Shrike	2022/3/12	1	0	22.30303563	114.25386750	https://www.inaturalist.org/observations/108504537
813	Lanius cristatus	Brown Shrike	2019/5/9	3	0	22.30582647	113.91985250	https://www.inaturalist.org/observations/24897927
814	Lanius cristatus	Brown Shrike	2022/11/5	2	0	22.30701076	114.19099470	https://www.inaturalist.org/observations/141133661
815	Lanius cristatus	Brown Shrike	2022/1/1	1	0	22.30704000	114.19035330	https://www.inaturalist.org/observations/105398815
816	Lanius cristatus	Brown Shrike	2022/11/6	1	0	22.30705472	114.19029310	https://www.inaturalist.org/observations/141249217
817	Lanius cristatus	Brown Shrike	2022/1/1	3	0	22.30706238	114.19036810	https://www.inaturalist.org/observations/104105808
818	Lanius cristatus	Brown Shrike	2021/12/30	2	0	22.30710600	114.19042800	https://www.inaturalist.org/observations/103945504
819	Lanius cristatus	Brown Shrike	2022/1/2	3	0	22.30735678	114.19076260	https://www.inaturalist.org/observations/104197324
820	Lanius cristatus	Brown Shrike	2022/1/1	2	0	22.30757940	114.19093750	https://www.inaturalist.org/observations/104154986
821	Lanius cristatus	Brown Shrike	2022/1/2	2	0	22.30757940	114.19093750	https://www.inaturalist.org/observations/112989284
822	Lanius cristatus	Brown Shrike	2021/12/27	3	0	22.30757964	114.19092890	https://www.inaturalist.org/observations/103810348
823	Lanius cristatus	Brown Shrike	2018/12/26	2	0	22.30877101	114.26533760	https://www.inaturalist.org/observations/19857422
824	Lanius cristatus	Brown Shrike	2022/1/4	4	0	22.31021000	73.29345333	https://www.inaturalist.org/observations/120809374
825	Lanius cristatus	Brown Shrike	2014/10/10	5	0	22.31061500	88.67460300	http://www.inaturalist.org/observations/918702
826	Lanius cristatus	Brown Shrike	2020/2/23	3	0	22.31306017	114.18327360	https://www.inaturalist.org/observations/101690541
827	Lanius cristatus	Brown Shrike	2020/2/23	1	0	22.31306017	114.18327360	https://www.inaturalist.org/observations/101690691

828	Lanius cristatus	Brown Shrike	2022/4/15	3	0	22.31332588	114.26065830	https://www.inaturalist.org/observations/116640835
829	Lanius cristatus lucionensis	Brown Shrike	2019/5/4	3	0	22.31772256	114.18321500	https://www.inaturalist.org/observations/30412508
830	Lanius cristatus	Brown Shrike	2020/9/16	2	0	22.31791707	114.18378230	https://www.inaturalist.org/observations/59858626
831	Lanius cristatus	Brown Shrike	2017/11/26	2	0	22.31794783	114.18379430	https://www.inaturalist.org/observations/9447636
832	Lanius cristatus	Brown Shrike	2021/11/21	1	0	22.31800453	114.18377310	https://www.inaturalist.org/observations/101790712
833	Lanius cristatus	Brown Shrike	2022/9/24	2	0	22.31818526	114.18449170	https://www.inaturalist.org/observations/136379700
834	Lanius cristatus	Brown Shrike	2022/9/26	2	0	22.31820616	114.18400460	https://www.inaturalist.org/observations/136569172
835	Lanius cristatus	Brown Shrike	2019/9/14	2	0	22.31820986	114.18346840	https://www.inaturalist.org/observations/32679460
836	Lanius cristatus	Brown Shrike	2022/10/13	1	0	22.31830601	87.86356855	https://www.inaturalist.org/observations/138600098
837	Lanius cristatus	Brown Shrike	2018/12/19	2	0	22.31833428	114.18329010	https://www.inaturalist.org/observations/30412523
838	Lanius cristatus	Brown Shrike	2018/10/1	3	0	22.31840330	114.18395360	https://www.inaturalist.org/observations/17091036
839	Lanius cristatus	Brown Shrike	2022/2/24	3	0	22.31840330	114.18395360	https://www.inaturalist.org/observations/107370651
840	Lanius cristatus	Brown Shrike	2020/4/13	2	0	22.31841084	114.18395350	https://www.inaturalist.org/observations/42084579
841	Lanius cristatus	Brown Shrike	2022/10/16	1	0	22.31850700	114.17553390	https://www.inaturalist.org/observations/139096401
842	Lanius cristatus	Brown Shrike	2022/9/23	2	0	22.31857191	114.18364420	https://www.inaturalist.org/observations/136125785
843	Lanius cristatus	Brown Shrike	2021/1/2	3	0	22.32022040	114.27603400	https://www.inaturalist.org/observations/70599790
844	Lanius cristatus	Brown Shrike	2016/11/29	3	0	22.32787411	80.58360099	https://www.inaturalist.org/observations/37750993
845	Lanius cristatus	Brown Shrike	2022/10/4	1	0	22.32819647	114.10197900	https://www.inaturalist.org/observations/140365391
846	Lanius cristatus	Brown Shrike	2014/3/26	4	0	22.32828400	114.20620700	http://www.inaturalist.org/observations/592256
847	Lanius cristatus	Brown Shrike	2017/3/8	5	0	22.32838936	80.60695410	https://www.inaturalist.org/observations/5464998
848	Lanius cristatus lucionensis	Brown Shrike	2019/9/7	1	0	22.33111112	114.18291910	https://www.inaturalist.org/observations/32260865
849	Lanius cristatus	Brown Shrike	2019/5/7	4	0	22.33157756	114.18269380	https://www.inaturalist.org/observations/24722298
850	Lanius cristatus	Brown Shrike	2019/5/5	2	0	22.33159612	114.18285100	https://www.inaturalist.org/observations/24578138

851	Lanius cristatus lucionensis	Brown Shrike	2022/9/20	1	0	22.33185830	114.18269930	https://www.inaturalist.org/observations/135768417
852	Lanius cristatus	Brown Shrike	2022/2/12	1	0	22.33203474	114.18919370	https://www.inaturalist.org/observations/106654148
853	Lanius cristatus	Brown Shrike	2020/2/1	2	0	22.33232134	114.18958660	https://www.inaturalist.org/observations/38178070
854	Lanius cristatus	Brown Shrike	2020/5/14	3	0	22.33326561	114.18540380	https://www.inaturalist.org/observations/45857769
855	Lanius cristatus	Brown Shrike	2022/3/6	1	0	22.33342500	114.14180000	https://www.inaturalist.org/observations/108021672
856	Lanius cristatus	Brown Shrike	2022/2/1	2	0	22.33351926	114.14178070	https://www.inaturalist.org/observations/113348457
857	Lanius cristatus	Brown Shrike	2021/3/20	1	0	22.33407770	80.61120460	https://www.inaturalist.org/observations/73365594
858	Lanius cristatus	Brown Shrike	2020/5/10	3	0	22.33414560	114.13846530	https://www.inaturalist.org/observations/45475605
859	Lanius cristatus	Brown Shrike	2022/3/11	2	0	22.33414907	114.18494610	https://www.inaturalist.org/observations/108342196
860	Lanius cristatus	Brown Shrike	2022/5/9	2	0	22.33416485	114.18507480	https://www.inaturalist.org/observations/116259463
861	Lanius cristatus	Brown Shrike	2022/12/6	2	0	22.33421395	114.13849930	https://www.inaturalist.org/observations/143802543
862	Lanius cristatus	Brown Shrike	2020/4/27	2	0	22.33428020	114.18511380	https://www.inaturalist.org/observations/43857133
863	Lanius cristatus lucionensis	Brown Shrike	2020/4/28	3	0	22.33440086	114.18508880	https://www.inaturalist.org/observations/44107689
864	Lanius cristatus	Brown Shrike	2020/3/21	2	0	22.33447561	114.13792030	https://www.inaturalist.org/observations/40454863
865	Lanius cristatus	Brown Shrike	2014/3/6	3	0	22.33451320	80.61151300	https://www.inaturalist.org/observations/52901137
866	Lanius cristatus	Brown Shrike	2019/2/12	1	0	22.33451320	80.61151300	https://www.inaturalist.org/observations/90521743
867	Lanius cristatus	Brown Shrike	2022/2/13	1	0	22.33461745	114.13961260	https://www.inaturalist.org/observations/107606785
868	Lanius cristatus	Brown Shrike	2022/2/11	2	0	22.33462681	114.13969780	https://www.inaturalist.org/observations/107086707
869	Lanius cristatus	Brown Shrike	2020/11/11	2	0	22.33465417	114.13844660	https://www.inaturalist.org/observations/82510759
870	Lanius cristatus	Brown Shrike	2020/2/21	1	0	22.33469328	114.13809380	https://www.inaturalist.org/observations/101690353
871	Lanius cristatus	Brown Shrike	2022/2/2	1	0	22.33469328	114.13809380	https://www.inaturalist.org/observations/106010187
872	Lanius cristatus	Brown Shrike	2020/3/15	2	0	22.33501058	114.13773910	https://www.inaturalist.org/observations/40063061
873	Lanius cristatus	Brown Shrike	2022/2/3	1	0	22.33501147	114.13737130	https://www.inaturalist.org/observations/123179640

874	Lanius cristatus	Brown Shrike	2011/11/4	3	0	22.33635907	80.60170874	https://www.inaturalist.org/observations/41553739
875	Lanius cristatus	Brown Shrike	2019/5/4	2	0	22.33726825	113.50081070	https://www.inaturalist.org/observations/67286809
876	Lanius cristatus	Brown Shrike	2021/5/5	2	0	22.33742871	114.13636610	https://www.inaturalist.org/observations/82284192
877	Lanius cristatus	Brown Shrike	2020/2/2	3	0	22.33795365	114.17453180	https://www.inaturalist.org/observations/38289556
878	Lanius cristatus	Brown Shrike	2021/1/5	2	0	22.33906596	114.17375210	https://www.inaturalist.org/observations/67730472
879	Lanius cristatus	Brown Shrike	2021/1/6	2	0	22.33907092	114.17375210	https://www.inaturalist.org/observations/67730530
880	Lanius cristatus	Brown Shrike	2022/3/9	1	0	22.34623333	120.36553330	https://www.inaturalist.org/observations/108213489
881	Lanius cristatus	Brown Shrike	2022/9/27	2	0	22.34652480	114.09830680	https://www.inaturalist.org/observations/136682604
882	Lanius cristatus	Brown Shrike	2020/11/1	3	0	22.34722947	80.44999849	https://www.inaturalist.org/observations/63946051
883	Lanius cristatus	Brown Shrike	2020/4/26	2	0	22.35256293	114.24333920	https://www.inaturalist.org/observations/44096630
884	Lanius cristatus	Brown Shrike	2020/3/25	2	0	22.35591033	114.10561320	https://www.inaturalist.org/observations/41012082
885	Lanius cristatus	Brown Shrike	2018/12/21	2	0	22.35609177	114.10585340	https://www.inaturalist.org/observations/68891460
886	Lanius cristatus	Brown Shrike	2021/1/14	2	0	22.35615969	114.10494270	https://www.inaturalist.org/observations/68136910
887	Lanius cristatus	Brown Shrike	2020/12/6	2	0	22.36420099	88.38824130	https://www.inaturalist.org/observations/111894239
888	Lanius cristatus	Brown Shrike	2021/5/7	1	0	22.37407044	114.26530080	https://www.inaturalist.org/observations/112031345
889	Lanius cristatus	Brown Shrike	2022/11/5	1	0	22.37409295	114.26505380	https://www.inaturalist.org/observations/141149055
890	Lanius cristatus	Brown Shrike	2022/5/2	1	0	22.37421637	114.26498320	https://www.inaturalist.org/observations/114635737
891	Lanius cristatus	Brown Shrike	2022/3/18	1	0	22.37487418	114.07809660	https://www.inaturalist.org/observations/108951183
892	Lanius cristatus	Brown Shrike	2022/1/19	2	0	22.37519033	114.26340600	https://www.inaturalist.org/observations/105217020
893	Lanius cristatus	Brown Shrike	2022/3/13	1	0	22.38111847	114.26518070	https://www.inaturalist.org/observations/108705689
894	Lanius cristatus	Brown Shrike	2022/12/24	0	0	22.38364390	114.27078670	https://www.inaturalist.org/observations/145246610
895	Lanius cristatus	Brown Shrike	2019/5/9	5	0	22.38417667	114.10797500	https://www.inaturalist.org/observations/24827793
896	Lanius cristatus	Brown Shrike	2022/1/24	2	0	22.38493080	114.27069740	https://www.inaturalist.org/observations/105478802

897	Lanius cristatus	Brown Shrike	2022/5/2	1	0	22.39042965	113.91989470	https://www.inaturalist.org/observations/114583958
898	Lanius cristatus	Brown Shrike	2004/4/4	5	0	22.39754498	114.20549170	https://www.inaturalist.org/observations/8555595
899	Lanius cristatus	Brown Shrike	2022/5/2	1	0	22.40052977	114.20761340	https://www.inaturalist.org/observations/114661491
900	Lanius cristatus	Brown Shrike	2022/12/2	1	0	22.40204164	114.09369400	https://www.inaturalist.org/observations/143486056
901	Lanius cristatus	Brown Shrike	2019/5/8	3	0	22.40246421	113.96160950	https://www.inaturalist.org/observations/25932606
902	Lanius cristatus	Brown Shrike	2021/3/13	2	0	22.40415284	114.22229760	https://www.inaturalist.org/observations/71121621
903	Lanius cristatus	Brown Shrike	2012/12/14	1	0	22.40934879	114.03158130	https://www.inaturalist.org/observations/92077917
904	Lanius cristatus	Brown Shrike	2021/5/4	1	0	22.41640046	114.20803630	https://www.inaturalist.org/observations/77238803
905	Lanius cristatus	Brown Shrike	2020/9/24	2	0	22.42574680	114.18120860	https://www.inaturalist.org/observations/63385039
906	Lanius cristatus	Brown Shrike	2021/10/29	1	0	22.42574680	114.18120860	https://www.inaturalist.org/observations/99708862
907	Lanius cristatus	Brown Shrike	2022/5/2	1	0	22.42574680	114.18120860	https://www.inaturalist.org/observations/114652786
908	Lanius cristatus lucionensis	Brown Shrike	2022/5/2	1	0	22.42574680	114.18120860	https://www.inaturalist.org/observations/114725273
909	Lanius cristatus	Brown Shrike	2020/9/12	2	0	22.42895906	114.23543980	https://www.inaturalist.org/observations/59453352
910	Lanius cristatus	Brown Shrike	2022/4/21	1	0	22.42918361	114.18250780	https://www.inaturalist.org/observations/112254993
911	Lanius cristatus	Brown Shrike	2018/12/14	3	0	22.42929167	114.18307170	https://www.inaturalist.org/observations/19010869
912	Lanius cristatus	Brown Shrike	2022/2/27	1	0	22.42958685	114.18250860	https://www.inaturalist.org/observations/107567249
913	Lanius cristatus	Brown Shrike	2022/4/24	1	0	22.42960201	114.18244250	https://www.inaturalist.org/observations/112636842
914	Lanius cristatus	Brown Shrike	2021/11/8	1	0	22.42993100	114.18316400	https://www.inaturalist.org/observations/100581170
915	Lanius cristatus	Brown Shrike	2021/10/2	1	0	22.42998419	114.18340620	https://www.inaturalist.org/observations/97017557
916	Lanius cristatus	Brown Shrike	2021/2/21	2	0	22.42998700	114.18233600	https://www.inaturalist.org/observations/69909683
917	Lanius cristatus	Brown Shrike	2018/12/22	3	0	22.43000800	114.18325500	https://www.inaturalist.org/observations/19211726
918	Lanius cristatus	Brown Shrike	2019/1/16	2	0	22.43009300	114.18321700	https://www.inaturalist.org/observations/19673932
919	Lanius cristatus	Brown Shrike	2022/3/15	1	0	22.43166800	114.18518100	https://www.inaturalist.org/observations/113722926

920	Lanius cristatus	Brown Shrike	2022/2/11	2	0	22.43179950	120.49248090	https://www.inaturalist.org/observations/106549428
921	Lanius cristatus	Brown Shrike	2019/11/20	2	0	22.43450028	120.49701030	https://www.inaturalist.org/observations/36124311
922	Lanius cristatus lucionensis	Brown Shrike	2022/10/9	1	0	22.43705450	114.18284290	https://www.inaturalist.org/observations/138235244
923	Lanius cristatus	Brown Shrike	2021/12/29	1	0	22.43741208	120.58041520	https://www.inaturalist.org/observations/104087173
924	Lanius cristatus	Brown Shrike	2021/12/13	1	0	22.43904803	120.58053270	https://www.inaturalist.org/observations/102980319
925	Lanius cristatus	Brown Shrike	2021/1/11	2	0	22.44232341	114.01969870	https://www.inaturalist.org/observations/67957848
926	Lanius cristatus	Brown Shrike	2020/12/6	3	0	22.44320958	88.30743541	https://www.inaturalist.org/observations/66251099
927	Lanius cristatus	Brown Shrike	2021/12/31	2	0	22.44335013	88.30729578	https://www.inaturalist.org/observations/104097484
928	Lanius cristatus	Brown Shrike	2022/4/20	2	0	22.44378100	120.49099200	https://www.inaturalist.org/observations/112147945
929	Lanius cristatus	Brown Shrike	2020/11/4	2	0	22.44378521	120.49099200	https://www.inaturalist.org/observations/64236003
930	Lanius cristatus	Brown Shrike	2020/4/25	3	0	22.44509900	114.13858700	https://www.inaturalist.org/observations/44734008
931	Lanius cristatus	Brown Shrike	2019/11/19	3	0	22.44553167	120.49166780	https://www.inaturalist.org/observations/36124667
932	Lanius cristatus lucionensis	Brown Shrike	2019/4/28	2	0	22.44701596	114.14113170	https://www.inaturalist.org/observations/24244324
933	Lanius cristatus lucionensis	Brown Shrike	2020/4/24	3	0	22.45060452	114.13615270	https://www.inaturalist.org/observations/43019924
934	Lanius cristatus	Brown Shrike	2022/11/20	2	0	22.45389760	114.15257450	https://www.inaturalist.org/observations/142736876
935	Lanius cristatus	Brown Shrike	2020/4/25	3	0	22.45816667	114.00898640	https://www.inaturalist.org/observations/43284914
936	Lanius cristatus	Brown Shrike	2018/11/22	3	0	22.46713560	114.17949120	https://www.inaturalist.org/observations/18554191
937	Lanius cristatus	Brown Shrike	2009/4/26	1	0	22.46929600	114.00932400	https://www.inaturalist.org/observations/143495354
938	Lanius cristatus	Brown Shrike	2021/10/24	1	0	22.46998523	88.32822383	https://www.inaturalist.org/observations/99363079
939	Lanius cristatus	Brown Shrike	2021/10/24	1	0	22.47078556	88.33058406	https://www.inaturalist.org/observations/99363092
940	Lanius cristatus	Brown Shrike	2021/11/22	1	0	22.47097000	114.04270500	https://www.inaturalist.org/observations/101699114
941	Lanius cristatus	Brown Shrike	2021/11/21	1	0	22.47097000	114.04270500	https://www.inaturalist.org/observations/101750866
942	Lanius cristatus	Brown Shrike	2022/12/26	3	0	22.47097000	114.04270500	https://www.inaturalist.org/observations/145099347

943	Lanius cristatus	Brown Shrike	2021/1/26	2	0	22.47218619	88.32627118	https://www.inaturalist.org/observations/68690032
944	Lanius cristatus	Brown Shrike	2021/5/6	2	0	22.47219321	114.00441510	https://www.inaturalist.org/observations/78199307
945	Lanius cristatus	Brown Shrike	2022/2/26	1	0	22.47338797	114.04183300	https://www.inaturalist.org/observations/107463578
946	Lanius cristatus	Brown Shrike	2020/10/4	2	0	22.47383728	88.31301446	https://www.inaturalist.org/observations/61791327
947	Lanius cristatus	Brown Shrike	2021/12/26	1	0	22.47512554	114.03990040	https://www.inaturalist.org/observations/103689693
948	Lanius cristatus	Brown Shrike	2021/2/7	3	0	22.47608974	114.04194250	https://www.inaturalist.org/observations/69187657
949	Lanius cristatus	Brown Shrike	2020/4/26	2	0	22.49386725	114.04037150	https://www.inaturalist.org/observations/44143216
950	Lanius cristatus	Brown Shrike	2021/5/1	1	0	22.49386725	114.04037150	https://www.inaturalist.org/observations/76853087
951	Lanius cristatus	Brown Shrike	2022/5/2	1	0	22.49386725	114.04037150	https://www.inaturalist.org/observations/116033909
952	Lanius cristatus	Brown Shrike	2018/12/11	5	0	22.49488833	114.10484330	https://www.inaturalist.org/observations/18957142
953	Lanius cristatus	Brown Shrike	2022/1/1	1	0	22.49506125	114.03393420	https://www.inaturalist.org/observations/104621201
954	Lanius cristatus	Brown Shrike	2021/1/30	2	0	22.49545556	114.10690000	https://www.inaturalist.org/observations/69756329
955	Lanius cristatus	Brown Shrike	2020/4/25	2	0	22.49553264	114.05427610	https://www.inaturalist.org/observations/44098245
956	Lanius cristatus	Brown Shrike	2021/12/16	2	0	22.49688080	114.03661840	https://www.inaturalist.org/observations/103183352
957	Lanius cristatus	Brown Shrike	2021/5/4	2	0	22.49830384	114.05344960	https://www.inaturalist.org/observations/116888084
958	Lanius cristatus	Brown Shrike	2021/11/16	1	0	22.49876414	114.04582750	https://www.inaturalist.org/observations/102230930
959	Lanius cristatus	Brown Shrike	2021/11/16	2	0	22.49876414	114.04582750	https://www.inaturalist.org/observations/102234090
960	Lanius cristatus	Brown Shrike	2005/3/5	2	0	22.50438700	114.11105600	https://www.inaturalist.org/observations/66960342
961	Lanius cristatus	Brown Shrike	2020/10/6	2	0	22.50489000	120.39480640	https://www.inaturalist.org/observations/61844202
962	Lanius cristatus	Brown Shrike	2021/10/22	1	0	22.50506576	114.11262200	https://www.inaturalist.org/observations/99107940
963	Lanius cristatus	Brown Shrike	2020/11/15	2	0	22.50532578	114.11082860	https://www.inaturalist.org/observations/64912092
964	Lanius cristatus	Brown Shrike	2021/4/30	1	0	22.50642304	114.11228900	https://www.inaturalist.org/observations/76011917
965	Lanius cristatus	Brown Shrike	2020/9/11	2	0	22.50686333	114.11221950	https://www.inaturalist.org/observations/59215519

966	Lanius cristatus	Brown Shrike	2020/11/2	2	0	22.50714500	114.11282830	https://www.inaturalist.org/observations/64033467
967	Lanius cristatus	Brown Shrike	2021/11/6	1	0	22.50760510	114.11318550	https://www.inaturalist.org/observations/100462159
968	Lanius cristatus	Brown Shrike	2020/12/4	1	0	22.50775354	114.11348400	https://www.inaturalist.org/observations/70599570
969	Lanius cristatus	Brown Shrike	2021/5/2	1	0	22.50780025	114.11199000	https://www.inaturalist.org/observations/76578953
970	Lanius cristatus	Brown Shrike	2020/11/1	2	0	22.50855500	114.11442830	https://www.inaturalist.org/observations/64032988
971	Lanius cristatus	Brown Shrike	2013/10/6	1	0	22.50879520	114.11297710	https://www.inaturalist.org/observations/78955605
972	Lanius cristatus	Brown Shrike	2020/12/2	2	0	22.50879930	114.11297690	https://www.inaturalist.org/observations/119038227
973	Lanius cristatus	Brown Shrike	2021/5/2	1	0	22.50906939	114.11361460	https://www.inaturalist.org/observations/76561767
974	Lanius cristatus	Brown Shrike	2020/11/18	2	0	22.51000467	114.11307900	https://www.inaturalist.org/observations/65183021
975	Lanius cristatus	Brown Shrike	2011/12/17	1	0	22.51115400	114.11452200	https://www.inaturalist.org/observations/95001557
976	Lanius cristatus	Brown Shrike	2020/9/24	1	0	22.51130336	120.36314870	https://www.inaturalist.org/observations/62109419
977	Lanius cristatus	Brown Shrike	2020/12/5	2	0	22.51261575	120.52806590	https://www.inaturalist.org/observations/66691481
978	Lanius cristatus	Brown Shrike	2020/3/8	2	0	22.51547718	114.24062830	https://www.inaturalist.org/observations/39688425
979	Lanius cristatus	Brown Shrike	2019/12/27	2	0	22.51564923	120.50517080	https://www.inaturalist.org/observations/36991502
980	Lanius cristatus	Brown Shrike	2022/3/17	1	0	22.51793447	120.46266500	https://www.inaturalist.org/observations/108768112
981	Lanius cristatus	Brown Shrike	2022/12/24	0	0	22.52623442	114.25953680	https://www.inaturalist.org/observations/145104355
982	Lanius cristatus	Brown Shrike	2020/1/16	2	0	22.53787538	120.58861470	https://www.inaturalist.org/observations/37653488
983	Lanius cristatus	Brown Shrike	2019/12/1	2	0	22.53866866	120.59045980	https://www.inaturalist.org/observations/45755794
984	Lanius cristatus	Brown Shrike	2017/11/17	2	0	22.53912970	120.39038550	https://www.inaturalist.org/observations/9457905
985	Lanius cristatus	Brown Shrike	2022/5/25	1	0	22.54309900	114.05786800	https://www.inaturalist.org/observations/118763116
986	Lanius cristatus	Brown Shrike	2013/2/11	1	0	22.54490349	88.34243774	https://www.inaturalist.org/observations/79812092
987	Lanius cristatus	Brown Shrike	2021/11/3	1	0	22.54682733	114.01722840	https://www.inaturalist.org/observations/100695542
988	Lanius cristatus	Brown Shrike	2017/11/17	2	0	22.55029385	120.39196010	https://www.inaturalist.org/observations/9457906

989	Lanius cristatus	Brown Shrike	2021/5/3	1	0	22.55143172	114.07936180	https://www.inaturalist.org/observations/77240811
990	Lanius cristatus	Brown Shrike	2022/9/20	2	0	22.55379126	120.31480380	https://www.inaturalist.org/observations/135775321
991	Lanius cristatus	Brown Shrike	2022/4/7	3	0	22.55385964	120.31508270	https://www.inaturalist.org/observations/110648910
992	Lanius cristatus	Brown Shrike	2020/11/27	2	0	22.55390918	120.31518590	https://www.inaturalist.org/observations/65748374
993	Lanius cristatus	Brown Shrike	2021/11/2	2	0	22.55390930	120.31518650	https://www.inaturalist.org/observations/100137904
994	Lanius cristatus	Brown Shrike	2022/12/16	1	0	22.55391673	120.31348330	https://www.inaturalist.org/observations/144470002
995	Lanius cristatus	Brown Shrike	2022/9/27	2	0	22.55415331	120.31446090	https://www.inaturalist.org/observations/136671946
996	Lanius cristatus	Brown Shrike	2022/2/17	3	0	22.55455028	120.39002030	https://www.inaturalist.org/observations/106908020
997	Lanius cristatus	Brown Shrike	2020/5/9	2	0	22.55513000	120.38903040	https://www.inaturalist.org/observations/45352943
998	Lanius cristatus	Brown Shrike	2016/9/24	0	0	22.55638056	120.38413330	https://www.inaturalist.org/observations/52771908
999	Lanius cristatus lucionensis	Brown Shrike	2018/12/15	1	0	22.55766400	120.38977400	https://www.inaturalist.org/observations/83732194
1000	Lanius cristatus	Brown Shrike	2021/2/20	2	0	22.55781439	120.44822560	https://www.inaturalist.org/observations/69917684
1001	Lanius cristatus	Brown Shrike	2021/3/11	2	0	22.56364634	120.53646050	https://www.inaturalist.org/observations/70994431
1002	Lanius cristatus	Brown Shrike	2022/12/2	1	0	22.56392860	113.97499080	https://www.inaturalist.org/observations/144148499
1003	Lanius cristatus	Brown Shrike	2020/10/20	2	0	22.56392860	120.53766680	https://www.inaturalist.org/observations/63170094
1004	Lanius cristatus	Brown Shrike	2022/4/2	2	0	22.56453479	120.36053070	https://www.inaturalist.org/observations/110162709
1005	Lanius cristatus	Brown Shrike	2019/1/8	2	0	22.57035833	120.34213170	https://www.inaturalist.org/observations/19606562
1006	Lanius cristatus	Brown Shrike	2020/10/27	2	0	22.57321417	120.33442420	https://www.inaturalist.org/observations/63963929
1007	Lanius cristatus	Brown Shrike	2019/10/15	1	0	22.57557411	88.47979032	https://www.inaturalist.org/observations/95556063
1008	Lanius cristatus	Brown Shrike	2020/10/27	2	0	22.57826630	120.34973240	https://www.inaturalist.org/observations/63963390
1009	Lanius cristatus	Brown Shrike	2020/10/27	2	0	22.57941940	120.35048730	https://www.inaturalist.org/observations/63963374
1010	Lanius cristatus	Brown Shrike	2020/10/26	2	0	22.57951417	120.35058830	https://www.inaturalist.org/observations/63950481
1011	Lanius cristatus	Brown Shrike	2020/2/16	2	0	22.58132701	88.28413021	https://www.inaturalist.org/observations/65212495

1012	Lanius cristatus	Brown Shrike	2020/10/26	2	0	22.58151556	120.34172830	https://www.inaturalist.org/observations/63950266
1013	Lanius cristatus	Brown Shrike	2020/10/26	2	0	22.58160833	120.34248560	https://www.inaturalist.org/observations/63950238
1014	Lanius cristatus	Brown Shrike	2020/10/26	2	0	22.58162306	120.34200970	https://www.inaturalist.org/observations/63950251
1015	Lanius cristatus	Brown Shrike	2020/10/26	2	0	22.58162444	120.34247530	https://www.inaturalist.org/observations/63950244
1016	Lanius cristatus	Brown Shrike	2021/2/17	1	0	22.58315550	88.47774721	https://www.inaturalist.org/observations/81277427
1017	Lanius cristatus	Brown Shrike	2021/4/13	2	0	22.58367882	120.57932920	https://www.inaturalist.org/observations/73930734
1018	Lanius cristatus	Brown Shrike	2021/2/1	2	0	22.58452150	88.32408650	https://www.inaturalist.org/observations/69797225
1019	Lanius cristatus	Brown Shrike	2022/4/19	1	0	22.58676000	88.41494000	https://www.inaturalist.org/observations/112182923
1020	Lanius cristatus	Brown Shrike	2021/3/9	3	0	22.58718339	120.35240940	https://www.inaturalist.org/observations/70936188
1021	Lanius cristatus	Brown Shrike	2022/1/1	1	0	22.58898684	120.62091540	https://www.inaturalist.org/observations/144122834
1022	Lanius cristatus	Brown Shrike	2022/10/26	1	0	22.59233254	120.35343630	https://www.inaturalist.org/observations/140032984
1023	Lanius cristatus	Brown Shrike	2018/12/31	3	0	22.59449726	120.35721220	https://www.inaturalist.org/observations/19333342
1024	Lanius cristatus	Brown Shrike	2022/12/20	1	0	22.59488589	120.35655120	https://www.inaturalist.org/observations/144711614
1025	Lanius cristatus	Brown Shrike	2021/3/9	2	0	22.59488990	120.35656140	https://www.inaturalist.org/observations/70936192
1026	Lanius cristatus	Brown Shrike	2021/11/27	2	0	22.59491471	120.35655500	https://www.inaturalist.org/observations/102089624
1027	Lanius cristatus	Brown Shrike	2022/11/14	3	0	22.59562073	120.35572850	https://www.inaturalist.org/observations/142114377
1028	Lanius cristatus	Brown Shrike	2022/3/4	1	0	22.59671978	120.30936180	https://www.inaturalist.org/observations/109640273
1029	Lanius cristatus	Brown Shrike	2020/11/27	2	0	22.59941166	120.27570250	https://www.inaturalist.org/observations/65748381
1030	Lanius cristatus	Brown Shrike	2021/12/24	1	0	22.60033375	88.47467434	https://www.inaturalist.org/observations/103572985
1031	Lanius cristatus	Brown Shrike	2022/4/10	2	0	22.60033530	88.47467434	https://www.inaturalist.org/observations/111169949
1032	Lanius cristatus	Brown Shrike	2021/11/27	2	0	22.60250194	120.35471250	https://www.inaturalist.org/observations/102089613
1033	Lanius cristatus	Brown Shrike	2021/2/21	0	0	22.60353600	70.20232420	https://www.inaturalist.org/observations/137943195
1034	Lanius cristatus	Brown Shrike	2019/12/24	3	0	22.60395035	88.47700252	https://www.inaturalist.org/observations/60430169

1035	Lanius cristatus	Brown Shrike	2020/2/7	2	0	22.60406095	120.27223570	https://www.inaturalist.org/observations/38386678
1036	Lanius cristatus	Brown Shrike	2021/11/27	2	0	22.60540700	120.35488150	https://www.inaturalist.org/observations/102089629
1037	Lanius cristatus	Brown Shrike	2017/3/4	2	0	22.60716146	120.30790640	https://www.inaturalist.org/observations/64109053
1038	Lanius cristatus	Brown Shrike	2021/12/28	2	0	22.60727602	120.30930520	https://www.inaturalist.org/observations/104404611
1039	Lanius cristatus	Brown Shrike	2019/2/18	2	0	22.60733911	120.30799720	https://www.inaturalist.org/observations/20448969
1040	Lanius cristatus	Brown Shrike	2022/12/21	2	0	22.60946000	88.46433667	https://www.inaturalist.org/observations/144925464
1041	Lanius cristatus	Brown Shrike	2021/12/28	2	0	22.61119812	120.35509590	https://www.inaturalist.org/observations/104404609
1042	Lanius cristatus	Brown Shrike	2021/3/8	3	0	22.61193320	120.35799060	https://www.inaturalist.org/observations/70936164
1043	Lanius cristatus	Brown Shrike	2022/2/27	1	0	22.61232974	120.35869800	https://www.inaturalist.org/observations/107538277
1044	Lanius cristatus	Brown Shrike	2022/11/1	1	0	22.61420148	88.46456623	https://www.inaturalist.org/observations/143942954
1045	Lanius cristatus	Brown Shrike	2020/2/7	2	0	22.61444406	120.26419230	https://www.inaturalist.org/observations/38386676
1046	Lanius cristatus	Brown Shrike	2018/9/26	2	0	22.61474320	120.37640970	https://www.inaturalist.org/observations/16927318
1047	Lanius cristatus	Brown Shrike	2022/12/18	1	0	22.61480302	120.26618620	https://www.inaturalist.org/observations/144597025
1048	Lanius cristatus	Brown Shrike	2021/11/2	1	0	22.61491199	120.26575390	https://www.inaturalist.org/observations/100137909
1049	Lanius cristatus	Brown Shrike	2020/9/16	2	0	22.61542187	120.32011680	https://www.inaturalist.org/observations/59763624
1050	Lanius cristatus	Brown Shrike	2021/1/13	2	0	22.61616269	120.37697450	https://www.inaturalist.org/observations/68054835
1051	Lanius cristatus	Brown Shrike	2017/6/30	1	0	22.61782882	120.33860490	https://www.inaturalist.org/observations/83144477
1052	Lanius cristatus	Brown Shrike	2020/4/4	2	0	22.61834583	120.37503360	https://www.inaturalist.org/observations/42094005
1053	Lanius cristatus	Brown Shrike	2022/1/11	1	0	22.61845333	120.33985830	https://www.inaturalist.org/observations/104745790
1054	Lanius cristatus	Brown Shrike	2021/2/16	1	0	22.61948061	88.44338325	https://www.inaturalist.org/observations/81268697
1055	Lanius cristatus	Brown Shrike	2018/2/20	2	0	22.61976820	120.33955290	https://www.inaturalist.org/observations/9930985
1056	Lanius cristatus	Brown Shrike	2018/4/29	2	0	22.61976820	120.33955290	https://www.inaturalist.org/observations/11724822
1057	Lanius cristatus	Brown Shrike	2018/11/19	2	0	22.61976820	120.33955290	https://www.inaturalist.org/observations/18494658

1058	Lanius cristatus	Brown Shrike	2018/11/30	3	0	22.61976820	120.33955290	https://www.inaturalist.org/observations/18732851
1059	Lanius cristatus	Brown Shrike	2022/11/1	1	0	22.61976820	120.33955290	https://www.inaturalist.org/observations/140736556
1060	Lanius cristatus	Brown Shrike	2020/2/6	3	0	22.61976991	120.33954980	https://www.inaturalist.org/observations/38354760
1061	Lanius cristatus	Brown Shrike	2019/12/8	2	0	22.61977486	120.33954980	https://www.inaturalist.org/observations/36460190
1062	Lanius cristatus	Brown Shrike	2019/12/8	2	0	22.61977486	120.33954980	https://www.inaturalist.org/observations/36460192
1063	Lanius cristatus	Brown Shrike	2019/12/16	2	0	22.61977486	120.33954980	https://www.inaturalist.org/observations/36698206
1064	Lanius cristatus	Brown Shrike	2020/4/9	2	0	22.61977486	120.33954980	https://www.inaturalist.org/observations/41758283
1065	Lanius cristatus	Brown Shrike	2017/9/14	2	0	22.61979467	120.33951760	https://www.inaturalist.org/observations/20022124
1066	Lanius cristatus	Brown Shrike	2021/1/3	2	0	22.61979467	120.33951760	https://www.inaturalist.org/observations/67570225
1067	Lanius cristatus	Brown Shrike	2021/3/8	3	0	22.61979467	120.33951760	https://www.inaturalist.org/observations/70936182
1068	Lanius cristatus	Brown Shrike	2020/1/5	2	0	22.61990358	120.34106370	https://www.inaturalist.org/observations/37295810
1069	Lanius cristatus	Brown Shrike	2018/1/22	2	0	22.62030965	120.34013990	https://www.inaturalist.org/observations/9561003
1070	Lanius cristatus	Brown Shrike	2018/1/11	1	0	22.62032216	120.33944320	https://www.inaturalist.org/observations/123674615
1071	Lanius cristatus	Brown Shrike	2017/6/30	1	0	22.62071761	120.34117310	https://www.inaturalist.org/observations/83144442
1072	Lanius cristatus	Brown Shrike	2020/2/17	2	0	22.62073910	120.36535730	https://www.inaturalist.org/observations/42911217
1073	Lanius cristatus	Brown Shrike	2018/1/11	1	0	22.62131309	120.34087000	https://www.inaturalist.org/observations/123674628
1074	Lanius cristatus	Brown Shrike	2018/1/31	1	0	22.62172494	120.34056070	https://www.inaturalist.org/observations/123676018
1075	Lanius cristatus lucionensis	Brown Shrike	2022/5/14	0	0	22.62174840	120.26858680	https://www.inaturalist.org/observations/116932064
1076	Lanius cristatus	Brown Shrike	2019/3/21	2	0	22.62202039	120.34014500	https://www.inaturalist.org/observations/21450492
1077	Lanius cristatus	Brown Shrike	2022/9/6	1	0	22.62215400	120.26905610	https://www.inaturalist.org/observations/133880270
1078	Lanius cristatus	Brown Shrike	2019/9/7	2	0	22.62221092	120.26916380	https://www.inaturalist.org/observations/32245936
1079	Lanius cristatus	Brown Shrike	2019/12/1	1	0	22.62223570	120.26791890	https://www.inaturalist.org/observations/117115080
1080	Lanius cristatus	Brown Shrike	2019/5/23	2	0	22.62232840	120.26909500	https://www.inaturalist.org/observations/30606532

1081	Lanius cristatus	Brown Shrike	2017/6/30	1	0	22.62235631	120.33888280	https://www.inaturalist.org/observations/83144462
1082	Lanius cristatus	Brown Shrike	2022/10/17	3	0	22.62241396	120.26877720	https://www.inaturalist.org/observations/139069042
1083	Lanius cristatus	Brown Shrike	2017/6/30	1	0	22.62242749	120.33910330	https://www.inaturalist.org/observations/83144464
1084	Lanius cristatus lucionensis	Brown Shrike	2022/3/18	0	0	22.62253125	120.33931680	https://www.inaturalist.org/observations/109148517
1085	Lanius cristatus	Brown Shrike	2022/9/13	2	0	22.62261203	120.26800470	https://www.inaturalist.org/observations/134851445
1086	Lanius cristatus	Brown Shrike	2022/2/11	2	0	22.62301323	120.38608760	https://www.inaturalist.org/observations/106549440
1087	Lanius cristatus	Brown Shrike	2020/11/1	2	0	22.62305790	120.38643660	https://www.inaturalist.org/observations/64032868
1088	Lanius cristatus	Brown Shrike	2022/2/14	3	0	22.62310937	120.36383190	https://www.inaturalist.org/observations/106733996
1089	Lanius cristatus lucionensis	Brown Shrike	2022/1/21	0	0	22.62344727	120.34377790	https://www.inaturalist.org/observations/105643509
1090	Lanius cristatus lucionensis	Brown Shrike	2022/1/21	0	0	22.62344727	120.34377790	https://www.inaturalist.org/observations/105643514
1091	Lanius cristatus	Brown Shrike	2020/10/10	3	0	22.62362295	88.44098009	https://www.inaturalist.org/observations/63711706
1092	Lanius cristatus	Brown Shrike	2019/4/20	2	0	22.62362870	88.44098000	https://www.inaturalist.org/observations/78030477
1093	Lanius cristatus	Brown Shrike	2019/12/29	4	0	22.62362883	88.44098009	https://www.inaturalist.org/observations/37304115
1094	Lanius cristatus	Brown Shrike	2019/11/1	1	0	22.62375231	88.44098009	https://www.inaturalist.org/observations/95556849
1095	Lanius cristatus	Brown Shrike	2022/9/15	1	0	22.62495910	120.26817640	https://www.inaturalist.org/observations/135117070
1096	Lanius cristatus	Brown Shrike	2021/2/16	1	0	22.62581875	88.43857673	https://www.inaturalist.org/observations/81268696
1097	Lanius cristatus	Brown Shrike	2020/10/3	3	0	22.62703180	120.28757460	https://www.inaturalist.org/observations/63562822
1098	Lanius cristatus	Brown Shrike	2021/12/27	2	0	22.62939441	120.34314660	https://www.inaturalist.org/observations/103739187
1099	Lanius cristatus	Brown Shrike	2022/2/15	2	0	22.62954400	120.26854800	https://www.inaturalist.org/observations/106789940
1100	Lanius cristatus	Brown Shrike	2022/2/14	3	0	22.62977640	120.36520520	https://www.inaturalist.org/observations/106733981
1101	Lanius cristatus	Brown Shrike	2017/1/24	2	0	22.62994585	120.28601450	https://www.inaturalist.org/observations/39050415
1102	Lanius cristatus	Brown Shrike	2020/9/3	1	0	22.63444466	120.60977740	https://www.inaturalist.org/observations/58402474
1103	Lanius cristatus	Brown Shrike	2020/3/3	2	0	22.63450630	120.27515420	https://www.inaturalist.org/observations/39566667

1104	Lanius cristatus	Brown Shrike	2020/2/10	2	0	22.63703597	120.28452490	https://www.inaturalist.org/observations/38536968
1105	Lanius cristatus	Brown Shrike	2022/2/7	1	0	22.63766743	114.40215590	https://www.inaturalist.org/observations/107693506
1106	Lanius cristatus	Brown Shrike	2022/4/21	1	0	22.63819326	114.40289650	https://www.inaturalist.org/observations/124994641
1107	Lanius cristatus	Brown Shrike	2019/12/27	2	0	22.63862020	120.61549010	https://www.inaturalist.org/observations/36991496
1108	Lanius cristatus	Brown Shrike	2018/1/3	2	0	22.63892354	120.29651060	https://www.inaturalist.org/observations/15778023
1109	Lanius cristatus	Brown Shrike	2021/4/1	1	0	22.63894220	120.60783370	https://www.inaturalist.org/observations/72600661
1110	Lanius cristatus	Brown Shrike	2022/1/21	1	0	22.63917261	114.39554500	https://www.inaturalist.org/observations/107372069
1111	Lanius cristatus	Brown Shrike	2020/4/27	1	0	22.63922755	114.35685090	https://www.inaturalist.org/observations/139437184
1112	Lanius cristatus	Brown Shrike	2020/2/28	1	0	22.64095961	120.27614400	https://www.inaturalist.org/observations/80227696
1113	Lanius cristatus	Brown Shrike	2019/12/2	2	0	22.64203479	120.59382090	https://www.inaturalist.org/observations/36287682
1114	Lanius cristatus	Brown Shrike	2020/10/10	2	0	22.64432171	120.60662300	https://www.inaturalist.org/observations/62372230
1115	Lanius cristatus	Brown Shrike	2018/2/6	1	0	22.64444400	120.60976400	https://www.inaturalist.org/observations/14414949
1116	Lanius cristatus	Brown Shrike	2018/2/6	2	0	22.64444400	120.60976400	https://www.inaturalist.org/observations/14414950
1117	Lanius cristatus	Brown Shrike	2017/10/7	2	0	22.64444400	120.60976400	https://www.inaturalist.org/observations/20004839
1118	Lanius cristatus	Brown Shrike	2022/12/15	1	0	22.64463916	120.27425540	https://www.inaturalist.org/observations/144414235
1119	Lanius cristatus	Brown Shrike	2022/12/13	2	0	22.64505215	120.28630520	https://www.inaturalist.org/observations/144294022
1120	Lanius cristatus	Brown Shrike	2020/9/24	2	0	22.64585666	120.60625340	https://www.inaturalist.org/observations/60686381
1121	Lanius cristatus	Brown Shrike	2018/3/29	2	0	22.64594400	120.28798600	https://www.inaturalist.org/observations/10505912
1122	Lanius cristatus	Brown Shrike	2019/12/21	3	0	22.64601361	120.27414450	https://www.inaturalist.org/observations/37062405
1123	Lanius cristatus	Brown Shrike	2021/3/9	2	0	22.64648074	120.61249480	https://www.inaturalist.org/observations/70844052
1124	Lanius cristatus	Brown Shrike	2022/11/19	1	0	22.64701355	120.35442880	https://www.inaturalist.org/observations/142343361
1125	Lanius cristatus	Brown Shrike	2022/11/11	2	0	22.64882871	120.61415580	https://www.inaturalist.org/observations/141648651
1126	Lanius cristatus	Brown Shrike	2020/11/18	2	0	22.64912100	120.29541500	https://www.inaturalist.org/observations/65137993

1127	Lanius cristatus	Brown Shrike	2021/11/25	1	0	22.65029112	120.60487550	https://www.inaturalist.org/observations/102296147
1128	Lanius cristatus	Brown Shrike	2019/12/10	2	0	22.65082590	120.60829260	https://www.inaturalist.org/observations/36587689
1129	Lanius cristatus	Brown Shrike	2011/12/30	3	0	22.65092100	120.25066100	https://www.inaturalist.org/observations/14643218
1130	Lanius cristatus	Brown Shrike	2020/2/9	2	0	22.65219392	120.27108510	https://www.inaturalist.org/observations/38536291
1131	Lanius cristatus	Brown Shrike	2020/9/18	1	0	22.65272231	120.35001800	https://www.inaturalist.org/observations/60507424
1132	Lanius cristatus	Brown Shrike	2020/4/28	2	0	22.65363333	120.27965500	https://www.inaturalist.org/observations/44253411
1133	Lanius cristatus	Brown Shrike	2021/10/27	1	0	22.65683727	120.28464240	https://www.inaturalist.org/observations/99543841
1134	Lanius cristatus	Brown Shrike	2022/11/14	1	0	22.65720275	120.28518280	https://www.inaturalist.org/observations/141990586
1135	Lanius cristatus	Brown Shrike	2022/11/10	1	0	22.65765382	120.42643700	https://www.inaturalist.org/observations/141606515
1136	Lanius cristatus	Brown Shrike	2022/3/14	1	0	22.65823611	120.29880280	https://www.inaturalist.org/observations/108569726
1137	Lanius cristatus	Brown Shrike	2022/1/1	1	0	22.65828339	120.28534030	https://www.inaturalist.org/observations/144124187
1138	Lanius cristatus	Brown Shrike	2022/1/1	1	0	22.65828339	120.28534030	https://www.inaturalist.org/observations/144124194
1139	Lanius cristatus	Brown Shrike	2022/1/1	1	0	22.65828339	120.28534030	https://www.inaturalist.org/observations/144124197
1140	Lanius cristatus	Brown Shrike	2022/1/1	1	0	22.65828339	120.28534030	https://www.inaturalist.org/observations/144124206
1141	Lanius cristatus	Brown Shrike	2022/1/1	1	0	22.65828339	120.28534030	https://www.inaturalist.org/observations/144124210
1142	Lanius cristatus	Brown Shrike	2021/12/31	2	0	22.65834500	120.28489830	https://www.inaturalist.org/observations/105646974
1143	Lanius cristatus	Brown Shrike	2022/2/4	0	0	22.65837874	120.31186820	https://www.inaturalist.org/observations/106110791
1144	Lanius cristatus	Brown Shrike	2022/3/14	1	0	22.65873333	120.29761110	https://www.inaturalist.org/observations/108569721
1145	Lanius cristatus	Brown Shrike	2022/3/14	1	0	22.65894444	120.29761670	https://www.inaturalist.org/observations/108569733
1146	Lanius cristatus	Brown Shrike	2022/11/23	1	0	22.65897668	120.29835380	https://www.inaturalist.org/observations/142652956
1147	Lanius cristatus	Brown Shrike	2022/3/14	2	0	22.65902500	120.30188610	https://www.inaturalist.org/observations/108569714
1148	Lanius cristatus	Brown Shrike	2019/2/13	2	0	22.65923808	120.28594170	https://www.inaturalist.org/observations/20320764
1149	Lanius cristatus	Brown Shrike	2022/4/12	2	0	22.65969948	120.28547590	https://www.inaturalist.org/observations/111178043

1150	Lanius cristatus	Brown Shrike	2020/2/9	1	0	22.65992700	120.57122800	https://www.inaturalist.org/observations/81452021
1151	Lanius cristatus	Brown Shrike	2022/4/5	1	0	22.66125400	120.43929900	https://www.inaturalist.org/observations/135082351
1152	Lanius cristatus	Brown Shrike	2016/10/31	2	0	22.66344125	120.63112530	https://www.inaturalist.org/observations/44365942
1153	Lanius cristatus	Brown Shrike	2021/1/10	1	0	22.66385267	120.44147030	https://www.inaturalist.org/observations/81264554
1154	Lanius cristatus	Brown Shrike	2021/1/16	2	0	22.66453386	120.38788550	https://www.inaturalist.org/observations/68152936
1155	Lanius cristatus	Brown Shrike	2021/3/7	3	0	22.67130000	120.30647170	https://www.inaturalist.org/observations/70734464
1156	Lanius cristatus	Brown Shrike	2022/3/16	2	0	22.67703143	120.50357160	https://www.inaturalist.org/observations/108801383
1157	Lanius cristatus	Brown Shrike	2022/3/16	2	0	22.67708464	120.50360010	https://www.inaturalist.org/observations/108801235
1158	Lanius cristatus	Brown Shrike	2021/1/14	2	0	22.67901225	120.30052970	https://www.inaturalist.org/observations/68040417
1159	Lanius cristatus	Brown Shrike	2022/4/30	1	0	22.67930000	120.32225830	https://www.inaturalist.org/observations/113698210
1160	Lanius cristatus	Brown Shrike	2021/11/3	3	0	22.67949777	120.30065340	https://www.inaturalist.org/observations/106847217
1161	Lanius cristatus	Brown Shrike	2020/1/18	2	0	22.68050670	120.29843890	https://www.inaturalist.org/observations/37703960
1162	Lanius cristatus lucionensis	Brown Shrike	2020/2/27	2	0	22.68052430	120.29832630	https://www.inaturalist.org/observations/39524351
1163	Lanius cristatus	Brown Shrike	2020/1/18	2	0	22.68055400	120.29821900	https://www.inaturalist.org/observations/37780919
1164	Lanius cristatus superciliosus	Brown Shrike	2015/5/4	1	0	22.68107330	88.31542690	https://www.inaturalist.org/observations/100378053
1165	Lanius cristatus	Brown Shrike	2021/2/27	2	0	22.68310277	120.32002660	https://www.inaturalist.org/observations/70436305
1166	Lanius cristatus	Brown Shrike	2020/12/20	3	0	22.68364621	88.32208366	https://www.inaturalist.org/observations/67046466
1167	Lanius cristatus	Brown Shrike	2022/2/20	1	0	22.68435500	120.32183000	https://www.inaturalist.org/observations/107096015
1168	Lanius cristatus	Brown Shrike	2021/1/3	2	0	22.68583250	120.29616530	https://www.inaturalist.org/observations/67570230
1169	Lanius cristatus	Brown Shrike	2021/3/20	2	0	22.69666565	121.06492670	https://www.inaturalist.org/observations/71625354
1170	Lanius cristatus	Brown Shrike	2021/3/11	3	0	22.69803910	120.64076210	https://www.inaturalist.org/observations/70994427
1171	Lanius cristatus	Brown Shrike	2022/4/28	2	0	22.69848667	120.31007170	https://www.inaturalist.org/observations/113211708
1172	Lanius cristatus	Brown Shrike	2022/9/17	2	0	22.69848833	120.31004500	https://www.inaturalist.org/observations/135342456

1173	Lanius cristatus	Brown Shrike	2020/11/13	2	0	22.69856080	120.64196080	https://www.inaturalist.org/observations/64848395
1174	Lanius cristatus	Brown Shrike	2022/9/30	1	0	22.70097417	88.37482106	https://www.inaturalist.org/observations/136993496
1175	Lanius cristatus	Brown Shrike	2020/10/16	1	0	22.70167984	120.29668370	https://www.inaturalist.org/observations/62790379
1176	Lanius cristatus	Brown Shrike	2021/10/21	2	0	22.70668333	120.30830330	https://www.inaturalist.org/observations/99205631
1177	Lanius cristatus	Brown Shrike	2020/11/10	3	0	22.70829085	120.54006040	https://www.inaturalist.org/observations/64727203
1178	Lanius cristatus	Brown Shrike	2021/1/12	2	0	22.70947644	120.53735820	https://www.inaturalist.org/observations/68054824
1179	Lanius cristatus	Brown Shrike	2020/1/22	2	0	22.70982090	120.36967100	https://www.inaturalist.org/observations/37979655
1180	Lanius cristatus	Brown Shrike	2022/2/1	1	0	22.71566667	120.52668330	https://www.inaturalist.org/observations/108288995
1181	Lanius cristatus	Brown Shrike	2018/10/6	2	0	22.71585056	120.47491600	https://www.inaturalist.org/observations/19673444
1182	Lanius cristatus	Brown Shrike	2020/2/10	2	0	22.71644565	120.47775810	https://www.inaturalist.org/observations/38503686
1183	Lanius cristatus	Brown Shrike	2020/11/24	2	0	22.72481086	120.61522250	https://www.inaturalist.org/observations/65581052
1184	Lanius cristatus	Brown Shrike	2021/4/19	1	0	22.72612848	115.34927410	https://www.inaturalist.org/observations/80717591
1185	Lanius cristatus	Brown Shrike	2022/10/12	2	0	22.72856944	120.29333890	https://www.inaturalist.org/observations/138482632
1186	Lanius cristatus	Brown Shrike	2014/9/4	2	0	22.72930500	113.77666500	https://www.inaturalist.org/observations/39808957
1187	Lanius cristatus	Brown Shrike	2015/1/20	3	0	22.73050931	120.27000960	https://www.inaturalist.org/observations/16456114
1188	Lanius cristatus	Brown Shrike	2020/1/11	2	0	22.73266987	120.31665210	https://www.inaturalist.org/observations/37481226
1189	Lanius cristatus	Brown Shrike	2017/1/22	2	0	22.73339700	120.31003310	https://www.inaturalist.org/observations/9457455
1190	Lanius cristatus	Brown Shrike	2021/12/12	2	0	22.73584071	120.30949940	https://www.inaturalist.org/observations/104853928
1191	Lanius cristatus	Brown Shrike	2022/1/16	1	0	22.73621368	121.05733490	https://www.inaturalist.org/observations/105017257
1192	Lanius cristatus	Brown Shrike	2020/1/28	4	0	22.73676024	121.06739720	https://www.inaturalist.org/observations/38054167
1193	Lanius cristatus	Brown Shrike	2020/5/14	4	0	22.73704586	121.06650960	https://www.inaturalist.org/observations/71014683
1194	Lanius cristatus	Brown Shrike	2021/12/6	2	0	22.74527488	120.37592810	https://www.inaturalist.org/observations/102580128
1195	Lanius cristatus	Brown Shrike	2022/2/8	1	0	22.75204086	120.31652650	https://www.inaturalist.org/observations/106371282

1196	Lanius cristatus	Brown Shrike	2020/9/25	2	0	22.75216903	120.31609570	https://www.inaturalist.org/observations/62639230
1197	Lanius cristatus	Brown Shrike	2021/3/28	2	0	22.75334180	121.14598110	https://www.inaturalist.org/observations/90224606
1198	Lanius cristatus	Brown Shrike	2022/2/8	1	0	22.75344084	120.31508880	https://www.inaturalist.org/observations/106371277
1199	Lanius cristatus	Brown Shrike	2019/12/14	2	0	22.75429523	120.24886700	https://www.inaturalist.org/observations/36774311
1200	Lanius cristatus	Brown Shrike	2020/9/26	1	0	22.75441013	120.26709270	https://www.inaturalist.org/observations/62768593
1201	Lanius cristatus	Brown Shrike	2022/2/8	1	0	22.75456381	120.31552530	https://www.inaturalist.org/observations/106371327
1202	Lanius cristatus	Brown Shrike	2020/9/26	2	0	22.75539304	120.26894210	https://www.inaturalist.org/observations/129821823
1203	Lanius cristatus	Brown Shrike	2020/9/26	1	0	22.75561717	120.26986070	https://www.inaturalist.org/observations/62770905
1204	Lanius cristatus	Brown Shrike	2022/12/19	1	0	22.76139420	121.15357270	https://www.inaturalist.org/observations/144642303
1205	Lanius cristatus	Brown Shrike	2021/1/19	3	0	22.76352882	121.05024720	https://www.inaturalist.org/observations/68368462
1206	Lanius cristatus	Brown Shrike	2022/12/18	1	0	22.76419220	121.16042700	https://www.inaturalist.org/observations/144575993
1207	Lanius cristatus	Brown Shrike	2020/3/19	2	0	22.76742782	88.38834543	https://www.inaturalist.org/observations/43658292
1208	Lanius cristatus	Brown Shrike	2022/1/19	1	0	22.77017091	121.15469690	https://www.inaturalist.org/observations/105210202
1209	Lanius cristatus	Brown Shrike	2021/12/7	1	0	22.77089500	121.14051950	https://www.inaturalist.org/observations/102647036
1210	Lanius cristatus	Brown Shrike	2020/3/21	3	0	22.77863500	120.28926000	https://www.inaturalist.org/observations/40349417
1211	Lanius cristatus	Brown Shrike	2022/11/17	1	0	22.78541667	120.29289440	https://www.inaturalist.org/observations/142207161
1212	Lanius cristatus	Brown Shrike	2020/3/21	2	0	22.78868500	88.42515100	https://www.inaturalist.org/observations/40358857
1213	Lanius cristatus	Brown Shrike	2020/9/12	2	0	22.79245360	88.29597950	https://www.inaturalist.org/observations/98007673
1214	Lanius cristatus	Brown Shrike	2022/9/7	2	0	22.79245360	88.29597950	https://www.inaturalist.org/observations/134010069
1215	Lanius cristatus	Brown Shrike	2022/1/3	1	0	22.79322464	121.12374770	https://www.inaturalist.org/observations/106016765
1216	Lanius cristatus	Brown Shrike	2021/1/7	2	0	22.79420100	88.29109600	https://www.inaturalist.org/observations/67714763
1217	Lanius cristatus	Brown Shrike	2022/10/9	1	0	22.80181170	88.33368621	https://www.inaturalist.org/observations/138744256
1218	Lanius cristatus	Brown Shrike	2019/6/19	2	0	22.80354226	112.07525580	https://www.inaturalist.org/observations/27260762

1219	Lanius cristatus	Brown Shrike	2022/11/27	2	0	22.80496181	88.33208207	https://www.inaturalist.org/observations/143038164
1220	Lanius cristatus	Brown Shrike	2022/10/13	1	0	22.81965597	72.29820975	https://www.inaturalist.org/observations/139507122
1221	Lanius cristatus	Brown Shrike	2020/10/6	2	0	22.82866813	120.26852090	https://www.inaturalist.org/observations/62287985
1222	Lanius cristatus	Brown Shrike	2020/10/6	2	0	22.82866923	120.26851960	https://www.inaturalist.org/observations/62287984
1223	Lanius cristatus	Brown Shrike	2020/10/6	2	0	22.82867032	120.26851830	https://www.inaturalist.org/observations/62287981
1224	Lanius cristatus	Brown Shrike	2021/2/6	1	0	22.83129413	120.29772640	https://www.inaturalist.org/observations/100153513
1225	Lanius cristatus	Brown Shrike	2021/11/23	1	0	22.83392748	86.72010499	https://www.inaturalist.org/observations/101823568
1226	Lanius cristatus	Brown Shrike	2022/2/24	2	0	22.83413328	120.21207440	https://www.inaturalist.org/observations/107369524
1227	Lanius cristatus	Brown Shrike	2017/11/27	2	0	22.83425359	120.21255390	https://www.inaturalist.org/observations/19940508
1228	Lanius cristatus	Brown Shrike	2019/10/13	2	0	22.83487092	120.21201730	https://www.inaturalist.org/observations/34298054
1229	Lanius cristatus	Brown Shrike	2022/11/6	1	0	22.83559147	120.45835240	https://www.inaturalist.org/observations/141244719
1230	Lanius cristatus	Brown Shrike	2020/10/5	2	0	22.83578754	120.20664840	https://www.inaturalist.org/observations/61765877
1231	Lanius cristatus	Brown Shrike	2020/1/5	2	0	22.83583260	120.45861050	https://www.inaturalist.org/observations/37297288
1232	Lanius cristatus	Brown Shrike	2022/2/18	1	0	22.83637304	120.27327540	https://www.inaturalist.org/observations/107117518
1233	Lanius cristatus	Brown Shrike	2022/9/17	1	0	22.84046755	120.58948980	https://www.inaturalist.org/observations/135992183
1234	Lanius cristatus	Brown Shrike	2022/10/8	2	0	22.84083855	120.58969670	https://www.inaturalist.org/observations/138452387
1235	Lanius cristatus	Brown Shrike	2022/3/17	1	0	22.84380817	120.29571970	https://www.inaturalist.org/observations/110005292
1236	Lanius cristatus	Brown Shrike	2020/3/16	2	0	22.86230635	120.53919440	https://www.inaturalist.org/observations/40692522
1237	Lanius cristatus	Brown Shrike	2020/10/15	2	0	22.86797782	120.54743630	https://www.inaturalist.org/observations/64034958
1238	Lanius cristatus	Brown Shrike	2020/10/15	1	0	22.86805320	120.54734510	https://www.inaturalist.org/observations/64034976
1239	Lanius cristatus	Brown Shrike	2019/9/20	1	0	22.87911681	120.65742320	https://www.inaturalist.org/observations/89996925
1240	Lanius cristatus	Brown Shrike	2021/2/2	2	0	22.88365082	120.20200420	https://www.inaturalist.org/observations/69143169
1241	Lanius cristatus	Brown Shrike	2022/1/18	2	0	22.88472300	120.44814870	https://www.inaturalist.org/observations/105158100

1242	Lanius cristatus	Brown Shrike	2019/3/26	2	0	22.88781233	120.62033960	https://www.inaturalist.org/observations/21630582
1243	Lanius cristatus	Brown Shrike	2017/12/5	2	0	22.89132472	120.57875850	https://www.inaturalist.org/observations/18209888
1244	Lanius cristatus	Brown Shrike	2005/3/26	2	0	22.89142776	120.51948060	https://www.inaturalist.org/observations/36017516
1245	Lanius cristatus	Brown Shrike	2017/12/5	2	0	22.89444688	120.57839780	https://www.inaturalist.org/observations/19940557
1246	Lanius cristatus	Brown Shrike	2020/9/20	2	0	22.89447447	120.52186090	https://www.inaturalist.org/observations/60319373
1247	Lanius cristatus	Brown Shrike	2021/1/28	4	0	22.90474401	121.12981070	https://www.inaturalist.org/observations/68834108
1248	Lanius cristatus	Brown Shrike	2021/11/6	1	0	22.90554463	120.55121590	https://www.inaturalist.org/observations/100680756
1249	Lanius cristatus	Brown Shrike	2019/12/28	2	0	22.90590667	120.68862500	https://www.inaturalist.org/observations/49692714
1250	Lanius cristatus	Brown Shrike	2021/12/21	2	0	22.90753631	83.91884811	https://www.inaturalist.org/observations/105913680
1251	Lanius cristatus	Brown Shrike	2021/2/20	1	0	22.90880158	120.31076290	https://www.inaturalist.org/observations/80767091
1252	Lanius cristatus	Brown Shrike	2020/11/24	2	0	22.90923099	120.55547430	https://www.inaturalist.org/observations/65581075
1253	Lanius cristatus	Brown Shrike	2020/1/8	2	0	22.91020102	120.55620320	https://www.inaturalist.org/observations/37394755
1254	Lanius cristatus	Brown Shrike	2021/9/9	2	0	22.91036944	120.55360280	https://www.inaturalist.org/observations/100500699
1255	Lanius cristatus	Brown Shrike	2022/4/16	1	0	22.91119120	120.55444820	https://www.inaturalist.org/observations/126333062
1256	Lanius cristatus	Brown Shrike	2021/11/6	1	0	22.91140727	120.55477790	https://www.inaturalist.org/observations/101166381
1257	Lanius cristatus	Brown Shrike	2022/9/16	2	0	22.91148000	120.55460830	https://www.inaturalist.org/observations/135621803
1258	Lanius cristatus	Brown Shrike	2021/1/17	2	0	22.91275983	121.25833280	https://www.inaturalist.org/observations/68477709
1259	Lanius cristatus	Brown Shrike	2021/5/2	1	0	22.91356833	120.31063890	https://www.inaturalist.org/observations/77705896
1260	Lanius cristatus	Brown Shrike	2020/4/25	3	0	22.91679000	120.26157500	https://www.inaturalist.org/observations/44091912
1261	Lanius cristatus	Brown Shrike	2018/9/29	2	0	22.91859013	120.31165730	https://www.inaturalist.org/observations/17025578
1262	Lanius cristatus	Brown Shrike	2018/10/21	2	0	22.92152162	120.29775760	https://www.inaturalist.org/observations/17710085
1263	Lanius cristatus	Brown Shrike	2020/9/27	2	0	22.92161167	120.26261720	https://www.inaturalist.org/observations/60964521
1264	Lanius cristatus	Brown Shrike	2019/4/27	2	0	22.92325800	120.31000500	https://www.inaturalist.org/observations/25060260

1265	Lanius cristatus	Brown Shrike	2018/5/10	1	0	22.92540785	120.30821300	https://www.inaturalist.org/observations/81665992
1266	Lanius cristatus	Brown Shrike	2018/3/30	1	0	22.92641868	120.30581800	https://www.inaturalist.org/observations/140768619
1267	Lanius cristatus	Brown Shrike	2020/2/28	2	0	22.92752848	120.25956250	https://www.inaturalist.org/observations/39403372
1268	Lanius cristatus	Brown Shrike	2020/2/16	2	0	22.92758467	120.26462180	https://www.inaturalist.org/observations/39405812
1269	Lanius cristatus	Brown Shrike	2020/10/4	1	0	22.92759910	88.55048970	https://www.inaturalist.org/observations/62284585
1270	Lanius cristatus	Brown Shrike	2019/10/26	3	0	22.92765834	120.30404170	https://www.inaturalist.org/observations/38213939
1271	Lanius cristatus	Brown Shrike	2019/10/26	2	0	22.92808333	120.30897000	https://www.inaturalist.org/observations/38216595
1272	Lanius cristatus	Brown Shrike	2018/5/10	1	0	22.92848511	120.30417170	https://www.inaturalist.org/observations/141776614
1273	Lanius cristatus	Brown Shrike	2020/10/30	2	0	22.92949528	120.26540860	https://www.inaturalist.org/observations/64038408
1274	Lanius cristatus	Brown Shrike	2022/9/30	1	0	22.92960505	88.42490762	https://www.inaturalist.org/observations/137111543
1275	Lanius cristatus	Brown Shrike	2019/1/26	1	0	22.93038094	120.30517960	https://www.inaturalist.org/observations/141360430
1276	Lanius cristatus	Brown Shrike	2020/1/4	2	0	22.93068321	120.30234820	https://www.inaturalist.org/observations/37266712
1277	Lanius cristatus	Brown Shrike	2020/4/25	2	0	22.93077014	120.26564550	https://www.inaturalist.org/observations/43282648
1278	Lanius cristatus	Brown Shrike	2020/1/4	2	0	22.93086143	120.29362800	https://www.inaturalist.org/observations/37265999
1279	Lanius cristatus	Brown Shrike	2020/4/25	2	0	22.93090932	120.26534180	https://www.inaturalist.org/observations/43283185
1280	Lanius cristatus	Brown Shrike	2020/4/25	2	0	22.93107285	120.26686930	https://www.inaturalist.org/observations/44074248
1281	Lanius cristatus	Brown Shrike	2020/10/30	2	0	22.93129012	120.26479390	https://www.inaturalist.org/observations/64036115
1282	Lanius cristatus	Brown Shrike	2020/1/4	2	0	22.93186250	120.29678830	https://www.inaturalist.org/observations/37266179
1283	Lanius cristatus	Brown Shrike	2022/1/9	2	0	22.93222167	120.22381670	https://www.inaturalist.org/observations/104649657
1284	Lanius cristatus	Brown Shrike	2020/4/14	3	0	22.93223190	120.22363280	https://www.inaturalist.org/observations/43198761
1285	Lanius cristatus	Brown Shrike	2020/1/4	2	0	22.93268024	120.29885630	https://www.inaturalist.org/observations/37295948
1286	Lanius cristatus	Brown Shrike	2020/1/4	2	0	22.93282470	120.29930410	https://www.inaturalist.org/observations/37296121
1287	Lanius cristatus	Brown Shrike	2022/4/4	3	0	22.93322298	120.22323110	https://www.inaturalist.org/observations/110622524

1288	Lanius cristatus	Brown Shrike	2021/1/2	3	0	22.93462778	120.22684720	https://www.inaturalist.org/observations/67706875
1289	Lanius cristatus	Brown Shrike	2021/9/25	1	0	22.93471917	120.30517580	https://www.inaturalist.org/observations/96364879
1290	Lanius cristatus	Brown Shrike	2021/1/2	3	0	22.93572222	120.22179170	https://www.inaturalist.org/observations/67706630
1291	Lanius cristatus	Brown Shrike	2021/11/21	2	0	22.93595050	120.22502190	https://www.inaturalist.org/observations/101704876
1292	Lanius cristatus	Brown Shrike	2021/1/22	2	0	22.93599073	120.22498520	https://www.inaturalist.org/observations/68437175
1293	Lanius cristatus	Brown Shrike	2020/1/4	2	0	22.93664861	120.30071720	https://www.inaturalist.org/observations/37370587
1294	Lanius cristatus	Brown Shrike	2018/10/27	2	0	22.93682586	120.22410580	https://www.inaturalist.org/observations/21022071
1295	Lanius cristatus	Brown Shrike	2019/11/23	2	0	22.93690500	120.30734330	https://www.inaturalist.org/observations/45045139
1296	Lanius cristatus	Brown Shrike	2021/5/5	1	0	22.94245357	120.53898030	https://www.inaturalist.org/observations/105256983
1297	Lanius cristatus	Brown Shrike	2020/4/13	2	0	22.94475405	121.12788960	https://www.inaturalist.org/observations/42938114
1298	Lanius cristatus	Brown Shrike	2017/2/3	3	0	22.94603105	120.23021820	https://www.inaturalist.org/observations/14779345
1299	Lanius cristatus	Brown Shrike	2018/3/31	1	0	22.95060400	88.53939600	https://www.inaturalist.org/observations/102363274
1300	Lanius cristatus	Brown Shrike	2021/1/24	2	0	22.95275200	88.53729439	https://www.inaturalist.org/observations/103891972
1301	Lanius cristatus	Brown Shrike	2022/1/2	2	0	22.95505943	88.53746586	https://www.inaturalist.org/observations/104753054
1302	Lanius cristatus	Brown Shrike	2020/12/31	2	0	22.95808313	120.52913880	https://www.inaturalist.org/observations/67573780
1303	Lanius cristatus	Brown Shrike	2020/3/4	2	0	22.96089180	120.24363270	https://www.inaturalist.org/observations/50386911
1304	Lanius cristatus	Brown Shrike	2022/5/2	1	0	22.96362260	120.24791650	https://www.inaturalist.org/observations/114669978
1305	Lanius cristatus	Brown Shrike	2018/2/15	2	0	22.96481018	86.89534058	https://www.inaturalist.org/observations/55191155
1306	Lanius cristatus	Brown Shrike	2018/2/16	2	0	22.96481018	86.89534058	https://www.inaturalist.org/observations/55191186
1307	Lanius cristatus	Brown Shrike	2018/2/16	2	0	22.96481018	86.89534058	https://www.inaturalist.org/observations/55191197
1308	Lanius cristatus	Brown Shrike	2019/1/19	4	0	22.96942470	86.99956125	https://www.inaturalist.org/observations/19729641
1309	Lanius cristatus	Brown Shrike	2022/1/23	1	0	22.97116964	120.22198660	https://www.inaturalist.org/observations/105479515
1310	Lanius cristatus	Brown Shrike	2022/5/16	1	0	22.97160433	120.22290080	https://www.inaturalist.org/observations/117260584

1311	Lanius cristatus	Brown Shrike	2022/5/18	2	0	22.97196333	120.22283500	https://www.inaturalist.org/observations/117613904
1312	Lanius cristatus	Brown Shrike	2017/2/18	2	0	22.97199290	120.22258740	https://www.inaturalist.org/observations/16286610
1313	Lanius cristatus	Brown Shrike	2017/3/27	2	0	22.97199290	120.22258740	https://www.inaturalist.org/observations/16286630
1314	Lanius cristatus	Brown Shrike	2020/10/30	2	0	22.97199290	120.22258740	https://www.inaturalist.org/observations/64032850
1315	Lanius cristatus	Brown Shrike	2018/5/10	1	0	22.97199290	120.22258740	https://www.inaturalist.org/observations/141775989
1316	Lanius cristatus	Brown Shrike	2018/5/10	1	0	22.97235651	120.22349250	https://www.inaturalist.org/observations/81665643
1317	Lanius cristatus	Brown Shrike	2022/4/30	1	0	22.97277167	120.22269500	https://www.inaturalist.org/observations/113727222
1318	Lanius cristatus	Brown Shrike	2018/5/10	1	0	22.97300311	120.22269760	https://www.inaturalist.org/observations/81665639
1319	Lanius cristatus	Brown Shrike	2018/4/18	3	0	22.97307987	120.22259720	https://www.inaturalist.org/observations/11219607
1320	Lanius cristatus	Brown Shrike	2020/10/9	2	0	22.97467013	120.21601490	https://www.inaturalist.org/observations/62105568
1321	Lanius cristatus	Brown Shrike	2020/4/24	2	0	22.97530610	120.22233060	https://www.inaturalist.org/observations/43020766
1322	Lanius cristatus	Brown Shrike	2019/10/27	2	0	22.98173811	120.26977130	https://www.inaturalist.org/observations/34944807
1323	Lanius cristatus	Brown Shrike	2022/2/24	2	0	22.98490278	120.20741110	https://www.inaturalist.org/observations/107359312
1324	Lanius cristatus	Brown Shrike	2020/11/23	3	0	22.98561152	86.99751615	https://www.inaturalist.org/observations/65470615
1325	Lanius cristatus	Brown Shrike	2018/1/17	2	0	22.98773148	87.00582041	https://www.inaturalist.org/observations/50670052
1326	Lanius cristatus	Brown Shrike	2019/3/2	4	0	22.98845494	87.00563283	https://www.inaturalist.org/observations/20876282
1327	Lanius cristatus	Brown Shrike	2019/3/2	4	0	22.98845494	87.00563283	https://www.inaturalist.org/observations/20876298
1328	Lanius cristatus	Brown Shrike	2020/11/13	2	0	22.98920315	87.00760241	https://www.inaturalist.org/observations/64846590
1329	Lanius cristatus	Brown Shrike	2018/1/24	3	0	22.98922533	87.00717779	https://www.inaturalist.org/observations/50670071
1330	Lanius cristatus	Brown Shrike	2022/9/16	0	0	22.98941287	120.22295620	https://www.inaturalist.org/observations/136879773
1331	Lanius cristatus	Brown Shrike	2022/10/1	1	0	22.98941287	120.22295620	https://www.inaturalist.org/observations/137142639
1332	Lanius cristatus	Brown Shrike	2019/10/10	3	0	22.98960065	87.00670572	https://www.inaturalist.org/observations/34497911
1333	Lanius cristatus	Brown Shrike	2020/10/11	3	0	22.98961286	86.99957087	https://www.inaturalist.org/observations/62407164

1334	Lanius cristatus	Brown Shrike	2018/2/5	5	0	22.98972087	87.00750233	https://www.inaturalist.org/observations/26958091
1335	Lanius cristatus	Brown Shrike	2017/11/5	4	0	22.99035383	87.00668158	https://www.inaturalist.org/observations/13358612
1336	Lanius cristatus	Brown Shrike	2022/11/28	2	0	22.99154438	87.00096677	https://www.inaturalist.org/observations/143234325
1337	Lanius cristatus	Brown Shrike	2020/9/12	5	0	22.99246973	87.00793727	https://www.inaturalist.org/observations/59412525
1338	Lanius cristatus	Brown Shrike	2016/5/9	3	0	22.99321226	120.21315600	https://www.inaturalist.org/observations/7927605
1339	Lanius cristatus	Brown Shrike	2020/11/20	3	0	22.99406212	86.99845614	https://www.inaturalist.org/observations/65338334
1340	Lanius cristatus	Brown Shrike	2019/2/23	2	0	22.99596574	120.41026330	https://www.inaturalist.org/observations/20653927
1341	Lanius cristatus	Brown Shrike	2019/2/11	4	0	22.99623975	87.02886781	https://www.inaturalist.org/observations/20281264
1342	Lanius cristatus	Brown Shrike	2018/9/12	1	0	22.99676250	120.21685640	https://www.inaturalist.org/observations/142661749
1343	Lanius cristatus	Brown Shrike	2018/4/13	1	0	22.99889450	120.21694500	https://www.inaturalist.org/observations/140379983
1344	Lanius cristatus	Brown Shrike	2021/1/21	2	0	23.00178104	120.20977140	https://www.inaturalist.org/observations/68404770
1345	Lanius cristatus	Brown Shrike	2021/1/4	3	0	23.00275400	120.24403900	https://www.inaturalist.org/observations/134715779
1346	Lanius cristatus	Brown Shrike	2019/2/23	2	0	23.00316625	120.15449910	https://www.inaturalist.org/observations/20684607
1347	Lanius cristatus	Brown Shrike	2021/1/4	1	0	23.00356076	120.24348120	https://www.inaturalist.org/observations/81662705
1348	Lanius cristatus	Brown Shrike	2022/12/24	1	0	23.00492700	120.14848300	https://www.inaturalist.org/observations/144973119
1349	Lanius cristatus	Brown Shrike	2021/3/10	3	0	23.00653462	120.25854370	https://www.inaturalist.org/observations/70917029
1350	Lanius cristatus	Brown Shrike	2020/12/18	2	0	23.00667500	120.27244500	https://www.inaturalist.org/observations/66718596
1351	Lanius cristatus	Brown Shrike	2020/2/8	0	0	23.00858209	120.17417960	https://www.inaturalist.org/observations/38563442
1352	Lanius cristatus	Brown Shrike	2019/12/8	3	0	23.01752713	120.19143150	https://www.inaturalist.org/observations/48753306
1353	Lanius cristatus	Brown Shrike	2019/12/8	2	0	23.02165184	120.20250380	https://www.inaturalist.org/observations/45952555
1354	Lanius cristatus	Brown Shrike	2021/1/11	1	0	23.02169341	120.33394610	https://www.inaturalist.org/observations/81661279
1355	Lanius cristatus	Brown Shrike	2019/9/29	2	0	23.02545480	120.22648860	https://www.inaturalist.org/observations/34629340
1356	Lanius cristatus	Brown Shrike	2022/1/10	1	0	23.04582646	121.15325120	https://www.inaturalist.org/observations/104692350

1357	Lanius cristatus	Brown Shrike	2021/12/29	2	0	23.04606433	121.15240830	https://www.inaturalist.org/observations/103871160
1358	Lanius cristatus	Brown Shrike	2022/1/22	0	0	23.04612397	121.15241500	https://www.inaturalist.org/observations/105351711
1359	Lanius cristatus	Brown Shrike	2022/1/3	1	0	23.04622000	121.15240300	https://www.inaturalist.org/observations/104249853
1360	Lanius cristatus	Brown Shrike	2020/9/3	2	0	23.05117024	120.18727700	https://www.inaturalist.org/observations/58375195
1361	Lanius cristatus	Brown Shrike	2018/3/19	2	0	23.05337601	120.18703940	https://www.inaturalist.org/observations/10353850
1362	Lanius cristatus	Brown Shrike	2019/10/6	2	0	23.05341100	120.24586200	https://www.inaturalist.org/observations/37518652
1363	Lanius cristatus	Brown Shrike	2020/5/2	2	0	23.05367806	120.24532310	https://www.inaturalist.org/observations/69364857
1364	Lanius cristatus	Brown Shrike	2018/12/22	2	0	23.05500667	120.24494500	https://www.inaturalist.org/observations/19490066
1365	Lanius cristatus	Brown Shrike	2020/2/10	2	0	23.05804854	120.18547820	https://www.inaturalist.org/observations/50401946
1366	Lanius cristatus	Brown Shrike	2017/1/7	3	0	23.06444100	120.19731100	https://www.inaturalist.org/observations/37360969
1367	Lanius cristatus	Brown Shrike	2018/11/9	3	0	23.06471870	120.19633860	https://www.inaturalist.org/observations/64482507
1368	Lanius cristatus	Brown Shrike	2017/9/21	2	0	23.06474567	120.19650440	https://www.inaturalist.org/observations/60418876
1369	Lanius cristatus	Brown Shrike	2018/11/9	3	0	23.06492862	120.19638690	https://www.inaturalist.org/observations/64482505
1370	Lanius cristatus	Brown Shrike	2019/1/10	2	0	23.06494500	120.19646500	https://www.inaturalist.org/observations/37441127
1371	Lanius cristatus	Brown Shrike	2020/9/25	5	0	23.06499315	120.19646980	https://www.inaturalist.org/observations/60800098
1372	Lanius cristatus	Brown Shrike	2020/9/16	4	0	23.06514260	120.19660160	https://www.inaturalist.org/observations/60800097
1373	Lanius cristatus	Brown Shrike	2017/11/12	2	0	23.07253283	120.17636820	https://www.inaturalist.org/observations/64729095
1374	Lanius cristatus	Brown Shrike	2020/2/12	2	0	23.07253648	120.17639860	https://www.inaturalist.org/observations/50371325
1375	Lanius cristatus	Brown Shrike	2019/4/27	1	0	23.07794780	120.06882180	https://www.inaturalist.org/observations/23260509
1376	Lanius cristatus	Brown Shrike	2020/10/9	2	0	23.08087133	120.16797950	https://www.inaturalist.org/observations/62112931
1377	Lanius cristatus	Brown Shrike	2020/10/9	2	0	23.08100848	120.16798360	https://www.inaturalist.org/observations/62112617
1378	Lanius cristatus	Brown Shrike	2020/1/19	1	0	23.08464717	113.45440000	https://www.inaturalist.org/observations/142747180
1379	Lanius cristatus	Brown Shrike	2018/9/25	1	0	23.09234930	120.12739690	https://www.inaturalist.org/observations/16893547

1380	Lanius cristatus	Brown Shrike	2021/12/23	2	0	23.09425556	120.35088060	https://www.inaturalist.org/observations/103525082
1381	Lanius cristatus	Brown Shrike	2022/12/22	1	0	23.09682500	120.36000820	https://www.inaturalist.org/observations/144856996
1382	Lanius cristatus	Brown Shrike	2020/2/1	3	0	23.09885283	120.36103000	https://www.inaturalist.org/observations/38217436
1383	Lanius cristatus	Brown Shrike	2022/4/21	1	0	23.10018407	113.29714290	https://www.inaturalist.org/observations/142639888
1384	Lanius cristatus	Brown Shrike	2022/9/17	1	0	23.10035865	113.29656930	https://www.inaturalist.org/observations/142432787
1385	Lanius cristatus	Brown Shrike	2020/1/31	2	0	23.10078460	120.28366380	https://www.inaturalist.org/observations/50402700
1386	Lanius cristatus	Brown Shrike	2018/9/3	1	0	23.10140291	113.29475710	https://www.inaturalist.org/observations/142752586
1387	Lanius cristatus	Brown Shrike	2022/10/14	1	0	23.10158388	113.29499320	https://www.inaturalist.org/observations/142432746
1388	Lanius cristatus	Brown Shrike	2022/5/17	1	0	23.10224713	113.29240460	https://www.inaturalist.org/observations/142601076
1389	Lanius cristatus	Brown Shrike	2022/5/19	1	0	23.10224713	113.29240460	https://www.inaturalist.org/observations/142601086
1390	Lanius cristatus	Brown Shrike	2020/1/26	4	0	23.10310343	86.86219661	https://www.inaturalist.org/observations/38902444
1391	Lanius cristatus	Brown Shrike	2019/12/15	2	0	23.10489203	121.22233330	https://www.inaturalist.org/observations/36773718
1392	Lanius cristatus	Brown Shrike	2019/4/8	2	0	23.10638986	120.27342310	https://www.inaturalist.org/observations/26744545
1393	Lanius cristatus	Brown Shrike	2020/10/6	2	0	23.11289480	120.28168910	https://www.inaturalist.org/observations/62295967
1394	Lanius cristatus	Brown Shrike	2022/10/27	1	0	23.11391389	120.29867780	https://www.inaturalist.org/observations/141581603
1395	Lanius cristatus	Brown Shrike	2015/4/10	2	0	23.11420260	120.29820010	https://www.inaturalist.org/observations/16280525
1396	Lanius cristatus	Brown Shrike	2019/1/3	4	0	23.11624833	121.22241500	https://www.inaturalist.org/observations/19722979
1397	Lanius cristatus	Brown Shrike	2019/1/3	2	0	23.11750000	121.22231000	https://www.inaturalist.org/observations/19722977
1398	Lanius cristatus	Brown Shrike	2020/4/3	2	0	23.11818228	121.22417450	https://www.inaturalist.org/observations/41665548
1399	Lanius cristatus	Brown Shrike	2022/1/6	1	0	23.11847744	121.22258760	https://www.inaturalist.org/observations/106103379
1400	Lanius cristatus	Brown Shrike	2020/4/3	2	0	23.11917769	121.22552390	https://www.inaturalist.org/observations/41665551
1401	Lanius cristatus	Brown Shrike	2021/9/3	1	0	23.11951660	120.10118360	https://www.inaturalist.org/observations/100826997
1402	Lanius cristatus	Brown Shrike	2021/3/11	2	0	23.12615270	121.36559890	https://www.inaturalist.org/observations/74822067

1403	Lanius cristatus	Brown Shrike	2021/3/12	1	0	23.12615270	121.36559890	https://www.inaturalist.org/observations/74822071
1404	Lanius cristatus	Brown Shrike	2021/10/2	1	0	23.12808108	120.12155390	https://www.inaturalist.org/observations/97101393
1405	Lanius cristatus	Brown Shrike	2021/10/2	1	0	23.12811623	120.12201120	https://www.inaturalist.org/observations/97101392
1406	Lanius cristatus	Brown Shrike	2012/4/13	2	0	23.13520050	120.49379730	https://www.inaturalist.org/observations/14643230
1407	Lanius cristatus	Brown Shrike	2012/2/1	2	0	23.13524818	120.49377440	https://www.inaturalist.org/observations/14643227
1408	Lanius cristatus	Brown Shrike	2011/9/24	2	0	23.13526917	120.49374390	https://www.inaturalist.org/observations/14643181
1409	Lanius cristatus	Brown Shrike	2011/10/15	2	0	23.13526917	120.49375150	https://www.inaturalist.org/observations/14643212
1410	Lanius cristatus	Brown Shrike	2022/9/12	2	0	23.15441600	121.28552490	https://www.inaturalist.org/observations/140126397
1411	Lanius cristatus	Brown Shrike	2022/11/18	2	0	23.15441600	121.28552490	https://www.inaturalist.org/observations/143926403
1412	Lanius cristatus	Brown Shrike	2022/10/31	1	0	23.15441600	121.28552490	https://www.inaturalist.org/observations/143989888
1413	Lanius cristatus	Brown Shrike	2022/10/17	1	0	23.15441600	121.28552490	https://www.inaturalist.org/observations/143997770
1414	Lanius cristatus	Brown Shrike	2022/11/7	1	0	23.15441600	121.28552490	https://www.inaturalist.org/observations/144001113
1415	Lanius cristatus	Brown Shrike	2022/1/13	1	0	23.16640300	77.37238000	https://www.inaturalist.org/observations/105019672
1416	Lanius cristatus	Brown Shrike	2020/9/13	1	0	23.16723299	69.18795511	https://www.inaturalist.org/observations/137051676
1417	Lanius cristatus	Brown Shrike	2016/12/1	6	0	23.17304100	87.96714200	http://www.inaturalist.org/observations/4679920
1418	Lanius cristatus	Brown Shrike	2018/1/1	2	0	23.17535500	87.96075400	https://www.inaturalist.org/observations/56031416
1419	Lanius cristatus	Brown Shrike	2016/9/17	5	0	23.17805200	87.96040400	http://www.inaturalist.org/observations/4130647
1420	Lanius cristatus	Brown Shrike	2019/11/21	1	0	23.18209413	120.31262240	https://www.inaturalist.org/observations/102379335
1421	Lanius cristatus	Brown Shrike	2014/10/14	4	0	23.18262091	120.31220790	https://www.inaturalist.org/observations/57529507
1422	Lanius cristatus	Brown Shrike	2022/1/9	2	0	23.18314712	120.31085460	https://www.inaturalist.org/observations/104643930
1423	Lanius cristatus	Brown Shrike	2019/11/14	2	0	23.18343102	120.31190110	https://www.inaturalist.org/observations/35663957
1424	Lanius cristatus	Brown Shrike	2019/11/14	2	0	23.18360369	120.31173330	https://www.inaturalist.org/observations/35663934
1425	Lanius cristatus	Brown Shrike	2019/12/22	2	0	23.18388025	120.31299360	https://www.inaturalist.org/observations/49688944

1426	Lanius cristatus	Brown Shrike	2022/4/3	1	0	23.18397497	113.47805500	https://www.inaturalist.org/observations/116052266
1427	Lanius cristatus	Brown Shrike	2019/12/12	2	0	23.18436487	120.31337560	https://www.inaturalist.org/observations/36606904
1428	Lanius cristatus	Brown Shrike	2022/9/28	3	0	23.18448667	120.09599170	https://www.inaturalist.org/observations/136755425
1429	Lanius cristatus	Brown Shrike	2019/11/19	2	0	23.18597363	120.31615880	https://www.inaturalist.org/observations/35875757
1430	Lanius cristatus	Brown Shrike	2020/3/1	2	0	23.18601150	120.37431650	https://www.inaturalist.org/observations/50386910
1431	Lanius cristatus	Brown Shrike	2022/10/6	2	0	23.19023471	120.24522360	https://www.inaturalist.org/observations/137714883
1432	Lanius cristatus	Brown Shrike	2019/11/14	2	0	23.19465178	120.29732430	https://www.inaturalist.org/observations/35672661
1433	Lanius cristatus	Brown Shrike	2018/9/30	0	0	23.20719655	69.26338028	https://www.inaturalist.org/observations/137872965
1434	Lanius cristatus	Brown Shrike	2022/4/5	1	0	23.20753083	120.36051190	https://www.inaturalist.org/observations/110825946
1435	Lanius cristatus	Brown Shrike	2022/4/5	1	0	23.20777639	120.35907000	https://www.inaturalist.org/observations/110825941
1436	Lanius cristatus	Brown Shrike	2022/4/5	1	0	23.20779583	120.35929030	https://www.inaturalist.org/observations/110825949
1437	Lanius cristatus	Brown Shrike	2022/4/5	1	0	23.20900583	120.35854030	https://www.inaturalist.org/observations/110825944
1438	Lanius cristatus	Brown Shrike	2020/12/26	2	0	23.22397660	87.89792480	https://www.inaturalist.org/observations/70433914
1439	Lanius cristatus	Brown Shrike	2020/9/10	5	0	23.23643156	120.11336470	https://www.inaturalist.org/observations/59418328
1440	Lanius cristatus	Brown Shrike	2020/5/13	2	0	23.25316806	119.66829310	https://www.inaturalist.org/observations/46204798
1441	Lanius cristatus	Brown Shrike	2020/5/15	2	0	23.25406642	119.66848280	https://www.inaturalist.org/observations/46210534
1442	Lanius cristatus	Brown Shrike	2020/5/15	2	0	23.25406642	119.66848280	https://www.inaturalist.org/observations/46210536
1443	Lanius cristatus	Brown Shrike	2020/5/15	2	0	23.25406642	119.66848280	https://www.inaturalist.org/observations/46210537
1444	Lanius cristatus	Brown Shrike	2020/1/10	2	0	23.26393477	120.12104930	https://www.inaturalist.org/observations/38117408
1445	Lanius cristatus	Brown Shrike	2020/9/26	2	0	23.26625653	120.50053250	https://www.inaturalist.org/observations/61281303
1446	Lanius cristatus	Brown Shrike	2020/2/29	4	0	23.27208380	86.70998007	https://www.inaturalist.org/observations/39575801
1447	Lanius cristatus	Brown Shrike	2016/10/30	2	0	23.28199956	71.68806233	https://www.inaturalist.org/observations/37247296
1448	Lanius cristatus	Brown Shrike	2019/12/7	2	0	23.28564833	120.39741830	https://www.inaturalist.org/observations/53311059

1449	Lanius cristatus	Brown Shrike	2019/12/7	1	0	23.28571167	120.39740330	https://www.inaturalist.org/observations/97093148
1450	Lanius cristatus	Brown Shrike	2019/12/7	2	0	23.28597500	120.39723500	https://www.inaturalist.org/observations/53311031
1451	Lanius cristatus	Brown Shrike	2019/10/24	2	0	23.29892880	121.26015330	https://www.inaturalist.org/observations/35709297
1452	Lanius cristatus	Brown Shrike	2020/4/8	4	0	23.32184861	120.16673890	https://www.inaturalist.org/observations/47271638
1453	Lanius cristatus	Brown Shrike	2021/4/10	2	0	23.32234754	120.31084080	https://www.inaturalist.org/observations/74816447
1454	Lanius cristatus	Brown Shrike	2021/4/10	1	0	23.32250500	120.31042100	https://www.inaturalist.org/observations/74816446
1455	Lanius cristatus	Brown Shrike	2020/11/24	2	0	23.33120556	120.32082220	https://www.inaturalist.org/observations/65505694
1456	Lanius cristatus	Brown Shrike	2020/10/1	2	0	23.33471528	120.18564440	https://www.inaturalist.org/observations/62285405
1457	Lanius cristatus	Brown Shrike	2021/11/23	3	0	23.35223500	116.68323400	https://www.inaturalist.org/observations/101766219
1458	Lanius cristatus	Brown Shrike	2021/11/23	4	0	23.35223500	116.68323400	https://www.inaturalist.org/observations/101766220
1459	Lanius cristatus lucionensis	Brown Shrike	2022/9/7	0	0	23.37895439	120.16068800	https://www.inaturalist.org/observations/134008701
1460	Lanius cristatus	Brown Shrike	2022/9/7	1	0	23.37919167	120.16201120	https://www.inaturalist.org/observations/134045674
1461	Lanius cristatus	Brown Shrike	2015/1/24	2	0	23.38889930	71.34159440	https://www.inaturalist.org/observations/33243087
1462	Lanius cristatus	Brown Shrike	2022/10/24	0	0	23.40400700	121.21684660	https://www.inaturalist.org/observations/143911187
1463	Lanius cristatus	Brown Shrike	2022/9/26	0	0	23.40400700	121.21684660	https://www.inaturalist.org/observations/143913570
1464	Lanius cristatus	Brown Shrike	2022/8/29	1	0	23.40400700	121.21684660	https://www.inaturalist.org/observations/144213659
1465	Lanius cristatus	Brown Shrike	2016/1/8	3	0	23.41182576	121.34753390	https://www.inaturalist.org/observations/66651967
1466	Lanius cristatus	Brown Shrike	2016/1/8	2	0	23.41591606	121.34987730	https://www.inaturalist.org/observations/14814724
1467	Lanius cristatus	Brown Shrike	2019/11/13	3	0	23.42501768	120.45499510	https://www.inaturalist.org/observations/35700457
1468	Lanius cristatus	Brown Shrike	2020/10/25	2	0	23.42624480	120.45410560	https://www.inaturalist.org/observations/63634179
1469	Lanius cristatus	Brown Shrike	2021/3/9	2	0	23.42886778	120.39780170	https://www.inaturalist.org/observations/72071844
1470	Lanius cristatus	Brown Shrike	2021/9/27	2	0	23.43158833	120.54754170	https://www.inaturalist.org/observations/98887683
1471	Lanius cristatus	Brown Shrike	2019/4/29	3	0	23.43171182	120.58721320	https://www.inaturalist.org/observations/23882456

1472	Lanius cristatus	Brown Shrike	2020/2/2	3	0	23.43781884	120.43521450	https://www.inaturalist.org/observations/38221404
1473	Lanius cristatus	Brown Shrike	2018/12/7	4	0	23.44457096	88.34205166	https://www.inaturalist.org/observations/19906188
1474	Lanius cristatus	Brown Shrike	2022/9/13	1	0	23.45053211	121.39663940	https://www.inaturalist.org/observations/140238875
1475	Lanius cristatus	Brown Shrike	2018/11/14	2	0	23.45286297	120.46302720	https://www.inaturalist.org/observations/19247004
1476	Lanius cristatus	Brown Shrike	2021/12/29	1	0	23.45317833	120.55983170	https://www.inaturalist.org/observations/108573438
1477	Lanius cristatus	Brown Shrike	2022/10/20	1	0	23.45318838	120.55944780	https://www.inaturalist.org/observations/139850618
1478	Lanius cristatus	Brown Shrike	2013/12/23	1	0	23.45325900	120.55941830	https://www.inaturalist.org/observations/118748187
1479	Lanius cristatus	Brown Shrike	2014/1/2	1	0	23.45327658	120.56031040	https://www.inaturalist.org/observations/98086546
1480	Lanius cristatus	Brown Shrike	2022/12/16	2	0	23.45334069	120.55982680	https://www.inaturalist.org/observations/144532617
1481	Lanius cristatus	Brown Shrike	2016/1/20	1	0	23.45345987	120.55891520	https://www.inaturalist.org/observations/96318683
1482	Lanius cristatus	Brown Shrike	2020/1/17	3	0	23.45347964	120.55923700	https://www.inaturalist.org/observations/60689549
1483	Lanius cristatus	Brown Shrike	2015/3/9	2	0	23.45348328	120.55889960	https://www.inaturalist.org/observations/97962308
1484	Lanius cristatus	Brown Shrike	2015/12/22	2	0	23.45348328	120.55889960	https://www.inaturalist.org/observations/97962313
1485	Lanius cristatus	Brown Shrike	2015/5/6	2	0	23.45348328	120.55889960	https://www.inaturalist.org/observations/97962317
1486	Lanius cristatus	Brown Shrike	2015/1/14	2	0	23.45348328	120.55889960	https://www.inaturalist.org/observations/97962318
1487	Lanius cristatus	Brown Shrike	2013/2/6	1	0	23.45353369	120.55886700	https://www.inaturalist.org/observations/101314986
1488	Lanius cristatus	Brown Shrike	2020/10/16	2	0	23.45362730	120.55910370	https://www.inaturalist.org/observations/63076078
1489	Lanius cristatus	Brown Shrike	2021/12/24	1	0	23.45629778	120.45702470	https://www.inaturalist.org/observations/103569233
1490	Lanius cristatus	Brown Shrike	2020/11/1	3	0	23.45644663	69.29472966	https://www.inaturalist.org/observations/136907808
1491	Lanius cristatus	Brown Shrike	2020/12/10	2	0	23.45686167	120.25689670	https://www.inaturalist.org/observations/66368130
1492	Lanius cristatus	Brown Shrike	2019/4/29	3	0	23.45914179	120.32477810	https://www.inaturalist.org/observations/24166898
1493	Lanius cristatus	Brown Shrike	2021/1/12	2	0	23.46054893	120.47043100	https://www.inaturalist.org/observations/79382125
1494	Lanius cristatus	Brown Shrike	2009/2/5	2	0	23.46155072	120.47521260	https://www.inaturalist.org/observations/16128393

1495	Lanius cristatus	Brown Shrike	2021/9/27	2	0	23.46158333	120.46821000	https://www.inaturalist.org/observations/98887671
1496	Lanius cristatus	Brown Shrike	2015/12/11	2	0	23.46182050	71.28742090	https://www.inaturalist.org/observations/56202829
1497	Lanius cristatus	Brown Shrike	2012/1/20	1	0	23.46182050	71.28742090	https://www.inaturalist.org/observations/101871656
1498	Lanius cristatus	Brown Shrike	2020/12/6	3	0	23.47062590	120.29346420	https://www.inaturalist.org/observations/134716011
1499	Lanius cristatus	Brown Shrike	2020/12/6	1	0	23.47395294	120.29165740	https://www.inaturalist.org/observations/81663367
1500	Lanius cristatus	Brown Shrike	2022/1/2	3	0	23.47545000	120.45109170	https://www.inaturalist.org/observations/104147882
1501	Lanius cristatus	Brown Shrike	2005/10/27	1	0	23.48007510	120.44911130	https://www.inaturalist.org/observations/77854881
1502	Lanius cristatus	Brown Shrike	2018/3/13	2	0	23.48104976	120.67368340	https://www.inaturalist.org/observations/65019653
1503	Lanius cristatus	Brown Shrike	2018/9/11	3	0	23.48235330	120.43590820	https://www.inaturalist.org/observations/16418732
1504	Lanius cristatus	Brown Shrike	2018/10/1	2	0	23.48351098	120.63085790	https://www.inaturalist.org/observations/17130399
1505	Lanius cristatus	Brown Shrike	2021/9/18	2	0	23.48473824	120.48721100	https://www.inaturalist.org/observations/95733356
1506	Lanius cristatus	Brown Shrike	2020/9/4	2	0	23.48593889	120.45275280	https://www.inaturalist.org/observations/58583305
1507	Lanius cristatus	Brown Shrike	2019/11/9	2	0	23.49306899	120.44718700	https://www.inaturalist.org/observations/49028704
1508	Lanius cristatus	Brown Shrike	2020/2/13	2	0	23.49322083	120.15019660	https://www.inaturalist.org/observations/38625223
1509	Lanius cristatus	Brown Shrike	2021/2/20	2	0	23.49447930	120.15382970	https://www.inaturalist.org/observations/70087297
1510	Lanius cristatus	Brown Shrike	2020/2/13	2	0	23.49977333	120.16634670	https://www.inaturalist.org/observations/38625247
1511	Lanius cristatus	Brown Shrike	2019/5/9	3	0	23.50248718	121.00195310	https://www.inaturalist.org/observations/25296180
1512	Lanius cristatus	Brown Shrike	2019/5/9	3	0	23.50249290	121.00195310	https://www.inaturalist.org/observations/25296119
1513	Lanius cristatus	Brown Shrike	2020/2/6	3	0	23.50275276	120.14879570	https://www.inaturalist.org/observations/40698641
1514	Lanius cristatus	Brown Shrike	2022/1/2	1	0	23.50456167	120.15588330	https://www.inaturalist.org/observations/104145260
1515	Lanius cristatus	Brown Shrike	2022/3/19	1	0	23.50717101	91.47139554	https://www.inaturalist.org/observations/111090543
1516	Lanius cristatus	Brown Shrike	2022/4/21	2	0	23.50981333	121.39056500	https://www.inaturalist.org/observations/112940629
1517	Lanius cristatus	Brown Shrike	2021/3/6	3	0	23.51000000	120.14800000	https://www.inaturalist.org/observations/70737127

1518	Lanius cristatus	Brown Shrike	2021/9/14	1	0	23.51035400	120.82310090	https://www.inaturalist.org/observations/102980388
1519	Lanius cristatus	Brown Shrike	2016/8/16	1	0	23.51103471	120.14556470	https://www.inaturalist.org/observations/80462969
1520	Lanius cristatus	Brown Shrike	2020/10/2	2	0	23.51579298	120.14485240	https://www.inaturalist.org/observations/81453016
1521	Lanius cristatus	Brown Shrike	2022/3/20	1	0	23.51944752	91.47531590	https://www.inaturalist.org/observations/111103331
1522	Lanius cristatus	Brown Shrike	2021/3/14	4	0	23.52044430	87.31192270	https://www.inaturalist.org/observations/71491479
1523	Lanius cristatus	Brown Shrike	2020/1/19	2	0	23.52521333	120.19414170	https://www.inaturalist.org/observations/37772093
1524	Lanius cristatus	Brown Shrike	2020/1/19	2	0	23.52715333	120.20526170	https://www.inaturalist.org/observations/37772107
1525	Lanius cristatus	Brown Shrike	2021/12/21	1	0	23.53392609	120.15069150	https://www.inaturalist.org/observations/108797158
1526	Lanius cristatus	Brown Shrike	2019/10/26	0	0	23.53686842	120.18564850	https://www.inaturalist.org/observations/34913449
1527	Lanius cristatus	Brown Shrike	2021/12/21	2	0	23.53913680	120.17323100	https://www.inaturalist.org/observations/103445238
1528	Lanius cristatus	Brown Shrike	2019/4/18	6	0	23.53949300	81.05517550	https://www.inaturalist.org/observations/123432311
1529	Lanius cristatus	Brown Shrike	2021/1/10	2	0	23.54173833	120.17365670	https://www.inaturalist.org/observations/67887583
1530	Lanius cristatus	Brown Shrike	2003/2/13	4	0	23.54364430	120.42336540	https://www.inaturalist.org/observations/13814506
1531	Lanius cristatus	Brown Shrike	2020/12/25	3	0	23.54427944	120.43752560	https://www.inaturalist.org/observations/67033934
1532	Lanius cristatus	Brown Shrike	2022/3/15	1	0	23.54651153	120.20760900	https://www.inaturalist.org/observations/108656309
1533	Lanius cristatus	Brown Shrike	2021/9/18	3	0	23.54861752	120.62842570	https://www.inaturalist.org/observations/95244279
1534	Lanius cristatus	Brown Shrike	2022/3/8	1	0	23.55205060	120.17409260	https://www.inaturalist.org/observations/108902875
1535	Lanius cristatus	Brown Shrike	2014/12/21	1	0	23.55784754	120.43544500	https://www.inaturalist.org/observations/56719660
1536	Lanius cristatus	Brown Shrike	2016/11/25	5	0	23.55805700	87.34445300	http://www.inaturalist.org/observations/4655679
1537	Lanius cristatus	Brown Shrike	2019/4/29	4	0	23.55999673	120.47171990	https://www.inaturalist.org/observations/23934812
1538	Lanius cristatus	Brown Shrike	2019/4/29	5	0	23.56013746	120.47181920	https://www.inaturalist.org/observations/24418352
1539	Lanius cristatus	Brown Shrike	2016/12/17	3	0	23.56669080	87.30347710	https://www.inaturalist.org/observations/5433660
1540	Lanius cristatus	Brown Shrike	2020/4/25	2	0	23.56758528	120.16587890	https://www.inaturalist.org/observations/44296061

1541	Lanius cristatus	Brown Shrike	2022/4/30	1	0	23.56879180	120.17157980	https://www.inaturalist.org/observations/114454862
1542	Lanius cristatus	Brown Shrike	2021/1/10	2	0	23.57048833	120.16400500	https://www.inaturalist.org/observations/67887558
1543	Lanius cristatus	Brown Shrike	2022/11/25	1	0	23.58519010	121.50274040	https://www.inaturalist.org/observations/143858210
1544	Lanius cristatus	Brown Shrike	2020/5/12	2	0	23.58747833	120.31786460	https://www.inaturalist.org/observations/45645943
1545	Lanius cristatus	Brown Shrike	2018/10/9	3	0	23.58877167	87.33724000	https://www.inaturalist.org/observations/17408746
1546	Lanius cristatus	Brown Shrike	2018/2/1	1	0	23.58881600	87.33407700	https://www.inaturalist.org/observations/102354495
1547	Lanius cristatus	Brown Shrike	2019/10/13	2	0	23.58905667	87.33467500	https://www.inaturalist.org/observations/35948254
1548	Lanius cristatus	Brown Shrike	2019/10/13	3	0	23.58914667	87.33467500	https://www.inaturalist.org/observations/34363325
1549	Lanius cristatus	Brown Shrike	2006/1/12	4	0	23.58916000	80.41992000	https://www.inaturalist.org/observations/9771870
1550	Lanius cristatus	Brown Shrike	2015/9/16	5	0	23.59202600	87.22679800	http://www.inaturalist.org/observations/1951301
1551	Lanius cristatus	Brown Shrike	2021/11/24	2	0	23.59528248	120.35679300	https://www.inaturalist.org/observations/126223396
1552	Lanius cristatus	Brown Shrike	2021/10/5	0	0	23.59577521	120.59822940	https://www.inaturalist.org/observations/97198016
1553	Lanius cristatus	Brown Shrike	2021/10/5	1	0	23.59584000	120.59814670	https://www.inaturalist.org/observations/97316871
1554	Lanius cristatus	Brown Shrike	2021/9/19	2	0	23.59662806	120.61230500	https://www.inaturalist.org/observations/95374166
1555	Lanius cristatus	Brown Shrike	2018/10/21	3	0	23.59790000	87.36432833	https://www.inaturalist.org/observations/18513396
1556	Lanius cristatus	Brown Shrike	2021/3/11	4	0	23.59908004	120.28085910	https://www.inaturalist.org/observations/71041156
1557	Lanius cristatus	Brown Shrike	2019/1/28	2	0	23.59937185	120.24248070	https://www.inaturalist.org/observations/19927422
1558	Lanius cristatus	Brown Shrike	2019/4/28	3	0	23.60009466	120.44479830	https://www.inaturalist.org/observations/24186173
1559	Lanius cristatus	Brown Shrike	2020/2/10	2	0	23.60231180	120.57932290	https://www.inaturalist.org/observations/38526533
1560	Lanius cristatus	Brown Shrike	2021/9/20	1	0	23.60776746	120.61046340	https://www.inaturalist.org/observations/95614628
1561	Lanius cristatus	Brown Shrike	2018/12/9	3	0	23.60809397	120.34711260	https://www.inaturalist.org/observations/18917157
1562	Lanius cristatus	Brown Shrike	2013/11/12	2	0	23.60850380	120.54458080	https://www.inaturalist.org/observations/8498315
1563	Lanius cristatus	Brown Shrike	2020/4/27	2	0	23.60911667	120.45938330	https://www.inaturalist.org/observations/44094299

1564	Lanius cristatus	Brown Shrike	2019/5/9	2	0	23.61039219	120.16134420	https://www.inaturalist.org/observations/24831342
1565	Lanius cristatus	Brown Shrike	2021/2/2	3	0	23.61190333	120.18977170	https://www.inaturalist.org/observations/68974251
1566	Lanius cristatus	Brown Shrike	2021/11/24	1	0	23.61230000	120.25061000	https://www.inaturalist.org/observations/102239252
1567	Lanius cristatus	Brown Shrike	2020/2/3	2	0	23.61330000	120.18077670	https://www.inaturalist.org/observations/38282497
1568	Lanius cristatus	Brown Shrike	2018/12/11	2	0	23.61663036	120.26265540	https://www.inaturalist.org/observations/18958355
1569	Lanius cristatus lucionensis	Brown Shrike	2016/11/23	2	0	23.61670635	87.24338750	https://www.inaturalist.org/observations/5433363
1570	Lanius cristatus	Brown Shrike	2020/3/5	2	0	23.61737667	120.26110500	https://www.inaturalist.org/observations/39604429
1571	Lanius cristatus	Brown Shrike	2020/3/23	2	0	23.61741848	120.26059800	https://www.inaturalist.org/observations/40620762
1572	Lanius cristatus	Brown Shrike	2022/3/13	1	0	23.61755407	120.26077090	https://www.inaturalist.org/observations/118892556
1573	Lanius cristatus	Brown Shrike	2022/3/13	2	0	23.61767203	120.26081380	https://www.inaturalist.org/observations/118892555
1574	Lanius cristatus	Brown Shrike	2022/3/6	1	0	23.61776166	120.26073960	https://www.inaturalist.org/observations/118892552
1575	Lanius cristatus	Brown Shrike	2022/3/2	1	0	23.61778363	120.26052820	https://www.inaturalist.org/observations/118892549
1576	Lanius cristatus	Brown Shrike	2022/3/14	1	0	23.61780965	120.26062070	https://www.inaturalist.org/observations/118892557
1577	Lanius cristatus	Brown Shrike	2022/3/6	1	0	23.61793860	120.26073960	https://www.inaturalist.org/observations/118892551
1578	Lanius cristatus	Brown Shrike	2022/3/30	1	0	23.61801956	120.26085010	https://www.inaturalist.org/observations/118892548
1579	Lanius cristatus	Brown Shrike	2002/3/27	2	0	23.61989424	120.26212590	https://www.inaturalist.org/observations/13942558
1580	Lanius cristatus	Brown Shrike	2021/3/24	2	0	23.61998174	120.26145080	https://www.inaturalist.org/observations/71934993
1581	Lanius cristatus	Brown Shrike	2013/1/29	3	0	23.62002270	120.26090940	https://www.inaturalist.org/observations/13203109
1582	Lanius cristatus	Brown Shrike	2021/9/22	1	0	23.62003559	120.26124010	https://www.inaturalist.org/observations/95723167
1583	Lanius cristatus	Brown Shrike	2020/3/20	1	0	23.62009755	120.26141430	https://www.inaturalist.org/observations/40620780
1584	Lanius cristatus	Brown Shrike	2020/4/14	2	0	23.62010167	120.26128830	https://www.inaturalist.org/observations/42155856
1585	Lanius cristatus	Brown Shrike	2020/3/12	2	0	23.62012273	120.26152380	https://www.inaturalist.org/observations/40119291
1586	Lanius cristatus	Brown Shrike	2021/9/14	1	0	23.62015267	120.26135360	https://www.inaturalist.org/observations/94783575

1587	Lanius cristatus	Brown Shrike	2021/3/24	2	0	23.62015716	120.26151520	https://www.inaturalist.org/observations/71934980
1588	Lanius cristatus	Brown Shrike	2021/2/23	2	0	23.62015853	120.26094000	https://www.inaturalist.org/observations/70026074
1589	Lanius cristatus	Brown Shrike	2020/4/28	3	0	23.62023833	120.26156330	https://www.inaturalist.org/observations/44289428
1590	Lanius cristatus	Brown Shrike	2019/5/10	2	0	23.62026752	120.26147520	https://www.inaturalist.org/observations/25051164
1591	Lanius cristatus	Brown Shrike	2022/5/2	0	0	23.62032518	120.26193980	https://www.inaturalist.org/observations/114494806
1592	Lanius cristatus	Brown Shrike	2019/10/4	3	0	23.62039797	120.26144640	https://www.inaturalist.org/observations/34105911
1593	Lanius cristatus	Brown Shrike	2021/5/4	3	0	23.62048667	120.26168330	https://www.inaturalist.org/observations/78402315
1594	Lanius cristatus	Brown Shrike	2020/5/8	2	0	23.62048970	120.26138390	https://www.inaturalist.org/observations/45247367
1595	Lanius cristatus	Brown Shrike	2021/4/20	1	0	23.62058603	120.26133790	https://www.inaturalist.org/observations/74594975
1596	Lanius cristatus	Brown Shrike	2021/1/21	1	0	23.62111350	120.26834090	https://www.inaturalist.org/observations/68403685
1597	Lanius cristatus	Brown Shrike	2020/1/15	2	0	23.62133047	120.23661030	https://www.inaturalist.org/observations/37772514
1598	Lanius cristatus	Brown Shrike	2021/3/28	1	0	23.62453842	120.26551820	https://www.inaturalist.org/observations/72237912
1599	Lanius cristatus	Brown Shrike	2020/2/12	2	0	23.63183333	120.15749170	https://www.inaturalist.org/observations/38625855
1600	Lanius cristatus	Brown Shrike	2022/3/26	1	0	23.63212639	120.28139310	https://www.inaturalist.org/observations/109518114
1601	Lanius cristatus	Brown Shrike	2022/4/1	2	0	23.63359172	120.48439640	https://www.inaturalist.org/observations/110073819
1602	Lanius cristatus	Brown Shrike	2020/2/25	2	0	23.63359500	120.22406000	https://www.inaturalist.org/observations/39198211
1603	Lanius cristatus	Brown Shrike	2021/2/2	3	0	23.63376500	120.28137670	https://www.inaturalist.org/observations/68974239
1604	Lanius cristatus	Brown Shrike	2021/4/20	1	0	23.63406999	120.52379910	https://www.inaturalist.org/observations/74605478
1605	Lanius cristatus	Brown Shrike	2020/2/8	4	0	23.63548873	120.49210010	https://www.inaturalist.org/observations/38536411
1606	Lanius cristatus	Brown Shrike	2021/1/5	2	0	23.64216167	120.48285170	https://www.inaturalist.org/observations/67617525
1607	Lanius cristatus	Brown Shrike	2022/10/14	1	0	23.64875579	121.41987060	https://www.inaturalist.org/observations/143991527
1608	Lanius cristatus	Brown Shrike	2022/3/26	2	0	23.65197500	120.30946000	https://www.inaturalist.org/observations/109518100
1609	Lanius cristatus	Brown Shrike	2022/2/9	1	0	23.65227500	120.30872310	https://www.inaturalist.org/observations/107169341

1610	Lanius cristatus	Brown Shrike	2021/1/28	3	0	23.65259532	120.30860400	https://www.inaturalist.org/observations/68704765
1611	Lanius cristatus	Brown Shrike	2021/4/19	2	0	23.65294937	120.30981100	https://www.inaturalist.org/observations/74475574
1612	Lanius cristatus	Brown Shrike	2020/2/18	2	0	23.65359167	120.23147000	https://www.inaturalist.org/observations/38816323
1613	Lanius cristatus	Brown Shrike	2018/10/31	1	0	23.65468025	121.41748050	https://www.inaturalist.org/observations/17979105
1614	Lanius cristatus	Brown Shrike	2021/9/27	1	0	23.66107167	120.50340170	https://www.inaturalist.org/observations/96464019
1615	Lanius cristatus	Brown Shrike	2019/12/3	2	0	23.66367113	120.48499120	https://www.inaturalist.org/observations/36418536
1616	Lanius cristatus	Brown Shrike	2020/2/19	2	0	23.66539333	120.29272830	https://www.inaturalist.org/observations/38817300
1617	Lanius cristatus	Brown Shrike	2022/9/11	3	0	23.66754588	69.08645187	https://www.inaturalist.org/observations/142267405
1618	Lanius cristatus	Brown Shrike	2015/4/10	4	0	23.66842411	81.03790283	https://www.inaturalist.org/observations/7613925
1619	Lanius cristatus	Brown Shrike	2013/5/4	2	0	23.67013402	120.28454360	https://www.inaturalist.org/observations/41217641
1620	Lanius cristatus	Brown Shrike	2020/2/14	2	0	23.67648667	120.47291330	https://www.inaturalist.org/observations/38655283
1621	Lanius cristatus	Brown Shrike	2020/3/3	2	0	23.67699833	120.47105670	https://www.inaturalist.org/observations/39485522
1622	Lanius cristatus	Brown Shrike	2022/4/30	2	0	23.67892606	120.55758850	https://www.inaturalist.org/observations/114923097
1623	Lanius cristatus	Brown Shrike	2021/11/10	1	0	23.67892930	120.55668160	https://www.inaturalist.org/observations/100815266
1624	Lanius cristatus	Brown Shrike	2022/5/1	1	0	23.67897537	120.55565670	https://www.inaturalist.org/observations/114964021
1625	Lanius cristatus	Brown Shrike	2022/5/2	1	0	23.67899502	120.55699780	https://www.inaturalist.org/observations/114964022
1626	Lanius cristatus	Brown Shrike	2022/4/30	1	0	23.67899502	120.56078510	https://www.inaturalist.org/observations/114964028
1627	Lanius cristatus	Brown Shrike	2021/1/30	2	0	23.67912268	120.31215350	https://www.inaturalist.org/observations/68806612
1628	Lanius cristatus	Brown Shrike	2019/10/23	2	0	23.67916696	120.55645060	https://www.inaturalist.org/observations/35662185
1629	Lanius cristatus	Brown Shrike	2019/10/23	2	0	23.67923236	120.55645570	https://www.inaturalist.org/observations/35532549
1630	Lanius cristatus	Brown Shrike	2018/11/22	2	0	23.67967451	120.56092360	https://www.inaturalist.org/observations/18858582
1631	Lanius cristatus	Brown Shrike	2019/11/1	4	0	23.67971412	120.55706150	https://www.inaturalist.org/observations/35161730
1632	Lanius cristatus	Brown Shrike	2019/9/23	4	0	23.67974084	120.55472500	https://www.inaturalist.org/observations/35232886

1633	Lanius cristatus	Brown Shrike	2014/12/5	3	0	23.67975700	120.55720500	https://www.inaturalist.org/observations/16710302
1634	Lanius cristatus	Brown Shrike	2019/11/8	6	0	23.67977308	120.55716310	https://www.inaturalist.org/observations/35563899
1635	Lanius cristatus	Brown Shrike	2014/10/31	2	0	23.67980900	120.55780800	https://www.inaturalist.org/observations/16709963
1636	Lanius cristatus	Brown Shrike	2020/11/20	4	0	23.67985291	120.55314110	https://www.inaturalist.org/observations/65257907
1637	Lanius cristatus	Brown Shrike	2022/4/30	1	0	23.67985291	120.55314110	https://www.inaturalist.org/observations/113872172
1638	Lanius cristatus	Brown Shrike	2022/4/30	2	0	23.67999061	120.55548990	https://www.inaturalist.org/observations/114923086
1639	Lanius cristatus	Brown Shrike	2020/10/12	2	0	23.68009351	120.55704950	https://www.inaturalist.org/observations/68158124
1640	Lanius cristatus	Brown Shrike	2022/5/2	1	0	23.68015443	120.55699780	https://www.inaturalist.org/observations/114964023
1641	Lanius cristatus	Brown Shrike	2022/4/29	2	0	23.68016507	120.55720440	https://www.inaturalist.org/observations/114923095
1642	Lanius cristatus	Brown Shrike	2020/12/19	3	0	23.68027440	120.47871200	https://www.inaturalist.org/observations/66760050
1643	Lanius cristatus	Brown Shrike	2020/1/19	4	0	23.68029069	120.32751480	https://www.inaturalist.org/observations/40169611
1644	Lanius cristatus	Brown Shrike	2013/12/6	2	0	23.68032049	120.55703120	https://www.inaturalist.org/observations/16787100
1645	Lanius cristatus	Brown Shrike	2022/1/29	2	0	23.68035415	120.55626230	https://www.inaturalist.org/observations/114923067
1646	Lanius cristatus	Brown Shrike	2014/1/9	2	0	23.68039535	120.55704730	https://www.inaturalist.org/observations/16787099
1647	Lanius cristatus	Brown Shrike	2022/5/2	1	0	23.68040801	120.55672510	https://www.inaturalist.org/observations/114964014
1648	Lanius cristatus	Brown Shrike	2014/1/22	2	0	23.68054970	120.55669980	https://www.inaturalist.org/observations/16787106
1649	Lanius cristatus	Brown Shrike	2014/1/20	2	0	23.68103014	120.55658060	https://www.inaturalist.org/observations/16787102
1650	Lanius cristatus	Brown Shrike	2009/2/8	4	0	23.68162954	81.02863431	https://www.inaturalist.org/observations/7458770
1651	Lanius cristatus	Brown Shrike	2020/11/20	3	0	23.68164329	120.55880730	https://www.inaturalist.org/observations/65274033
1652	Lanius cristatus	Brown Shrike	2020/4/20	2	0	23.68403633	120.24531860	https://www.inaturalist.org/observations/42665636
1653	Lanius cristatus	Brown Shrike	2015/2/24	2	0	23.68555030	81.02581680	https://www.inaturalist.org/observations/69242815
1654	Lanius cristatus	Brown Shrike	2021/11/10	1	0	23.68577980	120.62077280	https://www.inaturalist.org/observations/102745329
1655	Lanius cristatus	Brown Shrike	2021/11/11	1	0	23.68577980	120.62077280	https://www.inaturalist.org/observations/102745361

1656	Lanius cristatus	Brown Shrike	2021/11/12	1	0	23.68577980	120.62077280	https://www.inaturalist.org/observations/102745368
1657	Lanius cristatus	Brown Shrike	2021/9/9	1	0	23.68608480	120.62079450	https://www.inaturalist.org/observations/94299727
1658	Lanius cristatus	Brown Shrike	2020/3/3	2	0	23.68710667	120.53515830	https://www.inaturalist.org/observations/39517445
1659	Lanius cristatus	Brown Shrike	2021/2/22	2	0	23.68731032	120.54220620	https://www.inaturalist.org/observations/70025437
1660	Lanius cristatus	Brown Shrike	2019/5/14	1	0	23.69081328	120.53410770	https://www.inaturalist.org/observations/25101611
1661	Lanius cristatus	Brown Shrike	2020/2/12	2	0	23.69098368	120.31327320	https://www.inaturalist.org/observations/38559611
1662	Lanius cristatus	Brown Shrike	2019/10/27	1	0	23.69290907	120.68457260	https://www.inaturalist.org/observations/34955588
1663	Lanius cristatus	Brown Shrike	2019/10/27	1	0	23.69342894	120.68461730	https://www.inaturalist.org/observations/34955102
1664	Lanius cristatus	Brown Shrike	2022/1/1	1	0	23.69432306	120.31117970	https://www.inaturalist.org/observations/104341053
1665	Lanius cristatus	Brown Shrike	2018/12/12	2	0	23.69677285	120.53229560	https://www.inaturalist.org/observations/18978975
1666	Lanius cristatus	Brown Shrike	2019/4/30	2	0	23.69692022	120.53212400	https://www.inaturalist.org/observations/24177925
1667	Lanius cristatus	Brown Shrike	2020/3/12	2	0	23.70446932	120.46257730	https://www.inaturalist.org/observations/39875013
1668	Lanius cristatus	Brown Shrike	2021/9/11	1	0	23.70566811	120.51731490	https://www.inaturalist.org/observations/94401673
1669	Lanius cristatus	Brown Shrike	2021/9/27	1	0	23.70598500	120.51699670	https://www.inaturalist.org/observations/96373404
1670	Lanius cristatus	Brown Shrike	2018/1/21	1	0	23.70658224	120.53908170	https://www.inaturalist.org/observations/18926001
1671	Lanius cristatus	Brown Shrike	2021/12/24	2	0	23.71559929	120.43425080	https://www.inaturalist.org/observations/103562713
1672	Lanius cristatus	Brown Shrike	2020/3/15	2	0	23.71620684	120.43247060	https://www.inaturalist.org/observations/40009177
1673	Lanius cristatus	Brown Shrike	2021/12/25	1	0	23.71681139	120.40693640	https://www.inaturalist.org/observations/103626678
1674	Lanius cristatus	Brown Shrike	2020/5/9	2	0	23.71735833	120.36997330	https://www.inaturalist.org/observations/45345234
1675	Lanius cristatus	Brown Shrike	2019/12/29	2	0	23.72262314	81.02188697	https://www.inaturalist.org/observations/56496176
1676	Lanius cristatus	Brown Shrike	2018/1/4	4	0	23.72412559	120.63731700	https://www.inaturalist.org/observations/13145091
1677	Lanius cristatus	Brown Shrike	2021/9/18	2	0	23.72460139	120.42389140	https://www.inaturalist.org/observations/95249458
1678	Lanius cristatus	Brown Shrike	2021/12/26	3	0	23.72469000	120.42442970	https://www.inaturalist.org/observations/103669907

1679	Lanius cristatus	Brown Shrike	2022/3/5	1	0	23.72480306	120.42251640	https://www.inaturalist.org/observations/107919228
1680	Lanius cristatus	Brown Shrike	2021/12/11	2	0	23.72503639	120.42295000	https://www.inaturalist.org/observations/102920438
1681	Lanius cristatus	Brown Shrike	2022/4/4	1	0	23.72505472	120.42317640	https://www.inaturalist.org/observations/110356155
1682	Lanius cristatus	Brown Shrike	2022/2/27	1	0	23.72520639	120.42230310	https://www.inaturalist.org/observations/107534164
1683	Lanius cristatus	Brown Shrike	2022/9/25	1	0	23.72532139	120.42340140	https://www.inaturalist.org/observations/136409764
1684	Lanius cristatus	Brown Shrike	2021/3/14	2	0	23.72537086	120.42170220	https://www.inaturalist.org/observations/71178408
1685	Lanius cristatus	Brown Shrike	2018/1/4	4	0	23.72555340	120.62583050	https://www.inaturalist.org/observations/13145093
1686	Lanius cristatus	Brown Shrike	2018/1/11	2	0	23.72578418	120.62306900	https://www.inaturalist.org/observations/19485204
1687	Lanius cristatus	Brown Shrike	2014/5/7	2	0	23.72620080	120.62047290	https://www.inaturalist.org/observations/19485353
1688	Lanius cristatus	Brown Shrike	2018/1/6	2	0	23.72643333	120.42554830	https://www.inaturalist.org/observations/16584118
1689	Lanius cristatus	Brown Shrike	2021/12/19	1	0	23.72651639	120.42322000	https://www.inaturalist.org/observations/103291032
1690	Lanius cristatus	Brown Shrike	2020/12/20	3	0	23.72684863	120.42208660	https://www.inaturalist.org/observations/66818272
1691	Lanius cristatus	Brown Shrike	2022/11/25	1	0	23.72737497	120.42407730	https://www.inaturalist.org/observations/142798421
1692	Lanius cristatus	Brown Shrike	2019/12/11	2	0	23.72784999	120.42393210	https://www.inaturalist.org/observations/36550394
1693	Lanius cristatus	Brown Shrike	2019/1/25	2	0	23.72895388	120.36396120	https://www.inaturalist.org/observations/19872273
1694	Lanius cristatus	Brown Shrike	2022/2/17	1	0	23.72925498	120.42220460	https://www.inaturalist.org/observations/107239281
1695	Lanius cristatus	Brown Shrike	2021/11/25	2	0	23.73143650	120.58430760	https://www.inaturalist.org/observations/126223377
1696	Lanius cristatus	Brown Shrike	2019/11/29	2	0	23.73190330	120.53360220	https://www.inaturalist.org/observations/36938308
1697	Lanius cristatus	Brown Shrike	2021/9/12	1	0	23.73273972	120.39588140	https://www.inaturalist.org/observations/94528459
1698	Lanius cristatus	Brown Shrike	2022/9/4	1	0	23.73304639	120.39731640	https://www.inaturalist.org/observations/133564171
1699	Lanius cristatus	Brown Shrike	2022/12/20	2	0	23.73336310	120.39584710	https://www.inaturalist.org/observations/144724163
1700	Lanius cristatus	Brown Shrike	2021/10/16	2	0	23.73352658	120.78701630	https://www.inaturalist.org/observations/102274622
1701	Lanius cristatus	Brown Shrike	2019/1/30	2	0	23.73580181	120.39156880	https://www.inaturalist.org/observations/19982897

1702	Lanius cristatus	Brown Shrike	2021/12/31	1	0	23.73681639	120.39588810	https://www.inaturalist.org/observations/104016444
1703	Lanius cristatus	Brown Shrike	2022/4/17	1	0	23.73845833	121.45428000	https://www.inaturalist.org/observations/120114168
1704	Lanius cristatus	Brown Shrike	2021/10/10	1	0	23.74180451	120.44944030	https://www.inaturalist.org/observations/97721558
1705	Lanius cristatus	Brown Shrike	2019/8/8	2	0	23.74409888	120.27085150	https://www.inaturalist.org/observations/34288695
1706	Lanius cristatus	Brown Shrike	2021/10/14	1	0	23.74536167	120.62553000	https://www.inaturalist.org/observations/98163558
1707	Lanius cristatus	Brown Shrike	2022/5/2	1	0	23.75018197	120.63809100	https://www.inaturalist.org/observations/114633672
1708	Lanius cristatus	Brown Shrike	2019/2/2	2	0	23.75340684	120.62122160	https://www.inaturalist.org/observations/20117838
1709	Lanius cristatus	Brown Shrike	2022/12/4	1	0	23.75678382	120.21304320	https://www.inaturalist.org/observations/144396817
1710	Lanius cristatus	Brown Shrike	2018/12/30	1	0	23.75692029	121.47659080	https://www.inaturalist.org/observations/109835153
1711	Lanius cristatus	Brown Shrike	2020/2/3	2	0	23.75819522	120.61182310	https://www.inaturalist.org/observations/67037414
1712	Lanius cristatus	Brown Shrike	2022/9/14	1	0	23.76726296	120.45859140	https://www.inaturalist.org/observations/134924775
1713	Lanius cristatus	Brown Shrike	2020/12/27	3	0	23.77652804	120.34889480	https://www.inaturalist.org/observations/67517059
1714	Lanius cristatus	Brown Shrike	2022/1/14	2	0	23.78298577	120.61594380	https://www.inaturalist.org/observations/105264896
1715	Lanius cristatus	Brown Shrike	2021/3/27	3	0	23.79057469	120.31146380	https://www.inaturalist.org/observations/72158833
1716	Lanius cristatus	Brown Shrike	2022/1/23	1	0	23.79335123	120.42449810	https://www.inaturalist.org/observations/105555942
1717	Lanius cristatus	Brown Shrike	2016/4/23	4	0	23.79487999	120.47004110	https://www.inaturalist.org/observations/30582030
1718	Lanius cristatus	Brown Shrike	2021/10/11	2	0	23.80264551	120.22774730	https://www.inaturalist.org/observations/98658711
1719	Lanius cristatus	Brown Shrike	2021/1/1	2	0	23.80522167	120.36448830	https://www.inaturalist.org/observations/67768317
1720	Lanius cristatus	Brown Shrike	2017/5/2	2	0	23.80925439	120.27603560	https://www.inaturalist.org/observations/18516960
1721	Lanius cristatus	Brown Shrike	2018/10/21	3	0	23.81284764	120.68031660	https://www.inaturalist.org/observations/19796830
1722	Lanius cristatus	Brown Shrike	2019/1/15	1	0	23.82717563	120.80123440	https://www.inaturalist.org/observations/141034116
1723	Lanius cristatus	Brown Shrike	2017/3/11	2	0	23.83021408	120.52804920	https://www.inaturalist.org/observations/13519103
1724	Lanius cristatus lucionensis	Brown Shrike	2021/9/18	1	0	23.83287003	120.78889470	https://www.inaturalist.org/observations/95632289

1725	Lanius cristatus	Brown Shrike	2021/11/24	2	0	23.83720583	121.47504180	https://www.inaturalist.org/observations/101823094
1726	Lanius cristatus	Brown Shrike	2021/9/19	1	0	23.83865333	121.51096330	https://www.inaturalist.org/observations/96057254
1727	Lanius cristatus	Brown Shrike	2022/1/21	2	0	23.84026105	121.50330250	https://www.inaturalist.org/observations/105310077
1728	Lanius cristatus	Brown Shrike	2020/2/5	3	0	23.84133210	120.49830440	https://www.inaturalist.org/observations/38332945
1729	Lanius cristatus	Brown Shrike	2018/1/6	2	0	23.84232775	120.69159170	https://www.inaturalist.org/observations/19796829
1730	Lanius cristatus	Brown Shrike	2021/1/25	2	0	23.84409790	121.51629670	https://www.inaturalist.org/observations/69851799
1731	Lanius cristatus	Brown Shrike	2022/3/4	1	0	23.84671002	121.51213850	https://www.inaturalist.org/observations/115489287
1732	Lanius cristatus	Brown Shrike	2021/10/1	1	0	23.85484286	121.51700690	https://www.inaturalist.org/observations/96990395
1733	Lanius cristatus	Brown Shrike	2022/1/14	2	0	23.85881671	120.61189840	https://www.inaturalist.org/observations/105264860
1734	Lanius cristatus	Brown Shrike	2022/1/10	2	0	23.86553768	121.52296480	https://www.inaturalist.org/observations/104760030
1735	Lanius cristatus	Brown Shrike	2021/10/7	3	0	23.86584284	121.52474110	https://www.inaturalist.org/observations/97625225
1736	Lanius cristatus	Brown Shrike	2021/12/8	1	0	23.87208712	121.52359560	https://www.inaturalist.org/observations/106916718
1737	Lanius cristatus	Brown Shrike	2021/9/22	2	0	23.87242008	121.55861130	https://www.inaturalist.org/observations/95726230
1738	Lanius cristatus	Brown Shrike	2021/9/22	2	0	23.87352996	121.56024640	https://www.inaturalist.org/observations/95726231
1739	Lanius cristatus	Brown Shrike	2018/2/24	2	0	23.87433753	120.58006550	https://www.inaturalist.org/observations/62010445
1740	Lanius cristatus	Brown Shrike	2019/10/25	2	0	23.87454739	121.50920370	https://www.inaturalist.org/observations/34906306
1741	Lanius cristatus	Brown Shrike	2021/10/7	3	0	23.87968249	121.56584140	https://www.inaturalist.org/observations/97625214
1742	Lanius cristatus	Brown Shrike	2022/12/4	1	0	23.88486167	121.51892670	https://www.inaturalist.org/observations/143794207
1743	Lanius cristatus	Brown Shrike	2020/5/5	3	0	23.88869395	121.53597670	https://www.inaturalist.org/observations/46468774
1744	Lanius cristatus	Brown Shrike	2021/3/12	2	0	23.88898400	121.53521500	https://www.inaturalist.org/observations/71050615
1745	Lanius cristatus	Brown Shrike	2020/12/31	2	0	23.88904457	121.53659190	https://www.inaturalist.org/observations/69850288
1746	Lanius cristatus	Brown Shrike	2020/1/8	2	0	23.89020900	121.53766600	https://www.inaturalist.org/observations/37395076
1747	Lanius cristatus	Brown Shrike	2020/1/8	2	0	23.89097200	121.53473900	https://www.inaturalist.org/observations/37395079

1748	Lanius cristatus	Brown Shrike	2021/11/15	1	0	23.89148971	121.50692220	https://www.inaturalist.org/observations/103297250
1749	Lanius cristatus	Brown Shrike	2021/10/23	2	0	23.89171245	121.53846390	https://www.inaturalist.org/observations/99045210
1750	Lanius cristatus	Brown Shrike	2019/9/26	2	0	23.89194672	121.54076380	https://www.inaturalist.org/observations/33700774
1751	Lanius cristatus	Brown Shrike	2020/10/28	1	0	23.89232151	121.54116840	https://www.inaturalist.org/observations/67299897
1752	Lanius cristatus	Brown Shrike	2020/4/24	2	0	23.89319197	121.54485910	https://www.inaturalist.org/observations/43025284
1753	Lanius cristatus	Brown Shrike	2020/4/24	4	0	23.89343704	121.54564280	https://www.inaturalist.org/observations/47111659
1754	Lanius cristatus	Brown Shrike	2020/10/28	2	0	23.89348727	121.54164530	https://www.inaturalist.org/observations/63677510
1755	Lanius cristatus	Brown Shrike	2020/4/14	2	0	23.89394846	121.54902430	https://www.inaturalist.org/observations/44592630
1756	Lanius cristatus	Brown Shrike	2019/10/1	2	0	23.89417766	121.53832390	https://www.inaturalist.org/observations/33692781
1757	Lanius cristatus	Brown Shrike	2021/11/6	2	0	23.89452161	121.54996620	https://www.inaturalist.org/observations/100374360
1758	Lanius cristatus	Brown Shrike	2021/10/7	1	0	23.89455141	121.53696130	https://www.inaturalist.org/observations/100404911
1759	Lanius cristatus	Brown Shrike	2019/10/1	3	0	23.89535232	121.54838450	https://www.inaturalist.org/observations/33693250
1760	Lanius cristatus	Brown Shrike	2020/10/27	1	0	23.89601425	121.55033370	https://www.inaturalist.org/observations/63638752
1761	Lanius cristatus	Brown Shrike	2018/1/18	2	0	23.89639769	121.55110140	https://www.inaturalist.org/observations/68204379
1762	Lanius cristatus	Brown Shrike	2020/10/28	2	0	23.89641000	121.55090000	https://www.inaturalist.org/observations/63675684
1763	Lanius cristatus	Brown Shrike	2020/10/28	3	0	23.89641833	121.55088500	https://www.inaturalist.org/observations/63675686
1764	Lanius cristatus	Brown Shrike	2020/3/17	4	0	23.89646853	121.55072150	https://www.inaturalist.org/observations/40936203
1765	Lanius cristatus	Brown Shrike	2019/2/23	2	0	23.89675273	121.54484640	https://www.inaturalist.org/observations/21284194
1766	Lanius cristatus	Brown Shrike	2021/11/6	1	0	23.89705618	121.54476090	https://www.inaturalist.org/observations/100374359
1767	Lanius cristatus	Brown Shrike	2011/2/6	2	0	23.89753850	121.54180180	https://www.inaturalist.org/observations/55762465
1768	Lanius cristatus	Brown Shrike	2020/3/18	4	0	23.89756605	121.53560570	https://www.inaturalist.org/observations/47109114
1769	Lanius cristatus	Brown Shrike	2019/12/25	3	0	23.89847000	121.54413500	https://www.inaturalist.org/observations/36938595
1770	Lanius cristatus	Brown Shrike	2019/12/25	3	0	23.89855300	121.54427300	https://www.inaturalist.org/observations/36931991

1771	Lanius cristatus	Brown Shrike	2014/1/14	2	0	23.90019868	121.54642580	https://www.inaturalist.org/observations/12264597
1772	Lanius cristatus	Brown Shrike	2020/11/2	2	0	23.90025692	121.54969170	https://www.inaturalist.org/observations/64028628
1773	Lanius cristatus	Brown Shrike	2021/1/11	3	0	23.90043501	121.54947410	https://www.inaturalist.org/observations/67890143
1774	Lanius cristatus	Brown Shrike	2019/10/1	3	0	23.90055120	121.54473990	https://www.inaturalist.org/observations/33692771
1775	Lanius cristatus	Brown Shrike	2020/9/28	2	0	23.90065333	121.53740830	https://www.inaturalist.org/observations/61108742
1776	Lanius cristatus	Brown Shrike	2021/3/14	2	0	23.90076300	121.54849700	https://www.inaturalist.org/observations/71196667
1777	Lanius cristatus	Brown Shrike	2019/10/13	3	0	23.90080500	121.54223600	https://www.inaturalist.org/observations/34365660
1778	Lanius cristatus	Brown Shrike	2020/5/1	4	0	23.90085500	121.54378000	https://www.inaturalist.org/observations/44445711
1779	Lanius cristatus	Brown Shrike	2019/10/13	3	0	23.90102700	121.54216810	https://www.inaturalist.org/observations/34300054
1780	Lanius cristatus	Brown Shrike	2022/4/2	1	0	23.90121693	121.54560580	https://www.inaturalist.org/observations/119842721
1781	Lanius cristatus	Brown Shrike	2020/4/25	3	0	23.90126167	121.54610170	https://www.inaturalist.org/observations/43288695
1782	Lanius cristatus	Brown Shrike	2020/4/14	4	0	23.90129500	121.54774330	https://www.inaturalist.org/observations/42749781
1783	Lanius cristatus	Brown Shrike	2019/9/27	3	0	23.90145636	121.54327700	https://www.inaturalist.org/observations/33470654
1784	Lanius cristatus	Brown Shrike	2020/4/14	3	0	23.90153029	121.54784350	https://www.inaturalist.org/observations/42158203
1785	Lanius cristatus	Brown Shrike	2020/1/2	2	0	23.90173227	121.54537000	https://www.inaturalist.org/observations/42087398
1786	Lanius cristatus	Brown Shrike	2019/11/2	1	0	23.90180300	121.53898300	https://www.inaturalist.org/observations/35195393
1787	Lanius cristatus	Brown Shrike	2020/3/6	2	0	23.90182324	121.54363070	https://www.inaturalist.org/observations/44592621
1788	Lanius cristatus	Brown Shrike	2020/3/2	6	0	23.90279463	121.54786920	https://www.inaturalist.org/observations/39913698
1789	Lanius cristatus	Brown Shrike	2022/1/16	1	0	23.90400354	120.68275990	https://www.inaturalist.org/observations/105029529
1790	Lanius cristatus	Brown Shrike	2021/11/18	1	0	23.90609269	121.53758640	https://www.inaturalist.org/observations/101393988
1791	Lanius cristatus	Brown Shrike	2020/11/5	3	0	23.91232817	120.68122880	https://www.inaturalist.org/observations/66825010
1792	Lanius cristatus	Brown Shrike	2018/12/18	2	0	23.91509972	121.51384320	https://www.inaturalist.org/observations/34317897
1793	Lanius cristatus	Brown Shrike	2021/11/18	1	0	23.91560777	121.55859680	https://www.inaturalist.org/observations/101596501

1794	Lanius cristatus	Brown Shrike	2021/12/21	2	0	23.91593725	121.56295640	https://www.inaturalist.org/observations/103416930
1795	Lanius cristatus	Brown Shrike	2022/4/9	1	0	23.93607500	121.60174000	https://www.inaturalist.org/observations/119967843
1796	Lanius cristatus	Brown Shrike	2022/11/19	1	0	23.93638298	121.59902490	https://www.inaturalist.org/observations/142738808
1797	Lanius cristatus	Brown Shrike	2018/11/3	2	0	23.93666153	120.63010870	https://www.inaturalist.org/observations/19649517
1798	Lanius cristatus	Brown Shrike	2019/10/11	1	0	23.93742900	121.60098600	https://www.inaturalist.org/observations/34200255
1799	Lanius cristatus	Brown Shrike	2022/4/4	1	0	23.93764091	121.60115790	https://www.inaturalist.org/observations/119397359
1800	Lanius cristatus	Brown Shrike	2022/2/6	2	0	23.93882700	87.52484100	https://www.inaturalist.org/observations/106294653
1801	Lanius cristatus	Brown Shrike	2021/10/2	1	0	23.94828885	121.52341980	https://www.inaturalist.org/observations/96991484
1802	Lanius cristatus	Brown Shrike	2019/3/8	2	0	23.95582833	120.68718000	https://www.inaturalist.org/observations/21046148
1803	Lanius cristatus	Brown Shrike	2020/10/26	3	0	23.96513710	120.96737680	https://www.inaturalist.org/observations/63557662
1804	Lanius cristatus	Brown Shrike	2020/11/3	2	0	23.96513710	120.96737680	https://www.inaturalist.org/observations/64098659
1805	Lanius cristatus	Brown Shrike	2020/11/13	2	0	23.96513710	120.96737680	https://www.inaturalist.org/observations/65005460
1806	Lanius cristatus	Brown Shrike	2020/11/19	2	0	23.96513710	120.96737680	https://www.inaturalist.org/observations/65207111
1807	Lanius cristatus	Brown Shrike	2020/11/21	2	0	23.96513710	120.96737680	https://www.inaturalist.org/observations/65321826
1808	Lanius cristatus	Brown Shrike	2020/11/25	2	0	23.96513710	120.96737680	https://www.inaturalist.org/observations/65581531
1809	Lanius cristatus	Brown Shrike	2020/11/28	3	0	23.96513710	120.96737680	https://www.inaturalist.org/observations/65804933
1810	Lanius cristatus	Brown Shrike	2020/12/4	2	0	23.96513710	120.96737680	https://www.inaturalist.org/observations/66285044
1811	Lanius cristatus	Brown Shrike	2020/12/7	2	0	23.96513710	120.96737680	https://www.inaturalist.org/observations/66285055
1812	Lanius cristatus	Brown Shrike	2020/12/8	2	0	23.96513710	120.96737680	https://www.inaturalist.org/observations/66285057
1813	Lanius cristatus	Brown Shrike	2020/12/8	4	0	23.96513710	120.96737680	https://www.inaturalist.org/observations/66285058
1814	Lanius cristatus	Brown Shrike	2021/1/4	2	0	23.96513710	120.96737680	https://www.inaturalist.org/observations/67700392
1815	Lanius cristatus	Brown Shrike	2021/1/5	2	0	23.96513710	120.96737680	https://www.inaturalist.org/observations/67700394
1816	Lanius cristatus	Brown Shrike	2021/1/7	2	0	23.96513710	120.96737680	https://www.inaturalist.org/observations/67700396

1817	Lanius cristatus	Brown Shrike	2021/10/3	2	0	23.96696726	120.96615040	https://www.inaturalist.org/observations/97005172
1818	Lanius cristatus	Brown Shrike	2020/9/12	2	0	23.97225148	121.54529340	https://www.inaturalist.org/observations/59469091
1819	Lanius cristatus	Brown Shrike	2020/4/11	2	0	23.98036042	120.95245470	https://www.inaturalist.org/observations/41901036
1820	Lanius cristatus	Brown Shrike	2022/12/10	2	0	23.98224792	120.95562580	https://www.inaturalist.org/observations/144067585
1821	Lanius cristatus	Brown Shrike	2021/11/10	3	0	23.98243895	121.61633340	https://www.inaturalist.org/observations/100759503
1822	Lanius cristatus	Brown Shrike	2021/11/10	3	0	23.98339958	121.61605080	https://www.inaturalist.org/observations/100759504
1823	Lanius cristatus	Brown Shrike	2019/2/7	2	0	23.98380190	121.59849100	https://www.inaturalist.org/observations/20240473
1824	Lanius cristatus	Brown Shrike	2020/10/24	3	0	23.99560098	121.62872390	https://www.inaturalist.org/observations/66509346
1825	Lanius cristatus	Brown Shrike	2020/2/10	2	0	23.99994001	120.57147240	https://www.inaturalist.org/observations/38503083
1826	Lanius cristatus	Brown Shrike	2019/10/6	3	0	24.00111000	120.53464300	https://www.inaturalist.org/observations/33940159
1827	Lanius cristatus	Brown Shrike	2019/4/9	2	0	24.00156784	120.57415970	https://www.inaturalist.org/observations/22267914
1828	Lanius cristatus	Brown Shrike	2021/1/3	1	0	24.00177821	121.60228760	https://www.inaturalist.org/observations/107177028
1829	Lanius cristatus	Brown Shrike	2018/11/14	2	0	24.00506730	120.56655920	https://www.inaturalist.org/observations/19649522
1830	Lanius cristatus	Brown Shrike	2022/11/7	2	0	24.00555977	120.45498330	https://www.inaturalist.org/observations/141967632
1831	Lanius cristatus	Brown Shrike	2021/3/15	3	0	24.00921333	120.72152000	https://www.inaturalist.org/observations/71349876
1832	Lanius cristatus	Brown Shrike	2020/4/25	2	0	24.01234576	120.47661200	https://www.inaturalist.org/observations/43333488
1833	Lanius cristatus	Brown Shrike	2020/4/25	2	0	24.01451332	120.47308680	https://www.inaturalist.org/observations/43333487
1834	Lanius cristatus	Brown Shrike	2020/1/22	2	0	24.01566865	120.47126590	https://www.inaturalist.org/observations/37843165
1835	Lanius cristatus	Brown Shrike	2022/10/13	3	0	24.01592257	120.48349620	https://www.inaturalist.org/observations/138601500
1836	Lanius cristatus	Brown Shrike	2021/2/23	2	0	24.02067147	120.91295210	https://www.inaturalist.org/observations/105656681
1837	Lanius cristatus	Brown Shrike	2021/10/24	2	0	24.03660171	121.62485570	https://www.inaturalist.org/observations/99207248
1838	Lanius cristatus	Brown Shrike	2022/10/9	1	0	24.04039171	120.38548910	https://www.inaturalist.org/observations/139311915
1839	Lanius cristatus	Brown Shrike	2019/4/11	3	0	24.04251491	120.40168480	https://www.inaturalist.org/observations/22318893

1840	Lanius cristatus	Brown Shrike	2021/1/20	2	0	24.04510749	120.38716880	https://www.inaturalist.org/observations/68368875
1841	Lanius cristatus	Brown Shrike	2022/9/10	2	0	24.04616289	120.45184950	https://www.inaturalist.org/observations/134855913
1842	Lanius cristatus	Brown Shrike	2020/9/17	4	0	24.04877333	120.43910830	https://www.inaturalist.org/observations/59871457
1843	Lanius cristatus	Brown Shrike	2020/10/14	1	0	24.06945566	120.70315530	https://www.inaturalist.org/observations/62527202
1844	Lanius cristatus	Brown Shrike	2015/11/5	1	0	24.08605910	88.24229180	https://www.inaturalist.org/observations/117574689
1845	Lanius cristatus	Brown Shrike	2018/9/19	4	0	24.10574866	120.69046590	https://www.inaturalist.org/observations/16679791
1846	Lanius cristatus	Brown Shrike	2022/3/6	1	0	24.10663199	120.75996510	https://www.inaturalist.org/observations/109397819
1847	Lanius cristatus	Brown Shrike	2020/11/1	3	0	24.10817153	120.65205420	https://www.inaturalist.org/observations/65001593
1848	Lanius cristatus	Brown Shrike	2020/2/21	3	0	24.10925806	120.67229500	https://www.inaturalist.org/observations/39133301
1849	Lanius cristatus	Brown Shrike	2021/1/10	3	0	24.10948000	120.67089810	https://www.inaturalist.org/observations/67864882
1850	Lanius cristatus	Brown Shrike	2020/4/30	2	0	24.11139799	120.49395150	https://www.inaturalist.org/observations/44350152
1851	Lanius cristatus	Brown Shrike	2022/5/3	1	0	24.11246383	120.49366950	https://www.inaturalist.org/observations/114960400
1852	Lanius cristatus	Brown Shrike	2022/4/7	1	0	24.11251069	120.49346940	https://www.inaturalist.org/observations/110636165
1853	Lanius cristatus	Brown Shrike	2021/12/30	2	0	24.11296237	120.49388440	https://www.inaturalist.org/observations/103942708
1854	Lanius cristatus	Brown Shrike	2021/3/18	1	0	24.11313099	120.49380330	https://www.inaturalist.org/observations/71458875
1855	Lanius cristatus	Brown Shrike	2022/9/30	1	0	24.11318630	120.49355280	https://www.inaturalist.org/observations/136987574
1856	Lanius cristatus	Brown Shrike	2021/4/19	0	0	24.11319150	120.49360160	https://www.inaturalist.org/observations/74499004
1857	Lanius cristatus	Brown Shrike	2022/3/1	1	0	24.11319373	120.49363560	https://www.inaturalist.org/observations/107690166
1858	Lanius cristatus	Brown Shrike	2021/3/13	1	0	24.11345471	120.72331380	https://www.inaturalist.org/observations/72255745
1859	Lanius cristatus	Brown Shrike	2020/12/31	2	0	24.11364268	120.66173730	https://www.inaturalist.org/observations/67346246
1860	Lanius cristatus	Brown Shrike	2020/2/21	3	0	24.11448472	120.67149640	https://www.inaturalist.org/observations/39133303
1861	Lanius cristatus	Brown Shrike	2020/12/21	2	0	24.11460049	120.44323460	https://www.inaturalist.org/observations/66856507
1862	Lanius cristatus	Brown Shrike	2021/10/31	2	0	24.11462329	120.44325970	https://www.inaturalist.org/observations/99850287

1863	Lanius cristatus	Brown Shrike	2020/10/1	2	0	24.11471852	120.44319030	https://www.inaturalist.org/observations/61345160
1864	Lanius cristatus	Brown Shrike	2021/11/20	1	0	24.11472840	120.44338560	https://www.inaturalist.org/observations/101496659
1865	Lanius cristatus	Brown Shrike	2020/10/1	3	0	24.11478409	120.44322170	https://www.inaturalist.org/observations/61344692
1866	Lanius cristatus	Brown Shrike	2019/3/19	2	0	24.11541106	120.65947890	https://www.inaturalist.org/observations/21383088
1867	Lanius cristatus	Brown Shrike	2018/12/24	2	0	24.11681940	120.67416470	https://www.inaturalist.org/observations/62626309
1868	Lanius cristatus	Brown Shrike	2020/12/20	2	0	24.11732028	120.67716360	https://www.inaturalist.org/observations/66911332
1869	Lanius cristatus	Brown Shrike	2020/12/20	2	0	24.11735778	120.67720390	https://www.inaturalist.org/observations/66911331
1870	Lanius cristatus	Brown Shrike	2021/1/16	2	0	24.12077145	120.61813090	https://www.inaturalist.org/observations/99542532
1871	Lanius cristatus	Brown Shrike	2019/12/3	2	0	24.12125389	120.67264080	https://www.inaturalist.org/observations/36346094
1872	Lanius cristatus	Brown Shrike	2021/2/16	2	0	24.12195173	120.73025760	https://www.inaturalist.org/observations/70085519
1873	Lanius cristatus	Brown Shrike	2020/2/19	2	0	24.13107866	120.61930150	https://www.inaturalist.org/observations/44750740
1874	Lanius cristatus	Brown Shrike	2019/12/21	2	0	24.13186806	120.61942640	https://www.inaturalist.org/observations/36821160
1875	Lanius cristatus	Brown Shrike	2021/3/7	1	0	24.13424889	120.61934620	https://www.inaturalist.org/observations/95122127
1876	Lanius cristatus	Brown Shrike	2021/12/9	1	0	24.13843706	120.66935400	https://www.inaturalist.org/observations/103144015
1877	Lanius cristatus	Brown Shrike	2022/12/22	1	0	24.14305550	120.68464670	https://www.inaturalist.org/observations/144840406
1878	Lanius cristatus	Brown Shrike	2019/10/10	2	0	24.15351835	121.62416900	https://www.inaturalist.org/observations/34201142
1879	Lanius cristatus	Brown Shrike	2022/12/11	1	0	24.15565811	120.75824520	https://www.inaturalist.org/observations/144781644
1880	Lanius cristatus	Brown Shrike	2015/2/3	1	0	24.15857430	120.66834200	https://www.inaturalist.org/observations/110631024
1881	Lanius cristatus	Brown Shrike	2019/1/5	2	0	24.15874971	120.70448590	https://www.inaturalist.org/observations/19675750
1882	Lanius cristatus	Brown Shrike	2020/12/20	2	0	24.16012389	120.73487680	https://www.inaturalist.org/observations/67501693
1883	Lanius cristatus	Brown Shrike	2019/3/11	3	0	24.16024500	120.51828000	https://www.inaturalist.org/observations/34336931
1884	Lanius cristatus	Brown Shrike	2021/11/20	2	0	24.16491976	120.57114480	https://www.inaturalist.org/observations/101503346
1885	Lanius cristatus	Brown Shrike	2016/3/4	2	0	24.16602440	120.63214030	https://www.inaturalist.org/observations/102470192

1886	Lanius cristatus	Brown Shrike	2018/12/13	2	0	24.16749667	120.63897000	https://www.inaturalist.org/observations/18989960
1887	Lanius cristatus	Brown Shrike	2021/2/3	1	0	24.17063883	121.57260480	https://www.inaturalist.org/observations/79770256
1888	Lanius cristatus	Brown Shrike	2022/9/21	2	0	24.17481019	120.64909290	https://www.inaturalist.org/observations/136667153
1889	Lanius cristatus	Brown Shrike	2019/10/3	3	0	24.17781087	120.59790140	https://www.inaturalist.org/observations/33832968
1890	Lanius cristatus	Brown Shrike	2019/9/15	2	0	24.17850933	120.73808670	https://www.inaturalist.org/observations/33520180
1891	Lanius cristatus	Brown Shrike	2019/10/4	2	0	24.17939105	120.60028030	https://www.inaturalist.org/observations/33833681
1892	Lanius cristatus	Brown Shrike	2019/3/19	1	0	24.17948360	120.60423430	https://www.inaturalist.org/observations/99099306
1893	Lanius cristatus	Brown Shrike	2019/10/4	1	0	24.17951175	120.60024420	https://www.inaturalist.org/observations/33844559
1894	Lanius cristatus	Brown Shrike	2019/3/21	4	0	24.17960362	120.60006700	https://www.inaturalist.org/observations/21445144
1895	Lanius cristatus	Brown Shrike	2020/3/11	2	0	24.17986758	120.60449200	https://www.inaturalist.org/observations/39966547
1896	Lanius cristatus	Brown Shrike	2018/10/31	3	0	24.17989722	120.59876940	https://www.inaturalist.org/observations/19459006
1897	Lanius cristatus	Brown Shrike	2018/10/31	3	0	24.17989722	120.59876940	https://www.inaturalist.org/observations/19459701
1898	Lanius cristatus	Brown Shrike	2018/10/31	4	0	24.17990910	120.59872280	https://www.inaturalist.org/observations/17981127
1899	Lanius cristatus	Brown Shrike	2018/10/31	3	0	24.17991111	120.59876940	https://www.inaturalist.org/observations/19460548
1900	Lanius cristatus	Brown Shrike	2018/10/31	4	0	24.17991111	120.59876940	https://www.inaturalist.org/observations/19460550
1901	Lanius cristatus	Brown Shrike	2019/1/4	3	0	24.18025612	120.59780170	https://www.inaturalist.org/observations/18533613
1902	Lanius cristatus	Brown Shrike	2020/4/6	3	0	24.18042119	120.59872020	https://www.inaturalist.org/observations/41518382
1903	Lanius cristatus	Brown Shrike	2019/12/28	3	0	24.18043110	120.60137360	https://www.inaturalist.org/observations/37023942
1904	Lanius cristatus	Brown Shrike	2020/4/24	3	0	24.18044871	120.59998620	https://www.inaturalist.org/observations/43046718
1905	Lanius cristatus	Brown Shrike	2019/10/26	1	0	24.18053435	120.61082040	https://www.inaturalist.org/observations/34892374
1906	Lanius cristatus	Brown Shrike	2022/12/13	1	0	24.18080759	120.65535580	https://www.inaturalist.org/observations/144287191
1907	Lanius cristatus	Brown Shrike	2022/12/20	1	0	24.18087931	120.65519020	https://www.inaturalist.org/observations/144730556
1908	Lanius cristatus	Brown Shrike	2022/11/1	1	0	24.18090998	120.65526870	https://www.inaturalist.org/observations/140748597

1909	Lanius cristatus	Brown Shrike	2020/11/4	1	0	24.18113073	120.59885080	https://www.inaturalist.org/observations/98811726
1910	Lanius cristatus	Brown Shrike	2022/12/20	1	0	24.18120020	120.65497040	https://www.inaturalist.org/observations/144731556
1911	Lanius cristatus	Brown Shrike	2022/11/1	2	0	24.18210574	120.65581970	https://www.inaturalist.org/observations/140847366
1912	Lanius cristatus	Brown Shrike	2022/12/6	1	0	24.18219929	120.65513510	https://www.inaturalist.org/observations/143787980
1913	Lanius cristatus	Brown Shrike	2022/12/13	1	0	24.18259971	120.65599440	https://www.inaturalist.org/observations/144469978
1914	Lanius cristatus	Brown Shrike	2022/9/20	1	0	24.18421703	120.65533280	https://www.inaturalist.org/observations/135741714
1915	Lanius cristatus	Brown Shrike	2022/12/11	1	0	24.18426105	120.71918570	https://www.inaturalist.org/observations/144781641
1916	Lanius cristatus	Brown Shrike	2022/9/20	3	0	24.18497732	120.65421720	https://www.inaturalist.org/observations/135743669
1917	Lanius cristatus	Brown Shrike	2022/9/20	1	0	24.18510500	120.65391670	https://www.inaturalist.org/observations/135767361
1918	Lanius cristatus	Brown Shrike	2022/10/25	1	0	24.18684913	120.65365750	https://www.inaturalist.org/observations/139943466
1919	Lanius cristatus	Brown Shrike	2022/10/25	1	0	24.18721443	120.65328920	https://www.inaturalist.org/observations/139943485
1920	Lanius cristatus	Brown Shrike	2022/10/25	1	0	24.18850900	120.65318980	https://www.inaturalist.org/observations/139935334
1921	Lanius cristatus	Brown Shrike	2022/10/25	1	0	24.18851604	120.65237690	https://www.inaturalist.org/observations/139943449
1922	Lanius cristatus	Brown Shrike	2022/11/22	1	0	24.18870038	120.65322480	https://www.inaturalist.org/observations/142668652
1923	Lanius cristatus	Brown Shrike	2022/10/25	1	0	24.18953000	120.65431330	https://www.inaturalist.org/observations/139948193
1924	Lanius cristatus	Brown Shrike	2022/12/13	1	0	24.18968050	120.65298250	https://www.inaturalist.org/observations/144287153
1925	Lanius cristatus	Brown Shrike	2020/2/21	2	0	24.18984490	120.71207210	https://www.inaturalist.org/observations/38982318
1926	Lanius cristatus	Brown Shrike	2020/2/21	2	0	24.18984490	120.71207210	https://www.inaturalist.org/observations/38982948
1927	Lanius cristatus	Brown Shrike	2022/10/25	1	0	24.19006333	120.65261330	https://www.inaturalist.org/observations/139948259
1928	Lanius cristatus	Brown Shrike	2022/10/25	1	0	24.19010833	120.65347170	https://www.inaturalist.org/observations/139948300
1929	Lanius cristatus	Brown Shrike	2019/10/6	2	0	24.19480114	120.63564500	https://www.inaturalist.org/observations/35045015
1930	Lanius cristatus	Brown Shrike	2022/2/4	2	0	24.19486793	120.65073900	https://www.inaturalist.org/observations/112404580
1931	Lanius cristatus	Brown Shrike	2022/1/18	1	0	24.20423500	120.59991670	https://www.inaturalist.org/observations/105152578

1932	Lanius cristatus	Brown Shrike	2018/9/10	1	0	24.20424444	120.50547780	https://www.inaturalist.org/observations/16381038
1933	Lanius cristatus	Brown Shrike	2020/3/8	2	0	24.20469988	120.63744420	https://www.inaturalist.org/observations/39700732
1934	Lanius cristatus	Brown Shrike	2021/12/12	1	0	24.20471923	120.51420960	https://www.inaturalist.org/observations/102926208
1935	Lanius cristatus	Brown Shrike	2022/12/25	1	0	24.20684336	120.59713620	https://www.inaturalist.org/observations/145101928
1936	Lanius cristatus	Brown Shrike	2022/12/25	2	0	24.20693026	120.59894370	https://www.inaturalist.org/observations/145107917
1937	Lanius cristatus	Brown Shrike	2022/12/25	1	0	24.20697008	120.59886960	https://www.inaturalist.org/observations/145101701
1938	Lanius cristatus	Brown Shrike	2021/2/6	3	0	24.20721250	120.59751500	https://www.inaturalist.org/observations/69199069
1939	Lanius cristatus	Brown Shrike	2022/12/25	2	0	24.20737926	120.59886820	https://www.inaturalist.org/observations/145107656
1940	Lanius cristatus	Brown Shrike	2021/11/13	1	0	24.20752340	120.59926990	https://www.inaturalist.org/observations/101302883
1941	Lanius cristatus	Brown Shrike	2022/9/18	2	0	24.20756584	120.59728510	https://www.inaturalist.org/observations/135495973
1942	Lanius cristatus	Brown Shrike	2022/2/13	1	0	24.20782780	120.59881610	https://www.inaturalist.org/observations/106673556
1943	Lanius cristatus	Brown Shrike	2021/2/3	2	0	24.20782902	120.59881210	https://www.inaturalist.org/observations/69851819
1944	Lanius cristatus	Brown Shrike	2020/1/4	3	0	24.20783514	120.59881610	https://www.inaturalist.org/observations/37266518
1945	Lanius cristatus	Brown Shrike	2019/9/26	2	0	24.20787580	120.59851520	https://www.inaturalist.org/observations/33520193
1946	Lanius cristatus	Brown Shrike	2022/11/29	3	0	24.20896116	120.59943700	https://www.inaturalist.org/observations/143269068
1947	Lanius cristatus	Brown Shrike	2021/1/29	2	0	24.20922833	120.59824670	https://www.inaturalist.org/observations/68745795
1948	Lanius cristatus	Brown Shrike	2022/11/29	2	0	24.20927595	120.59923400	https://www.inaturalist.org/observations/143306610
1949	Lanius cristatus	Brown Shrike	2019/10/7	2	0	24.21056343	120.59672730	https://www.inaturalist.org/observations/34011430
1950	Lanius cristatus	Brown Shrike	2019/2/11	2	0	24.21386188	120.55844420	https://www.inaturalist.org/observations/20278192
1951	Lanius cristatus	Brown Shrike	2020/10/11	2	0	24.21429853	120.56223210	https://www.inaturalist.org/observations/62261309
1952	Lanius cristatus	Brown Shrike	2022/12/27	1	0	24.21867667	120.78961170	https://www.inaturalist.org/observations/145190521
1953	Lanius cristatus	Brown Shrike	2022/12/3	1	0	24.22803279	120.51919830	https://www.inaturalist.org/observations/144396204
1954	Lanius cristatus	Brown Shrike	2022/9/19	1	0	24.23038256	120.65628510	https://www.inaturalist.org/observations/136570432

1955	Lanius cristatus	Brown Shrike	2022/1/23	1	0	24.23463092	120.54643040	https://www.inaturalist.org/observations/105564850
1956	Lanius cristatus	Brown Shrike	2020/10/9	2	0	24.24097833	120.69950330	https://www.inaturalist.org/observations/62282402
1957	Lanius cristatus	Brown Shrike	2022/2/24	2	0	24.24342832	120.62408890	https://www.inaturalist.org/observations/107420769
1958	Lanius cristatus	Brown Shrike	2020/8/31	2	0	24.25071763	120.95585960	https://www.inaturalist.org/observations/59656971
1959	Lanius cristatus	Brown Shrike	2022/9/7	1	0	24.25224250	120.81087110	https://www.inaturalist.org/observations/136562230
1960	Lanius cristatus	Brown Shrike	2020/1/13	2	0	24.25637972	120.59933970	https://www.inaturalist.org/observations/37709106
1961	Lanius cristatus	Brown Shrike	2019/1/8	3	0	24.25727000	120.71637000	https://www.inaturalist.org/observations/19543532
1962	Lanius cristatus	Brown Shrike	2020/1/13	2	0	24.25803139	120.60000640	https://www.inaturalist.org/observations/37709104
1963	Lanius cristatus	Brown Shrike	2022/1/5	1	0	24.26918202	120.63554710	https://www.inaturalist.org/observations/104387465
1964	Lanius cristatus	Brown Shrike	2018/12/1	2	0	24.27039073	120.55741550	https://www.inaturalist.org/observations/18752969
1965	Lanius cristatus	Brown Shrike	2020/10/10	3	0	24.27326392	120.54679870	https://www.inaturalist.org/observations/62176881
1966	Lanius cristatus	Brown Shrike	2020/2/13	2	0	24.27483167	120.76082330	https://www.inaturalist.org/observations/38659785
1967	Lanius cristatus	Brown Shrike	2020/2/12	2	0	24.27499500	120.76031830	https://www.inaturalist.org/observations/38659779
1968	Lanius cristatus	Brown Shrike	2022/2/11	2	0	24.27525099	120.84533290	https://www.inaturalist.org/observations/106532928
1969	Lanius cristatus	Brown Shrike	2022/1/8	2	0	24.28133010	120.76942440	https://www.inaturalist.org/observations/104621457
1970	Lanius cristatus	Brown Shrike	2019/12/10	2	0	24.29413849	120.61040110	https://www.inaturalist.org/observations/37675715
1971	Lanius cristatus	Brown Shrike	2020/2/19	2	0	24.30784167	120.73778330	https://www.inaturalist.org/observations/39449285
1972	Lanius cristatus	Brown Shrike	2019/12/10	2	0	24.31194340	120.54964430	https://www.inaturalist.org/observations/37675462
1973	Lanius cristatus	Brown Shrike	2021/2/4	2	0	24.31440468	120.55144180	https://www.inaturalist.org/observations/69852294
1974	Lanius cristatus	Brown Shrike	2022/12/7	1	0	24.31776075	120.55235840	https://www.inaturalist.org/observations/144148819
1975	Lanius cristatus	Brown Shrike	2019/10/26	1	0	24.32305600	120.75976440	https://www.inaturalist.org/observations/35007659
1976	Lanius cristatus	Brown Shrike	2022/9/24	2	0	24.33699987	120.99514860	https://www.inaturalist.org/observations/141972476
1977	Lanius cristatus	Brown Shrike	2022/1/26	1	0	24.34500514	121.30820540	https://www.inaturalist.org/observations/105651217

1978	Lanius cristatus	Brown Shrike	2021/1/2	1	0	24.35057824	120.72566680	https://www.inaturalist.org/observations/95710553
1979	Lanius cristatus	Brown Shrike	2021/10/16	1	0	24.35267420	92.34481540	https://www.inaturalist.org/observations/101251428
1980	Lanius cristatus	Brown Shrike	2020/9/9	2	0	24.35301803	92.34154567	https://www.inaturalist.org/observations/59785140
1981	Lanius cristatus	Brown Shrike	2018/11/7	4	0	24.35648280	92.34330460	https://www.inaturalist.org/observations/19131913
1982	Lanius cristatus	Brown Shrike	2016/1/16	2	0	24.37695532	120.58277150	https://www.inaturalist.org/observations/10542042
1983	Lanius cristatus	Brown Shrike	2019/12/17	2	0	24.40014056	120.61787180	https://www.inaturalist.org/observations/36727434
1984	Lanius cristatus	Brown Shrike	2019/9/28	2	0	24.42563331	92.33881554	https://www.inaturalist.org/observations/33881543
1985	Lanius cristatus	Brown Shrike	2019/10/2	4	0	24.42813514	92.33542255	https://www.inaturalist.org/observations/34726159
1986	Lanius cristatus	Brown Shrike	2020/2/28	3	0	24.42813514	92.33542255	https://www.inaturalist.org/observations/46584991
1987	Lanius cristatus	Brown Shrike	2020/3/6	2	0	24.42813514	92.33542255	https://www.inaturalist.org/observations/46825167
1988	Lanius cristatus	Brown Shrike	2010/10/4	0	0	24.43202000	92.33068900	https://www.inaturalist.org/observations/103528439
1989	Lanius cristatus	Brown Shrike	2010/10/6	1	0	24.43202000	92.33068900	https://www.inaturalist.org/observations/103586527
1990	Lanius cristatus	Brown Shrike	2010/12/15	1	0	24.43202000	92.33068900	https://www.inaturalist.org/observations/103621319
1991	Lanius cristatus	Brown Shrike	2011/8/29	1	0	24.43202000	92.33068900	https://www.inaturalist.org/observations/103671162
1992	Lanius cristatus	Brown Shrike	2012/3/10	1	0	24.43202000	92.33068900	https://www.inaturalist.org/observations/103677426
1993	Lanius cristatus	Brown Shrike	2019/4/10	3	0	24.43334118	92.33028864	https://www.inaturalist.org/observations/33841627
1994	Lanius cristatus	Brown Shrike	2017/10/11	4	0	24.43341530	92.32874360	https://www.inaturalist.org/observations/13226823
1995	Lanius cristatus	Brown Shrike	2019/11/3	3	0	24.43353220	92.33039590	https://www.inaturalist.org/observations/35285253
1996	Lanius cristatus	Brown Shrike	2022/5/3	2	0	24.43756486	118.10350890	https://www.inaturalist.org/observations/115067614
1997	Lanius cristatus	Brown Shrike	2022/5/19	1	0	24.43908511	118.10224290	https://www.inaturalist.org/observations/118612680
1998	Lanius cristatus	Brown Shrike	2022/10/24	0	0	24.44023271	118.09844610	https://www.inaturalist.org/observations/139872628
1999	Lanius cristatus	Brown Shrike	2018/5/14	2	0	24.44095350	118.35205540	https://www.inaturalist.org/observations/15417147
2000	Lanius cristatus	Brown Shrike	2020/5/21	1	0	24.44305570	118.10020070	https://www.inaturalist.org/observations/112514801

2001	Lanius cristatus	Brown Shrike	2022/9/13	1	0	24.44555667	120.64006830	https://www.inaturalist.org/observations/134832909
2002	Lanius cristatus	Brown Shrike	2018/12/29	2	0	24.44647020	118.44712000	https://www.inaturalist.org/observations/19330592
2003	Lanius cristatus	Brown Shrike	2022/10/7	2	0	24.44705306	120.63607060	https://www.inaturalist.org/observations/137826018
2004	Lanius cristatus	Brown Shrike	2022/10/7	1	0	24.44969028	120.65082080	https://www.inaturalist.org/observations/137826005
2005	Lanius cristatus	Brown Shrike	2016/1/16	3	0	24.44995541	120.63946430	https://www.inaturalist.org/observations/11446746
2006	Lanius cristatus	Brown Shrike	2022/10/7	1	0	24.45128889	120.65126690	https://www.inaturalist.org/observations/137826003
2007	Lanius cristatus	Brown Shrike	2022/9/13	2	0	24.45179333	120.64231500	https://www.inaturalist.org/observations/134832972
2008	Lanius cristatus	Brown Shrike	2021/3/16	1	0	24.45944118	121.79799920	https://www.inaturalist.org/observations/78240891
2009	Lanius cristatus	Brown Shrike	2019/5/28	2	0	24.46388740	118.41440230	https://www.inaturalist.org/observations/30393798
2010	Lanius cristatus	Brown Shrike	2016/1/17	6	0	24.46555600	92.27400900	http://www.inaturalist.org/observations/4173782
2011	Lanius cristatus	Brown Shrike	2017/11/30	4	0	24.47433500	92.31172000	https://www.inaturalist.org/observations/13604563
2012	Lanius cristatus	Brown Shrike	2017/11/3	3	0	24.47873500	92.30142900	https://www.inaturalist.org/observations/13604469
2013	Lanius cristatus	Brown Shrike	2021/5/13	1	0	24.47990167	118.30830000	https://www.inaturalist.org/observations/79367509
2014	Lanius cristatus	Brown Shrike	2020/2/6	3	0	24.48294400	92.29071229	https://www.inaturalist.org/observations/46464766
2015	Lanius cristatus	Brown Shrike	2020/3/1	2	0	24.48294400	92.29071229	https://www.inaturalist.org/observations/46705496
2016	Lanius cristatus	Brown Shrike	2020/3/1	3	0	24.48294400	92.29071229	https://www.inaturalist.org/observations/46705498
2017	Lanius cristatus	Brown Shrike	2021/1/6	0	0	24.48294400	92.29071229	https://www.inaturalist.org/observations/95625304
2018	Lanius cristatus	Brown Shrike	2021/12/4	2	0	24.48294400	92.29071229	https://www.inaturalist.org/observations/104961935
2019	Lanius cristatus	Brown Shrike	2017/10/10	3	0	24.48298800	92.29041500	https://www.inaturalist.org/observations/13604595
2020	Lanius cristatus	Brown Shrike	2018/9/20	4	0	24.49770120	92.31476770	https://www.inaturalist.org/observations/19567360
2021	Lanius cristatus	Brown Shrike	2019/2/17	4	0	24.49770120	92.31476770	https://www.inaturalist.org/observations/20848156
2022	Lanius cristatus	Brown Shrike	2020/3/1	4	0	24.49781478	92.31476784	https://www.inaturalist.org/observations/39439412
2023	Lanius cristatus	Brown Shrike	2021/4/25	2	0	24.49930607	92.31533278	https://www.inaturalist.org/observations/75309154

2024	Lanius cristatus	Brown Shrike	2017/10/8	4	0	24.50061700	92.34840300	https://www.inaturalist.org/observations/9483738
2025	Lanius cristatus	Brown Shrike	2021/1/6	2	0	24.50145000	92.29966100	https://www.inaturalist.org/observations/69314270
2026	Lanius cristatus	Brown Shrike	2019/2/13	1	0	24.50395280	118.43707960	https://www.inaturalist.org/observations/141035392
2027	Lanius cristatus	Brown Shrike	2021/1/6	2	0	24.56047040	120.81591600	https://www.inaturalist.org/observations/67662294
2028	Lanius cristatus	Brown Shrike	2021/9/16	1	0	24.57079241	73.68263192	https://www.inaturalist.org/observations/105021711
2029	Lanius cristatus	Brown Shrike	2021/3/13	1	0	24.58434088	120.88638890	https://www.inaturalist.org/observations/103053680
2030	Lanius cristatus	Brown Shrike	2013/4/7	5	0	24.60728790	92.38344120	http://www.inaturalist.org/observations/231236
2031	Lanius cristatus	Brown Shrike	2022/1/30	1	0	24.61797922	121.85294140	https://www.inaturalist.org/observations/121402300
2032	Lanius cristatus	Brown Shrike	2022/12/22	1	0	24.62323304	120.75628690	https://www.inaturalist.org/observations/144850153
2033	Lanius cristatus	Brown Shrike	2022/12/22	1	0	24.62323304	120.75628690	https://www.inaturalist.org/observations/144850155
2034	Lanius cristatus	Brown Shrike	2021/1/10	2	0	24.65120833	121.81191000	https://www.inaturalist.org/observations/67851197
2035	Lanius cristatus	Brown Shrike	2019/9/23	3	0	24.65144684	121.82257180	https://www.inaturalist.org/observations/33694688
2036	Lanius cristatus	Brown Shrike	2021/1/24	3	0	24.65488810	121.81278390	https://www.inaturalist.org/observations/68688865
2037	Lanius cristatus	Brown Shrike	2018/10/2	3	0	24.65915700	121.82011300	https://www.inaturalist.org/observations/17123051
2038	Lanius cristatus	Brown Shrike	2019/1/23	2	0	24.65962638	121.82064540	https://www.inaturalist.org/observations/21222447
2039	Lanius cristatus	Brown Shrike	2020/9/6	2	0	24.66256807	121.81455750	https://www.inaturalist.org/observations/60299520
2040	Lanius cristatus	Brown Shrike	2019/12/16	2	0	24.66323250	121.81782690	https://www.inaturalist.org/observations/37515524
2041	Lanius cristatus	Brown Shrike	2021/1/26	2	0	24.66323250	121.81782690	https://www.inaturalist.org/observations/73182379
2042	Lanius cristatus	Brown Shrike	2021/1/26	1	0	24.66323250	121.81782690	https://www.inaturalist.org/observations/73182380
2043	Lanius cristatus	Brown Shrike	2020/9/18	2	0	24.66382132	121.81422570	https://www.inaturalist.org/observations/61280232
2044	Lanius cristatus	Brown Shrike	2020/10/8	2	0	24.66385300	121.81683790	https://www.inaturalist.org/observations/62481414
2045	Lanius cristatus	Brown Shrike	2021/10/30	2	0	24.67043017	121.81301980	https://www.inaturalist.org/observations/103819777
2046	Lanius cristatus	Brown Shrike	2021/2/7	3	0	24.67339667	121.81656170	https://www.inaturalist.org/observations/69194028

2047	Lanius cristatus	Brown Shrike	2017/3/3	2	0	24.67489044	121.76190770	https://www.inaturalist.org/observations/17378426
2048	Lanius cristatus	Brown Shrike	2020/10/1	3	0	24.67965096	121.75600560	https://www.inaturalist.org/observations/62012003
2049	Lanius cristatus	Brown Shrike	2019/11/27	2	0	24.68077057	121.73135230	https://www.inaturalist.org/observations/36317773
2050	Lanius cristatus	Brown Shrike	2022/3/19	2	0	24.68259000	121.75622000	https://www.inaturalist.org/observations/109344926
2051	Lanius cristatus	Brown Shrike	2022/11/10	2	0	24.68284500	121.76413670	https://www.inaturalist.org/observations/142054398
2052	Lanius cristatus	Brown Shrike	2009/12/27	2	0	24.68318617	121.77388150	https://www.inaturalist.org/observations/55455308
2053	Lanius cristatus	Brown Shrike	2021/1/1	3	0	24.68320475	121.77141520	https://www.inaturalist.org/observations/67381299
2054	Lanius cristatus	Brown Shrike	2009/12/26	2	0	24.68350786	121.77402630	https://www.inaturalist.org/observations/55454586
2055	Lanius cristatus	Brown Shrike	2009/12/26	2	0	24.68350786	121.77402630	https://www.inaturalist.org/observations/55454615
2056	Lanius cristatus	Brown Shrike	2022/3/6	2	0	24.68408030	121.77218500	https://www.inaturalist.org/observations/110253686
2057	Lanius cristatus	Brown Shrike	2020/2/8	2	0	24.68427801	121.77235800	https://www.inaturalist.org/observations/38605622
2058	Lanius cristatus	Brown Shrike	2021/2/18	2	0	24.68572667	121.77434830	https://www.inaturalist.org/observations/69924062
2059	Lanius cristatus	Brown Shrike	2022/10/10	1	0	24.68659000	121.82288670	https://www.inaturalist.org/observations/138250124
2060	Lanius cristatus	Brown Shrike	2016/2/13	2	0	24.69210559	120.88631170	https://www.inaturalist.org/observations/14561700
2061	Lanius cristatus	Brown Shrike	2022/10/14	1	0	24.69746546	121.80034920	https://www.inaturalist.org/observations/138698042
2062	Lanius cristatus	Brown Shrike	2022/5/4	3	0	24.70146561	120.86128120	https://www.inaturalist.org/observations/116296650
2063	Lanius cristatus	Brown Shrike	2022/4/30	1	0	24.70442010	121.15530740	https://www.inaturalist.org/observations/113799968
2064	Lanius cristatus	Brown Shrike	2020/1/9	2	0	24.70484667	121.80141670	https://www.inaturalist.org/observations/37632832
2065	Lanius cristatus	Brown Shrike	2020/1/9	2	0	24.70566000	121.80714500	https://www.inaturalist.org/observations/37632823
2066	Lanius cristatus	Brown Shrike	2020/4/25	3	0	24.70924107	121.72284440	https://www.inaturalist.org/observations/43286887
2067	Lanius cristatus	Brown Shrike	2022/10/23	3	0	24.71584137	121.78129710	https://www.inaturalist.org/observations/139708205
2068	Lanius cristatus	Brown Shrike	2022/11/22	1	0	24.71737845	121.81390180	https://www.inaturalist.org/observations/144910155
2069	Lanius cristatus	Brown Shrike	2022/1/16	1	0	24.71769500	121.81097670	https://www.inaturalist.org/observations/105029013

2070	Lanius cristatus	Brown Shrike	2021/11/25	1	0	24.72406590	121.76465290	https://www.inaturalist.org/observations/101932383
2071	Lanius cristatus	Brown Shrike	2021/12/29	2	0	24.73001000	120.96419500	https://www.inaturalist.org/observations/140156489
2072	Lanius cristatus	Brown Shrike	2018/11/5	2	0	24.73077225	121.08722430	https://www.inaturalist.org/observations/18158557
2073	Lanius cristatus	Brown Shrike	2021/12/4	2	0	24.73805560	121.75250000	https://www.inaturalist.org/observations/102524698
2074	Lanius cristatus	Brown Shrike	2021/2/19	2	0	24.74181000	121.69149830	https://www.inaturalist.org/observations/69924027
2075	Lanius cristatus	Brown Shrike	2021/11/7	1	0	24.74481322	121.72969150	https://www.inaturalist.org/observations/100471982
2076	Lanius cristatus	Brown Shrike	2022/2/15	1	0	24.74501610	121.73233190	https://www.inaturalist.org/observations/110252926
2077	Lanius cristatus	Brown Shrike	2021/4/19	1	0	24.74587640	121.75427270	https://www.inaturalist.org/observations/90241651
2078	Lanius cristatus	Brown Shrike	2021/2/20	2	0	24.74899833	121.63820670	https://www.inaturalist.org/observations/69924041
2079	Lanius cristatus	Brown Shrike	2019/11/5	2	0	24.74925548	121.76748310	https://www.inaturalist.org/observations/35526989
2080	Lanius cristatus	Brown Shrike	2018/1/17	1	0	24.74936050	121.63864890	https://www.inaturalist.org/observations/38792166
2081	Lanius cristatus	Brown Shrike	2020/9/18	2	0	24.74936050	121.63864890	https://www.inaturalist.org/observations/60696638
2082	Lanius cristatus	Brown Shrike	2020/10/23	1	0	24.74936050	121.63864890	https://www.inaturalist.org/observations/68398831
2083	Lanius cristatus	Brown Shrike	2019/10/17	2	0	24.74939529	121.76691380	https://www.inaturalist.org/observations/34558850
2084	Lanius cristatus	Brown Shrike	2021/2/20	3	0	24.74943500	121.63774330	https://www.inaturalist.org/observations/69924032
2085	Lanius cristatus	Brown Shrike	2021/1/12	2	0	24.74957667	121.76695750	https://www.inaturalist.org/observations/67952886
2086	Lanius cristatus	Brown Shrike	2021/1/12	2	0	24.74957667	121.76695750	https://www.inaturalist.org/observations/67952890
2087	Lanius cristatus	Brown Shrike	2021/1/12	1	0	24.74965462	121.76693600	https://www.inaturalist.org/observations/67953261
2088	Lanius cristatus	Brown Shrike	2020/10/17	2	0	24.74969390	121.76700950	https://www.inaturalist.org/observations/66509692
2089	Lanius cristatus	Brown Shrike	2021/1/19	3	0	24.74974710	121.76645040	https://www.inaturalist.org/observations/68320428
2090	Lanius cristatus	Brown Shrike	2021/1/19	3	0	24.74974710	121.76645040	https://www.inaturalist.org/observations/68320430
2091	Lanius cristatus	Brown Shrike	2021/11/15	1	0	24.74978128	121.76701110	https://www.inaturalist.org/observations/101259561
2092	Lanius cristatus	Brown Shrike	2021/1/18	3	0	24.74985428	121.76656840	https://www.inaturalist.org/observations/68320881

2093	Lanius cristatus	Brown Shrike	2021/1/19	3	0	24.74985428	121.76656840	https://www.inaturalist.org/observations/68320894
2094	Lanius cristatus	Brown Shrike	2021/1/14	1	0	24.74992110	121.76701400	https://www.inaturalist.org/observations/90108140
2095	Lanius cristatus	Brown Shrike	2021/1/14	1	0	24.74992110	121.76701400	https://www.inaturalist.org/observations/90108141
2096	Lanius cristatus	Brown Shrike	2021/1/15	1	0	24.74992110	121.76701400	https://www.inaturalist.org/observations/90108215
2097	Lanius cristatus	Brown Shrike	2021/1/15	1	0	24.74992110	121.76701400	https://www.inaturalist.org/observations/90108216
2098	Lanius cristatus	Brown Shrike	2018/11/15	2	0	24.75044800	121.63543700	https://www.inaturalist.org/observations/18428882
2099	Lanius cristatus	Brown Shrike	2018/1/12	4	0	24.75389400	121.63575500	https://www.inaturalist.org/observations/38779677
2100	Lanius cristatus	Brown Shrike	2020/4/28	2	0	24.75411667	121.14201380	https://www.inaturalist.org/observations/44105162
2101	Lanius cristatus	Brown Shrike	2015/10/15	2	0	24.75911500	121.75374000	https://www.inaturalist.org/observations/8837591
2102	Lanius cristatus	Brown Shrike	2022/2/16	1	0	24.77799031	121.02475700	https://www.inaturalist.org/observations/107299327
2103	Lanius cristatus	Brown Shrike	2018/1/22	2	0	24.78288469	120.99491690	https://www.inaturalist.org/observations/14196976
2104	Lanius cristatus	Brown Shrike	2021/12/25	2	0	24.78830667	120.99249500	https://www.inaturalist.org/observations/103623792
2105	Lanius cristatus	Brown Shrike	2011/9/28	1	0	24.78885800	92.79795400	https://www.inaturalist.org/observations/103754474
2106	Lanius cristatus	Brown Shrike	2021/12/2	1	0	24.79377939	120.91721930	https://www.inaturalist.org/observations/109989611
2107	Lanius cristatus	Brown Shrike	2021/8/30	2	0	24.79417500	120.95033830	https://www.inaturalist.org/observations/93012693
2108	Lanius cristatus	Brown Shrike	2012/2/28	3	0	24.79871140	121.79631110	https://www.inaturalist.org/observations/56209412
2109	Lanius cristatus	Brown Shrike	2022/12/4	1	0	24.79980901	121.80352090	https://www.inaturalist.org/observations/143627577
2110	Lanius cristatus	Brown Shrike	2020/1/23	2	0	24.80024910	121.73938750	https://www.inaturalist.org/observations/37872321
2111	Lanius cristatus	Brown Shrike	2020/2/12	3	0	24.80037117	121.73938750	https://www.inaturalist.org/observations/38750366
2112	Lanius cristatus	Brown Shrike	2020/2/8	4	0	24.80111178	120.97958150	https://www.inaturalist.org/observations/38416101
2113	Lanius cristatus	Brown Shrike	2020/11/22	3	0	24.80951990	121.80435010	https://www.inaturalist.org/observations/65402646
2114	Lanius cristatus	Brown Shrike	2018/12/18	3	0	24.81327529	120.93932860	https://www.inaturalist.org/observations/19636240
2115	Lanius cristatus	Brown Shrike	2021/1/13	2	0	24.81564833	121.04706830	https://www.inaturalist.org/observations/68007127

2116	Lanius cristatus	Brown Shrike	2022/9/12	1	0	24.82367500	120.98196940	https://www.inaturalist.org/observations/134692760
2117	Lanius cristatus	Brown Shrike	2021/3/8	3	0	24.83139727	121.00955300	https://www.inaturalist.org/observations/70914237
2118	Lanius cristatus	Brown Shrike	2021/2/2	1	0	24.83175088	121.75424680	https://www.inaturalist.org/observations/73368422
2119	Lanius cristatus	Brown Shrike	2020/9/8	2	0	24.83215052	121.00896740	https://www.inaturalist.org/observations/59201409
2120	Lanius cristatus	Brown Shrike	2022/4/23	1	0	24.83326912	121.01999660	https://www.inaturalist.org/observations/112484724
2121	Lanius cristatus	Brown Shrike	2021/12/2	1	0	24.91801165	121.53875440	https://www.inaturalist.org/observations/133072352
2122	Lanius cristatus	Brown Shrike	2020/11/5	2	0	24.93230894	74.96835183	https://www.inaturalist.org/observations/67467715
2123	Lanius cristatus	Brown Shrike	2022/3/1	1	0	24.93377590	121.06642710	https://www.inaturalist.org/observations/109988633
2124	Lanius cristatus	Brown Shrike	2019/9/23	0	0	24.93450203	121.24081790	https://www.inaturalist.org/observations/33238809
2125	Lanius cristatus	Brown Shrike	2021/10/16	1	0	24.93903425	121.52667180	https://www.inaturalist.org/observations/98801592
2126	Lanius cristatus	Brown Shrike	2020/9/27	3	0	24.94173832	121.36941000	https://www.inaturalist.org/observations/60932613
2127	Lanius cristatus	Brown Shrike	2022/4/30	2	0	24.94750730	121.21306040	https://www.inaturalist.org/observations/114622101
2128	Lanius cristatus	Brown Shrike	2021/12/28	1	0	24.95123807	121.22801690	https://www.inaturalist.org/observations/104091931
2129	Lanius cristatus	Brown Shrike	2022/11/18	1	0	24.95146807	121.22812510	https://www.inaturalist.org/observations/142599953
2130	Lanius cristatus	Brown Shrike	2022/4/30	2	0	24.95231963	121.21721300	https://www.inaturalist.org/observations/114620301
2131	Lanius cristatus	Brown Shrike	2014/3/22	2	0	24.96222172	121.20959310	https://www.inaturalist.org/observations/105655899
2132	Lanius cristatus	Brown Shrike	2022/4/24	1	0	24.97277910	121.42040420	https://www.inaturalist.org/observations/112833005
2133	Lanius cristatus	Brown Shrike	2018/2/1	2	0	24.97415783	121.54115310	https://www.inaturalist.org/observations/18616202
2134	Lanius cristatus	Brown Shrike	2018/2/1	2	0	24.97416148	121.54116920	https://www.inaturalist.org/observations/18616201
2135	Lanius cristatus	Brown Shrike	2019/2/20	3	0	24.97842600	121.55736020	https://www.inaturalist.org/observations/20505532
2136	Lanius cristatus	Brown Shrike	2019/4/21	2	0	24.97879434	121.27672470	https://www.inaturalist.org/observations/40930678
2137	Lanius cristatus	Brown Shrike	2022/3/13	3	0	24.97921612	121.52680730	https://www.inaturalist.org/observations/108484480
2138	Lanius cristatus	Brown Shrike	2019/1/19	3	0	24.98547658	121.45724340	https://www.inaturalist.org/observations/19771583

2139	Lanius cristatus	Brown Shrike	2018/2/16	2	0	24.98607255	121.45735110	https://www.inaturalist.org/observations/18616469
2140	Lanius cristatus	Brown Shrike	2021/2/23	1	0	24.98646139	121.55278800	https://www.inaturalist.org/observations/75019822
2141	Lanius cristatus	Brown Shrike	2021/11/8	2	0	24.98681225	121.55521230	https://www.inaturalist.org/observations/100846115
2142	Lanius cristatus	Brown Shrike	2022/11/10	1	0	24.98812490	121.48124220	https://www.inaturalist.org/observations/141586556
2143	Lanius cristatus	Brown Shrike	2021/1/1	2	0	24.98823780	121.53917460	https://www.inaturalist.org/observations/101893650
2144	Lanius cristatus	Brown Shrike	2021/11/18	2	0	24.98841321	121.57225700	https://www.inaturalist.org/observations/101715241
2145	Lanius cristatus	Brown Shrike	2021/12/20	2	0	24.98933848	121.53635380	https://www.inaturalist.org/observations/103428520
2146	Lanius cristatus	Brown Shrike	2021/12/22	2	0	24.98967530	121.53616440	https://www.inaturalist.org/observations/103476083
2147	Lanius cristatus	Brown Shrike	2021/12/22	2	0	24.98976564	121.53681330	https://www.inaturalist.org/observations/103476084
2148	Lanius cristatus	Brown Shrike	2021/12/22	2	0	24.99025282	121.53651690	https://www.inaturalist.org/observations/103476086
2149	Lanius cristatus	Brown Shrike	2009/11/20	2	0	24.99339645	121.32087420	https://www.inaturalist.org/observations/55451639
2150	Lanius cristatus	Brown Shrike	2012/12/9	2	0	24.99386120	121.33903610	https://www.inaturalist.org/observations/56615661
2151	Lanius cristatus	Brown Shrike	2010/12/19	2	0	24.99387154	121.33939140	https://www.inaturalist.org/observations/55758389
2152	Lanius cristatus	Brown Shrike	2021/4/7	1	0	24.99505806	121.49999240	https://www.inaturalist.org/observations/73214581
2153	Lanius cristatus	Brown Shrike	2022/3/8	1	0	24.99579876	121.32951520	https://www.inaturalist.org/observations/112512697
2154	Lanius cristatus	Brown Shrike	2019/12/9	2	0	24.99685000	121.43751170	https://www.inaturalist.org/observations/36495743
2155	Lanius cristatus	Brown Shrike	2019/4/20	2	0	24.99983040	121.43860320	https://www.inaturalist.org/observations/23911484
2156	Lanius cristatus	Brown Shrike	2009/10/22	3	0	25.00111912	121.32868850	https://www.inaturalist.org/observations/55378197
2157	Lanius cristatus	Brown Shrike	2021/1/15	0	0	25.00797197	121.53577770	https://www.inaturalist.org/observations/68400388
2158	Lanius cristatus	Brown Shrike	2019/2/3	2	0	25.01236667	121.32777500	https://www.inaturalist.org/observations/29153691
2159	Lanius cristatus	Brown Shrike	2017/3/24	1	0	25.01277532	121.46114400	https://www.inaturalist.org/observations/142124460
2160	Lanius cristatus	Brown Shrike	2021/1/1	1	0	25.01277760	121.44089040	https://www.inaturalist.org/observations/67705599
2161	Lanius cristatus	Brown Shrike	2021/11/5	1	0	25.01482588	121.91916440	https://www.inaturalist.org/observations/100305662

2162	Lanius cristatus	Brown Shrike	2021/4/11	1	0	25.01513611	121.92427140	https://www.inaturalist.org/observations/73718712
2163	Lanius cristatus	Brown Shrike	2005/4/8	2	0	25.01530236	121.53991860	https://www.inaturalist.org/observations/16706931
2164	Lanius cristatus	Brown Shrike	2022/3/14	3	0	25.01554323	121.91724690	https://www.inaturalist.org/observations/109358862
2165	Lanius cristatus	Brown Shrike	2019/1/22	2	0	25.01560738	121.53963310	https://www.inaturalist.org/observations/19814862
2166	Lanius cristatus	Brown Shrike	2021/12/15	1	0	25.01576667	121.54072000	https://www.inaturalist.org/observations/103082858
2167	Lanius cristatus	Brown Shrike	2021/1/25	2	0	25.01601991	121.54057990	https://www.inaturalist.org/observations/68583625
2168	Lanius cristatus	Brown Shrike	2021/2/2	2	0	25.01613020	121.54060200	https://www.inaturalist.org/observations/68948568
2169	Lanius cristatus	Brown Shrike	2021/11/16	1	0	25.01620676	121.54044950	https://www.inaturalist.org/observations/101245028
2170	Lanius cristatus	Brown Shrike	2019/11/12	2	0	25.01646085	121.54055290	https://www.inaturalist.org/observations/36285766
2171	Lanius cristatus	Brown Shrike	2021/11/16	1	0	25.01654161	121.54092390	https://www.inaturalist.org/observations/101391264
2172	Lanius cristatus	Brown Shrike	2022/11/13	1	0	25.01698222	121.92124940	https://www.inaturalist.org/observations/142273841
2173	Lanius cristatus	Brown Shrike	2022/1/3	1	0	25.01720950	121.92830650	https://www.inaturalist.org/observations/104971608
2174	Lanius cristatus	Brown Shrike	2022/3/6	2	0	25.01725730	85.41616420	https://www.inaturalist.org/observations/108017689
2175	Lanius cristatus	Brown Shrike	2021/10/6	2	0	25.01734397	121.53975180	https://www.inaturalist.org/observations/97297952
2176	Lanius cristatus	Brown Shrike	2017/12/24	2	0	25.01835597	121.92430280	https://www.inaturalist.org/observations/14127467
2177	Lanius cristatus	Brown Shrike	2022/1/10	1	0	25.01877131	121.53396730	https://www.inaturalist.org/observations/104685571
2178	Lanius cristatus	Brown Shrike	2014/10/8	4	0	25.01896872	121.92325030	https://www.inaturalist.org/observations/57317752
2179	Lanius cristatus	Brown Shrike	2020/3/15	2	0	25.01933990	121.92294210	https://www.inaturalist.org/observations/110839839
2180	Lanius cristatus	Brown Shrike	2019/10/4	2	0	25.01935768	121.94384000	https://www.inaturalist.org/observations/34299463
2181	Lanius cristatus	Brown Shrike	2019/3/12	2	0	25.01971651	121.53805160	https://www.inaturalist.org/observations/48522999
2182	Lanius cristatus	Brown Shrike	2020/4/10	2	0	25.02010594	121.92228580	https://www.inaturalist.org/observations/110656184
2183	Lanius cristatus	Brown Shrike	2019/11/12	2	0	25.02032319	121.53771400	https://www.inaturalist.org/observations/35739798
2184	Lanius cristatus	Brown Shrike	2019/11/18	2	0	25.02041100	121.92256700	https://www.inaturalist.org/observations/35852459

2185	Lanius cristatus	Brown Shrike	2022/9/10	3	0	25.02093056	121.31076390	https://www.inaturalist.org/observations/134709710
2186	Lanius cristatus	Brown Shrike	2022/2/4	2	0	25.02135593	121.54643760	https://www.inaturalist.org/observations/110064747
2187	Lanius cristatus	Brown Shrike	2022/2/11	1	0	25.02170856	121.50778380	https://www.inaturalist.org/observations/106550049
2188	Lanius cristatus	Brown Shrike	2022/12/3	2	0	25.02180489	121.30611810	https://www.inaturalist.org/observations/145176370
2189	Lanius cristatus	Brown Shrike	2020/4/25	2	0	25.02214029	121.51454110	https://www.inaturalist.org/observations/43264442
2190	Lanius cristatus	Brown Shrike	2022/2/11	1	0	25.02275837	121.51210200	https://www.inaturalist.org/observations/106593729
2191	Lanius cristatus lucionensis	Brown Shrike	2019/3/11	2	0	25.02327866	121.50471210	https://www.inaturalist.org/observations/65251136
2192	Lanius cristatus	Brown Shrike	2019/10/28	1	0	25.02341900	121.92460500	https://www.inaturalist.org/observations/35039466
2193	Lanius cristatus	Brown Shrike	2022/2/22	2	0	25.02413617	121.50502830	https://www.inaturalist.org/observations/107244509
2194	Lanius cristatus	Brown Shrike	2018/11/14	2	0	25.02450663	121.54553030	https://www.inaturalist.org/observations/18421270
2195	Lanius cristatus	Brown Shrike	2021/4/10	1	0	25.02455000	121.50502000	https://www.inaturalist.org/observations/76019811
2196	Lanius cristatus	Brown Shrike	2018/1/14	2	0	25.02574667	121.20277500	https://www.inaturalist.org/observations/14838294
2197	Lanius cristatus	Brown Shrike	2022/11/14	1	0	25.02744100	121.29676300	https://www.inaturalist.org/observations/142663611
2198	Lanius cristatus	Brown Shrike	2019/3/19	2	0	25.02933278	121.53563720	https://www.inaturalist.org/observations/21384692
2199	Lanius cristatus	Brown Shrike	2020/9/29	2	0	25.03048676	121.53577950	https://www.inaturalist.org/observations/63149498
2200	Lanius cristatus	Brown Shrike	2021/12/14	1	0	25.03074500	121.53554500	https://www.inaturalist.org/observations/104971591
2201	Lanius cristatus	Brown Shrike	2020/12/10	2	0	25.03171353	121.53601920	https://www.inaturalist.org/observations/66367808
2202	Lanius cristatus	Brown Shrike	2011/11/21	1	0	25.03185110	121.50945930	https://www.inaturalist.org/observations/130587685
2203	Lanius cristatus	Brown Shrike	2020/5/8	1	0	25.03204368	121.50802050	https://www.inaturalist.org/observations/102273241
2204	Lanius cristatus	Brown Shrike	2015/1/25	2	0	25.03214524	121.50976530	https://www.inaturalist.org/observations/19795456
2205	Lanius cristatus	Brown Shrike	2015/1/25	4	0	25.03226100	121.50914800	http://www.inaturalist.org/observations/1192730
2206	Lanius cristatus	Brown Shrike	2021/9/23	1	0	25.03246306	121.45840830	https://www.inaturalist.org/observations/98684290
2207	Lanius cristatus	Brown Shrike	2018/12/30	2	0	25.03279104	121.50878460	https://www.inaturalist.org/observations/19351733

2208	Lanius cristatus	Brown Shrike	2021/8/1	1	0	25.03296360	121.56542680	https://www.inaturalist.org/observations/89423648
2209	Lanius cristatus	Brown Shrike	2022/2/11	1	0	25.03296360	121.56542680	https://www.inaturalist.org/observations/106536216
2210	Lanius cristatus	Brown Shrike	2018/11/16	2	0	25.03344418	121.50944890	https://www.inaturalist.org/observations/18733650
2211	Lanius cristatus	Brown Shrike	2021/12/2	2	0	25.03444282	121.52245390	https://www.inaturalist.org/observations/102542459
2212	Lanius cristatus	Brown Shrike	2021/12/23	2	0	25.03487200	121.52268700	https://www.inaturalist.org/observations/103822422
2213	Lanius cristatus	Brown Shrike	2021/12/2	2	0	25.03515344	121.52273370	https://www.inaturalist.org/observations/102542462
2214	Lanius cristatus	Brown Shrike	2016/1/27	1	0	25.03531751	121.52327770	https://www.inaturalist.org/observations/142123861
2215	Lanius cristatus	Brown Shrike	2021/12/23	2	0	25.03541653	121.52156510	https://www.inaturalist.org/observations/103822421
2216	Lanius cristatus	Brown Shrike	2021/2/15	2	0	25.03574648	121.56161170	https://www.inaturalist.org/observations/69615302
2217	Lanius cristatus	Brown Shrike	2019/4/26	2	0	25.03623333	121.56495000	https://www.inaturalist.org/observations/23319347
2218	Lanius cristatus	Brown Shrike	2021/12/28	1	0	25.03623632	121.47872330	https://www.inaturalist.org/observations/104415659
2219	Lanius cristatus	Brown Shrike	2017/12/30	2	0	25.03651207	121.48956680	https://www.inaturalist.org/observations/14127250
2220	Lanius cristatus	Brown Shrike	2019/11/10	3	0	25.03696998	121.49060380	https://www.inaturalist.org/observations/36102315
2221	Lanius cristatus	Brown Shrike	2019/3/6	3	0	25.03798922	121.56711270	https://www.inaturalist.org/observations/70635966
2222	Lanius cristatus	Brown Shrike	2022/12/24	1	0	25.03835973	121.49090090	https://www.inaturalist.org/observations/144975413
2223	Lanius cristatus	Brown Shrike	2018/11/11	2	0	25.03836410	121.49148950	https://www.inaturalist.org/observations/19984105
2224	Lanius cristatus	Brown Shrike	2022/12/19	1	0	25.03836410	121.49148950	https://www.inaturalist.org/observations/144673970
2225	Lanius cristatus	Brown Shrike	2022/11/26	1	0	25.03839167	121.49258670	https://www.inaturalist.org/observations/142883565
2226	Lanius cristatus	Brown Shrike	2020/12/5	3	0	25.03840000	121.47271670	https://www.inaturalist.org/observations/66229575
2227	Lanius cristatus	Brown Shrike	2022/1/25	1	0	25.03856284	121.49191290	https://www.inaturalist.org/observations/105604327
2228	Lanius cristatus	Brown Shrike	2022/2/11	2	0	25.03857500	121.49234670	https://www.inaturalist.org/observations/106538939
2229	Lanius cristatus	Brown Shrike	2020/1/22	3	0	25.03870250	121.49522080	https://www.inaturalist.org/observations/37941206
2230	Lanius cristatus	Brown Shrike	2020/9/11	2	0	25.03870250	121.49522080	https://www.inaturalist.org/observations/110656185

2231	Lanius cristatus	Brown Shrike	2009/10/23	2	0	25.03891957	121.49012230	https://www.inaturalist.org/observations/55447255
2232	Lanius cristatus	Brown Shrike	2019/11/10	3	0	25.03943472	121.49499730	https://www.inaturalist.org/observations/36102321
2233	Lanius cristatus	Brown Shrike	2021/11/21	1	0	25.03949450	121.49553670	https://www.inaturalist.org/observations/101606962
2234	Lanius cristatus	Brown Shrike	2020/12/5	3	0	25.03956667	121.47820000	https://www.inaturalist.org/observations/66229573
2235	Lanius cristatus	Brown Shrike	2021/12/1	1	0	25.04212797	121.51542730	https://www.inaturalist.org/observations/102463677
2236	Lanius cristatus	Brown Shrike	2016/9/9	2	0	25.04311300	121.61643200	https://www.inaturalist.org/observations/9735562
2237	Lanius cristatus	Brown Shrike	2020/10/23	3	0	25.04560300	121.52400000	https://www.inaturalist.org/observations/63481406
2238	Lanius cristatus	Brown Shrike	2021/3/11	1	0	25.04562738	121.53013830	https://www.inaturalist.org/observations/82171608
2239	Lanius cristatus	Brown Shrike	2020/10/27	2	0	25.05135500	121.59030320	https://www.inaturalist.org/observations/63626004
2240	Lanius cristatus	Brown Shrike	2019/9/27	2	0	25.05189500	121.22428500	https://www.inaturalist.org/observations/66140138
2241	Lanius cristatus	Brown Shrike	2018/12/17	2	0	25.05250000	121.52611110	https://www.inaturalist.org/observations/19201087
2242	Lanius cristatus	Brown Shrike	2019/9/25	2	0	25.05533333	121.22509830	https://www.inaturalist.org/observations/66140132
2243	Lanius cristatus	Brown Shrike	2020/4/27	3	0	25.05743886	121.45315150	https://www.inaturalist.org/observations/43861400
2244	Lanius cristatus	Brown Shrike	2019/12/2	2	0	25.06012197	121.57162300	https://www.inaturalist.org/observations/36405022
2245	Lanius cristatus	Brown Shrike	2021/11/14	1	0	25.06062285	121.47858070	https://www.inaturalist.org/observations/101081773
2246	Lanius cristatus	Brown Shrike	2022/4/25	2	0	25.06203355	121.45995660	https://www.inaturalist.org/observations/112977106
2247	Lanius cristatus	Brown Shrike	2022/4/18	1	0	25.06212474	121.45995250	https://www.inaturalist.org/observations/111952961
2248	Lanius cristatus	Brown Shrike	2022/3/22	1	0	25.06215303	121.45989740	https://www.inaturalist.org/observations/109568729
2249	Lanius cristatus	Brown Shrike	2022/4/22	2	0	25.06216445	121.45994010	https://www.inaturalist.org/observations/112977067
2250	Lanius cristatus	Brown Shrike	2022/3/31	2	0	25.06232084	121.46029980	https://www.inaturalist.org/observations/110405181
2251	Lanius cristatus	Brown Shrike	2022/4/28	2	0	25.06263594	121.45966510	https://www.inaturalist.org/observations/113874523
2252	Lanius cristatus	Brown Shrike	2022/12/4	1	0	25.06346679	121.53914090	https://www.inaturalist.org/observations/143787184
2253	Lanius cristatus	Brown Shrike	2019/9/27	2	0	25.06424000	121.21365830	https://www.inaturalist.org/observations/66140142

2254	Lanius cristatus	Brown Shrike	2022/9/27	1	0	25.06461000	121.23190000	https://www.inaturalist.org/observations/142054444
2255	Lanius cristatus	Brown Shrike	2018/12/18	3	0	25.06721173	121.57177710	https://www.inaturalist.org/observations/19076425
2256	Lanius cristatus	Brown Shrike	2022/9/27	1	0	25.06776446	121.57384100	https://www.inaturalist.org/observations/136637846
2257	Lanius cristatus	Brown Shrike	2021/11/18	1	0	25.06812217	121.61875830	https://www.inaturalist.org/observations/101374263
2258	Lanius cristatus	Brown Shrike	2021/9/29	1	0	25.06898166	121.59090250	https://www.inaturalist.org/observations/96558461
2259	Lanius cristatus	Brown Shrike	2022/10/23	3	0	25.06945956	121.52970050	https://www.inaturalist.org/observations/139714779
2260	Lanius cristatus	Brown Shrike	2020/10/15	2	0	25.07125660	121.59671390	https://www.inaturalist.org/observations/69182126
2261	Lanius cristatus	Brown Shrike	2021/2/17	2	0	25.07125660	121.59671390	https://www.inaturalist.org/observations/69713836
2262	Lanius cristatus	Brown Shrike	2021/12/5	2	0	25.07161676	121.62124080	https://www.inaturalist.org/observations/102521888
2263	Lanius cristatus	Brown Shrike	2022/2/8	1	0	25.07274162	121.53777300	https://www.inaturalist.org/observations/106365599
2264	Lanius cristatus	Brown Shrike	2022/2/11	1	0	25.07542500	121.65994170	https://www.inaturalist.org/observations/106536277
2265	Lanius cristatus	Brown Shrike	2022/2/27	2	0	25.07698953	121.45731610	https://www.inaturalist.org/observations/107635157
2266	Lanius cristatus	Brown Shrike	2021/11/21	2	0	25.07740283	121.66636670	https://www.inaturalist.org/observations/101608717
2267	Lanius cristatus	Brown Shrike	2022/4/24	1	0	25.07778617	121.66653330	https://www.inaturalist.org/observations/112658159
2268	Lanius cristatus	Brown Shrike	2022/2/17	1	0	25.07795833	121.67403330	https://www.inaturalist.org/observations/106897522
2269	Lanius cristatus	Brown Shrike	2021/4/5	1	0	25.07997783	121.67146330	https://www.inaturalist.org/observations/72994994
2270	Lanius cristatus	Brown Shrike	2022/9/22	1	0	25.08099923	121.67086300	https://www.inaturalist.org/observations/135989147
2271	Lanius cristatus	Brown Shrike	2022/5/5	1	0	25.08100174	121.67091010	https://www.inaturalist.org/observations/115466459
2272	Lanius cristatus	Brown Shrike	2021/9/18	5	0	25.08104186	121.67081900	https://www.inaturalist.org/observations/95240748
2273	Lanius cristatus	Brown Shrike	2022/3/4	3	0	25.08104186	121.67081900	https://www.inaturalist.org/observations/107861192
2274	Lanius cristatus	Brown Shrike	2022/10/27	1	0	25.08118196	121.55614440	https://www.inaturalist.org/observations/140121940
2275	Lanius cristatus	Brown Shrike	2011/12/10	2	0	25.08228050	121.60445190	https://www.inaturalist.org/observations/130586407
2276	Lanius cristatus	Brown Shrike	2015/8/24	2	0	25.08272511	121.19819120	https://www.inaturalist.org/observations/58824867

2277	Lanius cristatus	Brown Shrike	2010/9/25	2	0	25.08319153	121.20093780	https://www.inaturalist.org/observations/55683838
2278	Lanius cristatus	Brown Shrike	2021/9/19	1	0	25.08358586	121.20360370	https://www.inaturalist.org/observations/95567075
2279	Lanius cristatus	Brown Shrike	2021/9/19	2	0	25.08504503	121.20137950	https://www.inaturalist.org/observations/95567095
2280	Lanius cristatus	Brown Shrike	2012/9/16	2	0	25.08587340	121.20014380	https://www.inaturalist.org/observations/56493050
2281	Lanius cristatus	Brown Shrike	2015/8/30	3	0	25.08587340	121.20014380	https://www.inaturalist.org/observations/58825257
2282	Lanius cristatus	Brown Shrike	2015/8/30	2	0	25.08587340	121.20014380	https://www.inaturalist.org/observations/58825258
2283	Lanius cristatus	Brown Shrike	2015/8/30	2	0	25.08587340	121.20014380	https://www.inaturalist.org/observations/58825260
2284	Lanius cristatus	Brown Shrike	2015/4/11	3	0	25.08641755	121.19904950	https://www.inaturalist.org/observations/57626791
2285	Lanius cristatus	Brown Shrike	2019/1/10	2	0	25.08727894	121.50433040	https://www.inaturalist.org/observations/139756618
2286	Lanius cristatus	Brown Shrike	2019/1/10	2	0	25.08735667	121.50433040	https://www.inaturalist.org/observations/139756616
2287	Lanius cristatus	Brown Shrike	2022/9/10	1	0	25.08748861	121.19861440	https://www.inaturalist.org/observations/144837985
2288	Lanius cristatus	Brown Shrike	2021/8/27	1	0	25.08751667	121.17767170	https://www.inaturalist.org/observations/92912746
2289	Lanius cristatus	Brown Shrike	2019/1/10	2	0	25.08782329	121.50416950	https://www.inaturalist.org/observations/139756613
2290	Lanius cristatus	Brown Shrike	2021/9/5	1	0	25.08842000	121.17948170	https://www.inaturalist.org/observations/93707512
2291	Lanius cristatus	Brown Shrike	2022/2/27	1	0	25.08865017	121.45452360	https://www.inaturalist.org/observations/107635166
2292	Lanius cristatus	Brown Shrike	2021/3/9	3	0	25.08873609	121.45036550	https://www.inaturalist.org/observations/71937177
2293	Lanius cristatus	Brown Shrike	2022/12/24	1	0	25.09062778	121.51157220	https://www.inaturalist.org/observations/145039696
2294	Lanius cristatus	Brown Shrike	2020/4/16	2	0	25.09109600	121.58130200	https://www.inaturalist.org/observations/59027288
2295	Lanius cristatus	Brown Shrike	2018/10/7	3	0	25.09130482	121.44999220	https://www.inaturalist.org/observations/18409163
2296	Lanius cristatus	Brown Shrike	2021/10/14	2	0	25.09159028	121.58306720	https://www.inaturalist.org/observations/98255810
2297	Lanius cristatus	Brown Shrike	2021/10/2	1	0	25.09160167	121.18476330	https://www.inaturalist.org/observations/96992826
2298	Lanius cristatus	Brown Shrike	2021/10/14	2	0	25.09160646	121.58306450	https://www.inaturalist.org/observations/98255811
2299	Lanius cristatus	Brown Shrike	2021/9/5	1	0	25.09182500	121.18505830	https://www.inaturalist.org/observations/93707509

2300	Lanius cristatus	Brown Shrike	2022/4/6	1	0	25.09220717	121.46309140	https://www.inaturalist.org/observations/110559711
2301	Lanius cristatus	Brown Shrike	2021/9/5	1	0	25.09223167	121.18613830	https://www.inaturalist.org/observations/93707510
2302	Lanius cristatus	Brown Shrike	2022/12/24	1	0	25.09277927	121.45397450	https://www.inaturalist.org/observations/145244852
2303	Lanius cristatus	Brown Shrike	2020/9/5	3	0	25.09285352	121.52768520	https://www.inaturalist.org/observations/58581807
2304	Lanius cristatus	Brown Shrike	2020/3/3	2	0	25.09307740	121.53239640	https://www.inaturalist.org/observations/39555441
2305	Lanius cristatus	Brown Shrike	2022/2/4	1	0	25.09309397	121.18899070	https://www.inaturalist.org/observations/110140444
2306	Lanius cristatus	Brown Shrike	2021/12/5	1	0	25.09445866	121.45077750	https://www.inaturalist.org/observations/105599712
2307	Lanius cristatus	Brown Shrike	2022/11/19	2	0	25.09447003	121.45101850	https://www.inaturalist.org/observations/142349551
2308	Lanius cristatus	Brown Shrike	2022/1/10	1	0	25.09458727	121.45776660	https://www.inaturalist.org/observations/104711078
2309	Lanius cristatus	Brown Shrike	2022/1/10	1	0	25.09459863	121.46061490	https://www.inaturalist.org/observations/104706082
2310	Lanius cristatus	Brown Shrike	2020/1/31	3	0	25.09497147	121.52950000	https://www.inaturalist.org/observations/38330284
2311	Lanius cristatus	Brown Shrike	2022/9/11	1	0	25.09513286	121.45084090	https://www.inaturalist.org/observations/136562964
2312	Lanius cristatus	Brown Shrike	2022/12/10	1	0	25.09521277	121.45086190	https://www.inaturalist.org/observations/145245417
2313	Lanius cristatus	Brown Shrike	2022/9/30	2	0	25.09538083	121.55698500	https://www.inaturalist.org/observations/142570600
2314	Lanius cristatus	Brown Shrike	2021/2/6	2	0	25.09567773	121.45105640	https://www.inaturalist.org/observations/71934680
2315	Lanius cristatus	Brown Shrike	2022/12/24	1	0	25.09707833	121.49708500	https://www.inaturalist.org/observations/145039695
2316	Lanius cristatus	Brown Shrike	2019/4/7	2	0	25.09769833	121.20677330	https://www.inaturalist.org/observations/22135434
2317	Lanius cristatus	Brown Shrike	2019/4/17	2	0	25.09784480	121.45682380	https://www.inaturalist.org/observations/22651096
2318	Lanius cristatus	Brown Shrike	2021/12/5	1	0	25.09807640	121.45508090	https://www.inaturalist.org/observations/105599711
2319	Lanius cristatus	Brown Shrike	2020/4/19	4	0	25.09813400	121.45389800	https://www.inaturalist.org/observations/45070042
2320	Lanius cristatus	Brown Shrike	2021/11/1	1	0	25.09814482	121.45647090	https://www.inaturalist.org/observations/100127245
2321	Lanius cristatus	Brown Shrike	2022/9/11	1	0	25.09818048	121.45606710	https://www.inaturalist.org/observations/136562975
2322	Lanius cristatus	Brown Shrike	2022/2/26	3	0	25.09842890	121.45625450	https://www.inaturalist.org/observations/107463600

2323	Lanius cristatus	Brown Shrike	2015/4/12	2	0	25.09865496	121.22174960	https://www.inaturalist.org/observations/57627325
2324	Lanius cristatus	Brown Shrike	2022/11/13	2	0	25.09914847	121.45327120	https://www.inaturalist.org/observations/141876815
2325	Lanius cristatus	Brown Shrike	2019/9/25	2	0	25.09916578	121.45762300	https://www.inaturalist.org/observations/34949710
2326	Lanius cristatus	Brown Shrike	2020/12/5	2	0	25.09964727	121.45476450	https://www.inaturalist.org/observations/66324515
2327	Lanius cristatus	Brown Shrike	2014/3/1	2	0	25.10000563	121.20730270	https://www.inaturalist.org/observations/56903936
2328	Lanius cristatus	Brown Shrike	2018/2/2	2	0	25.10569450	121.52396330	https://www.inaturalist.org/observations/9701053
2329	Lanius cristatus	Brown Shrike	2020/11/15	2	0	25.10856881	121.45607930	https://www.inaturalist.org/observations/64914421
2330	Lanius cristatus lucionensis	Brown Shrike	2021/2/5	1	0	25.11186330	121.49338170	https://www.inaturalist.org/observations/69084565
2331	Lanius cristatus	Brown Shrike	2018/10/6	3	0	25.11317053	121.48904610	https://www.inaturalist.org/observations/17236681
2332	Lanius cristatus	Brown Shrike	2018/4/15	2	0	25.11318450	121.48977680	https://www.inaturalist.org/observations/15778085
2333	Lanius cristatus	Brown Shrike	2019/12/9	3	0	25.11330205	121.48982900	https://www.inaturalist.org/observations/54244876
2334	Lanius cristatus	Brown Shrike	2021/3/29	1	0	25.11561495	121.53589830	https://www.inaturalist.org/observations/72344693
2335	Lanius cristatus	Brown Shrike	2020/12/21	2	0	25.11577300	121.49112000	https://www.inaturalist.org/observations/67755193
2336	Lanius cristatus	Brown Shrike	2022/1/20	2	0	25.11867231	121.47214300	https://www.inaturalist.org/observations/105421255
2337	Lanius cristatus	Brown Shrike	2020/3/5	3	0	25.11871422	121.47119520	https://www.inaturalist.org/observations/40942327
2338	Lanius cristatus	Brown Shrike	2020/2/15	3	0	25.11883100	121.47126600	https://www.inaturalist.org/observations/38898429
2339	Lanius cristatus	Brown Shrike	2022/1/20	2	0	25.11883845	121.47151940	https://www.inaturalist.org/observations/105421253
2340	Lanius cristatus lucionensis	Brown Shrike	2021/11/28	0	0	25.11902217	121.47228330	https://www.inaturalist.org/observations/102074322
2341	Lanius cristatus	Brown Shrike	2018/12/26	2	0	25.11913840	121.47201040	https://www.inaturalist.org/observations/33880399
2342	Lanius cristatus	Brown Shrike	2021/4/30	2	0	25.11914800	121.47248600	https://www.inaturalist.org/observations/76987132
2343	Lanius cristatus	Brown Shrike	2021/3/19	1	0	25.12245211	121.52697970	https://www.inaturalist.org/observations/107177027
2344	Lanius cristatus	Brown Shrike	2019/4/20	2	0	25.12616333	121.48742330	https://www.inaturalist.org/observations/22752880
2345	Lanius cristatus lucionensis	Brown Shrike	2021/4/26	0	0	25.12664450	121.48754170	https://www.inaturalist.org/observations/75285363

2346	Lanius cristatus	Brown Shrike	2021/4/2	1	0	25.12678722	121.48775500	https://www.inaturalist.org/observations/72706533
2347	Lanius cristatus	Brown Shrike	2009/10/20	2	0	25.12925344	121.48883560	https://www.inaturalist.org/observations/55377853
2348	Lanius cristatus	Brown Shrike	2008/10/8	1	0	25.13039010	121.49875050	https://www.inaturalist.org/observations/79223152
2349	Lanius cristatus	Brown Shrike	2021/1/1	2	0	25.13395833	121.80627500	https://www.inaturalist.org/observations/67385833
2350	Lanius cristatus	Brown Shrike	2021/11/14	1	0	25.14107960	121.79722590	https://www.inaturalist.org/observations/101059691
2351	Lanius cristatus	Brown Shrike	2022/3/18	1	0	25.14593809	121.80454270	https://www.inaturalist.org/observations/108856491
2352	Lanius cristatus	Brown Shrike	2022/8/25	1	0	25.14622500	121.79853610	https://www.inaturalist.org/observations/132277015
2353	Lanius cristatus	Brown Shrike	2017/12/28	2	0	25.14989273	121.45866710	https://www.inaturalist.org/observations/19940488
2354	Lanius cristatus	Brown Shrike	2022/4/3	1	0	25.15480049	121.45193820	https://www.inaturalist.org/observations/123794501
2355	Lanius cristatus	Brown Shrike	2018/10/1	2	0	25.16313333	121.41718830	https://www.inaturalist.org/observations/19047025
2356	Lanius cristatus	Brown Shrike	2022/4/3	2	0	25.16557423	121.49119750	https://www.inaturalist.org/observations/110282581
2357	Lanius cristatus	Brown Shrike	2021/11/25	1	0	25.17198050	121.44337060	https://www.inaturalist.org/observations/101876629
2358	Lanius cristatus	Brown Shrike	2020/9/1	2	0	25.17856279	121.65121700	https://www.inaturalist.org/observations/58323234
2359	Lanius cristatus	Brown Shrike	2018/11/25	4	0	25.19265640	75.10332514	https://www.inaturalist.org/observations/121574449
2360	Lanius cristatus	Brown Shrike	2021/9/15	1	0	25.22625167	121.64291830	https://www.inaturalist.org/observations/94909131
2361	Lanius cristatus	Brown Shrike	2021/9/15	1	0	25.22637333	121.64284330	https://www.inaturalist.org/observations/94909169
2362	Lanius cristatus	Brown Shrike	2020/10/10	1	0	25.22642274	121.64239770	https://www.inaturalist.org/observations/62276627
2363	Lanius cristatus	Brown Shrike	2010/11/13	2	0	25.22652275	121.64174320	https://www.inaturalist.org/observations/55754493
2364	Lanius cristatus	Brown Shrike	2022/1/10	1	0	25.22845418	121.64221170	https://www.inaturalist.org/observations/104699321
2365	Lanius cristatus	Brown Shrike	2015/10/14	4	0	25.23230900	121.62981700	https://www.inaturalist.org/observations/8750166
2366	Lanius cristatus	Brown Shrike	2020/10/30	1	0	25.31008387	91.90259714	https://www.inaturalist.org/observations/77865591
2367	Lanius cristatus	Brown Shrike	2020/3/19	1	0	25.31167448	91.89880550	https://www.inaturalist.org/observations/103251535
2368	Lanius cristatus	Brown Shrike	2018/12/16	5	0	25.52601990	92.13274910	https://www.inaturalist.org/observations/19859183

2369	Lanius cristatus	Brown Shrike	2021/1/23	3	0	25.55528523	91.90478677	https://www.inaturalist.org/observations/75878319
2370	Lanius cristatus	Brown Shrike	2019/1/24	4	0	25.60358665	91.89191373	https://www.inaturalist.org/observations/19842093
2371	Lanius cristatus	Brown Shrike	2019/12/13	2	0	25.63000526	88.12868111	https://www.inaturalist.org/observations/57908151
2372	Lanius cristatus	Brown Shrike	2022/7/8	0	0	25.70000000	100.10000000	https://www.inaturalist.org/observations/130510005
2373	Lanius cristatus	Brown Shrike	2022/7/6	0	0	25.70000000	100.10000000	https://www.inaturalist.org/observations/130514759
2374	Lanius cristatus	Brown Shrike	2013/7/26	2	0	25.70804937	100.18296120	https://www.inaturalist.org/observations/17168879
2375	Lanius cristatus	Brown Shrike	2013/4/26	1	0	25.76135405	110.12860210	https://www.inaturalist.org/observations/105586701
2376	Lanius cristatus	Brown Shrike	2020/9/14	2	0	25.82715009	118.48213790	https://www.inaturalist.org/observations/59679325
2377	Lanius cristatus	Brown Shrike	2020/9/10	2	0	25.82754843	118.48125180	https://www.inaturalist.org/observations/59677178
2378	Lanius cristatus	Brown Shrike	2011/1/14	2	0	25.84652500	93.42987500	https://www.inaturalist.org/observations/103884131
2379	Lanius cristatus	Brown Shrike	2017/10/19	1	0	25.95199649	119.97291390	https://www.inaturalist.org/observations/13259478
2380	Lanius cristatus	Brown Shrike	2021/12/26	1	0	26.17723724	91.76432122	https://www.inaturalist.org/observations/103677296
2381	Lanius cristatus	Brown Shrike	2022/2/12	2	0	26.19615435	91.69796102	https://www.inaturalist.org/observations/107013751
2382	Lanius cristatus	Brown Shrike	2015/12/16	1	0	26.21765237	91.63311252	https://www.inaturalist.org/observations/90516749
2383	Lanius cristatus lucionensis	Brown Shrike	2019/4/28	2	0	26.21829000	119.97498500	https://www.inaturalist.org/observations/24820036
2384	Lanius cristatus	Brown Shrike	2015/12/16	1	0	26.21895971	91.63298368	https://www.inaturalist.org/observations/129148175
2385	Lanius cristatus	Brown Shrike	2014/3/16	4	0	26.27863958	91.72485352	https://www.inaturalist.org/observations/9482581
2386	Lanius cristatus	Brown Shrike	2011/7/8	2	0	26.32099075	99.84962082	http://www.inaturalist.org/observations/4696423
2387	Lanius cristatus	Brown Shrike	2017/10/29	3	0	26.32700170	89.43855300	https://www.inaturalist.org/observations/58124876
2388	Lanius cristatus	Brown Shrike	2021/5/21	1	0	26.42342173	106.36952590	https://www.inaturalist.org/observations/80365549
2389	Lanius cristatus	Brown Shrike	2021/5/21	1	0	26.42342173	106.36952590	https://www.inaturalist.org/observations/80365563
2390	Lanius cristatus	Brown Shrike	2021/5/21	1	0	26.42342173	106.36952590	https://www.inaturalist.org/observations/80365570
2391	Lanius cristatus	Brown Shrike	2022/5/14	2	0	26.42342173	106.36952590	https://www.inaturalist.org/observations/118212128

2392	Lanius cristatus	Brown Shrike	2022/5/14	1	0	26.42342173	106.36952590	https://www.inaturalist.org/observations/123825434
2393	Lanius cristatus	Brown Shrike	2021/5/21	1	0	26.44083862	106.48329400	https://www.inaturalist.org/observations/80365505
2394	Lanius cristatus	Brown Shrike	2021/5/21	1	0	26.44083862	106.48329400	https://www.inaturalist.org/observations/80367568
2395	Lanius cristatus	Brown Shrike	2021/5/8	1	0	26.46619599	106.67869760	https://www.inaturalist.org/observations/78349351
2396	Lanius cristatus	Brown Shrike	2021/5/8	1	0	26.46619599	106.67869760	https://www.inaturalist.org/observations/78349366
2397	Lanius cristatus	Brown Shrike	2021/5/8	1	0	26.46619599	106.67869760	https://www.inaturalist.org/observations/78349373
2398	Lanius cristatus	Brown Shrike	2021/5/8	1	0	26.46619599	106.67869760	https://www.inaturalist.org/observations/78356774
2399	Lanius cristatus	Brown Shrike	2020/5/9	2	0	26.46889170	106.67842690	https://www.inaturalist.org/observations/45377440
2400	Lanius cristatus	Brown Shrike	2020/5/9	2	0	26.46889170	106.67842690	https://www.inaturalist.org/observations/45377490
2401	Lanius cristatus	Brown Shrike	2022/5/6	1	0	26.50934121	106.64975520	https://www.inaturalist.org/observations/117731315
2402	Lanius cristatus	Brown Shrike	2022/5/6	1	0	26.50934121	106.64975520	https://www.inaturalist.org/observations/117731322
2403	Lanius cristatus	Brown Shrike	2022/11/20	0	0	26.51933620	88.73813121	https://www.inaturalist.org/observations/142457050
2404	Lanius cristatus	Brown Shrike	2019/12/8	3	0	26.52528130	92.99380800	https://www.inaturalist.org/observations/39897402
2405	Lanius cristatus	Brown Shrike	2018/11/15	3	0	26.52528130	92.99380800	https://www.inaturalist.org/observations/69928280
2406	Lanius cristatus	Brown Shrike	2017/11/8	1	0	26.57753100	93.17112200	https://www.inaturalist.org/observations/82655848
2407	Lanius cristatus	Brown Shrike	2018/11/12	1	0	26.57753100	93.17112200	https://www.inaturalist.org/observations/100735663
2408	Lanius cristatus	Brown Shrike	2011/2/27	3	0	26.58990267	93.16069710	https://www.inaturalist.org/observations/55277147
2409	Lanius cristatus	Brown Shrike	2022/10/21	2	0	26.61378838	89.97772809	https://www.inaturalist.org/observations/140401733
2410	Lanius cristatus	Brown Shrike	2022/10/10	3	0	26.62545734	94.35860038	https://www.inaturalist.org/observations/138485826
2411	Lanius cristatus	Brown Shrike	2022/10/16	0	0	26.62602711	94.35869358	https://www.inaturalist.org/observations/139342450
2412	Lanius cristatus	Brown Shrike	2022/5/13	1	0	26.64680980	106.54746180	https://www.inaturalist.org/observations/117750661
2413	Lanius cristatus	Brown Shrike	2018/5/3	3	0	26.64733550	89.54833710	https://www.inaturalist.org/observations/29619329
2414	Lanius cristatus	Brown Shrike	2018/5/3	4	0	26.64766919	89.54829418	https://www.inaturalist.org/observations/29619330

2415	Lanius cristatus	Brown Shrike	2021/11/2	1	0	26.69715347	93.97693176	https://www.inaturalist.org/observations/100077250
2416	Lanius cristatus	Brown Shrike	2022/5/20	1	0	26.73460493	106.65555950	https://www.inaturalist.org/observations/119718912
2417	Lanius cristatus	Brown Shrike	2022/5/20	0	0	26.73460493	106.65555950	https://www.inaturalist.org/observations/119718925
2418	Lanius cristatus	Brown Shrike	2020/11/25	2	0	26.78856434	94.21399679	https://www.inaturalist.org/observations/65619812
2419	Lanius cristatus	Brown Shrike	2021/5/2	1	0	26.79601294	106.89834910	https://www.inaturalist.org/observations/78328292
2420	Lanius cristatus	Brown Shrike	2022/5/10	3	0	26.81517800	88.82479760	https://www.inaturalist.org/observations/118380459
2421	Lanius cristatus	Brown Shrike	2019/3/9	5	0	26.84144933	88.82592570	https://www.inaturalist.org/observations/26903554
2422	Lanius cristatus	Brown Shrike	2014/12/15	2	0	26.84452830	88.33971960	https://www.inaturalist.org/observations/126604485
2423	Lanius cristatus	Brown Shrike	2019/10/1	3	0	26.84953160	91.86521830	https://www.inaturalist.org/observations/33709851
2424	Lanius cristatus	Brown Shrike	2019/10/6	3	0	26.84990770	91.86460830	https://www.inaturalist.org/observations/34001222
2425	Lanius cristatus	Brown Shrike	2020/11/5	2	0	26.85023841	92.77330287	https://www.inaturalist.org/observations/64246952
2426	Lanius cristatus	Brown Shrike	2021/9/9	2	0	26.85198143	88.25151790	https://www.inaturalist.org/observations/94439065
2427	Lanius cristatus	Brown Shrike	2014/12/12	3	0	26.89552695	88.39755474	https://www.inaturalist.org/observations/126599383
2428	Lanius cristatus	Brown Shrike	2020/9/2	1	0	26.94237418	94.15179972	https://www.inaturalist.org/observations/93684285
2429	Lanius cristatus	Brown Shrike	2019/11/21	4	0	26.95662308	75.84354401	https://www.inaturalist.org/observations/35914818
2430	Lanius cristatus	Brown Shrike	2019/10/19	1	0	27.06117639	88.41054440	https://www.inaturalist.org/observations/93213720
2431	Lanius cristatus	Brown Shrike	2008/12/2	3	0	27.15926900	77.52319990	https://www.inaturalist.org/observations/55245734
2432	Lanius cristatus	Brown Shrike	2019/1/4	1	0	27.16647005	77.51560175	https://www.inaturalist.org/observations/120664100
2433	Lanius cristatus	Brown Shrike	1994/1/10	2	0	27.20324720	77.50702240	https://www.inaturalist.org/observations/89164824
2434	Lanius cristatus	Brown Shrike	2022/9/21	1	0	27.48153873	88.50563556	https://www.inaturalist.org/observations/136659550
2435	Lanius cristatus	Brown Shrike	2019/10/10	4	0	27.73505472	96.33909257	https://www.inaturalist.org/observations/39519335
2436	Lanius cristatus	Brown Shrike	2020/11/12	2	0	28.05539165	73.33982315	https://www.inaturalist.org/observations/64732232
2437	Lanius cristatus	Brown Shrike	2021/5/5	1	0	28.12676842	112.92630950	https://www.inaturalist.org/observations/77629717

2438	Lanius cristatus	Brown Shrike	2014/12/19	0	0	28.46770511	76.89167440	https://www.inaturalist.org/observations/144423901
2439	Lanius cristatus	Brown Shrike	2022/9/4	0	0	28.81518745	113.08782200	https://www.inaturalist.org/observations/133529532
2440	Lanius cristatus superciliosus	Brown Shrike	2022/9/4	0	0	28.90583333	105.44777780	https://www.inaturalist.org/observations/133584103
2441	Lanius cristatus	Brown Shrike	2020/2/18	1	0	28.94400616	77.10909091	https://www.inaturalist.org/observations/83430272
2442	Lanius cristatus	Brown Shrike	2022/9/16	0	0	29.47148140	118.66986560	https://www.inaturalist.org/observations/135502952
2443	Lanius cristatus	Brown Shrike	2008/12/19	2	0	29.53001380	78.77466030	https://www.inaturalist.org/observations/79496058
2444	Lanius cristatus	Brown Shrike	2017/5/19	1	0	29.68564800	102.84152100	https://www.inaturalist.org/observations/72087937
2445	Lanius cristatus	Brown Shrike	2017/5/19	1	0	29.68564800	102.84152100	https://www.inaturalist.org/observations/72087940
2446	Lanius cristatus	Brown Shrike	2021/5/12	1	0	29.80158837	121.56270550	https://www.inaturalist.org/observations/80099733
2447	Lanius cristatus	Brown Shrike	2021/5/12	1	0	29.80213038	121.56408650	https://www.inaturalist.org/observations/78666747
2448	Lanius cristatus	Brown Shrike	2021/5/1	1	0	29.81182328	121.53358410	https://www.inaturalist.org/observations/76049521
2449	Lanius cristatus	Brown Shrike	2021/8/18	1	0	29.81259301	121.53427440	https://www.inaturalist.org/observations/92643952
2450	Lanius cristatus	Brown Shrike	2021/2/8	1	0	30.08692810	78.26761160	https://www.inaturalist.org/observations/101884209
2451	Lanius cristatus	Brown Shrike	2022/9/5	1	0	30.23864740	119.75016590	https://www.inaturalist.org/observations/133744623
2452	Lanius cristatus	Brown Shrike	2019/9/15	2	0	30.26113000	120.14715550	https://www.inaturalist.org/observations/32761844
2453	Lanius cristatus	Brown Shrike	2022/5/13	1	0	30.28808431	114.27283170	https://www.inaturalist.org/observations/120629220
2454	Lanius cristatus	Brown Shrike	2020/5/23	2	0	30.39137208	103.80448020	https://www.inaturalist.org/observations/46924811
2455	Lanius cristatus	Brown Shrike	2019/9/12	1	0	30.44647707	114.40798820	https://www.inaturalist.org/observations/111394827
2456	Lanius cristatus	Brown Shrike	2022/9/17	0	0	30.54253735	114.36711160	https://www.inaturalist.org/observations/138473094
2457	Lanius cristatus	Brown Shrike	2020/7/12	1	0	30.63953933	104.19066050	https://www.inaturalist.org/observations/52781688
2458	Lanius cristatus	Brown Shrike	2022/9/6	1	0	30.93756839	121.87065140	https://www.inaturalist.org/observations/136660680
2459	Lanius cristatus	Brown Shrike	2020/5/7	2	0	31.11845386	117.17324480	https://www.inaturalist.org/observations/45163631
2460	Lanius cristatus	Brown Shrike	2020/5/10	3	0	31.11845581	117.17324360	https://www.inaturalist.org/observations/45449691

2461	Lanius cristatus	Brown Shrike	2020/5/10	2	0	31.11845581	117.17324360	https://www.inaturalist.org/observations/45450997
2462	Lanius cristatus	Brown Shrike	2020/5/10	3	0	31.11845581	117.17324360	https://www.inaturalist.org/observations/45451019
2463	Lanius cristatus	Brown Shrike	2018/9/4	2	0	31.22482485	121.41173140	https://www.inaturalist.org/observations/25152460
2464	Lanius cristatus	Brown Shrike	2017/5/13	3	0	31.31182038	119.71809370	https://www.inaturalist.org/observations/6715556
2465	Lanius cristatus	Brown Shrike	2021/5/25	2	0	31.34591667	121.35123330	https://www.inaturalist.org/observations/80217822
2466	Lanius cristatus	Brown Shrike	2021/5/18	1	0	31.40000000	119.40000000	https://www.inaturalist.org/observations/82256429
2467	Lanius cristatus	Brown Shrike	2021/5/23	1	0	31.49195884	121.34937970	https://www.inaturalist.org/observations/81023225
2468	Lanius cristatus	Brown Shrike	2020/5/20	2	0	31.49762391	120.44113480	https://www.inaturalist.org/observations/65411834
2469	Lanius cristatus	Brown Shrike	2018/6/13	2	0	31.74552048	120.92736480	https://www.inaturalist.org/observations/13416885
2470	Lanius cristatus	Brown Shrike	2019/9/14	2	0	31.75116230	117.27189300	https://www.inaturalist.org/observations/32686499
2471	Lanius cristatus	Brown Shrike	2021/8/25	1	0	31.81937000	118.52043330	https://www.inaturalist.org/observations/92647051
2472	Lanius cristatus	Brown Shrike	2021/6/12	1	0	31.93790667	119.17396170	https://www.inaturalist.org/observations/88347823
2473	Lanius cristatus	Brown Shrike	2022/5/22	1	0	31.97553167	118.63122160	https://www.inaturalist.org/observations/118484239
2474	Lanius cristatus	Brown Shrike	2021/5/27	2	0	31.98408667	118.77962500	https://www.inaturalist.org/observations/80446464
2475	Lanius cristatus	Brown Shrike	2022/5/22	1	0	31.98555500	118.63399330	https://www.inaturalist.org/observations/118481144
2476	Lanius cristatus	Brown Shrike	2022/5/8	1	0	32.00782667	118.63955490	https://www.inaturalist.org/observations/117969549
2477	Lanius cristatus	Brown Shrike	2022/6/9	1	0	32.03789529	118.66126950	https://www.inaturalist.org/observations/121375886
2478	Lanius cristatus	Brown Shrike	2022/5/14	1	0	32.04426833	118.71171830	https://www.inaturalist.org/observations/117324157
2479	Lanius cristatus	Brown Shrike	2022/5/8	2	0	32.05279831	118.66822000	https://www.inaturalist.org/observations/117969463
2480	Lanius cristatus	Brown Shrike	2022/5/20	1	0	32.05437897	118.49732300	https://www.inaturalist.org/observations/117947721
2481	Lanius cristatus	Brown Shrike	2022/6/22	2	0	32.13681969	118.75953390	https://www.inaturalist.org/observations/123032895
2482	Lanius cristatus	Brown Shrike	2022/6/25	1	0	32.15393360	118.76455800	https://www.inaturalist.org/observations/123829051
2483	Lanius cristatus	Brown Shrike	2021/6/29	1	0	32.19477667	117.10107670	https://www.inaturalist.org/observations/87946356

2484	Lanius cristatus	Brown Shrike	2022/4/30	1	0	32.20946904	119.01955720	https://www.inaturalist.org/observations/114084131
2485	Lanius cristatus	Brown Shrike	2021/9/4	2	0	32.24382200	77.17466300	https://www.inaturalist.org/observations/93559792
2486	Lanius cristatus	Brown Shrike	2020/8/30	0	0	32.42362976	119.56932070	https://www.inaturalist.org/observations/137733119
2487	Lanius cristatus	Brown Shrike	2021/7/17	1	0	32.60290833	115.83803830	https://www.inaturalist.org/observations/91264629
2488	Lanius cristatus	Brown Shrike	2021/7/17	1	0	32.60290833	115.83803830	https://www.inaturalist.org/observations/91264633
2489	Lanius cristatus	Brown Shrike	2021/7/17	1	0	32.64497000	115.60522500	https://www.inaturalist.org/observations/91264580
2490	Lanius cristatus	Brown Shrike	2021/7/17	1	0	32.64497000	115.60522500	https://www.inaturalist.org/observations/91264581
2491	Lanius cristatus	Brown Shrike	2022/5/25	2	0	32.86779370	120.82090670	https://www.inaturalist.org/observations/118649415
2492	Lanius cristatus	Brown Shrike	2020/7/10	2	0	34.08600723	74.84979920	https://www.inaturalist.org/observations/61381083
2493	Lanius cristatus	Brown Shrike	2021/8/21	1	0	34.59001833	113.84936170	https://www.inaturalist.org/observations/92213523
2494	Lanius cristatus	Brown Shrike	2021/7/18	1	0	34.59793500	113.85109500	https://www.inaturalist.org/observations/87531671
2495	Lanius cristatus	Brown Shrike	2022/6/12	1	0	34.59802000	113.85134830	https://www.inaturalist.org/observations/123362244
2496	Lanius cristatus	Brown Shrike	2021/7/28	1	0	34.59810336	113.85198760	https://www.inaturalist.org/observations/88928733
2497	Lanius cristatus	Brown Shrike	2021/7/31	1	0	34.59859353	113.85280200	https://www.inaturalist.org/observations/89473665
2498	Lanius cristatus	Brown Shrike	2021/8/7	1	0	34.59874667	113.85234170	https://www.inaturalist.org/observations/90484474
2499	Lanius cristatus	Brown Shrike	2022/5/14	1	0	34.59988396	113.86006330	https://www.inaturalist.org/observations/116967034
2500	Lanius cristatus	Brown Shrike	2022/5/14	1	0	34.59998833	113.85062500	https://www.inaturalist.org/observations/116967138
2501	Lanius cristatus	Brown Shrike	2022/5/14	1	0	34.60011333	113.85792330	https://www.inaturalist.org/observations/116967104
2502	Lanius cristatus	Brown Shrike	2022/5/14	1	0	34.60020833	113.85780000	https://www.inaturalist.org/observations/116967094
2503	Lanius cristatus	Brown Shrike	2022/5/14	1	0	34.60239201	113.85892610	https://www.inaturalist.org/observations/116967036
2504	Lanius cristatus	Brown Shrike	2022/6/12	1	0	34.60255833	113.85824000	https://www.inaturalist.org/observations/121852706
2505	Lanius cristatus	Brown Shrike	2022/6/12	1	0	34.60256167	113.85823000	https://www.inaturalist.org/observations/121852704
2506	Lanius cristatus	Brown Shrike	2022/8/17	1	0	34.72539000	113.57583350	https://www.inaturalist.org/observations/131322561

2507	Lanius cristatus	Brown Shrike	2022/7/11	1	0	34.76053845	113.65922320	https://www.inaturalist.org/observations/125774236
2508	Lanius cristatus	Brown Shrike	2022/9/1	0	0	34.76279833	113.68320170	https://www.inaturalist.org/observations/133195952
2509	Lanius cristatus	Brown Shrike	2021/6/9	1	0	34.76430667	113.66330670	https://www.inaturalist.org/observations/82264764
2510	Lanius cristatus	Brown Shrike	2009/8/2	2	0	34.95141144	113.51264330	https://www.inaturalist.org/observations/20309060
2511	Lanius cristatus	Brown Shrike	2021/7/4	2	0	35.11307390	116.58726570	https://www.inaturalist.org/observations/85610380
2512	Lanius cristatus	Brown Shrike	2022/7/30	0	0	35.55296015	118.43347620	https://www.inaturalist.org/observations/144031945
2513	Lanius cristatus	Brown Shrike	2022/7/30	1	0	35.55296015	118.43347620	https://www.inaturalist.org/observations/144031968
2514	Lanius cristatus	Brown Shrike	2022/7/31	0	0	35.55296015	118.43347620	https://www.inaturalist.org/observations/144031983
2515	Lanius cristatus	Brown Shrike	2022/7/31	0	0	35.55296015	118.43347620	https://www.inaturalist.org/observations/144031989
2516	Lanius cristatus	Brown Shrike	2019/5/26	2	0	35.56604427	113.80735870	https://www.inaturalist.org/observations/25802062
2517	Lanius cristatus	Brown Shrike	2019/8/22	3	0	36.06652044	120.43133520	https://www.inaturalist.org/observations/31263317
2518	Lanius cristatus	Brown Shrike	2019/8/23	2	0	36.09502133	120.47706480	https://www.inaturalist.org/observations/31336119
2519	Lanius cristatus	Brown Shrike	2020/5/16	2	0	36.20737000	120.40872500	https://www.inaturalist.org/observations/46075766
2520	Lanius cristatus	Brown Shrike	2019/5/12	1	0	36.37019000	120.68083100	https://www.inaturalist.org/observations/106869706
2521	Lanius cristatus	Brown Shrike	2020/9/21	1	0	36.37035100	120.68579400	https://www.inaturalist.org/observations/105263330
2522	Lanius cristatus	Brown Shrike	2019/5/21	1	0	36.37224600	120.68051500	https://www.inaturalist.org/observations/106916225
2523	Lanius cristatus	Brown Shrike	2021/5/8	1	0	36.37641200	120.68096100	https://www.inaturalist.org/observations/105432607
2524	Lanius cristatus	Brown Shrike	2012/5/5	1	0	36.67418100	117.02628900	https://www.inaturalist.org/observations/103203988
2525	Lanius cristatus	Brown Shrike	2021/5/7	2	0	37.39593060	121.33203640	https://www.inaturalist.org/observations/143824332
2526	Lanius cristatus	Brown Shrike	2021/5/14	1	0	38.68361500	117.48146500	https://www.inaturalist.org/observations/79203843
2527	Lanius cristatus	Brown Shrike	2021/5/13	1	0	38.77425509	117.39527130	https://www.inaturalist.org/observations/78902114
2528	Lanius cristatus	Brown Shrike	2021/5/13	1	0	38.78684333	117.39418830	https://www.inaturalist.org/observations/78828132
2529	Lanius cristatus	Brown Shrike	2021/5/16	1	0	39.13631383	118.26759500	https://www.inaturalist.org/observations/78955757

2530	Lanius cristatus	Brown Shrike	2021/5/10	1	0	39.15805667	118.25784000	https://www.inaturalist.org/observations/78284553
2531	Lanius cristatus	Brown Shrike	2020/7/15	0	0	39.70564700	116.32164800	https://www.inaturalist.org/observations/144468083
2532	Lanius cristatus	Brown Shrike	2022/6/4	1	0	39.73627292	116.91502470	https://www.inaturalist.org/observations/120945893
2533	Lanius cristatus	Brown Shrike	2012/5/6	2	0	39.82286969	119.51077240	https://www.inaturalist.org/observations/36408522
2534	Lanius cristatus	Brown Shrike	2013/9/20	2	0	39.90021729	119.54064320	https://www.inaturalist.org/observations/59373947
2535	Lanius cristatus	Brown Shrike	2022/5/14	1	0	39.90077814	116.78292170	https://www.inaturalist.org/observations/118004281
2536	Lanius cristatus	Brown Shrike	2021/8/26	1	0	39.97891990	119.77606000	https://www.inaturalist.org/observations/123852022
2537	Lanius cristatus	Brown Shrike	2022/5/20	2	0	39.99694771	116.30491350	https://www.inaturalist.org/observations/117828039
2538	Lanius cristatus	Brown Shrike	2022/9/3	1	0	40.01464407	116.38842810	https://www.inaturalist.org/observations/138498352
2539	Lanius cristatus	Brown Shrike	2022/5/2	1	0	40.01899144	116.39257700	https://www.inaturalist.org/observations/115224632
2540	Lanius cristatus	Brown Shrike	2021/7/30	1	0	40.10957164	116.71435120	https://www.inaturalist.org/observations/89282196
2541	Lanius cristatus	Brown Shrike	2020/5/14	2	0	40.11033667	116.69853830	https://www.inaturalist.org/observations/45971371
2542	Lanius cristatus	Brown Shrike	2019/6/6	3	0	40.11597000	116.69803500	https://www.inaturalist.org/observations/26918049
2543	Lanius cristatus	Brown Shrike	2020/8/16	3	0	40.11611658	116.70389820	https://www.inaturalist.org/observations/57454959
2544	Lanius cristatus	Brown Shrike	2020/8/16	3	0	40.11612096	116.70421090	https://www.inaturalist.org/observations/57454964
2545	Lanius cristatus	Brown Shrike	2021/6/26	2	0	40.11663671	116.70474040	https://www.inaturalist.org/observations/86032099
2546	Lanius cristatus cristatus	Brown Shrike	2019/5/26	3	0	40.11953833	116.69476670	https://www.inaturalist.org/observations/25942791
2547	Lanius cristatus	Brown Shrike	2019/5/26	2	0	40.12087000	116.69549000	https://www.inaturalist.org/observations/25942295
2548	Lanius cristatus	Brown Shrike	2019/6/2	2	0	40.12146333	116.69386500	https://www.inaturalist.org/observations/26379293
2549	Lanius cristatus	Brown Shrike	2019/5/26	3	0	40.12164333	116.69423500	https://www.inaturalist.org/observations/25942978
2550	Lanius cristatus	Brown Shrike	2019/5/26	3	0	40.12164333	116.69423500	https://www.inaturalist.org/observations/25942984
2551	Lanius cristatus	Brown Shrike	2019/5/26	3	0	40.12165333	116.69422170	https://www.inaturalist.org/observations/25942798
2552	Lanius cristatus	Brown Shrike	2019/5/26	2	0	40.12165667	116.69422000	https://www.inaturalist.org/observations/25942160

2553	Lanius cristatus	Brown Shrike	2018/8/20	2	0	40.13034700	116.65465000	https://www.inaturalist.org/observations/39608138
2554	Lanius cristatus	Brown Shrike	2018/8/19	2	0	40.13034700	116.65465000	https://www.inaturalist.org/observations/39608139
2555	Lanius cristatus	Brown Shrike	2021/8/27	1	0	40.13304615	116.31133980	https://www.inaturalist.org/observations/122030310
2556	Lanius cristatus	Brown Shrike	2021/8/30	1	0	40.13345818	116.29986590	https://www.inaturalist.org/observations/143235849
2557	Lanius cristatus	Brown Shrike	2021/9/11	1	0	40.13570499	116.29124890	https://www.inaturalist.org/observations/94421507
2558	Lanius cristatus	Brown Shrike	2019/5/18	2	0	40.17742833	116.67585000	https://www.inaturalist.org/observations/25624346
2559	Lanius cristatus	Brown Shrike	2021/9/5	1	0	40.18293590	116.67409730	https://www.inaturalist.org/observations/94319406
2560	Lanius cristatus	Brown Shrike	2022/8/26	1	0	40.36026600	116.00924800	https://www.inaturalist.org/observations/133481009
2561	Lanius cristatus	Brown Shrike	2021/8/27	1	0	40.38536530	115.83426430	https://www.inaturalist.org/observations/122030320
2562	Lanius cristatus confusus	Brown Shrike	2017/8/26	0	0	40.40536686	115.84512470	https://www.inaturalist.org/observations/8462844
2563	Lanius cristatus	Brown Shrike	2022/9/16	0	0	40.54371792	117.01492410	https://www.inaturalist.org/observations/135610040
2564	Lanius cristatus	Brown Shrike	2022/9/16	0	0	40.54403468	117.01454120	https://www.inaturalist.org/observations/135609988
2565	Lanius cristatus lucionensis	Brown Shrike	2014/6/20	1	0	41.04131965	121.70218720	https://www.inaturalist.org/observations/66869744
2566	Lanius cristatus	Brown Shrike	2016/6/18	2	0	42.77883118	123.27116710	https://www.inaturalist.org/observations/66884710
2567	Lanius cristatus	Brown Shrike	2022/7/17	1	0	43.62333452	126.17217280	https://www.inaturalist.org/observations/127839368
2568	Lanius cristatus	Brown Shrike	2019/7/4	1	0	43.98969071	125.35785370	https://www.inaturalist.org/observations/103744189
2569	Lanius cristatus	Brown Shrike	2007/5/21	2	0	47.02839700	129.02079300	https://www.inaturalist.org/observations/65334541
2570	Lanius cristatus	Brown Shrike	2007/5/21	2	0	47.02839700	129.02079300	https://www.inaturalist.org/observations/65334544
2571	Lanius cristatus	Brown Shrike	2007/5/22	2	0	47.02839700	129.02079300	https://www.inaturalist.org/observations/65335712
2572	Lanius cristatus	Brown Shrike	2006/6/1	2	0	47.02839700	129.02079300	https://www.inaturalist.org/observations/65470645

No	scientific name	common name	date	NIA	NID	latitude	longitude	url
1	Pitta brachyura	Indian Pitta	2019/11/7	5	0	6.22418210	80.11383690	https://www.inaturalist.org/observations/35452878
2	Pitta brachyura	Indian Pitta	2020/11/1	8	0	6.23051895	80.11060282	https://www.inaturalist.org/observations/64459965
3	Pitta brachyura	Indian Pitta	2020/11/11	8	0	6.23051895	80.11060282	https://www.inaturalist.org/observations/65029577
4	Pitta brachyura	Indian Pitta	2019/10/24	2	0	6.23059397	80.11046302	https://www.inaturalist.org/observations/34825614
5	Pitta brachyura	Indian Pitta	2018/10/17	5	0	6.26805833	81.35912833	https://www.inaturalist.org/observations/42732105
6	Pitta brachyura	Indian Pitta	2020/1/28	2	0	6.27841200	81.40194100	https://www.inaturalist.org/observations/38542456
7	Pitta brachyura	Indian Pitta	2022/2/17	2	0	6.31305715	81.42936587	https://www.inaturalist.org/observations/107457903
8	Pitta brachyura	Indian Pitta	2022/2/11	1	0	6.34846944	80.56666667	https://www.inaturalist.org/observations/144613165
9	Pitta brachyura	Indian Pitta	2021/12/20	4	0	6.41954613	80.86661530	https://www.inaturalist.org/observations/107902180
10	Pitta brachyura	Indian Pitta	2021/11/1	5	0	6.74757920	80.36864880	https://www.inaturalist.org/observations/101308794
11	Pitta brachyura	Indian Pitta	2020/1/3	4	0	6.90080434	81.06509699	https://www.inaturalist.org/observations/37294937
12	Pitta brachyura	Indian Pitta	2016/11/14	6	0	6.94971660	80.78910680	https://www.inaturalist.org/observations/125401137
13	Pitta brachyura	Indian Pitta	2015/2/17	3	0	6.96810457	80.76725861	https://www.inaturalist.org/observations/36774764
14	Pitta brachyura	Indian Pitta	2014/11/24	6	0	6.96875811	80.76844590	https://www.inaturalist.org/observations/70983316
15	Pitta brachyura	Indian Pitta	2011/1/17	5	0	6.96875957	80.76814544	https://www.inaturalist.org/observations/57826465
16	Pitta brachyura	Indian Pitta	2017/2/9	4	0	6.96951197	80.76814485	https://www.inaturalist.org/observations/80468090
17	Pitta brachyura	Indian Pitta	2018/12/7	4	0	6.96978788	80.76908458	https://www.inaturalist.org/observations/108652639
18	Pitta brachyura	Indian Pitta	2011/1/16	5	0	6.96979428	80.76802350	https://www.inaturalist.org/observations/57825702
19	Pitta brachyura	Indian Pitta	2013/11/23	7	0	6.97018700	80.76902700	https://www.inaturalist.org/observations/6505620
20	Pitta brachyura	Indian Pitta	2017/12/31	6	0	6.97169770	80.76833577	https://www.inaturalist.org/observations/15184951
21	Pitta brachyura	Indian Pitta	2017/12/31	6	0	6.97169770	80.76833577	https://www.inaturalist.org/observations/15184952
22	Pitta brachyura	Indian Pitta	2018/1/1	6	0	6.97169770	80.76833577	https://www.inaturalist.org/observations/15184953

23	Pitta brachyura	Indian Pitta	2015/1/17	7	0	6.98654700	80.42404200	https://www.inaturalist.org/observations/35084649
24	Pitta brachyura	Indian Pitta	2014/11/15	7	0	6.98793781	80.40601203	https://www.inaturalist.org/observations/70983347
25	Pitta brachyura	Indian Pitta	2012/3/10	5	0	6.98978450	80.42708850	https://www.inaturalist.org/observations/64664834
26	Pitta brachyura	Indian Pitta	2012/3/11	5	0	6.98978450	80.42708850	https://www.inaturalist.org/observations/64664914
27	Pitta brachyura	Indian Pitta	2021/12/12	3	0	6.99823480	80.40944220	https://www.inaturalist.org/observations/108640025
28	Pitta brachyura	Indian Pitta	2022/1/28	4	0	7.19124632	79.90239199	https://www.inaturalist.org/observations/133643741
29	Pitta brachyura	Indian Pitta	2021/1/30	6	0	7.23643803	80.31188082	https://www.inaturalist.org/observations/89150547
30	Pitta brachyura	Indian Pitta	2019/1/26	8	0	7.27213667	80.59504833	https://www.inaturalist.org/observations/34059496
31	Pitta brachyura	Indian Pitta	2022/4/12	1	0	7.27800556	80.21505259	https://www.inaturalist.org/observations/111891085
32	Pitta brachyura	Indian Pitta	2014/12/26	6	0	7.27870738	80.21439350	https://www.inaturalist.org/observations/33178690
33	Pitta brachyura	Indian Pitta	2020/3/23	6	0	7.29073528	80.63372612	https://www.inaturalist.org/observations/62020347
34	Pitta brachyura	Indian Pitta	2019/4/12	7	0	7.29475900	80.71190000	https://www.inaturalist.org/observations/22637444
35	Pitta brachyura	Indian Pitta	2018/11/20	4	0	7.29530810	80.66321030	https://www.inaturalist.org/observations/52445621
36	Pitta brachyura	Indian Pitta	2022/3/14	4	0	7.29613000	80.64378833	https://www.inaturalist.org/observations/109908795
37	Pitta brachyura	Indian Pitta	2015/11/16	5	0	7.30329573	80.64245566	https://www.inaturalist.org/observations/38067465
38	Pitta brachyura	Indian Pitta	2019/10/30	6	0	7.30344833	80.64323667	https://www.inaturalist.org/observations/41382368
39	Pitta brachyura	Indian Pitta	2022/3/4	5	0	7.47759988	80.32682771	https://www.inaturalist.org/observations/107862936
40	Pitta brachyura	Indian Pitta	2019/4/16	7	0	7.63247298	80.49803303	https://www.inaturalist.org/observations/22587466
41	Pitta brachyura	Indian Pitta	2014/12/17	5	0	7.87305400	80.77179700	https://www.inaturalist.org/observations/93801785
42	Pitta brachyura	Indian Pitta	2018/12/2	2	0	7.99624165	80.64150500	https://www.inaturalist.org/observations/121604031
43	Pitta brachyura	Indian Pitta	2018/12/16	7	0	8.03269013	80.74722440	https://www.inaturalist.org/observations/19569699
44	Pitta brachyura	Indian Pitta	2018/12/15	7	0	8.03465549	80.74704201	https://www.inaturalist.org/observations/19568924
45	Pitta brachyura	Indian Pitta	2019/11/16	8	0	8.04518617	79.85135500	https://www.inaturalist.org/observations/49329428

46	Pitta brachyura	Indian Pitta	2019/4/23	8	0	8.31202685	79.96946058	https://www.inaturalist.org/observations/22966824
47	Pitta brachyura	Indian Pitta	2021/10/17	2	0	8.32972514	77.50839798	https://www.inaturalist.org/observations/102798893
48	Pitta brachyura	Indian Pitta	2022/4/7	1	0	8.50757542	77.40882379	https://www.inaturalist.org/observations/142127219
49	Pitta brachyura	Indian Pitta	2021/4/15	7	0	8.58649761	77.02874132	https://www.inaturalist.org/observations/81521176
50	Pitta brachyura	Indian Pitta	2021/1/9	6	0	8.58651849	77.02900752	https://www.inaturalist.org/observations/81323304
51	Pitta brachyura	Indian Pitta	2016/2/3	5	0	8.70446035	77.27187356	https://www.inaturalist.org/observations/57533156
52	Pitta brachyura	Indian Pitta	2012/1/26	7	0	8.76736865	77.08084121	https://www.inaturalist.org/observations/82547234
53	Pitta brachyura	Indian Pitta	2016/2/14	4	0	8.85782870	77.21752230	https://www.inaturalist.org/observations/36321612
54	Pitta brachyura	Indian Pitta	2021/1/29	4	0	8.94900694	77.10936515	https://www.inaturalist.org/observations/116255338
55	Pitta brachyura	Indian Pitta	2022/2/26	5	0	8.96115117	77.03777632	https://www.inaturalist.org/observations/110923147
56	Pitta brachyura	Indian Pitta	2014/1/28	7	0	8.97506300	77.14944100	http://www.inaturalist.org/observations/520264
57	Pitta brachyura	Indian Pitta	2020/4/10	5	0	9.01761170	76.92609360	https://www.inaturalist.org/observations/41924938
58	Pitta brachyura	Indian Pitta	2017/2/12	6	0	9.26475820	76.78704140	https://www.inaturalist.org/observations/31912029
59	Pitta brachyura	Indian Pitta	2017/2/12	3	0	9.31590375	76.78785057	https://www.inaturalist.org/observations/127534959
60	Pitta brachyura	Indian Pitta	2013/1/5	3	0	9.45636096	77.18277407	https://www.inaturalist.org/observations/73936556
61	Pitta brachyura	Indian Pitta	2013/2/19	6	0	9.49782600	77.18444800	https://www.inaturalist.org/observations/9789153
62	Pitta brachyura	Indian Pitta	2015/1/27	6	0	9.49915100	77.17170200	https://www.inaturalist.org/observations/35325684
63	Pitta brachyura	Indian Pitta	2022/12/24	1	0	9.55111597	76.58494335	https://www.inaturalist.org/observations/144984130
64	Pitta brachyura	Indian Pitta	2014/2/18	6	0	9.69059098	80.01837753	https://www.inaturalist.org/observations/57833143
65	Pitta brachyura	Indian Pitta	2021/4/24	4	0	9.69062768	80.01858193	https://www.inaturalist.org/observations/75384848
66	Pitta brachyura	Indian Pitta	2021/12/5	6	0	9.83967275	76.78260484	https://www.inaturalist.org/observations/102768158
67	Pitta brachyura	Indian Pitta	2018/12/7	4	0	9.88868643	76.72812668	https://www.inaturalist.org/observations/91832929
68	Pitta brachyura	Indian Pitta	2018/12/8	5	0	9.88868643	76.72812668	https://www.inaturalist.org/observations/91832949

69	Pitta brachyura	Indian Pitta	2019/1/12	3	0	9.88868643	76.72812668	https://www.inaturalist.org/observations/97462860
70	Pitta brachyura	Indian Pitta	2022/2/26	4	0	9.89042690	76.69041728	https://www.inaturalist.org/observations/108286757
71	Pitta brachyura	Indian Pitta	2021/11/17	3	0	9.89081268	76.69041719	https://www.inaturalist.org/observations/101318616
72	Pitta brachyura	Indian Pitta	2022/2/12	5	0	9.89081268	76.69041719	https://www.inaturalist.org/observations/106805724
73	Pitta brachyura	Indian Pitta	2022/2/15	3	0	9.89081268	76.69041719	https://www.inaturalist.org/observations/106807384
74	Pitta brachyura	Indian Pitta	2022/4/19	2	0	9.89081268	76.69041719	https://www.inaturalist.org/observations/114307671
75	Pitta brachyura	Indian Pitta	2022/2/28	6	0	9.89084968	76.69077133	https://www.inaturalist.org/observations/108288382
76	Pitta brachyura	Indian Pitta	2022/2/18	4	0	10.03271500	76.33188900	https://www.inaturalist.org/observations/107040701
77	Pitta brachyura	Indian Pitta	2020/10/26	4	0	10.06058039	78.77006762	https://www.inaturalist.org/observations/76862243
78	Pitta brachyura	Indian Pitta	2018/12/16	6	0	10.10398030	76.70046050	https://www.inaturalist.org/observations/32152987
79	Pitta brachyura	Indian Pitta	2014/11/3	6	0	10.10398030	76.70046050	https://www.inaturalist.org/observations/70983379
80	Pitta brachyura	Indian Pitta	2018/3/3	5	0	10.10398030	76.70046050	https://www.inaturalist.org/observations/91649466
81	Pitta brachyura	Indian Pitta	2020/2/1	6	0	10.10398043	76.70046039	https://www.inaturalist.org/observations/38183624
82	Pitta brachyura	Indian Pitta	2017/2/12	4	0	10.11646740	76.67267890	https://www.inaturalist.org/observations/43904867
83	Pitta brachyura	Indian Pitta	2016/12/6	7	0	10.11709483	76.66989909	http://www.inaturalist.org/observations/4731094
84	Pitta brachyura	Indian Pitta	2017/12/17	7	0	10.12067400	76.75534700	https://www.inaturalist.org/observations/9914862
85	Pitta brachyura	Indian Pitta	2015/2/22	2	0	10.12903097	76.70651617	https://www.inaturalist.org/observations/44813529
86	Pitta brachyura	Indian Pitta	2018/4/5	5	0	10.12965278	76.68793889	https://www.inaturalist.org/observations/66526735
87	Pitta brachyura	Indian Pitta	2016/1/28	4	0	10.12979100	76.68711800	https://www.inaturalist.org/observations/7278942
88	Pitta brachyura	Indian Pitta	2014/12/23	3	0	10.12979140	76.68711840	https://www.inaturalist.org/observations/105781620
89	Pitta brachyura	Indian Pitta	2015/1/11	8	0	10.13000571	76.68728661	https://www.inaturalist.org/observations/83604919
90	Pitta brachyura	Indian Pitta	2022/12/10	2	0	10.13061022	76.68780673	https://www.inaturalist.org/observations/144141604
91	Pitta brachyura	Indian Pitta	2020/3/30	7	0	10.13098780	76.24104930	https://www.inaturalist.org/observations/41220153

92	Pitta brachyura	Indian Pitta	2020/4/1	5	0	10.24457702	76.35896024	https://www.inaturalist.org/observations/43541091
93	Pitta brachyura	Indian Pitta	2015/3/8	5	0	10.29336111	76.47048355	https://www.inaturalist.org/observations/46834843
94	Pitta brachyura	Indian Pitta	2017/3/9	4	0	10.32697340	76.95539670	https://www.inaturalist.org/observations/91817748
95	Pitta brachyura	Indian Pitta	2021/1/4	3	0	10.33141665	76.40064893	https://www.inaturalist.org/observations/67565844
96	Pitta brachyura	Indian Pitta	2016/12/23	5	0	10.35291658	77.21363068	https://www.inaturalist.org/observations/65120275
97	Pitta brachyura	Indian Pitta	2016/10/26	7	0	10.48510800	77.74806900	http://www.inaturalist.org/observations/4681070
98	Pitta brachyura	Indian Pitta	2021/4/8	4	0	10.52767097	76.34909197	https://www.inaturalist.org/observations/100228245
99	Pitta brachyura	Indian Pitta	2021/1/30	2	0	10.60074639	76.21713102	https://www.inaturalist.org/observations/139055606
100	Pitta brachyura	Indian Pitta	2018/2/7	5	0	10.61194637	76.35991738	https://www.inaturalist.org/observations/95834840
101	Pitta brachyura	Indian Pitta	2019/2/10	6	0	10.63174793	76.37262169	https://www.inaturalist.org/observations/48620065
102	Pitta brachyura	Indian Pitta	2019/3/23	5	0	10.73945143	76.15415409	https://www.inaturalist.org/observations/102456243
103	Pitta brachyura	Indian Pitta	2016/12/28	7	0	10.74753300	76.46716500	https://www.inaturalist.org/observations/87008573
104	Pitta brachyura	Indian Pitta	2014/3/22	5	0	10.74974237	76.37860390	https://www.inaturalist.org/observations/62118247
105	Pitta brachyura	Indian Pitta	2014/3/22	4	0	10.74974237	76.37860390	https://www.inaturalist.org/observations/98107003
106	Pitta brachyura	Indian Pitta	2014/3/22	5	0	10.74974237	76.37860390	https://www.inaturalist.org/observations/98107006
107	Pitta brachyura	Indian Pitta	2022/3/18	3	0	10.81723333	76.75621500	https://www.inaturalist.org/observations/115064553
108	Pitta brachyura	Indian Pitta	2018/12/28	5	0	10.82129581	76.64155137	https://www.inaturalist.org/observations/96858626
109	Pitta brachyura	Indian Pitta	2022/2/20	2	0	10.85834860	76.65722083	https://www.inaturalist.org/observations/136017722
110	Pitta brachyura	Indian Pitta	2018/12/9	6	0	10.88777170	76.07321890	https://www.inaturalist.org/observations/67399487
111	Pitta brachyura	Indian Pitta	2021/4/7	5	0	10.89101830	76.06852558	https://www.inaturalist.org/observations/73177451
112	Pitta brachyura	Indian Pitta	2019/2/16	3	0	11.01684450	76.95583210	https://www.inaturalist.org/observations/137613529
113	Pitta brachyura	Indian Pitta	2022/11/11	1	0	11.05044313	76.85487529	https://www.inaturalist.org/observations/141964438
114	Pitta brachyura	Indian Pitta	2020/10/26	6	0	11.07894586	79.83002432	https://www.inaturalist.org/observations/63578651

115	Pitta brachyura	Indian Pitta	2021/11/4	4	0	11.07894586	79.83002432	https://www.inaturalist.org/observations/100227211
116	Pitta brachyura	Indian Pitta	2018/1/28	4	0	11.08874798	76.45534521	https://www.inaturalist.org/observations/65731183
117	Pitta brachyura	Indian Pitta	2019/1/17	3	0	11.23986174	79.29021332	https://www.inaturalist.org/observations/54530035
118	Pitta brachyura	Indian Pitta	2011/3/9	2	0	11.35300220	76.79590950	https://www.inaturalist.org/observations/95520211
119	Pitta brachyura	Indian Pitta	2021/3/23	4	0	11.40664871	75.73826920	https://www.inaturalist.org/observations/79648465
120	Pitta brachyura	Indian Pitta	2020/10/21	4	0	11.42176660	76.86172750	https://www.inaturalist.org/observations/88796554
121	Pitta brachyura	Indian Pitta	2012/11/18	7	0	11.53174400	76.64753300	https://www.inaturalist.org/observations/133870427
122	Pitta brachyura	Indian Pitta	2014/1/21	3	0	11.55643790	76.64076801	https://www.inaturalist.org/observations/68292611
123	Pitta brachyura	Indian Pitta	2022/3/13	6	0	11.55956743	76.22497179	https://www.inaturalist.org/observations/125460591
124	Pitta brachyura	Indian Pitta	2013/11/6	7	0	11.57823125	76.65370137	https://www.inaturalist.org/observations/9896940
125	Pitta brachyura	Indian Pitta	2022/4/24	4	0	11.76094531	79.76539611	https://www.inaturalist.org/observations/112829925
126	Pitta brachyura	Indian Pitta	2020/3/28	5	0	11.98427631	75.39945457	https://www.inaturalist.org/observations/44398637
127	Pitta brachyura	Indian Pitta	2020/4/21	7	0	11.98947787	75.59228295	https://www.inaturalist.org/observations/44360551
128	Pitta brachyura	Indian Pitta	2012/11/14	5	0	11.99293100	76.37999200	https://www.inaturalist.org/observations/133913228
129	Pitta brachyura	Indian Pitta	2017/3/20	9	0	12.03447000	75.19677667	https://www.inaturalist.org/observations/30664112
130	Pitta brachyura	Indian Pitta	2017/3/19	7	0	12.03455500	75.19665167	https://www.inaturalist.org/observations/30664065
131	Pitta brachyura	Indian Pitta	2022/11/8	3	0	12.14316753	75.93861513	https://www.inaturalist.org/observations/142456788
132	Pitta brachyura	Indian Pitta	2022/3/12	2	0	12.20900400	75.73540400	https://www.inaturalist.org/observations/119195680
133	Pitta brachyura	Indian Pitta	2014/10/16	5	0	12.21329760	79.05880115	https://www.inaturalist.org/observations/122916039
134	Pitta brachyura	Indian Pitta	2015/11/4	3	0	12.22272330	76.62728760	https://www.inaturalist.org/observations/142800809
135	Pitta brachyura	Indian Pitta	2016/3/3	4	0	12.22920841	79.05243123	https://www.inaturalist.org/observations/96065224
136	Pitta brachyura	Indian Pitta	2011/2/12	3	0	12.26937200	76.61197800	https://www.inaturalist.org/observations/94578865
137	Pitta brachyura	Indian Pitta	2012/1/21	3	0	12.26937200	76.61197800	https://www.inaturalist.org/observations/94717033

138	Pitta brachyura	Indian Pitta	2022/10/22	3	0	12.27525460	76.67014750	https://www.inaturalist.org/observations/139637374
139	Pitta brachyura	Indian Pitta	2022/2/8	7	0	12.27680333	76.61296667	https://www.inaturalist.org/observations/108231661
140	Pitta brachyura	Indian Pitta	2019/5/1	6	0	12.28119801	77.39116236	https://www.inaturalist.org/observations/34773761
141	Pitta brachyura	Indian Pitta	2022/2/18	4	0	12.31016778	76.63113076	https://www.inaturalist.org/observations/106960065
142	Pitta brachyura	Indian Pitta	2019/2/25	1	0	12.35651812	76.67031576	https://www.inaturalist.org/observations/70264091
143	Pitta brachyura	Indian Pitta	2015/12/12	9	0	12.56311806	79.98715639	https://www.inaturalist.org/observations/22609688
144	Pitta brachyura	Indian Pitta	2013/1/26	2	0	12.64780281	76.62294091	https://www.inaturalist.org/observations/98110933
145	Pitta brachyura	Indian Pitta	2020/4/24	2	0	12.88437970	77.46371830	https://www.inaturalist.org/observations/44092527
146	Pitta brachyura	Indian Pitta	2022/4/24	1	0	12.88507929	77.46421002	https://www.inaturalist.org/observations/113745622
147	Pitta brachyura	Indian Pitta	2017/11/12	8	0	12.92898480	80.17216130	https://www.inaturalist.org/observations/24999701
148	Pitta brachyura	Indian Pitta	2021/2/9	7	0	12.94532610	79.23287340	https://www.inaturalist.org/observations/107632857
149	Pitta brachyura	Indian Pitta	2018/11/9	6	0	12.94982970	74.85656130	https://www.inaturalist.org/observations/18301126
150	Pitta brachyura	Indian Pitta	2022/2/9	5	0	12.95626600	75.38068500	https://www.inaturalist.org/observations/106438013
151	Pitta brachyura	Indian Pitta	2013/10/13	7	0	12.95812020	80.11922810	https://www.inaturalist.org/observations/22654990
152	Pitta brachyura	Indian Pitta	2019/2/17	8	0	12.98806353	80.22971290	https://www.inaturalist.org/observations/22552799
153	Pitta brachyura	Indian Pitta	2020/1/26	3	0	12.98933770	80.23774890	https://www.inaturalist.org/observations/38030169
154	Pitta brachyura	Indian Pitta	2022/2/27	6	0	12.99077680	80.23798700	https://www.inaturalist.org/observations/107752537
155	Pitta brachyura	Indian Pitta	2016/1/17	7	0	12.99701565	80.24066722	https://www.inaturalist.org/observations/31903372
156	Pitta brachyura	Indian Pitta	2018/3/10	8	0	13.00139119	80.23092585	https://www.inaturalist.org/observations/30877943
157	Pitta brachyura	Indian Pitta	2017/4/14	9	0	13.01677125	80.23569334	https://www.inaturalist.org/observations/23290692
158	Pitta brachyura	Indian Pitta	2018/5/5	3	0	13.01883817	77.56704990	https://www.inaturalist.org/observations/106329294
159	Pitta brachyura	Indian Pitta	2019/1/6	8	0	13.02109848	77.57077351	https://www.inaturalist.org/observations/26759180
160	Pitta brachyura	Indian Pitta	2021/10/24	4	0	13.05053719	80.25141276	https://www.inaturalist.org/observations/99195010

161	Pitta brachyura	Indian Pitta	2018/10/12	9	0	13.05784757	77.63975767	https://www.inaturalist.org/observations/28922788
162	Pitta brachyura	Indian Pitta	2021/2/12	4	0	13.08007180	77.57851510	https://www.inaturalist.org/observations/117712884
163	Pitta brachyura	Indian Pitta	2019/10/28	5	0	13.12686216	80.22766717	https://www.inaturalist.org/observations/35036076
164	Pitta brachyura	Indian Pitta	2019/4/28	3	0	13.12963810	80.13099400	https://www.inaturalist.org/observations/48412629
165	Pitta brachyura	Indian Pitta	2020/10/16	6	0	13.27142810	80.26393547	https://www.inaturalist.org/observations/62683788
166	Pitta brachyura	Indian Pitta	2018/2/18	3	0	13.43512480	75.64240880	https://www.inaturalist.org/observations/84867267
167	Pitta brachyura	Indian Pitta	2022/3/27	5	0	13.65182529	79.41621780	https://www.inaturalist.org/observations/109660330
168	Pitta brachyura	Indian Pitta	2022/3/26	1	0	13.65182529	79.41626072	https://www.inaturalist.org/observations/111062639
169	Pitta brachyura	Indian Pitta	2022/3/26	1	0	13.65182529	79.41626072	https://www.inaturalist.org/observations/111062641
170	Pitta brachyura	Indian Pitta	2022/3/24	8	0	13.65182529	79.41627145	https://www.inaturalist.org/observations/109363191
171	Pitta brachyura	Indian Pitta	2020/2/6	5	0	13.65413881	79.41542789	https://www.inaturalist.org/observations/38374931
172	Pitta brachyura	Indian Pitta	2021/3/24	2	0	13.65475328	79.41864627	https://www.inaturalist.org/observations/71941016
173	Pitta brachyura	Indian Pitta	2018/4/7	6	0	13.65762120	79.48696180	https://www.inaturalist.org/observations/11014813
174	Pitta brachyura	Indian Pitta	2022/3/12	3	0	13.74988551	79.45526842	https://www.inaturalist.org/observations/108479764
175	Pitta brachyura	Indian Pitta	2016/4/26	7	0	13.85362750	75.52241470	https://www.inaturalist.org/observations/9989151
176	Pitta brachyura	Indian Pitta	2021/1/29	5	0	14.14524639	77.82287139	https://www.inaturalist.org/observations/88228992
177	Pitta brachyura	Indian Pitta	2011/12/23	2	0	14.86613746	74.43937294	https://www.inaturalist.org/observations/103681909
178	Pitta brachyura	Indian Pitta	2020/4/25	3	0	15.21376257	73.93572807	https://www.inaturalist.org/observations/43662105
179	Pitta brachyura	Indian Pitta	2018/11/7	7	0	15.22097862	74.63251946	https://www.inaturalist.org/observations/18192076
180	Pitta brachyura	Indian Pitta	2018/5/22	5	0	15.22883840	73.97418740	https://www.inaturalist.org/observations/42656784
181	Pitta brachyura	Indian Pitta	2018/6/2	5	0	15.22883840	73.97418740	https://www.inaturalist.org/observations/42656785
182	Pitta brachyura	Indian Pitta	2018/11/15	7	0	15.24301160	74.62162970	https://www.inaturalist.org/observations/18568034
183	Pitta brachyura	Indian Pitta	2013/2/16	5	0	15.29168820	74.52099299	https://www.inaturalist.org/observations/127448563

184	Pitta brachyura	Indian Pitta	2008/1/16	7	0	15.43502400	74.24530500	http://www.inaturalist.org/observations/604614
185	Pitta brachyura	Indian Pitta	2019/6/2	9	0	15.53741413	73.83271662	https://www.inaturalist.org/observations/60168977
186	Pitta brachyura	Indian Pitta	2021/4/29	5	0	15.57145500	73.86578333	https://www.inaturalist.org/observations/75650387
187	Pitta brachyura	Indian Pitta	2019/3/19	7	0	15.67160881	73.73275390	https://www.inaturalist.org/observations/21384160
188	Pitta brachyura	Indian Pitta	2022/7/17	4	0	15.73395757	73.80246799	https://www.inaturalist.org/observations/128637375
189	Pitta brachyura	Indian Pitta	2020/5/24	5	0	15.88093717	73.73258438	https://www.inaturalist.org/observations/50128910
190	Pitta brachyura	Indian Pitta	2018/5/19	2	0	16.03724810	73.57708010	https://www.inaturalist.org/observations/56207387
191	Pitta brachyura	Indian Pitta	2018/5/19	2	0	16.03724810	73.57708010	https://www.inaturalist.org/observations/56207390
192	Pitta brachyura	Indian Pitta	2022/7/21	9	0	16.07211265	73.65276326	https://www.inaturalist.org/observations/127227905
193	Pitta brachyura	Indian Pitta	2017/4/27	9	0	16.07270584	73.65325554	https://www.inaturalist.org/observations/60170987
194	Pitta brachyura	Indian Pitta	2015/4/20	5	0	16.30689104	80.51252635	https://www.inaturalist.org/observations/87981075
195	Pitta brachyura	Indian Pitta	2021/10/9	7	0	16.51985804	80.67636050	https://www.inaturalist.org/observations/97970646
196	Pitta brachyura	Indian Pitta	2016/5/21	6	0	16.52154236	73.61417089	https://www.inaturalist.org/observations/131041147
197	Pitta brachyura	Indian Pitta	2019/6/26	4	0	16.52278155	73.53153180	https://www.inaturalist.org/observations/50233498
198	Pitta brachyura	Indian Pitta	2022/2/13	4	0	16.64674448	80.81858563	https://www.inaturalist.org/observations/106796077
199	Pitta brachyura	Indian Pitta	2022/2/19	5	0	16.64950208	80.49085768	https://www.inaturalist.org/observations/108083305
200	Pitta brachyura	Indian Pitta	2021/5/6	3	0	16.71209500	73.33980000	https://www.inaturalist.org/observations/77556103
201	Pitta brachyura	Indian Pitta	2022/6/5	5	0	16.71956910	73.35530210	https://www.inaturalist.org/observations/120298098
202	Pitta brachyura	Indian Pitta	2018/8/3	8	0	16.80923057	73.32867075	https://www.inaturalist.org/observations/30376128
203	Pitta brachyura	Indian Pitta	2022/7/30	5	0	16.94205780	73.37381690	https://www.inaturalist.org/observations/128762246
204	Pitta brachyura	Indian Pitta	2018/7/29	8	0	16.96124391	73.30489207	https://www.inaturalist.org/observations/14898603
205	Pitta brachyura	Indian Pitta	2006/5/3	8	0	16.98846626	73.31452179	http://www.inaturalist.org/observations/2351
206	Pitta brachyura	Indian Pitta	2022/10/3	7	0	16.99021500	73.31202330	https://www.inaturalist.org/observations/139126087

207	Pitta brachyura	Indian Pitta	2019/5/4	3	0	16.99567353	73.30502810	https://www.inaturalist.org/observations/55800952
208	Pitta brachyura	Indian Pitta	2020/6/10	4	0	17.16992167	73.53355333	https://www.inaturalist.org/observations/49960355
209	Pitta brachyura	Indian Pitta	2019/5/23	5	0	17.31866950	78.35862240	https://www.inaturalist.org/observations/63635300
210	Pitta brachyura	Indian Pitta	2022/6/10	1	0	17.34499793	73.33720867	https://www.inaturalist.org/observations/136292154
211	Pitta brachyura	Indian Pitta	2022/4/17	4	0	17.52246770	78.33443850	https://www.inaturalist.org/observations/111761107
212	Pitta brachyura	Indian Pitta	2015/7/5	3	0	17.53190370	73.51513410	https://www.inaturalist.org/observations/122790511
213	Pitta brachyura	Indian Pitta	2022/2/9	5	0	17.65949910	78.06029979	https://www.inaturalist.org/observations/126095875
214	Pitta brachyura	Indian Pitta	2022/4/10	6	0	17.84734150	73.08790320	https://www.inaturalist.org/observations/132024123
215	Pitta brachyura	Indian Pitta	2019/6/1	4	0	17.95258300	80.00597900	https://www.inaturalist.org/observations/63635593
216	Pitta brachyura	Indian Pitta	2017/5/14	6	0	18.04319766	79.13091118	https://www.inaturalist.org/observations/65885776
217	Pitta brachyura	Indian Pitta	2016/5/28	6	0	18.11243700	79.01930000	https://www.inaturalist.org/observations/63558643
218	Pitta brachyura	Indian Pitta	2018/6/30	5	0	18.11243720	79.01929970	https://www.inaturalist.org/observations/63556658
219	Pitta brachyura	Indian Pitta	2017/5/12	6	0	18.11243720	79.01929970	https://www.inaturalist.org/observations/63558542
220	Pitta brachyura	Indian Pitta	2015/3/13	10	0	18.44690320	73.43054390	https://www.inaturalist.org/observations/19696488
221	Pitta brachyura	Indian Pitta	2022/5/25	4	0	18.50735032	73.80765438	https://www.inaturalist.org/observations/118660444
222	Pitta brachyura	Indian Pitta	2011/5/5	6	0	18.65926661	72.93159704	https://www.inaturalist.org/observations/104699437
223	Pitta brachyura	Indian Pitta	2022/6/6	4	0	18.66130924	72.93403416	https://www.inaturalist.org/observations/121698011
224	Pitta brachyura	Indian Pitta	2014/6/9	7	0	18.69493988	72.92580150	https://www.inaturalist.org/observations/53129599
225	Pitta brachyura	Indian Pitta	2014/6/9	4	0	18.69549747	72.92534850	https://www.inaturalist.org/observations/103305169
226	Pitta brachyura	Indian Pitta	2009/5/27	3	0	18.74214080	72.86302355	https://www.inaturalist.org/observations/67030285
227	Pitta brachyura	Indian Pitta	2022/4/15	4	0	18.82090440	81.36942387	https://www.inaturalist.org/observations/116231941
228	Pitta brachyura	Indian Pitta	2019/6/9	8	0	18.85344575	81.36906613	https://www.inaturalist.org/observations/27277968
229	Pitta brachyura	Indian Pitta	2017/7/4	6	0	18.89066965	82.10339494	https://www.inaturalist.org/observations/7716760

230	Pitta brachyura	Indian Pitta	2012/7/14	3	0	18.89103790	73.12191850	https://www.inaturalist.org/observations/122790512
231	Pitta brachyura	Indian Pitta	2020/10/9	5	0	19.07757163	72.84200985	https://www.inaturalist.org/observations/65000246
232	Pitta brachyura	Indian Pitta	2013/7/12	5	0	19.15868740	73.52403490	https://www.inaturalist.org/observations/91012894
233	Pitta brachyura	Indian Pitta	2020/6/1	3	0	19.17645645	72.85428619	https://www.inaturalist.org/observations/50558256
234	Pitta brachyura	Indian Pitta	2020/6/1	5	0	19.17656872	72.85423928	https://www.inaturalist.org/observations/48114145
235	Pitta brachyura	Indian Pitta	2022/6/18	5	0	19.23376500	72.94458020	https://www.inaturalist.org/observations/122432690
236	Pitta brachyura	Indian Pitta	2018/5/27	3	0	19.23825890	72.96358970	https://www.inaturalist.org/observations/43620975
237	Pitta brachyura	Indian Pitta	2021/7/26	3	0	19.31100413	84.80094217	https://www.inaturalist.org/observations/88667313
238	Pitta brachyura	Indian Pitta	2018/8/4	9	0	19.44450005	72.92818105	https://www.inaturalist.org/observations/60170470
239	Pitta brachyura	Indian Pitta	2015/6/18	7	0	20.22289874	79.36008453	https://www.inaturalist.org/observations/12189321
240	Pitta brachyura	Indian Pitta	2010/5/23	3	0	20.24838270	79.36068900	https://www.inaturalist.org/observations/55252612
241	Pitta brachyura	Indian Pitta	2016/6/5	4	0	20.24838270	79.36068900	https://www.inaturalist.org/observations/55505184
242	Pitta brachyura	Indian Pitta	2015/6/13	3	0	20.24838270	79.36068900	https://www.inaturalist.org/observations/55897773
243	Pitta brachyura	Indian Pitta	2015/6/14	3	0	20.24838270	79.36068900	https://www.inaturalist.org/observations/55897790
244	Pitta brachyura	Indian Pitta	2014/6/25	5	0	20.24838270	79.36068900	https://www.inaturalist.org/observations/55899535
245	Pitta brachyura	Indian Pitta	2016/6/10	6	0	20.24838270	79.36068900	https://www.inaturalist.org/observations/58942631
246	Pitta brachyura	Indian Pitta	2019/5/27	9	0	20.37298664	79.35119095	https://www.inaturalist.org/observations/27587147
247	Pitta brachyura	Indian Pitta	2016/8/27	6	0	20.37816200	75.09774600	http://www.inaturalist.org/observations/4157418
248	Pitta brachyura	Indian Pitta	2011/5/15	6	0	20.49522540	79.37189860	https://www.inaturalist.org/observations/69553469
249	Pitta brachyura	Indian Pitta	2022/7/29	6	0	20.50009776	81.90122976	https://www.inaturalist.org/observations/128451548
250	Pitta brachyura	Indian Pitta	2021/5/29	5	0	20.59780733	82.05195419	https://www.inaturalist.org/observations/80688887
251	Pitta brachyura	Indian Pitta	2019/5/26	5	0	20.61087143	81.55033417	https://www.inaturalist.org/observations/37777578
252	Pitta brachyura	Indian Pitta	2019/5/29	4	0	21.16549961	81.39554054	https://www.inaturalist.org/observations/51160373

253	Pitta brachyura	Indian Pitta	2019/5/26	3	0	21.17295415	81.31154481	https://www.inaturalist.org/observations/72753794
254	Pitta brachyura	Indian Pitta	2022/5/19	5	0	21.19300697	80.76201115	https://www.inaturalist.org/observations/120312410
255	Pitta brachyura	Indian Pitta	2020/6/14	3	0	21.19999874	80.75930212	https://www.inaturalist.org/observations/49577634
256	Pitta brachyura	Indian Pitta	2021/5/20	5	0	21.20153666	80.71983241	https://www.inaturalist.org/observations/79494710
257	Pitta brachyura	Indian Pitta	2021/4/20	3	0	21.20608998	81.60781559	https://www.inaturalist.org/observations/74647043
258	Pitta brachyura	Indian Pitta	2019/1/1	3	0	21.21598352	81.44047827	https://www.inaturalist.org/observations/139441012
259	Pitta brachyura	Indian Pitta	2017/5/31	4	0	21.23559330	81.45205602	https://www.inaturalist.org/observations/78506852
260	Pitta brachyura	Indian Pitta	2021/4/23	5	0	21.23990900	81.63186900	https://www.inaturalist.org/observations/74866479
261	Pitta brachyura	Indian Pitta	2022/5/6	2	0	21.27257053	79.95874286	https://www.inaturalist.org/observations/115841580
262	Pitta brachyura	Indian Pitta	2019/6/14	9	0	21.27420338	80.00715225	https://www.inaturalist.org/observations/27696434
263	Pitta brachyura	Indian Pitta	2022/5/5	6	0	21.33454181	82.29921900	https://www.inaturalist.org/observations/116819023
264	Pitta brachyura	Indian Pitta	2022/5/7	2	0	21.41124237	86.71953198	https://www.inaturalist.org/observations/116947492
265	Pitta brachyura	Indian Pitta	2019/5/26	3	0	21.41711410	86.60062490	https://www.inaturalist.org/observations/97911996
266	Pitta brachyura	Indian Pitta	2022/5/7	2	0	21.41711410	86.60062490	https://www.inaturalist.org/observations/116264844
267	Pitta brachyura	Indian Pitta	2022/5/8	3	0	21.41711410	86.60062490	https://www.inaturalist.org/observations/116264888
268	Pitta brachyura	Indian Pitta	2022/5/6	4	0	21.41712041	86.60062496	https://www.inaturalist.org/observations/117096058
269	Pitta brachyura	Indian Pitta	2022/6/4	4	0	21.47491380	79.32120580	https://www.inaturalist.org/observations/121057074
270	Pitta brachyura	Indian Pitta	2022/6/4	3	0	21.47491380	79.32120580	https://www.inaturalist.org/observations/121057082
271	Pitta brachyura	Indian Pitta	2022/6/4	4	0	21.47491380	79.32120580	https://www.inaturalist.org/observations/121057084
272	Pitta brachyura	Indian Pitta	2013/6/9	3	0	21.50127398	75.78807947	https://www.inaturalist.org/observations/85669277
273	Pitta brachyura	Indian Pitta	2016/5/27	4	0	21.56551889	71.32569083	https://www.inaturalist.org/observations/74497824
274	Pitta brachyura	Indian Pitta	2013/6/9	3	0	21.57420865	75.63148867	https://www.inaturalist.org/observations/85669294
275	Pitta brachyura	Indian Pitta	2022/6/3	4	0	21.58683800	79.28303000	https://www.inaturalist.org/observations/121056840

276	Pitta brachyura	Indian Pitta	2018/6/8	3	0	21.63591260	79.23227860	https://www.inaturalist.org/observations/56209634
277	Pitta brachyura	Indian Pitta	2018/6/10	5	0	21.63591260	79.23227860	https://www.inaturalist.org/observations/56209673
278	Pitta brachyura	Indian Pitta	2022/5/10	3	0	21.63591260	79.23227860	https://www.inaturalist.org/observations/135771401
279	Pitta brachyura	Indian Pitta	2019/6/6	4	0	21.63591661	79.23227862	https://www.inaturalist.org/observations/101797778
280	Pitta brachyura	Indian Pitta	2022/6/3	3	0	21.66861570	79.36990140	https://www.inaturalist.org/observations/121056514
281	Pitta brachyura	Indian Pitta	2022/6/3	3	0	21.66861570	79.36990140	https://www.inaturalist.org/observations/121056524
282	Pitta brachyura	Indian Pitta	2022/6/3	3	0	21.66861570	79.36990140	https://www.inaturalist.org/observations/121056525
283	Pitta brachyura	Indian Pitta	2022/6/3	3	0	21.66861570	79.36990140	https://www.inaturalist.org/observations/121056527
284	Pitta brachyura	Indian Pitta	2022/6/3	3	0	21.66861570	79.36990140	https://www.inaturalist.org/observations/121056531
285	Pitta brachyura	Indian Pitta	2022/6/3	3	0	21.66861570	79.36990140	https://www.inaturalist.org/observations/121056532
286	Pitta brachyura	Indian Pitta	2020/5/14	6	0	21.67055556	71.85583333	https://www.inaturalist.org/observations/45869327
287	Pitta brachyura	Indian Pitta	2021/7/16	4	0	21.86744240	69.92674500	https://www.inaturalist.org/observations/137604567
288	Pitta brachyura	Indian Pitta	2014/6/26	7	0	22.15310500	71.31711600	http://www.inaturalist.org/observations/1062076
289	Pitta brachyura	Indian Pitta	2017/5/20	6	0	22.24651818	80.66129498	https://www.inaturalist.org/observations/6519549
290	Pitta brachyura	Indian Pitta	2009/5/20	4	0	22.33451320	80.61151300	https://www.inaturalist.org/observations/55159112
291	Pitta brachyura	Indian Pitta	2016/5/19	4	0	22.33451320	80.61151300	https://www.inaturalist.org/observations/56311387
292	Pitta brachyura	Indian Pitta	2016/5/20	6	0	22.33451320	80.61151300	https://www.inaturalist.org/observations/56311401
293	Pitta brachyura	Indian Pitta	2017/5/17	6	0	22.33451320	80.61151300	https://www.inaturalist.org/observations/56923097
294	Pitta brachyura	Indian Pitta	2021/4/3	3	0	22.33451320	80.61151300	https://www.inaturalist.org/observations/73485856
295	Pitta brachyura	Indian Pitta	2006/5/15	6	0	22.33483346	80.60943604	https://www.inaturalist.org/observations/54715636
296	Pitta brachyura	Indian Pitta	2022/6/11	5	0	22.35682333	80.54005167	https://www.inaturalist.org/observations/121699084
297	Pitta brachyura	Indian Pitta	2022/5/29	4	0	22.35709605	73.66443515	https://www.inaturalist.org/observations/119426838
298	Pitta brachyura	Indian Pitta	2014/3/11	6	0	22.36938060	73.71410460	https://www.inaturalist.org/observations/39916032

299	Pitta brachyura	Indian Pitta	2020/6/27	3	0	22.36938060	73.71410460	https://www.inaturalist.org/observations/53850268
300	Pitta brachyura	Indian Pitta	2022/4/24	5	0	22.42260016	88.38098578	https://www.inaturalist.org/observations/112834102
301	Pitta brachyura	Indian Pitta	2022/4/24	3	0	22.42347601	88.38202983	https://www.inaturalist.org/observations/113110618
302	Pitta brachyura	Indian Pitta	2019/5/23	7	0	22.43456300	81.70303300	https://www.inaturalist.org/observations/59106556
303	Pitta brachyura	Indian Pitta	2017/5/31	7	0	22.46939200	81.78125060	https://www.inaturalist.org/observations/60976391
304	Pitta brachyura	Indian Pitta	2019/5/24	5	0	22.46939200	81.78125060	https://www.inaturalist.org/observations/62090607
305	Pitta brachyura	Indian Pitta	2016/5/1	4	0	22.51199388	88.35611007	https://www.inaturalist.org/observations/97878472
306	Pitta brachyura	Indian Pitta	2019/4/22	2	0	22.51220567	88.35816087	https://www.inaturalist.org/observations/66408402
307	Pitta brachyura	Indian Pitta	2019/5/23	7	0	22.59873571	80.52728100	https://www.inaturalist.org/observations/34454680
308	Pitta brachyura	Indian Pitta	2019/5/16	7	0	22.79002338	80.60843135	https://www.inaturalist.org/observations/34454666
309	Pitta brachyura	Indian Pitta	2015/5/6	5	0	22.79245360	88.29597950	https://www.inaturalist.org/observations/100378056
310	Pitta brachyura	Indian Pitta	2022/4/28	3	0	22.80416040	88.33392340	https://www.inaturalist.org/observations/113701622
311	Pitta brachyura	Indian Pitta	2022/6/10	4	0	22.87806000	77.51555833	https://www.inaturalist.org/observations/124769503
312	Pitta brachyura	Indian Pitta	2020/6/28	5	0	22.89659541	86.20610286	https://www.inaturalist.org/observations/51345753
313	Pitta brachyura	Indian Pitta	2022/6/29	5	0	22.91585512	82.99684245	https://www.inaturalist.org/observations/128322223
314	Pitta brachyura	Indian Pitta	2021/5/9	5	0	22.91598081	86.14719383	https://www.inaturalist.org/observations/78038534
315	Pitta brachyura	Indian Pitta	2021/7/23	6	0	22.96549705	83.02855589	https://www.inaturalist.org/observations/88260758
316	Pitta brachyura	Indian Pitta	2018/8/24	7	0	22.96903936	86.89895969	https://www.inaturalist.org/observations/17156934
317	Pitta brachyura	Indian Pitta	2019/5/31	6	0	22.97063040	87.04712687	https://www.inaturalist.org/observations/26104805
318	Pitta brachyura	Indian Pitta	2019/5/31	7	0	22.97063040	87.04712687	https://www.inaturalist.org/observations/26104807
319	Pitta brachyura	Indian Pitta	2019/5/31	8	0	22.97063040	87.04712687	https://www.inaturalist.org/observations/26104808
320	Pitta brachyura	Indian Pitta	2022/6/5	3	0	22.97105296	83.03581730	https://www.inaturalist.org/observations/122490143
321	Pitta brachyura	Indian Pitta	2019/5/30	6	0	23.01424645	87.02129561	https://www.inaturalist.org/observations/26048777

322	Pitta brachyura	Indian Pitta	2019/5/30	6	0	23.01424645	87.02129561	https://www.inaturalist.org/observations/26048778
323	Pitta brachyura	Indian Pitta	2019/5/30	6	0	23.01424645	87.02129561	https://www.inaturalist.org/observations/26048779
324	Pitta brachyura	Indian Pitta	2019/5/30	6	0	23.01424645	87.02129561	https://www.inaturalist.org/observations/26048780
325	Pitta brachyura	Indian Pitta	2019/5/30	6	0	23.01424645	87.02129561	https://www.inaturalist.org/observations/26048782
326	Pitta brachyura	Indian Pitta	2019/5/30	6	0	23.01424645	87.02129561	https://www.inaturalist.org/observations/26048783
327	Pitta brachyura	Indian Pitta	2019/5/30	7	0	23.01424645	87.02129561	https://www.inaturalist.org/observations/26048784
328	Pitta brachyura	Indian Pitta	2019/5/30	7	0	23.01424645	87.02129561	https://www.inaturalist.org/observations/26048785
329	Pitta brachyura	Indian Pitta	2019/5/30	7	0	23.01424645	87.02129561	https://www.inaturalist.org/observations/26048786
330	Pitta brachyura	Indian Pitta	2019/5/30	7	0	23.01424645	87.02129561	https://www.inaturalist.org/observations/26048787
331	Pitta brachyura	Indian Pitta	2019/5/30	7	0	23.01424645	87.02129561	https://www.inaturalist.org/observations/26048788
332	Pitta brachyura	Indian Pitta	2019/5/30	8	0	23.01424645	87.02129561	https://www.inaturalist.org/observations/26048790
333	Pitta brachyura	Indian Pitta	2019/5/30	8	0	23.01424645	87.02129561	https://www.inaturalist.org/observations/26048792
334	Pitta brachyura	Indian Pitta	2019/5/30	9	0	23.01424645	87.02129561	https://www.inaturalist.org/observations/26048795
335	Pitta brachyura	Indian Pitta	2018/5/1	2	0	23.25136166	88.59443665	https://www.inaturalist.org/observations/74484623
336	Pitta brachyura	Indian Pitta	2021/5/28	3	0	23.25993330	77.41261500	https://www.inaturalist.org/observations/88270171
337	Pitta brachyura	Indian Pitta	2022/6/8	5	0	23.29875775	77.41721369	https://www.inaturalist.org/observations/120764305
338	Pitta brachyura	Indian Pitta	2021/5/29	1	0	23.29884859	77.41662595	https://www.inaturalist.org/observations/94000655
339	Pitta brachyura	Indian Pitta	2021/5/27	4	0	23.29888277	77.41761401	https://www.inaturalist.org/observations/105942605
340	Pitta brachyura	Indian Pitta	2021/5/15	6	0	23.52044430	87.31192270	https://www.inaturalist.org/observations/79957784
341	Pitta brachyura	Indian Pitta	2017/5/12	7	0	23.59520000	87.42664500	https://www.inaturalist.org/observations/8343108
342	Pitta brachyura	Indian Pitta	2016/5/10	5	0	23.60778840	87.44863080	https://www.inaturalist.org/observations/5904484
343	Pitta brachyura	Indian Pitta	2017/4/23	6	0	23.65813800	87.28251300	https://www.inaturalist.org/observations/5941634
344	Pitta brachyura	Indian Pitta	2019/5/24	4	0	23.69944400	80.96194400	https://www.inaturalist.org/observations/56496007

345	Pitta brachyura	Indian Pitta	2009/5/25	4	0	23.72221060	81.02327100	https://www.inaturalist.org/observations/55146100
346	Pitta brachyura	Indian Pitta	2018/5/21	10	0	23.72245400	81.02421900	https://www.inaturalist.org/observations/13142147
347	Pitta brachyura	Indian Pitta	2022/5/20	5	0	23.72381403	81.02348697	https://www.inaturalist.org/observations/118791165
348	Pitta brachyura	Indian Pitta	2020/9/1	7	0	24.92312262	76.84470791	https://www.inaturalist.org/observations/58506918
349	Pitta brachyura	Indian Pitta	2016/5/31	5	0	25.21169185	75.08970650	https://www.inaturalist.org/observations/123217563
350	Pitta brachyura	Indian Pitta	2022/7/7	3	0	26.01732619	76.50257405	https://www.inaturalist.org/observations/125884147
351	Pitta brachyura	Indian Pitta	2013/6/6	4	0	26.01732740	76.50257420	https://www.inaturalist.org/observations/55272192
352	Pitta brachyura	Indian Pitta	2015/5/25	3	0	26.01732740	76.50257420	https://www.inaturalist.org/observations/55474149
353	Pitta brachyura	Indian Pitta	2015/5/25	5	0	26.01732740	76.50257420	https://www.inaturalist.org/observations/56407988
354	Pitta brachyura	Indian Pitta	2013/6/6	4	0	26.01732740	76.50257420	https://www.inaturalist.org/observations/56619450
355	Pitta brachyura	Indian Pitta	2022/6/6	5	0	26.01732740	76.50257420	https://www.inaturalist.org/observations/130132488
356	Pitta brachyura	Indian Pitta	2015/5/17	4	0	26.01732740	76.50257420	https://www.inaturalist.org/observations/142000185
357	Pitta brachyura	Indian Pitta	2016/7/5	6	0	26.01936506	76.43667016	https://www.inaturalist.org/observations/21748446
358	Pitta brachyura	Indian Pitta	2022/10/23	2	0	26.51233900	80.23290000	https://www.inaturalist.org/observations/139977791
359	Pitta brachyura	Indian Pitta	2022/7/18	5	0	26.91686047	75.86473536	https://www.inaturalist.org/observations/128743834
360	Pitta brachyura	Indian Pitta	2022/7/18	2	0	26.91686047	75.86473536	https://www.inaturalist.org/observations/128743836
361	Pitta brachyura	Indian Pitta	2022/7/18	6	0	26.91686047	75.86473536	https://www.inaturalist.org/observations/128743837
362	Pitta brachyura	Indian Pitta	2021/6/26	5	0	28.35031350	77.06277145	https://www.inaturalist.org/observations/87819487
363	Pitta brachyura	Indian Pitta	2018/7/29	8	0	28.35095520	77.06148330	https://www.inaturalist.org/observations/15008608
364	Pitta brachyura	Indian Pitta	2020/6/14	6	0	28.36300890	77.04498209	https://www.inaturalist.org/observations/49930721
365	Pitta brachyura	Indian Pitta	2022/6/30	4	0	28.36301274	77.04498176	https://www.inaturalist.org/observations/127453896
366	Pitta brachyura	Indian Pitta	2020/8/2	4	0	28.37355947	77.17085112	https://www.inaturalist.org/observations/58117873
367	Pitta brachyura	Indian Pitta	2022/7/3	2	0	28.48183520	77.26527742	https://www.inaturalist.org/observations/132374777

368	Pitta brachyura	Indian Pitta	2008/5/29	4	0	29.53001380	78.77466030	https://www.inaturalist.org/observations/55248050
369	Pitta brachyura	Indian Pitta	2013/6/21	4	0	29.59191109	79.04433193	https://www.inaturalist.org/observations/105558375
370	Pitta brachyura	Indian Pitta	2020/5/4	6	0	29.60279948	79.07377616	https://www.inaturalist.org/observations/123682418
371	Pitta brachyura	Indian Pitta	2013/5/25	3	0	29.61269140	78.81177360	https://www.inaturalist.org/observations/122790510
372	Pitta brachyura	Indian Pitta	2022/5/8	4	0	30.29655558	77.88279925	https://www.inaturalist.org/observations/116067167
373	Pitta brachyura	Indian Pitta	2017/5/21	4	0	30.75405040	76.79280734	https://www.inaturalist.org/observations/128615240

No	scientific name	common name	date	NIA	NID	latitude	longitude	url
1	Sturnus pagodarum	Brahminy Starling	2011/1/22	3	0	6.25722147	81.38384608	https://www.inaturalist.org/observations/57912726
2	Sturnus pagodarum	Brahminy Starling	2018/10/24	3	0	6.27282560	81.38657213	https://www.inaturalist.org/observations/18263778
3	Sturnus pagodarum	Brahminy Starling	2017/3/26	4	0	6.27962074	81.40191293	https://www.inaturalist.org/observations/5505474
4	Sturnus pagodarum	Brahminy Starling	2020/12/14	6	0	6.30177324	81.36644707	https://www.inaturalist.org/observations/84043608
5	Sturnus pagodarum	Brahminy Starling	2022/2/17	2	0	6.34214678	81.45768832	https://www.inaturalist.org/observations/107540947
6	Sturnus pagodarum	Brahminy Starling	2014/2/13	4	0	6.39821167	81.46314333	https://www.inaturalist.org/observations/37148128
7	Sturnus pagodarum	Brahminy Starling	2016/12/24	5	0	6.52467110	81.68007463	https://www.inaturalist.org/observations/55770126
8	Sturnus pagodarum	Brahminy Starling	2021/11/8	4	0	7.00193553	81.85070248	https://www.inaturalist.org/observations/100611598
9	Sturnus pagodarum	Brahminy Starling	2021/1/21	2	0	8.44698648	76.98138792	https://www.inaturalist.org/observations/80755597
10	Sturnus pagodarum	Brahminy Starling	2019/11/17	2	0	8.45056153	76.97057661	https://www.inaturalist.org/observations/42916691
11	Sturnus pagodarum	Brahminy Starling	2022/4/8	3	0	8.49769802	77.45777061	https://www.inaturalist.org/observations/142139547
12	Sturnus pagodarum	Brahminy Starling	2014/10/4	3	0	8.69969770	77.63132000	https://www.inaturalist.org/observations/23921342
13	Sturnus pagodarum	Brahminy Starling	2021/12/23	2	0	8.70585758	77.61514621	https://www.inaturalist.org/observations/103674846
14	Sturnus pagodarum	Brahminy Starling	2021/4/25	1	0	9.36024228	77.92068515	https://www.inaturalist.org/observations/99902798
15	Sturnus pagodarum	Brahminy Starling	2021/4/25	1	0	9.36024228	77.92068515	https://www.inaturalist.org/observations/99902804
16	Sturnus pagodarum	Brahminy Starling	2021/12/5	3	0	9.83962543	76.78352549	https://www.inaturalist.org/observations/102766867
17	Sturnus pagodarum	Brahminy Starling	2022/12/9	2	0	10.05232199	76.33944813	https://www.inaturalist.org/observations/143991345
18	Sturnus pagodarum	Brahminy Starling	2016/1/27	1	0	10.12979100	76.68711800	https://www.inaturalist.org/observations/7278974
19	Sturnus pagodarum	Brahminy Starling	2020/3/8	1	0	10.12979136	76.68711841	https://www.inaturalist.org/observations/40601026
20	Sturnus pagodarum	Brahminy Starling	2022/10/24	1	0	10.13517211	76.38848886	https://www.inaturalist.org/observations/139951111
21	Sturnus pagodarum	Brahminy Starling	2018/1/26	4	0	10.36592393	76.24842564	https://www.inaturalist.org/observations/35271982
22	Sturnus pagodarum	Brahminy Starling	2015/1/8	1	0	10.41267546	77.24186803	https://www.inaturalist.org/observations/82346413

23	Sturnus pagodarum	Brahminy Starling	2022/11/22	2	0	10.44940699	76.41066916	https://www.inaturalist.org/observations/142733454
24	Sturnus pagodarum	Brahminy Starling	2020/3/1	2	0	10.53367812	76.17583808	https://www.inaturalist.org/observations/139509797
25	Sturnus pagodarum	Brahminy Starling	2020/12/8	3	0	10.53426833	76.12865500	https://www.inaturalist.org/observations/67002088
26	Sturnus pagodarum	Brahminy Starling	2021/5/13	3	0	10.61382704	76.19477921	https://www.inaturalist.org/observations/107043483
27	Sturnus pagodarum	Brahminy Starling	2021/5/14	3	0	10.61399875	76.19414621	https://www.inaturalist.org/observations/107045216
28	Sturnus pagodarum	Brahminy Starling	2021/6/3	3	0	10.61399875	76.19414621	https://www.inaturalist.org/observations/107045265
29	Sturnus pagodarum	Brahminy Starling	2019/5/28	6	0	10.62322000	76.18419833	https://www.inaturalist.org/observations/29235195
30	Sturnus pagodarum	Brahminy Starling	2022/5/4	3	0	10.66926492	78.60887883	https://www.inaturalist.org/observations/115264736
31	Sturnus pagodarum	Brahminy Starling	2016/12/30	3	0	10.74751762	76.46718671	https://www.inaturalist.org/observations/87028922
32	Sturnus pagodarum	Brahminy Starling	2016/12/29	3	0	10.74751762	76.46718671	https://www.inaturalist.org/observations/87028923
33	Sturnus pagodarum	Brahminy Starling	2018/2/10	3	0	10.74974237	76.37860390	https://www.inaturalist.org/observations/62641696
34	Sturnus pagodarum	Brahminy Starling	2019/10/20	3	0	10.76772010	75.92590130	https://www.inaturalist.org/observations/67397051
35	Sturnus pagodarum	Brahminy Starling	2020/7/11	1	0	10.77030003	78.64099778	https://www.inaturalist.org/observations/52648091
36	Sturnus pagodarum	Brahminy Starling	2022/5/4	3	0	10.78384664	78.79653922	https://www.inaturalist.org/observations/115271105
37	Sturnus pagodarum	Brahminy Starling	2022/4/17	1	0	10.80567810	79.13908580	https://www.inaturalist.org/observations/113704594
38	Sturnus pagodarum	Brahminy Starling	2022/3/19	3	0	10.81339167	76.73613167	https://www.inaturalist.org/observations/115065051
39	Sturnus pagodarum	Brahminy Starling	2022/3/19	2	0	10.81339167	76.73613167	https://www.inaturalist.org/observations/142799202
40	Sturnus pagodarum	Brahminy Starling	2022/3/19	3	0	10.81344333	76.73592167	https://www.inaturalist.org/observations/115065067
41	Sturnus pagodarum	Brahminy Starling	2022/3/19	2	0	10.81344333	76.73592167	https://www.inaturalist.org/observations/142799200
42	Sturnus pagodarum	Brahminy Starling	2016/2/2	3	0	10.83195700	76.71375300	https://www.inaturalist.org/observations/7275794
43	Sturnus pagodarum	Brahminy Starling	2015/3/5	3	0	10.87883627	78.70316722	https://www.inaturalist.org/observations/72024037
44	Sturnus pagodarum	Brahminy Starling	2020/10/2	1	0	10.97674599	76.87984203	https://www.inaturalist.org/observations/103461712
45	Sturnus pagodarum	Brahminy Starling	2020/10/2	3	0	11.07894586	79.83002432	https://www.inaturalist.org/observations/61542993

46	Sturnus pagodarum	Brahminy Starling	2020/12/13	2	0	11.07894586	79.83002432	https://www.inaturalist.org/observations/66528739
47	Sturnus pagodarum	Brahminy Starling	2021/7/24	1	0	11.07894586	79.83002432	https://www.inaturalist.org/observations/88418437
48	Sturnus pagodarum	Brahminy Starling	2021/7/24	1	0	11.07894586	79.83002432	https://www.inaturalist.org/observations/88418775
49	Sturnus pagodarum	Brahminy Starling	2022/2/13	1	0	11.11290667	76.64201500	https://www.inaturalist.org/observations/142754292
50	Sturnus pagodarum	Brahminy Starling	2022/2/13	2	0	11.11338500	76.64268167	https://www.inaturalist.org/observations/142754284
51	Sturnus pagodarum	Brahminy Starling	2019/9/15	3	0	11.23409110	77.65862357	https://www.inaturalist.org/observations/37122681
52	Sturnus pagodarum	Brahminy Starling	2019/6/9	3	0	11.23412300	77.65862357	https://www.inaturalist.org/observations/38785582
53	Sturnus pagodarum	Brahminy Starling	2021/10/10	2	0	11.25546043	79.52609383	https://www.inaturalist.org/observations/99330895
54	Sturnus pagodarum	Brahminy Starling	2022/2/26	3	0	11.25773689	79.52334389	https://www.inaturalist.org/observations/107461150
55	Sturnus pagodarum	Brahminy Starling	2020/3/13	2	0	11.31524644	79.43747688	https://www.inaturalist.org/observations/39918723
56	Sturnus pagodarum	Brahminy Starling	2022/12/4	3	0	11.32303840	79.24335279	https://www.inaturalist.org/observations/143645436
57	Sturnus pagodarum	Brahminy Starling	2020/11/12	4	0	11.32304662	79.24335279	https://www.inaturalist.org/observations/66090597
58	Sturnus pagodarum	Brahminy Starling	2021/1/11	3	0	11.32304662	79.24335279	https://www.inaturalist.org/observations/68401618
59	Sturnus pagodarum	Brahminy Starling	2021/7/17	1	0	11.32304662	79.24335279	https://www.inaturalist.org/observations/89279121
60	Sturnus pagodarum	Brahminy Starling	2022/1/13	1	0	11.32304662	79.24335279	https://www.inaturalist.org/observations/104855938
61	Sturnus pagodarum	Brahminy Starling	2021/1/15	1	0	11.33261638	79.22819730	https://www.inaturalist.org/observations/68323323
62	Sturnus pagodarum	Brahminy Starling	2022/5/8	3	0	11.43570920	78.03808820	https://www.inaturalist.org/observations/133725802
63	Sturnus pagodarum	Brahminy Starling	2019/6/19	6	0	11.50831916	76.65684879	https://www.inaturalist.org/observations/27794805
64	Sturnus pagodarum	Brahminy Starling	2016/8/12	1	0	11.51586789	79.32688951	https://www.inaturalist.org/observations/56494521
65	Sturnus pagodarum	Brahminy Starling	2022/11/6	2	0	11.53036832	76.64691063	https://www.inaturalist.org/observations/142718578
66	Sturnus pagodarum	Brahminy Starling	2012/11/17	6	0	11.53140300	76.64780100	https://www.inaturalist.org/observations/133870451
67	Sturnus pagodarum	Brahminy Starling	2015/1/6	1	0	11.53526164	76.65010023	https://www.inaturalist.org/observations/80580015
68	Sturnus pagodarum	Brahminy Starling	2015/2/3	4	0	11.56612300	76.63833800	https://www.inaturalist.org/observations/35345251

69	Sturnus pagodarum	Brahminy Starling	2022/12/24	2	0	11.56823216	76.92649383	https://www.inaturalist.org/observations/145182303
70	Sturnus pagodarum	Brahminy Starling	2014/11/10	2	0	11.57217680	76.64271520	https://www.inaturalist.org/observations/71324501
71	Sturnus pagodarum	Brahminy Starling	2021/6/30	2	0	11.57218127	76.64271511	https://www.inaturalist.org/observations/86837266
72	Sturnus pagodarum	Brahminy Starling	2018/12/28	4	0	11.57465100	76.91790800	https://www.inaturalist.org/observations/19400828
73	Sturnus pagodarum	Brahminy Starling	2022/1/23	3	0	11.58026500	76.58731900	https://www.inaturalist.org/observations/105409225
74	Sturnus pagodarum	Brahminy Starling	2021/1/23	1	0	11.58678333	76.66998167	https://www.inaturalist.org/observations/68720997
75	Sturnus pagodarum	Brahminy Starling	2014/12/25	5	0	11.59753800	78.66810700	http://www.inaturalist.org/observations/1537049
76	Sturnus pagodarum	Brahminy Starling	2018/8/29	4	0	11.59879585	76.67267505	https://www.inaturalist.org/observations/18406379
77	Sturnus pagodarum	Brahminy Starling	2019/5/28	5	0	11.60947439	76.55265194	https://www.inaturalist.org/observations/69359398
78	Sturnus pagodarum	Brahminy Starling	2022/12/20	2	0	11.63394782	76.66670836	https://www.inaturalist.org/observations/144714851
79	Sturnus pagodarum	Brahminy Starling	2021/6/26	3	0	11.63784405	76.52570073	https://www.inaturalist.org/observations/86833909
80	Sturnus pagodarum	Brahminy Starling	2019/10/6	2	0	11.65302908	76.60661090	https://www.inaturalist.org/observations/37091102
81	Sturnus pagodarum	Brahminy Starling	2019/1/23	2	0	11.66166667	76.62722222	https://www.inaturalist.org/observations/90266441
82	Sturnus pagodarum	Brahminy Starling	2022/2/25	3	0	11.66797900	78.05295600	https://www.inaturalist.org/observations/107408175
83	Sturnus pagodarum	Brahminy Starling	2019/12/7	2	0	11.66822060	76.63035407	https://www.inaturalist.org/observations/36616583
84	Sturnus pagodarum	Brahminy Starling	2020/11/25	4	0	11.68742128	78.18066828	https://www.inaturalist.org/observations/65576593
85	Sturnus pagodarum	Brahminy Starling	2021/3/21	2	0	11.68742128	78.18066828	https://www.inaturalist.org/observations/71686364
86	Sturnus pagodarum	Brahminy Starling	2021/12/26	2	0	11.68742128	78.18066828	https://www.inaturalist.org/observations/103682016
87	Sturnus pagodarum	Brahminy Starling	2019/10/15	3	0	11.70262274	76.57144709	https://www.inaturalist.org/observations/34861905
88	Sturnus pagodarum	Brahminy Starling	2016/12/28	2	0	11.70338737	76.61443630	https://www.inaturalist.org/observations/68734594
89	Sturnus pagodarum	Brahminy Starling	2019/5/28	4	0	11.71084550	76.48650527	https://www.inaturalist.org/observations/69376933
90	Sturnus pagodarum	Brahminy Starling	2017/6/21	3	0	11.74423266	78.05355422	https://www.inaturalist.org/observations/143067264
91	Sturnus pagodarum	Brahminy Starling	2018/11/6	2	0	11.75061048	76.60074304	https://www.inaturalist.org/observations/90630920

92	Sturnus pagodarum	Brahminy Starling	2021/7/10	1	0	11.81284564	77.05690613	https://www.inaturalist.org/observations/86539223
93	Sturnus pagodarum	Brahminy Starling	2021/12/30	2	0	11.86345130	78.12294647	https://www.inaturalist.org/observations/142127340
94	Sturnus pagodarum	Brahminy Starling	2018/6/15	2	0	11.87674169	76.28920439	https://www.inaturalist.org/observations/98035849
95	Sturnus pagodarum	Brahminy Starling	2021/10/23	1	0	11.92484330	76.59993914	https://www.inaturalist.org/observations/99193798
96	Sturnus pagodarum	Brahminy Starling	2015/3/15	2	0	11.93824046	76.24342185	https://www.inaturalist.org/observations/63158050
97	Sturnus pagodarum	Brahminy Starling	2022/7/9	3	0	11.93944667	79.80482500	https://www.inaturalist.org/observations/125393548
98	Sturnus pagodarum	Brahminy Starling	2022/7/11	3	0	11.94084250	79.80466564	https://www.inaturalist.org/observations/125715393
99	Sturnus pagodarum	Brahminy Starling	2022/3/28	1	0	11.94468432	75.38493812	https://www.inaturalist.org/observations/116540427
100	Sturnus pagodarum	Brahminy Starling	2022/3/28	1	0	11.94468432	75.38493812	https://www.inaturalist.org/observations/116540655
101	Sturnus pagodarum	Brahminy Starling	2022/3/28	2	0	11.94468432	75.38493812	https://www.inaturalist.org/observations/116540856
102	Sturnus pagodarum	Brahminy Starling	2022/11/1	1	0	11.94468432	75.38493812	https://www.inaturalist.org/observations/142732182
103	Sturnus pagodarum	Brahminy Starling	2022/11/1	1	0	11.94468432	75.38493812	https://www.inaturalist.org/observations/142732301
104	Sturnus pagodarum	Brahminy Starling	2022/11/1	2	0	11.94468432	75.38493812	https://www.inaturalist.org/observations/142741183
105	Sturnus pagodarum	Brahminy Starling	2022/11/1	2	0	11.94468432	75.38493812	https://www.inaturalist.org/observations/142741197
106	Sturnus pagodarum	Brahminy Starling	2022/11/7	2	0	11.94468432	75.38493812	https://www.inaturalist.org/observations/143256959
107	Sturnus pagodarum	Brahminy Starling	2022/11/7	2	0	11.94468432	75.38493812	https://www.inaturalist.org/observations/143256969
108	Sturnus pagodarum	Brahminy Starling	2022/11/7	2	0	11.94468432	75.38493812	https://www.inaturalist.org/observations/143257054
109	Sturnus pagodarum	Brahminy Starling	2022/11/7	3	0	11.94468432	75.38493812	https://www.inaturalist.org/observations/143257068
110	Sturnus pagodarum	Brahminy Starling	2022/11/7	3	0	11.94468432	75.38493812	https://www.inaturalist.org/observations/143257080
111	Sturnus pagodarum	Brahminy Starling	2022/11/7	3	0	11.94468432	75.38493812	https://www.inaturalist.org/observations/143257086
112	Sturnus pagodarum	Brahminy Starling	2022/11/18	2	0	11.94468432	75.38493812	https://www.inaturalist.org/observations/144065829
113	Sturnus pagodarum	Brahminy Starling	2022/11/18	2	0	11.94468432	75.38493812	https://www.inaturalist.org/observations/144065841
114	Sturnus pagodarum	Brahminy Starling	2022/11/18	2	0	11.94468432	75.38493812	https://www.inaturalist.org/observations/144065888

115	Sturnus pagodarum	Brahminy Starling	2022/11/22	2	0	11.94468432	75.38493812	https://www.inaturalist.org/observations/144412939
116	Sturnus pagodarum	Brahminy Starling	2022/11/22	2	0	11.94468432	75.38493812	https://www.inaturalist.org/observations/144412947
117	Sturnus pagodarum	Brahminy Starling	2022/11/22	4	0	11.94468432	75.38493812	https://www.inaturalist.org/observations/144414228
118	Sturnus pagodarum	Brahminy Starling	2022/11/22	4	0	11.94468432	75.38493812	https://www.inaturalist.org/observations/144414236
119	Sturnus pagodarum	Brahminy Starling	2019/12/29	6	0	11.94468464	75.38493812	https://www.inaturalist.org/observations/83242087
120	Sturnus pagodarum	Brahminy Starling	2018/3/27	7	0	11.97799167	76.17021667	https://www.inaturalist.org/observations/66385865
121	Sturnus pagodarum	Brahminy Starling	2020/12/13	3	0	11.98977370	76.37704070	https://www.inaturalist.org/observations/66723982
122	Sturnus pagodarum	Brahminy Starling	2022/10/23	2	0	12.00206860	76.28412804	https://www.inaturalist.org/observations/139943138
123	Sturnus pagodarum	Brahminy Starling	2018/2/3	3	0	12.00528210	79.81286490	https://www.inaturalist.org/observations/9715675
124	Sturnus pagodarum	Brahminy Starling	2018/2/3	3	0	12.00528210	79.81286490	https://www.inaturalist.org/observations/9715676
125	Sturnus pagodarum	Brahminy Starling	2018/2/3	4	0	12.00528210	79.81286490	https://www.inaturalist.org/observations/9715677
126	Sturnus pagodarum	Brahminy Starling	2018/2/3	3	0	12.00528210	79.81286490	https://www.inaturalist.org/observations/9715678
127	Sturnus pagodarum	Brahminy Starling	2017/2/27	2	0	12.01016190	76.25503900	https://www.inaturalist.org/observations/89289549
128	Sturnus pagodarum	Brahminy Starling	2009/6/6	2	0	12.03152720	77.59202950	https://www.inaturalist.org/observations/98179559
129	Sturnus pagodarum	Brahminy Starling	2019/10/15	4	0	12.03234670	75.25665640	https://www.inaturalist.org/observations/34574175
130	Sturnus pagodarum	Brahminy Starling	2021/8/31	3	0	12.03237190	75.25665645	https://www.inaturalist.org/observations/93471264
131	Sturnus pagodarum	Brahminy Starling	2022/10/25	2	0	12.04009244	75.29748503	https://www.inaturalist.org/observations/142200392
132	Sturnus pagodarum	Brahminy Starling	2016/1/3	1	0	12.21394126	79.02857030	https://www.inaturalist.org/observations/96052784
133	Sturnus pagodarum	Brahminy Starling	2021/10/28	2	0	12.24090600	76.59794450	https://www.inaturalist.org/observations/99606920
134	Sturnus pagodarum	Brahminy Starling	2022/11/23	2	0	12.24090600	76.59794450	https://www.inaturalist.org/observations/142665903
135	Sturnus pagodarum	Brahminy Starling	2020/10/4	1	0	12.26269942	76.56762058	https://www.inaturalist.org/observations/95042439
136	Sturnus pagodarum	Brahminy Starling	2020/10/4	1	0	12.26269942	76.56762058	https://www.inaturalist.org/observations/95042442
137	Sturnus pagodarum	Brahminy Starling	2020/11/14	1	0	12.26269942	76.56762058	https://www.inaturalist.org/observations/95042460

138	Sturnus pagodarum	Brahminy Starling	2020/10/29	1	0	12.26269942	76.56762058	https://www.inaturalist.org/observations/95042487
139	Sturnus pagodarum	Brahminy Starling	2021/12/14	2	0	12.26269942	76.56762058	https://www.inaturalist.org/observations/103041644
140	Sturnus pagodarum	Brahminy Starling	2022/1/23	1	0	12.26269942	76.56762058	https://www.inaturalist.org/observations/105417188
141	Sturnus pagodarum	Brahminy Starling	2008/12/30	2	0	12.26937200	76.61197800	https://www.inaturalist.org/observations/94574856
142	Sturnus pagodarum	Brahminy Starling	2014/10/30	1	0	12.26937200	76.61197800	https://www.inaturalist.org/observations/94791356
143	Sturnus pagodarum	Brahminy Starling	2014/6/8	2	0	12.26937200	76.61197800	https://www.inaturalist.org/observations/94817198
144	Sturnus pagodarum	Brahminy Starling	2018/11/23	4	0	12.27163500	76.61520333	https://www.inaturalist.org/observations/18774437
145	Sturnus pagodarum	Brahminy Starling	2018/12/30	4	0	12.27238167	76.61418333	https://www.inaturalist.org/observations/19331414
146	Sturnus pagodarum	Brahminy Starling	2020/4/7	2	0	12.27624403	76.61636609	https://www.inaturalist.org/observations/92211685
147	Sturnus pagodarum	Brahminy Starling	2020/10/10	2	0	12.27936203	76.58896975	https://www.inaturalist.org/observations/92779676
148	Sturnus pagodarum	Brahminy Starling	2018/2/19	3	0	12.28326434	76.61517620	https://www.inaturalist.org/observations/9931350
149	Sturnus pagodarum	Brahminy Starling	2021/11/14	2	0	12.28558200	76.56760710	https://www.inaturalist.org/observations/101074492
150	Sturnus pagodarum	Brahminy Starling	2021/12/15	2	0	12.29424209	76.56539693	https://www.inaturalist.org/observations/103086012
151	Sturnus pagodarum	Brahminy Starling	2022/8/16	5	0	12.29833014	76.58334777	https://www.inaturalist.org/observations/131048455
152	Sturnus pagodarum	Brahminy Starling	2020/10/4	2	0	12.29927196	76.69088789	https://www.inaturalist.org/observations/93859054
153	Sturnus pagodarum	Brahminy Starling	2021/5/23	2	0	12.32869760	76.55288440	https://www.inaturalist.org/observations/79924525
154	Sturnus pagodarum	Brahminy Starling	2022/11/27	2	0	12.33606885	77.81784051	https://www.inaturalist.org/observations/143637965
155	Sturnus pagodarum	Brahminy Starling	2022/11/27	2	0	12.33606885	77.81784051	https://www.inaturalist.org/observations/143637968
156	Sturnus pagodarum	Brahminy Starling	2018/11/24	6	0	12.33672760	80.01409243	https://www.inaturalist.org/observations/22611643
157	Sturnus pagodarum	Brahminy Starling	2022/11/27	2	0	12.36792833	77.86630667	https://www.inaturalist.org/observations/143014498
158	Sturnus pagodarum	Brahminy Starling	2022/11/27	2	0	12.39202167	77.86531667	https://www.inaturalist.org/observations/143014694
159	Sturnus pagodarum	Brahminy Starling	2022/10/23	2	0	12.40228230	77.88786348	https://www.inaturalist.org/observations/139961199
160	Sturnus pagodarum	Brahminy Starling	2022/10/23	3	0	12.40269591	77.84884959	https://www.inaturalist.org/observations/139739099

161	Sturnus pagodarum	Brahminy Starling	2020/4/19	2	0	12.42373333	77.66342500	https://www.inaturalist.org/observations/65137422
162	Sturnus pagodarum	Brahminy Starling	2017/9/22	3	0	12.45551770	75.95702340	https://www.inaturalist.org/observations/109829865
163	Sturnus pagodarum	Brahminy Starling	2021/12/5	2	0	12.48112667	77.61970167	https://www.inaturalist.org/observations/102524275
164	Sturnus pagodarum	Brahminy Starling	2022/8/5	3	0	12.51577333	77.63620167	https://www.inaturalist.org/observations/129513993
165	Sturnus pagodarum	Brahminy Starling	2018/7/8	1	0	12.51906682	77.68443134	https://www.inaturalist.org/observations/82426966
166	Sturnus pagodarum	Brahminy Starling	2021/4/18	2	0	12.55320736	77.61468228	https://www.inaturalist.org/observations/82860395
167	Sturnus pagodarum	Brahminy Starling	2020/3/17	2	0	12.55692070	78.24457764	https://www.inaturalist.org/observations/40126519
168	Sturnus pagodarum	Brahminy Starling	2018/7/26	5	0	12.57112306	79.84622639	https://www.inaturalist.org/observations/22501980
169	Sturnus pagodarum	Brahminy Starling	2017/5/28	2	0	12.67862582	77.47086824	https://www.inaturalist.org/observations/7262454
170	Sturnus pagodarum	Brahminy Starling	2021/4/10	1	0	12.68126667	76.65528589	https://www.inaturalist.org/observations/80697311
171	Sturnus pagodarum	Brahminy Starling	2013/6/29	4	0	12.68193720	79.98884130	https://www.inaturalist.org/observations/22556462
172	Sturnus pagodarum	Brahminy Starling	2020/1/12	4	0	12.68535257	80.15015150	https://www.inaturalist.org/observations/37521259
173	Sturnus pagodarum	Brahminy Starling	2022/2/19	2	0	12.68573455	80.12562154	https://www.inaturalist.org/observations/107244330
174	Sturnus pagodarum	Brahminy Starling	2021/3/6	5	0	12.71317244	77.68367574	https://www.inaturalist.org/observations/70670424
175	Sturnus pagodarum	Brahminy Starling	2015/10/14	2	0	12.73534289	77.79045663	https://www.inaturalist.org/observations/141769123
176	Sturnus pagodarum	Brahminy Starling	2022/2/27	4	0	12.77631000	77.86090833	https://www.inaturalist.org/observations/107552803
177	Sturnus pagodarum	Brahminy Starling	2020/11/21	2	0	12.81385331	76.94609644	https://www.inaturalist.org/observations/96775284
178	Sturnus pagodarum	Brahminy Starling	2020/11/2	1	0	12.81385331	76.94609644	https://www.inaturalist.org/observations/97213925
179	Sturnus pagodarum	Brahminy Starling	2020/8/29	2	0	12.81385331	76.94609644	https://www.inaturalist.org/observations/97434945
180	Sturnus pagodarum	Brahminy Starling	2020/12/29	3	0	12.81723531	79.15190237	https://www.inaturalist.org/observations/73007445
181	Sturnus pagodarum	Brahminy Starling	2020/1/16	2	0	12.81845600	79.69465860	https://www.inaturalist.org/observations/89745751
182	Sturnus pagodarum	Brahminy Starling	2020/11/7	1	0	12.84849973	76.80860198	https://www.inaturalist.org/observations/97213456
183	Sturnus pagodarum	Brahminy Starling	2020/12/31	2	0	12.85003961	79.29611735	https://www.inaturalist.org/observations/71958984

184	Sturnus pagodarum	Brahminy Starling	2021/2/7	5	0	12.85329009	79.13682047	https://www.inaturalist.org/observations/107961068
185	Sturnus pagodarum	Brahminy Starling	2020/12/29	2	0	12.85818595	79.08881312	https://www.inaturalist.org/observations/73007446
186	Sturnus pagodarum	Brahminy Starling	2022/1/17	5	0	12.86587830	79.37550440	https://www.inaturalist.org/observations/105607538
187	Sturnus pagodarum	Brahminy Starling	2021/11/14	4	0	12.87177648	77.52343252	https://www.inaturalist.org/observations/101102289
188	Sturnus pagodarum	Brahminy Starling	2021/3/28	2	0	12.87595329	77.49380479	https://www.inaturalist.org/observations/72247408
189	Sturnus pagodarum	Brahminy Starling	2021/7/31	4	0	12.87950540	79.40612680	https://www.inaturalist.org/observations/89471538
190	Sturnus pagodarum	Brahminy Starling	2021/8/15	4	0	12.87950540	79.40612680	https://www.inaturalist.org/observations/91937476
191	Sturnus pagodarum	Brahminy Starling	2021/9/29	3	0	12.87950540	79.40612680	https://www.inaturalist.org/observations/96660318
192	Sturnus pagodarum	Brahminy Starling	2020/11/24	2	0	12.89164450	79.01576637	https://www.inaturalist.org/observations/73001485
193	Sturnus pagodarum	Brahminy Starling	2020/12/29	3	0	12.90345288	79.13761659	https://www.inaturalist.org/observations/73007449
194	Sturnus pagodarum	Brahminy Starling	2020/7/12	2	0	12.90364742	79.11386108	https://www.inaturalist.org/observations/52789690
195	Sturnus pagodarum	Brahminy Starling	2021/1/16	3	0	12.91083150	79.40114880	https://www.inaturalist.org/observations/82160898
196	Sturnus pagodarum	Brahminy Starling	2022/3/18	3	0	12.91910172	77.67842102	https://www.inaturalist.org/observations/112367680
197	Sturnus pagodarum	Brahminy Starling	2020/10/8	3	0	12.94415991	79.18025047	https://www.inaturalist.org/observations/62006243
198	Sturnus pagodarum	Brahminy Starling	2018/12/27	4	0	12.94982970	74.85656130	https://www.inaturalist.org/observations/19252433
199	Sturnus pagodarum	Brahminy Starling	2022/12/25	1	0	12.95307150	79.13100300	https://www.inaturalist.org/observations/145026513
200	Sturnus pagodarum	Brahminy Starling	2013/8/14	3	0	12.96376290	80.11462210	https://www.inaturalist.org/observations/63089786
201	Sturnus pagodarum	Brahminy Starling	2019/8/4	3	0	12.97103486	79.23419138	https://www.inaturalist.org/observations/69613675
202	Sturnus pagodarum	Brahminy Starling	2021/9/21	4	0	12.97159870	77.59456270	https://www.inaturalist.org/observations/121979312
203	Sturnus pagodarum	Brahminy Starling	2021/8/17	2	0	12.98087120	77.62934113	https://www.inaturalist.org/observations/91619341
204	Sturnus pagodarum	Brahminy Starling	2020/10/17	2	0	12.99132563	80.23602959	https://www.inaturalist.org/observations/63144706
205	Sturnus pagodarum	Brahminy Starling	2020/10/17	4	0	12.99174909	80.23688083	https://www.inaturalist.org/observations/62792221
206	Sturnus pagodarum	Brahminy Starling	2022/6/9	2	0	12.99572489	79.03635450	https://www.inaturalist.org/observations/121379621

207	Sturnus pagodarum	Brahminy Starling	2019/11/1	1	0	12.99905550	79.30259573	https://www.inaturalist.org/observations/69760664
208	Sturnus pagodarum	Brahminy Starling	2020/8/13	1	0	13.06275397	77.74924837	https://www.inaturalist.org/observations/97153508
209	Sturnus pagodarum	Brahminy Starling	2016/11/21	2	0	13.08268020	80.27071840	https://www.inaturalist.org/observations/92325079
210	Sturnus pagodarum	Brahminy Starling	2021/7/31	1	0	13.08308237	80.27071830	https://www.inaturalist.org/observations/89999150
211	Sturnus pagodarum	Brahminy Starling	2021/9/23	3	0	13.14255810	77.58937073	https://www.inaturalist.org/observations/120216226
212	Sturnus pagodarum	Brahminy Starling	2017/11/12	1	0	13.14893047	80.05743265	https://www.inaturalist.org/observations/45230970
213	Sturnus pagodarum	Brahminy Starling	2017/2/19	1	0	13.14906308	80.05751006	https://www.inaturalist.org/observations/45849285
214	Sturnus pagodarum	Brahminy Starling	2009/6/29	1	0	13.25014327	76.33875201	https://www.inaturalist.org/observations/93147462
215	Sturnus pagodarum	Brahminy Starling	2022/3/27	3	0	13.59693430	79.36818328	https://www.inaturalist.org/observations/109660288
216	Sturnus pagodarum	Brahminy Starling	2019/1/25	2	0	13.65294753	76.06936908	https://www.inaturalist.org/observations/82860134
217	Sturnus pagodarum	Brahminy Starling	2021/9/15	1	0	13.66457030	77.46320460	https://www.inaturalist.org/observations/95110403
218	Sturnus pagodarum	Brahminy Starling	2021/4/22	3	0	13.73918015	77.32319358	https://www.inaturalist.org/observations/74793750
219	Sturnus pagodarum	Brahminy Starling	2022/5/15	3	0	13.77924891	79.17824094	https://www.inaturalist.org/observations/119023826
220	Sturnus pagodarum	Brahminy Starling	2022/6/30	6	0	13.98471500	78.91686500	https://www.inaturalist.org/observations/124065814
221	Sturnus pagodarum	Brahminy Starling	2022/2/17	2	0	14.23424946	75.64699329	https://www.inaturalist.org/observations/106894232
222	Sturnus pagodarum	Brahminy Starling	2022/1/25	3	0	14.24961331	76.96947397	https://www.inaturalist.org/observations/105550701
223	Sturnus pagodarum	Brahminy Starling	2020/12/5	2	0	14.70947300	75.64227400	https://www.inaturalist.org/observations/90125210
224	Sturnus pagodarum	Brahminy Starling	2022/10/31	3	0	14.81259020	76.80688695	https://www.inaturalist.org/observations/141458538
225	Sturnus pagodarum	Brahminy Starling	2021/1/30	1	0	15.18206000	75.54994000	https://www.inaturalist.org/observations/104333271
226	Sturnus pagodarum	Brahminy Starling	2017/12/24	2	0	15.25298045	76.36168586	https://www.inaturalist.org/observations/65646679
227	Sturnus pagodarum	Brahminy Starling	2022/9/27	1	0	15.26386164	76.48594664	https://www.inaturalist.org/observations/136898487
228	Sturnus pagodarum	Brahminy Starling	2019/7/4	4	0	15.32111900	76.47273680	https://www.inaturalist.org/observations/29233132
229	Sturnus pagodarum	Brahminy Starling	2017/8/19	1	0	15.32799100	76.47237800	https://www.inaturalist.org/observations/92918960

230	Sturnus pagodarum	Brahminy Starling	2012/10/8	2	0	15.33501320	76.46002400	https://www.inaturalist.org/observations/40577477
231	Sturnus pagodarum	Brahminy Starling	2019/1/30	1	0	15.33501320	76.46002400	https://www.inaturalist.org/observations/84849955
232	Sturnus pagodarum	Brahminy Starling	2020/1/24	3	0	15.33501320	76.46002400	https://www.inaturalist.org/observations/102094748
233	Sturnus pagodarum	Brahminy Starling	2019/3/19	4	0	15.33501320	76.46002400	https://www.inaturalist.org/observations/116086414
234	Sturnus pagodarum	Brahminy Starling	2022/7/31	3	0	15.33501320	76.46002400	https://www.inaturalist.org/observations/129444587
235	Sturnus pagodarum	Brahminy Starling	2022/10/28	2	0	15.33501320	76.46002400	https://www.inaturalist.org/observations/141982366
236	Sturnus pagodarum	Brahminy Starling	2022/10/28	2	0	15.33501320	76.46002400	https://www.inaturalist.org/observations/142220783
237	Sturnus pagodarum	Brahminy Starling	2022/10/28	2	0	15.33501320	76.46002400	https://www.inaturalist.org/observations/142220798
238	Sturnus pagodarum	Brahminy Starling	2022/7/22	3	0	15.33503666	76.46002382	https://www.inaturalist.org/observations/127468265
239	Sturnus pagodarum	Brahminy Starling	2020/12/30	1	0	15.33591306	76.46059306	https://www.inaturalist.org/observations/88432294
240	Sturnus pagodarum	Brahminy Starling	2020/2/5	2	0	15.34151198	76.47804254	https://www.inaturalist.org/observations/67404825
241	Sturnus pagodarum	Brahminy Starling	2016/4/30	4	0	15.38457047	75.10404198	https://www.inaturalist.org/observations/57623029
242	Sturnus pagodarum	Brahminy Starling	2015/9/10	4	0	15.39131900	76.47650444	https://www.inaturalist.org/observations/16126722
243	Sturnus pagodarum	Brahminy Starling	2020/12/6	1	0	15.42218000	75.01581500	https://www.inaturalist.org/observations/90238858
244	Sturnus pagodarum	Brahminy Starling	2015/5/6	5	0	15.45892400	75.00780800	http://www.inaturalist.org/observations/1463509
245	Sturnus pagodarum	Brahminy Starling	2021/5/12	4	0	15.45904614	75.00780813	https://www.inaturalist.org/observations/128266233
246	Sturnus pagodarum	Brahminy Starling	1999/11/29	1	0	15.56408571	73.75518348	https://www.inaturalist.org/observations/42317827
247	Sturnus pagodarum	Brahminy Starling	2022/10/22	2	0	15.82320186	74.48357809	https://www.inaturalist.org/observations/139592627
248	Sturnus pagodarum	Brahminy Starling	2016/12/12	1	0	15.91665060	75.69100430	https://www.inaturalist.org/observations/82685116
249	Sturnus pagodarum	Brahminy Starling	2015/12/31	1	0	15.94881800	75.81635200	https://www.inaturalist.org/observations/46196291
250	Sturnus pagodarum	Brahminy Starling	2020/3/8	1	0	16.14903760	77.35073486	https://www.inaturalist.org/observations/95628193
251	Sturnus pagodarum	Brahminy Starling	2020/10/23	3	0	16.16910489	75.66150270	https://www.inaturalist.org/observations/64435247
252	Sturnus pagodarum	Brahminy Starling	2020/4/6	2	0	16.20270966	77.36287147	https://www.inaturalist.org/observations/81855122

253	Sturnus pagodarum	Brahminy Starling	2018/12/16	3	0	16.22091644	74.74896446	https://www.inaturalist.org/observations/90276726
254	Sturnus pagodarum	Brahminy Starling	2019/2/17	2	0	16.35570560	78.83346130	https://www.inaturalist.org/observations/63621562
255	Sturnus pagodarum	Brahminy Starling	2022/10/27	2	0	16.35579612	74.29781271	https://www.inaturalist.org/observations/140219363
256	Sturnus pagodarum	Brahminy Starling	2021/12/26	2	0	16.38153544	81.58766080	https://www.inaturalist.org/observations/104907642
257	Sturnus pagodarum	Brahminy Starling	2022/11/27	2	0	16.38635333	81.58981667	https://www.inaturalist.org/observations/143026573
258	Sturnus pagodarum	Brahminy Starling	2013/12/1	3	0	16.40966969	80.70889656	https://www.inaturalist.org/observations/88106306
259	Sturnus pagodarum	Brahminy Starling	2020/12/20	2	0	16.55772920	80.72698540	https://www.inaturalist.org/observations/92475023
260	Sturnus pagodarum	Brahminy Starling	2020/12/6	3	0	16.55921794	80.76100097	https://www.inaturalist.org/observations/92452640
261	Sturnus pagodarum	Brahminy Starling	2020/12/1	2	0	16.55956663	80.76008636	https://www.inaturalist.org/observations/92473426
262	Sturnus pagodarum	Brahminy Starling	2021/12/5	3	0	16.57559146	80.68230191	https://www.inaturalist.org/observations/104498334
263	Sturnus pagodarum	Brahminy Starling	2020/11/29	2	0	16.57589266	80.68862217	https://www.inaturalist.org/observations/92670069
264	Sturnus pagodarum	Brahminy Starling	2020/8/31	2	0	16.57911333	80.70136500	https://www.inaturalist.org/observations/58122838
265	Sturnus pagodarum	Brahminy Starling	2021/12/29	2	0	16.59055924	80.82735576	https://www.inaturalist.org/observations/103879227
266	Sturnus pagodarum	Brahminy Starling	2020/12/27	2	0	16.59577924	80.61818580	https://www.inaturalist.org/observations/90049768
267	Sturnus pagodarum	Brahminy Starling	2022/1/9	3	0	16.59776527	80.61998585	https://www.inaturalist.org/observations/104863272
268	Sturnus pagodarum	Brahminy Starling	2014/3/2	3	0	16.63942370	80.79750281	https://www.inaturalist.org/observations/89171354
269	Sturnus pagodarum	Brahminy Starling	2022/3/13	4	0	16.66112798	80.47830352	https://www.inaturalist.org/observations/108944373
270	Sturnus pagodarum	Brahminy Starling	2022/1/4	1	0	16.68999444	80.77381944	https://www.inaturalist.org/observations/104401543
271	Sturnus pagodarum	Brahminy Starling	2022/2/8	2	0	16.69006111	80.77379167	https://www.inaturalist.org/observations/106383271
272	Sturnus pagodarum	Brahminy Starling	2021/12/27	1	0	16.69378611	80.77235833	https://www.inaturalist.org/observations/103754012
273	Sturnus pagodarum	Brahminy Starling	2022/1/5	3	0	16.69654722	80.76982778	https://www.inaturalist.org/observations/104401816
274	Sturnus pagodarum	Brahminy Starling	2021/12/22	3	0	16.69786043	82.12057114	https://www.inaturalist.org/observations/103876500
275	Sturnus pagodarum	Brahminy Starling	2021/10/24	3	0	16.73503224	80.65353531	https://www.inaturalist.org/observations/99225832

276	Sturnus pagodarum	Brahminy Starling	2016/11/12	4	0	16.79660260	74.29103570	https://www.inaturalist.org/observations/30795835
277	Sturnus pagodarum	Brahminy Starling	2016/11/14	4	0	16.79660260	74.29103570	https://www.inaturalist.org/observations/30796604
278	Sturnus pagodarum	Brahminy Starling	2020/4/4	1	0	17.02988897	74.61172645	https://www.inaturalist.org/observations/41347814
279	Sturnus pagodarum	Brahminy Starling	2010/11/6	1	0	17.27769300	74.18435350	https://www.inaturalist.org/observations/42480929
280	Sturnus pagodarum	Brahminy Starling	2022/5/22	2	0	17.31234335	77.84826880	https://www.inaturalist.org/observations/118330932
281	Sturnus pagodarum	Brahminy Starling	2022/5/22	2	0	17.31234335	77.84826880	https://www.inaturalist.org/observations/118330936
282	Sturnus pagodarum	Brahminy Starling	2022/5/22	2	0	17.31234335	77.84826880	https://www.inaturalist.org/observations/118330941
283	Sturnus pagodarum	Brahminy Starling	2022/1/2	1	0	17.31866950	78.35862240	https://www.inaturalist.org/observations/104171395
284	Sturnus pagodarum	Brahminy Starling	2022/1/2	2	0	17.31866950	78.35862240	https://www.inaturalist.org/observations/104905973
285	Sturnus pagodarum	Brahminy Starling	2022/1/2	4	0	17.31866950	78.35862240	https://www.inaturalist.org/observations/104905974
286	Sturnus pagodarum	Brahminy Starling	2022/1/2	3	0	17.31866950	78.35862240	https://www.inaturalist.org/observations/104905978
287	Sturnus pagodarum	Brahminy Starling	2021/12/26	3	0	17.37626560	78.29886370	https://www.inaturalist.org/observations/104752861
288	Sturnus pagodarum	Brahminy Starling	2021/12/26	2	0	17.37626560	78.29886370	https://www.inaturalist.org/observations/104752876
289	Sturnus pagodarum	Brahminy Starling	2021/12/26	2	0	17.37626560	78.29886370	https://www.inaturalist.org/observations/104753080
290	Sturnus pagodarum	Brahminy Starling	2021/12/26	4	0	17.37626560	78.29886370	https://www.inaturalist.org/observations/104753341
291	Sturnus pagodarum	Brahminy Starling	2021/12/26	2	0	17.38066800	78.31718980	https://www.inaturalist.org/observations/104691697
292	Sturnus pagodarum	Brahminy Starling	2021/12/26	3	0	17.38066800	78.31718980	https://www.inaturalist.org/observations/104692618
293	Sturnus pagodarum	Brahminy Starling	2022/7/31	4	0	17.43207510	78.47538770	https://www.inaturalist.org/observations/130092332
294	Sturnus pagodarum	Brahminy Starling	2022/10/12	2	0	17.45677890	78.23337430	https://www.inaturalist.org/observations/138803140
295	Sturnus pagodarum	Brahminy Starling	2022/10/12	1	0	17.45677890	78.23337430	https://www.inaturalist.org/observations/138803160
296	Sturnus pagodarum	Brahminy Starling	2020/12/3	3	0	17.47234320	78.79742500	https://www.inaturalist.org/observations/67861302
297	Sturnus pagodarum	Brahminy Starling	2022/6/25	1	0	17.49513825	78.40061143	https://www.inaturalist.org/observations/123361347
298	Sturnus pagodarum	Brahminy Starling	2020/6/24	1	0	17.54839900	78.46781740	https://www.inaturalist.org/observations/50747425

299	Sturnus pagodarum	Brahminy Starling	2020/3/10	1	0	17.57122191	76.90757765	https://www.inaturalist.org/observations/95631483
300	Sturnus pagodarum	Brahminy Starling	2021/12/19	4	0	17.64045667	78.06637500	https://www.inaturalist.org/observations/121718387
301	Sturnus pagodarum	Brahminy Starling	2021/12/19	4	0	17.64084167	78.06885500	https://www.inaturalist.org/observations/121718361
302	Sturnus pagodarum	Brahminy Starling	2022/1/21	2	0	17.65524480	78.07606880	https://www.inaturalist.org/observations/105428153
303	Sturnus pagodarum	Brahminy Starling	2020/4/3	6	0	17.68045874	74.01826110	https://www.inaturalist.org/observations/66384200
304	Sturnus pagodarum	Brahminy Starling	2020/7/8	4	0	17.68046390	74.01826100	https://www.inaturalist.org/observations/52527770
305	Sturnus pagodarum	Brahminy Starling	2020/7/8	2	0	17.68046390	74.01826100	https://www.inaturalist.org/observations/52529145
306	Sturnus pagodarum	Brahminy Starling	2020/11/13	2	0	17.68046390	74.01826100	https://www.inaturalist.org/observations/71119195
307	Sturnus pagodarum	Brahminy Starling	2020/11/13	1	0	17.68046390	74.01826100	https://www.inaturalist.org/observations/105498330
308	Sturnus pagodarum	Brahminy Starling	2013/2/22	4	0	17.68058160	75.31550350	https://www.inaturalist.org/observations/66917702
309	Sturnus pagodarum	Brahminy Starling	2022/6/26	3	0	17.68662580	78.32732450	https://www.inaturalist.org/observations/123486402
310	Sturnus pagodarum	Brahminy Starling	2022/6/26	3	0	17.68662580	78.32732450	https://www.inaturalist.org/observations/123486410
311	Sturnus pagodarum	Brahminy Starling	2022/6/26	2	0	17.68662580	78.32732450	https://www.inaturalist.org/observations/123486413
312	Sturnus pagodarum	Brahminy Starling	2022/6/26	4	0	17.68662580	78.32732450	https://www.inaturalist.org/observations/123486414
313	Sturnus pagodarum	Brahminy Starling	2015/3/15	4	0	17.82522113	75.86484911	https://www.inaturalist.org/observations/66421016
314	Sturnus pagodarum	Brahminy Starling	2017/4/27	2	0	17.82818757	75.86145125	https://www.inaturalist.org/observations/65517339
315	Sturnus pagodarum	Brahminy Starling	2021/8/22	1	0	17.93341333	83.37913833	https://www.inaturalist.org/observations/92532078
316	Sturnus pagodarum	Brahminy Starling	2021/8/29	2	0	18.05051356	83.43898918	https://www.inaturalist.org/observations/92895828
317	Sturnus pagodarum	Brahminy Starling	2021/10/16	2	0	18.05293432	78.26185305	https://www.inaturalist.org/observations/100763125
318	Sturnus pagodarum	Brahminy Starling	2021/10/16	2	0	18.05293432	78.26185305	https://www.inaturalist.org/observations/100764535
319	Sturnus pagodarum	Brahminy Starling	2021/8/29	1	0	18.06337120	83.41365304	https://www.inaturalist.org/observations/92904388
320	Sturnus pagodarum	Brahminy Starling	2018/7/19	5	0	18.11243720	79.01929970	https://www.inaturalist.org/observations/63558554
321	Sturnus pagodarum	Brahminy Starling	2021/6/11	2	0	18.14612333	73.84378833	https://www.inaturalist.org/observations/82637692

322	Sturnus pagodarum	Brahminy Starling	2021/6/16	2	0	18.15077440	74.08728920	https://www.inaturalist.org/observations/83913922
323	Sturnus pagodarum	Brahminy Starling	2020/11/15	3	0	18.15437575	74.59256161	https://www.inaturalist.org/observations/67232172
324	Sturnus pagodarum	Brahminy Starling	2018/7/22	2	0	18.17728139	74.95116306	https://www.inaturalist.org/observations/54378737
325	Sturnus pagodarum	Brahminy Starling	2018/7/3	2	0	18.17766500	74.95500306	https://www.inaturalist.org/observations/54374220
326	Sturnus pagodarum	Brahminy Starling	2018/9/1	1	0	18.17806306	74.96137639	https://www.inaturalist.org/observations/54154824
327	Sturnus pagodarum	Brahminy Starling	2018/11/20	1	0	18.17809000	74.96276167	https://www.inaturalist.org/observations/53711189
328	Sturnus pagodarum	Brahminy Starling	2020/10/2	6	0	18.17917625	74.60779689	https://www.inaturalist.org/observations/61546345
329	Sturnus pagodarum	Brahminy Starling	2020/7/11	4	0	18.17917912	74.60779723	https://www.inaturalist.org/observations/57982361
330	Sturnus pagodarum	Brahminy Starling	2020/2/22	0	0	18.26338600	74.80422700	https://www.inaturalist.org/observations/39575324
331	Sturnus pagodarum	Brahminy Starling	2018/12/12	1	0	18.26439328	74.76938926	https://www.inaturalist.org/observations/53452053
332	Sturnus pagodarum	Brahminy Starling	2018/2/3	2	0	18.27139188	74.79532800	https://www.inaturalist.org/observations/56114816
333	Sturnus pagodarum	Brahminy Starling	2021/1/13	2	0	18.27264780	74.32062080	https://www.inaturalist.org/observations/70932608
334	Sturnus pagodarum	Brahminy Starling	2021/10/25	3	0	18.27264780	74.32062080	https://www.inaturalist.org/observations/121383463
335	Sturnus pagodarum	Brahminy Starling	2020/8/31	1	0	18.27303995	84.00484376	https://www.inaturalist.org/observations/95508134
336	Sturnus pagodarum	Brahminy Starling	2020/8/31	2	0	18.27303995	84.00484376	https://www.inaturalist.org/observations/95645356
337	Sturnus pagodarum	Brahminy Starling	2017/11/16	2	0	18.27328806	74.75735306	https://www.inaturalist.org/observations/55011463
338	Sturnus pagodarum	Brahminy Starling	2014/3/16	3	0	18.27891820	74.78029550	https://www.inaturalist.org/observations/55372888
339	Sturnus pagodarum	Brahminy Starling	2016/2/3	4	0	18.29144000	74.77390900	http://www.inaturalist.org/observations/2649257
340	Sturnus pagodarum	Brahminy Starling	2011/3/6	2	0	18.29158270	74.77408000	https://www.inaturalist.org/observations/56006666
341	Sturnus pagodarum	Brahminy Starling	2012/3/10	2	0	18.29158270	74.77408000	https://www.inaturalist.org/observations/56113762
342	Sturnus pagodarum	Brahminy Starling	2012/3/10	2	0	18.29158300	74.77408000	https://www.inaturalist.org/observations/55702055
343	Sturnus pagodarum	Brahminy Starling	2014/1/5	3	0	18.29894780	74.75425710	https://www.inaturalist.org/observations/41029765
344	Sturnus pagodarum	Brahminy Starling	2021/12/20	2	0	18.31899690	73.87596292	https://www.inaturalist.org/observations/105859673

345	Sturnus pagodarum	Brahminy Starling	2021/1/28	3	0	18.33481840	73.67215150	https://www.inaturalist.org/observations/70931505
346	Sturnus pagodarum	Brahminy Starling	2018/10/7	5	0	18.34631300	74.03018300	https://www.inaturalist.org/observations/19463415
347	Sturnus pagodarum	Brahminy Starling	2020/9/28	4	0	18.34807574	75.97382348	https://www.inaturalist.org/observations/61317291
348	Sturnus pagodarum	Brahminy Starling	2019/9/30	3	0	18.35098139	74.35528306	https://www.inaturalist.org/observations/52245739
349	Sturnus pagodarum	Brahminy Starling	2017/10/28	3	0	18.35154150	74.36754460	https://www.inaturalist.org/observations/42755852
350	Sturnus pagodarum	Brahminy Starling	2021/1/6	1	0	18.35154150	74.36754460	https://www.inaturalist.org/observations/70980201
351	Sturnus pagodarum	Brahminy Starling	2012/11/25	4	0	18.36527342	73.70661791	https://www.inaturalist.org/observations/19405252
352	Sturnus pagodarum	Brahminy Starling	2017/11/12	3	0	18.37389901	74.06659464	https://www.inaturalist.org/observations/8860131
353	Sturnus pagodarum	Brahminy Starling	2022/8/6	1	0	18.37586280	73.76916470	https://www.inaturalist.org/observations/137316648
354	Sturnus pagodarum	Brahminy Starling	2018/2/5	5	0	18.37586300	73.76916500	https://www.inaturalist.org/observations/33243081
355	Sturnus pagodarum	Brahminy Starling	2017/4/1	2	0	18.38027930	73.76764300	https://www.inaturalist.org/observations/41811000
356	Sturnus pagodarum	Brahminy Starling	2021/10/15	3	0	18.41206170	74.04945000	https://www.inaturalist.org/observations/98586791
357	Sturnus pagodarum	Brahminy Starling	2020/7/15	4	0	18.46809050	73.92936630	https://www.inaturalist.org/observations/54617876
358	Sturnus pagodarum	Brahminy Starling	2019/7/16	2	0	18.47464060	73.91126201	https://www.inaturalist.org/observations/56002324
359	Sturnus pagodarum	Brahminy Starling	2013/4/3	3	0	18.47802300	73.84043600	https://www.inaturalist.org/observations/122174349
360	Sturnus pagodarum	Brahminy Starling	2016/1/20	4	0	18.48126541	73.83204103	http://www.inaturalist.org/observations/3735655
361	Sturnus pagodarum	Brahminy Starling	2011/5/15	2	0	18.48184261	73.81061714	https://www.inaturalist.org/observations/68086453
362	Sturnus pagodarum	Brahminy Starling	2020/6/11	1	0	18.48379500	73.89529500	https://www.inaturalist.org/observations/49194777
363	Sturnus pagodarum	Brahminy Starling	2019/6/29	7	0	18.49268310	73.78751190	https://www.inaturalist.org/observations/32817309
364	Sturnus pagodarum	Brahminy Starling	2021/6/14	2	0	18.49736978	73.90093177	https://www.inaturalist.org/observations/88495993
365	Sturnus pagodarum	Brahminy Starling	2014/10/11	4	0	18.51770300	73.82297500	http://www.inaturalist.org/observations/3923271
366	Sturnus pagodarum	Brahminy Starling	2022/7/16	2	0	18.52853621	73.81250520	https://www.inaturalist.org/observations/126456149
367	Sturnus pagodarum	Brahminy Starling	2020/11/10	6	0	18.54301526	73.78889277	https://www.inaturalist.org/observations/64783799

368	Sturnus pagodarum	Brahminy Starling	2022/5/20	6	0	18.55380500	73.84229150	https://www.inaturalist.org/observations/117873669
369	Sturnus pagodarum	Brahminy Starling	2016/3/9	2	0	18.55403120	73.79045760	https://www.inaturalist.org/observations/141622188
370	Sturnus pagodarum	Brahminy Starling	2021/4/2	2	0	18.55525360	73.77869828	https://www.inaturalist.org/observations/72783743
371	Sturnus pagodarum	Brahminy Starling	2014/4/30	2	0	18.55808650	73.78454730	https://www.inaturalist.org/observations/141622187
372	Sturnus pagodarum	Brahminy Starling	2020/6/21	2	0	18.57902320	73.78599120	https://www.inaturalist.org/observations/50369975
373	Sturnus pagodarum	Brahminy Starling	2022/8/11	2	0	18.57979200	73.78994900	https://www.inaturalist.org/observations/130460378
374	Sturnus pagodarum	Brahminy Starling	2020/6/27	2	0	18.58311100	73.78616100	https://www.inaturalist.org/observations/51189364
375	Sturnus pagodarum	Brahminy Starling	2020/3/17	4	0	18.58449835	73.73891439	https://www.inaturalist.org/observations/40124580
376	Sturnus pagodarum	Brahminy Starling	2022/6/21	5	0	18.58800103	73.97943377	https://www.inaturalist.org/observations/122820792
377	Sturnus pagodarum	Brahminy Starling	2022/12/11	2	0	18.60326575	73.89715575	https://www.inaturalist.org/observations/144136321
378	Sturnus pagodarum	Brahminy Starling	2020/1/17	1	0	18.62066316	73.73172717	https://www.inaturalist.org/observations/37706392
379	Sturnus pagodarum	Brahminy Starling	2014/3/3	3	0	18.62182959	72.89190161	https://www.inaturalist.org/observations/53128971
380	Sturnus pagodarum	Brahminy Starling	2019/1/16	3	0	18.62426000	73.67040000	https://www.inaturalist.org/observations/53333734
381	Sturnus pagodarum	Brahminy Starling	2021/3/27	6	0	18.63584057	74.02088446	https://www.inaturalist.org/observations/121120207
382	Sturnus pagodarum	Brahminy Starling	2010/1/16	3	0	18.64917810	73.77068180	https://www.inaturalist.org/observations/81737990
383	Sturnus pagodarum	Brahminy Starling	2019/9/20	3	0	18.65839890	72.87733460	https://www.inaturalist.org/observations/101310141
384	Sturnus pagodarum	Brahminy Starling	2010/4/1	3	0	18.65840439	73.85193718	https://www.inaturalist.org/observations/58140353
385	Sturnus pagodarum	Brahminy Starling	2015/3/25	1	0	18.77826659	74.28176031	https://www.inaturalist.org/observations/82316779
386	Sturnus pagodarum	Brahminy Starling	2022/11/27	0	0	18.77866000	81.99711080	https://www.inaturalist.org/observations/144013998
387	Sturnus pagodarum	Brahminy Starling	2019/5/1	5	0	18.79199106	81.98299695	https://www.inaturalist.org/observations/24276761
388	Sturnus pagodarum	Brahminy Starling	2012/9/26	3	0	18.81808760	74.15629900	https://www.inaturalist.org/observations/122264284
389	Sturnus pagodarum	Brahminy Starling	2022/3/19	5	0	18.96530630	73.22607085	https://www.inaturalist.org/observations/109206572
390	Sturnus pagodarum	Brahminy Starling	2020/10/28	2	0	18.99034551	75.72751790	https://www.inaturalist.org/observations/63685016

391	Sturnus pagodarum	Brahminy Starling	2020/10/28	2	0	18.99238272	75.75412206	https://www.inaturalist.org/observations/63685004
392	Sturnus pagodarum	Brahminy Starling	2018/4/1	5	0	19.08585050	73.74043060	https://www.inaturalist.org/observations/30471754
393	Sturnus pagodarum	Brahminy Starling	2018/7/7	5	0	19.08862900	74.43976700	https://www.inaturalist.org/observations/14127187
394	Sturnus pagodarum	Brahminy Starling	2020/3/1	1	0	19.09482870	74.74797890	https://www.inaturalist.org/observations/55131999
395	Sturnus pagodarum	Brahminy Starling	2020/9/27	5	0	19.13940104	82.10490905	https://www.inaturalist.org/observations/61079037
396	Sturnus pagodarum	Brahminy Starling	2020/9/27	4	0	19.13940104	82.10490905	https://www.inaturalist.org/observations/61079198
397	Sturnus pagodarum	Brahminy Starling	2020/3/29	3	0	19.14022852	74.72464536	https://www.inaturalist.org/observations/42006750
398	Sturnus pagodarum	Brahminy Starling	2018/9/21	2	0	19.14192483	72.96462536	https://www.inaturalist.org/observations/56299976
399	Sturnus pagodarum	Brahminy Starling	2018/9/29	2	0	19.14192483	72.96462536	https://www.inaturalist.org/observations/56299981
400	Sturnus pagodarum	Brahminy Starling	2022/10/2	1	0	19.14639878	72.96264544	https://www.inaturalist.org/observations/137278812
401	Sturnus pagodarum	Brahminy Starling	2014/10/5	3	0	19.15055340	72.95648930	https://www.inaturalist.org/observations/41423274
402	Sturnus pagodarum	Brahminy Starling	2015/8/13	5	0	19.17881600	72.95766400	http://www.inaturalist.org/observations/1866231
403	Sturnus pagodarum	Brahminy Starling	2018/10/6	2	0	19.19094485	72.98145019	https://www.inaturalist.org/observations/136795771
404	Sturnus pagodarum	Brahminy Starling	2022/11/10	2	0	19.22619435	73.11445791	https://www.inaturalist.org/observations/142048667
405	Sturnus pagodarum	Brahminy Starling	2018/2/18	4	0	19.23119238	72.87062366	https://www.inaturalist.org/observations/11732922
406	Sturnus pagodarum	Brahminy Starling	2020/10/22	2	0	19.24617639	72.86224511	https://www.inaturalist.org/observations/63566285
407	Sturnus pagodarum	Brahminy Starling	2017/10/8	2	0	19.38246770	72.77177750	https://www.inaturalist.org/observations/42584095
408	Sturnus pagodarum	Brahminy Starling	2018/6/3	1	0	19.47694673	73.26611102	https://www.inaturalist.org/observations/54235435
409	Sturnus pagodarum	Brahminy Starling	2022/4/12	1	0	19.50804444	73.01498897	https://www.inaturalist.org/observations/111430873
410	Sturnus pagodarum	Brahminy Starling	2022/7/9	2	0	19.58783191	81.74497317	https://www.inaturalist.org/observations/125435790
411	Sturnus pagodarum	Brahminy Starling	2019/10/16	5	0	19.74957269	81.39759377	https://www.inaturalist.org/observations/34870606
412	Sturnus pagodarum	Brahminy Starling	2016/7/12	4	0	19.79388769	75.53366493	http://www.inaturalist.org/observations/3665049
413	Sturnus pagodarum	Brahminy Starling	2019/11/12	4	0	19.85178330	82.57295380	https://www.inaturalist.org/observations/35602314

414	Sturnus pagodarum	Brahminy Starling	2022/2/5	1	0	19.88155000	75.32180200	https://www.inaturalist.org/observations/106156451
415	Sturnus pagodarum	Brahminy Starling	2018/4/29	6	0	19.89595106	82.66551858	https://www.inaturalist.org/observations/15995005
416	Sturnus pagodarum	Brahminy Starling	2021/5/20	3	0	19.91370362	82.64322940	https://www.inaturalist.org/observations/79511338
417	Sturnus pagodarum	Brahminy Starling	2021/5/20	2	0	19.91370362	82.64322940	https://www.inaturalist.org/observations/79512503
418	Sturnus pagodarum	Brahminy Starling	2021/5/21	5	0	19.91370362	82.64322940	https://www.inaturalist.org/observations/79633308
419	Sturnus pagodarum	Brahminy Starling	2021/5/21	2	0	19.91370362	82.64322940	https://www.inaturalist.org/observations/79633331
420	Sturnus pagodarum	Brahminy Starling	2014/12/3	1	0	19.92030268	82.64342721	https://www.inaturalist.org/observations/37634478
421	Sturnus pagodarum	Brahminy Starling	2021/5/1	2	0	19.94954675	73.75447959	https://www.inaturalist.org/observations/77216035
422	Sturnus pagodarum	Brahminy Starling	2019/5/7	2	0	19.97775833	79.36187000	https://www.inaturalist.org/observations/68926815
423	Sturnus pagodarum	Brahminy Starling	2018/7/1	3	0	20.06107167	74.07043333	https://www.inaturalist.org/observations/21643106
424	Sturnus pagodarum	Brahminy Starling	2022/5/13	4	0	20.09757400	78.53379800	https://www.inaturalist.org/observations/129688172
425	Sturnus pagodarum	Brahminy Starling	2015/6/17	5	0	20.22773101	79.35905495	https://www.inaturalist.org/observations/12188961
426	Sturnus pagodarum	Brahminy Starling	2010/5/23	2	0	20.24838270	79.36068900	https://www.inaturalist.org/observations/55252616
427	Sturnus pagodarum	Brahminy Starling	2016/6/12	3	0	20.24838270	79.36068900	https://www.inaturalist.org/observations/58942638
428	Sturnus pagodarum	Brahminy Starling	2014/3/13	1	0	20.33728167	79.30447833	https://www.inaturalist.org/observations/37713595
429	Sturnus pagodarum	Brahminy Starling	2014/6/24	4	0	20.34002560	79.30590060	https://www.inaturalist.org/observations/58578973
430	Sturnus pagodarum	Brahminy Starling	2019/5/27	5	0	20.39696859	79.36176000	https://www.inaturalist.org/observations/28291076
431	Sturnus pagodarum	Brahminy Starling	2018/4/22	5	0	20.43386500	79.37371000	https://www.inaturalist.org/observations/31326013
432	Sturnus pagodarum	Brahminy Starling	2021/9/20	1	0	20.46251066	85.88298921	https://www.inaturalist.org/observations/95553471
433	Sturnus pagodarum	Brahminy Starling	2018/8/16	3	0	20.55185820	75.70325196	https://www.inaturalist.org/observations/134020622
434	Sturnus pagodarum	Brahminy Starling	2020/10/26	4	0	20.58213290	79.68882144	https://www.inaturalist.org/observations/63569265
435	Sturnus pagodarum	Brahminy Starling	2020/10/24	4	0	20.63999485	79.64073647	https://www.inaturalist.org/observations/63378218
436	Sturnus pagodarum	Brahminy Starling	2020/10/31	4	0	20.66714959	77.11365304	https://www.inaturalist.org/observations/63877546

437	Sturnus pagodarum	Brahminy Starling	2020/10/31	2	0	20.67023191	77.09094906	https://www.inaturalist.org/observations/63876629
438	Sturnus pagodarum	Brahminy Starling	2021/2/27	3	0	20.67389178	81.57873943	https://www.inaturalist.org/observations/82512232
439	Sturnus pagodarum	Brahminy Starling	2020/10/30	3	0	20.69251565	77.01767435	https://www.inaturalist.org/observations/63805763
440	Sturnus pagodarum	Brahminy Starling	2016/3/22	3	0	20.73693667	82.44619833	https://www.inaturalist.org/observations/61469219
441	Sturnus pagodarum	Brahminy Starling	2017/8/3	3	0	20.74922679	78.60007190	https://www.inaturalist.org/observations/58982756
442	Sturnus pagodarum	Brahminy Starling	2017/8/1	3	0	20.75000381	78.59854889	https://www.inaturalist.org/observations/58982752
443	Sturnus pagodarum	Brahminy Starling	2017/7/29	3	0	20.75002669	78.59890744	https://www.inaturalist.org/observations/58982748
444	Sturnus pagodarum	Brahminy Starling	2017/7/30	3	0	20.75002669	78.59890744	https://www.inaturalist.org/observations/58982750
445	Sturnus pagodarum	Brahminy Starling	2021/5/27	1	0	20.78994569	81.94478658	https://www.inaturalist.org/observations/80470959
446	Sturnus pagodarum	Brahminy Starling	2020/4/1	2	0	20.79200373	78.13800372	https://www.inaturalist.org/observations/41214139
447	Sturnus pagodarum	Brahminy Starling	2020/9/21	3	0	20.83460333	81.71864833	https://www.inaturalist.org/observations/60302584
448	Sturnus pagodarum	Brahminy Starling	2020/9/20	3	0	20.83469500	81.71886000	https://www.inaturalist.org/observations/60160842
449	Sturnus pagodarum	Brahminy Starling	2020/12/16	1	0	20.98186032	75.53844295	https://www.inaturalist.org/observations/66960484
450	Sturnus pagodarum	Brahminy Starling	2021/3/15	4	0	20.98462279	75.53618079	https://www.inaturalist.org/observations/71348149
451	Sturnus pagodarum	Brahminy Starling	2021/3/25	3	0	20.98462279	75.53618079	https://www.inaturalist.org/observations/72079124
452	Sturnus pagodarum	Brahminy Starling	2020/12/14	2	0	20.98611643	75.53506203	https://www.inaturalist.org/observations/66575159
453	Sturnus pagodarum	Brahminy Starling	2020/12/31	3	0	20.98960524	75.54502911	https://www.inaturalist.org/observations/67352102
454	Sturnus pagodarum	Brahminy Starling	2020/12/31	4	0	20.98960524	75.54502911	https://www.inaturalist.org/observations/67354146
455	Sturnus pagodarum	Brahminy Starling	2021/1/16	3	0	20.98963028	75.54377920	https://www.inaturalist.org/observations/70596680
456	Sturnus pagodarum	Brahminy Starling	2021/3/13	3	0	20.99093930	75.53067092	https://www.inaturalist.org/observations/71116325
457	Sturnus pagodarum	Brahminy Starling	2021/1/28	2	0	20.99520391	81.50424846	https://www.inaturalist.org/observations/68724532
458	Sturnus pagodarum	Brahminy Starling	2021/12/9	1	0	21.01207667	78.22368333	https://www.inaturalist.org/observations/102761043
459	Sturnus pagodarum	Brahminy Starling	2021/12/8	1	0	21.01222138	78.22364096	https://www.inaturalist.org/observations/102694318

460	Sturnus pagodarum	Brahminy Starling	2021/12/9	2	0	21.01226333	78.22352000	https://www.inaturalist.org/observations/102761036
461	Sturnus pagodarum	Brahminy Starling	2021/6/19	3	0	21.04115872	81.53543718	https://www.inaturalist.org/observations/83754640
462	Sturnus pagodarum	Brahminy Starling	2020/6/4	4	0	21.04831568	75.84103737	https://www.inaturalist.org/observations/50949806
463	Sturnus pagodarum	Brahminy Starling	2021/1/28	1	0	21.05220535	81.46011254	https://www.inaturalist.org/observations/68719682
464	Sturnus pagodarum	Brahminy Starling	2021/4/5	2	0	21.08470186	81.04141384	https://www.inaturalist.org/observations/72986531
465	Sturnus pagodarum	Brahminy Starling	2020/10/23	5	0	21.08993500	79.12437697	https://www.inaturalist.org/observations/63308035
466	Sturnus pagodarum	Brahminy Starling	2021/6/7	2	0	21.09147222	79.99029444	https://www.inaturalist.org/observations/82392329
467	Sturnus pagodarum	Brahminy Starling	2018/6/2	5	0	21.12425570	79.04596653	https://www.inaturalist.org/observations/13184906
468	Sturnus pagodarum	Brahminy Starling	2020/2/29	2	0	21.12430540	70.82415070	https://www.inaturalist.org/observations/137947529
469	Sturnus pagodarum	Brahminy Starling	2018/7/6	3	0	21.14452950	79.05796966	https://www.inaturalist.org/observations/21644342
470	Sturnus pagodarum	Brahminy Starling	2018/7/6	3	0	21.14452950	79.05796966	https://www.inaturalist.org/observations/21644358
471	Sturnus pagodarum	Brahminy Starling	2021/5/12	1	0	21.16028900	79.63494650	https://www.inaturalist.org/observations/78582584
472	Sturnus pagodarum	Brahminy Starling	2021/3/10	2	0	21.16652857	81.24198407	https://www.inaturalist.org/observations/87371145
473	Sturnus pagodarum	Brahminy Starling	2019/3/25	3	0	21.17227540	79.03494883	https://www.inaturalist.org/observations/46680472
474	Sturnus pagodarum	Brahminy Starling	2019/1/11	5	0	21.17739330	81.77364718	https://www.inaturalist.org/observations/23018542
475	Sturnus pagodarum	Brahminy Starling	2021/1/27	1	0	21.18773686	81.35820884	https://www.inaturalist.org/observations/68667764
476	Sturnus pagodarum	Brahminy Starling	2021/1/17	2	0	21.19838267	80.76030593	https://www.inaturalist.org/observations/68212506
477	Sturnus pagodarum	Brahminy Starling	2021/3/18	3	0	21.20309738	81.35703843	https://www.inaturalist.org/observations/71463438
478	Sturnus pagodarum	Brahminy Starling	2019/5/25	3	0	21.20517289	78.98499504	https://www.inaturalist.org/observations/65210755
479	Sturnus pagodarum	Brahminy Starling	2022/8/28	2	0	21.20575620	81.60818640	https://www.inaturalist.org/observations/134176947
480	Sturnus pagodarum	Brahminy Starling	2022/8/28	2	0	21.20575620	81.60818640	https://www.inaturalist.org/observations/134176955
481	Sturnus pagodarum	Brahminy Starling	2021/2/3	1	0	21.20948261	81.43681504	https://www.inaturalist.org/observations/140751601
482	Sturnus pagodarum	Brahminy Starling	2020/6/9	2	0	21.22941515	80.78620400	https://www.inaturalist.org/observations/49047211

483	Sturnus pagodarum	Brahminy Starling	2022/6/15	4	0	21.24685990	81.59744520	https://www.inaturalist.org/observations/121903010
484	Sturnus pagodarum	Brahminy Starling	2022/8/7	5	0	21.24742480	81.64579940	https://www.inaturalist.org/observations/129995200
485	Sturnus pagodarum	Brahminy Starling	2022/7/5	3	0	21.25876286	81.62378308	https://www.inaturalist.org/observations/124861751
486	Sturnus pagodarum	Brahminy Starling	2022/7/7	1	0	21.25876286	81.62378308	https://www.inaturalist.org/observations/125801582
487	Sturnus pagodarum	Brahminy Starling	2022/7/12	3	0	21.25876286	81.62378308	https://www.inaturalist.org/observations/129098891
488	Sturnus pagodarum	Brahminy Starling	2022/8/11	4	0	21.25876286	81.62378308	https://www.inaturalist.org/observations/130389043
489	Sturnus pagodarum	Brahminy Starling	2022/8/11	4	0	21.25876286	81.62378308	https://www.inaturalist.org/observations/130389047
490	Sturnus pagodarum	Brahminy Starling	2022/8/11	4	0	21.25876286	81.62378308	https://www.inaturalist.org/observations/130389048
491	Sturnus pagodarum	Brahminy Starling	2022/8/12	3	0	21.25876286	81.62378308	https://www.inaturalist.org/observations/130532448
492	Sturnus pagodarum	Brahminy Starling	2022/8/12	2	0	21.25876286	81.62378308	https://www.inaturalist.org/observations/130532449
493	Sturnus pagodarum	Brahminy Starling	2022/8/21	4	0	21.25876286	81.62378308	https://www.inaturalist.org/observations/131788468
494	Sturnus pagodarum	Brahminy Starling	2021/8/18	1	0	21.26001460	81.35850325	https://www.inaturalist.org/observations/93377992
495	Sturnus pagodarum	Brahminy Starling	2022/7/8	2	0	21.27667700	81.64460490	https://www.inaturalist.org/observations/125802359
496	Sturnus pagodarum	Brahminy Starling	2022/7/8	2	0	21.27667700	81.64460490	https://www.inaturalist.org/observations/125802361
497	Sturnus pagodarum	Brahminy Starling	2018/2/17	5	0	21.28480333	81.68136333	https://www.inaturalist.org/observations/10257227
498	Sturnus pagodarum	Brahminy Starling	2021/9/21	3	0	21.29296160	81.38125340	https://www.inaturalist.org/observations/121445455
499	Sturnus pagodarum	Brahminy Starling	2020/9/26	5	0	21.29743334	80.33759180	https://www.inaturalist.org/observations/60807442
500	Sturnus pagodarum	Brahminy Starling	2020/10/8	6	0	21.29786389	80.33801111	https://www.inaturalist.org/observations/63224146
501	Sturnus pagodarum	Brahminy Starling	2020/10/31	2	0	21.29795000	80.33799167	https://www.inaturalist.org/observations/63862077
502	Sturnus pagodarum	Brahminy Starling	2020/9/11	4	0	21.29811484	80.33807443	https://www.inaturalist.org/observations/59188961
503	Sturnus pagodarum	Brahminy Starling	2020/10/25	7	0	21.29917703	80.34442037	https://www.inaturalist.org/observations/63498536
504	Sturnus pagodarum	Brahminy Starling	2019/2/9	1	0	21.30396780	80.49104700	https://www.inaturalist.org/observations/90532319
505	Sturnus pagodarum	Brahminy Starling	2021/2/23	1	0	21.49425275	81.69074278	https://www.inaturalist.org/observations/70022402

506	Sturnus pagodarum	Brahminy Starling	2018/6/8	3	0	21.63591260	79.23227860	https://www.inaturalist.org/observations/56209647
507	Sturnus pagodarum	Brahminy Starling	2014/5/10	2	0	21.63591260	79.23227860	https://www.inaturalist.org/observations/56218677
508	Sturnus pagodarum	Brahminy Starling	2020/11/7	1	0	21.63882833	87.55034667	https://www.inaturalist.org/observations/145037120
509	Sturnus pagodarum	Brahminy Starling	2020/5/17	3	0	21.66611111	71.85472222	https://www.inaturalist.org/observations/47700271
510	Sturnus pagodarum	Brahminy Starling	2022/1/30	3	0	21.67311494	81.31010111	https://www.inaturalist.org/observations/107025156
511	Sturnus pagodarum	Brahminy Starling	2017/6/5	4	0	21.74516604	72.96657251	https://www.inaturalist.org/observations/6560457
512	Sturnus pagodarum	Brahminy Starling	2014/1/3	2	0	21.75604860	72.12570720	https://www.inaturalist.org/observations/39746138
513	Sturnus pagodarum	Brahminy Starling	2020/10/27	5	0	21.77398110	87.87584504	https://www.inaturalist.org/observations/144669454
514	Sturnus pagodarum	Brahminy Starling	2021/5/21	5	0	21.80647450	83.30023138	https://www.inaturalist.org/observations/79793941
515	Sturnus pagodarum	Brahminy Starling	2021/5/18	2	0	21.80803032	83.30533683	https://www.inaturalist.org/observations/79667408
516	Sturnus pagodarum	Brahminy Starling	2019/4/11	4	0	21.84337000	79.47858500	https://www.inaturalist.org/observations/122260926
517	Sturnus pagodarum	Brahminy Starling	2021/8/27	2	0	21.85094423	83.29285374	https://www.inaturalist.org/observations/92756166
518	Sturnus pagodarum	Brahminy Starling	2021/8/7	2	0	21.85284500	83.29309833	https://www.inaturalist.org/observations/91033508
519	Sturnus pagodarum	Brahminy Starling	2017/3/12	4	0	22.00740148	79.80473432	https://www.inaturalist.org/observations/5547744
520	Sturnus pagodarum	Brahminy Starling	2021/3/9	1	0	22.01608917	83.37870117	https://www.inaturalist.org/observations/79762488
521	Sturnus pagodarum	Brahminy Starling	2021/3/14	1	0	22.02199943	83.38026490	https://www.inaturalist.org/observations/81179166
522	Sturnus pagodarum	Brahminy Starling	2017/4/16	2	0	22.02701726	73.09037989	https://www.inaturalist.org/observations/89290552
523	Sturnus pagodarum	Brahminy Starling	2010/5/13	2	0	22.04100240	79.44157490	https://www.inaturalist.org/observations/55249442
524	Sturnus pagodarum	Brahminy Starling	2010/5/13	2	0	22.04100240	79.44157490	https://www.inaturalist.org/observations/55249443
525	Sturnus pagodarum	Brahminy Starling	2022/5/17	3	0	22.05506400	77.86490100	https://www.inaturalist.org/observations/129802114
526	Sturnus pagodarum	Brahminy Starling	2021/9/5	1	0	22.06009167	82.20110000	https://www.inaturalist.org/observations/93744389
527	Sturnus pagodarum	Brahminy Starling	2021/8/15	1	0	22.06620000	82.21142833	https://www.inaturalist.org/observations/93617544
528	Sturnus pagodarum	Brahminy Starling	2021/8/15	1	0	22.06740500	82.20358000	https://www.inaturalist.org/observations/93615062

529	Sturnus pagodarum	Brahminy Starling	2021/12/26	1	0	22.07596639	81.55972306	https://www.inaturalist.org/observations/106623457
530	Sturnus pagodarum	Brahminy Starling	2019/10/2	3	0	22.07965450	82.14091520	https://www.inaturalist.org/observations/60433154
531	Sturnus pagodarum	Brahminy Starling	2017/2/9	4	0	22.07965450	82.14091520	https://www.inaturalist.org/observations/60975778
532	Sturnus pagodarum	Brahminy Starling	2019/9/11	4	0	22.13023950	82.13692356	https://www.inaturalist.org/observations/32634523
533	Sturnus pagodarum	Brahminy Starling	2021/8/31	0	0	22.13072167	82.13716500	https://www.inaturalist.org/observations/93439088
534	Sturnus pagodarum	Brahminy Starling	2019/9/11	4	0	22.13194921	82.13915918	https://www.inaturalist.org/observations/32974252
535	Sturnus pagodarum	Brahminy Starling	2022/6/27	3	0	22.15321934	71.31711602	https://www.inaturalist.org/observations/123620963
536	Sturnus pagodarum	Brahminy Starling	2021/8/4	3	0	22.17024929	75.58753137	https://www.inaturalist.org/observations/107754746
537	Sturnus pagodarum	Brahminy Starling	2020/7/18	1	0	22.18611111	76.06388889	https://www.inaturalist.org/observations/53468832
538	Sturnus pagodarum	Brahminy Starling	2021/11/20	2	0	22.20558378	81.39996383	https://www.inaturalist.org/observations/102643442
539	Sturnus pagodarum	Brahminy Starling	2021/4/17	2	0	22.23070704	81.42031141	https://www.inaturalist.org/observations/80220840
540	Sturnus pagodarum	Brahminy Starling	2021/9/3	1	0	22.28255667	82.06666667	https://www.inaturalist.org/observations/93622495
541	Sturnus pagodarum	Brahminy Starling	2021/9/1	1	0	22.29788500	82.08831167	https://www.inaturalist.org/observations/93446706
542	Sturnus pagodarum	Brahminy Starling	2021/9/1	1	0	22.29788500	82.08831167	https://www.inaturalist.org/observations/93446707
543	Sturnus pagodarum	Brahminy Starling	2021/9/1	1	0	22.29797000	82.08824000	https://www.inaturalist.org/observations/93440834
544	Sturnus pagodarum	Brahminy Starling	2021/9/1	1	0	22.30192667	82.08517667	https://www.inaturalist.org/observations/93442888
545	Sturnus pagodarum	Brahminy Starling	2021/4/13	4	0	22.31271588	73.28538168	https://www.inaturalist.org/observations/105208834
546	Sturnus pagodarum	Brahminy Starling	2019/2/13	1	0	22.34446500	80.45526990	https://www.inaturalist.org/observations/90508028
547	Sturnus pagodarum	Brahminy Starling	2022/6/10	5	0	22.36570667	80.53910167	https://www.inaturalist.org/observations/121596136
548	Sturnus pagodarum	Brahminy Starling	2016/6/5	2	0	22.37015650	70.10390320	https://www.inaturalist.org/observations/137947530
549	Sturnus pagodarum	Brahminy Starling	2020/12/11	1	0	22.38098147	69.83286001	https://www.inaturalist.org/observations/66459516
550	Sturnus pagodarum	Brahminy Starling	2022/2/20	4	0	22.41889400	74.56501400	https://www.inaturalist.org/observations/126818947
551	Sturnus pagodarum	Brahminy Starling	2022/2/20	3	0	22.42425167	73.60961333	https://www.inaturalist.org/observations/120020534

552	Sturnus pagodarum	Brahminy Starling	2022/2/20	3	0	22.42459667	73.60990000	https://www.inaturalist.org/observations/120020528
553	Sturnus pagodarum	Brahminy Starling	2022/10/25	6	0	22.46644500	73.48081333	https://www.inaturalist.org/observations/140639636
554	Sturnus pagodarum	Brahminy Starling	2022/4/5	2	0	22.48092000	73.49486833	https://www.inaturalist.org/observations/119413930
555	Sturnus pagodarum	Brahminy Starling	2022/4/5	2	0	22.48092500	73.49481167	https://www.inaturalist.org/observations/119413920
556	Sturnus pagodarum	Brahminy Starling	2022/6/5	2	0	22.48228000	73.52048333	https://www.inaturalist.org/observations/120643904
557	Sturnus pagodarum	Brahminy Starling	2016/5/7	2	0	22.50333100	78.43585080	https://www.inaturalist.org/observations/55504320
558	Sturnus pagodarum	Brahminy Starling	2021/3/25	1	0	22.50881333	73.47400833	https://www.inaturalist.org/observations/97887893
559	Sturnus pagodarum	Brahminy Starling	2022/2/8	3	0	22.50905500	73.47402833	https://www.inaturalist.org/observations/120502454
560	Sturnus pagodarum	Brahminy Starling	2022/2/8	3	0	22.50924833	73.47420667	https://www.inaturalist.org/observations/120502450
561	Sturnus pagodarum	Brahminy Starling	2022/7/3	4	0	22.54068700	72.96746350	https://www.inaturalist.org/observations/124504602
562	Sturnus pagodarum	Brahminy Starling	2021/9/22	1	0	22.55859095	78.48225038	https://www.inaturalist.org/observations/96865693
563	Sturnus pagodarum	Brahminy Starling	2021/7/11	1	0	22.59588034	120.35727250	https://www.inaturalist.org/observations/86520167
564	Sturnus pagodarum	Brahminy Starling	2020/12/27	1	0	22.66964531	85.62969206	https://www.inaturalist.org/observations/99643647
565	Sturnus pagodarum	Brahminy Starling	2011/6/8	4	0	22.71872910	71.63766940	https://www.inaturalist.org/observations/32860639
566	Sturnus pagodarum	Brahminy Starling	2022/1/9	2	0	22.74866167	74.33485333	https://www.inaturalist.org/observations/120793610
567	Sturnus pagodarum	Brahminy Starling	2022/10/24	3	0	22.76333305	86.45125897	https://www.inaturalist.org/observations/142660208
568	Sturnus pagodarum	Brahminy Starling	2020/8/2	3	0	22.80481964	86.20284028	https://www.inaturalist.org/observations/55386248
569	Sturnus pagodarum	Brahminy Starling	2021/12/18	3	0	22.91295408	83.92232493	https://www.inaturalist.org/observations/105911893
570	Sturnus pagodarum	Brahminy Starling	2021/4/18	4	0	22.97715962	83.03382911	https://www.inaturalist.org/observations/79672940
571	Sturnus pagodarum	Brahminy Starling	2020/4/16	6	0	22.98918582	87.00715633	https://www.inaturalist.org/observations/42942142
572	Sturnus pagodarum	Brahminy Starling	2020/3/7	1	0	22.98967966	87.00827213	https://www.inaturalist.org/observations/40590549
573	Sturnus pagodarum	Brahminy Starling	2020/4/6	3	0	22.98968126	87.00751440	https://www.inaturalist.org/observations/42086358
574	Sturnus pagodarum	Brahminy Starling	2018/4/4	4	0	22.98969309	87.00769545	https://www.inaturalist.org/observations/21107623

575	Sturnus pagodarum	Brahminy Starling	2018/4/13	2	0	22.98970339	87.00751191	https://www.inaturalist.org/observations/50587897
576	Sturnus pagodarum	Brahminy Starling	2018/4/13	2	0	22.98970339	87.00751191	https://www.inaturalist.org/observations/50587911
577	Sturnus pagodarum	Brahminy Starling	2018/5/14	1	0	22.99045004	87.00867982	https://www.inaturalist.org/observations/54556971
578	Sturnus pagodarum	Brahminy Starling	2018/5/15	1	0	22.99045004	87.00867982	https://www.inaturalist.org/observations/54556973
579	Sturnus pagodarum	Brahminy Starling	2019/5/25	2	0	23.01236553	72.50395361	https://www.inaturalist.org/observations/139519785
580	Sturnus pagodarum	Brahminy Starling	2020/10/18	5	0	23.02296084	72.57136188	https://www.inaturalist.org/observations/62870675
581	Sturnus pagodarum	Brahminy Starling	2019/5/25	4	0	23.14013739	72.39283908	https://www.inaturalist.org/observations/124542168
582	Sturnus pagodarum	Brahminy Starling	2019/5/26	4	0	23.14056223	72.40599364	https://www.inaturalist.org/observations/25788070
583	Sturnus pagodarum	Brahminy Starling	2020/1/5	4	0	23.14637500	72.42105556	https://www.inaturalist.org/observations/37344200
584	Sturnus pagodarum	Brahminy Starling	2018/11/15	2	0	23.15283350	71.39578430	https://www.inaturalist.org/observations/56308545
585	Sturnus pagodarum	Brahminy Starling	2020/2/22	5	0	23.15433302	86.74763516	https://www.inaturalist.org/observations/39227928
586	Sturnus pagodarum	Brahminy Starling	2021/12/26	0	0	23.18779333	87.97023196	https://www.inaturalist.org/observations/111617297
587	Sturnus pagodarum	Brahminy Starling	2022/1/13	2	0	23.19126600	77.40443700	https://www.inaturalist.org/observations/105020224
588	Sturnus pagodarum	Brahminy Starling	2012/2/29	4	0	23.20188699	75.76545101	https://www.inaturalist.org/observations/34000101
589	Sturnus pagodarum	Brahminy Starling	2021/11/5	4	0	23.23420087	87.05475781	https://www.inaturalist.org/observations/133196192
590	Sturnus pagodarum	Brahminy Starling	2022/10/24	3	0	23.25201463	87.06437462	https://www.inaturalist.org/observations/139852277
591	Sturnus pagodarum	Brahminy Starling	2018/8/19	2	0	23.25222222	87.06500000	https://www.inaturalist.org/observations/136253044
592	Sturnus pagodarum	Brahminy Starling	2022/10/27	2	0	23.25229366	87.06447931	https://www.inaturalist.org/observations/140131423
593	Sturnus pagodarum	Brahminy Starling	2021/5/23	2	0	23.25975327	69.37745295	https://www.inaturalist.org/observations/136010841
594	Sturnus pagodarum	Brahminy Starling	2022/10/18	1	0	23.26096643	71.73752351	https://www.inaturalist.org/observations/139719667
595	Sturnus pagodarum	Brahminy Starling	2017/6/12	4	0	23.31688780	86.37077860	https://www.inaturalist.org/observations/16761403
596	Sturnus pagodarum	Brahminy Starling	2021/5/21	3	0	23.34159183	82.57640585	https://www.inaturalist.org/observations/79650038
597	Sturnus pagodarum	Brahminy Starling	2021/9/4	2	0	23.34694546	77.78071333	https://www.inaturalist.org/observations/93734776

598	Sturnus pagodarum	Brahminy Starling	2019/9/29	2	0	23.36521925	69.26935303	https://www.inaturalist.org/observations/137607992
599	Sturnus pagodarum	Brahminy Starling	2021/2/5	1	0	23.37515227	72.03994621	https://www.inaturalist.org/observations/69196699
600	Sturnus pagodarum	Brahminy Starling	2014/1/4	2	0	23.39192948	69.25922243	https://www.inaturalist.org/observations/36295235
601	Sturnus pagodarum	Brahminy Starling	2016/12/17	2	0	23.46182050	71.28742090	https://www.inaturalist.org/observations/91123939
602	Sturnus pagodarum	Brahminy Starling	2022/2/10	4	0	23.48325600	77.50461000	https://www.inaturalist.org/observations/107295102
603	Sturnus pagodarum	Brahminy Starling	2015/5/6	4	0	23.48333000	87.31667000	http://www.inaturalist.org/observations/1464410
604	Sturnus pagodarum	Brahminy Starling	2016/8/18	4	0	23.48644200	87.30025000	http://www.inaturalist.org/observations/3960710
605	Sturnus pagodarum	Brahminy Starling	2014/7/5	5	0	23.49177500	87.30574300	http://www.inaturalist.org/observations/769791
606	Sturnus pagodarum	Brahminy Starling	2019/3/17	4	0	23.51517414	87.37993222	https://www.inaturalist.org/observations/21319280
607	Sturnus pagodarum	Brahminy Starling	2017/1/17	2	0	23.52044430	87.31192270	https://www.inaturalist.org/observations/6127444
608	Sturnus pagodarum	Brahminy Starling	2021/6/30	1	0	23.54014500	72.71786167	https://www.inaturalist.org/observations/96565674
609	Sturnus pagodarum	Brahminy Starling	2019/8/11	1	0	23.58142388	86.94135972	https://www.inaturalist.org/observations/97655729
610	Sturnus pagodarum	Brahminy Starling	2021/11/6	1	0	23.66021030	86.94684950	https://www.inaturalist.org/observations/100487419
611	Sturnus pagodarum	Brahminy Starling	2015/2/26	3	0	23.68555030	81.02581680	https://www.inaturalist.org/observations/69187551
612	Sturnus pagodarum	Brahminy Starling	2015/2/22	3	0	23.68555030	81.02581680	https://www.inaturalist.org/observations/69229855
613	Sturnus pagodarum	Brahminy Starling	2015/5/25	2	0	23.69778206	81.04236917	https://www.inaturalist.org/observations/139900518
614	Sturnus pagodarum	Brahminy Starling	2017/3/5	4	0	23.70658852	81.02660656	https://www.inaturalist.org/observations/5860496
615	Sturnus pagodarum	Brahminy Starling	2021/6/15	3	0	23.72221060	81.02327100	https://www.inaturalist.org/observations/83608154
616	Sturnus pagodarum	Brahminy Starling	2022/1/11	4	0	23.73982264	86.94924138	https://www.inaturalist.org/observations/104768166
617	Sturnus pagodarum	Brahminy Starling	2018/5/27	5	0	23.81931333	86.25328000	https://www.inaturalist.org/observations/12852548
618	Sturnus pagodarum	Brahminy Starling	2020/9/8	3	0	23.82550960	86.45462520	https://www.inaturalist.org/observations/59019193
619	Sturnus pagodarum	Brahminy Starling	2020/10/19	2	0	23.84950665	77.93709688	https://www.inaturalist.org/observations/64918300
620	Sturnus pagodarum	Brahminy Starling	2022/3/6	2	0	23.96961167	72.99303833	https://www.inaturalist.org/observations/120128416

621	Sturnus pagodarum	Brahminy Starling	2022/3/5	2	0	23.98740333	72.96817833	https://www.inaturalist.org/observations/120122870
622	Sturnus pagodarum	Brahminy Starling	2019/7/27	4	0	23.99049333	79.39657833	https://www.inaturalist.org/observations/29625045
623	Sturnus pagodarum	Brahminy Starling	2014/2/4	2	0	24.04542860	70.14558050	https://www.inaturalist.org/observations/139685262
624	Sturnus pagodarum	Brahminy Starling	2021/11/16	0	0	24.06749153	73.69206238	https://www.inaturalist.org/observations/101253027
625	Sturnus pagodarum	Brahminy Starling	2020/5/23	4	0	24.09147701	72.20644433	https://www.inaturalist.org/observations/47215057
626	Sturnus pagodarum	Brahminy Starling	2020/7/16	2	0	24.21068667	82.86453500	https://www.inaturalist.org/observations/72995532
627	Sturnus pagodarum	Brahminy Starling	2022/3/9	5	0	24.50151111	73.66327222	https://www.inaturalist.org/observations/108240905
628	Sturnus pagodarum	Brahminy Starling	2014/11/15	1	0	24.58544500	73.71247900	https://www.inaturalist.org/observations/95916244
629	Sturnus pagodarum	Brahminy Starling	2010/5/15	6	0	24.60168500	73.66032600	http://www.inaturalist.org/observations/326651
630	Sturnus pagodarum	Brahminy Starling	2016/9/22	2	0	24.62410000	73.65060000	https://www.inaturalist.org/observations/73924398
631	Sturnus pagodarum	Brahminy Starling	2019/12/19	5	0	24.67228273	77.13952395	https://www.inaturalist.org/observations/66196649
632	Sturnus pagodarum	Brahminy Starling	2021/7/5	1	0	24.78022167	71.64398000	https://www.inaturalist.org/observations/85756177
633	Sturnus pagodarum	Brahminy Starling	2020/5/20	2	0	24.78437064	85.00080463	https://www.inaturalist.org/observations/47003821
634	Sturnus pagodarum	Brahminy Starling	2018/10/26	4	0	24.84387500	79.92421300	https://www.inaturalist.org/observations/126892870
635	Sturnus pagodarum	Brahminy Starling	2019/4/10	3	0	24.85786333	79.92194500	https://www.inaturalist.org/observations/36820907
636	Sturnus pagodarum	Brahminy Starling	2009/10/13	2	0	24.88512903	73.69412794	https://www.inaturalist.org/observations/135534403
637	Sturnus pagodarum	Brahminy Starling	2019/2/14	5	0	24.93059540	75.59092590	https://www.inaturalist.org/observations/20335919
638	Sturnus pagodarum	Brahminy Starling	2019/2/14	5	0	24.93059540	75.59092590	https://www.inaturalist.org/observations/20339540
639	Sturnus pagodarum	Brahminy Starling	2021/4/14	1	0	25.01979577	85.42531047	https://www.inaturalist.org/observations/73939609
640	Sturnus pagodarum	Brahminy Starling	2020/6/28	2	0	25.02161591	74.87999316	https://www.inaturalist.org/observations/51335138
641	Sturnus pagodarum	Brahminy Starling	2020/3/11	4	0	25.05580498	73.14152305	https://www.inaturalist.org/observations/66088768
642	Sturnus pagodarum	Brahminy Starling	2018/7/8	1	0	25.11604070	73.47252700	https://www.inaturalist.org/observations/46895829
643	Sturnus pagodarum	Brahminy Starling	2014/12/27	5	0	25.23000000	73.23000000	http://www.inaturalist.org/observations/1160782

644	Sturnus pagodarum	Brahminy Starling	2020/12/29	3	0	25.24245305	86.98422544	https://www.inaturalist.org/observations/67242846
645	Sturnus pagodarum	Brahminy Starling	2015/1/18	4	0	25.35145000	78.64071000	http://www.inaturalist.org/observations/1555470
646	Sturnus pagodarum	Brahminy Starling	2021/9/11	3	0	25.41010684	81.84946451	https://www.inaturalist.org/observations/94902995
647	Sturnus pagodarum	Brahminy Starling	2018/11/5	4	0	25.45395470	81.84786224	https://www.inaturalist.org/observations/18124383
648	Sturnus pagodarum	Brahminy Starling	2011/10/31	1	0	25.45756382	75.64539819	https://www.inaturalist.org/observations/45506126
649	Sturnus pagodarum	Brahminy Starling	2020/5/10	2	0	25.61641281	81.62320208	https://www.inaturalist.org/observations/45429276
650	Sturnus pagodarum	Brahminy Starling	2022/4/30	3	0	25.88823833	77.07762667	https://www.inaturalist.org/observations/118519141
651	Sturnus pagodarum	Brahminy Starling	2022/5/1	5	0	25.89155000	77.01548000	https://www.inaturalist.org/observations/118519154
652	Sturnus pagodarum	Brahminy Starling	2022/5/1	4	0	25.89178667	77.02279500	https://www.inaturalist.org/observations/118519145
653	Sturnus pagodarum	Brahminy Starling	2022/4/30	4	0	25.89573167	77.04153000	https://www.inaturalist.org/observations/118519056
654	Sturnus pagodarum	Brahminy Starling	2022/4/30	4	0	25.89865167	77.04303000	https://www.inaturalist.org/observations/118519066
655	Sturnus pagodarum	Brahminy Starling	2015/5/24	3	0	25.93936626	76.37314689	https://www.inaturalist.org/observations/140578245
656	Sturnus pagodarum	Brahminy Starling	2018/9/25	3	0	26.01601500	76.50401970	https://www.inaturalist.org/observations/20249853
657	Sturnus pagodarum	Brahminy Starling	2014/5/28	2	0	26.01732740	76.50257420	https://www.inaturalist.org/observations/55378871
658	Sturnus pagodarum	Brahminy Starling	2014/5/29	2	0	26.01732740	76.50257420	https://www.inaturalist.org/observations/55379509
659	Sturnus pagodarum	Brahminy Starling	2015/5/27	2	0	26.01732740	76.50257420	https://www.inaturalist.org/observations/55474962
660	Sturnus pagodarum	Brahminy Starling	2016/4/21	2	0	26.01732740	76.50257420	https://www.inaturalist.org/observations/55500710
661	Sturnus pagodarum	Brahminy Starling	2015/5/24	2	0	26.01732740	76.50257420	https://www.inaturalist.org/observations/56407968
662	Sturnus pagodarum	Brahminy Starling	2015/5/26	2	0	26.01732740	76.50257420	https://www.inaturalist.org/observations/56408062
663	Sturnus pagodarum	Brahminy Starling	2018/5/28	3	0	26.01732740	76.50257420	https://www.inaturalist.org/observations/56627053
664	Sturnus pagodarum	Brahminy Starling	2018/5/28	2	0	26.01732740	76.50257420	https://www.inaturalist.org/observations/56627062
665	Sturnus pagodarum	Brahminy Starling	2022/6/5	5	0	26.01732740	76.50257420	https://www.inaturalist.org/observations/130130700
666	Sturnus pagodarum	Brahminy Starling	2019/12/29	5	0	26.01859798	76.37501493	https://www.inaturalist.org/observations/37098127

667	Sturnus pagodarum	Brahminy Starling	2015/11/12	4	0	26.02089961	76.38034987	http://conabio.inaturalist.org/observations/5522966
668	Sturnus pagodarum	Brahminy Starling	2019/12/28	2	0	26.02126050	76.45208040	https://www.inaturalist.org/observations/37780309
669	Sturnus pagodarum	Brahminy Starling	2019/11/17	3	0	26.02220641	76.45388807	https://www.inaturalist.org/observations/36216216
670	Sturnus pagodarum	Brahminy Starling	2014/8/22	2	0	26.02914524	76.44600112	https://www.inaturalist.org/observations/64933300
671	Sturnus pagodarum	Brahminy Starling	2017/6/5	2	0	26.03292110	76.50572583	https://www.inaturalist.org/observations/54063947
672	Sturnus pagodarum	Brahminy Starling	2014/8/20	2	0	26.05614200	76.42516800	https://www.inaturalist.org/observations/64930888
673	Sturnus pagodarum	Brahminy Starling	2019/8/17	3	0	26.21658570	74.86825690	https://www.inaturalist.org/observations/31340173
674	Sturnus pagodarum	Brahminy Starling	2020/2/20	1	0	26.21855298	78.18283081	https://www.inaturalist.org/observations/95512510
675	Sturnus pagodarum	Brahminy Starling	2019/8/17	1	0	26.45009294	74.63991635	https://www.inaturalist.org/observations/101817762
676	Sturnus pagodarum	Brahminy Starling	2019/11/14	3	0	26.45034239	74.63991635	https://www.inaturalist.org/observations/37034233
677	Sturnus pagodarum	Brahminy Starling	2019/10/12	2	0	26.48974900	74.55108560	https://www.inaturalist.org/observations/60390318
678	Sturnus pagodarum	Brahminy Starling	2019/10/12	2	0	26.48974900	74.55108560	https://www.inaturalist.org/observations/60390341
679	Sturnus pagodarum	Brahminy Starling	2019/10/12	2	0	26.49012453	74.55287342	https://www.inaturalist.org/observations/44347970
680	Sturnus pagodarum	Brahminy Starling	2022/10/23	2	0	26.69377250	75.87953780	https://www.inaturalist.org/observations/139717084
681	Sturnus pagodarum	Brahminy Starling	2013/12/23	4	0	26.78729922	78.47877502	https://www.inaturalist.org/observations/11402832
682	Sturnus pagodarum	Brahminy Starling	2019/8/2	4	0	26.80590833	75.81154167	https://www.inaturalist.org/observations/30142203
683	Sturnus pagodarum	Brahminy Starling	2016/12/19	2	0	26.80698780	80.94841950	https://www.inaturalist.org/observations/71553975
684	Sturnus pagodarum	Brahminy Starling	2022/7/5	3	0	26.81367778	75.84281667	https://www.inaturalist.org/observations/124831885
685	Sturnus pagodarum	Brahminy Starling	2016/9/20	1	0	26.84203461	75.82252537	https://www.inaturalist.org/observations/74110510
686	Sturnus pagodarum	Brahminy Starling	2016/9/20	2	0	26.84203461	75.82252537	https://www.inaturalist.org/observations/74110512
687	Sturnus pagodarum	Brahminy Starling	2022/3/15	1	0	26.84724150	75.74126900	https://www.inaturalist.org/observations/111075782
688	Sturnus pagodarum	Brahminy Starling	2022/3/15	1	0	26.84724150	75.74126900	https://www.inaturalist.org/observations/111075783
689	Sturnus pagodarum	Brahminy Starling	2022/3/15	1	0	26.84738310	75.73988480	https://www.inaturalist.org/observations/111077891

690	Sturnus pagodarum	Brahminy Starling	2022/8/15	3	0	26.85491140	75.83284110	https://www.inaturalist.org/observations/132146938
691	Sturnus pagodarum	Brahminy Starling	2022/8/15	4	0	26.85491140	75.83284110	https://www.inaturalist.org/observations/132146943
692	Sturnus pagodarum	Brahminy Starling	2022/7/19	2	0	26.85544160	75.83234180	https://www.inaturalist.org/observations/129019092
693	Sturnus pagodarum	Brahminy Starling	2019/5/11	2	0	26.85779228	80.97396386	https://www.inaturalist.org/observations/48326215
694	Sturnus pagodarum	Brahminy Starling	2020/3/10	2	0	26.86428460	80.96917638	https://www.inaturalist.org/observations/69848186
695	Sturnus pagodarum	Brahminy Starling	2010/7/17	3	0	26.87553495	75.78299150	https://www.inaturalist.org/observations/80290161
696	Sturnus pagodarum	Brahminy Starling	2018/4/15	2	0	26.87703720	75.81594930	https://www.inaturalist.org/observations/55582797
697	Sturnus pagodarum	Brahminy Starling	2019/7/7	4	0	26.87703729	75.81594933	https://www.inaturalist.org/observations/38450073
698	Sturnus pagodarum	Brahminy Starling	2019/7/7	3	0	26.87703729	75.81594933	https://www.inaturalist.org/observations/40858922
699	Sturnus pagodarum	Brahminy Starling	2013/10/23	3	0	26.89324887	78.34378163	https://www.inaturalist.org/observations/69357554
700	Sturnus pagodarum	Brahminy Starling	2018/3/14	4	0	26.90448699	75.80751646	https://www.inaturalist.org/observations/10585586
701	Sturnus pagodarum	Brahminy Starling	2022/7/12	2	0	26.91236591	75.78727081	https://www.inaturalist.org/observations/125888595
702	Sturnus pagodarum	Brahminy Starling	2016/7/13	3	0	26.91277011	75.78727081	https://www.inaturalist.org/observations/140983452
703	Sturnus pagodarum	Brahminy Starling	2022/7/18	3	0	26.91686047	75.86473536	https://www.inaturalist.org/observations/128741850
704	Sturnus pagodarum	Brahminy Starling	2022/7/18	3	0	26.91686047	75.86473536	https://www.inaturalist.org/observations/128741851
705	Sturnus pagodarum	Brahminy Starling	2022/7/18	3	0	26.91686047	75.86473536	https://www.inaturalist.org/observations/128741852
706	Sturnus pagodarum	Brahminy Starling	2022/7/18	3	0	26.91686047	75.86473536	https://www.inaturalist.org/observations/128741855
707	Sturnus pagodarum	Brahminy Starling	2022/3/25	6	0	26.91693580	75.85896961	https://www.inaturalist.org/observations/109449733
708	Sturnus pagodarum	Brahminy Starling	2022/6/29	3	0	26.91759486	75.85433645	https://www.inaturalist.org/observations/123970313
709	Sturnus pagodarum	Brahminy Starling	2022/3/26	3	0	26.92518901	75.80162298	https://www.inaturalist.org/observations/109506952
710	Sturnus pagodarum	Brahminy Starling	2014/5/26	2	0	26.92577193	75.82365632	https://www.inaturalist.org/observations/69292333
711	Sturnus pagodarum	Brahminy Starling	2012/2/19	3	0	26.93731300	75.81552600	https://www.inaturalist.org/observations/104812169
712	Sturnus pagodarum	Brahminy Starling	2013/12/24	2	0	26.96699140	75.84833530	https://www.inaturalist.org/observations/66942002

713	Sturnus pagodarum	Brahminy Starling	2022/7/19	4	0	27.09640516	76.28617074	https://www.inaturalist.org/observations/130084755
714	Sturnus pagodarum	Brahminy Starling	2020/6/30	1	0	27.15175687	77.52402403	https://www.inaturalist.org/observations/51487166
715	Sturnus pagodarum	Brahminy Starling	2016/2/9	3	0	27.15691986	77.52365112	https://www.inaturalist.org/observations/47798497
716	Sturnus pagodarum	Brahminy Starling	2011/1/27	5	0	27.15692000	77.52365100	https://www.inaturalist.org/observations/24371111
717	Sturnus pagodarum	Brahminy Starling	2011/1/27	5	0	27.15692000	77.52365100	https://www.inaturalist.org/observations/24371125
718	Sturnus pagodarum	Brahminy Starling	2016/12/19	3	0	27.15692000	77.52365100	https://www.inaturalist.org/observations/41366706
719	Sturnus pagodarum	Brahminy Starling	2016/12/20	3	0	27.15692000	77.52365100	https://www.inaturalist.org/observations/41366784
720	Sturnus pagodarum	Brahminy Starling	2010/8/30	3	0	27.15692000	77.52365100	https://www.inaturalist.org/observations/42171303
721	Sturnus pagodarum	Brahminy Starling	2019/3/12	3	0	27.15692000	77.52365100	https://www.inaturalist.org/observations/42552829
722	Sturnus pagodarum	Brahminy Starling	2014/12/6	4	0	27.15692000	77.52365100	https://www.inaturalist.org/observations/42928688
723	Sturnus pagodarum	Brahminy Starling	2019/12/16	1	0	27.15692000	77.52365100	https://www.inaturalist.org/observations/105767535
724	Sturnus pagodarum	Brahminy Starling	2016/12/10	1	0	27.15692000	77.52365100	https://www.inaturalist.org/observations/105767702
725	Sturnus pagodarum	Brahminy Starling	2017/2/4	4	0	27.15692463	77.52365112	https://www.inaturalist.org/observations/61104240
726	Sturnus pagodarum	Brahminy Starling	2019/1/4	5	0	27.15911519	77.52113996	https://www.inaturalist.org/observations/120664038
727	Sturnus pagodarum	Brahminy Starling	2019/12/17	1	0	27.15925594	77.52319984	https://www.inaturalist.org/observations/92876009
728	Sturnus pagodarum	Brahminy Starling	2017/2/8	2	0	27.15925594	77.52319984	https://www.inaturalist.org/observations/106900610
729	Sturnus pagodarum	Brahminy Starling	2017/2/6	3	0	27.15925594	77.52319984	https://www.inaturalist.org/observations/106962130
730	Sturnus pagodarum	Brahminy Starling	2016/2/3	3	0	27.15925594	77.52319984	https://www.inaturalist.org/observations/106962141
731	Sturnus pagodarum	Brahminy Starling	2016/1/31	1	0	27.15925594	77.52319984	https://www.inaturalist.org/observations/107034478
732	Sturnus pagodarum	Brahminy Starling	2017/2/7	6	0	27.15925594	77.52319984	https://www.inaturalist.org/observations/109024851
733	Sturnus pagodarum	Brahminy Starling	2019/11/14	4	0	27.15926900	77.52319990	https://www.inaturalist.org/observations/35983125
734	Sturnus pagodarum	Brahminy Starling	2009/11/20	4	0	27.15926900	77.52319990	https://www.inaturalist.org/observations/36401135
735	Sturnus pagodarum	Brahminy Starling	2015/3/3	3	0	27.15926900	77.52319990	https://www.inaturalist.org/observations/43871461

736	Sturnus pagodarum	Brahminy Starling	2008/12/1	2	0	27.15926900	77.52319990	https://www.inaturalist.org/observations/55245702
737	Sturnus pagodarum	Brahminy Starling	2008/12/2	4	0	27.15926900	77.52319990	https://www.inaturalist.org/observations/59031734
738	Sturnus pagodarum	Brahminy Starling	2008/12/3	2	0	27.15926900	77.52319990	https://www.inaturalist.org/observations/59033271
739	Sturnus pagodarum	Brahminy Starling	2014/12/16	3	0	27.15926900	77.52319990	https://www.inaturalist.org/observations/69152004
740	Sturnus pagodarum	Brahminy Starling	2014/12/15	3	0	27.15926900	77.52319990	https://www.inaturalist.org/observations/69152042
741	Sturnus pagodarum	Brahminy Starling	2022/3/12	0	0	27.15926900	77.52319990	https://www.inaturalist.org/observations/109822518
742	Sturnus pagodarum	Brahminy Starling	2022/3/13	1	0	27.15926900	77.52319990	https://www.inaturalist.org/observations/111064388
743	Sturnus pagodarum	Brahminy Starling	2015/11/29	3	0	27.15926900	77.52319990	https://www.inaturalist.org/observations/120790973
744	Sturnus pagodarum	Brahminy Starling	2020/2/18	3	0	27.15926900	77.52319990	https://www.inaturalist.org/observations/122774011
745	Sturnus pagodarum	Brahminy Starling	2018/3/5	1	0	27.15926900	77.52319990	https://www.inaturalist.org/observations/133425324
746	Sturnus pagodarum	Brahminy Starling	2012/10/17	2	0	27.15926900	77.52319990	https://www.inaturalist.org/observations/139746113
747	Sturnus pagodarum	Brahminy Starling	2005/12/14	3	0	27.15936423	77.52296448	https://www.inaturalist.org/observations/54715052
748	Sturnus pagodarum	Brahminy Starling	2017/3/3	1	0	27.15936423	77.52313614	https://www.inaturalist.org/observations/105808008
749	Sturnus pagodarum	Brahminy Starling	2022/2/8	2	0	27.15943314	77.52319984	https://www.inaturalist.org/observations/106907796
750	Sturnus pagodarum	Brahminy Starling	2012/4/27	5	0	27.15960850	77.52181270	http://www.inaturalist.org/observations/105910
751	Sturnus pagodarum	Brahminy Starling	2020/1/18	2	0	27.16415376	77.52865363	https://www.inaturalist.org/observations/56724086
752	Sturnus pagodarum	Brahminy Starling	2016/8/28	5	0	27.16516797	77.52502441	https://www.inaturalist.org/observations/17872953
753	Sturnus pagodarum	Brahminy Starling	2020/3/17	3	0	27.16598500	77.52460000	https://www.inaturalist.org/observations/48646163
754	Sturnus pagodarum	Brahminy Starling	2006/1/10	4	0	27.16700000	77.51800000	https://www.inaturalist.org/observations/9772238
755	Sturnus pagodarum	Brahminy Starling	2011/12/11	4	0	27.16758710	77.51501676	https://www.inaturalist.org/observations/22011627
756	Sturnus pagodarum	Brahminy Starling	2014/11/24	2	0	27.16761123	77.51918830	https://www.inaturalist.org/observations/142941249
757	Sturnus pagodarum	Brahminy Starling	2016/12/28	4	0	27.16801944	78.04891389	https://www.inaturalist.org/observations/69059255
758	Sturnus pagodarum	Brahminy Starling	2017/11/17	3	0	27.16857623	77.51730025	https://www.inaturalist.org/observations/65745580

759	Sturnus pagodarum	Brahminy Starling	2018/8/10	3	0	27.17042133	78.03267310	https://www.inaturalist.org/observations/59385738
760	Sturnus pagodarum	Brahminy Starling	2017/11/27	2	0	27.17070000	78.04810000	https://www.inaturalist.org/observations/69972826
761	Sturnus pagodarum	Brahminy Starling	2021/11/9	2	0	27.17131900	78.04864600	https://www.inaturalist.org/observations/100676217
762	Sturnus pagodarum	Brahminy Starling	2018/11/24	4	0	27.17177000	77.52163900	https://www.inaturalist.org/observations/36473751
763	Sturnus pagodarum	Brahminy Starling	2020/2/24	4	0	27.17309500	77.52094667	https://www.inaturalist.org/observations/39505511
764	Sturnus pagodarum	Brahminy Starling	2017/3/1	4	0	27.17451447	78.04761008	https://www.inaturalist.org/observations/5383819
765	Sturnus pagodarum	Brahminy Starling	2022/5/18	1	0	27.17654579	83.49675827	https://www.inaturalist.org/observations/139892635
766	Sturnus pagodarum	Brahminy Starling	2007/12/17	5	0	27.17820707	77.50328159	https://www.inaturalist.org/observations/7427714
767	Sturnus pagodarum	Brahminy Starling	2016/10/19	5	0	27.17868593	77.51810542	https://www.inaturalist.org/observations/20027072
768	Sturnus pagodarum	Brahminy Starling	2020/2/15	3	0	27.17894370	77.51706000	https://www.inaturalist.org/observations/62542539
769	Sturnus pagodarum	Brahminy Starling	2019/12/30	7	0	27.18128855	77.50292832	https://www.inaturalist.org/observations/37098182
770	Sturnus pagodarum	Brahminy Starling	2002/1/17	6	0	27.18492539	77.50809370	https://www.inaturalist.org/observations/40258468
771	Sturnus pagodarum	Brahminy Starling	2011/11/9	3	0	27.18970932	77.51294391	https://www.inaturalist.org/observations/37304740
772	Sturnus pagodarum	Brahminy Starling	2017/3/2	5	0	27.19162257	77.51448260	https://www.inaturalist.org/observations/5384793
773	Sturnus pagodarum	Brahminy Starling	2012/2/7	5	0	27.19234170	77.51078688	https://www.inaturalist.org/observations/18317242
774	Sturnus pagodarum	Brahminy Starling	2011/2/22	5	0	27.19296000	77.50988900	https://www.inaturalist.org/observations/17691648
775	Sturnus pagodarum	Brahminy Starling	2013/12/27	3	0	27.19815205	77.50672057	https://www.inaturalist.org/observations/67045980
776	Sturnus pagodarum	Brahminy Starling	2020/2/24	2	0	27.20018667	77.50748333	https://www.inaturalist.org/observations/39251510
777	Sturnus pagodarum	Brahminy Starling	2002/1/15	2	0	27.20052946	77.50447389	https://www.inaturalist.org/observations/104685904
778	Sturnus pagodarum	Brahminy Starling	2017/2/14	5	0	27.20177167	77.50752000	https://www.inaturalist.org/observations/5235268
779	Sturnus pagodarum	Brahminy Starling	2017/2/14	5	0	27.20183167	77.50746333	https://www.inaturalist.org/observations/5235269
780	Sturnus pagodarum	Brahminy Starling	2022/9/4	3	0	27.21518172	77.50299945	https://www.inaturalist.org/observations/133725985
781	Sturnus pagodarum	Brahminy Starling	2014/2/3	9	0	27.21731000	77.49009000	http://www.inaturalist.org/observations/1464581

782	Sturnus pagodarum	Brahminy Starling	2019/1/18	5	0	27.39170820	76.37565390	https://www.inaturalist.org/observations/19775112
783	Sturnus pagodarum	Brahminy Starling	2022/3/15	5	0	27.46868062	70.22892673	https://www.inaturalist.org/observations/108646557
784	Sturnus pagodarum	Brahminy Starling	2021/4/26	5	0	27.54301375	75.13829794	https://www.inaturalist.org/observations/75281091
785	Sturnus pagodarum	Brahminy Starling	2017/10/2	1	0	27.79715585	74.43376755	https://www.inaturalist.org/observations/72170679
786	Sturnus pagodarum	Brahminy Starling	2015/4/4	5	0	28.04965600	76.12129600	http://www.inaturalist.org/observations/1387124
787	Sturnus pagodarum	Brahminy Starling	2017/6/16	3	0	28.28839768	79.40163899	https://www.inaturalist.org/observations/6689433
788	Sturnus pagodarum	Brahminy Starling	2021/6/26	2	0	28.35031350	77.06277145	https://www.inaturalist.org/observations/89445321
789	Sturnus pagodarum	Brahminy Starling	2016/10/21	5	0	28.46855700	77.29069500	http://www.inaturalist.org/observations/4407861
790	Sturnus pagodarum	Brahminy Starling	2017/1/5	4	0	28.46927400	77.28904500	https://www.inaturalist.org/observations/4941409
791	Sturnus pagodarum	Brahminy Starling	2022/6/1	4	0	28.52631616	77.16528694	https://www.inaturalist.org/observations/119691965
792	Sturnus pagodarum	Brahminy Starling	2020/7/14	3	0	28.53773869	77.24305786	https://www.inaturalist.org/observations/53073165
793	Sturnus pagodarum	Brahminy Starling	2020/5/18	2	0	28.54128131	77.14341162	https://www.inaturalist.org/observations/46587555
794	Sturnus pagodarum	Brahminy Starling	2020/5/18	4	0	28.54131791	77.14339928	https://www.inaturalist.org/observations/46587378
795	Sturnus pagodarum	Brahminy Starling	2009/7/16	3	0	28.55407065	77.26079903	https://www.inaturalist.org/observations/61556747
796	Sturnus pagodarum	Brahminy Starling	2020/4/20	1	0	28.55536467	77.20767632	https://www.inaturalist.org/observations/59667058
797	Sturnus pagodarum	Brahminy Starling	2009/7/15	3	0	28.55720980	77.21278910	https://www.inaturalist.org/observations/31583629
798	Sturnus pagodarum	Brahminy Starling	2011/9/1	4	0	28.55816600	77.19715000	http://www.inaturalist.org/observations/30026
799	Sturnus pagodarum	Brahminy Starling	2019/5/7	4	0	28.56373500	77.22919667	https://www.inaturalist.org/observations/24882382
800	Sturnus pagodarum	Brahminy Starling	2022/5/10	1	0	28.59813497	77.22208897	https://www.inaturalist.org/observations/116400758
801	Sturnus pagodarum	Brahminy Starling	2020/5/9	2	0	28.59888667	77.19317000	https://www.inaturalist.org/observations/45352061
802	Sturnus pagodarum	Brahminy Starling	2022/4/18	1	0	28.61705208	77.23802948	https://www.inaturalist.org/observations/111897366
803	Sturnus pagodarum	Brahminy Starling	2020/4/12	3	0	28.63625650	77.07911080	https://www.inaturalist.org/observations/48093175
804	Sturnus pagodarum	Brahminy Starling	2021/10/19	1	0	28.74947929	76.97080994	https://www.inaturalist.org/observations/100934277

805	Sturnus pagodarum	Brahminy Starling	2020/4/9	2	0	28.89597315	76.60661090	https://www.inaturalist.org/observations/44880134
806	Sturnus pagodarum	Brahminy Starling	2020/3/30	1	0	29.02286260	79.48794120	https://www.inaturalist.org/observations/48679208
807	Sturnus pagodarum	Brahminy Starling	2021/7/21	2	0	29.21803066	79.47446767	https://www.inaturalist.org/observations/88066042
808	Sturnus pagodarum	Brahminy Starling	2019/12/28	1	0	29.53001380	78.77466030	https://www.inaturalist.org/observations/45902796
809	Sturnus pagodarum	Brahminy Starling	2021/5/3	3	0	29.61269140	78.81177360	https://www.inaturalist.org/observations/79267802
810	Sturnus pagodarum	Brahminy Starling	2021/5/3	1	0	29.61269140	78.81177360	https://www.inaturalist.org/observations/79741486
811	Sturnus pagodarum	Brahminy Starling	2019/12/7	2	0	29.80588790	78.22610910	https://www.inaturalist.org/observations/91721318
812	Sturnus pagodarum	Brahminy Starling	2021/10/31	1	0	29.91538060	78.09690760	https://www.inaturalist.org/observations/99987341
813	Sturnus pagodarum	Brahminy Starling	2022/10/24	2	0	29.91538060	78.09690760	https://www.inaturalist.org/observations/140050330
814	Sturnus pagodarum	Brahminy Starling	2017/10/8	1	0	29.95232895	78.08444380	https://www.inaturalist.org/observations/67239295
815	Sturnus pagodarum	Brahminy Starling	2014/3/27	2	0	30.13018680	78.32305590	https://www.inaturalist.org/observations/66447190
816	Sturnus pagodarum	Brahminy Starling	2020/9/22	3	0	30.31582622	78.05774264	https://www.inaturalist.org/observations/63170313
817	Sturnus pagodarum	Brahminy Starling	2021/9/5	1	0	30.31770112	78.05934191	https://www.inaturalist.org/observations/93693585
818	Sturnus pagodarum	Brahminy Starling	2015/5/17	4	0	30.37330100	78.09976800	http://www.inaturalist.org/observations/1505634
819	Sturnus pagodarum	Brahminy Starling	2020/3/14	3	0	30.44826650	77.75495360	https://www.inaturalist.org/observations/122466872
820	Sturnus pagodarum	Brahminy Starling	2022/8/6	1	0	30.45425900	78.11612670	https://www.inaturalist.org/observations/129766624
821	Sturnus pagodarum	Brahminy Starling	2022/5/30	1	0	30.74768842	76.73369698	https://www.inaturalist.org/observations/119420704
822	Sturnus pagodarum	Brahminy Starling	2018/12/31	2	0	30.79931764	76.71473065	https://www.inaturalist.org/observations/135485827
823	Sturnus pagodarum	Brahminy Starling	2018/6/17	4	0	31.09426150	75.58879890	https://www.inaturalist.org/observations/13884976
824	Sturnus pagodarum	Brahminy Starling	2020/2/5	3	0	31.28849660	75.62032830	https://www.inaturalist.org/observations/38327279
825	Sturnus pagodarum	Brahminy Starling	2016/6/23	1	0	31.29735380	75.61993450	https://www.inaturalist.org/observations/72139960
826	Sturnus pagodarum	Brahminy Starling	2022/6/18	2	0	31.56283019	75.92019405	https://www.inaturalist.org/observations/122792465
827	Sturnus pagodarum	Brahminy Starling	2022/4/23	0	0	31.56289972	76.86260000	https://www.inaturalist.org/observations/112506927

828	Sturnus pagodarum	Brahminy Starling	2021/4/9	2	0	32.18494009	75.97162065	https://www.inaturalist.org/observations/74161746
829	Sturnus pagodarum	Brahminy Starling	2016/6/16	4	0	33.63001889	78.31598443	https://www.inaturalist.org/observations/10524029

No	scientific name	common name	date	NIA	NID	latitude	longitude	url
1	Zoothera dauma	Scaly Thrush	2021/4/9	0	0	20.34002560	79.30590060	https://www.inaturalist.org/observations/137389874
2	Zoothera dauma	Scaly Thrush	2020/11/14	0	0	21.63864667	87.57434333	https://www.inaturalist.org/observations/141870559
3	Zoothera dauma	Scaly Thrush	2019/10/30	1	0	21.92905200	101.25512300	https://www.inaturalist.org/observations/35069928
4	Zoothera dauma	Scaly Thrush	2017/11/22	1	0	22.16941035	80.60857377	https://www.inaturalist.org/observations/75612543
5	Zoothera dauma	Scaly Thrush	2020/12/27	2	0	22.68107330	88.31542690	https://www.inaturalist.org/observations/97994069
6	Zoothera dauma	Scaly Thrush	2020/11/15	3	0	22.80744803	88.33444928	https://www.inaturalist.org/observations/97994044
7	Zoothera dauma	Scaly Thrush	2020/11/15	0	0	22.82111100	88.32028660	https://www.inaturalist.org/observations/100463691
8	Zoothera dauma dauma	Scaly Thrush	2019/10/18	1	0	22.88960999	120.66336010	https://www.inaturalist.org/observations/90024637
9	Zoothera dauma	Scaly Thrush	2019/11/1	0	0	22.89845033	120.66470400	https://www.inaturalist.org/observations/89329264
10	Zoothera dauma dauma	Scaly Thrush	2020/1/15	1	0	23.67195297	120.79705760	https://www.inaturalist.org/observations/40705248
11	Zoothera dauma dauma	Scaly Thrush	2020/2/8	1	0	23.67210721	120.79768000	https://www.inaturalist.org/observations/99598611
12	Zoothera dauma	Scaly Thrush	2015/3/30	1	0	23.67226932	120.79663200	https://www.inaturalist.org/observations/14408752
13	Zoothera dauma	Scaly Thrush	2021/1/18	1	0	23.67451780	120.79725150	https://www.inaturalist.org/observations/98774250
14	Zoothera dauma	Scaly Thrush	2017/5/3	1	0	23.67451800	120.79725200	https://www.inaturalist.org/observations/137201689
15	Zoothera dauma dauma	Scaly Thrush	2015/1/11	2	0	23.67486277	120.79865200	https://www.inaturalist.org/observations/15249883
16	Zoothera dauma	Scaly Thrush	2018/3/18	1	0	23.70942042	80.96692001	https://www.inaturalist.org/observations/13472061
17	Zoothera dauma	Scaly Thrush	2020/2/7	0	0	23.71775833	121.39712500	https://www.inaturalist.org/observations/38383440
18	Zoothera dauma	Scaly Thrush	2021/12/1	0	0	23.92500833	121.53785000	https://www.inaturalist.org/observations/109915966
19	Zoothera dauma	Scaly Thrush	2015/12/27	2	0	24.07827454	88.69165298	https://www.inaturalist.org/observations/117576673
20	Zoothera dauma dauma	Scaly Thrush	2022/7/4	1	0	24.22895139	120.98249310	https://www.inaturalist.org/observations/124709913
21	Zoothera dauma dauma	Scaly Thrush	2020/2/27	0	0	24.35235870	121.30859720	https://www.inaturalist.org/observations/39560162
22	Zoothera dauma	Scaly Thrush	2021/1/4	2	0	24.43202000	92.33068900	https://www.inaturalist.org/observations/69311740

23	Zoothera dauma	Scaly Thrush	2011/12/7	1	0	24.43202000	92.33068900	https://www.inaturalist.org/observations/103677179
24	Zoothera dauma dauma	Scaly Thrush	2011/3/8	2	0	24.51007938	121.61211230	https://www.inaturalist.org/observations/55775025
25	Zoothera dauma dauma	Scaly Thrush	2004/4/18	1	0	24.58809400	121.43420360	https://www.inaturalist.org/observations/16280096
26	Zoothera dauma dauma	Scaly Thrush	2020/2/9	1	0	24.75641799	121.59463280	https://www.inaturalist.org/observations/60854868
27	Zoothera dauma	Scaly Thrush	2018/5/18	1	0	25.65234300	94.02299200	https://www.inaturalist.org/observations/14281227
28	Zoothera dauma	Scaly Thrush	2022/1/24	3	0	26.85552014	75.84058440	https://www.inaturalist.org/observations/106661401
29	Zoothera dauma	Scaly Thrush	2012/1/7	0	0	26.93240100	92.87721600	https://www.inaturalist.org/observations/103804784
30	Zoothera dauma	Scaly Thrush	2014/1/15	4	0	26.93248900	92.87766400	http://www.inaturalist.org/observations/548834
31	Zoothera dauma	Scaly Thrush	2014/3/20	2	0	26.94035937	92.85798903	https://www.inaturalist.org/observations/7427098
32	Zoothera dauma	Scaly Thrush	2022/1/1	1	0	26.94543922	94.16107312	https://www.inaturalist.org/observations/104106880
33	Zoothera dauma	Scaly Thrush	2021/4/20	1	0	27.08326379	88.70029541	https://www.inaturalist.org/observations/97787062
34	Zoothera dauma	Scaly Thrush	2021/2/9	0	0	27.45433499	94.90310859	https://www.inaturalist.org/observations/93234676
35	Zoothera dauma	Scaly Thrush	2021/4/5	1	0	29.34430900	79.52943180	https://www.inaturalist.org/observations/101930393
36	Zoothera dauma	Scaly Thrush	2016/1/22	2	0	29.34430900	79.52943180	https://www.inaturalist.org/observations/127084473
37	Zoothera dauma	Scaly Thrush	2006/1/24	3	0	29.34730000	79.52920000	https://www.inaturalist.org/observations/104735051
38	Zoothera dauma	Scaly Thrush	2017/3/15	3	0	29.36891903	79.57329595	https://www.inaturalist.org/observations/109180484
39	Zoothera dauma	Scaly Thrush	2022/4/21	4	0	29.40366320	79.55927610	https://www.inaturalist.org/observations/113278401
40	Zoothera dauma	Scaly Thrush	2016/12/4	1	0	29.97111100	78.20360300	https://www.inaturalist.org/observations/19578120
41	Zoothera dauma	Scaly Thrush	2019/3/15	2	0	29.99089574	78.29011934	https://www.inaturalist.org/observations/87138102

No	scientific name	common name	date	NIA	NID	latitude	longitude	url
1	Corvus corone	Carrion Crow	2022/4/13	0	0	22.33959889	114.18926070	https://www.inaturalist.org/observations/114768353
2	Corvus corone	Carrion Crow	2022/3/16	1	0	22.47581188	114.03583120	https://www.inaturalist.org/observations/115135427
3	Corvus corone	Carrion Crow	2022/3/3	1	0	22.48713213	114.03205380	https://www.inaturalist.org/observations/118537826
4	Corvus corone	Carrion Crow	2021/11/25	1	0	22.57641306	114.08196800	https://www.inaturalist.org/observations/102084677
5	Corvus corone	Carrion Crow	2021/11/10	0	0	23.62802667	120.71205320	https://www.inaturalist.org/observations/100741548
6	Corvus corone	Carrion Crow	2022/5/8	0	0	24.57724614	118.07133800	https://www.inaturalist.org/observations/116055138
7	Corvus corone	Carrion Crow	2018/11/19	1	0	25.11146665	121.47095580	https://www.inaturalist.org/observations/18493721
8	Corvus corone orientalis	Carrion Crow	2019/3/10	2	0	25.11403259	121.49048780	https://www.inaturalist.org/observations/21144373
9	Corvus corone	Carrion Crow	2018/11/20	1	0	25.11576333	121.47145830	https://www.inaturalist.org/observations/18730617
10	Corvus corone	Carrion Crow	2022/1/22	0	0	26.84449356	104.24263370	https://www.inaturalist.org/observations/105712704
11	Corvus corone	Carrion Crow	2022/1/23	0	0	26.84449356	104.24263370	https://www.inaturalist.org/observations/105716513
12	Corvus corone	Carrion Crow	2022/1/23	1	0	26.84449356	104.24263370	https://www.inaturalist.org/observations/105758079
13	Corvus corone	Carrion Crow	2022/1/25	1	0	26.84449356	104.24263370	https://www.inaturalist.org/observations/107757275
14	Corvus corone	Carrion Crow	2022/1/25	1	0	26.84449356	104.24263370	https://www.inaturalist.org/observations/107757279
15	Corvus corone	Carrion Crow	2022/1/25	1	0	26.86281421	104.19353500	https://www.inaturalist.org/observations/107757288
16	Corvus corone	Carrion Crow	2022/1/23	1	0	26.86900759	104.15914740	https://www.inaturalist.org/observations/105758009
17	Corvus corone	Carrion Crow	2022/1/23	0	0	26.86900759	104.15914740	https://www.inaturalist.org/observations/105758020
18	Corvus corone	Carrion Crow	2022/1/23	0	0	26.89363246	104.20629510	https://www.inaturalist.org/observations/105757998
19	Corvus corone	Carrion Crow	2022/1/23	0	0	26.89363246	104.20629510	https://www.inaturalist.org/observations/105758122
20	Corvus corone	Carrion Crow	2020/8/13	1	0	27.48112378	95.58913497	https://www.inaturalist.org/observations/56377981
21	Corvus corone orientalis	Carrion Crow	2022/12/10	0	0	30.23864740	119.75016590	https://www.inaturalist.org/observations/144144878
22	Corvus corone	Carrion Crow	2021/10/23	0	0	32.20363101	119.01338020	https://www.inaturalist.org/observations/120273566

23	Corvus corone	Carrion Crow	2013/12/2	1	0	33.63001889	78.31598443	https://www.inaturalist.org/observations/10524035
24	Corvus corone	Carrion Crow	2019/6/26	0	0	34.05699393	77.64361890	https://www.inaturalist.org/observations/104204430
25	Corvus corone	Carrion Crow	2019/6/26	0	0	34.08592744	77.60142859	https://www.inaturalist.org/observations/104204421
26	Corvus corone	Carrion Crow	2020/12/11	1	0	34.76016235	113.65845490	https://www.inaturalist.org/observations/66412787
27	Corvus corone	Carrion Crow	2020/12/25	1	0	34.76357833	113.66932330	https://www.inaturalist.org/observations/67039575
28	Corvus corone	Carrion Crow	2021/12/17	1	0	34.76360667	113.66957000	https://www.inaturalist.org/observations/103193767
29	Corvus corone	Carrion Crow	2021/12/17	1	0	34.76361667	113.66944330	https://www.inaturalist.org/observations/103193768
30	Corvus corone	Carrion Crow	2021/12/17	0	0	34.76365500	113.66898330	https://www.inaturalist.org/observations/103193770
31	Corvus corone	Carrion Crow	2021/1/13	1	0	34.81084167	113.71343830	https://www.inaturalist.org/observations/68029887
32	Corvus corone	Carrion Crow	2021/1/10	0	0	34.81501333	113.70922830	https://www.inaturalist.org/observations/67841450
33	Corvus corone	Carrion Crow	2021/12/3	1	0	34.81984667	113.70502670	https://www.inaturalist.org/observations/102527238
34	Corvus corone	Carrion Crow	2021/3/8	1	0	34.82228333	113.71604170	https://www.inaturalist.org/observations/70811129
35	Corvus corone	Carrion Crow	2021/2/7	0	0	39.57724641	117.28704010	https://www.inaturalist.org/observations/142867116
36	Corvus corone	Carrion Crow	2022/11/17	1	0	39.73345924	116.02237780	https://www.inaturalist.org/observations/142218289
37	Corvus corone	Carrion Crow	2018/5/20	1	0	39.88152373	116.40669140	https://www.inaturalist.org/observations/104980206
38	Corvus corone	Carrion Crow	2022/2/19	1	0	39.94144568	116.33714370	https://www.inaturalist.org/observations/125245088
39	Corvus corone	Carrion Crow	2021/12/19	2	0	39.99966740	116.32644390	https://www.inaturalist.org/observations/144057175
40	Corvus corone	Carrion Crow	2021/2/7	0	0	40.00313439	116.33068750	https://www.inaturalist.org/observations/69482932
41	Corvus corone	Carrion Crow	2021/2/17	1	0	40.13304615	116.31133980	https://www.inaturalist.org/observations/122030264
42	Corvus corone	Carrion Crow	2019/2/8	2	0	40.13791500	116.30024500	https://www.inaturalist.org/observations/20201445
43	Corvus corone	Carrion Crow	2022/7/8	0	0	43.62929811	126.18407450	https://www.inaturalist.org/observations/128546351

No	scientific name	common name	date	NIA	NID	latitude	longitude	url
1	Emberiza rustica	Rustic Bunting	2021/11/25	1	0	22.24893438	113.83398930	https://www.inaturalist.org/observations/101873556
2	Emberiza rustica	Rustic Bunting	2022/12/6	4	0	22.43561851	114.16662610	https://www.inaturalist.org/observations/143777807
3	Emberiza rustica	Rustic Bunting	2019/11/27	2	0	22.43962292	114.13033840	https://www.inaturalist.org/observations/36126509
4	Emberiza rustica	Rustic Bunting	2020/11/29	1	0	22.45100631	114.19481980	https://www.inaturalist.org/observations/69293527
5	Emberiza rustica	Rustic Bunting	2021/11/16	1	0	22.45698562	114.14424200	https://www.inaturalist.org/observations/118537725
6	Emberiza rustica	Rustic Bunting	2019/11/10	1	0	22.48119162	114.16244750	https://www.inaturalist.org/observations/49838896
7	Emberiza rustica	Rustic Bunting	2021/11/23	1	0	22.49422885	114.03774380	https://www.inaturalist.org/observations/115141212
8	Emberiza rustica	Rustic Bunting	2019/11/20	3	0	22.50303437	114.08788210	https://www.inaturalist.org/observations/35880957
9	Emberiza rustica	Rustic Bunting	2020/11/19	1	0	22.52557277	114.05825530	https://www.inaturalist.org/observations/65223149
10	Emberiza rustica	Rustic Bunting	2020/11/14	1	0	22.53125789	114.16877760	https://www.inaturalist.org/observations/65148415
11	Emberiza rustica	Rustic Bunting	2021/11/21	3	0	22.53969150	114.04483450	https://www.inaturalist.org/observations/101701649
12	Emberiza rustica	Rustic Bunting	2022/2/13	1	0	22.54311806	114.21139800	https://www.inaturalist.org/observations/118537812
13	Emberiza rustica	Rustic Bunting	2020/11/14	2	0	22.55346383	114.05861720	https://www.inaturalist.org/observations/64848462
14	Emberiza rustica	Rustic Bunting	2021/11/26	1	0	22.56364055	114.12162540	https://www.inaturalist.org/observations/101948854
15	Emberiza rustica	Rustic Bunting	2020/12/8	1	0	22.56419333	114.10907670	https://www.inaturalist.org/observations/70608829
16	Emberiza rustica	Rustic Bunting	2021/11/29	2	0	22.57981286	114.15711310	https://www.inaturalist.org/observations/102171244
17	Emberiza rustica	Rustic Bunting	2021/11/13	1	0	22.58300354	114.10753920	https://www.inaturalist.org/observations/118537719
18	Emberiza rustica	Rustic Bunting	2018/11/25	2	0	22.59467072	114.00421880	https://www.inaturalist.org/observations/20970731
19	Emberiza rustica	Rustic Bunting	2022/12/2	1	0	22.59669824	114.12830620	https://www.inaturalist.org/observations/143473333
20	Emberiza rustica	Rustic Bunting	2021/12/3	2	0	23.89535679	121.59353950	https://www.inaturalist.org/observations/103079123
21	Emberiza rustica	Rustic Bunting	2021/12/17	2	0	23.95405760	121.57972210	https://www.inaturalist.org/observations/103416924
22	Emberiza rustica	Rustic Bunting	2021/12/3	1	0	23.96057053	121.54915480	https://www.inaturalist.org/observations/103079124

23	Emberiza rustica	Rustic Bunting	2021/12/3	2	0	23.98402975	121.40670510	https://www.inaturalist.org/observations/103079125
24	Emberiza rustica	Rustic Bunting	2017/11/2	1	0	25.06898776	121.19893420	https://www.inaturalist.org/observations/50201466
25	Emberiza rustica	Rustic Bunting	2022/12/26	1	0	25.08668527	121.87151700	https://www.inaturalist.org/observations/145104506
26	Emberiza rustica	Rustic Bunting	2019/4/18	2	0	25.10208420	121.84127780	https://www.inaturalist.org/observations/98798755
27	Emberiza rustica	Rustic Bunting	2019/12/14	2	0	28.62209091	116.04967290	https://www.inaturalist.org/observations/36730236
28	Emberiza rustica	Rustic Bunting	2022/12/8	1	0	29.03413842	116.15885400	https://www.inaturalist.org/observations/144411681
29	Emberiza rustica	Rustic Bunting	2022/12/8	1	0	29.16312959	116.07781100	https://www.inaturalist.org/observations/144411682
30	Emberiza rustica	Rustic Bunting	2021/11/19	1	0	29.69745392	121.41095270	https://www.inaturalist.org/observations/101608155
31	Emberiza rustica	Rustic Bunting	2022/2/28	1	0	29.80196253	120.99164560	https://www.inaturalist.org/observations/108161410
32	Emberiza rustica	Rustic Bunting	2022/2/28	1	0	29.87134419	120.96567530	https://www.inaturalist.org/observations/107693688
33	Emberiza rustica	Rustic Bunting	2022/2/28	1	0	30.06516841	120.88677420	https://www.inaturalist.org/observations/107693660
34	Emberiza rustica	Rustic Bunting	2009/11/6	2	0	30.22091006	121.11751770	https://www.inaturalist.org/observations/38630717
35	Emberiza rustica	Rustic Bunting	2022/1/17	1	0	30.22586642	119.81570300	https://www.inaturalist.org/observations/105207194
36	Emberiza rustica	Rustic Bunting	2021/12/6	1	0	30.22750061	119.91754730	https://www.inaturalist.org/observations/102643311
37	Emberiza rustica	Rustic Bunting	2021/12/18	1	0	30.22921826	119.92635530	https://www.inaturalist.org/observations/103516928
38	Emberiza rustica	Rustic Bunting	2022/1/17	1	0	30.23756161	119.88176850	https://www.inaturalist.org/observations/105207193
39	Emberiza rustica	Rustic Bunting	2021/12/15	1	0	30.24247150	119.86056030	https://www.inaturalist.org/observations/103185409
40	Emberiza rustica	Rustic Bunting	2022/2/5	1	0	30.25512650	119.80739300	https://www.inaturalist.org/observations/106368213
41	Emberiza rustica	Rustic Bunting	2022/12/25	1	0	30.27275375	119.91515270	https://www.inaturalist.org/observations/145199396
42	Emberiza rustica	Rustic Bunting	2022/2/14	1	0	30.27874971	119.80879900	https://www.inaturalist.org/observations/107364053
43	Emberiza rustica	Rustic Bunting	2022/12/25	1	0	30.27898113	120.16772610	https://www.inaturalist.org/observations/145200082
44	Emberiza rustica	Rustic Bunting	2021/12/15	1	0	30.28982228	119.95805470	https://www.inaturalist.org/observations/103185408
45	Emberiza rustica	Rustic Bunting	2021/12/6	1	0	30.29569142	119.84608440	https://www.inaturalist.org/observations/102691272

46	Emberiza rustica	Rustic Bunting	2022/11/1	1	0	30.30124407	120.13177070	https://www.inaturalist.org/observations/141382128
47	Emberiza rustica	Rustic Bunting	2021/12/27	2	0	30.32448010	119.85750250	https://www.inaturalist.org/observations/103797441
48	Emberiza rustica	Rustic Bunting	2021/12/15	1	0	30.32881614	119.87825060	https://www.inaturalist.org/observations/103185406
49	Emberiza rustica	Rustic Bunting	2021/12/15	1	0	30.32977169	119.88114440	https://www.inaturalist.org/observations/103185410
50	Emberiza rustica	Rustic Bunting	2021/12/6	1	0	30.33393964	119.85685100	https://www.inaturalist.org/observations/102643309
51	Emberiza rustica	Rustic Bunting	2021/1/7	2	0	30.34828723	119.96748630	https://www.inaturalist.org/observations/143821771
52	Emberiza rustica	Rustic Bunting	2021/12/18	1	0	30.36246987	119.97428960	https://www.inaturalist.org/observations/103516328
53	Emberiza rustica	Rustic Bunting	2022/1/17	1	0	30.37040115	119.87488700	https://www.inaturalist.org/observations/106849320
54	Emberiza rustica	Rustic Bunting	2022/1/17	1	0	30.37823485	119.81734200	https://www.inaturalist.org/observations/105213234
55	Emberiza rustica	Rustic Bunting	2021/12/27	1	0	30.38014700	119.98457220	https://www.inaturalist.org/observations/103797437
56	Emberiza rustica	Rustic Bunting	2022/12/3	2	0	30.38979096	119.83463170	https://www.inaturalist.org/observations/143997018
57	Emberiza rustica	Rustic Bunting	2021/12/15	1	0	30.39475355	119.84277280	https://www.inaturalist.org/observations/103185404
58	Emberiza rustica	Rustic Bunting	2019/12/22	1	0	30.68035473	114.16445320	https://www.inaturalist.org/observations/109638778
59	Emberiza rustica	Rustic Bunting	2018/1/20	1	0	30.98096460	121.89577630	https://www.inaturalist.org/observations/9597444
60	Emberiza rustica	Rustic Bunting	2021/11/26	1	0	30.98922046	121.80978670	https://www.inaturalist.org/observations/102001205
61	Emberiza rustica	Rustic Bunting	2020/2/29	2	0	31.02865621	117.08116440	https://www.inaturalist.org/observations/39348791
62	Emberiza rustica	Rustic Bunting	2021/1/12	1	0	31.43391688	120.42805150	https://www.inaturalist.org/observations/67947916
63	Emberiza rustica	Rustic Bunting	2020/11/13	1	0	31.55830589	119.52041310	https://www.inaturalist.org/observations/69653534
64	Emberiza rustica	Rustic Bunting	2022/1/16	1	0	31.83149443	118.73265560	https://www.inaturalist.org/observations/120274289
65	Emberiza rustica	Rustic Bunting	2022/2/19	2	0	31.98829776	118.71675840	https://www.inaturalist.org/observations/112548804
66	Emberiza rustica	Rustic Bunting	2022/2/19	2	0	32.06775501	118.40081390	https://www.inaturalist.org/observations/112548404
67	Emberiza rustica	Rustic Bunting	2022/2/2	2	0	32.26909422	119.15384350	https://www.inaturalist.org/observations/112935693
68	Emberiza rustica	Rustic Bunting	2021/1/3	1	0	32.35382147	119.18477160	https://www.inaturalist.org/observations/88710623

69	Emberiza rustica	Rustic Bunting	2021/2/16	1	0	32.41572914	119.45830850	https://www.inaturalist.org/observations/142645649
70	Emberiza rustica	Rustic Bunting	2019/1/6	2	0	32.46609872	121.04112000	https://www.inaturalist.org/observations/34010399
71	Emberiza rustica	Rustic Bunting	2017/12/2	1	0	33.47206701	120.45108470	https://www.inaturalist.org/observations/10232408
72	Emberiza rustica	Rustic Bunting	2022/2/26	1	0	34.40075738	119.37009450	https://www.inaturalist.org/observations/123573386
73	Emberiza rustica	Rustic Bunting	2022/2/20	1	0	34.81672794	113.73792890	https://www.inaturalist.org/observations/107090862
74	Emberiza rustica	Rustic Bunting	2021/1/10	2	0	34.87432871	113.60887650	https://www.inaturalist.org/observations/67841402
75	Emberiza rustica	Rustic Bunting	2022/2/20	1	0	34.92904251	113.66440220	https://www.inaturalist.org/observations/107090863
76	Emberiza rustica	Rustic Bunting	2020/11/1	1	0	36.21625635	120.63218270	https://www.inaturalist.org/observations/105315430
77	Emberiza rustica	Rustic Bunting	2021/11/16	2	0	36.39556897	120.33012700	https://www.inaturalist.org/observations/103684205
78	Emberiza rustica	Rustic Bunting	2021/2/26	1	0	39.12181143	117.00610730	https://www.inaturalist.org/observations/142867365
79	Emberiza rustica	Rustic Bunting	2021/2/7	1	0	39.51820006	118.03380250	https://www.inaturalist.org/observations/78557330
80	Emberiza rustica	Rustic Bunting	2021/2/21	1	0	39.85170267	116.38153770	https://www.inaturalist.org/observations/121418599
81	Emberiza rustica	Rustic Bunting	2021/1/3	1	0	40.00314305	116.74687200	https://www.inaturalist.org/observations/67959319
82	Emberiza rustica	Rustic Bunting	2022/12/4	1	0	40.02359303	116.77019800	https://www.inaturalist.org/observations/143672701
83	Emberiza rustica	Rustic Bunting	2019/2/5	1	0	40.04997745	116.78280110	https://www.inaturalist.org/observations/20121205
84	Emberiza rustica	Rustic Bunting	2020/2/19	2	0	40.05726532	116.78294740	https://www.inaturalist.org/observations/38832568
85	Emberiza rustica	Rustic Bunting	2018/12/31	2	0	40.07405730	116.86239340	https://www.inaturalist.org/observations/19332654
86	Emberiza rustica	Rustic Bunting	2021/3/7	2	0	40.09758767	116.76317700	https://www.inaturalist.org/observations/70879522
87	Emberiza rustica	Rustic Bunting	2022/10/30	1	0	40.16940623	116.75216280	https://www.inaturalist.org/observations/140526911
88	Emberiza rustica	Rustic Bunting	2022/1/15	1	0	40.17037687	116.72929260	https://www.inaturalist.org/observations/105907339
89	Emberiza rustica	Rustic Bunting	2020/2/18	0	0	40.17408300	116.61744740	https://www.inaturalist.org/observations/38792387
90	Emberiza rustica	Rustic Bunting	2021/12/4	1	0	40.38778629	115.95554670	https://www.inaturalist.org/observations/125272427
91	Emberiza rustica	Rustic Bunting	2022/2/5	1	0	40.58324366	117.19322520	https://www.inaturalist.org/observations/125106566

92	Emberiza rustica	Rustic Bunting	2005/3/31	2	0	40.74748567	125.54014090	https://www.inaturalist.org/observations/66610922
93	Emberiza rustica	Rustic Bunting	2005/3/31	2	0	40.77244964	125.45468820	https://www.inaturalist.org/observations/66610923
94	Emberiza rustica	Rustic Bunting	2017/3/26	1	0	42.77044872	123.39467840	https://www.inaturalist.org/observations/66884318
95	Emberiza rustica	Rustic Bunting	2017/3/27	2	0	42.77861513	123.39870480	https://www.inaturalist.org/observations/66884565
96	Emberiza rustica	Rustic Bunting	2012/11/18	1	0	43.85099277	126.43259210	https://www.inaturalist.org/observations/69409635
97	Emberiza rustica	Rustic Bunting	2022/4/1	2	0	49.28175457	129.65959600	https://www.inaturalist.org/observations/110074823

No	scientific name	common name	date	NIA	NID	latitude	longitude	url
1	Emberiza spodocephala	Black-faced Bunting	2021/1/11	1	0	21.69330820	111.76701700	https://www.inaturalist.org/observations/67958988
2	Emberiza spodocephala	Black-faced Bunting	2021/2/14	1	0	22.11255174	113.57003870	https://www.inaturalist.org/observations/88584092
3	Emberiza spodocephala	Black-faced Bunting	2021/12/12	1	0	22.12321530	113.56448680	https://www.inaturalist.org/observations/102915202
4	Emberiza spodocephala	Black-faced Bunting	2021/3/8	0	0	22.12552167	113.55976830	https://www.inaturalist.org/observations/70813367
5	Emberiza spodocephala	Black-faced Bunting	2021/3/30	1	0	22.12583188	113.55993160	https://www.inaturalist.org/observations/99561135
6	Emberiza spodocephala	Black-faced Bunting	2021/11/20	1	0	22.12602647	113.57483450	https://www.inaturalist.org/observations/101520264
7	Emberiza spodocephala	Black-faced Bunting	2021/4/10	1	0	22.15002763	114.33092760	https://www.inaturalist.org/observations/75853246
8	Emberiza spodocephala	Black-faced Bunting	2021/3/1	1	0	22.15242688	113.55987130	https://www.inaturalist.org/observations/92373915
9	Emberiza spodocephala	Black-faced Bunting	2021/4/24	2	0	22.16439223	114.25120640	https://www.inaturalist.org/observations/92349294
10	Emberiza spodocephala	Black-faced Bunting	2021/12/2	0	0	22.16514256	114.25469200	https://www.inaturalist.org/observations/108552291
11	Emberiza spodocephala	Black-faced Bunting	2021/4/20	2	0	22.16521816	114.25422570	https://www.inaturalist.org/observations/74690107
12	Emberiza spodocephala	Black-faced Bunting	2022/4/30	2	0	22.16522500	114.25263500	https://www.inaturalist.org/observations/114144838
13	Emberiza spodocephala	Black-faced Bunting	2009/12/25	1	0	22.16535700	114.25514300	https://www.inaturalist.org/observations/36901446
14	Emberiza spodocephala	Black-faced Bunting	2009/12/25	1	0	22.16539700	114.25524000	https://www.inaturalist.org/observations/36901536
15	Emberiza spodocephala	Black-faced Bunting	2022/4/30	1	0	22.16573067	114.25641470	https://www.inaturalist.org/observations/115522447
16	Emberiza spodocephala	Black-faced Bunting	2021/11/28	1	0	22.19599529	114.13798100	https://www.inaturalist.org/observations/102273561
17	Emberiza spodocephala	Black-faced Bunting	2022/2/12	1	0	22.20034479	113.54803200	https://www.inaturalist.org/observations/106591272
18	Emberiza spodocephala	Black-faced Bunting	2021/1/13	1	0	22.20046616	113.54779010	https://www.inaturalist.org/observations/67997214
19	Emberiza spodocephala	Black-faced Bunting	2021/1/22	1	0	22.20052821	113.54794080	https://www.inaturalist.org/observations/68442042
20	Emberiza spodocephala	Black-faced Bunting	2022/2/8	0	0	22.20054724	113.54801080	https://www.inaturalist.org/observations/106379497
21	Emberiza spodocephala	Black-faced Bunting	2021/11/19	0	0	22.20062072	113.54770900	https://www.inaturalist.org/observations/101455438
22	Emberiza spodocephala	Black-faced Bunting	2022/1/27	1	0	22.20062430	113.54785370	https://www.inaturalist.org/observations/105651752

23	Emberiza spodocephala	Black-faced Bunting	2021/11/23	1	0	22.21411297	114.12833900	https://www.inaturalist.org/observations/101752403
24	Emberiza spodocephala	Black-faced Bunting	2022/3/18	1	0	22.22147985	114.11543740	https://www.inaturalist.org/observations/108913802
25	Emberiza spodocephala	Black-faced Bunting	2020/1/4	1	0	22.22749210	113.99024770	https://www.inaturalist.org/observations/37300752
26	Emberiza spodocephala	Black-faced Bunting	2022/2/11	3	0	22.24313370	114.19572100	https://www.inaturalist.org/observations/118130550
27	Emberiza spodocephala	Black-faced Bunting	2020/2/8	0	0	22.24670695	113.91811010	https://www.inaturalist.org/observations/38662998
28	Emberiza spodocephala	Black-faced Bunting	2020/2/11	1	0	22.25529801	114.17167940	https://www.inaturalist.org/observations/38537451
29	Emberiza spodocephala	Black-faced Bunting	2022/3/12	2	0	22.25757169	114.16153540	https://www.inaturalist.org/observations/108422600
30	Emberiza spodocephala	Black-faced Bunting	2019/11/9	1	0	22.27267587	114.14755230	https://www.inaturalist.org/observations/35471624
31	Emberiza spodocephala	Black-faced Bunting	2022/3/1	1	0	22.27370900	114.21022600	https://www.inaturalist.org/observations/107683022
32	Emberiza spodocephala	Black-faced Bunting	2020/3/28	1	0	22.27430380	114.14366040	https://www.inaturalist.org/observations/40870161
33	Emberiza spodocephala	Black-faced Bunting	2019/11/9	1	0	22.27486475	114.14417670	https://www.inaturalist.org/observations/35486429
34	Emberiza spodocephala	Black-faced Bunting	2021/11/14	0	0	22.27607133	114.12159920	https://www.inaturalist.org/observations/101078876
35	Emberiza spodocephala	Black-faced Bunting	2013/4/7	1	0	22.27831510	114.17469500	http://www.inaturalist.org/observations/3945736
36	Emberiza spodocephala	Black-faced Bunting	2021/3/21	1	0	22.28750434	114.00527780	https://www.inaturalist.org/observations/71749132
37	Emberiza spodocephala	Black-faced Bunting	2021/3/28	0	0	22.28750434	114.00527780	https://www.inaturalist.org/observations/72356474
38	Emberiza spodocephala	Black-faced Bunting	2020/3/18	1	0	22.28754342	114.00805530	https://www.inaturalist.org/observations/40177639
39	Emberiza spodocephala	Black-faced Bunting	2019/3/9	1	0	22.31920110	114.16961210	https://www.inaturalist.org/observations/21077514
40	Emberiza spodocephala	Black-faced Bunting	2022/2/15	2	0	22.34717996	114.23457380	https://www.inaturalist.org/observations/106786625
41	Emberiza spodocephala	Black-faced Bunting	2022/1/31	1	0	22.35125266	114.23890870	https://www.inaturalist.org/observations/105961899
42	Emberiza spodocephala	Black-faced Bunting	2022/1/22	1	0	22.35127361	114.23892130	https://www.inaturalist.org/observations/105557971
43	Emberiza spodocephala	Black-faced Bunting	2022/2/12	1	0	22.35211261	114.24141670	https://www.inaturalist.org/observations/106683358
44	Emberiza spodocephala	Black-faced Bunting	2022/2/10	1	0	22.35306282	114.09658190	https://www.inaturalist.org/observations/106791765
45	Emberiza spodocephala	Black-faced Bunting	2020/2/22	1	0	22.37775102	114.09291000	https://www.inaturalist.org/observations/101690510

46	Emberiza spodocephala	Black-faced Bunting	2021/2/19	2	0	22.38162500	114.22233900	https://www.inaturalist.org/observations/104332973
47	Emberiza spodocephala	Black-faced Bunting	2018/12/16	3	0	22.39093100	114.23404400	https://www.inaturalist.org/observations/19063497
48	Emberiza spodocephala	Black-faced Bunting	2020/1/10	1	0	22.39200271	114.23383980	https://www.inaturalist.org/observations/37451714
49	Emberiza spodocephala	Black-faced Bunting	2019/11/25	1	0	22.40673188	114.07116900	https://www.inaturalist.org/observations/36173898
50	Emberiza spodocephala	Black-faced Bunting	2020/12/21	1	0	22.40696207	113.95337870	https://www.inaturalist.org/observations/70609434
51	Emberiza spodocephala	Black-faced Bunting	2021/11/25	1	0	22.40746970	113.95228980	https://www.inaturalist.org/observations/101882236
52	Emberiza spodocephala	Black-faced Bunting	2022/2/16	1	0	22.40746971	113.95104520	https://www.inaturalist.org/observations/106953675
53	Emberiza spodocephala	Black-faced Bunting	2021/12/25	1	0	22.40760856	113.95208590	https://www.inaturalist.org/observations/103662756
54	Emberiza spodocephala	Black-faced Bunting	2019/1/8	2	0	22.40853554	114.07316390	https://www.inaturalist.org/observations/19509993
55	Emberiza spodocephala	Black-faced Bunting	2021/12/21	1	0	22.40874630	114.11112350	https://www.inaturalist.org/observations/103405508
56	Emberiza spodocephala	Black-faced Bunting	2020/2/10	0	0	22.40884640	114.13794990	https://www.inaturalist.org/observations/38624616
57	Emberiza spodocephala	Black-faced Bunting	2018/2/15	1	0	22.40884640	114.13794990	https://www.inaturalist.org/observations/58928480
58	Emberiza spodocephala	Black-faced Bunting	2018/2/15	1	0	22.40884640	114.13794990	https://www.inaturalist.org/observations/58928481
59	Emberiza spodocephala	Black-faced Bunting	2018/11/2	1	0	22.41839716	114.08317890	https://www.inaturalist.org/observations/18087779
60	Emberiza spodocephala	Black-faced Bunting	2022/2/2	2	0	22.41877651	114.04568800	https://www.inaturalist.org/observations/106115528
61	Emberiza spodocephala	Black-faced Bunting	2018/11/2	1	0	22.41881992	114.08242620	https://www.inaturalist.org/observations/18087629
62	Emberiza spodocephala	Black-faced Bunting	2022/4/24	1	0	22.42135060	114.14197220	https://www.inaturalist.org/observations/112930955
63	Emberiza spodocephala	Black-faced Bunting	2019/12/18	1	0	22.42172204	113.92153930	https://www.inaturalist.org/observations/37116602
64	Emberiza spodocephala	Black-faced Bunting	2022/2/27	2	0	22.42211833	114.08748210	https://www.inaturalist.org/observations/107564127
65	Emberiza spodocephala	Black-faced Bunting	2018/11/13	1	0	22.42330152	114.08222970	https://www.inaturalist.org/observations/18423051
66	Emberiza spodocephala	Black-faced Bunting	2013/1/26	1	0	22.42709800	114.15947400	https://www.inaturalist.org/observations/29955344
67	Emberiza spodocephala	Black-faced Bunting	2021/3/20	1	0	22.43134804	114.09112960	https://www.inaturalist.org/observations/71685900
68	Emberiza spodocephala	Black-faced Bunting	2021/1/11	2	0	22.43250167	114.08892330	https://www.inaturalist.org/observations/69042285

69	Emberiza spodocephala	Black-faced Bunting	2022/1/31	1	0	22.43266475	114.08245720	https://www.inaturalist.org/observations/105901157
70	Emberiza spodocephala	Black-faced Bunting	2022/1/31	2	0	22.43268670	114.08392090	https://www.inaturalist.org/observations/105954696
71	Emberiza spodocephala	Black-faced Bunting	2021/1/19	1	0	22.43403360	114.01718720	https://www.inaturalist.org/observations/68404074
72	Emberiza spodocephala	Black-faced Bunting	2021/1/10	1	0	22.43485670	114.08495820	https://www.inaturalist.org/observations/67957005
73	Emberiza spodocephala	Black-faced Bunting	2022/2/2	1	0	22.43579172	114.05189600	https://www.inaturalist.org/observations/106021124
74	Emberiza spodocephala	Black-faced Bunting	2021/12/13	1	0	22.43852376	120.58027190	https://www.inaturalist.org/observations/102973769
75	Emberiza spodocephala	Black-faced Bunting	2022/4/2	2	0	22.43882323	114.03063320	https://www.inaturalist.org/observations/110243253
76	Emberiza spodocephala	Black-faced Bunting	2021/2/15	1	0	22.44196483	114.02008280	https://www.inaturalist.org/observations/69769953
77	Emberiza spodocephala	Black-faced Bunting	2020/3/28	1	0	22.44590940	114.14028140	https://www.inaturalist.org/observations/40882429
78	Emberiza spodocephala	Black-faced Bunting	2020/2/21	1	0	22.44601277	114.13775490	https://www.inaturalist.org/observations/44839084
79	Emberiza spodocephala	Black-faced Bunting	2019/11/24	1	0	22.44637100	114.13815900	https://www.inaturalist.org/observations/36661350
80	Emberiza spodocephala	Black-faced Bunting	2019/1/2	2	0	22.44711886	114.05116840	https://www.inaturalist.org/observations/19371339
81	Emberiza spodocephala	Black-faced Bunting	2019/11/17	1	0	22.44732325	114.14108970	https://www.inaturalist.org/observations/35773350
82	Emberiza spodocephala	Black-faced Bunting	2021/2/18	1	0	22.45268130	114.05272340	https://www.inaturalist.org/observations/69803725
83	Emberiza spodocephala	Black-faced Bunting	2022/3/26	1	0	22.45339167	114.05228520	https://www.inaturalist.org/observations/109817172
84	Emberiza spodocephala	Black-faced Bunting	2021/3/10	1	0	22.45372945	114.05251280	https://www.inaturalist.org/observations/71061818
85	Emberiza spodocephala	Black-faced Bunting	2022/1/29	1	0	22.45808321	114.02913220	https://www.inaturalist.org/observations/105955062
86	Emberiza spodocephala	Black-faced Bunting	2021/1/27	1	0	22.45867304	114.03848010	https://www.inaturalist.org/observations/70610374
87	Emberiza spodocephala	Black-faced Bunting	2021/12/2	1	0	22.46211310	114.15134060	https://www.inaturalist.org/observations/102392135
88	Emberiza spodocephala	Black-faced Bunting	2021/3/12	1	0	22.46521357	114.17793020	https://www.inaturalist.org/observations/71493464
89	Emberiza spodocephala	Black-faced Bunting	2022/1/29	0	0	22.46569500	114.01693620	https://www.inaturalist.org/observations/105956800
90	Emberiza spodocephala	Black-faced Bunting	2021/11/5	0	0	22.46842628	114.01638060	https://www.inaturalist.org/observations/100329662
91	Emberiza spodocephala	Black-faced Bunting	2022/2/26	1	0	22.46882417	114.01676060	https://www.inaturalist.org/observations/107463832

92	Emberiza spodocephala	Black-faced Bunting	2022/1/9	1	0	22.46983906	114.02096700	https://www.inaturalist.org/observations/104967214
93	Emberiza spodocephala	Black-faced Bunting	2012/1/11	1	0	22.47062472	114.00607030	https://www.inaturalist.org/observations/105007098
94	Emberiza spodocephala	Black-faced Bunting	2021/11/27	1	0	22.47110406	114.01758040	https://www.inaturalist.org/observations/102004282
95	Emberiza spodocephala	Black-faced Bunting	2021/11/27	1	0	22.47110406	114.01758040	https://www.inaturalist.org/observations/102004712
96	Emberiza spodocephala	Black-faced Bunting	2022/2/10	2	0	22.47110406	114.01758040	https://www.inaturalist.org/observations/106478044
97	Emberiza spodocephala	Black-faced Bunting	2022/4/2	1	0	22.47110406	114.01758040	https://www.inaturalist.org/observations/110159683
98	Emberiza spodocephala	Black-faced Bunting	2022/4/2	2	0	22.47110406	114.01758040	https://www.inaturalist.org/observations/110159689
99	Emberiza spodocephala	Black-faced Bunting	2021/1/1	3	0	22.47110430	114.01758040	https://www.inaturalist.org/observations/67439505
100	Emberiza spodocephala	Black-faced Bunting	2022/4/12	1	0	22.47110430	114.01758040	https://www.inaturalist.org/observations/111203447
101	Emberiza spodocephala	Black-faced Bunting	2021/11/15	2	0	22.47112110	114.01758040	https://www.inaturalist.org/observations/101235449
102	Emberiza spodocephala	Black-faced Bunting	2022/1/9	1	0	22.47138454	114.02265920	https://www.inaturalist.org/observations/104967212
103	Emberiza spodocephala	Black-faced Bunting	2021/2/13	1	0	22.47212007	114.01257280	https://www.inaturalist.org/observations/69551255
104	Emberiza spodocephala	Black-faced Bunting	2020/1/19	0	0	22.47313319	114.18297150	https://www.inaturalist.org/observations/37738773
105	Emberiza spodocephala	Black-faced Bunting	2021/11/20	1	0	22.47341986	114.01537090	https://www.inaturalist.org/observations/101548044
106	Emberiza spodocephala	Black-faced Bunting	2021/11/27	1	0	22.47359891	114.01599490	https://www.inaturalist.org/observations/102002223
107	Emberiza spodocephala	Black-faced Bunting	2021/11/27	1	0	22.47359891	114.01599490	https://www.inaturalist.org/observations/102002224
108	Emberiza spodocephala	Black-faced Bunting	2020/1/11	1	0	22.47387808	113.99562180	https://www.inaturalist.org/observations/37484189
109	Emberiza spodocephala	Black-faced Bunting	2021/12/18	1	0	22.47399801	114.03639880	https://www.inaturalist.org/observations/103254019
110	Emberiza spodocephala	Black-faced Bunting	2022/2/27	1	0	22.47445656	114.12378400	https://www.inaturalist.org/observations/107564161
111	Emberiza spodocephala	Black-faced Bunting	2020/12/24	1	0	22.47524833	114.00944080	https://www.inaturalist.org/observations/67003702
112	Emberiza spodocephala	Black-faced Bunting	2020/2/9	0	0	22.47589775	114.00826020	https://www.inaturalist.org/observations/38456638
113	Emberiza spodocephala	Black-faced Bunting	2022/4/3	1	0	22.47590069	114.04195630	https://www.inaturalist.org/observations/110261693
114	Emberiza spodocephala	Black-faced Bunting	2020/3/28	1	0	22.47612036	114.00825550	https://www.inaturalist.org/observations/40871647

115	Emberiza spodocephala	Black-faced Bunting	2020/2/2	0	0	22.48224153	114.01154550	https://www.inaturalist.org/observations/38662992
116	Emberiza spodocephala	Black-faced Bunting	2017/3/21	1	0	22.48419600	114.10110600	https://www.inaturalist.org/observations/5463266
117	Emberiza spodocephala	Black-faced Bunting	2020/11/15	2	0	22.48439674	114.03109950	https://www.inaturalist.org/observations/65072689
118	Emberiza spodocephala	Black-faced Bunting	2020/11/15	2	0	22.48439674	114.03109950	https://www.inaturalist.org/observations/65072693
119	Emberiza spodocephala	Black-faced Bunting	2022/1/12	1	0	22.48494641	114.04350500	https://www.inaturalist.org/observations/104808196
120	Emberiza spodocephala	Black-faced Bunting	2022/1/23	1	0	22.48658208	114.04099880	https://www.inaturalist.org/observations/105427880
121	Emberiza spodocephala	Black-faced Bunting	2021/12/29	1	0	22.48784197	114.04187960	https://www.inaturalist.org/observations/103885716
122	Emberiza spodocephala	Black-faced Bunting	2022/2/1	1	0	22.49145858	114.04413270	https://www.inaturalist.org/observations/106065667
123	Emberiza spodocephala	Black-faced Bunting	2022/3/7	1	0	22.49173572	114.04491500	https://www.inaturalist.org/observations/115135437
124	Emberiza spodocephala	Black-faced Bunting	2019/11/30	1	0	22.49177367	114.04434760	https://www.inaturalist.org/observations/36214291
125	Emberiza spodocephala	Black-faced Bunting	2016/4/2	1	0	22.49184506	114.03517880	https://www.inaturalist.org/observations/58912848
126	Emberiza spodocephala	Black-faced Bunting	2016/3/9	3	0	22.49210500	114.04454000	https://www.inaturalist.org/observations/12632303
127	Emberiza spodocephala	Black-faced Bunting	2020/12/17	1	0	22.49249053	114.04478380	https://www.inaturalist.org/observations/74344271
128	Emberiza spodocephala	Black-faced Bunting	2012/3/4	1	0	22.49293600	114.04499900	https://www.inaturalist.org/observations/35906172
129	Emberiza spodocephala	Black-faced Bunting	2022/4/6	2	0	22.49338700	114.04526700	https://www.inaturalist.org/observations/110659229
130	Emberiza spodocephala	Black-faced Bunting	2022/1/7	1	0	22.49344408	114.04566840	https://www.inaturalist.org/observations/104533236
131	Emberiza spodocephala	Black-faced Bunting	2019/12/6	1	0	22.49348232	114.03410140	https://www.inaturalist.org/observations/36556883
132	Emberiza spodocephala	Black-faced Bunting	2022/4/16	1	0	22.49381760	114.03468380	https://www.inaturalist.org/observations/111737490
133	Emberiza spodocephala	Black-faced Bunting	2021/4/4	1	0	22.49384622	114.17161370	https://www.inaturalist.org/observations/75421978
134	Emberiza spodocephala	Black-faced Bunting	2018/4/2	1	0	22.49419514	114.04608430	https://www.inaturalist.org/observations/10770109
135	Emberiza spodocephala	Black-faced Bunting	2021/12/9	1	0	22.49448333	114.04639450	https://www.inaturalist.org/observations/102861124
136	Emberiza spodocephala	Black-faced Bunting	2022/4/9	1	0	22.49452167	114.04641670	https://www.inaturalist.org/observations/111086373
137	Emberiza spodocephala	Black-faced Bunting	2022/1/31	2	0	22.49505690	114.03393420	https://www.inaturalist.org/observations/107483605

138	Emberiza spodocephala	Black-faced Bunting	2021/2/21	2	0	22.49506125	114.03393420	https://www.inaturalist.org/observations/69916919
139	Emberiza spodocephala	Black-faced Bunting	2022/1/27	1	0	22.49506125	114.03393420	https://www.inaturalist.org/observations/105696868
140	Emberiza spodocephala	Black-faced Bunting	2022/3/5	1	0	22.49506125	114.03393420	https://www.inaturalist.org/observations/108227561
141	Emberiza spodocephala	Black-faced Bunting	2022/2/27	1	0	22.49530405	114.12601880	https://www.inaturalist.org/observations/107564139
142	Emberiza spodocephala	Black-faced Bunting	2020/5/1	1	0	22.49849306	114.03948450	https://www.inaturalist.org/observations/44816750
143	Emberiza spodocephala	Black-faced Bunting	2021/11/24	1	0	22.49857516	114.04498160	https://www.inaturalist.org/observations/102240498
144	Emberiza spodocephala	Black-faced Bunting	2019/11/30	1	0	22.49861267	114.04336850	https://www.inaturalist.org/observations/36252238
145	Emberiza spodocephala	Black-faced Bunting	2022/1/2	1	0	22.49883848	114.04582750	https://www.inaturalist.org/observations/104166075
146	Emberiza spodocephala	Black-faced Bunting	2021/11/15	1	0	22.50004095	114.11666660	https://www.inaturalist.org/observations/101187381
147	Emberiza spodocephala	Black-faced Bunting	2013/1/19	1	0	22.50879520	114.11297710	https://www.inaturalist.org/observations/29955493
148	Emberiza spodocephala	Black-faced Bunting	2020/11/23	1	0	22.50879520	114.11297710	https://www.inaturalist.org/observations/65474073
149	Emberiza spodocephala	Black-faced Bunting	2022/4/4	1	0	22.50991806	114.08150550	https://www.inaturalist.org/observations/110798382
150	Emberiza spodocephala	Black-faced Bunting	2022/2/27	1	0	22.51158461	114.09094890	https://www.inaturalist.org/observations/107564109
151	Emberiza spodocephala	Black-faced Bunting	2022/1/25	1	0	22.51179700	114.06972000	https://www.inaturalist.org/observations/105554099
152	Emberiza spodocephala	Black-faced Bunting	2022/1/17	1	0	22.51398760	114.07001480	https://www.inaturalist.org/observations/105100619
153	Emberiza spodocephala	Black-faced Bunting	2021/3/17	1	0	22.52330736	114.08897700	https://www.inaturalist.org/observations/91070530
154	Emberiza spodocephala	Black-faced Bunting	2020/12/8	1	0	22.52601690	114.11515610	https://www.inaturalist.org/observations/66695792
155	Emberiza spodocephala	Black-faced Bunting	2021/11/21	1	0	22.53075558	114.19811940	https://www.inaturalist.org/observations/101617212
156	Emberiza spodocephala	Black-faced Bunting	2022/2/2	1	0	22.53106317	114.10793930	https://www.inaturalist.org/observations/106021141
157	Emberiza spodocephala	Black-faced Bunting	2022/4/13	1	0	22.54841064	114.02600210	https://www.inaturalist.org/observations/111392823
158	Emberiza spodocephala	Black-faced Bunting	2022/2/2	1	0	22.55812721	114.10004980	https://www.inaturalist.org/observations/106021140
159	Emberiza spodocephala	Black-faced Bunting	2022/2/2	1	0	22.57682493	114.00149850	https://www.inaturalist.org/observations/106021123
160	Emberiza spodocephala	Black-faced Bunting	2021/3/11	1	0	22.62605182	120.59678850	https://www.inaturalist.org/observations/70994421

161	Emberiza spodocephala	Black-faced Bunting	2021/2/25	1	0	22.62654295	120.59707760	https://www.inaturalist.org/observations/70249058
162	Emberiza spodocephala	Black-faced Bunting	2022/1/2	2	0	22.67296671	120.44341330	https://www.inaturalist.org/observations/134935099
163	Emberiza spodocephala	Black-faced Bunting	2022/1/8	1	0	22.68700071	120.45736010	https://www.inaturalist.org/observations/121970103
164	Emberiza spodocephala	Black-faced Bunting	2020/3/18	2	0	22.69308793	120.45967130	https://www.inaturalist.org/observations/40168623
165	Emberiza spodocephala	Black-faced Bunting	2021/3/20	1	0	22.69631333	121.05742670	https://www.inaturalist.org/observations/73725790
166	Emberiza spodocephala	Black-faced Bunting	2020/3/16	1	0	22.70137932	120.46323290	https://www.inaturalist.org/observations/40064903
167	Emberiza spodocephala	Black-faced Bunting	2005/4/4	1	0	22.87319939	120.51048350	https://www.inaturalist.org/observations/19192465
168	Emberiza spodocephala	Black-faced Bunting	2022/1/1	1	0	23.30167409	121.29887920	https://www.inaturalist.org/observations/105137910
169	Emberiza spodocephala	Black-faced Bunting	2022/2/10	1	0	23.44956500	121.39770170	https://www.inaturalist.org/observations/106475670
170	Emberiza spodocephala	Black-faced Bunting	2022/2/10	1	0	23.44966021	121.39744320	https://www.inaturalist.org/observations/106475674
171	Emberiza spodocephala	Black-faced Bunting	2021/11/23	3	0	23.45271405	116.73373030	https://www.inaturalist.org/observations/101763319
172	Emberiza spodocephala	Black-faced Bunting	2019/11/16	2	0	23.54899018	119.60679400	https://www.inaturalist.org/observations/102816346
173	Emberiza spodocephala	Black-faced Bunting	2019/11/2	2	0	23.55760528	119.61450440	https://www.inaturalist.org/observations/102816347
174	Emberiza spodocephala	Black-faced Bunting	2019/11/9	1	0	23.55761234	119.61447000	https://www.inaturalist.org/observations/102816344
175	Emberiza spodocephala	Black-faced Bunting	2019/11/9	1	0	23.55767472	119.61440310	https://www.inaturalist.org/observations/102816348
176	Emberiza spodocephala	Black-faced Bunting	2021/11/10	1	0	23.57839500	119.65962830	https://www.inaturalist.org/observations/118890107
177	Emberiza spodocephala	Black-faced Bunting	2022/4/15	2	0	23.61784897	120.26066360	https://www.inaturalist.org/observations/118892575
178	Emberiza spodocephala	Black-faced Bunting	2014/1/11	1	0	23.62321935	120.48650720	https://www.inaturalist.org/observations/122142920
179	Emberiza spodocephala	Black-faced Bunting	2019/12/14	2	0	23.63584042	120.52596730	https://www.inaturalist.org/observations/36630143
180	Emberiza spodocephala	Black-faced Bunting	2020/3/22	2	0	23.65233551	120.49228650	https://www.inaturalist.org/observations/45155867
181	Emberiza spodocephala	Black-faced Bunting	2020/11/28	1	0	23.67985914	120.17317960	https://www.inaturalist.org/observations/65741379
182	Emberiza spodocephala	Black-faced Bunting	2014/3/19	1	0	23.68464100	120.32053900	https://www.inaturalist.org/observations/15536684
183	Emberiza spodocephala	Black-faced Bunting	2012/12/18	1	0	23.72519640	120.62359190	https://www.inaturalist.org/observations/19485205

184	Emberiza spodocephala	Black-faced Bunting	2018/2/24	1	0	23.73131278	120.37956860	https://www.inaturalist.org/observations/18502116
185	Emberiza spodocephala	Black-faced Bunting	2022/4/17	1	0	23.74397667	121.46425170	https://www.inaturalist.org/observations/120114171
186	Emberiza spodocephala	Black-faced Bunting	2022/4/17	1	0	23.75759752	121.47268830	https://www.inaturalist.org/observations/126179591
187	Emberiza spodocephala	Black-faced Bunting	2019/3/8	1	0	23.77041210	121.47132650	https://www.inaturalist.org/observations/24596973
188	Emberiza spodocephala	Black-faced Bunting	2017/12/7	1	0	23.79820461	120.22152240	https://www.inaturalist.org/observations/18672516
189	Emberiza spodocephala	Black-faced Bunting	2021/12/20	1	0	23.80263582	120.22774620	https://www.inaturalist.org/observations/103941156
190	Emberiza spodocephala	Black-faced Bunting	2021/12/17	1	0	23.85256220	121.51257440	https://www.inaturalist.org/observations/103416918
191	Emberiza spodocephala	Black-faced Bunting	2022/4/9	1	0	23.88816464	121.54787370	https://www.inaturalist.org/observations/114310156
192	Emberiza spodocephala	Black-faced Bunting	2021/3/28	1	0	23.90963967	121.54208610	https://www.inaturalist.org/observations/72773114
193	Emberiza spodocephala	Black-faced Bunting	2018/12/23	1	0	23.91597636	121.56030040	https://www.inaturalist.org/observations/109835827
194	Emberiza spodocephala	Black-faced Bunting	2021/12/21	1	0	23.91663862	121.55969600	https://www.inaturalist.org/observations/103416928
195	Emberiza spodocephala	Black-faced Bunting	2021/12/21	1	0	23.91665529	121.55966190	https://www.inaturalist.org/observations/103416929
196	Emberiza spodocephala	Black-faced Bunting	2020/1/8	1	0	23.91745360	121.55954670	https://www.inaturalist.org/observations/107315227
197	Emberiza spodocephala	Black-faced Bunting	2020/1/6	1	0	23.91766600	121.54793900	https://www.inaturalist.org/observations/37395068
198	Emberiza spodocephala	Black-faced Bunting	2021/11/16	1	0	23.94745683	121.59455940	https://www.inaturalist.org/observations/101257543
199	Emberiza spodocephala	Black-faced Bunting	2019/1/4	1	0	23.95734201	120.84366860	https://www.inaturalist.org/observations/19414090
200	Emberiza spodocephala	Black-faced Bunting	2021/12/3	1	0	23.99641876	121.43236400	https://www.inaturalist.org/observations/103079127
201	Emberiza spodocephala	Black-faced Bunting	2020/4/15	1	0	23.99881376	120.57519760	https://www.inaturalist.org/observations/42245677
202	Emberiza spodocephala	Black-faced Bunting	2014/3/19	1	0	24.02858215	121.16448590	https://www.inaturalist.org/observations/92973737
203	Emberiza spodocephala	Black-faced Bunting	2013/11/15	1	0	24.04068100	121.15689900	https://www.inaturalist.org/observations/8674286
204	Emberiza spodocephala	Black-faced Bunting	2021/11/28	1	0	24.18405415	120.63506190	https://www.inaturalist.org/observations/102069503
205	Emberiza spodocephala	Black-faced Bunting	2020/11/11	1	0	24.19625568	121.42729690	https://www.inaturalist.org/observations/64664858
206	Emberiza spodocephala	Black-faced Bunting	2020/3/28	1	0	24.20698244	120.50437230	https://www.inaturalist.org/observations/40869061

207	Emberiza spodocephala	Black-faced Bunting	2022/1/8	1	0	24.20824683	120.59664730	https://www.inaturalist.org/observations/120297884
208	Emberiza spodocephala	Black-faced Bunting	2021/12/12	1	0	24.29148740	121.45107880	https://www.inaturalist.org/observations/103079147
209	Emberiza spodocephala	Black-faced Bunting	2018/4/4	2	0	24.31293399	120.54593470	https://www.inaturalist.org/observations/51749855
210	Emberiza spodocephala	Black-faced Bunting	2021/3/14	1	0	24.32838045	121.31083690	https://www.inaturalist.org/observations/71287716
211	Emberiza spodocephala	Black-faced Bunting	2019/3/1	0	0	24.35235870	121.30859720	https://www.inaturalist.org/observations/37993295
212	Emberiza spodocephala	Black-faced Bunting	2019/11/30	1	0	24.38262723	121.30559800	https://www.inaturalist.org/observations/36467794
213	Emberiza spodocephala	Black-faced Bunting	2021/12/10	1	0	24.41482396	118.28899700	https://www.inaturalist.org/observations/133921789
214	Emberiza spodocephala	Black-faced Bunting	2021/12/10	1	0	24.41485389	118.28901510	https://www.inaturalist.org/observations/133977383
215	Emberiza spodocephala	Black-faced Bunting	2021/1/21	1	0	24.42499458	118.30404420	https://www.inaturalist.org/observations/68912266
216	Emberiza spodocephala	Black-faced Bunting	2019/2/12	1	0	24.43820403	118.37250970	https://www.inaturalist.org/observations/135127227
217	Emberiza spodocephala	Black-faced Bunting	2014/12/27	1	0	24.43916050	118.37251380	https://www.inaturalist.org/observations/57535447
218	Emberiza spodocephala	Black-faced Bunting	2020/12/29	1	0	24.44383281	118.37524300	https://www.inaturalist.org/observations/67349816
219	Emberiza spodocephala	Black-faced Bunting	2017/2/6	1	0	24.44713762	118.44883840	https://www.inaturalist.org/observations/57000560
220	Emberiza spodocephala	Black-faced Bunting	2013/2/17	1	0	24.44839901	118.37920810	https://www.inaturalist.org/observations/56706647
221	Emberiza spodocephala	Black-faced Bunting	2014/12/17	1	0	24.44937260	118.37663520	https://www.inaturalist.org/observations/16787707
222	Emberiza spodocephala	Black-faced Bunting	2019/1/3	1	0	24.45516371	118.39649120	https://www.inaturalist.org/observations/19466281
223	Emberiza spodocephala	Black-faced Bunting	2019/1/3	1	0	24.45542167	118.39607170	https://www.inaturalist.org/observations/29001496
224	Emberiza spodocephala	Black-faced Bunting	2021/12/6	1	0	24.47088634	118.29140550	https://www.inaturalist.org/observations/133072369
225	Emberiza spodocephala	Black-faced Bunting	2021/12/6	1	0	24.47088634	118.29140550	https://www.inaturalist.org/observations/133072370
226	Emberiza spodocephala	Black-faced Bunting	2021/12/6	1	0	24.47088634	118.29140550	https://www.inaturalist.org/observations/133072371
227	Emberiza spodocephala	Black-faced Bunting	2021/12/6	1	0	24.47088634	118.29140550	https://www.inaturalist.org/observations/133072374
228	Emberiza spodocephala	Black-faced Bunting	2021/4/6	1	0	24.47355000	118.29054330	https://www.inaturalist.org/observations/73697432
229	Emberiza spodocephala	Black-faced Bunting	2015/2/23	1	0	24.47412600	118.32324420	https://www.inaturalist.org/observations/57606451

230	Emberiza spodocephala	Black-faced Bunting	2010/11/7	1	0	24.49340088	121.53592060	https://www.inaturalist.org/observations/55753344
231	Emberiza spodocephala	Black-faced Bunting	2021/3/31	1	0	24.50591200	121.11406780	https://www.inaturalist.org/observations/72672959
232	Emberiza spodocephala	Black-faced Bunting	2021/12/8	1	0	24.50874748	118.40754050	https://www.inaturalist.org/observations/133925125
233	Emberiza spodocephala	Black-faced Bunting	2022/4/9	1	0	24.53779012	120.70910210	https://www.inaturalist.org/observations/110845675
234	Emberiza spodocephala	Black-faced Bunting	2022/3/12	1	0	24.61332544	120.83118140	https://www.inaturalist.org/observations/118570545
235	Emberiza spodocephala	Black-faced Bunting	2019/2/25	1	0	24.61553606	121.84666460	https://www.inaturalist.org/observations/70640506
236	Emberiza spodocephala	Black-faced Bunting	2022/3/17	1	0	24.64897000	121.60018800	https://www.inaturalist.org/observations/123178541
237	Emberiza spodocephala	Black-faced Bunting	2021/12/20	1	0	24.65416424	121.82215630	https://www.inaturalist.org/observations/105309371
238	Emberiza spodocephala	Black-faced Bunting	2021/12/20	1	0	24.65416424	121.82215630	https://www.inaturalist.org/observations/105309372
239	Emberiza spodocephala	Black-faced Bunting	2019/3/25	1	0	24.65490061	121.81748900	https://www.inaturalist.org/observations/22230593
240	Emberiza spodocephala	Black-faced Bunting	2022/4/17	0	0	24.65900858	121.14062940	https://www.inaturalist.org/observations/111715172
241	Emberiza spodocephala	Black-faced Bunting	2021/1/2	1	0	24.66184797	121.81866380	https://www.inaturalist.org/observations/67477990
242	Emberiza spodocephala	Black-faced Bunting	2020/3/29	1	0	24.66316422	121.81805670	https://www.inaturalist.org/observations/49709152
243	Emberiza spodocephala	Black-faced Bunting	2019/12/21	1	0	24.66494600	121.81942800	https://www.inaturalist.org/observations/55162432
244	Emberiza spodocephala	Black-faced Bunting	2021/3/31	1	0	24.66788573	121.81971500	https://www.inaturalist.org/observations/79005003
245	Emberiza spodocephala	Black-faced Bunting	2022/3/19	1	0	24.67855926	120.83685850	https://www.inaturalist.org/observations/112803571
246	Emberiza spodocephala	Black-faced Bunting	2022/3/12	1	0	24.70987132	120.98042630	https://www.inaturalist.org/observations/118570546
247	Emberiza spodocephala	Black-faced Bunting	2021/11/19	1	0	24.71767148	121.81084070	https://www.inaturalist.org/observations/101521320
248	Emberiza spodocephala	Black-faced Bunting	2021/11/25	1	0	24.71904199	121.81301450	https://www.inaturalist.org/observations/101934633
249	Emberiza spodocephala	Black-faced Bunting	2014/3/25	1	0	24.73081428	121.75357380	https://www.inaturalist.org/observations/60852423
250	Emberiza spodocephala	Black-faced Bunting	2022/1/8	2	0	24.73458659	121.74636840	https://www.inaturalist.org/observations/105745256
251	Emberiza spodocephala	Black-faced Bunting	2022/1/8	2	0	24.73458659	121.74636840	https://www.inaturalist.org/observations/105745261
252	Emberiza spodocephala	Black-faced Bunting	2020/1/4	1	0	24.73637878	121.77082580	https://www.inaturalist.org/observations/37298647

253	Emberiza spodocephala	Black-faced Bunting	2018/11/15	2	0	24.74771200	121.63895500	https://www.inaturalist.org/observations/18428888
254	Emberiza spodocephala	Black-faced Bunting	2021/2/20	1	0	24.74900500	121.63821000	https://www.inaturalist.org/observations/69924038
255	Emberiza spodocephala	Black-faced Bunting	2021/12/5	2	0	24.74914614	121.64021530	https://www.inaturalist.org/observations/102523885
256	Emberiza spodocephala	Black-faced Bunting	2021/12/5	2	0	24.75226399	121.63646020	https://www.inaturalist.org/observations/102524709
257	Emberiza spodocephala	Black-faced Bunting	2021/12/5	2	0	24.75226399	121.63646020	https://www.inaturalist.org/observations/102524711
258	Emberiza spodocephala	Black-faced Bunting	2018/11/15	3	0	24.75295700	121.63788100	https://www.inaturalist.org/observations/18428898
259	Emberiza spodocephala	Black-faced Bunting	2019/4/6	2	0	24.75477620	121.63754530	https://www.inaturalist.org/observations/22345924
260	Emberiza spodocephala	Black-faced Bunting	2021/1/12	1	0	24.78171410	118.67394200	https://www.inaturalist.org/observations/69035988
261	Emberiza spodocephala	Black-faced Bunting	2022/1/10	2	0	24.79811027	121.03601910	https://www.inaturalist.org/observations/105137934
262	Emberiza spodocephala	Black-faced Bunting	2013/2/16	1	0	24.82503080	121.79718820	https://www.inaturalist.org/observations/56703185
263	Emberiza spodocephala	Black-faced Bunting	2013/2/16	1	0	24.82503080	121.79718820	https://www.inaturalist.org/observations/56703190
264	Emberiza spodocephala	Black-faced Bunting	2021/4/5	1	0	24.82578679	121.40911870	https://www.inaturalist.org/observations/72983196
265	Emberiza spodocephala	Black-faced Bunting	2022/4/3	1	0	24.83636803	121.81363350	https://www.inaturalist.org/observations/110268710
266	Emberiza spodocephala	Black-faced Bunting	2022/3/16	1	0	24.89550833	121.10747330	https://www.inaturalist.org/observations/109990313
267	Emberiza spodocephala	Black-faced Bunting	2022/4/13	2	0	24.97287902	121.42444590	https://www.inaturalist.org/observations/113349215
268	Emberiza spodocephala	Black-faced Bunting	2019/4/20	1	0	24.99983040	121.43860320	https://www.inaturalist.org/observations/23911448
269	Emberiza spodocephala	Black-faced Bunting	2021/11/25	1	0	25.00735021	121.59072590	https://www.inaturalist.org/observations/101934375
270	Emberiza spodocephala	Black-faced Bunting	2013/4/28	1	0	25.01120790	121.92426050	https://www.inaturalist.org/observations/45677047
271	Emberiza spodocephala	Black-faced Bunting	2020/11/26	2	0	25.01160118	121.92338300	https://www.inaturalist.org/observations/65625997
272	Emberiza spodocephala	Black-faced Bunting	2021/11/3	1	0	25.01325123	121.92070210	https://www.inaturalist.org/observations/134571088
273	Emberiza spodocephala	Black-faced Bunting	2021/12/13	1	0	25.01472023	121.91945910	https://www.inaturalist.org/observations/103051049
274	Emberiza spodocephala	Black-faced Bunting	2019/4/8	1	0	25.01482600	121.44341000	https://www.inaturalist.org/observations/65251147
275	Emberiza spodocephala	Black-faced Bunting	2019/4/19	1	0	25.01621667	121.92254830	https://www.inaturalist.org/observations/22752906

276	Emberiza spodocephala	Black-faced Bunting	2019/4/26	2	0	25.01692409	121.92605230	https://www.inaturalist.org/observations/48523520
277	Emberiza spodocephala	Black-faced Bunting	2020/11/15	1	0	25.01718928	121.92434570	https://www.inaturalist.org/observations/65402744
278	Emberiza spodocephala	Black-faced Bunting	2021/11/5	1	0	25.01764479	121.92217680	https://www.inaturalist.org/observations/100305655
279	Emberiza spodocephala	Black-faced Bunting	2021/11/5	1	0	25.01777262	121.92252180	https://www.inaturalist.org/observations/100305654
280	Emberiza spodocephala	Black-faced Bunting	2014/11/2	1	0	25.01789152	121.92384010	https://www.inaturalist.org/observations/57529100
281	Emberiza spodocephala	Black-faced Bunting	2020/11/7	1	0	25.01818528	121.92341420	https://www.inaturalist.org/observations/64453431
282	Emberiza spodocephala	Black-faced Bunting	2020/11/30	1	0	25.01867783	121.54324500	https://www.inaturalist.org/observations/65876252
283	Emberiza spodocephala	Black-faced Bunting	2015/1/1	1	0	25.01868650	121.92372340	https://www.inaturalist.org/observations/57601456
284	Emberiza spodocephala	Black-faced Bunting	2021/4/11	1	0	25.01937778	121.92177310	https://www.inaturalist.org/observations/73718710
285	Emberiza spodocephala	Black-faced Bunting	2010/11/6	1	0	25.01954809	121.92353430	https://www.inaturalist.org/observations/55753956
286	Emberiza spodocephala	Black-faced Bunting	2021/4/11	1	0	25.01958056	121.92188670	https://www.inaturalist.org/observations/73718688
287	Emberiza spodocephala	Black-faced Bunting	2020/11/26	1	0	25.02010058	121.92247920	https://www.inaturalist.org/observations/65625991
288	Emberiza spodocephala	Black-faced Bunting	2021/11/15	2	0	25.02016117	121.92233670	https://www.inaturalist.org/observations/101236167
289	Emberiza spodocephala	Black-faced Bunting	2017/2/18	1	0	25.02232745	121.92491450	https://www.inaturalist.org/observations/91611965
290	Emberiza spodocephala	Black-faced Bunting	2022/4/23	1	0	25.02888928	121.93027640	https://www.inaturalist.org/observations/121399482
291	Emberiza spodocephala	Black-faced Bunting	2020/11/14	1	0	25.03317071	121.26205840	https://www.inaturalist.org/observations/68752394
292	Emberiza spodocephala	Black-faced Bunting	2019/3/26	2	0	25.03802831	121.49104370	https://www.inaturalist.org/observations/21730990
293	Emberiza spodocephala	Black-faced Bunting	2021/12/28	1	0	25.03933241	121.49342780	https://www.inaturalist.org/observations/103886646
294	Emberiza spodocephala	Black-faced Bunting	2022/2/2	1	0	25.03994958	121.49686700	https://www.inaturalist.org/observations/106064399
295	Emberiza spodocephala	Black-faced Bunting	2019/12/24	1	0	25.06504667	121.23542670	https://www.inaturalist.org/observations/66245515
296	Emberiza spodocephala	Black-faced Bunting	2022/2/9	3	0	25.06744681	121.53918740	https://www.inaturalist.org/observations/106579702
297	Emberiza spodocephala	Black-faced Bunting	2019/5/10	1	0	25.06849705	121.61980170	https://www.inaturalist.org/observations/24884004
298	Emberiza spodocephala	Black-faced Bunting	2020/11/16	0	0	25.07095300	121.78063900	https://www.inaturalist.org/observations/66328374

299	Emberiza spodocephala	Black-faced Bunting	2020/12/31	1	0	25.08365945	121.18321190	https://www.inaturalist.org/observations/67353630
300	Emberiza spodocephala	Black-faced Bunting	2021/4/5	1	0	25.08674268	121.17516760	https://www.inaturalist.org/observations/73082284
301	Emberiza spodocephala	Black-faced Bunting	2022/3/30	1	0	25.08733380	121.17625240	https://www.inaturalist.org/observations/113782307
302	Emberiza spodocephala	Black-faced Bunting	2022/4/17	1	0	25.08938432	121.45177710	https://www.inaturalist.org/observations/112129502
303	Emberiza spodocephala	Black-faced Bunting	2021/11/9	1	0	25.09137421	121.58301580	https://www.inaturalist.org/observations/100671722
304	Emberiza spodocephala	Black-faced Bunting	2019/12/14	1	0	25.09849587	121.45511260	https://www.inaturalist.org/observations/37191642
305	Emberiza spodocephala	Black-faced Bunting	2021/12/5	1	0	25.09862461	121.45505780	https://www.inaturalist.org/observations/105604604
306	Emberiza spodocephala	Black-faced Bunting	2019/4/10	1	0	25.09906251	121.45755740	https://www.inaturalist.org/observations/22651438
307	Emberiza spodocephala	Black-faced Bunting	2019/4/19	2	0	25.10168979	121.45466700	https://www.inaturalist.org/observations/24286239
308	Emberiza spodocephala	Black-faced Bunting	2020/1/27	0	0	25.10822500	121.86374170	https://www.inaturalist.org/observations/38028982
309	Emberiza spodocephala	Black-faced Bunting	2019/1/5	0	0	25.10868732	121.45620550	https://www.inaturalist.org/observations/19530961
310	Emberiza spodocephala	Black-faced Bunting	2020/4/4	1	0	25.11230933	121.49062700	https://www.inaturalist.org/observations/63752551
311	Emberiza spodocephala	Black-faced Bunting	2020/4/4	1	0	25.11230933	121.49062700	https://www.inaturalist.org/observations/63752552
312	Emberiza spodocephala	Black-faced Bunting	2019/12/7	1	0	25.11326041	121.48979340	https://www.inaturalist.org/observations/54244186
313	Emberiza spodocephala	Black-faced Bunting	2019/12/7	1	0	25.11327831	121.48971040	https://www.inaturalist.org/observations/54244192
314	Emberiza spodocephala	Black-faced Bunting	2021/1/21	1	0	25.11543667	121.47226330	https://www.inaturalist.org/observations/68633932
315	Emberiza spodocephala	Black-faced Bunting	2022/5/1	1	0	25.12345161	121.86994440	https://www.inaturalist.org/observations/114637789
316	Emberiza spodocephala	Black-faced Bunting	2020/12/10	1	0	25.12532500	121.48161670	https://www.inaturalist.org/observations/66454343
317	Emberiza spodocephala	Black-faced Bunting	2020/12/10	1	0	25.12606611	121.48518500	https://www.inaturalist.org/observations/66453453
318	Emberiza spodocephala	Black-faced Bunting	2020/12/10	0	0	25.12607694	121.48509420	https://www.inaturalist.org/observations/66453450
319	Emberiza spodocephala	Black-faced Bunting	2020/12/10	1	0	25.12608861	121.48507280	https://www.inaturalist.org/observations/66453451
320	Emberiza spodocephala	Black-faced Bunting	2020/12/10	0	0	25.12615278	121.48534670	https://www.inaturalist.org/observations/66453449
321	Emberiza spodocephala	Black-faced Bunting	2021/4/2	1	0	25.12623167	121.48549360	https://www.inaturalist.org/observations/72706563

322	Emberiza spodocephala	Black-faced Bunting	2019/11/24	1	0	25.12914628	121.47698750	https://www.inaturalist.org/observations/36012650
323	Emberiza spodocephala	Black-faced Bunting	2021/1/26	1	0	25.13011000	121.48831130	https://www.inaturalist.org/observations/68685492
324	Emberiza spodocephala	Black-faced Bunting	2021/1/26	1	0	25.13011000	121.48831130	https://www.inaturalist.org/observations/68685496
325	Emberiza spodocephala	Black-faced Bunting	2021/1/26	1	0	25.13011000	121.48831130	https://www.inaturalist.org/observations/68685497
326	Emberiza spodocephala	Black-faced Bunting	2021/1/26	1	0	25.13011000	121.48831130	https://www.inaturalist.org/observations/68685498
327	Emberiza spodocephala	Black-faced Bunting	2021/1/26	1	0	25.13011000	121.48831130	https://www.inaturalist.org/observations/68685508
328	Emberiza spodocephala	Black-faced Bunting	2022/2/12	2	0	25.13096667	121.49022830	https://www.inaturalist.org/observations/106598859
329	Emberiza spodocephala	Black-faced Bunting	2021/12/18	1	0	25.13375809	121.48921320	https://www.inaturalist.org/observations/134568322
330	Emberiza spodocephala	Black-faced Bunting	2020/3/9	1	0	25.13881574	121.52380940	https://www.inaturalist.org/observations/49694634
331	Emberiza spodocephala	Black-faced Bunting	2019/4/14	2	0	25.14451943	121.46347260	https://www.inaturalist.org/observations/30657019
332	Emberiza spodocephala	Black-faced Bunting	2022/5/5	1	0	25.14580360	121.79830340	https://www.inaturalist.org/observations/115639458
333	Emberiza spodocephala	Black-faced Bunting	2022/5/17	1	0	25.14644167	121.79724440	https://www.inaturalist.org/observations/117472265
334	Emberiza spodocephala	Black-faced Bunting	2010/3/14	1	0	25.15690148	121.54647220	https://www.inaturalist.org/observations/55473167
335	Emberiza spodocephala	Black-faced Bunting	2017/11/15	1	0	25.16946720	121.55556740	https://www.inaturalist.org/observations/14127757
336	Emberiza spodocephala	Black-faced Bunting	2021/1/18	1	0	25.22344286	121.64123960	https://www.inaturalist.org/observations/68264494
337	Emberiza spodocephala	Black-faced Bunting	2019/11/10	1	0	25.22397383	121.64182210	https://www.inaturalist.org/observations/35524276
338	Emberiza spodocephala	Black-faced Bunting	2022/3/7	2	0	25.22594741	121.64086150	https://www.inaturalist.org/observations/131190608
339	Emberiza spodocephala	Black-faced Bunting	2022/3/6	1	0	25.22608158	121.64126490	https://www.inaturalist.org/observations/131190598
340	Emberiza spodocephala	Black-faced Bunting	2022/4/16	2	0	25.22639195	121.63994360	https://www.inaturalist.org/observations/111762899
341	Emberiza spodocephala	Black-faced Bunting	2021/4/7	1	0	25.22639694	121.64164830	https://www.inaturalist.org/observations/73207479
342	Emberiza spodocephala	Black-faced Bunting	2022/4/16	2	0	25.22646503	121.64152860	https://www.inaturalist.org/observations/111762904
343	Emberiza spodocephala	Black-faced Bunting	2021/11/16	1	0	25.22646814	121.64116350	https://www.inaturalist.org/observations/104805315
344	Emberiza spodocephala	Black-faced Bunting	2012/3/25	1	0	25.22656088	121.64161200	https://www.inaturalist.org/observations/56213510

345	Emberiza spodocephala	Black-faced Bunting	2013/3/12	1	0	25.22667963	121.64127010	https://www.inaturalist.org/observations/41172780
346	Emberiza spodocephala	Black-faced Bunting	2013/3/12	1	0	25.22667963	121.64127010	https://www.inaturalist.org/observations/41172781
347	Emberiza spodocephala	Black-faced Bunting	2022/4/3	1	0	25.22700759	121.64128280	https://www.inaturalist.org/observations/110276592
348	Emberiza spodocephala	Black-faced Bunting	2021/3/1	1	0	25.22716561	121.64243180	https://www.inaturalist.org/observations/70393019
349	Emberiza spodocephala	Black-faced Bunting	2018/3/6	1	0	25.22777012	121.63993270	https://www.inaturalist.org/observations/103242161
350	Emberiza spodocephala	Black-faced Bunting	2020/11/20	1	0	25.22965000	121.63826100	https://www.inaturalist.org/observations/65456622
351	Emberiza spodocephala	Black-faced Bunting	2019/12/11	0	0	25.23028167	121.64592270	https://www.inaturalist.org/observations/36555972
352	Emberiza spodocephala	Black-faced Bunting	2013/4/20	1	0	25.23229915	121.63363620	https://www.inaturalist.org/observations/56722412
353	Emberiza spodocephala	Black-faced Bunting	2022/2/9	1	0	25.23662196	121.55095950	https://www.inaturalist.org/observations/106580058
354	Emberiza spodocephala	Black-faced Bunting	2020/4/26	1	0	25.24593316	121.62843990	https://www.inaturalist.org/observations/107175242
355	Emberiza spodocephala	Black-faced Bunting	2020/4/20	2	0	25.25970470	121.47582790	https://www.inaturalist.org/observations/42660893
356	Emberiza spodocephala	Black-faced Bunting	2021/2/8	1	0	25.26169016	121.62010930	https://www.inaturalist.org/observations/69298960
357	Emberiza spodocephala	Black-faced Bunting	2022/4/18	2	0	25.96788494	119.93370400	https://www.inaturalist.org/observations/115350650
358	Emberiza spodocephala	Black-faced Bunting	2022/4/18	2	0	25.96831285	119.93341570	https://www.inaturalist.org/observations/115350652
359	Emberiza spodocephala	Black-faced Bunting	2022/4/18	2	0	25.97014400	119.93332600	https://www.inaturalist.org/observations/115350644
360	Emberiza spodocephala	Black-faced Bunting	2014/3/29	0	0	26.15259872	119.28760990	https://www.inaturalist.org/observations/39030440
361	Emberiza spodocephala	Black-faced Bunting	2021/12/12	1	0	26.15677600	119.95203900	https://www.inaturalist.org/observations/103087341
362	Emberiza spodocephala	Black-faced Bunting	2018/4/29	1	0	26.15695553	119.95160670	https://www.inaturalist.org/observations/56821672
363	Emberiza spodocephala	Black-faced Bunting	2019/4/29	1	0	26.21612500	119.97563830	https://www.inaturalist.org/observations/24835024
364	Emberiza spodocephala	Black-faced Bunting	2019/4/29	1	0	26.21612500	119.97563830	https://www.inaturalist.org/observations/24835025
365	Emberiza spodocephala	Black-faced Bunting	2019/4/29	1	0	26.21612500	119.97563830	https://www.inaturalist.org/observations/24835031
366	Emberiza spodocephala	Black-faced Bunting	2019/4/29	1	0	26.21612500	119.97563830	https://www.inaturalist.org/observations/24835035
367	Emberiza spodocephala	Black-faced Bunting	2019/4/29	1	0	26.21612500	119.97563830	https://www.inaturalist.org/observations/24835036

368	Emberiza spodocephala	Black-faced Bunting	2019/4/28	1	0	26.21655333	119.97501670	https://www.inaturalist.org/observations/24820025
369	Emberiza spodocephala	Black-faced Bunting	2019/4/29	1	0	26.21710333	119.97473500	https://www.inaturalist.org/observations/24835012
370	Emberiza spodocephala	Black-faced Bunting	2019/4/28	2	0	26.21719000	119.97513830	https://www.inaturalist.org/observations/24820032
371	Emberiza spodocephala	Black-faced Bunting	2019/4/29	1	0	26.21719000	119.97520000	https://www.inaturalist.org/observations/24835018
372	Emberiza spodocephala spodocephala	Black-faced Bunting	2019/4/28	1	0	26.21722000	119.97514000	https://www.inaturalist.org/observations/24820034
373	Emberiza spodocephala	Black-faced Bunting	2015/4/5	1	0	26.36967470	120.49624170	https://www.inaturalist.org/observations/57625625
374	Emberiza spodocephala	Black-faced Bunting	2017/4/23	1	0	26.37148800	120.48603900	https://www.inaturalist.org/observations/15321017
375	Emberiza spodocephala	Black-faced Bunting	2022/4/22	1	0	26.42342173	106.36952590	https://www.inaturalist.org/observations/112481382
376	Emberiza spodocephala	Black-faced Bunting	2022/4/22	2	0	26.42342173	106.36952590	https://www.inaturalist.org/observations/112481405
377	Emberiza spodocephala	Black-faced Bunting	2021/5/21	1	0	26.44083862	106.48329400	https://www.inaturalist.org/observations/80365494
378	Emberiza spodocephala	Black-faced Bunting	2020/4/28	1	0	26.44292183	106.67568310	https://www.inaturalist.org/observations/44117158
379	Emberiza spodocephala	Black-faced Bunting	2020/7/15	1	0	26.46259214	106.67465200	https://www.inaturalist.org/observations/53155162
380	Emberiza spodocephala	Black-faced Bunting	2021/4/2	1	0	26.46619599	106.67869760	https://www.inaturalist.org/observations/74017562
381	Emberiza spodocephala	Black-faced Bunting	2021/4/2	1	0	26.46619599	106.67869760	https://www.inaturalist.org/observations/74017570
382	Emberiza spodocephala	Black-faced Bunting	2021/4/16	1	0	26.46619599	106.67869760	https://www.inaturalist.org/observations/74530128
383	Emberiza spodocephala	Black-faced Bunting	2021/4/16	1	0	26.46619599	106.67869760	https://www.inaturalist.org/observations/74530158
384	Emberiza spodocephala	Black-faced Bunting	2021/4/23	0	0	26.46619599	106.67869760	https://www.inaturalist.org/observations/75181123
385	Emberiza spodocephala	Black-faced Bunting	2021/4/23	1	0	26.46619599	106.67869760	https://www.inaturalist.org/observations/75181128
386	Emberiza spodocephala	Black-faced Bunting	2021/5/8	1	0	26.46619599	106.67869760	https://www.inaturalist.org/observations/78349369
387	Emberiza spodocephala	Black-faced Bunting	2021/9/3	2	0	26.46619599	106.67869760	https://www.inaturalist.org/observations/94074823
388	Emberiza spodocephala	Black-faced Bunting	2022/4/4	1	0	26.46619599	106.67869760	https://www.inaturalist.org/observations/110573711
389	Emberiza spodocephala	Black-faced Bunting	2022/4/14	1	0	26.46619599	106.67869760	https://www.inaturalist.org/observations/112055220
390	Emberiza spodocephala	Black-faced Bunting	2022/2/11	1	0	26.50934121	106.64975520	https://www.inaturalist.org/observations/108662408

391	Emberiza spodocephala	Black-faced Bunting	2022/2/12	1	0	26.50934121	106.64975520	https://www.inaturalist.org/observations/108779840
392	Emberiza spodocephala	Black-faced Bunting	2022/4/11	1	0	26.50934121	106.64975520	https://www.inaturalist.org/observations/112045231
393	Emberiza spodocephala	Black-faced Bunting	2022/5/6	1	0	26.50934121	106.64975520	https://www.inaturalist.org/observations/117731333
394	Emberiza spodocephala	Black-faced Bunting	2022/6/9	1	0	26.50934121	106.64975520	https://www.inaturalist.org/observations/125022333
395	Emberiza spodocephala	Black-faced Bunting	2015/3/10	2	0	26.66259870	94.35360990	https://www.inaturalist.org/observations/58330051
396	Emberiza spodocephala	Black-faced Bunting	2022/1/22	1	0	26.84449356	104.24263370	https://www.inaturalist.org/observations/105712655
397	Emberiza spodocephala	Black-faced Bunting	2022/1/22	0	0	26.84449356	104.24263370	https://www.inaturalist.org/observations/105712682
398	Emberiza spodocephala	Black-faced Bunting	2022/1/22	1	0	26.84449356	104.24263370	https://www.inaturalist.org/observations/105712686
399	Emberiza spodocephala	Black-faced Bunting	2022/1/23	2	0	26.84449356	104.24263370	https://www.inaturalist.org/observations/105758107
400	Emberiza spodocephala	Black-faced Bunting	2020/4/1	1	0	26.92666766	112.61178700	https://www.inaturalist.org/observations/43062166
401	Emberiza spodocephala	Black-faced Bunting	2021/3/3	1	0	27.57563090	95.39526400	https://www.inaturalist.org/observations/101927042
402	Emberiza spodocephala	Black-faced Bunting	2016/12/18	1	0	27.57563090	95.39526400	https://www.inaturalist.org/observations/106175140
403	Emberiza spodocephala	Black-faced Bunting	2016/4/17	1	0	27.57886829	95.39174592	https://www.inaturalist.org/observations/19516787
404	Emberiza spodocephala	Black-faced Bunting	2016/12/18	1	0	27.58080467	95.39286074	https://www.inaturalist.org/observations/106175149
405	Emberiza spodocephala	Black-faced Bunting	2018/12/30	2	0	28.06598806	95.71248806	https://www.inaturalist.org/observations/103046272
406	Emberiza spodocephala	Black-faced Bunting	2022/2/14	0	0	28.10095799	112.98501720	https://www.inaturalist.org/observations/106739736
407	Emberiza spodocephala	Black-faced Bunting	2022/2/22	1	0	28.12676842	112.92630950	https://www.inaturalist.org/observations/107551041
408	Emberiza spodocephala	Black-faced Bunting	2022/2/22	1	0	28.12676842	112.92630950	https://www.inaturalist.org/observations/107551042
409	Emberiza spodocephala	Black-faced Bunting	2022/2/27	2	0	28.45137800	119.92535400	https://www.inaturalist.org/observations/136556284
410	Emberiza spodocephala	Black-faced Bunting	2021/4/16	2	0	28.79970644	119.07782430	https://www.inaturalist.org/observations/74818469
411	Emberiza spodocephala	Black-faced Bunting	2021/4/16	1	0	28.79976333	119.07779500	https://www.inaturalist.org/observations/74820256
412	Emberiza spodocephala	Black-faced Bunting	2017/12/14	1	0	28.86685762	116.40463770	https://www.inaturalist.org/observations/10413753
413	Emberiza spodocephala	Black-faced Bunting	2021/12/18	1	0	29.07812000	119.64759000	https://www.inaturalist.org/observations/119071490

414	Emberiza spodocephala	Black-faced Bunting	2005/12/10	1	0	29.24808500	117.86179700	https://www.inaturalist.org/observations/66185296
415	Emberiza spodocephala	Black-faced Bunting	2019/1/11	1	0	29.24883400	117.85029600	https://www.inaturalist.org/observations/34113740
416	Emberiza spodocephala	Black-faced Bunting	2019/1/2	1	0	29.78778924	115.67092840	https://www.inaturalist.org/observations/36380616
417	Emberiza spodocephala	Black-faced Bunting	2021/3/29	1	0	29.79924456	121.56018250	https://www.inaturalist.org/observations/72518913
418	Emberiza spodocephala	Black-faced Bunting	2021/3/6	1	0	29.79929053	121.56345520	https://www.inaturalist.org/observations/70651365
419	Emberiza spodocephala	Black-faced Bunting	2021/4/10	1	0	29.79954248	121.56407780	https://www.inaturalist.org/observations/73593488
420	Emberiza spodocephala	Black-faced Bunting	2021/3/20	1	0	29.80084153	121.56173550	https://www.inaturalist.org/observations/71604484
421	Emberiza spodocephala	Black-faced Bunting	2021/3/21	1	0	29.80108854	121.56185930	https://www.inaturalist.org/observations/71673588
422	Emberiza spodocephala	Black-faced Bunting	2021/1/29	2	0	29.80166081	121.56172410	https://www.inaturalist.org/observations/69806297
423	Emberiza spodocephala	Black-faced Bunting	2021/3/14	1	0	29.80201808	121.56351920	https://www.inaturalist.org/observations/71201803
424	Emberiza spodocephala	Black-faced Bunting	2021/11/14	2	0	29.81181746	121.53410980	https://www.inaturalist.org/observations/101296732
425	Emberiza spodocephala	Black-faced Bunting	2021/5/2	1	0	29.81215928	121.54695590	https://www.inaturalist.org/observations/76394881
426	Emberiza spodocephala	Black-faced Bunting	2021/11/3	1	0	29.81235884	121.52950110	https://www.inaturalist.org/observations/100187007
427	Emberiza spodocephala	Black-faced Bunting	2021/3/15	2	0	29.81281294	121.53784740	https://www.inaturalist.org/observations/71297546
428	Emberiza spodocephala	Black-faced Bunting	2021/12/9	2	0	30.08487167	116.70894170	https://www.inaturalist.org/observations/104350924
429	Emberiza spodocephala	Black-faced Bunting	2017/5/22	2	0	30.13161700	103.18451600	https://www.inaturalist.org/observations/36149383
430	Emberiza spodocephala	Black-faced Bunting	2022/2/3	1	0	30.20844227	120.14087790	https://www.inaturalist.org/observations/106355830
431	Emberiza spodocephala	Black-faced Bunting	2022/2/24	1	0	30.20844227	120.14087790	https://www.inaturalist.org/observations/107551061
432	Emberiza spodocephala	Black-faced Bunting	2021/3/29	2	0	30.20971277	119.33037090	https://www.inaturalist.org/observations/72599513
433	Emberiza spodocephala	Black-faced Bunting	2021/3/15	1	0	30.23679364	119.75188260	https://www.inaturalist.org/observations/73608370
434	Emberiza spodocephala	Black-faced Bunting	2021/2/20	1	0	30.23736825	119.75422160	https://www.inaturalist.org/observations/69875868
435	Emberiza spodocephala	Black-faced Bunting	2021/1/4	1	0	30.23798011	119.76158130	https://www.inaturalist.org/observations/68362667
436	Emberiza spodocephala	Black-faced Bunting	2021/1/29	1	0	30.23798011	119.76158130	https://www.inaturalist.org/observations/68851525

437	Emberiza spodocephala	Black-faced Bunting	2021/2/3	1	0	30.23798011	119.76158130	https://www.inaturalist.org/observations/69033880
438	Emberiza spodocephala	Black-faced Bunting	2022/1/3	1	0	30.23864740	119.75016590	https://www.inaturalist.org/observations/104278488
439	Emberiza spodocephala	Black-faced Bunting	2022/1/3	1	0	30.23864740	119.75016590	https://www.inaturalist.org/observations/104278532
440	Emberiza spodocephala	Black-faced Bunting	2022/1/3	1	0	30.23864740	119.75016590	https://www.inaturalist.org/observations/104278665
441	Emberiza spodocephala	Black-faced Bunting	2022/1/11	1	0	30.23864740	119.75016590	https://www.inaturalist.org/observations/104852811
442	Emberiza spodocephala	Black-faced Bunting	2022/1/11	1	0	30.23864740	119.75016590	https://www.inaturalist.org/observations/104852814
443	Emberiza spodocephala	Black-faced Bunting	2022/1/21	1	0	30.23864740	119.75016590	https://www.inaturalist.org/observations/105413452
444	Emberiza spodocephala	Black-faced Bunting	2022/2/5	1	0	30.23864740	119.75016590	https://www.inaturalist.org/observations/106368231
445	Emberiza spodocephala	Black-faced Bunting	2022/3/8	1	0	30.23864740	119.75016590	https://www.inaturalist.org/observations/108295547
446	Emberiza spodocephala	Black-faced Bunting	2022/3/29	2	0	30.23864740	119.75016590	https://www.inaturalist.org/observations/111363788
447	Emberiza spodocephala	Black-faced Bunting	2021/12/20	1	0	30.23900000	119.76300000	https://www.inaturalist.org/observations/103575874
448	Emberiza spodocephala	Black-faced Bunting	2021/4/19	1	0	30.23985140	120.12789460	https://www.inaturalist.org/observations/75434734
449	Emberiza spodocephala	Black-faced Bunting	2022/4/4	1	0	30.23985140	120.12789460	https://www.inaturalist.org/observations/112541064
450	Emberiza spodocephala	Black-faced Bunting	2022/2/20	1	0	30.25100000	120.11300000	https://www.inaturalist.org/observations/107103749
451	Emberiza spodocephala	Black-faced Bunting	2022/1/7	1	0	30.25100000	120.11300000	https://www.inaturalist.org/observations/131045139
452	Emberiza spodocephala	Black-faced Bunting	2021/10/25	2	0	30.25140029	119.90746640	https://www.inaturalist.org/observations/99610871
453	Emberiza spodocephala	Black-faced Bunting	2021/10/25	1	0	30.25140029	119.90746640	https://www.inaturalist.org/observations/99610880
454	Emberiza spodocephala	Black-faced Bunting	2021/3/12	1	0	30.25287500	120.11697780	https://www.inaturalist.org/observations/73090701
455	Emberiza spodocephala	Black-faced Bunting	2021/3/12	1	0	30.25287500	120.11697780	https://www.inaturalist.org/observations/73090702
456	Emberiza spodocephala	Black-faced Bunting	2022/2/19	1	0	30.25351764	120.11996840	https://www.inaturalist.org/observations/107103928
457	Emberiza spodocephala	Black-faced Bunting	2021/11/19	1	0	30.25418228	120.12014610	https://www.inaturalist.org/observations/101611137
458	Emberiza spodocephala	Black-faced Bunting	2022/2/19	1	0	30.25418228	120.12014610	https://www.inaturalist.org/observations/107367606
459	Emberiza spodocephala	Black-faced Bunting	2022/2/20	1	0	30.25418228	120.12014610	https://www.inaturalist.org/observations/107367647

460	Emberiza spodocephala	Black-faced Bunting	2022/2/5	1	0	30.25472961	119.91609230	https://www.inaturalist.org/observations/106368216
461	Emberiza spodocephala	Black-faced Bunting	2022/3/15	0	0	30.25472961	119.91609230	https://www.inaturalist.org/observations/108719643
462	Emberiza spodocephala	Black-faced Bunting	2022/3/15	1	0	30.25472961	119.91609230	https://www.inaturalist.org/observations/108719646
463	Emberiza spodocephala	Black-faced Bunting	2022/3/29	1	0	30.25472961	119.91609230	https://www.inaturalist.org/observations/110656218
464	Emberiza spodocephala	Black-faced Bunting	2021/10/25	2	0	30.25722790	119.91305830	https://www.inaturalist.org/observations/99610893
465	Emberiza spodocephala	Black-faced Bunting	2022/1/17	1	0	30.25933000	119.91529000	https://www.inaturalist.org/observations/107363964
466	Emberiza spodocephala	Black-faced Bunting	2021/10/25	2	0	30.26404689	119.91855860	https://www.inaturalist.org/observations/99610897
467	Emberiza spodocephala	Black-faced Bunting	2021/10/25	2	0	30.26404689	119.91855860	https://www.inaturalist.org/observations/99610898
468	Emberiza spodocephala	Black-faced Bunting	2021/10/25	2	0	30.26404689	119.91855860	https://www.inaturalist.org/observations/99610900
469	Emberiza spodocephala	Black-faced Bunting	2021/4/6	1	0	30.26433033	119.93016870	https://www.inaturalist.org/observations/75429046
470	Emberiza spodocephala	Black-faced Bunting	2022/1/17	1	0	30.26433033	119.93016870	https://www.inaturalist.org/observations/105213233
471	Emberiza spodocephala	Black-faced Bunting	2021/3/18	1	0	30.30204987	120.08709080	https://www.inaturalist.org/observations/71610771
472	Emberiza spodocephala	Black-faced Bunting	2021/1/16	1	0	30.31777220	113.83765220	https://www.inaturalist.org/observations/120634659
473	Emberiza spodocephala	Black-faced Bunting	2022/1/17	1	0	30.32795000	119.90878000	https://www.inaturalist.org/observations/106849668
474	Emberiza spodocephala	Black-faced Bunting	2022/1/17	1	0	30.33300005	119.92555030	https://www.inaturalist.org/observations/105207230
475	Emberiza spodocephala	Black-faced Bunting	2021/12/15	1	0	30.34818400	119.94872400	https://www.inaturalist.org/observations/103185122
476	Emberiza spodocephala	Black-faced Bunting	2021/12/15	1	0	30.34818400	119.94872400	https://www.inaturalist.org/observations/103185123
477	Emberiza spodocephala	Black-faced Bunting	2021/12/18	1	0	30.34818400	119.94872400	https://www.inaturalist.org/observations/103516327
478	Emberiza spodocephala	Black-faced Bunting	2022/1/17	1	0	30.34818400	119.94872400	https://www.inaturalist.org/observations/106849321
479	Emberiza spodocephala	Black-faced Bunting	2022/1/17	1	0	30.34818400	119.94872400	https://www.inaturalist.org/observations/106849322
480	Emberiza spodocephala	Black-faced Bunting	2022/1/17	1	0	30.34818400	119.94872400	https://www.inaturalist.org/observations/106849323
481	Emberiza spodocephala	Black-faced Bunting	2022/2/14	1	0	30.34818400	119.94872400	https://www.inaturalist.org/observations/107364061
482	Emberiza spodocephala	Black-faced Bunting	2021/12/6	1	0	30.35211150	119.93181640	https://www.inaturalist.org/observations/102643310

483	Emberiza spodocephala	Black-faced Bunting	2022/1/17	1	0	30.35211150	119.93181640	https://www.inaturalist.org/observations/105207195
484	Emberiza spodocephala	Black-faced Bunting	2022/1/17	1	0	30.35211150	119.93181640	https://www.inaturalist.org/observations/105207198
485	Emberiza spodocephala	Black-faced Bunting	2022/1/17	1	0	30.35211150	119.93181640	https://www.inaturalist.org/observations/105207217
486	Emberiza spodocephala	Black-faced Bunting	2022/1/17	1	0	30.35211150	119.93181640	https://www.inaturalist.org/observations/105207222
487	Emberiza spodocephala	Black-faced Bunting	2022/1/17	1	0	30.35211150	119.93181640	https://www.inaturalist.org/observations/105207225
488	Emberiza spodocephala	Black-faced Bunting	2022/4/14	1	0	30.38425336	119.98458850	https://www.inaturalist.org/observations/111401833
489	Emberiza spodocephala	Black-faced Bunting	2019/7/30	2	0	30.46179632	109.17712830	https://www.inaturalist.org/observations/30539557
490	Emberiza spodocephala	Black-faced Bunting	2020/1/6	1	0	30.47192559	114.36207730	https://www.inaturalist.org/observations/109638779
491	Emberiza spodocephala	Black-faced Bunting	2019/12/2	1	0	30.47336454	114.36214520	https://www.inaturalist.org/observations/109636534
492	Emberiza spodocephala	Black-faced Bunting	2021/3/4	1	0	30.51394500	120.05323800	https://www.inaturalist.org/observations/98361718
493	Emberiza spodocephala	Black-faced Bunting	2021/3/4	2	0	30.51989538	120.05109640	https://www.inaturalist.org/observations/70646652
494	Emberiza spodocephala	Black-faced Bunting	2021/3/4	1	0	30.52094886	120.05652520	https://www.inaturalist.org/observations/70646644
495	Emberiza spodocephala	Black-faced Bunting	2022/3/2	1	0	30.60786246	119.43257910	https://www.inaturalist.org/observations/107817580
496	Emberiza spodocephala	Black-faced Bunting	2022/3/2	1	0	30.60900400	119.46580960	https://www.inaturalist.org/observations/108643522
497	Emberiza spodocephala	Black-faced Bunting	2020/4/22	1	0	30.85457954	120.48811510	https://www.inaturalist.org/observations/42830277
498	Emberiza spodocephala	Black-faced Bunting	2020/12/30	1	0	30.90413000	119.60569200	https://www.inaturalist.org/observations/67907339
499	Emberiza spodocephala	Black-faced Bunting	2016/11/24	1	0	31.02448241	121.44864030	https://www.inaturalist.org/observations/19692352
500	Emberiza spodocephala	Black-faced Bunting	2017/11/1	1	0	31.04646308	121.22481820	https://www.inaturalist.org/observations/8641956
501	Emberiza spodocephala	Black-faced Bunting	2017/11/1	1	0	31.04665835	121.22464230	https://www.inaturalist.org/observations/8641959
502	Emberiza spodocephala	Black-faced Bunting	2017/10/23	1	0	31.07460200	121.18206600	https://www.inaturalist.org/observations/8554991
503	Emberiza spodocephala	Black-faced Bunting	2022/2/18	1	0	31.07612998	121.17615160	https://www.inaturalist.org/observations/108301797
504	Emberiza spodocephala	Black-faced Bunting	2014/3/29	2	0	31.14697656	121.44440080	https://www.inaturalist.org/observations/93592614
505	Emberiza spodocephala	Black-faced Bunting	2022/4/2	2	0	31.18613948	120.96015980	https://www.inaturalist.org/observations/110160372

506	Emberiza spodocephala	Black-faced Bunting	2022/3/4	1	0	31.21213400	121.54372400	https://www.inaturalist.org/observations/109542422
507	Emberiza spodocephala personata	Black-faced Bunting	2006/3/24	0	0	31.21522181	121.55213990	https://www.inaturalist.org/observations/91612183
508	Emberiza spodocephala	Black-faced Bunting	2019/1/19	1	0	31.21539400	121.55215700	https://www.inaturalist.org/observations/19726558
509	Emberiza spodocephala	Black-faced Bunting	2021/2/3	1	0	31.21539400	121.55215700	https://www.inaturalist.org/observations/68994999
510	Emberiza spodocephala	Black-faced Bunting	2021/2/9	1	0	31.21539400	121.55215700	https://www.inaturalist.org/observations/69301598
511	Emberiza spodocephala	Black-faced Bunting	2021/4/16	1	0	31.21539400	121.55215700	https://www.inaturalist.org/observations/74239847
512	Emberiza spodocephala	Black-faced Bunting	2021/11/3	2	0	31.21539400	121.55215700	https://www.inaturalist.org/observations/101882591
513	Emberiza spodocephala	Black-faced Bunting	2022/1/8	1	0	31.21539400	121.55215700	https://www.inaturalist.org/observations/104705264
514	Emberiza spodocephala	Black-faced Bunting	2022/2/10	1	0	31.21637304	121.55577290	https://www.inaturalist.org/observations/106607997
515	Emberiza spodocephala	Black-faced Bunting	2022/2/10	1	0	31.21665154	121.55572730	https://www.inaturalist.org/observations/106607874
516	Emberiza spodocephala	Black-faced Bunting	2022/2/9	1	0	31.21716974	121.55518890	https://www.inaturalist.org/observations/106604674
517	Emberiza spodocephala	Black-faced Bunting	2022/2/24	1	0	31.36246888	120.68288700	https://www.inaturalist.org/observations/107445915
518	Emberiza spodocephala	Black-faced Bunting	2018/2/1	1	0	31.38484070	121.52915000	https://www.inaturalist.org/observations/9691161
519	Emberiza spodocephala	Black-faced Bunting	2019/4/10	2	0	31.41543296	120.89790320	https://www.inaturalist.org/observations/22271795
520	Emberiza spodocephala	Black-faced Bunting	2021/1/11	1	0	31.45706529	120.34806290	https://www.inaturalist.org/observations/67900590
521	Emberiza spodocephala	Black-faced Bunting	2021/11/28	1	0	31.49410828	121.34581120	https://www.inaturalist.org/observations/102227672
522	Emberiza spodocephala	Black-faced Bunting	2021/5/3	1	0	31.64968004	119.21509050	https://www.inaturalist.org/observations/77310272
523	Emberiza spodocephala	Black-faced Bunting	2021/5/2	1	0	31.65490872	119.22105170	https://www.inaturalist.org/observations/77309625
524	Emberiza spodocephala	Black-faced Bunting	2020/4/21	1	0	31.70969776	119.57638730	https://www.inaturalist.org/observations/42756272
525	Emberiza spodocephala	Black-faced Bunting	2021/2/12	1	0	31.90253115	118.91454360	https://www.inaturalist.org/observations/101876193
526	Emberiza spodocephala	Black-faced Bunting	2022/2/19	1	0	32.13896500	118.52879170	https://www.inaturalist.org/observations/112548346
527	Emberiza spodocephala	Black-faced Bunting	2022/4/30	1	0	32.20946833	119.01806830	https://www.inaturalist.org/observations/114084117
528	Emberiza spodocephala	Black-faced Bunting	2022/4/30	0	0	32.21337833	119.01424500	https://www.inaturalist.org/observations/114084176

529	Emberiza spodocephala	Black-faced Bunting	2017/12/2	1	0	33.58902600	120.50139400	https://www.inaturalist.org/observations/10232413
530	Emberiza spodocephala	Black-faced Bunting	2019/1/8	1	0	33.60056600	120.50859000	https://www.inaturalist.org/observations/34012686
531	Emberiza spodocephala	Black-faced Bunting	2021/12/20	1	0	34.81488797	113.70426350	https://www.inaturalist.org/observations/103410858
532	Emberiza spodocephala	Black-faced Bunting	2021/10/29	1	0	34.81809000	113.70654500	https://www.inaturalist.org/observations/99704407
533	Emberiza spodocephala	Black-faced Bunting	2021/1/10	1	0	34.82312167	113.70414500	https://www.inaturalist.org/observations/67841408
534	Emberiza spodocephala	Black-faced Bunting	2021/1/13	1	0	34.82332500	113.70526330	https://www.inaturalist.org/observations/68029829
535	Emberiza spodocephala	Black-faced Bunting	2021/1/10	2	0	34.82345167	113.70284170	https://www.inaturalist.org/observations/67841401
536	Emberiza spodocephala	Black-faced Bunting	2021/12/20	1	0	34.82432500	113.70668000	https://www.inaturalist.org/observations/103443263
537	Emberiza spodocephala	Black-faced Bunting	2021/1/13	1	0	34.82465000	113.70965000	https://www.inaturalist.org/observations/68029852
538	Emberiza spodocephala	Black-faced Bunting	2019/4/11	0	0	36.06284000	120.34510000	https://www.inaturalist.org/observations/134868743
539	Emberiza spodocephala	Black-faced Bunting	2019/4/11	1	0	36.06395000	120.34636000	https://www.inaturalist.org/observations/134868681
540	Emberiza spodocephala	Black-faced Bunting	2022/4/8	1	0	36.15036625	120.36829030	https://www.inaturalist.org/observations/111034974
541	Emberiza spodocephala	Black-faced Bunting	2018/10/14	1	0	36.15932000	120.50131000	https://www.inaturalist.org/observations/133339662
542	Emberiza spodocephala	Black-faced Bunting	2018/10/14	1	0	36.15939000	120.50127000	https://www.inaturalist.org/observations/133339664
543	Emberiza spodocephala	Black-faced Bunting	2018/10/14	1	0	36.15965000	120.50117000	https://www.inaturalist.org/observations/133339897
544	Emberiza spodocephala	Black-faced Bunting	2020/4/16	1	0	36.17858167	120.39747670	https://www.inaturalist.org/observations/42467953
545	Emberiza spodocephala	Black-faced Bunting	2019/10/23	0	0	36.36735600	120.68541500	https://www.inaturalist.org/observations/107476095
546	Emberiza spodocephala	Black-faced Bunting	2020/9/30	0	0	36.36737600	120.68514300	https://www.inaturalist.org/observations/105300874
547	Emberiza spodocephala	Black-faced Bunting	2019/4/14	0	0	36.36854000	120.67865000	https://www.inaturalist.org/observations/134872931
548	Emberiza spodocephala	Black-faced Bunting	2021/4/18	1	0	36.37044600	120.68158000	https://www.inaturalist.org/observations/105410258
549	Emberiza spodocephala	Black-faced Bunting	2020/10/5	0	0	36.37159200	120.67989600	https://www.inaturalist.org/observations/105308925
550	Emberiza spodocephala	Black-faced Bunting	2021/1/1	1	0	36.37511100	120.68414300	https://www.inaturalist.org/observations/105354154
551	Emberiza spodocephala	Black-faced Bunting	2022/5/12	1	0	39.88382680	116.36450740	https://www.inaturalist.org/observations/125387988

552	Emberiza spodocephala	Black-faced Bunting	2021/10/6	1	0	39.97891990	119.77606000	https://www.inaturalist.org/observations/123573408
553	Emberiza spodocephala	Black-faced Bunting	2022/10/1	1	0	40.01354379	116.37954950	https://www.inaturalist.org/observations/137158747
554	Emberiza spodocephala	Black-faced Bunting	2022/1/7	1	0	40.01643167	116.37702670	https://www.inaturalist.org/observations/125272413
555	Emberiza spodocephala	Black-faced Bunting	2022/1/10	1	0	40.01821529	116.38538950	https://www.inaturalist.org/observations/122024980
556	Emberiza spodocephala	Black-faced Bunting	2022/5/12	1	0	40.07971223	116.47367790	https://www.inaturalist.org/observations/116787262
557	Emberiza spodocephala	Black-faced Bunting	2019/5/19	3	0	40.11598500	116.70157170	https://www.inaturalist.org/observations/25624786
558	Emberiza spodocephala	Black-faced Bunting	2019/11/2	1	0	40.12162833	116.69463670	https://www.inaturalist.org/observations/35193830
559	Emberiza spodocephala	Black-faced Bunting	2022/5/7	1	0	40.13797161	116.31906060	https://www.inaturalist.org/observations/117040051
560	Emberiza spodocephala	Black-faced Bunting	2022/5/7	1	0	40.13810754	116.31962820	https://www.inaturalist.org/observations/117039985
561	Emberiza spodocephala	Black-faced Bunting	2021/10/5	2	0	40.18056583	116.68115900	https://www.inaturalist.org/observations/100235781
562	Emberiza spodocephala	Black-faced Bunting	2021/10/5	2	0	40.18056583	116.68115900	https://www.inaturalist.org/observations/100235782
563	Emberiza spodocephala	Black-faced Bunting	2021/10/5	1	0	40.18056583	116.68115900	https://www.inaturalist.org/observations/100235787
564	Emberiza spodocephala	Black-faced Bunting	2015/4/14	1	0	42.70462190	123.19261430	https://www.inaturalist.org/observations/64268886
565	Emberiza spodocephala	Black-faced Bunting	2013/6/5	1	0	45.40412771	127.66336200	https://www.inaturalist.org/observations/18903570
566	Emberiza spodocephala	Black-faced Bunting	2007/5/21	1	0	47.02839700	129.02079300	https://www.inaturalist.org/observations/65334527
567	Emberiza spodocephala	Black-faced Bunting	2006/6/1	1	0	47.02839700	129.02079300	https://www.inaturalist.org/observations/65470647
568	Emberiza spodocephala	Black-faced Bunting	2005/7/1	1	0	47.04063990	129.10726990	https://www.inaturalist.org/observations/110660117

No	scientific name	common name	date	NIA	NID	latitude	longitude	url
1	Halcyon coromanda	Ruddy Kingfisher	2022/2/21	5	0	11.79615407	92.71869267	https://www.inaturalist.org/observations/107877471
2	Halcyon coromanda	Ruddy Kingfisher	2020/2/22	1	0	11.83116810	92.65864010	https://www.inaturalist.org/observations/102092125
3	Halcyon coromanda	Ruddy Kingfisher	2017/12/13	5	0	11.84390466	92.72980121	https://www.inaturalist.org/observations/9558297
4	Halcyon coromanda	Ruddy Kingfisher	2019/10/15	3	0	22.75834493	121.14518980	https://www.inaturalist.org/observations/44369220
5	Halcyon coromanda	Ruddy Kingfisher	2018/4/28	4	0	22.80673652	120.34529760	https://www.inaturalist.org/observations/14162966
6	Halcyon coromanda	Ruddy Kingfisher	2018/4/28	3	0	22.99182678	120.22505330	https://www.inaturalist.org/observations/14538315
7	Halcyon coromanda	Ruddy Kingfisher	2013/4/3	3	0	23.72642984	120.62768110	https://www.inaturalist.org/observations/19485348
8	Halcyon coromanda	Ruddy Kingfisher	2005/4/30	4	0	23.74206450	120.62574230	https://www.inaturalist.org/observations/13697374
9	Halcyon coromanda	Ruddy Kingfisher	2020/4/28	1	0	23.94987953	121.48016720	https://www.inaturalist.org/observations/44125258
10	Halcyon coromanda	Ruddy Kingfisher	2021/3/22	1	0	24.08236403	120.60645340	https://www.inaturalist.org/observations/72250684
11	Halcyon coromanda	Ruddy Kingfisher	2022/5/2	2	0	24.61194167	121.73056110	https://www.inaturalist.org/observations/116994232
12	Halcyon coromanda	Ruddy Kingfisher	2020/5/16	2	0	24.65471198	121.65404980	https://www.inaturalist.org/observations/46048706
13	Halcyon coromanda	Ruddy Kingfisher	2022/5/2	3	0	24.68038337	121.68187050	https://www.inaturalist.org/observations/115576219
14	Halcyon coromanda	Ruddy Kingfisher	2020/10/25	2	0	24.68383866	121.77445330	https://www.inaturalist.org/observations/63490826
15	Halcyon coromanda	Ruddy Kingfisher	2019/10/8	3	0	25.03253921	121.50968990	https://www.inaturalist.org/observations/34107701
16	Halcyon coromanda	Ruddy Kingfisher	2021/9/26	2	0	25.06072217	121.49875500	https://www.inaturalist.org/observations/96212541
17	Halcyon coromanda	Ruddy Kingfisher	2021/5/25	1	0	25.15186200	121.49077000	https://www.inaturalist.org/observations/80176009
18	Halcyon coromanda	Ruddy Kingfisher	2022/4/22	5	0	26.64733550	89.54833710	https://www.inaturalist.org/observations/127829143
19	Halcyon coromanda	Ruddy Kingfisher	2022/5/10	3	0	26.75635740	88.79747930	https://www.inaturalist.org/observations/118380497
20	Halcyon coromanda	Ruddy Kingfisher	2022/5/10	6	0	26.76176968	88.83392036	https://www.inaturalist.org/observations/119778630
21	Halcyon coromanda	Ruddy Kingfisher	2022/5/28	4	0	26.85108540	88.85510190	https://www.inaturalist.org/observations/119361493
22	Halcyon coromanda	Ruddy Kingfisher	2022/4/21	3	0	27.09835550	92.81782910	https://www.inaturalist.org/observations/115542967

23	Halcyon coromanda	Ruddy Kingfisher	2022/4/23	4	0	27.09835550	92.81782910	https://www.inaturalist.org/observations/115543654
24	Halcyon coromanda	Ruddy Kingfisher	2017/5/1	4	0	27.34694687	95.50881958	https://www.inaturalist.org/observations/6271982

No	scientific name	common name	date	NIA	NID	latitude	longitude	url
1	Pitta nympha	Fairy Pitta	2022/9/16	1	0	22.18382150	113.52210480	https://www.inaturalist.org/observations/136389590
2	Pitta nympha	Fairy Pitta	2020/9/16	1	0	22.20078448	114.15539130	https://www.inaturalist.org/observations/118553086
3	Pitta nympha	Fairy Pitta	2020/9/16	1	0	22.20416479	114.05716170	https://www.inaturalist.org/observations/110850976
4	Pitta nympha	Fairy Pitta	2020/10/12	3	0	22.21644179	114.18454080	https://www.inaturalist.org/observations/62754568
5	Pitta nympha	Fairy Pitta	2020/10/2	4	0	22.27234533	113.41073390	https://www.inaturalist.org/observations/62252790
6	Pitta nympha	Fairy Pitta	2020/10/2	3	0	22.27736192	113.51916830	https://www.inaturalist.org/observations/62252792
7	Pitta nympha	Fairy Pitta	2020/10/1	3	0	22.35307364	113.43537050	https://www.inaturalist.org/observations/61474729
8	Pitta nympha	Fairy Pitta	2022/5/17	4	0	22.36376094	114.30576010	https://www.inaturalist.org/observations/117539579
9	Pitta nympha	Fairy Pitta	2019/10/2	7	0	22.53525823	114.11682390	https://www.inaturalist.org/observations/33739744
10	Pitta nympha	Fairy Pitta	2017/5/23	4	0	22.75163221	120.60252860	https://www.inaturalist.org/observations/14020025
11	Pitta nympha	Fairy Pitta	2018/5/16	4	0	22.78721537	120.73202180	https://www.inaturalist.org/observations/14538312
12	Pitta nympha	Fairy Pitta	2018/5/16	3	0	22.78996524	120.63413060	https://www.inaturalist.org/observations/43326461
13	Pitta nympha	Fairy Pitta	2017/5/23	4	0	22.79605714	120.64745830	https://www.inaturalist.org/observations/14163345
14	Pitta nympha	Fairy Pitta	2014/4/27	2	0	23.01074072	113.26538700	https://www.inaturalist.org/observations/32843872
15	Pitta nympha	Fairy Pitta	2019/5/7	2	0	23.01651805	113.27794720	https://www.inaturalist.org/observations/98606512
16	Pitta nympha	Fairy Pitta	2022/5/15	1	0	23.11886891	120.33251220	https://www.inaturalist.org/observations/117059767
17	Pitta nympha	Fairy Pitta	2022/5/15	1	0	23.17141518	120.21877020	https://www.inaturalist.org/observations/117052198
18	Pitta nympha	Fairy Pitta	2020/6/7	5	0	23.41503871	120.44246670	https://www.inaturalist.org/observations/48749463
19	Pitta nympha	Fairy Pitta	2014/6/4	1	0	23.42574805	111.93653500	https://www.inaturalist.org/observations/33482183
20	Pitta nympha	Fairy Pitta	2007/5/19	5	0	23.55011757	120.20829640	https://www.inaturalist.org/observations/18754337
21	Pitta nympha	Fairy Pitta	2020/6/18	2	0	23.61068274	120.70010270	https://www.inaturalist.org/observations/50373210
22	Pitta nympha	Fairy Pitta	2022/4/28	1	0	23.65397303	120.79219410	https://www.inaturalist.org/observations/113157164

23	Pitta nympha	Fairy Pitta	2020/7/18	3	0	23.68247507	120.76660050	https://www.inaturalist.org/observations/53443969
24	Pitta nympha	Fairy Pitta	2022/4/28	1	0	23.69053526	120.70228510	https://www.inaturalist.org/observations/113160089
25	Pitta nympha	Fairy Pitta	2021/5/6	1	0	23.69974905	121.54322510	https://www.inaturalist.org/observations/77556865
26	Pitta nympha	Fairy Pitta	2020/6/7	3	0	23.70338259	120.81717160	https://www.inaturalist.org/observations/48716434
27	Pitta nympha	Fairy Pitta	2015/7/22	2	0	23.70742676	120.70069170	https://www.inaturalist.org/observations/48617761
28	Pitta nympha	Fairy Pitta	2013/5/29	1	0	23.71673164	120.63632760	https://www.inaturalist.org/observations/38376610
29	Pitta nympha	Fairy Pitta	2021/5/3	2	0	23.73070559	120.74055290	https://www.inaturalist.org/observations/98284196
30	Pitta nympha	Fairy Pitta	2014/5/5	3	0	23.73567506	120.70015870	https://www.inaturalist.org/observations/19484487
31	Pitta nympha	Fairy Pitta	2005/6/4	7	0	23.73915767	120.74921830	https://www.inaturalist.org/observations/13783836
32	Pitta nympha	Fairy Pitta	2015/7/22	3	0	23.76623594	120.71973450	https://www.inaturalist.org/observations/81540293
33	Pitta nympha	Fairy Pitta	2019/4/24	4	0	23.76709362	113.76587330	https://www.inaturalist.org/observations/24716465
34	Pitta nympha	Fairy Pitta	2022/4/30	2	0	23.76900361	120.72147350	https://www.inaturalist.org/observations/113609134
35	Pitta nympha	Fairy Pitta	2013/8/8	5	0	23.79164958	120.61785410	http://www.inaturalist.org/observations/3518419
36	Pitta nympha	Fairy Pitta	2015/7/18	5	0	23.79558836	120.63314610	https://www.inaturalist.org/observations/20070310
37	Pitta nympha	Fairy Pitta	2019/4/19	1	0	23.91012459	120.65397390	https://www.inaturalist.org/observations/22874451
38	Pitta nympha	Fairy Pitta	2022/4/27	1	0	23.94844787	121.46455870	https://www.inaturalist.org/observations/113065962
39	Pitta nympha	Fairy Pitta	2021/6/23	1	0	23.95012854	120.62211240	https://www.inaturalist.org/observations/84141348
40	Pitta nympha	Fairy Pitta	2019/6/19	4	0	24.11753778	121.46619950	https://www.inaturalist.org/observations/28168757
41	Pitta nympha	Fairy Pitta	2022/6/14	2	0	24.61801862	121.78634180	https://www.inaturalist.org/observations/121678530
42	Pitta nympha	Fairy Pitta	2011/7/2	2	0	24.65026756	121.75514490	https://www.inaturalist.org/observations/55872786
43	Pitta nympha	Fairy Pitta	2020/5/31	2	0	24.67109133	121.67128150	https://www.inaturalist.org/observations/48909587
44	Pitta nympha	Fairy Pitta	2019/5/25	5	0	24.73595046	121.18039690	https://www.inaturalist.org/observations/25942021
45	Pitta nympha	Fairy Pitta	2017/6/6	4	0	24.77060256	121.62663370	https://www.inaturalist.org/observations/65250026

46	Pitta nympha	Fairy Pitta	2011/6/26	6	0	24.77689934	121.61073890	https://www.inaturalist.org/observations/83232048
47	Pitta nympha	Fairy Pitta	2017/6/9	5	0	24.77851035	121.68214390	https://www.inaturalist.org/observations/40126214
48	Pitta nympha	Fairy Pitta	2020/5/24	2	0	24.86297876	121.30806500	https://www.inaturalist.org/observations/47103197
49	Pitta nympha	Fairy Pitta	2021/5/21	2	0	24.98580718	121.29844910	https://www.inaturalist.org/observations/79661089
50	Pitta nympha	Fairy Pitta	2011/6/27	3	0	29.22686052	118.09414180	https://www.inaturalist.org/observations/27037787
51	Pitta nympha	Fairy Pitta	2013/5/18	1	0	30.08440191	120.07739470	https://www.inaturalist.org/observations/33482182
52	Pitta nympha	Fairy Pitta	2022/9/3	2	0	31.20653631	121.57776780	https://www.inaturalist.org/observations/133912168
53	Pitta nympha	Fairy Pitta	2021/7/4	0	0	31.66920147	104.41802250	https://www.inaturalist.org/observations/128600559
54	Pitta nympha	Fairy Pitta	2021/5/29	3	0	32.12220463	118.45548840	https://www.inaturalist.org/observations/88350785
55	Pitta nympha	Fairy Pitta	2021/5/28	1	0	32.16120987	118.62771680	https://www.inaturalist.org/observations/115531952

No	scientific name	common name	date	NIA	NID	latitude	longitude	url
1	Spilopelia chinensis	Eastern Spotted Dove	2022/2/4	2	0	5.94673667	80.46399667	https://www.inaturalist.org/observations/106111278
2	Spilopelia chinensis	Eastern Spotted Dove	2019/4/9	6	0	5.94684006	80.63111349	https://www.inaturalist.org/observations/61253530
3	Spilopelia chinensis	Eastern Spotted Dove	2022/1/25	2	0	5.95895500	80.41196333	https://www.inaturalist.org/observations/113957574
4	Spilopelia chinensis	Eastern Spotted Dove	2021/6/18	4	0	5.97667239	80.53462788	https://www.inaturalist.org/observations/84189424
5	Spilopelia chinensis	Eastern Spotted Dove	2017/2/6	5	0	6.00333768	80.34324412	https://www.inaturalist.org/observations/5061582
6	Spilopelia chinensis	Eastern Spotted Dove	2022/2/7	2	0	6.00415278	80.31827500	https://www.inaturalist.org/observations/106307577
7	Spilopelia chinensis	Eastern Spotted Dove	2022/4/14	1	0	6.01166790	80.24819445	https://www.inaturalist.org/observations/111423442
8	Spilopelia chinensis	Eastern Spotted Dove	2019/12/16	6	0	6.01496485	80.24786272	https://www.inaturalist.org/observations/90042026
9	Spilopelia chinensis	Eastern Spotted Dove	2019/12/25	5	0	6.01744658	80.24885964	https://www.inaturalist.org/observations/65523660
10	Spilopelia chinensis	Eastern Spotted Dove	2020/3/3	3	0	6.02690420	80.21496569	https://www.inaturalist.org/observations/39494153
11	Spilopelia chinensis	Eastern Spotted Dove	2017/7/26	5	0	6.03868822	80.21718979	https://www.inaturalist.org/observations/10297257
12	Spilopelia chinensis	Eastern Spotted Dove	2019/3/7	3	0	6.04717777	80.85577803	https://www.inaturalist.org/observations/22137718
13	Spilopelia chinensis	Eastern Spotted Dove	2019/3/10	3	0	6.04728366	80.85555994	https://www.inaturalist.org/observations/22205222
14	Spilopelia chinensis	Eastern Spotted Dove	2019/7/25	3	0	6.11225000	81.06211667	https://www.inaturalist.org/observations/51229568
15	Spilopelia chinensis	Eastern Spotted Dove	2019/7/25	5	0	6.11225000	81.06211667	https://www.inaturalist.org/observations/51814334
16	Spilopelia chinensis	Eastern Spotted Dove	2017/8/25	6	0	6.11605966	81.06306912	https://www.inaturalist.org/observations/69669113
17	Spilopelia chinensis	Eastern Spotted Dove	2021/12/24	2	0	6.12333050	80.11516667	https://www.inaturalist.org/observations/103575607
18	Spilopelia chinensis	Eastern Spotted Dove	2022/3/9	3	0	6.12555539	80.10881760	https://www.inaturalist.org/observations/108236408
19	Spilopelia chinensis	Eastern Spotted Dove	2014/2/15	3	0	6.13946760	80.10628610	https://www.inaturalist.org/observations/39273175
20	Spilopelia chinensis	Eastern Spotted Dove	2022/2/4	1	0	6.14547204	80.10472108	https://www.inaturalist.org/observations/106154904
21	Spilopelia chinensis	Eastern Spotted Dove	2018/12/12	4	0	6.18032866	81.22043736	https://www.inaturalist.org/observations/19441734
22	Spilopelia chinensis	Eastern Spotted Dove	2007/7/25	4	0	6.18600000	81.19400000	https://www.inaturalist.org/observations/53893540

23	Spilopelia chinensis	Eastern Spotted Dove	2019/11/28	3	0	6.18706169	81.22094283	https://www.inaturalist.org/observations/37123137
24	Spilopelia chinensis	Eastern Spotted Dove	2015/11/11	2	0	6.19742233	81.21126595	https://www.inaturalist.org/observations/63709887
25	Spilopelia chinensis	Eastern Spotted Dove	2019/11/28	2	0	6.19900930	81.21015260	https://www.inaturalist.org/observations/36898330
26	Spilopelia chinensis	Eastern Spotted Dove	2019/7/17	6	0	6.23066000	80.11067167	https://www.inaturalist.org/observations/29967779
27	Spilopelia chinensis	Eastern Spotted Dove	2019/8/11	3	0	6.23466944	81.36834722	https://www.inaturalist.org/observations/32796746
28	Spilopelia chinensis	Eastern Spotted Dove	2019/8/11	4	0	6.23467778	81.36833333	https://www.inaturalist.org/observations/32796736
29	Spilopelia chinensis	Eastern Spotted Dove	2013/1/2	4	0	6.25222222	81.37138889	https://www.inaturalist.org/observations/45713396
30	Spilopelia chinensis	Eastern Spotted Dove	2018/10/24	5	0	6.26103443	81.40035821	https://www.inaturalist.org/observations/17979122
31	Spilopelia chinensis	Eastern Spotted Dove	2019/12/21	4	0	6.26496022	81.41133988	https://www.inaturalist.org/observations/36826139
32	Spilopelia chinensis	Eastern Spotted Dove	2017/3/26	3	0	6.26575960	81.40837669	https://www.inaturalist.org/observations/5505218
33	Spilopelia chinensis	Eastern Spotted Dove	2018/10/24	3	0	6.26918778	81.39203637	https://www.inaturalist.org/observations/18136632
34	Spilopelia chinensis	Eastern Spotted Dove	2016/1/6	4	0	6.27615077	81.29917366	https://www.inaturalist.org/observations/119056129
35	Spilopelia chinensis	Eastern Spotted Dove	2020/7/3	5	0	6.27841172	81.40194096	https://www.inaturalist.org/observations/63234158
36	Spilopelia chinensis	Eastern Spotted Dove	2020/1/31	5	0	6.27842205	81.40193224	https://www.inaturalist.org/observations/40045581
37	Spilopelia chinensis	Eastern Spotted Dove	2019/11/28	2	0	6.28958180	81.28216470	https://www.inaturalist.org/observations/37123393
38	Spilopelia chinensis	Eastern Spotted Dove	2011/1/21	4	0	6.29302248	81.26677764	https://www.inaturalist.org/observations/57910362
39	Spilopelia chinensis	Eastern Spotted Dove	2019/8/12	4	0	6.30466389	81.44143611	https://www.inaturalist.org/observations/32800811
40	Spilopelia chinensis	Eastern Spotted Dove	2019/11/29	3	0	6.30527804	81.44139801	https://www.inaturalist.org/observations/37258623
41	Spilopelia chinensis	Eastern Spotted Dove	2014/4/22	3	0	6.31166951	80.03226144	https://www.inaturalist.org/observations/19174672
42	Spilopelia chinensis	Eastern Spotted Dove	2019/11/29	3	0	6.31223484	81.42931703	https://www.inaturalist.org/observations/37258622
43	Spilopelia chinensis	Eastern Spotted Dove	2022/4/25	5	0	6.31255320	81.40243271	https://www.inaturalist.org/observations/116614968
44	Spilopelia chinensis	Eastern Spotted Dove	2015/11/10	4	0	6.31774392	81.36216974	https://www.inaturalist.org/observations/63415554
45	Spilopelia chinensis	Eastern Spotted Dove	2019/4/2	4	0	6.34351255	80.56191462	https://www.inaturalist.org/observations/50934658

46	Spilopelia chinensis	Eastern Spotted Dove	2019/2/4	3	0	6.34420365	81.49715712	https://www.inaturalist.org/observations/20671961
47	Spilopelia chinensis	Eastern Spotted Dove	2018/10/24	4	0	6.34767843	80.02230336	https://www.inaturalist.org/observations/18278809
48	Spilopelia chinensis	Eastern Spotted Dove	2017/7/14	7	0	6.35436496	80.73593043	https://www.inaturalist.org/observations/67201114
49	Spilopelia chinensis	Eastern Spotted Dove	2022/2/23	3	0	6.35548632	80.49008079	https://www.inaturalist.org/observations/109444889
50	Spilopelia chinensis	Eastern Spotted Dove	2014/2/14	5	0	6.36224000	81.45610167	https://www.inaturalist.org/observations/37174040
51	Spilopelia chinensis	Eastern Spotted Dove	2019/10/6	6	0	6.36286807	81.51891084	https://www.inaturalist.org/observations/34450168
52	Spilopelia chinensis	Eastern Spotted Dove	2019/7/16	3	0	6.36582857	81.24188161	https://www.inaturalist.org/observations/51872421
53	Spilopelia chinensis	Eastern Spotted Dove	2014/4/18	2	0	6.37241551	81.50028828	https://www.inaturalist.org/observations/19157598
54	Spilopelia chinensis	Eastern Spotted Dove	2016/6/12	4	0	6.37277778	81.51694444	https://www.inaturalist.org/observations/14250213
55	Spilopelia chinensis	Eastern Spotted Dove	2020/1/25	3	0	6.38972300	80.50138900	https://www.inaturalist.org/observations/38297522
56	Spilopelia chinensis	Eastern Spotted Dove	2014/2/14	4	0	6.39029333	81.47000667	https://www.inaturalist.org/observations/37118825
57	Spilopelia chinensis	Eastern Spotted Dove	2019/2/4	3	0	6.39547128	81.47917813	https://www.inaturalist.org/observations/20671994
58	Spilopelia chinensis	Eastern Spotted Dove	2019/12/7	3	0	6.41595000	80.81806111	https://www.inaturalist.org/observations/104809865
59	Spilopelia chinensis	Eastern Spotted Dove	2019/4/13	3	0	6.41726635	81.57324359	https://www.inaturalist.org/observations/22556970
60	Spilopelia chinensis	Eastern Spotted Dove	2019/2/10	2	0	6.41855041	80.82393799	https://www.inaturalist.org/observations/89523116
61	Spilopelia chinensis	Eastern Spotted Dove	2019/9/3	4	0	6.42606810	80.85650437	https://www.inaturalist.org/observations/67640438
62	Spilopelia chinensis	Eastern Spotted Dove	2014/11/21	2	0	6.42677800	81.30804700	https://www.inaturalist.org/observations/70878401
63	Spilopelia chinensis	Eastern Spotted Dove	2016/9/18	5	0	6.42678442	81.30804688	https://www.inaturalist.org/observations/100019069
64	Spilopelia chinensis	Eastern Spotted Dove	2016/4/15	4	0	6.43485967	80.90633426	https://www.inaturalist.org/observations/58177919
65	Spilopelia chinensis	Eastern Spotted Dove	2019/3/13	5	0	6.43814260	80.88861140	https://www.inaturalist.org/observations/54360187
66	Spilopelia chinensis	Eastern Spotted Dove	2020/4/25	5	0	6.43814869	80.88861141	https://www.inaturalist.org/observations/43293200
67	Spilopelia chinensis	Eastern Spotted Dove	2012/12/8	4	0	6.43815136	80.88861108	https://www.inaturalist.org/observations/54633158
68	Spilopelia chinensis	Eastern Spotted Dove	2021/12/28	3	0	6.43847024	79.98995125	https://www.inaturalist.org/observations/105034168

69	Spilopelia chinensis	Eastern Spotted Dove	2020/1/27	4	0	6.44049000	80.89069010	https://www.inaturalist.org/observations/38023208
70	Spilopelia chinensis	Eastern Spotted Dove	2019/12/1	4	0	6.44325068	80.41702039	https://www.inaturalist.org/observations/37422045
71	Spilopelia chinensis	Eastern Spotted Dove	2019/11/30	4	0	6.44336806	80.41758632	https://www.inaturalist.org/observations/37422044
72	Spilopelia chinensis	Eastern Spotted Dove	2018/3/6	4	0	6.45126443	80.31057358	https://www.inaturalist.org/observations/10180287
73	Spilopelia chinensis	Eastern Spotted Dove	2013/11/26	4	0	6.45194600	80.89472600	https://www.inaturalist.org/observations/13634051
74	Spilopelia chinensis	Eastern Spotted Dove	2018/11/30	4	0	6.45219793	80.30811299	https://www.inaturalist.org/observations/125268774
75	Spilopelia chinensis	Eastern Spotted Dove	2018/3/6	4	0	6.45223000	80.30824167	https://www.inaturalist.org/observations/10179840
76	Spilopelia chinensis	Eastern Spotted Dove	2015/11/26	3	0	6.45323147	80.89513081	https://www.inaturalist.org/observations/37617560
77	Spilopelia chinensis	Eastern Spotted Dove	2022/3/14	4	0	6.45385217	80.89094731	https://www.inaturalist.org/observations/109189520
78	Spilopelia chinensis	Eastern Spotted Dove	2012/3/5	4	0	6.45460310	80.89238783	https://www.inaturalist.org/observations/64525814
79	Spilopelia chinensis	Eastern Spotted Dove	2022/3/14	4	0	6.45577114	80.89090438	https://www.inaturalist.org/observations/109186573
80	Spilopelia chinensis	Eastern Spotted Dove	2018/1/15	4	0	6.45741500	80.89007833	https://www.inaturalist.org/observations/41520151
81	Spilopelia chinensis	Eastern Spotted Dove	2016/4/5	4	0	6.46562611	80.92092611	https://www.inaturalist.org/observations/127139882
82	Spilopelia chinensis	Eastern Spotted Dove	2021/5/28	5	0	6.47091375	79.98069003	https://www.inaturalist.org/observations/99856621
83	Spilopelia chinensis	Eastern Spotted Dove	2017/7/28	3	0	6.47382494	80.89112237	https://www.inaturalist.org/observations/7655761
84	Spilopelia chinensis	Eastern Spotted Dove	2018/7/16	5	0	6.47468800	80.89882600	https://www.inaturalist.org/observations/15036727
85	Spilopelia chinensis	Eastern Spotted Dove	2018/7/16	5	0	6.47468800	80.89882600	https://www.inaturalist.org/observations/15037545
86	Spilopelia chinensis	Eastern Spotted Dove	2019/1/19	5	0	6.47468840	80.89882570	https://www.inaturalist.org/observations/24798655
87	Spilopelia chinensis	Eastern Spotted Dove	2017/7/28	4	0	6.47764135	80.90509131	https://www.inaturalist.org/observations/7655750
88	Spilopelia chinensis	Eastern Spotted Dove	2017/12/27	4	0	6.48910000	80.86580000	https://www.inaturalist.org/observations/10793938
89	Spilopelia chinensis	Eastern Spotted Dove	2018/3/5	3	0	6.52433667	80.21452500	https://www.inaturalist.org/observations/10168603
90	Spilopelia chinensis	Eastern Spotted Dove	2018/3/7	4	0	6.52652667	80.09291667	https://www.inaturalist.org/observations/10183063
91	Spilopelia chinensis	Eastern Spotted Dove	2020/3/27	3	0	6.53332500	80.14109167	https://www.inaturalist.org/observations/41679064

92	Spilopelia chinensis	Eastern Spotted Dove	2022/7/17	2	0	6.55800315	80.15292768	https://www.inaturalist.org/observations/127082890
93	Spilopelia chinensis	Eastern Spotted Dove	2018/7/17	5	0	6.58539500	79.96074000	https://www.inaturalist.org/observations/15081758
94	Spilopelia chinensis	Eastern Spotted Dove	2018/7/19	5	0	6.58539500	79.96074000	https://www.inaturalist.org/observations/15082054
95	Spilopelia chinensis	Eastern Spotted Dove	2018/7/19	4	0	6.58539500	79.96074000	https://www.inaturalist.org/observations/15082184
96	Spilopelia chinensis	Eastern Spotted Dove	2020/3/25	3	0	6.60139190	79.95274525	https://www.inaturalist.org/observations/40861627
97	Spilopelia chinensis	Eastern Spotted Dove	2020/3/27	4	0	6.60141322	79.95274525	https://www.inaturalist.org/observations/40862285
98	Spilopelia chinensis	Eastern Spotted Dove	2020/3/28	4	0	6.60145585	79.95285254	https://www.inaturalist.org/observations/40937175
99	Spilopelia chinensis	Eastern Spotted Dove	2020/3/28	6	0	6.60149848	79.95285254	https://www.inaturalist.org/observations/40938817
100	Spilopelia chinensis	Eastern Spotted Dove	2020/3/25	3	0	6.60150836	79.95289911	https://www.inaturalist.org/observations/40861087
101	Spilopelia chinensis	Eastern Spotted Dove	2021/7/13	3	0	6.60157847	79.95263567	https://www.inaturalist.org/observations/87234415
102	Spilopelia chinensis	Eastern Spotted Dove	2020/5/8	6	0	6.60163391	79.95267637	https://www.inaturalist.org/observations/46439969
103	Spilopelia chinensis	Eastern Spotted Dove	2020/3/25	5	0	6.60163779	79.95273879	https://www.inaturalist.org/observations/41276508
104	Spilopelia chinensis	Eastern Spotted Dove	2020/5/3	7	0	6.60166373	79.95272150	https://www.inaturalist.org/observations/44845758
105	Spilopelia chinensis	Eastern Spotted Dove	2022/8/21	4	0	6.60352860	80.15960410	https://www.inaturalist.org/observations/131889002
106	Spilopelia chinensis	Eastern Spotted Dove	2019/4/20	2	0	6.62693100	79.94398600	https://www.inaturalist.org/observations/22749945
107	Spilopelia chinensis	Eastern Spotted Dove	2019/5/19	5	0	6.66686110	80.70480840	https://www.inaturalist.org/observations/33367881
108	Spilopelia chinensis	Eastern Spotted Dove	2015/1/18	3	0	6.70557400	80.38473400	https://www.inaturalist.org/observations/35098378
109	Spilopelia chinensis	Eastern Spotted Dove	2020/4/24	6	0	6.71141528	79.90742616	https://www.inaturalist.org/observations/43274164
110	Spilopelia chinensis	Eastern Spotted Dove	2020/1/29	6	0	6.76541360	80.95256550	https://www.inaturalist.org/observations/39831107
111	Spilopelia chinensis	Eastern Spotted Dove	2016/8/2	6	0	6.77103244	80.93688548	https://www.inaturalist.org/observations/16337280
112	Spilopelia chinensis	Eastern Spotted Dove	2022/3/19	4	0	6.78034333	80.90363333	https://www.inaturalist.org/observations/110185511
113	Spilopelia chinensis	Eastern Spotted Dove	2020/12/22	6	0	6.78086352	80.11302574	https://www.inaturalist.org/observations/67578815
114	Spilopelia chinensis	Eastern Spotted Dove	2020/12/16	5	0	6.79710426	80.09433372	https://www.inaturalist.org/observations/67578823

115	Spilopelia chinensis	Eastern Spotted Dove	2021/8/9	1	0	6.80163343	79.89233103	https://www.inaturalist.org/observations/92234342
116	Spilopelia chinensis	Eastern Spotted Dove	2020/12/23	2	0	6.81011155	79.96188683	https://www.inaturalist.org/observations/66959933
117	Spilopelia chinensis	Eastern Spotted Dove	2021/2/3	5	0	6.83294522	80.09039971	https://www.inaturalist.org/observations/71321542
118	Spilopelia chinensis	Eastern Spotted Dove	2021/10/5	5	0	6.83667750	80.09696419	https://www.inaturalist.org/observations/129535313
119	Spilopelia chinensis	Eastern Spotted Dove	2018/7/22	4	0	6.83726482	79.89177547	https://www.inaturalist.org/observations/16213199
120	Spilopelia chinensis	Eastern Spotted Dove	2018/7/21	5	0	6.84116600	79.90247800	https://www.inaturalist.org/observations/15095495
121	Spilopelia chinensis	Eastern Spotted Dove	2018/7/21	5	0	6.84116600	79.90247800	https://www.inaturalist.org/observations/15103568
122	Spilopelia chinensis	Eastern Spotted Dove	2018/7/21	5	0	6.84116600	79.90247800	https://www.inaturalist.org/observations/15104202
123	Spilopelia chinensis	Eastern Spotted Dove	2021/2/21	6	0	6.84522740	79.98896640	https://www.inaturalist.org/observations/69915047
124	Spilopelia chinensis	Eastern Spotted Dove	2020/8/29	5	0	6.84880985	79.95625962	https://www.inaturalist.org/observations/57894068
125	Spilopelia chinensis	Eastern Spotted Dove	2021/11/21	4	0	6.84893894	79.95642090	https://www.inaturalist.org/observations/101605145
126	Spilopelia chinensis	Eastern Spotted Dove	2020/4/30	5	0	6.84919700	79.95633729	https://www.inaturalist.org/observations/44352006
127	Spilopelia chinensis	Eastern Spotted Dove	2021/3/18	2	0	6.85029500	79.98678667	https://www.inaturalist.org/observations/71482788
128	Spilopelia chinensis	Eastern Spotted Dove	2020/1/30	5	0	6.85083030	81.05236060	https://www.inaturalist.org/observations/39960521
129	Spilopelia chinensis	Eastern Spotted Dove	2021/2/19	4	0	6.85267167	79.90075500	https://www.inaturalist.org/observations/69799700
130	Spilopelia chinensis	Eastern Spotted Dove	2021/2/20	4	0	6.85457000	79.90374667	https://www.inaturalist.org/observations/69847908
131	Spilopelia chinensis	Eastern Spotted Dove	2019/4/7	2	0	6.85915800	79.92777200	https://www.inaturalist.org/observations/22255831
132	Spilopelia chinensis	Eastern Spotted Dove	2018/4/8	5	0	6.86113589	81.05050452	https://www.inaturalist.org/observations/10698964
133	Spilopelia chinensis	Eastern Spotted Dove	2022/2/21	0	0	6.86260748	81.03906250	https://www.inaturalist.org/observations/108435849
134	Spilopelia chinensis	Eastern Spotted Dove	2012/3/30	3	0	6.86361005	81.04639077	https://www.inaturalist.org/observations/39463108
135	Spilopelia chinensis	Eastern Spotted Dove	2019/4/18	5	0	6.86669880	81.04655300	https://www.inaturalist.org/observations/49864972
136	Spilopelia chinensis	Eastern Spotted Dove	2018/12/24	3	0	6.86677091	81.04681049	https://www.inaturalist.org/observations/19461978
137	Spilopelia chinensis	Eastern Spotted Dove	2020/1/29	5	0	6.86752453	81.04689766	https://www.inaturalist.org/observations/39919629

138	Spilopelia chinensis	Eastern Spotted Dove	2020/2/13	3	0	6.86886101	81.06062253	https://www.inaturalist.org/observations/39425600
139	Spilopelia chinensis	Eastern Spotted Dove	2019/9/1	3	0	6.87058346	81.05276965	https://www.inaturalist.org/observations/41473828
140	Spilopelia chinensis	Eastern Spotted Dove	2022/2/22	3	0	6.87065667	81.05662333	https://www.inaturalist.org/observations/107418445
141	Spilopelia chinensis	Eastern Spotted Dove	2022/4/9	1	0	6.87162000	81.04839450	https://www.inaturalist.org/observations/110927519
142	Spilopelia chinensis	Eastern Spotted Dove	2019/8/30	3	0	6.87174251	81.05686069	https://www.inaturalist.org/observations/39900970
143	Spilopelia chinensis	Eastern Spotted Dove	2019/12/27	4	0	6.87180667	81.04754000	https://www.inaturalist.org/observations/37194962
144	Spilopelia chinensis	Eastern Spotted Dove	2022/7/25	3	0	6.87977953	79.92923990	https://www.inaturalist.org/observations/127857549
145	Spilopelia chinensis	Eastern Spotted Dove	2022/1/15	2	0	6.88536167	79.85597167	https://www.inaturalist.org/observations/104959029
146	Spilopelia chinensis	Eastern Spotted Dove	2022/2/13	2	0	6.88676126	79.94759995	https://www.inaturalist.org/observations/107409377
147	Spilopelia chinensis	Eastern Spotted Dove	2021/12/12	3	0	6.89485855	79.97509311	https://www.inaturalist.org/observations/102912335
148	Spilopelia chinensis	Eastern Spotted Dove	2021/10/22	2	0	6.90349667	79.89177000	https://www.inaturalist.org/observations/98945126
149	Spilopelia chinensis	Eastern Spotted Dove	2020/11/8	6	0	6.90955833	79.87546667	https://www.inaturalist.org/observations/64428506
150	Spilopelia chinensis	Eastern Spotted Dove	2020/11/10	5	0	6.91002500	79.87536667	https://www.inaturalist.org/observations/64566135
151	Spilopelia chinensis	Eastern Spotted Dove	2020/4/1	3	0	6.91573758	79.87411934	https://www.inaturalist.org/observations/45271961
152	Spilopelia chinensis	Eastern Spotted Dove	2020/4/17	4	0	6.91575888	79.87411934	https://www.inaturalist.org/observations/45271971
153	Spilopelia chinensis	Eastern Spotted Dove	2020/11/6	2	0	6.91577820	79.87416270	https://www.inaturalist.org/observations/64281295
154	Spilopelia chinensis	Eastern Spotted Dove	2020/11/6	5	0	6.91637500	79.87429167	https://www.inaturalist.org/observations/64280088
155	Spilopelia chinensis	Eastern Spotted Dove	2020/4/18	5	0	6.91893281	79.99478303	https://www.inaturalist.org/observations/42606224
156	Spilopelia chinensis	Eastern Spotted Dove	2012/8/22	5	0	6.92122207	80.92041118	https://www.inaturalist.org/observations/31923872
157	Spilopelia chinensis	Eastern Spotted Dove	2012/1/2	5	0	6.93158472	80.10586567	https://www.inaturalist.org/observations/18998192
158	Spilopelia chinensis	Eastern Spotted Dove	2022/7/27	4	0	6.93397103	79.92867999	https://www.inaturalist.org/observations/128168620
159	Spilopelia chinensis	Eastern Spotted Dove	2016/1/23	3	0	6.94971660	80.78910680	https://www.inaturalist.org/observations/42007769
160	Spilopelia chinensis	Eastern Spotted Dove	2019/11/26	3	0	6.97912225	80.76372739	https://www.inaturalist.org/observations/36640036

161	Spilopelia chinensis	Eastern Spotted Dove	2017/6/22	5	0	6.98278610	80.42374134	https://www.inaturalist.org/observations/6870245
162	Spilopelia chinensis	Eastern Spotted Dove	2021/9/19	4	0	6.98515215	80.87780938	https://www.inaturalist.org/observations/95411287
163	Spilopelia chinensis	Eastern Spotted Dove	2015/1/15	3	0	6.98654700	80.42404200	https://www.inaturalist.org/observations/35084692
164	Spilopelia chinensis	Eastern Spotted Dove	2018/10/10	4	0	7.00013667	79.86842167	https://www.inaturalist.org/observations/67193173
165	Spilopelia chinensis	Eastern Spotted Dove	2020/4/20	5	0	7.06679840	79.90409320	https://www.inaturalist.org/observations/42735219
166	Spilopelia chinensis	Eastern Spotted Dove	2021/5/12	3	0	7.06679840	79.90409320	https://www.inaturalist.org/observations/102456837
167	Spilopelia chinensis	Eastern Spotted Dove	2022/5/29	5	0	7.06679840	79.90409320	https://www.inaturalist.org/observations/119963070
168	Spilopelia chinensis	Eastern Spotted Dove	2019/4/5	2	0	7.15338200	80.03156400	https://www.inaturalist.org/observations/22186762
169	Spilopelia chinensis	Eastern Spotted Dove	2019/7/9	6	0	7.21444927	80.78309331	https://www.inaturalist.org/observations/62040735
170	Spilopelia chinensis	Eastern Spotted Dove	2019/4/23	2	0	7.22534700	80.53311100	https://www.inaturalist.org/observations/22982384
171	Spilopelia chinensis	Eastern Spotted Dove	2020/2/10	3	0	7.26305031	80.60792300	https://www.inaturalist.org/observations/39424819
172	Spilopelia chinensis	Eastern Spotted Dove	2019/4/6	3	0	7.26343163	80.59625110	https://www.inaturalist.org/observations/22773290
173	Spilopelia chinensis	Eastern Spotted Dove	2017/7/2	5	0	7.26521900	80.58611565	https://www.inaturalist.org/observations/37120093
174	Spilopelia chinensis	Eastern Spotted Dove	2019/4/17	4	0	7.26605060	80.59470060	https://www.inaturalist.org/observations/22989523
175	Spilopelia chinensis	Eastern Spotted Dove	2018/12/14	4	0	7.26797481	80.59464635	https://www.inaturalist.org/observations/19568130
176	Spilopelia chinensis	Eastern Spotted Dove	2019/12/24	3	0	7.26885667	80.59538833	https://www.inaturalist.org/observations/37193763
177	Spilopelia chinensis	Eastern Spotted Dove	2020/2/11	3	0	7.26903524	80.59586895	https://www.inaturalist.org/observations/39425292
178	Spilopelia chinensis	Eastern Spotted Dove	2022/2/2	1	0	7.26976052	80.59663008	https://www.inaturalist.org/observations/106520170
179	Spilopelia chinensis	Eastern Spotted Dove	2017/7/7	2	0	7.27195860	80.59533940	https://www.inaturalist.org/observations/11672830
180	Spilopelia chinensis	Eastern Spotted Dove	2017/7/7	3	0	7.28597528	80.62884168	https://www.inaturalist.org/observations/40832431
181	Spilopelia chinensis	Eastern Spotted Dove	2013/4/16	4	0	7.28955507	80.64567672	https://www.inaturalist.org/observations/97041700
182	Spilopelia chinensis	Eastern Spotted Dove	2014/3/4	4	0	7.29057150	80.63372620	https://www.inaturalist.org/observations/39241101
183	Spilopelia chinensis	Eastern Spotted Dove	2018/10/27	4	0	7.29244862	79.84277656	https://www.inaturalist.org/observations/18355031

184	Spilopelia chinensis	Eastern Spotted Dove	2018/11/21	4	0	7.29530810	80.66321030	https://www.inaturalist.org/observations/52446277
185	Spilopelia chinensis	Eastern Spotted Dove	2021/12/16	2	0	7.29746303	79.88218792	https://www.inaturalist.org/observations/103134517
186	Spilopelia chinensis	Eastern Spotted Dove	2019/12/28	4	0	7.30559132	80.62346564	https://www.inaturalist.org/observations/37051863
187	Spilopelia chinensis	Eastern Spotted Dove	2019/4/22	2	0	7.30684500	80.73315900	https://www.inaturalist.org/observations/22990334
188	Spilopelia chinensis	Eastern Spotted Dove	2021/12/25	1	0	7.31260158	80.66383995	https://www.inaturalist.org/observations/103636453
189	Spilopelia chinensis	Eastern Spotted Dove	2022/2/4	1	0	7.31261489	80.66390432	https://www.inaturalist.org/observations/106177659
190	Spilopelia chinensis	Eastern Spotted Dove	2021/12/25	2	0	7.31265080	80.66382151	https://www.inaturalist.org/observations/103718086
191	Spilopelia chinensis	Eastern Spotted Dove	2019/5/18	3	0	7.31266653	80.66381395	https://www.inaturalist.org/observations/29313414
192	Spilopelia chinensis	Eastern Spotted Dove	2022/7/6	3	0	7.34061830	79.84823082	https://www.inaturalist.org/observations/125019621
193	Spilopelia chinensis	Eastern Spotted Dove	2022/7/8	4	0	7.34936171	79.83527675	https://www.inaturalist.org/observations/125398539
194	Spilopelia chinensis	Eastern Spotted Dove	2019/12/29	4	0	7.39313225	80.72407551	https://www.inaturalist.org/observations/37294899
195	Spilopelia chinensis	Eastern Spotted Dove	2019/12/31	3	0	7.39320063	80.72405115	https://www.inaturalist.org/observations/37294906
196	Spilopelia chinensis	Eastern Spotted Dove	2022/6/28	4	0	7.47792896	80.32702951	https://www.inaturalist.org/observations/123856676
197	Spilopelia chinensis	Eastern Spotted Dove	2022/7/10	2	0	7.68441194	79.92579695	https://www.inaturalist.org/observations/125744883
198	Spilopelia chinensis	Eastern Spotted Dove	2019/4/28	2	0	7.71000700	80.24504800	https://www.inaturalist.org/observations/24170045
199	Spilopelia chinensis	Eastern Spotted Dove	2021/10/24	2	0	7.79911724	80.80455668	https://www.inaturalist.org/observations/99186254
200	Spilopelia chinensis	Eastern Spotted Dove	2017/11/27	4	0	7.87305400	80.77179700	https://www.inaturalist.org/observations/9951459
201	Spilopelia chinensis	Eastern Spotted Dove	2017/11/29	4	0	7.87305400	80.77179700	https://www.inaturalist.org/observations/9951568
202	Spilopelia chinensis	Eastern Spotted Dove	2017/11/29	2	0	7.87305400	80.77179700	https://www.inaturalist.org/observations/9951570
203	Spilopelia chinensis	Eastern Spotted Dove	2017/8/7	4	0	7.88756401	80.77179685	https://www.inaturalist.org/observations/44566311
204	Spilopelia chinensis	Eastern Spotted Dove	2018/8/12	5	0	7.92848803	80.72167656	https://www.inaturalist.org/observations/68680934
205	Spilopelia chinensis	Eastern Spotted Dove	2019/9/6	4	0	7.93169333	80.01829158	https://www.inaturalist.org/observations/32899331
206	Spilopelia chinensis	Eastern Spotted Dove	2019/8/28	2	0	7.93606963	80.71342561	https://www.inaturalist.org/observations/38547937

207	Spilopelia chinensis	Eastern Spotted Dove	2019/8/27	3	0	7.93986146	80.72236542	https://www.inaturalist.org/observations/37646496
208	Spilopelia chinensis	Eastern Spotted Dove	2019/8/29	3	0	7.94074375	80.72161272	https://www.inaturalist.org/observations/38579922
209	Spilopelia chinensis	Eastern Spotted Dove	2020/1/21	0	0	7.95027309	80.74905591	https://www.inaturalist.org/observations/38806291
210	Spilopelia chinensis	Eastern Spotted Dove	2022/3/12	1	0	7.95080228	80.75952857	https://www.inaturalist.org/observations/109821202
211	Spilopelia chinensis	Eastern Spotted Dove	2012/8/28	5	0	7.95183000	80.76187100	https://www.inaturalist.org/observations/25933817
212	Spilopelia chinensis	Eastern Spotted Dove	2021/8/9	3	0	7.95308690	80.48413250	https://www.inaturalist.org/observations/91718121
213	Spilopelia chinensis	Eastern Spotted Dove	2010/4/29	3	0	7.95410800	80.75469800	https://www.inaturalist.org/observations/134848413
214	Spilopelia chinensis	Eastern Spotted Dove	2020/1/26	5	0	7.95608031	80.75367071	https://www.inaturalist.org/observations/39528338
215	Spilopelia chinensis	Eastern Spotted Dove	2012/12/25	6	0	7.95672721	80.75886726	http://www.inaturalist.org/observations/183274
216	Spilopelia chinensis	Eastern Spotted Dove	2016/1/19	5	0	7.95811600	81.00554500	http://www.inaturalist.org/observations/2969420
217	Spilopelia chinensis	Eastern Spotted Dove	2020/2/7	2	0	7.99457500	80.73264000	https://www.inaturalist.org/observations/39243513
218	Spilopelia chinensis	Eastern Spotted Dove	2016/3/28	4	0	7.99975028	80.74644194	https://www.inaturalist.org/observations/127139812
219	Spilopelia chinensis	Eastern Spotted Dove	2014/1/22	2	0	8.02700528	80.75095374	https://www.inaturalist.org/observations/92594207
220	Spilopelia chinensis	Eastern Spotted Dove	2016/9/13	3	0	8.03298297	80.82443252	https://www.inaturalist.org/observations/100476606
221	Spilopelia chinensis	Eastern Spotted Dove	2019/4/12	3	0	8.03441100	80.90706500	https://www.inaturalist.org/observations/22384724
222	Spilopelia chinensis	Eastern Spotted Dove	2019/7/30	3	0	8.04100550	79.71232000	https://www.inaturalist.org/observations/29836479
223	Spilopelia chinensis	Eastern Spotted Dove	2019/7/28	1	0	8.04124667	79.71228667	https://www.inaturalist.org/observations/29910028
224	Spilopelia chinensis	Eastern Spotted Dove	2019/4/11	5	0	8.12391300	79.70800900	https://www.inaturalist.org/observations/22320258
225	Spilopelia chinensis	Eastern Spotted Dove	2015/11/7	6	0	8.16152787	80.91296138	https://www.inaturalist.org/observations/63261120
226	Spilopelia chinensis	Eastern Spotted Dove	2020/2/3	1	0	8.20802833	80.07042333	https://www.inaturalist.org/observations/39206832
227	Spilopelia chinensis	Eastern Spotted Dove	2020/2/5	2	0	8.20961000	80.07441833	https://www.inaturalist.org/observations/39242241
228	Spilopelia chinensis	Eastern Spotted Dove	2021/11/10	2	0	8.21746076	77.44944949	https://www.inaturalist.org/observations/104865078
229	Spilopelia chinensis	Eastern Spotted Dove	2018/6/13	2	0	8.21794281	77.40831140	https://www.inaturalist.org/observations/135099708

230	Spilopelia chinensis	Eastern Spotted Dove	2021/12/5	2	0	8.22188889	77.57524609	https://www.inaturalist.org/observations/104167103
231	Spilopelia chinensis	Eastern Spotted Dove	2021/12/5	2	0	8.22922137	77.55943419	https://www.inaturalist.org/observations/104167104
232	Spilopelia chinensis	Eastern Spotted Dove	2021/11/10	2	0	8.22946396	77.45999953	https://www.inaturalist.org/observations/104865071
233	Spilopelia chinensis	Eastern Spotted Dove	2021/12/5	2	0	8.26312017	77.45656672	https://www.inaturalist.org/observations/104167102
234	Spilopelia chinensis	Eastern Spotted Dove	2021/11/9	1	0	8.27456406	77.48428570	https://www.inaturalist.org/observations/105084457
235	Spilopelia chinensis	Eastern Spotted Dove	2021/12/6	2	0	8.30225570	77.55617174	https://www.inaturalist.org/observations/103956039
236	Spilopelia chinensis	Eastern Spotted Dove	2019/3/2	2	0	8.32763000	80.37745000	https://www.inaturalist.org/observations/20866297
237	Spilopelia chinensis	Eastern Spotted Dove	2020/2/4	1	0	8.33318500	80.13050000	https://www.inaturalist.org/observations/39205801
238	Spilopelia chinensis	Eastern Spotted Dove	2019/3/2	3	0	8.33472167	80.39051167	https://www.inaturalist.org/observations/20928693
239	Spilopelia chinensis	Eastern Spotted Dove	2019/2/15	3	0	8.34870695	80.40429913	https://www.inaturalist.org/observations/20733712
240	Spilopelia chinensis	Eastern Spotted Dove	2018/12/2	5	0	8.36041766	80.10764256	https://www.inaturalist.org/observations/108580515
241	Spilopelia chinensis	Eastern Spotted Dove	2019/4/25	3	0	8.36229700	80.41896200	https://www.inaturalist.org/observations/23105032
242	Spilopelia chinensis	Eastern Spotted Dove	2021/12/24	1	0	8.37647059	78.05625200	https://www.inaturalist.org/observations/104153017
243	Spilopelia chinensis	Eastern Spotted Dove	2022/3/26	4	0	8.38833300	77.52138900	https://www.inaturalist.org/observations/109844670
244	Spilopelia chinensis	Eastern Spotted Dove	2021/10/19	2	0	8.44482054	77.50892826	https://www.inaturalist.org/observations/102859016
245	Spilopelia chinensis	Eastern Spotted Dove	2022/1/28	1	0	8.45637230	80.04763830	https://www.inaturalist.org/observations/107418491
246	Spilopelia chinensis	Eastern Spotted Dove	2019/12/18	5	0	8.47767467	80.13976979	https://www.inaturalist.org/observations/69579871
247	Spilopelia chinensis	Eastern Spotted Dove	2022/2/12	1	0	8.49590833	77.75911000	https://www.inaturalist.org/observations/106982270
248	Spilopelia chinensis	Eastern Spotted Dove	2022/2/12	1	0	8.49593667	77.75913833	https://www.inaturalist.org/observations/106982273
249	Spilopelia chinensis	Eastern Spotted Dove	2017/11/14	2	0	8.49622960	77.75593700	https://www.inaturalist.org/observations/92338200
250	Spilopelia chinensis	Eastern Spotted Dove	2016/2/5	5	0	8.50883014	80.54077807	https://www.inaturalist.org/observations/33178927
251	Spilopelia chinensis	Eastern Spotted Dove	2021/11/12	4	0	8.55916003	77.46961960	https://www.inaturalist.org/observations/104509437
252	Spilopelia chinensis	Eastern Spotted Dove	2020/5/14	3	0	8.57546817	77.02952553	https://www.inaturalist.org/observations/81547789

253	Spilopelia chinensis	Eastern Spotted Dove	2021/11/12	4	0	8.58137654	77.51437398	https://www.inaturalist.org/observations/104509438
254	Spilopelia chinensis	Eastern Spotted Dove	2021/1/23	0	0	8.59184197	81.18810654	https://www.inaturalist.org/observations/68539400
255	Spilopelia chinensis	Eastern Spotted Dove	2019/11/16	5	0	8.63204447	76.92037437	https://www.inaturalist.org/observations/59487280
256	Spilopelia chinensis	Eastern Spotted Dove	2019/12/24	1	0	8.65577000	77.44803200	https://www.inaturalist.org/observations/56766344
257	Spilopelia chinensis	Eastern Spotted Dove	2020/8/27	2	0	8.65577000	77.44803200	https://www.inaturalist.org/observations/58132925
258	Spilopelia chinensis	Eastern Spotted Dove	2011/8/2	5	0	8.65616235	77.44776821	https://www.inaturalist.org/observations/66742950
259	Spilopelia chinensis	Eastern Spotted Dove	2018/12/2	2	0	8.65777711	77.44755741	https://www.inaturalist.org/observations/63620919
260	Spilopelia chinensis	Eastern Spotted Dove	2021/6/19	1	0	8.65948037	77.40341715	https://www.inaturalist.org/observations/84734324
261	Spilopelia chinensis	Eastern Spotted Dove	2014/9/29	2	0	8.69969770	77.63132000	https://www.inaturalist.org/observations/23914844
262	Spilopelia chinensis	Eastern Spotted Dove	2019/9/19	5	0	8.70518112	77.73418427	https://www.inaturalist.org/observations/32988947
263	Spilopelia chinensis	Eastern Spotted Dove	2019/8/26	3	0	8.71182948	81.18337277	https://www.inaturalist.org/observations/36542666
264	Spilopelia chinensis	Eastern Spotted Dove	2021/10/31	2	0	8.72714200	77.73027500	https://www.inaturalist.org/observations/102397621
265	Spilopelia chinensis	Eastern Spotted Dove	2022/7/5	3	0	8.78171805	76.95558946	https://www.inaturalist.org/observations/127968722
266	Spilopelia chinensis	Eastern Spotted Dove	2011/9/7	3	0	8.78406613	77.32605083	https://www.inaturalist.org/observations/66659978
267	Spilopelia chinensis	Eastern Spotted Dove	2018/1/20	3	0	8.95867959	77.14746329	https://www.inaturalist.org/observations/65983835
268	Spilopelia chinensis	Eastern Spotted Dove	2022/3/2	4	0	8.96151150	77.03464350	https://www.inaturalist.org/observations/111746858
269	Spilopelia chinensis	Eastern Spotted Dove	2014/1/28	7	0	8.97506300	77.14944100	http://www.inaturalist.org/observations/520156
270	Spilopelia chinensis	Eastern Spotted Dove	2022/2/5	2	0	9.01330607	76.66644484	https://www.inaturalist.org/observations/106829268
271	Spilopelia chinensis	Eastern Spotted Dove	2020/1/22	1	0	9.01761170	76.92609360	https://www.inaturalist.org/observations/42019478
272	Spilopelia chinensis	Eastern Spotted Dove	2021/12/17	4	0	9.01761170	76.92609360	https://www.inaturalist.org/observations/103241421
273	Spilopelia chinensis	Eastern Spotted Dove	2021/9/3	3	0	9.17041874	76.55848694	https://www.inaturalist.org/observations/95231809
274	Spilopelia chinensis	Eastern Spotted Dove	2019/4/14	4	0	9.21122903	76.65644376	https://www.inaturalist.org/observations/33358901
275	Spilopelia chinensis	Eastern Spotted Dove	2016/12/3	5	0	9.46215500	77.23684740	https://www.inaturalist.org/observations/33246784

276	Spilopelia chinensis	Eastern Spotted Dove	2021/3/1	3	0	9.49368750	77.48022980	https://www.inaturalist.org/observations/71935032
277	Spilopelia chinensis	Eastern Spotted Dove	2021/3/1	2	0	9.49368750	77.48022980	https://www.inaturalist.org/observations/71938092
278	Spilopelia chinensis	Eastern Spotted Dove	2015/1/27	4	0	9.51498100	77.18191600	https://www.inaturalist.org/observations/35239521
279	Spilopelia chinensis	Eastern Spotted Dove	2018/7/28	1	0	9.57682514	77.17989349	https://www.inaturalist.org/observations/55931112
280	Spilopelia chinensis	Eastern Spotted Dove	2012/8/14	4	0	9.58391637	77.11829323	https://www.inaturalist.org/observations/98477822
281	Spilopelia chinensis	Eastern Spotted Dove	2018/5/11	2	0	9.59454362	77.16532329	https://www.inaturalist.org/observations/42821636
282	Spilopelia chinensis	Eastern Spotted Dove	2022/5/12	3	0	9.60310828	77.16145802	https://www.inaturalist.org/observations/118884283
283	Spilopelia chinensis	Eastern Spotted Dove	2020/1/24	3	0	9.60468091	77.17219293	https://www.inaturalist.org/observations/59451167
284	Spilopelia chinensis	Eastern Spotted Dove	2020/1/24	3	0	9.60471129	77.17202757	https://www.inaturalist.org/observations/59451148
285	Spilopelia chinensis	Eastern Spotted Dove	2021/4/4	3	0	9.64575218	77.17881747	https://www.inaturalist.org/observations/73600023
286	Spilopelia chinensis	Eastern Spotted Dove	2019/6/25	3	0	9.65635000	77.62782167	https://www.inaturalist.org/observations/27611020
287	Spilopelia chinensis	Eastern Spotted Dove	2015/12/26	2	0	9.81380760	76.92973540	https://www.inaturalist.org/observations/91676775
288	Spilopelia chinensis	Eastern Spotted Dove	2022/4/17	3	0	9.83123041	76.78898888	https://www.inaturalist.org/observations/114303459
289	Spilopelia chinensis	Eastern Spotted Dove	2022/4/16	2	0	9.84003461	76.78300106	https://www.inaturalist.org/observations/114299900
290	Spilopelia chinensis	Eastern Spotted Dove	2019/5/29	2	0	9.88330103	78.07305409	https://www.inaturalist.org/observations/60396719
291	Spilopelia chinensis	Eastern Spotted Dove	2021/7/26	2	0	9.89081268	76.69041719	https://www.inaturalist.org/observations/88714655
292	Spilopelia chinensis	Eastern Spotted Dove	2021/8/10	1	0	9.89081268	76.69041719	https://www.inaturalist.org/observations/91750829
293	Spilopelia chinensis	Eastern Spotted Dove	2021/9/10	3	0	9.89081268	76.69041719	https://www.inaturalist.org/observations/94352477
294	Spilopelia chinensis	Eastern Spotted Dove	2021/9/25	1	0	9.89081268	76.69041719	https://www.inaturalist.org/observations/96106958
295	Spilopelia chinensis	Eastern Spotted Dove	2021/10/20	3	0	9.89081268	76.69041719	https://www.inaturalist.org/observations/98805722
296	Spilopelia chinensis	Eastern Spotted Dove	2021/11/10	3	0	9.89081268	76.69041719	https://www.inaturalist.org/observations/100763458
297	Spilopelia chinensis	Eastern Spotted Dove	2022/4/7	2	0	9.89081268	76.69041719	https://www.inaturalist.org/observations/114273754
298	Spilopelia chinensis	Eastern Spotted Dove	2022/4/19	2	0	9.89081268	76.69041719	https://www.inaturalist.org/observations/114307668

299	Spilopelia chinensis	Eastern Spotted Dove	2021/11/26	1	0	9.94155833	78.15813333	https://www.inaturalist.org/observations/101932547
300	Spilopelia chinensis	Eastern Spotted Dove	2021/1/5	4	0	9.94835159	78.14931694	https://www.inaturalist.org/observations/67621520
301	Spilopelia chinensis	Eastern Spotted Dove	2022/3/25	2	0	10.05215922	76.33863608	https://www.inaturalist.org/observations/110468623
302	Spilopelia chinensis	Eastern Spotted Dove	2020/1/31	2	0	10.05349167	76.34194000	https://www.inaturalist.org/observations/67019341
303	Spilopelia chinensis	Eastern Spotted Dove	2014/12/31	2	0	10.05414773	77.06824755	https://www.inaturalist.org/observations/79699655
304	Spilopelia chinensis	Eastern Spotted Dove	2022/4/3	4	0	10.06025360	77.00739190	https://www.inaturalist.org/observations/110418049
305	Spilopelia chinensis	Eastern Spotted Dove	2021/3/1	2	0	10.06120630	76.34652816	https://www.inaturalist.org/observations/70401194
306	Spilopelia chinensis	Eastern Spotted Dove	2020/1/28	3	0	10.08732307	77.07113081	https://www.inaturalist.org/observations/66773424
307	Spilopelia chinensis	Eastern Spotted Dove	2016/4/17	6	0	10.08893300	77.05952500	http://www.inaturalist.org/observations/3594665
308	Spilopelia chinensis	Eastern Spotted Dove	2017/4/1	2	0	10.08893330	77.05952480	https://www.inaturalist.org/observations/64363248
309	Spilopelia chinensis	Eastern Spotted Dove	2017/3/30	2	0	10.08893330	77.05952480	https://www.inaturalist.org/observations/64365106
310	Spilopelia chinensis	Eastern Spotted Dove	2021/1/31	1	0	10.08897943	77.05952469	https://www.inaturalist.org/observations/68922144
311	Spilopelia chinensis	Eastern Spotted Dove	2020/2/7	5	0	10.08980405	77.05986946	https://www.inaturalist.org/observations/81605845
312	Spilopelia chinensis	Eastern Spotted Dove	2020/1/26	2	0	10.09031211	76.78242894	https://www.inaturalist.org/observations/39200897
313	Spilopelia chinensis	Eastern Spotted Dove	2020/5/12	8	0	10.09379346	77.05295563	https://www.inaturalist.org/observations/45659613
314	Spilopelia chinensis	Eastern Spotted Dove	2019/1/26	1	0	10.09489859	76.92231587	https://www.inaturalist.org/observations/49152745
315	Spilopelia chinensis	Eastern Spotted Dove	2019/1/27	4	0	10.09522520	76.93191555	https://www.inaturalist.org/observations/121761011
316	Spilopelia chinensis	Eastern Spotted Dove	2020/12/31	4	0	10.10398010	76.70046039	https://www.inaturalist.org/observations/129569752
317	Spilopelia chinensis	Eastern Spotted Dove	2018/12/15	3	0	10.10398030	76.70046050	https://www.inaturalist.org/observations/32153134
318	Spilopelia chinensis	Eastern Spotted Dove	2020/1/19	4	0	10.10398030	76.70046050	https://www.inaturalist.org/observations/51566614
319	Spilopelia chinensis	Eastern Spotted Dove	2019/9/13	3	0	10.11054025	77.05457398	https://www.inaturalist.org/observations/36405621
320	Spilopelia chinensis	Eastern Spotted Dove	2020/12/12	1	0	10.11595215	76.67348356	https://www.inaturalist.org/observations/66516990
321	Spilopelia chinensis	Eastern Spotted Dove	2020/12/12	1	0	10.11609644	76.67469755	https://www.inaturalist.org/observations/66996865

322	Spilopelia chinensis	Eastern Spotted Dove	2022/5/10	4	0	10.11746540	76.67468556	https://www.inaturalist.org/observations/117595407
323	Spilopelia chinensis	Eastern Spotted Dove	2020/12/31	5	0	10.12979136	76.68711841	https://www.inaturalist.org/observations/125054337
324	Spilopelia chinensis	Eastern Spotted Dove	2022/2/12	5	0	10.13022505	76.68713752	https://www.inaturalist.org/observations/106656304
325	Spilopelia chinensis	Eastern Spotted Dove	2020/2/21	2	0	10.13098780	76.24104930	https://www.inaturalist.org/observations/41221484
326	Spilopelia chinensis	Eastern Spotted Dove	2021/3/4	4	0	10.18497500	77.25920000	https://www.inaturalist.org/observations/70883502
327	Spilopelia chinensis	Eastern Spotted Dove	2022/9/11	2	0	10.18715861	77.25441809	https://www.inaturalist.org/observations/136777088
328	Spilopelia chinensis	Eastern Spotted Dove	2022/8/15	4	0	10.19553878	76.49679188	https://www.inaturalist.org/observations/130948602
329	Spilopelia chinensis	Eastern Spotted Dove	2021/11/7	5	0	10.20606635	76.50078937	https://www.inaturalist.org/observations/100844677
330	Spilopelia chinensis	Eastern Spotted Dove	2014/1/3	2	0	10.21351920	77.19721950	https://www.inaturalist.org/observations/48609801
331	Spilopelia chinensis	Eastern Spotted Dove	2019/3/1	2	0	10.21425843	77.48530928	https://www.inaturalist.org/observations/21225592
332	Spilopelia chinensis	Eastern Spotted Dove	2020/2/10	0	0	10.22988667	77.49367500	https://www.inaturalist.org/observations/38502739
333	Spilopelia chinensis	Eastern Spotted Dove	2015/6/8	3	0	10.23004080	77.49363590	https://www.inaturalist.org/observations/22598335
334	Spilopelia chinensis	Eastern Spotted Dove	2018/5/6	5	0	10.23033140	77.48435790	https://www.inaturalist.org/observations/12668041
335	Spilopelia chinensis	Eastern Spotted Dove	2017/7/1	3	0	10.23088455	77.49417877	https://www.inaturalist.org/observations/20845853
336	Spilopelia chinensis	Eastern Spotted Dove	2010/3/22	1	0	10.23212102	77.49460726	https://www.inaturalist.org/observations/93533748
337	Spilopelia chinensis	Eastern Spotted Dove	2019/3/16	4	0	10.23270306	77.45959863	https://www.inaturalist.org/observations/64726885
338	Spilopelia chinensis	Eastern Spotted Dove	2018/8/14	6	0	10.23315949	77.49429362	https://www.inaturalist.org/observations/15841363
339	Spilopelia chinensis	Eastern Spotted Dove	2022/4/17	3	0	10.23487186	77.48770142	https://www.inaturalist.org/observations/111767734
340	Spilopelia chinensis	Eastern Spotted Dove	2020/11/28	2	0	10.23537962	77.18615849	https://www.inaturalist.org/observations/66173783
341	Spilopelia chinensis	Eastern Spotted Dove	2019/8/11	4	0	10.23811360	77.48918220	https://www.inaturalist.org/observations/31532726
342	Spilopelia chinensis	Eastern Spotted Dove	2016/12/19	1	0	10.23811360	77.48918220	https://www.inaturalist.org/observations/92319674
343	Spilopelia chinensis	Eastern Spotted Dove	2019/4/8	3	0	10.26038230	77.16759130	https://www.inaturalist.org/observations/22191597
344	Spilopelia chinensis	Eastern Spotted Dove	2020/11/2	3	0	10.26754555	77.50030920	https://www.inaturalist.org/observations/64029148

345	Spilopelia chinensis	Eastern Spotted Dove	2022/7/2	2	0	10.26882500	77.15628167	https://www.inaturalist.org/observations/126917626
346	Spilopelia chinensis	Eastern Spotted Dove	2021/4/30	3	0	10.27630211	77.16136150	https://www.inaturalist.org/observations/117098421
347	Spilopelia chinensis	Eastern Spotted Dove	2021/11/9	3	0	10.29247036	76.39274865	https://www.inaturalist.org/observations/103521661
348	Spilopelia chinensis	Eastern Spotted Dove	2020/10/3	3	0	10.29525960	77.36546110	https://www.inaturalist.org/observations/62098293
349	Spilopelia chinensis	Eastern Spotted Dove	2020/10/2	3	0	10.29525960	77.36546110	https://www.inaturalist.org/observations/62098309
350	Spilopelia chinensis	Eastern Spotted Dove	2021/12/25	1	0	10.29600337	79.81704712	https://www.inaturalist.org/observations/103956411
351	Spilopelia chinensis	Eastern Spotted Dove	2021/9/12	1	0	10.29911739	79.84560728	https://www.inaturalist.org/observations/94714092
352	Spilopelia chinensis	Eastern Spotted Dove	2021/11/26	5	0	10.30214265	76.42738435	https://www.inaturalist.org/observations/101949013
353	Spilopelia chinensis	Eastern Spotted Dove	2021/12/31	3	0	10.30214265	76.42738435	https://www.inaturalist.org/observations/104025514
354	Spilopelia chinensis	Eastern Spotted Dove	2022/1/12	4	0	10.30214265	76.42738435	https://www.inaturalist.org/observations/104817256
355	Spilopelia chinensis	Eastern Spotted Dove	2022/2/25	5	0	10.30214265	76.42738435	https://www.inaturalist.org/observations/107414818
356	Spilopelia chinensis	Eastern Spotted Dove	2022/2/27	5	0	10.30214265	76.42738435	https://www.inaturalist.org/observations/107550901
357	Spilopelia chinensis	Eastern Spotted Dove	2022/3/7	4	0	10.30214265	76.42738435	https://www.inaturalist.org/observations/108105666
358	Spilopelia chinensis	Eastern Spotted Dove	2022/9/14	2	0	10.30219148	76.42738435	https://www.inaturalist.org/observations/135078926
359	Spilopelia chinensis	Eastern Spotted Dove	2022/9/28	2	0	10.30219148	76.42738435	https://www.inaturalist.org/observations/136812237
360	Spilopelia chinensis	Eastern Spotted Dove	2022/9/28	2	0	10.30219148	76.42738435	https://www.inaturalist.org/observations/136887142
361	Spilopelia chinensis	Eastern Spotted Dove	2022/9/28	2	0	10.30219148	76.42738435	https://www.inaturalist.org/observations/136887352
362	Spilopelia chinensis	Eastern Spotted Dove	2015/6/4	4	0	10.30679740	77.20602410	https://www.inaturalist.org/observations/12149091
363	Spilopelia chinensis	Eastern Spotted Dove	2013/11/29	2	0	10.30679740	77.20602410	https://www.inaturalist.org/observations/81671689
364	Spilopelia chinensis	Eastern Spotted Dove	2021/11/12	1	0	10.30679740	77.20602410	https://www.inaturalist.org/observations/129437031
365	Spilopelia chinensis	Eastern Spotted Dove	2013/11/29	3	0	10.30681260	77.20601320	https://www.inaturalist.org/observations/81671708
366	Spilopelia chinensis	Eastern Spotted Dove	2016/12/24	2	0	10.30986500	76.92666500	https://www.inaturalist.org/observations/64753825
367	Spilopelia chinensis	Eastern Spotted Dove	2021/12/25	1	0	10.31622667	79.83669829	https://www.inaturalist.org/observations/103752063

368	Spilopelia chinensis	Eastern Spotted Dove	2020/1/29	1	0	10.32359080	76.95100300	https://www.inaturalist.org/observations/44604300
369	Spilopelia chinensis	Eastern Spotted Dove	2019/10/12	2	0	10.32697340	76.95539670	https://www.inaturalist.org/observations/56023063
370	Spilopelia chinensis	Eastern Spotted Dove	2021/3/29	3	0	10.32697340	76.95539670	https://www.inaturalist.org/observations/105206848
371	Spilopelia chinensis	Eastern Spotted Dove	2021/3/29	2	0	10.32697340	76.95539670	https://www.inaturalist.org/observations/105256382
372	Spilopelia chinensis	Eastern Spotted Dove	2021/2/3	1	0	10.33630520	76.95485350	https://www.inaturalist.org/observations/79244228
373	Spilopelia chinensis	Eastern Spotted Dove	2022/5/1	2	0	10.36569167	76.24873500	https://www.inaturalist.org/observations/114746624
374	Spilopelia chinensis	Eastern Spotted Dove	2021/3/27	3	0	10.36828538	76.33538727	https://www.inaturalist.org/observations/73264079
375	Spilopelia chinensis	Eastern Spotted Dove	2019/8/3	2	0	10.39286550	76.77562192	https://www.inaturalist.org/observations/48201809
376	Spilopelia chinensis	Eastern Spotted Dove	2020/1/7	2	0	10.39720500	76.22623167	https://www.inaturalist.org/observations/44164574
377	Spilopelia chinensis	Eastern Spotted Dove	2011/2/13	1	0	10.42409900	77.12556000	https://www.inaturalist.org/observations/91250216
378	Spilopelia chinensis	Eastern Spotted Dove	2021/5/13	4	0	10.44361120	76.23179633	https://www.inaturalist.org/observations/99608887
379	Spilopelia chinensis	Eastern Spotted Dove	2021/10/31	2	0	10.44464520	76.23342779	https://www.inaturalist.org/observations/99965658
380	Spilopelia chinensis	Eastern Spotted Dove	2020/11/29	4	0	10.46857731	76.15747782	https://www.inaturalist.org/observations/67708507
381	Spilopelia chinensis	Eastern Spotted Dove	2014/12/29	2	0	10.46937591	76.84236702	https://www.inaturalist.org/observations/45472982
382	Spilopelia chinensis	Eastern Spotted Dove	2019/9/9	4	0	10.47282333	76.19793000	https://www.inaturalist.org/observations/32704976
383	Spilopelia chinensis	Eastern Spotted Dove	2020/1/13	2	0	10.52820000	76.27412500	https://www.inaturalist.org/observations/67014874
384	Spilopelia chinensis	Eastern Spotted Dove	2021/2/27	3	0	10.53029080	76.34731870	https://www.inaturalist.org/observations/100293353
385	Spilopelia chinensis	Eastern Spotted Dove	2021/12/2	2	0	10.53029087	76.34731840	https://www.inaturalist.org/observations/104843180
386	Spilopelia chinensis	Eastern Spotted Dove	2020/3/20	2	0	10.53029449	76.34731840	https://www.inaturalist.org/observations/100075231
387	Spilopelia chinensis	Eastern Spotted Dove	2020/1/30	3	0	10.54093167	76.24092167	https://www.inaturalist.org/observations/67019392
388	Spilopelia chinensis	Eastern Spotted Dove	2020/1/5	2	0	10.54161833	76.15446667	https://www.inaturalist.org/observations/44104671
389	Spilopelia chinensis	Eastern Spotted Dove	2019/10/4	3	0	10.54213922	76.15429484	https://www.inaturalist.org/observations/34218259
390	Spilopelia chinensis	Eastern Spotted Dove	2019/9/15	4	0	10.54911500	76.28487333	https://www.inaturalist.org/observations/33021464

391	Spilopelia chinensis	Eastern Spotted Dove	2014/5/14	3	0	10.58321956	76.14201026	https://www.inaturalist.org/observations/30260282
392	Spilopelia chinensis	Eastern Spotted Dove	2017/6/18	1	0	10.59842855	76.03892364	https://www.inaturalist.org/observations/30260541
393	Spilopelia chinensis	Eastern Spotted Dove	2021/5/14	3	0	10.61399875	76.19414621	https://www.inaturalist.org/observations/107045211
394	Spilopelia chinensis	Eastern Spotted Dove	2020/12/11	2	0	10.61491000	76.19266333	https://www.inaturalist.org/observations/67002155
395	Spilopelia chinensis	Eastern Spotted Dove	2020/6/30	1	0	10.61566106	76.19416515	https://www.inaturalist.org/observations/51495315
396	Spilopelia chinensis	Eastern Spotted Dove	2020/4/18	3	0	10.61582030	76.19352223	https://www.inaturalist.org/observations/48353977
397	Spilopelia chinensis	Eastern Spotted Dove	2019/1/24	5	0	10.63750217	76.30570555	https://www.inaturalist.org/observations/19876518
398	Spilopelia chinensis	Eastern Spotted Dove	2022/8/17	3	0	10.64121920	76.30836602	https://www.inaturalist.org/observations/132082252
399	Spilopelia chinensis	Eastern Spotted Dove	2021/8/30	2	0	10.68515779	76.52486357	https://www.inaturalist.org/observations/93924815
400	Spilopelia chinensis	Eastern Spotted Dove	2021/8/30	2	0	10.68515779	76.52486357	https://www.inaturalist.org/observations/93924837
401	Spilopelia chinensis	Eastern Spotted Dove	2019/9/12	1	0	10.69413692	76.34636220	https://www.inaturalist.org/observations/78827708
402	Spilopelia chinensis	Eastern Spotted Dove	2021/12/24	1	0	10.70020365	79.32311940	https://www.inaturalist.org/observations/103949682
403	Spilopelia chinensis	Eastern Spotted Dove	2021/12/24	2	0	10.70075000	79.32228667	https://www.inaturalist.org/observations/103745301
404	Spilopelia chinensis	Eastern Spotted Dove	2016/12/29	2	0	10.74712700	76.46723400	https://www.inaturalist.org/observations/87008575
405	Spilopelia chinensis	Eastern Spotted Dove	2016/12/30	2	0	10.74723400	76.46719600	https://www.inaturalist.org/observations/87008596
406	Spilopelia chinensis	Eastern Spotted Dove	2016/12/18	2	0	10.74744183	76.46735703	https://www.inaturalist.org/observations/65605239
407	Spilopelia chinensis	Eastern Spotted Dove	2021/1/24	2	0	10.74960298	76.37829512	https://www.inaturalist.org/observations/99718444
408	Spilopelia chinensis	Eastern Spotted Dove	2020/8/22	2	0	10.74974237	76.37860390	https://www.inaturalist.org/observations/61872152
409	Spilopelia chinensis	Eastern Spotted Dove	2014/5/24	2	0	10.74974237	76.37860390	https://www.inaturalist.org/observations/98113865
410	Spilopelia chinensis	Eastern Spotted Dove	2021/3/10	4	0	10.75818940	76.11749880	https://www.inaturalist.org/observations/71037451
411	Spilopelia chinensis	Eastern Spotted Dove	2022/8/22	4	0	10.79878611	79.13876944	https://www.inaturalist.org/observations/131962735
412	Spilopelia chinensis	Eastern Spotted Dove	2022/2/9	1	0	10.80113333	78.76062500	https://www.inaturalist.org/observations/106411137
413	Spilopelia chinensis	Eastern Spotted Dove	2021/12/26	2	0	10.80336050	76.08453635	https://www.inaturalist.org/observations/103888775

414	Spilopelia chinensis	Eastern Spotted Dove	2021/11/11	2	0	10.82377949	76.68334320	https://www.inaturalist.org/observations/101502208
415	Spilopelia chinensis	Eastern Spotted Dove	2020/11/8	3	0	10.83880570	76.69035700	https://www.inaturalist.org/observations/67399674
416	Spilopelia chinensis	Eastern Spotted Dove	2022/3/14	4	0	10.84015309	76.83562236	https://www.inaturalist.org/observations/115189882
417	Spilopelia chinensis	Eastern Spotted Dove	2022/4/2	2	0	10.84320590	76.84617680	https://www.inaturalist.org/observations/110561991
418	Spilopelia chinensis	Eastern Spotted Dove	2019/12/23	2	0	10.84845193	76.43446589	https://www.inaturalist.org/observations/52681584
419	Spilopelia chinensis	Eastern Spotted Dove	2022/4/3	2	0	10.86576010	76.65920730	https://www.inaturalist.org/observations/110563600
420	Spilopelia chinensis	Eastern Spotted Dove	2022/3/15	3	0	10.86624972	76.64740575	https://www.inaturalist.org/observations/115064230
421	Spilopelia chinensis	Eastern Spotted Dove	2015/3/5	1	0	10.87917342	78.70316722	https://www.inaturalist.org/observations/72021976
422	Spilopelia chinensis	Eastern Spotted Dove	2021/5/9	1	0	10.88471926	76.06608879	https://www.inaturalist.org/observations/78016353
423	Spilopelia chinensis	Eastern Spotted Dove	2021/6/10	1	0	10.88505740	76.06598720	https://www.inaturalist.org/observations/82398998
424	Spilopelia chinensis	Eastern Spotted Dove	2021/12/31	1	0	10.88709380	76.06606850	https://www.inaturalist.org/observations/104009232
425	Spilopelia chinensis	Eastern Spotted Dove	2021/8/24	2	0	10.88748260	76.06472830	https://www.inaturalist.org/observations/92324098
426	Spilopelia chinensis	Eastern Spotted Dove	2021/7/6	2	0	10.88749857	76.06633992	https://www.inaturalist.org/observations/85893816
427	Spilopelia chinensis	Eastern Spotted Dove	2022/8/6	4	0	10.88791500	76.06467400	https://www.inaturalist.org/observations/129642778
428	Spilopelia chinensis	Eastern Spotted Dove	2021/7/11	3	0	10.88826110	76.06684090	https://www.inaturalist.org/observations/86531885
429	Spilopelia chinensis	Eastern Spotted Dove	2020/10/17	1	0	10.98630000	76.90633900	https://www.inaturalist.org/observations/107245276
430	Spilopelia chinensis	Eastern Spotted Dove	2020/8/29	2	0	11.01684450	76.95583210	https://www.inaturalist.org/observations/118348924
431	Spilopelia chinensis	Eastern Spotted Dove	2019/1/24	2	0	11.01684450	76.95583210	https://www.inaturalist.org/observations/135547289
432	Spilopelia chinensis	Eastern Spotted Dove	2021/12/9	5	0	11.01717987	76.95583183	https://www.inaturalist.org/observations/132372649
433	Spilopelia chinensis	Eastern Spotted Dove	2020/3/28	2	0	11.07894586	79.83002432	https://www.inaturalist.org/observations/48747971
434	Spilopelia chinensis	Eastern Spotted Dove	2020/6/14	2	0	11.07894586	79.83002432	https://www.inaturalist.org/observations/49541132
435	Spilopelia chinensis	Eastern Spotted Dove	2018/1/27	4	0	11.08598366	76.46006088	https://www.inaturalist.org/observations/65743568
436	Spilopelia chinensis	Eastern Spotted Dove	2018/1/28	3	0	11.09079800	76.44972183	https://www.inaturalist.org/observations/65725302

437	Spilopelia chinensis	Eastern Spotted Dove	2022/6/5	5	0	11.10741096	79.78331365	https://www.inaturalist.org/observations/120295771
438	Spilopelia chinensis	Eastern Spotted Dove	2021/8/1	1	0	11.11049397	79.70172353	https://www.inaturalist.org/observations/89437931
439	Spilopelia chinensis	Eastern Spotted Dove	2022/4/2	2	0	11.13291820	75.89125469	https://www.inaturalist.org/observations/110278078
440	Spilopelia chinensis	Eastern Spotted Dove	2020/11/25	2	0	11.13389588	75.89402609	https://www.inaturalist.org/observations/70706994
441	Spilopelia chinensis	Eastern Spotted Dove	2020/4/27	4	0	11.18829610	76.26094630	https://www.inaturalist.org/observations/58726979
442	Spilopelia chinensis	Eastern Spotted Dove	2019/10/29	3	0	11.23409110	77.65862357	https://www.inaturalist.org/observations/37123165
443	Spilopelia chinensis	Eastern Spotted Dove	2022/2/13	3	0	11.25282833	77.65826667	https://www.inaturalist.org/observations/106983071
444	Spilopelia chinensis	Eastern Spotted Dove	2021/1/14	1	0	11.32303840	79.24335279	https://www.inaturalist.org/observations/68127316
445	Spilopelia chinensis	Eastern Spotted Dove	2020/11/12	4	0	11.32304662	79.24335279	https://www.inaturalist.org/observations/66090919
446	Spilopelia chinensis	Eastern Spotted Dove	2021/7/20	2	0	11.32304662	79.24335279	https://www.inaturalist.org/observations/89325462
447	Spilopelia chinensis	Eastern Spotted Dove	2021/7/20	2	0	11.32304662	79.24335279	https://www.inaturalist.org/observations/89329497
448	Spilopelia chinensis	Eastern Spotted Dove	2022/8/17	2	0	11.32304662	79.24335279	https://www.inaturalist.org/observations/131674468
449	Spilopelia chinensis	Eastern Spotted Dove	2022/8/15	3	0	11.32304662	79.24335279	https://www.inaturalist.org/observations/131713270
450	Spilopelia chinensis	Eastern Spotted Dove	2022/8/15	5	0	11.32304662	79.24335279	https://www.inaturalist.org/observations/131737241
451	Spilopelia chinensis	Eastern Spotted Dove	2016/12/29	1	0	11.32598793	79.24508952	https://www.inaturalist.org/observations/54542317
452	Spilopelia chinensis	Eastern Spotted Dove	2022/7/10	1	0	11.33563200	76.81587400	https://www.inaturalist.org/observations/136108411
453	Spilopelia chinensis	Eastern Spotted Dove	2021/1/18	1	0	11.33996667	76.75147500	https://www.inaturalist.org/observations/68271527
454	Spilopelia chinensis	Eastern Spotted Dove	2019/1/2	3	0	11.34094277	76.86998347	https://www.inaturalist.org/observations/19781704
455	Spilopelia chinensis	Eastern Spotted Dove	2021/7/9	4	0	11.37319358	75.84196169	https://www.inaturalist.org/observations/87826445
456	Spilopelia chinensis	Eastern Spotted Dove	2022/7/1	5	0	11.39116287	79.70322781	https://www.inaturalist.org/observations/124579094
457	Spilopelia chinensis	Eastern Spotted Dove	2020/4/4	2	0	11.41214736	76.80158705	https://www.inaturalist.org/observations/41353502
458	Spilopelia chinensis	Eastern Spotted Dove	2020/10/16	3	0	11.41522053	76.71850059	https://www.inaturalist.org/observations/63328212
459	Spilopelia chinensis	Eastern Spotted Dove	2021/6/30	1	0	11.41522053	76.71850059	https://www.inaturalist.org/observations/86871868

460	Spilopelia chinensis	Eastern Spotted Dove	2021/7/21	4	0	11.41522053	76.71850059	https://www.inaturalist.org/observations/88105066
461	Spilopelia chinensis	Eastern Spotted Dove	2020/1/17	3	0	11.42188896	76.86172754	https://www.inaturalist.org/observations/55793634
462	Spilopelia chinensis	Eastern Spotted Dove	2019/3/24	3	0	11.47320910	76.67901380	https://www.inaturalist.org/observations/23016008
463	Spilopelia chinensis	Eastern Spotted Dove	2019/3/24	2	0	11.47320910	76.67901380	https://www.inaturalist.org/observations/23017490
464	Spilopelia chinensis	Eastern Spotted Dove	2019/1/27	1	0	11.53107778	76.64257222	https://www.inaturalist.org/observations/102544364
465	Spilopelia chinensis	Eastern Spotted Dove	2015/1/6	1	0	11.53526164	76.65010023	https://www.inaturalist.org/observations/80580030
466	Spilopelia chinensis	Eastern Spotted Dove	2011/3/3	6	0	11.56614300	76.64794900	https://www.inaturalist.org/observations/17690584
467	Spilopelia chinensis	Eastern Spotted Dove	2016/4/15	3	0	11.57500960	76.55543800	https://www.inaturalist.org/observations/34053159
468	Spilopelia chinensis	Eastern Spotted Dove	2014/12/22	7	0	11.57598200	78.69927400	http://www.inaturalist.org/observations/1555397
469	Spilopelia chinensis	Eastern Spotted Dove	2013/11/7	6	0	11.57823125	76.65370137	https://www.inaturalist.org/observations/9896943
470	Spilopelia chinensis	Eastern Spotted Dove	2014/8/2	4	0	11.57922579	76.58381220	https://www.inaturalist.org/observations/20034214
471	Spilopelia chinensis	Eastern Spotted Dove	2018/12/29	1	0	11.58161710	76.99886690	https://www.inaturalist.org/observations/92214309
472	Spilopelia chinensis	Eastern Spotted Dove	2011/8/21	6	0	11.60346600	76.14237200	http://www.inaturalist.org/observations/29919
473	Spilopelia chinensis	Eastern Spotted Dove	2020/10/17	3	0	11.61821948	76.17221508	https://www.inaturalist.org/observations/63304022
474	Spilopelia chinensis	Eastern Spotted Dove	2018/9/14	5	0	11.63759180	76.52570070	https://www.inaturalist.org/observations/17124436
475	Spilopelia chinensis	Eastern Spotted Dove	2021/7/3	1	0	11.63784405	76.52570073	https://www.inaturalist.org/observations/86954445
476	Spilopelia chinensis	Eastern Spotted Dove	2019/5/6	2	0	11.64951111	76.05216111	https://www.inaturalist.org/observations/78674223
477	Spilopelia chinensis	Eastern Spotted Dove	2018/10/22	2	0	11.65302908	76.60661090	https://www.inaturalist.org/observations/37091507
478	Spilopelia chinensis	Eastern Spotted Dove	2017/8/21	2	0	11.67683741	75.95238330	https://www.inaturalist.org/observations/65888348
479	Spilopelia chinensis	Eastern Spotted Dove	2021/4/9	1	0	11.69837952	76.48135376	https://www.inaturalist.org/observations/74604938
480	Spilopelia chinensis	Eastern Spotted Dove	2020/10/24	2	0	11.70175426	79.75943897	https://www.inaturalist.org/observations/67136720
481	Spilopelia chinensis	Eastern Spotted Dove	2022/4/9	3	0	11.70910400	78.18970129	https://www.inaturalist.org/observations/110841594
482	Spilopelia chinensis	Eastern Spotted Dove	2021/8/22	2	0	11.73561617	78.20203878	https://www.inaturalist.org/observations/92074334

483	Spilopelia chinensis	Eastern Spotted Dove	2022/7/24	4	0	11.73561617	78.20203878	https://www.inaturalist.org/observations/128550995
484	Spilopelia chinensis	Eastern Spotted Dove	2021/12/18	1	0	11.74613203	78.17025661	https://www.inaturalist.org/observations/103635692
485	Spilopelia chinensis	Eastern Spotted Dove	2017/8/20	1	0	11.74613235	78.17025661	https://www.inaturalist.org/observations/81318815
486	Spilopelia chinensis	Eastern Spotted Dove	2021/8/29	3	0	11.75915032	75.55387102	https://www.inaturalist.org/observations/107238389
487	Spilopelia chinensis	Eastern Spotted Dove	2021/1/13	3	0	11.80617155	76.02141023	https://www.inaturalist.org/observations/68047954
488	Spilopelia chinensis	Eastern Spotted Dove	2020/7/15	1	0	11.80868214	76.02043457	https://www.inaturalist.org/observations/56095949
489	Spilopelia chinensis	Eastern Spotted Dove	2021/9/19	2	0	11.80880581	76.05844919	https://www.inaturalist.org/observations/95505941
490	Spilopelia chinensis	Eastern Spotted Dove	2022/8/3	4	0	11.80927800	76.05887886	https://www.inaturalist.org/observations/129238516
491	Spilopelia chinensis	Eastern Spotted Dove	2022/7/10	4	0	11.81961070	78.26983630	https://www.inaturalist.org/observations/132934459
492	Spilopelia chinensis	Eastern Spotted Dove	2020/7/6	4	0	11.84297340	75.55311727	https://www.inaturalist.org/observations/52183362
493	Spilopelia chinensis	Eastern Spotted Dove	2021/8/19	1	0	11.84425186	75.55307278	https://www.inaturalist.org/observations/93126467
494	Spilopelia chinensis	Eastern Spotted Dove	2018/6/15	2	0	11.87245961	76.27940882	https://www.inaturalist.org/observations/97920242
495	Spilopelia chinensis	Eastern Spotted Dove	2015/3/13	2	0	11.88517097	76.17806269	https://www.inaturalist.org/observations/62471641
496	Spilopelia chinensis	Eastern Spotted Dove	2019/7/6	4	0	11.90304137	77.12219417	https://www.inaturalist.org/observations/28556248
497	Spilopelia chinensis	Eastern Spotted Dove	2015/3/13	2	0	11.93864094	76.24365709	https://www.inaturalist.org/observations/62471638
498	Spilopelia chinensis	Eastern Spotted Dove	2018/3/26	6	0	11.93892500	76.27041667	https://www.inaturalist.org/observations/66384402
499	Spilopelia chinensis	Eastern Spotted Dove	2015/3/14	2	0	11.93942070	76.21612846	https://www.inaturalist.org/observations/62471640
500	Spilopelia chinensis	Eastern Spotted Dove	2022/6/6	1	0	11.94468432	75.38493812	https://www.inaturalist.org/observations/121998345
501	Spilopelia chinensis	Eastern Spotted Dove	2021/6/2	4	0	11.94468432	75.38493812	https://www.inaturalist.org/observations/126191291
502	Spilopelia chinensis	Eastern Spotted Dove	2022/7/15	1	0	11.94468432	75.38493812	https://www.inaturalist.org/observations/127094489
503	Spilopelia chinensis	Eastern Spotted Dove	2022/7/16	2	0	11.94468432	75.38493812	https://www.inaturalist.org/observations/127098692
504	Spilopelia chinensis	Eastern Spotted Dove	2022/7/15	4	0	11.94468432	75.38493812	https://www.inaturalist.org/observations/127101796
505	Spilopelia chinensis	Eastern Spotted Dove	2021/6/2	2	0	11.94468432	75.38493812	https://www.inaturalist.org/observations/127203772

506	Spilopelia chinensis	Eastern Spotted Dove	2021/6/4	1	0	11.94468432	75.38493812	https://www.inaturalist.org/observations/127349417
507	Spilopelia chinensis	Eastern Spotted Dove	2021/6/4	1	0	11.94468432	75.38493812	https://www.inaturalist.org/observations/127349682
508	Spilopelia chinensis	Eastern Spotted Dove	2022/7/5	2	0	11.94468432	75.38493812	https://www.inaturalist.org/observations/127470815
509	Spilopelia chinensis	Eastern Spotted Dove	2022/7/5	2	0	11.94468432	75.38493812	https://www.inaturalist.org/observations/127470878
510	Spilopelia chinensis	Eastern Spotted Dove	2022/6/6	2	0	11.94468432	75.38493812	https://www.inaturalist.org/observations/127975758
511	Spilopelia chinensis	Eastern Spotted Dove	2022/6/6	1	0	11.94468432	75.38493812	https://www.inaturalist.org/observations/127975851
512	Spilopelia chinensis	Eastern Spotted Dove	2022/6/6	2	0	11.94468432	75.38493812	https://www.inaturalist.org/observations/127975927
513	Spilopelia chinensis	Eastern Spotted Dove	2022/8/20	3	0	11.94468432	75.38493812	https://www.inaturalist.org/observations/135645245
514	Spilopelia chinensis	Eastern Spotted Dove	2022/8/25	2	0	11.94468432	75.38493812	https://www.inaturalist.org/observations/135886775
515	Spilopelia chinensis	Eastern Spotted Dove	2022/8/25	2	0	11.94468432	75.38493812	https://www.inaturalist.org/observations/135886782
516	Spilopelia chinensis	Eastern Spotted Dove	2022/8/25	2	0	11.94468432	75.38493812	https://www.inaturalist.org/observations/135886802
517	Spilopelia chinensis	Eastern Spotted Dove	2020/5/9	3	0	11.94474565	75.38493812	https://www.inaturalist.org/observations/45592396
518	Spilopelia chinensis	Eastern Spotted Dove	2022/8/5	3	0	11.95245630	77.04970618	https://www.inaturalist.org/observations/129807841
519	Spilopelia chinensis	Eastern Spotted Dove	2017/7/14	2	0	11.98427631	75.39945457	https://www.inaturalist.org/observations/44400493
520	Spilopelia chinensis	Eastern Spotted Dove	2020/11/7	4	0	11.98741331	75.32260627	https://www.inaturalist.org/observations/64352649
521	Spilopelia chinensis	Eastern Spotted Dove	2021/4/28	1	0	11.98810401	75.32233771	https://www.inaturalist.org/observations/75516536
522	Spilopelia chinensis	Eastern Spotted Dove	2021/4/27	2	0	11.98831423	75.32184117	https://www.inaturalist.org/observations/75403030
523	Spilopelia chinensis	Eastern Spotted Dove	2021/7/7	2	0	11.98838573	75.32161318	https://www.inaturalist.org/observations/86027940
524	Spilopelia chinensis	Eastern Spotted Dove	2019/12/28	1	0	11.98977400	76.37704100	https://www.inaturalist.org/observations/40870655
525	Spilopelia chinensis	Eastern Spotted Dove	2020/10/5	3	0	11.99509856	79.82263435	https://www.inaturalist.org/observations/62022723
526	Spilopelia chinensis	Eastern Spotted Dove	2006/8/29	5	0	11.99877500	77.13983110	https://www.inaturalist.org/observations/31928286
527	Spilopelia chinensis	Eastern Spotted Dove	2014/6/23	1	0	11.99877500	77.13983110	https://www.inaturalist.org/observations/86172370
528	Spilopelia chinensis	Eastern Spotted Dove	2015/10/31	1	0	11.99877500	77.13983110	https://www.inaturalist.org/observations/86172395

529	Spilopelia chinensis	Eastern Spotted Dove	2019/6/3	7	0	12.02524960	75.50335765	https://www.inaturalist.org/observations/26528233
530	Spilopelia chinensis	Eastern Spotted Dove	2022/6/22	2	0	12.02665167	75.26125000	https://www.inaturalist.org/observations/126932680
531	Spilopelia chinensis	Eastern Spotted Dove	2019/5/4	5	0	12.02960500	75.25159000	https://www.inaturalist.org/observations/29242844
532	Spilopelia chinensis	Eastern Spotted Dove	2017/3/17	3	0	12.03000078	75.20706394	https://www.inaturalist.org/observations/30603887
533	Spilopelia chinensis	Eastern Spotted Dove	2017/3/17	3	0	12.03000078	75.20706394	https://www.inaturalist.org/observations/30603894
534	Spilopelia chinensis	Eastern Spotted Dove	2022/6/25	3	0	12.03004333	75.25172000	https://www.inaturalist.org/observations/126933068
535	Spilopelia chinensis	Eastern Spotted Dove	2019/9/5	3	0	12.03083000	75.25211333	https://www.inaturalist.org/observations/32201060
536	Spilopelia chinensis	Eastern Spotted Dove	2022/6/12	2	0	12.03234633	75.25665645	https://www.inaturalist.org/observations/130749587
537	Spilopelia chinensis	Eastern Spotted Dove	2019/10/15	3	0	12.03234670	75.25665640	https://www.inaturalist.org/observations/34574260
538	Spilopelia chinensis	Eastern Spotted Dove	2021/9/11	1	0	12.03234670	75.25665640	https://www.inaturalist.org/observations/94542605
539	Spilopelia chinensis	Eastern Spotted Dove	2017/5/18	3	0	12.03391833	75.19422833	https://www.inaturalist.org/observations/30658452
540	Spilopelia chinensis	Eastern Spotted Dove	2017/3/25	5	0	12.03414500	75.19368333	https://www.inaturalist.org/observations/30664118
541	Spilopelia chinensis	Eastern Spotted Dove	2022/6/22	3	0	12.03574333	75.25799000	https://www.inaturalist.org/observations/126932770
542	Spilopelia chinensis	Eastern Spotted Dove	2020/4/6	2	0	12.03683768	75.24725094	https://www.inaturalist.org/observations/45573604
543	Spilopelia chinensis	Eastern Spotted Dove	2022/6/22	2	0	12.03728833	75.25807167	https://www.inaturalist.org/observations/126932743
544	Spilopelia chinensis	Eastern Spotted Dove	2022/6/22	3	0	12.03824167	75.25788167	https://www.inaturalist.org/observations/126932708
545	Spilopelia chinensis	Eastern Spotted Dove	2020/2/6	4	0	12.03997500	75.25872833	https://www.inaturalist.org/observations/67039944
546	Spilopelia chinensis	Eastern Spotted Dove	2021/2/21	3	0	12.04009244	75.29748503	https://www.inaturalist.org/observations/82039107
547	Spilopelia chinensis	Eastern Spotted Dove	2019/9/5	3	0	12.04595500	75.29944833	https://www.inaturalist.org/observations/32201148
548	Spilopelia chinensis	Eastern Spotted Dove	2013/10/30	7	0	12.05128200	79.87603400	http://www.inaturalist.org/observations/444342
549	Spilopelia chinensis	Eastern Spotted Dove	2017/1/1	4	0	12.05498333	75.20164833	https://www.inaturalist.org/observations/30660607
550	Spilopelia chinensis	Eastern Spotted Dove	2017/2/18	4	0	12.05517667	75.20043500	https://www.inaturalist.org/observations/30660629
551	Spilopelia chinensis	Eastern Spotted Dove	2020/4/29	1	0	12.05749173	75.20062657	https://www.inaturalist.org/observations/44273916

552	Spilopelia chinensis	Eastern Spotted Dove	2017/10/18	4	0	12.06395452	75.19410296	https://www.inaturalist.org/observations/30601933
553	Spilopelia chinensis	Eastern Spotted Dove	2014/10/25	1	0	12.08230054	76.71351780	https://www.inaturalist.org/observations/95515519
554	Spilopelia chinensis	Eastern Spotted Dove	2019/7/4	3	0	12.10229667	75.23720500	https://www.inaturalist.org/observations/29236194
555	Spilopelia chinensis	Eastern Spotted Dove	2020/2/17	1	0	12.10328000	75.23647833	https://www.inaturalist.org/observations/39321568
556	Spilopelia chinensis	Eastern Spotted Dove	2021/10/2	0	0	12.10347138	75.23360022	https://www.inaturalist.org/observations/96904677
557	Spilopelia chinensis	Eastern Spotted Dove	2021/2/18	1	0	12.14058883	75.21942017	https://www.inaturalist.org/observations/69754910
558	Spilopelia chinensis	Eastern Spotted Dove	2022/7/22	2	0	12.14382373	75.93982179	https://www.inaturalist.org/observations/127330147
559	Spilopelia chinensis	Eastern Spotted Dove	2020/2/3	0	0	12.14454516	75.93820442	https://www.inaturalist.org/observations/38260190
560	Spilopelia chinensis	Eastern Spotted Dove	2022/3/8	3	0	12.14945333	75.19513833	https://www.inaturalist.org/observations/115189684
561	Spilopelia chinensis	Eastern Spotted Dove	2021/9/12	1	0	12.14979833	75.19504502	https://www.inaturalist.org/observations/95049462
562	Spilopelia chinensis	Eastern Spotted Dove	2018/5/5	2	0	12.15093825	76.52898616	https://www.inaturalist.org/observations/90641715
563	Spilopelia chinensis	Eastern Spotted Dove	2022/3/8	3	0	12.15114500	75.19610500	https://www.inaturalist.org/observations/115189701
564	Spilopelia chinensis	Eastern Spotted Dove	2022/1/20	1	0	12.15146074	75.94302770	https://www.inaturalist.org/observations/106382747
565	Spilopelia chinensis	Eastern Spotted Dove	2022/4/14	3	0	12.15150466	75.94302770	https://www.inaturalist.org/observations/131546366
566	Spilopelia chinensis	Eastern Spotted Dove	2012/9/2	7	0	12.20010501	77.58948561	https://www.inaturalist.org/observations/66363228
567	Spilopelia chinensis	Eastern Spotted Dove	2021/9/10	1	0	12.20925291	75.73551352	https://www.inaturalist.org/observations/95252412
568	Spilopelia chinensis	Eastern Spotted Dove	2016/1/3	1	0	12.22528410	79.07469570	https://www.inaturalist.org/observations/96338221
569	Spilopelia chinensis	Eastern Spotted Dove	2020/12/20	2	0	12.25227833	75.27653833	https://www.inaturalist.org/observations/66994047
570	Spilopelia chinensis	Eastern Spotted Dove	2018/3/8	5	0	12.26937200	76.61197800	https://www.inaturalist.org/observations/10138018
571	Spilopelia chinensis	Eastern Spotted Dove	2018/1/20	1	0	12.26937200	76.61197800	https://www.inaturalist.org/observations/93867733
572	Spilopelia chinensis	Eastern Spotted Dove	2012/1/14	1	0	12.26937200	76.61197800	https://www.inaturalist.org/observations/94711066
573	Spilopelia chinensis	Eastern Spotted Dove	2014/9/6	1	0	12.26937200	76.61197800	https://www.inaturalist.org/observations/94790685
574	Spilopelia chinensis	Eastern Spotted Dove	2014/6/8	1	0	12.26937200	76.61197800	https://www.inaturalist.org/observations/94817201

575	Spilopelia chinensis	Eastern Spotted Dove	2009/12/31	1	0	12.26937200	76.61197800	https://www.inaturalist.org/observations/94821397
576	Spilopelia chinensis	Eastern Spotted Dove	2014/5/25	2	0	12.26937200	76.61197800	https://www.inaturalist.org/observations/94834958
577	Spilopelia chinensis	Eastern Spotted Dove	2021/12/16	3	0	12.26937200	76.61197800	https://www.inaturalist.org/observations/103140743
578	Spilopelia chinensis	Eastern Spotted Dove	2022/1/30	1	0	12.26937200	76.61197800	https://www.inaturalist.org/observations/105954078
579	Spilopelia chinensis	Eastern Spotted Dove	2021/9/8	2	0	12.27042491	76.77563072	https://www.inaturalist.org/observations/94095180
580	Spilopelia chinensis	Eastern Spotted Dove	2018/2/24	4	0	12.27057398	76.61383270	https://www.inaturalist.org/observations/10030289
581	Spilopelia chinensis	Eastern Spotted Dove	2018/12/30	4	0	12.27238500	76.61419667	https://www.inaturalist.org/observations/19331415
582	Spilopelia chinensis	Eastern Spotted Dove	2014/7/26	2	0	12.27525460	76.67014750	https://www.inaturalist.org/observations/87711998
583	Spilopelia chinensis	Eastern Spotted Dove	2017/9/21	3	0	12.27525460	76.67014750	https://www.inaturalist.org/observations/88501806
584	Spilopelia chinensis	Eastern Spotted Dove	2020/10/22	2	0	12.27525460	76.67014750	https://www.inaturalist.org/observations/88506960
585	Spilopelia chinensis	Eastern Spotted Dove	2019/2/25	5	0	12.27677739	76.61203234	https://www.inaturalist.org/observations/69669064
586	Spilopelia chinensis	Eastern Spotted Dove	2020/6/13	2	0	12.27936203	76.58896975	https://www.inaturalist.org/observations/92779670
587	Spilopelia chinensis	Eastern Spotted Dove	2022/9/21	2	0	12.28217170	76.59072926	https://www.inaturalist.org/observations/136249232
588	Spilopelia chinensis	Eastern Spotted Dove	2018/2/12	5	0	12.28300014	76.61500451	https://www.inaturalist.org/observations/9864526
589	Spilopelia chinensis	Eastern Spotted Dove	2020/7/24	1	0	12.28607705	76.61092624	https://www.inaturalist.org/observations/95925083
590	Spilopelia chinensis	Eastern Spotted Dove	2022/9/4	3	0	12.28706207	77.84838524	https://www.inaturalist.org/observations/133581614
591	Spilopelia chinensis	Eastern Spotted Dove	2015/5/23	6	0	12.28919600	76.56372100	http://www.inaturalist.org/observations/1530071
592	Spilopelia chinensis	Eastern Spotted Dove	2020/8/17	1	0	12.28966880	76.60732135	https://www.inaturalist.org/observations/96066508
593	Spilopelia chinensis	Eastern Spotted Dove	2022/6/18	5	0	12.29248167	76.61502833	https://www.inaturalist.org/observations/123187192
594	Spilopelia chinensis	Eastern Spotted Dove	2021/11/5	1	0	12.29355833	76.57409451	https://www.inaturalist.org/observations/100308392
595	Spilopelia chinensis	Eastern Spotted Dove	2019/10/6	2	0	12.29581040	76.63938050	https://www.inaturalist.org/observations/100075203
596	Spilopelia chinensis	Eastern Spotted Dove	2019/1/20	3	0	12.30753930	76.63534507	https://www.inaturalist.org/observations/19780268
597	Spilopelia chinensis	Eastern Spotted Dove	2016/12/25	1	0	12.31016778	76.63113076	https://www.inaturalist.org/observations/93559164

598	Spilopelia chinensis	Eastern Spotted Dove	2019/5/20	1	0	12.31016778	76.63113076	https://www.inaturalist.org/observations/93559531
599	Spilopelia chinensis	Eastern Spotted Dove	2021/8/20	2	0	12.31193100	77.27692050	https://www.inaturalist.org/observations/106424529
600	Spilopelia chinensis	Eastern Spotted Dove	2018/5/26	4	0	12.31404667	76.62966000	https://www.inaturalist.org/observations/12820451
601	Spilopelia chinensis	Eastern Spotted Dove	2018/7/22	2	0	12.31685280	76.59830560	https://www.inaturalist.org/observations/86150189
602	Spilopelia chinensis	Eastern Spotted Dove	2021/8/30	1	0	12.31685280	76.59830560	https://www.inaturalist.org/observations/93030178
603	Spilopelia chinensis	Eastern Spotted Dove	2020/2/17	1	0	12.31716500	75.10982500	https://www.inaturalist.org/observations/39321536
604	Spilopelia chinensis	Eastern Spotted Dove	2012/2/18	1	0	12.32531077	76.67042708	https://www.inaturalist.org/observations/48926219
605	Spilopelia chinensis	Eastern Spotted Dove	2020/4/2	2	0	12.32630220	76.61146101	https://www.inaturalist.org/observations/41348861
606	Spilopelia chinensis	Eastern Spotted Dove	2022/7/30	3	0	12.35073910	77.64725740	https://www.inaturalist.org/observations/128611422
607	Spilopelia chinensis	Eastern Spotted Dove	2019/5/17	1	0	12.35806928	76.61007194	https://www.inaturalist.org/observations/91852316
608	Spilopelia chinensis	Eastern Spotted Dove	2020/3/1	2	0	12.36009865	76.54254396	https://www.inaturalist.org/observations/39528543
609	Spilopelia chinensis	Eastern Spotted Dove	2012/5/4	8	0	12.36012451	76.61109924	http://www.inaturalist.org/observations/122109
610	Spilopelia chinensis	Eastern Spotted Dove	2011/6/24	1	0	12.37043930	76.48801310	https://www.inaturalist.org/observations/96097670
611	Spilopelia chinensis	Eastern Spotted Dove	2019/12/23	1	0	12.39444281	76.77467678	https://www.inaturalist.org/observations/38053177
612	Spilopelia chinensis	Eastern Spotted Dove	2011/6/19	2	0	12.41666670	77.75000000	https://www.inaturalist.org/observations/63564395
613	Spilopelia chinensis	Eastern Spotted Dove	2011/6/19	4	0	12.41666670	77.75000000	https://www.inaturalist.org/observations/64120797
614	Spilopelia chinensis	Eastern Spotted Dove	2013/6/19	2	0	12.41666670	77.75000000	https://www.inaturalist.org/observations/64164551
615	Spilopelia chinensis	Eastern Spotted Dove	2019/12/25	1	0	12.42183100	75.91892400	https://www.inaturalist.org/observations/40687912
616	Spilopelia chinensis	Eastern Spotted Dove	2019/1/22	4	0	12.42370709	76.65633436	https://www.inaturalist.org/observations/19820748
617	Spilopelia chinensis	Eastern Spotted Dove	2020/11/22	4	0	12.42738666	77.65511524	https://www.inaturalist.org/observations/65467262
618	Spilopelia chinensis	Eastern Spotted Dove	2016/8/14	0	0	12.42952672	77.65473184	https://www.inaturalist.org/observations/65137436
619	Spilopelia chinensis	Eastern Spotted Dove	2022/2/6	1	0	12.43756642	75.74141583	https://www.inaturalist.org/observations/112939324
620	Spilopelia chinensis	Eastern Spotted Dove	2017/9/22	3	0	12.45551770	75.95702340	https://www.inaturalist.org/observations/109829864

621	Spilopelia chinensis	Eastern Spotted Dove	2015/3/15	6	0	12.46700000	75.96700000	http://www.inaturalist.org/observations/1366852
622	Spilopelia chinensis	Eastern Spotted Dove	2021/11/16	2	0	12.48648902	75.67278439	https://www.inaturalist.org/observations/102292452
623	Spilopelia chinensis	Eastern Spotted Dove	2022/8/5	3	0	12.50065167	77.61493833	https://www.inaturalist.org/observations/129513545
624	Spilopelia chinensis	Eastern Spotted Dove	2020/12/13	2	0	12.53765667	80.10910342	https://www.inaturalist.org/observations/78228076
625	Spilopelia chinensis	Eastern Spotted Dove	2022/2/20	5	0	12.54341786	74.96370022	https://www.inaturalist.org/observations/107700577
626	Spilopelia chinensis	Eastern Spotted Dove	2021/11/16	2	0	12.56258631	75.71239223	https://www.inaturalist.org/observations/102292470
627	Spilopelia chinensis	Eastern Spotted Dove	2018/7/27	4	0	12.57606639	79.82380306	https://www.inaturalist.org/observations/22503061
628	Spilopelia chinensis	Eastern Spotted Dove	2022/6/22	4	0	12.58107667	76.17493667	https://www.inaturalist.org/observations/124899537
629	Spilopelia chinensis	Eastern Spotted Dove	2021/10/16	1	0	12.59844046	77.94741365	https://www.inaturalist.org/observations/98356348
630	Spilopelia chinensis	Eastern Spotted Dove	2022/6/21	5	0	12.61327000	76.08523000	https://www.inaturalist.org/observations/124086970
631	Spilopelia chinensis	Eastern Spotted Dove	2021/7/2	1	0	12.61706667	77.73832333	https://www.inaturalist.org/observations/85582630
632	Spilopelia chinensis	Eastern Spotted Dove	2022/6/21	4	0	12.62108333	76.05286833	https://www.inaturalist.org/observations/123239332
633	Spilopelia chinensis	Eastern Spotted Dove	2021/11/28	1	0	12.62326582	77.94588290	https://www.inaturalist.org/observations/102135378
634	Spilopelia chinensis	Eastern Spotted Dove	2021/10/3	2	0	12.62829834	74.94471985	https://www.inaturalist.org/observations/97151857
635	Spilopelia chinensis	Eastern Spotted Dove	2020/4/26	3	0	12.63000000	74.98000000	https://www.inaturalist.org/observations/44238322
636	Spilopelia chinensis	Eastern Spotted Dove	2020/7/25	1	0	12.63000000	74.98000000	https://www.inaturalist.org/observations/54363432
637	Spilopelia chinensis	Eastern Spotted Dove	2021/9/8	2	0	12.65550130	77.66681110	https://www.inaturalist.org/observations/103202534
638	Spilopelia chinensis	Eastern Spotted Dove	2021/10/3	2	0	12.67749487	77.91260335	https://www.inaturalist.org/observations/96993885
639	Spilopelia chinensis	Eastern Spotted Dove	2021/10/10	2	0	12.68003667	77.91864667	https://www.inaturalist.org/observations/97753486
640	Spilopelia chinensis	Eastern Spotted Dove	2022/8/14	3	0	12.68004333	77.91831000	https://www.inaturalist.org/observations/130746563
641	Spilopelia chinensis	Eastern Spotted Dove	2020/3/26	3	0	12.68371110	77.81224680	https://www.inaturalist.org/observations/55927205
642	Spilopelia chinensis	Eastern Spotted Dove	2022/2/19	2	0	12.71264500	77.80773333	https://www.inaturalist.org/observations/107120038
643	Spilopelia chinensis	Eastern Spotted Dove	2022/5/8	2	0	12.71382000	77.81439167	https://www.inaturalist.org/observations/119070837

644	Spilopelia chinensis	Eastern Spotted Dove	2022/1/21	1	0	12.71384972	77.81438833	https://www.inaturalist.org/observations/106980828
645	Spilopelia chinensis	Eastern Spotted Dove	2022/1/21	1	0	12.71384972	77.81438833	https://www.inaturalist.org/observations/106980831
646	Spilopelia chinensis	Eastern Spotted Dove	2022/1/29	1	0	12.71384972	77.81438833	https://www.inaturalist.org/observations/106980862
647	Spilopelia chinensis	Eastern Spotted Dove	2022/5/7	2	0	12.71385167	77.81439500	https://www.inaturalist.org/observations/115847657
648	Spilopelia chinensis	Eastern Spotted Dove	2022/5/8	2	0	12.71385833	77.81434500	https://www.inaturalist.org/observations/119070824
649	Spilopelia chinensis	Eastern Spotted Dove	2022/5/7	4	0	12.71387333	77.81437667	https://www.inaturalist.org/observations/115847636
650	Spilopelia chinensis	Eastern Spotted Dove	2015/4/4	4	0	12.72048776	77.56077331	https://www.inaturalist.org/observations/52037607
651	Spilopelia chinensis	Eastern Spotted Dove	2021/6/25	1	0	12.72654817	77.99171190	https://www.inaturalist.org/observations/84660636
652	Spilopelia chinensis	Eastern Spotted Dove	2020/12/31	4	0	12.75320165	77.30136741	https://www.inaturalist.org/observations/67378418
653	Spilopelia chinensis	Eastern Spotted Dove	2022/5/7	4	0	12.75736983	77.86978615	https://www.inaturalist.org/observations/119229940
654	Spilopelia chinensis	Eastern Spotted Dove	2022/4/24	0	0	12.76971000	77.87755833	https://www.inaturalist.org/observations/112636769
655	Spilopelia chinensis	Eastern Spotted Dove	2022/7/6	3	0	12.77371074	77.61810542	https://www.inaturalist.org/observations/124990229
656	Spilopelia chinensis	Eastern Spotted Dove	2021/12/12	1	0	12.77821635	77.82059743	https://www.inaturalist.org/observations/102921100
657	Spilopelia chinensis	Eastern Spotted Dove	2021/12/12	1	0	12.78532306	77.95126050	https://www.inaturalist.org/observations/102917205
658	Spilopelia chinensis	Eastern Spotted Dove	2021/10/29	2	0	12.78806036	76.93665770	https://www.inaturalist.org/observations/99774533
659	Spilopelia chinensis	Eastern Spotted Dove	2022/5/4	3	0	12.79134284	77.82513249	https://www.inaturalist.org/observations/119229904
660	Spilopelia chinensis	Eastern Spotted Dove	2022/7/28	3	0	12.80324282	77.56302930	https://www.inaturalist.org/observations/128593115
661	Spilopelia chinensis	Eastern Spotted Dove	2022/6/30	4	0	12.80725762	77.56099720	https://www.inaturalist.org/observations/124705284
662	Spilopelia chinensis	Eastern Spotted Dove	2019/8/12	1	0	12.80738909	76.73563921	https://www.inaturalist.org/observations/95275792
663	Spilopelia chinensis	Eastern Spotted Dove	2017/6/11	5	0	12.80783943	77.56465430	https://www.inaturalist.org/observations/69361758
664	Spilopelia chinensis	Eastern Spotted Dove	2022/2/9	2	0	12.80834931	77.52646055	https://www.inaturalist.org/observations/106440952
665	Spilopelia chinensis	Eastern Spotted Dove	2022/2/3	1	0	12.80880063	77.52785474	https://www.inaturalist.org/observations/106078045
666	Spilopelia chinensis	Eastern Spotted Dove	2021/10/2	1	0	12.80965801	77.62658456	https://www.inaturalist.org/observations/97729377

667	Spilopelia chinensis	Eastern Spotted Dove	2018/1/14	0	0	12.81385331	76.94609644	https://www.inaturalist.org/observations/96773263
668	Spilopelia chinensis	Eastern Spotted Dove	2020/6/1	2	0	12.81385331	76.94609644	https://www.inaturalist.org/observations/96783997
669	Spilopelia chinensis	Eastern Spotted Dove	2019/4/22	2	0	12.82552510	77.03485730	https://www.inaturalist.org/observations/92326550
670	Spilopelia chinensis	Eastern Spotted Dove	2019/6/9	2	0	12.82552510	77.03485730	https://www.inaturalist.org/observations/92329138
671	Spilopelia chinensis	Eastern Spotted Dove	2022/6/26	2	0	12.82565567	77.73247457	https://www.inaturalist.org/observations/127757533
672	Spilopelia chinensis	Eastern Spotted Dove	2020/8/16	3	0	12.83853523	77.65206955	https://www.inaturalist.org/observations/84542209
673	Spilopelia chinensis	Eastern Spotted Dove	2020/8/25	3	0	12.83864704	77.65198675	https://www.inaturalist.org/observations/84538562
674	Spilopelia chinensis	Eastern Spotted Dove	2022/2/26	1	0	12.84162626	77.77524635	https://www.inaturalist.org/observations/107467074
675	Spilopelia chinensis	Eastern Spotted Dove	2022/7/29	6	0	12.84240270	77.72698600	https://www.inaturalist.org/observations/132941581
676	Spilopelia chinensis	Eastern Spotted Dove	2021/8/16	1	0	12.84275000	77.72446300	https://www.inaturalist.org/observations/92644365
677	Spilopelia chinensis	Eastern Spotted Dove	2022/1/5	5	0	12.84275000	77.72446300	https://www.inaturalist.org/observations/104404248
678	Spilopelia chinensis	Eastern Spotted Dove	2022/2/1	2	0	12.84275000	77.72446300	https://www.inaturalist.org/observations/105953541
679	Spilopelia chinensis	Eastern Spotted Dove	2017/8/13	2	0	12.84290300	77.47106450	https://www.inaturalist.org/observations/66178140
680	Spilopelia chinensis	Eastern Spotted Dove	2021/11/21	1	0	12.84303427	80.17864268	https://www.inaturalist.org/observations/101611060
681	Spilopelia chinensis	Eastern Spotted Dove	2022/2/20	4	0	12.84463597	77.77340200	https://www.inaturalist.org/observations/107361829
682	Spilopelia chinensis	Eastern Spotted Dove	2022/4/3	1	0	12.84597197	77.77160997	https://www.inaturalist.org/observations/110264381
683	Spilopelia chinensis	Eastern Spotted Dove	2020/11/7	1	0	12.84849973	76.80860198	https://www.inaturalist.org/observations/97213467
684	Spilopelia chinensis	Eastern Spotted Dove	2022/6/10	1	0	12.85061333	77.77928833	https://www.inaturalist.org/observations/121093875
685	Spilopelia chinensis	Eastern Spotted Dove	2021/7/27	1	0	12.85081333	77.78189667	https://www.inaturalist.org/observations/88785764
686	Spilopelia chinensis	Eastern Spotted Dove	2018/8/5	4	0	12.85312333	77.50383833	https://www.inaturalist.org/observations/15161513
687	Spilopelia chinensis	Eastern Spotted Dove	2020/2/1	2	0	12.85400090	77.50871148	https://www.inaturalist.org/observations/61773024
688	Spilopelia chinensis	Eastern Spotted Dove	2022/1/2	1	0	12.85529794	77.50802483	https://www.inaturalist.org/observations/104173252
689	Spilopelia chinensis	Eastern Spotted Dove	2021/3/21	3	0	12.85718650	77.50891100	https://www.inaturalist.org/observations/71692536

690	Spilopelia chinensis	Eastern Spotted Dove	2021/10/31	1	0	12.85718650	77.50891100	https://www.inaturalist.org/observations/99861613
691	Spilopelia chinensis	Eastern Spotted Dove	2020/11/8	2	0	12.86523000	77.56809333	https://www.inaturalist.org/observations/64535854
692	Spilopelia chinensis	Eastern Spotted Dove	2020/9/26	2	0	12.86877172	77.64412656	https://www.inaturalist.org/observations/60988373
693	Spilopelia chinensis	Eastern Spotted Dove	2022/5/1	1	0	12.87381851	77.52592295	https://www.inaturalist.org/observations/114142704
694	Spilopelia chinensis	Eastern Spotted Dove	2020/12/12	1	0	12.87407590	77.52710920	https://www.inaturalist.org/observations/66456722
695	Spilopelia chinensis	Eastern Spotted Dove	2021/12/21	0	0	12.87414820	75.76445320	https://www.inaturalist.org/observations/103677707
696	Spilopelia chinensis	Eastern Spotted Dove	2021/12/21	1	0	12.87414820	75.76445320	https://www.inaturalist.org/observations/103677716
697	Spilopelia chinensis	Eastern Spotted Dove	2021/12/21	1	0	12.87414820	75.76445320	https://www.inaturalist.org/observations/103678231
698	Spilopelia chinensis	Eastern Spotted Dove	2021/12/24	2	0	12.87414820	75.76445320	https://www.inaturalist.org/observations/103683690
699	Spilopelia chinensis	Eastern Spotted Dove	2020/12/25	2	0	12.87420550	77.52724260	https://www.inaturalist.org/observations/67044249
700	Spilopelia chinensis	Eastern Spotted Dove	2020/6/6	1	0	12.87470852	77.52157137	https://www.inaturalist.org/observations/48625202
701	Spilopelia chinensis	Eastern Spotted Dove	2020/12/20	4	0	12.87498745	77.62998562	https://www.inaturalist.org/observations/66882289
702	Spilopelia chinensis	Eastern Spotted Dove	2020/10/25	4	0	12.87502333	77.58001833	https://www.inaturalist.org/observations/63558285
703	Spilopelia chinensis	Eastern Spotted Dove	2021/8/28	2	0	12.87864161	77.52998391	https://www.inaturalist.org/observations/92778168
704	Spilopelia chinensis	Eastern Spotted Dove	2020/12/26	4	0	12.87884033	77.52970496	https://www.inaturalist.org/observations/67081939
705	Spilopelia chinensis	Eastern Spotted Dove	2021/2/7	2	0	12.87888216	77.53004828	https://www.inaturalist.org/observations/69198756
706	Spilopelia chinensis	Eastern Spotted Dove	2018/8/4	4	0	12.87888700	77.52640200	https://www.inaturalist.org/observations/15102177
707	Spilopelia chinensis	Eastern Spotted Dove	2021/7/31	2	0	12.87890308	77.52986589	https://www.inaturalist.org/observations/89328262
708	Spilopelia chinensis	Eastern Spotted Dove	2021/2/28	1	0	12.87892400	77.53009120	https://www.inaturalist.org/observations/70749990
709	Spilopelia chinensis	Eastern Spotted Dove	2021/3/12	3	0	12.87892400	77.53009120	https://www.inaturalist.org/observations/71202352
710	Spilopelia chinensis	Eastern Spotted Dove	2021/3/20	2	0	12.87892400	77.53009120	https://www.inaturalist.org/observations/71611421
711	Spilopelia chinensis	Eastern Spotted Dove	2021/7/3	2	0	12.87892400	77.53009120	https://www.inaturalist.org/observations/85460239
712	Spilopelia chinensis	Eastern Spotted Dove	2022/2/5	2	0	12.87892400	77.53009120	https://www.inaturalist.org/observations/106171517

713	Spilopelia chinensis	Eastern Spotted Dove	2020/9/26	5	0	12.87894620	77.52977930	https://www.inaturalist.org/observations/60814569
714	Spilopelia chinensis	Eastern Spotted Dove	2020/10/10	2	0	12.87894620	77.52977930	https://www.inaturalist.org/observations/62180986
715	Spilopelia chinensis	Eastern Spotted Dove	2021/4/3	2	0	12.87896584	77.52959767	https://www.inaturalist.org/observations/72766381
716	Spilopelia chinensis	Eastern Spotted Dove	2020/11/28	2	0	12.87941685	77.52982221	https://www.inaturalist.org/observations/65761552
717	Spilopelia chinensis	Eastern Spotted Dove	2020/12/29	4	0	12.87946786	77.52971569	https://www.inaturalist.org/observations/67245052
718	Spilopelia chinensis	Eastern Spotted Dove	2020/10/2	3	0	12.87950540	79.40612680	https://www.inaturalist.org/observations/83090792
719	Spilopelia chinensis	Eastern Spotted Dove	2021/10/24	2	0	12.88013724	77.52939383	https://www.inaturalist.org/observations/99234731
720	Spilopelia chinensis	Eastern Spotted Dove	2020/10/24	3	0	12.88028500	77.52939300	https://www.inaturalist.org/observations/63388634
721	Spilopelia chinensis	Eastern Spotted Dove	2019/1/12	3	0	12.88035796	77.52937493	https://www.inaturalist.org/observations/19590306
722	Spilopelia chinensis	Eastern Spotted Dove	2021/1/24	3	0	12.88158836	79.12976425	https://www.inaturalist.org/observations/68551387
723	Spilopelia chinensis	Eastern Spotted Dove	2017/3/4	2	0	12.88259664	77.52360254	https://www.inaturalist.org/observations/64917453
724	Spilopelia chinensis	Eastern Spotted Dove	2021/8/28	2	0	12.88458640	77.46480580	https://www.inaturalist.org/observations/93390133
725	Spilopelia chinensis	Eastern Spotted Dove	2021/5/22	2	0	12.88541756	77.46574592	https://www.inaturalist.org/observations/80154185
726	Spilopelia chinensis	Eastern Spotted Dove	2016/4/16	1	0	12.88662979	77.67267399	https://www.inaturalist.org/observations/79310223
727	Spilopelia chinensis	Eastern Spotted Dove	2012/3/8	1	0	12.89041400	77.58719000	https://www.inaturalist.org/observations/86257060
728	Spilopelia chinensis	Eastern Spotted Dove	2021/4/18	1	0	12.89050000	77.56400000	https://www.inaturalist.org/observations/80296053
729	Spilopelia chinensis	Eastern Spotted Dove	2021/4/18	2	0	12.89050000	77.56400000	https://www.inaturalist.org/observations/80296054
730	Spilopelia chinensis	Eastern Spotted Dove	2019/9/15	2	0	12.89180091	79.10718043	https://www.inaturalist.org/observations/73587009
731	Spilopelia chinensis	Eastern Spotted Dove	2019/8/4	3	0	12.89308790	79.28769389	https://www.inaturalist.org/observations/69613684
732	Spilopelia chinensis	Eastern Spotted Dove	2021/8/6	1	0	12.89859382	77.54075289	https://www.inaturalist.org/observations/90119343
733	Spilopelia chinensis	Eastern Spotted Dove	2021/8/13	2	0	12.89861768	77.54075859	https://www.inaturalist.org/observations/90987745
734	Spilopelia chinensis	Eastern Spotted Dove	2021/6/30	2	0	12.89863958	77.54074886	https://www.inaturalist.org/observations/86963658
735	Spilopelia chinensis	Eastern Spotted Dove	2021/6/28	2	0	12.89864089	77.54076764	https://www.inaturalist.org/observations/86955123

736	Spilopelia chinensis	Eastern Spotted Dove	2021/8/16	1	0	12.89864415	77.54073646	https://www.inaturalist.org/observations/91394409
737	Spilopelia chinensis	Eastern Spotted Dove	2020/2/22	0	0	12.89878109	77.54062884	https://www.inaturalist.org/observations/39250637
738	Spilopelia chinensis	Eastern Spotted Dove	2020/6/1	3	0	12.89917930	77.66699090	https://www.inaturalist.org/observations/48154026
739	Spilopelia chinensis	Eastern Spotted Dove	2021/6/26	1	0	12.89950401	77.54156694	https://www.inaturalist.org/observations/86955015
740	Spilopelia chinensis	Eastern Spotted Dove	2020/9/26	2	0	12.90080270	80.21644568	https://www.inaturalist.org/observations/60819473
741	Spilopelia chinensis	Eastern Spotted Dove	2015/8/25	8	0	12.90206794	77.62719807	https://www.inaturalist.org/observations/17836220
742	Spilopelia chinensis	Eastern Spotted Dove	2020/8/20	3	0	12.90263889	79.11374722	https://www.inaturalist.org/observations/57304466
743	Spilopelia chinensis	Eastern Spotted Dove	2018/6/4	6	0	12.90470726	77.43077496	https://www.inaturalist.org/observations/13368640
744	Spilopelia chinensis	Eastern Spotted Dove	2022/9/11	0	0	12.90529248	80.20354606	https://www.inaturalist.org/observations/134604815
745	Spilopelia chinensis	Eastern Spotted Dove	2020/12/27	2	0	12.90556861	77.66945167	https://www.inaturalist.org/observations/67501563
746	Spilopelia chinensis	Eastern Spotted Dove	2020/7/9	2	0	12.90657222	77.67716389	https://www.inaturalist.org/observations/52433433
747	Spilopelia chinensis	Eastern Spotted Dove	2020/8/1	1	0	12.90666103	77.67614775	https://www.inaturalist.org/observations/55006703
748	Spilopelia chinensis	Eastern Spotted Dove	2022/4/28	2	0	12.90701639	75.66369139	https://www.inaturalist.org/observations/114661907
749	Spilopelia chinensis	Eastern Spotted Dove	2020/2/1	1	0	12.90893832	77.66249299	https://www.inaturalist.org/observations/38172927
750	Spilopelia chinensis	Eastern Spotted Dove	2020/4/15	2	0	12.90898530	77.49020970	https://www.inaturalist.org/observations/44570211
751	Spilopelia chinensis	Eastern Spotted Dove	2020/4/15	4	0	12.90898530	77.49020970	https://www.inaturalist.org/observations/44573067
752	Spilopelia chinensis	Eastern Spotted Dove	2019/12/15	4	0	12.91236155	77.67258916	https://www.inaturalist.org/observations/58830509
753	Spilopelia chinensis	Eastern Spotted Dove	2020/9/6	1	0	12.91269250	77.67329750	https://www.inaturalist.org/observations/58689211
754	Spilopelia chinensis	Eastern Spotted Dove	2019/9/26	2	0	12.91506285	77.66319036	https://www.inaturalist.org/observations/52767401
755	Spilopelia chinensis	Eastern Spotted Dove	2021/10/2	1	0	12.91509453	77.46969799	https://www.inaturalist.org/observations/96892170
756	Spilopelia chinensis	Eastern Spotted Dove	2021/3/3	3	0	12.91732262	77.66494074	https://www.inaturalist.org/observations/70548849
757	Spilopelia chinensis	Eastern Spotted Dove	2019/6/30	5	0	12.91769500	77.66520500	https://www.inaturalist.org/observations/28348835
758	Spilopelia chinensis	Eastern Spotted Dove	2018/11/18	2	0	12.91831260	77.68009700	https://www.inaturalist.org/observations/65007294

759	Spilopelia chinensis	Eastern Spotted Dove	2021/8/1	3	0	12.91889375	77.67817280	https://www.inaturalist.org/observations/90113590
760	Spilopelia chinensis	Eastern Spotted Dove	2021/6/18	2	0	12.92001561	77.64769351	https://www.inaturalist.org/observations/83630610
761	Spilopelia chinensis	Eastern Spotted Dove	2021/4/18	2	0	12.92002500	77.64769476	https://www.inaturalist.org/observations/80872080
762	Spilopelia chinensis	Eastern Spotted Dove	2022/4/29	2	0	12.92006810	77.64772743	https://www.inaturalist.org/observations/114628052
763	Spilopelia chinensis	Eastern Spotted Dove	2022/8/30	4	0	12.92024716	77.64776576	https://www.inaturalist.org/observations/133574277
764	Spilopelia chinensis	Eastern Spotted Dove	2022/9/26	2	0	12.92046917	77.69176472	https://www.inaturalist.org/observations/136768833
765	Spilopelia chinensis	Eastern Spotted Dove	2020/7/15	3	0	12.92288978	77.58277573	https://www.inaturalist.org/observations/53132419
766	Spilopelia chinensis	Eastern Spotted Dove	2021/5/19	6	0	12.92459872	77.58783361	https://www.inaturalist.org/observations/109357389
767	Spilopelia chinensis	Eastern Spotted Dove	2021/5/23	5	0	12.92462954	77.58797965	https://www.inaturalist.org/observations/109361810
768	Spilopelia chinensis	Eastern Spotted Dove	2021/5/23	4	0	12.92464382	77.58797711	https://www.inaturalist.org/observations/109357390
769	Spilopelia chinensis	Eastern Spotted Dove	2021/5/23	5	0	12.92467029	77.58798047	https://www.inaturalist.org/observations/109357391
770	Spilopelia chinensis	Eastern Spotted Dove	2021/1/11	4	0	12.93276500	77.53203101	https://www.inaturalist.org/observations/67946913
771	Spilopelia chinensis	Eastern Spotted Dove	2021/7/19	2	0	12.93297849	77.73646203	https://www.inaturalist.org/observations/87703117
772	Spilopelia chinensis	Eastern Spotted Dove	2021/1/21	2	0	12.93334577	79.23422425	https://www.inaturalist.org/observations/71931103
773	Spilopelia chinensis	Eastern Spotted Dove	2022/4/14	2	0	12.93441683	77.60601707	https://www.inaturalist.org/observations/111481483
774	Spilopelia chinensis	Eastern Spotted Dove	2019/2/2	1	0	12.93489120	80.21371240	https://www.inaturalist.org/observations/64436363
775	Spilopelia chinensis	Eastern Spotted Dove	2020/5/13	2	0	12.93531446	77.62448072	https://www.inaturalist.org/observations/46572173
776	Spilopelia chinensis	Eastern Spotted Dove	2022/8/9	1	0	12.93697740	77.56838990	https://www.inaturalist.org/observations/133053990
777	Spilopelia chinensis	Eastern Spotted Dove	2020/2/14	0	0	12.94242500	77.51245833	https://www.inaturalist.org/observations/38954370
778	Spilopelia chinensis	Eastern Spotted Dove	2020/11/11	3	0	12.94270833	77.57262500	https://www.inaturalist.org/observations/64785260
779	Spilopelia chinensis	Eastern Spotted Dove	2013/2/2	6	0	12.94415110	75.78656530	https://www.inaturalist.org/observations/28936142
780	Spilopelia chinensis	Eastern Spotted Dove	2021/7/20	3	0	12.94621080	74.92069340	https://www.inaturalist.org/observations/87958430
781	Spilopelia chinensis	Eastern Spotted Dove	2014/3/20	3	0	12.94657610	80.22376600	https://www.inaturalist.org/observations/22692267

782	Spilopelia chinensis	Eastern Spotted Dove	2020/12/29	3	0	12.94835134	79.11517912	https://www.inaturalist.org/observations/73007451
783	Spilopelia chinensis	Eastern Spotted Dove	2017/10/12	5	0	12.94883393	77.58704702	https://www.inaturalist.org/observations/8385069
784	Spilopelia chinensis	Eastern Spotted Dove	2020/2/12	0	0	12.95117500	77.51457500	https://www.inaturalist.org/observations/38945613
785	Spilopelia chinensis	Eastern Spotted Dove	2020/6/29	1	0	12.95785678	77.70670158	https://www.inaturalist.org/observations/51351769
786	Spilopelia chinensis	Eastern Spotted Dove	2020/6/30	1	0	12.95793950	77.70674475	https://www.inaturalist.org/observations/51463150
787	Spilopelia chinensis	Eastern Spotted Dove	2020/8/9	3	0	12.95796323	77.70672023	https://www.inaturalist.org/observations/55903009
788	Spilopelia chinensis	Eastern Spotted Dove	2020/8/8	1	0	12.95803981	77.70664598	https://www.inaturalist.org/observations/55768708
789	Spilopelia chinensis	Eastern Spotted Dove	2020/11/2	1	0	12.96765063	77.71209542	https://www.inaturalist.org/observations/64338017
790	Spilopelia chinensis	Eastern Spotted Dove	2018/9/14	4	0	12.97159870	77.59456270	https://www.inaturalist.org/observations/37222806
791	Spilopelia chinensis	Eastern Spotted Dove	2019/4/7	1	0	12.97159870	77.59456270	https://www.inaturalist.org/observations/56023058
792	Spilopelia chinensis	Eastern Spotted Dove	2019/4/7	1	0	12.97159870	77.59456270	https://www.inaturalist.org/observations/56023060
793	Spilopelia chinensis	Eastern Spotted Dove	2020/4/24	2	0	12.97159870	77.59456270	https://www.inaturalist.org/observations/56023062
794	Spilopelia chinensis	Eastern Spotted Dove	2021/9/15	4	0	12.97159870	77.59456270	https://www.inaturalist.org/observations/121526733
795	Spilopelia chinensis	Eastern Spotted Dove	2022/8/17	1	0	12.97159870	77.59456270	https://www.inaturalist.org/observations/136245327
796	Spilopelia chinensis	Eastern Spotted Dove	2021/1/10	2	0	12.97227898	77.59456269	https://www.inaturalist.org/observations/81796486
797	Spilopelia chinensis	Eastern Spotted Dove	2021/7/5	1	0	12.97262171	77.86725249	https://www.inaturalist.org/observations/85714024
798	Spilopelia chinensis	Eastern Spotted Dove	2022/5/10	3	0	12.97512424	77.68837206	https://www.inaturalist.org/observations/120647803
799	Spilopelia chinensis	Eastern Spotted Dove	2022/3/26	3	0	12.97615437	77.61759751	https://www.inaturalist.org/observations/109637715
800	Spilopelia chinensis	Eastern Spotted Dove	2018/9/29	4	0	12.97757833	77.68486167	https://www.inaturalist.org/observations/17019963
801	Spilopelia chinensis	Eastern Spotted Dove	2019/2/17	3	0	12.98141060	77.67007660	https://www.inaturalist.org/observations/20420922
802	Spilopelia chinensis	Eastern Spotted Dove	2021/9/30	1	0	12.98363483	77.67974094	https://www.inaturalist.org/observations/98024476
803	Spilopelia chinensis	Eastern Spotted Dove	2021/9/30	3	0	12.98363510	77.67974110	https://www.inaturalist.org/observations/96888560
804	Spilopelia chinensis	Eastern Spotted Dove	2021/12/9	3	0	12.98471949	77.57670287	https://www.inaturalist.org/observations/102762216

805	Spilopelia chinensis	Eastern Spotted Dove	2016/2/16	4	0	12.98587333	77.59097500	http://www.inaturalist.org/observations/2727266
806	Spilopelia chinensis	Eastern Spotted Dove	2018/10/14	3	0	12.99298331	77.61977043	https://www.inaturalist.org/observations/51035189
807	Spilopelia chinensis	Eastern Spotted Dove	2022/4/12	2	0	12.99801073	77.66139714	https://www.inaturalist.org/observations/111167127
808	Spilopelia chinensis	Eastern Spotted Dove	2022/2/15	2	0	13.00029229	80.26669834	https://www.inaturalist.org/observations/106842084
809	Spilopelia chinensis	Eastern Spotted Dove	2020/10/17	3	0	13.00352740	77.57794138	https://www.inaturalist.org/observations/62777959
810	Spilopelia chinensis	Eastern Spotted Dove	2021/9/23	1	0	13.00482500	77.61914667	https://www.inaturalist.org/observations/95831112
811	Spilopelia chinensis	Eastern Spotted Dove	2019/12/13	1	0	13.00980441	80.27526124	https://www.inaturalist.org/observations/45147403
812	Spilopelia chinensis	Eastern Spotted Dove	2021/11/26	1	0	13.01075039	77.55653253	https://www.inaturalist.org/observations/101989239
813	Spilopelia chinensis	Eastern Spotted Dove	2021/3/17	1	0	13.01745305	77.56859344	https://www.inaturalist.org/observations/105159382
814	Spilopelia chinensis	Eastern Spotted Dove	2019/9/1	1	0	13.01833608	78.33677237	https://www.inaturalist.org/observations/93050158
815	Spilopelia chinensis	Eastern Spotted Dove	2016/2/19	4	0	13.02182500	77.57092833	http://www.inaturalist.org/observations/2729051
816	Spilopelia chinensis	Eastern Spotted Dove	2021/9/22	1	0	13.02692379	77.54651225	https://www.inaturalist.org/observations/95724175
817	Spilopelia chinensis	Eastern Spotted Dove	2019/1/21	4	0	13.03353630	77.57907133	https://www.inaturalist.org/observations/108354716
818	Spilopelia chinensis	Eastern Spotted Dove	2022/3/2	4	0	13.03387070	77.57297736	https://www.inaturalist.org/observations/109911012
819	Spilopelia chinensis	Eastern Spotted Dove	2021/2/14	1	0	13.03448965	77.57224044	https://www.inaturalist.org/observations/105090561
820	Spilopelia chinensis	Eastern Spotted Dove	2021/2/17	1	0	13.03448965	77.57224044	https://www.inaturalist.org/observations/105090565
821	Spilopelia chinensis	Eastern Spotted Dove	2022/3/28	3	0	13.03468610	77.57302024	https://www.inaturalist.org/observations/113195994
822	Spilopelia chinensis	Eastern Spotted Dove	2021/2/8	1	0	13.04125000	77.56901389	https://www.inaturalist.org/observations/69235732
823	Spilopelia chinensis	Eastern Spotted Dove	2019/1/21	2	0	13.05054078	77.55335501	https://www.inaturalist.org/observations/103525132
824	Spilopelia chinensis	Eastern Spotted Dove	2021/9/19	1	0	13.05092740	77.60715710	https://www.inaturalist.org/observations/95376241
825	Spilopelia chinensis	Eastern Spotted Dove	2021/6/1	1	0	13.05633384	77.40826762	https://www.inaturalist.org/observations/81312781
826	Spilopelia chinensis	Eastern Spotted Dove	2022/1/14	4	0	13.05686824	77.53580153	https://www.inaturalist.org/observations/109361216
827	Spilopelia chinensis	Eastern Spotted Dove	2021/3/26	1	0	13.05956388	77.42572173	https://www.inaturalist.org/observations/72059782

828	Spilopelia chinensis	Eastern Spotted Dove	2022/7/20	1	0	13.06038369	77.65698319	https://www.inaturalist.org/observations/127202229
829	Spilopelia chinensis	Eastern Spotted Dove	2021/7/28	2	0	13.06045580	77.65708400	https://www.inaturalist.org/observations/88934139
830	Spilopelia chinensis	Eastern Spotted Dove	2021/2/28	1	0	13.06275397	77.74924837	https://www.inaturalist.org/observations/97246787
831	Spilopelia chinensis	Eastern Spotted Dove	2022/9/30	2	0	13.06373181	77.56247643	https://www.inaturalist.org/observations/136985544
832	Spilopelia chinensis	Eastern Spotted Dove	2021/3/20	3	0	13.06506800	77.64797197	https://www.inaturalist.org/observations/71608407
833	Spilopelia chinensis	Eastern Spotted Dove	2021/3/22	1	0	13.06510600	77.64790800	https://www.inaturalist.org/observations/71786915
834	Spilopelia chinensis	Eastern Spotted Dove	2016/3/27	3	0	13.06528285	77.56265280	https://www.inaturalist.org/observations/34053174
835	Spilopelia chinensis	Eastern Spotted Dove	2020/7/22	1	0	13.06529510	77.61480870	https://www.inaturalist.org/observations/53914647
836	Spilopelia chinensis	Eastern Spotted Dove	2021/9/1	1	0	13.06681667	77.55715278	https://www.inaturalist.org/observations/93447701
837	Spilopelia chinensis	Eastern Spotted Dove	2018/10/27	5	0	13.07029150	77.76952550	https://www.inaturalist.org/observations/18056071
838	Spilopelia chinensis	Eastern Spotted Dove	2016/9/24	5	0	13.07765910	77.58046590	http://www.inaturalist.org/observations/4211992
839	Spilopelia chinensis	Eastern Spotted Dove	2021/12/13	2	0	13.07847503	77.57789612	https://www.inaturalist.org/observations/103039170
840	Spilopelia chinensis	Eastern Spotted Dove	2015/1/14	5	0	13.07989186	77.58415235	https://www.inaturalist.org/observations/29638300
841	Spilopelia chinensis	Eastern Spotted Dove	2015/2/7	5	0	13.07997751	77.58384121	https://www.inaturalist.org/observations/29640354
842	Spilopelia chinensis	Eastern Spotted Dove	2020/4/24	2	0	13.08128186	77.64389231	https://www.inaturalist.org/observations/43871337
843	Spilopelia chinensis	Eastern Spotted Dove	2020/4/24	1	0	13.08157653	77.64400058	https://www.inaturalist.org/observations/43871351
844	Spilopelia chinensis	Eastern Spotted Dove	2020/4/24	1	0	13.08164169	77.64403733	https://www.inaturalist.org/observations/43871354
845	Spilopelia chinensis	Eastern Spotted Dove	2021/8/1	2	0	13.08268020	80.27071840	https://www.inaturalist.org/observations/89616121
846	Spilopelia chinensis	Eastern Spotted Dove	2021/9/19	2	0	13.08308237	80.27071830	https://www.inaturalist.org/observations/96450563
847	Spilopelia chinensis	Eastern Spotted Dove	2020/4/26	1	0	13.08336590	77.64049230	https://www.inaturalist.org/observations/43874998
848	Spilopelia chinensis	Eastern Spotted Dove	2020/4/26	1	0	13.08336590	77.64049230	https://www.inaturalist.org/observations/43875002
849	Spilopelia chinensis	Eastern Spotted Dove	2020/4/26	1	0	13.08336590	77.64049230	https://www.inaturalist.org/observations/43875004
850	Spilopelia chinensis	Eastern Spotted Dove	2019/12/20	3	0	13.08736710	77.60993500	https://www.inaturalist.org/observations/45945919

851	Spilopelia chinensis	Eastern Spotted Dove	2019/12/20	3	0	13.08736710	77.60993500	https://www.inaturalist.org/observations/45950756
852	Spilopelia chinensis	Eastern Spotted Dove	2019/12/20	3	0	13.08736710	77.60993500	https://www.inaturalist.org/observations/45950757
853	Spilopelia chinensis	Eastern Spotted Dove	2020/5/2	1	0	13.10567783	74.97238833	https://www.inaturalist.org/observations/64839814
854	Spilopelia chinensis	Eastern Spotted Dove	2018/12/15	1	0	13.11538315	77.50293732	https://www.inaturalist.org/observations/22642969
855	Spilopelia chinensis	Eastern Spotted Dove	2016/8/19	2	0	13.11841849	76.93742144	https://www.inaturalist.org/observations/92438078
856	Spilopelia chinensis	Eastern Spotted Dove	2021/7/30	2	0	13.13003667	80.19144000	https://www.inaturalist.org/observations/89427952
857	Spilopelia chinensis	Eastern Spotted Dove	2020/10/22	2	0	13.13443751	79.19192172	https://www.inaturalist.org/observations/75296741
858	Spilopelia chinensis	Eastern Spotted Dove	2021/2/18	1	0	13.13581669	77.52574988	https://www.inaturalist.org/observations/69754159
859	Spilopelia chinensis	Eastern Spotted Dove	2020/12/30	2	0	13.13628397	77.57092097	https://www.inaturalist.org/observations/69502975
860	Spilopelia chinensis	Eastern Spotted Dove	2021/3/8	2	0	13.15004332	77.49000013	https://www.inaturalist.org/observations/70800822
861	Spilopelia chinensis	Eastern Spotted Dove	2022/8/20	2	0	13.17101800	76.43101400	https://www.inaturalist.org/observations/131689605
862	Spilopelia chinensis	Eastern Spotted Dove	2020/9/18	3	0	13.22328762	77.19087549	https://www.inaturalist.org/observations/62285161
863	Spilopelia chinensis	Eastern Spotted Dove	2019/12/21	3	0	13.22648167	77.67459937	https://www.inaturalist.org/observations/46056422
864	Spilopelia chinensis	Eastern Spotted Dove	2021/11/7	1	0	13.25283620	76.52105530	https://www.inaturalist.org/observations/100492013
865	Spilopelia chinensis	Eastern Spotted Dove	2020/1/15	2	0	13.30336660	77.18673830	https://www.inaturalist.org/observations/38358223
866	Spilopelia chinensis	Eastern Spotted Dove	2020/10/20	3	0	13.31296638	77.54213657	https://www.inaturalist.org/observations/115744722
867	Spilopelia chinensis	Eastern Spotted Dove	2019/12/22	4	0	13.37041630	77.68430732	https://www.inaturalist.org/observations/46067013
868	Spilopelia chinensis	Eastern Spotted Dove	2020/12/27	2	0	13.50266350	75.09031540	https://www.inaturalist.org/observations/67999060
869	Spilopelia chinensis	Eastern Spotted Dove	2022/3/23	1	0	13.51900000	79.32344167	https://www.inaturalist.org/observations/111898679
870	Spilopelia chinensis	Eastern Spotted Dove	2022/9/24	1	0	13.56304800	79.30651000	https://www.inaturalist.org/observations/136408152
871	Spilopelia chinensis	Eastern Spotted Dove	2017/10/24	1	0	13.56508150	74.93695220	https://www.inaturalist.org/observations/41211257
872	Spilopelia chinensis	Eastern Spotted Dove	2021/9/11	2	0	13.57885933	79.37742871	https://www.inaturalist.org/observations/94405371
873	Spilopelia chinensis	Eastern Spotted Dove	2022/6/10	3	0	13.62492572	74.69310723	https://www.inaturalist.org/observations/125133102

874	Spilopelia chinensis	Eastern Spotted Dove	2022/2/7	3	0	13.62813000	79.37454000	https://www.inaturalist.org/observations/112129140
875	Spilopelia chinensis	Eastern Spotted Dove	2022/3/26	3	0	13.63040397	79.38621380	https://www.inaturalist.org/observations/111534944
876	Spilopelia chinensis	Eastern Spotted Dove	2022/3/26	3	0	13.63043218	79.38603503	https://www.inaturalist.org/observations/111534945
877	Spilopelia chinensis	Eastern Spotted Dove	2022/2/20	2	0	13.64311965	79.39796805	https://www.inaturalist.org/observations/108231004
878	Spilopelia chinensis	Eastern Spotted Dove	2022/3/9	3	0	13.65038429	79.46692914	https://www.inaturalist.org/observations/108228629
879	Spilopelia chinensis	Eastern Spotted Dove	2022/2/21	3	0	13.65048015	79.33129025	https://www.inaturalist.org/observations/109821256
880	Spilopelia chinensis	Eastern Spotted Dove	2022/6/12	3	0	13.65191802	79.41631128	https://www.inaturalist.org/observations/121892196
881	Spilopelia chinensis	Eastern Spotted Dove	2022/6/13	5	0	13.65322548	79.41788422	https://www.inaturalist.org/observations/122601849
882	Spilopelia chinensis	Eastern Spotted Dove	2020/3/23	2	0	13.65384647	74.93372638	https://www.inaturalist.org/observations/51989288
883	Spilopelia chinensis	Eastern Spotted Dove	2022/2/21	2	0	13.65620078	79.33500272	https://www.inaturalist.org/observations/112250410
884	Spilopelia chinensis	Eastern Spotted Dove	2021/5/2	1	0	13.65668000	79.26958000	https://www.inaturalist.org/observations/76480507
885	Spilopelia chinensis	Eastern Spotted Dove	2021/5/2	1	0	13.65682600	79.26944000	https://www.inaturalist.org/observations/76480522
886	Spilopelia chinensis	Eastern Spotted Dove	2021/5/2	2	0	13.65752400	79.27092100	https://www.inaturalist.org/observations/76480502
887	Spilopelia chinensis	Eastern Spotted Dove	2019/2/17	1	0	13.65793887	79.48242778	https://www.inaturalist.org/observations/55892158
888	Spilopelia chinensis	Eastern Spotted Dove	2022/9/27	3	0	13.74163700	79.60654500	https://www.inaturalist.org/observations/136707945
889	Spilopelia chinensis	Eastern Spotted Dove	2021/4/18	1	0	13.76268300	79.46905100	https://www.inaturalist.org/observations/74357759
890	Spilopelia chinensis	Eastern Spotted Dove	2017/4/8	2	0	13.84077511	75.51342264	https://www.inaturalist.org/observations/50272830
891	Spilopelia chinensis	Eastern Spotted Dove	2019/5/22	4	0	13.85308382	76.45052411	https://www.inaturalist.org/observations/132921971
892	Spilopelia chinensis	Eastern Spotted Dove	2016/4/26	5	0	13.85362750	75.52241470	https://www.inaturalist.org/observations/9989173
893	Spilopelia chinensis	Eastern Spotted Dove	2022/6/29	3	0	13.87184500	75.55673333	https://www.inaturalist.org/observations/125886469
894	Spilopelia chinensis	Eastern Spotted Dove	2011/9/18	2	0	14.22943890	74.81252290	https://www.inaturalist.org/observations/92573231
895	Spilopelia chinensis	Eastern Spotted Dove	2020/3/19	5	0	14.23268336	74.44286149	https://www.inaturalist.org/observations/40276694
896	Spilopelia chinensis	Eastern Spotted Dove	2019/12/24	2	0	14.23533847	74.44059804	https://www.inaturalist.org/observations/36934756

897	Spilopelia chinensis	Eastern Spotted Dove	2021/11/10	2	0	14.24893722	74.65306990	https://www.inaturalist.org/observations/100778538
898	Spilopelia chinensis	Eastern Spotted Dove	2020/11/8	3	0	14.64665467	74.90408605	https://www.inaturalist.org/observations/64510565
899	Spilopelia chinensis	Eastern Spotted Dove	2020/12/5	1	0	14.73555335	75.62983056	https://www.inaturalist.org/observations/90235001
900	Spilopelia chinensis	Eastern Spotted Dove	2022/8/29	3	0	14.75865909	74.92655497	https://www.inaturalist.org/observations/132908314
901	Spilopelia chinensis	Eastern Spotted Dove	2022/1/2	4	0	14.79370650	74.68688150	https://www.inaturalist.org/observations/121135396
902	Spilopelia chinensis	Eastern Spotted Dove	2021/2/13	1	0	14.99809980	74.35874730	https://www.inaturalist.org/observations/70862968
903	Spilopelia chinensis	Eastern Spotted Dove	2020/2/16	2	0	15.21376257	73.93572807	https://www.inaturalist.org/observations/39694712
904	Spilopelia chinensis	Eastern Spotted Dove	2020/4/15	1	0	15.21376257	73.93572807	https://www.inaturalist.org/observations/42250323
905	Spilopelia chinensis	Eastern Spotted Dove	2019/2/16	6	0	15.24967750	74.61737380	https://www.inaturalist.org/observations/30354683
906	Spilopelia chinensis	Eastern Spotted Dove	2018/11/13	6	0	15.29200100	74.52086100	https://www.inaturalist.org/observations/18586210
907	Spilopelia chinensis	Eastern Spotted Dove	2019/11/23	1	0	15.29932665	74.12399612	https://www.inaturalist.org/observations/36174574
908	Spilopelia chinensis	Eastern Spotted Dove	2008/11/9	2	0	15.39934400	73.84417300	https://www.inaturalist.org/observations/57421341
909	Spilopelia chinensis	Eastern Spotted Dove	2018/12/14	5	0	15.51577572	73.89166781	https://www.inaturalist.org/observations/20104377
910	Spilopelia chinensis	Eastern Spotted Dove	2015/12/31	1	0	15.91731100	75.68585400	https://www.inaturalist.org/observations/46196284
911	Spilopelia chinensis	Eastern Spotted Dove	2019/9/1	3	0	16.05845255	73.65312260	https://www.inaturalist.org/observations/32323179
912	Spilopelia chinensis	Eastern Spotted Dove	2021/12/6	2	0	16.26581320	78.85631371	https://www.inaturalist.org/observations/106477082
913	Spilopelia chinensis	Eastern Spotted Dove	2020/10/18	3	0	16.26615778	73.68010919	https://www.inaturalist.org/observations/63060145
914	Spilopelia chinensis	Eastern Spotted Dove	2019/8/19	4	0	16.30165617	80.43817928	https://www.inaturalist.org/observations/31401811
915	Spilopelia chinensis	Eastern Spotted Dove	2019/3/1	1	0	16.35570560	78.83346130	https://www.inaturalist.org/observations/87986710
916	Spilopelia chinensis	Eastern Spotted Dove	2020/7/18	2	0	16.36097700	74.29613500	https://www.inaturalist.org/observations/53445864
917	Spilopelia chinensis	Eastern Spotted Dove	2021/10/9	2	0	16.38617960	81.47662170	https://www.inaturalist.org/observations/97902113
918	Spilopelia chinensis	Eastern Spotted Dove	2013/9/30	2	0	16.41060994	80.70892527	https://www.inaturalist.org/observations/88105648
919	Spilopelia chinensis	Eastern Spotted Dove	2012/9/1	1	0	16.49730598	80.59512800	https://www.inaturalist.org/observations/93610164

920	Spilopelia chinensis	Eastern Spotted Dove	2012/3/29	2	0	16.49979669	80.59578562	https://www.inaturalist.org/observations/93593891
921	Spilopelia chinensis	Eastern Spotted Dove	2021/12/28	2	0	16.51799610	80.66480390	https://www.inaturalist.org/observations/103803529
922	Spilopelia chinensis	Eastern Spotted Dove	2022/1/2	1	0	16.51898000	81.51410000	https://www.inaturalist.org/observations/111197908
923	Spilopelia chinensis	Eastern Spotted Dove	2022/1/2	2	0	16.51903333	81.51410000	https://www.inaturalist.org/observations/104192847
924	Spilopelia chinensis	Eastern Spotted Dove	2022/1/2	2	0	16.51903333	81.51410000	https://www.inaturalist.org/observations/104348364
925	Spilopelia chinensis	Eastern Spotted Dove	2022/1/2	2	0	16.51903333	81.51410000	https://www.inaturalist.org/observations/104411677
926	Spilopelia chinensis	Eastern Spotted Dove	2021/7/6	2	0	16.51980013	80.67626028	https://www.inaturalist.org/observations/85859941
927	Spilopelia chinensis	Eastern Spotted Dove	2020/8/26	1	0	16.52000000	80.67600000	https://www.inaturalist.org/observations/57607256
928	Spilopelia chinensis	Eastern Spotted Dove	2022/7/24	4	0	16.52264888	73.53148177	https://www.inaturalist.org/observations/129242595
929	Spilopelia chinensis	Eastern Spotted Dove	2021/7/18	1	0	16.52274471	80.63134938	https://www.inaturalist.org/observations/95113834
930	Spilopelia chinensis	Eastern Spotted Dove	2021/12/20	1	0	16.52359650	80.63777792	https://www.inaturalist.org/observations/103360156
931	Spilopelia chinensis	Eastern Spotted Dove	2018/1/6	1	0	16.52925276	80.67580696	https://www.inaturalist.org/observations/49218867
932	Spilopelia chinensis	Eastern Spotted Dove	2020/4/13	2	0	16.52943607	80.59093245	https://www.inaturalist.org/observations/42163773
933	Spilopelia chinensis	Eastern Spotted Dove	2022/5/6	4	0	16.53181896	80.60471401	https://www.inaturalist.org/observations/117331089
934	Spilopelia chinensis	Eastern Spotted Dove	2022/3/15	5	0	16.53690333	81.55579667	https://www.inaturalist.org/observations/108648093
935	Spilopelia chinensis	Eastern Spotted Dove	2022/3/15	1	0	16.53690333	81.55579667	https://www.inaturalist.org/observations/114316822
936	Spilopelia chinensis	Eastern Spotted Dove	2022/3/15	2	0	16.53710167	81.55571333	https://www.inaturalist.org/observations/114289989
937	Spilopelia chinensis	Eastern Spotted Dove	2021/12/31	2	0	16.53965944	81.50560364	https://www.inaturalist.org/observations/104111778
938	Spilopelia chinensis	Eastern Spotted Dove	2022/1/2	1	0	16.53968001	81.50569115	https://www.inaturalist.org/observations/104166652
939	Spilopelia chinensis	Eastern Spotted Dove	2021/12/23	1	0	16.54489085	81.52124092	https://www.inaturalist.org/observations/103530579
940	Spilopelia chinensis	Eastern Spotted Dove	2021/12/31	2	0	16.55703790	81.49404097	https://www.inaturalist.org/observations/104023134
941	Spilopelia chinensis	Eastern Spotted Dove	2021/12/23	2	0	16.55704167	81.49411000	https://www.inaturalist.org/observations/106079131
942	Spilopelia chinensis	Eastern Spotted Dove	2021/12/23	3	0	16.55704167	81.49411000	https://www.inaturalist.org/observations/111966881

943	Spilopelia chinensis	Eastern Spotted Dove	2021/12/23	1	0	16.55704500	81.49412667	https://www.inaturalist.org/observations/104648173
944	Spilopelia chinensis	Eastern Spotted Dove	2021/12/23	1	0	16.55705833	81.49408833	https://www.inaturalist.org/observations/103760743
945	Spilopelia chinensis	Eastern Spotted Dove	2021/12/23	1	0	16.55705833	81.49408833	https://www.inaturalist.org/observations/104352325
946	Spilopelia chinensis	Eastern Spotted Dove	2022/2/6	1	0	16.55730667	81.49229500	https://www.inaturalist.org/observations/106237081
947	Spilopelia chinensis	Eastern Spotted Dove	2021/9/1	1	0	16.55745279	81.49806127	https://www.inaturalist.org/observations/95744321
948	Spilopelia chinensis	Eastern Spotted Dove	2020/12/20	1	0	16.55772920	80.72698540	https://www.inaturalist.org/observations/92475075
949	Spilopelia chinensis	Eastern Spotted Dove	2021/10/10	2	0	16.55811900	81.49816353	https://www.inaturalist.org/observations/97895462
950	Spilopelia chinensis	Eastern Spotted Dove	2021/10/28	2	0	16.55811900	81.49816353	https://www.inaturalist.org/observations/101397653
951	Spilopelia chinensis	Eastern Spotted Dove	2021/12/31	1	0	16.55879167	81.49852000	https://www.inaturalist.org/observations/104118228
952	Spilopelia chinensis	Eastern Spotted Dove	2021/12/31	1	0	16.55879167	81.49852000	https://www.inaturalist.org/observations/104348072
953	Spilopelia chinensis	Eastern Spotted Dove	2021/12/31	1	0	16.55879167	81.49852000	https://www.inaturalist.org/observations/104411437
954	Spilopelia chinensis	Eastern Spotted Dove	2020/12/1	1	0	16.55956663	80.76008636	https://www.inaturalist.org/observations/92473424
955	Spilopelia chinensis	Eastern Spotted Dove	2020/11/22	3	0	16.56399833	80.74516500	https://www.inaturalist.org/observations/65397649
956	Spilopelia chinensis	Eastern Spotted Dove	2020/11/29	1	0	16.57589266	80.68862217	https://www.inaturalist.org/observations/92670130
957	Spilopelia chinensis	Eastern Spotted Dove	2020/9/18	3	0	16.62166000	80.51394000	https://www.inaturalist.org/observations/59955410
958	Spilopelia chinensis	Eastern Spotted Dove	2021/7/24	3	0	16.62166000	80.51394000	https://www.inaturalist.org/observations/88412710
959	Spilopelia chinensis	Eastern Spotted Dove	2021/12/28	1	0	16.62712500	80.78196111	https://www.inaturalist.org/observations/103819850
960	Spilopelia chinensis	Eastern Spotted Dove	2017/12/22	4	0	16.62928639	73.34324139	https://www.inaturalist.org/observations/30800922
961	Spilopelia chinensis	Eastern Spotted Dove	2018/8/6	5	0	16.63080806	73.35558972	https://www.inaturalist.org/observations/30916429
962	Spilopelia chinensis	Eastern Spotted Dove	2020/11/8	1	0	16.63578069	80.79800606	https://www.inaturalist.org/observations/92484764
963	Spilopelia chinensis	Eastern Spotted Dove	2021/6/20	1	0	16.63773941	80.79677278	https://www.inaturalist.org/observations/91646598
964	Spilopelia chinensis	Eastern Spotted Dove	2017/12/22	5	0	16.64066639	73.35301139	https://www.inaturalist.org/observations/30800876
965	Spilopelia chinensis	Eastern Spotted Dove	2021/8/15	1	0	16.64249177	80.48667067	https://www.inaturalist.org/observations/91540414

966	Spilopelia chinensis	Eastern Spotted Dove	2021/9/12	2	0	16.64309974	80.48695564	https://www.inaturalist.org/observations/94807578
967	Spilopelia chinensis	Eastern Spotted Dove	2021/9/24	1	0	16.64336083	80.48831037	https://www.inaturalist.org/observations/95970265
968	Spilopelia chinensis	Eastern Spotted Dove	2021/8/15	1	0	16.64457999	80.48804998	https://www.inaturalist.org/observations/91255062
969	Spilopelia chinensis	Eastern Spotted Dove	2021/8/22	1	0	16.64467134	80.48805617	https://www.inaturalist.org/observations/92343646
970	Spilopelia chinensis	Eastern Spotted Dove	2021/7/4	2	0	16.64507460	80.48777312	https://www.inaturalist.org/observations/90636951
971	Spilopelia chinensis	Eastern Spotted Dove	2018/8/6	5	0	16.64598306	73.36884000	https://www.inaturalist.org/observations/30916161
972	Spilopelia chinensis	Eastern Spotted Dove	2022/2/19	3	0	16.64950208	80.49085768	https://www.inaturalist.org/observations/108083315
973	Spilopelia chinensis	Eastern Spotted Dove	2022/3/6	5	0	16.65391605	80.46723317	https://www.inaturalist.org/observations/108249515
974	Spilopelia chinensis	Eastern Spotted Dove	2020/12/13	1	0	16.65462089	80.46307863	https://www.inaturalist.org/observations/92659377
975	Spilopelia chinensis	Eastern Spotted Dove	2020/12/18	3	0	16.66244810	80.69972200	https://www.inaturalist.org/observations/67304121
976	Spilopelia chinensis	Eastern Spotted Dove	2017/10/20	1	0	16.66497301	81.18106842	https://www.inaturalist.org/observations/98112207
977	Spilopelia chinensis	Eastern Spotted Dove	2018/2/18	1	0	16.66497301	81.18106842	https://www.inaturalist.org/observations/98114809
978	Spilopelia chinensis	Eastern Spotted Dove	2021/12/22	3	0	16.69786043	82.12057114	https://www.inaturalist.org/observations/103876501
979	Spilopelia chinensis	Eastern Spotted Dove	2022/1/5	2	0	16.69901389	80.76662500	https://www.inaturalist.org/observations/104401864
980	Spilopelia chinensis	Eastern Spotted Dove	2022/2/17	2	0	16.69901944	80.76675833	https://www.inaturalist.org/observations/106913151
981	Spilopelia chinensis	Eastern Spotted Dove	2022/3/8	3	0	16.69909444	80.76635833	https://www.inaturalist.org/observations/108172041
982	Spilopelia chinensis	Eastern Spotted Dove	2022/3/11	2	0	16.69921111	80.76580278	https://www.inaturalist.org/observations/108362233
983	Spilopelia chinensis	Eastern Spotted Dove	2022/1/4	1	0	16.70608889	80.75481944	https://www.inaturalist.org/observations/104401164
984	Spilopelia chinensis	Eastern Spotted Dove	2022/1/27	1	0	16.70613333	80.75464167	https://www.inaturalist.org/observations/105713284
985	Spilopelia chinensis	Eastern Spotted Dove	2021/4/24	3	0	16.71195833	73.34098333	https://www.inaturalist.org/observations/75036618
986	Spilopelia chinensis	Eastern Spotted Dove	2021/3/1	3	0	16.71956910	73.35530210	https://www.inaturalist.org/observations/71128071
987	Spilopelia chinensis	Eastern Spotted Dove	2020/6/28	1	0	16.94133758	73.67566681	https://www.inaturalist.org/observations/51194044
988	Spilopelia chinensis	Eastern Spotted Dove	2015/1/26	4	0	16.94806230	73.51523810	https://www.inaturalist.org/observations/107811354

989	Spilopelia chinensis	Eastern Spotted Dove	2022/7/29	2	0	17.09470270	74.02088550	https://www.inaturalist.org/observations/129520497
990	Spilopelia chinensis	Eastern Spotted Dove	2020/6/26	2	0	17.17096167	73.53648333	https://www.inaturalist.org/observations/52009085
991	Spilopelia chinensis	Eastern Spotted Dove	2021/7/31	1	0	17.18129697	78.80309701	https://www.inaturalist.org/observations/90165653
992	Spilopelia chinensis	Eastern Spotted Dove	2021/12/12	1	0	17.26026167	81.50225167	https://www.inaturalist.org/observations/103527522
993	Spilopelia chinensis	Eastern Spotted Dove	2022/5/22	3	0	17.31234335	77.84826880	https://www.inaturalist.org/observations/118331011
994	Spilopelia chinensis	Eastern Spotted Dove	2022/9/28	1	0	17.32109100	78.39382120	https://www.inaturalist.org/observations/137013590
995	Spilopelia chinensis	Eastern Spotted Dove	2022/9/21	3	0	17.35067800	78.45130680	https://www.inaturalist.org/observations/137030959
996	Spilopelia chinensis	Eastern Spotted Dove	2017/12/1	6	0	17.35731065	78.47317632	https://www.inaturalist.org/observations/9014975
997	Spilopelia chinensis	Eastern Spotted Dove	2017/11/29	6	0	17.35923703	78.30090299	https://www.inaturalist.org/observations/9111397
998	Spilopelia chinensis	Eastern Spotted Dove	2021/4/29	2	0	17.37000604	78.35701879	https://www.inaturalist.org/observations/75614477
999	Spilopelia chinensis	Eastern Spotted Dove	2017/10/27	2	0	17.37272613	73.22309182	https://www.inaturalist.org/observations/55030548
1000	Spilopelia chinensis	Eastern Spotted Dove	2020/12/6	5	0	17.37587568	73.21191296	https://www.inaturalist.org/observations/66288088
1001	Spilopelia chinensis	Eastern Spotted Dove	2021/10/20	3	0	17.37595760	78.53705600	https://www.inaturalist.org/observations/99893677
1002	Spilopelia chinensis	Eastern Spotted Dove	2019/5/13	3	0	17.37908314	78.30266370	https://www.inaturalist.org/observations/25072327
1003	Spilopelia chinensis	Eastern Spotted Dove	2020/8/25	3	0	17.38494038	78.48667111	https://www.inaturalist.org/observations/65020906
1004	Spilopelia chinensis	Eastern Spotted Dove	2021/1/31	0	0	17.38494038	78.48667111	https://www.inaturalist.org/observations/96977340
1005	Spilopelia chinensis	Eastern Spotted Dove	2012/10/9	6	0	17.38504400	78.48667100	https://www.inaturalist.org/observations/19189587
1006	Spilopelia chinensis	Eastern Spotted Dove	2021/10/18	1	0	17.39327770	78.34114480	https://www.inaturalist.org/observations/99242580
1007	Spilopelia chinensis	Eastern Spotted Dove	2021/5/23	3	0	17.39578470	78.43119440	https://www.inaturalist.org/observations/80241347
1008	Spilopelia chinensis	Eastern Spotted Dove	2019/6/1	3	0	17.39694100	78.51966600	https://www.inaturalist.org/observations/26165313
1009	Spilopelia chinensis	Eastern Spotted Dove	2019/5/31	3	0	17.39698500	78.51975000	https://www.inaturalist.org/observations/26162134
1010	Spilopelia chinensis	Eastern Spotted Dove	2012/4/8	1	0	17.40534260	73.54174400	https://www.inaturalist.org/observations/41611272
1011	Spilopelia chinensis	Eastern Spotted Dove	2012/4/8	1	0	17.40534260	73.54174400	https://www.inaturalist.org/observations/41611311

1012	Spilopelia chinensis	Eastern Spotted Dove	2019/1/17	5	0	17.40602427	78.39477960	https://www.inaturalist.org/observations/19898711
1013	Spilopelia chinensis	Eastern Spotted Dove	2007/3/4	6	0	17.42450200	78.46375400	https://www.inaturalist.org/observations/18798203
1014	Spilopelia chinensis	Eastern Spotted Dove	2020/8/5	1	0	17.43041121	78.45883369	https://www.inaturalist.org/observations/56042387
1015	Spilopelia chinensis	Eastern Spotted Dove	2022/7/31	4	0	17.43207510	78.47538770	https://www.inaturalist.org/observations/130092333
1016	Spilopelia chinensis	Eastern Spotted Dove	2022/7/31	4	0	17.43207510	78.47538770	https://www.inaturalist.org/observations/130092334
1017	Spilopelia chinensis	Eastern Spotted Dove	2020/2/6	0	0	17.43427833	78.38288000	https://www.inaturalist.org/observations/38530057
1018	Spilopelia chinensis	Eastern Spotted Dove	2017/12/1	6	0	17.43774264	78.38744466	https://www.inaturalist.org/observations/9014942
1019	Spilopelia chinensis	Eastern Spotted Dove	2022/4/10	2	0	17.44147023	81.52410083	https://www.inaturalist.org/observations/111893967
1020	Spilopelia chinensis	Eastern Spotted Dove	2017/11/30	4	0	17.44159224	78.38192850	https://www.inaturalist.org/observations/9112336
1021	Spilopelia chinensis	Eastern Spotted Dove	2019/10/15	4	0	17.44275982	78.36853687	https://www.inaturalist.org/observations/34398169
1022	Spilopelia chinensis	Eastern Spotted Dove	2021/1/17	1	0	17.44952640	78.36371697	https://www.inaturalist.org/observations/115310214
1023	Spilopelia chinensis	Eastern Spotted Dove	2022/8/8	4	0	17.45104722	78.39385000	https://www.inaturalist.org/observations/130074068
1024	Spilopelia chinensis	Eastern Spotted Dove	2019/1/13	3	0	17.45189108	78.23120478	https://www.inaturalist.org/observations/19788110
1025	Spilopelia chinensis	Eastern Spotted Dove	2021/1/31	1	0	17.45398981	78.35721463	https://www.inaturalist.org/observations/98784320
1026	Spilopelia chinensis	Eastern Spotted Dove	2020/4/30	4	0	17.45426231	78.54788516	https://www.inaturalist.org/observations/62920320
1027	Spilopelia chinensis	Eastern Spotted Dove	2022/8/28	3	0	17.45436626	78.35679285	https://www.inaturalist.org/observations/132780547
1028	Spilopelia chinensis	Eastern Spotted Dove	2019/1/17	5	0	17.45517541	78.25529616	https://www.inaturalist.org/observations/19834026
1029	Spilopelia chinensis	Eastern Spotted Dove	2022/9/6	3	0	17.45673625	78.32638767	https://www.inaturalist.org/observations/133929580
1030	Spilopelia chinensis	Eastern Spotted Dove	2018/12/3	3	0	17.45805239	78.37074126	https://www.inaturalist.org/observations/18791361
1031	Spilopelia chinensis	Eastern Spotted Dove	2012/10/9	6	0	17.47336200	78.37290200	http://www.inaturalist.org/observations/250788
1032	Spilopelia chinensis	Eastern Spotted Dove	2019/1/13	4	0	17.47431526	78.27746772	https://www.inaturalist.org/observations/19766292
1033	Spilopelia chinensis	Eastern Spotted Dove	2020/1/9	3	0	17.48342117	78.59797824	https://www.inaturalist.org/observations/37422776
1034	Spilopelia chinensis	Eastern Spotted Dove	2021/2/2	2	0	17.48377370	78.31583351	https://www.inaturalist.org/observations/68949238

1035	Spilopelia chinensis	Eastern Spotted Dove	2022/3/5	3	0	17.48463980	78.24298670	https://www.inaturalist.org/observations/107946729
1036	Spilopelia chinensis	Eastern Spotted Dove	2021/12/4	1	0	17.48571797	78.34817200	https://www.inaturalist.org/observations/102462559
1037	Spilopelia chinensis	Eastern Spotted Dove	2022/2/5	2	0	17.48605690	78.34653110	https://www.inaturalist.org/observations/106486633
1038	Spilopelia chinensis	Eastern Spotted Dove	2022/9/3	2	0	17.48605690	78.34653110	https://www.inaturalist.org/observations/133431105
1039	Spilopelia chinensis	Eastern Spotted Dove	2022/9/3	5	0	17.48605690	78.34653110	https://www.inaturalist.org/observations/133432807
1040	Spilopelia chinensis	Eastern Spotted Dove	2021/12/4	2	0	17.48605696	78.34653106	https://www.inaturalist.org/observations/102468513
1041	Spilopelia chinensis	Eastern Spotted Dove	2021/12/4	2	0	17.48605696	78.34653106	https://www.inaturalist.org/observations/102472284
1042	Spilopelia chinensis	Eastern Spotted Dove	2021/8/20	2	0	17.48907377	78.34631749	https://www.inaturalist.org/observations/91843911
1043	Spilopelia chinensis	Eastern Spotted Dove	2020/6/2	5	0	17.49002352	78.53177642	https://www.inaturalist.org/observations/48202672
1044	Spilopelia chinensis	Eastern Spotted Dove	2021/1/31	4	0	17.49004289	78.53207728	https://www.inaturalist.org/observations/68844797
1045	Spilopelia chinensis	Eastern Spotted Dove	2021/7/28	2	0	17.49087167	78.53409500	https://www.inaturalist.org/observations/88931486
1046	Spilopelia chinensis	Eastern Spotted Dove	2021/3/13	2	0	17.49127997	78.33826100	https://www.inaturalist.org/observations/86250684
1047	Spilopelia chinensis	Eastern Spotted Dove	2022/9/2	4	0	17.49230278	78.33621667	https://www.inaturalist.org/observations/133321221
1048	Spilopelia chinensis	Eastern Spotted Dove	2022/9/2	2	0	17.49230278	78.33621667	https://www.inaturalist.org/observations/133321272
1049	Spilopelia chinensis	Eastern Spotted Dove	2022/8/29	4	0	17.49263889	78.33600556	https://www.inaturalist.org/observations/132782776
1050	Spilopelia chinensis	Eastern Spotted Dove	2022/9/4	2	0	17.49281389	78.33744444	https://www.inaturalist.org/observations/133566588
1051	Spilopelia chinensis	Eastern Spotted Dove	2022/2/25	3	0	17.49368560	78.34012930	https://www.inaturalist.org/observations/108249076
1052	Spilopelia chinensis	Eastern Spotted Dove	2021/11/24	1	0	17.49670160	78.50662330	https://www.inaturalist.org/observations/103686994
1053	Spilopelia chinensis	Eastern Spotted Dove	2014/12/11	5	0	17.50002296	81.38143463	https://www.inaturalist.org/observations/66881019
1054	Spilopelia chinensis	Eastern Spotted Dove	2021/12/2	2	0	17.51635452	78.32887843	https://www.inaturalist.org/observations/105550210
1055	Spilopelia chinensis	Eastern Spotted Dove	2022/6/5	3	0	17.51714280	78.31574200	https://www.inaturalist.org/observations/120287100
1056	Spilopelia chinensis	Eastern Spotted Dove	2022/1/16	3	0	17.52246770	78.33443850	https://www.inaturalist.org/observations/105039242
1057	Spilopelia chinensis	Eastern Spotted Dove	2022/4/17	1	0	17.52246770	78.33443850	https://www.inaturalist.org/observations/111761122

1058	Spilopelia chinensis	Eastern Spotted Dove	2020/9/27	4	0	17.53266850	78.37488537	https://www.inaturalist.org/observations/61297003
1059	Spilopelia chinensis	Eastern Spotted Dove	2019/1/17	4	0	17.53325935	78.23338502	https://www.inaturalist.org/observations/19834044
1060	Spilopelia chinensis	Eastern Spotted Dove	2021/12/10	2	0	17.59550124	81.68398600	https://www.inaturalist.org/observations/103412889
1061	Spilopelia chinensis	Eastern Spotted Dove	2018/9/21	4	0	17.59927845	81.68741867	https://www.inaturalist.org/observations/64935902
1062	Spilopelia chinensis	Eastern Spotted Dove	2022/6/24	4	0	17.63509667	78.53748833	https://www.inaturalist.org/observations/123211316
1063	Spilopelia chinensis	Eastern Spotted Dove	2019/11/2	1	0	17.67067090	81.70914260	https://www.inaturalist.org/observations/92889162
1064	Spilopelia chinensis	Eastern Spotted Dove	2020/11/13	1	0	17.68046390	74.01826100	https://www.inaturalist.org/observations/71119196
1065	Spilopelia chinensis	Eastern Spotted Dove	2020/11/13	1	0	17.68046390	74.01826100	https://www.inaturalist.org/observations/105498334
1066	Spilopelia chinensis	Eastern Spotted Dove	2020/11/13	1	0	17.68046390	74.01826100	https://www.inaturalist.org/observations/105498338
1067	Spilopelia chinensis	Eastern Spotted Dove	2021/12/12	4	0	17.68661679	78.32732443	https://www.inaturalist.org/observations/104472223
1068	Spilopelia chinensis	Eastern Spotted Dove	2022/2/27	3	0	17.68662580	78.32732450	https://www.inaturalist.org/observations/107689546
1069	Spilopelia chinensis	Eastern Spotted Dove	2022/6/26	2	0	17.68662580	78.32732450	https://www.inaturalist.org/observations/123486419
1070	Spilopelia chinensis	Eastern Spotted Dove	2021/7/30	2	0	17.73106766	83.33222961	https://www.inaturalist.org/observations/89175274
1071	Spilopelia chinensis	Eastern Spotted Dove	2022/6/24	5	0	17.73940000	83.32820000	https://www.inaturalist.org/observations/123199095
1072	Spilopelia chinensis	Eastern Spotted Dove	2021/9/1	1	0	17.74200161	83.32744885	https://www.inaturalist.org/observations/93376040
1073	Spilopelia chinensis	Eastern Spotted Dove	2020/12/24	3	0	17.76913300	73.74210180	https://www.inaturalist.org/observations/68050059
1074	Spilopelia chinensis	Eastern Spotted Dove	2021/7/31	1	0	17.86485518	82.69698718	https://www.inaturalist.org/observations/90024042
1075	Spilopelia chinensis	Eastern Spotted Dove	2017/10/1	2	0	17.98149900	73.79544500	https://www.inaturalist.org/observations/45665898
1076	Spilopelia chinensis	Eastern Spotted Dove	2022/1/2	1	0	17.99041898	82.97922067	https://www.inaturalist.org/observations/104173476
1077	Spilopelia chinensis	Eastern Spotted Dove	2020/10/2	2	0	18.09901203	83.41638275	https://www.inaturalist.org/observations/61467895
1078	Spilopelia chinensis	Eastern Spotted Dove	2018/7/7	3	0	18.11243720	79.01929970	https://www.inaturalist.org/observations/63556665
1079	Spilopelia chinensis	Eastern Spotted Dove	2016/10/29	4	0	18.11243720	79.01929970	https://www.inaturalist.org/observations/63558506
1080	Spilopelia chinensis	Eastern Spotted Dove	2022/3/1	2	0	18.14128328	73.79158091	https://www.inaturalist.org/observations/111675249

1081	Spilopelia chinensis	Eastern Spotted Dove	2018/12/23	3	0	18.16823210	72.99018980	https://www.inaturalist.org/observations/67147398
1082	Spilopelia chinensis	Eastern Spotted Dove	2017/10/12	4	0	18.19868240	109.48975330	https://www.inaturalist.org/observations/8382926
1083	Spilopelia chinensis	Eastern Spotted Dove	2017/11/13	3	0	18.20059851	109.48977480	https://www.inaturalist.org/observations/8802099
1084	Spilopelia chinensis	Eastern Spotted Dove	2017/3/10	4	0	18.22663796	109.50661180	https://www.inaturalist.org/observations/5400841
1085	Spilopelia chinensis	Eastern Spotted Dove	2016/12/7	4	0	18.25284700	109.51190900	http://www.inaturalist.org/observations/4735422
1086	Spilopelia chinensis	Eastern Spotted Dove	2017/6/12	4	0	18.25284700	109.51190900	https://www.inaturalist.org/observations/6620149
1087	Spilopelia chinensis	Eastern Spotted Dove	2017/6/22	4	0	18.25284700	109.51190900	https://www.inaturalist.org/observations/6764332
1088	Spilopelia chinensis	Eastern Spotted Dove	2019/4/26	1	0	18.25902284	109.53557980	https://www.inaturalist.org/observations/24882983
1089	Spilopelia chinensis	Eastern Spotted Dove	2022/2/17	1	0	18.28877980	73.13136383	https://www.inaturalist.org/observations/107090522
1090	Spilopelia chinensis	Eastern Spotted Dove	2022/1/15	4	0	18.28911907	73.13576672	https://www.inaturalist.org/observations/104970709
1091	Spilopelia chinensis	Eastern Spotted Dove	2021/9/15	1	0	18.28939792	73.13555751	https://www.inaturalist.org/observations/95295581
1092	Spilopelia chinensis	Eastern Spotted Dove	2021/9/3	2	0	18.29018231	73.13359413	https://www.inaturalist.org/observations/95023184
1093	Spilopelia chinensis	Eastern Spotted Dove	2022/4/10	3	0	18.32106860	73.66612970	https://www.inaturalist.org/observations/121449012
1094	Spilopelia chinensis	Eastern Spotted Dove	2021/1/28	2	0	18.33481840	73.67215150	https://www.inaturalist.org/observations/70931525
1095	Spilopelia chinensis	Eastern Spotted Dove	2019/2/14	3	0	18.33760984	109.58740490	https://www.inaturalist.org/observations/20339260
1096	Spilopelia chinensis	Eastern Spotted Dove	2019/2/14	2	0	18.33760984	109.58740490	https://www.inaturalist.org/observations/20339261
1097	Spilopelia chinensis	Eastern Spotted Dove	2020/11/29	3	0	18.35312390	75.63916110	https://www.inaturalist.org/observations/75145437
1098	Spilopelia chinensis	Eastern Spotted Dove	2020/10/27	1	0	18.35425091	75.96868370	https://www.inaturalist.org/observations/63619800
1099	Spilopelia chinensis	Eastern Spotted Dove	2012/1/29	3	0	18.37499735	73.76974406	https://www.inaturalist.org/observations/128263448
1100	Spilopelia chinensis	Eastern Spotted Dove	2018/3/31	2	0	18.37586280	73.76916470	https://www.inaturalist.org/observations/44360082
1101	Spilopelia chinensis	Eastern Spotted Dove	2017/10/29	4	0	18.37586280	73.76916470	https://www.inaturalist.org/observations/44361547
1102	Spilopelia chinensis	Eastern Spotted Dove	2018/3/31	2	0	18.37586280	73.76916470	https://www.inaturalist.org/observations/67006336
1103	Spilopelia chinensis	Eastern Spotted Dove	2017/10/29	3	0	18.37586280	73.76916470	https://www.inaturalist.org/observations/67006886

1104	Spilopelia chinensis	Eastern Spotted Dove	2021/3/12	3	0	18.37586280	73.76916470	https://www.inaturalist.org/observations/71121908
1105	Spilopelia chinensis	Eastern Spotted Dove	2021/2/13	2	0	18.37586280	73.76916470	https://www.inaturalist.org/observations/75058833
1106	Spilopelia chinensis	Eastern Spotted Dove	2022/3/22	6	0	18.37586782	73.76915932	https://www.inaturalist.org/observations/109300968
1107	Spilopelia chinensis	Eastern Spotted Dove	2016/4/9	3	0	18.37846650	73.76782250	https://www.inaturalist.org/observations/44859673
1108	Spilopelia chinensis	Eastern Spotted Dove	2017/4/1	2	0	18.38027930	73.76764300	https://www.inaturalist.org/observations/41810990
1109	Spilopelia chinensis	Eastern Spotted Dove	2022/2/24	2	0	18.38027930	73.76764300	https://www.inaturalist.org/observations/121368356
1110	Spilopelia chinensis	Eastern Spotted Dove	2022/4/12	2	0	18.44690320	73.43054390	https://www.inaturalist.org/observations/135571079
1111	Spilopelia chinensis	Eastern Spotted Dove	2022/4/12	2	0	18.44690320	73.43054390	https://www.inaturalist.org/observations/135571080
1112	Spilopelia chinensis	Eastern Spotted Dove	2017/10/29	5	0	18.45127779	73.77890110	https://www.inaturalist.org/observations/8601144
1113	Spilopelia chinensis	Eastern Spotted Dove	2017/11/5	4	0	18.45640707	73.78134727	https://www.inaturalist.org/observations/8705776
1114	Spilopelia chinensis	Eastern Spotted Dove	2012/9/2	5	0	18.46009619	73.45490601	https://www.inaturalist.org/observations/17446520
1115	Spilopelia chinensis	Eastern Spotted Dove	2016/12/15	2	0	18.46571306	73.46555472	https://www.inaturalist.org/observations/94905371
1116	Spilopelia chinensis	Eastern Spotted Dove	2016/6/15	1	0	18.47670806	73.47459000	https://www.inaturalist.org/observations/95640141
1117	Spilopelia chinensis	Eastern Spotted Dove	2015/8/16	5	0	18.52548990	73.81538450	http://www.inaturalist.org/observations/3910577
1118	Spilopelia chinensis	Eastern Spotted Dove	2019/8/16	4	0	18.54137667	82.61475500	https://www.inaturalist.org/observations/35539469
1119	Spilopelia chinensis	Eastern Spotted Dove	2019/8/15	4	0	18.57338167	82.49817167	https://www.inaturalist.org/observations/35537312
1120	Spilopelia chinensis	Eastern Spotted Dove	2019/8/15	4	0	18.57451000	82.49688667	https://www.inaturalist.org/observations/35537633
1121	Spilopelia chinensis	Eastern Spotted Dove	2018/2/9	2	0	18.60315524	108.71063240	https://www.inaturalist.org/observations/10243251
1122	Spilopelia chinensis	Eastern Spotted Dove	2013/4/3	4	0	18.69415352	72.93129234	https://www.inaturalist.org/observations/104699133
1123	Spilopelia chinensis	Eastern Spotted Dove	2022/9/23	2	0	18.70543726	73.45919974	https://www.inaturalist.org/observations/136671772
1124	Spilopelia chinensis	Eastern Spotted Dove	2022/8/11	5	0	18.73185333	73.47917167	https://www.inaturalist.org/observations/130368229
1125	Spilopelia chinensis	Eastern Spotted Dove	2011/12/21	1	0	18.74218140	72.86276575	https://www.inaturalist.org/observations/70428982
1126	Spilopelia chinensis	Eastern Spotted Dove	2010/2/26	1	0	18.75572370	73.40907570	https://www.inaturalist.org/observations/38153616

1127	Spilopelia chinensis	Eastern Spotted Dove	2010/2/26	3	0	18.76510960	73.40783550	https://www.inaturalist.org/observations/69552804
1128	Spilopelia chinensis	Eastern Spotted Dove	2019/12/16	2	0	18.89103790	73.12191850	https://www.inaturalist.org/observations/52641370
1129	Spilopelia chinensis	Eastern Spotted Dove	2016/10/30	4	0	18.89992803	72.95202702	https://www.inaturalist.org/observations/64533141
1130	Spilopelia chinensis	Eastern Spotted Dove	2016/10/23	1	0	18.89992803	72.95202702	https://www.inaturalist.org/observations/100514836
1131	Spilopelia chinensis	Eastern Spotted Dove	2022/3/23	4	0	18.90064326	73.25803565	https://www.inaturalist.org/observations/110304527
1132	Spilopelia chinensis	Eastern Spotted Dove	2019/8/11	3	0	18.90524167	83.46250667	https://www.inaturalist.org/observations/34634576
1133	Spilopelia chinensis	Eastern Spotted Dove	2020/4/17	2	0	18.91921965	81.83882955	https://www.inaturalist.org/observations/42957217
1134	Spilopelia chinensis	Eastern Spotted Dove	2020/9/18	1	0	18.93908882	84.58715057	https://www.inaturalist.org/observations/79110245
1135	Spilopelia chinensis	Eastern Spotted Dove	2020/2/20	2	0	18.98912191	82.15650482	https://www.inaturalist.org/observations/39064776
1136	Spilopelia chinensis	Eastern Spotted Dove	2019/12/29	1	0	19.01659084	73.00593976	https://www.inaturalist.org/observations/104542980
1137	Spilopelia chinensis	Eastern Spotted Dove	2016/1/26	2	0	19.01828952	73.00556157	https://www.inaturalist.org/observations/68670391
1138	Spilopelia chinensis	Eastern Spotted Dove	2021/2/2	2	0	19.03197498	73.03356249	https://www.inaturalist.org/observations/69174507
1139	Spilopelia chinensis	Eastern Spotted Dove	2019/8/14	4	0	19.03617333	83.16652667	https://www.inaturalist.org/observations/34638530
1140	Spilopelia chinensis	Eastern Spotted Dove	2019/8/12	3	0	19.11192167	83.85526333	https://www.inaturalist.org/observations/34635612
1141	Spilopelia chinensis	Eastern Spotted Dove	2012/11/8	3	0	19.11480815	73.56371228	https://www.inaturalist.org/observations/108014658
1142	Spilopelia chinensis	Eastern Spotted Dove	2019/12/22	4	0	19.14190380	72.96462540	https://www.inaturalist.org/observations/36905324
1143	Spilopelia chinensis	Eastern Spotted Dove	2021/11/21	1	0	19.14190551	72.96462838	https://www.inaturalist.org/observations/101681346
1144	Spilopelia chinensis	Eastern Spotted Dove	2019/11/30	4	0	19.14191121	72.96462536	https://www.inaturalist.org/observations/36458970
1145	Spilopelia chinensis	Eastern Spotted Dove	2017/10/7	2	0	19.14192483	72.96462536	https://www.inaturalist.org/observations/56393936
1146	Spilopelia chinensis	Eastern Spotted Dove	2017/11/12	2	0	19.14192483	72.96462536	https://www.inaturalist.org/observations/56404031
1147	Spilopelia chinensis	Eastern Spotted Dove	2017/11/26	2	0	19.14192483	72.96462536	https://www.inaturalist.org/observations/56404336
1148	Spilopelia chinensis	Eastern Spotted Dove	2017/12/2	2	0	19.14192483	72.96462536	https://www.inaturalist.org/observations/56406574
1149	Spilopelia chinensis	Eastern Spotted Dove	2020/10/2	2	0	19.14192483	72.96462536	https://www.inaturalist.org/observations/62011527

1150	Spilopelia chinensis	Eastern Spotted Dove	2021/10/15	1	0	19.14192483	72.96462536	https://www.inaturalist.org/observations/98696877
1151	Spilopelia chinensis	Eastern Spotted Dove	2020/12/12	3	0	19.14951660	73.15698430	https://www.inaturalist.org/observations/66466945
1152	Spilopelia chinensis	Eastern Spotted Dove	2021/10/31	3	0	19.16457805	73.31106415	https://www.inaturalist.org/observations/132288285
1153	Spilopelia chinensis	Eastern Spotted Dove	2017/9/11	3	0	19.18916729	72.98159990	https://www.inaturalist.org/observations/136710765
1154	Spilopelia chinensis	Eastern Spotted Dove	2019/8/12	4	0	19.18984500	83.72661333	https://www.inaturalist.org/observations/34635249
1155	Spilopelia chinensis	Eastern Spotted Dove	2019/8/12	3	0	19.19120167	83.72539167	https://www.inaturalist.org/observations/34635192
1156	Spilopelia chinensis	Eastern Spotted Dove	2019/8/12	3	0	19.19143333	83.72515167	https://www.inaturalist.org/observations/34635107
1157	Spilopelia chinensis	Eastern Spotted Dove	2020/11/21	3	0	19.19257320	73.33353690	https://www.inaturalist.org/observations/65591039
1158	Spilopelia chinensis	Eastern Spotted Dove	2020/11/22	3	0	19.19257320	73.33353690	https://www.inaturalist.org/observations/65591042
1159	Spilopelia chinensis	Eastern Spotted Dove	2020/11/23	4	0	19.19257320	73.33353690	https://www.inaturalist.org/observations/65638454
1160	Spilopelia chinensis	Eastern Spotted Dove	2013/3/27	1	0	19.20571630	73.01624180	https://www.inaturalist.org/observations/41678088
1161	Spilopelia chinensis	Eastern Spotted Dove	2020/11/7	3	0	19.21942330	73.12483440	https://www.inaturalist.org/observations/64660732
1162	Spilopelia chinensis	Eastern Spotted Dove	2021/11/27	2	0	19.23196227	73.11217636	https://www.inaturalist.org/observations/102156563
1163	Spilopelia chinensis	Eastern Spotted Dove	2020/3/7	2	0	19.23376500	72.94458020	https://www.inaturalist.org/observations/39641097
1164	Spilopelia chinensis	Eastern Spotted Dove	2021/3/6	3	0	19.23376500	72.94458020	https://www.inaturalist.org/observations/70659871
1165	Spilopelia chinensis	Eastern Spotted Dove	2022/4/23	1	0	19.23376500	72.94458020	https://www.inaturalist.org/observations/112497239
1166	Spilopelia chinensis	Eastern Spotted Dove	2021/7/31	1	0	19.23595411	73.47399998	https://www.inaturalist.org/observations/89747256
1167	Spilopelia chinensis	Eastern Spotted Dove	2010/1/10	2	0	19.23675100	72.96424900	https://www.inaturalist.org/observations/70255694
1168	Spilopelia chinensis	Eastern Spotted Dove	2010/1/10	1	0	19.23821100	72.96496100	https://www.inaturalist.org/observations/70297506
1169	Spilopelia chinensis	Eastern Spotted Dove	2018/10/28	5	0	19.23979254	72.99714843	https://www.inaturalist.org/observations/17900020
1170	Spilopelia chinensis	Eastern Spotted Dove	2021/3/14	2	0	19.24161420	72.99651240	https://www.inaturalist.org/observations/71205854
1171	Spilopelia chinensis	Eastern Spotted Dove	2018/10/28	2	0	19.24515270	72.99792940	https://www.inaturalist.org/observations/75050245
1172	Spilopelia chinensis	Eastern Spotted Dove	2019/8/19	4	0	19.25161833	84.90056833	https://www.inaturalist.org/observations/35542210

1173	Spilopelia chinensis	Eastern Spotted Dove	2020/9/19	4	0	19.25727500	73.64362008	https://www.inaturalist.org/observations/60504560
1174	Spilopelia chinensis	Eastern Spotted Dove	2019/8/18	4	0	19.25749500	84.89829667	https://www.inaturalist.org/observations/35541429
1175	Spilopelia chinensis	Eastern Spotted Dove	2019/8/18	5	0	19.25757500	84.89850667	https://www.inaturalist.org/observations/35541408
1176	Spilopelia chinensis	Eastern Spotted Dove	2019/10/23	1	0	19.25834100	110.47464800	https://www.inaturalist.org/observations/64989407
1177	Spilopelia chinensis	Eastern Spotted Dove	2022/4/30	1	0	19.26258900	73.21923874	https://www.inaturalist.org/observations/113773663
1178	Spilopelia chinensis	Eastern Spotted Dove	2012/12/16	3	0	19.26803820	73.14045390	https://www.inaturalist.org/observations/41427646
1179	Spilopelia chinensis	Eastern Spotted Dove	2019/8/13	5	0	19.28452333	83.43202667	https://www.inaturalist.org/observations/34636532
1180	Spilopelia chinensis	Eastern Spotted Dove	2021/8/5	2	0	19.33218279	84.80703078	https://www.inaturalist.org/observations/90020289
1181	Spilopelia chinensis	Eastern Spotted Dove	2021/8/4	0	0	19.33309647	84.81291857	https://www.inaturalist.org/observations/89992765
1182	Spilopelia chinensis	Eastern Spotted Dove	2018/10/14	2	0	19.38246770	72.77177750	https://www.inaturalist.org/observations/42587045
1183	Spilopelia chinensis	Eastern Spotted Dove	2021/3/18	3	0	19.40330945	72.95786753	https://www.inaturalist.org/observations/72062809
1184	Spilopelia chinensis	Eastern Spotted Dove	2022/8/31	1	0	19.40409623	81.79774224	https://www.inaturalist.org/observations/133053627
1185	Spilopelia chinensis	Eastern Spotted Dove	2015/3/9	4	0	19.42437300	80.56492700	http://www.inaturalist.org/observations/3360185
1186	Spilopelia chinensis	Eastern Spotted Dove	2019/8/14	5	0	19.42660667	83.45390000	https://www.inaturalist.org/observations/34638301
1187	Spilopelia chinensis	Eastern Spotted Dove	2019/1/27	1	0	19.43723083	108.84164990	https://www.inaturalist.org/observations/39043228
1188	Spilopelia chinensis	Eastern Spotted Dove	2019/8/13	4	0	19.48814833	83.46916667	https://www.inaturalist.org/observations/34636885
1189	Spilopelia chinensis	Eastern Spotted Dove	2019/8/13	4	0	19.48817333	83.46922500	https://www.inaturalist.org/observations/34636821
1190	Spilopelia chinensis	Eastern Spotted Dove	2022/6/2	2	0	19.50195557	73.01743948	https://www.inaturalist.org/observations/119882697
1191	Spilopelia chinensis	Eastern Spotted Dove	2022/7/21	4	0	19.50842894	73.01576235	https://www.inaturalist.org/observations/130097464
1192	Spilopelia chinensis	Eastern Spotted Dove	2022/3/27	4	0	19.58225707	81.66132230	https://www.inaturalist.org/observations/109836406
1193	Spilopelia chinensis	Eastern Spotted Dove	2021/5/19	2	0	19.64109996	81.47324044	https://www.inaturalist.org/observations/79425427
1194	Spilopelia chinensis	Eastern Spotted Dove	2017/3/27	4	0	19.74710870	73.19064280	https://www.inaturalist.org/observations/8600239
1195	Spilopelia chinensis	Eastern Spotted Dove	2019/10/16	4	0	19.74750643	81.41837921	https://www.inaturalist.org/observations/34865318

1196	Spilopelia chinensis	Eastern Spotted Dove	2019/10/16	4	0	19.75422043	81.38775777	https://www.inaturalist.org/observations/34865548
1197	Spilopelia chinensis	Eastern Spotted Dove	2019/1/14	3	0	19.88722848	82.69527767	https://www.inaturalist.org/observations/19639636
1198	Spilopelia chinensis	Eastern Spotted Dove	2019/8/20	6	0	19.88737167	86.09672667	https://www.inaturalist.org/observations/35545828
1199	Spilopelia chinensis	Eastern Spotted Dove	2019/12/17	4	0	19.88759530	86.09453640	https://www.inaturalist.org/observations/40360266
1200	Spilopelia chinensis	Eastern Spotted Dove	2016/12/12	3	0	19.89071839	86.09029370	https://www.inaturalist.org/observations/110214498
1201	Spilopelia chinensis	Eastern Spotted Dove	2019/8/19	6	0	19.89520000	85.43975167	https://www.inaturalist.org/observations/35543660
1202	Spilopelia chinensis	Eastern Spotted Dove	2020/9/29	3	0	19.90581986	75.31999178	https://www.inaturalist.org/observations/61234907
1203	Spilopelia chinensis	Eastern Spotted Dove	2021/5/21	3	0	19.91370362	82.64322940	https://www.inaturalist.org/observations/79633065
1204	Spilopelia chinensis	Eastern Spotted Dove	2021/12/25	1	0	19.94971085	110.12321470	https://www.inaturalist.org/observations/103622679
1205	Spilopelia chinensis	Eastern Spotted Dove	2019/1/23	0	0	20.01766302	110.24571840	https://www.inaturalist.org/observations/39138030
1206	Spilopelia chinensis	Eastern Spotted Dove	2022/1/2	1	0	20.03119119	110.30215220	https://www.inaturalist.org/observations/104328604
1207	Spilopelia chinensis	Eastern Spotted Dove	2022/1/2	1	0	20.03119119	110.30215220	https://www.inaturalist.org/observations/104328609
1208	Spilopelia chinensis	Eastern Spotted Dove	2022/2/19	2	0	20.03751807	110.31572560	https://www.inaturalist.org/observations/107033962
1209	Spilopelia chinensis	Eastern Spotted Dove	2021/2/16	1	0	20.04540390	110.20894590	https://www.inaturalist.org/observations/69651313
1210	Spilopelia chinensis	Eastern Spotted Dove	2020/5/18	1	0	20.04707100	110.22265230	https://www.inaturalist.org/observations/46323599
1211	Spilopelia chinensis	Eastern Spotted Dove	2022/7/31	5	0	20.12782020	73.24778580	https://www.inaturalist.org/observations/130125633
1212	Spilopelia chinensis	Eastern Spotted Dove	2015/6/17	5	0	20.22998607	79.35905457	https://www.inaturalist.org/observations/12189129
1213	Spilopelia chinensis	Eastern Spotted Dove	2017/5/2	1	0	20.24838270	79.36068900	https://www.inaturalist.org/observations/57004999
1214	Spilopelia chinensis	Eastern Spotted Dove	2011/4/14	2	0	20.34002560	79.30590060	https://www.inaturalist.org/observations/41762177
1215	Spilopelia chinensis	Eastern Spotted Dove	2017/5/1	1	0	20.34002560	79.30590060	https://www.inaturalist.org/observations/56308251
1216	Spilopelia chinensis	Eastern Spotted Dove	2014/6/24	3	0	20.34002560	79.30590060	https://www.inaturalist.org/observations/58578970
1217	Spilopelia chinensis	Eastern Spotted Dove	2016/12/19	1	0	20.40032712	85.82660573	https://www.inaturalist.org/observations/110214499
1218	Spilopelia chinensis	Eastern Spotted Dove	2022/9/25	2	0	20.50353906	84.90677811	https://www.inaturalist.org/observations/136394229

1219	Spilopelia chinensis	Eastern Spotted Dove	2022/2/23	3	0	20.52056100	81.64409120	https://www.inaturalist.org/observations/107305784
1220	Spilopelia chinensis	Eastern Spotted Dove	2021/2/28	4	0	20.60345828	81.52928855	https://www.inaturalist.org/observations/70540289
1221	Spilopelia chinensis	Eastern Spotted Dove	2020/9/18	3	0	20.63999485	79.64073647	https://www.inaturalist.org/observations/60070247
1222	Spilopelia chinensis	Eastern Spotted Dove	2020/10/31	2	0	20.66697517	77.11278282	https://www.inaturalist.org/observations/63877544
1223	Spilopelia chinensis	Eastern Spotted Dove	2020/10/31	3	0	20.67094429	77.08813961	https://www.inaturalist.org/observations/63876623
1224	Spilopelia chinensis	Eastern Spotted Dove	2021/9/15	1	0	20.72258215	81.19167522	https://www.inaturalist.org/observations/94900512
1225	Spilopelia chinensis	Eastern Spotted Dove	2021/9/26	1	0	20.72311493	81.18983388	https://www.inaturalist.org/observations/96327081
1226	Spilopelia chinensis	Eastern Spotted Dove	2018/9/18	1	0	20.72709677	81.20557979	https://www.inaturalist.org/observations/95794352
1227	Spilopelia chinensis	Eastern Spotted Dove	2016/3/21	3	0	20.73274500	82.43611833	https://www.inaturalist.org/observations/61468465
1228	Spilopelia chinensis	Eastern Spotted Dove	2020/9/18	1	0	20.78376283	79.45872111	https://www.inaturalist.org/observations/60070123
1229	Spilopelia chinensis	Eastern Spotted Dove	2022/8/23	3	0	20.79071700	82.06450960	https://www.inaturalist.org/observations/133784637
1230	Spilopelia chinensis	Eastern Spotted Dove	2022/8/23	3	0	20.79071700	82.06450960	https://www.inaturalist.org/observations/133784639
1231	Spilopelia chinensis	Eastern Spotted Dove	2022/8/23	3	0	20.79071700	82.06450960	https://www.inaturalist.org/observations/133784641
1232	Spilopelia chinensis	Eastern Spotted Dove	2022/8/23	2	0	20.79071700	82.06450960	https://www.inaturalist.org/observations/133784645
1233	Spilopelia chinensis	Eastern Spotted Dove	2022/8/23	3	0	20.79071700	82.06450960	https://www.inaturalist.org/observations/133784646
1234	Spilopelia chinensis	Eastern Spotted Dove	2022/8/23	4	0	20.79071700	82.06450960	https://www.inaturalist.org/observations/133784649
1235	Spilopelia chinensis	Eastern Spotted Dove	2022/8/23	4	0	20.79071700	82.06450960	https://www.inaturalist.org/observations/133784650
1236	Spilopelia chinensis	Eastern Spotted Dove	2022/8/23	4	0	20.79071700	82.06450960	https://www.inaturalist.org/observations/133784651
1237	Spilopelia chinensis	Eastern Spotted Dove	2017/12/2	4	0	20.82537810	73.70070880	https://www.inaturalist.org/observations/31116453
1238	Spilopelia chinensis	Eastern Spotted Dove	2018/3/30	5	0	20.90834153	85.00923279	https://www.inaturalist.org/observations/13185076
1239	Spilopelia chinensis	Eastern Spotted Dove	2021/5/2	2	0	20.94526962	72.95346905	https://www.inaturalist.org/observations/89026327
1240	Spilopelia chinensis	Eastern Spotted Dove	2020/1/13	2	0	20.94531409	72.95325011	https://www.inaturalist.org/observations/79400567
1241	Spilopelia chinensis	Eastern Spotted Dove	2013/2/12	1	0	20.97549643	77.49699674	https://www.inaturalist.org/observations/85625277

1242	Spilopelia chinensis	Eastern Spotted Dove	2017/1/4	1	0	21.00490556	70.75978889	https://www.inaturalist.org/observations/74618073
1243	Spilopelia chinensis	Eastern Spotted Dove	2020/2/23	3	0	21.05405500	75.88339500	https://www.inaturalist.org/observations/51030027
1244	Spilopelia chinensis	Eastern Spotted Dove	2021/7/10	1	0	21.06592278	81.24488153	https://www.inaturalist.org/observations/86421007
1245	Spilopelia chinensis	Eastern Spotted Dove	2021/2/2	3	0	21.07782947	75.94527975	https://www.inaturalist.org/observations/70595951
1246	Spilopelia chinensis	Eastern Spotted Dove	2021/5/19	2	0	21.08411468	80.66351205	https://www.inaturalist.org/observations/79376197
1247	Spilopelia chinensis	Eastern Spotted Dove	2021/1/11	2	0	21.09352478	80.15796897	https://www.inaturalist.org/observations/68052622
1248	Spilopelia chinensis	Eastern Spotted Dove	2019/12/11	4	0	21.14819293	80.65339711	https://www.inaturalist.org/observations/41443032
1249	Spilopelia chinensis	Eastern Spotted Dove	2020/9/27	4	0	21.17244392	80.76369758	https://www.inaturalist.org/observations/61300035
1250	Spilopelia chinensis	Eastern Spotted Dove	2019/1/17	4	0	21.18293953	72.80736542	https://www.inaturalist.org/observations/19690900
1251	Spilopelia chinensis	Eastern Spotted Dove	2016/2/5	7	0	21.18700646	80.60116611	https://www.inaturalist.org/observations/6546062
1252	Spilopelia chinensis	Eastern Spotted Dove	2020/8/7	2	0	21.18708525	80.76186363	https://www.inaturalist.org/observations/56762385
1253	Spilopelia chinensis	Eastern Spotted Dove	2019/10/9	2	0	21.19335583	79.04573869	https://www.inaturalist.org/observations/34104768
1254	Spilopelia chinensis	Eastern Spotted Dove	2020/3/15	2	0	21.20567301	81.17750578	https://www.inaturalist.org/observations/51234023
1255	Spilopelia chinensis	Eastern Spotted Dove	2013/2/10	1	0	21.40703127	77.16112333	https://www.inaturalist.org/observations/85331563
1256	Spilopelia chinensis	Eastern Spotted Dove	2021/8/25	1	0	21.45841973	83.17351327	https://www.inaturalist.org/observations/92514921
1257	Spilopelia chinensis	Eastern Spotted Dove	2022/2/8	1	0	21.47974470	82.53135810	https://www.inaturalist.org/observations/135563690
1258	Spilopelia chinensis	Eastern Spotted Dove	2018/10/8	6	0	21.54234452	81.80025201	https://www.inaturalist.org/observations/17315783
1259	Spilopelia chinensis	Eastern Spotted Dove	2021/12/12	3	0	21.60331639	82.72325139	https://www.inaturalist.org/observations/106690456
1260	Spilopelia chinensis	Eastern Spotted Dove	2022/8/12	5	0	21.75938167	83.09079167	https://www.inaturalist.org/observations/131690245
1261	Spilopelia chinensis	Eastern Spotted Dove	2021/5/8	2	0	21.80517459	83.30696690	https://www.inaturalist.org/observations/79921752
1262	Spilopelia chinensis	Eastern Spotted Dove	2021/5/8	2	0	21.80558180	83.30273190	https://www.inaturalist.org/observations/79921751
1263	Spilopelia chinensis	Eastern Spotted Dove	2021/8/4	1	0	21.81894667	83.32227500	https://www.inaturalist.org/observations/89949311
1264	Spilopelia chinensis	Eastern Spotted Dove	2021/2/20	1	0	21.83584645	88.88420105	https://www.inaturalist.org/observations/81275846

1265	Spilopelia chinensis	Eastern Spotted Dove	2017/1/27	2	0	21.83585950	88.88420110	https://www.inaturalist.org/observations/54038632
1266	Spilopelia chinensis	Eastern Spotted Dove	2011/12/26	2	0	21.83585950	88.88420110	https://www.inaturalist.org/observations/55264897
1267	Spilopelia chinensis	Eastern Spotted Dove	2011/12/26	2	0	21.83585950	88.88420110	https://www.inaturalist.org/observations/55264905
1268	Spilopelia chinensis	Eastern Spotted Dove	2022/3/9	4	0	21.83585950	88.88420110	https://www.inaturalist.org/observations/132027162
1269	Spilopelia chinensis	Eastern Spotted Dove	2021/8/26	0	0	21.84819180	83.29253761	https://www.inaturalist.org/observations/92915791
1270	Spilopelia chinensis	Eastern Spotted Dove	2021/8/7	1	0	21.87114667	83.26999167	https://www.inaturalist.org/observations/91651982
1271	Spilopelia chinensis	Eastern Spotted Dove	2022/7/1	2	0	21.88590710	69.76369070	https://www.inaturalist.org/observations/136883480
1272	Spilopelia chinensis	Eastern Spotted Dove	2009/9/26	2	0	21.94414553	120.81257710	https://www.inaturalist.org/observations/14756987
1273	Spilopelia chinensis	Eastern Spotted Dove	2011/6/15	1	0	21.95305816	120.72614870	https://www.inaturalist.org/observations/21294625
1274	Spilopelia chinensis	Eastern Spotted Dove	2022/4/17	1	0	21.99853500	73.47864000	https://www.inaturalist.org/observations/119406716
1275	Spilopelia chinensis	Eastern Spotted Dove	2019/10/22	2	0	22.00244363	120.74333410	https://www.inaturalist.org/observations/35709288
1276	Spilopelia chinensis	Eastern Spotted Dove	2014/3/13	2	0	22.02162625	120.75537970	https://www.inaturalist.org/observations/92882864
1277	Spilopelia chinensis	Eastern Spotted Dove	2019/10/20	0	0	22.02698667	120.85553670	https://www.inaturalist.org/observations/38184064
1278	Spilopelia chinensis	Eastern Spotted Dove	2021/9/4	1	0	22.05899167	82.05880333	https://www.inaturalist.org/observations/93748701
1279	Spilopelia chinensis	Eastern Spotted Dove	2019/12/19	3	0	22.07965450	82.14091520	https://www.inaturalist.org/observations/60917720
1280	Spilopelia chinensis	Eastern Spotted Dove	2022/4/17	1	0	22.08876152	88.75787269	https://www.inaturalist.org/observations/112352274
1281	Spilopelia chinensis	Eastern Spotted Dove	2022/4/2	4	0	22.09058134	88.76197813	https://www.inaturalist.org/observations/115794877
1282	Spilopelia chinensis	Eastern Spotted Dove	2022/4/16	1	0	22.09079617	88.76220349	https://www.inaturalist.org/observations/112496648
1283	Spilopelia chinensis	Eastern Spotted Dove	2022/4/16	2	0	22.09083500	88.76131333	https://www.inaturalist.org/observations/112701785
1284	Spilopelia chinensis	Eastern Spotted Dove	2022/4/16	2	0	22.09085000	88.76130500	https://www.inaturalist.org/observations/112701792
1285	Spilopelia chinensis	Eastern Spotted Dove	2022/4/17	2	0	22.09094310	88.76133710	https://www.inaturalist.org/observations/115079208
1286	Spilopelia chinensis	Eastern Spotted Dove	2022/4/16	3	0	22.09751674	88.76416656	https://www.inaturalist.org/observations/112254730
1287	Spilopelia chinensis	Eastern Spotted Dove	2021/9/4	0	0	22.09932500	82.07050333	https://www.inaturalist.org/observations/93748643

1288	Spilopelia chinensis	Eastern Spotted Dove	2022/2/13	1	0	22.11026780	113.55442910	https://www.inaturalist.org/observations/106670999
1289	Spilopelia chinensis	Eastern Spotted Dove	2021/2/14	1	0	22.11502599	113.57226470	https://www.inaturalist.org/observations/88584072
1290	Spilopelia chinensis	Eastern Spotted Dove	2021/9/6	1	0	22.11783422	113.56453500	https://www.inaturalist.org/observations/93933538
1291	Spilopelia chinensis	Eastern Spotted Dove	2021/4/3	1	0	22.11961222	113.56992460	https://www.inaturalist.org/observations/72759253
1292	Spilopelia chinensis	Eastern Spotted Dove	2021/12/20	1	0	22.12435266	88.83100009	https://www.inaturalist.org/observations/103967910
1293	Spilopelia chinensis	Eastern Spotted Dove	2021/8/30	1	0	22.12473333	82.14267333	https://www.inaturalist.org/observations/93207095
1294	Spilopelia chinensis	Eastern Spotted Dove	2021/8/30	1	0	22.12476333	82.14378667	https://www.inaturalist.org/observations/93207105
1295	Spilopelia chinensis	Eastern Spotted Dove	2021/8/30	0	0	22.12572500	82.14075000	https://www.inaturalist.org/observations/93207063
1296	Spilopelia chinensis	Eastern Spotted Dove	2020/11/28	1	0	22.12597533	113.57635530	https://www.inaturalist.org/observations/75069785
1297	Spilopelia chinensis	Eastern Spotted Dove	2019/9/11	3	0	22.12975656	82.13542353	https://www.inaturalist.org/observations/32520019
1298	Spilopelia chinensis	Eastern Spotted Dove	2021/8/31	3	0	22.13044167	82.13685833	https://www.inaturalist.org/observations/93448483
1299	Spilopelia chinensis	Eastern Spotted Dove	2021/8/31	3	0	22.13044167	82.13685833	https://www.inaturalist.org/observations/93448484
1300	Spilopelia chinensis	Eastern Spotted Dove	2019/9/5	4	0	22.13223090	82.13859692	https://www.inaturalist.org/observations/32274628
1301	Spilopelia chinensis	Eastern Spotted Dove	2021/12/20	2	0	22.13613162	88.79021687	https://www.inaturalist.org/observations/103961597
1302	Spilopelia chinensis	Eastern Spotted Dove	2021/12/20	1	0	22.13613162	88.79021687	https://www.inaturalist.org/observations/103961606
1303	Spilopelia chinensis	Eastern Spotted Dove	2021/12/20	1	0	22.13613162	88.79021687	https://www.inaturalist.org/observations/103961610
1304	Spilopelia chinensis	Eastern Spotted Dove	2021/12/20	2	0	22.13613162	88.79021687	https://www.inaturalist.org/observations/103961615
1305	Spilopelia chinensis	Eastern Spotted Dove	2021/5/3	2	0	22.15408281	113.56082020	https://www.inaturalist.org/observations/77315133
1306	Spilopelia chinensis	Eastern Spotted Dove	2021/2/1	2	0	22.15420348	113.56105970	https://www.inaturalist.org/observations/85112920
1307	Spilopelia chinensis	Eastern Spotted Dove	2021/5/22	3	0	22.15619167	113.55691670	https://www.inaturalist.org/observations/80362087
1308	Spilopelia chinensis	Eastern Spotted Dove	2019/4/27	1	0	22.15629450	113.55706170	https://www.inaturalist.org/observations/23331972
1309	Spilopelia chinensis	Eastern Spotted Dove	2019/7/8	3	0	22.15636731	113.55712980	https://www.inaturalist.org/observations/28418258
1310	Spilopelia chinensis	Eastern Spotted Dove	2022/5/7	2	0	22.15649690	113.55699320	https://www.inaturalist.org/observations/115860849

1311	Spilopelia chinensis	Eastern Spotted Dove	2019/6/2	1	0	22.15740437	113.56678710	https://www.inaturalist.org/observations/26389507
1312	Spilopelia chinensis	Eastern Spotted Dove	2022/5/14	3	0	22.15814158	113.54852660	https://www.inaturalist.org/observations/116948679
1313	Spilopelia chinensis	Eastern Spotted Dove	2019/10/24	2	0	22.15815000	113.55313000	https://www.inaturalist.org/observations/34820882
1314	Spilopelia chinensis	Eastern Spotted Dove	2022/5/6	3	0	22.15847200	113.55456300	https://www.inaturalist.org/observations/115708891
1315	Spilopelia chinensis	Eastern Spotted Dove	2022/4/21	3	0	22.15857896	113.55667440	https://www.inaturalist.org/observations/115322003
1316	Spilopelia chinensis	Eastern Spotted Dove	2022/5/5	2	0	22.15858211	113.55679290	https://www.inaturalist.org/observations/115731710
1317	Spilopelia chinensis	Eastern Spotted Dove	2021/4/5	2	0	22.15858786	113.55422090	https://www.inaturalist.org/observations/72992063
1318	Spilopelia chinensis	Eastern Spotted Dove	2021/3/17	1	0	22.15867639	113.56320070	https://www.inaturalist.org/observations/71677981
1319	Spilopelia chinensis	Eastern Spotted Dove	2021/5/26	2	0	22.15956875	113.55391270	https://www.inaturalist.org/observations/80345761
1320	Spilopelia chinensis	Eastern Spotted Dove	2022/4/30	2	0	22.16072138	113.55853320	https://www.inaturalist.org/observations/113781757
1321	Spilopelia chinensis	Eastern Spotted Dove	2021/1/23	1	0	22.16288754	113.55470590	https://www.inaturalist.org/observations/76903835
1322	Spilopelia chinensis	Eastern Spotted Dove	2020/8/30	1	0	22.16466639	113.55552550	https://www.inaturalist.org/observations/61467276
1323	Spilopelia chinensis	Eastern Spotted Dove	2021/3/28	1	0	22.16466750	114.25612150	https://www.inaturalist.org/observations/72335901
1324	Spilopelia chinensis	Eastern Spotted Dove	2020/8/30	1	0	22.16522312	113.55552630	https://www.inaturalist.org/observations/61414336
1325	Spilopelia chinensis	Eastern Spotted Dove	2022/2/20	1	0	22.16751500	73.49982333	https://www.inaturalist.org/observations/120020368
1326	Spilopelia chinensis	Eastern Spotted Dove	2021/8/26	1	0	22.16927353	114.25985330	https://www.inaturalist.org/observations/92559901
1327	Spilopelia chinensis	Eastern Spotted Dove	2022/4/5	1	0	22.18047525	113.47738140	https://www.inaturalist.org/observations/110498268
1328	Spilopelia chinensis	Eastern Spotted Dove	2019/10/13	2	0	22.18796626	113.54644720	https://www.inaturalist.org/observations/34576650
1329	Spilopelia chinensis	Eastern Spotted Dove	2021/5/2	1	0	22.18808611	113.55136940	https://www.inaturalist.org/observations/76534238
1330	Spilopelia chinensis	Eastern Spotted Dove	2021/5/2	1	0	22.18831389	113.55123060	https://www.inaturalist.org/observations/76534076
1331	Spilopelia chinensis	Eastern Spotted Dove	2021/5/2	1	0	22.18872778	113.55062780	https://www.inaturalist.org/observations/76534075
1332	Spilopelia chinensis	Eastern Spotted Dove	2022/4/16	2	0	22.18880369	113.55097190	https://www.inaturalist.org/observations/111624413
1333	Spilopelia chinensis	Eastern Spotted Dove	2021/3/13	1	0	22.19263010	113.54503780	https://www.inaturalist.org/observations/71109178

1334	Spilopelia chinensis	Eastern Spotted Dove	2021/3/13	1	0	22.19270740	113.54494700	https://www.inaturalist.org/observations/71110239
1335	Spilopelia chinensis	Eastern Spotted Dove	2021/10/6	2	0	22.19388889	113.54111110	https://www.inaturalist.org/observations/97457917
1336	Spilopelia chinensis	Eastern Spotted Dove	2021/3/28	2	0	22.19627073	113.54652250	https://www.inaturalist.org/observations/72234851
1337	Spilopelia chinensis	Eastern Spotted Dove	2020/1/29	1	0	22.19663811	113.54473880	https://www.inaturalist.org/observations/43346223
1338	Spilopelia chinensis	Eastern Spotted Dove	2020/4/26	1	0	22.19671440	113.54496770	https://www.inaturalist.org/observations/43568991
1339	Spilopelia chinensis	Eastern Spotted Dove	2010/6/6	1	0	22.19687969	113.54210240	https://www.inaturalist.org/observations/120204808
1340	Spilopelia chinensis	Eastern Spotted Dove	2022/5/2	2	0	22.19691849	113.54089360	https://www.inaturalist.org/observations/114610813
1341	Spilopelia chinensis	Eastern Spotted Dove	2020/10/3	1	0	22.19694444	113.54944440	https://www.inaturalist.org/observations/61550074
1342	Spilopelia chinensis	Eastern Spotted Dove	2020/4/4	1	0	22.19709899	113.54620300	https://www.inaturalist.org/observations/43631694
1343	Spilopelia chinensis	Eastern Spotted Dove	2019/11/5	2	0	22.19717284	113.54095400	https://www.inaturalist.org/observations/36617813
1344	Spilopelia chinensis	Eastern Spotted Dove	2021/4/2	1	0	22.19724722	113.55157500	https://www.inaturalist.org/observations/72760120
1345	Spilopelia chinensis	Eastern Spotted Dove	2022/3/13	2	0	22.19775833	113.55251330	https://www.inaturalist.org/observations/108490393
1346	Spilopelia chinensis	Eastern Spotted Dove	2020/3/14	1	0	22.19777778	113.55000000	https://www.inaturalist.org/observations/56125937
1347	Spilopelia chinensis	Eastern Spotted Dove	2022/3/8	2	0	22.19848854	113.55149640	https://www.inaturalist.org/observations/110086116
1348	Spilopelia chinensis	Eastern Spotted Dove	2022/4/3	1	0	22.19879549	113.55172470	https://www.inaturalist.org/observations/110390957
1349	Spilopelia chinensis	Eastern Spotted Dove	2022/3/21	1	0	22.19882000	113.54869170	https://www.inaturalist.org/observations/109626660
1350	Spilopelia chinensis	Eastern Spotted Dove	2022/3/27	1	0	22.19884426	113.55149780	https://www.inaturalist.org/observations/109739184
1351	Spilopelia chinensis	Eastern Spotted Dove	2021/4/2	2	0	22.19889874	113.55268120	https://www.inaturalist.org/observations/72749734
1352	Spilopelia chinensis	Eastern Spotted Dove	2022/3/31	1	0	22.19921000	113.54484600	https://www.inaturalist.org/observations/110142990
1353	Spilopelia chinensis	Eastern Spotted Dove	2019/6/3	1	0	22.19948667	113.55165000	https://www.inaturalist.org/observations/26415958
1354	Spilopelia chinensis	Eastern Spotted Dove	2019/6/1	2	0	22.19962883	113.55156710	https://www.inaturalist.org/observations/26166817
1355	Spilopelia chinensis	Eastern Spotted Dove	2019/5/18	1	0	22.19971765	113.55158800	https://www.inaturalist.org/observations/26148613
1356	Spilopelia chinensis	Eastern Spotted Dove	2019/5/18	2	0	22.19972339	113.55157240	https://www.inaturalist.org/observations/26174290

1357	Spilopelia chinensis	Eastern Spotted Dove	2019/5/18	2	0	22.19973324	113.55146470	https://www.inaturalist.org/observations/26148594
1358	Spilopelia chinensis	Eastern Spotted Dove	2019/5/18	2	0	22.19979286	113.55152130	https://www.inaturalist.org/observations/25299198
1359	Spilopelia chinensis	Eastern Spotted Dove	2019/6/7	2	0	22.19981947	113.55355210	https://www.inaturalist.org/observations/26539543
1360	Spilopelia chinensis	Eastern Spotted Dove	2019/6/6	2	0	22.19989881	113.55128480	https://www.inaturalist.org/observations/26493594
1361	Spilopelia chinensis	Eastern Spotted Dove	2020/8/8	1	0	22.20000000	113.55000000	https://www.inaturalist.org/observations/55767535
1362	Spilopelia chinensis	Eastern Spotted Dove	2019/7/7	2	0	22.20000560	114.13501730	https://www.inaturalist.org/observations/28719478
1363	Spilopelia chinensis	Eastern Spotted Dove	2015/12/12	2	0	22.20000560	114.13501730	https://www.inaturalist.org/observations/59101089
1364	Spilopelia chinensis	Eastern Spotted Dove	2021/5/2	1	0	22.20000560	114.13501730	https://www.inaturalist.org/observations/76837192
1365	Spilopelia chinensis	Eastern Spotted Dove	2022/4/3	2	0	22.20009500	113.55196400	https://www.inaturalist.org/observations/110733730
1366	Spilopelia chinensis	Eastern Spotted Dove	2021/3/28	1	0	22.20017833	113.54819500	https://www.inaturalist.org/observations/78853818
1367	Spilopelia chinensis	Eastern Spotted Dove	2021/3/28	1	0	22.20019667	113.54830830	https://www.inaturalist.org/observations/76493537
1368	Spilopelia chinensis	Eastern Spotted Dove	2021/2/18	1	0	22.20025983	113.55146080	https://www.inaturalist.org/observations/69767784
1369	Spilopelia chinensis	Eastern Spotted Dove	2019/6/3	1	0	22.20026333	113.55379500	https://www.inaturalist.org/observations/26317084
1370	Spilopelia chinensis	Eastern Spotted Dove	2019/6/3	2	0	22.20026333	113.55379500	https://www.inaturalist.org/observations/26438380
1371	Spilopelia chinensis	Eastern Spotted Dove	2020/3/6	1	0	22.20027939	113.55142590	https://www.inaturalist.org/observations/67285491
1372	Spilopelia chinensis	Eastern Spotted Dove	2021/5/8	0	0	22.20029428	113.55104480	https://www.inaturalist.org/observations/77839953
1373	Spilopelia chinensis	Eastern Spotted Dove	2022/3/28	2	0	22.20030753	113.54739340	https://www.inaturalist.org/observations/109740342
1374	Spilopelia chinensis	Eastern Spotted Dove	2021/2/28	0	0	22.20030911	113.55379820	https://www.inaturalist.org/observations/70292641
1375	Spilopelia chinensis	Eastern Spotted Dove	2019/1/2	1	0	22.20031416	113.54797590	https://www.inaturalist.org/observations/23901197
1376	Spilopelia chinensis	Eastern Spotted Dove	2020/12/28	1	0	22.20035716	113.54758380	https://www.inaturalist.org/observations/67182263
1377	Spilopelia chinensis	Eastern Spotted Dove	2022/3/31	2	0	22.20041333	113.54741670	https://www.inaturalist.org/observations/110017798
1378	Spilopelia chinensis	Eastern Spotted Dove	2021/5/8	2	0	22.20045937	113.55069440	https://www.inaturalist.org/observations/78823854
1379	Spilopelia chinensis	Eastern Spotted Dove	2021/5/8	3	0	22.20046842	113.55095840	https://www.inaturalist.org/observations/77849077

1380	Spilopelia chinensis	Eastern Spotted Dove	2019/6/2	2	0	22.20049327	113.54830930	https://www.inaturalist.org/observations/26239371
1381	Spilopelia chinensis	Eastern Spotted Dove	2021/5/8	1	0	22.20050500	113.55086330	https://www.inaturalist.org/observations/77849109
1382	Spilopelia chinensis	Eastern Spotted Dove	2022/2/2	1	0	22.20059167	113.54829170	https://www.inaturalist.org/observations/106694696
1383	Spilopelia chinensis	Eastern Spotted Dove	2021/4/30	1	0	22.20066544	113.55135230	https://www.inaturalist.org/observations/75781300
1384	Spilopelia chinensis	Eastern Spotted Dove	2021/3/27	1	0	22.20068550	113.54907230	https://www.inaturalist.org/observations/72132024
1385	Spilopelia chinensis	Eastern Spotted Dove	2022/6/18	1	0	22.20073555	113.54781630	https://www.inaturalist.org/observations/122356694
1386	Spilopelia chinensis	Eastern Spotted Dove	2021/5/8	2	0	22.20074100	113.55110960	https://www.inaturalist.org/observations/77842221
1387	Spilopelia chinensis	Eastern Spotted Dove	2022/3/28	2	0	22.20075082	113.54828290	https://www.inaturalist.org/observations/109740115
1388	Spilopelia chinensis	Eastern Spotted Dove	2022/3/28	1	0	22.20078833	113.55140000	https://www.inaturalist.org/observations/110002735
1389	Spilopelia chinensis	Eastern Spotted Dove	2019/6/1	1	0	22.20080500	113.54837000	https://www.inaturalist.org/observations/26165541
1390	Spilopelia chinensis	Eastern Spotted Dove	2021/5/8	1	0	22.20085736	113.55180810	https://www.inaturalist.org/observations/78552803
1391	Spilopelia chinensis	Eastern Spotted Dove	2022/3/24	2	0	22.20095650	113.54821730	https://www.inaturalist.org/observations/109611366
1392	Spilopelia chinensis	Eastern Spotted Dove	2022/3/24	2	0	22.20099899	113.54809820	https://www.inaturalist.org/observations/109348342
1393	Spilopelia chinensis	Eastern Spotted Dove	2022/3/24	1	0	22.20103833	113.54812500	https://www.inaturalist.org/observations/109348659
1394	Spilopelia chinensis	Eastern Spotted Dove	2021/3/26	1	0	22.20226900	114.13047700	https://www.inaturalist.org/observations/72141056
1395	Spilopelia chinensis	Eastern Spotted Dove	2019/4/26	1	0	22.20383871	113.54909850	https://www.inaturalist.org/observations/23101330
1396	Spilopelia chinensis	Eastern Spotted Dove	2020/6/21	2	0	22.20388000	113.54927000	https://www.inaturalist.org/observations/50367862
1397	Spilopelia chinensis	Eastern Spotted Dove	2021/4/2	1	0	22.20411333	113.55259670	https://www.inaturalist.org/observations/72685824
1398	Spilopelia chinensis	Eastern Spotted Dove	2022/4/5	2	0	22.20421667	113.55221670	https://www.inaturalist.org/observations/110636079
1399	Spilopelia chinensis	Eastern Spotted Dove	2022/4/5	2	0	22.20428075	113.55253880	https://www.inaturalist.org/observations/110575538
1400	Spilopelia chinensis	Eastern Spotted Dove	2022/6/11	2	0	22.20428838	113.55219230	https://www.inaturalist.org/observations/121204909
1401	Spilopelia chinensis	Eastern Spotted Dove	2022/4/3	1	0	22.20436000	113.55258000	https://www.inaturalist.org/observations/110473093
1402	Spilopelia chinensis	Eastern Spotted Dove	2022/4/3	2	0	22.20436730	113.55259230	https://www.inaturalist.org/observations/110379701

1403	Spilopelia chinensis	Eastern Spotted Dove	2022/4/3	2	0	22.20443333	113.55221670	https://www.inaturalist.org/observations/110284426
1404	Spilopelia chinensis	Eastern Spotted Dove	2022/4/5	2	0	22.20443333	113.55286330	https://www.inaturalist.org/observations/110468382
1405	Spilopelia chinensis	Eastern Spotted Dove	2022/4/3	2	0	22.20447114	113.55256270	https://www.inaturalist.org/observations/110581045
1406	Spilopelia chinensis	Eastern Spotted Dove	2022/4/3	2	0	22.20447833	113.55298670	https://www.inaturalist.org/observations/110285216
1407	Spilopelia chinensis	Eastern Spotted Dove	2022/4/5	2	0	22.20449667	113.55291170	https://www.inaturalist.org/observations/110642698
1408	Spilopelia chinensis	Eastern Spotted Dove	2021/4/2	1	0	22.20455342	113.55248520	https://www.inaturalist.org/observations/72978007
1409	Spilopelia chinensis	Eastern Spotted Dove	2021/4/2	1	0	22.20460400	113.55272300	https://www.inaturalist.org/observations/73032200
1410	Spilopelia chinensis	Eastern Spotted Dove	2020/12/25	1	0	22.20469167	113.55243000	https://www.inaturalist.org/observations/67223617
1411	Spilopelia chinensis	Eastern Spotted Dove	2021/3/11	1	0	22.20554700	114.13146800	https://www.inaturalist.org/observations/71008471
1412	Spilopelia chinensis	Eastern Spotted Dove	2017/5/10	3	0	22.20615989	114.02418140	https://www.inaturalist.org/observations/6244832
1413	Spilopelia chinensis	Eastern Spotted Dove	2019/5/4	2	0	22.20656057	113.54867360	https://www.inaturalist.org/observations/24501908
1414	Spilopelia chinensis	Eastern Spotted Dove	2021/5/9	1	0	22.20691077	113.54724600	https://www.inaturalist.org/observations/78046119
1415	Spilopelia chinensis	Eastern Spotted Dove	2020/3/7	1	0	22.20700203	113.54733760	https://www.inaturalist.org/observations/41826796
1416	Spilopelia chinensis	Eastern Spotted Dove	2021/2/17	2	0	22.20729430	113.54885390	https://www.inaturalist.org/observations/69709237
1417	Spilopelia chinensis	Eastern Spotted Dove	2022/4/30	2	0	22.20756746	114.12334430	https://www.inaturalist.org/observations/115135599
1418	Spilopelia chinensis	Eastern Spotted Dove	2019/5/11	1	0	22.20795176	113.54717510	https://www.inaturalist.org/observations/24933536
1419	Spilopelia chinensis	Eastern Spotted Dove	2021/3/20	1	0	22.20797267	113.55239960	https://www.inaturalist.org/observations/71578579
1420	Spilopelia chinensis	Eastern Spotted Dove	2021/4/2	1	0	22.20800276	113.54818210	https://www.inaturalist.org/observations/72699071
1421	Spilopelia chinensis	Eastern Spotted Dove	2022/4/3	2	0	22.20804667	113.54733330	https://www.inaturalist.org/observations/110635903
1422	Spilopelia chinensis	Eastern Spotted Dove	2022/4/3	2	0	22.20807500	113.54725500	https://www.inaturalist.org/observations/110368408
1423	Spilopelia chinensis	Eastern Spotted Dove	2019/11/6	2	0	22.20816452	114.13583020	https://www.inaturalist.org/observations/35392948
1424	Spilopelia chinensis	Eastern Spotted Dove	2019/5/9	2	0	22.20841212	113.54820020	https://www.inaturalist.org/observations/24834168
1425	Spilopelia chinensis	Eastern Spotted Dove	2021/4/5	1	0	22.20861126	113.54735610	https://www.inaturalist.org/observations/72997985

1426	Spilopelia chinensis	Eastern Spotted Dove	2018/5/1	4	0	22.20870274	114.02881620	https://www.inaturalist.org/observations/12060663
1427	Spilopelia chinensis	Eastern Spotted Dove	2019/6/2	1	0	22.20878333	113.55395500	https://www.inaturalist.org/observations/26380397
1428	Spilopelia chinensis	Eastern Spotted Dove	2019/5/27	3	0	22.21030617	113.55294040	https://www.inaturalist.org/observations/25865911
1429	Spilopelia chinensis	Eastern Spotted Dove	2019/5/27	1	0	22.21030617	113.55294040	https://www.inaturalist.org/observations/25866036
1430	Spilopelia chinensis	Eastern Spotted Dove	2021/4/30	1	0	22.21083333	113.55326170	https://www.inaturalist.org/observations/76884676
1431	Spilopelia chinensis	Eastern Spotted Dove	2019/6/9	2	0	22.21109370	113.55220540	https://www.inaturalist.org/observations/26660304
1432	Spilopelia chinensis	Eastern Spotted Dove	2019/6/1	1	0	22.21118000	113.55255000	https://www.inaturalist.org/observations/26150134
1433	Spilopelia chinensis	Eastern Spotted Dove	2019/6/5	1	0	22.21131667	113.55252000	https://www.inaturalist.org/observations/26432842
1434	Spilopelia chinensis	Eastern Spotted Dove	2021/4/26	2	0	22.21133318	113.55283100	https://www.inaturalist.org/observations/75308017
1435	Spilopelia chinensis	Eastern Spotted Dove	2019/6/5	2	0	22.21138055	113.54290790	https://www.inaturalist.org/observations/26434169
1436	Spilopelia chinensis	Eastern Spotted Dove	2021/11/30	1	0	22.21155128	113.54264840	https://www.inaturalist.org/observations/104395892
1437	Spilopelia chinensis	Eastern Spotted Dove	2020/12/10	1	0	22.21281075	114.19749950	https://www.inaturalist.org/observations/66418065
1438	Spilopelia chinensis	Eastern Spotted Dove	2019/6/5	1	0	22.21472100	113.54723600	https://www.inaturalist.org/observations/26430086
1439	Spilopelia chinensis	Eastern Spotted Dove	2019/6/2	2	0	22.21585575	113.54636280	https://www.inaturalist.org/observations/26237839
1440	Spilopelia chinensis	Eastern Spotted Dove	2019/6/2	2	0	22.21585587	113.54643500	https://www.inaturalist.org/observations/26237871
1441	Spilopelia chinensis	Eastern Spotted Dove	2019/6/5	2	0	22.21608000	113.54592830	https://www.inaturalist.org/observations/26488500
1442	Spilopelia chinensis	Eastern Spotted Dove	2019/6/5	0	0	22.21615196	113.54594240	https://www.inaturalist.org/observations/26430084
1443	Spilopelia chinensis	Eastern Spotted Dove	2019/5/13	1	0	22.21620561	113.54600950	https://www.inaturalist.org/observations/25042974
1444	Spilopelia chinensis	Eastern Spotted Dove	2021/6/11	1	0	22.21638333	113.54535000	https://www.inaturalist.org/observations/82524235
1445	Spilopelia chinensis	Eastern Spotted Dove	2012/12/26	4	0	22.21744777	114.12029730	http://www.inaturalist.org/observations/166874
1446	Spilopelia chinensis	Eastern Spotted Dove	2019/4/28	2	0	22.21771485	114.21013910	https://www.inaturalist.org/observations/23649827
1447	Spilopelia chinensis	Eastern Spotted Dove	2022/7/29	2	0	22.21774667	114.20970330	https://www.inaturalist.org/observations/128388202
1448	Spilopelia chinensis	Eastern Spotted Dove	2019/3/18	2	0	22.21817500	114.21203670	https://www.inaturalist.org/observations/51286954

1449	Spilopelia chinensis	Eastern Spotted Dove	2017/3/7	1	0	22.21829571	114.03137780	https://www.inaturalist.org/observations/73209500
1450	Spilopelia chinensis	Eastern Spotted Dove	2022/7/23	2	0	22.21831640	114.20699330	https://www.inaturalist.org/observations/127469551
1451	Spilopelia chinensis	Eastern Spotted Dove	2021/11/18	1	0	22.21874619	114.21015170	https://www.inaturalist.org/observations/101393409
1452	Spilopelia chinensis	Eastern Spotted Dove	2021/3/26	2	0	22.21930200	114.11890900	https://www.inaturalist.org/observations/73364231
1453	Spilopelia chinensis	Eastern Spotted Dove	2021/4/26	1	0	22.21979113	113.54821730	https://www.inaturalist.org/observations/75246499
1454	Spilopelia chinensis	Eastern Spotted Dove	2020/5/3	1	0	22.22261054	113.91454910	https://www.inaturalist.org/observations/45149503
1455	Spilopelia chinensis	Eastern Spotted Dove	2022/5/2	2	0	22.22280261	114.11891990	https://www.inaturalist.org/observations/116055019
1456	Spilopelia chinensis	Eastern Spotted Dove	2018/4/29	3	0	22.22361183	114.11389160	https://www.inaturalist.org/observations/11825945
1457	Spilopelia chinensis	Eastern Spotted Dove	2020/4/24	2	0	22.22388374	114.11245690	https://www.inaturalist.org/observations/43028912
1458	Spilopelia chinensis	Eastern Spotted Dove	2019/4/27	2	0	22.22394900	114.11513420	https://www.inaturalist.org/observations/23322441
1459	Spilopelia chinensis	Eastern Spotted Dove	2020/10/16	1	0	22.22406282	114.11218730	https://www.inaturalist.org/observations/62750619
1460	Spilopelia chinensis	Eastern Spotted Dove	2021/2/4	1	0	22.22418400	114.11423700	https://www.inaturalist.org/observations/69048910
1461	Spilopelia chinensis	Eastern Spotted Dove	2019/5/13	1	0	22.22430333	114.11504450	https://www.inaturalist.org/observations/25050778
1462	Spilopelia chinensis	Eastern Spotted Dove	2018/5/7	4	0	22.22445000	114.11074720	https://www.inaturalist.org/observations/12318255
1463	Spilopelia chinensis	Eastern Spotted Dove	2020/4/25	1	0	22.22450086	114.11589220	https://www.inaturalist.org/observations/43592619
1464	Spilopelia chinensis	Eastern Spotted Dove	2018/4/29	5	0	22.22455500	114.11315280	https://www.inaturalist.org/observations/11727794
1465	Spilopelia chinensis	Eastern Spotted Dove	2021/3/12	1	0	22.22478777	114.30043100	https://www.inaturalist.org/observations/71494581
1466	Spilopelia chinensis	Eastern Spotted Dove	2018/4/28	2	0	22.22514365	114.11509320	https://www.inaturalist.org/observations/11569153
1467	Spilopelia chinensis	Eastern Spotted Dove	2012/10/14	4	0	22.22683346	114.11340940	http://www.inaturalist.org/observations/135100
1468	Spilopelia chinensis	Eastern Spotted Dove	2014/6/9	5	0	22.22769800	114.11426100	http://www.inaturalist.org/observations/725738
1469	Spilopelia chinensis	Eastern Spotted Dove	2018/4/27	3	0	22.22913008	114.11570110	https://www.inaturalist.org/observations/11468618
1470	Spilopelia chinensis	Eastern Spotted Dove	2020/7/21	2	0	22.22978900	113.93190000	https://www.inaturalist.org/observations/53787473
1471	Spilopelia chinensis	Eastern Spotted Dove	2020/3/29	1	0	22.22991090	114.11026640	https://www.inaturalist.org/observations/44865344

1472	Spilopelia chinensis	Eastern Spotted Dove	2018/4/27	5	0	22.23289143	114.17155270	https://www.inaturalist.org/observations/11482287
1473	Spilopelia chinensis	Eastern Spotted Dove	2018/4/27	1	0	22.23377000	114.17086670	https://www.inaturalist.org/observations/11503913
1474	Spilopelia chinensis	Eastern Spotted Dove	2022/8/27	2	0	22.23669241	114.16496950	https://www.inaturalist.org/observations/132898696
1475	Spilopelia chinensis	Eastern Spotted Dove	2021/6/8	3	0	22.23910333	114.19608330	https://www.inaturalist.org/observations/83011474
1476	Spilopelia chinensis	Eastern Spotted Dove	2021/12/7	1	0	22.24060633	113.97595920	https://www.inaturalist.org/observations/102689739
1477	Spilopelia chinensis	Eastern Spotted Dove	2021/5/3	2	0	22.24060833	114.22370830	https://www.inaturalist.org/observations/76851800
1478	Spilopelia chinensis	Eastern Spotted Dove	2021/4/11	1	0	22.24071431	114.15021120	https://www.inaturalist.org/observations/78538321
1479	Spilopelia chinensis	Eastern Spotted Dove	2022/5/2	1	0	22.24114836	114.15002280	https://www.inaturalist.org/observations/114617805
1480	Spilopelia chinensis	Eastern Spotted Dove	2018/4/21	5	0	22.24120833	114.15521950	https://www.inaturalist.org/observations/11251747
1481	Spilopelia chinensis	Eastern Spotted Dove	2016/3/20	2	0	22.24144884	113.97766630	https://www.inaturalist.org/observations/49217091
1482	Spilopelia chinensis	Eastern Spotted Dove	2020/4/25	2	0	22.24159477	113.97451770	https://www.inaturalist.org/observations/43307581
1483	Spilopelia chinensis	Eastern Spotted Dove	2021/11/17	2	0	22.24249359	113.97847970	https://www.inaturalist.org/observations/101521526
1484	Spilopelia chinensis	Eastern Spotted Dove	2018/4/29	3	0	22.24250208	114.14736720	https://www.inaturalist.org/observations/11883638
1485	Spilopelia chinensis	Eastern Spotted Dove	2018/4/28	4	0	22.24326893	114.14703670	https://www.inaturalist.org/observations/11579055
1486	Spilopelia chinensis	Eastern Spotted Dove	2020/2/22	1	0	22.24337600	113.97534900	https://www.inaturalist.org/observations/41153303
1487	Spilopelia chinensis	Eastern Spotted Dove	2021/3/27	1	0	22.24425797	114.15272300	https://www.inaturalist.org/observations/72141772
1488	Spilopelia chinensis	Eastern Spotted Dove	2020/10/8	1	0	22.24429167	114.16405550	https://www.inaturalist.org/observations/62010977
1489	Spilopelia chinensis	Eastern Spotted Dove	2018/4/29	6	0	22.24438073	114.17536940	https://www.inaturalist.org/observations/11879201
1490	Spilopelia chinensis	Eastern Spotted Dove	2021/3/27	1	0	22.24451144	114.15404060	https://www.inaturalist.org/observations/72142138
1491	Spilopelia chinensis	Eastern Spotted Dove	2021/5/2	2	0	22.24451284	114.15399430	https://www.inaturalist.org/observations/76455896
1492	Spilopelia chinensis	Eastern Spotted Dove	2020/11/12	1	0	22.24456993	114.17642870	https://www.inaturalist.org/observations/64716735
1493	Spilopelia chinensis	Eastern Spotted Dove	2020/4/27	1	0	22.24459521	114.17660370	https://www.inaturalist.org/observations/44049316
1494	Spilopelia chinensis	Eastern Spotted Dove	2020/4/10	2	0	22.24461506	114.17649640	https://www.inaturalist.org/observations/41808866

1495	Spilopelia chinensis	Eastern Spotted Dove	2018/4/29	2	0	22.24467833	114.17662830	https://www.inaturalist.org/observations/11763601
1496	Spilopelia chinensis	Eastern Spotted Dove	2020/11/9	1	0	22.24473430	114.14933090	https://www.inaturalist.org/observations/64501593
1497	Spilopelia chinensis	Eastern Spotted Dove	2020/11/27	1	0	22.24485400	114.15069800	https://www.inaturalist.org/observations/65662582
1498	Spilopelia chinensis	Eastern Spotted Dove	2020/11/27	1	0	22.24485400	114.15069800	https://www.inaturalist.org/observations/65662830
1499	Spilopelia chinensis	Eastern Spotted Dove	2021/5/2	1	0	22.24491916	114.15281640	https://www.inaturalist.org/observations/76454317
1500	Spilopelia chinensis	Eastern Spotted Dove	2020/11/27	1	0	22.24495997	114.15059800	https://www.inaturalist.org/observations/65662546
1501	Spilopelia chinensis	Eastern Spotted Dove	2020/11/18	2	0	22.24496040	114.15021860	https://www.inaturalist.org/observations/65113596
1502	Spilopelia chinensis	Eastern Spotted Dove	2019/11/1	1	0	22.24498758	114.17621870	https://www.inaturalist.org/observations/35185103
1503	Spilopelia chinensis	Eastern Spotted Dove	2020/3/29	1	0	22.24511157	114.17623890	https://www.inaturalist.org/observations/41218876
1504	Spilopelia chinensis	Eastern Spotted Dove	2019/11/7	2	0	22.24513985	114.14927360	https://www.inaturalist.org/observations/35397127
1505	Spilopelia chinensis	Eastern Spotted Dove	2019/11/7	1	0	22.24515439	114.14931090	https://www.inaturalist.org/observations/35397165
1506	Spilopelia chinensis	Eastern Spotted Dove	2018/11/6	2	0	22.24517666	114.14903480	https://www.inaturalist.org/observations/18157695
1507	Spilopelia chinensis	Eastern Spotted Dove	2019/4/26	1	0	22.24518833	114.17607830	https://www.inaturalist.org/observations/23097773
1508	Spilopelia chinensis	Eastern Spotted Dove	2019/11/7	4	0	22.24520615	114.14921190	https://www.inaturalist.org/observations/35402313
1509	Spilopelia chinensis	Eastern Spotted Dove	2020/11/26	1	0	22.24523997	114.15076600	https://www.inaturalist.org/observations/65608344
1510	Spilopelia chinensis	Eastern Spotted Dove	2019/4/26	2	0	22.24524123	114.17603990	https://www.inaturalist.org/observations/23097782
1511	Spilopelia chinensis	Eastern Spotted Dove	2019/4/26	1	0	22.24524169	114.17610920	https://www.inaturalist.org/observations/23108293
1512	Spilopelia chinensis	Eastern Spotted Dove	2018/4/29	3	0	22.24524667	114.17614670	https://www.inaturalist.org/observations/11759317
1513	Spilopelia chinensis	Eastern Spotted Dove	2019/4/26	1	0	22.24525500	114.17606330	https://www.inaturalist.org/observations/23097861
1514	Spilopelia chinensis	Eastern Spotted Dove	2019/4/26	1	0	22.24526667	114.17604170	https://www.inaturalist.org/observations/23098114
1515	Spilopelia chinensis	Eastern Spotted Dove	2020/6/24	2	0	22.24585333	114.18608000	https://www.inaturalist.org/observations/50859982
1516	Spilopelia chinensis	Eastern Spotted Dove	2021/5/2	1	0	22.24603269	114.18633430	https://www.inaturalist.org/observations/76458422
1517	Spilopelia chinensis	Eastern Spotted Dove	2021/5/2	2	0	22.24612500	114.18635500	https://www.inaturalist.org/observations/76868685

1518	Spilopelia chinensis	Eastern Spotted Dove	2020/10/20	1	0	22.24667667	113.86154670	https://www.inaturalist.org/observations/63103832
1519	Spilopelia chinensis	Eastern Spotted Dove	2018/4/11	1	0	22.24680633	114.17493430	https://www.inaturalist.org/observations/10771692
1520	Spilopelia chinensis	Eastern Spotted Dove	2021/4/25	1	0	22.24718225	114.15417380	https://www.inaturalist.org/observations/75160597
1521	Spilopelia chinensis	Eastern Spotted Dove	2020/12/14	1	0	22.24722833	114.16136950	https://www.inaturalist.org/observations/66558004
1522	Spilopelia chinensis	Eastern Spotted Dove	2018/4/1	3	0	22.24731333	114.15878330	https://www.inaturalist.org/observations/10540559
1523	Spilopelia chinensis	Eastern Spotted Dove	2019/11/12	2	0	22.24747239	114.15259340	https://www.inaturalist.org/observations/35603188
1524	Spilopelia chinensis	Eastern Spotted Dove	2020/10/20	1	0	22.24787889	113.86467860	https://www.inaturalist.org/observations/63105596
1525	Spilopelia chinensis	Eastern Spotted Dove	2018/1/10	4	0	22.24810833	114.15414440	https://www.inaturalist.org/observations/16276737
1526	Spilopelia chinensis	Eastern Spotted Dove	2019/4/18	2	0	22.24830500	114.15683050	https://www.inaturalist.org/observations/22783816
1527	Spilopelia chinensis	Eastern Spotted Dove	2019/8/26	3	0	22.24830680	114.15243980	https://www.inaturalist.org/observations/31597819
1528	Spilopelia chinensis	Eastern Spotted Dove	2021/2/12	1	0	22.24853000	114.15679830	https://www.inaturalist.org/observations/97978506
1529	Spilopelia chinensis	Eastern Spotted Dove	2020/12/14	2	0	22.24928672	114.13602310	https://www.inaturalist.org/observations/66563574
1530	Spilopelia chinensis	Eastern Spotted Dove	2020/12/14	2	0	22.24929758	114.13599660	https://www.inaturalist.org/observations/66564283
1531	Spilopelia chinensis	Eastern Spotted Dove	2022/2/27	2	0	22.24989680	114.15624400	https://www.inaturalist.org/observations/107530331
1532	Spilopelia chinensis	Eastern Spotted Dove	2019/4/10	1	0	22.25019500	114.15678330	https://www.inaturalist.org/observations/22262412
1533	Spilopelia chinensis	Eastern Spotted Dove	2019/2/7	3	0	22.25073300	114.13712160	https://www.inaturalist.org/observations/20173544
1534	Spilopelia chinensis	Eastern Spotted Dove	2022/1/23	2	0	22.25151094	114.18414840	https://www.inaturalist.org/observations/105487060
1535	Spilopelia chinensis	Eastern Spotted Dove	2020/9/10	2	0	22.25231950	113.86611950	https://www.inaturalist.org/observations/115532166
1536	Spilopelia chinensis	Eastern Spotted Dove	2021/5/7	1	0	22.25274460	114.13430970	https://www.inaturalist.org/observations/77687463
1537	Spilopelia chinensis	Eastern Spotted Dove	2019/11/4	1	0	22.25292434	114.15852470	https://www.inaturalist.org/observations/35265636
1538	Spilopelia chinensis	Eastern Spotted Dove	2022/8/16	3	0	22.25323548	114.15842420	https://www.inaturalist.org/observations/131157219
1539	Spilopelia chinensis	Eastern Spotted Dove	2013/6/30	3	0	22.25397172	113.91109000	https://www.inaturalist.org/observations/17165294
1540	Spilopelia chinensis	Eastern Spotted Dove	2018/11/9	3	0	22.25438604	114.23916770	https://www.inaturalist.org/observations/18410532

1541	Spilopelia chinensis	Eastern Spotted Dove	2021/6/28	1	0	22.25455500	113.86095330	https://www.inaturalist.org/observations/84839871
1542	Spilopelia chinensis	Eastern Spotted Dove	2012/5/13	1	0	22.25505524	113.90473480	https://www.inaturalist.org/observations/108242945
1543	Spilopelia chinensis	Eastern Spotted Dove	2019/8/22	1	0	22.25513333	113.90651670	https://www.inaturalist.org/observations/132517942
1544	Spilopelia chinensis	Eastern Spotted Dove	2018/5/26	4	0	22.25549370	114.13854590	https://www.inaturalist.org/observations/12823929
1545	Spilopelia chinensis	Eastern Spotted Dove	2021/9/8	1	0	22.25564300	113.86682600	https://www.inaturalist.org/observations/94099624
1546	Spilopelia chinensis	Eastern Spotted Dove	2018/3/30	1	0	22.25566795	114.16108360	https://www.inaturalist.org/observations/10506663
1547	Spilopelia chinensis	Eastern Spotted Dove	2018/12/31	5	0	22.25580000	113.90777000	https://www.inaturalist.org/observations/19393529
1548	Spilopelia chinensis	Eastern Spotted Dove	2018/3/29	2	0	22.25604527	114.16099780	https://www.inaturalist.org/observations/10506564
1549	Spilopelia chinensis	Eastern Spotted Dove	2021/5/1	1	0	22.25670833	114.13160000	https://www.inaturalist.org/observations/76020831
1550	Spilopelia chinensis	Eastern Spotted Dove	2021/5/2	1	0	22.25673872	114.16443580	https://www.inaturalist.org/observations/77238741
1551	Spilopelia chinensis	Eastern Spotted Dove	2022/3/31	1	0	22.25685009	114.21064250	https://www.inaturalist.org/observations/110003433
1552	Spilopelia chinensis	Eastern Spotted Dove	2022/3/31	2	0	22.25685009	114.21064250	https://www.inaturalist.org/observations/110003604
1553	Spilopelia chinensis	Eastern Spotted Dove	2019/8/10	4	0	22.25685901	113.85802820	https://www.inaturalist.org/observations/30573163
1554	Spilopelia chinensis	Eastern Spotted Dove	2022/4/29	2	0	22.25686929	114.13188730	https://www.inaturalist.org/observations/113348627
1555	Spilopelia chinensis	Eastern Spotted Dove	2018/10/11	4	0	22.25789269	114.13921660	https://www.inaturalist.org/observations/17451634
1556	Spilopelia chinensis	Eastern Spotted Dove	2020/4/2	1	0	22.25795474	114.13898260	https://www.inaturalist.org/observations/41221168
1557	Spilopelia chinensis	Eastern Spotted Dove	2020/5/2	1	0	22.25801804	114.13913010	https://www.inaturalist.org/observations/44572676
1558	Spilopelia chinensis	Eastern Spotted Dove	2018/5/6	4	0	22.25827790	114.13854460	https://www.inaturalist.org/observations/12179456
1559	Spilopelia chinensis	Eastern Spotted Dove	2022/6/24	2	0	22.25902167	114.12962220	https://www.inaturalist.org/observations/123207412
1560	Spilopelia chinensis	Eastern Spotted Dove	2019/5/25	3	0	22.25908638	114.13981710	https://www.inaturalist.org/observations/25733440
1561	Spilopelia chinensis	Eastern Spotted Dove	2020/4/25	2	0	22.26020467	114.13685900	https://www.inaturalist.org/observations/43292184
1562	Spilopelia chinensis	Eastern Spotted Dove	2021/5/1	2	0	22.26072976	114.16675390	https://www.inaturalist.org/observations/76071924
1563	Spilopelia chinensis	Eastern Spotted Dove	2018/12/26	3	0	22.26082866	114.14047560	https://www.inaturalist.org/observations/19228465

1564	Spilopelia chinensis	Eastern Spotted Dove	2016/12/5	2	0	22.26102743	114.25068860	https://www.inaturalist.org/observations/9006960
1565	Spilopelia chinensis	Eastern Spotted Dove	2021/5/1	1	0	22.26126667	114.13179720	https://www.inaturalist.org/observations/76022823
1566	Spilopelia chinensis	Eastern Spotted Dove	2022/2/13	2	0	22.26154376	114.26821190	https://www.inaturalist.org/observations/106663350
1567	Spilopelia chinensis	Eastern Spotted Dove	2020/4/26	1	0	22.26188000	114.24975500	https://www.inaturalist.org/observations/43532674
1568	Spilopelia chinensis	Eastern Spotted Dove	2019/4/27	2	0	22.26203649	114.25251480	https://www.inaturalist.org/observations/23325971
1569	Spilopelia chinensis	Eastern Spotted Dove	2019/11/4	3	0	22.26258741	114.25397040	https://www.inaturalist.org/observations/35280650
1570	Spilopelia chinensis	Eastern Spotted Dove	2018/11/8	3	0	22.26285769	114.24842280	https://www.inaturalist.org/observations/18222329
1571	Spilopelia chinensis	Eastern Spotted Dove	2018/11/19	5	0	22.26286258	114.23898910	https://www.inaturalist.org/observations/18491303
1572	Spilopelia chinensis	Eastern Spotted Dove	2018/11/8	4	0	22.26298249	114.24973390	https://www.inaturalist.org/observations/18245306
1573	Spilopelia chinensis	Eastern Spotted Dove	2021/10/11	1	0	22.26298588	114.24904310	https://www.inaturalist.org/observations/97868515
1574	Spilopelia chinensis	Eastern Spotted Dove	2020/11/29	1	0	22.26304023	114.25380200	https://www.inaturalist.org/observations/65816375
1575	Spilopelia chinensis	Eastern Spotted Dove	2020/11/16	2	0	22.26312907	114.24911690	https://www.inaturalist.org/observations/64999587
1576	Spilopelia chinensis	Eastern Spotted Dove	2018/11/8	4	0	22.26322653	114.24971490	https://www.inaturalist.org/observations/18245913
1577	Spilopelia chinensis	Eastern Spotted Dove	2018/11/8	4	0	22.26324765	114.24966760	https://www.inaturalist.org/observations/18245301
1578	Spilopelia chinensis	Eastern Spotted Dove	2020/11/8	1	0	22.26324780	114.15879830	https://www.inaturalist.org/observations/96349505
1579	Spilopelia chinensis	Eastern Spotted Dove	2020/11/4	2	0	22.26327374	114.24921100	https://www.inaturalist.org/observations/64136848
1580	Spilopelia chinensis	Eastern Spotted Dove	2020/12/28	1	0	22.26329167	114.18747000	https://www.inaturalist.org/observations/67238048
1581	Spilopelia chinensis	Eastern Spotted Dove	2019/11/9	3	0	22.26330313	114.25368680	https://www.inaturalist.org/observations/35484221
1582	Spilopelia chinensis	Eastern Spotted Dove	2021/3/25	1	0	22.26334724	114.23613860	https://www.inaturalist.org/observations/72000341
1583	Spilopelia chinensis	Eastern Spotted Dove	2018/11/8	4	0	22.26335846	114.24941610	https://www.inaturalist.org/observations/18241495
1584	Spilopelia chinensis	Eastern Spotted Dove	2021/9/30	1	0	22.26336567	113.99293560	https://www.inaturalist.org/observations/96703867
1585	Spilopelia chinensis	Eastern Spotted Dove	2019/11/9	3	0	22.26338773	114.25362780	https://www.inaturalist.org/observations/35484223
1586	Spilopelia chinensis	Eastern Spotted Dove	2022/6/3	2	0	22.26350891	114.24838100	https://www.inaturalist.org/observations/119984867

1587	Spilopelia chinensis	Eastern Spotted Dove	2019/3/21	2	0	22.26355430	114.13695330	https://www.inaturalist.org/observations/21447816
1588	Spilopelia chinensis	Eastern Spotted Dove	2019/11/9	5	0	22.26355511	114.25347360	https://www.inaturalist.org/observations/35484479
1589	Spilopelia chinensis	Eastern Spotted Dove	2019/11/9	6	0	22.26364944	114.25345620	https://www.inaturalist.org/observations/35484486
1590	Spilopelia chinensis	Eastern Spotted Dove	2020/11/26	1	0	22.26365442	114.24910640	https://www.inaturalist.org/observations/65628154
1591	Spilopelia chinensis	Eastern Spotted Dove	2019/3/21	2	0	22.26367490	114.13688730	https://www.inaturalist.org/observations/21437037
1592	Spilopelia chinensis	Eastern Spotted Dove	2021/11/12	0	0	22.26372249	114.25267560	https://www.inaturalist.org/observations/100913112
1593	Spilopelia chinensis	Eastern Spotted Dove	2019/11/9	4	0	22.26373462	114.25343470	https://www.inaturalist.org/observations/35484467
1594	Spilopelia chinensis	Eastern Spotted Dove	2022/4/30	2	0	22.26375375	114.24819980	https://www.inaturalist.org/observations/113711930
1595	Spilopelia chinensis	Eastern Spotted Dove	2020/11/29	1	0	22.26386812	114.25198360	https://www.inaturalist.org/observations/65816102
1596	Spilopelia chinensis	Eastern Spotted Dove	2020/11/29	1	0	22.26388798	114.25197890	https://www.inaturalist.org/observations/65816101
1597	Spilopelia chinensis	Eastern Spotted Dove	2020/11/22	2	0	22.26391729	114.25334800	https://www.inaturalist.org/observations/65397105
1598	Spilopelia chinensis	Eastern Spotted Dove	2020/5/6	1	0	22.26394453	114.13773000	https://www.inaturalist.org/observations/45065463
1599	Spilopelia chinensis	Eastern Spotted Dove	2019/11/9	5	0	22.26396155	114.25252780	https://www.inaturalist.org/observations/35474864
1600	Spilopelia chinensis	Eastern Spotted Dove	2020/11/18	1	0	22.26400250	114.24970850	https://www.inaturalist.org/observations/65114444
1601	Spilopelia chinensis	Eastern Spotted Dove	2021/11/12	1	0	22.26400300	114.24987600	https://www.inaturalist.org/observations/100889056
1602	Spilopelia chinensis	Eastern Spotted Dove	2016/10/10	4	0	22.26400800	114.14278900	http://www.inaturalist.org/observations/4312079
1603	Spilopelia chinensis	Eastern Spotted Dove	2020/11/13	1	0	22.26403265	114.24909700	https://www.inaturalist.org/observations/64774620
1604	Spilopelia chinensis	Eastern Spotted Dove	2021/11/12	1	0	22.26404464	114.25328660	https://www.inaturalist.org/observations/100919014
1605	Spilopelia chinensis	Eastern Spotted Dove	2021/11/12	1	0	22.26404464	114.25328660	https://www.inaturalist.org/observations/100919015
1606	Spilopelia chinensis	Eastern Spotted Dove	2018/11/7	5	0	22.26404507	114.24989490	https://www.inaturalist.org/observations/18189953
1607	Spilopelia chinensis	Eastern Spotted Dove	2019/11/8	4	0	22.26405531	114.24951080	https://www.inaturalist.org/observations/35441735
1608	Spilopelia chinensis	Eastern Spotted Dove	2018/11/7	2	0	22.26406908	114.24963570	https://www.inaturalist.org/observations/18185115
1609	Spilopelia chinensis	Eastern Spotted Dove	2019/11/4	2	0	22.26407160	114.24937280	https://www.inaturalist.org/observations/35267412

1610	Spilopelia chinensis	Eastern Spotted Dove	2021/11/9	1	0	22.26408713	114.24953370	https://www.inaturalist.org/observations/100683170
1611	Spilopelia chinensis	Eastern Spotted Dove	2020/11/18	1	0	22.26409300	114.24892900	https://www.inaturalist.org/observations/65136205
1612	Spilopelia chinensis	Eastern Spotted Dove	2021/11/11	1	0	22.26413098	114.24918610	https://www.inaturalist.org/observations/100808155
1613	Spilopelia chinensis	Eastern Spotted Dove	2021/11/9	1	0	22.26413638	114.24953770	https://www.inaturalist.org/observations/100676315
1614	Spilopelia chinensis	Eastern Spotted Dove	2021/11/11	2	0	22.26417189	114.24941060	https://www.inaturalist.org/observations/100808338
1615	Spilopelia chinensis	Eastern Spotted Dove	2021/11/9	1	0	22.26417732	114.24964090	https://www.inaturalist.org/observations/100676326
1616	Spilopelia chinensis	Eastern Spotted Dove	2021/11/9	1	0	22.26417732	114.24964090	https://www.inaturalist.org/observations/100676328
1617	Spilopelia chinensis	Eastern Spotted Dove	2018/4/30	4	0	22.26417991	114.25274850	https://www.inaturalist.org/observations/11870979
1618	Spilopelia chinensis	Eastern Spotted Dove	2019/11/5	4	0	22.26418170	114.24962870	https://www.inaturalist.org/observations/35306363
1619	Spilopelia chinensis	Eastern Spotted Dove	2021/11/12	1	0	22.26418612	114.24978900	https://www.inaturalist.org/observations/100919001
1620	Spilopelia chinensis	Eastern Spotted Dove	2019/11/8	6	0	22.26422956	114.24930940	https://www.inaturalist.org/observations/35447852
1621	Spilopelia chinensis	Eastern Spotted Dove	2019/11/8	7	0	22.26422956	114.24930940	https://www.inaturalist.org/observations/35447853
1622	Spilopelia chinensis	Eastern Spotted Dove	2021/11/9	1	0	22.26423565	114.24959800	https://www.inaturalist.org/observations/100676316
1623	Spilopelia chinensis	Eastern Spotted Dove	2021/11/9	1	0	22.26423565	114.24959800	https://www.inaturalist.org/observations/100676320
1624	Spilopelia chinensis	Eastern Spotted Dove	2021/11/12	1	0	22.26424398	114.24933110	https://www.inaturalist.org/observations/100918962
1625	Spilopelia chinensis	Eastern Spotted Dove	2020/11/22	3	0	22.26426203	114.25244570	https://www.inaturalist.org/observations/65397037
1626	Spilopelia chinensis	Eastern Spotted Dove	2021/11/11	1	0	22.26426995	114.24924560	https://www.inaturalist.org/observations/100807575
1627	Spilopelia chinensis	Eastern Spotted Dove	2021/11/11	1	0	22.26427477	114.24922360	https://www.inaturalist.org/observations/100807731
1628	Spilopelia chinensis	Eastern Spotted Dove	2020/11/29	1	0	22.26427622	114.25334800	https://www.inaturalist.org/observations/65816356
1629	Spilopelia chinensis	Eastern Spotted Dove	2021/11/11	1	0	22.26428344	114.24921820	https://www.inaturalist.org/observations/100807608
1630	Spilopelia chinensis	Eastern Spotted Dove	2019/11/5	3	0	22.26429060	114.24947600	https://www.inaturalist.org/observations/35312916
1631	Spilopelia chinensis	Eastern Spotted Dove	2021/11/11	1	0	22.26429896	114.24919180	https://www.inaturalist.org/observations/100807752
1632	Spilopelia chinensis	Eastern Spotted Dove	2021/11/9	2	0	22.26430143	114.24971740	https://www.inaturalist.org/observations/100676339

1633	Spilopelia chinensis	Eastern Spotted Dove	2021/11/9	1	0	22.26430143	114.24971740	https://www.inaturalist.org/observations/100676343
1634	Spilopelia chinensis	Eastern Spotted Dove	2021/11/11	2	0	22.26430741	114.24922800	https://www.inaturalist.org/observations/100807519
1635	Spilopelia chinensis	Eastern Spotted Dove	2021/11/11	2	0	22.26431074	114.24931020	https://www.inaturalist.org/observations/100808320
1636	Spilopelia chinensis	Eastern Spotted Dove	2021/11/9	2	0	22.26431386	114.24980320	https://www.inaturalist.org/observations/100676303
1637	Spilopelia chinensis	Eastern Spotted Dove	2021/11/9	1	0	22.26431386	114.24980320	https://www.inaturalist.org/observations/100676304
1638	Spilopelia chinensis	Eastern Spotted Dove	2021/11/11	1	0	22.26431870	114.24937250	https://www.inaturalist.org/observations/100807521
1639	Spilopelia chinensis	Eastern Spotted Dove	2021/11/11	2	0	22.26431966	114.24926560	https://www.inaturalist.org/observations/100807532
1640	Spilopelia chinensis	Eastern Spotted Dove	2018/4/28	6	0	22.26436889	113.98956300	https://www.inaturalist.org/observations/11592974
1641	Spilopelia chinensis	Eastern Spotted Dove	2020/11/26	1	0	22.26442463	114.24929730	https://www.inaturalist.org/observations/65630791
1642	Spilopelia chinensis	Eastern Spotted Dove	2019/11/7	4	0	22.26443190	114.25155750	https://www.inaturalist.org/observations/35403211
1643	Spilopelia chinensis	Eastern Spotted Dove	2020/12/26	1	0	22.26444710	73.04697050	https://www.inaturalist.org/observations/67847754
1644	Spilopelia chinensis	Eastern Spotted Dove	2021/11/4	1	0	22.26444713	114.24912420	https://www.inaturalist.org/observations/100211165
1645	Spilopelia chinensis	Eastern Spotted Dove	2019/11/4	3	0	22.26444736	114.25004550	https://www.inaturalist.org/observations/35266855
1646	Spilopelia chinensis	Eastern Spotted Dove	2020/12/14	2	0	22.26444802	114.24918580	https://www.inaturalist.org/observations/66560955
1647	Spilopelia chinensis	Eastern Spotted Dove	2019/11/8	4	0	22.26446270	114.24922510	https://www.inaturalist.org/observations/35438265
1648	Spilopelia chinensis	Eastern Spotted Dove	2021/11/2	1	0	22.26446651	114.24967230	https://www.inaturalist.org/observations/100055801
1649	Spilopelia chinensis	Eastern Spotted Dove	2021/11/23	1	0	22.26448534	114.24825970	https://www.inaturalist.org/observations/101746323
1650	Spilopelia chinensis	Eastern Spotted Dove	2021/11/11	1	0	22.26449333	114.24924670	https://www.inaturalist.org/observations/100807711
1651	Spilopelia chinensis	Eastern Spotted Dove	2019/11/4	2	0	22.26450217	114.25009480	https://www.inaturalist.org/observations/35266908
1652	Spilopelia chinensis	Eastern Spotted Dove	2021/11/8	2	0	22.26451359	114.24934110	https://www.inaturalist.org/observations/100544427
1653	Spilopelia chinensis	Eastern Spotted Dove	2018/11/8	3	0	22.26452573	114.25246640	https://www.inaturalist.org/observations/18232517
1654	Spilopelia chinensis	Eastern Spotted Dove	2020/11/7	1	0	22.26452672	114.25214220	https://www.inaturalist.org/observations/64345155
1655	Spilopelia chinensis	Eastern Spotted Dove	2019/11/4	4	0	22.26453590	114.24980580	https://www.inaturalist.org/observations/35266808

1656	Spilopelia chinensis	Eastern Spotted Dove	2019/11/4	4	0	22.26454970	114.24977710	https://www.inaturalist.org/observations/35266812
1657	Spilopelia chinensis	Eastern Spotted Dove	2019/11/4	2	0	22.26456591	114.24925290	https://www.inaturalist.org/observations/35266875
1658	Spilopelia chinensis	Eastern Spotted Dove	2019/11/4	3	0	22.26456680	114.24977430	https://www.inaturalist.org/observations/35266795
1659	Spilopelia chinensis	Eastern Spotted Dove	2021/10/11	1	0	22.26457167	114.24769670	https://www.inaturalist.org/observations/97868537
1660	Spilopelia chinensis	Eastern Spotted Dove	2020/11/23	1	0	22.26459880	114.24913400	https://www.inaturalist.org/observations/65458894
1661	Spilopelia chinensis	Eastern Spotted Dove	2019/11/7	4	0	22.26460733	114.25299020	https://www.inaturalist.org/observations/35408719
1662	Spilopelia chinensis	Eastern Spotted Dove	2019/11/7	4	0	22.26460733	114.25299020	https://www.inaturalist.org/observations/35408721
1663	Spilopelia chinensis	Eastern Spotted Dove	2021/11/8	1	0	22.26460977	114.24932230	https://www.inaturalist.org/observations/100544473
1664	Spilopelia chinensis	Eastern Spotted Dove	2019/11/8	4	0	22.26462334	114.25189110	https://www.inaturalist.org/observations/35446395
1665	Spilopelia chinensis	Eastern Spotted Dove	2019/11/4	4	0	22.26462639	114.25033310	https://www.inaturalist.org/observations/35266790
1666	Spilopelia chinensis	Eastern Spotted Dove	2019/11/4	2	0	22.26464056	114.24999040	https://www.inaturalist.org/observations/35266821
1667	Spilopelia chinensis	Eastern Spotted Dove	2020/11/26	1	0	22.26464803	114.24943820	https://www.inaturalist.org/observations/65630790
1668	Spilopelia chinensis	Eastern Spotted Dove	2021/11/10	1	0	22.26468356	114.24945920	https://www.inaturalist.org/observations/100731959
1669	Spilopelia chinensis	Eastern Spotted Dove	2020/11/19	2	0	22.26468833	114.24934000	https://www.inaturalist.org/observations/65182928
1670	Spilopelia chinensis	Eastern Spotted Dove	2021/11/8	1	0	22.26471898	114.24943510	https://www.inaturalist.org/observations/100544015
1671	Spilopelia chinensis	Eastern Spotted Dove	2019/11/4	2	0	22.26473205	114.25215230	https://www.inaturalist.org/observations/35278689
1672	Spilopelia chinensis	Eastern Spotted Dove	2020/11/22	2	0	22.26473449	114.25231290	https://www.inaturalist.org/observations/65397048
1673	Spilopelia chinensis	Eastern Spotted Dove	2019/11/5	3	0	22.26474687	114.25292390	https://www.inaturalist.org/observations/35320296
1674	Spilopelia chinensis	Eastern Spotted Dove	2020/11/23	3	0	22.26475833	114.24945000	https://www.inaturalist.org/observations/65458931
1675	Spilopelia chinensis	Eastern Spotted Dove	2021/11/12	1	0	22.26475837	114.25309530	https://www.inaturalist.org/observations/100919838
1676	Spilopelia chinensis	Eastern Spotted Dove	2020/12/1	2	0	22.26476333	114.25288330	https://www.inaturalist.org/observations/65937652
1677	Spilopelia chinensis	Eastern Spotted Dove	2020/11/22	1	0	22.26476406	114.25226170	https://www.inaturalist.org/observations/65397046
1678	Spilopelia chinensis	Eastern Spotted Dove	2021/11/12	1	0	22.26476522	114.25296840	https://www.inaturalist.org/observations/100919805

1679	Spilopelia chinensis	Eastern Spotted Dove	2020/11/17	1	0	22.26478833	114.25304500	https://www.inaturalist.org/observations/65179493
1680	Spilopelia chinensis	Eastern Spotted Dove	2020/11/7	0	0	22.26479800	114.25238220	https://www.inaturalist.org/observations/64344839
1681	Spilopelia chinensis	Eastern Spotted Dove	2020/11/22	1	0	22.26480688	114.25226110	https://www.inaturalist.org/observations/65397050
1682	Spilopelia chinensis	Eastern Spotted Dove	2020/12/1	1	0	22.26482000	114.25288330	https://www.inaturalist.org/observations/65937323
1683	Spilopelia chinensis	Eastern Spotted Dove	2019/11/23	1	0	22.26483099	114.25295420	https://www.inaturalist.org/observations/36404578
1684	Spilopelia chinensis	Eastern Spotted Dove	2021/11/12	1	0	22.26483703	114.25294430	https://www.inaturalist.org/observations/100919804
1685	Spilopelia chinensis	Eastern Spotted Dove	2019/11/5	3	0	22.26484776	114.24955600	https://www.inaturalist.org/observations/35315065
1686	Spilopelia chinensis	Eastern Spotted Dove	2020/12/1	1	0	22.26496333	114.25248670	https://www.inaturalist.org/observations/65937641
1687	Spilopelia chinensis	Eastern Spotted Dove	2020/11/29	1	0	22.26498112	114.25290010	https://www.inaturalist.org/observations/65816318
1688	Spilopelia chinensis	Eastern Spotted Dove	2019/11/7	4	0	22.26500649	114.25238450	https://www.inaturalist.org/observations/35403866
1689	Spilopelia chinensis	Eastern Spotted Dove	2020/11/29	1	0	22.26502355	114.25289010	https://www.inaturalist.org/observations/65816320
1690	Spilopelia chinensis	Eastern Spotted Dove	2020/11/29	1	0	22.26504722	114.25289400	https://www.inaturalist.org/observations/65816323
1691	Spilopelia chinensis	Eastern Spotted Dove	2020/11/29	1	0	22.26504837	114.25286730	https://www.inaturalist.org/observations/65816317
1692	Spilopelia chinensis	Eastern Spotted Dove	2022/5/3	2	0	22.26508331	114.25184630	https://www.inaturalist.org/observations/115005524
1693	Spilopelia chinensis	Eastern Spotted Dove	2019/11/7	5	0	22.26509530	114.25185650	https://www.inaturalist.org/observations/35403424
1694	Spilopelia chinensis	Eastern Spotted Dove	2019/11/7	5	0	22.26512237	114.25227470	https://www.inaturalist.org/observations/35405128
1695	Spilopelia chinensis	Eastern Spotted Dove	2022/9/10	1	0	22.26514371	114.25275700	https://www.inaturalist.org/observations/134584745
1696	Spilopelia chinensis	Eastern Spotted Dove	2019/11/7	4	0	22.26515337	114.25228160	https://www.inaturalist.org/observations/35408711
1697	Spilopelia chinensis	Eastern Spotted Dove	2019/11/7	5	0	22.26525029	114.25235010	https://www.inaturalist.org/observations/35408698
1698	Spilopelia chinensis	Eastern Spotted Dove	2019/11/7	4	0	22.26525029	114.25235010	https://www.inaturalist.org/observations/35408700
1699	Spilopelia chinensis	Eastern Spotted Dove	2020/11/7	2	0	22.26525400	114.25147600	https://www.inaturalist.org/observations/64345159
1700	Spilopelia chinensis	Eastern Spotted Dove	2019/11/8	4	0	22.26528649	114.25004990	https://www.inaturalist.org/observations/35443697
1701	Spilopelia chinensis	Eastern Spotted Dove	2020/4/10	1	0	22.26539167	114.13745000	https://www.inaturalist.org/observations/41817518

1702	Spilopelia chinensis	Eastern Spotted Dove	2020/4/26	1	0	22.26539167	114.13745000	https://www.inaturalist.org/observations/43588432
1703	Spilopelia chinensis	Eastern Spotted Dove	2020/5/2	1	0	22.26539167	114.13745000	https://www.inaturalist.org/observations/44572561
[truncated: 445,490 more chars]
